# Nutritional Balancing Strategies in Low-Fishmeal Aquafeeds: A PRISMA 2020-Based Systematic Review Beyond Lysine and Methionine Supplementation

**DOI:** 10.3390/vetsci13080762

**Published:** 2026-07-30

**Authors:** Md Hashibur Rahman, Hyuncheol Jeon, Haham Kim, Seunghyung Lee

**Affiliations:** 1 Major of Aquaculture and Applied Life Sciences, Division of Fisheries Life Sciences, Pukyong National University, Busan 48547, Republic of Korea; hashibur@pukyong.ac.kr (M.H.R.); conjp@naver.com (H.J.); haham7@naver.com (H.K.); 2 Interdisciplinary Program of Marine and Fisheries Sciences and Convergent Technology, Pukyong National University, Busan 48513, Republic of Korea; 3 Feeds and Foods Nutrition Research Center, Pukyong National University, Busan 48547, Republic of Korea

**Keywords:** nutritional equivalence, lysine, methionine, taurine, phytase, functional amino acids, gut health

## Abstract

Fishmeal reduction is central to sustainable aquafeed development, but low-fishmeal diets may not reproduce all the nutritional and functional properties of fishmeal after lysine and methionine correction. This PRISMA 2020-based systematic review synthesized peer-reviewed feeding trials evaluating nutritional-balancing strategies beyond these two amino acids in low-fishmeal, fishmeal-free, or high-alternative-protein diets. Alternative protein ingredients were treated as dietary matrices rather than interventions, and studies evaluating ingredient replacement alone were excluded. Twenty-three studies were included. Study-level effect sizes were estimable for 21 studies, providing 24 representative performance-related comparisons. Eight comparisons had 95% confidence intervals excluding zero in favor of the intervention, whereas 16 crossed the null; estimates were not pooled because of substantial heterogeneity in species, diet formulation, intervention, dose, and outcome definition. The evidence covered essential and functional amino acids, taurine and related nitrogenous compounds, palatability enhancers, enzymes, phosphorus and mineral balance, antioxidant support, lipid-associated nutrients, and gut-targeted strategies. Phytase provides the clearest mechanism through which phosphorus utilization can be improved in phytate-rich or phosphorus-limited diets. Taurine responses were generally favorable but imprecise in the four marine finfish species. Protein hydrolysates, fermented ingredients, yeast products, probiotics, hydroxyproline, and other additives produce context-dependent benefits for performance, nutrient utilization, intestinal conditions, immunity, stress resistance, or product quality. Across strategies, efficacy depended mainly on matching the intervention to the nutritional or functional limitation created by the complete diet. Nutritional equivalence should therefore be evaluated via integrated production, mechanism-specific, health, product-quality, environmental, and economic outcomes rather than the fishmeal-replacement percentage alone.

## Simple Summary

Fishmeal remains a key functional ingredient in aquafeeds, yet its limited availability and price volatility have accelerated the transition toward low-fishmeal formulations. However, effective fishmeal reduction requires more than protein replacement, as fishmeal provides essential nutritional and functional components that support growth, nutrient utilization, intestinal integrity, immunity, and stress resistance. This review highlights nutritional strategies that enhance the performance of low-fishmeal diets, including taurine, phosphorus-mobilizing enzymes, protein hydrolysates, fermented ingredients, yeast-derived products, probiotics, minerals, vitamins, and gut health-promoting additives. Within the species, life stages, diet matrices, and experimental conditions represented in the available evidence, future low-fishmeal feeds should be developed through context-specific formulations and validation aimed at restoring the relevant biological functions of fishmeal-based diets rather than through universal ingredient or additive recommendations.

## 1. Introduction

Aquaculture has become a major source of aquatic food, with farmed aquatic animal production recently exceeding capture fisheries [1]. This expansion has increased the demand for nutritionally adequate, cost-effective, and sustainable aquafeeds. Fishmeal remains a valuable feed ingredient because it provides highly digestible protein, a balanced essential amino acid profile, strong palatability, minerals, taurine, peptides, and other functional nitrogenous compounds [2,3,4]. However, supply constraints, price volatility, and sustainability concerns have accelerated the use of plant proteins, resulting in the production of animal byproducts, insect meals, microbial ingredients, and algal products in low-fishmeal and fishmeal-free diets [5,6].

Alternative ingredients can supply substantial amounts of crude protein but may not reproduce the full nutritional and functional value of fishmeal. Plant-derived ingredients may have imbalanced amino acid profiles, lower digestibility and palatability, antinutritional factors, and limited phosphorus and trace mineral availability [5,7,8]. Consequently, fishmeal replacement should be evaluated according to the nutritional performance of the complete diet rather than the proportion of fishmeal removed. Lysine and methionine supplementation can correct common first-limiting amino acid deficiencies [3,9], yet highly reduced-fishmeal diets may still impair feed intake, growth, nutrient utilization, metabolism, intestinal integrity, immunity, and stress tolerance. These effects indicate that relevant limitations often persist after lysine and methionine have been balanced.

Depending on the species, life stage, ingredient matrix, and culture conditions, other indispensable amino acids, including histidine, arginine, threonine, tryptophan, and branched-chain amino acids, may become limiting. In addition to protein synthesis, these nutrients contribute to mucosal integrity, immune regulation, appetite, antioxidant defense, nitrogen metabolism, and stress responses [9,10,11,12,13,14,15,16,17,18]. A reduction in fishmeal may also lower the dietary supply of taurine, glycine, proline, hydroxyproline, creatine-related compounds, nucleotides, and bioactive peptides [19,20]. Taurine is particularly relevant because of its roles in bile acid conjugation, lipid digestion, osmoregulation, antioxidant defense, and stress physiology [19].

The nutritional consequences of fishmeal reduction extend beyond amino acid balance. Greater inclusion of plant ingredients can increase phytate and other antinutritional factors, thereby reducing phosphorus and trace mineral availability and affecting digestion and intestinal function [7,8]. The reduced use of marine ingredients may also alter the supply of EPA, DHA, phospholipids, cholesterol, and feeding stimulants, particularly when fishmeal reduction coincides with fish-oil replacement [21,22,23]. Potential corrective strategies therefore include phytase, protein hydrolysates, feed attractants, processed or fermented ingredients, mineral and lipid balancing, functional amino acids, and gut-targeted additives.

Low-fishmeal formulations should consequently be considered a whole-diet challenge rather than simple protein substitution. Alternative ingredients may simultaneously alter nutrient availability, amino acid ratios, palatability, intestinal morphology, microbiota, immunity, and lipid metabolism [24,25,26,27,28]. Moreover, nutritional inadequacies may first appear in mechanism-specific outcomes rather than in growth. For example, inadequate long-chain polyunsaturated fatty acid supply may alter tissue lipid composition, phytate-associated mineral limitation may reduce phosphorus retention, and intestinal disturbance may affect mucosal structure or microbiota before body weight gain decreases [22,29,30]. Evaluations based only on growth or feed conversion may therefore overlook biologically and commercially relevant effects on health, resilience, product quality, and nutrient discharge.

Previous reviews have generally examined individual alternative ingredients, nutrients, additives, or biological functions. Consequently, the evidence remains fragmented across species, diet matrices, interventions, and outcome domains. No systematic synthesis has critically linked the nutritional and functional limitations created by fishmeal reduction with mechanism-matched strategies and the extent to which these strategies restore production, nutrient utilization, health, product quality, or sustainability outcomes.

This review addresses this gap by systematically evaluating nutritional-balancing strategies beyond lysine and methionine in low-fishmeal, fishmeal-free, and high-alternative-protein aquafeeds. Specifically, it aims to (1) identify the principal nutritional and functional limitations associated with fishmeal reduction; (2) assess the consistency and strength of evidence for targeted corrective strategies; (3) determine how species, life stage, diet matrix, replacement level, dose, and culture conditions influence efficacy; and (4) develop a mechanism- and outcome-based framework for evaluating nutritional equivalence.

The primary review questions were as follows: Which nutritional-balancing strategies beyond lysine and methionine improve or restore production, nutrient utilization, physiological conditions, intestinal health, immune and antioxidant capacity, stress and disease resilience, product quality, or sustainability-related outcomes in aquatic animals fed low-fishmeal diets? The secondary questions include which limitations remain after lysine and methionine correction, whether the available evidence supports equivalence or noninferiority to fishmeal-based reference diets and which mechanism-aligned end points are most appropriate for evaluating efficacy and safety.

In this review, nutritional equivalence is defined as the capacity of a low-fishmeal diet to maintain the biologically and practically important functions of an appropriate fishmeal-based reference diet under comparable conditions. It is considered context-specific and dependent on species, life stage, diet matrix, culture environment, and production objective. The operational criteria used to distinguish formal equivalence, functional parity, superiority, and failure to demonstrate equivalence are described in Section 2.3.

## 2. Materials and Methods

### 2.1. Reporting Guidelines

This systematic review was conducted and reported in accordance with the Preferred Reporting Items for Systematic Reviews and Meta-Analyses 2020 statement (PRISMA 2020) [31]. The reporting of the search strategy followed the recommendations of the PRISMA-S extension where applicable [32]. The study identification and selection process was summarized via a PRISMA 2020 flow diagram. The review protocol was retrospectively registered on the Open Science Framework (OSF) for transparency, and the complete protocol and registration records are publicly available (DOI: 10.17605/OSF.IO/RCUZX accessed on 24 July 2026). The eligibility criteria, search strategy, outcomes of interest, data-extraction categories, and synthesis approach were established before study screening.

### 2.2. Review Questions

The review was guided by the primary and secondary research questions stated at the end of the Introduction. Briefly, the review evaluated which nutritional-balancing strategies beyond lysine and methionine improve or restore production; nutritional, physiological, health, and product-quality; and sustainability-related outcomes in low-fishmeal aquafeeds. It also examined the nutritional gaps associated with fishmeal reduction, contextual modifiers of intervention responses, evidence for nutritional equivalence, and appropriate mechanism-aligned and veterinary-relevant end points.

### 2.3. Operational Definition of Nutritional Equivalence

Nutritional equivalence was defined as the maintenance of prespecified biological and production functions of an appropriate fishmeal-based reference diet in the same species, life stage, culture condition, and experimental period. The preferred benchmark was a contemporaneous fishmeal-based positive-control diet. Because fishmeal quality varies with raw material source, freshness, processing, storage, and composition, each positive control was interpreted as a study-specific benchmark rather than a uniform fishmeal standard. Equivalence was considered across three levels: (1) core production outcomes, including growth, feed conversion ratio, feed intake, survival, and, where relevant, whole-body composition; (2) mechanism-specific outcomes aligned with the tested strategy; and (3) health and safety outcomes used to detect adverse physiological, intestinal, immune, or pathological effects.

For outcomes with a clearly favorable direction, formal equivalence or noninferiority should ideally be evaluated using a prespecified biologically or economically justified margin and a 95% confidence interval. None of the included studies were designed as formal equivalence or noninferiority trials with prospectively defined margins. Therefore, nonsignificant differences from a fishmeal-based control were not interpreted as proof of equivalence. A pragmatic 5% margin was retained as an illustrative framework for future study design and cautious retrospective interpretation, but it was not used to establish formal nutritional equivalence in the present review. Where the available controls and data permitted interpretation, studies were instead described in terms of functional parity, potential superiority, lack of equivalence, or failure to demonstrate equivalence. The operational categories are summarized in Table 1.

### 2.4. Eligibility Framework

Eligibility was structured according to the PICOS. Populations included fish, shrimp, and other aquaculture species fed low-fishmeal, fishmeal-reduced, fishmeal-free, fishmeal-replacement, or high-alternative-protein diets. Eligible interventions included taurine, other essential or functional amino acids, nonprotein nitrogenous compounds, phytase and other enzymes, minerals, vitamins, antioxidants, feed attractants, protein hydrolysates, fermented ingredients, probiotics, prebiotics, nucleotides, organic acids, lipid-associated nutrients, and other functional additives.

Alternative protein ingredients were treated as components of the dietary matrix rather than interventions. Studies of plants, animals, insects, microbes, algae, or other alternative ingredients were eligible only when an additional nutritional-balancing strategy beyond lysine and methionine was tested. Eligible comparators included fishmeal-based controls, non-supplemented low-fishmeal diets, diets balanced only with lysine and/or methionine, or different levels of the same intervention. Eligible outcomes included production; digestibility; retention; and physiological, immune, antioxidant, intestinal, microbiota, disease, stress, product-quality, environmental, and economic measures. Only peer-reviewed original feeding trials were included.

### 2.5. Information Sources

A systematic literature search was conducted in the Web of Science, Scopus, PubMed, ScienceDirect, and CAB abstracts. Additional searches used Google Scholar and publisher platforms, including SpringerLink, the Wiley Online Library, Taylor & Francis Online, and Elsevier journals. The reference lists of the included studies and relevant reviews were screened, and forward citation tracking was performed to identify additional records. The initial search was conducted on 17 December 2025 and updated on 18 June 2026, with no lower publication date restrictions. All records identified through the updated search were screened using the predefined eligibility criteria, and the final study selection results are presented in the PRISMA 2020 flow diagram. The ASFA, AGRICOLA, and FSTA were not included. Although the multisource strategy provided broad coverage of aquaculture nutrition and feed research, the omission of these specialized databases may have limited the retrieval of older, regionally indexed, less-cited, or non-English publications.

### 2.6. Search Strategy

The search strategy combined terms related to fishmeal reduction, aquaculture species, nutritional-balancing interventions, and biological or production outcomes. Search terms were adapted to the syntax and available search fields of each database and were applied to titles, abstracts, and keywords where possible. The core search string was as follows: (“fishmeal replacement” OR “fish meal replacement” OR “low fishmeal” OR “low-fishmeal” OR “fishmeal-free” OR “fishmeal reduction” OR “plant protein” OR “alternative protein” OR “alternative ingredient”) AND (aquafeed OR aquaculture OR fish OR shrimp OR crustacean OR “fish culture” OR “fish farming”) AND (taurine OR histidine OR arginine OR threonine OR tryptophan OR leucine OR isoleucine OR valine OR “branched-chain amino acid” OR “functional amino acid” OR “crystalline amino acid” OR “protected amino acid” OR phytase OR phosphorus OR phosphate OR mineral OR vitamin OR antioxidant OR attractant OR palatability OR hydrolysate OR fermented OR fermentation OR enzyme OR probiotic OR prebiotic OR nucleotide OR “organic acid” OR microbiota OR “gut health”) AND (growth OR “weight gain” OR SGR OR “specific growth rate” OR “feed intake” OR “feed efficiency” OR “feed conversion ratio” OR FCR OR digestibility OR “apparent digestibility” OR “apparent digestibility coefficient” OR availability OR bioavailability OR absorption OR utilization OR “nutrient retention” OR immunity OR antioxidant OR stress OR “stress tolerance” OR “gut morphology” OR “intestinal morphology” OR microbiota OR “blood biochemistry” OR survival OR “disease resistance” OR “product quality” OR “phosphorus retention” OR “phosphorus discharge”).

Study identification was not based solely on exact keyword matching. The database searches were supplemented by Google Scholar, publisher-platform searches, backward reference screening, and forward citation tracking. Search performance was evaluated using eight known eligible benchmark studies, and the retrieval pathways of all 23 included studies were audited. A second author independently reviewed the search concepts, synonyms, Boolean operators, phrase searching, field restrictions, and database-specific syntax.

The complete database-specific search strings, search dates, search fields, filters, and numbers of retrieved records are provided in Supplementary Table S4. Google Scholar and publisher-platform searches are also reported with the search terms, dates, and screening limits applied. Records were exported to Mendeley Reference Manager [version 2.148.0], and duplicate records were removed before screening.

### 2.7. Inclusion and Exclusion Criteria

Studies were excluded when lysine and/or methionine supplementation was the sole experimental intervention but remained eligible when these amino acids balanced the basal diet, formed part of a broader strategy, or served as comparators for an additional intervention. Studies evaluating ingredient replacement alone were excluded, as were nonfeeding studies, records without clear diet formulation or fishmeal information, and studies without relevant biological outcomes. Reviews, meta-analyses, book chapters, theses, conference abstracts, editorials, technical reports, and other nonpeer-reviewed publications were excluded. Fish-oil replacement studies were excluded unless they were directly integrated into a low-fishmeal formulation.

### 2.8. Study Selection

After duplicate removal, study selection was conducted in two stages using the predefined eligibility criteria. First, one reviewer screened the titles and abstracts to exclude clearly irrelevant records. Second, the same reviewer assessed the full texts of potentially eligible articles and recorded the reasons for exclusion. A second reviewer subsequently verified all full-text eligibility decisions and a random 20% sample of the title-and-abstract screening decisions, as well as the recorded reasons for exclusion and the final set of included studies. Any disagreements were resolved through discussion and consensus between the reviewers. No automated screening tools were used.

**Figure 1:**
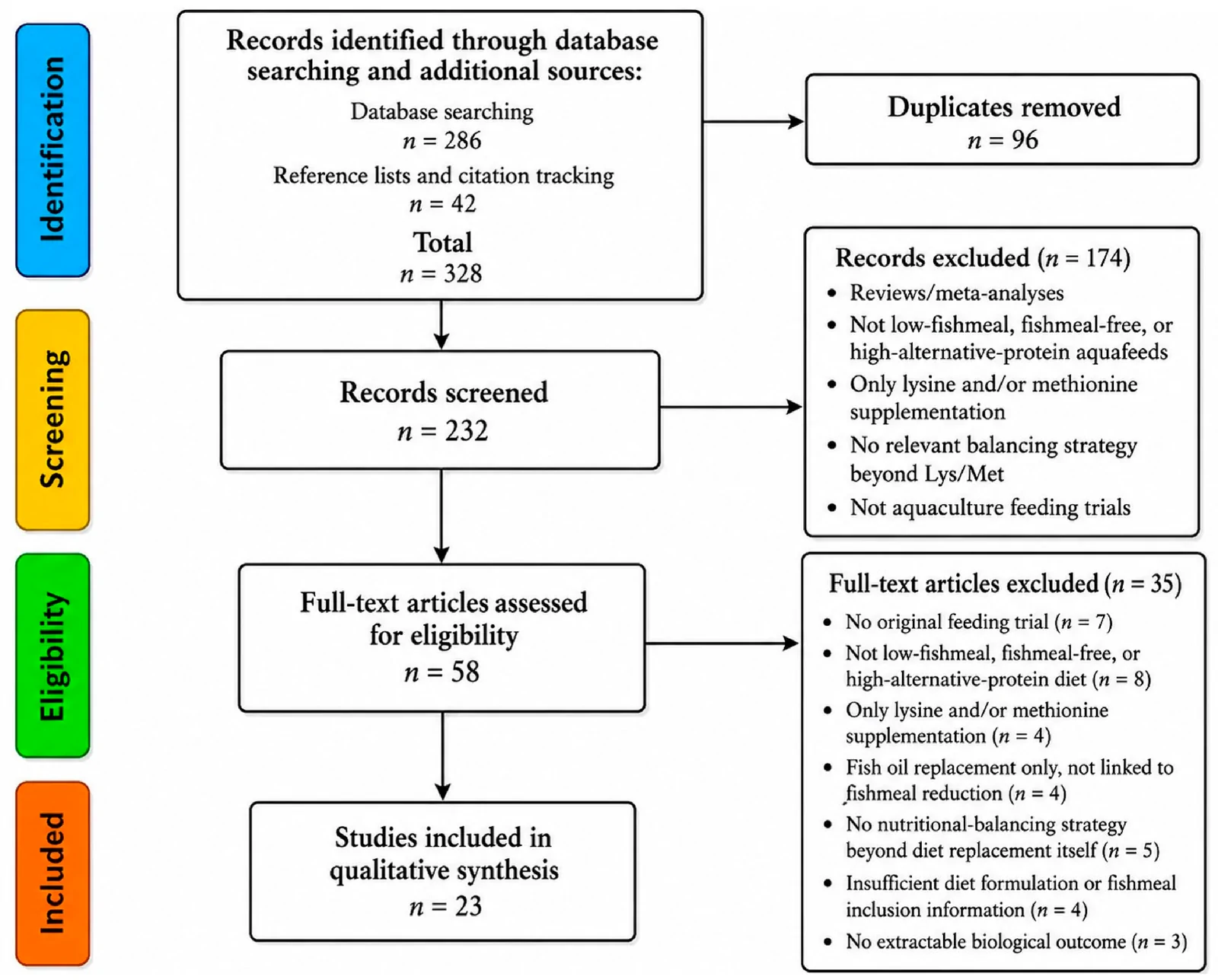
PRISMA flow diagram of study identification, screening, eligibility assessment, and inclusion for the systematic review.

Among the 328 records identified through database searches and additional sources, 96 duplicates were removed, leaving 232 records for title-and-abstract screening. After 174 records were excluded, 58 full-text articles were assessed for eligibility. Of these, 35 were excluded because they did not meet the predefined eligibility criteria, resulting in 23 studies being included in the qualitative synthesis. The complete study-selection process and reasons for full-text exclusion are presented in Figure 1.

### 2.9. Data Extraction

A standardized extraction form was developed before final data extraction and used to collect information on study and animal characteristics, diet formulation and fishmeal inclusion, alternative-protein matrices, intervention type and dose, experimental design, culture conditions, statistical methods, and reported outcomes. Extracted diet-level information included proximate composition, amino acid profiles, phosphorus or mineral composition, replacement ingredients, and other relevant nutrient characteristics where reported.

Outcome data were extracted for production performance, feed and nutrient utilization, physiological and metabolic status, intestinal condition, microbiota, immune and antioxidant responses, disease and stress resilience, product quality, environmental performance, and economic outcomes. The replicate tank or cage was treated as the experimental unit where applicable. Missing or insufficiently reported information was recorded as not reported and was not inferred from unrelated studies or external sources.

One reviewer conducted the primary data extraction, and a second reviewer subsequently checked the extracted study characteristics, diet information, outcome data, experimental-unit assignments, and effect-size inputs against the original articles. Disagreements or unclear information were resolved through discussion and consensus between the reviewers. When numerical results were available only in figures, values were obtained using WebPlotDigitizer, version 5 (Ankit Rohatgi, Pacifica, CA, USA) and checked against the original graphical presentation by the second reviewer. When reliable numerical extraction was not possible, the direction and nature of the reported response were recorded qualitatively. No automated data-extraction tools were used. The complete study-level extraction matrix is provided in Supplementary Table S1.

### 2.10. Classification of Nutritional Balance Strategies

Studies were classified into eight categories according to their primary intervention objective: essential amino acids beyond lysine and methionine; functional amino acids and nonprotein nitrogenous compounds; palatability and feed-intake enhancers; digestibility and enzyme strategies; mineral and phosphorus balancing; vitamin and antioxidant support; lipid-associated nutrient balancing; and gut health or microbiome-targeted strategies. Multifunctional studies were assigned to one primary category according to the dominant experimental objective for study-count and effect-size summaries. Secondary mechanisms and outcome domains were recorded separately in Supplementary Table S1. This approach prevented the same study from being counted repeatedly across primary strategy categories while preserving information on multifunctional effects.

### 2.11. Quality Assessment

Methodological quality and reporting completeness were assessed across the following domains: animal and experimental design; dietary treatment structure; replication and identification of the experimental unit; randomization; diet formulation and analytical verification; characterization of fishmeal replacement and alternative ingredients; trial duration; culture conditions; statistical analysis; outcome reporting; and availability of variance data.

Each domain was rated as low concern, some concern, high concern, or insufficient reporting. A low-concern judgment indicated that the relevant design or reporting information was sufficiently described and appropriate for interpretation. Some concern indicated a limitation that reduced confidence but did not invalidate the study. High concern indicated a design, analysis, or reporting limitation that could materially affect interpretation. Insufficient reporting was assigned when the available information did not permit a reliable judgment.

One reviewer conducted the initial methodological-quality and reporting assessment, and a second reviewer subsequently verified the domain-level judgments and supporting information against the original articles. Any differences were resolved through discussion and consensus between the reviewers. Studies were not excluded solely because of methodological-quality concerns unless essential information on the diet, fishmeal inclusion, comparator, experimental unit, or relevant outcomes was unavailable. Detailed study-level judgments and supporting explanations are provided in Supplementary Table S2.

### 2.12. Causal Pathway Mapping and Evidence Classification

To distinguish mechanistically supported nutritional strategies from associations based only on outcome improvement, each included study was mapped across six predefined causal elements: (1) the reduction in fishmeal or inclusion of an alternative ingredient; (2) the resulting nutrient or functional gap; (3) the biological mechanism expected to be affected; (4) the impaired biological or production outcome observed in the non-supplemented low-fishmeal treatment; (5) the nutritional correction strategy applied; and (6) the extent to which the impaired outcome was restored relative to the non-supplemented low-fishmeal treatment and, where available, the fishmeal-based positive control.

Evidence was classified as mechanistically supported when the diet-induced nutrient or functional gap, a corresponding mechanism-aligned intermediate response, the impaired outcome, and subsequent restoration were demonstrated within the same experimental framework. Evidence was classified as partially supported when restoration was demonstrated but the initial gap or one or more intermediate mechanisms were not directly measured. Evidence was classified as associative when an improvement was reported without direct confirmation of the proposed nutritional limitation or biological mechanism.

The classification also considered comparator structure, tank- or cage-level replication, methodological quality, availability of mechanism-specific endpoints, and consistency between intermediate and final outcomes. These classifications represent the completeness of the proposed biological pathway and should not be interpreted as formal risk-of-bias or GRADE certainty ratings. Causal attribution was considered limited for multicomponent interventions unless the contribution of individual components was separated experimentally.

### 2.13. Data Synthesis

The primary synthesis was conducted as a structured narrative comparison organized by intervention category, species group, diet matrix, and outcome domain. For each nutritional-balancing strategy, the evidence was interpreted according to the number of contributing studies and represented species, the direction and consistency of the reported responses, the precision of estimable effect sizes, methodological quality and reporting completeness, the appropriateness of comparator diets and identification of experimental units, the completeness of the proposed causal pathway, and the extent to which findings were replicated across diet matrices, doses, life stages, and culture conditions.

Responses were classified as consistently favorable, mixed, neutral, unfavorable, or insufficient. The overall review-level interpretation described each strategy as having relatively coherent evidence, promising but limited evidence, mixed or context-dependent evidence, preliminary evidence, or insufficient support. These classifications were descriptive and were not intended to represent formal GRADE certainty ratings.

Statistical pooling was not performed because the included studies differed substantially in species, life stage, diet matrix, intervention type, dose, comparator, trial duration, culture condition, and outcome definition.

### 2.14. Study-Level Effect Size Estimation and Graphical Presentation

Where group means, variability estimates, and experimental-unit sample sizes were available, standardized mean differences were calculated as Hedges’ *g* with 95% confidence intervals using R version 4.6.1 (R Core Team, Vienna, Austria) and the metafor package. Effect sizes and their corresponding sampling variances were calculated using the escalc function with small-sample bias correction. Standard errors were converted to standard deviations using SD = SE × √*n*.

The replicate tank or cage was treated as the experimental unit. Individual animals sampled from the same tank or cage were not treated as independent experimental units unless independent tank- or cage-level summary data were available. When studies reported sample sizes for both replicate tanks or cages and individual animals, the number of replicate tanks or cages was used for effect-size estimation.

For outcomes with an unambiguous favorable direction, effect directions were standardized. The signs of the feed conversion ratio and other outcomes for which lower values indicated improvement were reversed so that positive values consistently represented a more favorable intervention response. Outcomes without a universally favorable direction were interpreted according to their biological context.

For graded-dose studies, the highest tested intervention dose and, where applicable, the greatest fishmeal-replacement level were selected as the representative comparison for the descriptive forest plots to apply a consistent graphical rule across studies. This selection was made solely for descriptive presentation and does not indicate that the highest tested dose was biologically optimal, commercially recommended, or superior to all other tested doses. All extractable dose-level data, effect-size calculations, and selection notes are reported in Supplementary Table S3.

Effect estimates were displayed descriptively as forest plots generated using R. No pooled estimates, study weights, heterogeneity statistics, overall effect tests, or formal statistical comparisons among nutritional-balancing strategies were calculated. Outcomes were classified as non-estimable when the required group means, variability estimates, or experimental-unit sample sizes were unavailable.

### 2.15. Exploratory Subgroup Analysis

Post hoc exploratory subgroups were defined by taxonomic or production group, reported life stage, and primary diet matrix. The 24 representative performance comparisons selected for the descriptive forest plots were summarized within these groups, while the two studies without estimable effects remained in the study-level classification. Study counts, comparison counts, effect size ranges, and the number of confidence intervals excluding zero were described without subgroup pooling, formal interaction tests, or meta-regression because the subgroups were small and the intervention type, diet matrix, dose, fishmeal level, and outcome definition were confounded. The detailed assignments are provided in Supplementary Table S5. The subgroup findings were used only to describe the distribution of the evidence and were not incorporated as independent evidence of taxonomic, life-stage, or diet-matrix effect modification.

## 3. Results

### 3.1. Study Selection Based on PRISMA 2020

The search identified 328 records, including 286 from scientific databases and 42 from additional sources. After 96 duplicates were removed, 232 records were screened, 174 of which were excluded. Among the 58 full-text articles assessed, 35 were excluded because they lacked an original feeding trial (*n* = 7), an eligible low-fishmeal or alternative-protein diet model (*n* = 8), an intervention beyond lysine and/or methionine alone (*n* = 4), a direct link between fish-oil replacement and fishmeal reduction (*n* = 4), a nutritional-balancing strategy beyond ingredient replacement (*n* = 5), sufficient diet information (*n* = 4), or extractable biological outcomes (*n* = 3). The updated search, which was completed on 18 June 2026, identified no additional eligible studies. Consequently, 23 studies were included in the qualitative synthesis. Owing to substantial heterogeneity in species, diets, interventions, and outcomes, the results were synthesized narratively rather than pooled quantitatively. The selection process is shown in Figure 1.

### 3.2. General Characteristics of Included Studies

Twenty-three studies were published between 2004 and 2024 and represented 11 aquatic species or experimental taxa. Most involved juvenile finfish, particularly marine non-salmonid species, whereas crustaceans were represented mainly by Pacific white shrimp [33,34,35,36,37,38,39]. The feeding trials lasted 4–20 weeks, and the reported percentage of fishmeal included ranged from 0% to 64%. The main diet matrices included soybean meal, soy protein concentrate, canola meal, fermented plant ingredients, mixed plant-protein formulations, fishmeal-free diets, low-phosphorus diets, and practical mixed-protein formulations. Several studies used both a fishmeal-based positive control and a non-supplemented low-fishmeal negative control, enabling direct assessment of outcome restoration.

Most studies used completely randomized designs with triplicate tanks per treatment. Growth, the feed conversion ratio, feed intake, survival, protein efficiency, digestibility, and whole-body composition were reported most frequently. Fewer studies assessed blood biochemistry, immune or antioxidant responses, intestinal morphology, the microbiota, disease resistance, stress tolerance, or molecular endpoints. Rendered animal, insect, gluten, microbial, and algal matrices were insufficiently represented for ingredient-specific synthesis. Table 2 summarizes the study characteristics and strategy groups.

#### Methodological Quality and Reporting Limitations

Methodological quality and reporting completeness varied among the 23 included studies. The principal concerns identified in Supplementary Table S2 involved limited tank- or cage-level replication, incomplete reporting of randomization and allocation procedures, insufficient identification of the experimental unit, incomplete analytical characterization of experimental diets, and limited reporting of variance data or mechanism-specific outcomes. Diet formulation and fishmeal inclusion were generally reported sufficiently for eligibility assessment, but information on digestible amino acids, available phosphorus, ingredient quality, antinutritional factors, additive stability, and nutrient retention was frequently incomplete.

These limitations did not affect all studies or outcome domains equally. However, they reduced confidence in broad conclusions when a strategy was represented by few studies, when estimates were based on two or three replicate tanks per treatment, or when the proposed nutrient gap and intermediate mechanism were not measured directly. Methodological-quality judgments were therefore considered together with response consistency, statistical precision, comparator structure, species and diet coverage, and causal-pathway completeness when interpreting the strategy-level evidence. 

### 3.3. Overview of Nutritional Balance Strategies

**Figure 2:**
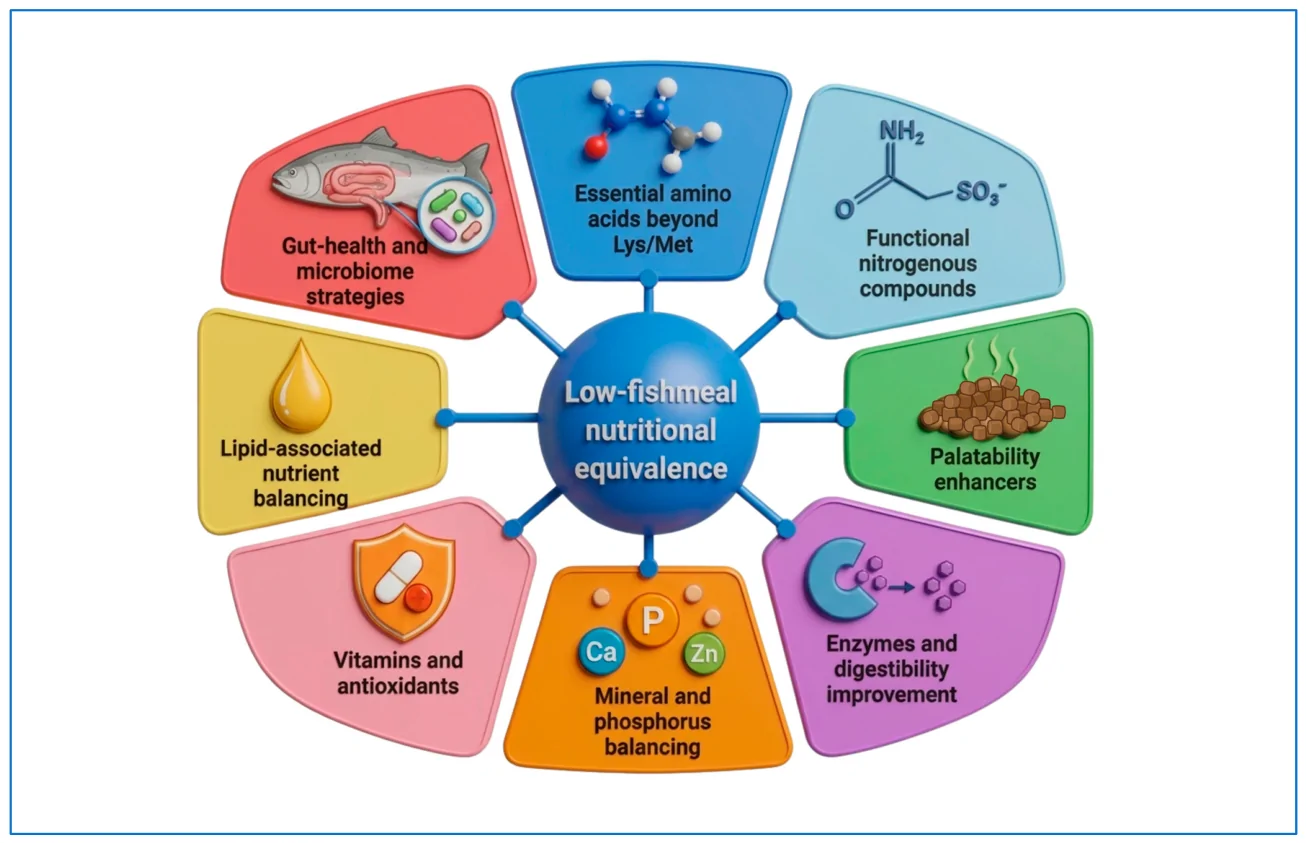
Eight-category framework of nutritional-balancing strategies identified in the included studies. Studies have been classified according to the primary function of the intervention, although several strategies may act through overlapping biological pathways.

The included studies were classified into eight categories according to the primary function of each intervention: essential amino acids beyond lysine and methionine; functional nitrogenous compounds; palatability enhancers; enzymes and digestibility strategies; mineral and phosphorus balance; vitamin and antioxidant support; lipid-associated nutrient balance; and gut health or microbiome-targeted strategies. Because some interventions act through multiple pathways, classification was based on the dominant experimental objective (Figure 2).

The evidence varies among categories. Amino acids other than lysine and methionine affect metabolic or immune responses, with inconsistent growth effects [35,36,40]. Phytase generally improves phosphorus utilization, although growth responses depend on the diet matrix [34,37]. Hydrolysates and attractant-rich ingredients improved feed utilization and selected health outcomes [41], whereas fermented soybean meal, yeast hydrolysate, and probiotics produced context-dependent intestinal, immune, and disease resistance benefits [38,39,42]. Taurine showed favorable but imprecise responses in a limited marine-finfish evidence base, whereas phytase provided clearer mechanism-aligned evidence. However, evidence for histidine, threonine, tryptophan, hydroxyproline, vitamin-antioxidant strategies, and lipid-associated nutrients remains limited. The strategy-level findings, including study counts, Hedges’ *g* ranges, and confidence intervals, are summarized in Table 3 and detailed in Section 3.4.1, Section 3.4.2, Section 3.4.3, Section 3.4.4, Section 3.4.5, Section 3.4.6, Section 3.4.7 and Section 3.4.8.

#### Cross-Strategy Consistency, Precision, and Strength of Evidence

The strength of evidence differed substantially among nutritional-balancing strategies. Of the 24 representative comparisons from 21 estimable studies, eight had 95% confidence intervals excluding zero in favor of the intervention, whereas 16 crossed the null. These estimates were interpreted descriptively because several comparisons originated from multi-arm studies and because interventions, species, diet matrices, doses, comparators, and outcomes were not sufficiently comparable for statistical pooling.

Phytase-based phosphorus balancing provided the most coherent mechanism-aligned evidence. Three of five representative comparisons had confidence intervals excluding zero, and the observed responses were consistent with improved phosphorus availability, retention, mineralization, or reduced phosphorus loss in phytate-rich or phosphorus-limited diets. Protein hydrolysates and attractant-rich ingredients also showed a consistently favorable performance-related pattern, with all three representative comparisons having confidence intervals excluding zero. However, the hydrolysate evidence remained limited to three studies conducted in different species and diet matrices.

Taurine responses were directionally favorable but imprecise, as none of the three representative comparisons had confidence intervals excluding zero. The evidence was also restricted to a small number of marine finfish species. Essential amino acids beyond lysine and methionine and gut-targeted strategies produced mixed performance responses, although some studies reported mechanism-specific metabolic, immune, intestinal, or disease-resistance benefits. Evidence for hydroxyproline, lipid-source balancing, and integrated strategies under environmental stress remained preliminary because each category contained few studies.

Accordingly, the current evidence is strongest for the mechanism-matched use of phytase in diets with limited phosphorus availability and is consistently favorable but less generalizable for protein hydrolysates. Evidence for taurine and other functional strategies supports context-specific testing rather than universal supplementation. These conclusions reflect response consistency, statistical precision, methodological quality, species coverage, diet context, and mechanistic alignment rather than effect magnitude alone.

### 3.4. Nutritional Balancing Strategies Beyond Lysine and Methionine

The six-stage causal framework is as follows: (1) fishmeal reduction or alternative-ingredient inclusion; (2) development of a nutrient or functional gap; (3) disruption of the corresponding biological mechanism; (4) impaired production or health outcomes; (5) targeted nutritional correction; and (6) restoration of mechanism-specific or final outcomes. These pathways include altered amino acid and nitrogenous compound supplies; reduced palatability; impaired nutrient and mineral availability; disrupted lipid metabolism; and compromised intestinal, microbial, and immune functions [8,9,10,19,22,24,25,26,27,28,29,30,56,57,58,59,60,61].

**Figure 3:**
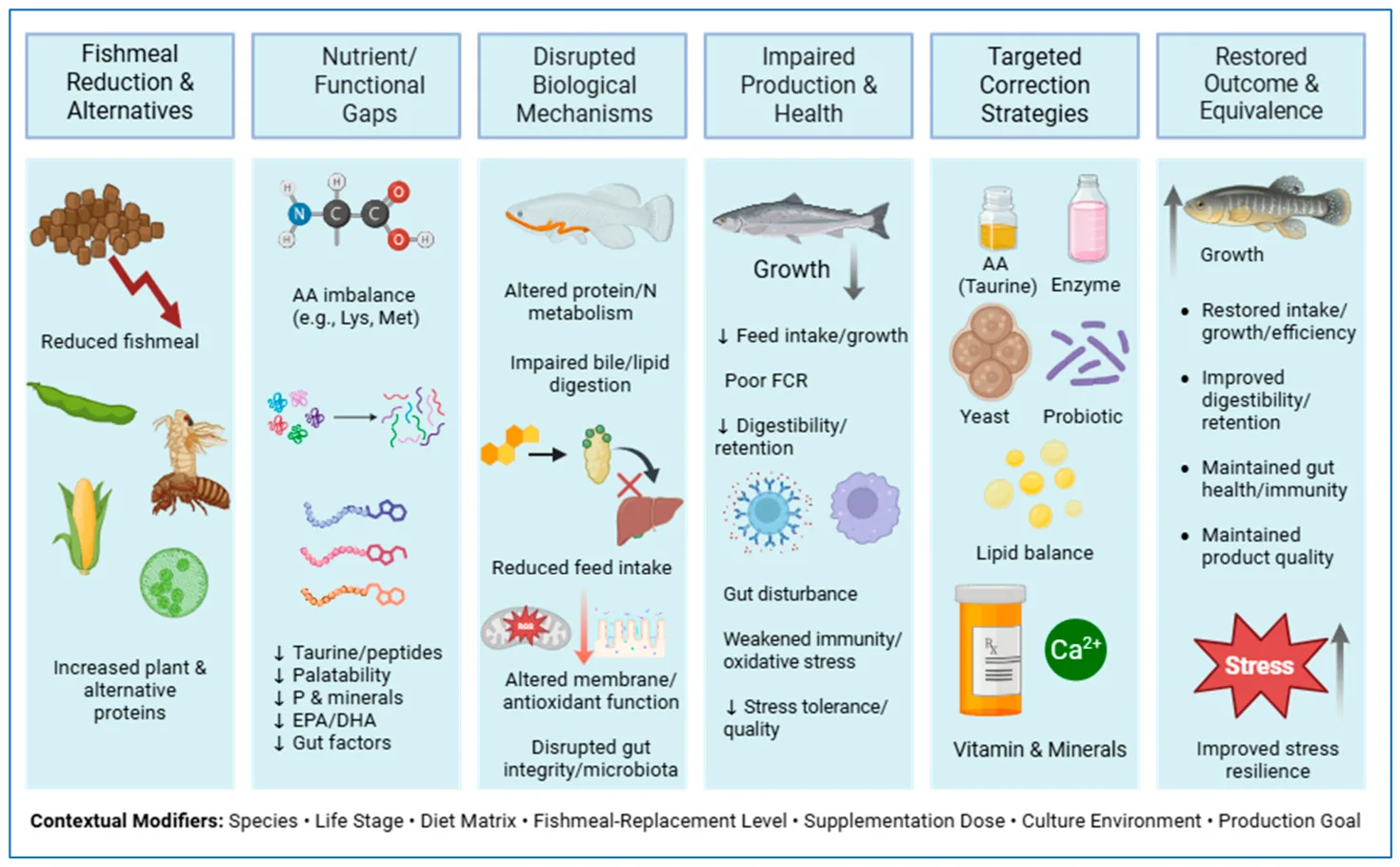
Causal framework linking fishmeal reduction to targeted nutritional correction and outcome restoration. Fishmeal reduction or alternative-ingredient inclusion may create nutrient or functional gaps that disrupt biological mechanisms and impair production, physiological, health, product-quality, or environmental outcomes. Targeted strategies are applied to address these limitations, and restoration is evaluated using mechanism-aligned intermediate and final outcomes. Causal support depends on whether the nutritional gap, affected mechanism, impaired outcome, and restoration were measured within the same experimental framework. Species, life stage, diet matrix, replacement level, dose, culture environment, and production objective act as contextual modifiers. The framework illustrates the analytical pathway and does not imply that all links are equally supported. Strategy-specific causal-support classifications are presented in Table 4.

Causal support was considered stronger when diet-induced limitations, intermediate mechanisms, impaired outcomes, and subsequent restoration were demonstrated within the same experiment. Pathways with unmeasured links were classified as partially supported or associative. Figure 3 illustrates the framework, and Table 4 summarizes the pathway-specific evidence and strength of causal support.

The major nutritional gaps associated with fishmeal reduction, their mechanistic consequences, corresponding balancing strategies, and recommended validation endpoints are summarized in Table 4.

Causal support varied among strategies (Table 4). Mineral and phosphorus balance had the strongest support because phosphorus limitation, digestibility, retention, fecal loss, and growth were evaluated within aligned experimental models [34,37,49,50,51]. Taurine, hydroxyproline, and gut-targeted strategies have provided partial to mechanistic support, although key intermediate mechanisms have been inconsistently measured [33,43,44,45,46,47,48]. Palatability, digestibility, and essential amino acid strategies generally lack direct evidence for one or more causal links, whereas vitamin-antioxidant and lipid-associated strategies remain largely associative or hypothesis-generating.

#### 3.4.1. Essential Amino Acids Beyond Lysine and Methionine

Evidence for essential amino acids beyond lysine and methionine was limited and mixed. Histidine supplementation in yellowtail altered feed intake and tissue-free amino acid profiles without consistently improving growth [35]. In fishmeal-free diets for gilthead seabream, tryptophan transiently enhanced immune responses, whereas histidine, threonine, and taurine affected selected immune indices without uniform production benefits [36,40]. Branched-chain amino acid imbalance also altered amino acid metabolism and growth-related responses in turbot [46]. These findings indicate that additional amino acid balancing may produce clearer metabolic or immune effects than growth responses and should be based on a demonstrated imbalance in the complete diet. However, the small evidence base, incomplete reporting of digestible amino acid ratios, and limited dose–response information prevent general supplementation recommendations.

#### 3.4.2. Functional Amino Acids and Nonprotein Nitrogenous Compounds

Taurine showed generally favorable but context-dependent responses in red seabream, olive flounder, European seabass, and gilthead seabream. Reported benefits included improvements in growth, feed utilization, hematological status, immunity, and low-temperature performance [33,43,44,45]. These responses suggest that taurine may be most relevant in marine finfish diets containing high levels of plant protein or reduced marine ingredients, particularly when taurine-dependent metabolism or stress resilience is compromised. Nevertheless, the evidence remained limited in precision, species coverage, and direct measurement of intermediate mechanisms. Hydroxyproline also improved collagen synthesis, muscle development, texture, or growth in triploid crucian carp and Pacific white shrimp [47,48], but evidence was restricted to two studies. These findings are consistent with the recognized roles of glycine, proline, and hydroxyproline in collagen synthesis and animal growth [62]. Glutamate and glutamine also have important nutritional and metabolic roles in fish [63]. Taurine and hydroxyproline should therefore be considered promising, mechanism-specific strategies rather than universally applicable supplements.

#### 3.4.3. Palatability and Feed-Intake Balance

Protein hydrolysates and attractant-rich ingredients showed the most consistently favorable performance-related pattern among the reviewed strategies. Shrimp protein hydrolysate and krill meal improved growth, feed utilization, digestibility, immune responses, antioxidant activity, and hepatic IGF-I expression in red seabream [41]. Protein hydrolysates also improved growth or health-related outcomes in olive flounder [52] and supported plant-protein diets in Atlantic salmon [53]. These benefits may reflect improved palatability, feed intake, digestibility, peptide availability, or combined effects. Marine side streams are recognized as sources of functional and bioactive peptides [64]. Enzymatic fish protein hydrolysates have been reviewed specifically for their application in finfish aquaculture [65]. Bioactive peptides derived from marine by-products have also been reviewed in relation to their production, health benefits, and potential biomedical applications [66]. However, only three studies were available, and they differed in species, hydrolysate source, product composition, and diet matrix. The evidence is therefore consistently favorable but insufficient to identify a universal inclusion level or a single dominant mechanism.

#### 3.4.4. Digestibility Improvement and Enzyme Supplementation

Digestibility and enzyme-based strategies produced generally favorable but diet-dependent responses. Phytase improved phosphorus digestibility in olive flounder without affecting growth [34], whereas it improved growth, feed conversion, phosphorus utilization, and fecal phosphorus output in Atlantic salmon [37]. Combined phytase and protease supplementation also improved growth and nutrient utilization in tilapia fed low-phosphorus, fishmeal-free diets [49]. These findings indicate that enzyme efficacy depends on whether the relevant substrate or nutritional constraint is present in the basal diet. Other digestibility-oriented approaches were represented by isolated models: fermented soybean meal affected antioxidant and lysozyme responses in whiteleg shrimp [38], whereas tributyrin and alanyl-glutamine alleviated intestinal impairment in largemouth bass fed high-soybean-meal diets [54]. Accordingly, enzyme and processing strategies should be matched to the specific antinutritional or digestive limitation of the complete diet.

#### 3.4.5. Mineral and Phosphorus Balance

Mineral and phosphorus balancing provided the clearest mechanism-aligned evidence in the review. In plant-rich diets, total phosphorus may overestimate biologically available phosphorus because a substantial proportion is bound to phytate. Across studies, phytase generally improved phosphorus digestibility, retention, mineral utilization, or fecal phosphorus output, although growth responses were not uniform [34,37,49,50,51]. For example, phytase improved growth and phosphorus utilization in Atlantic salmon [37] but increased phosphorus digestibility without improving growth in olive flounder [34]. These findings show that phytase should be evaluated primarily through available phosphorus, retention, mineralization, and discharge rather than as a universal growth promoter. Despite relatively coherent mechanistic support, optimal responses remain dependent on species, phytate concentration, phosphorus status, enzyme dose, application method, and feed-processing conditions.

#### 3.4.6. Vitamin, Antioxidant, and Lipid-Associated Nutrient Balance

Evidence for vitamin-, antioxidant-, and lipid-associated nutrient balancing was sparse. Changes in superoxide dismutase, catalase, glutathione peroxidase, lysozyme, or related responses were reported following supplementation with taurine, hydrolysates, fermented ingredients, or yeast-derived products [38,39,41,43]. However, these multifunctional interventions did not permit the antioxidant effects to be attributed to correction of a specific vitamin or antioxidant deficiency. Lipid-related evidence was also limited: taurine affected growth and organoleptic traits in European seabass [44], alternative lipid sources were evaluated in gilthead seabream [45], and hydroxyproline influenced collagen synthesis and muscle texture [47,48]. The available evidence therefore remains insufficient for general recommendations concerning vitamins, phospholipids, cholesterol, or individual antioxidant nutrients. Future studies should directly characterize dietary and tissue lipid composition and mechanism-specific metabolic outcomes.

#### 3.4.7. Gut Health and Microbiome-Targeted Strategies

Gut and microbiome-targeted strategies produced mixed growth responses but more frequent intestinal, immune, or disease-related benefits. Multistrain probiotics improved nutrient utilization, immunity, and gut microbiota in olive flounder [42]. Yeast hydrolysate improved growth, intestinal morphology, and resistance to *Vibrio parahaemolyticus* in whiteleg shrimp [39], whereas fermented soybean meal affected antioxidant and immune responses [38]. Tributyrin and alanyl-glutamine also alleviated intestinal impairment in largemouth bass fed high-soybean-meal diets [54]. Previous reviews have highlighted the immunomodulatory roles of probiotics in fish [67], the interactions between gut microbiota and innate immunity [68], and the potential application of probiotics for disease control in aquaculture [69]. In shrimp, the gut microbiome has likewise been implicated in host health and disease [70]. Yeast-derived products may also contribute to aquafeed functionality, with β-glucans acting as immunomodulators [71] and whole yeast serving as a protein-rich feed ingredient [72].

These strategies may therefore be most relevant when alternative ingredients create a measurable intestinal or microbial challenge. However, evidence was heterogeneous in product type, microbial strain, dose, diet matrix, culture condition, and challenge model. Current findings support context-specific gut and health benefits but do not identify a universally effective additive or supplementation protocol.

#### 3.4.8. Integrated Nutritional Strategies Under Environmental Stress

Evidence for integrated strategies under environmental stress was favorable but preliminary. Taurine improved performance or feed utilization in red seabream and olive flounder under low-temperature conditions [33,43]. A multicomponent strategy containing betaine, krill meal, lecithin, macroalgae, and taurine also supported gilthead seabream performance at low temperature [55]. These findings suggest that environmental stress may expose nutritional limitations that remain undetected under optimal rearing conditions and may increase the value of palatability, metabolic, lipid-associated, or immune-supporting nutrients. However, the small number of studies and the multicomponent nature of the interventions limit causal attribution and generalization. Further factorial or component-removal studies are required to distinguish the effects of individual ingredients and determine whether similar benefits occur under heat, salinity, hypoxia, handling, transport, or disease stress.

#### 3.4.9. Overall Strategy-Level Interpretation

The strategy-level evidence supports targeted correction rather than universal supplementation. Phytase had the most coherent mechanism-aligned support when phosphorus availability was limited, whereas protein hydrolysates showed a consistently favorable but smaller evidence base. Taurine was promising but imprecise and restricted mainly to marine finfish. Evidence for the remaining strategies was mixed, preliminary, or strongly dependent on the diet matrix, species, product, and outcome evaluated.

Accordingly, no additive should be selected solely according to the percentage of fishmeal replaced. The intervention should address a demonstrated nutritional or functional limitation, and its efficacy should be confirmed using production outcomes together with relevant mechanism-specific, health, product-quality, environmental, or economic endpoints.

### 3.5. Biological Outcomes of Nutritional Balance

Outcome domains were interpreted according to their biological relevance. Growth and FCR integrate feed intake, digestibility, and metabolism; digestibility and retention indicate nutrient availability; blood indices reflect metabolic status; gut morphology and microbiota indicate intestinal compatibility; immune and antioxidant markers reflect physiological resilience; and product-quality and sustainability outcomes indicate practical and environmental performance [22,24,29,30].

Across the included studies, nutritional balance affected production, nutrient utilization, metabolism, intestinal health, immunity, stress resilience, and sustainability. Therefore, low-fishmeal diets should not be evaluated solely by body weight or fishmeal replacement level but through integrated production, physiological, health, and environmental endpoints. The recommended validation domains are summarized in Table 5.

**Figure 4:**
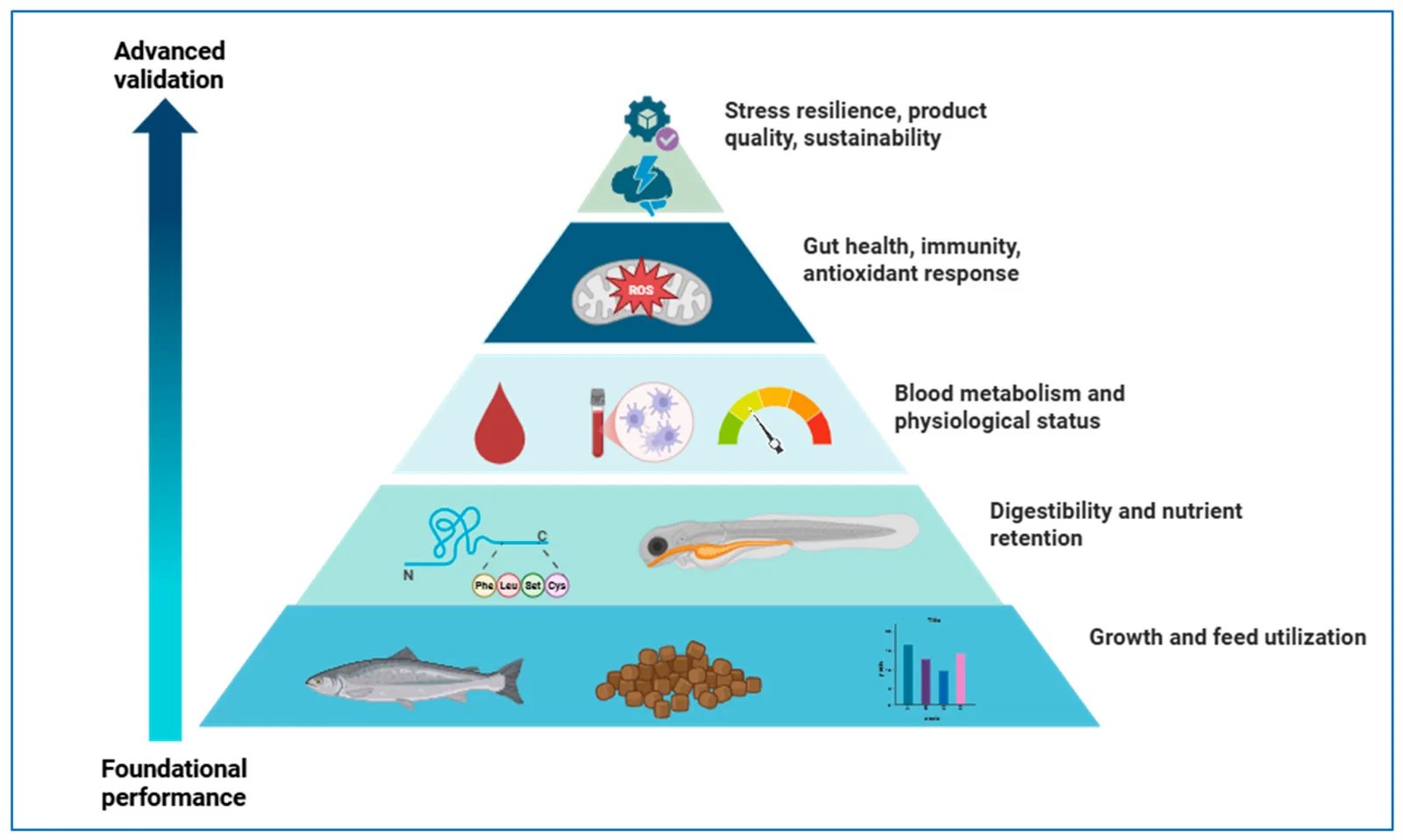
Outcome-validation framework for low-fishmeal feeds. The framework summarizes the breadth of outcome domains that may be required to evaluate nutritional equivalence, progressing from foundational production and feed-utilization outcomes to nutrient digestibility and retention, physiological and metabolic status, intestinal condition, immune and antioxidant responses, and advanced validation under stress, disease, product-quality, environmental, and economic conditions. The levels represent increasing breadth of biological and practical validation rather than a formal ranking of evidence certainty or endpoint importance.

The hierarchical relationships among these validation domains are illustrated in Figure 4. The pyramid emphasizes that low-fishmeal aquafeed evaluation should progress from foundational production responses toward more advanced indicators of nutritional equivalence.

#### 3.5.1. Growth Performance and Feed Utilization

Growth performance and feed utilization were the most frequently reported outcomes across the included studies. Taurine supplementation improved the growth and feed utilization of red seabream fed soybean meal-based low-fishmeal diets during the low-temperature season, and taurine also improved the feed utilization of juvenile olive flounder under low-temperature conditions [33,43]. Protein hydrolysates, shrimp hydrolysate, krill meal, probiotics, fermented soybean meal, and yeast hydrolysate also improved growth or feed efficiency in selected low-fishmeal models [38,39,41,42,52]. However, amino acids other than lysine and methionine do not uniformly improve growth, even when they affect immune or metabolic responses [35,36,40].

Overall, growth responses are useful for identifying whether a balancing strategy improves production performance, but they do not always reveal the mechanism involved. Similar growth improvements could result from better feed intake, improved digestibility, restored phosphorus availability, enhanced gut function, or improved physiological resilience. Therefore, growth and FCR should be interpreted together with feed intake, digestibility, immune status, gut morphology, and nutrient-retention endpoints.

#### 3.5.2. Digestibility and Nutrient Retention

Digestibility and retention outcomes were less frequently reported than growth, but they provided stronger evidence of nutrient-use correction. Phytase supplementation improved apparent phosphorus digestibility in olive flounder, and in Atlantic salmon, it improved phosphorus digestibility, phosphorus retention, and fecal phosphorus loss in addition to growth and FCR [34,37]. Phytase and protease also improved nutrient utilization in tilapia fed low-phosphorus and fishmeal-free diets [49]. Shrimp protein hydrolysate increased protein digestibility in red seabream fed a low-fishmeal diet [41].

These findings indicate that digestibility data are essential for distinguishing crude nutrient equivalence from functional nutrient equivalence. A low-fishmeal diet may meet formulated crude protein or total phosphorus levels but still provides relatively low levels of digestible amino acids or available phosphorus. Across the included studies, incomplete reporting of amino acid digestibility, available phosphorus, mineral retention, and nitrogen or phosphorus outputs limited the possibility of quantitative comparison.

#### 3.5.3. Physiological, Immune, Antioxidant, and Gut Health Outcomes

Health-related endpoints revealed that responses are not always reflected in growth. Taurine improved hematological and innate immune indices in olive flounder [43] and enhanced feed utilization with numerical improvements in innate immunity in red seabream [33]. Shrimp protein hydrolysate and krill meal also improved hematological, immune, and antioxidant responses, with shrimp protein hydrolysate increasing catalase and glutathione peroxidase activities [41].

Gut-targeted strategies have shown similar benefits. Multiple strains of probiotics improved nutrient utilization, immunity, and the gut microbiota in olive flounder [42], whereas yeast hydrolysate enhanced the intestinal morphology and resistance to *Vibrio parahaemolyticus* in whiteleg shrimp [39]. Fermented soybean meal also affected antioxidant and immune responses in shrimp [38].

#### 3.5.4. Stress Resilience, Product Quality, and Sustainability Outcomes

Stress-related outcomes were assessed less frequently than growth. Taurine improved performance and immune responses in red seabream and olive flounder under low-temperature conditions [33,43], whereas an integrated nutritional strategy supported gilthead seabream performance at low temperature [55]. Yeast hydrolysate also increased resistance to Vibrio parahaemolyticus in whiteleg shrimp [39].

The evidence for product quality and sustainability is limited. Hydroxyproline improved collagen synthesis, myofiber development, and muscle texture [47,48], whereas taurine studies have addressed product quality and alternative lipid sources [44,45]. Phytase provides the clearest environmental benefit by improving phosphorus utilization and reducing fecal phosphorus output in Atlantic salmon [37]. The economic, life-cycle, sensory, fatty acid, and broader nutrient-waste outcomes have rarely been reported.

#### 3.5.5. Implications of Outcome Selection for Evidence Interpretation

The consistency of evidence depended partly on whether the outcomes matched the intended function of the intervention. Phytase studies most clearly demonstrated this principle because phosphorus digestibility, retention, mineralization, and discharge outcomes directly tested the proposed mechanism. By contrast, several taurine, amino acid, hydrolysate, and gut-targeted studies measured growth without consistently measuring the nutrient gap or intermediate biological pathway.

An unchanged growth response should therefore not be interpreted automatically as an absence of biological value when mechanism-specific nutrient, physiological, intestinal, immune, or environmental outcomes improve. Conversely, improved growth without measurements of feed intake, digestibility, nutrient retention, or relevant physiological responses cannot establish the responsible mechanism. Future studies should combine standardized core production outcomes with intervention-specific endpoints selected before the feeding trial.

#### 3.5.6. Study-Level Effect Sizes and Precision

Effect sizes and 95% confidence intervals were estimated for 21 of the 23 studies, yielding 24 intervention–comparator comparisons because two multiarm studies contributed multiple estimates. Comparisons sharing a control were presented descriptively but were not treated as independent or pooled.

**Figure 5:**
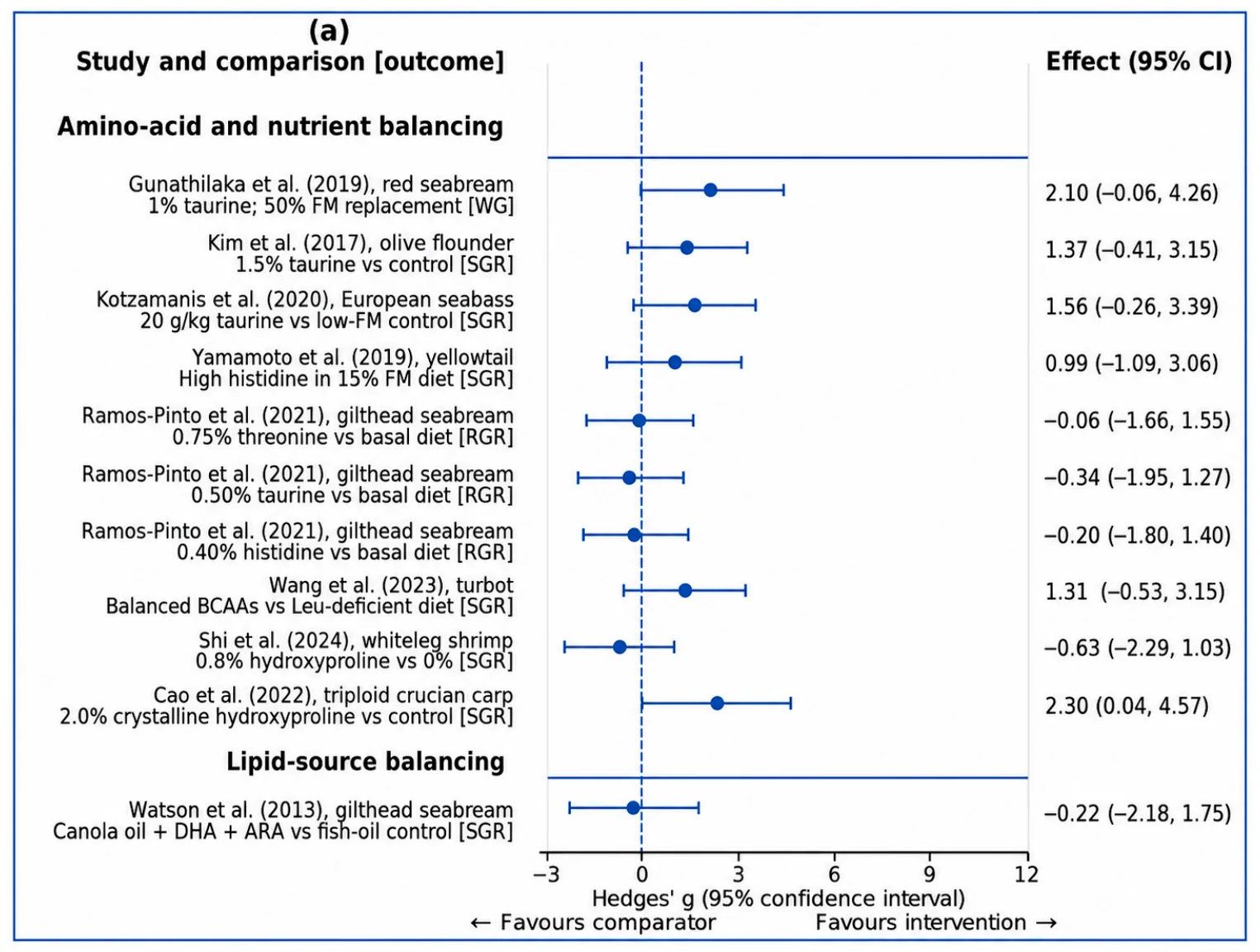

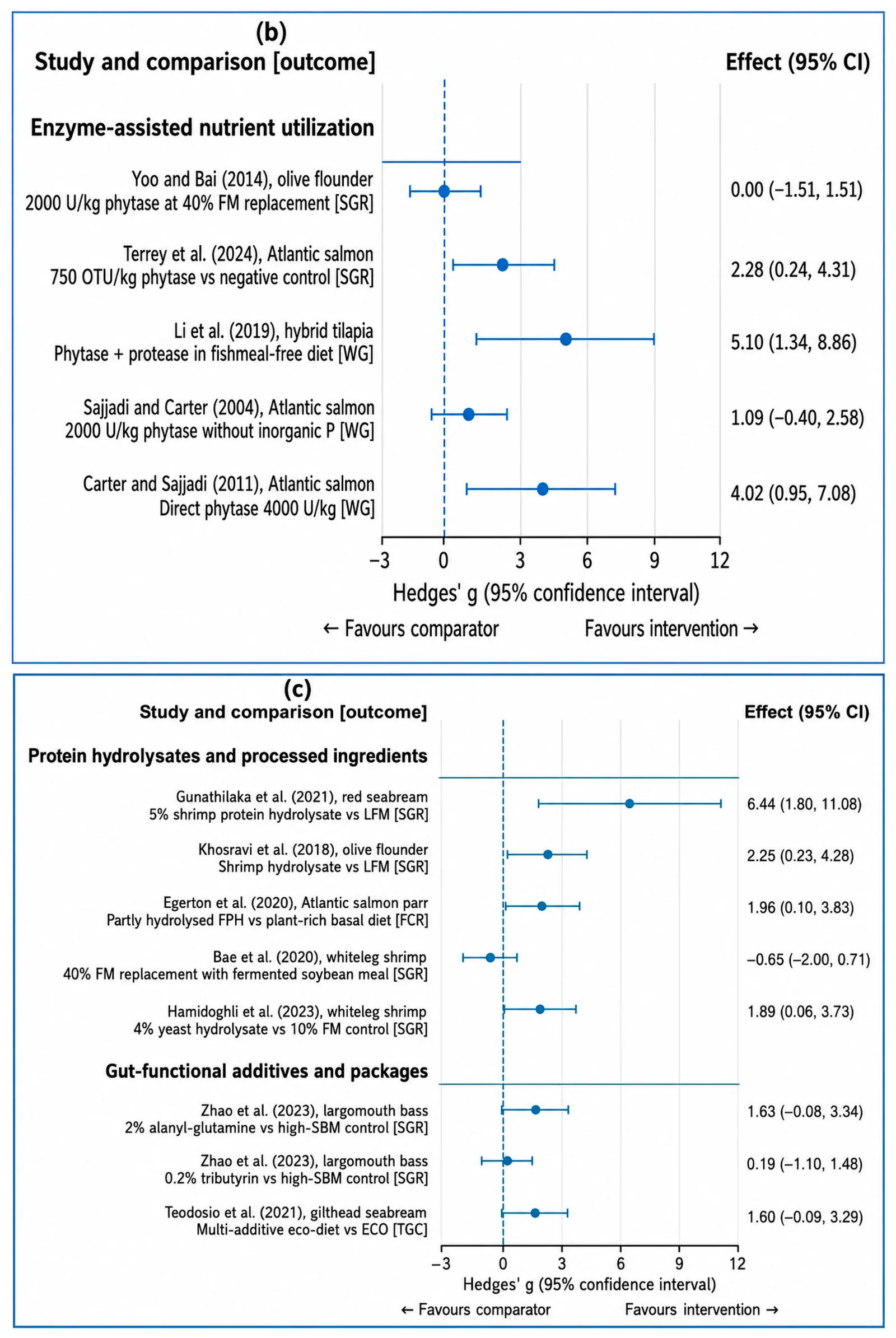
Descriptive forest plots of selected study-level performance effects of nutritional-balancing strategies in low-fishmeal aquafeeds. The studies included in panels (**a**–**c**) are cited in [33,34,35,37,38,39,40,41,43,44,45,46,47,48,49,50,51,52,53,54,55]. The panels present (**a**) amino acid, nutrient, and lipid-source balancing strategies; (**b**) enzyme-assisted nutrient-utilization strategies; and (**c**) protein hydrolysates, processed ingredients, and gut-functional additive strategies. Points represent Hedges’ *g*, and horizontal lines represent unadjusted 95% confidence intervals. Positive values indicate a more favorable intervention response, whereas negative values favor the comparator. The direction of feed conversion ratio and other outcomes for which lower values were favorable was reversed so that positive values indicate improvement. The plots contain 24 intervention-comparator comparisons from 21 of the 23 included studies. Multiple comparisons from the same study represent distinct intervention groups and should not be interpreted as independent studies. No pooled estimates, study weights, heterogeneity statistics, overall effect tests, or formal comparisons among strategies were calculated. For graded-dose studies, the highest tested dose and, where applicable, the greatest fishmeal-replacement level were selected as representative comparisons; this does not imply that these levels were optimal or commercially recommended. Confidence intervals crossing zero were interpreted as statistical imprecision rather than proof of no biological effect. Strategy-level interpretation also considered study number, methodological quality, response consistency, mechanistic alignment, species coverage, and diet context. Complete effect-size estimates, data-processing notes, and reasons for nonestimability are provided in Supplementary Table S3.

Figure 5a–c present selected performance-related effects by strategy category. Effect magnitude and precision varied substantially, with wide confidence intervals common because most studies used only two or three replicate tanks or cages per treatment. Two studies were excluded from the forest plots because the means, variance estimates, or experimental-unit data were unavailable. The complete estimates, sample sizes, variability measures, and reasons for nonestimability are provided in Supplementary Table S3.

None of the included studies were designed as formal equivalence or noninferiority trials with prespecified margins, and many estimates were imprecise because of limited replication. Effect sizes were therefore used only to identify patterns of inferiority, functional parity, or potential superiority, not to confirm formal nutritional equivalence. Retrospective confirmation would require an appropriate fishmeal-based comparator, experimental-unit variance data, sufficiently precise confidence intervals, and the decision criteria defined in Section 2.3.

#### 3.5.7. Exploratory Subgroup Patterns According to Taxonomic Group, Reported Life Stage, and Diet Matrix

Marine non-salmonid finfish dominated the evidence base, comprising 13 studies and 13 estimable comparisons from 11 studies; Hedges’ *g* ranged from −0.34–6.44, with 2 of 13 confidence intervals excluding zero. Salmonids contributed to four studies and four comparisons (*g* = 1.09–4.02; 3/4 intervals excluding zero), three freshwater finfish studies and four comparisons (*g* = 0.19–5.10; 2/4), and three crustacean studies and three comparisons (*g* = −0.65–1.89; 1/3). These differences should not be interpreted as taxonomic effects because the intervention type and diet matrix were strongly confounded.

Eleven studies explicitly evaluated juveniles, fingerlings, or parrs, contributing nine comparisons (*g* = −0.65–2.25; 3/9 intervals excluding zero). Twelve studies did not clearly report life stage and contributed 15 comparisons (*g* = −0.34–6.44; 5/15). Adult and broodstock evidence was absent; therefore, conclusions apply mainly to juvenile production stages.

Soy-dominant diets were represented by seven studies and eight comparisons (*g* = −0.65–6.44; 3/8 intervals excluding zero), mixed plant-protein or fishmeal-free diets were represented by nine studies and eight comparisons (*g* = −0.22–5.10; 4/8), and other mixed alternative-protein diets were represented by seven studies and eight comparisons (*g* = −0.63–1.89; 1/8). Wide within-group ranges indicated that broad matrix categories did not explain the responses. The benefits were most evident when interventions addressed specific limitations, such as phytase for phosphorus limitation, hydrolysates for palatability or peptide supply, and gut-targeted additives for intestinal challenges. The complete subgroup assignments and estimates are provided in Supplementary Table S5.

#### 3.5.8. Integrative Cross-Strategy Synthesis and General Principles

Despite differences in species, diet matrices, interventions, and measured outcomes, three general principles emerged. First, nutritional-balancing strategies were most effective when they addressed a defined diet-induced limitation. Phytase has the clearest effects on phytate-rich or phosphorus-limited diets; hydrolysates and attractant-rich ingredients are most useful because palatability, intake, peptide supply, or digestibility are limiting; taurine is most relevant in marine finfish diets, in which reduced marine ingredients affect taurine-related physiological functions; and gut-targeted additives are most informative in intestinal or disease challenge models. Intervention efficacy therefore depended more on matching the strategy to the nutritional or functional gap than on the additive itself.

Second, mechanism-aligned outcomes are often more informative than growth alone. Improvements in phosphorus utilization, nutrient retention, tissue amino acid status, hematology, immunity, the intestinal condition, the microbiota, and disease resistance sometimes occur without clear growth enhancement. Conversely, growth improvement without measurements of intake, digestibility, retention, or physiological responses did not reveal the responsible mechanism. The evidence was strongest when the low-fishmeal diet produced an identifiable limitation, a corresponding mechanism-specific response was demonstrated, and supplementation restored both intermediate and final outcomes.

**Figure 6:**
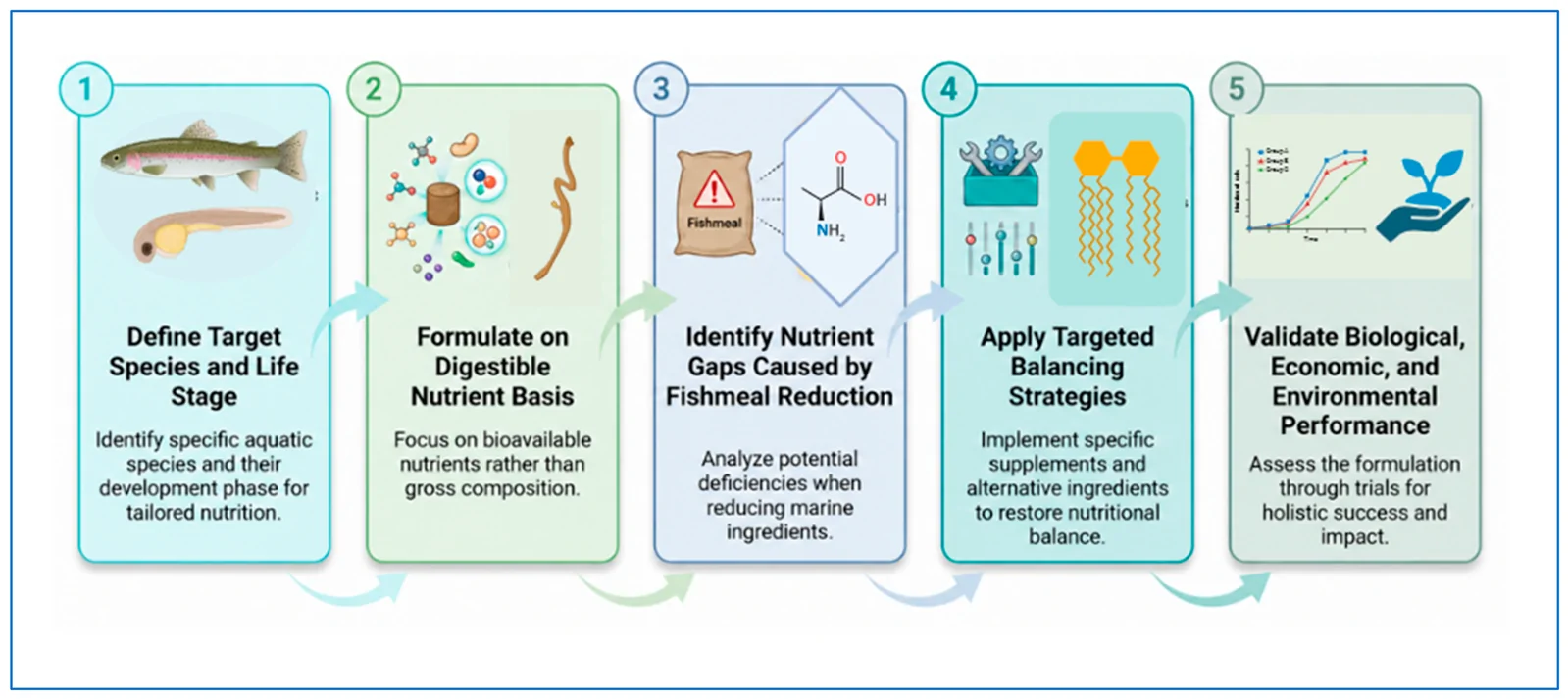
Five-step practical formulation framework for developing low-fishmeal aquafeeds. The framework outlines a stepwise approach for achieving nutritional equivalence, including defining the target species and life stage; formulating diets on a digestible nutrient basis; identifying nutrient gaps caused by fishmeal reduction; applying targeted balancing strategies; and validating biological, economic, and environmental performance.

Third, extensively reduced or fishmeal-free diets may create several concurrent limitations involving amino acid balance, taurine, palatability, phosphorus availability, lipid-associated nutrients, and intestinal compatibility. Such diets are therefore unlikely to be corrected by a universal single additive and may require coordinated whole-diet formulation using complementary ingredients and targeted supplementation. Together, Figure 3, Figure 4 and Figure 6 present an integrated framework linking the diagnosis of diet-induced limitations, mechanism-aligned validation, and practical formulation.

### 3.6. Practical Framework for Low-Fishmeal Aquafeed Formulation

The proposed framework follows a cause-and-correction approach: identify the function lost through fishmeal reduction, apply a mechanism-matched intervention, and validate the response via relevant endpoints. For example, phytase should be assessed through phosphorus availability and excretion, attractants through feed intake, taurine through lipid utilization and stress responses, and gut-targeted additives through microbiota, intestinal morphology, immunity, and disease resistance [24,30,33,43,57,59,73]. Because efficacy depends on the species, life stage, ingredient matrix, processing, dose, culture conditions, and production goals, the framework is intended as a diagnostic tool rather than a universal prescription.

Accordingly, low-fishmeal diets should be evaluated for their ability to maintain nutrient supply, feed intake, physiological and intestinal function, stress resilience, product quality, and nutrient-use efficiency, not simply by the percentage of fishmeal removed. The five-step framework therefore progresses from defining the target species and life stage to digestible nutrient formulation, nutrient-gap identification, targeted correction, and integrated biological, economic, and environmental validation (Figure 6).

#### 3.6.1. Step 1: Define the Species, Life Stage, and Production Goal

The first step is to define the target animal, developmental stage, culture condition, and production objective. Nutritional gaps caused by fishmeal reduction are not universal: marine carnivorous fish may be more sensitive to taurine, palatability, digestible protein quality, phosphorus availability, EPA/DHA supply, and gut disturbance, whereas dietary cholesterol and phospholipid balance may require particular attention in some crustaceans [74]. The production goal should also be explicit. A diet designed for maximum growth may require different balancing priorities from a diet designed for disease resistance, low-temperature feeding, fillet quality, or nutrient-waste reduction.

#### 3.6.2. Step 2: Formulate on a Digestible Nutrient Basis

Low-fishmeal diets should be formulated using digestible nutrient values rather than crude protein, total amino acids, or total phosphorus alone. Particular attention should be given to digestible indispensable amino acid ratios when crystalline amino acids are included because their absorption, leaching, and utilization may differ from those of protein-bound amino acids. Available phosphorus should likewise be prioritized because much of the phosphorus in plant ingredients is phytate-bound. The reviewed phytase studies revealed that phosphorus digestibility and retention may improve without parallel changes in growth, indicating that nutrient adequacy should be assessed directly through digestibility and retention endpoints.

#### 3.6.3. Step 3: Identify Nutrient Gaps Caused by Fishmeal Reduction

The third step is to identify which fishmeal-derived functions are lost in the specific formulation. These gaps may include digestible amino acid imbalance, reduced taurine and small peptides, lower palatability, lower available phosphorus and trace mineral bioavailability, reduced marine lipid-associated nutrients, increased antinutritional factors, and altered gut microbial or mucosal function. This gap analysis should be diet-specific. For example, a soybean meal-based diet may primarily require phytase, gut health support, and palatability restoration, whereas a fishmeal-free marine fish diet may additionally require taurine and careful lipid-associated nutrient balancing.

#### 3.6.4. Step 4: Apply Targeted Balancing Strategies

Balancing strategies should address the specific limitations identified in the diet. Whole-formula optimization using complementary ingredients should precede supplementation and account for digestible amino acids, available phosphorus, essential fatty acids, micronutrients, ingredient quality, processing, and practical inclusion limits. Targeted additives should be used only when relevant gaps remain. Taurine may reduce the taurine supply or impair lipid utilization; phytase can improve phytate–phosphorus availability; and hydrolysates, fermented ingredients, yeast products, or gut-targeted additives may support palatability, digestibility, and intestinal health. Highly reduced-fishmeal diets may require complementary interventions because no single additive restores all fishmeal functions.

#### 3.6.5. Step 5: Validate Biological, Economic, and Environmental Performance

The final step is integrated validation. A low-fishmeal diet should not be considered successful solely because it meets the calculated nutrient specifications. It should maintain growth, feed intake, FCR, survival, digestibility, nutrient retention, blood metabolic status, gut condition, immune and antioxidant capacity, stress and disease resilience, product quality, and nutrient waste outputs. Commercial validation should also include feed cost, cost per biomass gain, ingredient availability, and production-scale feasibility. This step distinguishes simple fishmeal replacement from true nutritional equivalence.

## 4. Discussion

### 4.1. Integrative Interpretation of the Evidence

The evidence was not equally strong across nutritional-balancing strategies. Phytase provided the most coherent mechanism-aligned support because favorable responses occurred mainly in phytate-rich or phosphorus-limited diets and were confirmed through phosphorus digestibility, retention, mineralization, or discharge-related outcomes. Protein hydrolysates showed consistently favorable performance-related estimates, but the small number of studies and differences among species, hydrolysate products, and diet matrices limited generalization. Taurine responses were generally favorable in direction but imprecise and restricted to a small marine-finfish evidence base. Evidence for essential amino acids beyond lysine and methionine, hydroxyproline, fermented ingredients, probiotics, yeast products, lipid-associated nutrients, and integrated stress-oriented strategies remained mixed, preliminary, or context-dependent.

Three general principles emerged from this evidence. First, intervention efficacy depended more on matching the strategy to the dominant diet-induced limitation than on the additive itself. Phytase was most informative when phytate-bound phosphorus limited availability; hydrolysates and attractant-rich ingredients were most relevant when palatability, intake, peptide supply, or digestibility was constrained; taurine was most relevant when reduced marine ingredients plausibly affected taurine-dependent functions; and gut-targeted strategies were most informative in diets associated with intestinal or disease-related challenges.

Second, mechanism-aligned outcomes were often more informative than growth alone. Improvements in phosphorus utilization, nutrient retention, tissue amino acid status, hematology, intestinal morphology, microbiota, immunity, or disease resistance sometimes occurred without a clear growth response. Conversely, growth improvement without measurements of feed intake, digestibility, retention, or relevant physiological responses did not establish why the intervention was effective. Evidence was therefore strongest when the low-fishmeal diet produced an identifiable limitation, the corresponding intermediate response was measured, and the intervention restored both the mechanism-specific and final outcomes.

Third, extensively reduced-fishmeal or fishmeal-free diets may create several simultaneous limitations involving amino acid balance, taurine, palatability, available phosphorus, lipid-associated nutrients, and intestinal compatibility. Such diets are unlikely to be corrected consistently by one universal additive. Complementary ingredient selection and whole-diet optimization should precede targeted supplementation, with additives used to address residual limitations that remain after digestible nutrient, lipid, micronutrient, processing, palatability, and cost constraints have been considered.

Within this framework, nutritional equivalence should not be interpreted as numerical identity between a low-fishmeal diet and a fishmeal-based reference diet. It should be evaluated according to whether the diet maintains prespecified production, nutrient-utilization, health, product-quality, environmental, and economic functions within an acceptable margin. Because none of the included studies were designed as formal equivalence or noninferiority trials, the current evidence supports targeted functional improvement and context-specific parity rather than confirmed universal nutritional equivalence.

### 4.2. Comparison with Previous Reviews and the Contributions of This Review

The present findings generally agree with previous reviews that identified taurine as an important functional nutrient in plant-based fish diets, phytase as an effective strategy for improving phytate-phosphorus utilization, protein hydrolysates as sources of digestible peptides and feeding stimulants, and probiotics or yeast-derived products as potential modulators of intestinal health and immunity [19,29,65,69,71]. However, those reviews focused primarily on individual nutrients, additives, ingredients, or biological mechanisms. They did not directly compare multiple nutritional-balancing strategies within a common low-fishmeal framework or evaluate whether these strategies restored functions lost through fishmeal reduction.

This review extends the previous literature by integrating eight nutritional-balancing categories beyond lysine and methionine, estimating study-level effect sizes where possible, mapping the causal pathway from fishmeal reduction to nutrient or functional loss and outcome restoration, and proposing operational criteria for nutritional equivalence. The findings also provide a more cautious interpretation of the literature. For example, earlier reviews emphasized the biological importance of taurine, whereas the present synthesis shows that direct low-fishmeal evidence remains limited to four marine finfish species and imprecise performance estimates. Similarly, the findings support previous conclusions regarding phytase, hydrolysates, and gut-targeted additives but demonstrate that their efficacy depends on whether the intervention matches the specific limitation created by the diet matrix. The principal contribution of this review is therefore an integrated, mechanism-aligned, and veterinary-relevant framework for selecting and evaluating nutritional strategies rather than reviewing each additive in isolation.

### 4.3. Evidence Interpretation in a Systematic Narrative Synthesis

Substantial heterogeneity in species, life stage, diet matrix, fishmeal inclusion, intervention, dose, comparator, trial duration, culture condition, and outcome definition precluded a meaningful pooled meta-analysis. The evidence was therefore interpreted using a structured narrative approach that considered response direction and consistency, effect-size precision, methodological quality, comparator structure, and mechanistic alignment.

The exploratory subgroup findings were interpreted cautiously because taxonomic group, life stage, diet matrix, and intervention type were strongly confounded. Salmonid studies predominantly evaluated phytase or hydrolysates, whereas marine-finfish studies more often evaluated taurine and amino acid strategies. Crustacean studies more frequently evaluated fermented or yeast-derived products. Consequently, differences among subgroup summaries cannot be attributed independently to taxonomy or production group and should be regarded as hypothesis-generating.

The evidence base also did not represent the full diversity of alternative-protein matrices. Rendered animal proteins were minimally represented, and insect, gluten, single-cell, and algal proteins were absent or insufficient for ingredient-specific synthesis. The conclusions therefore apply primarily to the species, juvenile life stages, plant-rich diets, interventions, and experimental conditions represented in the included studies.

### 4.4. Mechanism-Matched Interpretation of Nutritional Strategies

The causal framework was used to evaluate pathway completeness rather than to establish definitive causality. Evidence was strongest when fishmeal reduction or alternative-ingredient inclusion produced a measurable nutritional or functional limitation, altered a corresponding intermediate endpoint, impaired a relevant outcome, and the targeted strategy restored both the intermediate and final responses.

When the initial nutrient gap or intermediate mechanism was not measured, favorable growth, feed-efficiency, immune, or intestinal responses remained biologically plausible but associative. This distinction was particularly important for multifunctional products such as hydrolysates, fermented ingredients, yeast products, probiotics, and integrated additive mixtures, for which several mechanisms may operate simultaneously.

Intervention efficacy should therefore be interpreted in relation to the complete diet and intended biological function. A nonsignificant growth response should not be considered treatment failure when a prespecified mechanism-specific outcome improves, but such an improvement should not be generalized beyond the relevant nutrient limitation, species, diet matrix, and production condition.

### 4.5. Veterinary-Science Relevance: Gut Health, Immunity, Disease Resistance, and Resilience

Low-fishmeal formulations are relevant not only to production and sustainability but also to the maintenance of aquatic animal health. Several studies, including feeding trials, have evaluated intestinal morphology, the microbiota, hematological and immune indices, antioxidant responses, disease resistance, and stress tolerance in addition to growth and feed utilization. These endpoints may reveal functional effects of low-fishmeal diets that are not captured by short-term production measures alone.

The included studies provide examples of this broader response. Probiotic supplementation improved growth, nutrient utilization, immune responses, and the gut microbiota in juvenile olive flounder, whereas yeast hydrolysate improved the intestinal morphology and resistance to *Vibrio parahaemolyticus* in whiteleg shrimp. Fermented soybean meal, taurine, and protein hydrolysates also affect selected hematological, immune, antioxidant, intestinal, or stress-related outcomes [38,39,41,42,43]. These findings indicate that nutritional-balancing strategies may influence physiological resilience and disease-related responses as well as production performance.

Accordingly, when the intended function of an intervention involves gut health, immunity, or stress resilience, nutritional equivalence should not be evaluated solely on the basis of growth or feed conversion. A diet that maintains short-term growth but compromises the intestinal condition, immune competence, oxidative balance, or disease resistance may not provide equivalent biological function. Conversely, an intervention with modest effects on growth may still have practical value when it improves mechanism-aligned health or resilience outcomes. Therefore, health-related endpoints should be incorporated as complementary indicators of nutritional equivalence when they are directly relevant to the nutritional strategy being evaluated.

### 4.6. Interpretation of Outcome Domains

Growth, feed intake, feed conversion, and survival remain essential production outcomes, but they are insufficient as stand-alone measures of nutritional equivalence. Digestibility and nutrient retention provide more direct evidence of nutrient correction, whereas physiological, intestinal, immune, antioxidant, disease-resistance, and stress-related outcomes indicate whether production is maintained without hidden biological impairment [22,24,29,30].

The appropriate outcome set should be determined by the intended mechanism of the intervention. Phytase should be evaluated primarily through phosphorus digestibility, retention, mineralization, and discharge-related outcomes [34,37,49,50,51]. Palatability strategies require feed-intake measurements, whereas protein hydrolysates should be assessed through feed intake, digestibility, nutrient utilization, and relevant health outcomes [41,52,53]. Taurine should be evaluated using metabolic, lipid-related, physiological, immune, or stress-response endpoints [33,43,44,45]. Gut-targeted additives require intestinal morphology, microbiota, immune, inflammatory, and disease-challenge outcomes [39,42,54]. Standardized production outcomes should therefore be combined with prespecified mechanism-specific endpoints.

### 4.7. Formulation Implications for Low-Fishmeal Diets

Commercial formulations should not be interpreted as a choice between fishmeal and a single replacement ingredient followed by additive supplementation. In practice, nutritionists commonly combine multiple protein sources with complementary nutrient profiles and formulate under simultaneous constraints for digestible indispensable amino acids, available phosphorus, essential fatty acids, vitamins, minerals, palatability, ingredient availability, processing characteristics, and cost. Such multi-ingredient formulations may reduce or eliminate the need for some crystalline nutrients or functional additives. Accordingly, supplementation should be viewed as one component of a broader least-cost formulation process and should be applied only when ingredient combinations alone cannot achieve the required nutritional, functional, health, or production targets.

Targeted supplementation should therefore be based on diet-matrix-specific limitations rather than on the percentage of fishmeal replacement alone. Any proposed strategy should be validated in the intended species, life stage, ingredient matrix, replacement level, and production environment using appropriate performance, health, economic, and environmental endpoints. Positive responses observed in one species or formulation should not be transferred directly to other aquaculture systems, and commercial evaluation should consider ingredient quality, additive form and dose, processing stability, availability, and cost per unit of biomass gain.

### 4.8. Cost–Benefit Considerations for Commercial Adoption

Commercial adoption of nutritional-balancing strategies depends on whether their biological benefits justify the added costs of supplementation, processing, feed manufacture, and quality control. Economic value should therefore be assessed at the complete-diet and production-system levels rather than by additive price alone. A more costly strategy may still be worthwhile if it improves growth, FCR, survival, disease resistance, product quality, or nutrient retention. The key indicators should include diet cost, feed cost per biomass gain, additive cost, FCR, survival-adjusted production, and product-quality benefits. The economic comparator should not be limited to a non-supplemented low-fishmeal diet. Where possible, the tested supplementation strategy should also be compared with a least-cost reformulated diet using complementary protein and micronutrient sources because the same nutritional target may sometimes be achieved more economically through ingredient blending than through direct supplementation.

The economic value of nutritional-balancing strategies varies by intervention. Phytase may reduce phosphorus supplementation and discharge, whereas the cost-effectiveness of taurine, amino acids, hydrolysates, attractants, and gut-targeted additives depends on their effects on growth, feed utilization, health, and scalability. The feed cost per biomass gain is a useful basic indicator, but survival, production duration, health-management costs, ingredient availability, and environmental benefits should also be considered. Because economic outcomes are inconsistently reported, no definitive ranking was possible, highlighting the need for standardized economic evaluation in future trials.

### 4.9. Regulatory Status, Safety Considerations, and Upper Safe Limits

Commercial use of nutritional-balancing strategies must comply with region-, product-, species-, and use-specific regulations, and experimental efficacy does not confirm legal authorization of an additive, dose, formulation, or claim in all markets. Safety assessment should also be intervention-specific. Excessive nutrient supplementation may cause imbalance or environmental discharge, whereas enzymes, hydrolysates, fermented products, marine-derived ingredients, and probiotics require appropriate evaluations of their activity, stability, contamination, consistency, traceability, and microbial safety. Importantly, the highest experimental dose should not be considered a recommended, legally permitted, or safe upper limit. Because tolerance data were generally limited, the inclusion levels in Table 2 represent only those tested in the reviewed studies. Future work should include dose–response safety assessments, nutrient interactions, environmental outputs, and verification of additive stability after feed processing.

### 4.10. Knowledge Gaps in the Current Evidence Base

Several limitations constrain the strength and generalizability of the available evidence. Many studies inadequately report digestible amino acids, available phosphorus, mineral bioavailability, fatty acid and phospholipid composition, additive stability, and antinutritional factor profiles. This limits the assessment of whether experimental diets are functionally comparable. Dose–response evidence is also insufficient for many nutrients beyond lysine and methionine, including taurine, histidine, threonine, tryptophan, branched-chain amino acids, hydroxyproline, and other functional nitrogenous compounds.

Additional evidence gaps included limited evaluation under realistic production stressors; inadequate reporting of product-quality, environmental, and economic outcomes; insufficient mechanistic validation; and inconsistent reporting of experimental design and mechanism-aligned outcomes. Evidence distinguishing the restoration of functions lost through fishmeal reduction from compensation for limitations introduced by alternative ingredients or enhancement beyond the reference diet is also limited.

### 4.11. Strengths and Limitations of the Review

This review followed PRISMA 2020, used a broad multisource search supported by known-item testing and citation tracking, applied standardized quality assessment, and calculated study-level effect sizes where possible. It also integrates diverse nutritional strategies within a common mechanism-based framework, distinguishes associative from mechanistically supported evidence, and applies context-specific criteria for nutritional equivalence.

Several limitations should be considered. First, omission of the ASFA, AGRICOLA, and FSTA may have introduced retrieval bias, particularly for older, regionally indexed, less-cited, or non-English studies. Although multidisciplinary databases, CAB abstracts, Google Scholar, publisher searches, reference screening, and citation tracking were used, the evidence base cannot be considered exhaustive. Variation in terminology may also have reduced retrieval because eligible studies were sometimes indexed by ingredient, additive, processing method, or physiological outcome rather than by “low-fishmeal” or “fishmeal replacement”.

Second, substantial heterogeneity in species, life stage, diet matrix, fishmeal inclusion, supplementation dose, trial duration, culture conditions, and outcome reporting limits generalizability and quantitative synthesis. The core outcomes were inconsistently reported, and mechanism-specific endpoints differed among studies. Fishmeal and plant protein quality are also poorly characterized, despite potential variations in source, processing, storage, nutrient availability, palatability, and antinutritional factors. Accordingly, fishmeal controls and soy-dominant diets should be regarded as study-specific benchmarks and broad matrix categories rather than standardized treatments.

Methodological quality and reporting completeness also affected the strength of the review-level conclusions. Confidence was reduced when studies used limited tank- or cage-level replication, did not clearly identify the experimental unit, incompletely described randomization, lacked analytical confirmation of relevant diet characteristics, or did not report sufficient variance data. These limitations were particularly important for strategies represented by one or two studies and for apparently large effects accompanied by wide confidence intervals. The review-level interpretations in Table 3 therefore considered methodological credibility and reporting completeness in addition to effect direction, consistency, and magnitude.

Third, the evidence was uneven across strategies and diet matrices. Taurine studies have been limited to four marine finfish species, mainly juveniles, and supplementation levels of approximately 0.25–2.0%. The evidence for several amino acids, vitamin-antioxidant strategies, lipid-associated nutrients, product-quality outcomes, rendered animal proteins, insect meals, and other alternative protein matrices is sparse or absent. These gaps partly reflect the intervention-focused eligibility criteria and restrict extrapolation beyond the represented species and formulations.

The subgroup analyses were post hoc, based on broad classifications, and confounded by intervention type, diet matrix, and incomplete life-stage reporting. They should therefore be considered descriptive and hypothesis-generating rather than evidence of formal effect modification. Publication bias and incomplete reporting of feed intake, digestible nutrients, mineral retention, fatty acid composition, additive stability, gut health, disease resistance, and economic outcomes may also influence synthesis.

Finally, causal inference remains limited because nutrient gaps and intermediate mechanisms are often not measured directly, control structures are sometimes incomplete, and some interventions are multicomponent. Thus, most pathways were partially supported or associative rather than definitively causal. The proposed 5% noninferiority margin should likewise be interpreted cautiously because it has not been validated across species, life stages, production systems, or outcome domains. Overall, this review provides a systematic narrative synthesis and mechanism-based formulation framework but not a definitive quantitative ranking of nutritional-balancing strategies or proof of formal nutritional equivalence.

## 5. Conclusions

Sustainable fishmeal reduction requires attention to nutritional and functional limitations beyond those associated with lysine and methionine. Across the 23 included studies, intervention efficacy depended primarily on the alignment among the diet-induced limitation, the selected strategy, and the endpoints used to confirm restoration. However, the evidence has been dominated by short-term trials in juvenile finfish, particularly marine non-salmonids, and remains limited for adults, broodstock, crustaceans, commercial production systems, and poorly represented diet matrices such as rendered animal byproducts and insect meals.

Phytase provides the clearest mechanism-aligned evidence in plant-rich or phosphorus-limited diets. Taurine produced favorable but imprecise responses in four marine finfish species, whereas protein hydrolysates, fermented ingredients, yeast-derived products, probiotics, hydroxyproline, and other functional additives showed context-dependent benefits. The available evidence therefore does not support a universal additive combination, supplementation dose, or fishmeal-replacement threshold.

Nutritional balancing should instead be integrated with complementary ingredient selection and whole-diet optimization. Strategies should be selected according to the specific limitations created by the diet matrix and validated in the target species and life stage using mechanism-specific, production, health, economic, safety, and environmental outcomes. Because most studies were not designed as formal equivalence or noninferiority trials, the current evidence supports targeted functional improvement rather than confirmed universal nutritional equivalence.

Future research should prioritize species- and life-stage-specific dose–response and tolerance studies, longer production-scale trials, and validation under commercially relevant environmental and disease challenges. Improved reporting of digestible nutrient composition, ingredient quality, mechanism-specific responses, product quality, economics, and environmental effects is also needed before broad commercial recommendations can be established.

## Figures and Tables

**Table 1:** Operational classification of nutritional equivalence in low-fishmeal aquafeeds.

Classification	Operational Meaning
Formal nutritional equivalence	The 95% confidence interval meets a predefined equivalence or noninferiority margin for all core outcomes, with no biologically meaningful deterioration in critical health or safety outcomes.
Functional parity	Responses are descriptively similar to the fishmeal control, but formal equivalence cannot be established because of insufficient variance data, wide confidence intervals, absence of a predefined margin, or lack of an appropriate reference control.
Superiority	At least one prespecified primary outcome exceeds the favorable benchmark with confidence-interval support, without important deterioration in other core outcomes.
Not equivalent	One or more critical outcomes fall outside the predefined acceptable margin or show biologically meaningful deterioration.
Equivalence not demonstrated	The study design or reporting does not provide sufficient information to confirm or reject equivalence.

**Table 2:** General and quantitative characteristics of the 23 studies included in the systematic review, grouped according to the primary nutritional-balancing strategy.

Primary Strategy, No. of Studies and Species	Species Represented	Main Diet Context and Fm Inclusion Range (%)	Duration (Weeks)	Primary Supplementation or Intervention Range	Main Outcome Domains
Taurine supplementation (*n* = 4 studies; 4 species)	Red seabream (*Pagrus major*); olive flounder (*Paralichthys olivaceus*); European seabass (*Dicentrarchus labrax*); gilthead seabream (*Sparus aurata*)	LFM, high-SBM, high-plant-protein and FM-free diets; FM: 0–50%	10–20	Taurine: 0.25–2.0% diet or 2.5–20 g/kg	Growth and feed utilization; immunity; antioxidant status; lipid metabolism; fillet quality; stress responses
Essential and functional amino acids beyond lysine and methionine (*n* = 4; 3 species)	Yellowtail (*Seriola quinqueradiata*); gilthead seabream (*Sparus aurata*); turbot (*Scophthalmus maximus*)	LFM, FM-free, plant-protein and amino-acid-balanced diets; FM: 0–15%	4–13.1	Histidine: variable; threonine: 0.75%; taurine: 0.50%; BCAAs: deficient-to-excess levels; tryptophan: supplemented.	Growth; feed intake; amino-acid metabolism; tissue amino acids; hematology; immunity; antioxidant responses; gene expression
Hydroxyproline and collagen-related nutrients (*n* = 2; 2 species)	Triploid crucian carp (triploid *Carassius auratus*); Pacific white shrimp (*Litopenaeus vannamei*)	Very-LFM plant-based and LFM shrimp diets; FM: 0.5–25%	8	Hydroxyproline: 0.2–2.0% diet	Growth; collagen synthesis; muscle development and texture; product quality; growth-signaling pathways
Phytase and enzyme-based improvement of phosphorus availability and utilization (*n* = 5; 3 species)	Olive flounder (*Paralichthys olivaceus*); Atlantic salmon (*Salmo salar*); hybrid tilapia (*Oreochromis niloticus* × *O. aureus*)	SBM, SPC, canola-meal, high-plant-meal, low-P and FM-free diets; FM: 0–64%	8–12	Phytase: 250–4000 U/kg, 750 OTU/kg, or 400 mg/kg; protease: 175 mg/kg; phytase pretreatment: 250–1000 U/kg SPC	Growth and feed utilization; nutrient and P digestibility; P retention and discharge; bone mineralization; intestinal morphology
Protein hydrolysates and attractant-rich ingredients (*n* = 3; 3 species)	Red seabream (*Pagrus major*); olive flounder (*Paralichthys olivaceus*); Atlantic salmon (*Salmo salar*)	LFM, SPC and high-plant-protein diets; FM: 5–40%	11–15	Protein hydrolysates: 2.88–10% diet; krill meal: 5% diet	Growth/feed use; digestibility; gut health and microbiota; immunity, antioxidant response, and disease resistance.
Fermented ingredients, yeast products, probiotics and gut-targeted additives (*n* = 4; 3 species)	Pacific white shrimp (*Litopenaeus vannamei*); olive flounder (*Paralichthys olivaceus*); largemouth bass (*Micropterus salmoides*)	FM-replacement, LFM, high-SBM and fermented-plant-protein diets; FM: 10–35%	7–12	Fermented ingredients: 20–40% FM replacement; YH: 0.5–4%; probiotics: 10^8^–10^9^ CFU/kg; TB: 0.1–0.2%; AGn: 1–2%.	Growth/feed use; gut health and microbiota; immunity, oxidative/inflammatory status, apoptosis, and disease resistance.
Integrated multiadditive strategy under environmental stress (*n* = 1; 1 species)	Gilthead seabream (*Sparus aurata*)	Low-protein, LFM poultry-meal diet under low temperature; FM: 7.5–27.5%	12	ECOSup mixture containing betaine, krill meal, lecithin, macroalgae and taurine; component doses reported where available	Low-temperature growth; feed/nutrient use; body indices; *n* losses; sustainability and cost.
Overall evidence base	11 aquatic species or experimental taxa	LFM/FM-free plant-based, SBM/SPC, canola, fermented, low-P, and functional diets; FM: 0–64%.	4–20	Strategy-specific units; no combined dose range calculated	Growth and nutrient use; health and immunity; stress tolerance; product quality; sustainability.

Abbreviations: AGn, alanyl-glutamine; BCAA, branched-chain amino acid; CFU, colony-forming unit; ECOSup, supplemented eco-friendly diet; FM, fishmeal; LFM, low-fishmeal; N, nitrogen; OTU, OptiPhos phytase activity unit; P, phosphorus; SBM, soybean meal; SPC, soy protein concentrate; TB, tributyrin; U, enzyme activity unit; YH, yeast hydrolysate; *n*, number of studies. Footnote: The supplementation ranges represent doses evaluated in the included experimental studies and should not be interpreted as recommended commercial inclusion levels, legally authorized maximum contents, or established upper safe limits.

**Table 3:** Strategy-level consistency, precision, and review-level interpretation of evidence for nutritional-balancing strategies in low-fishmeal aquafeeds.

Strategy	Synthesized Response Pattern	Quantitative Evidence Support	Interpretation	References
Taurine supplementation	Taurine showed favorable but imprecise responses in a limited marine-finfish evidence base.	3 studies; 3 comparisons; g = 1.37–2.10; CI+ = 0/3	Promising but limited. Responses favored supplementation directionally, but all representative confidence intervals crossed zero, and evidence was restricted to a small number of marine finfish species and diet models.	[33,43,44]
Lipid-source balancing	Growth was maintained, with no clear intervention effect.	1 study; 1 comparison; g = −0.22; CI+ = 0/1	Insufficient evidence. The conclusion was based on one study and did not demonstrate a clear performance benefit.	[45]
Essential amino acids beyond lysine and methionine	Growth responses were mixed, although mechanism-specific metabolic or immune effects were reported.	4 studies; 3 estimable; 5 comparisons; g = −0.34–1.31; CI+ = 0/5	Limited and mixed. The evidence suggests possible mechanism-specific effects but does not support consistent production benefits or general dose recommendations.	[35,36,40,46]
Hydroxyproline and collagen-related nutrients	Growth responses varied, and one comparison showed a clear favorable effect.	2 studies; 2 comparisons; g = −0.63–2.30; CI+ = 1/2	Preliminary and mixed. Potential benefits for collagen synthesis and product quality were reported, but evidence was limited to two species and two studies.	[47,48]
Phytase and enzyme-based phosphorus balancing	Responses were generally favorable, particularly when phosphorus availability was limited.	5 studies; 5 comparisons; g = 0.00–5.10; CI+ = 3/5	Most coherent mechanism-aligned evidence. Effects were strongest in phytate-rich or phosphorus-limited diets and were supported by phosphorus digestibility, retention, mineralization, or discharge-related outcomes. Growth responses remained diet-dependent.	[34,37,49,50,51]
Protein hydrolysates and attractant-rich ingredients	Growth or feed-utilization responses were consistently favorable.	3 studies; 3 comparisons; g = 1.96–6.44; CI+ = 3/3	Consistently favorable but limited. All representative estimates favored the intervention with confidence-interval support, but only three studies and heterogeneous products and diet matrices were represented.	[41,52,53]
Fermented ingredients, yeast products, probiotics, and gut-targeted additives	Growth responses were mixed, although intestinal, immune, or disease-resistance benefits were frequently reported.	4 studies; 3 estimable; 4 comparisons; g = −0.65–1.89; CI+ = 1/4	Mixed and context-dependent. Evidence was stronger for mechanism-specific intestinal and health outcomes than for consistent growth improvement, and products and experimental models were highly heterogeneous.	[38,39,42,54]
Integrated strategy under environmental stress	A favorable but imprecise response was observed under low temperature.	1 study; 1 comparison; g = 1.60; CI+ = 0/1	Preliminary and insufficient for generalization. The evidence was based on one multicomponent intervention, preventing attribution to individual components.	[55]
Overall evidence base	Most estimates favored the intervention directionally, but many were imprecise.	23 studies; 21 estimable; 24 comparisons; g = −0.65–6.44; CI+ = 8/24	The evidence supports targeted, diet-specific correction rather than universal supplementation. Confidence was highest when the intervention addressed a defined limitation and mechanism-aligned outcomes confirmed restoration.	[33,34,35,36,37,38,39,40,41,42,43,44,45,46,47,48,49,50,51,52,53,54,55]

Footnote: *g*, Hedges’ *g*; CI+, number of comparisons whose 95% confidence intervals excluded zero in favor of the intervention. Effect-size ranges are descriptive and were not pooled. Review-level interpretations considered the number of studies, response direction and consistency, effect-size precision, methodological limitations, species and diet coverage, and mechanistic alignment. These interpretations are not formal GRADE certainty ratings. Complete study- and outcome-level estimates are provided in Supplementary Table S3.

**Table 4:** Causal pathways linking fishmeal reduction, nutritional gaps, biological mechanisms, targeted correction strategies, and restored outcomes.

Nutritional-Balancing Category	Proposed Causal Pathway	Causal Support	References
Essential amino acids beyond lysine and methionine	Fishmeal reduction → amino acid imbalance → impaired metabolism and growth → amino acid supplementation → improved physiological responses	Partially supported/associative	[35,36,40,46]
Functional amino acids and nonprotein nitrogenous compounds	Fishmeal reduction → reduced functional nitrogenous compounds → impaired metabolism, stress resilience, or collagen synthesis → supplementation → improved performance and tissue quality	Partially to mechanistically supported	[33,43,44,45,46,47,48]
Palatability and feed-intake enhancers	Fishmeal reduction → reduced attractants → lower feed intake → attractant supplementation → improved intake and performance	Partially supported	[39,41,52,53]
Digestibility improvement and enzyme supplementation	Alternative ingredients → antinutritional factors and poor digestion → reduced nutrient utilization → processing or enzyme supplementation → improved digestibility	Partially supported	[38,49,54]
Mineral and phosphorus balancing	Plant ingredients → phytate-bound phosphorus → reduced phosphorus availability → phytase or phosphorus balancing → improved utilization and reduced discharge	Mechanistically supported	[34,37,49,50,51]
Vitamin and antioxidant support	Fishmeal reduction → micronutrient limitation or oxidative stress → weakened antioxidant and immune defenses → supplementation → improved protection	Associative	[38,39,41,43]
Lipid-associated nutrient balancing	Reduced marine ingredients → lower lipid-associated nutrients → impaired lipid metabolism and tissue quality → supplementation → improved lipid-related outcomes	Partially supported/associative	[44,45]
Gut-health and microbiome-targeted strategies	Alternative ingredients → intestinal stress and microbiota disruption → impaired gut health and immunity → gut-targeted additives → improved intestinal function and disease resistance	Partially to mechanistically supported	[39,42,54]

Footnote: Arrows indicate the proposed sequential pathway and do not imply that every causal link was directly demonstrated. Mechanistically supported evidence indicates that the nutritional gap, a mechanism-aligned response, the impaired outcome, and restoration were demonstrated within the same experimental framework. Partially supported evidence indicates that one or more links were not measured directly. Associative evidence indicates that improvement was reported without direct confirmation of the proposed nutritional gap or intermediate mechanism. Causal-support classifications describe pathway completeness and should not be interpreted as formal risk-of-bias or certainty ratings. Environmental and biological stresses were treated as contextual modifiers rather than separate nutritional-balancing categories.

**Table 5:** Recommended outcome domains for validating nutritional equivalence in low-fishmeal aquafeeds.

Validation Domain	Core Endpoints	Supporting Endpoints	Relevance to Nutritional Equivalence	Representative Studies
Growth and feed utilization	Weight gain, SGR, FCR, feed intake, survival	PER, condition factor, whole-body composition	Confirms whether fishmeal reduction maintains basic production performance	[33,38,39,41,42,43,44,52,55]
Digestibility and nutrient retention	Apparent digestibility coefficients, phosphorus digestibility, phosphorus retention, protein efficiency	Amino acid digestibility, nitrogen retention, fecal phosphorus loss	Determines whether the diet achieves digestible nutrient equivalence rather than crude nutrient equivalence	[34,37,41,42,49,50,51]
Physiological and metabolic status	Hematology, plasma cholesterol, glucose, triglycerides, liver-related indicators	Tissue free amino acids, lipid metabolism indicators, metabolic enzyme activity	Detects hidden metabolic imbalance that may not be reflected by growth alone	[35,41,43,44,45]
Immune and antioxidant capacity	Lysozyme, NBT, MPO, immunoglobulin, SOD, CAT, GPx	Cytokine expression, complement activity, lipid peroxidation markers	Evaluates whether low-fishmeal diets maintain immune competence and oxidative balance	[36,38,39,40,41,43]
Intestinal condition and microbiota	Gut morphology, intestinal barrier status, microbiota composition	Digestive enzyme activity, inflammatory markers, mucosal indicators	Assesses whether alternative ingredients compromise gut integrity or microbial stability	[39,42,53,54]
Stress and disease resilience	Low-temperature performance, bacterial challenge, survival after stress	Hematology, immune markers, stress-related biomarkers	Confirms whether the diet performs under practical farming stressors rather than only under optimal laboratory conditions	[33,39,43,55]
Product quality	Muscle texture, collagen synthesis, fatty acid profile, organoleptic traits	Fillet composition, shelf-life indicators, consumer-relevant nutritional value	Determines whether fishmeal reduction maintains market quality and nutritional value	[44,45,47,48]
Environmental and economic relevance	Phosphorus retention, fecal phosphorus output, nutrient retention, cost per gain	FIFO, life-cycle indicators, ingredient-use efficiency	Links nutritional balancing to sustainability, waste reduction, and commercial feasibility	[34,37,49,50,51]

Abbreviations: CAT, catalase; FCR, feed conversion ratio; FIFO, fish-in fish-out ratio; GPx, glutathione peroxidase; MPO, myeloperoxidase; NBT, nitro-blue tetrazolium; PER, protein efficiency ratio; SGR, specific growth rate; SOD, superoxide dismutase.

## Data Availability

The original contributions presented in this study are included in the article/Supplementary Material. Further inquiries can be directed to the corresponding author.

## Supplementary Materials

The following supporting information can be downloaded at https://www.mdpi.com/article/10.3390/vetsci13080762/s1, Table S1, expanded study-level extraction and evidence-role matrix of the 23 feeding trials included in the qualitative synthesis; Table S2, methodological quality and reporting assessment of the 23 included feeding trials; Table S3, Study-level effect-size estimates and 95% confidence intervals for nutritional-balancing strategies in low-fishmeal aquafeeds; Table S4, Search-strategy validation and retrieval audit of all 23 studies included in the systematic review; Table S5, exploratory subgroup classification and descriptive response patterns according to taxonomic group, reported life stage, and primary diet matrix.
