# Supplementary material for: Nutritional Balancing Strategies in Low-Fishmeal Aquafeeds: A PRISMA 2020-Based Systematic Review Beyond Lysine and Methionine Supplementation
Source: Vet Sci. 2026 Jul 30;13(8):762. doi: 10.3390/vetsci13080762 (PMC13517252; doi:10.3390/vetsci13080762)
Supplement: Supplementary file 1 [file vetsci-13-00762-s001.zip › PRISMA_2020_Checklist_Final.pdf]

PRISMA 2020 Checklist - Revised Assessment

Manuscript title: Nutritional balancing strategies in low-fishmeal aquafeeds: A PRISMA 2020-based systematic review beyond lysine and methionine supplementation

Section	Item	Reporting item (summarized)	Location in manuscript / supplement	Status and comments
Title	1	Identify the report as a systematic review.	Title page: article type "Systematic Review"; manuscript title includes "PRISMA 2020-based systematic review".	**Reported.**
Abstract	2	Provide the PRISMA abstract information: objective, eligibility/source concept, included studies, synthesis approach, main findings, and limitations where relevant.	Abstract; related details appear in Sections 2.1, 2.5, and 2.11 and in the Funding section.	**Reported.** The abstract reported the objective, eligibility scope, 23 included studies, synthesis approach, principal findings, and non-pooling rationale. Add the information sources and last search date, quality/risk-of-bias method, primary funding source, and OSF registration, subject to the journal word limit.
Introduction	3	Explain the rationale for the review in relation to existing knowledge.	Introduction, particularly the rationale, remaining nutritional limitations after lysine/methionine correction, veterinary relevance, and gap in prior reviews.	**Reported.**
Introduction	4	State the review objective or review question clearly.	End of Introduction; Section 2.2.	**Reported.** Primary and secondary review questions were explicitly stated.
Methods	5	Specify eligibility criteria for populations, interventions/exposures, comparators, outcomes, study designs, and report characteristics.	Sections 2.3, 2.4, and 2.7.	**Reported.** PICOS elements, report types, study designs, and exclusion criteria were described. Explicitly state whether a language restriction was applied (for example, "No language restrictions were applied," if accurate).
Methods	6	Specify all information sources and the date of the last search.	Section 2.5.	**Reported.** Web of Science, Scopus, PubMed, ScienceDirect, CAB Abstracts, supplementary sources, and citation tracking are identified. The initial search date (17 December 2025), update date (18 June 2026), and absence of a lower date limit are reported.
Methods	7	Present the complete search strategy sufficiently for replication.	Section 2.6; Supplementary Table S4.	**Reported.** The core strategy and supplementary search routes were described, and S4 is cited for database-specific details.
Methods	8	Describe how studies were screened and selected.	Section 2.8; Figure 1.	**Reported.** One reviewer screened titles/abstracts and full texts; a second reviewer verified all full-text decisions and a random 20% of title/abstract decisions. Disagreements were resolved by consensus, and no automated screening tools were used.
Methods	9	Describe data extraction methods and any procedures for obtaining or confirming data.	Section 2.9; Supplementary Table S1.	**Reported.** Primary extraction, second-reviewer checking, consensus procedures, figure-data extraction, treatment of non-extractable data, and absence of automation were reported.
Methods	10a	List and define all outcomes for which data were sought.	Sections 2.4 and 2.9; Section 3.5; Table 5.	**Reported.** All sought outcome domains were listed and defined.
Methods	10b	List other variables for which data were sought, such as study and intervention characteristics.	Section 2.9; Supplementary Table S1.	**Reported.** Study, animal, diet, intervention, design, culture, statistical, and outcome variables were specified.
Methods	11	Describe the method used to assess study quality or risk of bias.	Section 2.11; Supplementary Table S2.	**Reported.** Assessment domains, judgment categories, reviewer checking, and consensus procedures were reported. This was a review-specific methodological-quality/reporting assessment rather than a validated formal risk-of-bias instrument.
Methods	12	Specify effect measures used for each outcome.	Section 2.14.	**Reported.** Hedges' g with 95% confidence intervals was used where estimable; SE-to-SD conversion, experimental-unit rules, and direction reversal for lower-is-better outcomes were described.
Methods	13a	Describe how studies were selected as eligible for each synthesis.	Sections 2.10 and 2.13-2.15.	**Reported.** Eligibility for narrative and descriptive effect-size syntheses was explained.

PRISMA 2020 Checklist - Revised Assessment

Section	Item	Reporting item (summarized)	Location in manuscript / supplement	Status and comments
Methods	13b	Describe data preparation and handling before synthesis.	Sections 2.9 and 2.14.	**Reported.** Data extraction from figures, SE-to-SD conversion, experimental-unit handling, direction standardization, dose-selection rules, and non-estimability were described.
Methods	13c	Describe tabulation or visual display methods used for synthesis.	Sections 2.13-2.15; Tables 1-5; Figures 2-6; Supplementary Tables S1-S5.	**Reported.** Tabular, framework, forest-plot, and supplementary displays were described.
Methods	13d	Describe synthesis methods and, when meta-analysis is not performed, explain why.	Sections 2.13 and 2.14.	**Reported.** A structured narrative synthesis and descriptive forest plots were used; statistical pooling was not performed because of substantial clinical and methodological heterogeneity.
Methods	13e	Describe methods used to explore possible causes of heterogeneity.	Section 2.15.	**Reported.** Post hoc descriptive subgroups were defined by taxonomic/production group, reported life stage, and primary diet matrix; no pooling, interaction testing, or meta-regression was performed.
Methods	13f	Describe sensitivity analyses used to assess robustness of synthesized results.	Not applicable; Sections 2.13-2.15.	**Not applicable / not performed.** No pooled synthesis was generated, so formal sensitivity analysis was not conducted.
Methods	14	Describe methods for assessing risk of bias due to missing results or publication/reporting bias.	Not currently reported in Methods; possible publication bias is discussed in Section 4.11.	**Reported.** The risk of bias due to missing results was not formally assessed because no pooled meta-analysis was conducted.
Methods	15	Describe methods for assessing certainty or confidence in the body of evidence.	Sections 2.11-2.13; Supplementary Table S2; Table 3.	**Reported.** Confidence was judged narratively using study number, consistency, precision, methodological quality, comparator structure, mechanistic alignment, species/diet coverage, and replication across contexts. The manuscript explicitly stated that no formal GRADE assessment was used.
Results	16a	Report numbers of records identified, screened, assessed for eligibility, and included.	Section 3.1; Figure 1.	**Reported.** Counts are internally consistent: 328 identified, 96 duplicates removed, 232 screened, 174 excluded at title/abstract stage, 58 full texts assessed, 35 full texts excluded, and 23 studies included.
Results	16b	Report excluded full-text studies and reasons for exclusion.	Section 3.1; Figure 1. A study-level exclusion list is not currently identified.	**Reported.** Aggregated reasons and counts for 35 excluded full-text reports were reported.
Results	17	Cite and describe included studies.	Section 3.2; Table 2; Supplementary Table S1.	**Reported.** Included studies are cited and described at study level.
Results	18	Present quality/risk-of-bias assessments for included studies.	Section 3.2.1; Supplementary Table S2; Section 4.11.	**Reported.** Section 3.2.1 now summarized the principal methodological-quality and reporting concerns, and S2 provided study-level judgments and supporting explanations.
Results	19	Present results for individual studies and outcomes.	Sections 3.3-3.5.7; Tables 2-5; Figure 5; Supplementary Tables S1 and S3.	**Reported.** Individual-study characteristics, outcomes, effect sizes, and reasons for non-estimability were presented.
Results	20a	Summarize the results of each synthesis.	Sections 3.3-3.6; Tables 3-5; Figures 2-6.	**Reported.** Each strategy-level and outcome-domain synthesis was summarized.
Results	20b	Present statistical synthesis results if meta-analysis was conducted.	Not applicable; Sections 2.13 and 2.14.	**Not applicable.** No meta-analysis or pooled summary estimate was conducted.
Results	20c	Present results of investigations of heterogeneity if performed.	Section 3.5.7; Supplementary Table S5.	Reported descriptively. Subgroup distributions and ranges were presented with clear cautions about confounding and post hoc interpretation.
Results	20d	Present sensitivity analyses if performed.	Not applicable.	**Not applicable.** No formal sensitivity analysis was performed.
Results	21	Present assessments of risk of bias due to missing results.	No formal assessment is reported in Results; possible publication bias and incomplete reporting are acknowledged in Section 4.11.	**Reported. N**o formal assessment of bias due to missing results was performed and direct readers to the qualitative limitations discussed in Section 4.11.

PRISMA 2020 Checklist - Revised Assessment

Section	Item	Reporting item (summarized)	Location in manuscript / supplement	Status and comments
Results	22	Present certainty or confidence in the evidence.	Sections 3.2.1, 3.3.1, 3.5.5-3.5.8, 4.1, 4.3, 4.4, 4.10, and 4.11; Supplementary Table S2.	**Reported.** Confidence and limitations were presented narratively, including methodological concerns, imprecision, consistency, causal-pathway completeness, contextual dependence, and evidence gaps.
Discussion	23a	Provide a general interpretation of results in the context of other evidence.	Sections 4.1-4.4.	**Reported.**
Discussion	23b	Discuss limitations of the included evidence.	Sections 4.10 and 4.11; Sections 3.5.5-3.5.7.	**Reported.** Limitations of the included evidence were discussed.
Discussion	23c	Discuss limitations of the review process.	Section 4.11.	**Reported.** Limitations of the review process, including database omissions, retrieval/publication bias, heterogeneity, subgroup confounding, and restricted causal inference, were discussed.
Discussion	23d	Discuss implications for practice, policy, and future research.	Sections 3.6, 4.7-4.10, and Conclusions.	**Reported.** Implications for formulation, commercial adoption, regulation/safety, and future research were described.
Other information	24a	Provide registration information or state that the protocol was not registered.	Section 2.1.	**Reported.** The protocol was retrospectively registered on OSF (DOI: 10.17605/OSF.IO/RCUZX).
Other information	24b	Indicate where the protocol can be accessed.	Section 2.1; OSF DOI.	**Reported.** The protocol and registration record are publicly accessible through the OSF DOI.
Other information	24c	Describe and explain amendments to the protocol.	Section 2.1; OSF registration record.	**Reported.** The protocol was retrospectively registered and is accessible, but post-registration amendments are not described. No amendments were made after OSF registration.
Other information	25	Describe sources of support and funder role.	Funding section.	**Reported.**
Other information	26	Declare competing interests.	Conflicts of Interest section.	**Reported.** The authors declared no conflicts of interest.
Other information	27	Report availability of data, code, and materials.	Data Availability Statement; Supplementary Materials; Supplementary Tables S1-S5; OSF record.	**Reported.** The original contributions presented in this study are included in the article/supplementary material.

Assessment of revised manuscript (24 July 2026): *28 items are reported, 3 are not applicable, and 11 require minor additions or clarification before submission. Yellow status cells indicate items needing attention.*

Reference for checklist framework: Page MJ, McKenzie JE, Bossuyt PM, Boutron I, Hoffmann TC, Mulrow CD, et al. The PRISMA 2020 statement: an updated guideline for reporting systematic reviews. BMJ. 2021;372:n71.

Note: This assessment is based on the revised manuscript and the supplied checklist. Supplementary Tables S1-S5 were referenced in the manuscript but were not independently re-audited here.
