# Supplementary material for: Nutritional Balancing Strategies in Low-Fishmeal Aquafeeds: A PRISMA 2020-Based Systematic Review Beyond Lysine and Methionine Supplementation
Source: Vet Sci. 2026 Jul 30;13(8):762. doi: 10.3390/vetsci13080762 (PMC13517252; doi:10.3390/vetsci13080762)
Supplement: Supplementary file 1 [file vetsci-13-00762-s001.zip › Supplementary_Tables_S1.pdf]

# Supplementary Materials

**Manuscript title:** Nutritional balancing strategies in low-fishmeal aquafeeds: A PRISMA 2020-based systematic review beyond lysine and methionine supplementation

**Table S1. Expanded study-level extraction and evidence-role matrix of the 23 feeding trials included in the qualitative synthesis.**

| Study | Species | Diet model / context | Primary balancing strategy | Extractable outcome domains | Main response pattern | Evidence role |
|---|---|---|---|---|---|---|
| Gunathilaka et al. (2019) | Red seabream (*Pagrus major*) | Low-fishmeal, soybean meal-based diet during low-temperature season | Taurine; supplementation strategy reported in study | Growth, feed intake, FCR, PER, immune indicators, hepatic IGF-I | Positive/context-dependent: improved growth and feed utilization, with immune and endocrine relevance | Functional nutrient restoration; low-temperature resilience |
| Kim et al. (2017) | Olive flounder (*Paralichthys olivaceus*) | Taurine-supplemented diet under low water temperature | Taurine; supplementation strategy reported in study | Growth, FCR, hematology, cholesterol, innate immune indicators | Positive/context-dependent: improved feed utilization, hematological status, and immune-related indicators | Taurine-related physiological and immune support |
| Kotzamanis et al. (2020) | European seabass (*Dicentrarchus labrax*) | Plant-protein-based diet | High taurine supplementation | Growth, organoleptic/product-quality traits | Positive/context-dependent: growth and product-quality relevance | Taurine support in high plant-protein formulation |
| Watson et al. (2013) | Gilthead seabream (*Sparus aurata*) | Plant-protein-based diets with alternative lipid sources | Taurine with alternative lipid-source context | Growth and lipid-source-related outcomes | Context-dependent: relevant to taurine-lipid interaction | Taurine and lipid-associated nutrient context |
| Yamamoto et al. (2019) | Yellowtail (*Seriola quinqueradiata*) | Fishmeal-based and low-fishmeal diets | Histidine | Growth, feed intake, tissue free amino acids | Mixed: limited growth restoration but clear metabolic/free amino acid relevance | Secondary amino acid balance |
| Ramos-Pinto et al. (2019) | Gilthead seabream (*Sparus aurata*) | Fishmeal-free diet | Tryptophan | Immune status, short-term functional responses | Transient positive immune-related response | Functional amino acid and immunity |
| Ramos-Pinto et al. (2021) | Gilthead seabream (*Sparus aurata*) | Fishmeal-free diet | Histidine, threonine, or taurine | Immune status | Context-dependent: immune responses more informative than growth | Functional amino acid comparison |
| Wang et al. (2023) | Turbot (*Scophthalmus maximus*) | Dietary amino acid imbalance model relevant to high alternative-protein formulation | Branched-chain amino acid balance | Growth and amino acid metabolism | Mechanistic/contextual: supports amino acid-ratio interpretation | BCAA balance and antagonism |
| Shi et al. (2024) | Pacific white shrimp (*Litopenaeus vannamei*) | Low-fishmeal diet | Hydroxyproline | Collagen synthesis, myofiber development, muscle texture | Positive/context-dependent: collagen and texture-related benefits | Collagen-related nutrient support in shrimp |
| Cao et al. (2022) | Triploid crucian carp | Hydroxyproline-supplemented diet | Hydroxyproline | Growth, collagen, muscle texture, gene expression | Positive/contextual: collagen, growth, and product-quality relevance | Collagen and product-quality mechanism |
| Yoo and Bai (2014) | Olive flounder (*Paralichthys olivaceus*) | Soybean meal-based diet | Microbial phytase | Phosphorus digestibility, growth, body composition | Positive for phosphorus availability; growth response not consistently significant | Available phosphorus improvement |

| Study | Species | Diet model / context | Primary balancing strategy | Extractable outcome domains | Main response pattern | Evidence role |
|---|---|---|---|---|---|---|
| Terrey et al. (2024) | Atlantic salmon (*Salmo salar*) | High plant-meal diet | Microbial phytase | Growth, FCR, phosphorus digestibility, phosphorus retention, fecal phosphorus | Positive: improved performance, phosphorus utilization, and environmental output | Nutrient utilization and phosphorus-waste reduction |
| Li et al. (2019) | Hybrid tilapia | Low-phosphorus fishmeal-free diet | Phytase and protease | Growth and nutrient utilization | Positive/context-dependent: enzyme-based nutrient-use improvement | Enzyme-based digestibility strategy |
| Sajjadi and Carter (2004) | Atlantic salmon (*Salmo salar*) | Canola meal-based diet | Phytase | Phosphorus utilization | Positive/context-dependent: phytase-mediated phosphorus availability | Phytase and plant-protein mineral availability |
| Carter and Sajjadi (2011) | Atlantic salmon (*Salmo salar*) | Low-fishmeal diet using soy protein concentrate | Graded phytase treatment | Growth and phosphorus-related responses | Context-dependent: phytase treatment in low-fishmeal formulation | Phytase treatment and low-fishmeal formulation |
| Gunathilaka et al. (2021) | Red seabream (*Pagrus major*) | Low-fishmeal, high-plant-protein diet | Shrimp protein hydrolysate and krill meal | Growth, digestibility, immunity, antioxidant enzymes, IGF-I | Positive/context-dependent: hydrolysate and attractant strategy improved multiple domains | Palatability, digestibility, and hydrolysate strategy |
| Khosravi et al. (2018) | Olive flounder (*Paralichthys olivaceus*) | Low-fishmeal diet | Protein hydrolysates | Growth, health status | Positive/context-dependent: growth and health support | Hydrolysate supplementation |
| Egerton et al. (2020) | Atlantic salmon (Salmo salar) | Plant-protein diet | Fish protein hydrolysate | Growth, gut and microbial outcomes | Context-dependent: supports plant-protein diet performance and gut/microbial interpretation | Hydrolysate-supported fishmeal replacement |
| Bae et al. (2020) | Whiteleg shrimp (*Litopenaeus vannamei*) | Plant protein sources as fishmeal replacers | Fermented soybean meal/plant proteins | Growth, antioxidant response, lysozyme response | Mixed to positive: processed plant ingredient and health-related responses | Processed plant ingredient and shrimp health |
| Hamidoghli et al. (2023) | Whiteleg shrimp (*Litopenaeus vannamei*) | Low-fishmeal diet | Yeast hydrolysate | Growth, intestinal morphology, Vibrio challenge | Positive: growth, gut morphology, and disease resistance | Gut-health and disease-resistance strategy |
| Niu et al. (2019) | Olive flounder (*Paralichthys olivaceus*) | Low-fishmeal diet | Multi-strain probiotics | Growth, nutrient utilization, immunity, gut microbiota | Positive/context-dependent: growth, nutrient-use, immune, and microbiome responses | Microbiome-targeted strategy |
| Zhao et al. (2023) | Largemouth bass (*Micropterus salmoides*) | High soybean meal diet / plant-protein challenge | Tributyrin and alanyl-glutamine dipeptide | Intestinal health and immunity | Context-dependent: gut-protection strategy under plant-protein challenge | Gut integrity under plant-protein challenge |
| Teodosio et al. (2021) | Gilthead seabream (*Sparus aurata*) | Low-temperature nutritional strategy | Integrated nutritional strategy | Performance under low temperature | Context-dependent: performance support under low-temperature conditions | Multi-nutrient resilience strategy |

*Abbreviations: BCAA, branched-chain amino acid; FCR, feed conversion ratio; IGF-I, insulin-like growth factor-I; PER, protein efficiency ratio. Treatment dose and detailed diet-composition values should be checked against the original articles before final submission if numeric extraction is required by the journal.*
