# Supplementary material for: Nutritional Balancing Strategies in Low-Fishmeal Aquafeeds: A PRISMA 2020-Based Systematic Review Beyond Lysine and Methionine Supplementation
Source: Vet Sci. 2026 Jul 30;13(8):762. doi: 10.3390/vetsci13080762 (PMC13517252; doi:10.3390/vetsci13080762)
Supplement: Supplementary file 1 [file vetsci-13-00762-s001.zip › Supplementary_Tables_S2.pdf]

Table S2. Methodological quality and reporting assessment of the 23 included feeding trials.

Ratings were assigned to support interpretation of the systematic narrative synthesis. Studies were not excluded solely on quality unless essential eligibility information was missing. "Contextual" indicates that the study was relevant to the review framework but the low-fishmeal/high-alternative-protein connection should be clearly stated in the manuscript or extraction file.

Study	Species / life stage reporting	Replication / experimental unit	Diet and fishmeal-context reporting	Mechanism-endpoint alignment	Outcome reporting	Overall concern	Main interpretation note
Gunathilaka et al. (2019)	Clear	Clear/adequate	Clear	Good	Good	Low concern	Mechanism-aligned taurine and low-temperature endpoints
Kim et al. (2017)	Clear	Clear/adequate	Clear	Good	Good	Low concern	Useful physiological and immune endpoints
Kotzamanis et al. (2020)	Clear	Clear/adequate	Clear	Moderate	Good	Some concern	Product-quality outcomes useful; low-fishmeal context should be stated clearly
Watson et al. (2013)	Clear	Clear/adequate	Clear	Moderate	Moderate	Some concern	Useful taurine-lipid context; endpoint coverage is narrower
Yamamoto et al. (2019)	Clear	Clear/adequate	Clear	Good	Moderate	Some concern	Growth response limited; metabolic endpoints more informative
Ramos-Pinto et al. (2019)	Clear	Clear/adequate	Clear	Good	Moderate	Some concern	Short-term immune response; limited production endpoints
Ramos-Pinto et al. (2021)	Clear	Clear/adequate	Clear	Good	Moderate	Some concern	Immune-focused comparison; limited growth/digestibility endpoints
Wang et al. (2023)	Clear	Clear/adequate	Contextual	Moderate	Moderate	Some concern	Mechanistic amino acid-imbalance support; low-fishmeal eligibility should be clarified
Shi et al. (2024)	Clear	Clear/adequate	Clear	Good	Good	Low concern	Low-fishmeal shrimp and collagen/product endpoints align well
Cao et al. (2022)	Clear	Clear/adequate	Contextual	Good	Good	Some concern	Hydroxyproline mechanism is strong; low-fishmeal connection should be clarified
Yoo and Bai (2014)	Clear	Clear/adequate	Clear	Good	Good	Low concern	Phosphorus digestibility endpoint aligns with phytase mechanism
Terrey et al. (2024)	Clear	Clear/adequate	Clear	Good	Good	Low concern	Strong mechanism-aligned phosphorus and fecal-output endpoints

Study	Species / life stage reporting	Replication / experimental unit	Diet and fishmeal-context reporting	Mechanism-endpoint alignment	Outcome reporting	Overall concern	Main interpretation note
Li et al. (2019)	Clear	Clear/adequate	Clear	Good	Good	Low concern	Fishmeal-free/low-phosphorus enzyme strategy is clearly relevant
Sajjadi and Carter (2004)	Clear	Clear/adequate	Clear	Good	Moderate	Low concern	Phosphorus-utilization endpoint aligns with phytase mechanism
Carter and Sajjadi (2011)	Clear	Clear/adequate	Clear	Good	Moderate	Low concern	Low-fishmeal phytase treatment is clearly relevant
Gunathilaka et al. (2021)	Clear	Clear/adequate	Clear	Good	Good	Low concern	Multiple endpoints support palatability/digestibility/health interpretation
Khosravi et al. (2018)	Clear	Clear/adequate	Clear	Moderate	Moderate	Some concern	Hydrolysate response is useful; endpoint detail is less broad
Egerton et al. (2020)	Clear	Clear/adequate	Clear	Good	Good	Low concern	Gut/microbial endpoints strengthen hydrolysate interpretation
Bae et al. (2020)	Clear	Clear/adequate	Clear	Moderate	Good	Low concern	Fishmeal-replacement context in shrimp is clear
Hamidoghli et al. (2023)	Clear	Clear/adequate	Clear	Good	Good	Low concern	Gut morphology and Vibrio challenge strengthen veterinary relevance
Niu et al. (2019)	Clear	Clear/adequate	Clear	Good	Good	Low concern	Growth, immune, nutrient-use, and microbiota endpoints align well
Zhao et al. (2023)	Clear	Clear/adequate	Contextual	Good	Good	Some concern	High soybean meal gut-challenge model; low-fishmeal link should be stated
Teodosio et al. (2021)	Clear	Clear/adequate	Contextual	Moderate	Moderate	Some concern	Low-temperature resilience is relevant; low-fishmeal context should be clarified

Note: Low concern = the study provides sufficient information for inclusion and interpretation in the present qualitative synthesis; Some concern = the study is usable but has narrower endpoint coverage, more contextual relevance, or a reporting feature that should be interpreted cautiously.
