# Supplementary material for: Nutritional Balancing Strategies in Low-Fishmeal Aquafeeds: A PRISMA 2020-Based Systematic Review Beyond Lysine and Methionine Supplementation
Source: Vet Sci. 2026 Jul 30;13(8):762. doi: 10.3390/vetsci13080762 (PMC13517252; doi:10.3390/vetsci13080762)
Supplement: Supplementary file 1 [file vetsci-13-00762-s001.zip › Supplementary_Table_S3.pdf]

Supplementary Table S3. Study-level effect-size estimates and 95% confidence intervals for nutritional-balancing strategies in low-fishmeal aquafeeds.

Complete compilation of all 23 included studies. Study-level estimates are reported where sufficient numerical data were available; outcomes without sufficient numerical information are retained and annotated with the reason for non-estimability.

Study 1. Gunathilaka et al. (2019): matched taurine-supplemented versus non-supplemented low-fishmeal diets

Study	Species	Strategy	Intervention	Comparator	Outcome and unit	Intervention mean ± SD	Comparator mean ± SD	n intervention (tanks)	n comparator (tanks)	Effect-size metric	Effect size	95% CI	Interpretation	Reason not estimated
Gunathilaka et al. (2019)	Red seabream (Pagrus major)	1% dietary taurine supplementation	SM20-T (1% taurine; 20% FM replacement)	SM20 (0% taurine; 20% FM replacement)	Weight gain (%)	82.20 ± 5.10	69.50 ± 5.00	3	3	Hedges' g	2.01	-0.11 to 4.13	Higher measured value with taurine; 95% CI included 0 (imprecise estimate)	Not applicable—effect size estimated
Gunathilaka et al. (2019)	Red seabream (Pagrus major)	1% dietary taurine supplementation	SM20-T (1% taurine; 20% FM replacement)	SM20 (0% taurine; 20% FM replacement)	Feed intake (g/fish)	75.30 ± 3.70	71.40 ± 4.70	3	3	Hedges' g	0.74	-0.94 to 2.42	Higher measured value with taurine; 95% CI included 0 (imprecise estimate)	Not applicable—effect size estimated
Gunathilaka et al. (2019)	Red seabream (Pagrus major)	1% dietary taurine supplementation	SM20-T (1% taurine; 20% FM replacement)	SM20 (0% taurine; 20% FM replacement)	FCR	1.06 ± 0.02	1.39 ± 0.25	3	3	Hedges' g (direction reversed)	1.49	-0.42 to 3.39	Lower, more favorable FCR with taurine; 95% CI included 0 (imprecise estimate)	Not applicable—effect size estimated
Gunathilaka et al. (2019)	Red seabream (Pagrus major)	1% dietary taurine supplementation	SM20-T (1% taurine; 20% FM replacement)	SM20 (0% taurine; 20% FM replacement)	PER	1.96 ± 0.04	1.53 ± 0.29	3	3	Hedges' g	1.66	-0.31 to 3.63	Higher measured value with taurine; 95% CI included 0 (imprecise estimate)	Not applicable—effect size estimated
Gunathilaka et al. (2019)	Red seabream (Pagrus major)	1% dietary taurine supplementation	SM20-T (1% taurine; 20% FM replacement)	SM20 (0% taurine; 20% FM replacement)	Survival (%)	96.70 ± 2.90	91.70 ± 5.80	3	3	Hedges' g	0.87	-0.84 to 2.58	Higher measured value with taurine; 95% CI included 0 (imprecise estimate)	Not applicable—effect size estimated
Gunathilaka et al. (2019)	Red seabream (Pagrus major)	1% dietary taurine supplementation	SM20-T (1% taurine; 20% FM replacement)	SM20 (0% taurine; 20% FM replacement)	Antiprotease (% inhibition)	19.70 ± 0.30	18.80 ± 0.07	3	3	Hedges' g	3.31	0.51 to 6.10	Higher measured value with taurine; 95% CI excluded 0	Not applicable—effect size estimated
Gunathilaka et al. (2019)	Red seabream (Pagrus major)	1% dietary taurine supplementation	SM20-T (1% taurine; 20% FM replacement)	SM20 (0% taurine; 20% FM replacement)	GPx (mU/mL)	92.20 ± 1.71	85.80 ± 2.43	3	3	Hedges' g	2.44	0.11 to 4.76	Higher measured value with taurine; 95% CI excluded 0	Not applicable—effect size estimated
Gunathilaka et al. (2019)	Red seabream (Pagrus major)	1% dietary taurine supplementation	SM20-T (1% taurine; 20% FM replacement)	SM20 (0% taurine; 20% FM replacement)	Lysozyme (µg/mL)	5.10 ± 0.22	4.60 ± 0.25	3	3	Hedges' g	1.7	-0.29 to 3.69	Higher measured value with taurine; 95% CI included 0 (imprecise estimate)	Not applicable—effect size estimated
Gunathilaka et al. (2019)	Red seabream (Pagrus major)	1% dietary taurine supplementation	SM20-T (1% taurine; 20% FM replacement)	SM20 (0% taurine; 20% FM replacement)	MPO (reported unit)	1.42 ± 0.20	1.36 ± 0.06	3	3	Hedges' g	0.33	-1.29 to 1.94	Higher measured value with taurine; 95% CI included 0 (imprecise estimate)	Not applicable—effect size estimated
Gunathilaka et al. (2019)	Red seabream (Pagrus major)	1% dietary taurine supplementation	SM20-T (1% taurine; 20% FM replacement)	SM20 (0% taurine; 20% FM replacement)	NBT (reported unit)	0.91 ± 0.17	0.95 ± 0.09	3	3	Hedges' g	-0.24	-1.84 to 1.37	Lower measured value with taurine; 95% CI included 0 (imprecise estimate)	Not applicable—effect size estimated
Gunathilaka et al. (2019)	Red seabream (Pagrus major)	1% dietary taurine supplementation	SM20-T (1% taurine; 20% FM replacement)	SM20 (0% taurine; 20% FM replacement)	SOD (% inhibition)	67.20 ± 1.90	64.40 ± 1.50	3	3	Hedges' g	1.31	-0.53 to 3.15	Higher measured value with taurine; 95% CI included 0 (imprecise estimate)	Not applicable—effect size estimated
Gunathilaka et al. (2019)	Red seabream (Pagrus major)	1% dietary taurine supplementation	SM20-T (1% taurine; 20% FM replacement)	SM20 (0% taurine; 20% FM replacement)	Total Ig (mg/mL)	21.60 ± 0.83	20.20 ± 0.40	3	3	Hedges' g	1.72	-0.28 to 3.71	Higher measured value with taurine; 95% CI included 0 (imprecise estimate)	Not applicable—effect size estimated
Gunathilaka et al. (2019)	Red seabream (Pagrus major)	1% dietary taurine supplementation	SM35-T (1% taurine; 35% FM replacement)	SM35 (0% taurine; 35% FM replacement)	Weight gain (%)	78.10 ± 4.00	64.80 ± 1.10	3	3	Hedges' g	3.63	0.65 to 6.61	Higher measured value with taurine; 95% CI excluded 0	Not applicable—effect size estimated
Gunathilaka et al. (2019)	Red seabream (Pagrus major)	1% dietary taurine supplementation	SM35-T (1% taurine; 35% FM replacement)	SM35 (0% taurine; 35% FM replacement)	Feed intake (g/fish)	73.60 ± 9.80	66.50 ± 4.10	3	3	Hedges' g	0.76	-0.93 to 2.44	Higher measured value with taurine; 95% CI included 0 (imprecise estimate)	Not applicable—effect size estimated
Gunathilaka et al. (2019)	Red seabream (Pagrus major)	1% dietary taurine supplementation	SM35-T (1% taurine; 35% FM replacement)	SM35 (0% taurine; 35% FM replacement)	FCR	1.14 ± 0.04	1.25 ± 0.15	3	3	Hedges' g (direction reversed)	0.8	-0.89 to 2.50	Lower, more favorable FCR with taurine; 95% CI included 0 (imprecise estimate)	Not applicable—effect size estimated
Gunathilaka et al. (2019)	Red seabream (Pagrus major)	1% dietary taurine supplementation	SM35-T (1% taurine; 35% FM replacement)	SM35 (0% taurine; 35% FM replacement)	PER	1.82 ± 0.07	1.68 ± 0.18	3	3	Hedges' g	0.82	-0.88 to 2.52	Higher measured value with taurine; 95% CI included 0 (imprecise estimate)	Not applicable—effect size estimated
Gunathilaka et al. (2019)	Red seabream (Pagrus major)	1% dietary taurine supplementation	SM35-T (1% taurine; 35% FM replacement)	SM35 (0% taurine; 35% FM replacement)	Survival (%)	96.70 ± 5.80	96.70 ± 5.80	3	3	Hedges' g	0	-1.60 to 1.60	No observed mean difference; 95% CI included 0 (imprecise estimate)	Not applicable—effect size estimated
Gunathilaka et al. (2019)	Red seabream (Pagrus major)	1% dietary taurine supplementation	SM35-T (1% taurine; 35% FM replacement)	SM35 (0% taurine; 35% FM replacement)	Antiprotease (% inhibition)	19.60 ± 0.79	17.10 ± 1.23	3	3	Hedges' g	1.93	-0.15 to 4.02	Higher measured value with taurine; 95% CI included 0 (imprecise estimate)	Not applicable—effect size estimated
Gunathilaka et al. (2019)	Red seabream (Pagrus major)	1% dietary taurine supplementation	SM35-T (1% taurine; 35% FM replacement)	SM35 (0% taurine; 35% FM replacement)	GPx (mU/mL)	87.60 ± 0.76	82.50 ± 3.47	3	3	Hedges' g	1.62	-0.33 to 3.58	Higher measured value with taurine; 95% CI included 0 (imprecise estimate)	Not applicable—effect size estimated
Gunathilaka et al. (2019)	Red seabream (Pagrus major)	1% dietary taurine supplementation	SM35-T (1% taurine; 35% FM replacement)	SM35 (0% taurine; 35% FM replacement)	Lysozyme (µg/mL)	5.00 ± 0.08	4.70 ± 0.10	3	3	Hedges' g	2.65	0.21 to 5.09	Higher measured value with taurine; 95% CI excluded 0	Not applicable—effect size estimated
Gunathilaka et al. (2019)	Red seabream (Pagrus major)	1% dietary taurine supplementation	SM35-T (1% taurine; 35% FM replacement)	SM35 (0% taurine; 35% FM replacement)	MPO (reported unit)	1.37 ± 0.03	1.31 ± 0.05	3	3	Hedges' g	1.16	-0.63 to 2.96	Higher measured value with taurine; 95% CI included 0 (imprecise estimate)	Not applicable—effect size estimated
Gunathilaka et al. (2019)	Red seabream (Pagrus major)	1% dietary taurine supplementation	SM35-T (1% taurine; 35% FM replacement)	SM35 (0% taurine; 35% FM replacement)	NBT (reported unit)	0.94 ± 0.01	0.88 ± 0.07	3	3	Hedges' g	0.96	-0.77 to 2.69	Higher measured value with taurine; 95% CI included 0 (imprecise estimate)	Not applicable—effect size estimated
Gunathilaka et al. (2019)	Red seabream (Pagrus major)	1% dietary taurine supplementation	SM35-T (1% taurine; 35% FM replacement)	SM35 (0% taurine; 35% FM replacement)	SOD (% inhibition)	64.60 ± 1.60	62.60 ± 0.60	3	3	Hedges' g	1.32	-0.52 to 3.17	Higher measured value with taurine; 95% CI included 0 (imprecise estimate)	Not applicable—effect size estimated
Gunathilaka et al. (2019)	Red seabream (Pagrus major)	1% dietary taurine supplementation	SM35-T (1% taurine; 35% FM replacement)	SM35 (0% taurine; 35% FM replacement)	Total Ig (mg/mL)	21.30 ± 1.57	19.90 ± 2.00	3	3	Hedges' g	0.62	-1.03 to 2.28	Higher measured value with taurine; 95% CI included 0 (imprecise estimate)	Not applicable—effect size estimated
Gunathilaka et al. (2019)	Red seabream (Pagrus major)	1% dietary taurine supplementation	SM50-T (1% taurine; 50% FM replacement)	SM50 (0% taurine; 50% FM replacement)	Weight gain (%)	70.80 ± 2.70	56.70 ± 7.10	3	3	Hedges' g	2.1	-0.06 to 4.26	Higher measured value with taurine; 95% CI included 0 (imprecise estimate)	Not applicable—effect size estimated
Gunathilaka et al. (2019)	Red seabream (Pagrus major)	1% dietary taurine supplementation	SM50-T (1% taurine; 50% FM replacement)	SM50 (0% taurine; 50% FM replacement)	Feed intake (g/fish)	65.40 ± 1.30	62.60 ± 3.30	3	3	Hedges' g	0.89	-0.82 to 2.61	Higher measured value with taurine; 95% CI included 0 (imprecise estimate)	Not applicable—effect size estimated
Gunathilaka et al. (2019)	Red seabream (Pagrus major)	1% dietary taurine supplementation	SM50-T (1% taurine; 50% FM replacement)	SM50 (0% taurine; 50% FM replacement)	FCR	1.46 ± 0.27	1.62 ± 0.07	3	3	Hedges' g (direction reversed)	0.65	-1.01 to 2.31	Lower, more favorable FCR with taurine; 95% CI included 0 (imprecise estimate)	Not applicable—effect size estimated
Gunathilaka et al. (2019)	Red seabream (Pagrus major)	1% dietary taurine supplementation	SM50-T (1% taurine; 50% FM replacement)	SM50 (0% taurine; 50% FM replacement)	PER	1.46 ± 0.30	1.29 ± 0.06	3	3	Hedges' g	0.63	-1.03 to 2.29	Higher measured value with taurine; 95% CI included 0 (imprecise estimate)	Not applicable—effect size estimated
Gunathilaka et al. (2019)	Red seabream (Pagrus major)	1% dietary taurine supplementation	SM50-T (1% taurine; 50% FM replacement)	SM50 (0% taurine; 50% FM replacement)	Survival (%)	88.30 ± 7.60	95.00 ± 5.00	3	3	Hedges' g	-0.83	-2.53 to 0.87	Lower measured value with taurine; 95% CI included 0 (imprecise estimate)	Not applicable—effect size estimated
Gunathilaka et al. (2019)	Red seabream (Pagrus major)	1% dietary taurine supplementation	SM50-T (1% taurine; 50% FM replacement)	SM50 (0% taurine; 50% FM replacement)	Antiprotease (% inhibition)	16.70 ± 1.56	15.40 ± 1.68	3	3	Hedges' g	0.64	-1.02 to 2.30	Higher measured value with taurine; 95% CI included 0 (imprecise estimate)	Not applicable—effect size estimated
Gunathilaka et al. (2019)	Red seabream (Pagrus major)	1% dietary taurine supplementation	SM50-T (1% taurine; 50% FM replacement)	SM50 (0% taurine; 50% FM replacement)	GPx (mU/mL)	84.00 ± 3.39	81.00 ± 2.29	3	3	Hedges' g	0.83	-0.87 to 2.53	Higher measured value with taurine; 95% CI included 0 (imprecise estimate)	Not applicable—effect size estimated
Gunathilaka et al. (2019)	Red seabream (Pagrus major)	1% dietary taurine supplementation	SM50-T (1% taurine; 50% FM replacement)	SM50 (0% taurine; 50% FM replacement)	Lysozyme (µg/mL)	4.70 ± 0.09	4.20 ± 0.48	3	3	Hedges' g	1.16	-0.63 to 2.95	Higher measured value with taurine; 95% CI included 0 (imprecise estimate)	Not applicable—effect size estimated
Gunathilaka et al. (2019)	Red seabream (Pagrus major)	1% dietary taurine supplementation	SM50-T (1% taurine; 50% FM replacement)	SM50 (0% taurine; 50% FM replacement)	MPO (reported unit)	1.32 ± 0.02	1.29 ± 0.05	3	3	Hedges' g	0.63	-1.03 to 2.29	Higher measured value with taurine; 95% CI included 0 (imprecise estimate)	Not applicable—effect size estimated
Gunathilaka et al. (2019)	Red seabream (Pagrus major)	1% dietary taurine supplementation	SM50-T (1% taurine; 50% FM replacement)	SM50 (0% taurine; 50% FM replacement)	NBT (reported unit)	0.90 ± 0.19	0.98 ± 0.05	3	3	Hedges' g	-0.46	-2.09 to 1.17	Lower measured value with taurine; 95% CI included 0 (imprecise estimate)	Not applicable—effect size estimated
Gunathilaka et al. (2019)	Red seabream (Pagrus major)	1% dietary taurine supplementation	SM50-T (1% taurine; 50% FM replacement)	SM50 (0% taurine; 50% FM replacement)	SOD (% inhibition)	60.60 ± 1.80	59.60 ± 0.80	3	3	Hedges' g	0.57	-1.07 to 2.22	Higher measured value with taurine; 95% CI included 0 (imprecise estimate)	Not applicable—effect size estimated
Gunathilaka et al. (2019)	Red seabream (Pagrus major)	1% dietary taurine supplementation	SM50-T (1% taurine; 50% FM replacement)	SM50 (0% taurine; 50% FM replacement)	Total Ig (mg/mL)	21.30 ± 0.29	19.80 ± 2.15	3	3	Hedges' g	0.78	-0.91 to 2.47	Higher measured value with taurine; 95% CI included 0 (imprecise estimate)	Not applicable—effect size estimated
Gunathilaka et al.	Red seabream (Pagrus	1% dietary taurine	SM20-T (1% taurine; 20% FM	SM20 (0% taurine; 20% FM	Hepatic IGF-I mRNA	Not reported	Not reported	3	3	Not estimable	Not	Not estimable	The original study reported higher IGF-I	Exact group means and SDs were presented

Study	Species	Strategy	Intervention	Comparator	Outcome and unit	Intervention mean ± SD	Comparator mean ± SD	n intervention (tanks)	n comparator (tanks)	Effect-size metric	Effect size	95% CI	Interpretation	Reason not estimated
(2019)	major)	supplementation	replacement)	replacement)	expression	numerically	numerically				estimable		expression with taurine relative to the corresponding unsupplemented diet	only graphically and were not reliably extractable
Gunathilaka et al. (2019)	Red seabream (Pagrus major)	1% dietary taurine supplementation	SM35-T (1% taurine; 35% FM replacement)	SM35 (0% taurine; 35% FM replacement)	Hepatic IGF-I mRNA expression	Not reported numerically	Not reported numerically	3	3	Not estimable	Not estimable	Not estimable	The original study reported higher IGF-I expression with taurine relative to the corresponding unsupplemented diet	Exact group means and SDs were presented only graphically and were not reliably extractable
Gunathilaka et al. (2019)	Red seabream (Pagrus major)	1% dietary taurine supplementation	SM50-T (1% taurine; 50% FM replacement)	SM50 (0% taurine; 50% FM replacement)	Hepatic IGF-I mRNA expression	Not reported numerically	Not reported numerically	3	3	Not estimable	Not estimable	Not estimable	The original study reported higher IGF-I expression with taurine relative to the corresponding unsupplemented diet	Exact group means and SDs were presented only graphically and were not reliably extractable

Note: Continuous outcomes were summarized using Hedges' g and 95% confidence intervals. The replicate tank was treated as the experimental unit (n = 3 per treatment). Effect directions were standardized so that positive values favored the nutritional-balancing intervention; for FCR, the sign was reversed because lower values indicate improvement. Survival was treated as a continuous tank-level percentage because tank-specific event counts were unavailable. Effect sizes were not estimated when exact numerical means and SDs were not reported. Unadjusted pairwise confidence intervals may not correspond exactly to the original one-way ANOVA and Tukey HSD results.

Source: Gunathilaka et al. (2019), https://doi.org/10.1186/s41240-019-0138-z

Study 2. Kim et al. (2017): graded taurine supplementation in olive flounder at low water temperature

Study	Species	Strategy	Intervention	Comparator	Outcome and unit	Intervention mean ± SD	Comparator mean ± SD	n intervention	n comparator	Effect-size metric	Effect size	95% CI	Interpretation	Reason not estimated
Kim et al. (2017)	Olive flounder (Paralichthys olivaceus)	Graded taurine supplementation (0.25–1.50%) under low-temperature conditions	T1 (0.25% added taurine; analyzed 0.33%)	Control (0% added taurine; analyzed 0.12%)	Final body weight (g)	41.70 ± 3.70	38.10 ± 0.66	3	3	Hedges' g (LS variance)	1.08	-0.63 to 2.79	Higher growth outcome with taurine; 95% CI included 0 (imprecise estimate)	Not applicable—effect size estimated
Kim et al. (2017)	Olive flounder (Paralichthys olivaceus)	Graded taurine supplementation (0.25–1.50%) under low-temperature conditions	T1 (0.25% added taurine; analyzed 0.33%)	Control (0% added taurine; analyzed 0.12%)	Weight gain (%)	113.00 ± 18.90	96.50 ± 3.39	3	3	Hedges' g (LS variance)	0.97	-0.72 to 2.66	Higher growth outcome with taurine; 95% CI included 0 (imprecise estimate)	Not applicable—effect size estimated
Kim et al. (2017)	Olive flounder (Paralichthys olivaceus)	Graded taurine supplementation (0.25–1.50%) under low-temperature conditions	T1 (0.25% added taurine; analyzed 0.33%)	Control (0% added taurine; analyzed 0.12%)	Specific growth rate (%/day)	1.13 ± 0.13	1.01 ± 0.03	3	3	Hedges' g (LS variance)	1.01	-0.69 to 2.72	Higher growth outcome with taurine; 95% CI included 0 (imprecise estimate)	Not applicable—effect size estimated
Kim et al. (2017)	Olive flounder (Paralichthys olivaceus)	Graded taurine supplementation (0.25–1.50%) under low-temperature conditions	T1 (0.25% added taurine; analyzed 0.33%)	Control (0% added taurine; analyzed 0.12%)	Feed intake (g/fish)	35.10 ± 5.85	30.60 ± 1.26	3	3	Hedges' g (LS variance)	0.85	-0.82 to 2.52	Higher feed intake with taurine; 95% CI included 0 (imprecise estimate)	Not applicable—effect size estimated
Kim et al. (2017)	Olive flounder (Paralichthys olivaceus)	Graded taurine supplementation (0.25–1.50%) under low-temperature conditions	T1 (0.25% added taurine; analyzed 0.33%)	Control (0% added taurine; analyzed 0.12%)	FCR	1.58 ± 0.00	1.63 ± 0.02	3	3	Hedges' g (direction reversed; LS variance)	2.82	0.56 to 5.08	Lower, more favorable FCR with taurine; intervention SD was reported as 0.00, so the standardized estimate may be inflated by rounding; 95% CI excluded 0	Not applicable—effect size estimated
Kim et al. (2017)	Olive flounder (Paralichthys olivaceus)	Graded taurine supplementation (0.25–1.50%) under low-temperature conditions	T1 (0.25% added taurine; analyzed 0.33%)	Control (0% added taurine; analyzed 0.12%)	PER	1.37 ± 0.00	1.32 ± 0.01	3	3	Hedges' g (LS variance)	5.64	2.07 to 9.21	Higher, more favorable PER with taurine; intervention SD was reported as 0.00, so the standardized estimate may be inflated by rounding; 95% CI excluded 0	Not applicable—effect size estimated
Kim et al. (2017)	Olive flounder (Paralichthys olivaceus)	Graded taurine supplementation (0.25–1.50%) under low-temperature conditions	T1 (0.25% added taurine; analyzed 0.33%)	Control (0% added taurine; analyzed 0.12%)	Survival (%)	57.20 ± 14.30	60.00 ± 5.71	3	3	Hedges' g (LS variance)	-0.21	-1.81 to 1.40	Lower survival with taurine; 95% CI included 0 (imprecise estimate)	Not applicable—effect size estimated
Kim et al. (2017)	Olive flounder (Paralichthys olivaceus)	Graded taurine supplementation (0.25–1.50%) under low-temperature conditions	T1 (0.25% added taurine; analyzed 0.33%)	Control (0% added taurine; analyzed 0.12%)	Hematocrit (%)	20.10 ± 0.56	15.80 ± 1.58	3	3	Hedges' g (LS variance)	2.89	0.60 to 5.18	Higher hematological value with taurine; 95% CI excluded 0	Not applicable—effect size estimated
Kim et al. (2017)	Olive flounder (Paralichthys olivaceus)	Graded taurine supplementation (0.25–1.50%) under low-temperature conditions	T1 (0.25% added taurine; analyzed 0.33%)	Control (0% added taurine; analyzed 0.12%)	Hemoglobin (g/dL)	3.54 ± 0.17	2.81 ± 0.17	3	3	Hedges' g (LS variance)	3.43	0.91 to 5.94	Higher hematological value with taurine; 95% CI excluded 0	Not applicable—effect size estimated
Kim et al. (2017)	Olive flounder (Paralichthys olivaceus)	Graded taurine supplementation (0.25–1.50%) under low-temperature conditions	T1 (0.25% added taurine; analyzed 0.33%)	Control (0% added taurine; analyzed 0.12%)	Glucose (mg/dL)	48.90 ± 2.87	53.60 ± 5.66	3	3	Hedges' g (LS variance)	-0.84	-2.50 to 0.83	Lower measured value with taurine; 95% CI included 0 (imprecise estimate)	Not applicable—effect size estimated
Kim et al. (2017)	Olive flounder (Paralichthys olivaceus)	Graded taurine supplementation (0.25–1.50%) under low-temperature conditions	T1 (0.25% added taurine; analyzed 0.33%)	Control (0% added taurine; analyzed 0.12%)	Total protein (g/dL)	3.86 ± 0.04	3.86 ± 0.26	3	3	Hedges' g (LS variance)	0.00	-1.60 to 1.60	No observed mean difference; 95% CI included 0 (imprecise estimate)	Not applicable—effect size estimated
Kim et al. (2017)	Olive flounder (Paralichthys olivaceus)	Graded taurine supplementation (0.25–1.50%) under low-temperature conditions	T1 (0.25% added taurine; analyzed 0.33%)	Control (0% added taurine; analyzed 0.12%)	Total cholesterol (mg/dL)	68.30 ± 10.30	82.00 ± 2.53	3	3	Hedges' g (LS variance)	-1.46	-3.26 to 0.34	Lower measured plasma cholesterol with taurine; 95% CI included 0 (imprecise estimate)	Not applicable—effect size estimated
Kim et al. (2017)	Olive flounder (Paralichthys olivaceus)	Graded taurine supplementation (0.25–1.50%) under low-temperature conditions	T1 (0.25% added taurine; analyzed 0.33%)	Control (0% added taurine; analyzed 0.12%)	NBT (reported unit)	0.51 ± 0.01	0.41 ± 0.01	3	3	Hedges' g (LS variance)	7.98	3.19 to 12.77	Higher measured immune/antioxidant response with taurine; 95% CI excluded 0	Not applicable—effect size estimated
Kim et al. (2017)	Olive flounder (Paralichthys olivaceus)	Graded taurine supplementation (0.25–1.50%) under low-temperature conditions	T1 (0.25% added taurine; analyzed 0.33%)	Control (0% added taurine; analyzed 0.12%)	MPO (reported unit)	1.80 ± 0.09	1.39 ± 0.08	3	3	Hedges' g (LS variance)	3.84	1.14 to 6.54	Higher measured immune/antioxidant response with taurine; 95% CI excluded 0	Not applicable—effect size estimated
Kim et al. (2017)	Olive flounder (Paralichthys olivaceus)	Graded taurine supplementation (0.25–1.50%) under low-temperature conditions	T1 (0.25% added taurine; analyzed 0.33%)	Control (0% added taurine; analyzed 0.12%)	SOD (% inhibition)	75.00 ± 2.56	70.60 ± 2.50	3	3	Hedges' g (LS variance)	1.39	-0.39 to 3.17	Higher measured immune/antioxidant response with taurine; 95% CI included 0 (imprecise estimate)	Not applicable—effect size estimated
Kim et al. (2017)	Olive flounder (Paralichthys olivaceus)	Graded taurine supplementation (0.25–1.50%) under low-temperature conditions	T1 (0.25% added taurine; analyzed 0.33%)	Control (0% added taurine; analyzed 0.12%)	Total Ig (mg/mL)	58.40 ± 1.10	20.40 ± 0.35	3	3	Hedges' g (LS variance)	37.15	16.07 to 58.22	Higher measured immune/antioxidant response with taurine; 95% CI excluded 0	Not applicable—effect size estimated
Kim et al. (2017)	Olive flounder (Paralichthys olivaceus)	Graded taurine supplementation (0.25–1.50%) under low-temperature conditions	T1 (0.25% added taurine; analyzed 0.33%)	Control (0% added taurine; analyzed 0.12%)	Lysozyme (μg/mL)	16.90 ± 0.11	15.30 ± 0.70	3	3	Hedges' g (LS variance)	2.55	0.39 to 4.70	Higher measured immune/antioxidant response with taurine; 95% CI excluded 0	Not applicable—effect size estimated
Kim et al. (2017)	Olive flounder (Paralichthys olivaceus)	Graded taurine supplementation (0.25–1.50%) under low-temperature conditions	T1 (0.25% added taurine; analyzed 0.33%)	Control (0% added taurine; analyzed 0.12%)	Antiprotease (% inhibition)	20.60 ± 0.61	13.80 ± 0.63	3	3	Hedges' g (LS variance)	8.75	3.55 to 13.95	Higher measured immune/antioxidant response with taurine; 95% CI excluded 0	Not applicable—effect size estimated
Kim et al. (2017)	Olive flounder (Paralichthys olivaceus)	Graded taurine supplementation (0.25–1.50%) under low-temperature conditions	T1 (0.25% added taurine; analyzed 0.33%)	Control (0% added taurine; analyzed 0.12%)	Whole-body dry matter (% DM)	76.10 ± 0.45	75.40 ± 0.53	3	3	Hedges' g (LS variance)	1.14	-0.59 to 2.86	Higher measured value with taurine; 95% CI included 0 (imprecise estimate)	Not applicable—effect size estimated
Kim et al. (2017)	Olive flounder (Paralichthys olivaceus)	Graded taurine supplementation (0.25–1.50%) under low-temperature conditions	T1 (0.25% added taurine; analyzed 0.33%)	Control (0% added taurine; analyzed 0.12%)	Whole-body protein (% DM)	69.20 ± 1.80	66.40 ± 0.74	3	3	Hedges' g (LS variance)	1.62	-0.22 to 3.47	Higher measured value with taurine; 95% CI included 0 (imprecise estimate)	Not applicable—effect size estimated
Kim et al. (2017)	Olive flounder (Paralichthys olivaceus)	Graded taurine supplementation (0.25–1.50%) under low-temperature conditions	T1 (0.25% added taurine; analyzed 0.33%)	Control (0% added taurine; analyzed 0.12%)	Whole-body lipid (% DM)	23.90 ± 1.92	25.70 ± 1.60	3	3	Hedges' g (LS variance)	-0.81	-2.48 to 0.85	Lower measured value with taurine; 95% CI included 0 (imprecise estimate)	Not applicable—effect size estimated
Kim et al. (2017)	Olive flounder (Paralichthys olivaceus)	Graded taurine supplementation (0.25–1.50%) under low-temperature conditions	T1 (0.25% added taurine; analyzed 0.33%)	Control (0% added taurine; analyzed 0.12%)	Whole-body ash (% DM)	11.80 ± 2.85	14.20 ± 0.06	3	3	Hedges' g (LS variance)	-0.95	-2.64 to 0.74	Lower measured value with taurine; 95% CI included 0 (imprecise estimate)	Not applicable—effect size estimated
Kim et al. (2017)	Olive flounder (Paralichthys olivaceus)	Graded taurine supplementation (0.25–1.50%) under low-temperature conditions	T2 (0.50% added taurine; analyzed 0.42%)	Control (0% added taurine; analyzed 0.12%)	Final body weight (g)	39.80 ± 7.89	38.10 ± 0.66	3	3	Hedges' g (LS variance)	0.24	-1.36 to 1.85	Higher growth outcome with taurine; 95% CI included 0 (imprecise estimate)	Not applicable—effect size estimated

Study	Species	Strategy	Intervention	Comparator	Outcome and unit	Intervention mean ± SD	Comparator mean ± SD	n intervention	n comparator	Effect-size metric	Effect size	95% CI	Interpretation	Reason not estimated
Kim et al. (2017)	Olive flounder (Paralichthys olivaceus)	Graded taurine supplementation (0.25–1.50%) under low-temperature conditions	T2 (0.50% added taurine; analyzed 0.42%)	Control (0% added taurine; analyzed 0.12%)	Weight gain (%)	103.00 ± 40.30	96.50 ± 3.39	3	3	Hedges' g (LS variance)	0.18	-1.42 to 1.78	Higher growth outcome with taurine; 95% CI included 0 (imprecise estimate)	Not applicable—effect size estimated
Kim et al. (2017)	Olive flounder (Paralichthys olivaceus)	Graded taurine supplementation (0.25–1.50%) under low-temperature conditions	T2 (0.50% added taurine; analyzed 0.42%)	Control (0% added taurine; analyzed 0.12%)	Specific growth rate (%/day)	1.04 ± 0.30	1.01 ± 0.03	3	3	Hedges' g (LS variance)	0.11	-1.49 to 1.71	Higher growth outcome with taurine; 95% CI included 0 (imprecise estimate)	Not applicable—effect size estimated
Kim et al. (2017)	Olive flounder (Paralichthys olivaceus)	Graded taurine supplementation (0.25–1.50%) under low-temperature conditions	T2 (0.50% added taurine; analyzed 0.42%)	Control (0% added taurine; analyzed 0.12%)	Feed intake (g/fish)	30.80 ± 12.13	30.60 ± 1.26	3	3	Hedges' g (LS variance)	0.02	-1.58 to 1.62	Higher feed intake with taurine; 95% CI included 0 (imprecise estimate)	Not applicable—effect size estimated
Kim et al. (2017)	Olive flounder (Paralichthys olivaceus)	Graded taurine supplementation (0.25–1.50%) under low-temperature conditions	T2 (0.50% added taurine; analyzed 0.42%)	Control (0% added taurine; analyzed 0.12%)	FCR	1.52 ± 0.01	1.63 ± 0.02	3	3	Hedges' g (direction reversed; LS variance)	5.55	2.03 to 9.08	Lower, more favorable FCR with taurine; 95% CI excluded 0	Not applicable—effect size estimated
Kim et al. (2017)	Olive flounder (Paralichthys olivaceus)	Graded taurine supplementation (0.25–1.50%) under low-temperature conditions	T2 (0.50% added taurine; analyzed 0.42%)	Control (0% added taurine; analyzed 0.12%)	PER	1.42 ± 0.01	1.32 ± 0.01	3	3	Hedges' g (LS variance)	7.98	3.19 to 12.77	Higher, more favorable PER with taurine; 95% CI excluded 0	Not applicable—effect size estimated
Kim et al. (2017)	Olive flounder (Paralichthys olivaceus)	Graded taurine supplementation (0.25–1.50%) under low-temperature conditions	T2 (0.50% added taurine; analyzed 0.42%)	Control (0% added taurine; analyzed 0.12%)	Survival (%)	63.80 ± 13.20	60.00 ± 5.71	3	3	Hedges' g (LS variance)	0.30	-1.31 to 1.91	Higher survival with taurine; 95% CI included 0 (imprecise estimate)	Not applicable—effect size estimated
Kim et al. (2017)	Olive flounder (Paralichthys olivaceus)	Graded taurine supplementation (0.25–1.50%) under low-temperature conditions	T2 (0.50% added taurine; analyzed 0.42%)	Control (0% added taurine; analyzed 0.12%)	Hematocrit (%)	20.90 ± 2.47	15.80 ± 1.58	3	3	Hedges' g (LS variance)	1.96	0.01 to 3.91	Higher hematological value with taurine; 95% CI excluded 0	Not applicable—effect size estimated
Kim et al. (2017)	Olive flounder (Paralichthys olivaceus)	Graded taurine supplementation (0.25–1.50%) under low-temperature conditions	T2 (0.50% added taurine; analyzed 0.42%)	Control (0% added taurine; analyzed 0.12%)	Hemoglobin (g/dL)	3.00 ± 0.28	2.81 ± 0.17	3	3	Hedges' g (LS variance)	0.65	-0.99 to 2.30	Higher hematological value with taurine; 95% CI included 0 (imprecise estimate)	Not applicable—effect size estimated
Kim et al. (2017)	Olive flounder (Paralichthys olivaceus)	Graded taurine supplementation (0.25–1.50%) under low-temperature conditions	T2 (0.50% added taurine; analyzed 0.42%)	Control (0% added taurine; analyzed 0.12%)	Glucose (mg/dL)	47.10 ± 1.81	53.60 ± 5.66	3	3	Hedges' g (LS variance)	-1.23	-2.98 to 0.51	Lower measured value with taurine; 95% CI included 0 (imprecise estimate)	Not applicable—effect size estimated
Kim et al. (2017)	Olive flounder (Paralichthys olivaceus)	Graded taurine supplementation (0.25–1.50%) under low-temperature conditions	T2 (0.50% added taurine; analyzed 0.42%)	Control (0% added taurine; analyzed 0.12%)	Total protein (g/dL)	4.16 ± 0.26	3.86 ± 0.26	3	3	Hedges' g (LS variance)	0.92	-0.76 to 2.60	Higher measured value with taurine; 95% CI included 0 (imprecise estimate)	Not applicable—effect size estimated
Kim et al. (2017)	Olive flounder (Paralichthys olivaceus)	Graded taurine supplementation (0.25–1.50%) under low-temperature conditions	T2 (0.50% added taurine; analyzed 0.42%)	Control (0% added taurine; analyzed 0.12%)	Total cholesterol (mg/dL)	71.60 ± 5.76	82.00 ± 2.53	3	3	Hedges' g (LS variance)	-1.87	-3.78 to 0.05	Lower measured plasma cholesterol with taurine; 95% CI included 0 (imprecise estimate)	Not applicable—effect size estimated
Kim et al. (2017)	Olive flounder (Paralichthys olivaceus)	Graded taurine supplementation (0.25–1.50%) under low-temperature conditions	T2 (0.50% added taurine; analyzed 0.42%)	Control (0% added taurine; analyzed 0.12%)	NBT (reported unit)	0.53 ± 0.02	0.41 ± 0.01	3	3	Hedges' g (LS variance)	6.06	2.27 to 9.84	Higher measured immune/antioxidant response with taurine; 95% CI excluded 0	Not applicable—effect size estimated
Kim et al. (2017)	Olive flounder (Paralichthys olivaceus)	Graded taurine supplementation (0.25–1.50%) under low-temperature conditions	T2 (0.50% added taurine; analyzed 0.42%)	Control (0% added taurine; analyzed 0.12%)	MPO (reported unit)	2.01 ± 0.08	1.39 ± 0.08	3	3	Hedges' g (LS variance)	6.18	2.34 to 10.03	Higher measured immune/antioxidant response with taurine; 95% CI excluded 0	Not applicable—effect size estimated
Kim et al. (2017)	Olive flounder (Paralichthys olivaceus)	Graded taurine supplementation (0.25–1.50%) under low-temperature conditions	T2 (0.50% added taurine; analyzed 0.42%)	Control (0% added taurine; analyzed 0.12%)	SOD (% inhibition)	77.30 ± 2.01	70.60 ± 2.50	3	3	Hedges' g (LS variance)	2.36	0.27 to 4.44	Higher measured immune/antioxidant response with taurine; 95% CI excluded 0	Not applicable—effect size estimated
Kim et al. (2017)	Olive flounder (Paralichthys olivaceus)	Graded taurine supplementation (0.25–1.50%) under low-temperature conditions	T2 (0.50% added taurine; analyzed 0.42%)	Control (0% added taurine; analyzed 0.12%)	Total Ig (mg/mL)	78.90 ± 1.38	20.40 ± 0.35	3	3	Hedges' g (LS variance)	46.37	20.08 to 72.65	Higher measured immune/antioxidant response with taurine; 95% CI excluded 0	Not applicable—effect size estimated
Kim et al. (2017)	Olive flounder (Paralichthys olivaceus)	Graded taurine supplementation (0.25–1.50%) under low-temperature conditions	T2 (0.50% added taurine; analyzed 0.42%)	Control (0% added taurine; analyzed 0.12%)	Lysozyme (µg/mL)	22.40 ± 1.76	15.30 ± 0.70	3	3	Hedges' g (LS variance)	4.23	1.35 to 7.11	Higher measured immune/antioxidant response with taurine; 95% CI excluded 0	Not applicable—effect size estimated
Kim et al. (2017)	Olive flounder (Paralichthys olivaceus)	Graded taurine supplementation (0.25–1.50%) under low-temperature conditions	T2 (0.50% added taurine; analyzed 0.42%)	Control (0% added taurine; analyzed 0.12%)	Antiprotease (% inhibition)	24.10 ± 0.86	13.80 ± 0.63	3	3	Hedges' g (LS variance)	10.90	4.53 to 17.27	Higher measured immune/antioxidant response with taurine; 95% CI excluded 0	Not applicable—effect size estimated
Kim et al. (2017)	Olive flounder (Paralichthys olivaceus)	Graded taurine supplementation (0.25–1.50%) under low-temperature conditions	T2 (0.50% added taurine; analyzed 0.42%)	Control (0% added taurine; analyzed 0.12%)	Whole-body dry matter (% DM)	75.40 ± 0.68	75.40 ± 0.53	3	3	Hedges' g (LS variance)	0.00	-1.60 to 1.60	No observed mean difference; 95% CI included 0 (imprecise estimate)	Not applicable—effect size estimated
Kim et al. (2017)	Olive flounder (Paralichthys olivaceus)	Graded taurine supplementation (0.25–1.50%) under low-temperature conditions	T2 (0.50% added taurine; analyzed 0.42%)	Control (0% added taurine; analyzed 0.12%)	Whole-body protein (% DM)	67.70 ± 1.55	66.40 ± 0.74	3	3	Hedges' g (LS variance)	0.85	-0.82 to 2.53	Higher measured value with taurine; 95% CI included 0 (imprecise estimate)	Not applicable—effect size estimated
Kim et al. (2017)	Olive flounder (Paralichthys olivaceus)	Graded taurine supplementation (0.25–1.50%) under low-temperature conditions	T2 (0.50% added taurine; analyzed 0.42%)	Control (0% added taurine; analyzed 0.12%)	Whole-body lipid (% DM)	22.80 ± 2.56	25.70 ± 1.60	3	3	Hedges' g (LS variance)	-1.08	-2.80 to 0.63	Lower measured value with taurine; 95% CI included 0 (imprecise estimate)	Not applicable—effect size estimated
Kim et al. (2017)	Olive flounder (Paralichthys olivaceus)	Graded taurine supplementation (0.25–1.50%) under low-temperature conditions	T2 (0.50% added taurine; analyzed 0.42%)	Control (0% added taurine; analyzed 0.12%)	Whole-body ash (% DM)	10.20 ± 2.15	14.20 ± 0.06	3	3	Hedges' g (LS variance)	-2.10	-4.09 to -0.11	Lower measured value with taurine; 95% CI excluded 0	Not applicable—effect size estimated
Kim et al. (2017)	Olive flounder (Paralichthys olivaceus)	Graded taurine supplementation (0.25–1.50%) under low-temperature conditions	T3 (1.00% added taurine; analyzed 0.92%)	Control (0% added taurine; analyzed 0.12%)	Final body weight (g)	46.40 ± 3.45	38.10 ± 0.66	3	3	Hedges' g (LS variance)	2.67	0.47 to 4.87	Higher growth outcome with taurine; 95% CI excluded 0	Not applicable—effect size estimated
Kim et al. (2017)	Olive flounder (Paralichthys olivaceus)	Graded taurine supplementation (0.25–1.50%) under low-temperature conditions	T3 (1.00% added taurine; analyzed 0.92%)	Control (0% added taurine; analyzed 0.12%)	Weight gain (%)	137.00 ± 17.60	96.50 ± 3.39	3	3	Hedges' g (LS variance)	2.55	0.40 to 4.70	Higher growth outcome with taurine; 95% CI excluded 0	Not applicable—effect size estimated

Study	Species	Strategy	Intervention	Comparator	Outcome and unit	Intervention mean ± SD	Comparator mean ± SD	n intervention	n comparator	Effect-size metric	Effect size	95% CI	Interpretation	Reason not estimated
Kim et al. (2017)	Olive flounder (Paralichthys olivaceus)	Graded taurine supplementation (0.25–1.50%) under low-temperature conditions	T3 (1.00% added taurine; analyzed 0.92%)	Control (0% added taurine; analyzed 0.12%)	Specific growth rate (%/day)	1.29 ± 0.11	1.01 ± 0.03	3	3	Hedges' g (LS variance)	2.77	0.53 to 5.01	Higher growth outcome with taurine; 95% CI excluded 0	Not applicable—effect size estimated
Kim et al. (2017)	Olive flounder (Paralichthys olivaceus)	Graded taurine supplementation (0.25–1.50%) under low-temperature conditions	T3 (1.00% added taurine; analyzed 0.92%)	Control (0% added taurine; analyzed 0.12%)	Feed intake (g/fish)	33.30 ± 4.04	30.60 ± 1.26	3	3	Hedges' g (LS variance)	0.72	-0.93 to 2.37	Higher feed intake with taurine; 95% CI included 0 (imprecise estimate)	Not applicable—effect size estimated
Kim et al. (2017)	Olive flounder (Paralichthys olivaceus)	Graded taurine supplementation (0.25–1.50%) under low-temperature conditions	T3 (1.00% added taurine; analyzed 0.92%)	Control (0% added taurine; analyzed 0.12%)	FCR	1.24 ± 0.01	1.63 ± 0.02	3	3	Hedges' g (direction reversed; LS variance)	19.68	8.43 to 30.93	Lower, more favorable FCR with taurine; 95% CI excluded 0	Not applicable—effect size estimated
Kim et al. (2017)	Olive flounder (Paralichthys olivaceus)	Graded taurine supplementation (0.25–1.50%) under low-temperature conditions	T3 (1.00% added taurine; analyzed 0.92%)	Control (0% added taurine; analyzed 0.12%)	PER	1.74 ± 0.01	1.32 ± 0.01	3	3	Hedges' g (LS variance)	33.51	14.48 to 52.54	Higher, more favorable PER with taurine; 95% CI excluded 0	Not applicable—effect size estimated
Kim et al. (2017)	Olive flounder (Paralichthys olivaceus)	Graded taurine supplementation (0.25–1.50%) under low-temperature conditions	T3 (1.00% added taurine; analyzed 0.92%)	Control (0% added taurine; analyzed 0.12%)	Survival (%)	66.70 ± 5.95	60.00 ± 5.71	3	3	Hedges' g (LS variance)	0.92	-0.77 to 2.60	Higher survival with taurine; 95% CI included 0 (imprecise estimate)	Not applicable—effect size estimated
Kim et al. (2017)	Olive flounder (Paralichthys olivaceus)	Graded taurine supplementation (0.25–1.50%) under low-temperature conditions	T3 (1.00% added taurine; analyzed 0.92%)	Control (0% added taurine; analyzed 0.12%)	Hematocrit (%)	24.20 ± 0.51	15.80 ± 1.58	3	3	Hedges' g (LS variance)	5.71	2.10 to 9.31	Higher hematological value with taurine; 95% CI excluded 0	Not applicable—effect size estimated
Kim et al. (2017)	Olive flounder (Paralichthys olivaceus)	Graded taurine supplementation (0.25–1.50%) under low-temperature conditions	T3 (1.00% added taurine; analyzed 0.92%)	Control (0% added taurine; analyzed 0.12%)	Hemoglobin (g/dL)	4.66 ± 0.47	2.81 ± 0.17	3	3	Hedges' g (LS variance)	4.18	1.32 to 7.03	Higher hematological value with taurine; 95% CI excluded 0	Not applicable—effect size estimated
Kim et al. (2017)	Olive flounder (Paralichthys olivaceus)	Graded taurine supplementation (0.25–1.50%) under low-temperature conditions	T3 (1.00% added taurine; analyzed 0.92%)	Control (0% added taurine; analyzed 0.12%)	Glucose (mg/dL)	56.30 ± 6.69	53.60 ± 5.66	3	3	Hedges' g (LS variance)	0.35	-1.26 to 1.96	Higher measured value with taurine; 95% CI included 0 (imprecise estimate)	Not applicable—effect size estimated
Kim et al. (2017)	Olive flounder (Paralichthys olivaceus)	Graded taurine supplementation (0.25–1.50%) under low-temperature conditions	T3 (1.00% added taurine; analyzed 0.92%)	Control (0% added taurine; analyzed 0.12%)	Total protein (g/dL)	4.16 ± 0.19	3.86 ± 0.26	3	3	Hedges' g (LS variance)	1.05	-0.66 to 2.76	Higher measured value with taurine; 95% CI included 0 (imprecise estimate)	Not applicable—effect size estimated
Kim et al. (2017)	Olive flounder (Paralichthys olivaceus)	Graded taurine supplementation (0.25–1.50%) under low-temperature conditions	T3 (1.00% added taurine; analyzed 0.92%)	Control (0% added taurine; analyzed 0.12%)	Total cholesterol (mg/dL)	67.70 ± 3.04	82.00 ± 2.53	3	3	Hedges' g (LS variance)	-4.08	-6.89 to -1.27	Lower measured plasma cholesterol with taurine; 95% CI excluded 0	Not applicable—effect size estimated
Kim et al. (2017)	Olive flounder (Paralichthys olivaceus)	Graded taurine supplementation (0.25–1.50%) under low-temperature conditions	T3 (1.00% added taurine; analyzed 0.92%)	Control (0% added taurine; analyzed 0.12%)	NBT (reported unit)	0.58 ± 0.02	0.41 ± 0.01	3	3	Hedges' g (LS variance)	8.58	3.47 to 13.69	Higher measured immune/antioxidant response with taurine; 95% CI excluded 0	Not applicable—effect size estimated
Kim et al. (2017)	Olive flounder (Paralichthys olivaceus)	Graded taurine supplementation (0.25–1.50%) under low-temperature conditions	T3 (1.00% added taurine; analyzed 0.92%)	Control (0% added taurine; analyzed 0.12%)	MPO (reported unit)	2.05 ± 0.06	1.39 ± 0.08	3	3	Hedges' g (LS variance)	7.45	2.94 to 11.95	Higher measured immune/antioxidant response with taurine; 95% CI excluded 0	Not applicable—effect size estimated
Kim et al. (2017)	Olive flounder (Paralichthys olivaceus)	Graded taurine supplementation (0.25–1.50%) under low-temperature conditions	T3 (1.00% added taurine; analyzed 0.92%)	Control (0% added taurine; analyzed 0.12%)	SOD (% inhibition)	76.50 ± 1.55	70.60 ± 2.50	3	3	Hedges' g (LS variance)	2.26	0.21 to 4.31	Higher measured immune/antioxidant response with taurine; 95% CI excluded 0	Not applicable—effect size estimated
Kim et al. (2017)	Olive flounder (Paralichthys olivaceus)	Graded taurine supplementation (0.25–1.50%) under low-temperature conditions	T3 (1.00% added taurine; analyzed 0.92%)	Control (0% added taurine; analyzed 0.12%)	Total Ig (mg/mL)	74.90 ± 1.83	20.40 ± 0.35	3	3	Hedges' g (LS variance)	33.01	14.26 to 51.75	Higher measured immune/antioxidant response with taurine; 95% CI excluded 0	Not applicable—effect size estimated
Kim et al. (2017)	Olive flounder (Paralichthys olivaceus)	Graded taurine supplementation (0.25–1.50%) under low-temperature conditions	T3 (1.00% added taurine; analyzed 0.92%)	Control (0% added taurine; analyzed 0.12%)	Lysozyme (µg/mL)	27.10 ± 0.79	15.30 ± 0.70	3	3	Hedges' g (LS variance)	12.61	5.30 to 19.93	Higher measured immune/antioxidant response with taurine; 95% CI excluded 0	Not applicable—effect size estimated
Kim et al. (2017)	Olive flounder (Paralichthys olivaceus)	Graded taurine supplementation (0.25–1.50%) under low-temperature conditions	T3 (1.00% added taurine; analyzed 0.92%)	Control (0% added taurine; analyzed 0.12%)	Antiprotease (% inhibition)	23.00 ± 0.81	13.80 ± 0.63	3	3	Hedges' g (LS variance)	10.12	4.17 to 16.06	Higher measured immune/antioxidant response with taurine; 95% CI excluded 0	Not applicable—effect size estimated
Kim et al. (2017)	Olive flounder (Paralichthys olivaceus)	Graded taurine supplementation (0.25–1.50%) under low-temperature conditions	T3 (1.00% added taurine; analyzed 0.92%)	Control (0% added taurine; analyzed 0.12%)	Whole-body dry matter (% DM)	75.50 ± 0.78	75.40 ± 0.53	3	3	Hedges' g (LS variance)	0.12	-1.48 to 1.72	Higher measured value with taurine; 95% CI included 0 (imprecise estimate)	Not applicable—effect size estimated
Kim et al. (2017)	Olive flounder (Paralichthys olivaceus)	Graded taurine supplementation (0.25–1.50%) under low-temperature conditions	T3 (1.00% added taurine; analyzed 0.92%)	Control (0% added taurine; analyzed 0.12%)	Whole-body protein (% DM)	67.30 ± 0.85	66.40 ± 0.74	3	3	Hedges' g (LS variance)	0.90	-0.78 to 2.58	Higher measured value with taurine; 95% CI included 0 (imprecise estimate)	Not applicable—effect size estimated
Kim et al. (2017)	Olive flounder (Paralichthys olivaceus)	Graded taurine supplementation (0.25–1.50%) under low-temperature conditions	T3 (1.00% added taurine; analyzed 0.92%)	Control (0% added taurine; analyzed 0.12%)	Whole-body lipid (% DM)	27.70 ± 1.58	25.70 ± 1.60	3	3	Hedges' g (LS variance)	1.00	-0.69 to 2.70	Higher measured value with taurine; 95% CI included 0 (imprecise estimate)	Not applicable—effect size estimated
Kim et al. (2017)	Olive flounder (Paralichthys olivaceus)	Graded taurine supplementation (0.25–1.50%) under low-temperature conditions	T3 (1.00% added taurine; analyzed 0.92%)	Control (0% added taurine; analyzed 0.12%)	Whole-body ash (% DM)	12.50 ± 0.83	14.20 ± 0.06	3	3	Hedges' g (LS variance)	-2.31	-4.37 to -0.24	Lower measured value with taurine; 95% CI excluded 0	Not applicable—effect size estimated
Kim et al. (2017)	Olive flounder (Paralichthys olivaceus)	Graded taurine supplementation (0.25–1.50%) under low-temperature conditions	T4 (1.50% added taurine; analyzed 1.34%)	Control (0% added taurine; analyzed 0.12%)	Final body weight (g)	44.60 ± 5.10	38.10 ± 0.66	3	3	Hedges' g (LS variance)	1.43	-0.37 to 3.22	Higher growth outcome with taurine; 95% CI included 0 (imprecise estimate)	Not applicable—effect size estimated
Kim et al. (2017)	Olive flounder (Paralichthys olivaceus)	Graded taurine supplementation (0.25–1.50%) under low-temperature conditions	T4 (1.50% added taurine; analyzed 1.34%)	Control (0% added taurine; analyzed 0.12%)	Weight gain (%)	128.00 ± 26.00	96.50 ± 3.39	3	3	Hedges' g (LS variance)	1.36	-0.42 to 3.13	Higher growth outcome with taurine; 95% CI included 0 (imprecise estimate)	Not applicable—effect size estimated
Kim et al. (2017)	Olive flounder (Paralichthys olivaceus)	Graded taurine supplementation (0.25–1.50%) under low-temperature conditions	T4 (1.50% added taurine; analyzed 1.34%)	Control (0% added taurine; analyzed 0.12%)	Specific growth rate (%/day)	1.22 ± 0.17	1.01 ± 0.03	3	3	Hedges' g (LS variance)	1.37	-0.41 to 3.15	Higher growth outcome with taurine; 95% CI included 0 (imprecise estimate)	Not applicable—effect size estimated

Study	Species	Strategy	Intervention	Comparator	Outcome and unit	Intervention mean ± SD	Comparator mean ± SD	n intervention	n comparator	Effect-size metric	Effect size	95% CI	Interpretation	Reason not estimated
Kim et al. (2017)	Olive flounder (Paralichthys olivaceus)	Graded taurine supplementation (0.25–1.50%) under low-temperature conditions	T4 (1.50% added taurine; analyzed 1.34%)	Control (0% added taurine; analyzed 0.12%)	Feed intake (g/fish)	30.60 ± 6.12	30.60 ± 1.26	3	3	Hedges' g (LS variance)	0.00	-1.60 to 1.60	No observed mean difference in feed intake; 95% CI included 0 (imprecise estimate)	Not applicable—effect size estimated
Kim et al. (2017)	Olive flounder (Paralichthys olivaceus)	Graded taurine supplementation (0.25–1.50%) under low-temperature conditions	T4 (1.50% added taurine; analyzed 1.34%)	Control (0% added taurine; analyzed 0.12%)	FCR	1.22 ± 0.03	1.63 ± 0.02	3	3	Hedges' g (direction reversed; LS variance)	12.83	5.40 to 20.27	Lower, more favorable FCR with taurine; 95% CI excluded 0	Not applicable—effect size estimated
Kim et al. (2017)	Olive flounder (Paralichthys olivaceus)	Graded taurine supplementation (0.25–1.50%) under low-temperature conditions	T4 (1.50% added taurine; analyzed 1.34%)	Control (0% added taurine; analyzed 0.12%)	PER	1.77 ± 0.04	1.32 ± 0.01	3	3	Hedges' g (LS variance)	12.32	5.17 to 19.46	Higher, more favorable PER with taurine; 95% CI excluded 0	Not applicable—effect size estimated
Kim et al. (2017)	Olive flounder (Paralichthys olivaceus)	Graded taurine supplementation (0.25–1.50%) under low-temperature conditions	T4 (1.50% added taurine; analyzed 1.34%)	Control (0% added taurine; analyzed 0.12%)	Survival (%)	57.50 ± 15.90	60.00 ± 5.71	3	3	Hedges' g (LS variance)	-0.17	-1.77 to 1.44	Lower survival with taurine; 95% CI included 0 (imprecise estimate)	Not applicable—effect size estimated
Kim et al. (2017)	Olive flounder (Paralichthys olivaceus)	Graded taurine supplementation (0.25–1.50%) under low-temperature conditions	T4 (1.50% added taurine; analyzed 1.34%)	Control (0% added taurine; analyzed 0.12%)	Hematocrit (%)	22.80 ± 0.95	15.80 ± 1.58	3	3	Hedges' g (LS variance)	4.28	1.38 to 7.19	Higher hematological value with taurine; 95% CI excluded 0	Not applicable—effect size estimated
Kim et al. (2017)	Olive flounder (Paralichthys olivaceus)	Graded taurine supplementation (0.25–1.50%) under low-temperature conditions	T4 (1.50% added taurine; analyzed 1.34%)	Control (0% added taurine; analyzed 0.12%)	Hemoglobin (g/dL)	3.81 ± 0.12	2.81 ± 0.17	3	3	Hedges' g (LS variance)	5.42	1.96 to 8.88	Higher hematological value with taurine; 95% CI excluded 0	Not applicable—effect size estimated
Kim et al. (2017)	Olive flounder (Paralichthys olivaceus)	Graded taurine supplementation (0.25–1.50%) under low-temperature conditions	T4 (1.50% added taurine; analyzed 1.34%)	Control (0% added taurine; analyzed 0.12%)	Glucose (mg/dL)	51.10 ± 2.95	53.60 ± 5.66	3	3	Hedges' g (LS variance)	-0.44	-2.06 to 1.18	Lower measured value with taurine; 95% CI included 0 (imprecise estimate)	Not applicable—effect size estimated
Kim et al. (2017)	Olive flounder (Paralichthys olivaceus)	Graded taurine supplementation (0.25–1.50%) under low-temperature conditions	T4 (1.50% added taurine; analyzed 1.34%)	Control (0% added taurine; analyzed 0.12%)	Total protein (g/dL)	3.97 ± 0.25	3.86 ± 0.26	3	3	Hedges' g (LS variance)	0.34	-1.27 to 1.96	Higher measured value with taurine; 95% CI included 0 (imprecise estimate)	Not applicable—effect size estimated
Kim et al. (2017)	Olive flounder (Paralichthys olivaceus)	Graded taurine supplementation (0.25–1.50%) under low-temperature conditions	T4 (1.50% added taurine; analyzed 1.34%)	Control (0% added taurine; analyzed 0.12%)	Total cholesterol (mg/dL)	67.00 ± 0.76	82.00 ± 2.53	3	3	Hedges' g (LS variance)	-6.41	-10.37 to -2.44	Lower measured plasma cholesterol with taurine; 95% CI excluded 0	Not applicable—effect size estimated
Kim et al. (2017)	Olive flounder (Paralichthys olivaceus)	Graded taurine supplementation (0.25–1.50%) under low-temperature conditions	T4 (1.50% added taurine; analyzed 1.34%)	Control (0% added taurine; analyzed 0.12%)	NBT (reported unit)	0.52 ± 0.02	0.41 ± 0.01	3	3	Hedges' g (LS variance)	5.55	2.03 to 9.08	Higher measured immune/antioxidant response with taurine; 95% CI excluded 0	Not applicable—effect size estimated
Kim et al. (2017)	Olive flounder (Paralichthys olivaceus)	Graded taurine supplementation (0.25–1.50%) under low-temperature conditions	T4 (1.50% added taurine; analyzed 1.34%)	Control (0% added taurine; analyzed 0.12%)	MPO (reported unit)	1.64 ± 0.04	1.39 ± 0.08	3	3	Hedges' g (LS variance)	3.15	0.76 to 5.55	Higher measured immune/antioxidant response with taurine; 95% CI excluded 0	Not applicable—effect size estimated
Kim et al. (2017)	Olive flounder (Paralichthys olivaceus)	Graded taurine supplementation (0.25–1.50%) under low-temperature conditions	T4 (1.50% added taurine; analyzed 1.34%)	Control (0% added taurine; analyzed 0.12%)	SOD (% inhibition)	71.70 ± 1.90	70.60 ± 2.50	3	3	Hedges' g (LS variance)	0.40	-1.22 to 2.01	Higher measured immune/antioxidant response with taurine; 95% CI included 0 (imprecise estimate)	Not applicable—effect size estimated
Kim et al. (2017)	Olive flounder (Paralichthys olivaceus)	Graded taurine supplementation (0.25–1.50%) under low-temperature conditions	T4 (1.50% added taurine; analyzed 1.34%)	Control (0% added taurine; analyzed 0.12%)	Total Ig (mg/mL)	59.40 ± 0.15	20.40 ± 0.35	3	3	Hedges' g (LS variance)	115.57	50.16 to 180.98	Higher measured immune/antioxidant response with taurine; 95% CI excluded 0	Not applicable—effect size estimated
Kim et al. (2017)	Olive flounder (Paralichthys olivaceus)	Graded taurine supplementation (0.25–1.50%) under low-temperature conditions	T4 (1.50% added taurine; analyzed 1.34%)	Control (0% added taurine; analyzed 0.12%)	Lysozyme (μg/mL)	21.80 ± 0.92	15.30 ± 0.70	3	3	Hedges' g (LS variance)	6.34	2.41 to 10.27	Higher measured immune/antioxidant response with taurine; 95% CI excluded 0	Not applicable—effect size estimated
Kim et al. (2017)	Olive flounder (Paralichthys olivaceus)	Graded taurine supplementation (0.25–1.50%) under low-temperature conditions	T4 (1.50% added taurine; analyzed 1.34%)	Control (0% added taurine; analyzed 0.12%)	Antiprotease (% inhibition)	22.60 ± 0.95	13.80 ± 0.63	3	3	Hedges' g (LS variance)	8.71	3.53 to 13.89	Higher measured immune/antioxidant response with taurine; 95% CI excluded 0	Not applicable—effect size estimated
Kim et al. (2017)	Olive flounder (Paralichthys olivaceus)	Graded taurine supplementation (0.25–1.50%) under low-temperature conditions	T4 (1.50% added taurine; analyzed 1.34%)	Control (0% added taurine; analyzed 0.12%)	Whole-body dry matter (% DM)	75.90 ± 0.20	75.40 ± 0.53	3	3	Hedges' g (LS variance)	1.00	-0.70 to 2.69	Higher measured value with taurine; 95% CI included 0 (imprecise estimate)	Not applicable—effect size estimated
Kim et al. (2017)	Olive flounder (Paralichthys olivaceus)	Graded taurine supplementation (0.25–1.50%) under low-temperature conditions	T4 (1.50% added taurine; analyzed 1.34%)	Control (0% added taurine; analyzed 0.12%)	Whole-body protein (% DM)	67.90 ± 0.45	66.40 ± 0.74	3	3	Hedges' g (LS variance)	1.95	0.01 to 3.90	Higher measured value with taurine; 95% CI excluded 0	Not applicable—effect size estimated
Kim et al. (2017)	Olive flounder (Paralichthys olivaceus)	Graded taurine supplementation (0.25–1.50%) under low-temperature conditions	T4 (1.50% added taurine; analyzed 1.34%)	Control (0% added taurine; analyzed 0.12%)	Whole-body lipid (% DM)	25.30 ± 1.82	25.70 ± 1.60	3	3	Hedges' g (LS variance)	-0.19	-1.79 to 1.42	Lower measured value with taurine; 95% CI included 0 (imprecise estimate)	Not applicable—effect size estimated
Kim et al. (2017)	Olive flounder (Paralichthys olivaceus)	Graded taurine supplementation (0.25–1.50%) under low-temperature conditions	T4 (1.50% added taurine; analyzed 1.34%)	Control (0% added taurine; analyzed 0.12%)	Whole-body ash (% DM)	10.00 ± 2.59	14.20 ± 0.06	3	3	Hedges' g (LS variance)	-1.83	-3.74 to 0.08	Lower measured value with taurine; 95% CI included 0 (imprecise estimate)	Not applicable—effect size estimated
Kim et al. (2017)	Olive flounder (Paralichthys olivaceus)	Graded taurine supplementation (0.25–1.50%) under low-temperature conditions	T1 (0.25% added taurine; analyzed 0.33%)	Control (0% added taurine; analyzed 0.12%)	Hepatic IGF-I mRNA expression	Not reported numerically	Not reported numerically	3	3	Not estimable	Not estimable	Not estimable	The study reported a significantly higher IGF-I expression peak only in T4; exact numerical effect magnitude was unavailable	Exact group means and SDs were presented only graphically and were not reliably extractable
Kim et al. (2017)	Olive flounder (Paralichthys olivaceus)	Graded taurine supplementation (0.25–1.50%) under low-temperature conditions	T2 (0.50% added taurine; analyzed 0.42%)	Control (0% added taurine; analyzed 0.12%)	Hepatic IGF-I mRNA expression	Not reported numerically	Not reported numerically	3	3	Not estimable	Not estimable	Not estimable	The study reported a significantly higher IGF-I expression peak only in T4; exact numerical effect magnitude was unavailable	Exact group means and SDs were presented only graphically and were not reliably extractable
Kim et al. (2017)	Olive flounder (Paralichthys olivaceus)	Graded taurine supplementation (0.25–1.50%) under low-temperature conditions	T3 (1.00% added taurine; analyzed 0.92%)	Control (0% added taurine; analyzed 0.12%)	Hepatic IGF-I mRNA expression	Not reported numerically	Not reported numerically	3	3	Not estimable	Not estimable	Not estimable	The study reported a significantly higher IGF-I expression peak only in T4; exact numerical effect magnitude was unavailable	Exact group means and SDs were presented only graphically and were not reliably extractable
Kim et al. (2017)	Olive flounder (Paralichthys olivaceus)	Graded taurine supplementation (0.25–1.50%) under low-temperature conditions	T4 (1.50% added taurine; analyzed 1.34%)	Control (0% added taurine; analyzed 0.12%)	Hepatic IGF-I mRNA expression	Not reported numerically	Not reported numerically	3	3	Not estimable	Not estimable	Not estimable	The study reported a significantly higher IGF-I expression peak only in T4; exact numerical effect magnitude was unavailable	Exact group means and SDs were presented only graphically and were not reliably extractable

Notes: Continuous outcomes were summarized using Hedges' g and 95% confidence intervals, with the replicate tank treated as the independent experimental unit (n = 3 per treatment). Positive values indicate a higher intervention value; FCR was direction-reversed so that positive values indicate a lower, more favorable FCR. Survival was treated as a continuous tank-level percentage because tank-specific event counts were not reported. Some FCR and PER SDs were reported as 0.00 and may reflect rounding; standardized estimates for those rows should be interpreted cautiously. IGF-I effect sizes were not estimated because exact group means and SDs were not numerically reported.

Supplementary Table S3

Study 3. Kotzamanis et al. (2020): taurine supplementation in a low-fishmeal, high-plant-protein diet

Primary comparisons: T5, T10, and T20 versus the matched low-fishmeal comparator C−. Yellow-shaded rows indicate outcomes for which a study-level effect size was not estimable.

Study	Species	Strategy	Intervention	Comparator	Outcome and unit	Intervention mean ± SD	Comparator mean ± SD	n intervention	n comparator	Effect-size metric	Effect size	95% CI	Interpretation	Reason not estimated
Kotzamanis et al. (2020)	European seabass (Dicentrarchus labrax)	Taurine supplementation (5–20 g/kg) in a 15% fishmeal, high-plant-protein diet	T5 (5 g/kg added taurine; analyzed 0.58%)	C− (15% fishmeal; no added taurine; analyzed taurine 0.11%)	Survival (%)	92.000 ± 3.500	87.000 ± 9.600	3	3	Hedges' g (LS variance)	0.55	-1.08 to 2.18	Higher survival with taurine; 95% CI included 0 (imprecise estimate)	Not applicable—effect size estimated
Kotzamanis et al. (2020)	European seabass (Dicentrarchus labrax)	Taurine supplementation (5–20 g/kg) in a 15% fishmeal, high-plant-protein diet	T5 (5 g/kg added taurine; analyzed 0.58%)	C− (15% fishmeal; no added taurine; analyzed taurine 0.11%)	Final body weight (g)	160.000 ± 2.100	161.000 ± 2.200	3	3	Hedges' g (LS variance)	-0.37	-1.99 to 1.24	Lower growth outcome with taurine; 95% CI included 0 (imprecise estimate)	Not applicable—effect size estimated
Kotzamanis et al. (2020)	European seabass (Dicentrarchus labrax)	Taurine supplementation (5–20 g/kg) in a 15% fishmeal, high-plant-protein diet	T5 (5 g/kg added taurine; analyzed 0.58%)	C− (15% fishmeal; no added taurine; analyzed taurine 0.11%)	Weight gain (g)	83.000 ± 1.900	84.000 ± 2.700	3	3	Hedges' g (LS variance)	-0.34	-1.95 to 1.27	Lower growth outcome with taurine; 95% CI included 0 (imprecise estimate)	Not applicable—effect size estimated
Kotzamanis et al. (2020)	European seabass (Dicentrarchus labrax)	Taurine supplementation (5–20 g/kg) in a 15% fishmeal, high-plant-protein diet	T5 (5 g/kg added taurine; analyzed 0.58%)	C− (15% fishmeal; no added taurine; analyzed taurine 0.11%)	Daily growth index (%)	1.660 ± 0.030	1.660 ± 0.050	3	3	Hedges' g (LS variance)	0.0	-1.60 to 1.60	No observed mean difference; 95% CI included 0 (imprecise estimate)	Not applicable—effect size estimated
Kotzamanis et al. (2020)	European seabass (Dicentrarchus labrax)	Taurine supplementation (5–20 g/kg) in a 15% fishmeal, high-plant-protein diet	T5 (5 g/kg added taurine; analyzed 0.58%)	C− (15% fishmeal; no added taurine; analyzed taurine 0.11%)	Total feed intake (g DM/fish)	115.000 ± 12.400	129.000 ± 15.100	3	3	Hedges' g (LS variance)	-0.81	-2.47 to 0.86	Lower feed intake with taurine; 95% CI included 0 (imprecise estimate)	Not applicable—effect size estimated
Kotzamanis et al. (2020)	European seabass (Dicentrarchus labrax)	Taurine supplementation (5–20 g/kg) in a 15% fishmeal, high-plant-protein diet	T5 (5 g/kg added taurine; analyzed 0.58%)	C− (15% fishmeal; no added taurine; analyzed taurine 0.11%)	Relative feed intake (% DM)	149.100 ± 15.900	166.200 ± 20.500	3	3	Hedges' g (LS variance)	-0.74	-2.40 to 0.91	Lower feed intake with taurine; 95% CI included 0 (imprecise estimate)	Not applicable—effect size estimated
Kotzamanis et al. (2020)	European seabass (Dicentrarchus labrax)	Taurine supplementation (5–20 g/kg) in a 15% fishmeal, high-plant-protein diet	T5 (5 g/kg added taurine; analyzed 0.58%)	C− (15% fishmeal; no added taurine; analyzed taurine 0.11%)	Specific growth rate (%/day)	1.030 ± 0.020	1.030 ± 0.030	3	3	Hedges' g (LS variance)	0.0	-1.60 to 1.60	No observed mean difference; 95% CI included 0 (imprecise estimate)	Not applicable—effect size estimated
Kotzamanis et al. (2020)	European seabass (Dicentrarchus labrax)	Taurine supplementation (5–20 g/kg) in a 15% fishmeal, high-plant-protein diet	T5 (5 g/kg added taurine; analyzed 0.58%)	C− (15% fishmeal; no added taurine; analyzed taurine 0.11%)	FCR	1.380 ± 0.120	1.530 ± 0.220	3	3	Hedges' g (direction reversed; LS variance)	0.68	-0.97 to 2.32	Lower, more favorable FCR with taurine; 95% CI included 0 (imprecise estimate)	Not applicable—effect size estimated
Kotzamanis et al. (2020)	European seabass (Dicentrarchus labrax)	Taurine supplementation (5–20 g/kg) in a 15% fishmeal, high-plant-protein diet	T5 (5 g/kg added taurine; analyzed 0.58%)	C− (15% fishmeal; no added taurine; analyzed taurine 0.11%)	PER	1.640 ± 0.140	1.540 ± 0.420	3	3	Hedges' g (LS variance)	0.25	-1.35 to 1.86	Higher, more favorable PER with taurine; 95% CI included 0 (imprecise estimate)	Not applicable—effect size estimated
Kotzamanis et al. (2020)	European seabass (Dicentrarchus labrax)	Taurine supplementation (5–20 g/kg) in a 15% fishmeal, high-plant-protein diet	T5 (5 g/kg added taurine; analyzed 0.58%)	C− (15% fishmeal; no added taurine; analyzed taurine 0.11%)	Thermal growth coefficient (×1,000)	0.600 ± 0.010	0.600 ± 0.020	3	3	Hedges' g (LS variance)	0.0	-1.60 to 1.60	No observed mean difference; 95% CI included 0 (imprecise estimate)	Not applicable—effect size estimated
Kotzamanis et al. (2020)	European seabass (Dicentrarchus labrax)	Taurine supplementation (5–20 g/kg) in a 15% fishmeal, high-plant-protein diet	T5 (5 g/kg added taurine; analyzed 0.58%)	C− (15% fishmeal; no added taurine; analyzed taurine 0.11%)	Lipidosomatic index (%)	9.880 ± 0.240	9.510 ± 2.440	3	3	Hedges' g (LS variance)	0.17	-1.43 to 1.77	Higher measured value with taurine; 95% CI included 0 (imprecise estimate)	Not applicable—effect size estimated
Kotzamanis et al. (2020)	European seabass (Dicentrarchus labrax)	Taurine supplementation (5–20 g/kg) in a 15% fishmeal, high-plant-protein diet	T5 (5 g/kg added taurine; analyzed 0.58%)	C− (15% fishmeal; no added taurine; analyzed taurine 0.11%)	Hepatosomatic index (%)	2.230 ± 0.260	2.160 ± 0.230	3	3	Hedges' g (LS variance)	0.23	-1.38 to 1.83	Higher measured value with taurine; 95% CI included 0 (imprecise estimate)	Not applicable—effect size estimated
Kotzamanis et al. (2020)	European seabass (Dicentrarchus labrax)	Taurine supplementation (5–20 g/kg) in a 15% fishmeal, high-plant-protein diet	T5 (5 g/kg added taurine; analyzed 0.58%)	C− (15% fishmeal; no added taurine; analyzed taurine 0.11%)	Whole-body moisture (g/kg fresh weight)	637.000 ± 9.000	607.000 ± 21.000	3	3	Hedges' g (LS variance)	1.48	-0.32 to 3.29	Higher measured value with taurine; 95% CI included 0 (imprecise estimate)	Not applicable—effect size estimated
Kotzamanis et al. (2020)	European seabass (Dicentrarchus labrax)	Taurine supplementation (5–20 g/kg) in a 15% fishmeal, high-plant-protein diet	T5 (5 g/kg added taurine; analyzed 0.58%)	C− (15% fishmeal; no added taurine; analyzed taurine 0.11%)	Whole-body protein (g/kg fresh weight)	158.000 ± 3.000	171.000 ± 6.000	3	3	Hedges' g (LS variance)	-2.19	-4.21 to -0.16	Lower measured value with taurine; 95% CI excluded 0	Not applicable—effect size estimated
Kotzamanis et al. (2020)	European seabass (Dicentrarchus labrax)	Taurine supplementation (5–20 g/kg) in a 15% fishmeal, high-plant-protein diet	T5 (5 g/kg added taurine; analyzed 0.58%)	C− (15% fishmeal; no added taurine; analyzed taurine 0.11%)	Whole-body lipid (g/kg fresh weight)	143.000 ± 10.000	160.000 ± 4.000	3	3	Hedges' g (LS variance)	-1.78	-3.67 to 0.11	Lower measured value with taurine; 95% CI included 0 (imprecise estimate)	Not applicable—effect size estimated
Kotzamanis et al. (2020)	European seabass (Dicentrarchus labrax)	Taurine supplementation (5–20 g/kg) in a 15% fishmeal, high-plant-protein diet	T5 (5 g/kg added taurine; analyzed 0.58%)	C− (15% fishmeal; no added taurine; analyzed taurine 0.11%)	Whole-body ash (g/kg fresh weight)	40.000 ± 2.000	25.000 ± 9.000	3	3	Hedges' g (LS variance)	1.84	-0.07 to 3.74	Higher measured value with taurine; 95% CI included 0 (imprecise estimate)	Not applicable—effect size estimated
Kotzamanis et al. (2020)	European seabass (Dicentrarchus labrax)	Taurine supplementation (5–20 g/kg) in a 15% fishmeal, high-plant-protein diet	T5 (5 g/kg added taurine; analyzed 0.58%)	C− (15% fishmeal; no added taurine; analyzed taurine 0.11%)	Muscle water (g/kg fresh weight)	749.000 ± 1.800	741.000 ± 2.300	3	3	Hedges' g (LS variance)	3.09	0.72 to 5.46	Higher measured value with taurine; 95% CI excluded 0	Not applicable—effect size estimated
Kotzamanis et al. (2020)	European seabass (Dicentrarchus labrax)	Taurine supplementation (5–20 g/kg) in a 15% fishmeal, high-plant-protein diet	T5 (5 g/kg added taurine; analyzed 0.58%)	C− (15% fishmeal; no added taurine; analyzed taurine 0.11%)	Muscle protein (g/kg fresh weight)	214.000 ± 10.500	227.000 ± 17.000	3	3	Hedges' g (LS variance)	-0.73	-2.39 to 0.92	Lower measured value with taurine; 95% CI included 0 (imprecise estimate)	Not applicable—effect size estimated
Kotzamanis et al. (2020)	European seabass (Dicentrarchus labrax)	Taurine supplementation (5–20 g/kg) in a 15% fishmeal, high-plant-protein diet	T5 (5 g/kg added taurine; analyzed 0.58%)	C− (15% fishmeal; no added taurine; analyzed taurine 0.11%)	Muscle lipid (g/kg fresh weight)	28.000 ± 0.100	26.000 ± 0.100	3	3	Hedges' g (LS variance)	15.96	6.79 to 25.13	Higher measured value with taurine; 95% CI excluded 0	Not applicable—effect size estimated
Kotzamanis et al. (2020)	European seabass (Dicentrarchus labrax)	Taurine supplementation (5–20 g/kg) in a 15% fishmeal, high-plant-protein diet	T5 (5 g/kg added taurine; analyzed 0.58%)	C− (15% fishmeal; no added taurine; analyzed taurine 0.11%)	Muscle ash (g/kg fresh weight)	15.000 ± 0.200	14.000 ± 0.400	3	3	Hedges' g (LS variance)	2.52	0.38 to 4.67	Higher measured value with taurine; 95% CI excluded 0	Not applicable—effect size estimated
Kotzamanis et al. (2020)	European seabass (Dicentrarchus labrax)	Taurine supplementation (5–20 g/kg) in a 15% fishmeal, high-plant-protein diet	T5 (5 g/kg added taurine; analyzed 0.58%)	C− (15% fishmeal; no added taurine; analyzed taurine 0.11%)	Fillet histidine (g/kg muscle)	5.000 ± 0.300	4.800 ± 1.100	3	3	Hedges' g (LS variance)	0.2	-1.41 to 1.80	Higher measured value with taurine; 95% CI included 0 (imprecise estimate)	Not applicable—effect size estimated
Kotzamanis et al. (2020)	European seabass (Dicentrarchus labrax)	Taurine supplementation (5–20 g/kg) in a 15% fishmeal, high-plant-protein diet	T5 (5 g/kg added taurine; analyzed 0.58%)	C− (15% fishmeal; no added taurine; analyzed taurine 0.11%)	Fillet taurine (g/kg muscle)	4.600 ± 0.200	2.300 ± 0.300	3	3	Hedges' g (LS variance)	7.2	2.82 to 11.57	Higher muscle taurine deposition with taurine; 95% CI excluded 0	Not applicable—effect size estimated
Kotzamanis et al. (2020)	European seabass (Dicentrarchus labrax)	Taurine supplementation (5–20 g/kg) in a 15% fishmeal, high-plant-protein diet	T5 (5 g/kg added taurine; analyzed 0.58%)	C− (15% fishmeal; no added taurine; analyzed taurine 0.11%)	Fillet serine (g/kg muscle)	9.100 ± 0.280	8.500 ± 0.600	3	3	Hedges' g (LS variance)	1.02	-0.68 to 2.72	Higher measured value with taurine; 95% CI included 0 (imprecise estimate)	Not applicable—effect size estimated
Kotzamanis et al. (2020)	European seabass (Dicentrarchus labrax)	Taurine supplementation (5–20 g/kg) in a 15% fishmeal, high-plant-protein diet	T5 (5 g/kg added taurine; analyzed 0.58%)	C− (15% fishmeal; no added taurine; analyzed taurine 0.11%)	Fillet arginine (g/kg muscle)	13.900 ± 0.580	14.000 ± 0.510	3	3	Hedges' g (LS variance)	-0.15	-1.75 to 1.46	Lower measured value with taurine; 95% CI included 0 (imprecise estimate)	Not applicable—effect size estimated
Kotzamanis et al. (2020)	European seabass (Dicentrarchus labrax)	Taurine supplementation (5–20 g/kg) in a 15% fishmeal, high-plant-protein diet	T5 (5 g/kg added taurine; analyzed 0.58%)	C− (15% fishmeal; no added taurine; analyzed taurine 0.11%)	Fillet glycine (g/kg muscle)	11.300 ± 1.050	11.700 ± 0.200	3	3	Hedges' g (LS variance)	-0.42	-2.04 to 1.20	Lower measured value with taurine; 95% CI included 0 (imprecise estimate)	Not applicable—effect size estimated
Kotzamanis et al. (2020)	European seabass (Dicentrarchus labrax)	Taurine supplementation (5–20 g/kg) in a 15% fishmeal, high-plant-protein diet	T5 (5 g/kg added taurine; analyzed 0.58%)	C− (15% fishmeal; no added taurine; analyzed taurine 0.11%)	Fillet Asx (g/kg muscle)	23.900 ± 0.500	24.300 ± 1.260	3	3	Hedges' g (LS variance)	-0.33	-1.94 to 1.28	Lower measured value with taurine; 95% CI included 0 (imprecise estimate)	Not applicable—effect size estimated
Kotzamanis et al. (2020)	European seabass (Dicentrarchus labrax)	Taurine supplementation (5–20 g/kg) in a 15% fishmeal, high-plant-protein diet	T5 (5 g/kg added taurine; analyzed 0.58%)	C− (15% fishmeal; no added taurine; analyzed taurine 0.11%)	Fillet Glx (g/kg muscle)	34.200 ± 0.900	34.800 ± 1.590	3	3	Hedges' g (LS variance)	-0.37	-1.98 to 1.24	Lower measured value with taurine; 95% CI included 0 (imprecise estimate)	Not applicable—effect size estimated
Kotzamanis et al. (2020)	European seabass (Dicentrarchus labrax)	Taurine supplementation (5–20 g/kg) in a 15% fishmeal,	T5 (5 g/kg added taurine; analyzed 0.58%)	C− (15% fishmeal; no added taurine; analyzed taurine 0.11%)	Fillet threonine (g/kg muscle)	10.100 ± 0.260	10.100 ± 0.430	3	3	Hedges' g (LS variance)	0.0	-1.60 to 1.60	No observed mean difference; 95% CI included 0 (imprecise estimate)	Not applicable—effect size estimated

Study	Species	Strategy	Intervention	Comparator	Outcome and unit	Intervention mean ± SD	Comparator mean ± SD	n intervention	n comparator	Effect-size metric	Effect size	95% CI	Interpretation	Reason not estimated
		high-plant-protein diet												
Kotzamanis et al. (2020)	European seabass (Dicentrarchus labrax)	Taurine supplementation (5–20 g/kg) in a 15% fishmeal, high-plant-protein diet	T5 (5 g/kg added taurine; analyzed 0.58%)	C− (15% fishmeal; no added taurine; analyzed taurine 0.11%)	Fillet alanine (g/kg muscle)	14.000 ± 0.110	13.900 ± 0.520	3	3	Hedges' g (LS variance)	0.21	-1.39 to 1.82	Higher measured value with taurine; 95% CI included 0 (imprecise estimate)	Not applicable—effect size estimated
Kotzamanis et al. (2020)	European seabass (Dicentrarchus labrax)	Taurine supplementation (5–20 g/kg) in a 15% fishmeal, high-plant-protein diet	T5 (5 g/kg added taurine; analyzed 0.58%)	C− (15% fishmeal; no added taurine; analyzed taurine 0.11%)	Fillet proline (g/kg muscle)	7.300 ± 0.300	7.500 ± 0.170	3	3	Hedges' g (LS variance)	-0.65	-2.30 to 0.99	Lower measured value with taurine; 95% CI included 0 (imprecise estimate)	Not applicable—effect size estimated
Kotzamanis et al. (2020)	European seabass (Dicentrarchus labrax)	Taurine supplementation (5–20 g/kg) in a 15% fishmeal, high-plant-protein diet	T5 (5 g/kg added taurine; analyzed 0.58%)	C− (15% fishmeal; no added taurine; analyzed taurine 0.11%)	Fillet cystine (g/kg muscle)	0.700 ± 0.000	0.800 ± 0.400	3	3	Hedges' g (LS variance)	-0.28	-1.89 to 1.33	Lower measured value with taurine; intervention SD was reported as 0.00, so the standardized estimate may be sensitive to rounding; 95% CI included 0 (imprecise estimate)	Not applicable—effect size estimated
Kotzamanis et al. (2020)	European seabass (Dicentrarchus labrax)	Taurine supplementation (5–20 g/kg) in a 15% fishmeal, high-plant-protein diet	T5 (5 g/kg added taurine; analyzed 0.58%)	C− (15% fishmeal; no added taurine; analyzed taurine 0.11%)	Fillet lysine (g/kg muscle)	20.300 ± 0.500	20.200 ± 9.600	3	3	Hedges' g (LS variance)	0.01	-1.59 to 1.61	Higher measured value with taurine; 95% CI included 0 (imprecise estimate)	Not applicable—effect size estimated
Kotzamanis et al. (2020)	European seabass (Dicentrarchus labrax)	Taurine supplementation (5–20 g/kg) in a 15% fishmeal, high-plant-protein diet	T5 (5 g/kg added taurine; analyzed 0.58%)	C− (15% fishmeal; no added taurine; analyzed taurine 0.11%)	Fillet tyrosine (g/kg muscle)	7.700 ± 0.170	7.800 ± 0.410	3	3	Hedges' g (LS variance)	-0.25	-1.86 to 1.35	Lower measured value with taurine; 95% CI included 0 (imprecise estimate)	Not applicable—effect size estimated
Kotzamanis et al. (2020)	European seabass (Dicentrarchus labrax)	Taurine supplementation (5–20 g/kg) in a 15% fishmeal, high-plant-protein diet	T5 (5 g/kg added taurine; analyzed 0.58%)	C− (15% fishmeal; no added taurine; analyzed taurine 0.11%)	Fillet methionine (g/kg muscle)	6.900 ± 0.050	7.000 ± 0.360	3	3	Hedges' g (LS variance)	-0.31	-1.92 to 1.30	Lower measured value with taurine; 95% CI included 0 (imprecise estimate)	Not applicable—effect size estimated
Kotzamanis et al. (2020)	European seabass (Dicentrarchus labrax)	Taurine supplementation (5–20 g/kg) in a 15% fishmeal, high-plant-protein diet	T5 (5 g/kg added taurine; analyzed 0.58%)	C− (15% fishmeal; no added taurine; analyzed taurine 0.11%)	Fillet valine (g/kg muscle)	10.400 ± 0.400	10.500 ± 0.510	3	3	Hedges' g (LS variance)	-0.17	-1.78 to 1.43	Lower measured value with taurine; 95% CI included 0 (imprecise estimate)	Not applicable—effect size estimated
Kotzamanis et al. (2020)	European seabass (Dicentrarchus labrax)	Taurine supplementation (5–20 g/kg) in a 15% fishmeal, high-plant-protein diet	T5 (5 g/kg added taurine; analyzed 0.58%)	C− (15% fishmeal; no added taurine; analyzed taurine 0.11%)	Fillet isoleucine (g/kg muscle)	9.900 ± 0.280	9.700 ± 6.400	3	3	Hedges' g (LS variance)	0.04	-1.57 to 1.64	Higher measured value with taurine; 95% CI included 0 (imprecise estimate)	Not applicable—effect size estimated
Kotzamanis et al. (2020)	European seabass (Dicentrarchus labrax)	Taurine supplementation (5–20 g/kg) in a 15% fishmeal, high-plant-protein diet	T5 (5 g/kg added taurine; analyzed 0.58%)	C− (15% fishmeal; no added taurine; analyzed taurine 0.11%)	Fillet leucine (g/kg muscle)	17.700 ± 0.460	17.800 ± 0.920	3	3	Hedges' g (LS variance)	-0.11	-1.71 to 1.49	Lower measured value with taurine; 95% CI included 0 (imprecise estimate)	Not applicable—effect size estimated
Kotzamanis et al. (2020)	European seabass (Dicentrarchus labrax)	Taurine supplementation (5–20 g/kg) in a 15% fishmeal, high-plant-protein diet	T5 (5 g/kg added taurine; analyzed 0.58%)	C− (15% fishmeal; no added taurine; analyzed taurine 0.11%)	Fillet phenylalanine (g/kg muscle)	9.400 ± 0.100	9.400 ± 0.500	3	3	Hedges' g (LS variance)	0.0	-1.60 to 1.60	No observed mean difference; 95% CI included 0 (imprecise estimate)	Not applicable—effect size estimated
Kotzamanis et al. (2020)	European seabass (Dicentrarchus labrax)	Taurine supplementation (5–20 g/kg) in a 15% fishmeal, high-plant-protein diet	T5 (5 g/kg added taurine; analyzed 0.58%)	C− (15% fishmeal; no added taurine; analyzed taurine 0.11%)	Muscle taurine (% of muscle protein)	0.213 ± 0.010	0.102 ± 0.013	3	3	Hedges' g (LS variance)	7.64	3.03 to 12.24	Higher muscle taurine deposition with taurine; 95% CI excluded 0	Not applicable—effect size estimated
Kotzamanis et al. (2020)	European seabass (Dicentrarchus labrax)	Taurine supplementation (5–20 g/kg) in a 15% fishmeal, high-plant-protein diet	T10 (10 g/kg added taurine; analyzed 1.06%)	C− (15% fishmeal; no added taurine; analyzed taurine 0.11%)	Survival (%)	88.000 ± 10.500	87.000 ± 9.600	3	3	Hedges' g (LS variance)	0.08	-1.52 to 1.68	Higher survival with taurine; 95% CI included 0 (imprecise estimate)	Not applicable—effect size estimated
Kotzamanis et al. (2020)	European seabass (Dicentrarchus labrax)	Taurine supplementation (5–20 g/kg) in a 15% fishmeal, high-plant-protein diet	T10 (10 g/kg added taurine; analyzed 1.06%)	C− (15% fishmeal; no added taurine; analyzed taurine 0.11%)	Final body weight (g)	166.000 ± 0.900	161.000 ± 2.200	3	3	Hedges' g (LS variance)	2.37	0.28 to 4.46	Higher growth outcome with taurine; 95% CI excluded 0	Not applicable—effect size estimated
Kotzamanis et al. (2020)	European seabass (Dicentrarchus labrax)	Taurine supplementation (5–20 g/kg) in a 15% fishmeal, high-plant-protein diet	T10 (10 g/kg added taurine; analyzed 1.06%)	C− (15% fishmeal; no added taurine; analyzed taurine 0.11%)	Weight gain (g)	88.000 ± 1.300	84.000 ± 2.700	3	3	Hedges' g (LS variance)	1.51	-0.31 to 3.32	Higher growth outcome with taurine; 95% CI included 0 (imprecise estimate)	Not applicable—effect size estimated
Kotzamanis et al. (2020)	European seabass (Dicentrarchus labrax)	Taurine supplementation (5–20 g/kg) in a 15% fishmeal, high-plant-protein diet	T10 (10 g/kg added taurine; analyzed 1.06%)	C− (15% fishmeal; no added taurine; analyzed taurine 0.11%)	Daily growth index (%)	1.730 ± 0.020	1.660 ± 0.050	3	3	Hedges' g (LS variance)	1.47	-0.34 to 3.27	Higher growth outcome with taurine; 95% CI included 0 (imprecise estimate)	Not applicable—effect size estimated
Kotzamanis et al. (2020)	European seabass (Dicentrarchus labrax)	Taurine supplementation (5–20 g/kg) in a 15% fishmeal, high-plant-protein diet	T10 (10 g/kg added taurine; analyzed 1.06%)	C− (15% fishmeal; no added taurine; analyzed taurine 0.11%)	Total feed intake (g DM/fish)	117.000 ± 15.500	129.000 ± 15.100	3	3	Hedges' g (LS variance)	-0.63	-2.26 to 1.01	Lower feed intake with taurine; 95% CI included 0 (imprecise estimate)	Not applicable—effect size estimated
Kotzamanis et al. (2020)	European seabass (Dicentrarchus labrax)	Taurine supplementation (5–20 g/kg) in a 15% fishmeal, high-plant-protein diet	T10 (10 g/kg added taurine; analyzed 1.06%)	C− (15% fishmeal; no added taurine; analyzed taurine 0.11%)	Relative feed intake (% DM)	151.000 ± 20.800	166.200 ± 20.500	3	3	Hedges' g (LS variance)	-0.59	-2.22 to 1.05	Lower feed intake with taurine; 95% CI included 0 (imprecise estimate)	Not applicable—effect size estimated
Kotzamanis et al. (2020)	European seabass (Dicentrarchus labrax)	Taurine supplementation (5–20 g/kg) in a 15% fishmeal, high-plant-protein diet	T10 (10 g/kg added taurine; analyzed 1.06%)	C− (15% fishmeal; no added taurine; analyzed taurine 0.11%)	Specific growth rate (%/day)	1.070 ± 0.010	1.030 ± 0.030	3	3	Hedges' g (LS variance)	1.43	-0.37 to 3.22	Higher growth outcome with taurine; 95% CI included 0 (imprecise estimate)	Not applicable—effect size estimated
Kotzamanis et al. (2020)	European seabass (Dicentrarchus labrax)	Taurine supplementation (5–20 g/kg) in a 15% fishmeal, high-plant-protein diet	T10 (10 g/kg added taurine; analyzed 1.06%)	C− (15% fishmeal; no added taurine; analyzed taurine 0.11%)	FCR	1.330 ± 0.160	1.530 ± 0.220	3	3	Hedges' g (direction reversed; LS variance)	0.83	-0.84 to 2.50	Lower, more favorable FCR with taurine; 95% CI included 0 (imprecise estimate)	Not applicable—effect size estimated
Kotzamanis et al. (2020)	European seabass (Dicentrarchus labrax)	Taurine supplementation (5–20 g/kg) in a 15% fishmeal, high-plant-protein diet	T10 (10 g/kg added taurine; analyzed 1.06%)	C− (15% fishmeal; no added taurine; analyzed taurine 0.11%)	PER	1.750 ± 0.200	1.540 ± 0.420	3	3	Hedges' g (LS variance)	0.51	-1.12 to 2.14	Higher, more favorable PER with taurine; 95% CI included 0 (imprecise estimate)	Not applicable—effect size estimated
Kotzamanis et al. (2020)	European seabass (Dicentrarchus labrax)	Taurine supplementation (5–20 g/kg) in a 15% fishmeal, high-plant-protein diet	T10 (10 g/kg added taurine; analyzed 1.06%)	C− (15% fishmeal; no added taurine; analyzed taurine 0.11%)	Thermal growth coefficient (×1,000)	0.620 ± 0.010	0.600 ± 0.020	3	3	Hedges' g (LS variance)	1.01	-0.69 to 2.71	Higher growth outcome with taurine; 95% CI included 0 (imprecise estimate)	Not applicable—effect size estimated
Kotzamanis et al. (2020)	European seabass (Dicentrarchus labrax)	Taurine supplementation (5–20 g/kg) in a 15% fishmeal, high-plant-protein diet	T10 (10 g/kg added taurine; analyzed 1.06%)	C− (15% fishmeal; no added taurine; analyzed taurine 0.11%)	Lipidosomatic index (%)	9.630 ± 0.900	9.510 ± 2.440	3	3	Hedges' g (LS variance)	0.05	-1.55 to 1.65	Higher measured value with taurine; 95% CI included 0 (imprecise estimate)	Not applicable—effect size estimated
Kotzamanis et al. (2020)	European seabass (Dicentrarchus labrax)	Taurine supplementation (5–20 g/kg) in a 15% fishmeal, high-plant-protein diet	T10 (10 g/kg added taurine; analyzed 1.06%)	C− (15% fishmeal; no added taurine; analyzed taurine 0.11%)	Hepatosomatic index (%)	2.260 ± 0.210	2.160 ± 0.230	3	3	Hedges' g (LS variance)	0.36	-1.25 to 1.98	Higher measured value with taurine; 95% CI included 0 (imprecise estimate)	Not applicable—effect size estimated
Kotzamanis et al. (2020)	European seabass (Dicentrarchus labrax)	Taurine supplementation (5–20 g/kg) in a 15% fishmeal, high-plant-protein diet	T10 (10 g/kg added taurine; analyzed 1.06%)	C− (15% fishmeal; no added taurine; analyzed taurine 0.11%)	Whole-body moisture (g/kg fresh weight)	636.000 ± 9.000	607.000 ± 21.000	3	3	Hedges' g (LS variance)	1.43	-0.36 to 3.23	Higher measured value with taurine; 95% CI included 0 (imprecise estimate)	Not applicable—effect size estimated
Kotzamanis et al. (2020)	European seabass (Dicentrarchus labrax)	Taurine supplementation (5–20 g/kg) in a 15% fishmeal, high-plant-protein diet	T10 (10 g/kg added taurine; analyzed 1.06%)	C− (15% fishmeal; no added taurine; analyzed taurine 0.11%)	Whole-body protein (g/kg fresh weight)	165.000 ± 7.000	171.000 ± 6.000	3	3	Hedges' g (LS variance)	-0.73	-2.39 to 0.92	Lower measured value with taurine; 95% CI included 0 (imprecise estimate)	Not applicable—effect size estimated
Kotzamanis et al. (2020)	European seabass (Dicentrarchus labrax)	Taurine supplementation (5–20 g/kg) in a 15% fishmeal, high-plant-protein diet	T10 (10 g/kg added taurine; analyzed 1.06%)	C− (15% fishmeal; no added taurine; analyzed taurine 0.11%)	Whole-body lipid (g/kg fresh weight)	154.000 ± 2.000	160.000 ± 4.000	3	3	Hedges' g (LS variance)	-1.51	-3.33 to 0.30	Lower measured value with taurine; 95% CI included 0 (imprecise estimate)	Not applicable—effect size estimated
Kotzamanis et al. (2020)	European seabass (Dicentrarchus labrax)	Taurine supplementation (5–20 g/kg) in a 15% fishmeal, high-plant-protein diet	T10 (10 g/kg added taurine; analyzed 1.06%)	C− (15% fishmeal; no added taurine; analyzed taurine 0.11%)	Whole-body ash (g/kg fresh weight)	41.000 ± 10.000	25.000 ± 9.000	3	3	Hedges' g (LS variance)	1.34	-0.43 to 3.11	Higher measured value with taurine; 95% CI included 0 (imprecise estimate)	Not applicable—effect size estimated
Kotzamanis et al. (2020)	European seabass (Dicentrarchus labrax)	Taurine supplementation (5–20 g/kg) in a 15% fishmeal, high-plant-protein diet	T10 (10 g/kg added taurine; analyzed 1.06%)	C− (15% fishmeal; no added taurine; analyzed taurine 0.11%)	Muscle water (g/kg fresh weight)	752.000 ± 3.500	741.000 ± 2.300	3	3	Hedges' g (LS variance)	2.96	0.65 to 5.28	Higher measured value with taurine; 95% CI excluded 0	Not applicable—effect size estimated

Study	Species	Strategy	Intervention	Comparator	Outcome and unit	Intervention mean ± SD	Comparator mean ± SD	n intervention	n comparator	Effect-size metric	Effect size	95% CI	Interpretation	Reason not estimated
Kotzamanis et al. (2020)	European seabass (Dicentrarchus labrax)	Taurine supplementation (5–20 g/kg) in a 15% fishmeal, high-plant-protein diet	T10 (10 g/kg added taurine; analyzed 1.06%)	C− (15% fishmeal; no added taurine; analyzed taurine 0.11%)	Muscle protein (g/kg fresh weight)	219.000 ± 14.100	227.000 ± 17.000	3	3	Hedges' g (LS variance)	-0.41	-2.03 to 1.21	Lower measured value with taurine; 95% CI included 0 (imprecise estimate)	Not applicable—effect size estimated
Kotzamanis et al. (2020)	European seabass (Dicentrarchus labrax)	Taurine supplementation (5–20 g/kg) in a 15% fishmeal, high-plant-protein diet	T10 (10 g/kg added taurine; analyzed 1.06%)	C− (15% fishmeal; no added taurine; analyzed taurine 0.11%)	Muscle lipid (g/kg fresh weight)	26.000 ± 3.200	26.000 ± 0.100	3	3	Hedges' g (LS variance)	0.0	-1.60 to 1.60	No observed mean difference; 95% CI included 0 (imprecise estimate)	Not applicable—effect size estimated
Kotzamanis et al. (2020)	European seabass (Dicentrarchus labrax)	Taurine supplementation (5–20 g/kg) in a 15% fishmeal, high-plant-protein diet	T10 (10 g/kg added taurine; analyzed 1.06%)	C− (15% fishmeal; no added taurine; analyzed taurine 0.11%)	Muscle ash (g/kg fresh weight)	15.000 ± 0.800	14.000 ± 0.400	3	3	Hedges' g (LS variance)	1.26	-0.49 to 3.01	Higher measured value with taurine; 95% CI included 0 (imprecise estimate)	Not applicable—effect size estimated
Kotzamanis et al. (2020)	European seabass (Dicentrarchus labrax)	Taurine supplementation (5–20 g/kg) in a 15% fishmeal, high-plant-protein diet	T10 (10 g/kg added taurine; analyzed 1.06%)	C− (15% fishmeal; no added taurine; analyzed taurine 0.11%)	Fillet histidine (g/kg muscle)	5.400 ± 0.400	4.800 ± 1.100	3	3	Hedges' g (LS variance)	0.58	-1.06 to 2.21	Higher measured value with taurine; 95% CI included 0 (imprecise estimate)	Not applicable—effect size estimated
Kotzamanis et al. (2020)	European seabass (Dicentrarchus labrax)	Taurine supplementation (5–20 g/kg) in a 15% fishmeal, high-plant-protein diet	T10 (10 g/kg added taurine; analyzed 1.06%)	C− (15% fishmeal; no added taurine; analyzed taurine 0.11%)	Fillet taurine (g/kg muscle)	5.200 ± 0.260	2.300 ± 0.300	3	3	Hedges' g (LS variance)	8.24	3.31 to 13.17	Higher muscle taurine deposition with taurine; 95% CI excluded 0	Not applicable—effect size estimated
Kotzamanis et al. (2020)	European seabass (Dicentrarchus labrax)	Taurine supplementation (5–20 g/kg) in a 15% fishmeal, high-plant-protein diet	T10 (10 g/kg added taurine; analyzed 1.06%)	C− (15% fishmeal; no added taurine; analyzed taurine 0.11%)	Fillet serine (g/kg muscle)	9.200 ± 0.490	8.500 ± 0.600	3	3	Hedges' g (LS variance)	1.02	-0.68 to 2.72	Higher measured value with taurine; 95% CI included 0 (imprecise estimate)	Not applicable—effect size estimated
Kotzamanis et al. (2020)	European seabass (Dicentrarchus labrax)	Taurine supplementation (5–20 g/kg) in a 15% fishmeal, high-plant-protein diet	T10 (10 g/kg added taurine; analyzed 1.06%)	C− (15% fishmeal; no added taurine; analyzed taurine 0.11%)	Fillet arginine (g/kg muscle)	14.200 ± 0.820	14.000 ± 0.510	3	3	Hedges' g (LS variance)	0.23	-1.37 to 1.84	Higher measured value with taurine; 95% CI included 0 (imprecise estimate)	Not applicable—effect size estimated
Kotzamanis et al. (2020)	European seabass (Dicentrarchus labrax)	Taurine supplementation (5–20 g/kg) in a 15% fishmeal, high-plant-protein diet	T10 (10 g/kg added taurine; analyzed 1.06%)	C− (15% fishmeal; no added taurine; analyzed taurine 0.11%)	Fillet glycine (g/kg muscle)	11.000 ± 0.550	11.700 ± 0.200	3	3	Hedges' g (LS variance)	-1.35	-3.12 to 0.42	Lower measured value with taurine; 95% CI included 0 (imprecise estimate)	Not applicable—effect size estimated
Kotzamanis et al. (2020)	European seabass (Dicentrarchus labrax)	Taurine supplementation (5–20 g/kg) in a 15% fishmeal, high-plant-protein diet	T10 (10 g/kg added taurine; analyzed 1.06%)	C− (15% fishmeal; no added taurine; analyzed taurine 0.11%)	Fillet Asx (g/kg muscle)	24.800 ± 0.570	24.300 ± 1.260	3	3	Hedges' g (LS variance)	0.41	-1.21 to 2.02	Higher measured value with taurine; 95% CI included 0 (imprecise estimate)	Not applicable—effect size estimated
Kotzamanis et al. (2020)	European seabass (Dicentrarchus labrax)	Taurine supplementation (5–20 g/kg) in a 15% fishmeal, high-plant-protein diet	T10 (10 g/kg added taurine; analyzed 1.06%)	C− (15% fishmeal; no added taurine; analyzed taurine 0.11%)	Fillet Glx (g/kg muscle)	35.300 ± 1.210	34.800 ± 1.590	3	3	Hedges' g (LS variance)	0.28	-1.33 to 1.89	Higher measured value with taurine; 95% CI included 0 (imprecise estimate)	Not applicable—effect size estimated
Kotzamanis et al. (2020)	European seabass (Dicentrarchus labrax)	Taurine supplementation (5–20 g/kg) in a 15% fishmeal, high-plant-protein diet	T10 (10 g/kg added taurine; analyzed 1.06%)	C− (15% fishmeal; no added taurine; analyzed taurine 0.11%)	Fillet threonine (g/kg muscle)	10.400 ± 0.280	10.100 ± 0.430	3	3	Hedges' g (LS variance)	0.66	-0.98 to 2.30	Higher measured value with taurine; 95% CI included 0 (imprecise estimate)	Not applicable—effect size estimated
Kotzamanis et al. (2020)	European seabass (Dicentrarchus labrax)	Taurine supplementation (5–20 g/kg) in a 15% fishmeal, high-plant-protein diet	T10 (10 g/kg added taurine; analyzed 1.06%)	C− (15% fishmeal; no added taurine; analyzed taurine 0.11%)	Fillet alanine (g/kg muscle)	14.100 ± 0.530	13.900 ± 0.520	3	3	Hedges' g (LS variance)	0.3	-1.31 to 1.91	Higher measured value with taurine; 95% CI included 0 (imprecise estimate)	Not applicable—effect size estimated
Kotzamanis et al. (2020)	European seabass (Dicentrarchus labrax)	Taurine supplementation (5–20 g/kg) in a 15% fishmeal, high-plant-protein diet	T10 (10 g/kg added taurine; analyzed 1.06%)	C− (15% fishmeal; no added taurine; analyzed taurine 0.11%)	Fillet proline (g/kg muscle)	7.100 ± 0.460	7.500 ± 0.170	3	3	Hedges' g (LS variance)	-0.92	-2.60 to 0.76	Lower measured value with taurine; 95% CI included 0 (imprecise estimate)	Not applicable—effect size estimated
Kotzamanis et al. (2020)	European seabass (Dicentrarchus labrax)	Taurine supplementation (5–20 g/kg) in a 15% fishmeal, high-plant-protein diet	T10 (10 g/kg added taurine; analyzed 1.06%)	C− (15% fishmeal; no added taurine; analyzed taurine 0.11%)	Fillet cystine (g/kg muscle)	0.900 ± 0.120	0.800 ± 0.400	3	3	Hedges' g (LS variance)	0.27	-1.34 to 1.88	Higher measured value with taurine; 95% CI included 0 (imprecise estimate)	Not applicable—effect size estimated
Kotzamanis et al. (2020)	European seabass (Dicentrarchus labrax)	Taurine supplementation (5–20 g/kg) in a 15% fishmeal, high-plant-protein diet	T10 (10 g/kg added taurine; analyzed 1.06%)	C− (15% fishmeal; no added taurine; analyzed taurine 0.11%)	Fillet lysine (g/kg muscle)	21.000 ± 0.500	20.200 ± 9.600	3	3	Hedges' g (LS variance)	0.09	-1.51 to 1.70	Higher measured value with taurine; 95% CI included 0 (imprecise estimate)	Not applicable—effect size estimated
Kotzamanis et al. (2020)	European seabass (Dicentrarchus labrax)	Taurine supplementation (5–20 g/kg) in a 15% fishmeal, high-plant-protein diet	T10 (10 g/kg added taurine; analyzed 1.06%)	C− (15% fishmeal; no added taurine; analyzed taurine 0.11%)	Fillet tyrosine (g/kg muscle)	8.100 ± 0.190	7.800 ± 0.410	3	3	Hedges' g (LS variance)	0.75	-0.91 to 2.40	Higher measured value with taurine; 95% CI included 0 (imprecise estimate)	Not applicable—effect size estimated
Kotzamanis et al. (2020)	European seabass (Dicentrarchus labrax)	Taurine supplementation (5–20 g/kg) in a 15% fishmeal, high-plant-protein diet	T10 (10 g/kg added taurine; analyzed 1.06%)	C− (15% fishmeal; no added taurine; analyzed taurine 0.11%)	Fillet methionine (g/kg muscle)	7.100 ± 0.170	7.000 ± 0.360	3	3	Hedges' g (LS variance)	0.28	-1.32 to 1.89	Higher measured value with taurine; 95% CI included 0 (imprecise estimate)	Not applicable—effect size estimated
Kotzamanis et al. (2020)	European seabass (Dicentrarchus labrax)	Taurine supplementation (5–20 g/kg) in a 15% fishmeal, high-plant-protein diet	T10 (10 g/kg added taurine; analyzed 1.06%)	C− (15% fishmeal; no added taurine; analyzed taurine 0.11%)	Fillet valine (g/kg muscle)	11.000 ± 0.200	10.500 ± 0.510	3	3	Hedges' g (LS variance)	1.03	-0.67 to 2.73	Higher measured value with taurine; 95% CI included 0 (imprecise estimate)	Not applicable—effect size estimated
Kotzamanis et al. (2020)	European seabass (Dicentrarchus labrax)	Taurine supplementation (5–20 g/kg) in a 15% fishmeal, high-plant-protein diet	T10 (10 g/kg added taurine; analyzed 1.06%)	C− (15% fishmeal; no added taurine; analyzed taurine 0.11%)	Fillet isoleucine (g/kg muscle)	10.300 ± 0.220	9.700 ± 6.400	3	3	Hedges' g (LS variance)	0.11	-1.50 to 1.71	Higher measured value with taurine; 95% CI included 0 (imprecise estimate)	Not applicable—effect size estimated
Kotzamanis et al. (2020)	European seabass (Dicentrarchus labrax)	Taurine supplementation (5–20 g/kg) in a 15% fishmeal, high-plant-protein diet	T10 (10 g/kg added taurine; analyzed 1.06%)	C− (15% fishmeal; no added taurine; analyzed taurine 0.11%)	Fillet leucine (g/kg muscle)	18.400 ± 0.410	17.800 ± 0.920	3	3	Hedges' g (LS variance)	0.67	-0.97 to 2.32	Higher measured value with taurine; 95% CI included 0 (imprecise estimate)	Not applicable—effect size estimated
Kotzamanis et al. (2020)	European seabass (Dicentrarchus labrax)	Taurine supplementation (5–20 g/kg) in a 15% fishmeal, high-plant-protein diet	T10 (10 g/kg added taurine; analyzed 1.06%)	C− (15% fishmeal; no added taurine; analyzed taurine 0.11%)	Fillet phenylalanine (g/kg muscle)	9.600 ± 0.170	9.400 ± 0.500	3	3	Hedges' g (LS variance)	0.43	-1.19 to 2.05	Higher measured value with taurine; 95% CI included 0 (imprecise estimate)	Not applicable—effect size estimated
Kotzamanis et al. (2020)	European seabass (Dicentrarchus labrax)	Taurine supplementation (5–20 g/kg) in a 15% fishmeal, high-plant-protein diet	T10 (10 g/kg added taurine; analyzed 1.06%)	C− (15% fishmeal; no added taurine; analyzed taurine 0.11%)	Muscle taurine (% of muscle protein)	0.233 ± 0.013	0.102 ± 0.013	3	3	Hedges' g (LS variance)	7.92	3.16 to 12.67	Higher muscle taurine deposition with taurine; 95% CI excluded 0	Not applicable—effect size estimated
Kotzamanis et al. (2020)	European seabass (Dicentrarchus labrax)	Taurine supplementation (5–20 g/kg) in a 15% fishmeal, high-plant-protein diet	T20 (20 g/kg added taurine; analyzed 2.02%)	C− (15% fishmeal; no added taurine; analyzed taurine 0.11%)	Survival (%)	94.000 ± 5.300	87.000 ± 9.600	3	3	Hedges' g (LS variance)	0.72	-0.93 to 2.37	Higher survival with taurine; 95% CI included 0 (imprecise estimate)	Not applicable—effect size estimated
Kotzamanis et al. (2020)	European seabass (Dicentrarchus labrax)	Taurine supplementation (5–20 g/kg) in a 15% fishmeal, high-plant-protein diet	T20 (20 g/kg added taurine; analyzed 2.02%)	C− (15% fishmeal; no added taurine; analyzed taurine 0.11%)	Final body weight (g)	169.000 ± 2.700	161.000 ± 2.200	3	3	Hedges' g (LS variance)	2.59	0.42 to 4.76	Higher growth outcome with taurine; 95% CI excluded 0	Not applicable—effect size estimated
Kotzamanis et al. (2020)	European seabass (Dicentrarchus labrax)	Taurine supplementation (5–20 g/kg) in a 15% fishmeal, high-plant-protein diet	T20 (20 g/kg added taurine; analyzed 2.02%)	C− (15% fishmeal; no added taurine; analyzed taurine 0.11%)	Weight gain (g)	91.000 ± 2.700	84.000 ± 2.700	3	3	Hedges' g (LS variance)	2.07	0.09 to 4.05	Higher growth outcome with taurine; 95% CI excluded 0	Not applicable—effect size estimated
Kotzamanis et al. (2020)	European seabass (Dicentrarchus labrax)	Taurine supplementation (5–20 g/kg) in a 15% fishmeal, high-plant-protein diet	T20 (20 g/kg added taurine; analyzed 2.02%)	C− (15% fishmeal; no added taurine; analyzed taurine 0.11%)	Daily growth index (%)	1.760 ± 0.040	1.660 ± 0.050	3	3	Hedges' g (LS variance)	1.76	-0.12 to 3.65	Higher growth outcome with taurine; 95% CI included 0 (imprecise estimate)	Not applicable—effect size estimated
Kotzamanis et al. (2020)	European seabass (Dicentrarchus labrax)	Taurine supplementation (5–20 g/kg) in a 15% fishmeal, high-plant-protein diet	T20 (20 g/kg added taurine; analyzed 2.02%)	C− (15% fishmeal; no added taurine; analyzed taurine 0.11%)	Total feed intake (g DM/fish)	113.000 ± 3.100	129.000 ± 15.100	3	3	Hedges' g (LS variance)	-1.17	-2.90 to 0.56	Lower feed intake with taurine; 95% CI included 0 (imprecise estimate)	Not applicable—effect size estimated
Kotzamanis et al. (2020)	European seabass (Dicentrarchus labrax)	Taurine supplementation (5–20 g/kg) in a 15% fishmeal, high-plant-protein diet	T20 (20 g/kg added taurine; analyzed 2.02%)	C− (15% fishmeal; no added taurine; analyzed taurine 0.11%)	Relative feed intake (% DM)	144.500 ± 3.800	166.200 ± 20.500	3	3	Hedges' g (LS variance)	-1.17	-2.91 to 0.56	Lower feed intake with taurine; 95% CI included 0 (imprecise estimate)	Not applicable—effect size estimated
Kotzamanis et al. (2020)	European seabass (Dicentrarchus labrax)	Taurine supplementation (5–20 g/kg) in a 15% fishmeal, high-plant-protein diet	T20 (20 g/kg added taurine; analyzed 2.02%)	C− (15% fishmeal; no added taurine; analyzed taurine 0.11%)	Specific growth rate (%/day)	1.080 ± 0.020	1.030 ± 0.030	3	3	Hedges' g (LS variance)	1.56	-0.26 to 3.39	Higher growth outcome with taurine; 95% CI included 0 (imprecise estimate)	Not applicable—effect size estimated

Study	Species	Strategy	Intervention	Comparator	Outcome and unit	Intervention mean ± SD	Comparator mean ± SD	n intervention	n comparator	Effect-size metric	Effect size	95% CI	Interpretation	Reason not estimated
Kotzamanis et al. (2020)	European seabass (Dicentrarchus labrax)	Taurine supplementation (5–20 g/kg) in a 15% fishmeal, high-plant-protein diet	T20 (20 g/kg added taurine; analyzed 2.02%)	C− (15% fishmeal; no added taurine; analyzed taurine 0.11%)	FCR	1.250 ± 0.060	1.530 ± 0.220	3	3	Hedges' g (direction reversed; LS variance)	1.39	-0.40 to 3.17	Lower, more favorable FCR with taurine; 95% CI included 0 (imprecise estimate)	Not applicable—effect size estimated
Kotzamanis et al. (2020)	European seabass (Dicentrarchus labrax)	Taurine supplementation (5–20 g/kg) in a 15% fishmeal, high-plant-protein diet	T20 (20 g/kg added taurine; analyzed 2.02%)	C− (15% fishmeal; no added taurine; analyzed taurine 0.11%)	PER	1.840 ± 0.060	1.540 ± 0.420	3	3	Hedges' g (LS variance)	0.8	-0.86 to 2.46	Higher, more favorable PER with taurine; 95% CI included 0 (imprecise estimate)	Not applicable—effect size estimated
Kotzamanis et al. (2020)	European seabass (Dicentrarchus labrax)	Taurine supplementation (5–20 g/kg) in a 15% fishmeal, high-plant-protein diet	T20 (20 g/kg added taurine; analyzed 2.02%)	C− (15% fishmeal; no added taurine; analyzed taurine 0.11%)	Thermal growth coefficient (×1,000)	0.640 ± 0.020	0.600 ± 0.020	3	3	Hedges' g (LS variance)	1.6	-0.24 to 3.43	Higher growth outcome with taurine; 95% CI included 0 (imprecise estimate)	Not applicable—effect size estimated
Kotzamanis et al. (2020)	European seabass (Dicentrarchus labrax)	Taurine supplementation (5–20 g/kg) in a 15% fishmeal, high-plant-protein diet	T20 (20 g/kg added taurine; analyzed 2.02%)	C− (15% fishmeal; no added taurine; analyzed taurine 0.11%)	Lipidosomatic index (%)	9.030 ± 1.120	9.510 ± 2.440	3	3	Hedges' g (LS variance)	-0.2	-1.81 to 1.40	Lower measured value with taurine; 95% CI included 0 (imprecise estimate)	Not applicable—effect size estimated
Kotzamanis et al. (2020)	European seabass (Dicentrarchus labrax)	Taurine supplementation (5–20 g/kg) in a 15% fishmeal, high-plant-protein diet	T20 (20 g/kg added taurine; analyzed 2.02%)	C− (15% fishmeal; no added taurine; analyzed taurine 0.11%)	Hepatosomatic index (%)	2.480 ± 0.110	2.160 ± 0.230	3	3	Hedges' g (LS variance)	1.42	-0.37 to 3.21	Higher measured value with taurine; 95% CI included 0 (imprecise estimate)	Not applicable—effect size estimated
Kotzamanis et al. (2020)	European seabass (Dicentrarchus labrax)	Taurine supplementation (5–20 g/kg) in a 15% fishmeal, high-plant-protein diet	T20 (20 g/kg added taurine; analyzed 2.02%)	C− (15% fishmeal; no added taurine; analyzed taurine 0.11%)	Whole-body moisture (g/kg fresh weight)	633.000 ± 2.000	607.000 ± 21.000	3	3	Hedges' g (LS variance)	1.39	-0.39 to 3.17	Higher measured value with taurine; 95% CI included 0 (imprecise estimate)	Not applicable—effect size estimated
Kotzamanis et al. (2020)	European seabass (Dicentrarchus labrax)	Taurine supplementation (5–20 g/kg) in a 15% fishmeal, high-plant-protein diet	T20 (20 g/kg added taurine; analyzed 2.02%)	C− (15% fishmeal; no added taurine; analyzed taurine 0.11%)	Whole-body protein (g/kg fresh weight)	158.000 ± 5.000	171.000 ± 6.000	3	3	Hedges' g (LS variance)	-1.88	-3.80 to 0.04	Lower measured value with taurine; 95% CI included 0 (imprecise estimate)	Not applicable—effect size estimated
Kotzamanis et al. (2020)	European seabass (Dicentrarchus labrax)	Taurine supplementation (5–20 g/kg) in a 15% fishmeal, high-plant-protein diet	T20 (20 g/kg added taurine; analyzed 2.02%)	C− (15% fishmeal; no added taurine; analyzed taurine 0.11%)	Whole-body lipid (g/kg fresh weight)	147.000 ± 10.000	160.000 ± 4.000	3	3	Hedges' g (LS variance)	-1.36	-3.14 to 0.41	Lower measured value with taurine; 95% CI included 0 (imprecise estimate)	Not applicable—effect size estimated
Kotzamanis et al. (2020)	European seabass (Dicentrarchus labrax)	Taurine supplementation (5–20 g/kg) in a 15% fishmeal, high-plant-protein diet	T20 (20 g/kg added taurine; analyzed 2.02%)	C− (15% fishmeal; no added taurine; analyzed taurine 0.11%)	Whole-body ash (g/kg fresh weight)	38.000 ± 5.000	25.000 ± 9.000	3	3	Hedges' g (LS variance)	1.42	-0.37 to 3.22	Higher measured value with taurine; 95% CI included 0 (imprecise estimate)	Not applicable—effect size estimated
Kotzamanis et al. (2020)	European seabass (Dicentrarchus labrax)	Taurine supplementation (5–20 g/kg) in a 15% fishmeal, high-plant-protein diet	T20 (20 g/kg added taurine; analyzed 2.02%)	C− (15% fishmeal; no added taurine; analyzed taurine 0.11%)	Muscle water (g/kg fresh weight)	762.000 ± 13.000	741.000 ± 2.300	3	3	Hedges' g (LS variance)	1.79	-0.10 to 3.69	Higher measured value with taurine; 95% CI included 0 (imprecise estimate)	Not applicable—effect size estimated
Kotzamanis et al. (2020)	European seabass (Dicentrarchus labrax)	Taurine supplementation (5–20 g/kg) in a 15% fishmeal, high-plant-protein diet	T20 (20 g/kg added taurine; analyzed 2.02%)	C− (15% fishmeal; no added taurine; analyzed taurine 0.11%)	Muscle protein (g/kg fresh weight)	215.000 ± 16.600	227.000 ± 17.000	3	3	Hedges' g (LS variance)	-0.57	-2.20 to 1.06	Lower measured value with taurine; 95% CI included 0 (imprecise estimate)	Not applicable—effect size estimated
Kotzamanis et al. (2020)	European seabass (Dicentrarchus labrax)	Taurine supplementation (5–20 g/kg) in a 15% fishmeal, high-plant-protein diet	T20 (20 g/kg added taurine; analyzed 2.02%)	C− (15% fishmeal; no added taurine; analyzed taurine 0.11%)	Muscle lipid (g/kg fresh weight)	25.000 ± 0.800	26.000 ± 0.100	3	3	Hedges' g (LS variance)	-1.4	-3.19 to 0.39	Lower measured value with taurine; 95% CI included 0 (imprecise estimate)	Not applicable—effect size estimated
Kotzamanis et al. (2020)	European seabass (Dicentrarchus labrax)	Taurine supplementation (5–20 g/kg) in a 15% fishmeal, high-plant-protein diet	T20 (20 g/kg added taurine; analyzed 2.02%)	C− (15% fishmeal; no added taurine; analyzed taurine 0.11%)	Muscle ash (g/kg fresh weight)	14.000 ± 0.300	14.000 ± 0.400	3	3	Hedges' g (LS variance)	0.0	-1.60 to 1.60	No observed mean difference; 95% CI included 0 (imprecise estimate)	Not applicable—effect size estimated
Kotzamanis et al. (2020)	European seabass (Dicentrarchus labrax)	Taurine supplementation (5–20 g/kg) in a 15% fishmeal, high-plant-protein diet	T20 (20 g/kg added taurine; analyzed 2.02%)	C− (15% fishmeal; no added taurine; analyzed taurine 0.11%)	Fillet histidine (g/kg muscle)	4.900 ± 0.400	4.800 ± 1.100	3	3	Hedges' g (LS variance)	0.1	-1.50 to 1.70	Higher measured value with taurine; 95% CI included 0 (imprecise estimate)	Not applicable—effect size estimated
Kotzamanis et al. (2020)	European seabass (Dicentrarchus labrax)	Taurine supplementation (5–20 g/kg) in a 15% fishmeal, high-plant-protein diet	T20 (20 g/kg added taurine; analyzed 2.02%)	C− (15% fishmeal; no added taurine; analyzed taurine 0.11%)	Fillet taurine (g/kg muscle)	5.300 ± 0.660	2.300 ± 0.300	3	3	Hedges' g (LS variance)	4.67	1.58 to 7.76	Higher muscle taurine deposition with taurine; 95% CI excluded 0	Not applicable—effect size estimated
Kotzamanis et al. (2020)	European seabass (Dicentrarchus labrax)	Taurine supplementation (5–20 g/kg) in a 15% fishmeal, high-plant-protein diet	T20 (20 g/kg added taurine; analyzed 2.02%)	C− (15% fishmeal; no added taurine; analyzed taurine 0.11%)	Fillet serine (g/kg muscle)	8.600 ± 0.350	8.500 ± 0.600	3	3	Hedges' g (LS variance)	0.16	-1.44 to 1.77	Higher measured value with taurine; 95% CI included 0 (imprecise estimate)	Not applicable—effect size estimated
Kotzamanis et al. (2020)	European seabass (Dicentrarchus labrax)	Taurine supplementation (5–20 g/kg) in a 15% fishmeal, high-plant-protein diet	T20 (20 g/kg added taurine; analyzed 2.02%)	C− (15% fishmeal; no added taurine; analyzed taurine 0.11%)	Fillet arginine (g/kg muscle)	13.400 ± 0.650	14.000 ± 0.510	3	3	Hedges' g (LS variance)	-0.82	-2.49 to 0.85	Lower measured value with taurine; 95% CI included 0 (imprecise estimate)	Not applicable—effect size estimated
Kotzamanis et al. (2020)	European seabass (Dicentrarchus labrax)	Taurine supplementation (5–20 g/kg) in a 15% fishmeal, high-plant-protein diet	T20 (20 g/kg added taurine; analyzed 2.02%)	C− (15% fishmeal; no added taurine; analyzed taurine 0.11%)	Fillet glycine (g/kg muscle)	10.100 ± 0.360	11.700 ± 0.200	3	3	Hedges' g (LS variance)	-4.38	-7.34 to -1.43	Lower measured value with taurine; 95% CI excluded 0	Not applicable—effect size estimated
Kotzamanis et al. (2020)	European seabass (Dicentrarchus labrax)	Taurine supplementation (5–20 g/kg) in a 15% fishmeal, high-plant-protein diet	T20 (20 g/kg added taurine; analyzed 2.02%)	C− (15% fishmeal; no added taurine; analyzed taurine 0.11%)	Fillet Asx (g/kg muscle)	22.600 ± 0.570	24.300 ± 1.260	3	3	Hedges' g (LS variance)	-1.39	-3.17 to 0.40	Lower measured value with taurine; 95% CI included 0 (imprecise estimate)	Not applicable—effect size estimated
Kotzamanis et al. (2020)	European seabass (Dicentrarchus labrax)	Taurine supplementation (5–20 g/kg) in a 15% fishmeal, high-plant-protein diet	T20 (20 g/kg added taurine; analyzed 2.02%)	C− (15% fishmeal; no added taurine; analyzed taurine 0.11%)	Fillet Glx (g/kg muscle)	32.400 ± 1.040	34.800 ± 1.590	3	3	Hedges' g (LS variance)	-1.43	-3.22 to 0.37	Lower measured value with taurine; 95% CI included 0 (imprecise estimate)	Not applicable—effect size estimated
Kotzamanis et al. (2020)	European seabass (Dicentrarchus labrax)	Taurine supplementation (5–20 g/kg) in a 15% fishmeal, high-plant-protein diet	T20 (20 g/kg added taurine; analyzed 2.02%)	C− (15% fishmeal; no added taurine; analyzed taurine 0.11%)	Fillet threonine (g/kg muscle)	9.700 ± 0.280	10.100 ± 0.430	3	3	Hedges' g (LS variance)	-0.88	-2.56 to 0.80	Lower measured value with taurine; 95% CI included 0 (imprecise estimate)	Not applicable—effect size estimated
Kotzamanis et al. (2020)	European seabass (Dicentrarchus labrax)	Taurine supplementation (5–20 g/kg) in a 15% fishmeal, high-plant-protein diet	T20 (20 g/kg added taurine; analyzed 2.02%)	C− (15% fishmeal; no added taurine; analyzed taurine 0.11%)	Fillet alanine (g/kg muscle)	13.000 ± 0.340	13.900 ± 0.520	3	3	Hedges' g (LS variance)	-1.63	-3.48 to 0.21	Lower measured value with taurine; 95% CI included 0 (imprecise estimate)	Not applicable—effect size estimated
Kotzamanis et al. (2020)	European seabass (Dicentrarchus labrax)	Taurine supplementation (5–20 g/kg) in a 15% fishmeal, high-plant-protein diet	T20 (20 g/kg added taurine; analyzed 2.02%)	C− (15% fishmeal; no added taurine; analyzed taurine 0.11%)	Fillet proline (g/kg muscle)	6.800 ± 0.320	7.500 ± 0.170	3	3	Hedges' g (LS variance)	-2.18	-4.20 to -0.16	Lower measured value with taurine; 95% CI excluded 0	Not applicable—effect size estimated
Kotzamanis et al. (2020)	European seabass (Dicentrarchus labrax)	Taurine supplementation (5–20 g/kg) in a 15% fishmeal, high-plant-protein diet	T20 (20 g/kg added taurine; analyzed 2.02%)	C− (15% fishmeal; no added taurine; analyzed taurine 0.11%)	Fillet cystine (g/kg muscle)	0.800 ± 0.100	0.800 ± 0.400	3	3	Hedges' g (LS variance)	0.0	-1.60 to 1.60	No observed mean difference; 95% CI included 0 (imprecise estimate)	Not applicable—effect size estimated
Kotzamanis et al. (2020)	European seabass (Dicentrarchus labrax)	Taurine supplementation (5–20 g/kg) in a 15% fishmeal, high-plant-protein diet	T20 (20 g/kg added taurine; analyzed 2.02%)	C− (15% fishmeal; no added taurine; analyzed taurine 0.11%)	Fillet lysine (g/kg muscle)	19.400 ± 0.870	20.200 ± 9.600	3	3	Hedges' g (LS variance)	-0.09	-1.69 to 1.51	Lower measured value with taurine; 95% CI included 0 (imprecise estimate)	Not applicable—effect size estimated
Kotzamanis et al. (2020)	European seabass (Dicentrarchus labrax)	Taurine supplementation (5–20 g/kg) in a 15% fishmeal, high-plant-protein diet	T20 (20 g/kg added taurine; analyzed 2.02%)	C− (15% fishmeal; no added taurine; analyzed taurine 0.11%)	Fillet tyrosine (g/kg muscle)	7.400 ± 0.170	7.800 ± 0.410	3	3	Hedges' g (LS variance)	-1.02	-2.72 to 0.68	Lower measured value with taurine; 95% CI included 0 (imprecise estimate)	Not applicable—effect size estimated
Kotzamanis et al. (2020)	European seabass (Dicentrarchus labrax)	Taurine supplementation (5–20 g/kg) in a 15% fishmeal, high-plant-protein diet	T20 (20 g/kg added taurine; analyzed 2.02%)	C− (15% fishmeal; no added taurine; analyzed taurine 0.11%)	Fillet methionine (g/kg muscle)	6.600 ± 0.150	7.000 ± 0.360	3	3	Hedges' g (LS variance)	-1.16	-2.89 to 0.57	Lower measured value with taurine; 95% CI included 0 (imprecise estimate)	Not applicable—effect size estimated
Kotzamanis et al. (2020)	European seabass (Dicentrarchus labrax)	Taurine supplementation (5–20 g/kg) in a 15% fishmeal, high-plant-protein diet	T20 (20 g/kg added taurine; analyzed 2.02%)	C− (15% fishmeal; no added taurine; analyzed taurine 0.11%)	Fillet valine (g/kg muscle)	10.100 ± 0.230	10.500 ± 0.510	3	3	Hedges' g (LS variance)	-0.81	-2.47 to 0.86	Lower measured value with taurine; 95% CI included 0 (imprecise estimate)	Not applicable—effect size estimated
Kotzamanis et al. (2020)	European seabass (Dicentrarchus labrax)	Taurine supplementation (5–20 g/kg) in a 15% fishmeal, high-plant-protein diet	T20 (20 g/kg added taurine; analyzed 2.02%)	C− (15% fishmeal; no added taurine; analyzed taurine 0.11%)	Fillet isoleucine (g/kg muscle)	9.400 ± 0.250	9.700 ± 6.400	3	3	Hedges' g (LS variance)	-0.05	-1.65 to 1.55	Lower measured value with taurine; 95% CI included 0 (imprecise estimate)	Not applicable—effect size estimated

Supplementary Table S3

Study	Species	Strategy	Intervention	Comparator	Outcome and unit	Intervention mean ± SD	Comparator mean ± SD	n intervention	n comparator	Effect-size metric	Effect size	95% CI	Interpretation	Reason not estimated
Kotzamanis et al. (2020)	European seabass (Dicentrarchus labrax)	Taurine supplementation (5–20 g/kg) in a 15% fishmeal, high-plant-protein diet	T20 (20 g/kg added taurine; analyzed 2.02%)	C− (15% fishmeal; no added taurine; analyzed taurine 0.11%)	Fillet leucine (g/kg muscle)	16.900 ± 0.470	17.800 ± 0.920	3	3	Hedges' g (LS variance)	-0.98	-2.68 to 0.71	Lower measured value with taurine; 95% CI included 0 (imprecise estimate)	Not applicable—effect size estimated
Kotzamanis et al. (2020)	European seabass (Dicentrarchus labrax)	Taurine supplementation (5–20 g/kg) in a 15% fishmeal, high-plant-protein diet	T20 (20 g/kg added taurine; analyzed 2.02%)	C− (15% fishmeal; no added taurine; analyzed taurine 0.11%)	Fillet phenylalanine (g/kg muscle)	8.800 ± 0.200	9.400 ± 0.500	3	3	Hedges' g (LS variance)	-1.26	-3.01 to 0.49	Lower measured value with taurine; 95% CI included 0 (imprecise estimate)	Not applicable—effect size estimated
Kotzamanis et al. (2020)	European seabass (Dicentrarchus labrax)	Taurine supplementation (5–20 g/kg) in a 15% fishmeal, high-plant-protein diet	T20 (20 g/kg added taurine; analyzed 2.02%)	C− (15% fishmeal; no added taurine; analyzed taurine 0.11%)	Muscle taurine (% of muscle protein)	0.248 ± 0.031	0.102 ± 0.013	3	3	Hedges' g (LS variance)	4.9	1.70 to 8.10	Higher muscle taurine deposition with taurine; 95% CI excluded 0	Not applicable—effect size estimated
Kotzamanis et al. (2020)	European seabass (Dicentrarchus labrax)	Taurine supplementation (5–20 g/kg) in a 15% fishmeal, high-plant-protein diet	T10 (10 g/kg added taurine; analyzed 1.06%)	C− (15% fishmeal; no added taurine; analyzed taurine 0.11%)	Fillet instrumental hardness	2.650 ± 0.100	2.050 ± 0.050	3	3	Hedges' g (LS variance)	6.06	2.27 to 9.84	Higher instrumental fillet hardness with taurine; 95% CI excluded 0	Not applicable—effect size estimated
Kotzamanis et al. (2020)	European seabass (Dicentrarchus labrax)	Taurine supplementation (5–20 g/kg) in a 15% fishmeal, high-plant-protein diet	T10 (10 g/kg added taurine; analyzed 1.06%)	C− (15% fishmeal; no added taurine; analyzed taurine 0.11%)	Fillet instrumental chewiness	0.790 ± 0.080	0.560 ± 0.070	3	3	Hedges' g (LS variance)	2.44	0.33 to 4.56	Higher instrumental fillet chewiness with taurine; 95% CI excluded 0	Not applicable—effect size estimated
Kotzamanis et al. (2020)	European seabass (Dicentrarchus labrax)	Taurine supplementation (5–20 g/kg) in a 15% fishmeal, high-plant-protein diet	T10 (10 g/kg added taurine; analyzed 1.06%)	C− (15% fishmeal; no added taurine; analyzed taurine 0.11%)	Fillet instrumental adhesiveness	-0.050 ± 0.010	-0.018 ± 0.002	3	3	Hedges' g (direction reversed; LS variance)	3.54	0.98 to 6.10	Lower (more negative) adhesiveness with taurine; 95% CI excluded 0	Not applicable—effect size estimated
Kotzamanis et al. (2020)	European seabass (Dicentrarchus labrax)	Taurine supplementation (5–20 g/kg) in a 15% fishmeal, high-plant-protein diet	T20 (20 g/kg added taurine; analyzed 2.02%)	C− (15% fishmeal; no added taurine; analyzed taurine 0.11%)	Fillet instrumental hardness	3.730 ± 0.190	2.050 ± 0.050	3	3	Hedges' g (LS variance)	9.65	3.96 to 15.34	Higher instrumental fillet hardness with taurine; 95% CI excluded 0	Not applicable—effect size estimated
Kotzamanis et al. (2020)	European seabass (Dicentrarchus labrax)	Taurine supplementation (5–20 g/kg) in a 15% fishmeal, high-plant-protein diet	T20 (20 g/kg added taurine; analyzed 2.02%)	C− (15% fishmeal; no added taurine; analyzed taurine 0.11%)	Fillet instrumental chewiness	1.070 ± 0.170	0.560 ± 0.070	3	3	Hedges' g (LS variance)	3.13	0.74 to 5.52	Higher instrumental fillet chewiness with taurine; 95% CI excluded 0	Not applicable—effect size estimated
Kotzamanis et al. (2020)	European seabass (Dicentrarchus labrax)	Taurine supplementation (5–20 g/kg) in a 15% fishmeal, high-plant-protein diet	T20 (20 g/kg added taurine; analyzed 2.02%)	C− (15% fishmeal; no added taurine; analyzed taurine 0.11%)	Fillet instrumental adhesiveness	-0.063 ± 0.009	-0.018 ± 0.002	3	3	Hedges' g (direction reversed; LS variance)	5.51	2.00 to 9.01	Lower (more negative) adhesiveness with taurine; 95% CI excluded 0	Not applicable—effect size estimated
Kotzamanis et al. (2020)	European seabass (Dicentrarchus labrax)	Taurine supplementation (5–20 g/kg) in a 15% fishmeal, high-plant-protein diet	T10 (10 g/kg added taurine; analyzed 1.06%)	C− (15% fishmeal; no added taurine; analyzed taurine 0.11%)	Sensory darkness score	3.83 ± 3.04	5.33 ± 3.39	Not reported at tank level	Not reported at tank level	Not estimable	Not estimable	Not estimable	Descriptive sensory score reported by the original study	Scores were generated by a 20-person taste panel; independent tank-level variance and sample size were not reported
Kotzamanis et al. (2020)	European seabass (Dicentrarchus labrax)	Taurine supplementation (5–20 g/kg) in a 15% fishmeal, high-plant-protein diet	T10 (10 g/kg added taurine; analyzed 1.06%)	C− (15% fishmeal; no added taurine; analyzed taurine 0.11%)	Sensory flavor score	7.68 ± 2.68	6.42 ± 2.29	Not reported at tank level	Not reported at tank level	Not estimable	Not estimable	Not estimable	Descriptive sensory score reported by the original study	Scores were generated by a 20-person taste panel; independent tank-level variance and sample size were not reported
Kotzamanis et al. (2020)	European seabass (Dicentrarchus labrax)	Taurine supplementation (5–20 g/kg) in a 15% fishmeal, high-plant-protein diet	T10 (10 g/kg added taurine; analyzed 1.06%)	C− (15% fishmeal; no added taurine; analyzed taurine 0.11%)	Sensory taste score	7.21 ± 2.90	6.53 ± 2.57	Not reported at tank level	Not reported at tank level	Not estimable	Not estimable	Not estimable	Descriptive sensory score reported by the original study	Scores were generated by a 20-person taste panel; independent tank-level variance and sample size were not reported
Kotzamanis et al. (2020)	European seabass (Dicentrarchus labrax)	Taurine supplementation (5–20 g/kg) in a 15% fishmeal, high-plant-protein diet	T10 (10 g/kg added taurine; analyzed 1.06%)	C− (15% fishmeal; no added taurine; analyzed taurine 0.11%)	Sensory hardness score	7.00 ± 2.27	5.26 ± 2.36	Not reported at tank level	Not reported at tank level	Not estimable	Not estimable	Not estimable	Descriptive sensory score reported by the original study	Scores were generated by a 20-person taste panel; independent tank-level variance and sample size were not reported
Kotzamanis et al. (2020)	European seabass (Dicentrarchus labrax)	Taurine supplementation (5–20 g/kg) in a 15% fishmeal, high-plant-protein diet	T10 (10 g/kg added taurine; analyzed 1.06%)	C− (15% fishmeal; no added taurine; analyzed taurine 0.11%)	Sensory elasticity score	6.84 ± 2.70	6.42 ± 2.58	Not reported at tank level	Not reported at tank level	Not estimable	Not estimable	Not estimable	Descriptive sensory score reported by the original study	Scores were generated by a 20-person taste panel; independent tank-level variance and sample size were not reported
Kotzamanis et al. (2020)	European seabass (Dicentrarchus labrax)	Taurine supplementation (5–20 g/kg) in a 15% fishmeal, high-plant-protein diet	T10 (10 g/kg added taurine; analyzed 1.06%)	C− (15% fishmeal; no added taurine; analyzed taurine 0.11%)	Sensory cohesiveness score	6.53 ± 2.48	6.58 ± 2.50	Not reported at tank level	Not reported at tank level	Not estimable	Not estimable	Not estimable	Descriptive sensory score reported by the original study	Scores were generated by a 20-person taste panel; independent tank-level variance and sample size were not reported
Kotzamanis et al. (2020)	European seabass (Dicentrarchus labrax)	Taurine supplementation (5–20 g/kg) in a 15% fishmeal, high-plant-protein diet	T10 (10 g/kg added taurine; analyzed 1.06%)	C− (15% fishmeal; no added taurine; analyzed taurine 0.11%)	Sensory chewiness score	7.05 ± 2.88	6.47 ± 2.57	Not reported at tank level	Not reported at tank level	Not estimable	Not estimable	Not estimable	Descriptive sensory score reported by the original study	Scores were generated by a 20-person taste panel; independent tank-level variance and sample size were not reported
Kotzamanis et al. (2020)	European seabass (Dicentrarchus labrax)	Taurine supplementation (5–20 g/kg) in a 15% fishmeal, high-plant-protein diet	T10 (10 g/kg added taurine; analyzed 1.06%)	C− (15% fishmeal; no added taurine; analyzed taurine 0.11%)	Sensory likeness score	6.21 ± 2.41	5.58 ± 2.38	Not reported at tank level	Not reported at tank level	Not estimable	Not estimable	Not estimable	Descriptive sensory score reported by the original study	Scores were generated by a 20-person taste panel; independent tank-level variance and sample size were not reported
Kotzamanis et al. (2020)	European seabass (Dicentrarchus labrax)	Taurine supplementation (5–20 g/kg) in a 15% fishmeal, high-plant-protein diet	T20 (20 g/kg added taurine; analyzed 2.02%)	C− (15% fishmeal; no added taurine; analyzed taurine 0.11%)	Sensory darkness score	4.75 ± 3.13	5.33 ± 3.39	Not reported at tank level	Not reported at tank level	Not estimable	Not estimable	Not estimable	Descriptive sensory score reported by the original study	Scores were generated by a 20-person taste panel; independent tank-level variance and sample size were not reported
Kotzamanis et al. (2020)	European seabass (Dicentrarchus labrax)	Taurine supplementation (5–20 g/kg) in a 15% fishmeal, high-plant-protein diet	T20 (20 g/kg added taurine; analyzed 2.02%)	C− (15% fishmeal; no added taurine; analyzed taurine 0.11%)	Sensory flavor score	6.32 ± 2.65	6.42 ± 2.29	Not reported at tank level	Not reported at tank level	Not estimable	Not estimable	Not estimable	Descriptive sensory score reported by the original study	Scores were generated by a 20-person taste panel; independent tank-level variance and sample size were not reported
Kotzamanis et al. (2020)	European seabass (Dicentrarchus labrax)	Taurine supplementation (5–20 g/kg) in a 15% fishmeal, high-plant-protein diet	T20 (20 g/kg added taurine; analyzed 2.02%)	C− (15% fishmeal; no added taurine; analyzed taurine 0.11%)	Sensory taste score	5.84 ± 2.33	6.53 ± 2.57	Not reported at tank level	Not reported at tank level	Not estimable	Not estimable	Not estimable	Descriptive sensory score reported by the original study	Scores were generated by a 20-person taste panel; independent tank-level variance and sample size were not reported
Kotzamanis et al. (2020)	European seabass (Dicentrarchus labrax)	Taurine supplementation (5–20 g/kg) in a 15% fishmeal, high-plant-protein diet	T20 (20 g/kg added taurine; analyzed 2.02%)	C− (15% fishmeal; no added taurine; analyzed taurine 0.11%)	Sensory hardness score	6.00 ± 2.12	5.26 ± 2.36	Not reported at tank level	Not reported at tank level	Not estimable	Not estimable	Not estimable	Descriptive sensory score reported by the original study	Scores were generated by a 20-person taste panel; independent tank-level variance and sample size were not reported
Kotzamanis et al. (2020)	European seabass (Dicentrarchus labrax)	Taurine supplementation (5–20 g/kg) in a 15% fishmeal, high-plant-protein diet	T20 (20 g/kg added taurine; analyzed 2.02%)	C− (15% fishmeal; no added taurine; analyzed taurine 0.11%)	Sensory elasticity score	6.26 ± 2.62	6.42 ± 2.58	Not reported at tank level	Not reported at tank level	Not estimable	Not estimable	Not estimable	Descriptive sensory score reported by the original study	Scores were generated by a 20-person taste panel; independent tank-level variance and sample size were not reported
Kotzamanis et al. (2020)	European seabass (Dicentrarchus labrax)	Taurine supplementation (5–20 g/kg) in a 15% fishmeal, high-plant-protein diet	T20 (20 g/kg added taurine; analyzed 2.02%)	C− (15% fishmeal; no added taurine; analyzed taurine 0.11%)	Sensory cohesiveness score	6.74 ± 2.24	6.58 ± 2.50	Not reported at tank level	Not reported at tank level	Not estimable	Not estimable	Not estimable	Descriptive sensory score reported by the original study	Scores were generated by a 20-person taste panel; independent tank-level variance and sample size were not reported
Kotzamanis et al. (2020)	European seabass (Dicentrarchus labrax)	Taurine supplementation (5–20 g/kg) in a 15% fishmeal, high-plant-protein diet	T20 (20 g/kg added taurine; analyzed 2.02%)	C− (15% fishmeal; no added taurine; analyzed taurine 0.11%)	Sensory chewiness score	6.58 ± 2.83	6.47 ± 2.57	Not reported at tank level	Not reported at tank level	Not estimable	Not estimable	Not estimable	Descriptive sensory score reported by the original study	Scores were generated by a 20-person taste panel; independent tank-level variance and sample size were not reported
Kotzamanis et al. (2020)	European seabass (Dicentrarchus labrax)	Taurine supplementation (5–20 g/kg) in a 15% fishmeal, high-plant-protein diet	T20 (20 g/kg added taurine; analyzed 2.02%)	C− (15% fishmeal; no added taurine; analyzed taurine 0.11%)	Sensory likeness score	5.95 ± 1.78	5.58 ± 2.38	Not reported at tank level	Not reported at tank level	Not estimable	Not estimable	Not estimable	Descriptive sensory score reported by the original study	Scores were generated by a 20-person taste panel; independent tank-level variance and sample size were not reported
Kotzamanis et al. (2020)	European seabass (Dicentrarchus labrax)	Taurine supplementation (5–20 g/kg) in a 15% fishmeal, high-plant-protein diet	T10 (10 g/kg added taurine; analyzed 1.06%)	C− (15% fishmeal; no added taurine; analyzed taurine 0.11%)	Total viable count growth rate at 0 °C (d−1)	0.43 ± 0.09	0.43 ± 0.12	Unclear	Unclear	Not estimable	Not estimable	Not estimable	No significant dietary effect was reported	SD reflected regression-model parameter variation rather than independent tank-level replication; experimental-unit n was unclear
Kotzamanis et al. (2020)	European seabass (Dicentrarchus labrax)	Taurine supplementation (5–20 g/kg) in a 15% fishmeal, high-plant-protein diet	T10 (10 g/kg added taurine; analyzed 1.06%)	C− (15% fishmeal; no added taurine; analyzed taurine 0.11%)	Total viable count growth rate at 5 °C (d−1)	0.48 ± 0.22	0.46 ± 0.12	Unclear	Unclear	Not estimable	Not estimable	Not estimable	No significant dietary effect was reported	SD reflected regression-model parameter variation rather than independent tank-level replication; experimental-unit n was unclear
Kotzamanis et al. (2020)	European seabass (Dicentrarchus labrax)	Taurine supplementation (5–20 g/kg) in a 15% fishmeal, high-plant-protein diet	T10 (10 g/kg added taurine; analyzed 1.06%)	C− (15% fishmeal; no added taurine; analyzed taurine 0.11%)	Pseudomonas growth rate at 0 °C (d−1)	0.49 ± 0.08	0.48 ± 0.15	Unclear	Unclear	Not estimable	Not estimable	Not estimable	No significant dietary effect was reported	SD reflected regression-model parameter variation rather than independent tank-level replication; experimental-unit n was unclear
Kotzamanis et al. (2020)	European seabass (Dicentrarchus labrax)	Taurine supplementation (5–20 g/kg) in a 15% fishmeal, high-plant-protein diet	T10 (10 g/kg added taurine; analyzed 1.06%)	C− (15% fishmeal; no added taurine; analyzed taurine 0.11%)	Pseudomonas growth rate at 5 °C (d−1)	0.68 ± 0.12	0.59 ± 0.10	Unclear	Unclear	Not estimable	Not estimable	Not estimable	No significant dietary effect was reported	SD reflected regression-model parameter variation rather than independent tank-level replication; experimental-unit n was unclear

Supplementary Table S3

Study	Species	Strategy	Intervention	Comparator	Outcome and unit	Intervention mean ± SD	Comparator mean ± SD	n intervention	n comparator	Effect-size metric	Effect size	95% CI	Interpretation	Reason not estimated
Kotzamanis et al. (2020)	European seabass (Dicentrarchus labrax)	Taurine supplementation (5–20 g/kg) in a 15% fishmeal, high-plant-protein diet	T10 (10 g/kg added taurine; analyzed 1.06%)	C− (15% fishmeal; no added taurine; analyzed taurine 0.11%)	Enterobacteriaceae growth rate at 0 °C (d−1)	0.59 ± 0.13	0.47 ± 0.11	Unclear	Unclear	Not estimable	Not estimable	Not estimable	No significant dietary effect was reported	SD reflected regression-model parameter variation rather than independent tank-level replication; experimental-unit n was unclear
Kotzamanis et al. (2020)	European seabass (Dicentrarchus labrax)	Taurine supplementation (5–20 g/kg) in a 15% fishmeal, high-plant-protein diet	T10 (10 g/kg added taurine; analyzed 1.06%)	C− (15% fishmeal; no added taurine; analyzed taurine 0.11%)	Enterobacteriaceae growth rate at 5 °C (d−1)	0.85 ± 0.01	0.78 ± 0.01	Unclear	Unclear	Not estimable	Not estimable	Not estimable	No significant dietary effect was reported	SD reflected regression-model parameter variation rather than independent tank-level replication; experimental-unit n was unclear
Kotzamanis et al. (2020)	European seabass (Dicentrarchus labrax)	Taurine supplementation (5–20 g/kg) in a 15% fishmeal, high-plant-protein diet	T20 (20 g/kg added taurine; analyzed 2.02%)	C− (15% fishmeal; no added taurine; analyzed taurine 0.11%)	Total viable count growth rate at 0 °C (d−1)	0.45 ± 0.08	0.43 ± 0.12	Unclear	Unclear	Not estimable	Not estimable	Not estimable	No significant dietary effect was reported	SD reflected regression-model parameter variation rather than independent tank-level replication; experimental-unit n was unclear
Kotzamanis et al. (2020)	European seabass (Dicentrarchus labrax)	Taurine supplementation (5–20 g/kg) in a 15% fishmeal, high-plant-protein diet	T20 (20 g/kg added taurine; analyzed 2.02%)	C− (15% fishmeal; no added taurine; analyzed taurine 0.11%)	Total viable count growth rate at 5 °C (d−1)	0.46 ± 0.17	0.46 ± 0.12	Unclear	Unclear	Not estimable	Not estimable	Not estimable	No significant dietary effect was reported	SD reflected regression-model parameter variation rather than independent tank-level replication; experimental-unit n was unclear
Kotzamanis et al. (2020)	European seabass (Dicentrarchus labrax)	Taurine supplementation (5–20 g/kg) in a 15% fishmeal, high-plant-protein diet	T20 (20 g/kg added taurine; analyzed 2.02%)	C− (15% fishmeal; no added taurine; analyzed taurine 0.11%)	Pseudomonas growth rate at 0 °C (d−1)	0.51 ± 0.06	0.48 ± 0.15	Unclear	Unclear	Not estimable	Not estimable	Not estimable	No significant dietary effect was reported	SD reflected regression-model parameter variation rather than independent tank-level replication; experimental-unit n was unclear
Kotzamanis et al. (2020)	European seabass (Dicentrarchus labrax)	Taurine supplementation (5–20 g/kg) in a 15% fishmeal, high-plant-protein diet	T20 (20 g/kg added taurine; analyzed 2.02%)	C− (15% fishmeal; no added taurine; analyzed taurine 0.11%)	Pseudomonas growth rate at 5 °C (d−1)	0.59 ± 0.24	0.59 ± 0.10	Unclear	Unclear	Not estimable	Not estimable	Not estimable	No significant dietary effect was reported	SD reflected regression-model parameter variation rather than independent tank-level replication; experimental-unit n was unclear
Kotzamanis et al. (2020)	European seabass (Dicentrarchus labrax)	Taurine supplementation (5–20 g/kg) in a 15% fishmeal, high-plant-protein diet	T20 (20 g/kg added taurine; analyzed 2.02%)	C− (15% fishmeal; no added taurine; analyzed taurine 0.11%)	Enterobacteriaceae growth rate at 0 °C (d−1)	0.51 ± 0.16	0.47 ± 0.11	Unclear	Unclear	Not estimable	Not estimable	Not estimable	No significant dietary effect was reported	SD reflected regression-model parameter variation rather than independent tank-level replication; experimental-unit n was unclear
Kotzamanis et al. (2020)	European seabass (Dicentrarchus labrax)	Taurine supplementation (5–20 g/kg) in a 15% fishmeal, high-plant-protein diet	T20 (20 g/kg added taurine; analyzed 2.02%)	C− (15% fishmeal; no added taurine; analyzed taurine 0.11%)	Enterobacteriaceae growth rate at 5 °C (d−1)	0.74 ± 0.10	0.78 ± 0.01	Unclear	Unclear	Not estimable	Not estimable	Not estimable	No significant dietary effect was reported	SD reflected regression-model parameter variation rather than independent tank-level replication; experimental-unit n was unclear
Kotzamanis et al. (2020)	European seabass (Dicentrarchus labrax)	Taurine supplementation (5–20 g/kg) in a 15% fishmeal, high-plant-protein diet	T10 (10 g/kg added taurine; analyzed 1.06%)	C− (15% fishmeal; no added taurine; analyzed taurine 0.11%)	Shelf life at 0 °C (days)	12 days; SD not reported	11 days; SD not reported	Not reported	Not reported	Not estimable	Not estimable	Not estimable	Shelf-life duration reported descriptively	Variance and independent experimental-unit sample size were not reported
Kotzamanis et al. (2020)	European seabass (Dicentrarchus labrax)	Taurine supplementation (5–20 g/kg) in a 15% fishmeal, high-plant-protein diet	T10 (10 g/kg added taurine; analyzed 1.06%)	C− (15% fishmeal; no added taurine; analyzed taurine 0.11%)	Shelf life at 5 °C (days)	6 days; SD not reported	6 days; SD not reported	Not reported	Not reported	Not estimable	Not estimable	Not estimable	Shelf-life duration reported descriptively	Variance and independent experimental-unit sample size were not reported
Kotzamanis et al. (2020)	European seabass (Dicentrarchus labrax)	Taurine supplementation (5–20 g/kg) in a 15% fishmeal, high-plant-protein diet	T20 (20 g/kg added taurine; analyzed 2.02%)	C− (15% fishmeal; no added taurine; analyzed taurine 0.11%)	Shelf life at 0 °C (days)	11 days; SD not reported	11 days; SD not reported	Not reported	Not reported	Not estimable	Not estimable	Not estimable	Shelf-life duration reported descriptively	Variance and independent experimental-unit sample size were not reported
Kotzamanis et al. (2020)	European seabass (Dicentrarchus labrax)	Taurine supplementation (5–20 g/kg) in a 15% fishmeal, high-plant-protein diet	T20 (20 g/kg added taurine; analyzed 2.02%)	C− (15% fishmeal; no added taurine; analyzed taurine 0.11%)	Shelf life at 5 °C (days)	5 days; SD not reported	6 days; SD not reported	Not reported	Not reported	Not estimable	Not estimable	Not estimable	Shelf-life duration reported descriptively	Variance and independent experimental-unit sample size were not reported
Kotzamanis et al. (2020)	European seabass (Dicentrarchus labrax)	Taurine supplementation (5–20 g/kg) in a 15% fishmeal, high-plant-protein diet	T10 (10 g/kg added taurine; analyzed 1.06%)	C− (15% fishmeal; no added taurine; analyzed taurine 0.11%)	TVBN time series at 0 °C	Figure-only time-series data	Figure-only time-series data	Unclear	Unclear	Not estimable	Not estimable	Not estimable	The original study reported no dietary effect on TVBN; drip loss was reduced at selected conditions	Exact time-specific numerical values and independent experimental-unit variance were not tabulated
Kotzamanis et al. (2020)	European seabass (Dicentrarchus labrax)	Taurine supplementation (5–20 g/kg) in a 15% fishmeal, high-plant-protein diet	T10 (10 g/kg added taurine; analyzed 1.06%)	C− (15% fishmeal; no added taurine; analyzed taurine 0.11%)	TVBN time series at 5 °C	Figure-only time-series data	Figure-only time-series data	Unclear	Unclear	Not estimable	Not estimable	Not estimable	The original study reported no dietary effect on TVBN; drip loss was reduced at selected conditions	Exact time-specific numerical values and independent experimental-unit variance were not tabulated
Kotzamanis et al. (2020)	European seabass (Dicentrarchus labrax)	Taurine supplementation (5–20 g/kg) in a 15% fishmeal, high-plant-protein diet	T10 (10 g/kg added taurine; analyzed 1.06%)	C− (15% fishmeal; no added taurine; analyzed taurine 0.11%)	Drip-loss time series at 0 °C	Figure-only time-series data	Figure-only time-series data	Unclear	Unclear	Not estimable	Not estimable	Not estimable	The original study reported no dietary effect on TVBN; drip loss was reduced at selected conditions	Exact time-specific numerical values and independent experimental-unit variance were not tabulated
Kotzamanis et al. (2020)	European seabass (Dicentrarchus labrax)	Taurine supplementation (5–20 g/kg) in a 15% fishmeal, high-plant-protein diet	T10 (10 g/kg added taurine; analyzed 1.06%)	C− (15% fishmeal; no added taurine; analyzed taurine 0.11%)	Drip-loss time series at 5 °C	Figure-only time-series data	Figure-only time-series data	Unclear	Unclear	Not estimable	Not estimable	Not estimable	The original study reported no dietary effect on TVBN; drip loss was reduced at selected conditions	Exact time-specific numerical values and independent experimental-unit variance were not tabulated
Kotzamanis et al. (2020)	European seabass (Dicentrarchus labrax)	Taurine supplementation (5–20 g/kg) in a 15% fishmeal, high-plant-protein diet	T20 (20 g/kg added taurine; analyzed 2.02%)	C− (15% fishmeal; no added taurine; analyzed taurine 0.11%)	TVBN time series at 0 °C	Figure-only time-series data	Figure-only time-series data	Unclear	Unclear	Not estimable	Not estimable	Not estimable	The original study reported no dietary effect on TVBN; drip loss was reduced at selected conditions	Exact time-specific numerical values and independent experimental-unit variance were not tabulated
Kotzamanis et al. (2020)	European seabass (Dicentrarchus labrax)	Taurine supplementation (5–20 g/kg) in a 15% fishmeal, high-plant-protein diet	T20 (20 g/kg added taurine; analyzed 2.02%)	C− (15% fishmeal; no added taurine; analyzed taurine 0.11%)	TVBN time series at 5 °C	Figure-only time-series data	Figure-only time-series data	Unclear	Unclear	Not estimable	Not estimable	Not estimable	The original study reported no dietary effect on TVBN; drip loss was reduced at selected conditions	Exact time-specific numerical values and independent experimental-unit variance were not tabulated
Kotzamanis et al. (2020)	European seabass (Dicentrarchus labrax)	Taurine supplementation (5–20 g/kg) in a 15% fishmeal, high-plant-protein diet	T20 (20 g/kg added taurine; analyzed 2.02%)	C− (15% fishmeal; no added taurine; analyzed taurine 0.11%)	Drip-loss time series at 0 °C	Figure-only time-series data	Figure-only time-series data	Unclear	Unclear	Not estimable	Not estimable	Not estimable	The original study reported no dietary effect on TVBN; drip loss was reduced at selected conditions	Exact time-specific numerical values and independent experimental-unit variance were not tabulated
Kotzamanis et al. (2020)	European seabass (Dicentrarchus labrax)	Taurine supplementation (5–20 g/kg) in a 15% fishmeal, high-plant-protein diet	T20 (20 g/kg added taurine; analyzed 2.02%)	C− (15% fishmeal; no added taurine; analyzed taurine 0.11%)	Drip-loss time series at 5 °C	Figure-only time-series data	Figure-only time-series data	Unclear	Unclear	Not estimable	Not estimable	Not estimable	The original study reported no dietary effect on TVBN; drip loss was reduced at selected conditions	Exact time-specific numerical values and independent experimental-unit variance were not tabulated
Kotzamanis et al. (2020)	European seabass (Dicentrarchus labrax)	Taurine supplementation (5–20 g/kg) in a 15% fishmeal, high-plant-protein diet	T5 (5 g/kg added taurine; analyzed 0.58%)	C− (15% fishmeal; no added taurine; analyzed taurine 0.11%)	Intestinal histological score	Categorical counts shown graphically	Categorical counts shown graphically	3	3	Not estimable	Not estimable	Not estimable	No clear treatment-related histological pattern was reported	Ordinal category counts were presented without a compatible continuous mean/SD or binary event structure
Kotzamanis et al. (2020)	European seabass (Dicentrarchus labrax)	Taurine supplementation (5–20 g/kg) in a 15% fishmeal, high-plant-protein diet	T5 (5 g/kg added taurine; analyzed 0.58%)	C− (15% fishmeal; no added taurine; analyzed taurine 0.11%)	Liver histological score	Categorical counts shown graphically	Categorical counts shown graphically	3	3	Not estimable	Not estimable	Not estimable	No clear treatment-related histological pattern was reported	Ordinal category counts were presented without a compatible continuous mean/SD or binary event structure
Kotzamanis et al. (2020)	European seabass (Dicentrarchus labrax)	Taurine supplementation (5–20 g/kg) in a 15% fishmeal, high-plant-protein diet	T10 (10 g/kg added taurine; analyzed 1.06%)	C− (15% fishmeal; no added taurine; analyzed taurine 0.11%)	Intestinal histological score	Categorical counts shown graphically	Categorical counts shown graphically	3	3	Not estimable	Not estimable	Not estimable	No clear treatment-related histological pattern was reported	Ordinal category counts were presented without a compatible continuous mean/SD or binary event structure
Kotzamanis et al. (2020)	European seabass (Dicentrarchus labrax)	Taurine supplementation (5–20 g/kg) in a 15% fishmeal, high-plant-protein diet	T10 (10 g/kg added taurine; analyzed 1.06%)	C− (15% fishmeal; no added taurine; analyzed taurine 0.11%)	Liver histological score	Categorical counts shown graphically	Categorical counts shown graphically	3	3	Not estimable	Not estimable	Not estimable	No clear treatment-related histological pattern was reported	Ordinal category counts were presented without a compatible continuous mean/SD or binary event structure
Kotzamanis et al. (2020)	European seabass (Dicentrarchus labrax)	Taurine supplementation (5–20 g/kg) in a 15% fishmeal, high-plant-protein diet	T20 (20 g/kg added taurine; analyzed 2.02%)	C− (15% fishmeal; no added taurine; analyzed taurine 0.11%)	Intestinal histological score	Categorical counts shown graphically	Categorical counts shown graphically	3	3	Not estimable	Not estimable	Not estimable	No clear treatment-related histological pattern was reported	Ordinal category counts were presented without a compatible continuous mean/SD or binary event structure
Kotzamanis et al. (2020)	European seabass (Dicentrarchus labrax)	Taurine supplementation (5–20 g/kg) in a 15% fishmeal, high-plant-protein diet	T20 (20 g/kg added taurine; analyzed 2.02%)	C− (15% fishmeal; no added taurine; analyzed taurine 0.11%)	Liver histological score	Categorical counts shown graphically	Categorical counts shown graphically	3	3	Not estimable	Not estimable	Not estimable	No clear treatment-related histological pattern was reported	Ordinal category counts were presented without a compatible continuous mean/SD or binary event structure

Notes. Continuous tank-level outcomes were summarized as Hedges' g with 95% confidence intervals. The replicate tank was the independent unit (n = 3 per treatment). Positive values indicate a higher intervention value; FCR and instrumental adhesiveness were sign-reversed. Sensory-panel, microbial-model, shelf-life, figure-only, and ordinal histology outcomes were retained descriptively when a valid tank-level effect estimate could not be calculated. C+, the 30% fishmeal positive control, was not used as the primary comparator.

Study 4. Watson et al. (2013): fishmeal-free plant-protein diets containing alternative lipid sources and a constant taurine level

Primary comparisons: MSC versus ARS control and CO+EFA versus ARS control. Yellow rows indicate outcomes for which a valid tank-level effect estimate was not possible.

Study	Species	Strategy	Intervention	Comparator	Outcome and unit	Intervention mean ± SD	Comparator mean ± SD	n intervention	n comparator	Effect-size metric	Effect size	95% CI	Interpretation	Reason not estimated
Watson et al. (2013)	Gilthead seabream (Sparus aurata)	Alternative lipid sources in taurine-supplemented, fishmeal-free plant-protein diets	MSC (thraustochytrid meal + soybean oil; 15 g/kg taurine)	ARS control (fish oil; 15 g/kg taurine; fishmeal-free)	Weight gain (%)	785.69 ± 3.84	696.33 ± 0.05	2	2	Hedges' g (LS variance)	18.57	5.55 to 31.58	Higher growth outcome with the alternative lipid source; 95% CI excluded 0	Not applicable—effect size estimated
Watson et al. (2013)	Gilthead seabream (Sparus aurata)	Alternative lipid sources in taurine-supplemented, fishmeal-free plant-protein diets	MSC (thraustochytrid meal + soybean oil; 15 g/kg taurine)	ARS control (fish oil; 15 g/kg taurine; fishmeal-free)	Fillet yield (%)	27.28 ± 1.30	27.66 ± 1.09	2 tanks; analysis-level n unclear	2 tanks; analysis-level n unclear	Not estimable	Not estimable	Not estimable	Outcome retained descriptively	The outcome was measured on sampled fish, but independent tank-level means and variance were not reported
Watson et al. (2013)	Gilthead seabream (Sparus aurata)	Alternative lipid sources in taurine-supplemented, fishmeal-free plant-protein diets	MSC (thraustochytrid meal + soybean oil; 15 g/kg taurine)	ARS control (fish oil; 15 g/kg taurine; fishmeal-free)	FCR	1.27 ± 0.01	1.36 ± 0.01	2	2	Hedges' g (direction reversed; LS variance)	5.08	1.05 to 9.11	Lower, more favorable FCR with the alternative lipid source; 95% CI excluded 0	Not applicable—effect size estimated
Watson et al. (2013)	Gilthead seabream (Sparus aurata)	Alternative lipid sources in taurine-supplemented, fishmeal-free plant-protein diets	MSC (thraustochytrid meal + soybean oil; 15 g/kg taurine)	ARS control (fish oil; 15 g/kg taurine; fishmeal-free)	PER	1.61 ± 0.04	1.64 ± 0.01	2	2	Hedges' g (LS variance)	-0.58	-2.58 to 1.42	Lower PER with the alternative lipid source; 95% CI included 0 (very imprecise estimate)	Not applicable—effect size estimated
Watson et al. (2013)	Gilthead seabream (Sparus aurata)	Alternative lipid sources in taurine-supplemented, fishmeal-free plant-protein diets	MSC (thraustochytrid meal + soybean oil; 15 g/kg taurine)	ARS control (fish oil; 15 g/kg taurine; fishmeal-free)	Hepatosomatic index (%)	1.41 ± 0.18	2.11 ± 0.50	2 tanks; analysis-level n unclear	2 tanks; analysis-level n unclear	Not estimable	Not estimable	Not estimable	Outcome retained descriptively	The outcome was measured on sampled fish, but independent tank-level means and variance were not reported
Watson et al. (2013)	Gilthead seabream (Sparus aurata)	Alternative lipid sources in taurine-supplemented, fishmeal-free plant-protein diets	MSC (thraustochytrid meal + soybean oil; 15 g/kg taurine)	ARS control (fish oil; 15 g/kg taurine; fishmeal-free)	Specific growth rate (%/day)	2.48 ± 0.00	2.36 ± 0.00	2 tanks; analysis-level n unclear	2 tanks; analysis-level n unclear	Not estimable	Not estimable	Not estimable	Outcome retained descriptively	Both treatment SDs were reported as 0.00, producing a zero pooled SD; the standardized effect cannot be calculated
Watson et al. (2013)	Gilthead seabream (Sparus aurata)	Alternative lipid sources in taurine-supplemented, fishmeal-free plant-protein diets	MSC (thraustochytrid meal + soybean oil; 15 g/kg taurine)	ARS control (fish oil; 15 g/kg taurine; fishmeal-free)	Condition factor	1.56 ± 0.15	1.68 ± 0.13	2 tanks; analysis-level n unclear	2 tanks; analysis-level n unclear	Not estimable	Not estimable	Not estimable	Outcome retained descriptively	The outcome was measured on sampled fish, but independent tank-level means and variance were not reported
Watson et al. (2013)	Gilthead seabream (Sparus aurata)	Alternative lipid sources in taurine-supplemented, fishmeal-free plant-protein diets	MSC (thraustochytrid meal + soybean oil; 15 g/kg taurine)	ARS control (fish oil; 15 g/kg taurine; fishmeal-free)	Survival (%)	97.50; SD not reported	95.50; SD not reported	2 tanks; analysis-level n unclear	2 tanks; analysis-level n unclear	Not estimable	Not estimable	Not estimable	Outcome retained descriptively	Treatment percentages were reported without tank-level variance; cluster-adjusted event data were unavailable
Watson et al. (2013)	Gilthead seabream (Sparus aurata)	Alternative lipid sources in taurine-supplemented, fishmeal-free plant-protein diets	CO+EFA (canola oil + DHA + ARA; 15 g/kg taurine)	ARS control (fish oil; 15 g/kg taurine; fishmeal-free)	Weight gain (%)	680.34 ± 72.01	696.33 ± 0.05	2	2	Hedges' g (LS variance)	-0.18	-2.14 to 1.79	Lower growth outcome with the alternative lipid source; 95% CI included 0 (very imprecise estimate)	Not applicable—effect size estimated
Watson et al. (2013)	Gilthead seabream (Sparus aurata)	Alternative lipid sources in taurine-supplemented, fishmeal-free plant-protein diets	CO+EFA (canola oil + DHA + ARA; 15 g/kg taurine)	ARS control (fish oil; 15 g/kg taurine; fishmeal-free)	Fillet yield (%)	27.50 ± 2.97	27.66 ± 1.09	2 tanks; analysis-level n unclear	2 tanks; analysis-level n unclear	Not estimable	Not estimable	Not estimable	Outcome retained descriptively	The outcome was measured on sampled fish, but independent tank-level means and variance were not reported
Watson et al. (2013)	Gilthead seabream (Sparus aurata)	Alternative lipid sources in taurine-supplemented, fishmeal-free plant-protein diets	CO+EFA (canola oil + DHA + ARA; 15 g/kg taurine)	ARS control (fish oil; 15 g/kg taurine; fishmeal-free)	FCR	1.37 ± 0.04	1.36 ± 0.01	2	2	Hedges' g (direction reversed; LS variance)	-0.19	-2.16 to 1.77	Higher, less favorable FCR with the alternative lipid source; 95% CI included 0 (very imprecise estimate)	Not applicable—effect size estimated
Watson et al. (2013)	Gilthead seabream (Sparus aurata)	Alternative lipid sources in taurine-supplemented, fishmeal-free plant-protein diets	CO+EFA (canola oil + DHA + ARA; 15 g/kg taurine)	ARS control (fish oil; 15 g/kg taurine; fishmeal-free)	PER	1.42 ± 0.04	1.64 ± 0.01	2	2	Hedges' g (LS variance)	-4.26	-7.80 to -0.72	Lower PER with the alternative lipid source; 95% CI excluded 0	Not applicable—effect size estimated
Watson et al. (2013)	Gilthead seabream (Sparus aurata)	Alternative lipid sources in taurine-supplemented, fishmeal-free plant-protein diets	CO+EFA (canola oil + DHA + ARA; 15 g/kg taurine)	ARS control (fish oil; 15 g/kg taurine; fishmeal-free)	Hepatosomatic index (%)	2.14 ± 0.39	2.11 ± 0.50	2 tanks; analysis-level n unclear	2 tanks; analysis-level n unclear	Not estimable	Not estimable	Not estimable	Outcome retained descriptively	The outcome was measured on sampled fish, but independent tank-level means and variance were not reported
Watson et al. (2013)	Gilthead seabream (Sparus aurata)	Alternative lipid sources in taurine-supplemented, fishmeal-free plant-protein diets	CO+EFA (canola oil + DHA + ARA; 15 g/kg taurine)	ARS control (fish oil; 15 g/kg taurine; fishmeal-free)	Specific growth rate (%/day)	2.33 ± 0.11	2.36 ± 0.00	2	2	Hedges' g (LS variance)	-0.22	-2.18 to 1.75	Lower growth outcome with the alternative lipid source; at least one SD was reported as 0.00 and the estimate is sensitive to rounding; 95% CI included 0 (very imprecise estimate)	Not applicable—effect size estimated
Watson et al. (2013)	Gilthead seabream (Sparus aurata)	Alternative lipid sources in taurine-supplemented, fishmeal-free plant-protein diets	CO+EFA (canola oil + DHA + ARA; 15 g/kg taurine)	ARS control (fish oil; 15 g/kg taurine; fishmeal-free)	Condition factor	1.67 ± 0.13	1.68 ± 0.13	2 tanks; analysis-level n unclear	2 tanks; analysis-level n unclear	Not estimable	Not estimable	Not estimable	Outcome retained descriptively	The outcome was measured on sampled fish, but independent tank-level means and variance were not reported
Watson et al. (2013)	Gilthead seabream (Sparus aurata)	Alternative lipid sources in taurine-supplemented, fishmeal-free plant-protein diets	CO+EFA (canola oil + DHA + ARA; 15 g/kg taurine)	ARS control (fish oil; 15 g/kg taurine; fishmeal-free)	Survival (%)	94.50; SD not reported	95.50; SD not reported	2 tanks; analysis-level n unclear	2 tanks; analysis-level n unclear	Not estimable	Not estimable	Not estimable	Outcome retained descriptively	Treatment percentages were reported without tank-level variance; cluster-adjusted event data were unavailable
Watson et al. (2013)	Gilthead seabream (Sparus aurata)	Alternative lipid sources in taurine-supplemented, fishmeal-free plant-protein diets	MSC (thraustochytrid meal + soybean oil; 15 g/kg taurine)	ARS control (fish oil; 15 g/kg taurine; fishmeal-free)	Whole-body lipid (g/100 g dry matter)	26.94 ± 5.55	29.90 ± 6.65	6 fish nested in 2 tanks	6 fish nested in 2 tanks	Not estimable	Not estimable	Not estimable	Whole-body outcome retained descriptively	Six fish per diet were sampled from two tanks, but tank-level means and variance were not reported; using fish-level SD would risk pseudoreplication
Watson et al. (2013)	Gilthead seabream (Sparus aurata)	Alternative lipid sources in taurine-supplemented, fishmeal-free plant-protein diets	MSC (thraustochytrid meal + soybean oil; 15 g/kg taurine)	ARS control (fish oil; 15 g/kg taurine; fishmeal-free)	Whole-body ash (g/100 g dry matter)	10.14 ± 1.66	10.58 ± 1.22	6 fish nested in 2 tanks	6 fish nested in 2 tanks	Not estimable	Not estimable	Not estimable	Whole-body outcome retained descriptively	Six fish per diet were sampled from two tanks, but tank-level means and variance were not reported; using fish-level SD would risk pseudoreplication
Watson et al. (2013)	Gilthead seabream (Sparus aurata)	Alternative lipid sources in taurine-supplemented, fishmeal-free plant-protein diets	MSC (thraustochytrid meal + soybean oil; 15 g/kg taurine)	ARS control (fish oil; 15 g/kg taurine; fishmeal-free)	Whole-body protein (g/100 g dry matter)	52.64 ± 5.88	52.33 ± 4.52	6 fish nested in 2 tanks	6 fish nested in 2 tanks	Not estimable	Not estimable	Not estimable	Whole-body outcome retained descriptively	Six fish per diet were sampled from two tanks, but tank-level means and variance were not reported; using fish-level SD would risk pseudoreplication
Watson et al. (2013)	Gilthead seabream (Sparus aurata)	Alternative lipid sources in taurine-supplemented, fishmeal-free plant-protein diets	MSC (thraustochytrid meal + soybean oil; 15 g/kg taurine)	ARS control (fish oil; 15 g/kg taurine; fishmeal-free)	Whole-body carbohydrate/fiber (g/100 g dry matter)	10.28; SD not reported	7.19; SD not reported	6 fish nested in 2 tanks	6 fish nested in 2 tanks	Not estimable	Not estimable	Not estimable	Whole-body outcome retained descriptively	Variance was not reported, and tank-level summary data were unavailable
Watson et al. (2013)	Gilthead seabream (Sparus aurata)	Alternative lipid sources in taurine-supplemented, fishmeal-free plant-protein diets	MSC (thraustochytrid meal + soybean oil; 15 g/kg taurine)	ARS control (fish oil; 15 g/kg taurine; fishmeal-free)	Whole-body moisture (g/100 g dry matter)	62.88 ± 2.48	65.59 ± 1.05	6 fish nested in 2 tanks	6 fish nested in 2 tanks	Not estimable	Not estimable	Not estimable	Whole-body outcome retained descriptively	Six fish per diet were sampled from two tanks, but tank-level means and variance were not reported; using fish-level SD would risk pseudoreplication
Watson et al.	Gilthead seabream	Alternative lipid sources in	MSC (thraustochytrid meal +	ARS control (fish oil; 15 g/kg	Whole-body energy	23.02 ± 1.07	24.08 ± 0.84	6 fish	6 fish	Not estimable	Not	Not estimable	Whole-body outcome retained	Six fish per diet were sampled from two

Study	Species	Strategy	Intervention	Comparator	Outcome and unit	Intervention mean ± SD	Comparator mean ± SD	n intervention	n comparator	Effect-size metric	Effect size	95% CI	Interpretation	Reason not estimated
(2013)	(Sparus aurata)	taurine-supplemented, fishmeal-free plant-protein diets	soybean oil; 15 g/kg taurine)	taurine; fishmeal-free)	(MJ/kg)			nested in 2 tanks	nested in 2 tanks		estimable		descriptively	tanks, but tank-level means and variance were not reported; using fish-level SD would risk pseudoreplication
Watson et al. (2013)	Gilthead seabream (Sparus aurata)	Alternative lipid sources in taurine-supplemented, fishmeal-free plant-protein diets	MSC (thraustochytrid meal + soybean oil; 15 g/kg taurine)	ARS control (fish oil; 15 g/kg taurine; fishmeal-free)	Body weight of analyzed fish (g)	111.33 ± 8.76	92.78 ± 13.79	6 fish nested in 2 tanks	6 fish nested in 2 tanks	Not estimable	Not estimable	Not estimable	Whole-body outcome retained descriptively	Six fish per diet were sampled from two tanks, but tank-level means and variance were not reported; using fish-level SD would risk pseudoreplication
Watson et al. (2013)	Gilthead seabream (Sparus aurata)	Alternative lipid sources in taurine-supplemented, fishmeal-free plant-protein diets	MSC (thraustochytrid meal + soybean oil; 15 g/kg taurine)	ARS control (fish oil; 15 g/kg taurine; fishmeal-free)	Fillet lipid (g/100 g dry matter)	15.28 ± 4.54	14.46 ± 3.93	6 fish nested in 2 tanks	6 fish nested in 2 tanks	Not estimable	Not estimable	Not estimable	Fillet-composition outcome retained descriptively	Six fish per diet were sampled from two tanks, but tank-level means and variance were not reported; using fish-level SD would risk pseudoreplication
Watson et al. (2013)	Gilthead seabream (Sparus aurata)	Alternative lipid sources in taurine-supplemented, fishmeal-free plant-protein diets	MSC (thraustochytrid meal + soybean oil; 15 g/kg taurine)	ARS control (fish oil; 15 g/kg taurine; fishmeal-free)	Fillet ash (g/100 g dry matter)	4.23 ± 0.87	4.15 ± 1.15	6 fish nested in 2 tanks	6 fish nested in 2 tanks	Not estimable	Not estimable	Not estimable	Fillet-composition outcome retained descriptively	Six fish per diet were sampled from two tanks, but tank-level means and variance were not reported; using fish-level SD would risk pseudoreplication
Watson et al. (2013)	Gilthead seabream (Sparus aurata)	Alternative lipid sources in taurine-supplemented, fishmeal-free plant-protein diets	MSC (thraustochytrid meal + soybean oil; 15 g/kg taurine)	ARS control (fish oil; 15 g/kg taurine; fishmeal-free)	Fillet protein (g/100 g dry matter)	71.54 ± 5.43	78.02 ± 2.51	6 fish nested in 2 tanks	6 fish nested in 2 tanks	Not estimable	Not estimable	Not estimable	Fillet-composition outcome retained descriptively	Six fish per diet were sampled from two tanks, but tank-level means and variance were not reported; using fish-level SD would risk pseudoreplication
Watson et al. (2013)	Gilthead seabream (Sparus aurata)	Alternative lipid sources in taurine-supplemented, fishmeal-free plant-protein diets	MSC (thraustochytrid meal + soybean oil; 15 g/kg taurine)	ARS control (fish oil; 15 g/kg taurine; fishmeal-free)	Fillet carbohydrate/fiber (g/100 g dry matter)	8.95; SD not reported	3.37; SD not reported	6 fish nested in 2 tanks	6 fish nested in 2 tanks	Not estimable	Not estimable	Not estimable	Fillet-composition outcome retained descriptively	Variance was not reported, and tank-level summary data were unavailable
Watson et al. (2013)	Gilthead seabream (Sparus aurata)	Alternative lipid sources in taurine-supplemented, fishmeal-free plant-protein diets	MSC (thraustochytrid meal + soybean oil; 15 g/kg taurine)	ARS control (fish oil; 15 g/kg taurine; fishmeal-free)	Fillet moisture (g/100 g dry matter)	69.69 ± 2.92	71.06 ± 2.31	6 fish nested in 2 tanks	6 fish nested in 2 tanks	Not estimable	Not estimable	Not estimable	Fillet-composition outcome retained descriptively	Six fish per diet were sampled from two tanks, but tank-level means and variance were not reported; using fish-level SD would risk pseudoreplication
Watson et al. (2013)	Gilthead seabream (Sparus aurata)	Alternative lipid sources in taurine-supplemented, fishmeal-free plant-protein diets	MSC (thraustochytrid meal + soybean oil; 15 g/kg taurine)	ARS control (fish oil; 15 g/kg taurine; fishmeal-free)	Fillet energy (MJ/kg)	25.39 ± 0.80	24.70 ± 0.84	6 fish nested in 2 tanks	6 fish nested in 2 tanks	Not estimable	Not estimable	Not estimable	Fillet-composition outcome retained descriptively	Six fish per diet were sampled from two tanks, but tank-level means and variance were not reported; using fish-level SD would risk pseudoreplication
Watson et al. (2013)	Gilthead seabream (Sparus aurata)	Alternative lipid sources in taurine-supplemented, fishmeal-free plant-protein diets	MSC (thraustochytrid meal + soybean oil; 15 g/kg taurine)	ARS control (fish oil; 15 g/kg taurine; fishmeal-free)	Body weight of fillet-analysis fish (g)	118.72 ± 13.10	113.47 ± 11.26	6 fish nested in 2 tanks	6 fish nested in 2 tanks	Not estimable	Not estimable	Not estimable	Fillet-composition outcome retained descriptively	Six fish per diet were sampled from two tanks, but tank-level means and variance were not reported; using fish-level SD would risk pseudoreplication
Watson et al. (2013)	Gilthead seabream (Sparus aurata)	Alternative lipid sources in taurine-supplemented, fishmeal-free plant-protein diets	CO+EFA (canola oil + DHA + ARA; 15 g/kg taurine)	ARS control (fish oil; 15 g/kg taurine; fishmeal-free)	Whole-body lipid (g/100 g dry matter)	27.40 ± 4.48	29.90 ± 6.65	6 fish nested in 2 tanks	6 fish nested in 2 tanks	Not estimable	Not estimable	Not estimable	Whole-body outcome retained descriptively	Six fish per diet were sampled from two tanks, but tank-level means and variance were not reported; using fish-level SD would risk pseudoreplication
Watson et al. (2013)	Gilthead seabream (Sparus aurata)	Alternative lipid sources in taurine-supplemented, fishmeal-free plant-protein diets	CO+EFA (canola oil + DHA + ARA; 15 g/kg taurine)	ARS control (fish oil; 15 g/kg taurine; fishmeal-free)	Whole-body ash (g/100 g dry matter)	10.51 ± 1.29	10.58 ± 1.22	6 fish nested in 2 tanks	6 fish nested in 2 tanks	Not estimable	Not estimable	Not estimable	Whole-body outcome retained descriptively	Six fish per diet were sampled from two tanks, but tank-level means and variance were not reported; using fish-level SD would risk pseudoreplication
Watson et al. (2013)	Gilthead seabream (Sparus aurata)	Alternative lipid sources in taurine-supplemented, fishmeal-free plant-protein diets	CO+EFA (canola oil + DHA + ARA; 15 g/kg taurine)	ARS control (fish oil; 15 g/kg taurine; fishmeal-free)	Whole-body protein (g/100 g dry matter)	51.42 ± 5.77	52.33 ± 4.52	6 fish nested in 2 tanks	6 fish nested in 2 tanks	Not estimable	Not estimable	Not estimable	Whole-body outcome retained descriptively	Six fish per diet were sampled from two tanks, but tank-level means and variance were not reported; using fish-level SD would risk pseudoreplication
Watson et al. (2013)	Gilthead seabream (Sparus aurata)	Alternative lipid sources in taurine-supplemented, fishmeal-free plant-protein diets	CO+EFA (canola oil + DHA + ARA; 15 g/kg taurine)	ARS control (fish oil; 15 g/kg taurine; fishmeal-free)	Whole-body carbohydrate/fiber (g/100 g dry matter)	10.67; SD not reported	7.19; SD not reported	6 fish nested in 2 tanks	6 fish nested in 2 tanks	Not estimable	Not estimable	Not estimable	Whole-body outcome retained descriptively	Variance was not reported, and tank-level summary data were unavailable
Watson et al. (2013)	Gilthead seabream (Sparus aurata)	Alternative lipid sources in taurine-supplemented, fishmeal-free plant-protein diets	CO+EFA (canola oil + DHA + ARA; 15 g/kg taurine)	ARS control (fish oil; 15 g/kg taurine; fishmeal-free)	Whole-body moisture (g/100 g dry matter)	64.71 ± 0.81	65.59 ± 1.05	6 fish nested in 2 tanks	6 fish nested in 2 tanks	Not estimable	Not estimable	Not estimable	Whole-body outcome retained descriptively	Six fish per diet were sampled from two tanks, but tank-level means and variance were not reported; using fish-level SD would risk pseudoreplication
Watson et al. (2013)	Gilthead seabream (Sparus aurata)	Alternative lipid sources in taurine-supplemented, fishmeal-free plant-protein diets	CO+EFA (canola oil + DHA + ARA; 15 g/kg taurine)	ARS control (fish oil; 15 g/kg taurine; fishmeal-free)	Whole-body energy (MJ/kg)	24.11 ± 0.73	24.08 ± 0.84	6 fish nested in 2 tanks	6 fish nested in 2 tanks	Not estimable	Not estimable	Not estimable	Whole-body outcome retained descriptively	Six fish per diet were sampled from two tanks, but tank-level means and variance were not reported; using fish-level SD would risk pseudoreplication
Watson et al. (2013)	Gilthead seabream (Sparus aurata)	Alternative lipid sources in taurine-supplemented, fishmeal-free plant-protein diets	CO+EFA (canola oil + DHA + ARA; 15 g/kg taurine)	ARS control (fish oil; 15 g/kg taurine; fishmeal-free)	Body weight of analyzed fish (g)	94.75 ± 11.06	92.78 ± 13.79	6 fish nested in 2 tanks	6 fish nested in 2 tanks	Not estimable	Not estimable	Not estimable	Whole-body outcome retained descriptively	Six fish per diet were sampled from two tanks, but tank-level means and variance were not reported; using fish-level SD would risk pseudoreplication
Watson et al. (2013)	Gilthead seabream (Sparus aurata)	Alternative lipid sources in taurine-supplemented, fishmeal-free plant-protein diets	CO+EFA (canola oil + DHA + ARA; 15 g/kg taurine)	ARS control (fish oil; 15 g/kg taurine; fishmeal-free)	Fillet lipid (g/100 g dry matter)	16.33 ± 4.50	14.46 ± 3.93	6 fish nested in 2 tanks	6 fish nested in 2 tanks	Not estimable	Not estimable	Not estimable	Fillet-composition outcome retained descriptively	Six fish per diet were sampled from two tanks, but tank-level means and variance were not reported; using fish-level SD would risk pseudoreplication
Watson et al. (2013)	Gilthead seabream (Sparus aurata)	Alternative lipid sources in taurine-supplemented, fishmeal-free plant-protein diets	CO+EFA (canola oil + DHA + ARA; 15 g/kg taurine)	ARS control (fish oil; 15 g/kg taurine; fishmeal-free)	Fillet ash (g/100 g dry matter)	4.07 ± 0.77	4.15 ± 1.15	6 fish nested in 2 tanks	6 fish nested in 2 tanks	Not estimable	Not estimable	Not estimable	Fillet-composition outcome retained descriptively	Six fish per diet were sampled from two tanks, but tank-level means and variance were not reported; using fish-level SD would risk pseudoreplication
Watson et al. (2013)	Gilthead seabream (Sparus aurata)	Alternative lipid sources in taurine-supplemented, fishmeal-free plant-protein diets	CO+EFA (canola oil + DHA + ARA; 15 g/kg taurine)	ARS control (fish oil; 15 g/kg taurine; fishmeal-free)	Fillet protein (g/100 g dry matter)	73.65 ± 2.64	78.02 ± 2.51	6 fish nested in 2 tanks	6 fish nested in 2 tanks	Not estimable	Not estimable	Not estimable	Fillet-composition outcome retained descriptively	Six fish per diet were sampled from two tanks, but tank-level means and variance were not reported; using fish-level SD would risk pseudoreplication
Watson et al. (2013)	Gilthead seabream (Sparus aurata)	Alternative lipid sources in taurine-supplemented, fishmeal-free plant-protein diets	CO+EFA (canola oil + DHA + ARA; 15 g/kg taurine)	ARS control (fish oil; 15 g/kg taurine; fishmeal-free)	Fillet carbohydrate/fiber (g/100 g dry matter)	5.95; SD not reported	3.37; SD not reported	6 fish nested in 2 tanks	6 fish nested in 2 tanks	Not estimable	Not estimable	Not estimable	Fillet-composition outcome retained descriptively	Variance was not reported, and tank-level summary data were unavailable
Watson et al. (2013)	Gilthead seabream (Sparus aurata)	Alternative lipid sources in taurine-supplemented, fishmeal-free plant-protein diets	CO+EFA (canola oil + DHA + ARA; 15 g/kg taurine)	ARS control (fish oil; 15 g/kg taurine; fishmeal-free)	Fillet moisture (g/100 g dry matter)	71.61 ± 0.62	71.06 ± 2.31	6 fish nested in 2 tanks	6 fish nested in 2 tanks	Not estimable	Not estimable	Not estimable	Fillet-composition outcome retained descriptively	Six fish per diet were sampled from two tanks, but tank-level means and variance were not reported; using fish-level SD would risk pseudoreplication
Watson et al. (2013)	Gilthead seabream (Sparus aurata)	Alternative lipid sources in taurine-supplemented, fishmeal-free plant-protein diets	CO+EFA (canola oil + DHA + ARA; 15 g/kg taurine)	ARS control (fish oil; 15 g/kg taurine; fishmeal-free)	Fillet energy (MJ/kg)	24.64 ± 0.59	24.70 ± 0.84	6 fish nested in 2 tanks	6 fish nested in 2 tanks	Not estimable	Not estimable	Not estimable	Fillet-composition outcome retained descriptively	Six fish per diet were sampled from two tanks, but tank-level means and variance were not reported; using fish-level SD would risk pseudoreplication
Watson et al. (2013)	Gilthead seabream (Sparus aurata)	Alternative lipid sources in taurine-supplemented, fishmeal-free plant-protein	CO+EFA (canola oil + DHA + ARA; 15 g/kg taurine)	ARS control (fish oil; 15 g/kg taurine; fishmeal-free)	Body weight of fillet-analysis fish (g)	104.33 ± 12.52	113.47 ± 11.26	6 fish nested in 2 tanks	6 fish nested in 2 tanks	Not estimable	Not estimable	Not estimable	Fillet-composition outcome retained descriptively	Six fish per diet were sampled from two tanks, but tank-level means and variance were not reported; using fish-level SD

Study	Species	Strategy	Intervention	Comparator	Outcome and unit	Intervention mean ± SD	Comparator mean ± SD	n intervention	n comparator	Effect-size metric	Effect size	95% CI	Interpretation	Reason not estimated
		diets												would risk pseudoreplication
Watson et al. (2013)	Gilthead seabream (Sparus aurata)	Alternative lipid sources in taurine-supplemented, fishmeal-free plant-protein diets	MSC (thraustochytrid meal + soybean oil; 15 g/kg taurine)	ARS control (fish oil; 15 g/kg taurine; fishmeal-free)	Fillet fatty acid 12:0 (g/100 g total fatty acids)	0.10 ± 0.01	0.09 ± 0.01	6 fish nested in 2 tanks	6 fish nested in 2 tanks	Not estimable	Not estimable	Not estimable	Fillet fatty-acid profile retained descriptively	Tank-level means and variance were not reported; reported SD appears to reflect fish-level observations nested within two tanks
Watson et al. (2013)	Gilthead seabream (Sparus aurata)	Alternative lipid sources in taurine-supplemented, fishmeal-free plant-protein diets	MSC (thraustochytrid meal + soybean oil; 15 g/kg taurine)	ARS control (fish oil; 15 g/kg taurine; fishmeal-free)	Whole-body fatty acid 12:0 (g/100 g total fatty acids)	0.11 ± 0.03	0.09 ± 0.01	6 fish nested in 2 tanks	6 fish nested in 2 tanks	Not estimable	Not estimable	Not estimable	Whole-body fatty-acid profile retained descriptively	Tank-level means and variance were not reported; reported SD appears to reflect fish-level observations nested within two tanks
Watson et al. (2013)	Gilthead seabream (Sparus aurata)	Alternative lipid sources in taurine-supplemented, fishmeal-free plant-protein diets	MSC (thraustochytrid meal + soybean oil; 15 g/kg taurine)	ARS control (fish oil; 15 g/kg taurine; fishmeal-free)	Fillet fatty acid 14:0 (g/100 g total fatty acids)	5.48 ± 0.61	4.99 ± 0.23	6 fish nested in 2 tanks	6 fish nested in 2 tanks	Not estimable	Not estimable	Not estimable	Fillet fatty-acid profile retained descriptively	Tank-level means and variance were not reported; reported SD appears to reflect fish-level observations nested within two tanks
Watson et al. (2013)	Gilthead seabream (Sparus aurata)	Alternative lipid sources in taurine-supplemented, fishmeal-free plant-protein diets	MSC (thraustochytrid meal + soybean oil; 15 g/kg taurine)	ARS control (fish oil; 15 g/kg taurine; fishmeal-free)	Whole-body fatty acid 14:0 (g/100 g total fatty acids)	6.50 ± 1.14	5.36 ± 0.59	6 fish nested in 2 tanks	6 fish nested in 2 tanks	Not estimable	Not estimable	Not estimable	Whole-body fatty-acid profile retained descriptively	Tank-level means and variance were not reported; reported SD appears to reflect fish-level observations nested within two tanks
Watson et al. (2013)	Gilthead seabream (Sparus aurata)	Alternative lipid sources in taurine-supplemented, fishmeal-free plant-protein diets	MSC (thraustochytrid meal + soybean oil; 15 g/kg taurine)	ARS control (fish oil; 15 g/kg taurine; fishmeal-free)	Fillet fatty acid 16:0 (g/100 g total fatty acids)	24.93 ± 2.01	26.08 ± 1.16	6 fish nested in 2 tanks	6 fish nested in 2 tanks	Not estimable	Not estimable	Not estimable	Fillet fatty-acid profile retained descriptively	Tank-level means and variance were not reported; reported SD appears to reflect fish-level observations nested within two tanks
Watson et al. (2013)	Gilthead seabream (Sparus aurata)	Alternative lipid sources in taurine-supplemented, fishmeal-free plant-protein diets	MSC (thraustochytrid meal + soybean oil; 15 g/kg taurine)	ARS control (fish oil; 15 g/kg taurine; fishmeal-free)	Whole-body fatty acid 16:0 (g/100 g total fatty acids)	29.60 ± 4.21	27.82 ± 3.13	6 fish nested in 2 tanks	6 fish nested in 2 tanks	Not estimable	Not estimable	Not estimable	Whole-body fatty-acid profile retained descriptively	Tank-level means and variance were not reported; reported SD appears to reflect fish-level observations nested within two tanks
Watson et al. (2013)	Gilthead seabream (Sparus aurata)	Alternative lipid sources in taurine-supplemented, fishmeal-free plant-protein diets	MSC (thraustochytrid meal + soybean oil; 15 g/kg taurine)	ARS control (fish oil; 15 g/kg taurine; fishmeal-free)	Fillet fatty acid 17:0 (g/100 g total fatty acids)	0.23 ± 0.02	0.25 ± 0.01	6 fish nested in 2 tanks	6 fish nested in 2 tanks	Not estimable	Not estimable	Not estimable	Fillet fatty-acid profile retained descriptively	Tank-level means and variance were not reported; reported SD appears to reflect fish-level observations nested within two tanks
Watson et al. (2013)	Gilthead seabream (Sparus aurata)	Alternative lipid sources in taurine-supplemented, fishmeal-free plant-protein diets	MSC (thraustochytrid meal + soybean oil; 15 g/kg taurine)	ARS control (fish oil; 15 g/kg taurine; fishmeal-free)	Whole-body fatty acid 17:0 (g/100 g total fatty acids)	0.27 ± 0.03	0.29 ± 0.03	6 fish nested in 2 tanks	6 fish nested in 2 tanks	Not estimable	Not estimable	Not estimable	Whole-body fatty-acid profile retained descriptively	Tank-level means and variance were not reported; reported SD appears to reflect fish-level observations nested within two tanks
Watson et al. (2013)	Gilthead seabream (Sparus aurata)	Alternative lipid sources in taurine-supplemented, fishmeal-free plant-protein diets	MSC (thraustochytrid meal + soybean oil; 15 g/kg taurine)	ARS control (fish oil; 15 g/kg taurine; fishmeal-free)	Fillet fatty acid 18:0 (g/100 g total fatty acids)	4.08 ± 0.31	4.57 ± 0.29	6 fish nested in 2 tanks	6 fish nested in 2 tanks	Not estimable	Not estimable	Not estimable	Fillet fatty-acid profile retained descriptively	Tank-level means and variance were not reported; reported SD appears to reflect fish-level observations nested within two tanks
Watson et al. (2013)	Gilthead seabream (Sparus aurata)	Alternative lipid sources in taurine-supplemented, fishmeal-free plant-protein diets	MSC (thraustochytrid meal + soybean oil; 15 g/kg taurine)	ARS control (fish oil; 15 g/kg taurine; fishmeal-free)	Whole-body fatty acid 18:0 (g/100 g total fatty acids)	5.15 ± 0.70	5.17 ± 0.51	6 fish nested in 2 tanks	6 fish nested in 2 tanks	Not estimable	Not estimable	Not estimable	Whole-body fatty-acid profile retained descriptively	Tank-level means and variance were not reported; reported SD appears to reflect fish-level observations nested within two tanks
Watson et al. (2013)	Gilthead seabream (Sparus aurata)	Alternative lipid sources in taurine-supplemented, fishmeal-free plant-protein diets	MSC (thraustochytrid meal + soybean oil; 15 g/kg taurine)	ARS control (fish oil; 15 g/kg taurine; fishmeal-free)	Fillet fatty acid 20:0 (g/100 g total fatty acids)	0.22 ± 0.04	0.24 ± 0.05	6 fish nested in 2 tanks	6 fish nested in 2 tanks	Not estimable	Not estimable	Not estimable	Fillet fatty-acid profile retained descriptively	Tank-level means and variance were not reported; reported SD appears to reflect fish-level observations nested within two tanks
Watson et al. (2013)	Gilthead seabream (Sparus aurata)	Alternative lipid sources in taurine-supplemented, fishmeal-free plant-protein diets	MSC (thraustochytrid meal + soybean oil; 15 g/kg taurine)	ARS control (fish oil; 15 g/kg taurine; fishmeal-free)	Whole-body fatty acid 20:0 (g/100 g total fatty acids)	0.26 ± 0.04	0.27 ± 0.04	6 fish nested in 2 tanks	6 fish nested in 2 tanks	Not estimable	Not estimable	Not estimable	Whole-body fatty-acid profile retained descriptively	Tank-level means and variance were not reported; reported SD appears to reflect fish-level observations nested within two tanks
Watson et al. (2013)	Gilthead seabream (Sparus aurata)	Alternative lipid sources in taurine-supplemented, fishmeal-free plant-protein diets	MSC (thraustochytrid meal + soybean oil; 15 g/kg taurine)	ARS control (fish oil; 15 g/kg taurine; fishmeal-free)	Fillet fatty acid 22:0 (g/100 g total fatty acids)	0.27 ± 0.08	0.40 ± 0.07	6 fish nested in 2 tanks	6 fish nested in 2 tanks	Not estimable	Not estimable	Not estimable	Fillet fatty-acid profile retained descriptively	Tank-level means and variance were not reported; reported SD appears to reflect fish-level observations nested within two tanks
Watson et al. (2013)	Gilthead seabream (Sparus aurata)	Alternative lipid sources in taurine-supplemented, fishmeal-free plant-protein diets	MSC (thraustochytrid meal + soybean oil; 15 g/kg taurine)	ARS control (fish oil; 15 g/kg taurine; fishmeal-free)	Whole-body fatty acid 22:0 (g/100 g total fatty acids)	0.24 ± 0.11	0.43 ± 0.05	6 fish nested in 2 tanks	6 fish nested in 2 tanks	Not estimable	Not estimable	Not estimable	Whole-body fatty-acid profile retained descriptively	Tank-level means and variance were not reported; reported SD appears to reflect fish-level observations nested within two tanks
Watson et al. (2013)	Gilthead seabream (Sparus aurata)	Alternative lipid sources in taurine-supplemented, fishmeal-free plant-protein diets	MSC (thraustochytrid meal + soybean oil; 15 g/kg taurine)	ARS control (fish oil; 15 g/kg taurine; fishmeal-free)	Fillet fatty acid SFA (g/100 g total fatty acids)	35.32 ± 2.88	36.61 ± 1.47	6 fish nested in 2 tanks	6 fish nested in 2 tanks	Not estimable	Not estimable	Not estimable	Fillet fatty-acid profile retained descriptively	Tank-level means and variance were not reported; reported SD appears to reflect fish-level observations nested within two tanks
Watson et al. (2013)	Gilthead seabream (Sparus aurata)	Alternative lipid sources in taurine-supplemented, fishmeal-free plant-protein diets	MSC (thraustochytrid meal + soybean oil; 15 g/kg taurine)	ARS control (fish oil; 15 g/kg taurine; fishmeal-free)	Whole-body fatty acid SFA (g/100 g total fatty acids)	41.99 ± 6.10	39.42 ± 4.24	6 fish nested in 2 tanks	6 fish nested in 2 tanks	Not estimable	Not estimable	Not estimable	Whole-body fatty-acid profile retained descriptively	Tank-level means and variance were not reported; reported SD appears to reflect fish-level observations nested within two tanks
Watson et al. (2013)	Gilthead seabream (Sparus aurata)	Alternative lipid sources in taurine-supplemented, fishmeal-free plant-protein diets	MSC (thraustochytrid meal + soybean oil; 15 g/kg taurine)	ARS control (fish oil; 15 g/kg taurine; fishmeal-free)	Fillet fatty acid 16:1n-7 (g/100 g total fatty acids)	6.25 ± 0.55	9.13 ± 0.41	6 fish nested in 2 tanks	6 fish nested in 2 tanks	Not estimable	Not estimable	Not estimable	Fillet fatty-acid profile retained descriptively	Tank-level means and variance were not reported; reported SD appears to reflect fish-level observations nested within two tanks
Watson et al. (2013)	Gilthead seabream (Sparus aurata)	Alternative lipid sources in taurine-supplemented, fishmeal-free plant-protein diets	MSC (thraustochytrid meal + soybean oil; 15 g/kg taurine)	ARS control (fish oil; 15 g/kg taurine; fishmeal-free)	Whole-body fatty acid 16:1n-7 (g/100 g total fatty acids)	6.37 ± 0.69	9.08 ± 0.57	6 fish nested in 2 tanks	6 fish nested in 2 tanks	Not estimable	Not estimable	Not estimable	Whole-body fatty-acid profile retained descriptively	Tank-level means and variance were not reported; reported SD appears to reflect fish-level observations nested within two tanks
Watson et al. (2013)	Gilthead seabream (Sparus aurata)	Alternative lipid sources in taurine-supplemented, fishmeal-free plant-protein diets	MSC (thraustochytrid meal + soybean oil; 15 g/kg taurine)	ARS control (fish oil; 15 g/kg taurine; fishmeal-free)	Fillet fatty acid 18:1n-7 (g/100 g total fatty acids)	2.17 ± 0.16	3.07 ± 0.11	6 fish nested in 2 tanks	6 fish nested in 2 tanks	Not estimable	Not estimable	Not estimable	Fillet fatty-acid profile retained descriptively	Tank-level means and variance were not reported; reported SD appears to reflect fish-level observations nested within two tanks
Watson et al. (2013)	Gilthead seabream (Sparus aurata)	Alternative lipid sources in taurine-supplemented, fishmeal-free plant-protein diets	MSC (thraustochytrid meal + soybean oil; 15 g/kg taurine)	ARS control (fish oil; 15 g/kg taurine; fishmeal-free)	Whole-body fatty acid 18:1n-7 (g/100 g total fatty acids)	2.46 ± 0.19	3.23 ± 0.16	6 fish nested in 2 tanks	6 fish nested in 2 tanks	Not estimable	Not estimable	Not estimable	Whole-body fatty-acid profile retained descriptively	Tank-level means and variance were not reported; reported SD appears to reflect fish-level observations nested within two tanks
Watson et al. (2013)	Gilthead seabream (Sparus aurata)	Alternative lipid sources in taurine-supplemented, fishmeal-free plant-protein diets	MSC (thraustochytrid meal + soybean oil; 15 g/kg taurine)	ARS control (fish oil; 15 g/kg taurine; fishmeal-free)	Fillet fatty acid 18:1n-9+6 (g/100 g total fatty acids)	22.39 ± 1.48	30.20 ± 0.69	6 fish nested in 2 tanks	6 fish nested in 2 tanks	Not estimable	Not estimable	Not estimable	Fillet fatty-acid profile retained descriptively	Tank-level means and variance were not reported; reported SD appears to reflect fish-level observations nested within two tanks
Watson et al. (2013)	Gilthead seabream (Sparus aurata)	Alternative lipid sources in taurine-supplemented, fishmeal-free plant-protein diets	MSC (thraustochytrid meal + soybean oil; 15 g/kg taurine)	ARS control (fish oil; 15 g/kg taurine; fishmeal-free)	Whole-body fatty acid 18:1n-9+6 (g/100 g total fatty acids)	23.65 ± 2.04	31.13 ± 1.87	6 fish nested in 2 tanks	6 fish nested in 2 tanks	Not estimable	Not estimable	Not estimable	Whole-body fatty-acid profile retained descriptively	Tank-level means and variance were not reported; reported SD appears to reflect fish-level observations nested within two tanks

Study	Species	Strategy	Intervention	Comparator	Outcome and unit	Intervention mean ± SD	Comparator mean ± SD	n intervention	n comparator	Effect-size metric	Effect size	95% CI	Interpretation	Reason not estimated
Watson et al. (2013)	Gilthead seabream (Sparus aurata)	Alternative lipid sources in taurine-supplemented, fishmeal-free plant-protein diets	MSC (thraustochytrid meal + soybean oil; 15 g/kg taurine)	ARS control (fish oil; 15 g/kg taurine; fishmeal-free)	Fillet fatty acid 20:1n-15+cis-8 (g/100 g total fatty acids)	0.11 ± 0.02	0.23 ± 0.06	6 fish nested in 2 tanks	6 fish nested in 2 tanks	Not estimable	Not estimable	Not estimable	Fillet fatty-acid profile retained descriptively	Tank-level means and variance were not reported; reported SD appears to reflect fish-level observations nested within two tanks
Watson et al. (2013)	Gilthead seabream (Sparus aurata)	Alternative lipid sources in taurine-supplemented, fishmeal-free plant-protein diets	MSC (thraustochytrid meal + soybean oil; 15 g/kg taurine)	ARS control (fish oil; 15 g/kg taurine; fishmeal-free)	Whole-body fatty acid 20:1n-15+cis-8 (g/100 g total fatty acids)	0.16 ± 0.03	0.26 ± 0.04	6 fish nested in 2 tanks	6 fish nested in 2 tanks	Not estimable	Not estimable	Not estimable	Whole-body fatty-acid profile retained descriptively	Tank-level means and variance were not reported; reported SD appears to reflect fish-level observations nested within two tanks
Watson et al. (2013)	Gilthead seabream (Sparus aurata)	Alternative lipid sources in taurine-supplemented, fishmeal-free plant-protein diets	MSC (thraustochytrid meal + soybean oil; 15 g/kg taurine)	ARS control (fish oil; 15 g/kg taurine; fishmeal-free)	Fillet fatty acid 20:1n-9 (g/100 g total fatty acids)	0.71 ± 0.08	0.81 ± 0.12	6 fish nested in 2 tanks	6 fish nested in 2 tanks	Not estimable	Not estimable	Not estimable	Fillet fatty-acid profile retained descriptively	Tank-level means and variance were not reported; reported SD appears to reflect fish-level observations nested within two tanks
Watson et al. (2013)	Gilthead seabream (Sparus aurata)	Alternative lipid sources in taurine-supplemented, fishmeal-free plant-protein diets	MSC (thraustochytrid meal + soybean oil; 15 g/kg taurine)	ARS control (fish oil; 15 g/kg taurine; fishmeal-free)	Whole-body fatty acid 20:1n-9 (g/100 g total fatty acids)	0.91 ± 0.08	0.91 ± 0.13	6 fish nested in 2 tanks	6 fish nested in 2 tanks	Not estimable	Not estimable	Not estimable	Whole-body fatty-acid profile retained descriptively	Tank-level means and variance were not reported; reported SD appears to reflect fish-level observations nested within two tanks
Watson et al. (2013)	Gilthead seabream (Sparus aurata)	Alternative lipid sources in taurine-supplemented, fishmeal-free plant-protein diets	MSC (thraustochytrid meal + soybean oil; 15 g/kg taurine)	ARS control (fish oil; 15 g/kg taurine; fishmeal-free)	Fillet fatty acid 24:1n-9 (g/100 g total fatty acids)	0.20 ± 0.04	0.29 ± 0.07	6 fish nested in 2 tanks	6 fish nested in 2 tanks	Not estimable	Not estimable	Not estimable	Fillet fatty-acid profile retained descriptively	Tank-level means and variance were not reported; reported SD appears to reflect fish-level observations nested within two tanks
Watson et al. (2013)	Gilthead seabream (Sparus aurata)	Alternative lipid sources in taurine-supplemented, fishmeal-free plant-protein diets	MSC (thraustochytrid meal + soybean oil; 15 g/kg taurine)	ARS control (fish oil; 15 g/kg taurine; fishmeal-free)	Whole-body fatty acid 24:1n-9 (g/100 g total fatty acids)	0.33 ± 0.08	0.37 ± 0.08	6 fish nested in 2 tanks	6 fish nested in 2 tanks	Not estimable	Not estimable	Not estimable	Whole-body fatty-acid profile retained descriptively	Tank-level means and variance were not reported; reported SD appears to reflect fish-level observations nested within two tanks
Watson et al. (2013)	Gilthead seabream (Sparus aurata)	Alternative lipid sources in taurine-supplemented, fishmeal-free plant-protein diets	MSC (thraustochytrid meal + soybean oil; 15 g/kg taurine)	ARS control (fish oil; 15 g/kg taurine; fishmeal-free)	Fillet fatty acid MUFA (g/100 g total fatty acids)	31.84 ± 2.09	43.72 ± 0.76	6 fish nested in 2 tanks	6 fish nested in 2 tanks	Not estimable	Not estimable	Not estimable	Fillet fatty-acid profile retained descriptively	Tank-level means and variance were not reported; reported SD appears to reflect fish-level observations nested within two tanks
Watson et al. (2013)	Gilthead seabream (Sparus aurata)	Alternative lipid sources in taurine-supplemented, fishmeal-free plant-protein diets	MSC (thraustochytrid meal + soybean oil; 15 g/kg taurine)	ARS control (fish oil; 15 g/kg taurine; fishmeal-free)	Whole-body fatty acid MUFA (g/100 g total fatty acids)	33.87 ± 3.02	44.98 ± 2.46	6 fish nested in 2 tanks	6 fish nested in 2 tanks	Not estimable	Not estimable	Not estimable	Whole-body fatty-acid profile retained descriptively	Tank-level means and variance were not reported; reported SD appears to reflect fish-level observations nested within two tanks
Watson et al. (2013)	Gilthead seabream (Sparus aurata)	Alternative lipid sources in taurine-supplemented, fishmeal-free plant-protein diets	MSC (thraustochytrid meal + soybean oil; 15 g/kg taurine)	ARS control (fish oil; 15 g/kg taurine; fishmeal-free)	Fillet fatty acid 16:3n-4 (g/100 g total fatty acids)	0.39 ± 0.04	0.53 ± 0.04	6 fish nested in 2 tanks	6 fish nested in 2 tanks	Not estimable	Not estimable	Not estimable	Fillet fatty-acid profile retained descriptively	Tank-level means and variance were not reported; reported SD appears to reflect fish-level observations nested within two tanks
Watson et al. (2013)	Gilthead seabream (Sparus aurata)	Alternative lipid sources in taurine-supplemented, fishmeal-free plant-protein diets	MSC (thraustochytrid meal + soybean oil; 15 g/kg taurine)	ARS control (fish oil; 15 g/kg taurine; fishmeal-free)	Whole-body fatty acid 16:3n-4 (g/100 g total fatty acids)	0.31 ± 0.08	0.49 ± 0.13	6 fish nested in 2 tanks	6 fish nested in 2 tanks	Not estimable	Not estimable	Not estimable	Whole-body fatty-acid profile retained descriptively	Tank-level means and variance were not reported; reported SD appears to reflect fish-level observations nested within two tanks
Watson et al. (2013)	Gilthead seabream (Sparus aurata)	Alternative lipid sources in taurine-supplemented, fishmeal-free plant-protein diets	MSC (thraustochytrid meal + soybean oil; 15 g/kg taurine)	ARS control (fish oil; 15 g/kg taurine; fishmeal-free)	Fillet fatty acid 16:4n-1 (g/100 g total fatty acids)	0.34 ± 0.04	0.38 ± 0.04	6 fish nested in 2 tanks	6 fish nested in 2 tanks	Not estimable	Not estimable	Not estimable	Fillet fatty-acid profile retained descriptively	Tank-level means and variance were not reported; reported SD appears to reflect fish-level observations nested within two tanks
Watson et al. (2013)	Gilthead seabream (Sparus aurata)	Alternative lipid sources in taurine-supplemented, fishmeal-free plant-protein diets	MSC (thraustochytrid meal + soybean oil; 15 g/kg taurine)	ARS control (fish oil; 15 g/kg taurine; fishmeal-free)	Whole-body fatty acid 16:4n-1 (g/100 g total fatty acids)	0.24 ± 0.05	0.42 ± 0.03	6 fish nested in 2 tanks	6 fish nested in 2 tanks	Not estimable	Not estimable	Not estimable	Whole-body fatty-acid profile retained descriptively	Tank-level means and variance were not reported; reported SD appears to reflect fish-level observations nested within two tanks
Watson et al. (2013)	Gilthead seabream (Sparus aurata)	Alternative lipid sources in taurine-supplemented, fishmeal-free plant-protein diets	MSC (thraustochytrid meal + soybean oil; 15 g/kg taurine)	ARS control (fish oil; 15 g/kg taurine; fishmeal-free)	Fillet fatty acid 18:2n-6 (g/100 g total fatty acids)	8.20 ± 0.60	6.97 ± 1.99	6 fish nested in 2 tanks	6 fish nested in 2 tanks	Not estimable	Not estimable	Not estimable	Fillet fatty-acid profile retained descriptively	Tank-level means and variance were not reported; reported SD appears to reflect fish-level observations nested within two tanks
Watson et al. (2013)	Gilthead seabream (Sparus aurata)	Alternative lipid sources in taurine-supplemented, fishmeal-free plant-protein diets	MSC (thraustochytrid meal + soybean oil; 15 g/kg taurine)	ARS control (fish oil; 15 g/kg taurine; fishmeal-free)	Whole-body fatty acid 18:2n-6 (g/100 g total fatty acids)	6.35 ± 3.40	6.40 ± 1.98	6 fish nested in 2 tanks	6 fish nested in 2 tanks	Not estimable	Not estimable	Not estimable	Whole-body fatty-acid profile retained descriptively	Tank-level means and variance were not reported; reported SD appears to reflect fish-level observations nested within two tanks
Watson et al. (2013)	Gilthead seabream (Sparus aurata)	Alternative lipid sources in taurine-supplemented, fishmeal-free plant-protein diets	MSC (thraustochytrid meal + soybean oil; 15 g/kg taurine)	ARS control (fish oil; 15 g/kg taurine; fishmeal-free)	Fillet fatty acid 18:3n-3 (g/100 g total fatty acids)	0.73 ± 0.10	0.69 ± 0.04	6 fish nested in 2 tanks	6 fish nested in 2 tanks	Not estimable	Not estimable	Not estimable	Fillet fatty-acid profile retained descriptively	Tank-level means and variance were not reported; reported SD appears to reflect fish-level observations nested within two tanks
Watson et al. (2013)	Gilthead seabream (Sparus aurata)	Alternative lipid sources in taurine-supplemented, fishmeal-free plant-protein diets	MSC (thraustochytrid meal + soybean oil; 15 g/kg taurine)	ARS control (fish oil; 15 g/kg taurine; fishmeal-free)	Whole-body fatty acid 18:3n-3 (g/100 g total fatty acids)	0.76 ± 0.12	0.55 ± 0.21	6 fish nested in 2 tanks	6 fish nested in 2 tanks	Not estimable	Not estimable	Not estimable	Whole-body fatty-acid profile retained descriptively	Tank-level means and variance were not reported; reported SD appears to reflect fish-level observations nested within two tanks
Watson et al. (2013)	Gilthead seabream (Sparus aurata)	Alternative lipid sources in taurine-supplemented, fishmeal-free plant-protein diets	MSC (thraustochytrid meal + soybean oil; 15 g/kg taurine)	ARS control (fish oil; 15 g/kg taurine; fishmeal-free)	Fillet fatty acid 20:2n-6 (g/100 g total fatty acids)	0.14 ± 0.03	0.30 ± 0.39	6 fish nested in 2 tanks	6 fish nested in 2 tanks	Not estimable	Not estimable	Not estimable	Fillet fatty-acid profile retained descriptively	Tank-level means and variance were not reported; reported SD appears to reflect fish-level observations nested within two tanks
Watson et al. (2013)	Gilthead seabream (Sparus aurata)	Alternative lipid sources in taurine-supplemented, fishmeal-free plant-protein diets	MSC (thraustochytrid meal + soybean oil; 15 g/kg taurine)	ARS control (fish oil; 15 g/kg taurine; fishmeal-free)	Whole-body fatty acid 20:2n-6 (g/100 g total fatty acids)	0.15 ± 0.04	0.15 ± 0.04	6 fish nested in 2 tanks	6 fish nested in 2 tanks	Not estimable	Not estimable	Not estimable	Whole-body fatty-acid profile retained descriptively	Tank-level means and variance were not reported; reported SD appears to reflect fish-level observations nested within two tanks
Watson et al. (2013)	Gilthead seabream (Sparus aurata)	Alternative lipid sources in taurine-supplemented, fishmeal-free plant-protein diets	MSC (thraustochytrid meal + soybean oil; 15 g/kg taurine)	ARS control (fish oil; 15 g/kg taurine; fishmeal-free)	Fillet fatty acid 20:4n-6 (g/100 g total fatty acids)	0.79 ± 0.12	0.39 ± 0.06	6 fish nested in 2 tanks	6 fish nested in 2 tanks	Not estimable	Not estimable	Not estimable	Fillet fatty-acid profile retained descriptively	Tank-level means and variance were not reported; reported SD appears to reflect fish-level observations nested within two tanks
Watson et al. (2013)	Gilthead seabream (Sparus aurata)	Alternative lipid sources in taurine-supplemented, fishmeal-free plant-protein diets	MSC (thraustochytrid meal + soybean oil; 15 g/kg taurine)	ARS control (fish oil; 15 g/kg taurine; fishmeal-free)	Whole-body fatty acid 20:4n-6 (g/100 g total fatty acids)	0.48 ± 0.38	0.32 ± 0.13	6 fish nested in 2 tanks	6 fish nested in 2 tanks	Not estimable	Not estimable	Not estimable	Whole-body fatty-acid profile retained descriptively	Tank-level means and variance were not reported; reported SD appears to reflect fish-level observations nested within two tanks
Watson et al. (2013)	Gilthead seabream (Sparus aurata)	Alternative lipid sources in taurine-supplemented, fishmeal-free plant-protein diets	MSC (thraustochytrid meal + soybean oil; 15 g/kg taurine)	ARS control (fish oil; 15 g/kg taurine; fishmeal-free)	Fillet fatty acid 20:5n-3 (g/100 g total fatty acids)	3.42 ± 1.09	4.22 ± 0.17	6 fish nested in 2 tanks	6 fish nested in 2 tanks	Not estimable	Not estimable	Not estimable	Fillet fatty-acid profile retained descriptively	Tank-level means and variance were not reported; reported SD appears to reflect fish-level observations nested within two tanks
Watson et al. (2013)	Gilthead seabream (Sparus aurata)	Alternative lipid sources in taurine-supplemented, fishmeal-free plant-protein diets	MSC (thraustochytrid meal + soybean oil; 15 g/kg taurine)	ARS control (fish oil; 15 g/kg taurine; fishmeal-free)	Whole-body fatty acid 20:5n-3 (g/100 g total fatty acids)	3.21 ± 1.02	3.04 ± 1.65	6 fish nested in 2 tanks	6 fish nested in 2 tanks	Not estimable	Not estimable	Not estimable	Whole-body fatty-acid profile retained descriptively	Tank-level means and variance were not reported; reported SD appears to reflect fish-level observations nested within two tanks
Watson et al. (2013)	Gilthead seabream (Sparus aurata)	Alternative lipid sources in taurine-supplemented,	MSC (thraustochytrid meal + soybean oil; 15 g/kg taurine)	ARS control (fish oil; 15 g/kg taurine; fishmeal-free)	Fillet fatty acid 22:5n-6 (g/100 g total fatty acids)	3.73 ± 0.59	0.33 ± 0.09	6 fish nested in 2	6 fish nested in 2	Not estimable	Not estimable	Not estimable	Fillet fatty-acid profile retained descriptively	Tank-level means and variance were not reported; reported SD appears to reflect

Study	Species	Strategy	Intervention	Comparator	Outcome and unit	Intervention mean ± SD	Comparator mean ± SD	n intervention	n comparator	Effect-size metric	Effect size	95% CI	Interpretation	Reason not estimated
		fishmeal-free plant-protein diets						tanks	tanks					fish-level observations nested within two tanks
Watson et al. (2013)	Gilthead seabream (Sparus aurata)	Alternative lipid sources in taurine-supplemented, fishmeal-free plant-protein diets	MSC (thraustochytrid meal + soybean oil; 15 g/kg taurine)	ARS control (fish oil; 15 g/kg taurine; fishmeal-free)	Whole-body fatty acid 22:5n-6 (g/100 g total fatty acids)	1.97 ± 1.66	0.29 ± 0.08	6 fish nested in 2 tanks	6 fish nested in 2 tanks	Not estimable	Not estimable	Not estimable	Whole-body fatty-acid profile retained descriptively	Tank-level means and variance were not reported; reported SD appears to reflect fish-level observations nested within two tanks
Watson et al. (2013)	Gilthead seabream (Sparus aurata)	Alternative lipid sources in taurine-supplemented, fishmeal-free plant-protein diets	MSC (thraustochytrid meal + soybean oil; 15 g/kg taurine)	ARS control (fish oil; 15 g/kg taurine; fishmeal-free)	Fillet fatty acid 22:5n-3 (g/100 g total fatty acids)	1.21 ± 0.17	1.14 ± 0.14	6 fish nested in 2 tanks	6 fish nested in 2 tanks	Not estimable	Not estimable	Not estimable	Fillet fatty-acid profile retained descriptively	Tank-level means and variance were not reported; reported SD appears to reflect fish-level observations nested within two tanks
Watson et al. (2013)	Gilthead seabream (Sparus aurata)	Alternative lipid sources in taurine-supplemented, fishmeal-free plant-protein diets	MSC (thraustochytrid meal + soybean oil; 15 g/kg taurine)	ARS control (fish oil; 15 g/kg taurine; fishmeal-free)	Whole-body fatty acid 22:5n-3 (g/100 g total fatty acids)	0.82 ± 0.65	1.04 ± 0.37	6 fish nested in 2 tanks	6 fish nested in 2 tanks	Not estimable	Not estimable	Not estimable	Whole-body fatty-acid profile retained descriptively	Tank-level means and variance were not reported; reported SD appears to reflect fish-level observations nested within two tanks
Watson et al. (2013)	Gilthead seabream (Sparus aurata)	Alternative lipid sources in taurine-supplemented, fishmeal-free plant-protein diets	MSC (thraustochytrid meal + soybean oil; 15 g/kg taurine)	ARS control (fish oil; 15 g/kg taurine; fishmeal-free)	Fillet fatty acid 22:6n-3 (g/100 g total fatty acids)	13.46 ± 2.37	4.23 ± 0.25	6 fish nested in 2 tanks	6 fish nested in 2 tanks	Not estimable	Not estimable	Not estimable	Fillet fatty-acid profile retained descriptively	Tank-level means and variance were not reported; reported SD appears to reflect fish-level observations nested within two tanks
Watson et al. (2013)	Gilthead seabream (Sparus aurata)	Alternative lipid sources in taurine-supplemented, fishmeal-free plant-protein diets	MSC (thraustochytrid meal + soybean oil; 15 g/kg taurine)	ARS control (fish oil; 15 g/kg taurine; fishmeal-free)	Whole-body fatty acid 22:6n-3 (g/100 g total fatty acids)	11.92 ± 2.81	2.90 ± 1.58	6 fish nested in 2 tanks	6 fish nested in 2 tanks	Not estimable	Not estimable	Not estimable	Whole-body fatty-acid profile retained descriptively	Tank-level means and variance were not reported; reported SD appears to reflect fish-level observations nested within two tanks
Watson et al. (2013)	Gilthead seabream (Sparus aurata)	Alternative lipid sources in taurine-supplemented, fishmeal-free plant-protein diets	MSC (thraustochytrid meal + soybean oil; 15 g/kg taurine)	ARS control (fish oil; 15 g/kg taurine; fishmeal-free)	Fillet fatty acid PUFA (g/100 g total fatty acids)	32.84 ± 4.38	19.67 ± 2.03	6 fish nested in 2 tanks	6 fish nested in 2 tanks	Not estimable	Not estimable	Not estimable	Fillet fatty-acid profile retained descriptively	Tank-level means and variance were not reported; reported SD appears to reflect fish-level observations nested within two tanks
Watson et al. (2013)	Gilthead seabream (Sparus aurata)	Alternative lipid sources in taurine-supplemented, fishmeal-free plant-protein diets	MSC (thraustochytrid meal + soybean oil; 15 g/kg taurine)	ARS control (fish oil; 15 g/kg taurine; fishmeal-free)	Whole-body fatty acid PUFA (g/100 g total fatty acids)	24.14 ± 9.01	15.60 ± 6.62	6 fish nested in 2 tanks	6 fish nested in 2 tanks	Not estimable	Not estimable	Not estimable	Whole-body fatty-acid profile retained descriptively	Tank-level means and variance were not reported; reported SD appears to reflect fish-level observations nested within two tanks
Watson et al. (2013)	Gilthead seabream (Sparus aurata)	Alternative lipid sources in taurine-supplemented, fishmeal-free plant-protein diets	CO+EFA (canola oil + DHA + ARA; 15 g/kg taurine)	ARS control (fish oil; 15 g/kg taurine; fishmeal-free)	Fillet fatty acid 12:0 (g/100 g total fatty acids)	0.11 ± 0.01	0.09 ± 0.01	6 fish nested in 2 tanks	6 fish nested in 2 tanks	Not estimable	Not estimable	Not estimable	Fillet fatty-acid profile retained descriptively	Tank-level means and variance were not reported; reported SD appears to reflect fish-level observations nested within two tanks
Watson et al. (2013)	Gilthead seabream (Sparus aurata)	Alternative lipid sources in taurine-supplemented, fishmeal-free plant-protein diets	CO+EFA (canola oil + DHA + ARA; 15 g/kg taurine)	ARS control (fish oil; 15 g/kg taurine; fishmeal-free)	Whole-body fatty acid 12:0 (g/100 g total fatty acids)	0.11 ± 0.01	0.09 ± 0.01	6 fish nested in 2 tanks	6 fish nested in 2 tanks	Not estimable	Not estimable	Not estimable	Whole-body fatty-acid profile retained descriptively	Tank-level means and variance were not reported; reported SD appears to reflect fish-level observations nested within two tanks
Watson et al. (2013)	Gilthead seabream (Sparus aurata)	Alternative lipid sources in taurine-supplemented, fishmeal-free plant-protein diets	CO+EFA (canola oil + DHA + ARA; 15 g/kg taurine)	ARS control (fish oil; 15 g/kg taurine; fishmeal-free)	Fillet fatty acid 14:0 (g/100 g total fatty acids)	2.37 ± 0.20	4.99 ± 0.23	6 fish nested in 2 tanks	6 fish nested in 2 tanks	Not estimable	Not estimable	Not estimable	Fillet fatty-acid profile retained descriptively	Tank-level means and variance were not reported; reported SD appears to reflect fish-level observations nested within two tanks
Watson et al. (2013)	Gilthead seabream (Sparus aurata)	Alternative lipid sources in taurine-supplemented, fishmeal-free plant-protein diets	CO+EFA (canola oil + DHA + ARA; 15 g/kg taurine)	ARS control (fish oil; 15 g/kg taurine; fishmeal-free)	Whole-body fatty acid 14:0 (g/100 g total fatty acids)	2.20 ± 0.19	5.36 ± 0.59	6 fish nested in 2 tanks	6 fish nested in 2 tanks	Not estimable	Not estimable	Not estimable	Whole-body fatty-acid profile retained descriptively	Tank-level means and variance were not reported; reported SD appears to reflect fish-level observations nested within two tanks
Watson et al. (2013)	Gilthead seabream (Sparus aurata)	Alternative lipid sources in taurine-supplemented, fishmeal-free plant-protein diets	CO+EFA (canola oil + DHA + ARA; 15 g/kg taurine)	ARS control (fish oil; 15 g/kg taurine; fishmeal-free)	Fillet fatty acid 16:0 (g/100 g total fatty acids)	17.12 ± 0.55	26.08 ± 1.16	6 fish nested in 2 tanks	6 fish nested in 2 tanks	Not estimable	Not estimable	Not estimable	Fillet fatty-acid profile retained descriptively	Tank-level means and variance were not reported; reported SD appears to reflect fish-level observations nested within two tanks
Watson et al. (2013)	Gilthead seabream (Sparus aurata)	Alternative lipid sources in taurine-supplemented, fishmeal-free plant-protein diets	CO+EFA (canola oil + DHA + ARA; 15 g/kg taurine)	ARS control (fish oil; 15 g/kg taurine; fishmeal-free)	Whole-body fatty acid 16:0 (g/100 g total fatty acids)	16.74 ± 1.78	27.82 ± 3.13	6 fish nested in 2 tanks	6 fish nested in 2 tanks	Not estimable	Not estimable	Not estimable	Whole-body fatty-acid profile retained descriptively	Tank-level means and variance were not reported; reported SD appears to reflect fish-level observations nested within two tanks
Watson et al. (2013)	Gilthead seabream (Sparus aurata)	Alternative lipid sources in taurine-supplemented, fishmeal-free plant-protein diets	CO+EFA (canola oil + DHA + ARA; 15 g/kg taurine)	ARS control (fish oil; 15 g/kg taurine; fishmeal-free)	Fillet fatty acid 17:0 (g/100 g total fatty acids)	0.11 ± 0.01	0.25 ± 0.01	6 fish nested in 2 tanks	6 fish nested in 2 tanks	Not estimable	Not estimable	Not estimable	Fillet fatty-acid profile retained descriptively	Tank-level means and variance were not reported; reported SD appears to reflect fish-level observations nested within two tanks
Watson et al. (2013)	Gilthead seabream (Sparus aurata)	Alternative lipid sources in taurine-supplemented, fishmeal-free plant-protein diets	CO+EFA (canola oil + DHA + ARA; 15 g/kg taurine)	ARS control (fish oil; 15 g/kg taurine; fishmeal-free)	Whole-body fatty acid 17:0 (g/100 g total fatty acids)	0.13 ± 0.02	0.29 ± 0.03	6 fish nested in 2 tanks	6 fish nested in 2 tanks	Not estimable	Not estimable	Not estimable	Whole-body fatty-acid profile retained descriptively	Tank-level means and variance were not reported; reported SD appears to reflect fish-level observations nested within two tanks
Watson et al. (2013)	Gilthead seabream (Sparus aurata)	Alternative lipid sources in taurine-supplemented, fishmeal-free plant-protein diets	CO+EFA (canola oil + DHA + ARA; 15 g/kg taurine)	ARS control (fish oil; 15 g/kg taurine; fishmeal-free)	Fillet fatty acid 18:0 (g/100 g total fatty acids)	3.10 ± 0.13	4.57 ± 0.29	6 fish nested in 2 tanks	6 fish nested in 2 tanks	Not estimable	Not estimable	Not estimable	Fillet fatty-acid profile retained descriptively	Tank-level means and variance were not reported; reported SD appears to reflect fish-level observations nested within two tanks
Watson et al. (2013)	Gilthead seabream (Sparus aurata)	Alternative lipid sources in taurine-supplemented, fishmeal-free plant-protein diets	CO+EFA (canola oil + DHA + ARA; 15 g/kg taurine)	ARS control (fish oil; 15 g/kg taurine; fishmeal-free)	Whole-body fatty acid 18:0 (g/100 g total fatty acids)	3.54 ± 0.43	5.17 ± 0.51	6 fish nested in 2 tanks	6 fish nested in 2 tanks	Not estimable	Not estimable	Not estimable	Whole-body fatty-acid profile retained descriptively	Tank-level means and variance were not reported; reported SD appears to reflect fish-level observations nested within two tanks
Watson et al. (2013)	Gilthead seabream (Sparus aurata)	Alternative lipid sources in taurine-supplemented, fishmeal-free plant-protein diets	CO+EFA (canola oil + DHA + ARA; 15 g/kg taurine)	ARS control (fish oil; 15 g/kg taurine; fishmeal-free)	Fillet fatty acid 20:0 (g/100 g total fatty acids)	0.27 ± 0.02	0.24 ± 0.05	6 fish nested in 2 tanks	6 fish nested in 2 tanks	Not estimable	Not estimable	Not estimable	Fillet fatty-acid profile retained descriptively	Tank-level means and variance were not reported; reported SD appears to reflect fish-level observations nested within two tanks
Watson et al. (2013)	Gilthead seabream (Sparus aurata)	Alternative lipid sources in taurine-supplemented, fishmeal-free plant-protein diets	CO+EFA (canola oil + DHA + ARA; 15 g/kg taurine)	ARS control (fish oil; 15 g/kg taurine; fishmeal-free)	Whole-body fatty acid 20:0 (g/100 g total fatty acids)	0.29 ± 0.05	0.27 ± 0.04	6 fish nested in 2 tanks	6 fish nested in 2 tanks	Not estimable	Not estimable	Not estimable	Whole-body fatty-acid profile retained descriptively	Tank-level means and variance were not reported; reported SD appears to reflect fish-level observations nested within two tanks
Watson et al. (2013)	Gilthead seabream (Sparus aurata)	Alternative lipid sources in taurine-supplemented, fishmeal-free plant-protein diets	CO+EFA (canola oil + DHA + ARA; 15 g/kg taurine)	ARS control (fish oil; 15 g/kg taurine; fishmeal-free)	Fillet fatty acid 22:0 (g/100 g total fatty acids)	0.20 ± 0.12	0.40 ± 0.07	6 fish nested in 2 tanks	6 fish nested in 2 tanks	Not estimable	Not estimable	Not estimable	Fillet fatty-acid profile retained descriptively	Tank-level means and variance were not reported; reported SD appears to reflect fish-level observations nested within two tanks
Watson et al. (2013)	Gilthead seabream (Sparus aurata)	Alternative lipid sources in taurine-supplemented, fishmeal-free plant-protein diets	CO+EFA (canola oil + DHA + ARA; 15 g/kg taurine)	ARS control (fish oil; 15 g/kg taurine; fishmeal-free)	Whole-body fatty acid 22:0 (g/100 g total fatty acids)	0.25 ± 0.26	0.43 ± 0.05	6 fish nested in 2 tanks	6 fish nested in 2 tanks	Not estimable	Not estimable	Not estimable	Whole-body fatty-acid profile retained descriptively	Tank-level means and variance were not reported; reported SD appears to reflect fish-level observations nested within two tanks
Watson et al. (2013)	Gilthead seabream (Sparus aurata)	Alternative lipid sources in taurine-supplemented, fishmeal-free plant-protein diets	CO+EFA (canola oil + DHA + ARA; 15 g/kg taurine)	ARS control (fish oil; 15 g/kg taurine; fishmeal-free)	Fillet fatty acid SFA (g/100 g total fatty acids)	23.18 ± 0.60	36.61 ± 1.47	6 fish nested in 2 tanks	6 fish nested in 2 tanks	Not estimable	Not estimable	Not estimable	Fillet fatty-acid profile retained descriptively	Tank-level means and variance were not reported; reported SD appears to reflect fish-level observations nested within two tanks

Study	Species	Strategy	Intervention	Comparator	Outcome and unit	Intervention mean ± SD	Comparator mean ± SD	n intervention	n comparator	Effect-size metric	Effect size	95% CI	Interpretation	Reason not estimated
Watson et al. (2013)	Gilthead seabream (Sparus aurata)	Alternative lipid sources in taurine-supplemented, fishmeal-free plant-protein diets	CO+EFA (canola oil + DHA + ARA; 15 g/kg taurine)	ARS control (fish oil; 15 g/kg taurine; fishmeal-free)	Whole-body fatty acid SFA (g/100 g total fatty acids)	23.10 ± 2.43	39.42 ± 4.24	6 fish nested in 2 tanks	6 fish nested in 2 tanks	Not estimable	Not estimable	Not estimable	Whole-body fatty-acid profile retained descriptively	Tank-level means and variance were not reported; reported SD appears to reflect fish-level observations nested within two tanks
Watson et al. (2013)	Gilthead seabream (Sparus aurata)	Alternative lipid sources in taurine-supplemented, fishmeal-free plant-protein diets	CO+EFA (canola oil + DHA + ARA; 15 g/kg taurine)	ARS control (fish oil; 15 g/kg taurine; fishmeal-free)	Fillet fatty acid 16:1n-7 (g/100 g total fatty acids)	3.71 ± 0.40	9.13 ± 0.41	6 fish nested in 2 tanks	6 fish nested in 2 tanks	Not estimable	Not estimable	Not estimable	Fillet fatty-acid profile retained descriptively	Tank-level means and variance were not reported; reported SD appears to reflect fish-level observations nested within two tanks
Watson et al. (2013)	Gilthead seabream (Sparus aurata)	Alternative lipid sources in taurine-supplemented, fishmeal-free plant-protein diets	CO+EFA (canola oil + DHA + ARA; 15 g/kg taurine)	ARS control (fish oil; 15 g/kg taurine; fishmeal-free)	Whole-body fatty acid 16:1n-7 (g/100 g total fatty acids)	3.55 ± 0.48	9.08 ± 0.57	6 fish nested in 2 tanks	6 fish nested in 2 tanks	Not estimable	Not estimable	Not estimable	Whole-body fatty-acid profile retained descriptively	Tank-level means and variance were not reported; reported SD appears to reflect fish-level observations nested within two tanks
Watson et al. (2013)	Gilthead seabream (Sparus aurata)	Alternative lipid sources in taurine-supplemented, fishmeal-free plant-protein diets	CO+EFA (canola oil + DHA + ARA; 15 g/kg taurine)	ARS control (fish oil; 15 g/kg taurine; fishmeal-free)	Fillet fatty acid 18:1n-7 (g/100 g total fatty acids)	2.43 ± 0.05	3.07 ± 0.11	6 fish nested in 2 tanks	6 fish nested in 2 tanks	Not estimable	Not estimable	Not estimable	Fillet fatty-acid profile retained descriptively	Tank-level means and variance were not reported; reported SD appears to reflect fish-level observations nested within two tanks
Watson et al. (2013)	Gilthead seabream (Sparus aurata)	Alternative lipid sources in taurine-supplemented, fishmeal-free plant-protein diets	CO+EFA (canola oil + DHA + ARA; 15 g/kg taurine)	ARS control (fish oil; 15 g/kg taurine; fishmeal-free)	Whole-body fatty acid 18:1n-7 (g/100 g total fatty acids)	2.58 ± 0.13	3.23 ± 0.16	6 fish nested in 2 tanks	6 fish nested in 2 tanks	Not estimable	Not estimable	Not estimable	Whole-body fatty-acid profile retained descriptively	Tank-level means and variance were not reported; reported SD appears to reflect fish-level observations nested within two tanks
Watson et al. (2013)	Gilthead seabream (Sparus aurata)	Alternative lipid sources in taurine-supplemented, fishmeal-free plant-protein diets	CO+EFA (canola oil + DHA + ARA; 15 g/kg taurine)	ARS control (fish oil; 15 g/kg taurine; fishmeal-free)	Fillet fatty acid 18:1n-9+6 (g/100 g total fatty acids)	43.53 ± 0.48	30.20 ± 0.69	6 fish nested in 2 tanks	6 fish nested in 2 tanks	Not estimable	Not estimable	Not estimable	Fillet fatty-acid profile retained descriptively	Tank-level means and variance were not reported; reported SD appears to reflect fish-level observations nested within two tanks
Watson et al. (2013)	Gilthead seabream (Sparus aurata)	Alternative lipid sources in taurine-supplemented, fishmeal-free plant-protein diets	CO+EFA (canola oil + DHA + ARA; 15 g/kg taurine)	ARS control (fish oil; 15 g/kg taurine; fishmeal-free)	Whole-body fatty acid 18:1n-9+6 (g/100 g total fatty acids)	45.97 ± 2.71	31.13 ± 1.87	6 fish nested in 2 tanks	6 fish nested in 2 tanks	Not estimable	Not estimable	Not estimable	Whole-body fatty-acid profile retained descriptively	Tank-level means and variance were not reported; reported SD appears to reflect fish-level observations nested within two tanks
Watson et al. (2013)	Gilthead seabream (Sparus aurata)	Alternative lipid sources in taurine-supplemented, fishmeal-free plant-protein diets	CO+EFA (canola oil + DHA + ARA; 15 g/kg taurine)	ARS control (fish oil; 15 g/kg taurine; fishmeal-free)	Fillet fatty acid 20:1n-15+cis-8 (g/100 g total fatty acids)	0.09 ± 0.03	0.23 ± 0.06	6 fish nested in 2 tanks	6 fish nested in 2 tanks	Not estimable	Not estimable	Not estimable	Fillet fatty-acid profile retained descriptively	Tank-level means and variance were not reported; reported SD appears to reflect fish-level observations nested within two tanks
Watson et al. (2013)	Gilthead seabream (Sparus aurata)	Alternative lipid sources in taurine-supplemented, fishmeal-free plant-protein diets	CO+EFA (canola oil + DHA + ARA; 15 g/kg taurine)	ARS control (fish oil; 15 g/kg taurine; fishmeal-free)	Whole-body fatty acid 20:1n-15+cis-8 (g/100 g total fatty acids)	0.13 ± 0.07	0.26 ± 0.04	6 fish nested in 2 tanks	6 fish nested in 2 tanks	Not estimable	Not estimable	Not estimable	Whole-body fatty-acid profile retained descriptively	Tank-level means and variance were not reported; reported SD appears to reflect fish-level observations nested within two tanks
Watson et al. (2013)	Gilthead seabream (Sparus aurata)	Alternative lipid sources in taurine-supplemented, fishmeal-free plant-protein diets	CO+EFA (canola oil + DHA + ARA; 15 g/kg taurine)	ARS control (fish oil; 15 g/kg taurine; fishmeal-free)	Fillet fatty acid 20:1n-9 (g/100 g total fatty acids)	0.72 ± 0.12	0.81 ± 0.12	6 fish nested in 2 tanks	6 fish nested in 2 tanks	Not estimable	Not estimable	Not estimable	Fillet fatty-acid profile retained descriptively	Tank-level means and variance were not reported; reported SD appears to reflect fish-level observations nested within two tanks
Watson et al. (2013)	Gilthead seabream (Sparus aurata)	Alternative lipid sources in taurine-supplemented, fishmeal-free plant-protein diets	CO+EFA (canola oil + DHA + ARA; 15 g/kg taurine)	ARS control (fish oil; 15 g/kg taurine; fishmeal-free)	Whole-body fatty acid 20:1n-9 (g/100 g total fatty acids)	0.84 ± 0.10	0.91 ± 0.13	6 fish nested in 2 tanks	6 fish nested in 2 tanks	Not estimable	Not estimable	Not estimable	Whole-body fatty-acid profile retained descriptively	Tank-level means and variance were not reported; reported SD appears to reflect fish-level observations nested within two tanks
Watson et al. (2013)	Gilthead seabream (Sparus aurata)	Alternative lipid sources in taurine-supplemented, fishmeal-free plant-protein diets	CO+EFA (canola oil + DHA + ARA; 15 g/kg taurine)	ARS control (fish oil; 15 g/kg taurine; fishmeal-free)	Fillet fatty acid 24:1n-9 (g/100 g total fatty acids)	0.20 ± 0.04	0.29 ± 0.07	6 fish nested in 2 tanks	6 fish nested in 2 tanks	Not estimable	Not estimable	Not estimable	Fillet fatty-acid profile retained descriptively	Tank-level means and variance were not reported; reported SD appears to reflect fish-level observations nested within two tanks
Watson et al. (2013)	Gilthead seabream (Sparus aurata)	Alternative lipid sources in taurine-supplemented, fishmeal-free plant-protein diets	CO+EFA (canola oil + DHA + ARA; 15 g/kg taurine)	ARS control (fish oil; 15 g/kg taurine; fishmeal-free)	Whole-body fatty acid 24:1n-9 (g/100 g total fatty acids)	0.26 ± 0.06	0.37 ± 0.08	6 fish nested in 2 tanks	6 fish nested in 2 tanks	Not estimable	Not estimable	Not estimable	Whole-body fatty-acid profile retained descriptively	Tank-level means and variance were not reported; reported SD appears to reflect fish-level observations nested within two tanks
Watson et al. (2013)	Gilthead seabream (Sparus aurata)	Alternative lipid sources in taurine-supplemented, fishmeal-free plant-protein diets	CO+EFA (canola oil + DHA + ARA; 15 g/kg taurine)	ARS control (fish oil; 15 g/kg taurine; fishmeal-free)	Fillet fatty acid MUFA (g/100 g total fatty acids)	50.67 ± 0.42	43.72 ± 0.76	6 fish nested in 2 tanks	6 fish nested in 2 tanks	Not estimable	Not estimable	Not estimable	Fillet fatty-acid profile retained descriptively	Tank-level means and variance were not reported; reported SD appears to reflect fish-level observations nested within two tanks
Watson et al. (2013)	Gilthead seabream (Sparus aurata)	Alternative lipid sources in taurine-supplemented, fishmeal-free plant-protein diets	CO+EFA (canola oil + DHA + ARA; 15 g/kg taurine)	ARS control (fish oil; 15 g/kg taurine; fishmeal-free)	Whole-body fatty acid MUFA (g/100 g total fatty acids)	53.34 ± 3.25	44.98 ± 2.46	6 fish nested in 2 tanks	6 fish nested in 2 tanks	Not estimable	Not estimable	Not estimable	Whole-body fatty-acid profile retained descriptively	Tank-level means and variance were not reported; reported SD appears to reflect fish-level observations nested within two tanks
Watson et al. (2013)	Gilthead seabream (Sparus aurata)	Alternative lipid sources in taurine-supplemented, fishmeal-free plant-protein diets	CO+EFA (canola oil + DHA + ARA; 15 g/kg taurine)	ARS control (fish oil; 15 g/kg taurine; fishmeal-free)	Fillet fatty acid 16:3n-4 (g/100 g total fatty acids)	0.16 ± 0.04	0.53 ± 0.04	6 fish nested in 2 tanks	6 fish nested in 2 tanks	Not estimable	Not estimable	Not estimable	Fillet fatty-acid profile retained descriptively	Tank-level means and variance were not reported; reported SD appears to reflect fish-level observations nested within two tanks
Watson et al. (2013)	Gilthead seabream (Sparus aurata)	Alternative lipid sources in taurine-supplemented, fishmeal-free plant-protein diets	CO+EFA (canola oil + DHA + ARA; 15 g/kg taurine)	ARS control (fish oil; 15 g/kg taurine; fishmeal-free)	Whole-body fatty acid 16:3n-4 (g/100 g total fatty acids)	0.25 ± 0.10	0.49 ± 0.13	6 fish nested in 2 tanks	6 fish nested in 2 tanks	Not estimable	Not estimable	Not estimable	Whole-body fatty-acid profile retained descriptively	Tank-level means and variance were not reported; reported SD appears to reflect fish-level observations nested within two tanks
Watson et al. (2013)	Gilthead seabream (Sparus aurata)	Alternative lipid sources in taurine-supplemented, fishmeal-free plant-protein diets	CO+EFA (canola oil + DHA + ARA; 15 g/kg taurine)	ARS control (fish oil; 15 g/kg taurine; fishmeal-free)	Fillet fatty acid 16:4n-1 (g/100 g total fatty acids)	0.12 ± 0.04	0.38 ± 0.04	6 fish nested in 2 tanks	6 fish nested in 2 tanks	Not estimable	Not estimable	Not estimable	Fillet fatty-acid profile retained descriptively	Tank-level means and variance were not reported; reported SD appears to reflect fish-level observations nested within two tanks
Watson et al. (2013)	Gilthead seabream (Sparus aurata)	Alternative lipid sources in taurine-supplemented, fishmeal-free plant-protein diets	CO+EFA (canola oil + DHA + ARA; 15 g/kg taurine)	ARS control (fish oil; 15 g/kg taurine; fishmeal-free)	Whole-body fatty acid 16:4n-1 (g/100 g total fatty acids)	0.14 ± 0.03	0.42 ± 0.03	6 fish nested in 2 tanks	6 fish nested in 2 tanks	Not estimable	Not estimable	Not estimable	Whole-body fatty-acid profile retained descriptively	Tank-level means and variance were not reported; reported SD appears to reflect fish-level observations nested within two tanks
Watson et al. (2013)	Gilthead seabream (Sparus aurata)	Alternative lipid sources in taurine-supplemented, fishmeal-free plant-protein diets	CO+EFA (canola oil + DHA + ARA; 15 g/kg taurine)	ARS control (fish oil; 15 g/kg taurine; fishmeal-free)	Fillet fatty acid 18:2n-6 (g/100 g total fatty acids)	17.12 ± 0.43	6.97 ± 1.99	6 fish nested in 2 tanks	6 fish nested in 2 tanks	Not estimable	Not estimable	Not estimable	Fillet fatty-acid profile retained descriptively	Tank-level means and variance were not reported; reported SD appears to reflect fish-level observations nested within two tanks
Watson et al. (2013)	Gilthead seabream (Sparus aurata)	Alternative lipid sources in taurine-supplemented, fishmeal-free plant-protein diets	CO+EFA (canola oil + DHA + ARA; 15 g/kg taurine)	ARS control (fish oil; 15 g/kg taurine; fishmeal-free)	Whole-body fatty acid 18:2n-6 (g/100 g total fatty acids)	15.59 ± 3.13	6.40 ± 1.98	6 fish nested in 2 tanks	6 fish nested in 2 tanks	Not estimable	Not estimable	Not estimable	Whole-body fatty-acid profile retained descriptively	Tank-level means and variance were not reported; reported SD appears to reflect fish-level observations nested within two tanks
Watson et al. (2013)	Gilthead seabream (Sparus aurata)	Alternative lipid sources in taurine-supplemented, fishmeal-free plant-protein diets	CO+EFA (canola oil + DHA + ARA; 15 g/kg taurine)	ARS control (fish oil; 15 g/kg taurine; fishmeal-free)	Fillet fatty acid 18:3n-3 (g/100 g total fatty acids)	3.93 ± 0.13	0.69 ± 0.04	6 fish nested in 2 tanks	6 fish nested in 2 tanks	Not estimable	Not estimable	Not estimable	Fillet fatty-acid profile retained descriptively	Tank-level means and variance were not reported; reported SD appears to reflect fish-level observations nested within two tanks
Watson et al. (2013)	Gilthead seabream (Sparus aurata)	Alternative lipid sources in taurine-supplemented,	CO+EFA (canola oil + DHA + ARA; 15 g/kg taurine)	ARS control (fish oil; 15 g/kg taurine; fishmeal-free)	Whole-body fatty acid 18:3n-3 (g/100 g total fatty	3.56 ± 1.01	0.55 ± 0.21	6 fish nested in 2	6 fish nested in 2	Not estimable	Not estimable	Not estimable	Whole-body fatty-acid profile retained descriptively	Tank-level means and variance were not reported; reported SD appears to reflect

Study	Species	Strategy	Intervention	Comparator	Outcome and unit	Intervention mean ± SD	Comparator mean ± SD	n intervention	n comparator	Effect-size metric	Effect size	95% CI	Interpretation	Reason not estimated
		fishmeal-free plant-protein diets			acids)			tanks	tanks					fish-level observations nested within two tanks
Watson et al. (2013)	Gilthead seabream (Sparus aurata)	Alternative lipid sources in taurine-supplemented, fishmeal-free plant-protein diets	CO+EFA (canola oil + DHA + ARA; 15 g/kg taurine)	ARS control (fish oil; 15 g/kg taurine; fishmeal-free)	Fillet fatty acid 20:2n-6 (g/100 g total fatty acids)	0.25 ± 0.06	0.30 ± 0.39	6 fish nested in 2 tanks	6 fish nested in 2 tanks	Not estimable	Not estimable	Not estimable	Fillet fatty-acid profile retained descriptively	Tank-level means and variance were not reported; reported SD appears to reflect fish-level observations nested within two tanks
Watson et al. (2013)	Gilthead seabream (Sparus aurata)	Alternative lipid sources in taurine-supplemented, fishmeal-free plant-protein diets	CO+EFA (canola oil + DHA + ARA; 15 g/kg taurine)	ARS control (fish oil; 15 g/kg taurine; fishmeal-free)	Whole-body fatty acid 20:2n-6 (g/100 g total fatty acids)	0.30 ± 0.07	0.15 ± 0.04	6 fish nested in 2 tanks	6 fish nested in 2 tanks	Not estimable	Not estimable	Not estimable	Whole-body fatty-acid profile retained descriptively	Tank-level means and variance were not reported; reported SD appears to reflect fish-level observations nested within two tanks
Watson et al. (2013)	Gilthead seabream (Sparus aurata)	Alternative lipid sources in taurine-supplemented, fishmeal-free plant-protein diets	CO+EFA (canola oil + DHA + ARA; 15 g/kg taurine)	ARS control (fish oil; 15 g/kg taurine; fishmeal-free)	Fillet fatty acid 20:4n-6 (g/100 g total fatty acids)	0.22 ± 0.03	0.39 ± 0.06	6 fish nested in 2 tanks	6 fish nested in 2 tanks	Not estimable	Not estimable	Not estimable	Fillet fatty-acid profile retained descriptively	Tank-level means and variance were not reported; reported SD appears to reflect fish-level observations nested within two tanks
Watson et al. (2013)	Gilthead seabream (Sparus aurata)	Alternative lipid sources in taurine-supplemented, fishmeal-free plant-protein diets	CO+EFA (canola oil + DHA + ARA; 15 g/kg taurine)	ARS control (fish oil; 15 g/kg taurine; fishmeal-free)	Whole-body fatty acid 20:4n-6 (g/100 g total fatty acids)	0.23 ± 0.09	0.32 ± 0.13	6 fish nested in 2 tanks	6 fish nested in 2 tanks	Not estimable	Not estimable	Not estimable	Whole-body fatty-acid profile retained descriptively	Tank-level means and variance were not reported; reported SD appears to reflect fish-level observations nested within two tanks
Watson et al. (2013)	Gilthead seabream (Sparus aurata)	Alternative lipid sources in taurine-supplemented, fishmeal-free plant-protein diets	CO+EFA (canola oil + DHA + ARA; 15 g/kg taurine)	ARS control (fish oil; 15 g/kg taurine; fishmeal-free)	Fillet fatty acid 20:5n-3 (g/100 g total fatty acids)	0.55 ± 0.16	4.22 ± 0.17	6 fish nested in 2 tanks	6 fish nested in 2 tanks	Not estimable	Not estimable	Not estimable	Fillet fatty-acid profile retained descriptively	Tank-level means and variance were not reported; reported SD appears to reflect fish-level observations nested within two tanks
Watson et al. (2013)	Gilthead seabream (Sparus aurata)	Alternative lipid sources in taurine-supplemented, fishmeal-free plant-protein diets	CO+EFA (canola oil + DHA + ARA; 15 g/kg taurine)	ARS control (fish oil; 15 g/kg taurine; fishmeal-free)	Whole-body fatty acid 20:5n-3 (g/100 g total fatty acids)	0.61 ± 0.35	3.04 ± 1.65	6 fish nested in 2 tanks	6 fish nested in 2 tanks	Not estimable	Not estimable	Not estimable	Whole-body fatty-acid profile retained descriptively	Tank-level means and variance were not reported; reported SD appears to reflect fish-level observations nested within two tanks
Watson et al. (2013)	Gilthead seabream (Sparus aurata)	Alternative lipid sources in taurine-supplemented, fishmeal-free plant-protein diets	CO+EFA (canola oil + DHA + ARA; 15 g/kg taurine)	ARS control (fish oil; 15 g/kg taurine; fishmeal-free)	Fillet fatty acid 22:5n-6 (g/100 g total fatty acids)	0.33 ± 0.12	0.33 ± 0.09	6 fish nested in 2 tanks	6 fish nested in 2 tanks	Not estimable	Not estimable	Not estimable	Fillet fatty-acid profile retained descriptively	Tank-level means and variance were not reported; reported SD appears to reflect fish-level observations nested within two tanks
Watson et al. (2013)	Gilthead seabream (Sparus aurata)	Alternative lipid sources in taurine-supplemented, fishmeal-free plant-protein diets	CO+EFA (canola oil + DHA + ARA; 15 g/kg taurine)	ARS control (fish oil; 15 g/kg taurine; fishmeal-free)	Whole-body fatty acid 22:5n-6 (g/100 g total fatty acids)	0.37 ± 0.09	0.29 ± 0.08	6 fish nested in 2 tanks	6 fish nested in 2 tanks	Not estimable	Not estimable	Not estimable	Whole-body fatty-acid profile retained descriptively	Tank-level means and variance were not reported; reported SD appears to reflect fish-level observations nested within two tanks
Watson et al. (2013)	Gilthead seabream (Sparus aurata)	Alternative lipid sources in taurine-supplemented, fishmeal-free plant-protein diets	CO+EFA (canola oil + DHA + ARA; 15 g/kg taurine)	ARS control (fish oil; 15 g/kg taurine; fishmeal-free)	Fillet fatty acid 22:5n-3 (g/100 g total fatty acids)	0.25 ± 0.04	1.14 ± 0.14	6 fish nested in 2 tanks	6 fish nested in 2 tanks	Not estimable	Not estimable	Not estimable	Fillet fatty-acid profile retained descriptively	Tank-level means and variance were not reported; reported SD appears to reflect fish-level observations nested within two tanks
Watson et al. (2013)	Gilthead seabream (Sparus aurata)	Alternative lipid sources in taurine-supplemented, fishmeal-free plant-protein diets	CO+EFA (canola oil + DHA + ARA; 15 g/kg taurine)	ARS control (fish oil; 15 g/kg taurine; fishmeal-free)	Whole-body fatty acid 22:5n-3 (g/100 g total fatty acids)	0.27 ± 0.10	1.04 ± 0.37	6 fish nested in 2 tanks	6 fish nested in 2 tanks	Not estimable	Not estimable	Not estimable	Whole-body fatty-acid profile retained descriptively	Tank-level means and variance were not reported; reported SD appears to reflect fish-level observations nested within two tanks
Watson et al. (2013)	Gilthead seabream (Sparus aurata)	Alternative lipid sources in taurine-supplemented, fishmeal-free plant-protein diets	CO+EFA (canola oil + DHA + ARA; 15 g/kg taurine)	ARS control (fish oil; 15 g/kg taurine; fishmeal-free)	Fillet fatty acid 22:6n-3 (g/100 g total fatty acids)	3.21 ± 0.41	4.23 ± 0.25	6 fish nested in 2 tanks	6 fish nested in 2 tanks	Not estimable	Not estimable	Not estimable	Fillet fatty-acid profile retained descriptively	Tank-level means and variance were not reported; reported SD appears to reflect fish-level observations nested within two tanks
Watson et al. (2013)	Gilthead seabream (Sparus aurata)	Alternative lipid sources in taurine-supplemented, fishmeal-free plant-protein diets	CO+EFA (canola oil + DHA + ARA; 15 g/kg taurine)	ARS control (fish oil; 15 g/kg taurine; fishmeal-free)	Whole-body fatty acid 22:6n-3 (g/100 g total fatty acids)	2.53 ± 0.98	2.90 ± 1.58	6 fish nested in 2 tanks	6 fish nested in 2 tanks	Not estimable	Not estimable	Not estimable	Whole-body fatty-acid profile retained descriptively	Tank-level means and variance were not reported; reported SD appears to reflect fish-level observations nested within two tanks
Watson et al. (2013)	Gilthead seabream (Sparus aurata)	Alternative lipid sources in taurine-supplemented, fishmeal-free plant-protein diets	CO+EFA (canola oil + DHA + ARA; 15 g/kg taurine)	ARS control (fish oil; 15 g/kg taurine; fishmeal-free)	Fillet fatty acid PUFA (g/100 g total fatty acids)	26.16 ± 0.74	19.67 ± 2.03	6 fish nested in 2 tanks	6 fish nested in 2 tanks	Not estimable	Not estimable	Not estimable	Fillet fatty-acid profile retained descriptively	Tank-level means and variance were not reported; reported SD appears to reflect fish-level observations nested within two tanks
Watson et al. (2013)	Gilthead seabream (Sparus aurata)	Alternative lipid sources in taurine-supplemented, fishmeal-free plant-protein diets	CO+EFA (canola oil + DHA + ARA; 15 g/kg taurine)	ARS control (fish oil; 15 g/kg taurine; fishmeal-free)	Whole-body fatty acid PUFA (g/100 g total fatty acids)	23.56 ± 5.55	15.60 ± 6.62	6 fish nested in 2 tanks	6 fish nested in 2 tanks	Not estimable	Not estimable	Not estimable	Whole-body fatty-acid profile retained descriptively	Tank-level means and variance were not reported; reported SD appears to reflect fish-level observations nested within two tanks
Watson et al. (2013)	Gilthead seabream (Sparus aurata)	Alternative lipid sources in taurine-supplemented, fishmeal-free plant-protein diets	MSC recovery after switch to ARS control	No concurrent ARS-control recovery group reported	Recovery-fillet fatty acid 12:0 (g/100 g total fatty acids)	0.08 ± 0.01	Not reported	6 fish nested in 2 tanks	Not available	Not estimable	Not estimable	Not estimable	Recovery-period fatty-acid value retained descriptively	No concurrent matched comparator was reported for the recovery period, and tank-level variance was unavailable
Watson et al. (2013)	Gilthead seabream (Sparus aurata)	Alternative lipid sources in taurine-supplemented, fishmeal-free plant-protein diets	MSC recovery after switch to ARS control	No concurrent ARS-control recovery group reported	Recovery-fillet fatty acid 14:0 (g/100 g total fatty acids)	4.18 ± 0.15	Not reported	6 fish nested in 2 tanks	Not available	Not estimable	Not estimable	Not estimable	Recovery-period fatty-acid value retained descriptively	No concurrent matched comparator was reported for the recovery period, and tank-level variance was unavailable
Watson et al. (2013)	Gilthead seabream (Sparus aurata)	Alternative lipid sources in taurine-supplemented, fishmeal-free plant-protein diets	MSC recovery after switch to ARS control	No concurrent ARS-control recovery group reported	Recovery-fillet fatty acid 16:0 (g/100 g total fatty acids)	22.05 ± 0.73	Not reported	6 fish nested in 2 tanks	Not available	Not estimable	Not estimable	Not estimable	Recovery-period fatty-acid value retained descriptively	No concurrent matched comparator was reported for the recovery period, and tank-level variance was unavailable
Watson et al. (2013)	Gilthead seabream (Sparus aurata)	Alternative lipid sources in taurine-supplemented, fishmeal-free plant-protein diets	MSC recovery after switch to ARS control	No concurrent ARS-control recovery group reported	Recovery-fillet fatty acid 17:0 (g/100 g total fatty acids)	0.26 ± 0.01	Not reported	6 fish nested in 2 tanks	Not available	Not estimable	Not estimable	Not estimable	Recovery-period fatty-acid value retained descriptively	No concurrent matched comparator was reported for the recovery period, and tank-level variance was unavailable
Watson et al. (2013)	Gilthead seabream (Sparus aurata)	Alternative lipid sources in taurine-supplemented, fishmeal-free plant-protein diets	MSC recovery after switch to ARS control	No concurrent ARS-control recovery group reported	Recovery-fillet fatty acid 18:0 (g/100 g total fatty acids)	3.87 ± 0.25	Not reported	6 fish nested in 2 tanks	Not available	Not estimable	Not estimable	Not estimable	Recovery-period fatty-acid value retained descriptively	No concurrent matched comparator was reported for the recovery period, and tank-level variance was unavailable
Watson et al. (2013)	Gilthead seabream (Sparus aurata)	Alternative lipid sources in taurine-supplemented, fishmeal-free plant-protein diets	MSC recovery after switch to ARS control	No concurrent ARS-control recovery group reported	Recovery-fillet fatty acid 20:0 (g/100 g total fatty acids)	0.22 ± 0.03	Not reported	6 fish nested in 2 tanks	Not available	Not estimable	Not estimable	Not estimable	Recovery-period fatty-acid value retained descriptively	No concurrent matched comparator was reported for the recovery period, and tank-level variance was unavailable
Watson et al. (2013)	Gilthead seabream (Sparus aurata)	Alternative lipid sources in taurine-supplemented, fishmeal-free plant-protein diets	MSC recovery after switch to ARS control	No concurrent ARS-control recovery group reported	Recovery-fillet fatty acid 22:0 (g/100 g total fatty acids)	0.33 ± 0.06	Not reported	6 fish nested in 2 tanks	Not available	Not estimable	Not estimable	Not estimable	Recovery-period fatty-acid value retained descriptively	No concurrent matched comparator was reported for the recovery period, and tank-level variance was unavailable
Watson et al. (2013)	Gilthead seabream (Sparus aurata)	Alternative lipid sources in taurine-supplemented, fishmeal-free plant-protein diets	MSC recovery after switch to ARS control	No concurrent ARS-control recovery group reported	Recovery-fillet fatty acid SFA (g/100 g total fatty acids)	30.98 ± 0.60	Not reported	6 fish nested in 2 tanks	Not available	Not estimable	Not estimable	Not estimable	Recovery-period fatty-acid value retained descriptively	No concurrent matched comparator was reported for the recovery period, and tank-level variance was unavailable

Study	Species	Strategy	Intervention	Comparator	Outcome and unit	Intervention mean ± SD	Comparator mean ± SD	n intervention	n comparator	Effect-size metric	Effect size	95% CI	Interpretation	Reason not estimated
Watson et al. (2013)	Gilthead seabream (Sparus aurata)	Alternative lipid sources in taurine-supplemented, fishmeal-free plant-protein diets	MSC recovery after switch to ARS control	No concurrent ARS-control recovery group reported	Recovery-fillet fatty acid 16:1n-7 (g/100 g total fatty acids)	6.77 ± 0.61	Not reported	6 fish nested in 2 tanks	Not available	Not estimable	Not estimable	Not estimable	Recovery-period fatty-acid value retained descriptively	No concurrent matched comparator was reported for the recovery period, and tank-level variance was unavailable
Watson et al. (2013)	Gilthead seabream (Sparus aurata)	Alternative lipid sources in taurine-supplemented, fishmeal-free plant-protein diets	MSC recovery after switch to ARS control	No concurrent ARS-control recovery group reported	Recovery-fillet fatty acid 18:1n-7 (g/100 g total fatty acids)	2.48 ± 0.07	Not reported	6 fish nested in 2 tanks	Not available	Not estimable	Not estimable	Not estimable	Recovery-period fatty-acid value retained descriptively	No concurrent matched comparator was reported for the recovery period, and tank-level variance was unavailable
Watson et al. (2013)	Gilthead seabream (Sparus aurata)	Alternative lipid sources in taurine-supplemented, fishmeal-free plant-protein diets	MSC recovery after switch to ARS control	No concurrent ARS-control recovery group reported	Recovery-fillet fatty acid 18:1n-9+6 (g/100 g total fatty acids)	23.66 ± 0.85	Not reported	6 fish nested in 2 tanks	Not available	Not estimable	Not estimable	Not estimable	Recovery-period fatty-acid value retained descriptively	No concurrent matched comparator was reported for the recovery period, and tank-level variance was unavailable
Watson et al. (2013)	Gilthead seabream (Sparus aurata)	Alternative lipid sources in taurine-supplemented, fishmeal-free plant-protein diets	MSC recovery after switch to ARS control	No concurrent ARS-control recovery group reported	Recovery-fillet fatty acid 20:1n-15+cis-8 (g/100 g total fatty acids)	0.15 ± 0.03	Not reported	6 fish nested in 2 tanks	Not available	Not estimable	Not estimable	Not estimable	Recovery-period fatty-acid value retained descriptively	No concurrent matched comparator was reported for the recovery period, and tank-level variance was unavailable
Watson et al. (2013)	Gilthead seabream (Sparus aurata)	Alternative lipid sources in taurine-supplemented, fishmeal-free plant-protein diets	MSC recovery after switch to ARS control	No concurrent ARS-control recovery group reported	Recovery-fillet fatty acid 20:1n-9 (g/100 g total fatty acids)	0.91 ± 0.06	Not reported	6 fish nested in 2 tanks	Not available	Not estimable	Not estimable	Not estimable	Recovery-period fatty-acid value retained descriptively	No concurrent matched comparator was reported for the recovery period, and tank-level variance was unavailable
Watson et al. (2013)	Gilthead seabream (Sparus aurata)	Alternative lipid sources in taurine-supplemented, fishmeal-free plant-protein diets	MSC recovery after switch to ARS control	No concurrent ARS-control recovery group reported	Recovery-fillet fatty acid 24:1n-9 (g/100 g total fatty acids)	0.28 ± 0.06	Not reported	6 fish nested in 2 tanks	Not available	Not estimable	Not estimable	Not estimable	Recovery-period fatty-acid value retained descriptively	No concurrent matched comparator was reported for the recovery period, and tank-level variance was unavailable
Watson et al. (2013)	Gilthead seabream (Sparus aurata)	Alternative lipid sources in taurine-supplemented, fishmeal-free plant-protein diets	MSC recovery after switch to ARS control	No concurrent ARS-control recovery group reported	Recovery-fillet fatty acid MUFA (g/100 g total fatty acids)	34.24 ± 1.39	Not reported	6 fish nested in 2 tanks	Not available	Not estimable	Not estimable	Not estimable	Recovery-period fatty-acid value retained descriptively	No concurrent matched comparator was reported for the recovery period, and tank-level variance was unavailable
Watson et al. (2013)	Gilthead seabream (Sparus aurata)	Alternative lipid sources in taurine-supplemented, fishmeal-free plant-protein diets	MSC recovery after switch to ARS control	No concurrent ARS-control recovery group reported	Recovery-fillet fatty acid 16:3n-4 (g/100 g total fatty acids)	0.34 ± 0.12	Not reported	6 fish nested in 2 tanks	Not available	Not estimable	Not estimable	Not estimable	Recovery-period fatty-acid value retained descriptively	No concurrent matched comparator was reported for the recovery period, and tank-level variance was unavailable
Watson et al. (2013)	Gilthead seabream (Sparus aurata)	Alternative lipid sources in taurine-supplemented, fishmeal-free plant-protein diets	MSC recovery after switch to ARS control	No concurrent ARS-control recovery group reported	Recovery-fillet fatty acid 16:4n-1 (g/100 g total fatty acids)	0.31 ± 0.01	Not reported	6 fish nested in 2 tanks	Not available	Not estimable	Not estimable	Not estimable	Recovery-period fatty-acid value retained descriptively	No concurrent matched comparator was reported for the recovery period, and tank-level variance was unavailable
Watson et al. (2013)	Gilthead seabream (Sparus aurata)	Alternative lipid sources in taurine-supplemented, fishmeal-free plant-protein diets	MSC recovery after switch to ARS control	No concurrent ARS-control recovery group reported	Recovery-fillet fatty acid 18:2n-6 (g/100 g total fatty acids)	10.16 ± 0.35	Not reported	6 fish nested in 2 tanks	Not available	Not estimable	Not estimable	Not estimable	Recovery-period fatty-acid value retained descriptively	No concurrent matched comparator was reported for the recovery period, and tank-level variance was unavailable
Watson et al. (2013)	Gilthead seabream (Sparus aurata)	Alternative lipid sources in taurine-supplemented, fishmeal-free plant-protein diets	MSC recovery after switch to ARS control	No concurrent ARS-control recovery group reported	Recovery-fillet fatty acid 18:3n-3 (g/100 g total fatty acids)	0.89 ± 0.04	Not reported	6 fish nested in 2 tanks	Not available	Not estimable	Not estimable	Not estimable	Recovery-period fatty-acid value retained descriptively	No concurrent matched comparator was reported for the recovery period, and tank-level variance was unavailable
Watson et al. (2013)	Gilthead seabream (Sparus aurata)	Alternative lipid sources in taurine-supplemented, fishmeal-free plant-protein diets	MSC recovery after switch to ARS control	No concurrent ARS-control recovery group reported	Recovery-fillet fatty acid 20:2n-6 (g/100 g total fatty acids)	0.24 ± 0.03	Not reported	6 fish nested in 2 tanks	Not available	Not estimable	Not estimable	Not estimable	Recovery-period fatty-acid value retained descriptively	No concurrent matched comparator was reported for the recovery period, and tank-level variance was unavailable
Watson et al. (2013)	Gilthead seabream (Sparus aurata)	Alternative lipid sources in taurine-supplemented, fishmeal-free plant-protein diets	MSC recovery after switch to ARS control	No concurrent ARS-control recovery group reported	Recovery-fillet fatty acid 20:4n-6 (g/100 g total fatty acids)	0.93 ± 0.05	Not reported	6 fish nested in 2 tanks	Not available	Not estimable	Not estimable	Not estimable	Recovery-period fatty-acid value retained descriptively	No concurrent matched comparator was reported for the recovery period, and tank-level variance was unavailable
Watson et al. (2013)	Gilthead seabream (Sparus aurata)	Alternative lipid sources in taurine-supplemented, fishmeal-free plant-protein diets	MSC recovery after switch to ARS control	No concurrent ARS-control recovery group reported	Recovery-fillet fatty acid 20:5n-3 (g/100 g total fatty acids)	4.82 ± 0.38	Not reported	6 fish nested in 2 tanks	Not available	Not estimable	Not estimable	Not estimable	Recovery-period fatty-acid value retained descriptively	No concurrent matched comparator was reported for the recovery period, and tank-level variance was unavailable
Watson et al. (2013)	Gilthead seabream (Sparus aurata)	Alternative lipid sources in taurine-supplemented, fishmeal-free plant-protein diets	MSC recovery after switch to ARS control	No concurrent ARS-control recovery group reported	Recovery-fillet fatty acid 22:5n-6 (g/100 g total fatty acids)	2.40 ± 0.22	Not reported	6 fish nested in 2 tanks	Not available	Not estimable	Not estimable	Not estimable	Recovery-period fatty-acid value retained descriptively	No concurrent matched comparator was reported for the recovery period, and tank-level variance was unavailable
Watson et al. (2013)	Gilthead seabream (Sparus aurata)	Alternative lipid sources in taurine-supplemented, fishmeal-free plant-protein diets	MSC recovery after switch to ARS control	No concurrent ARS-control recovery group reported	Recovery-fillet fatty acid 22:5n-3 (g/100 g total fatty acids)	1.80 ± 0.11	Not reported	6 fish nested in 2 tanks	Not available	Not estimable	Not estimable	Not estimable	Recovery-period fatty-acid value retained descriptively	No concurrent matched comparator was reported for the recovery period, and tank-level variance was unavailable
Watson et al. (2013)	Gilthead seabream (Sparus aurata)	Alternative lipid sources in taurine-supplemented, fishmeal-free plant-protein diets	MSC recovery after switch to ARS control	No concurrent ARS-control recovery group reported	Recovery-fillet fatty acid 22:6n-3 (g/100 g total fatty acids)	12.33 ± 1.04	Not reported	6 fish nested in 2 tanks	Not available	Not estimable	Not estimable	Not estimable	Recovery-period fatty-acid value retained descriptively	No concurrent matched comparator was reported for the recovery period, and tank-level variance was unavailable
Watson et al. (2013)	Gilthead seabream (Sparus aurata)	Alternative lipid sources in taurine-supplemented, fishmeal-free plant-protein diets	MSC recovery after switch to ARS control	No concurrent ARS-control recovery group reported	Recovery-fillet fatty acid PUFA (g/100 g total fatty acids)	34.78 ± 1.84	Not reported	6 fish nested in 2 tanks	Not available	Not estimable	Not estimable	Not estimable	Recovery-period fatty-acid value retained descriptively	No concurrent matched comparator was reported for the recovery period, and tank-level variance was unavailable
Watson et al. (2013)	Gilthead seabream (Sparus aurata)	Alternative lipid sources in taurine-supplemented, fishmeal-free plant-protein diets	CO+EFA recovery after switch to ARS control	No concurrent ARS-control recovery group reported	Recovery-fillet fatty acid 12:0 (g/100 g total fatty acids)	0.08 ± 0.01	Not reported	6 fish nested in 2 tanks	Not available	Not estimable	Not estimable	Not estimable	Recovery-period fatty-acid value retained descriptively	No concurrent matched comparator was reported for the recovery period, and tank-level variance was unavailable
Watson et al. (2013)	Gilthead seabream (Sparus aurata)	Alternative lipid sources in taurine-supplemented, fishmeal-free plant-protein diets	CO+EFA recovery after switch to ARS control	No concurrent ARS-control recovery group reported	Recovery-fillet fatty acid 14:0 (g/100 g total fatty acids)	2.68 ± 0.11	Not reported	6 fish nested in 2 tanks	Not available	Not estimable	Not estimable	Not estimable	Recovery-period fatty-acid value retained descriptively	No concurrent matched comparator was reported for the recovery period, and tank-level variance was unavailable
Watson et al. (2013)	Gilthead seabream (Sparus aurata)	Alternative lipid sources in taurine-supplemented, fishmeal-free plant-protein diets	CO+EFA recovery after switch to ARS control	No concurrent ARS-control recovery group reported	Recovery-fillet fatty acid 16:0 (g/100 g total fatty acids)	18.61 ± 0.61	Not reported	6 fish nested in 2 tanks	Not available	Not estimable	Not estimable	Not estimable	Recovery-period fatty-acid value retained descriptively	No concurrent matched comparator was reported for the recovery period, and tank-level variance was unavailable
Watson et al. (2013)	Gilthead seabream (Sparus aurata)	Alternative lipid sources in taurine-supplemented, fishmeal-free plant-protein diets	CO+EFA recovery after switch to ARS control	No concurrent ARS-control recovery group reported	Recovery-fillet fatty acid 17:0 (g/100 g total fatty acids)	0.21 ± 0.02	Not reported	6 fish nested in 2 tanks	Not available	Not estimable	Not estimable	Not estimable	Recovery-period fatty-acid value retained descriptively	No concurrent matched comparator was reported for the recovery period, and tank-level variance was unavailable
Watson et al. (2013)	Gilthead seabream (Sparus aurata)	Alternative lipid sources in taurine-supplemented,	CO+EFA recovery after switch to ARS control	No concurrent ARS-control recovery group reported	Recovery-fillet fatty acid 18:0 (g/100 g total fatty	3.82 ± 0.11	Not reported	6 fish nested in 2	Not available	Not estimable	Not estimable	Not estimable	Recovery-period fatty-acid value retained descriptively	No concurrent matched comparator was reported for the recovery period, and tank-

Study	Species	Strategy	Intervention	Comparator	Outcome and unit	Intervention mean ± SD	Comparator mean ± SD	n intervention	n comparator	Effect-size metric	Effect size	95% CI	Interpretation	Reason not estimated
		fishmeal-free plant-protein diets			acids)			tanks						level variance was unavailable
Watson et al. (2013)	Gilthead seabream (Sparus aurata)	Alternative lipid sources in taurine-supplemented, fishmeal-free plant-protein diets	CO+EFA recovery after switch to ARS control	No concurrent ARS-control recovery group reported	Recovery-fillet fatty acid 20:0 (g/100 g total fatty acids)	0.31 ± 0.05	Not reported	6 fish nested in 2 tanks	Not available	Not estimable	Not estimable	Not estimable	Recovery-period fatty-acid value retained descriptively	No concurrent matched comparator was reported for the recovery period, and tank-level variance was unavailable
Watson et al. (2013)	Gilthead seabream (Sparus aurata)	Alternative lipid sources in taurine-supplemented, fishmeal-free plant-protein diets	CO+EFA recovery after switch to ARS control	No concurrent ARS-control recovery group reported	Recovery-fillet fatty acid 22:0 (g/100 g total fatty acids)	0.28 ± 0.06	Not reported	6 fish nested in 2 tanks	Not available	Not estimable	Not estimable	Not estimable	Recovery-period fatty-acid value retained descriptively	No concurrent matched comparator was reported for the recovery period, and tank-level variance was unavailable
Watson et al. (2013)	Gilthead seabream (Sparus aurata)	Alternative lipid sources in taurine-supplemented, fishmeal-free plant-protein diets	CO+EFA recovery after switch to ARS control	No concurrent ARS-control recovery group reported	Recovery-fillet fatty acid SFA (g/100 g total fatty acids)	26.00 ± 0.79	Not reported	6 fish nested in 2 tanks	Not available	Not estimable	Not estimable	Not estimable	Recovery-period fatty-acid value retained descriptively	No concurrent matched comparator was reported for the recovery period, and tank-level variance was unavailable
Watson et al. (2013)	Gilthead seabream (Sparus aurata)	Alternative lipid sources in taurine-supplemented, fishmeal-free plant-protein diets	CO+EFA recovery after switch to ARS control	No concurrent ARS-control recovery group reported	Recovery-fillet fatty acid 16:1n-7 (g/100 g total fatty acids)	5.16 ± 0.16	Not reported	6 fish nested in 2 tanks	Not available	Not estimable	Not estimable	Not estimable	Recovery-period fatty-acid value retained descriptively	No concurrent matched comparator was reported for the recovery period, and tank-level variance was unavailable
Watson et al. (2013)	Gilthead seabream (Sparus aurata)	Alternative lipid sources in taurine-supplemented, fishmeal-free plant-protein diets	CO+EFA recovery after switch to ARS control	No concurrent ARS-control recovery group reported	Recovery-fillet fatty acid 18:1n-7 (g/100 g total fatty acids)	2.64 ± 0.07	Not reported	6 fish nested in 2 tanks	Not available	Not estimable	Not estimable	Not estimable	Recovery-period fatty-acid value retained descriptively	No concurrent matched comparator was reported for the recovery period, and tank-level variance was unavailable
Watson et al. (2013)	Gilthead seabream (Sparus aurata)	Alternative lipid sources in taurine-supplemented, fishmeal-free plant-protein diets	CO+EFA recovery after switch to ARS control	No concurrent ARS-control recovery group reported	Recovery-fillet fatty acid 18:1n-9+6 (g/100 g total fatty acids)	34.57 ± 1.05	Not reported	6 fish nested in 2 tanks	Not available	Not estimable	Not estimable	Not estimable	Recovery-period fatty-acid value retained descriptively	No concurrent matched comparator was reported for the recovery period, and tank-level variance was unavailable
Watson et al. (2013)	Gilthead seabream (Sparus aurata)	Alternative lipid sources in taurine-supplemented, fishmeal-free plant-protein diets	CO+EFA recovery after switch to ARS control	No concurrent ARS-control recovery group reported	Recovery-fillet fatty acid 20:1n-15+cis-8 (g/100 g total fatty acids)	0.15 ± 0.03	Not reported	6 fish nested in 2 tanks	Not available	Not estimable	Not estimable	Not estimable	Recovery-period fatty-acid value retained descriptively	No concurrent matched comparator was reported for the recovery period, and tank-level variance was unavailable
Watson et al. (2013)	Gilthead seabream (Sparus aurata)	Alternative lipid sources in taurine-supplemented, fishmeal-free plant-protein diets	CO+EFA recovery after switch to ARS control	No concurrent ARS-control recovery group reported	Recovery-fillet fatty acid 20:1n-9 (g/100 g total fatty acids)	1.04 ± 0.08	Not reported	6 fish nested in 2 tanks	Not available	Not estimable	Not estimable	Not estimable	Recovery-period fatty-acid value retained descriptively	No concurrent matched comparator was reported for the recovery period, and tank-level variance was unavailable
Watson et al. (2013)	Gilthead seabream (Sparus aurata)	Alternative lipid sources in taurine-supplemented, fishmeal-free plant-protein diets	CO+EFA recovery after switch to ARS control	No concurrent ARS-control recovery group reported	Recovery-fillet fatty acid 24:1n-9 (g/100 g total fatty acids)	0.33 ± 0.08	Not reported	6 fish nested in 2 tanks	Not available	Not estimable	Not estimable	Not estimable	Recovery-period fatty-acid value retained descriptively	No concurrent matched comparator was reported for the recovery period, and tank-level variance was unavailable
Watson et al. (2013)	Gilthead seabream (Sparus aurata)	Alternative lipid sources in taurine-supplemented, fishmeal-free plant-protein diets	CO+EFA recovery after switch to ARS control	No concurrent ARS-control recovery group reported	Recovery-fillet fatty acid MUFA (g/100 g total fatty acids)	43.89 ± 1.12	Not reported	6 fish nested in 2 tanks	Not available	Not estimable	Not estimable	Not estimable	Recovery-period fatty-acid value retained descriptively	No concurrent matched comparator was reported for the recovery period, and tank-level variance was unavailable
Watson et al. (2013)	Gilthead seabream (Sparus aurata)	Alternative lipid sources in taurine-supplemented, fishmeal-free plant-protein diets	CO+EFA recovery after switch to ARS control	No concurrent ARS-control recovery group reported	Recovery-fillet fatty acid 16:3n-4 (g/100 g total fatty acids)	0.35 ± 0.03	Not reported	6 fish nested in 2 tanks	Not available	Not estimable	Not estimable	Not estimable	Recovery-period fatty-acid value retained descriptively	No concurrent matched comparator was reported for the recovery period, and tank-level variance was unavailable
Watson et al. (2013)	Gilthead seabream (Sparus aurata)	Alternative lipid sources in taurine-supplemented, fishmeal-free plant-protein diets	CO+EFA recovery after switch to ARS control	No concurrent ARS-control recovery group reported	Recovery-fillet fatty acid 16:4n-1 (g/100 g total fatty acids)	0.19 ± 0.02	Not reported	6 fish nested in 2 tanks	Not available	Not estimable	Not estimable	Not estimable	Recovery-period fatty-acid value retained descriptively	No concurrent matched comparator was reported for the recovery period, and tank-level variance was unavailable
Watson et al. (2013)	Gilthead seabream (Sparus aurata)	Alternative lipid sources in taurine-supplemented, fishmeal-free plant-protein diets	CO+EFA recovery after switch to ARS control	No concurrent ARS-control recovery group reported	Recovery-fillet fatty acid 18:2n-6 (g/100 g total fatty acids)	14.45 ± 0.14	Not reported	6 fish nested in 2 tanks	Not available	Not estimable	Not estimable	Not estimable	Recovery-period fatty-acid value retained descriptively	No concurrent matched comparator was reported for the recovery period, and tank-level variance was unavailable
Watson et al. (2013)	Gilthead seabream (Sparus aurata)	Alternative lipid sources in taurine-supplemented, fishmeal-free plant-protein diets	CO+EFA recovery after switch to ARS control	No concurrent ARS-control recovery group reported	Recovery-fillet fatty acid 18:3n-3 (g/100 g total fatty acids)	2.40 ± 0.08	Not reported	6 fish nested in 2 tanks	Not available	Not estimable	Not estimable	Not estimable	Recovery-period fatty-acid value retained descriptively	No concurrent matched comparator was reported for the recovery period, and tank-level variance was unavailable
Watson et al. (2013)	Gilthead seabream (Sparus aurata)	Alternative lipid sources in taurine-supplemented, fishmeal-free plant-protein diets	CO+EFA recovery after switch to ARS control	No concurrent ARS-control recovery group reported	Recovery-fillet fatty acid 20:2n-6 (g/100 g total fatty acids)	0.36 ± 0.05	Not reported	6 fish nested in 2 tanks	Not available	Not estimable	Not estimable	Not estimable	Recovery-period fatty-acid value retained descriptively	No concurrent matched comparator was reported for the recovery period, and tank-level variance was unavailable
Watson et al. (2013)	Gilthead seabream (Sparus aurata)	Alternative lipid sources in taurine-supplemented, fishmeal-free plant-protein diets	CO+EFA recovery after switch to ARS control	No concurrent ARS-control recovery group reported	Recovery-fillet fatty acid 20:4n-6 (g/100 g total fatty acids)	0.58 ± 0.08	Not reported	6 fish nested in 2 tanks	Not available	Not estimable	Not estimable	Not estimable	Recovery-period fatty-acid value retained descriptively	No concurrent matched comparator was reported for the recovery period, and tank-level variance was unavailable
Watson et al. (2013)	Gilthead seabream (Sparus aurata)	Alternative lipid sources in taurine-supplemented, fishmeal-free plant-protein diets	CO+EFA recovery after switch to ARS control	No concurrent ARS-control recovery group reported	Recovery-fillet fatty acid 20:5n-3 (g/100 g total fatty acids)	3.39 ± 0.29	Not reported	6 fish nested in 2 tanks	Not available	Not estimable	Not estimable	Not estimable	Recovery-period fatty-acid value retained descriptively	No concurrent matched comparator was reported for the recovery period, and tank-level variance was unavailable
Watson et al. (2013)	Gilthead seabream (Sparus aurata)	Alternative lipid sources in taurine-supplemented, fishmeal-free plant-protein diets	CO+EFA recovery after switch to ARS control	No concurrent ARS-control recovery group reported	Recovery-fillet fatty acid 22:5n-6 (g/100 g total fatty acids)	0.36 ± 0.06	Not reported	6 fish nested in 2 tanks	Not available	Not estimable	Not estimable	Not estimable	Recovery-period fatty-acid value retained descriptively	No concurrent matched comparator was reported for the recovery period, and tank-level variance was unavailable
Watson et al. (2013)	Gilthead seabream (Sparus aurata)	Alternative lipid sources in taurine-supplemented, fishmeal-free plant-protein diets	CO+EFA recovery after switch to ARS control	No concurrent ARS-control recovery group reported	Recovery-fillet fatty acid 22:5n-3 (g/100 g total fatty acids)	1.39 ± 0.14	Not reported	6 fish nested in 2 tanks	Not available	Not estimable	Not estimable	Not estimable	Recovery-period fatty-acid value retained descriptively	No concurrent matched comparator was reported for the recovery period, and tank-level variance was unavailable
Watson et al. (2013)	Gilthead seabream (Sparus aurata)	Alternative lipid sources in taurine-supplemented, fishmeal-free plant-protein diets	CO+EFA recovery after switch to ARS control	No concurrent ARS-control recovery group reported	Recovery-fillet fatty acid 22:6n-3 (g/100 g total fatty acids)	6.21 ± 0.57	Not reported	6 fish nested in 2 tanks	Not available	Not estimable	Not estimable	Not estimable	Recovery-period fatty-acid value retained descriptively	No concurrent matched comparator was reported for the recovery period, and tank-level variance was unavailable
Watson et al. (2013)	Gilthead seabream (Sparus aurata)	Alternative lipid sources in taurine-supplemented, fishmeal-free plant-protein diets	CO+EFA recovery after switch to ARS control	No concurrent ARS-control recovery group reported	Recovery-fillet fatty acid PUFA (g/100 g total fatty acids)	30.11 ± 1.13	Not reported	6 fish nested in 2 tanks	Not available	Not estimable	Not estimable	Not estimable	Recovery-period fatty-acid value retained descriptively	No concurrent matched comparator was reported for the recovery period, and tank-level variance was unavailable

Notes. Hedges' g was calculated only for clearly tank-derived outcomes using two tanks per diet. FCR was sign-reversed. All diets contained 15 g/kg taurine; therefore, the study does not estimate an independent taurine effect. Sampled-fish and fatty-acid outcomes were retained descriptively because tank-level variance was not reported. Recovery-period fatty acids lacked a concurrent matched comparator.

Study 5. Yamamoto et al. (2019): histidine supplementation in fishmeal-based and 15% fishmeal diets for yellowtail

Matched comparisons: FM1H vs FM1, LFMH1 vs LFM, and LFMH2 vs LFM. *SD was derived from the pooled SEM reported in the article: SD = SEM × sqrt(2).

Study	Species	Strategy	Intervention	Comparator	Outcome and unit	Intervention mean ± SD	Comparator mean ± SD	n intervention	n comparator	Effect-size metric	Effect size	95% CI	Interpretation	Reason not estimated
Yamamoto et al. (2019)	Yellowtail (Seriola quinqueradiata)	Histidine supplementation to fishmeal-based and 15% fishmeal diets	FM1H (50% anchovy meal; analyzed histidine 1.89%)	FM1 (50% anchovy meal; analyzed histidine 1.27%)	Final body weight (g)	50.500 ± 0.962*	47.600 ± 0.962*	2	2	Hedges' g (SD derived from pooled SEM; LS variance)	1.7	-0.59 to 3.99	Higher growth outcome with histidine supplementation; 95% CI included 0 (very imprecise estimate)	Not applicable—effect size estimated
Yamamoto et al. (2019)	Yellowtail (Seriola quinqueradiata)	Histidine supplementation to fishmeal-based and 15% fishmeal diets	FM1H (50% anchovy meal; analyzed histidine 1.89%)	FM1 (50% anchovy meal; analyzed histidine 1.27%)	Weight gain (%)	986.000 ± 18.809*	923.000 ± 18.809*	2	2	Hedges' g (SD derived from pooled SEM; LS variance)	1.89	-0.47 to 4.25	Higher growth outcome with histidine supplementation; 95% CI included 0 (very imprecise estimate)	Not applicable—effect size estimated
Yamamoto et al. (2019)	Yellowtail (Seriola quinqueradiata)	Histidine supplementation to fishmeal-based and 15% fishmeal diets	FM1H (50% anchovy meal; analyzed histidine 1.89%)	FM1 (50% anchovy meal; analyzed histidine 1.27%)	Specific growth rate (% BW/day)	5.300 ± 0.115*	5.170 ± 0.115*	2	2	Hedges' g (SD derived from pooled SEM; LS variance)	0.64	-1.37 to 2.65	Higher growth outcome with histidine supplementation; 95% CI included 0 (very imprecise estimate)	Not applicable—effect size estimated
Yamamoto et al. (2019)	Yellowtail (Seriola quinqueradiata)	Histidine supplementation to fishmeal-based and 15% fishmeal diets	FM1H (50% anchovy meal; analyzed histidine 1.89%)	FM1 (50% anchovy meal; analyzed histidine 1.27%)	Feed efficiency ratio	1.060 ± 0.034*	1.110 ± 0.034*	2	2	Hedges' g (SD derived from pooled SEM; LS variance)	-0.83	-2.87 to 1.21	Lower feed efficiency ratio with histidine supplementation; 95% CI included 0 (very imprecise estimate)	Not applicable—effect size estimated
Yamamoto et al. (2019)	Yellowtail (Seriola quinqueradiata)	Histidine supplementation to fishmeal-based and 15% fishmeal diets	FM1H (50% anchovy meal; analyzed histidine 1.89%)	FM1 (50% anchovy meal; analyzed histidine 1.27%)	Feed intake (% BW/day)	3.920 ± 0.071*	3.700 ± 0.071*	2	2	Hedges' g (SD derived from pooled SEM; LS variance)	1.76	-0.55 to 4.06	Higher feed intake with histidine supplementation; 95% CI included 0 (very imprecise estimate)	Not applicable—effect size estimated
Yamamoto et al. (2019)	Yellowtail (Seriola quinqueradiata)	Histidine supplementation to fishmeal-based and 15% fishmeal diets	FM1H (50% anchovy meal; analyzed histidine 1.89%)	FM1 (50% anchovy meal; analyzed histidine 1.27%)	Whole-body moisture (%)	73.200 ± 0.382*	73.900 ± 0.382*	2	2	Hedges' g (SD derived from pooled SEM; LS variance)	-1.03	-3.12 to 1.05	Lower value with histidine supplementation; 95% CI included 0 (very imprecise estimate)	Not applicable—effect size estimated
Yamamoto et al. (2019)	Yellowtail (Seriola quinqueradiata)	Histidine supplementation to fishmeal-based and 15% fishmeal diets	FM1H (50% anchovy meal; analyzed histidine 1.89%)	FM1 (50% anchovy meal; analyzed histidine 1.27%)	Whole-body crude protein (%)	18.000 ± 0.099*	18.100 ± 0.099*	2	2	Hedges' g (SD derived from pooled SEM; LS variance)	-0.57	-2.57 to 1.43	Lower value with histidine supplementation; 95% CI included 0 (very imprecise estimate)	Not applicable—effect size estimated
Yamamoto et al. (2019)	Yellowtail (Seriola quinqueradiata)	Histidine supplementation to fishmeal-based and 15% fishmeal diets	FM1H (50% anchovy meal; analyzed histidine 1.89%)	FM1 (50% anchovy meal; analyzed histidine 1.27%)	Whole-body crude fat (%)	5.100 ± 0.198*	4.600 ± 0.198*	2	2	Hedges' g (SD derived from pooled SEM; LS variance)	1.42	-0.77 to 3.62	Higher value with histidine supplementation; 95% CI included 0 (very imprecise estimate)	Not applicable—effect size estimated
Yamamoto et al. (2019)	Yellowtail (Seriola quinqueradiata)	Histidine supplementation to fishmeal-based and 15% fishmeal diets	FM1H (50% anchovy meal; analyzed histidine 1.89%)	FM1 (50% anchovy meal; analyzed histidine 1.27%)	Whole-body ash (%)	2.800 ± 0.071*	2.900 ± 0.071*	2	2	Hedges' g (SD derived from pooled SEM; LS variance)	-0.8	-2.83 to 1.24	Lower value with histidine supplementation; 95% CI included 0 (very imprecise estimate)	Not applicable—effect size estimated
Yamamoto et al. (2019)	Yellowtail (Seriola quinqueradiata)	Histidine supplementation to fishmeal-based and 15% fishmeal diets	FM1H (50% anchovy meal; analyzed histidine 1.89%)	FM1 (50% anchovy meal; analyzed histidine 1.27%)	Hepatosomatic index (% BW)	1.000 ± 0.061*	0.970 ± 0.061*	2	2	Hedges' g (SD derived from pooled SEM; LS variance)	0.28	-1.69 to 2.25	Higher value with histidine supplementation; 95% CI included 0 (very imprecise estimate)	Not applicable—effect size estimated
Yamamoto et al. (2019)	Yellowtail (Seriola quinqueradiata)	Histidine supplementation to fishmeal-based and 15% fishmeal diets	FM1H (50% anchovy meal; analyzed histidine 1.89%)	FM1 (50% anchovy meal; analyzed histidine 1.27%)	Hemoglobin (g/dL)	10.000 ± 0.322*	9.800 ± 0.322*	2	2	Hedges' g (SD derived from pooled SEM; LS variance)	0.35	-1.62 to 2.32	Higher value with histidine supplementation; 95% CI included 0 (very imprecise estimate)	Not applicable—effect size estimated
Yamamoto et al. (2019)	Yellowtail (Seriola quinqueradiata)	Histidine supplementation to fishmeal-based and 15% fishmeal diets	FM1H (50% anchovy meal; analyzed histidine 1.89%)	FM1 (50% anchovy meal; analyzed histidine 1.27%)	Plasma total protein (g/dL)	3.100 ± 0.127*	3.000 ± 0.127*	2	2	Hedges' g (SD derived from pooled SEM; LS variance)	0.44	-1.54 to 2.43	Higher value with histidine supplementation; 95% CI included 0 (very imprecise estimate)	Not applicable—effect size estimated
Yamamoto et al. (2019)	Yellowtail (Seriola quinqueradiata)	Histidine supplementation to fishmeal-based and 15% fishmeal diets	FM1H (50% anchovy meal; analyzed histidine 1.89%)	FM1 (50% anchovy meal; analyzed histidine 1.27%)	Plasma total cholesterol (mg/dL)	296.000 ± 12.304*	304.000 ± 12.304*	2	2	Hedges' g (SD derived from pooled SEM; LS variance)	-0.37	-2.34 to 1.61	Lower value with histidine supplementation; 95% CI included 0 (very imprecise estimate)	Not applicable—effect size estimated
Yamamoto et al. (2019)	Yellowtail (Seriola quinqueradiata)	Histidine supplementation to fishmeal-based and 15% fishmeal diets	FM1H (50% anchovy meal; analyzed histidine 1.89%)	FM1 (50% anchovy meal; analyzed histidine 1.27%)	Plasma triacylglycerol (mg/dL)	64.000 ± 4.384*	54.000 ± 4.384*	2	2	Hedges' g (SD derived from pooled SEM; LS variance)	1.29	-0.87 to 3.44	Higher value with histidine supplementation; 95% CI included 0 (very imprecise estimate)	Not applicable—effect size estimated
Yamamoto et al. (2019)	Yellowtail (Seriola quinqueradiata)	Histidine supplementation to fishmeal-based and 15% fishmeal diets	FM1H (50% anchovy meal; analyzed histidine 1.89%)	FM1 (50% anchovy meal; analyzed histidine 1.27%)	Plasma glucose (mg/dL)	133.000 ± 6.505*	151.000 ± 6.505*	2	2	Hedges' g (SD derived from pooled SEM; LS variance)	-1.56	-3.80 to 0.68	Lower value with histidine supplementation; 95% CI included 0 (very imprecise estimate)	Not applicable—effect size estimated
Yamamoto et al. (2019)	Yellowtail (Seriola quinqueradiata)	Histidine supplementation to fishmeal-based and 15% fishmeal diets	FM1H (50% anchovy meal; analyzed histidine 1.89%)	FM1 (50% anchovy meal; analyzed histidine 1.27%)	Liver taurine (mg/g)	5.320 ± 0.175*	5.570 ± 0.175*	2	2	Hedges' g (SD derived from pooled SEM; LS variance)	-0.8	-2.84 to 1.23	Lower value with histidine supplementation; 95% CI included 0 (very imprecise estimate)	Not applicable—effect size estimated
Yamamoto et al. (2019)	Yellowtail (Seriola quinqueradiata)	Histidine supplementation to fishmeal-based and 15% fishmeal diets	FM1H (50% anchovy meal; analyzed histidine 1.89%)	FM1 (50% anchovy meal; analyzed histidine 1.27%)	Liver Glx (mg/g)	1.850 ± 0.079*	1.570 ± 0.079*	2	2	Hedges' g (SD derived from pooled SEM; LS variance)	1.99	-0.40 to 4.39	Higher value with histidine supplementation; 95% CI included 0 (very imprecise estimate)	Not applicable—effect size estimated
Yamamoto et al. (2019)	Yellowtail (Seriola quinqueradiata)	Histidine supplementation to fishmeal-based and 15% fishmeal diets	FM1H (50% anchovy meal; analyzed histidine 1.89%)	FM1 (50% anchovy meal; analyzed histidine 1.27%)	Liver Gly+Ala (mg/g)	0.980 ± 0.177*	1.300 ± 0.177*	2	2	Hedges' g (SD derived from pooled SEM; LS variance)	-1.02	-3.11 to 1.06	Lower value with histidine supplementation; 95% CI included 0 (very imprecise estimate)	Not applicable—effect size estimated
Yamamoto et al. (2019)	Yellowtail (Seriola quinqueradiata)	Histidine supplementation to fishmeal-based and 15% fishmeal diets	FM1H (50% anchovy meal; analyzed histidine 1.89%)	FM1 (50% anchovy meal; analyzed histidine 1.27%)	Liver histidine (mg/g)	0.110 ± 0.040*	0.100 ± 0.040*	2	2	Hedges' g (SD derived from pooled SEM; LS variance)	0.14	-1.82 to 2.10	Higher value with histidine supplementation; 95% CI included 0 (very imprecise estimate)	Not applicable—effect size estimated
Yamamoto et al. (2019)	Yellowtail (Seriola quinqueradiata)	Histidine supplementation to fishmeal-based and 15% fishmeal diets	FM1H (50% anchovy meal; analyzed histidine 1.89%)	FM1 (50% anchovy meal; analyzed histidine 1.27%)	Liver EAA (mg/g)	0.890 ± 0.158*	0.910 ± 0.158*	2	2	Hedges' g (SD derived from pooled SEM; LS variance)	-0.07	-2.03 to 1.89	Lower value with histidine supplementation; 95% CI included 0 (very imprecise estimate)	Not applicable—effect size estimated
Yamamoto et al. (2019)	Yellowtail (Seriola quinqueradiata)	Histidine supplementation to fishmeal-based and 15% fishmeal diets	FM1H (50% anchovy meal; analyzed histidine 1.89%)	FM1 (50% anchovy meal; analyzed histidine 1.27%)	Liver NEAA (mg/g)	3.190 ± 0.194*	3.200 ± 0.194*	2	2	Hedges' g (SD derived from pooled SEM; LS variance)	-0.03	-1.99 to 1.93	Lower value with histidine supplementation; 95% CI included 0 (very imprecise estimate)	Not applicable—effect size estimated
Yamamoto et al. (2019)	Yellowtail (Seriola quinqueradiata)	Histidine supplementation to fishmeal-based and 15%	FM1H (50% anchovy meal; analyzed histidine 1.89%)	FM1 (50% anchovy meal; analyzed histidine 1.27%)	Muscle taurine (mg/g)	3.620 ± 0.164*	4.500 ± 0.164*	2	2	Hedges' g (SD derived from	-3.03	-5.90 to -0.16	Lower muscle taurine with supplementation; 95% CI excluded 0	Not applicable—effect size estimated

Study	Species	Strategy	Intervention	Comparator	Outcome and unit	Intervention mean ± SD	Comparator mean ± SD	n intervention	n comparator	Effect-size metric	Effect size	95% CI	Interpretation	Reason not estimated
		fishmeal diets								pooled SEM; LS variance)				
Yamamoto et al. (2019)	Yellowtail (Seriola quinqueradiata)	Histidine supplementation to fishmeal-based and 15% fishmeal diets	FM1H (50% anchovy meal; analyzed histidine 1.89%)	FM1 (50% anchovy meal; analyzed histidine 1.27%)	Muscle Glx (mg/g)	0.340 ± 0.065*	0.330 ± 0.065*	2	2	Hedges' g (SD derived from pooled SEM; LS variance)	0.09	-1.87 to 2.05	Higher value with histidine supplementation; 95% CI included 0 (very imprecise estimate)	Not applicable—effect size estimated
Yamamoto et al. (2019)	Yellowtail (Seriola quinqueradiata)	Histidine supplementation to fishmeal-based and 15% fishmeal diets	FM1H (50% anchovy meal; analyzed histidine 1.89%)	FM1 (50% anchovy meal; analyzed histidine 1.27%)	Muscle Gly+Ala (mg/g)	0.430 ± 0.034*	0.530 ± 0.034*	2	2	Hedges' g (SD derived from pooled SEM; LS variance)	-1.66	-3.94 to 0.61	Lower value with histidine supplementation; 95% CI included 0 (very imprecise estimate)	Not applicable—effect size estimated
Yamamoto et al. (2019)	Yellowtail (Seriola quinqueradiata)	Histidine supplementation to fishmeal-based and 15% fishmeal diets	FM1H (50% anchovy meal; analyzed histidine 1.89%)	FM1 (50% anchovy meal; analyzed histidine 1.27%)	Muscle histidine (mg/g)	7.170 ± 0.168*	4.460 ± 0.168*	2	2	Hedges' g (SD derived from pooled SEM; LS variance)	9.09	2.49 to 15.68	Higher muscle free histidine with supplementation; 95% CI excluded 0	Not applicable—effect size estimated
Yamamoto et al. (2019)	Yellowtail (Seriola quinqueradiata)	Histidine supplementation to fishmeal-based and 15% fishmeal diets	FM1H (50% anchovy meal; analyzed histidine 1.89%)	FM1 (50% anchovy meal; analyzed histidine 1.27%)	Muscle EAA excluding histidine (mg/g)	0.410 ± 0.025*	0.400 ± 0.025*	2	2	Hedges' g (SD derived from pooled SEM; LS variance)	0.22	-1.74 to 2.19	Higher value with histidine supplementation; 95% CI included 0 (very imprecise estimate)	Not applicable—effect size estimated
Yamamoto et al. (2019)	Yellowtail (Seriola quinqueradiata)	Histidine supplementation to fishmeal-based and 15% fishmeal diets	FM1H (50% anchovy meal; analyzed histidine 1.89%)	FM1 (50% anchovy meal; analyzed histidine 1.27%)	Muscle NEAA (mg/g)	0.960 ± 0.099*	1.160 ± 0.099*	2	2	Hedges' g (SD derived from pooled SEM; LS variance)	-1.14	-3.25 to 0.97	Lower value with histidine supplementation; 95% CI included 0 (very imprecise estimate)	Not applicable—effect size estimated
Yamamoto et al. (2019)	Yellowtail (Seriola quinqueradiata)	Histidine supplementation to fishmeal-based and 15% fishmeal diets	LFMH1 (15% fishmeal; analyzed histidine 1.22%)	LFM (15% fishmeal; analyzed histidine 1.12%)	Final body weight (g)	37.300 ± 0.962*	34.500 ± 0.962*	2	2	Hedges' g (SD derived from pooled SEM; LS variance)	1.64	-0.62 to 3.91	Higher growth outcome with histidine supplementation; 95% CI included 0 (very imprecise estimate)	Not applicable—effect size estimated
Yamamoto et al. (2019)	Yellowtail (Seriola quinqueradiata)	Histidine supplementation to fishmeal-based and 15% fishmeal diets	LFMH1 (15% fishmeal; analyzed histidine 1.22%)	LFM (15% fishmeal; analyzed histidine 1.12%)	Weight gain (%)	706.000 ± 18.809*	651.000 ± 18.809*	2	2	Hedges' g (SD derived from pooled SEM; LS variance)	1.65	-0.62 to 3.92	Higher growth outcome with histidine supplementation; 95% CI included 0 (very imprecise estimate)	Not applicable—effect size estimated
Yamamoto et al. (2019)	Yellowtail (Seriola quinqueradiata)	Histidine supplementation to fishmeal-based and 15% fishmeal diets	LFMH1 (15% fishmeal; analyzed histidine 1.22%)	LFM (15% fishmeal; analyzed histidine 1.12%)	Specific growth rate (% BW/day)	4.640 ± 0.115*	4.480 ± 0.115*	2	2	Hedges' g (SD derived from pooled SEM; LS variance)	0.79	-1.25 to 2.82	Higher growth outcome with histidine supplementation; 95% CI included 0 (very imprecise estimate)	Not applicable—effect size estimated
Yamamoto et al. (2019)	Yellowtail (Seriola quinqueradiata)	Histidine supplementation to fishmeal-based and 15% fishmeal diets	LFMH1 (15% fishmeal; analyzed histidine 1.22%)	LFM (15% fishmeal; analyzed histidine 1.12%)	Feed efficiency ratio	1.020 ± 0.034*	1.030 ± 0.034*	2	2	Hedges' g (SD derived from pooled SEM; LS variance)	-0.17	-2.13 to 1.80	Lower feed efficiency ratio with histidine supplementation; 95% CI included 0 (very imprecise estimate)	Not applicable—effect size estimated
Yamamoto et al. (2019)	Yellowtail (Seriola quinqueradiata)	Histidine supplementation to fishmeal-based and 15% fishmeal diets	LFMH1 (15% fishmeal; analyzed histidine 1.22%)	LFM (15% fishmeal; analyzed histidine 1.12%)	Feed intake (% BW/day)	3.790 ± 0.071*	3.720 ± 0.071*	2	2	Hedges' g (SD derived from pooled SEM; LS variance)	0.56	-1.44 to 2.56	Higher feed intake with histidine supplementation; 95% CI included 0 (very imprecise estimate)	Not applicable—effect size estimated
Yamamoto et al. (2019)	Yellowtail (Seriola quinqueradiata)	Histidine supplementation to fishmeal-based and 15% fishmeal diets	LFMH1 (15% fishmeal; analyzed histidine 1.22%)	LFM (15% fishmeal; analyzed histidine 1.12%)	Whole-body moisture (%)	75.000 ± 0.382*	74.600 ± 0.382*	2	2	Hedges' g (SD derived from pooled SEM; LS variance)	0.59	-1.41 to 2.59	Higher value with histidine supplementation; 95% CI included 0 (very imprecise estimate)	Not applicable—effect size estimated
Yamamoto et al. (2019)	Yellowtail (Seriola quinqueradiata)	Histidine supplementation to fishmeal-based and 15% fishmeal diets	LFMH1 (15% fishmeal; analyzed histidine 1.22%)	LFM (15% fishmeal; analyzed histidine 1.12%)	Whole-body crude protein (%)	17.600 ± 0.099*	17.700 ± 0.099*	2	2	Hedges' g (SD derived from pooled SEM; LS variance)	-0.57	-2.57 to 1.43	Lower value with histidine supplementation; 95% CI included 0 (very imprecise estimate)	Not applicable—effect size estimated
Yamamoto et al. (2019)	Yellowtail (Seriola quinqueradiata)	Histidine supplementation to fishmeal-based and 15% fishmeal diets	LFMH1 (15% fishmeal; analyzed histidine 1.22%)	LFM (15% fishmeal; analyzed histidine 1.12%)	Whole-body crude fat (%)	4.600 ± 0.198*	4.300 ± 0.198*	2	2	Hedges' g (SD derived from pooled SEM; LS variance)	0.85	-1.19 to 2.90	Higher value with histidine supplementation; 95% CI included 0 (very imprecise estimate)	Not applicable—effect size estimated
Yamamoto et al. (2019)	Yellowtail (Seriola quinqueradiata)	Histidine supplementation to fishmeal-based and 15% fishmeal diets	LFMH1 (15% fishmeal; analyzed histidine 1.22%)	LFM (15% fishmeal; analyzed histidine 1.12%)	Whole-body ash (%)	2.800 ± 0.071*	2.800 ± 0.071*	2	2	Hedges' g (SD derived from pooled SEM; LS variance)	0.0	-1.96 to 1.96	No observed mean difference; 95% CI included 0 (very imprecise estimate)	Not applicable—effect size estimated
Yamamoto et al. (2019)	Yellowtail (Seriola quinqueradiata)	Histidine supplementation to fishmeal-based and 15% fishmeal diets	LFMH1 (15% fishmeal; analyzed histidine 1.22%)	LFM (15% fishmeal; analyzed histidine 1.12%)	Hepatosomatic index (% BW)	0.880 ± 0.061*	0.850 ± 0.061*	2	2	Hedges' g (SD derived from pooled SEM; LS variance)	0.28	-1.69 to 2.25	Higher value with histidine supplementation; 95% CI included 0 (very imprecise estimate)	Not applicable—effect size estimated
Yamamoto et al. (2019)	Yellowtail (Seriola quinqueradiata)	Histidine supplementation to fishmeal-based and 15% fishmeal diets	LFMH1 (15% fishmeal; analyzed histidine 1.22%)	LFM (15% fishmeal; analyzed histidine 1.12%)	Hemoglobin (g/dL)	9.100 ± 0.322*	9.500 ± 0.322*	2	2	Hedges' g (SD derived from pooled SEM; LS variance)	-0.7	-2.72 to 1.32	Lower value with histidine supplementation; 95% CI included 0 (very imprecise estimate)	Not applicable—effect size estimated
Yamamoto et al. (2019)	Yellowtail (Seriola quinqueradiata)	Histidine supplementation to fishmeal-based and 15% fishmeal diets	LFMH1 (15% fishmeal; analyzed histidine 1.22%)	LFM (15% fishmeal; analyzed histidine 1.12%)	Plasma total protein (g/dL)	2.500 ± 0.127*	2.500 ± 0.127*	2	2	Hedges' g (SD derived from pooled SEM; LS variance)	0.0	-1.96 to 1.96	No observed mean difference; 95% CI included 0 (very imprecise estimate)	Not applicable—effect size estimated
Yamamoto et al. (2019)	Yellowtail (Seriola quinqueradiata)	Histidine supplementation to fishmeal-based and 15% fishmeal diets	LFMH1 (15% fishmeal; analyzed histidine 1.22%)	LFM (15% fishmeal; analyzed histidine 1.12%)	Plasma total cholesterol (mg/dL)	199.000 ± 12.304*	190.000 ± 12.304*	2	2	Hedges' g (SD derived from pooled SEM; LS variance)	0.41	-1.57 to 2.39	Higher value with histidine supplementation; 95% CI included 0 (very imprecise estimate)	Not applicable—effect size estimated
Yamamoto et al. (2019)	Yellowtail (Seriola quinqueradiata)	Histidine supplementation to fishmeal-based and 15% fishmeal diets	LFMH1 (15% fishmeal; analyzed histidine 1.22%)	LFM (15% fishmeal; analyzed histidine 1.12%)	Plasma triacylglycerol (mg/dL)	76.000 ± 4.384*	75.000 ± 4.384*	2	2	Hedges' g (SD derived from pooled SEM; LS variance)	0.13	-1.83 to 2.09	Higher value with histidine supplementation; 95% CI included 0 (very imprecise estimate)	Not applicable—effect size estimated
Yamamoto et al. (2019)	Yellowtail (Seriola quinqueradiata)	Histidine supplementation to fishmeal-based and 15% fishmeal diets	LFMH1 (15% fishmeal; analyzed histidine 1.22%)	LFM (15% fishmeal; analyzed histidine 1.12%)	Plasma glucose (mg/dL)	113.000 ± 6.505*	128.000 ± 6.505*	2	2	Hedges' g (SD derived from pooled SEM; LS variance)	-1.3	-3.46 to 0.86	Lower value with histidine supplementation; 95% CI included 0 (very imprecise estimate)	Not applicable—effect size estimated
Yamamoto et al. (2019)	Yellowtail (Seriola quinqueradiata)	Histidine supplementation to fishmeal-based and 15% fishmeal diets	LFMH1 (15% fishmeal; analyzed histidine 1.22%)	LFM (15% fishmeal; analyzed histidine 1.12%)	Liver taurine (mg/g)	5.640 ± 0.175*	5.990 ± 0.175*	2	2	Hedges' g (SD derived from pooled SEM; LS variance)	-1.13	-3.24 to 0.98	Lower value with histidine supplementation; 95% CI included 0 (very imprecise estimate)	Not applicable—effect size estimated
Yamamoto et al. (2019)	Yellowtail (Seriola quinqueradiata)	Histidine supplementation to fishmeal-based and 15% fishmeal diets	LFMH1 (15% fishmeal; analyzed histidine 1.22%)	LFM (15% fishmeal; analyzed histidine 1.12%)	Liver Glx (mg/g)	1.390 ± 0.079*	1.130 ± 0.079*	2	2	Hedges' g (SD derived from pooled SEM; LS variance)	1.85	-0.49 to 4.20	Higher value with histidine supplementation; 95% CI included 0 (very imprecise estimate)	Not applicable—effect size estimated

Study	Species	Strategy	Intervention	Comparator	Outcome and unit	Intervention mean ± SD	Comparator mean ± SD	n intervention	n comparator	Effect-size metric	Effect size	95% CI	Interpretation	Reason not estimated
Yamamoto et al. (2019)	Yellowtail (Seriola quinqueradiata)	Histidine supplementation to fishmeal-based and 15% fishmeal diets	LFMH1 (15% fishmeal; analyzed histidine 1.22%)	LFM (15% fishmeal; analyzed histidine 1.12%)	Liver Gly+Ala (mg/g)	1.090 ± 0.177*	1.320 ± 0.177*	2	2	Hedges' g (SD derived from pooled SEM; LS variance)	-0.73	-2.76 to 1.29	Lower value with histidine supplementation; 95% CI included 0 (very imprecise estimate)	Not applicable—effect size estimated
Yamamoto et al. (2019)	Yellowtail (Seriola quinqueradiata)	Histidine supplementation to fishmeal-based and 15% fishmeal diets	LFMH1 (15% fishmeal; analyzed histidine 1.22%)	LFM (15% fishmeal; analyzed histidine 1.12%)	Liver histidine (mg/g)	0.100 ± 0.040*	0.110 ± 0.040*	2	2	Hedges' g (SD derived from pooled SEM; LS variance)	-0.14	-2.10 to 1.82	Lower value with histidine supplementation; 95% CI included 0 (very imprecise estimate)	Not applicable—effect size estimated
Yamamoto et al. (2019)	Yellowtail (Seriola quinqueradiata)	Histidine supplementation to fishmeal-based and 15% fishmeal diets	LFMH1 (15% fishmeal; analyzed histidine 1.22%)	LFM (15% fishmeal; analyzed histidine 1.12%)	Liver EAA (mg/g)	0.900 ± 0.158*	1.050 ± 0.158*	2	2	Hedges' g (SD derived from pooled SEM; LS variance)	-0.53	-2.53 to 1.46	Lower value with histidine supplementation; 95% CI included 0 (very imprecise estimate)	Not applicable—effect size estimated
Yamamoto et al. (2019)	Yellowtail (Seriola quinqueradiata)	Histidine supplementation to fishmeal-based and 15% fishmeal diets	LFMH1 (15% fishmeal; analyzed histidine 1.22%)	LFM (15% fishmeal; analyzed histidine 1.12%)	Liver NEAA (mg/g)	2.860 ± 0.194*	2.880 ± 0.194*	2	2	Hedges' g (SD derived from pooled SEM; LS variance)	-0.06	-2.02 to 1.90	Lower value with histidine supplementation; 95% CI included 0 (very imprecise estimate)	Not applicable—effect size estimated
Yamamoto et al. (2019)	Yellowtail (Seriola quinqueradiata)	Histidine supplementation to fishmeal-based and 15% fishmeal diets	LFMH1 (15% fishmeal; analyzed histidine 1.22%)	LFM (15% fishmeal; analyzed histidine 1.12%)	Muscle taurine (mg/g)	4.840 ± 0.164*	5.250 ± 0.164*	2	2	Hedges' g (SD derived from pooled SEM; LS variance)	-1.41	-3.60 to 0.78	Lower muscle taurine with supplementation; 95% CI included 0 (very imprecise estimate)	Not applicable—effect size estimated
Yamamoto et al. (2019)	Yellowtail (Seriola quinqueradiata)	Histidine supplementation to fishmeal-based and 15% fishmeal diets	LFMH1 (15% fishmeal; analyzed histidine 1.22%)	LFM (15% fishmeal; analyzed histidine 1.12%)	Muscle Glx (mg/g)	0.470 ± 0.065*	0.400 ± 0.065*	2	2	Hedges' g (SD derived from pooled SEM; LS variance)	0.61	-1.40 to 2.61	Higher value with histidine supplementation; 95% CI included 0 (very imprecise estimate)	Not applicable—effect size estimated
Yamamoto et al. (2019)	Yellowtail (Seriola quinqueradiata)	Histidine supplementation to fishmeal-based and 15% fishmeal diets	LFMH1 (15% fishmeal; analyzed histidine 1.22%)	LFM (15% fishmeal; analyzed histidine 1.12%)	Muscle Gly+Ala (mg/g)	0.620 ± 0.034*	0.670 ± 0.034*	2	2	Hedges' g (SD derived from pooled SEM; LS variance)	-0.83	-2.87 to 1.21	Lower value with histidine supplementation; 95% CI included 0 (very imprecise estimate)	Not applicable—effect size estimated
Yamamoto et al. (2019)	Yellowtail (Seriola quinqueradiata)	Histidine supplementation to fishmeal-based and 15% fishmeal diets	LFMH1 (15% fishmeal; analyzed histidine 1.22%)	LFM (15% fishmeal; analyzed histidine 1.12%)	Muscle histidine (mg/g)	4.740 ± 0.168*	3.930 ± 0.168*	2	2	Hedges' g (SD derived from pooled SEM; LS variance)	2.72	-0.00 to 5.43	Higher muscle free histidine with supplementation; 95% CI included 0 (very imprecise estimate)	Not applicable—effect size estimated
Yamamoto et al. (2019)	Yellowtail (Seriola quinqueradiata)	Histidine supplementation to fishmeal-based and 15% fishmeal diets	LFMH1 (15% fishmeal; analyzed histidine 1.22%)	LFM (15% fishmeal; analyzed histidine 1.12%)	Muscle EAA excluding histidine (mg/g)	0.580 ± 0.025*	0.580 ± 0.025*	2	2	Hedges' g (SD derived from pooled SEM; LS variance)	0.0	-1.96 to 1.96	No observed mean difference; 95% CI included 0 (very imprecise estimate)	Not applicable—effect size estimated
Yamamoto et al. (2019)	Yellowtail (Seriola quinqueradiata)	Histidine supplementation to fishmeal-based and 15% fishmeal diets	LFMH1 (15% fishmeal; analyzed histidine 1.22%)	LFM (15% fishmeal; analyzed histidine 1.12%)	Muscle NEAA (mg/g)	1.410 ± 0.099*	1.400 ± 0.099*	2	2	Hedges' g (SD derived from pooled SEM; LS variance)	0.06	-1.90 to 2.02	Higher value with histidine supplementation; 95% CI included 0 (very imprecise estimate)	Not applicable—effect size estimated
Yamamoto et al. (2019)	Yellowtail (Seriola quinqueradiata)	Histidine supplementation to fishmeal-based and 15% fishmeal diets	LFMH2 (15% fishmeal; analyzed histidine 1.92%)	LFM (15% fishmeal; analyzed histidine 1.12%)	Final body weight (g)	38.300 ± 0.962*	34.500 ± 0.962*	2	2	Hedges' g (SD derived from pooled SEM; LS variance)	2.23	-0.27 to 4.73	Higher growth outcome with histidine supplementation; 95% CI included 0 (very imprecise estimate)	Not applicable—effect size estimated
Yamamoto et al. (2019)	Yellowtail (Seriola quinqueradiata)	Histidine supplementation to fishmeal-based and 15% fishmeal diets	LFMH2 (15% fishmeal; analyzed histidine 1.92%)	LFM (15% fishmeal; analyzed histidine 1.12%)	Weight gain (%)	721.000 ± 18.809*	651.000 ± 18.809*	2	2	Hedges' g (SD derived from pooled SEM; LS variance)	2.1	-0.34 to 4.54	Higher growth outcome with histidine supplementation; 95% CI included 0 (very imprecise estimate)	Not applicable—effect size estimated
Yamamoto et al. (2019)	Yellowtail (Seriola quinqueradiata)	Histidine supplementation to fishmeal-based and 15% fishmeal diets	LFMH2 (15% fishmeal; analyzed histidine 1.92%)	LFM (15% fishmeal; analyzed histidine 1.12%)	Specific growth rate (% BW/day)	4.680 ± 0.115*	4.480 ± 0.115*	2	2	Hedges' g (SD derived from pooled SEM; LS variance)	0.99	-1.09 to 3.06	Higher growth outcome with histidine supplementation; 95% CI included 0 (very imprecise estimate)	Not applicable—effect size estimated
Yamamoto et al. (2019)	Yellowtail (Seriola quinqueradiata)	Histidine supplementation to fishmeal-based and 15% fishmeal diets	LFMH2 (15% fishmeal; analyzed histidine 1.92%)	LFM (15% fishmeal; analyzed histidine 1.12%)	Feed efficiency ratio	1.020 ± 0.034*	1.030 ± 0.034*	2	2	Hedges' g (SD derived from pooled SEM; LS variance)	-0.17	-2.13 to 1.80	Lower feed efficiency ratio with histidine supplementation; 95% CI included 0 (very imprecise estimate)	Not applicable—effect size estimated
Yamamoto et al. (2019)	Yellowtail (Seriola quinqueradiata)	Histidine supplementation to fishmeal-based and 15% fishmeal diets	LFMH2 (15% fishmeal; analyzed histidine 1.92%)	LFM (15% fishmeal; analyzed histidine 1.12%)	Feed intake (% BW/day)	3.840 ± 0.071*	3.720 ± 0.071*	2	2	Hedges' g (SD derived from pooled SEM; LS variance)	0.96	-1.11 to 3.03	Higher feed intake with histidine supplementation; 95% CI included 0 (very imprecise estimate)	Not applicable—effect size estimated
Yamamoto et al. (2019)	Yellowtail (Seriola quinqueradiata)	Histidine supplementation to fishmeal-based and 15% fishmeal diets	LFMH2 (15% fishmeal; analyzed histidine 1.92%)	LFM (15% fishmeal; analyzed histidine 1.12%)	Whole-body moisture (%)	74.600 ± 0.382*	74.600 ± 0.382*	2	2	Hedges' g (SD derived from pooled SEM; LS variance)	0.0	-1.96 to 1.96	No observed mean difference; 95% CI included 0 (very imprecise estimate)	Not applicable—effect size estimated
Yamamoto et al. (2019)	Yellowtail (Seriola quinqueradiata)	Histidine supplementation to fishmeal-based and 15% fishmeal diets	LFMH2 (15% fishmeal; analyzed histidine 1.92%)	LFM (15% fishmeal; analyzed histidine 1.12%)	Whole-body crude protein (%)	17.700 ± 0.099*	17.700 ± 0.099*	2	2	Hedges' g (SD derived from pooled SEM; LS variance)	0.0	-1.96 to 1.96	No observed mean difference; 95% CI included 0 (very imprecise estimate)	Not applicable—effect size estimated
Yamamoto et al. (2019)	Yellowtail (Seriola quinqueradiata)	Histidine supplementation to fishmeal-based and 15% fishmeal diets	LFMH2 (15% fishmeal; analyzed histidine 1.92%)	LFM (15% fishmeal; analyzed histidine 1.12%)	Whole-body crude fat (%)	4.700 ± 0.198*	4.300 ± 0.198*	2	2	Hedges' g (SD derived from pooled SEM; LS variance)	1.14	-0.97 to 3.25	Higher value with histidine supplementation; 95% CI included 0 (very imprecise estimate)	Not applicable—effect size estimated
Yamamoto et al. (2019)	Yellowtail (Seriola quinqueradiata)	Histidine supplementation to fishmeal-based and 15% fishmeal diets	LFMH2 (15% fishmeal; analyzed histidine 1.92%)	LFM (15% fishmeal; analyzed histidine 1.12%)	Whole-body ash (%)	2.900 ± 0.071*	2.800 ± 0.071*	2	2	Hedges' g (SD derived from pooled SEM; LS variance)	0.8	-1.24 to 2.83	Higher value with histidine supplementation; 95% CI included 0 (very imprecise estimate)	Not applicable—effect size estimated
Yamamoto et al. (2019)	Yellowtail (Seriola quinqueradiata)	Histidine supplementation to fishmeal-based and 15% fishmeal diets	LFMH2 (15% fishmeal; analyzed histidine 1.92%)	LFM (15% fishmeal; analyzed histidine 1.12%)	Hepatosomatic index (% BW)	0.840 ± 0.061*	0.850 ± 0.061*	2	2	Hedges' g (SD derived from pooled SEM; LS variance)	-0.09	-2.05 to 1.87	Lower value with histidine supplementation; 95% CI included 0 (very imprecise estimate)	Not applicable—effect size estimated
Yamamoto et al. (2019)	Yellowtail (Seriola quinqueradiata)	Histidine supplementation to fishmeal-based and 15% fishmeal diets	LFMH2 (15% fishmeal; analyzed histidine 1.92%)	LFM (15% fishmeal; analyzed histidine 1.12%)	Hemoglobin (g/dL)	9.500 ± 0.322*	9.500 ± 0.322*	2	2	Hedges' g (SD derived from pooled SEM; LS variance)	0.0	-1.96 to 1.96	No observed mean difference; 95% CI included 0 (very imprecise estimate)	Not applicable—effect size estimated
Yamamoto et al. (2019)	Yellowtail (Seriola quinqueradiata)	Histidine supplementation to fishmeal-based and 15% fishmeal diets	LFMH2 (15% fishmeal; analyzed histidine 1.92%)	LFM (15% fishmeal; analyzed histidine 1.12%)	Plasma total protein (g/dL)	2.500 ± 0.127*	2.500 ± 0.127*	2	2	Hedges' g (SD derived from pooled SEM; LS variance)	0.0	-1.96 to 1.96	No observed mean difference; 95% CI included 0 (very imprecise estimate)	Not applicable—effect size estimated
Yamamoto et al. (2019)	Yellowtail (Seriola quinqueradiata)	Histidine supplementation to fishmeal-based and 15%	LFMH2 (15% fishmeal; analyzed histidine 1.92%)	LFM (15% fishmeal; analyzed histidine 1.12%)	Plasma total cholesterol (mg/dL)	210.000 ± 12.304*	190.000 ± 12.304*	2	2	Hedges' g (SD derived from	0.92	-1.14 to 2.98	Higher value with histidine supplementation; 95% CI included 0	Not applicable—effect size estimated

Study	Species	Strategy	Intervention	Comparator	Outcome and unit	Intervention mean ± SD	Comparator mean ± SD	n intervention	n comparator	Effect-size metric	Effect size	95% CI	Interpretation	Reason not estimated
		fishmeal diets								pooled SEM; LS variance)			(very imprecise estimate)	
Yamamoto et al. (2019)	Yellowtail (Seriola quinqueradiata)	Histidine supplementation to fishmeal-based and 15% fishmeal diets	LFMH2 (15% fishmeal; analyzed histidine 1.92%)	LFM (15% fishmeal; analyzed histidine 1.12%)	Plasma triacylglycerol (mg/dL)	71.000 ± 4.384*	75.000 ± 4.384*	2	2	Hedges' g (SD derived from pooled SEM; LS variance)	-0.51	-2.51 to 1.48	Lower value with histidine supplementation; 95% CI included 0 (very imprecise estimate)	Not applicable—effect size estimated
Yamamoto et al. (2019)	Yellowtail (Seriola quinqueradiata)	Histidine supplementation to fishmeal-based and 15% fishmeal diets	LFMH2 (15% fishmeal; analyzed histidine 1.92%)	LFM (15% fishmeal; analyzed histidine 1.12%)	Plasma glucose (mg/dL)	109.000 ± 6.505*	128.000 ± 6.505*	2	2	Hedges' g (SD derived from pooled SEM; LS variance)	-1.65	-3.92 to 0.62	Lower value with histidine supplementation; 95% CI included 0 (very imprecise estimate)	Not applicable—effect size estimated
Yamamoto et al. (2019)	Yellowtail (Seriola quinqueradiata)	Histidine supplementation to fishmeal-based and 15% fishmeal diets	LFMH2 (15% fishmeal; analyzed histidine 1.92%)	LFM (15% fishmeal; analyzed histidine 1.12%)	Liver taurine (mg/g)	5.670 ± 0.175*	5.990 ± 0.175*	2	2	Hedges' g (SD derived from pooled SEM; LS variance)	-1.03	-3.12 to 1.06	Lower value with histidine supplementation; 95% CI included 0 (very imprecise estimate)	Not applicable—effect size estimated
Yamamoto et al. (2019)	Yellowtail (Seriola quinqueradiata)	Histidine supplementation to fishmeal-based and 15% fishmeal diets	LFMH2 (15% fishmeal; analyzed histidine 1.92%)	LFM (15% fishmeal; analyzed histidine 1.12%)	Liver Glx (mg/g)	1.880 ± 0.079*	1.130 ± 0.079*	2	2	Hedges' g (SD derived from pooled SEM; LS variance)	5.34	1.15 to 9.53	Higher value with histidine supplementation; 95% CI excluded 0	Not applicable—effect size estimated
Yamamoto et al. (2019)	Yellowtail (Seriola quinqueradiata)	Histidine supplementation to fishmeal-based and 15% fishmeal diets	LFMH2 (15% fishmeal; analyzed histidine 1.92%)	LFM (15% fishmeal; analyzed histidine 1.12%)	Liver Gly+Ala (mg/g)	1.000 ± 0.177*	1.320 ± 0.177*	2	2	Hedges' g (SD derived from pooled SEM; LS variance)	-1.02	-3.11 to 1.06	Lower value with histidine supplementation; 95% CI included 0 (very imprecise estimate)	Not applicable—effect size estimated
Yamamoto et al. (2019)	Yellowtail (Seriola quinqueradiata)	Histidine supplementation to fishmeal-based and 15% fishmeal diets	LFMH2 (15% fishmeal; analyzed histidine 1.92%)	LFM (15% fishmeal; analyzed histidine 1.12%)	Liver histidine (mg/g)	0.110 ± 0.040*	0.110 ± 0.040*	2	2	Hedges' g (SD derived from pooled SEM; LS variance)	0.0	-1.96 to 1.96	No observed mean difference; 95% CI included 0 (very imprecise estimate)	Not applicable—effect size estimated
Yamamoto et al. (2019)	Yellowtail (Seriola quinqueradiata)	Histidine supplementation to fishmeal-based and 15% fishmeal diets	LFMH2 (15% fishmeal; analyzed histidine 1.92%)	LFM (15% fishmeal; analyzed histidine 1.12%)	Liver EAA (mg/g)	0.990 ± 0.158*	1.050 ± 0.158*	2	2	Hedges' g (SD derived from pooled SEM; LS variance)	-0.21	-2.18 to 1.75	Lower value with histidine supplementation; 95% CI included 0 (very imprecise estimate)	Not applicable—effect size estimated
Yamamoto et al. (2019)	Yellowtail (Seriola quinqueradiata)	Histidine supplementation to fishmeal-based and 15% fishmeal diets	LFMH2 (15% fishmeal; analyzed histidine 1.92%)	LFM (15% fishmeal; analyzed histidine 1.12%)	Liver NEAA (mg/g)	3.310 ± 0.194*	2.880 ± 0.194*	2	2	Hedges' g (SD derived from pooled SEM; LS variance)	1.25	-0.89 to 3.40	Higher value with histidine supplementation; 95% CI included 0 (very imprecise estimate)	Not applicable—effect size estimated
Yamamoto et al. (2019)	Yellowtail (Seriola quinqueradiata)	Histidine supplementation to fishmeal-based and 15% fishmeal diets	LFMH2 (15% fishmeal; analyzed histidine 1.92%)	LFM (15% fishmeal; analyzed histidine 1.12%)	Muscle taurine (mg/g)	3.960 ± 0.164*	5.250 ± 0.164*	2	2	Hedges' g (SD derived from pooled SEM; LS variance)	-4.44	-8.08 to -0.79	Lower muscle taurine with supplementation; 95% CI excluded 0	Not applicable—effect size estimated
Yamamoto et al. (2019)	Yellowtail (Seriola quinqueradiata)	Histidine supplementation to fishmeal-based and 15% fishmeal diets	LFMH2 (15% fishmeal; analyzed histidine 1.92%)	LFM (15% fishmeal; analyzed histidine 1.12%)	Muscle Glx (mg/g)	0.450 ± 0.065*	0.400 ± 0.065*	2	2	Hedges' g (SD derived from pooled SEM; LS variance)	0.43	-1.55 to 2.42	Higher value with histidine supplementation; 95% CI included 0 (very imprecise estimate)	Not applicable—effect size estimated
Yamamoto et al. (2019)	Yellowtail (Seriola quinqueradiata)	Histidine supplementation to fishmeal-based and 15% fishmeal diets	LFMH2 (15% fishmeal; analyzed histidine 1.92%)	LFM (15% fishmeal; analyzed histidine 1.12%)	Muscle Gly+Ala (mg/g)	0.530 ± 0.034*	0.670 ± 0.034*	2	2	Hedges' g (SD derived from pooled SEM; LS variance)	-2.33	-4.87 to 0.21	Lower value with histidine supplementation; 95% CI included 0 (very imprecise estimate)	Not applicable—effect size estimated
Yamamoto et al. (2019)	Yellowtail (Seriola quinqueradiata)	Histidine supplementation to fishmeal-based and 15% fishmeal diets	LFMH2 (15% fishmeal; analyzed histidine 1.92%)	LFM (15% fishmeal; analyzed histidine 1.12%)	Muscle histidine (mg/g)	6.830 ± 0.168*	3.930 ± 0.168*	2	2	Hedges' g (SD derived from pooled SEM; LS variance)	9.72	2.71 to 16.74	Higher muscle free histidine with supplementation; 95% CI excluded 0	Not applicable—effect size estimated
Yamamoto et al. (2019)	Yellowtail (Seriola quinqueradiata)	Histidine supplementation to fishmeal-based and 15% fishmeal diets	LFMH2 (15% fishmeal; analyzed histidine 1.92%)	LFM (15% fishmeal; analyzed histidine 1.12%)	Muscle EAA excluding histidine (mg/g)	0.510 ± 0.025*	0.580 ± 0.025*	2	2	Hedges' g (SD derived from pooled SEM; LS variance)	-1.55	-3.79 to 0.68	Lower value with histidine supplementation; 95% CI included 0 (very imprecise estimate)	Not applicable—effect size estimated
Yamamoto et al. (2019)	Yellowtail (Seriola quinqueradiata)	Histidine supplementation to fishmeal-based and 15% fishmeal diets	LFMH2 (15% fishmeal; analyzed histidine 1.92%)	LFM (15% fishmeal; analyzed histidine 1.12%)	Muscle NEAA (mg/g)	1.240 ± 0.099*	1.400 ± 0.099*	2	2	Hedges' g (SD derived from pooled SEM; LS variance)	-0.91	-2.97 to 1.15	Lower value with histidine supplementation; 95% CI included 0 (very imprecise estimate)	Not applicable—effect size estimated

Notes. Means were reported with pooled SEM. SDs in this table were derived using SD = SEM × sqrt(2), because each diet had two independent replicate tanks. Hedges' g retains the natural direction of each outcome. Positive values therefore indicate a higher value in the histidine-supplemented group and do not necessarily indicate a beneficial biological response. Estimates are highly uncertain because n = 2 tanks per group.

Study 6. Ramos-Pinto et al. (2019): additional tryptophan in an amino-acid-balanced, fishmeal-free diet

A. Growth, feed utilization, and whole-body composition

Study	Species	Strategy	Intervention	Comparator	Outcome and unit	Intervention mean ± SD	Comparator mean ± SD	n intervention	n comparator	Effect-size metric	Effect size	95% CI	Interpretation	Reason not estimated
Ramos-Pinto et al. (2019)	Gilthead seabream (Sparus aurata)	Additional dietary tryptophan in an amino-acid-balanced, fishmeal-free diet	SUP (0% fishmeal; additional tryptophan)	CTRL (0% fishmeal; amino-acid-balanced basal diet)	Final body weight (g) — 15 days	Not reported numerically in supplied preview	Not reported numerically in supplied preview	3 tanks	3 tanks	Not estimable	Not estimable	Not estimable	The original article reported no significant difference between SUP and CTRL at this sampling time	The supplied PDF is an article preview and does not contain the exact treatment means and variance estimates required for a study-level effect-size calculation
Ramos-Pinto et al. (2019)	Gilthead seabream (Sparus aurata)	Additional dietary tryptophan in an amino-acid-balanced, fishmeal-free diet	SUP (0% fishmeal; additional tryptophan)	CTRL (0% fishmeal; amino-acid-balanced basal diet)	Daily growth index — 15 days	Not reported numerically in supplied preview	Not reported numerically in supplied preview	3 tanks	3 tanks	Not estimable	Not estimable	Not estimable	The original article reported no significant difference between SUP and CTRL at this sampling time	The supplied PDF is an article preview and does not contain the exact treatment means and variance estimates required for a study-level effect-size calculation
Ramos-Pinto et al. (2019)	Gilthead seabream (Sparus aurata)	Additional dietary tryptophan in an amino-acid-balanced, fishmeal-free diet	SUP (0% fishmeal; additional tryptophan)	CTRL (0% fishmeal; amino-acid-balanced basal diet)	Relative growth rate — 15 days	Not reported numerically in supplied preview	Not reported numerically in supplied preview	3 tanks	3 tanks	Not estimable	Not estimable	Not estimable	The original article reported no significant difference between SUP and CTRL at this sampling time	The supplied PDF is an article preview and does not contain the exact treatment means and variance estimates required for a study-level effect-size calculation
Ramos-Pinto et al. (2019)	Gilthead seabream (Sparus aurata)	Additional dietary tryptophan in an amino-acid-balanced, fishmeal-free diet	SUP (0% fishmeal; additional tryptophan)	CTRL (0% fishmeal; amino-acid-balanced basal diet)	Voluntary feed intake — 15 days	Not reported numerically in supplied preview	Not reported numerically in supplied preview	3 tanks	3 tanks	Not estimable	Not estimable	Not estimable	The original article reported no significant difference between SUP and CTRL at this sampling time	The supplied PDF is an article preview and does not contain the exact treatment means and variance estimates required for a study-level effect-size calculation
Ramos-Pinto et al. (2019)	Gilthead seabream (Sparus aurata)	Additional dietary tryptophan in an amino-acid-balanced, fishmeal-free diet	SUP (0% fishmeal; additional tryptophan)	CTRL (0% fishmeal; amino-acid-balanced basal diet)	Final body weight (g) — 92 days	Not reported numerically in supplied preview	Not reported numerically in supplied preview	3 tanks	3 tanks	Not estimable	Not estimable	Not estimable	The original article reported no significant difference between SUP and CTRL at this sampling time	The supplied PDF is an article preview and does not contain the exact treatment means and variance estimates required for a study-level effect-size calculation
Ramos-Pinto et al. (2019)	Gilthead seabream (Sparus aurata)	Additional dietary tryptophan in an amino-acid-balanced, fishmeal-free diet	SUP (0% fishmeal; additional tryptophan)	CTRL (0% fishmeal; amino-acid-balanced basal diet)	Daily growth index — 92 days	Not reported numerically in supplied preview	Not reported numerically in supplied preview	3 tanks	3 tanks	Not estimable	Not estimable	Not estimable	The original article reported no significant difference between SUP and CTRL at this sampling time	The supplied PDF is an article preview and does not contain the exact treatment means and variance estimates required for a study-level effect-size calculation
Ramos-Pinto et al. (2019)	Gilthead seabream (Sparus aurata)	Additional dietary tryptophan in an amino-acid-balanced, fishmeal-free diet	SUP (0% fishmeal; additional tryptophan)	CTRL (0% fishmeal; amino-acid-balanced basal diet)	Relative growth rate — 92 days	Not reported numerically in supplied preview	Not reported numerically in supplied preview	3 tanks	3 tanks	Not estimable	Not estimable	Not estimable	The original article reported no significant difference between SUP and CTRL at this sampling time	The supplied PDF is an article preview and does not contain the exact treatment means and variance estimates required for a study-level effect-size calculation
Ramos-Pinto et al. (2019)	Gilthead seabream (Sparus aurata)	Additional dietary tryptophan in an amino-acid-balanced, fishmeal-free diet	SUP (0% fishmeal; additional tryptophan)	CTRL (0% fishmeal; amino-acid-balanced basal diet)	Voluntary feed intake — 92 days	Not reported numerically in supplied preview	Not reported numerically in supplied preview	3 tanks	3 tanks	Not estimable	Not estimable	Not estimable	The original article reported no significant difference between SUP and CTRL at this sampling time	The supplied PDF is an article preview and does not contain the exact treatment means and variance estimates required for a study-level effect-size calculation
Ramos-Pinto et al. (2019)	Gilthead seabream (Sparus aurata)	Additional dietary tryptophan in an amino-acid-balanced, fishmeal-free diet	SUP (0% fishmeal; additional tryptophan)	CTRL (0% fishmeal; amino-acid-balanced basal diet)	Feed conversion ratio — 15 days	Not reported numerically in supplied preview	Not reported numerically in supplied preview	3 tanks	3 tanks	Not estimable	Not estimable	Not estimable	SUP showed a tendency toward a higher, less favorable FCR than CTRL; exact magnitude was unavailable	The supplied PDF is an article preview and does not contain the exact treatment means and variance estimates required for a study-level effect-size calculation
Ramos-Pinto et al. (2019)	Gilthead seabream (Sparus aurata)	Additional dietary tryptophan in an amino-acid-balanced, fishmeal-free diet	SUP (0% fishmeal; additional tryptophan)	CTRL (0% fishmeal; amino-acid-balanced basal diet)	Feed conversion ratio — 92 days	Not reported numerically in supplied preview	Not reported numerically in supplied preview	3 tanks	3 tanks	Not estimable	Not estimable	Not estimable	The short-term tendency toward a higher FCR in SUP was no longer observed	The supplied PDF is an article preview and does not contain the exact treatment means and variance estimates required for a study-level effect-size calculation
Ramos-Pinto et al. (2019)	Gilthead seabream (Sparus aurata)	Additional dietary tryptophan in an amino-acid-balanced, fishmeal-free diet	SUP (0% fishmeal; additional tryptophan)	CTRL (0% fishmeal; amino-acid-balanced basal diet)	Whole-body moisture (% wet weight) — 15 days	Not reported numerically in supplied preview	Not reported numerically in supplied preview	3 tanks	3 tanks	Not estimable	Not estimable	Not estimable	Outcome was assessed, but the numerical treatment result was unavailable in the supplied preview	The supplied PDF is an article preview and does not contain the exact treatment means and variance estimates required for a study-level effect-size calculation
Ramos-Pinto et al. (2019)	Gilthead seabream (Sparus aurata)	Additional dietary tryptophan in an amino-acid-balanced, fishmeal-free diet	SUP (0% fishmeal; additional tryptophan)	CTRL (0% fishmeal; amino-acid-balanced basal diet)	Whole-body crude protein (% wet weight) — 15 days	Not reported numerically in supplied preview	Not reported numerically in supplied preview	3 tanks	3 tanks	Not estimable	Not estimable	Not estimable	Outcome was assessed, but the numerical treatment result was unavailable in the supplied preview	The supplied PDF is an article preview and does not contain the exact treatment means and variance estimates required for a study-level effect-size calculation
Ramos-Pinto et al. (2019)	Gilthead seabream (Sparus aurata)	Additional dietary tryptophan in an amino-acid-balanced, fishmeal-free diet	SUP (0% fishmeal; additional tryptophan)	CTRL (0% fishmeal; amino-acid-balanced basal diet)	Whole-body crude lipid (% wet weight) — 15 days	Not reported numerically in supplied preview	Not reported numerically in supplied preview	3 tanks	3 tanks	Not estimable	Not estimable	Not estimable	Outcome was assessed, but the numerical treatment result was unavailable in the supplied preview	The supplied PDF is an article preview and does not contain the exact treatment means and variance estimates required for a study-level effect-size calculation
Ramos-Pinto et al. (2019)	Gilthead seabream (Sparus aurata)	Additional dietary tryptophan in an amino-acid-balanced, fishmeal-free diet	SUP (0% fishmeal; additional tryptophan)	CTRL (0% fishmeal; amino-acid-balanced basal diet)	Whole-body ash (% wet weight) — 15 days	Not reported numerically in supplied preview	Not reported numerically in supplied preview	3 tanks	3 tanks	Not estimable	Not estimable	Not estimable	Outcome was assessed, but the numerical treatment result was unavailable in the supplied preview	The supplied PDF is an article preview and does not contain the exact treatment means and variance estimates required for a study-level effect-size calculation
Ramos-Pinto et al. (2019)	Gilthead seabream (Sparus aurata)	Additional dietary tryptophan in an amino-acid-balanced, fishmeal-free diet	SUP (0% fishmeal; additional tryptophan)	CTRL (0% fishmeal; amino-acid-balanced basal diet)	Whole-body moisture (% wet weight) — 92 days	Not reported numerically in supplied preview	Not reported numerically in supplied preview	3 tanks	3 tanks	Not estimable	Not estimable	Not estimable	Outcome was assessed, but the numerical treatment result was unavailable in the supplied preview	The supplied PDF is an article preview and does not contain the exact treatment means and variance estimates required for a study-level effect-size calculation
Ramos-Pinto et al. (2019)	Gilthead seabream (Sparus aurata)	Additional dietary tryptophan in an amino-acid-balanced, fishmeal-free diet	SUP (0% fishmeal; additional tryptophan)	CTRL (0% fishmeal; amino-acid-balanced basal diet)	Whole-body crude protein (% wet weight) — 92 days	Not reported numerically in supplied preview	Not reported numerically in supplied preview	3 tanks	3 tanks	Not estimable	Not estimable	Not estimable	Outcome was assessed, but the numerical treatment result was unavailable in the supplied preview	The supplied PDF is an article preview and does not contain the exact treatment means and variance estimates required for a study-level effect-size calculation
Ramos-Pinto et al. (2019)	Gilthead seabream (Sparus aurata)	Additional dietary tryptophan in an amino-acid-balanced, fishmeal-free diet	SUP (0% fishmeal; additional tryptophan)	CTRL (0% fishmeal; amino-acid-balanced basal diet)	Whole-body crude lipid (% wet weight) — 92 days	Not reported numerically in supplied preview	Not reported numerically in supplied preview	3 tanks	3 tanks	Not estimable	Not estimable	Not estimable	Outcome was assessed, but the numerical treatment result was unavailable in the supplied preview	The supplied PDF is an article preview and does not contain the exact treatment means and variance estimates required for a study-level effect-size calculation

B. Remaining composition, hematological, and plasma humoral outcomes

Study	Species	Strategy	Intervention	Comparator	Outcome and unit	Intervention mean ± SD	Comparator mean ± SD	n intervention	n comparator	Effect-size metric	Effect size	95% CI	Interpretation	Reason not estimated
Ramos-Pinto et al. (2019)	Gilthead seabream (Sparus aurata)	Additional dietary tryptophan in an amino-acid-balanced, fishmeal-free diet	SUP (0% fishmeal; additional tryptophan)	CTRL (0% fishmeal; amino-acid-balanced basal diet)	Whole-body ash (% wet weight) — 92 days	Not reported numerically in supplied preview	Not reported numerically in supplied preview	3 tanks	3 tanks	Not estimable	Not estimable	Not estimable	Outcome was assessed, but the numerical treatment result was unavailable in the supplied preview	The supplied PDF is an article preview and does not contain the exact treatment means and variance estimates required for a study-level effect-size calculation
Ramos-Pinto et al. (2019)	Gilthead seabream (Sparus aurata)	Additional dietary tryptophan in an amino-acid-balanced, fishmeal-free diet	SUP (0% fishmeal; additional tryptophan)	CTRL (0% fishmeal; amino-acid-balanced basal diet)	Total white blood cell count — 15 days	Not reported numerically in supplied preview	Not reported numerically in supplied preview	3 tanks; analysis-level n unclear	3 tanks; analysis-level n unclear	Not estimable	Not estimable	Not estimable	Hematological outcome was assessed, but exact group values and the complete result were unavailable in the supplied preview	The supplied PDF is an article preview and does not contain the exact treatment means and variance estimates required for a study-level effect-size calculation
Ramos-Pinto et al. (2019)	Gilthead seabream (Sparus aurata)	Additional dietary tryptophan in an amino-acid-balanced, fishmeal-free diet	SUP (0% fishmeal; additional tryptophan)	CTRL (0% fishmeal; amino-acid-balanced basal diet)	Total red blood cell count — 15 days	Not reported numerically in supplied preview	Not reported numerically in supplied preview	3 tanks; analysis-level n unclear	3 tanks; analysis-level n unclear	Not estimable	Not estimable	Not estimable	Hematological outcome was assessed, but exact group values and the complete result were unavailable in the supplied preview	The supplied PDF is an article preview and does not contain the exact treatment means and variance estimates required for a study-level effect-size calculation

Study	Species	Strategy	Intervention	Comparator	Outcome and unit	Intervention mean ± SD	Comparator mean ± SD	n intervention	n comparator	Effect-size metric	Effect size	95% CI	Interpretation	Reason not estimated
Ramos-Pinto et al. (2019)	Gilthead seabream (Sparus aurata)	Additional dietary tryptophan in an amino-acid-balanced, fishmeal-free diet	SUP (0% fishmeal; additional tryptophan)	CTRL (0% fishmeal; amino-acid-balanced basal diet)	Hemoglobin — 15 days	Not reported numerically in supplied preview	Not reported numerically in supplied preview	3 tanks; analysis-level n unclear	3 tanks; analysis-level n unclear	Not estimable	Not estimable	Not estimable	Hematological outcome was assessed, but exact group values and the complete result were unavailable in the supplied preview	The supplied PDF is an article preview and does not contain the exact treatment means and variance estimates required for a study-level effect-size calculation
Ramos-Pinto et al. (2019)	Gilthead seabream (Sparus aurata)	Additional dietary tryptophan in an amino-acid-balanced, fishmeal-free diet	SUP (0% fishmeal; additional tryptophan)	CTRL (0% fishmeal; amino-acid-balanced basal diet)	Total white blood cell count — 92 days	Not reported numerically in supplied preview	Not reported numerically in supplied preview	3 tanks; analysis-level n unclear	3 tanks; analysis-level n unclear	Not estimable	Not estimable	Not estimable	Hematological outcome was assessed, but exact group values and the complete result were unavailable in the supplied preview	The supplied PDF is an article preview and does not contain the exact treatment means and variance estimates required for a study-level effect-size calculation
Ramos-Pinto et al. (2019)	Gilthead seabream (Sparus aurata)	Additional dietary tryptophan in an amino-acid-balanced, fishmeal-free diet	SUP (0% fishmeal; additional tryptophan)	CTRL (0% fishmeal; amino-acid-balanced basal diet)	Total red blood cell count — 92 days	Not reported numerically in supplied preview	Not reported numerically in supplied preview	3 tanks; analysis-level n unclear	3 tanks; analysis-level n unclear	Not estimable	Not estimable	Not estimable	Hematological outcome was assessed, but exact group values and the complete result were unavailable in the supplied preview	The supplied PDF is an article preview and does not contain the exact treatment means and variance estimates required for a study-level effect-size calculation
Ramos-Pinto et al. (2019)	Gilthead seabream (Sparus aurata)	Additional dietary tryptophan in an amino-acid-balanced, fishmeal-free diet	SUP (0% fishmeal; additional tryptophan)	CTRL (0% fishmeal; amino-acid-balanced basal diet)	Hemoglobin — 92 days	Not reported numerically in supplied preview	Not reported numerically in supplied preview	3 tanks; analysis-level n unclear	3 tanks; analysis-level n unclear	Not estimable	Not estimable	Not estimable	Hematological outcome was assessed, but exact group values and the complete result were unavailable in the supplied preview	The supplied PDF is an article preview and does not contain the exact treatment means and variance estimates required for a study-level effect-size calculation
Ramos-Pinto et al. (2019)	Gilthead seabream (Sparus aurata)	Additional dietary tryptophan in an amino-acid-balanced, fishmeal-free diet	SUP (0% fishmeal; additional tryptophan)	CTRL (0% fishmeal; amino-acid-balanced basal diet)	Plasma antiprotease activity — 15 days	Not reported numerically in supplied preview	Not reported numerically in supplied preview	3 tanks; analysis-level n unclear	3 tanks; analysis-level n unclear	Not estimable	Not estimable	Not estimable	No major diet-related difference was reported	The supplied PDF is an article preview and does not contain the exact treatment means and variance estimates required for a study-level effect-size calculation
Ramos-Pinto et al. (2019)	Gilthead seabream (Sparus aurata)	Additional dietary tryptophan in an amino-acid-balanced, fishmeal-free diet	SUP (0% fishmeal; additional tryptophan)	CTRL (0% fishmeal; amino-acid-balanced basal diet)	Plasma peroxidase activity — 15 days	Not reported numerically in supplied preview	Not reported numerically in supplied preview	3 tanks; analysis-level n unclear	3 tanks; analysis-level n unclear	Not estimable	Not estimable	Not estimable	No major diet-related difference was reported	The supplied PDF is an article preview and does not contain the exact treatment means and variance estimates required for a study-level effect-size calculation
Ramos-Pinto et al. (2019)	Gilthead seabream (Sparus aurata)	Additional dietary tryptophan in an amino-acid-balanced, fishmeal-free diet	SUP (0% fishmeal; additional tryptophan)	CTRL (0% fishmeal; amino-acid-balanced basal diet)	Plasma antiprotease activity — 92 days	Not reported numerically in supplied preview	Not reported numerically in supplied preview	3 tanks; analysis-level n unclear	3 tanks; analysis-level n unclear	Not estimable	Not estimable	Not estimable	No major diet-related difference was reported; activity increased at 92 days regardless of dietary treatment	The supplied PDF is an article preview and does not contain the exact treatment means and variance estimates required for a study-level effect-size calculation
Ramos-Pinto et al. (2019)	Gilthead seabream (Sparus aurata)	Additional dietary tryptophan in an amino-acid-balanced, fishmeal-free diet	SUP (0% fishmeal; additional tryptophan)	CTRL (0% fishmeal; amino-acid-balanced basal diet)	Plasma peroxidase activity — 92 days	Not reported numerically in supplied preview	Not reported numerically in supplied preview	3 tanks; analysis-level n unclear	3 tanks; analysis-level n unclear	Not estimable	Not estimable	Not estimable	No major diet-related difference was reported; activity increased at 92 days regardless of dietary treatment	The supplied PDF is an article preview and does not contain the exact treatment means and variance estimates required for a study-level effect-size calculation

C. Tissue multigene-expression profiles

Study	Species	Strategy	Intervention	Comparator	Outcome and unit	Intervention mean ± SD	Comparator mean ± SD	n intervention	n comparator	Effect-size metric	Effect size	95% CI	Interpretation	Reason not estimated
Ramos-Pinto et al. (2019)	Gilthead seabream (Sparus aurata)	Additional dietary tryptophan in an amino-acid-balanced, fishmeal-free diet	SUP (0% fishmeal; additional tryptophan)	CTRL (0% fishmeal; amino-acid-balanced basal diet)	White skeletal muscle multigene-expression profile — 15 days	Not reported numerically in supplied preview	Not reported numerically in supplied preview	3 tanks; tissue-sampling n unclear	3 tanks; tissue-sampling n unclear	Not estimable	Not estimable	Not estimable	Diet-related transcriptional modulation was detected during short-term feeding; the head-kidney profile was consistent with a transient immunostimulated state in SUP fish	The supplied PDF is an article preview and does not contain the exact treatment means and variance estimates required for a study-level effect-size calculation
Ramos-Pinto et al. (2019)	Gilthead seabream (Sparus aurata)	Additional dietary tryptophan in an amino-acid-balanced, fishmeal-free diet	SUP (0% fishmeal; additional tryptophan)	CTRL (0% fishmeal; amino-acid-balanced basal diet)	White skeletal muscle multigene-expression profile — 92 days	Not reported numerically in supplied preview	Not reported numerically in supplied preview	3 tanks; tissue-sampling n unclear	3 tanks; tissue-sampling n unclear	Not estimable	Not estimable	Not estimable	The short-term diet-related transcriptional effects were largely attenuated or absent after long-term feeding	The supplied PDF is an article preview and does not contain the exact treatment means and variance estimates required for a study-level effect-size calculation
Ramos-Pinto et al. (2019)	Gilthead seabream (Sparus aurata)	Additional dietary tryptophan in an amino-acid-balanced, fishmeal-free diet	SUP (0% fishmeal; additional tryptophan)	CTRL (0% fishmeal; amino-acid-balanced basal diet)	Liver multigene-expression profile — 15 days	Not reported numerically in supplied preview	Not reported numerically in supplied preview	3 tanks; tissue-sampling n unclear	3 tanks; tissue-sampling n unclear	Not estimable	Not estimable	Not estimable	Diet-related transcriptional modulation was detected during short-term feeding; the head-kidney profile was consistent with a transient immunostimulated state in SUP fish	The supplied PDF is an article preview and does not contain the exact treatment means and variance estimates required for a study-level effect-size calculation
Ramos-Pinto et al. (2019)	Gilthead seabream (Sparus aurata)	Additional dietary tryptophan in an amino-acid-balanced, fishmeal-free diet	SUP (0% fishmeal; additional tryptophan)	CTRL (0% fishmeal; amino-acid-balanced basal diet)	Liver multigene-expression profile — 92 days	Not reported numerically in supplied preview	Not reported numerically in supplied preview	3 tanks; tissue-sampling n unclear	3 tanks; tissue-sampling n unclear	Not estimable	Not estimable	Not estimable	The short-term diet-related transcriptional effects were largely attenuated or absent after long-term feeding	The supplied PDF is an article preview and does not contain the exact treatment means and variance estimates required for a study-level effect-size calculation
Ramos-Pinto et al. (2019)	Gilthead seabream (Sparus aurata)	Additional dietary tryptophan in an amino-acid-balanced, fishmeal-free diet	SUP (0% fishmeal; additional tryptophan)	CTRL (0% fishmeal; amino-acid-balanced basal diet)	Head-kidney multigene-expression profile — 15 days	Not reported numerically in supplied preview	Not reported numerically in supplied preview	3 tanks; tissue-sampling n unclear	3 tanks; tissue-sampling n unclear	Not estimable	Not estimable	Not estimable	Diet-related transcriptional modulation was detected during short-term feeding; the head-kidney profile was consistent with a transient immunostimulated state in SUP fish	The supplied PDF is an article preview and does not contain the exact treatment means and variance estimates required for a study-level effect-size calculation
Ramos-Pinto et al. (2019)	Gilthead seabream (Sparus aurata)	Additional dietary tryptophan in an amino-acid-balanced, fishmeal-free diet	SUP (0% fishmeal; additional tryptophan)	CTRL (0% fishmeal; amino-acid-balanced basal diet)	Head-kidney multigene-expression profile — 92 days	Not reported numerically in supplied preview	Not reported numerically in supplied preview	3 tanks; tissue-sampling n unclear	3 tanks; tissue-sampling n unclear	Not estimable	Not estimable	Not estimable	The short-term diet-related transcriptional effects were largely attenuated or absent after long-term feeding	The supplied PDF is an article preview and does not contain the exact treatment means and variance estimates required for a study-level effect-size calculation

Notes. CTRL and SUP were both fishmeal-free diets; SUP contained additional tryptophan. Triplicate tanks were reported for each diet. Exact treatment means and tank-level variance were absent from the supplied preview, so no Hedges' g or 95% CI was calculated. These rows must be updated from the complete article before final submission of Supplementary Table S3.

Study 7. Ramos-Pinto et al. (2021): histidine, taurine, or threonine supplementation in a practical low-fishmeal diet

A. Growth and survival outcomes

Study	Species	Strategy	Intervention	Comparator	Outcome and unit	Intervention mean ± SD	Comparator mean ± SD	n intervention	n comparator	Effect-size metric	Effect size	95% CI	Interpretation	Reason not estimated
Ramos-Pinto et al. (2021)	Gilthead seabream (Sparus aurata)	Histidine, taurine, or threonine supplementation in a 12% fishmeal practical diet	THR (0.75% added threonine)	CTRL (same basal diet; no added test amino acid)	Final body weight (g/fish) — 2 weeks	10.89 ± 0.30	11.07 ± 0.23	12 fish nested in 3 tanks	12 fish nested in 3 tanks	Not estimable	Not estimable	Not estimable	Lower value with supplementation	The reported dispersion was based on sampled fish rather than independent tank means; using fish-level n would risk pseudoreplication
Ramos-Pinto et al. (2021)	Gilthead seabream (Sparus aurata)	Histidine, taurine, or threonine supplementation in a 12% fishmeal practical diet	THR (0.75% added threonine)	CTRL (same basal diet; no added test amino acid)	Relative growth rate (%/day) — 2 weeks	1.65 ± 0.51	1.63 ± 0.28	3 tanks	3 tanks	Hedges' g (provisional tank-level interpretation; LS variance)	0.04	-1.56 to 1.64	Higher relative growth rate with supplementation; 95% CI included 0 (imprecise estimate)	Not applicable—effect size estimated
Ramos-Pinto et al. (2021)	Gilthead seabream (Sparus aurata)	Histidine, taurine, or threonine supplementation in a 12% fishmeal practical diet	THR (0.75% added threonine)	CTRL (same basal diet; no added test amino acid)	Final body weight (g/fish) — 4 weeks	14.04 ± 1.49	14.49 ± 2.10	12 fish nested in 3 tanks	12 fish nested in 3 tanks	Not estimable	Not estimable	Not estimable	Lower value with supplementation	The reported dispersion was based on sampled fish rather than independent tank means; using fish-level n would risk pseudoreplication
Ramos-Pinto et al. (2021)	Gilthead seabream (Sparus aurata)	Histidine, taurine, or threonine supplementation in a 12% fishmeal practical diet	THR (0.75% added threonine)	CTRL (same basal diet; no added test amino acid)	Relative growth rate (%/day) — 4 weeks	1.73 ± 0.35	1.76 ± 0.50	3 tanks	3 tanks	Hedges' g (provisional tank-level interpretation; LS variance)	-0.06	-1.66 to 1.55	Lower relative growth rate with supplementation; 95% CI included 0 (imprecise estimate)	Not applicable—effect size estimated
Ramos-Pinto et al. (2021)	Gilthead seabream (Sparus aurata)	Histidine, taurine, or threonine supplementation in a 12% fishmeal practical diet	TAU (0.50% added taurine)	CTRL (same basal diet; no added test amino acid)	Final body weight (g/fish) — 2 weeks	11.08 ± 0.33	11.07 ± 0.23	12 fish nested in 3 tanks	12 fish nested in 3 tanks	Not estimable	Not estimable	Not estimable	Higher value with supplementation	The reported dispersion was based on sampled fish rather than independent tank means; using fish-level n would risk pseudoreplication
Ramos-Pinto et al. (2021)	Gilthead seabream (Sparus aurata)	Histidine, taurine, or threonine supplementation in a 12% fishmeal practical diet	TAU (0.50% added taurine)	CTRL (same basal diet; no added test amino acid)	Relative growth rate (%/day) — 2 weeks	1.54 ± 0.18	1.63 ± 0.28	3 tanks	3 tanks	Hedges' g (provisional tank-level interpretation; LS variance)	-0.31	-1.91 to 1.30	Lower relative growth rate with supplementation; 95% CI included 0 (imprecise estimate)	Not applicable—effect size estimated
Ramos-Pinto et al. (2021)	Gilthead seabream (Sparus aurata)	Histidine, taurine, or threonine supplementation in a 12% fishmeal practical diet	TAU (0.50% added taurine)	CTRL (same basal diet; no added test amino acid)	Final body weight (g/fish) — 4 weeks	13.89 ± 1.08	14.49 ± 2.10	12 fish nested in 3 tanks	12 fish nested in 3 tanks	Not estimable	Not estimable	Not estimable	Lower value with supplementation	The reported dispersion was based on sampled fish rather than independent tank means; using fish-level n would risk pseudoreplication
Ramos-Pinto et al. (2021)	Gilthead seabream (Sparus aurata)	Histidine, taurine, or threonine supplementation in a 12% fishmeal practical diet	TAU (0.50% added taurine)	CTRL (same basal diet; no added test amino acid)	Relative growth rate (%/day) — 4 weeks	1.58 ± 0.32	1.76 ± 0.50	3 tanks	3 tanks	Hedges' g (provisional tank-level interpretation; LS variance)	-0.34	-1.95 to 1.27	Lower relative growth rate with supplementation; 95% CI included 0 (imprecise estimate)	Not applicable—effect size estimated
Ramos-Pinto et al. (2021)	Gilthead seabream (Sparus aurata)	Histidine, taurine, or threonine supplementation in a 12% fishmeal practical diet	HIS (0.40% added histidine)	CTRL (same basal diet; no added test amino acid)	Final body weight (g/fish) — 2 weeks	11.13 ± 0.49	11.07 ± 0.23	12 fish nested in 3 tanks	12 fish nested in 3 tanks	Not estimable	Not estimable	Not estimable	Higher value with supplementation	The reported dispersion was based on sampled fish rather than independent tank means; using fish-level n would risk pseudoreplication
Ramos-Pinto et al. (2021)	Gilthead seabream (Sparus aurata)	Histidine, taurine, or threonine supplementation in a 12% fishmeal practical diet	HIS (0.40% added histidine)	CTRL (same basal diet; no added test amino acid)	Relative growth rate (%/day) — 2 weeks	1.84 ± 0.48	1.63 ± 0.28	3 tanks	3 tanks	Hedges' g (provisional tank-level interpretation; LS variance)	0.43	-1.19 to 2.04	Higher relative growth rate with supplementation; 95% CI included 0 (imprecise estimate)	Not applicable—effect size estimated
Ramos-Pinto et al. (2021)	Gilthead seabream (Sparus aurata)	Histidine, taurine, or threonine supplementation in a 12% fishmeal practical diet	HIS (0.40% added histidine)	CTRL (same basal diet; no added test amino acid)	Final body weight (g/fish) — 4 weeks	13.70 ± 0.10	14.49 ± 2.10	12 fish nested in 3 tanks	12 fish nested in 3 tanks	Not estimable	Not estimable	Not estimable	Lower value with supplementation	The reported dispersion was based on sampled fish rather than independent tank means; using fish-level n would risk pseudoreplication
Ramos-Pinto et al. (2021)	Gilthead seabream (Sparus aurata)	Histidine, taurine, or threonine supplementation in a 12% fishmeal practical diet	HIS (0.40% added histidine)	CTRL (same basal diet; no added test amino acid)	Relative growth rate (%/day) — 4 weeks	1.67 ± 0.09	1.76 ± 0.50	3 tanks	3 tanks	Hedges' g (provisional tank-level interpretation; LS variance)	-0.2	-1.80 to 1.40	Lower relative growth rate with supplementation; 95% CI included 0 (imprecise estimate)	Not applicable—effect size estimated
Ramos-Pinto et al. (2021)	Gilthead seabream (Sparus aurata)	Histidine, taurine, or threonine supplementation in a 12% fishmeal practical diet	THR (0.75% added threonine)	CTRL (same basal diet; no added test amino acid)	Survival over the 4-week trial (%)	100%; SD not reported	100%; SD not reported	3 tanks; 70 fish/tank at stocking	3 tanks; 70 fish/tank at stocking	Not estimable	Not estimable	Not estimable	No deaths were observed in either group	No mortality events occurred in either arm; a conventional risk ratio is undefined
Ramos-Pinto et al. (2021)	Gilthead seabream (Sparus aurata)	Histidine, taurine, or threonine supplementation in a 12% fishmeal practical diet	TAU (0.50% added taurine)	CTRL (same basal diet; no added test amino acid)	Survival over the 4-week trial (%)	100%; SD not reported	100%; SD not reported	3 tanks; 70 fish/tank at stocking	3 tanks; 70 fish/tank at stocking	Not estimable	Not estimable	Not estimable	No deaths were observed in either group	No mortality events occurred in either arm; a conventional risk ratio is undefined
Ramos-Pinto et al. (2021)	Gilthead seabream (Sparus aurata)	Histidine, taurine, or threonine supplementation in a 12% fishmeal practical diet	HIS (0.40% added histidine)	CTRL (same basal diet; no added test amino acid)	Survival over the 4-week trial (%)	100%; SD not reported	100%; SD not reported	3 tanks; 70 fish/tank at stocking	3 tanks; 70 fish/tank at stocking	Not estimable	Not estimable	Not estimable	No deaths were observed in either group	No mortality events occurred in either arm; a conventional risk ratio is undefined

B. Hematological outcomes

Study	Species	Strategy	Intervention	Comparator	Outcome and unit	Intervention mean ± SD	Comparator mean ± SD	n intervention	n comparator	Effect-size metric	Effect size	95% CI	Interpretation	Reason not estimated
Ramos-Pinto et al. (2021)	Gilthead seabream (Sparus aurata)	Histidine, taurine, or threonine supplementation in a 12% fishmeal practical diet	THR (0.75% added threonine)	CTRL (same basal diet; no added test amino acid)	Hemoglobin (g/dL) — 2 weeks	0.650 ± 0.270*	0.600 ± 0.180*	9 fish nested in 3 tanks	9 fish nested in 3 tanks	Not estimable	Not estimable	Not estimable	Higher value with supplementation	SD was derived from fish-level SE, but independent tank-level means and variance were not reported; a tank-level effect size could not be estimated without pseudoreplication
Ramos-Pinto et al. (2021)	Gilthead seabream (Sparus aurata)	Histidine, taurine, or threonine supplementation in a 12% fishmeal practical diet	THR (0.75% added threonine)	CTRL (same basal diet; no added test amino acid)	WBC (×10^4/μL) — 2 weeks	3.340 ± 0.600*	4.530 ± 1.800*	9 fish nested in 3 tanks	9 fish nested in 3 tanks	Not estimable	Not estimable	Not estimable	Lower value with supplementation	SD was derived from fish-level SE, but independent tank-level means and variance were not reported; a tank-level effect size could not be estimated without pseudoreplication
Ramos-Pinto et al. (2021)	Gilthead seabream (Sparus aurata)	Histidine, taurine, or threonine supplementation in a 12% fishmeal practical diet	THR (0.75% added threonine)	CTRL (same basal diet; no added test amino acid)	RBC (×10^6/μL) — 2 weeks	1.280 ± 0.330*	1.140 ± 0.270*	9 fish nested in 3 tanks	9 fish nested in 3 tanks	Not estimable	Not estimable	Not estimable	Higher value with supplementation	SD was derived from fish-level SE, but independent tank-level means and variance were not reported; a tank-level effect size could not be estimated without pseudoreplication
Ramos-Pinto et al. (2021)	Gilthead seabream (Sparus aurata)	Histidine, taurine, or threonine supplementation in a 12% fishmeal practical diet	THR (0.75% added threonine)	CTRL (same basal diet; no added test amino acid)	Thrombocytes (×10^4/μL) — 2 weeks	2.460 ± 0.540*	3.460 ± 1.710*	9 fish nested in 3 tanks	9 fish nested in 3 tanks	Not estimable	Not estimable	Not estimable	Lower value with supplementation	SD was derived from fish-level SE, but independent tank-level means and variance were not reported; a tank-level effect size could not be estimated without pseudoreplication
Ramos-Pinto et al. (2021)	Gilthead seabream (Sparus aurata)	Histidine, taurine, or threonine supplementation in a 12% fishmeal practical diet	THR (0.75% added threonine)	CTRL (same basal diet; no added test amino acid)	Lymphocytes (×10^4/μL) — 2 weeks	0.700 ± 1.020*	0.930 ± 0.420*	9 fish nested in 3 tanks	9 fish nested in 3 tanks	Not estimable	Not estimable	Not estimable	Lower value with supplementation	SD was derived from fish-level SE, but independent tank-level means and variance were not reported; a tank-level effect size could not be estimated without

Study	Species	Strategy	Intervention	Comparator	Outcome and unit	Intervention mean ± SD	Comparator mean ± SD	n intervention	n comparator	Effect-size metric	Effect size	95% CI	Interpretation	Reason not estimated
														pseudoreplication
Ramos-Pinto et al. (2021)	Gilthead seabream (Sparus aurata)	Histidine, taurine, or threonine supplementation in a 12% fishmeal practical diet	THR (0.75% added threonine)	CTRL (same basal diet; no added test amino acid)	Monocytes (×10^4/µL) — 2 weeks	0.020 ± 0.030*	0.020 ± 0.030*	9 fish nested in 3 tanks	9 fish nested in 3 tanks	Not estimable	Not estimable	Not estimable	No observed mean difference	SD was derived from fish-level SE, but independent tank-level means and variance were not reported; a tank-level effect size could not be estimated without pseudoreplication
Ramos-Pinto et al. (2021)	Gilthead seabream (Sparus aurata)	Histidine, taurine, or threonine supplementation in a 12% fishmeal practical diet	THR (0.75% added threonine)	CTRL (same basal diet; no added test amino acid)	Neutrophils (×10^4/µL) — 2 weeks	0.080 ± 0.090*	0.070 ± 0.060*	9 fish nested in 3 tanks	9 fish nested in 3 tanks	Not estimable	Not estimable	Not estimable	Higher value with supplementation	SD was derived from fish-level SE, but independent tank-level means and variance were not reported; a tank-level effect size could not be estimated without pseudoreplication
Ramos-Pinto et al. (2021)	Gilthead seabream (Sparus aurata)	Histidine, taurine, or threonine supplementation in a 12% fishmeal practical diet	THR (0.75% added threonine)	CTRL (same basal diet; no added test amino acid)	Hemoglobin (g/dL) — 4 weeks	0.650 ± 0.120*	0.600 ± 0.150*	9 fish nested in 3 tanks	9 fish nested in 3 tanks	Not estimable	Not estimable	Not estimable	Higher value with supplementation	SD was derived from fish-level SE, but independent tank-level means and variance were not reported; a tank-level effect size could not be estimated without pseudoreplication
Ramos-Pinto et al. (2021)	Gilthead seabream (Sparus aurata)	Histidine, taurine, or threonine supplementation in a 12% fishmeal practical diet	THR (0.75% added threonine)	CTRL (same basal diet; no added test amino acid)	WBC (×10^4/µL) — 4 weeks	7.110 ± 1.680*	6.210 ± 1.080*	9 fish nested in 3 tanks	9 fish nested in 3 tanks	Not estimable	Not estimable	Not estimable	Higher value with supplementation	SD was derived from fish-level SE, but independent tank-level means and variance were not reported; a tank-level effect size could not be estimated without pseudoreplication
Ramos-Pinto et al. (2021)	Gilthead seabream (Sparus aurata)	Histidine, taurine, or threonine supplementation in a 12% fishmeal practical diet	THR (0.75% added threonine)	CTRL (same basal diet; no added test amino acid)	RBC (×10^6/µL) — 4 weeks	1.580 ± 0.120*	1.440 ± 0.300*	9 fish nested in 3 tanks	9 fish nested in 3 tanks	Not estimable	Not estimable	Not estimable	Higher value with supplementation	SD was derived from fish-level SE, but independent tank-level means and variance were not reported; a tank-level effect size could not be estimated without pseudoreplication
Ramos-Pinto et al. (2021)	Gilthead seabream (Sparus aurata)	Histidine, taurine, or threonine supplementation in a 12% fishmeal practical diet	THR (0.75% added threonine)	CTRL (same basal diet; no added test amino acid)	Thrombocytes (×10^4/µL) — 4 weeks	4.770 ± 1.080*	4.540 ± 0.780*	9 fish nested in 3 tanks	9 fish nested in 3 tanks	Not estimable	Not estimable	Not estimable	Higher value with supplementation	SD was derived from fish-level SE, but independent tank-level means and variance were not reported; a tank-level effect size could not be estimated without pseudoreplication
Ramos-Pinto et al. (2021)	Gilthead seabream (Sparus aurata)	Histidine, taurine, or threonine supplementation in a 12% fishmeal practical diet	THR (0.75% added threonine)	CTRL (same basal diet; no added test amino acid)	Lymphocytes (×10^4/µL) — 4 weeks	1.950 ± 1.470*	1.370 ± 0.540*	9 fish nested in 3 tanks	9 fish nested in 3 tanks	Not estimable	Not estimable	Not estimable	Higher value with supplementation	SD was derived from fish-level SE, but independent tank-level means and variance were not reported; a tank-level effect size could not be estimated without pseudoreplication
Ramos-Pinto et al. (2021)	Gilthead seabream (Sparus aurata)	Histidine, taurine, or threonine supplementation in a 12% fishmeal practical diet	THR (0.75% added threonine)	CTRL (same basal diet; no added test amino acid)	Monocytes (×10^4/µL) — 4 weeks	0.060 ± 0.060*	0.060 ± 0.060*	9 fish nested in 3 tanks	9 fish nested in 3 tanks	Not estimable	Not estimable	Not estimable	No observed mean difference	SD was derived from fish-level SE, but independent tank-level means and variance were not reported; a tank-level effect size could not be estimated without pseudoreplication
Ramos-Pinto et al. (2021)	Gilthead seabream (Sparus aurata)	Histidine, taurine, or threonine supplementation in a 12% fishmeal practical diet	THR (0.75% added threonine)	CTRL (same basal diet; no added test amino acid)	Neutrophils (×10^4/µL) — 4 weeks	0.180 ± 0.060*	0.220 ± 0.120*	9 fish nested in 3 tanks	9 fish nested in 3 tanks	Not estimable	Not estimable	Not estimable	Lower value with supplementation	SD was derived from fish-level SE, but independent tank-level means and variance were not reported; a tank-level effect size could not be estimated without pseudoreplication
Ramos-Pinto et al. (2021)	Gilthead seabream (Sparus aurata)	Histidine, taurine, or threonine supplementation in a 12% fishmeal practical diet	TAU (0.50% added taurine)	CTRL (same basal diet; no added test amino acid)	Hemoglobin (g/dL) — 2 weeks	0.570 ± 0.090*	0.600 ± 0.180*	9 fish nested in 3 tanks	9 fish nested in 3 tanks	Not estimable	Not estimable	Not estimable	Lower value with supplementation	SD was derived from fish-level SE, but independent tank-level means and variance were not reported; a tank-level effect size could not be estimated without pseudoreplication
Ramos-Pinto et al. (2021)	Gilthead seabream (Sparus aurata)	Histidine, taurine, or threonine supplementation in a 12% fishmeal practical diet	TAU (0.50% added taurine)	CTRL (same basal diet; no added test amino acid)	WBC (×10^4/µL) — 2 weeks	4.850 ± 0.840*	4.530 ± 1.800*	9 fish nested in 3 tanks	9 fish nested in 3 tanks	Not estimable	Not estimable	Not estimable	Higher value with supplementation	SD was derived from fish-level SE, but independent tank-level means and variance were not reported; a tank-level effect size could not be estimated without pseudoreplication
Ramos-Pinto et al. (2021)	Gilthead seabream (Sparus aurata)	Histidine, taurine, or threonine supplementation in a 12% fishmeal practical diet	TAU (0.50% added taurine)	CTRL (same basal diet; no added test amino acid)	RBC (×10^6/µL) — 2 weeks	1.000 ± 0.180*	1.140 ± 0.270*	9 fish nested in 3 tanks	9 fish nested in 3 tanks	Not estimable	Not estimable	Not estimable	Lower value with supplementation	SD was derived from fish-level SE, but independent tank-level means and variance were not reported; a tank-level effect size could not be estimated without pseudoreplication
Ramos-Pinto et al. (2021)	Gilthead seabream (Sparus aurata)	Histidine, taurine, or threonine supplementation in a 12% fishmeal practical diet	TAU (0.50% added taurine)	CTRL (same basal diet; no added test amino acid)	Thrombocytes (×10^4/µL) — 2 weeks	3.620 ± 0.810*	3.460 ± 1.710*	9 fish nested in 3 tanks	9 fish nested in 3 tanks	Not estimable	Not estimable	Not estimable	Higher value with supplementation	SD was derived from fish-level SE, but independent tank-level means and variance were not reported; a tank-level effect size could not be estimated without pseudoreplication
Ramos-Pinto et al. (2021)	Gilthead seabream (Sparus aurata)	Histidine, taurine, or threonine supplementation in a 12% fishmeal practical diet	TAU (0.50% added taurine)	CTRL (same basal diet; no added test amino acid)	Lymphocytes (×10^4/µL) — 2 weeks	0.980 ± 1.380*	0.930 ± 0.420*	9 fish nested in 3 tanks	9 fish nested in 3 tanks	Not estimable	Not estimable	Not estimable	Higher value with supplementation	SD was derived from fish-level SE, but independent tank-level means and variance were not reported; a tank-level effect size could not be estimated without pseudoreplication
Ramos-Pinto et al. (2021)	Gilthead seabream (Sparus aurata)	Histidine, taurine, or threonine supplementation in a 12% fishmeal practical diet	TAU (0.50% added taurine)	CTRL (same basal diet; no added test amino acid)	Monocytes (×10^4/µL) — 2 weeks	0.040 ± 0.030*	0.020 ± 0.030*	9 fish nested in 3 tanks	9 fish nested in 3 tanks	Not estimable	Not estimable	Not estimable	Higher value with supplementation	SD was derived from fish-level SE, but independent tank-level means and variance were not reported; a tank-level effect size could not be estimated without pseudoreplication
Ramos-Pinto et al. (2021)	Gilthead seabream (Sparus aurata)	Histidine, taurine, or threonine supplementation in a 12% fishmeal practical diet	TAU (0.50% added taurine)	CTRL (same basal diet; no added test amino acid)	Neutrophils (×10^4/µL) — 2 weeks	0.180 ± 0.090*	0.070 ± 0.060*	9 fish nested in 3 tanks	9 fish nested in 3 tanks	Not estimable	Not estimable	Not estimable	Higher value with supplementation	SD was derived from fish-level SE, but independent tank-level means and variance were not reported; a tank-level effect size could not be estimated without pseudoreplication
Ramos-Pinto et al. (2021)	Gilthead seabream (Sparus aurata)	Histidine, taurine, or threonine supplementation in a 12% fishmeal practical diet	TAU (0.50% added taurine)	CTRL (same basal diet; no added test amino acid)	Hemoglobin (g/dL) — 4 weeks	0.560 ± 0.150*	0.600 ± 0.150*	9 fish nested in 3 tanks	9 fish nested in 3 tanks	Not estimable	Not estimable	Not estimable	Lower value with supplementation	SD was derived from fish-level SE, but independent tank-level means and variance were not reported; a tank-level effect size could not be estimated without pseudoreplication
Ramos-Pinto et al. (2021)	Gilthead seabream (Sparus aurata)	Histidine, taurine, or threonine supplementation in a 12% fishmeal practical diet	TAU (0.50% added taurine)	CTRL (same basal diet; no added test amino acid)	WBC (×10^4/µL) — 4 weeks	7.000 ± 1.200*	6.210 ± 1.080*	9 fish nested in 3 tanks	9 fish nested in 3 tanks	Not estimable	Not estimable	Not estimable	Higher value with supplementation	SD was derived from fish-level SE, but independent tank-level means and variance were not reported; a tank-level effect size could not be estimated without pseudoreplication
Ramos-Pinto et al.	Gilthead seabream	Histidine, taurine, or	TAU (0.50% added taurine)	CTRL (same basal diet; no added	RBC (×10^6/µL) — 4	1.420 ± 0.270*	1.440 ± 0.300*	9 fish	9 fish	Not estimable	Not	Not estimable	Lower value with supplementation	SD was derived from fish-level SE, but

Study	Species	Strategy	Intervention	Comparator	Outcome and unit	Intervention mean ± SD	Comparator mean ± SD	n intervention	n comparator	Effect-size metric	Effect size	95% CI	Interpretation	Reason not estimated
(2021)	(Sparus aurata)	threonine supplementation in a 12% fishmeal practical diet		test amino acid)	weeks			nested in 3 tanks	nested in 3 tanks		estimable			independent tank-level means and variance were not reported; a tank-level effect size could not be estimated without pseudoreplication
Ramos-Pinto et al. (2021)	Gilthead seabream (Sparus aurata)	Histidine, taurine, or threonine supplementation in a 12% fishmeal practical diet	TAU (0.50% added taurine)	CTRL (same basal diet; no added test amino acid)	Thrombocytes (×10^4/µL) — 4 weeks	5.190 ± 1.200*	4.540 ± 0.780*	9 fish nested in 3 tanks	9 fish nested in 3 tanks	Not estimable	Not estimable	Not estimable	Higher value with supplementation	SD was derived from fish-level SE, but independent tank-level means and variance were not reported; a tank-level effect size could not be estimated without pseudoreplication
Ramos-Pinto et al. (2021)	Gilthead seabream (Sparus aurata)	Histidine, taurine, or threonine supplementation in a 12% fishmeal practical diet	TAU (0.50% added taurine)	CTRL (same basal diet; no added test amino acid)	Lymphocytes (×10^4/µL) — 4 weeks	1.490 ± 1.440*	1.370 ± 0.540*	9 fish nested in 3 tanks	9 fish nested in 3 tanks	Not estimable	Not estimable	Not estimable	Higher value with supplementation	SD was derived from fish-level SE, but independent tank-level means and variance were not reported; a tank-level effect size could not be estimated without pseudoreplication
Ramos-Pinto et al. (2021)	Gilthead seabream (Sparus aurata)	Histidine, taurine, or threonine supplementation in a 12% fishmeal practical diet	TAU (0.50% added taurine)	CTRL (same basal diet; no added test amino acid)	Monocytes (×10^4/µL) — 4 weeks	0.080 ± 0.060*	0.060 ± 0.060*	9 fish nested in 3 tanks	9 fish nested in 3 tanks	Not estimable	Not estimable	Not estimable	Higher value with supplementation	SD was derived from fish-level SE, but independent tank-level means and variance were not reported; a tank-level effect size could not be estimated without pseudoreplication
Ramos-Pinto et al. (2021)	Gilthead seabream (Sparus aurata)	Histidine, taurine, or threonine supplementation in a 12% fishmeal practical diet	TAU (0.50% added taurine)	CTRL (same basal diet; no added test amino acid)	Neutrophils (×10^4/µL) — 4 weeks	0.250 ± 0.060*	0.220 ± 0.120*	9 fish nested in 3 tanks	9 fish nested in 3 tanks	Not estimable	Not estimable	Not estimable	Higher value with supplementation	SD was derived from fish-level SE, but independent tank-level means and variance were not reported; a tank-level effect size could not be estimated without pseudoreplication
Ramos-Pinto et al. (2021)	Gilthead seabream (Sparus aurata)	Histidine, taurine, or threonine supplementation in a 12% fishmeal practical diet	HIS (0.40% added histidine)	CTRL (same basal diet; no added test amino acid)	Hemoglobin (g/dL) — 2 weeks	0.540 ± 0.180*	0.600 ± 0.180*	9 fish nested in 3 tanks	9 fish nested in 3 tanks	Not estimable	Not estimable	Not estimable	Lower value with supplementation	SD was derived from fish-level SE, but independent tank-level means and variance were not reported; a tank-level effect size could not be estimated without pseudoreplication
Ramos-Pinto et al. (2021)	Gilthead seabream (Sparus aurata)	Histidine, taurine, or threonine supplementation in a 12% fishmeal practical diet	HIS (0.40% added histidine)	CTRL (same basal diet; no added test amino acid)	WBC (×10^4/µL) — 2 weeks	4.940 ± 1.410*	4.530 ± 1.800*	9 fish nested in 3 tanks	9 fish nested in 3 tanks	Not estimable	Not estimable	Not estimable	Higher value with supplementation	SD was derived from fish-level SE, but independent tank-level means and variance were not reported; a tank-level effect size could not be estimated without pseudoreplication
Ramos-Pinto et al. (2021)	Gilthead seabream (Sparus aurata)	Histidine, taurine, or threonine supplementation in a 12% fishmeal practical diet	HIS (0.40% added histidine)	CTRL (same basal diet; no added test amino acid)	RBC (×10^6/µL) — 2 weeks	1.240 ± 0.330*	1.140 ± 0.270*	9 fish nested in 3 tanks	9 fish nested in 3 tanks	Not estimable	Not estimable	Not estimable	Higher value with supplementation	SD was derived from fish-level SE, but independent tank-level means and variance were not reported; a tank-level effect size could not be estimated without pseudoreplication
Ramos-Pinto et al. (2021)	Gilthead seabream (Sparus aurata)	Histidine, taurine, or threonine supplementation in a 12% fishmeal practical diet	HIS (0.40% added histidine)	CTRL (same basal diet; no added test amino acid)	Thrombocytes (×10^4/µL) — 2 weeks	3.560 ± 1.050*	3.460 ± 1.710*	9 fish nested in 3 tanks	9 fish nested in 3 tanks	Not estimable	Not estimable	Not estimable	Higher value with supplementation	SD was derived from fish-level SE, but independent tank-level means and variance were not reported; a tank-level effect size could not be estimated without pseudoreplication
Ramos-Pinto et al. (2021)	Gilthead seabream (Sparus aurata)	Histidine, taurine, or threonine supplementation in a 12% fishmeal practical diet	HIS (0.40% added histidine)	CTRL (same basal diet; no added test amino acid)	Lymphocytes (×10^4/µL) — 2 weeks	1.220 ± 1.470*	0.930 ± 0.420*	9 fish nested in 3 tanks	9 fish nested in 3 tanks	Not estimable	Not estimable	Not estimable	Higher value with supplementation	SD was derived from fish-level SE, but independent tank-level means and variance were not reported; a tank-level effect size could not be estimated without pseudoreplication
Ramos-Pinto et al. (2021)	Gilthead seabream (Sparus aurata)	Histidine, taurine, or threonine supplementation in a 12% fishmeal practical diet	HIS (0.40% added histidine)	CTRL (same basal diet; no added test amino acid)	Monocytes (×10^4/µL) — 2 weeks	0.070 ± 0.060*	0.020 ± 0.030*	9 fish nested in 3 tanks	9 fish nested in 3 tanks	Not estimable	Not estimable	Not estimable	Higher value with supplementation	SD was derived from fish-level SE, but independent tank-level means and variance were not reported; a tank-level effect size could not be estimated without pseudoreplication
Ramos-Pinto et al. (2021)	Gilthead seabream (Sparus aurata)	Histidine, taurine, or threonine supplementation in a 12% fishmeal practical diet	HIS (0.40% added histidine)	CTRL (same basal diet; no added test amino acid)	Neutrophils (×10^4/µL) — 2 weeks	0.090 ± 0.030*	0.070 ± 0.060*	9 fish nested in 3 tanks	9 fish nested in 3 tanks	Not estimable	Not estimable	Not estimable	Higher value with supplementation	SD was derived from fish-level SE, but independent tank-level means and variance were not reported; a tank-level effect size could not be estimated without pseudoreplication
Ramos-Pinto et al. (2021)	Gilthead seabream (Sparus aurata)	Histidine, taurine, or threonine supplementation in a 12% fishmeal practical diet	HIS (0.40% added histidine)	CTRL (same basal diet; no added test amino acid)	Hemoglobin (g/dL) — 4 weeks	0.670 ± 0.300*	0.600 ± 0.150*	9 fish nested in 3 tanks	9 fish nested in 3 tanks	Not estimable	Not estimable	Not estimable	Higher value with supplementation	SD was derived from fish-level SE, but independent tank-level means and variance were not reported; a tank-level effect size could not be estimated without pseudoreplication
Ramos-Pinto et al. (2021)	Gilthead seabream (Sparus aurata)	Histidine, taurine, or threonine supplementation in a 12% fishmeal practical diet	HIS (0.40% added histidine)	CTRL (same basal diet; no added test amino acid)	WBC (×10^4/µL) — 4 weeks	5.940 ± 1.680*	6.210 ± 1.080*	9 fish nested in 3 tanks	9 fish nested in 3 tanks	Not estimable	Not estimable	Not estimable	Lower value with supplementation	SD was derived from fish-level SE, but independent tank-level means and variance were not reported; a tank-level effect size could not be estimated without pseudoreplication
Ramos-Pinto et al. (2021)	Gilthead seabream (Sparus aurata)	Histidine, taurine, or threonine supplementation in a 12% fishmeal practical diet	HIS (0.40% added histidine)	CTRL (same basal diet; no added test amino acid)	RBC (×10^6/µL) — 4 weeks	1.660 ± 0.360*	1.440 ± 0.300*	9 fish nested in 3 tanks	9 fish nested in 3 tanks	Not estimable	Not estimable	Not estimable	Higher value with supplementation	SD was derived from fish-level SE, but independent tank-level means and variance were not reported; a tank-level effect size could not be estimated without pseudoreplication
Ramos-Pinto et al. (2021)	Gilthead seabream (Sparus aurata)	Histidine, taurine, or threonine supplementation in a 12% fishmeal practical diet	HIS (0.40% added histidine)	CTRL (same basal diet; no added test amino acid)	Thrombocytes (×10^4/µL) — 4 weeks	4.600 ± 1.410*	4.540 ± 0.780*	9 fish nested in 3 tanks	9 fish nested in 3 tanks	Not estimable	Not estimable	Not estimable	Higher value with supplementation	SD was derived from fish-level SE, but independent tank-level means and variance were not reported; a tank-level effect size could not be estimated without pseudoreplication
Ramos-Pinto et al. (2021)	Gilthead seabream (Sparus aurata)	Histidine, taurine, or threonine supplementation in a 12% fishmeal practical diet	HIS (0.40% added histidine)	CTRL (same basal diet; no added test amino acid)	Lymphocytes (×10^4/µL) — 4 weeks	1.110 ± 1.290*	1.370 ± 0.540*	9 fish nested in 3 tanks	9 fish nested in 3 tanks	Not estimable	Not estimable	Not estimable	Lower value with supplementation	SD was derived from fish-level SE, but independent tank-level means and variance were not reported; a tank-level effect size could not be estimated without pseudoreplication
Ramos-Pinto et al. (2021)	Gilthead seabream (Sparus aurata)	Histidine, taurine, or threonine supplementation in a 12% fishmeal practical diet	HIS (0.40% added histidine)	CTRL (same basal diet; no added test amino acid)	Monocytes (×10^4/µL) — 4 weeks	0.060 ± 0.060*	0.060 ± 0.060*	9 fish nested in 3 tanks	9 fish nested in 3 tanks	Not estimable	Not estimable	Not estimable	No observed mean difference	SD was derived from fish-level SE, but independent tank-level means and variance were not reported; a tank-level effect size could not be estimated without pseudoreplication
Ramos-Pinto et al. (2021)	Gilthead seabream (Sparus aurata)	Histidine, taurine, or threonine supplementation in a 12% fishmeal practical diet	HIS (0.40% added histidine)	CTRL (same basal diet; no added test amino acid)	Neutrophils (×10^4/µL) — 4 weeks	0.150 ± 0.120*	0.220 ± 0.120*	9 fish nested in 3 tanks	9 fish nested in 3 tanks	Not estimable	Not estimable	Not estimable	Lower value with supplementation	SD was derived from fish-level SE, but independent tank-level means and variance were not reported; a tank-level effect size

Study	Species	Strategy	Intervention	Comparator	Outcome and unit	Intervention mean ± SD	Comparator mean ± SD	n intervention	n comparator	Effect-size metric	Effect size	95% CI	Interpretation	Reason not estimated
														could not be estimated without pseudoreplication

C. Plasma and skin-mucus humoral outcomes

Study	Species	Strategy	Intervention	Comparator	Outcome and unit	Intervention mean ± SD	Comparator mean ± SD	n intervention	n comparator	Effect-size metric	Effect size	95% CI	Interpretation	Reason not estimated
Ramos-Pinto et al. (2021)	Gilthead seabream (Sparus aurata)	Histidine, taurine, or threonine supplementation in a 12% fishmeal practical diet	THR (0.75% added threonine)	CTRL (same basal diet; no added test amino acid)	Plasma bactericidal activity (%) — 2 weeks	37.650 ± 8.868*	43.750 ± 7.413*	12 fish nested in 3 tanks	12 fish nested in 3 tanks	Not estimable	Not estimable	Not estimable	Lower value with supplementation	SD was derived from fish-level SE, but independent tank-level means and variance were not reported; a tank-level effect size could not be estimated without pseudoreplication
Ramos-Pinto et al. (2021)	Gilthead seabream (Sparus aurata)	Histidine, taurine, or threonine supplementation in a 12% fishmeal practical diet	THR (0.75% added threonine)	CTRL (same basal diet; no added test amino acid)	Plasma antiprotease activity (%) — 2 weeks	94.600 ± 2.044*	92.020 ± 3.429*	12 fish nested in 3 tanks	12 fish nested in 3 tanks	Not estimable	Not estimable	Not estimable	Higher value with supplementation	SD was derived from fish-level SE, but independent tank-level means and variance were not reported; a tank-level effect size could not be estimated without pseudoreplication
Ramos-Pinto et al. (2021)	Gilthead seabream (Sparus aurata)	Histidine, taurine, or threonine supplementation in a 12% fishmeal practical diet	THR (0.75% added threonine)	CTRL (same basal diet; no added test amino acid)	Plasma peroxidase activity (U/mL) — 2 weeks	6.600 ± 2.737*	6.290 ± 2.460*	12 fish nested in 3 tanks	12 fish nested in 3 tanks	Not estimable	Not estimable	Not estimable	Higher value with supplementation	SD was derived from fish-level SE, but independent tank-level means and variance were not reported; a tank-level effect size could not be estimated without pseudoreplication
Ramos-Pinto et al. (2021)	Gilthead seabream (Sparus aurata)	Histidine, taurine, or threonine supplementation in a 12% fishmeal practical diet	THR (0.75% added threonine)	CTRL (same basal diet; no added test amino acid)	Plasma IgM (absorbance) — 2 weeks	0.770 ± 0.139*	0.850 ± 0.069*	12 fish nested in 3 tanks	12 fish nested in 3 tanks	Not estimable	Not estimable	Not estimable	Lower value with supplementation	SD was derived from fish-level SE, but independent tank-level means and variance were not reported; a tank-level effect size could not be estimated without pseudoreplication
Ramos-Pinto et al. (2021)	Gilthead seabream (Sparus aurata)	Histidine, taurine, or threonine supplementation in a 12% fishmeal practical diet	THR (0.75% added threonine)	CTRL (same basal diet; no added test amino acid)	Mucus bactericidal activity (%) — 2 weeks	64.570 ± 3.083*	66.410 ± 2.182*	12 fish nested in 3 tanks	12 fish nested in 3 tanks	Not estimable	Not estimable	Not estimable	Lower value with supplementation	SD was derived from fish-level SE, but independent tank-level means and variance were not reported; a tank-level effect size could not be estimated without pseudoreplication
Ramos-Pinto et al. (2021)	Gilthead seabream (Sparus aurata)	Histidine, taurine, or threonine supplementation in a 12% fishmeal practical diet	THR (0.75% added threonine)	CTRL (same basal diet; no added test amino acid)	Mucus antiprotease activity (%) — 2 weeks	28.100 ± 6.824*	25.520 ± 9.353*	12 fish nested in 3 tanks	12 fish nested in 3 tanks	Not estimable	Not estimable	Not estimable	Higher value with supplementation	SD was derived from fish-level SE, but independent tank-level means and variance were not reported; a tank-level effect size could not be estimated without pseudoreplication
Ramos-Pinto et al. (2021)	Gilthead seabream (Sparus aurata)	Histidine, taurine, or threonine supplementation in a 12% fishmeal practical diet	THR (0.75% added threonine)	CTRL (same basal diet; no added test amino acid)	Mucus peroxidase activity (U/mg) — 2 weeks	0.890 ± 0.346*	0.730 ± 0.312*	12 fish nested in 3 tanks	12 fish nested in 3 tanks	Not estimable	Not estimable	Not estimable	Higher value with supplementation	SD was derived from fish-level SE, but independent tank-level means and variance were not reported; a tank-level effect size could not be estimated without pseudoreplication
Ramos-Pinto et al. (2021)	Gilthead seabream (Sparus aurata)	Histidine, taurine, or threonine supplementation in a 12% fishmeal practical diet	THR (0.75% added threonine)	CTRL (same basal diet; no added test amino acid)	Plasma bactericidal activity (%) — 4 weeks	48.250 ± 9.561*	47.560 ± 10.427*	12 fish nested in 3 tanks	12 fish nested in 3 tanks	Not estimable	Not estimable	Not estimable	Higher value with supplementation	SD was derived from fish-level SE, but independent tank-level means and variance were not reported; a tank-level effect size could not be estimated without pseudoreplication
Ramos-Pinto et al. (2021)	Gilthead seabream (Sparus aurata)	Histidine, taurine, or threonine supplementation in a 12% fishmeal practical diet	THR (0.75% added threonine)	CTRL (same basal diet; no added test amino acid)	Plasma antiprotease activity (%) — 4 weeks	95.450 ± 3.499*	92.690 ± 4.954*	12 fish nested in 3 tanks	12 fish nested in 3 tanks	Not estimable	Not estimable	Not estimable	Higher value with supplementation	SD was derived from fish-level SE, but independent tank-level means and variance were not reported; a tank-level effect size could not be estimated without pseudoreplication
Ramos-Pinto et al. (2021)	Gilthead seabream (Sparus aurata)	Histidine, taurine, or threonine supplementation in a 12% fishmeal practical diet	THR (0.75% added threonine)	CTRL (same basal diet; no added test amino acid)	Plasma peroxidase activity (U/mL) — 4 weeks	7.180 ± 1.905*	7.070 ± 2.460*	12 fish nested in 3 tanks	12 fish nested in 3 tanks	Not estimable	Not estimable	Not estimable	Higher value with supplementation	SD was derived from fish-level SE, but independent tank-level means and variance were not reported; a tank-level effect size could not be estimated without pseudoreplication
Ramos-Pinto et al. (2021)	Gilthead seabream (Sparus aurata)	Histidine, taurine, or threonine supplementation in a 12% fishmeal practical diet	THR (0.75% added threonine)	CTRL (same basal diet; no added test amino acid)	Plasma IgM (absorbance) — 4 weeks	0.680 ± 0.208*	0.720 ± 0.173*	12 fish nested in 3 tanks	12 fish nested in 3 tanks	Not estimable	Not estimable	Not estimable	Lower value with supplementation	SD was derived from fish-level SE, but independent tank-level means and variance were not reported; a tank-level effect size could not be estimated without pseudoreplication
Ramos-Pinto et al. (2021)	Gilthead seabream (Sparus aurata)	Histidine, taurine, or threonine supplementation in a 12% fishmeal practical diet	THR (0.75% added threonine)	CTRL (same basal diet; no added test amino acid)	Mucus bactericidal activity (%) — 4 weeks	59.110 ± 5.508*	55.830 ± 6.097*	12 fish nested in 3 tanks	12 fish nested in 3 tanks	Not estimable	Not estimable	Not estimable	Higher value with supplementation	SD was derived from fish-level SE, but independent tank-level means and variance were not reported; a tank-level effect size could not be estimated without pseudoreplication
Ramos-Pinto et al. (2021)	Gilthead seabream (Sparus aurata)	Histidine, taurine, or threonine supplementation in a 12% fishmeal practical diet	THR (0.75% added threonine)	CTRL (same basal diet; no added test amino acid)	Mucus antiprotease activity (%) — 4 weeks	26.880 ± 4.538*	26.990 ± 4.607*	12 fish nested in 3 tanks	12 fish nested in 3 tanks	Not estimable	Not estimable	Not estimable	Lower value with supplementation	SD was derived from fish-level SE, but independent tank-level means and variance were not reported; a tank-level effect size could not be estimated without pseudoreplication
Ramos-Pinto et al. (2021)	Gilthead seabream (Sparus aurata)	Histidine, taurine, or threonine supplementation in a 12% fishmeal practical diet	THR (0.75% added threonine)	CTRL (same basal diet; no added test amino acid)	Mucus peroxidase activity (U/mg) — 4 weeks	0.780 ± 0.381*	0.690 ± 0.312*	12 fish nested in 3 tanks	12 fish nested in 3 tanks	Not estimable	Not estimable	Not estimable	Higher value with supplementation	SD was derived from fish-level SE, but independent tank-level means and variance were not reported; a tank-level effect size could not be estimated without pseudoreplication
Ramos-Pinto et al. (2021)	Gilthead seabream (Sparus aurata)	Histidine, taurine, or threonine supplementation in a 12% fishmeal practical diet	TAU (0.50% added taurine)	CTRL (same basal diet; no added test amino acid)	Plasma bactericidal activity (%) — 2 weeks	38.470 ± 7.621*	43.750 ± 7.413*	12 fish nested in 3 tanks	12 fish nested in 3 tanks	Not estimable	Not estimable	Not estimable	Lower value with supplementation	SD was derived from fish-level SE, but independent tank-level means and variance were not reported; a tank-level effect size could not be estimated without pseudoreplication
Ramos-Pinto et al. (2021)	Gilthead seabream (Sparus aurata)	Histidine, taurine, or threonine supplementation in a 12% fishmeal practical diet	TAU (0.50% added taurine)	CTRL (same basal diet; no added test amino acid)	Plasma antiprotease activity (%) — 2 weeks	90.060 ± 2.944*	92.020 ± 3.429*	12 fish nested in 3 tanks	12 fish nested in 3 tanks	Not estimable	Not estimable	Not estimable	Lower value with supplementation	SD was derived from fish-level SE, but independent tank-level means and variance were not reported; a tank-level effect size could not be estimated without pseudoreplication
Ramos-Pinto et al. (2021)	Gilthead seabream (Sparus aurata)	Histidine, taurine, or threonine supplementation in a 12% fishmeal practical diet	TAU (0.50% added taurine)	CTRL (same basal diet; no added test amino acid)	Plasma peroxidase activity (U/mL) — 2 weeks	6.340 ± 2.217*	6.290 ± 2.460*	12 fish nested in 3 tanks	12 fish nested in 3 tanks	Not estimable	Not estimable	Not estimable	Higher value with supplementation	SD was derived from fish-level SE, but independent tank-level means and variance were not reported; a tank-level effect size could not be estimated without

Study	Species	Strategy	Intervention	Comparator	Outcome and unit	Intervention mean ± SD	Comparator mean ± SD	n intervention	n comparator	Effect-size metric	Effect size	95% CI	Interpretation	Reason not estimated
														pseudoreplication
Ramos-Pinto et al. (2021)	Gilthead seabream (Sparus aurata)	Histidine, taurine, or threonine supplementation in a 12% fishmeal practical diet	TAU (0.50% added taurine)	CTRL (same basal diet; no added test amino acid)	Plasma IgM (absorbance) — 2 weeks	0.660 ± 0.139*	0.850 ± 0.069*	12 fish nested in 3 tanks	12 fish nested in 3 tanks	Not estimable	Not estimable	Not estimable	Lower value with supplementation	SD was derived from fish-level SE, but independent tank-level means and variance were not reported; a tank-level effect size could not be estimated without pseudoreplication
Ramos-Pinto et al. (2021)	Gilthead seabream (Sparus aurata)	Histidine, taurine, or threonine supplementation in a 12% fishmeal practical diet	TAU (0.50% added taurine)	CTRL (same basal diet; no added test amino acid)	Mucus bactericidal activity (%) — 2 weeks	60.800 ± 4.642*	66.410 ± 2.182*	12 fish nested in 3 tanks	12 fish nested in 3 tanks	Not estimable	Not estimable	Not estimable	Lower value with supplementation	SD was derived from fish-level SE, but independent tank-level means and variance were not reported; a tank-level effect size could not be estimated without pseudoreplication
Ramos-Pinto et al. (2021)	Gilthead seabream (Sparus aurata)	Histidine, taurine, or threonine supplementation in a 12% fishmeal practical diet	TAU (0.50% added taurine)	CTRL (same basal diet; no added test amino acid)	Mucus antiprotease activity (%) — 2 weeks	28.360 ± 7.275*	25.520 ± 9.353*	12 fish nested in 3 tanks	12 fish nested in 3 tanks	Not estimable	Not estimable	Not estimable	Higher value with supplementation	SD was derived from fish-level SE, but independent tank-level means and variance were not reported; a tank-level effect size could not be estimated without pseudoreplication
Ramos-Pinto et al. (2021)	Gilthead seabream (Sparus aurata)	Histidine, taurine, or threonine supplementation in a 12% fishmeal practical diet	TAU (0.50% added taurine)	CTRL (same basal diet; no added test amino acid)	Mucus peroxidase activity (U/mg) — 2 weeks	1.000 ± 0.416*	0.730 ± 0.312*	12 fish nested in 3 tanks	12 fish nested in 3 tanks	Not estimable	Not estimable	Not estimable	Higher value with supplementation	SD was derived from fish-level SE, but independent tank-level means and variance were not reported; a tank-level effect size could not be estimated without pseudoreplication
Ramos-Pinto et al. (2021)	Gilthead seabream (Sparus aurata)	Histidine, taurine, or threonine supplementation in a 12% fishmeal practical diet	TAU (0.50% added taurine)	CTRL (same basal diet; no added test amino acid)	Plasma bactericidal activity (%) — 4 weeks	39.920 ± 11.432*	47.560 ± 10.427*	12 fish nested in 3 tanks	12 fish nested in 3 tanks	Not estimable	Not estimable	Not estimable	Lower value with supplementation	SD was derived from fish-level SE, but independent tank-level means and variance were not reported; a tank-level effect size could not be estimated without pseudoreplication
Ramos-Pinto et al. (2021)	Gilthead seabream (Sparus aurata)	Histidine, taurine, or threonine supplementation in a 12% fishmeal practical diet	TAU (0.50% added taurine)	CTRL (same basal diet; no added test amino acid)	Plasma antiprotease activity (%) — 4 weeks	94.560 ± 2.044*	92.690 ± 4.954*	12 fish nested in 3 tanks	12 fish nested in 3 tanks	Not estimable	Not estimable	Not estimable	Higher value with supplementation	SD was derived from fish-level SE, but independent tank-level means and variance were not reported; a tank-level effect size could not be estimated without pseudoreplication
Ramos-Pinto et al. (2021)	Gilthead seabream (Sparus aurata)	Histidine, taurine, or threonine supplementation in a 12% fishmeal practical diet	TAU (0.50% added taurine)	CTRL (same basal diet; no added test amino acid)	Plasma peroxidase activity (U/mL) — 4 weeks	6.440 ± 1.490*	7.070 ± 2.460*	12 fish nested in 3 tanks	12 fish nested in 3 tanks	Not estimable	Not estimable	Not estimable	Lower value with supplementation	SD was derived from fish-level SE, but independent tank-level means and variance were not reported; a tank-level effect size could not be estimated without pseudoreplication
Ramos-Pinto et al. (2021)	Gilthead seabream (Sparus aurata)	Histidine, taurine, or threonine supplementation in a 12% fishmeal practical diet	TAU (0.50% added taurine)	CTRL (same basal diet; no added test amino acid)	Plasma IgM (absorbance) — 4 weeks	0.740 ± 0.139*	0.720 ± 0.173*	12 fish nested in 3 tanks	12 fish nested in 3 tanks	Not estimable	Not estimable	Not estimable	Higher value with supplementation	SD was derived from fish-level SE, but independent tank-level means and variance were not reported; a tank-level effect size could not be estimated without pseudoreplication
Ramos-Pinto et al. (2021)	Gilthead seabream (Sparus aurata)	Histidine, taurine, or threonine supplementation in a 12% fishmeal practical diet	TAU (0.50% added taurine)	CTRL (same basal diet; no added test amino acid)	Mucus bactericidal activity (%) — 4 weeks	59.080 ± 4.122*	55.830 ± 6.097*	12 fish nested in 3 tanks	12 fish nested in 3 tanks	Not estimable	Not estimable	Not estimable	Higher value with supplementation	SD was derived from fish-level SE, but independent tank-level means and variance were not reported; a tank-level effect size could not be estimated without pseudoreplication
Ramos-Pinto et al. (2021)	Gilthead seabream (Sparus aurata)	Histidine, taurine, or threonine supplementation in a 12% fishmeal practical diet	TAU (0.50% added taurine)	CTRL (same basal diet; no added test amino acid)	Mucus antiprotease activity (%) — 4 weeks	28.690 ± 8.626*	26.990 ± 4.607*	12 fish nested in 3 tanks	12 fish nested in 3 tanks	Not estimable	Not estimable	Not estimable	Higher value with supplementation	SD was derived from fish-level SE, but independent tank-level means and variance were not reported; a tank-level effect size could not be estimated without pseudoreplication
Ramos-Pinto et al. (2021)	Gilthead seabream (Sparus aurata)	Histidine, taurine, or threonine supplementation in a 12% fishmeal practical diet	TAU (0.50% added taurine)	CTRL (same basal diet; no added test amino acid)	Mucus peroxidase activity (U/mg) — 4 weeks	0.710 ± 0.277*	0.690 ± 0.312*	12 fish nested in 3 tanks	12 fish nested in 3 tanks	Not estimable	Not estimable	Not estimable	Higher value with supplementation	SD was derived from fish-level SE, but independent tank-level means and variance were not reported; a tank-level effect size could not be estimated without pseudoreplication
Ramos-Pinto et al. (2021)	Gilthead seabream (Sparus aurata)	Histidine, taurine, or threonine supplementation in a 12% fishmeal practical diet	HIS (0.40% added histidine)	CTRL (same basal diet; no added test amino acid)	Plasma bactericidal activity (%) — 2 weeks	42.390 ± 11.778*	43.750 ± 7.413*	12 fish nested in 3 tanks	12 fish nested in 3 tanks	Not estimable	Not estimable	Not estimable	Lower value with supplementation	SD was derived from fish-level SE, but independent tank-level means and variance were not reported; a tank-level effect size could not be estimated without pseudoreplication
Ramos-Pinto et al. (2021)	Gilthead seabream (Sparus aurata)	Histidine, taurine, or threonine supplementation in a 12% fishmeal practical diet	HIS (0.40% added histidine)	CTRL (same basal diet; no added test amino acid)	Plasma antiprotease activity (%) — 2 weeks	91.710 ± 3.603*	92.020 ± 3.429*	12 fish nested in 3 tanks	12 fish nested in 3 tanks	Not estimable	Not estimable	Not estimable	Lower value with supplementation	SD was derived from fish-level SE, but independent tank-level means and variance were not reported; a tank-level effect size could not be estimated without pseudoreplication
Ramos-Pinto et al. (2021)	Gilthead seabream (Sparus aurata)	Histidine, taurine, or threonine supplementation in a 12% fishmeal practical diet	HIS (0.40% added histidine)	CTRL (same basal diet; no added test amino acid)	Plasma peroxidase activity (U/mL) — 2 weeks	6.950 ± 1.490*	6.290 ± 2.460*	12 fish nested in 3 tanks	12 fish nested in 3 tanks	Not estimable	Not estimable	Not estimable	Higher value with supplementation	SD was derived from fish-level SE, but independent tank-level means and variance were not reported; a tank-level effect size could not be estimated without pseudoreplication
Ramos-Pinto et al. (2021)	Gilthead seabream (Sparus aurata)	Histidine, taurine, or threonine supplementation in a 12% fishmeal practical diet	HIS (0.40% added histidine)	CTRL (same basal diet; no added test amino acid)	Plasma IgM (absorbance) — 2 weeks	0.810 ± 0.173*	0.850 ± 0.069*	12 fish nested in 3 tanks	12 fish nested in 3 tanks	Not estimable	Not estimable	Not estimable	Lower value with supplementation	SD was derived from fish-level SE, but independent tank-level means and variance were not reported; a tank-level effect size could not be estimated without pseudoreplication
Ramos-Pinto et al. (2021)	Gilthead seabream (Sparus aurata)	Histidine, taurine, or threonine supplementation in a 12% fishmeal practical diet	HIS (0.40% added histidine)	CTRL (same basal diet; no added test amino acid)	Mucus bactericidal activity (%) — 2 weeks	59.400 ± 4.365*	66.410 ± 2.182*	12 fish nested in 3 tanks	12 fish nested in 3 tanks	Not estimable	Not estimable	Not estimable	Lower value with supplementation	SD was derived from fish-level SE, but independent tank-level means and variance were not reported; a tank-level effect size could not be estimated without pseudoreplication
Ramos-Pinto et al. (2021)	Gilthead seabream (Sparus aurata)	Histidine, taurine, or threonine supplementation in a 12% fishmeal practical diet	HIS (0.40% added histidine)	CTRL (same basal diet; no added test amino acid)	Mucus antiprotease activity (%) — 2 weeks	27.520 ± 3.118*	25.520 ± 9.353*	12 fish nested in 3 tanks	12 fish nested in 3 tanks	Not estimable	Not estimable	Not estimable	Higher value with supplementation	SD was derived from fish-level SE, but independent tank-level means and variance were not reported; a tank-level effect size could not be estimated without pseudoreplication
Ramos-Pinto et al. (2021)	Gilthead seabream (Sparus aurata)	Histidine, taurine, or threonine supplementation in a 12% fishmeal practical diet	HIS (0.40% added histidine)	CTRL (same basal diet; no added test amino acid)	Mucus peroxidase activity (U/mg) — 2 weeks	0.810 ± 0.381*	0.730 ± 0.312*	12 fish nested in 3 tanks	12 fish nested in 3 tanks	Not estimable	Not estimable	Not estimable	Higher value with supplementation	SD was derived from fish-level SE, but independent tank-level means and variance were not reported; a tank-level effect size could not be estimated without pseudoreplication
Ramos-Pinto et al.	Gilthead seabream	Histidine, taurine, or	HIS (0.40% added histidine)	CTRL (same basal diet; no added	Plasma bactericidal activity	43.540 ± 10.046*	47.560 ± 10.427*	12 fish	12 fish	Not estimable	Not	Not estimable	Lower value with supplementation	SD was derived from fish-level SE, but

Study	Species	Strategy	Intervention	Comparator	Outcome and unit	Intervention mean ± SD	Comparator mean ± SD	n intervention	n comparator	Effect-size metric	Effect size	95% CI	Interpretation	Reason not estimated
(2021)	(Sparus aurata)	threonine supplementation in a 12% fishmeal practical diet		test amino acid)	(%) — 4 weeks			nested in 3 tanks	nested in 3 tanks		estimable			independent tank-level means and variance were not reported; a tank-level effect size could not be estimated without pseudoreplication
Ramos-Pinto et al. (2021)	Gilthead seabream (Sparus aurata)	Histidine, taurine, or threonine supplementation in a 12% fishmeal practical diet	HIS (0.40% added histidine)	CTRL (same basal diet; no added test amino acid)	Plasma antiprotease activity (%) — 4 weeks	95.970 ± 1.732*	92.690 ± 4.954*	12 fish nested in 3 tanks	12 fish nested in 3 tanks	Not estimable	Not estimable	Not estimable	Higher value with supplementation	SD was derived from fish-level SE, but independent tank-level means and variance were not reported; a tank-level effect size could not be estimated without pseudoreplication
Ramos-Pinto et al. (2021)	Gilthead seabream (Sparus aurata)	Histidine, taurine, or threonine supplementation in a 12% fishmeal practical diet	HIS (0.40% added histidine)	CTRL (same basal diet; no added test amino acid)	Plasma peroxidase activity (U/mL) — 4 weeks	7.940 ± 3.464*	7.070 ± 2.460*	12 fish nested in 3 tanks	12 fish nested in 3 tanks	Not estimable	Not estimable	Not estimable	Higher value with supplementation	SD was derived from fish-level SE, but independent tank-level means and variance were not reported; a tank-level effect size could not be estimated without pseudoreplication
Ramos-Pinto et al. (2021)	Gilthead seabream (Sparus aurata)	Histidine, taurine, or threonine supplementation in a 12% fishmeal practical diet	HIS (0.40% added histidine)	CTRL (same basal diet; no added test amino acid)	Plasma IgM (absorbance) — 4 weeks	0.660 ± 0.173*	0.720 ± 0.173*	12 fish nested in 3 tanks	12 fish nested in 3 tanks	Not estimable	Not estimable	Not estimable	Lower value with supplementation	SD was derived from fish-level SE, but independent tank-level means and variance were not reported; a tank-level effect size could not be estimated without pseudoreplication
Ramos-Pinto et al. (2021)	Gilthead seabream (Sparus aurata)	Histidine, taurine, or threonine supplementation in a 12% fishmeal practical diet	HIS (0.40% added histidine)	CTRL (same basal diet; no added test amino acid)	Mucus bactericidal activity (%) — 4 weeks	57.690 ± 5.162*	55.830 ± 6.097*	12 fish nested in 3 tanks	12 fish nested in 3 tanks	Not estimable	Not estimable	Not estimable	Higher value with supplementation	SD was derived from fish-level SE, but independent tank-level means and variance were not reported; a tank-level effect size could not be estimated without pseudoreplication
Ramos-Pinto et al. (2021)	Gilthead seabream (Sparus aurata)	Histidine, taurine, or threonine supplementation in a 12% fishmeal practical diet	HIS (0.40% added histidine)	CTRL (same basal diet; no added test amino acid)	Mucus antiprotease activity (%) — 4 weeks	30.620 ± 9.007*	26.990 ± 4.607*	12 fish nested in 3 tanks	12 fish nested in 3 tanks	Not estimable	Not estimable	Not estimable	Higher value with supplementation	SD was derived from fish-level SE, but independent tank-level means and variance were not reported; a tank-level effect size could not be estimated without pseudoreplication
Ramos-Pinto et al. (2021)	Gilthead seabream (Sparus aurata)	Histidine, taurine, or threonine supplementation in a 12% fishmeal practical diet	HIS (0.40% added histidine)	CTRL (same basal diet; no added test amino acid)	Mucus peroxidase activity (U/mg) — 4 weeks	0.780 ± 1.420*	0.690 ± 0.312*	12 fish nested in 3 tanks	12 fish nested in 3 tanks	Not estimable	Not estimable	Not estimable	Higher value with supplementation	SD was derived from fish-level SE, but independent tank-level means and variance were not reported; a tank-level effect size could not be estimated without pseudoreplication

D. Figure-only head-kidney gene-expression outcomes

Study	Species	Strategy	Intervention	Comparator	Outcome and unit	Intervention mean ± SD	Comparator mean ± SD	n intervention	n comparator	Effect-size metric	Effect size	95% CI	Interpretation	Reason not estimated
Ramos-Pinto et al. (2021)	Gilthead seabream (Sparus aurata)	Histidine, taurine, or threonine supplementation in a 12% fishmeal practical diet	THR (0.75% added threonine)	CTRL (same basal diet; no added test amino acid)	Head-kidney IgT-m relative expression — 2 weeks	Figure-only; exact value not tabulated in the uploaded PDF	Figure-only; exact value not tabulated in the uploaded PDF	9 fish nested in 3 tanks	9 fish nested in 3 tanks	Not estimable	Not estimable	Not estimable	Gene expression was shown graphically; no clearly significant difference from CTRL was reported for this comparison	Exact numerical means and variance were not available in the uploaded PDF; the full raw-expression table is supplementary
Ramos-Pinto et al. (2021)	Gilthead seabream (Sparus aurata)	Histidine, taurine, or threonine supplementation in a 12% fishmeal practical diet	THR (0.75% added threonine)	CTRL (same basal diet; no added test amino acid)	Head-kidney clec10a relative expression — 2 weeks	Figure-only; exact value not tabulated in the uploaded PDF	Figure-only; exact value not tabulated in the uploaded PDF	9 fish nested in 3 tanks	9 fish nested in 3 tanks	Not estimable	Not estimable	Not estimable	Lower clec10a expression than CTRL at 2 weeks according to the original post-hoc comparison	Exact numerical means and variance were not available in the uploaded PDF; the full raw-expression table is supplementary
Ramos-Pinto et al. (2021)	Gilthead seabream (Sparus aurata)	Histidine, taurine, or threonine supplementation in a 12% fishmeal practical diet	THR (0.75% added threonine)	CTRL (same basal diet; no added test amino acid)	Head-kidney IgT-m relative expression — 4 weeks	Figure-only; exact value not tabulated in the uploaded PDF	Figure-only; exact value not tabulated in the uploaded PDF	9 fish nested in 3 tanks	9 fish nested in 3 tanks	Not estimable	Not estimable	Not estimable	Gene expression was shown graphically; no clearly significant difference from CTRL was reported for this comparison	Exact numerical means and variance were not available in the uploaded PDF; the full raw-expression table is supplementary
Ramos-Pinto et al. (2021)	Gilthead seabream (Sparus aurata)	Histidine, taurine, or threonine supplementation in a 12% fishmeal practical diet	THR (0.75% added threonine)	CTRL (same basal diet; no added test amino acid)	Head-kidney clec10a relative expression — 4 weeks	Figure-only; exact value not tabulated in the uploaded PDF	Figure-only; exact value not tabulated in the uploaded PDF	9 fish nested in 3 tanks	9 fish nested in 3 tanks	Not estimable	Not estimable	Not estimable	Gene expression was shown graphically; no clearly significant difference from CTRL was reported for this comparison	Exact numerical means and variance were not available in the uploaded PDF; the full raw-expression table is supplementary
Ramos-Pinto et al. (2021)	Gilthead seabream (Sparus aurata)	Histidine, taurine, or threonine supplementation in a 12% fishmeal practical diet	TAU (0.50% added taurine)	CTRL (same basal diet; no added test amino acid)	Head-kidney IgT-m relative expression — 2 weeks	Figure-only; exact value not tabulated in the uploaded PDF	Figure-only; exact value not tabulated in the uploaded PDF	9 fish nested in 3 tanks	9 fish nested in 3 tanks	Not estimable	Not estimable	Not estimable	Gene expression was shown graphically; no clearly significant difference from CTRL was reported for this comparison	Exact numerical means and variance were not available in the uploaded PDF; the full raw-expression table is supplementary
Ramos-Pinto et al. (2021)	Gilthead seabream (Sparus aurata)	Histidine, taurine, or threonine supplementation in a 12% fishmeal practical diet	TAU (0.50% added taurine)	CTRL (same basal diet; no added test amino acid)	Head-kidney clec10a relative expression — 2 weeks	Figure-only; exact value not tabulated in the uploaded PDF	Figure-only; exact value not tabulated in the uploaded PDF	9 fish nested in 3 tanks	9 fish nested in 3 tanks	Not estimable	Not estimable	Not estimable	Gene expression was shown graphically; no clearly significant difference from CTRL was reported for this comparison	Exact numerical means and variance were not available in the uploaded PDF; the full raw-expression table is supplementary
Ramos-Pinto et al. (2021)	Gilthead seabream (Sparus aurata)	Histidine, taurine, or threonine supplementation in a 12% fishmeal practical diet	TAU (0.50% added taurine)	CTRL (same basal diet; no added test amino acid)	Head-kidney IgT-m relative expression — 4 weeks	Figure-only; exact value not tabulated in the uploaded PDF	Figure-only; exact value not tabulated in the uploaded PDF	9 fish nested in 3 tanks	9 fish nested in 3 tanks	Not estimable	Not estimable	Not estimable	Lower IgT-m expression than THR at 4 weeks according to the original post-hoc comparison	Exact numerical means and variance were not available in the uploaded PDF; the full raw-expression table is supplementary
Ramos-Pinto et al. (2021)	Gilthead seabream (Sparus aurata)	Histidine, taurine, or threonine supplementation in a 12% fishmeal practical diet	TAU (0.50% added taurine)	CTRL (same basal diet; no added test amino acid)	Head-kidney clec10a relative expression — 4 weeks	Figure-only; exact value not tabulated in the uploaded PDF	Figure-only; exact value not tabulated in the uploaded PDF	9 fish nested in 3 tanks	9 fish nested in 3 tanks	Not estimable	Not estimable	Not estimable	Gene expression was shown graphically; no clearly significant difference from CTRL was reported for this comparison	Exact numerical means and variance were not available in the uploaded PDF; the full raw-expression table is supplementary
Ramos-Pinto et al. (2021)	Gilthead seabream (Sparus aurata)	Histidine, taurine, or threonine supplementation in a 12% fishmeal practical diet	HIS (0.40% added histidine)	CTRL (same basal diet; no added test amino acid)	Head-kidney IgT-m relative expression — 2 weeks	Figure-only; exact value not tabulated in the uploaded PDF	Figure-only; exact value not tabulated in the uploaded PDF	9 fish nested in 3 tanks	9 fish nested in 3 tanks	Not estimable	Not estimable	Not estimable	Gene expression was shown graphically; no clearly significant difference from CTRL was reported for this comparison	Exact numerical means and variance were not available in the uploaded PDF; the full raw-expression table is supplementary
Ramos-Pinto et al. (2021)	Gilthead seabream (Sparus aurata)	Histidine, taurine, or threonine supplementation in a 12% fishmeal practical diet	HIS (0.40% added histidine)	CTRL (same basal diet; no added test amino acid)	Head-kidney clec10a relative expression — 2 weeks	Figure-only; exact value not tabulated in the uploaded PDF	Figure-only; exact value not tabulated in the uploaded PDF	9 fish nested in 3 tanks	9 fish nested in 3 tanks	Not estimable	Not estimable	Not estimable	Gene expression was shown graphically; no clearly significant difference from CTRL was reported for this comparison	Exact numerical means and variance were not available in the uploaded PDF; the full raw-expression table is supplementary
Ramos-Pinto et al. (2021)	Gilthead seabream (Sparus aurata)	Histidine, taurine, or threonine supplementation in a 12% fishmeal practical diet	HIS (0.40% added histidine)	CTRL (same basal diet; no added test amino acid)	Head-kidney IgT-m relative expression — 4 weeks	Figure-only; exact value not tabulated in the uploaded PDF	Figure-only; exact value not tabulated in the uploaded PDF	9 fish nested in 3 tanks	9 fish nested in 3 tanks	Not estimable	Not estimable	Not estimable	Gene expression was shown graphically; no clearly significant difference from CTRL was reported for this comparison	Exact numerical means and variance were not available in the uploaded PDF; the full raw-expression table is supplementary
Ramos-Pinto et al. (2021)	Gilthead seabream (Sparus aurata)	Histidine, taurine, or threonine supplementation in a 12% fishmeal practical diet	HIS (0.40% added histidine)	CTRL (same basal diet; no added test amino acid)	Head-kidney clec10a relative expression — 4 weeks	Figure-only; exact value not tabulated in the uploaded PDF	Figure-only; exact value not tabulated in the uploaded PDF	9 fish nested in 3 tanks	9 fish nested in 3 tanks	Not estimable	Not estimable	Not estimable	Gene expression was shown graphically; no clearly significant difference from CTRL was reported for this comparison	Exact numerical means and variance were not available in the uploaded PDF; the full raw-expression table is supplementary

Notes. Green rows contain provisional tank-level Hedges' g estimates for relative growth rate. Yellow rows were not assigned effect sizes because the reported observations were fish nested within tanks, mortality was absent in both groups, or exact gene-expression values were not numerically available. *SD derived from the reported SE.

Study 8. Wang et al. (2023): balanced control versus Leu-deficient or single-BCAA-excess diets

Study	Species	Strategy	Intervention	Comparator	Outcome and unit	Intervention mean ± SD	Comparator mean ± SD	n intervention (tanks)	n comparator (tanks)	Effect-size metric	Effect size	95% CI	Interpretation	Reason not estimated
Wang et al. (2023)	Turbot (Scophthalmus maximus)	BCAA balancing to estimated requirement levels	Control (balanced BCAAs: Leu 30.4, Ile 19.7, Val 17.4 g/kg)	Leu-L (Leu deficient: 20.9 g/kg)	Survival (%)	98.70 ± 2.25	98.70 ± 2.25	3	3	Hedges' g	0.0	-1.60 to 1.60	No observed mean difference; 95% CI included 0 (imprecise estimate)	Not applicable - effect size estimated after converting SE to SD
Wang et al. (2023)	Turbot (Scophthalmus maximus)	BCAA balancing to estimated requirement levels	Control (balanced BCAAs: Leu 30.4, Ile 19.7, Val 17.4 g/kg)	Leu-L (Leu deficient: 20.9 g/kg)	Weight gain (%)	117.80 ± 13.16	96.20 ± 12.12	3	3	Hedges' g	1.37	-0.49 to 3.22	Higher, more favorable value with the balanced BCAA profile; 95% CI included 0 (imprecise estimate)	Not applicable - effect size estimated after converting SE to SD
Wang et al. (2023)	Turbot (Scophthalmus maximus)	BCAA balancing to estimated requirement levels	Control (balanced BCAAs: Leu 30.4, Ile 19.7, Val 17.4 g/kg)	Leu-L (Leu deficient: 20.9 g/kg)	Specific growth rate (%/day)	1.29 ± 0.10	1.12 ± 0.10	3	3	Hedges' g	1.31	-0.53 to 3.15	Higher, more favorable value with the balanced BCAA profile; 95% CI included 0 (imprecise estimate)	Not applicable - effect size estimated after converting SE to SD
Wang et al. (2023)	Turbot (Scophthalmus maximus)	BCAA balancing to estimated requirement levels	Control (balanced BCAAs: Leu 30.4, Ile 19.7, Val 17.4 g/kg)	Leu-L (Leu deficient: 20.9 g/kg)	Feed intake (% body weight/day)	1.12 ± 0.03	1.10 ± 0.05	3	3	Hedges' g	0.36	-1.26 to 1.98	Higher measured value with the balanced BCAA profile; 95% CI included 0 (imprecise estimate)	Not applicable - effect size estimated after converting SE to SD
Wang et al. (2023)	Turbot (Scophthalmus maximus)	BCAA balancing to estimated requirement levels	Control (balanced BCAAs: Leu 30.4, Ile 19.7, Val 17.4 g/kg)	Leu-L (Leu deficient: 20.9 g/kg)	Feed efficiency	1.11 ± 0.05	1.00 ± 0.07	3	3	Hedges' g	1.44	-0.45 to 3.32	Higher, more favorable value with the balanced BCAA profile; 95% CI included 0 (imprecise estimate)	Not applicable - effect size estimated after converting SE to SD
Wang et al. (2023)	Turbot (Scophthalmus maximus)	BCAA balancing to estimated requirement levels	Control (balanced BCAAs: Leu 30.4, Ile 19.7, Val 17.4 g/kg)	Leu-L (Leu deficient: 20.9 g/kg)	Protein efficiency ratio	2.17 ± 0.16	1.93 ± 0.16	3	3	Hedges' g	1.23	-0.58 to 3.05	Higher, more favorable value with the balanced BCAA profile; 95% CI included 0 (imprecise estimate)	Not applicable - effect size estimated after converting SE to SD
Wang et al. (2023)	Turbot (Scophthalmus maximus)	BCAA balancing to estimated requirement levels	Control (balanced BCAAs: Leu 30.4, Ile 19.7, Val 17.4 g/kg)	Leu-L (Leu deficient: 20.9 g/kg)	Protein productive value (%)	34.40 ± 2.08	32.80 ± 1.91	3	3	Hedges' g	0.64	-1.02 to 2.30	Higher, more favorable value with the balanced BCAA profile; 95% CI included 0 (imprecise estimate)	Not applicable - effect size estimated after converting SE to SD
Wang et al. (2023)	Turbot (Scophthalmus maximus)	BCAA balancing to estimated requirement levels	Control (balanced BCAAs: Leu 30.4, Ile 19.7, Val 17.4 g/kg)	Leu-L (Leu deficient: 20.9 g/kg)	Moisture (g/kg)	774.70 ± 5.54	772.00 ± 11.95	3	3	Hedges' g	0.23	-1.38 to 1.84	Higher measured value with the balanced BCAA profile; 95% CI included 0 (imprecise estimate)	Not applicable - effect size estimated after converting SE to SD
Wang et al. (2023)	Turbot (Scophthalmus maximus)	BCAA balancing to estimated requirement levels	Control (balanced BCAAs: Leu 30.4, Ile 19.7, Val 17.4 g/kg)	Leu-L (Leu deficient: 20.9 g/kg)	Crude protein (g/kg)	153.00 ± 2.60	157.70 ± 7.45	3	3	Hedges' g	-0.67	-2.34 to 0.99	Lower measured value with the balanced BCAA profile; 95% CI included 0 (imprecise estimate)	Not applicable - effect size estimated after converting SE to SD
Wang et al. (2023)	Turbot (Scophthalmus maximus)	BCAA balancing to estimated requirement levels	Control (balanced BCAAs: Leu 30.4, Ile 19.7, Val 17.4 g/kg)	Leu-L (Leu deficient: 20.9 g/kg)	Crude lipid (g/kg)	26.10 ± 4.85	22.30 ± 3.64	3	3	Hedges' g	0.71	-0.96 to 2.38	Higher measured value with the balanced BCAA profile; 95% CI included 0 (imprecise estimate)	Not applicable - effect size estimated after converting SE to SD
Wang et al. (2023)	Turbot (Scophthalmus maximus)	BCAA balancing to estimated requirement levels	Control (balanced BCAAs: Leu 30.4, Ile 19.7, Val 17.4 g/kg)	Leu-L (Leu deficient: 20.9 g/kg)	Ash (g/kg)	37.20 ± 2.08	42.20 ± 3.29	3	3	Hedges' g	-1.45	-3.34 to 0.44	Lower measured value with the balanced BCAA profile; 95% CI included 0 (imprecise estimate)	Not applicable - effect size estimated after converting SE to SD
Wang et al. (2023)	Turbot (Scophthalmus maximus)	BCAA balancing to estimated requirement levels	Control (balanced BCAAs: Leu 30.4, Ile 19.7, Val 17.4 g/kg)	Leu-L (Leu deficient: 20.9 g/kg)	Plasma free Leu, 2 h after feeding (µg/mL)	57.23 ± 7.60	23.73 ± 5.84	3	3	Hedges' g	3.95	0.78 to 7.13	Higher measured value with the balanced BCAA profile; 95% CI excluded 0	Not applicable - effect size estimated after converting SE to SD
Wang et al. (2023)	Turbot (Scophthalmus maximus)	BCAA balancing to estimated requirement levels	Control (balanced BCAAs: Leu 30.4, Ile 19.7, Val 17.4 g/kg)	Leu-L (Leu deficient: 20.9 g/kg)	Plasma free Ile, 2 h after feeding (µg/mL)	36.40 ± 4.82	47.20 ± 13.08	3	3	Hedges' g	-0.88	-2.59 to 0.83	Lower measured value with the balanced BCAA profile; 95% CI included 0 (imprecise estimate)	Not applicable - effect size estimated after converting SE to SD
Wang et al. (2023)	Turbot (Scophthalmus maximus)	BCAA balancing to estimated requirement levels	Control (balanced BCAAs: Leu 30.4, Ile 19.7, Val 17.4 g/kg)	Leu-L (Leu deficient: 20.9 g/kg)	Plasma free Val, 2 h after feeding (µg/mL)	40.85 ± 4.31	48.65 ± 10.62	3	3	Hedges' g	-0.77	-2.46 to 0.92	Lower measured value with the balanced BCAA profile; 95% CI included 0 (imprecise estimate)	Not applicable - effect size estimated after converting SE to SD
Wang et al. (2023)	Turbot (Scophthalmus maximus)	BCAA balancing to estimated requirement levels	Control (balanced BCAAs: Leu 30.4, Ile 19.7, Val 17.4 g/kg)	Leu-L (Leu deficient: 20.9 g/kg)	Plasma free Leu, 6 h after feeding (µg/mL)	54.19 ± 16.26	22.07 ± 9.08	3	3	Hedges' g	1.95	-0.14 to 4.05	Higher measured value with the balanced BCAA profile; 95% CI included 0 (imprecise estimate)	Not applicable - effect size estimated after converting SE to SD
Wang et al. (2023)	Turbot (Scophthalmus maximus)	BCAA balancing to estimated requirement levels	Control (balanced BCAAs: Leu 30.4, Ile 19.7, Val 17.4 g/kg)	Leu-L (Leu deficient: 20.9 g/kg)	Plasma free Ile, 6 h after feeding (µg/mL)	37.73 ± 13.41	38.26 ± 10.20	3	3	Hedges' g	-0.04	-1.64 to 1.56	Lower measured value with the balanced BCAA profile; 95% CI included 0 (imprecise estimate)	Not applicable - effect size estimated after converting SE to SD
Wang et al. (2023)	Turbot (Scophthalmus maximus)	BCAA balancing to estimated requirement levels	Control (balanced BCAAs: Leu 30.4, Ile 19.7, Val 17.4 g/kg)	Leu-L (Leu deficient: 20.9 g/kg)	Plasma free Val, 6 h after feeding (µg/mL)	40.54 ± 11.73	49.35 ± 12.61	3	3	Hedges' g	-0.58	-2.23 to 1.07	Lower measured value with the balanced BCAA profile; 95% CI included 0 (imprecise estimate)	Not applicable - effect size estimated after converting SE to SD
Wang et al. (2023)	Turbot (Scophthalmus maximus)	BCAA balancing to estimated requirement levels	Control (balanced BCAAs: Leu 30.4, Ile 19.7, Val 17.4 g/kg)	Leu-L (Leu deficient: 20.9 g/kg)	Plasma free Leu, 24 h after feeding (µg/mL)	10.42 ± 0.73	11.13 ± 3.17	3	3	Hedges' g	-0.25	-1.86 to 1.36	Lower measured value with the balanced BCAA profile; 95% CI included 0 (imprecise estimate)	Not applicable - effect size estimated after converting SE to SD
Wang et al. (2023)	Turbot (Scophthalmus maximus)	BCAA balancing to estimated requirement levels	Control (balanced BCAAs: Leu 30.4, Ile 19.7, Val 17.4 g/kg)	Leu-L (Leu deficient: 20.9 g/kg)	Plasma free Ile, 24 h after feeding (µg/mL)	5.64 ± 0.64	6.19 ± 1.77	3	3	Hedges' g	-0.33	-1.95 to 1.29	Lower measured value with the balanced BCAA profile; 95% CI included 0 (imprecise estimate)	Not applicable - effect size estimated after converting SE to SD
Wang et al. (2023)	Turbot (Scophthalmus maximus)	BCAA balancing to estimated requirement levels	Control (balanced BCAAs: Leu 30.4, Ile 19.7, Val 17.4 g/kg)	Leu-L (Leu deficient: 20.9 g/kg)	Plasma free Val, 24 h after feeding (µg/mL)	10.17 ± 1.20	10.56 ± 1.80	3	3	Hedges' g	-0.2	-1.81 to 1.40	Lower measured value with the balanced BCAA profile; 95% CI included 0 (imprecise estimate)	Not applicable - effect size estimated after converting SE to SD
Wang et al. (2023)	Turbot (Scophthalmus maximus)	BCAA balancing to estimated requirement levels	Control (balanced BCAAs: Leu 30.4, Ile 19.7, Val 17.4 g/kg)	Leu-L (Leu deficient: 20.9 g/kg)	Liver free Leu, 2 h after feeding (µg/g)	740.38 ± 282.01	932.79 ± 212.78	3	3	Hedges' g	-0.62	-2.27 to 1.04	Lower measured value with the balanced BCAA profile; 95% CI included 0 (imprecise estimate)	Not applicable - effect size estimated after converting SE to SD
Wang et al. (2023)	Turbot (Scophthalmus maximus)	BCAA balancing to estimated requirement levels	Control (balanced BCAAs: Leu 30.4, Ile 19.7, Val 17.4 g/kg)	Leu-L (Leu deficient: 20.9 g/kg)	Liver free Ile, 2 h after feeding (µg/g)	386.80 ± 135.31	542.40 ± 165.67	3	3	Hedges' g	-0.82	-2.52 to 0.88	Lower measured value with the balanced BCAA profile; 95% CI included 0 (imprecise estimate)	Not applicable - effect size estimated after converting SE to SD
Wang et al. (2023)	Turbot (Scophthalmus maximus)	BCAA balancing to estimated requirement levels	Control (balanced BCAAs: Leu 30.4, Ile 19.7, Val 17.4 g/kg)	Leu-L (Leu deficient: 20.9 g/kg)	Liver free Val, 2 h after feeding (µg/g)	631.12 ± 247.86	962.63 ± 182.38	3	3	Hedges' g	-1.22	-3.03 to 0.59	Lower measured value with the balanced BCAA profile; 95% CI included 0 (imprecise estimate)	Not applicable - effect size estimated after converting SE to SD
Wang et al. (2023)	Turbot (Scophthalmus maximus)	BCAA balancing to estimated requirement levels	Control (balanced BCAAs: Leu 30.4, Ile 19.7, Val 17.4 g/kg)	Leu-L (Leu deficient: 20.9 g/kg)	Liver free Leu, 6 h after feeding (µg/g)	646.81 ± 172.81	707.05 ± 129.30	3	3	Hedges' g	-0.32	-1.93 to 1.30	Lower measured value with the balanced BCAA profile; 95% CI included 0 (imprecise estimate)	Not applicable - effect size estimated after converting SE to SD
Wang et al. (2023)	Turbot (Scophthalmus maximus)	BCAA balancing to estimated requirement levels	Control (balanced BCAAs: Leu 30.4, Ile 19.7, Val 17.4 g/kg)	Leu-L (Leu deficient: 20.9 g/kg)	Liver free Ile, 6 h after feeding (µg/g)	344.65 ± 108.51	407.99 ± 59.39	3	3	Hedges' g	-0.58	-2.23 to 1.07	Lower measured value with the balanced BCAA profile; 95% CI included 0 (imprecise estimate)	Not applicable - effect size estimated after converting SE to SD
Wang et al. (2023)	Turbot (Scophthalmus maximus)	BCAA balancing to estimated requirement levels	Control (balanced BCAAs: Leu 30.4, Ile 19.7, Val 17.4 g/kg)	Leu-L (Leu deficient: 20.9 g/kg)	Liver free Val, 6 h after feeding (µg/g)	611.17 ± 136.33	809.13 ± 83.40	3	3	Hedges' g	-1.4	-3.27 to 0.47	Lower measured value with the balanced BCAA profile; 95% CI included 0 (imprecise estimate)	Not applicable - effect size estimated after converting SE to SD
Wang et al. (2023)	Turbot (Scophthalmus maximus)	BCAA balancing to estimated requirement levels	Control (balanced BCAAs: Leu 30.4, Ile 19.7, Val 17.4 g/kg)	Leu-L (Leu deficient: 20.9 g/kg)	Liver free Leu, 24 h after feeding (µg/g)	1367.52 ± 221.67	1054.28 ± 229.79	3	3	Hedges' g	1.11	-0.67 to 2.89	Higher measured value with the balanced BCAA profile; 95% CI included 0 (imprecise estimate)	Not applicable - effect size estimated after converting SE to SD
Wang et al. (2023)	Turbot (Scophthalmus maximus)	BCAA balancing to estimated requirement levels	Control (balanced BCAAs: Leu 30.4, Ile 19.7, Val 17.4 g/kg)	Leu-L (Leu deficient: 20.9 g/kg)	Liver free Ile, 24 h after feeding (µg/g)	657.02 ± 91.40	495.51 ± 68.54	3	3	Hedges' g	1.6	-0.35 to 3.55	Higher measured value with the balanced BCAA profile; 95% CI included 0 (imprecise estimate)	Not applicable - effect size estimated after converting SE to SD
Wang et al. (2023)	Turbot (Scophthalmus maximus)	BCAA balancing to estimated requirement levels	Control (balanced BCAAs: Leu 30.4, Ile 19.7, Val 17.4 g/kg)	Leu-L (Leu deficient: 20.9 g/kg)	Liver free Val, 24 h after feeding (µg/g)	1163.91 ± 145.84	953.66 ± 171.01	3	3	Hedges' g	1.06	-0.70 to 2.82	Higher measured value with the balanced BCAA profile; 95% CI included 0 (imprecise estimate)	Not applicable - effect size estimated after converting SE to SD
Wang et al. (2023)	Turbot (Scophthalmus maximus)	BCAA balancing to estimated requirement levels	Control (balanced BCAAs: Leu 30.4, Ile 19.7, Val 17.4 g/kg)	Leu-L (Leu deficient: 20.9 g/kg)	Muscle free Leu, 2 h after feeding (µg/g)	144.73 ± 8.12	53.40 ± 34.95	3	3	Hedges' g	2.88	0.32 to 5.44	Higher measured value with the balanced BCAA profile; 95% CI excluded 0	Not applicable - effect size estimated after converting SE to SD

Study	Species	Strategy	Intervention	Comparator	Outcome and unit	Intervention mean ± SD	Comparator mean ± SD	n intervention (tanks)	n comparator (tanks)	Effect-size metric	Effect size	95% CI	Interpretation	Reason not estimated
Wang et al. (2023)	Turbot (Scophthalmus maximus)	BCAA balancing to estimated requirement levels	Control (balanced BCAAs: Leu 30.4, Ile 19.7, Val 17.4 g/kg)	Leu-L (Leu deficient: 20.9 g/kg)	Muscle free Ile, 2 h after feeding (μg/g)	103.55 ± 10.25	112.04 ± 19.40	3	3	Hedges' g	-0.44	-2.07 to 1.19	Lower measured value with the balanced BCAA profile; 95% CI included 0 (imprecise estimate)	Not applicable - effect size estimated after converting SE to SD
Wang et al. (2023)	Turbot (Scophthalmus maximus)	BCAA balancing to estimated requirement levels	Control (balanced BCAAs: Leu 30.4, Ile 19.7, Val 17.4 g/kg)	Leu-L (Leu deficient: 20.9 g/kg)	Muscle free Val, 2 h after feeding (μg/g)	1940.63 ± 202.27	2124.21 ± 246.92	3	3	Hedges' g	-0.65	-2.31 to 1.01	Lower measured value with the balanced BCAA profile; 95% CI included 0 (imprecise estimate)	Not applicable - effect size estimated after converting SE to SD
Wang et al. (2023)	Turbot (Scophthalmus maximus)	BCAA balancing to estimated requirement levels	Control (balanced BCAAs: Leu 30.4, Ile 19.7, Val 17.4 g/kg)	Leu-L (Leu deficient: 20.9 g/kg)	Muscle free Leu, 6 h after feeding (μg/g)	169.94 ± 42.61	69.11 ± 11.69	3	3	Hedges' g	2.58	0.18 to 4.98	Higher measured value with the balanced BCAA profile; 95% CI excluded 0	Not applicable - effect size estimated after converting SE to SD
Wang et al. (2023)	Turbot (Scophthalmus maximus)	BCAA balancing to estimated requirement levels	Control (balanced BCAAs: Leu 30.4, Ile 19.7, Val 17.4 g/kg)	Leu-L (Leu deficient: 20.9 g/kg)	Muscle free Ile, 6 h after feeding (μg/g)	108.29 ± 25.50	136.45 ± 11.28	3	3	Hedges' g	-1.14	-2.93 to 0.64	Lower measured value with the balanced BCAA profile; 95% CI included 0 (imprecise estimate)	Not applicable - effect size estimated after converting SE to SD
Wang et al. (2023)	Turbot (Scophthalmus maximus)	BCAA balancing to estimated requirement levels	Control (balanced BCAAs: Leu 30.4, Ile 19.7, Val 17.4 g/kg)	Leu-L (Leu deficient: 20.9 g/kg)	Muscle free Val, 6 h after feeding (μg/g)	2219.39 ± 534.30	2074.76 ± 488.37	3	3	Hedges' g	0.23	-1.38 to 1.83	Higher measured value with the balanced BCAA profile; 95% CI included 0 (imprecise estimate)	Not applicable - effect size estimated after converting SE to SD
Wang et al. (2023)	Turbot (Scophthalmus maximus)	BCAA balancing to estimated requirement levels	Control (balanced BCAAs: Leu 30.4, Ile 19.7, Val 17.4 g/kg)	Leu-L (Leu deficient: 20.9 g/kg)	Muscle free Leu, 24 h after feeding (μg/g)	69.30 ± 21.20	74.11 ± 7.95	3	3	Hedges' g	-0.24	-1.85 to 1.37	Lower measured value with the balanced BCAA profile; 95% CI included 0 (imprecise estimate)	Not applicable - effect size estimated after converting SE to SD
Wang et al. (2023)	Turbot (Scophthalmus maximus)	BCAA balancing to estimated requirement levels	Control (balanced BCAAs: Leu 30.4, Ile 19.7, Val 17.4 g/kg)	Leu-L (Leu deficient: 20.9 g/kg)	Muscle free Ile, 24 h after feeding (μg/g)	48.99 ± 18.74	50.36 ± 11.12	3	3	Hedges' g	-0.07	-1.67 to 1.53	Lower measured value with the balanced BCAA profile; 95% CI included 0 (imprecise estimate)	Not applicable - effect size estimated after converting SE to SD
Wang et al. (2023)	Turbot (Scophthalmus maximus)	BCAA balancing to estimated requirement levels	Control (balanced BCAAs: Leu 30.4, Ile 19.7, Val 17.4 g/kg)	Leu-L (Leu deficient: 20.9 g/kg)	Muscle free Val, 24 h after feeding (μg/g)	1772.95 ± 160.06	1996.23 ± 539.60	3	3	Hedges' g	-0.45	-2.08 to 1.18	Lower measured value with the balanced BCAA profile; 95% CI included 0 (imprecise estimate)	Not applicable - effect size estimated after converting SE to SD
Wang et al. (2023)	Turbot (Scophthalmus maximus)	BCAA balancing to estimated requirement levels	Control (balanced BCAAs: Leu 30.4, Ile 19.7, Val 17.4 g/kg)	Leu-H (Leu excess: 75.5 g/kg)	Survival (%)	98.70 ± 2.25	97.30 ± 4.68	3	3	Hedges' g	0.31	-1.31 to 1.92	Higher, more favorable value with the balanced BCAA profile; 95% CI included 0 (imprecise estimate)	Not applicable - effect size estimated after converting SE to SD
Wang et al. (2023)	Turbot (Scophthalmus maximus)	BCAA balancing to estimated requirement levels	Control (balanced BCAAs: Leu 30.4, Ile 19.7, Val 17.4 g/kg)	Leu-H (Leu excess: 75.5 g/kg)	Weight gain (%)	117.80 ± 13.16	106.70 ± 11.60	3	3	Hedges' g	0.72	-0.96 to 2.39	Higher, more favorable value with the balanced BCAA profile; 95% CI included 0 (imprecise estimate)	Not applicable - effect size estimated after converting SE to SD
Wang et al. (2023)	Turbot (Scophthalmus maximus)	BCAA balancing to estimated requirement levels	Control (balanced BCAAs: Leu 30.4, Ile 19.7, Val 17.4 g/kg)	Leu-H (Leu excess: 75.5 g/kg)	Specific growth rate (%/day)	1.29 ± 0.10	1.21 ± 0.09	3	3	Hedges' g	0.67	-1.00 to 2.34	Higher, more favorable value with the balanced BCAA profile; 95% CI included 0 (imprecise estimate)	Not applicable - effect size estimated after converting SE to SD
Wang et al. (2023)	Turbot (Scophthalmus maximus)	BCAA balancing to estimated requirement levels	Control (balanced BCAAs: Leu 30.4, Ile 19.7, Val 17.4 g/kg)	Leu-H (Leu excess: 75.5 g/kg)	Feed intake (% body weight/day)	1.12 ± 0.03	1.04 ± 0.02	3	3	Hedges' g	2.34	0.06 to 4.61	Higher measured value with the balanced BCAA profile; 95% CI excluded 0	Not applicable - effect size estimated after converting SE to SD
Wang et al. (2023)	Turbot (Scophthalmus maximus)	BCAA balancing to estimated requirement levels	Control (balanced BCAAs: Leu 30.4, Ile 19.7, Val 17.4 g/kg)	Leu-H (Leu excess: 75.5 g/kg)	Feed efficiency	1.11 ± 0.05	1.12 ± 0.05	3	3	Hedges' g	-0.15	-1.76 to 1.45	Lower, less favorable value with the balanced BCAA profile; 95% CI included 0 (imprecise estimate)	Not applicable - effect size estimated after converting SE to SD
Wang et al. (2023)	Turbot (Scophthalmus maximus)	BCAA balancing to estimated requirement levels	Control (balanced BCAAs: Leu 30.4, Ile 19.7, Val 17.4 g/kg)	Leu-H (Leu excess: 75.5 g/kg)	Protein efficiency ratio	2.17 ± 0.16	2.20 ± 0.10	3	3	Hedges' g	-0.18	-1.79 to 1.42	Lower, less favorable value with the balanced BCAA profile; 95% CI included 0 (imprecise estimate)	Not applicable - effect size estimated after converting SE to SD
Wang et al. (2023)	Turbot (Scophthalmus maximus)	BCAA balancing to estimated requirement levels	Control (balanced BCAAs: Leu 30.4, Ile 19.7, Val 17.4 g/kg)	Leu-H (Leu excess: 75.5 g/kg)	Protein productive value (%)	34.40 ± 2.08	33.40 ± 2.42	3	3	Hedges' g	0.35	-1.26 to 1.97	Higher, more favorable value with the balanced BCAA profile; 95% CI included 0 (imprecise estimate)	Not applicable - effect size estimated after converting SE to SD
Wang et al. (2023)	Turbot (Scophthalmus maximus)	BCAA balancing to estimated requirement levels	Control (balanced BCAAs: Leu 30.4, Ile 19.7, Val 17.4 g/kg)	Leu-H (Leu excess: 75.5 g/kg)	Moisture (g/kg)	774.70 ± 5.54	779.00 ± 3.64	3	3	Hedges' g	-0.73	-2.41 to 0.95	Lower measured value with the balanced BCAA profile; 95% CI included 0 (imprecise estimate)	Not applicable - effect size estimated after converting SE to SD
Wang et al. (2023)	Turbot (Scophthalmus maximus)	BCAA balancing to estimated requirement levels	Control (balanced BCAAs: Leu 30.4, Ile 19.7, Val 17.4 g/kg)	Leu-H (Leu excess: 75.5 g/kg)	Crude protein (g/kg)	153.00 ± 2.60	148.70 ± 1.73	3	3	Hedges' g	1.56	-0.37 to 3.49	Higher measured value with the balanced BCAA profile; 95% CI included 0 (imprecise estimate)	Not applicable - effect size estimated after converting SE to SD
Wang et al. (2023)	Turbot (Scophthalmus maximus)	BCAA balancing to estimated requirement levels	Control (balanced BCAAs: Leu 30.4, Ile 19.7, Val 17.4 g/kg)	Leu-H (Leu excess: 75.5 g/kg)	Crude lipid (g/kg)	26.10 ± 4.85	24.30 ± 1.73	3	3	Hedges' g	0.4	-1.23 to 2.02	Higher measured value with the balanced BCAA profile; 95% CI included 0 (imprecise estimate)	Not applicable - effect size estimated after converting SE to SD
Wang et al. (2023)	Turbot (Scophthalmus maximus)	BCAA balancing to estimated requirement levels	Control (balanced BCAAs: Leu 30.4, Ile 19.7, Val 17.4 g/kg)	Leu-H (Leu excess: 75.5 g/kg)	Ash (g/kg)	37.20 ± 2.08	37.50 ± 1.56	3	3	Hedges' g	-0.13	-1.73 to 1.47	Lower measured value with the balanced BCAA profile; 95% CI included 0 (imprecise estimate)	Not applicable - effect size estimated after converting SE to SD
Wang et al. (2023)	Turbot (Scophthalmus maximus)	BCAA balancing to estimated requirement levels	Control (balanced BCAAs: Leu 30.4, Ile 19.7, Val 17.4 g/kg)	Leu-H (Leu excess: 75.5 g/kg)	Plasma free Leu, 2 h after feeding (μg/mL)	57.23 ± 7.60	225.56 ± 13.13	3	3	Hedges' g	-12.55	-21.40 to -3.71	Lower measured value with the balanced BCAA profile; 95% CI excluded 0	Not applicable - effect size estimated after converting SE to SD
Wang et al. (2023)	Turbot (Scophthalmus maximus)	BCAA balancing to estimated requirement levels	Control (balanced BCAAs: Leu 30.4, Ile 19.7, Val 17.4 g/kg)	Leu-H (Leu excess: 75.5 g/kg)	Plasma free Ile, 2 h after feeding (μg/mL)	36.40 ± 4.82	31.90 ± 11.33	3	3	Hedges' g	0.41	-1.21 to 2.04	Higher measured value with the balanced BCAA profile; 95% CI included 0 (imprecise estimate)	Not applicable - effect size estimated after converting SE to SD
Wang et al. (2023)	Turbot (Scophthalmus maximus)	BCAA balancing to estimated requirement levels	Control (balanced BCAAs: Leu 30.4, Ile 19.7, Val 17.4 g/kg)	Leu-H (Leu excess: 75.5 g/kg)	Plasma free Val, 2 h after feeding (μg/mL)	40.85 ± 4.31	35.84 ± 9.54	3	3	Hedges' g	0.54	-1.10 to 2.18	Higher measured value with the balanced BCAA profile; 95% CI included 0 (imprecise estimate)	Not applicable - effect size estimated after converting SE to SD
Wang et al. (2023)	Turbot (Scophthalmus maximus)	BCAA balancing to estimated requirement levels	Control (balanced BCAAs: Leu 30.4, Ile 19.7, Val 17.4 g/kg)	Leu-H (Leu excess: 75.5 g/kg)	Plasma free Leu, 6 h after feeding (μg/mL)	54.19 ± 16.26	419.36 ± 87.88	3	3	Hedges' g	-4.62	-8.20 to -1.04	Lower measured value with the balanced BCAA profile; 95% CI excluded 0	Not applicable - effect size estimated after converting SE to SD
Wang et al. (2023)	Turbot (Scophthalmus maximus)	BCAA balancing to estimated requirement levels	Control (balanced BCAAs: Leu 30.4, Ile 19.7, Val 17.4 g/kg)	Leu-H (Leu excess: 75.5 g/kg)	Plasma free Ile, 6 h after feeding (μg/mL)	37.73 ± 13.41	61.31 ± 17.37	3	3	Hedges' g	-1.22	-3.02 to 0.59	Lower measured value with the balanced BCAA profile; 95% CI included 0 (imprecise estimate)	Not applicable - effect size estimated after converting SE to SD
Wang et al. (2023)	Turbot (Scophthalmus maximus)	BCAA balancing to estimated requirement levels	Control (balanced BCAAs: Leu 30.4, Ile 19.7, Val 17.4 g/kg)	Leu-H (Leu excess: 75.5 g/kg)	Plasma free Val, 6 h after feeding (μg/mL)	40.54 ± 11.73	60.39 ± 16.18	3	3	Hedges' g	-1.12	-2.90 to 0.66	Lower measured value with the balanced BCAA profile; 95% CI included 0 (imprecise estimate)	Not applicable - effect size estimated after converting SE to SD
Wang et al. (2023)	Turbot (Scophthalmus maximus)	BCAA balancing to estimated requirement levels	Control (balanced BCAAs: Leu 30.4, Ile 19.7, Val 17.4 g/kg)	Leu-H (Leu excess: 75.5 g/kg)	Plasma free Leu, 24 h after feeding (μg/mL)	10.42 ± 0.73	19.34 ± 7.21	3	3	Hedges' g	-1.39	-3.26 to 0.48	Lower measured value with the balanced BCAA profile; 95% CI included 0 (imprecise estimate)	Not applicable - effect size estimated after converting SE to SD
Wang et al. (2023)	Turbot (Scophthalmus maximus)	BCAA balancing to estimated requirement levels	Control (balanced BCAAs: Leu 30.4, Ile 19.7, Val 17.4 g/kg)	Leu-H (Leu excess: 75.5 g/kg)	Plasma free Ile, 24 h after feeding (μg/mL)	5.64 ± 0.64	5.30 ± 1.26	3	3	Hedges' g	0.27	-1.34 to 1.88	Higher measured value with the balanced BCAA profile; 95% CI included 0 (imprecise estimate)	Not applicable - effect size estimated after converting SE to SD
Wang et al. (2023)	Turbot (Scophthalmus maximus)	BCAA balancing to estimated requirement levels	Control (balanced BCAAs: Leu 30.4, Ile 19.7, Val 17.4 g/kg)	Leu-H (Leu excess: 75.5 g/kg)	Plasma free Val, 24 h after feeding (μg/mL)	10.17 ± 1.20	9.63 ± 1.52	3	3	Hedges' g	0.32	-1.30 to 1.93	Higher measured value with the balanced BCAA profile; 95% CI included 0 (imprecise estimate)	Not applicable - effect size estimated after converting SE to SD
Wang et al. (2023)	Turbot (Scophthalmus maximus)	BCAA balancing to estimated requirement levels	Control (balanced BCAAs: Leu 30.4, Ile 19.7, Val 17.4 g/kg)	Leu-H (Leu excess: 75.5 g/kg)	Liver free Leu, 2 h after feeding (μg/g)	740.38 ± 282.01	1641.08 ± 152.23	3	3	Hedges' g	-3.18	-5.90 to -0.46	Lower measured value with the balanced BCAA profile; 95% CI excluded 0	Not applicable - effect size estimated after converting SE to SD
Wang et al. (2023)	Turbot (Scophthalmus maximus)	BCAA balancing to estimated requirement levels	Control (balanced BCAAs: Leu 30.4, Ile 19.7, Val 17.4 g/kg)	Leu-H (Leu excess: 75.5 g/kg)	Liver free Ile, 2 h after feeding (μg/g)	386.80 ± 135.31	511.02 ± 65.11	3	3	Hedges' g	-0.94	-2.66 to 0.79	Lower measured value with the balanced BCAA profile; 95% CI included 0 (imprecise estimate)	Not applicable - effect size estimated after converting SE to SD
Wang et al. (2023)	Turbot (Scophthalmus maximus)	BCAA balancing to estimated requirement levels	Control (balanced BCAAs: Leu 30.4, Ile 19.7, Val 17.4 g/kg)	Leu-H (Leu excess: 75.5 g/kg)	Liver free Val, 2 h after feeding (μg/g)	631.12 ± 247.86	818.41 ± 98.59	3	3	Hedges' g	-0.79	-2.49 to 0.90	Lower measured value with the balanced BCAA profile; 95% CI included 0 (imprecise estimate)	Not applicable - effect size estimated after converting SE to SD
Wang et al. (2023)	Turbot (Scophthalmus maximus)	BCAA balancing to estimated requirement levels	Control (balanced BCAAs: Leu 30.4, Ile 19.7, Val 17.4 g/kg)	Leu-H (Leu excess: 75.5 g/kg)	Liver free Leu, 6 h after feeding (μg/g)	646.81 ± 172.81	1452.26 ± 261.63	3	3	Hedges' g	-2.91	-5.48 to -0.33	Lower measured value with the balanced BCAA profile; 95% CI excluded 0	Not applicable - effect size estimated after converting SE to SD

Study	Species	Strategy	Intervention	Comparator	Outcome and unit	Intervention mean ± SD	Comparator mean ± SD	n intervention (tanks)	n comparator (tanks)	Effect-size metric	Effect size	95% CI	Interpretation	Reason not estimated
Wang et al. (2023)	Turbot (Scophthalmus maximus)	BCAA balancing to estimated requirement levels	Control (balanced BCAAs: Leu 30.4, Ile 19.7, Val 17.4 g/kg)	Leu-H (Leu excess: 75.5 g/kg)	Liver free Ile, 6 h after feeding (µg/g)	344.65 ± 108.51	460.81 ± 146.67	3	3	Hedges' g	-0.72	-2.40 to 0.96	Lower measured value with the balanced BCAA profile; 95% CI included 0 (imprecise estimate)	Not applicable - effect size estimated after converting SE to SD
Wang et al. (2023)	Turbot (Scophthalmus maximus)	BCAA balancing to estimated requirement levels	Control (balanced BCAAs: Leu 30.4, Ile 19.7, Val 17.4 g/kg)	Leu-H (Leu excess: 75.5 g/kg)	Liver free Val, 6 h after feeding (µg/g)	611.17 ± 136.33	753.11 ± 150.83	3	3	Hedges' g	-0.79	-2.48 to 0.90	Lower measured value with the balanced BCAA profile; 95% CI included 0 (imprecise estimate)	Not applicable - effect size estimated after converting SE to SD
Wang et al. (2023)	Turbot (Scophthalmus maximus)	BCAA balancing to estimated requirement levels	Control (balanced BCAAs: Leu 30.4, Ile 19.7, Val 17.4 g/kg)	Leu-H (Leu excess: 75.5 g/kg)	Liver free Leu, 24 h after feeding (µg/g)	1367.52 ± 221.67	1270.64 ± 94.52	3	3	Hedges' g	0.45	-1.18 to 2.09	Higher measured value with the balanced BCAA profile; 95% CI included 0 (imprecise estimate)	Not applicable - effect size estimated after converting SE to SD
Wang et al. (2023)	Turbot (Scophthalmus maximus)	BCAA balancing to estimated requirement levels	Control (balanced BCAAs: Leu 30.4, Ile 19.7, Val 17.4 g/kg)	Leu-H (Leu excess: 75.5 g/kg)	Liver free Ile, 24 h after feeding (µg/g)	657.02 ± 91.40	588.33 ± 32.77	3	3	Hedges' g	0.8	-0.89 to 2.49	Higher measured value with the balanced BCAA profile; 95% CI included 0 (imprecise estimate)	Not applicable - effect size estimated after converting SE to SD
Wang et al. (2023)	Turbot (Scophthalmus maximus)	BCAA balancing to estimated requirement levels	Control (balanced BCAAs: Leu 30.4, Ile 19.7, Val 17.4 g/kg)	Leu-H (Leu excess: 75.5 g/kg)	Liver free Val, 24 h after feeding (µg/g)	1163.91 ± 145.84	1086.95 ± 81.49	3	3	Hedges' g	0.52	-1.12 to 2.16	Higher measured value with the balanced BCAA profile; 95% CI included 0 (imprecise estimate)	Not applicable - effect size estimated after converting SE to SD
Wang et al. (2023)	Turbot (Scophthalmus maximus)	BCAA balancing to estimated requirement levels	Control (balanced BCAAs: Leu 30.4, Ile 19.7, Val 17.4 g/kg)	Leu-H (Leu excess: 75.5 g/kg)	Muscle free Leu, 2 h after feeding (µg/g)	144.73 ± 8.12	546.82 ± 39.04	3	3	Hedges' g	-11.41	-19.47 to -3.34	Lower measured value with the balanced BCAA profile; 95% CI excluded 0	Not applicable - effect size estimated after converting SE to SD
Wang et al. (2023)	Turbot (Scophthalmus maximus)	BCAA balancing to estimated requirement levels	Control (balanced BCAAs: Leu 30.4, Ile 19.7, Val 17.4 g/kg)	Leu-H (Leu excess: 75.5 g/kg)	Muscle free Ile, 2 h after feeding (µg/g)	103.55 ± 10.25	104.42 ± 8.61	3	3	Hedges' g	-0.07	-1.67 to 1.53	Lower measured value with the balanced BCAA profile; 95% CI included 0 (imprecise estimate)	Not applicable - effect size estimated after converting SE to SD
Wang et al. (2023)	Turbot (Scophthalmus maximus)	BCAA balancing to estimated requirement levels	Control (balanced BCAAs: Leu 30.4, Ile 19.7, Val 17.4 g/kg)	Leu-H (Leu excess: 75.5 g/kg)	Muscle free Val, 2 h after feeding (µg/g)	1940.63 ± 202.27	2050.21 ± 233.12	3	3	Hedges' g	-0.4	-2.03 to 1.22	Lower measured value with the balanced BCAA profile; 95% CI included 0 (imprecise estimate)	Not applicable - effect size estimated after converting SE to SD
Wang et al. (2023)	Turbot (Scophthalmus maximus)	BCAA balancing to estimated requirement levels	Control (balanced BCAAs: Leu 30.4, Ile 19.7, Val 17.4 g/kg)	Leu-H (Leu excess: 75.5 g/kg)	Muscle free Leu, 6 h after feeding (µg/g)	169.94 ± 42.61	843.85 ± 56.83	3	3	Hedges' g	-10.73	-18.34 to -3.13	Lower measured value with the balanced BCAA profile; 95% CI excluded 0	Not applicable - effect size estimated after converting SE to SD
Wang et al. (2023)	Turbot (Scophthalmus maximus)	BCAA balancing to estimated requirement levels	Control (balanced BCAAs: Leu 30.4, Ile 19.7, Val 17.4 g/kg)	Leu-H (Leu excess: 75.5 g/kg)	Muscle free Ile, 6 h after feeding (µg/g)	108.29 ± 25.50	136.96 ± 21.49	3	3	Hedges' g	-0.97	-2.71 to 0.76	Lower measured value with the balanced BCAA profile; 95% CI included 0 (imprecise estimate)	Not applicable - effect size estimated after converting SE to SD
Wang et al. (2023)	Turbot (Scophthalmus maximus)	BCAA balancing to estimated requirement levels	Control (balanced BCAAs: Leu 30.4, Ile 19.7, Val 17.4 g/kg)	Leu-H (Leu excess: 75.5 g/kg)	Muscle free Val, 6 h after feeding (µg/g)	2219.39 ± 534.30	1708.33 ± 397.97	3	3	Hedges' g	0.87	-0.84 to 2.58	Higher measured value with the balanced BCAA profile; 95% CI included 0 (imprecise estimate)	Not applicable - effect size estimated after converting SE to SD
Wang et al. (2023)	Turbot (Scophthalmus maximus)	BCAA balancing to estimated requirement levels	Control (balanced BCAAs: Leu 30.4, Ile 19.7, Val 17.4 g/kg)	Leu-H (Leu excess: 75.5 g/kg)	Muscle free Leu, 24 h after feeding (µg/g)	69.30 ± 21.20	114.88 ± 27.44	3	3	Hedges' g	-1.49	-3.39 to 0.42	Lower measured value with the balanced BCAA profile; 95% CI included 0 (imprecise estimate)	Not applicable - effect size estimated after converting SE to SD
Wang et al. (2023)	Turbot (Scophthalmus maximus)	BCAA balancing to estimated requirement levels	Control (balanced BCAAs: Leu 30.4, Ile 19.7, Val 17.4 g/kg)	Leu-H (Leu excess: 75.5 g/kg)	Muscle free Ile, 24 h after feeding (µg/g)	48.99 ± 18.74	81.72 ± 24.32	3	3	Hedges' g	-1.21	-3.01 to 0.60	Lower measured value with the balanced BCAA profile; 95% CI included 0 (imprecise estimate)	Not applicable - effect size estimated after converting SE to SD
Wang et al. (2023)	Turbot (Scophthalmus maximus)	BCAA balancing to estimated requirement levels	Control (balanced BCAAs: Leu 30.4, Ile 19.7, Val 17.4 g/kg)	Leu-H (Leu excess: 75.5 g/kg)	Muscle free Val, 24 h after feeding (µg/g)	1772.95 ± 160.06	1705.03 ± 189.71	3	3	Hedges' g	0.31	-1.31 to 1.92	Higher measured value with the balanced BCAA profile; 95% CI included 0 (imprecise estimate)	Not applicable - effect size estimated after converting SE to SD
Wang et al. (2023)	Turbot (Scophthalmus maximus)	BCAA balancing to estimated requirement levels	Control (balanced BCAAs: Leu 30.4, Ile 19.7, Val 17.4 g/kg)	Ile-H (Ile excess: 52.6 g/kg)	Survival (%)	98.70 ± 2.25	100.00 ± 0.00	3	3	Hedges' g	-0.65	-2.32 to 1.01	Lower, less favorable value with the balanced BCAA profile; 95% CI included 0 (imprecise estimate)	Not applicable - effect size estimated after converting SE to SD
Wang et al. (2023)	Turbot (Scophthalmus maximus)	BCAA balancing to estimated requirement levels	Control (balanced BCAAs: Leu 30.4, Ile 19.7, Val 17.4 g/kg)	Ile-H (Ile excess: 52.6 g/kg)	Weight gain (%)	117.80 ± 13.16	116.50 ± 9.18	3	3	Hedges' g	0.09	-1.51 to 1.69	Higher, more favorable value with the balanced BCAA profile; 95% CI included 0 (imprecise estimate)	Not applicable - effect size estimated after converting SE to SD
Wang et al. (2023)	Turbot (Scophthalmus maximus)	BCAA balancing to estimated requirement levels	Control (balanced BCAAs: Leu 30.4, Ile 19.7, Val 17.4 g/kg)	Ile-H (Ile excess: 52.6 g/kg)	Specific growth rate (%/day)	1.29 ± 0.10	1.29 ± 0.07	3	3	Hedges' g	0.0	-1.60 to 1.60	No observed mean difference; 95% CI included 0 (imprecise estimate)	Not applicable - effect size estimated after converting SE to SD
Wang et al. (2023)	Turbot (Scophthalmus maximus)	BCAA balancing to estimated requirement levels	Control (balanced BCAAs: Leu 30.4, Ile 19.7, Val 17.4 g/kg)	Ile-H (Ile excess: 52.6 g/kg)	Feed intake (% body weight/day)	1.12 ± 0.03	1.04 ± 0.05	3	3	Hedges' g	1.45	-0.44 to 3.34	Higher measured value with the balanced BCAA profile; 95% CI included 0 (imprecise estimate)	Not applicable - effect size estimated after converting SE to SD
Wang et al. (2023)	Turbot (Scophthalmus maximus)	BCAA balancing to estimated requirement levels	Control (balanced BCAAs: Leu 30.4, Ile 19.7, Val 17.4 g/kg)	Ile-H (Ile excess: 52.6 g/kg)	Feed efficiency	1.11 ± 0.05	1.19 ± 0.09	3	3	Hedges' g	-0.9	-2.61 to 0.82	Lower, less favorable value with the balanced BCAA profile; 95% CI included 0 (imprecise estimate)	Not applicable - effect size estimated after converting SE to SD
Wang et al. (2023)	Turbot (Scophthalmus maximus)	BCAA balancing to estimated requirement levels	Control (balanced BCAAs: Leu 30.4, Ile 19.7, Val 17.4 g/kg)	Ile-H (Ile excess: 52.6 g/kg)	Protein efficiency ratio	2.17 ± 0.16	2.40 ± 0.17	3	3	Hedges' g	-1.12	-2.89 to 0.66	Lower, less favorable value with the balanced BCAA profile; 95% CI included 0 (imprecise estimate)	Not applicable - effect size estimated after converting SE to SD
Wang et al. (2023)	Turbot (Scophthalmus maximus)	BCAA balancing to estimated requirement levels	Control (balanced BCAAs: Leu 30.4, Ile 19.7, Val 17.4 g/kg)	Ile-H (Ile excess: 52.6 g/kg)	Protein productive value (%)	34.40 ± 2.08	38.60 ± 3.98	3	3	Hedges' g	-1.06	-2.82 to 0.70	Lower, less favorable value with the balanced BCAA profile; 95% CI included 0 (imprecise estimate)	Not applicable - effect size estimated after converting SE to SD
Wang et al. (2023)	Turbot (Scophthalmus maximus)	BCAA balancing to estimated requirement levels	Control (balanced BCAAs: Leu 30.4, Ile 19.7, Val 17.4 g/kg)	Ile-H (Ile excess: 52.6 g/kg)	Moisture (g/kg)	774.70 ± 5.54	775.20 ± 4.50	3	3	Hedges' g	-0.08	-1.68 to 1.52	Lower measured value with the balanced BCAA profile; 95% CI included 0 (imprecise estimate)	Not applicable - effect size estimated after converting SE to SD
Wang et al. (2023)	Turbot (Scophthalmus maximus)	BCAA balancing to estimated requirement levels	Control (balanced BCAAs: Leu 30.4, Ile 19.7, Val 17.4 g/kg)	Ile-H (Ile excess: 52.6 g/kg)	Crude protein (g/kg)	153.00 ± 2.60	154.10 ± 3.98	3	3	Hedges' g	-0.26	-1.87 to 1.35	Lower measured value with the balanced BCAA profile; 95% CI included 0 (imprecise estimate)	Not applicable - effect size estimated after converting SE to SD
Wang et al. (2023)	Turbot (Scophthalmus maximus)	BCAA balancing to estimated requirement levels	Control (balanced BCAAs: Leu 30.4, Ile 19.7, Val 17.4 g/kg)	Ile-H (Ile excess: 52.6 g/kg)	Crude lipid (g/kg)	26.10 ± 4.85	23.80 ± 0.69	3	3	Hedges' g	0.53	-1.11 to 2.17	Higher measured value with the balanced BCAA profile; 95% CI included 0 (imprecise estimate)	Not applicable - effect size estimated after converting SE to SD
Wang et al. (2023)	Turbot (Scophthalmus maximus)	BCAA balancing to estimated requirement levels	Control (balanced BCAAs: Leu 30.4, Ile 19.7, Val 17.4 g/kg)	Ile-H (Ile excess: 52.6 g/kg)	Ash (g/kg)	37.20 ± 2.08	36.70 ± 1.73	3	3	Hedges' g	0.21	-1.40 to 1.82	Higher measured value with the balanced BCAA profile; 95% CI included 0 (imprecise estimate)	Not applicable - effect size estimated after converting SE to SD
Wang et al. (2023)	Turbot (Scophthalmus maximus)	BCAA balancing to estimated requirement levels	Control (balanced BCAAs: Leu 30.4, Ile 19.7, Val 17.4 g/kg)	Ile-H (Ile excess: 52.6 g/kg)	Plasma free Leu, 2 h after feeding (µg/mL)	57.23 ± 7.60	87.48 ± 10.31	3	3	Hedges' g	-2.67	-5.12 to -0.22	Lower measured value with the balanced BCAA profile; 95% CI excluded 0	Not applicable - effect size estimated after converting SE to SD
Wang et al. (2023)	Turbot (Scophthalmus maximus)	BCAA balancing to estimated requirement levels	Control (balanced BCAAs: Leu 30.4, Ile 19.7, Val 17.4 g/kg)	Ile-H (Ile excess: 52.6 g/kg)	Plasma free Ile, 2 h after feeding (µg/mL)	36.40 ± 4.82	194.57 ± 23.14	3	3	Hedges' g	-7.57	-13.06 to -2.09	Lower measured value with the balanced BCAA profile; 95% CI excluded 0	Not applicable - effect size estimated after converting SE to SD
Wang et al. (2023)	Turbot (Scophthalmus maximus)	BCAA balancing to estimated requirement levels	Control (balanced BCAAs: Leu 30.4, Ile 19.7, Val 17.4 g/kg)	Ile-H (Ile excess: 52.6 g/kg)	Plasma free Val, 2 h after feeding (µg/mL)	40.85 ± 4.31	56.67 ± 13.49	3	3	Hedges' g	-1.26	-3.09 to 0.56	Lower measured value with the balanced BCAA profile; 95% CI included 0 (imprecise estimate)	Not applicable - effect size estimated after converting SE to SD
Wang et al. (2023)	Turbot (Scophthalmus maximus)	BCAA balancing to estimated requirement levels	Control (balanced BCAAs: Leu 30.4, Ile 19.7, Val 17.4 g/kg)	Ile-H (Ile excess: 52.6 g/kg)	Plasma free Leu, 6 h after feeding (µg/mL)	54.19 ± 16.26	96.66 ± 29.62	3	3	Hedges' g	-1.42	-3.30 to 0.46	Lower measured value with the balanced BCAA profile; 95% CI included 0 (imprecise estimate)	Not applicable - effect size estimated after converting SE to SD
Wang et al. (2023)	Turbot (Scophthalmus maximus)	BCAA balancing to estimated requirement levels	Control (balanced BCAAs: Leu 30.4, Ile 19.7, Val 17.4 g/kg)	Ile-H (Ile excess: 52.6 g/kg)	Plasma free Ile, 6 h after feeding (µg/mL)	37.73 ± 13.41	268.31 ± 28.98	3	3	Hedges' g	-8.17	-14.05 to -2.29	Lower measured value with the balanced BCAA profile; 95% CI excluded 0	Not applicable - effect size estimated after converting SE to SD
Wang et al. (2023)	Turbot (Scophthalmus maximus)	BCAA balancing to estimated requirement levels	Control (balanced BCAAs: Leu 30.4, Ile 19.7, Val 17.4 g/kg)	Ile-H (Ile excess: 52.6 g/kg)	Plasma free Val, 6 h after feeding (µg/mL)	40.54 ± 11.73	69.57 ± 28.18	3	3	Hedges' g	-1.08	-2.84 to 0.69	Lower measured value with the balanced BCAA profile; 95% CI included 0 (imprecise estimate)	Not applicable - effect size estimated after converting SE to SD
Wang et al. (2023)	Turbot (Scophthalmus maximus)	BCAA balancing to estimated requirement levels	Control (balanced BCAAs: Leu 30.4, Ile 19.7, Val 17.4 g/kg)	Ile-H (Ile excess: 52.6 g/kg)	Plasma free Leu, 24 h after feeding (µg/mL)	10.42 ± 0.73	10.79 ± 1.39	3	3	Hedges' g	-0.27	-1.88 to 1.34	Lower measured value with the balanced BCAA profile; 95% CI included 0 (imprecise estimate)	Not applicable - effect size estimated after converting SE to SD

Study	Species	Strategy	Intervention	Comparator	Outcome and unit	Intervention mean ± SD	Comparator mean ± SD	n intervention (tanks)	n comparator (tanks)	Effect-size metric	Effect size	95% CI	Interpretation	Reason not estimated
Wang et al. (2023)	Turbot (Scophthalmus maximus)	BCAA balancing to estimated requirement levels	Control (balanced BCAAs: Leu 30.4, Ile 19.7, Val 17.4 g/kg)	Ile-H (Ile excess: 52.6 g/kg)	Plasma free Ile, 24 h after feeding (µg/mL)	5.64 ± 0.64	7.02 ± 0.55	3	3	Hedges' g	-1.84	-3.89 to 0.20	Lower measured value with the balanced BCAA profile; 95% CI included 0 (imprecise estimate)	Not applicable - effect size estimated after converting SE to SD
Wang et al. (2023)	Turbot (Scophthalmus maximus)	BCAA balancing to estimated requirement levels	Control (balanced BCAAs: Leu 30.4, Ile 19.7, Val 17.4 g/kg)	Ile-H (Ile excess: 52.6 g/kg)	Plasma free Val, 24 h after feeding (µg/mL)	10.17 ± 1.20	11.01 ± 0.33	3	3	Hedges' g	-0.77	-2.45 to 0.92	Lower measured value with the balanced BCAA profile; 95% CI included 0 (imprecise estimate)	Not applicable - effect size estimated after converting SE to SD
Wang et al. (2023)	Turbot (Scophthalmus maximus)	BCAA balancing to estimated requirement levels	Control (balanced BCAAs: Leu 30.4, Ile 19.7, Val 17.4 g/kg)	Ile-H (Ile excess: 52.6 g/kg)	Liver free Leu, 2 h after feeding (µg/g)	740.38 ± 282.01	956.04 ± 222.22	3	3	Hedges' g	-0.68	-2.35 to 0.99	Lower measured value with the balanced BCAA profile; 95% CI included 0 (imprecise estimate)	Not applicable - effect size estimated after converting SE to SD
Wang et al. (2023)	Turbot (Scophthalmus maximus)	BCAA balancing to estimated requirement levels	Control (balanced BCAAs: Leu 30.4, Ile 19.7, Val 17.4 g/kg)	Ile-H (Ile excess: 52.6 g/kg)	Liver free Ile, 2 h after feeding (µg/g)	386.80 ± 135.31	993.16 ± 100.42	3	3	Hedges' g	-4.07	-7.31 to -0.83	Lower measured value with the balanced BCAA profile; 95% CI excluded 0	Not applicable - effect size estimated after converting SE to SD
Wang et al. (2023)	Turbot (Scophthalmus maximus)	BCAA balancing to estimated requirement levels	Control (balanced BCAAs: Leu 30.4, Ile 19.7, Val 17.4 g/kg)	Ile-H (Ile excess: 52.6 g/kg)	Liver free Val, 2 h after feeding (µg/g)	631.12 ± 247.86	835.97 ± 165.22	3	3	Hedges' g	-0.78	-2.47 to 0.91	Lower measured value with the balanced BCAA profile; 95% CI included 0 (imprecise estimate)	Not applicable - effect size estimated after converting SE to SD
Wang et al. (2023)	Turbot (Scophthalmus maximus)	BCAA balancing to estimated requirement levels	Control (balanced BCAAs: Leu 30.4, Ile 19.7, Val 17.4 g/kg)	Ile-H (Ile excess: 52.6 g/kg)	Liver free Leu, 6 h after feeding (µg/g)	646.81 ± 172.81	722.37 ± 260.99	3	3	Hedges' g	-0.27	-1.88 to 1.34	Lower measured value with the balanced BCAA profile; 95% CI included 0 (imprecise estimate)	Not applicable - effect size estimated after converting SE to SD
Wang et al. (2023)	Turbot (Scophthalmus maximus)	BCAA balancing to estimated requirement levels	Control (balanced BCAAs: Leu 30.4, Ile 19.7, Val 17.4 g/kg)	Ile-H (Ile excess: 52.6 g/kg)	Liver free Ile, 6 h after feeding (µg/g)	344.65 ± 108.51	797.87 ± 147.28	3	3	Hedges' g	-2.8	-5.32 to -0.29	Lower measured value with the balanced BCAA profile; 95% CI excluded 0	Not applicable - effect size estimated after converting SE to SD
Wang et al. (2023)	Turbot (Scophthalmus maximus)	BCAA balancing to estimated requirement levels	Control (balanced BCAAs: Leu 30.4, Ile 19.7, Val 17.4 g/kg)	Ile-H (Ile excess: 52.6 g/kg)	Liver free Val, 6 h after feeding (µg/g)	611.17 ± 136.33	606.43 ± 168.22	3	3	Hedges' g	0.02	-1.58 to 1.63	Higher measured value with the balanced BCAA profile; 95% CI included 0 (imprecise estimate)	Not applicable - effect size estimated after converting SE to SD
Wang et al. (2023)	Turbot (Scophthalmus maximus)	BCAA balancing to estimated requirement levels	Control (balanced BCAAs: Leu 30.4, Ile 19.7, Val 17.4 g/kg)	Ile-H (Ile excess: 52.6 g/kg)	Liver free Leu, 24 h after feeding (µg/g)	1367.52 ± 221.67	882.02 ± 126.75	3	3	Hedges' g	2.15	-0.04 to 4.34	Higher measured value with the balanced BCAA profile; 95% CI included 0 (imprecise estimate)	Not applicable - effect size estimated after converting SE to SD
Wang et al. (2023)	Turbot (Scophthalmus maximus)	BCAA balancing to estimated requirement levels	Control (balanced BCAAs: Leu 30.4, Ile 19.7, Val 17.4 g/kg)	Ile-H (Ile excess: 52.6 g/kg)	Liver free Ile, 24 h after feeding (µg/g)	657.02 ± 91.40	432.07 ± 119.63	3	3	Hedges' g	1.69	-0.29 to 3.67	Higher measured value with the balanced BCAA profile; 95% CI included 0 (imprecise estimate)	Not applicable - effect size estimated after converting SE to SD
Wang et al. (2023)	Turbot (Scophthalmus maximus)	BCAA balancing to estimated requirement levels	Control (balanced BCAAs: Leu 30.4, Ile 19.7, Val 17.4 g/kg)	Ile-H (Ile excess: 52.6 g/kg)	Liver free Val, 24 h after feeding (µg/g)	1163.91 ± 145.84	818.78 ± 125.89	3	3	Hedges' g	2.03	-0.10 to 4.16	Higher measured value with the balanced BCAA profile; 95% CI included 0 (imprecise estimate)	Not applicable - effect size estimated after converting SE to SD
Wang et al. (2023)	Turbot (Scophthalmus maximus)	BCAA balancing to estimated requirement levels	Control (balanced BCAAs: Leu 30.4, Ile 19.7, Val 17.4 g/kg)	Ile-H (Ile excess: 52.6 g/kg)	Muscle free Leu, 2 h after feeding (µg/g)	144.73 ± 8.12	186.53 ± 24.49	3	3	Hedges' g	-1.83	-3.88 to 0.21	Lower measured value with the balanced BCAA profile; 95% CI included 0 (imprecise estimate)	Not applicable - effect size estimated after converting SE to SD
Wang et al. (2023)	Turbot (Scophthalmus maximus)	BCAA balancing to estimated requirement levels	Control (balanced BCAAs: Leu 30.4, Ile 19.7, Val 17.4 g/kg)	Ile-H (Ile excess: 52.6 g/kg)	Muscle free Ile, 2 h after feeding (µg/g)	103.55 ± 10.25	455.06 ± 23.54	3	3	Hedges' g	-15.49	-26.34 to -4.64	Lower measured value with the balanced BCAA profile; 95% CI excluded 0	Not applicable - effect size estimated after converting SE to SD
Wang et al. (2023)	Turbot (Scophthalmus maximus)	BCAA balancing to estimated requirement levels	Control (balanced BCAAs: Leu 30.4, Ile 19.7, Val 17.4 g/kg)	Ile-H (Ile excess: 52.6 g/kg)	Muscle free Val, 2 h after feeding (µg/g)	1940.63 ± 202.27	2137.86 ± 250.40	3	3	Hedges' g	-0.69	-2.36 to 0.98	Lower measured value with the balanced BCAA profile; 95% CI included 0 (imprecise estimate)	Not applicable - effect size estimated after converting SE to SD
Wang et al. (2023)	Turbot (Scophthalmus maximus)	BCAA balancing to estimated requirement levels	Control (balanced BCAAs: Leu 30.4, Ile 19.7, Val 17.4 g/kg)	Ile-H (Ile excess: 52.6 g/kg)	Muscle free Leu, 6 h after feeding (µg/g)	169.94 ± 42.61	251.48 ± 32.94	3	3	Hedges' g	-1.71	-3.71 to 0.28	Lower measured value with the balanced BCAA profile; 95% CI included 0 (imprecise estimate)	Not applicable - effect size estimated after converting SE to SD
Wang et al. (2023)	Turbot (Scophthalmus maximus)	BCAA balancing to estimated requirement levels	Control (balanced BCAAs: Leu 30.4, Ile 19.7, Val 17.4 g/kg)	Ile-H (Ile excess: 52.6 g/kg)	Muscle free Ile, 6 h after feeding (µg/g)	108.29 ± 25.50	713.13 ± 43.49	3	3	Hedges' g	-13.57	-23.11 to -4.03	Lower measured value with the balanced BCAA profile; 95% CI excluded 0	Not applicable - effect size estimated after converting SE to SD
Wang et al. (2023)	Turbot (Scophthalmus maximus)	BCAA balancing to estimated requirement levels	Control (balanced BCAAs: Leu 30.4, Ile 19.7, Val 17.4 g/kg)	Ile-H (Ile excess: 52.6 g/kg)	Muscle free Val, 6 h after feeding (µg/g)	2219.39 ± 534.30	2754.15 ± 320.65	3	3	Hedges' g	-0.97	-2.71 to 0.77	Lower measured value with the balanced BCAA profile; 95% CI included 0 (imprecise estimate)	Not applicable - effect size estimated after converting SE to SD
Wang et al. (2023)	Turbot (Scophthalmus maximus)	BCAA balancing to estimated requirement levels	Control (balanced BCAAs: Leu 30.4, Ile 19.7, Val 17.4 g/kg)	Ile-H (Ile excess: 52.6 g/kg)	Muscle free Leu, 24 h after feeding (µg/g)	69.30 ± 21.20	70.53 ± 7.74	3	3	Hedges' g	-0.06	-1.66 to 1.54	Lower measured value with the balanced BCAA profile; 95% CI included 0 (imprecise estimate)	Not applicable - effect size estimated after converting SE to SD
Wang et al. (2023)	Turbot (Scophthalmus maximus)	BCAA balancing to estimated requirement levels	Control (balanced BCAAs: Leu 30.4, Ile 19.7, Val 17.4 g/kg)	Ile-H (Ile excess: 52.6 g/kg)	Muscle free Ile, 24 h after feeding (µg/g)	48.99 ± 18.74	78.35 ± 16.78	3	3	Hedges' g	-1.32	-3.16 to 0.52	Lower measured value with the balanced BCAA profile; 95% CI included 0 (imprecise estimate)	Not applicable - effect size estimated after converting SE to SD
Wang et al. (2023)	Turbot (Scophthalmus maximus)	BCAA balancing to estimated requirement levels	Control (balanced BCAAs: Leu 30.4, Ile 19.7, Val 17.4 g/kg)	Ile-H (Ile excess: 52.6 g/kg)	Muscle free Val, 24 h after feeding (µg/g)	1772.95 ± 160.06	2316.37 ± 448.51	3	3	Hedges' g	-1.29	-3.12 to 0.54	Lower measured value with the balanced BCAA profile; 95% CI included 0 (imprecise estimate)	Not applicable - effect size estimated after converting SE to SD
Wang et al. (2023)	Turbot (Scophthalmus maximus)	BCAA balancing to estimated requirement levels	Control (balanced BCAAs: Leu 30.4, Ile 19.7, Val 17.4 g/kg)	Val-H (Val excess: 46.6 g/kg)	Survival (%)	98.70 ± 2.25	98.70 ± 2.25	3	3	Hedges' g	0.0	-1.60 to 1.60	No observed mean difference; 95% CI included 0 (imprecise estimate)	Not applicable - effect size estimated after converting SE to SD
Wang et al. (2023)	Turbot (Scophthalmus maximus)	BCAA balancing to estimated requirement levels	Control (balanced BCAAs: Leu 30.4, Ile 19.7, Val 17.4 g/kg)	Val-H (Val excess: 46.6 g/kg)	Weight gain (%)	117.80 ± 13.16	110.40 ± 7.45	3	3	Hedges' g	0.55	-1.09 to 2.20	Higher, more favorable value with the balanced BCAA profile; 95% CI included 0 (imprecise estimate)	Not applicable - effect size estimated after converting SE to SD
Wang et al. (2023)	Turbot (Scophthalmus maximus)	BCAA balancing to estimated requirement levels	Control (balanced BCAAs: Leu 30.4, Ile 19.7, Val 17.4 g/kg)	Val-H (Val excess: 46.6 g/kg)	Specific growth rate (%/day)	1.29 ± 0.10	1.24 ± 0.07	3	3	Hedges' g	0.45	-1.18 to 2.08	Higher, more favorable value with the balanced BCAA profile; 95% CI included 0 (imprecise estimate)	Not applicable - effect size estimated after converting SE to SD
Wang et al. (2023)	Turbot (Scophthalmus maximus)	BCAA balancing to estimated requirement levels	Control (balanced BCAAs: Leu 30.4, Ile 19.7, Val 17.4 g/kg)	Val-H (Val excess: 46.6 g/kg)	Feed intake (% body weight/day)	1.12 ± 0.03	1.04 ± 0.02	3	3	Hedges' g	2.34	0.06 to 4.61	Higher measured value with the balanced BCAA profile; 95% CI excluded 0	Not applicable - effect size estimated after converting SE to SD
Wang et al. (2023)	Turbot (Scophthalmus maximus)	BCAA balancing to estimated requirement levels	Control (balanced BCAAs: Leu 30.4, Ile 19.7, Val 17.4 g/kg)	Val-H (Val excess: 46.6 g/kg)	Feed efficiency	1.11 ± 0.05	1.15 ± 0.07	3	3	Hedges' g	-0.52	-2.16 to 1.12	Lower, less favorable value with the balanced BCAA profile; 95% CI included 0 (imprecise estimate)	Not applicable - effect size estimated after converting SE to SD
Wang et al. (2023)	Turbot (Scophthalmus maximus)	BCAA balancing to estimated requirement levels	Control (balanced BCAAs: Leu 30.4, Ile 19.7, Val 17.4 g/kg)	Val-H (Val excess: 46.6 g/kg)	Protein efficiency ratio	2.17 ± 0.16	2.27 ± 0.12	3	3	Hedges' g	-0.57	-2.22 to 1.08	Lower, less favorable value with the balanced BCAA profile; 95% CI included 0 (imprecise estimate)	Not applicable - effect size estimated after converting SE to SD
Wang et al. (2023)	Turbot (Scophthalmus maximus)	BCAA balancing to estimated requirement levels	Control (balanced BCAAs: Leu 30.4, Ile 19.7, Val 17.4 g/kg)	Val-H (Val excess: 46.6 g/kg)	Protein productive value (%)	34.40 ± 2.08	34.60 ± 1.91	3	3	Hedges' g	-0.08	-1.68 to 1.52	Lower, less favorable value with the balanced BCAA profile; 95% CI included 0 (imprecise estimate)	Not applicable - effect size estimated after converting SE to SD
Wang et al. (2023)	Turbot (Scophthalmus maximus)	BCAA balancing to estimated requirement levels	Control (balanced BCAAs: Leu 30.4, Ile 19.7, Val 17.4 g/kg)	Val-H (Val excess: 46.6 g/kg)	Moisture (g/kg)	774.70 ± 5.54	780.80 ± 0.17	3	3	Hedges' g	-1.24	-3.06 to 0.57	Lower measured value with the balanced BCAA profile; 95% CI included 0 (imprecise estimate)	Not applicable - effect size estimated after converting SE to SD
Wang et al. (2023)	Turbot (Scophthalmus maximus)	BCAA balancing to estimated requirement levels	Control (balanced BCAAs: Leu 30.4, Ile 19.7, Val 17.4 g/kg)	Val-H (Val excess: 46.6 g/kg)	Crude protein (g/kg)	153.00 ± 2.60	149.70 ± 1.04	3	3	Hedges' g	1.33	-0.51 to 3.18	Higher measured value with the balanced BCAA profile; 95% CI included 0 (imprecise estimate)	Not applicable - effect size estimated after converting SE to SD
Wang et al. (2023)	Turbot (Scophthalmus maximus)	BCAA balancing to estimated requirement levels	Control (balanced BCAAs: Leu 30.4, Ile 19.7, Val 17.4 g/kg)	Val-H (Val excess: 46.6 g/kg)	Crude lipid (g/kg)	26.10 ± 4.85	23.00 ± 1.91	3	3	Hedges' g	0.67	-0.99 to 2.34	Higher measured value with the balanced BCAA profile; 95% CI included 0 (imprecise estimate)	Not applicable - effect size estimated after converting SE to SD
Wang et al. (2023)	Turbot (Scophthalmus maximus)	BCAA balancing to estimated requirement levels	Control (balanced BCAAs: Leu 30.4, Ile 19.7, Val 17.4 g/kg)	Val-H (Val excess: 46.6 g/kg)	Ash (g/kg)	37.20 ± 2.08	36.70 ± 0.35	3	3	Hedges' g	0.27	-1.34 to 1.88	Higher measured value with the balanced BCAA profile; 95% CI included 0 (imprecise estimate)	Not applicable - effect size estimated after converting SE to SD
Wang et al. (2023)	Turbot (Scophthalmus maximus)	BCAA balancing to estimated requirement levels	Control (balanced BCAAs: Leu 30.4, Ile 19.7, Val 17.4 g/kg)	Val-H (Val excess: 46.6 g/kg)	Plasma free Leu, 2 h after feeding (µg/mL)	57.23 ± 7.60	66.38 ± 3.38	3	3	Hedges' g	-1.24	-3.06 to 0.57	Lower measured value with the balanced BCAA profile; 95% CI included 0 (imprecise estimate)	Not applicable - effect size estimated after converting SE to SD

Study	Species	Strategy	Intervention	Comparator	Outcome and unit	Intervention mean ± SD	Comparator mean ± SD	n intervention (tanks)	n comparator (tanks)	Effect-size metric	Effect size	95% CI	Interpretation	Reason not estimated
Wang et al. (2023)	Turbot (Scophthalmus maximus)	BCAA balancing to estimated requirement levels	Control (balanced BCAAs: Leu 30.4, Ile 19.7, Val 17.4 g/kg)	Val-H (Val excess: 46.6 g/kg)	Plasma free Ile, 2 h after feeding (µg/mL)	36.40 ± 4.82	46.62 ± 8.49	3	3	Hedges' g	-1.18	-2.98 to 0.61	Lower measured value with the balanced BCAA profile; 95% CI included 0 (imprecise estimate)	Not applicable - effect size estimated after converting SE to SD
Wang et al. (2023)	Turbot (Scophthalmus maximus)	BCAA balancing to estimated requirement levels	Control (balanced BCAAs: Leu 30.4, Ile 19.7, Val 17.4 g/kg)	Val-H (Val excess: 46.6 g/kg)	Plasma free Val, 2 h after feeding (µg/mL)	40.85 ± 4.31	218.93 ± 17.49	3	3	Hedges' g	-11.18	-19.09 to -3.27	Lower measured value with the balanced BCAA profile; 95% CI excluded 0	Not applicable - effect size estimated after converting SE to SD
Wang et al. (2023)	Turbot (Scophthalmus maximus)	BCAA balancing to estimated requirement levels	Control (balanced BCAAs: Leu 30.4, Ile 19.7, Val 17.4 g/kg)	Val-H (Val excess: 46.6 g/kg)	Plasma free Leu, 6 h after feeding (µg/mL)	54.19 ± 16.26	87.41 ± 6.30	3	3	Hedges' g	-2.15	-4.34 to 0.03	Lower measured value with the balanced BCAA profile; 95% CI included 0 (imprecise estimate)	Not applicable - effect size estimated after converting SE to SD
Wang et al. (2023)	Turbot (Scophthalmus maximus)	BCAA balancing to estimated requirement levels	Control (balanced BCAAs: Leu 30.4, Ile 19.7, Val 17.4 g/kg)	Val-H (Val excess: 46.6 g/kg)	Plasma free Ile, 6 h after feeding (µg/mL)	37.73 ± 13.41	56.32 ± 3.78	3	3	Hedges' g	-1.51	-3.42 to 0.40	Lower measured value with the balanced BCAA profile; 95% CI included 0 (imprecise estimate)	Not applicable - effect size estimated after converting SE to SD
Wang et al. (2023)	Turbot (Scophthalmus maximus)	BCAA balancing to estimated requirement levels	Control (balanced BCAAs: Leu 30.4, Ile 19.7, Val 17.4 g/kg)	Val-H (Val excess: 46.6 g/kg)	Plasma free Val, 6 h after feeding (µg/mL)	40.54 ± 11.73	280.91 ± 40.22	3	3	Hedges' g	-6.49	-11.27 to -1.72	Lower measured value with the balanced BCAA profile; 95% CI excluded 0	Not applicable - effect size estimated after converting SE to SD
Wang et al. (2023)	Turbot (Scophthalmus maximus)	BCAA balancing to estimated requirement levels	Control (balanced BCAAs: Leu 30.4, Ile 19.7, Val 17.4 g/kg)	Val-H (Val excess: 46.6 g/kg)	Plasma free Leu, 24 h after feeding (µg/mL)	10.42 ± 0.73	13.89 ± 4.92	3	3	Hedges' g	-0.79	-2.48 to 0.90	Lower measured value with the balanced BCAA profile; 95% CI included 0 (imprecise estimate)	Not applicable - effect size estimated after converting SE to SD
Wang et al. (2023)	Turbot (Scophthalmus maximus)	BCAA balancing to estimated requirement levels	Control (balanced BCAAs: Leu 30.4, Ile 19.7, Val 17.4 g/kg)	Val-H (Val excess: 46.6 g/kg)	Plasma free Ile, 24 h after feeding (µg/mL)	5.64 ± 0.64	7.50 ± 2.27	3	3	Hedges' g	-0.89	-2.61 to 0.82	Lower measured value with the balanced BCAA profile; 95% CI included 0 (imprecise estimate)	Not applicable - effect size estimated after converting SE to SD
Wang et al. (2023)	Turbot (Scophthalmus maximus)	BCAA balancing to estimated requirement levels	Control (balanced BCAAs: Leu 30.4, Ile 19.7, Val 17.4 g/kg)	Val-H (Val excess: 46.6 g/kg)	Plasma free Val, 24 h after feeding (µg/mL)	10.17 ± 1.20	18.47 ± 5.59	3	3	Hedges' g	-1.64	-3.60 to 0.32	Lower measured value with the balanced BCAA profile; 95% CI included 0 (imprecise estimate)	Not applicable - effect size estimated after converting SE to SD
Wang et al. (2023)	Turbot (Scophthalmus maximus)	BCAA balancing to estimated requirement levels	Control (balanced BCAAs: Leu 30.4, Ile 19.7, Val 17.4 g/kg)	Val-H (Val excess: 46.6 g/kg)	Liver free Leu, 2 h after feeding (µg/g)	740.38 ± 282.01	935.50 ± 142.89	3	3	Hedges' g	-0.7	-2.37 to 0.97	Lower measured value with the balanced BCAA profile; 95% CI included 0 (imprecise estimate)	Not applicable - effect size estimated after converting SE to SD
Wang et al. (2023)	Turbot (Scophthalmus maximus)	BCAA balancing to estimated requirement levels	Control (balanced BCAAs: Leu 30.4, Ile 19.7, Val 17.4 g/kg)	Val-H (Val excess: 46.6 g/kg)	Liver free Ile, 2 h after feeding (µg/g)	386.80 ± 135.31	508.65 ± 80.80	3	3	Hedges' g	-0.87	-2.59 to 0.84	Lower measured value with the balanced BCAA profile; 95% CI included 0 (imprecise estimate)	Not applicable - effect size estimated after converting SE to SD
Wang et al. (2023)	Turbot (Scophthalmus maximus)	BCAA balancing to estimated requirement levels	Control (balanced BCAAs: Leu 30.4, Ile 19.7, Val 17.4 g/kg)	Val-H (Val excess: 46.6 g/kg)	Liver free Val, 2 h after feeding (µg/g)	631.12 ± 247.86	1279.68 ± 160.02	3	3	Hedges' g	-2.49	-4.84 to -0.14	Lower measured value with the balanced BCAA profile; 95% CI excluded 0	Not applicable - effect size estimated after converting SE to SD
Wang et al. (2023)	Turbot (Scophthalmus maximus)	BCAA balancing to estimated requirement levels	Control (balanced BCAAs: Leu 30.4, Ile 19.7, Val 17.4 g/kg)	Val-H (Val excess: 46.6 g/kg)	Liver free Leu, 6 h after feeding (µg/g)	646.81 ± 172.81	708.10 ± 107.40	3	3	Hedges' g	-0.34	-1.96 to 1.28	Lower measured value with the balanced BCAA profile; 95% CI included 0 (imprecise estimate)	Not applicable - effect size estimated after converting SE to SD
Wang et al. (2023)	Turbot (Scophthalmus maximus)	BCAA balancing to estimated requirement levels	Control (balanced BCAAs: Leu 30.4, Ile 19.7, Val 17.4 g/kg)	Val-H (Val excess: 46.6 g/kg)	Liver free Ile, 6 h after feeding (µg/g)	344.65 ± 108.51	362.69 ± 60.15	3	3	Hedges' g	-0.16	-1.77 to 1.44	Lower measured value with the balanced BCAA profile; 95% CI included 0 (imprecise estimate)	Not applicable - effect size estimated after converting SE to SD
Wang et al. (2023)	Turbot (Scophthalmus maximus)	BCAA balancing to estimated requirement levels	Control (balanced BCAAs: Leu 30.4, Ile 19.7, Val 17.4 g/kg)	Val-H (Val excess: 46.6 g/kg)	Liver free Val, 6 h after feeding (µg/g)	611.17 ± 136.33	985.93 ± 109.81	3	3	Hedges' g	-2.42	-4.74 to -0.10	Lower measured value with the balanced BCAA profile; 95% CI excluded 0	Not applicable - effect size estimated after converting SE to SD
Wang et al. (2023)	Turbot (Scophthalmus maximus)	BCAA balancing to estimated requirement levels	Control (balanced BCAAs: Leu 30.4, Ile 19.7, Val 17.4 g/kg)	Val-H (Val excess: 46.6 g/kg)	Liver free Leu, 24 h after feeding (µg/g)	1367.52 ± 221.67	916.47 ± 165.88	3	3	Hedges' g	1.84	-0.20 to 3.89	Higher measured value with the balanced BCAA profile; 95% CI included 0 (imprecise estimate)	Not applicable - effect size estimated after converting SE to SD
Wang et al. (2023)	Turbot (Scophthalmus maximus)	BCAA balancing to estimated requirement levels	Control (balanced BCAAs: Leu 30.4, Ile 19.7, Val 17.4 g/kg)	Val-H (Val excess: 46.6 g/kg)	Liver free Ile, 24 h after feeding (µg/g)	657.02 ± 91.40	441.64 ± 61.23	3	3	Hedges' g	2.21	-0.00 to 4.43	Higher measured value with the balanced BCAA profile; 95% CI included 0 (imprecise estimate)	Not applicable - effect size estimated after converting SE to SD
Wang et al. (2023)	Turbot (Scophthalmus maximus)	BCAA balancing to estimated requirement levels	Control (balanced BCAAs: Leu 30.4, Ile 19.7, Val 17.4 g/kg)	Val-H (Val excess: 46.6 g/kg)	Liver free Val, 24 h after feeding (µg/g)	1163.91 ± 145.84	875.54 ± 127.18	3	3	Hedges' g	1.69	-0.30 to 3.67	Higher measured value with the balanced BCAA profile; 95% CI included 0 (imprecise estimate)	Not applicable - effect size estimated after converting SE to SD
Wang et al. (2023)	Turbot (Scophthalmus maximus)	BCAA balancing to estimated requirement levels	Control (balanced BCAAs: Leu 30.4, Ile 19.7, Val 17.4 g/kg)	Val-H (Val excess: 46.6 g/kg)	Muscle free Leu, 2 h after feeding (µg/g)	144.73 ± 8.12	145.08 ± 16.58	3	3	Hedges' g	-0.02	-1.62 to 1.58	Lower measured value with the balanced BCAA profile; 95% CI included 0 (imprecise estimate)	Not applicable - effect size estimated after converting SE to SD
Wang et al. (2023)	Turbot (Scophthalmus maximus)	BCAA balancing to estimated requirement levels	Control (balanced BCAAs: Leu 30.4, Ile 19.7, Val 17.4 g/kg)	Val-H (Val excess: 46.6 g/kg)	Muscle free Ile, 2 h after feeding (µg/g)	103.55 ± 10.25	116.91 ± 12.04	3	3	Hedges' g	-0.96	-2.69 to 0.78	Lower measured value with the balanced BCAA profile; 95% CI included 0 (imprecise estimate)	Not applicable - effect size estimated after converting SE to SD
Wang et al. (2023)	Turbot (Scophthalmus maximus)	BCAA balancing to estimated requirement levels	Control (balanced BCAAs: Leu 30.4, Ile 19.7, Val 17.4 g/kg)	Val-H (Val excess: 46.6 g/kg)	Muscle free Val, 2 h after feeding (µg/g)	1940.63 ± 202.27	2605.17 ± 130.22	3	3	Hedges' g	-3.13	-5.82 to -0.43	Lower measured value with the balanced BCAA profile; 95% CI excluded 0	Not applicable - effect size estimated after converting SE to SD
Wang et al. (2023)	Turbot (Scophthalmus maximus)	BCAA balancing to estimated requirement levels	Control (balanced BCAAs: Leu 30.4, Ile 19.7, Val 17.4 g/kg)	Val-H (Val excess: 46.6 g/kg)	Muscle free Leu, 6 h after feeding (µg/g)	169.94 ± 42.61	195.03 ± 29.74	3	3	Hedges' g	-0.55	-2.19 to 1.10	Lower measured value with the balanced BCAA profile; 95% CI included 0 (imprecise estimate)	Not applicable - effect size estimated after converting SE to SD
Wang et al. (2023)	Turbot (Scophthalmus maximus)	BCAA balancing to estimated requirement levels	Control (balanced BCAAs: Leu 30.4, Ile 19.7, Val 17.4 g/kg)	Val-H (Val excess: 46.6 g/kg)	Muscle free Ile, 6 h after feeding (µg/g)	108.29 ± 25.50	148.39 ± 34.47	3	3	Hedges' g	-1.06	-2.82 to 0.70	Lower measured value with the balanced BCAA profile; 95% CI included 0 (imprecise estimate)	Not applicable - effect size estimated after converting SE to SD
Wang et al. (2023)	Turbot (Scophthalmus maximus)	BCAA balancing to estimated requirement levels	Control (balanced BCAAs: Leu 30.4, Ile 19.7, Val 17.4 g/kg)	Val-H (Val excess: 46.6 g/kg)	Muscle free Val, 6 h after feeding (µg/g)	2219.39 ± 534.30	3098.80 ± 212.73	3	3	Hedges' g	-1.73	-3.73 to 0.27	Lower measured value with the balanced BCAA profile; 95% CI included 0 (imprecise estimate)	Not applicable - effect size estimated after converting SE to SD
Wang et al. (2023)	Turbot (Scophthalmus maximus)	BCAA balancing to estimated requirement levels	Control (balanced BCAAs: Leu 30.4, Ile 19.7, Val 17.4 g/kg)	Val-H (Val excess: 46.6 g/kg)	Muscle free Leu, 24 h after feeding (µg/g)	69.30 ± 21.20	91.67 ± 11.99	3	3	Hedges' g	-1.04	-2.79 to 0.72	Lower measured value with the balanced BCAA profile; 95% CI included 0 (imprecise estimate)	Not applicable - effect size estimated after converting SE to SD
Wang et al. (2023)	Turbot (Scophthalmus maximus)	BCAA balancing to estimated requirement levels	Control (balanced BCAAs: Leu 30.4, Ile 19.7, Val 17.4 g/kg)	Val-H (Val excess: 46.6 g/kg)	Muscle free Ile, 24 h after feeding (µg/g)	48.99 ± 18.74	67.39 ± 11.17	3	3	Hedges' g	-0.95	-2.69 to 0.78	Lower measured value with the balanced BCAA profile; 95% CI included 0 (imprecise estimate)	Not applicable - effect size estimated after converting SE to SD
Wang et al. (2023)	Turbot (Scophthalmus maximus)	BCAA balancing to estimated requirement levels	Control (balanced BCAAs: Leu 30.4, Ile 19.7, Val 17.4 g/kg)	Val-H (Val excess: 46.6 g/kg)	Muscle free Val, 24 h after feeding (µg/g)	1772.95 ± 160.06	2451.37 ± 549.94	3	3	Hedges' g	-1.34	-3.19 to 0.51	Lower measured value with the balanced BCAA profile; 95% CI included 0 (imprecise estimate)	Not applicable - effect size estimated after converting SE to SD
Wang et al. (2023)	Turbot (Scophthalmus maximus)	BCAA balancing to estimated requirement levels	Control (balanced BCAAs: Leu 30.4, Ile 19.7, Val 17.4 g/kg)	Leu-L (Leu deficient: 20.9 g/kg)	b0at1 mRNA expression (Figure 1A)	Not reported numerically	Not reported numerically	3	3	Not estimable	Not estimable	Not estimable	The outcome was presented graphically with Tukey-letter annotations; an exact pairwise effect size was not calculated	Exact group means and SEs were presented only in Figures 1-3 and were not reliably extractable
Wang et al. (2023)	Turbot (Scophthalmus maximus)	BCAA balancing to estimated requirement levels	Control (balanced BCAAs: Leu 30.4, Ile 19.7, Val 17.4 g/kg)	Leu-L (Leu deficient: 20.9 g/kg)	b0at2 mRNA expression (Figure 1A)	Not reported numerically	Not reported numerically	3	3	Not estimable	Not estimable	Not estimable	The outcome was presented graphically with Tukey-letter annotations; an exact pairwise effect size was not calculated	Exact group means and SEs were presented only in Figures 1-3 and were not reliably extractable
Wang et al. (2023)	Turbot (Scophthalmus maximus)	BCAA balancing to estimated requirement levels	Control (balanced BCAAs: Leu 30.4, Ile 19.7, Val 17.4 g/kg)	Leu-L (Leu deficient: 20.9 g/kg)	lat4 mRNA expression (Figure 1A)	Not reported numerically	Not reported numerically	3	3	Not estimable	Not estimable	Not estimable	The outcome was presented graphically with Tukey-letter annotations; an exact pairwise effect size was not calculated	Exact group means and SEs were presented only in Figures 1-3 and were not reliably extractable
Wang et al. (2023)	Turbot (Scophthalmus maximus)	BCAA balancing to estimated requirement levels	Control (balanced BCAAs: Leu 30.4, Ile 19.7, Val 17.4 g/kg)	Leu-L (Leu deficient: 20.9 g/kg)	pept1a mRNA expression (Figure 1B)	Not reported numerically	Not reported numerically	3	3	Not estimable	Not estimable	Not estimable	The outcome was presented graphically with Tukey-letter annotations; an exact pairwise effect size was not calculated	Exact group means and SEs were presented only in Figures 1-3 and were not reliably extractable
Wang et al. (2023)	Turbot (Scophthalmus maximus)	BCAA balancing to estimated requirement levels	Control (balanced BCAAs: Leu 30.4, Ile 19.7, Val 17.4 g/kg)	Leu-L (Leu deficient: 20.9 g/kg)	pept1b mRNA expression (Figure 1B)	Not reported numerically	Not reported numerically	3	3	Not estimable	Not estimable	Not estimable	The outcome was presented graphically with Tukey-letter annotations; an exact pairwise effect size was not calculated	Exact group means and SEs were presented only in Figures 1-3 and were not reliably extractable
Wang et al. (2023)	Turbot (Scophthalmus maximus)	BCAA balancing to estimated requirement levels	Control (balanced BCAAs: Leu 30.4, Ile 19.7, Val 17.4 g/kg)	Leu-L (Leu deficient: 20.9 g/kg)	Muscle bcat2 mRNA expression (Figure 2)	Not reported numerically	Not reported numerically	3	3	Not estimable	Not estimable	Not estimable	The outcome was presented graphically with Tukey-letter annotations; an exact pairwise effect size was not calculated	Exact group means and SEs were presented only in Figures 1-3 and were not reliably extractable

Study	Species	Strategy	Intervention	Comparator	Outcome and unit	Intervention mean ± SD	Comparator mean ± SD	n intervention (tanks)	n comparator (tanks)	Effect-size metric	Effect size	95% CI	Interpretation	Reason not estimated
Wang et al. (2023)	Turbot (Scophthalmus maximus)	BCAA balancing to estimated requirement levels	Control (balanced BCAAs: Leu 30.4, Ile 19.7, Val 17.4 g/kg)	Leu-L (Leu deficient: 20.9 g/kg)	Liver bckdha mRNA expression (Figure 2)	Not reported numerically	Not reported numerically	3	3	Not estimable	Not estimable	Not estimable	The outcome was presented graphically with Tukey-letter annotations; an exact pairwise effect size was not calculated	Exact group means and SEs were presented only in Figures 1-3 and were not reliably extractable
Wang et al. (2023)	Turbot (Scophthalmus maximus)	BCAA balancing to estimated requirement levels	Control (balanced BCAAs: Leu 30.4, Ile 19.7, Val 17.4 g/kg)	Leu-L (Leu deficient: 20.9 g/kg)	Liver bckdhb mRNA expression (Figure 2)	Not reported numerically	Not reported numerically	3	3	Not estimable	Not estimable	Not estimable	The outcome was presented graphically with Tukey-letter annotations; an exact pairwise effect size was not calculated	Exact group means and SEs were presented only in Figures 1-3 and were not reliably extractable
Wang et al. (2023)	Turbot (Scophthalmus maximus)	BCAA balancing to estimated requirement levels	Control (balanced BCAAs: Leu 30.4, Ile 19.7, Val 17.4 g/kg)	Leu-L (Leu deficient: 20.9 g/kg)	Liver bckdk mRNA expression (Figure 2)	Not reported numerically	Not reported numerically	3	3	Not estimable	Not estimable	Not estimable	The outcome was presented graphically with Tukey-letter annotations; an exact pairwise effect size was not calculated	Exact group means and SEs were presented only in Figures 1-3 and were not reliably extractable
Wang et al. (2023)	Turbot (Scophthalmus maximus)	BCAA balancing to estimated requirement levels	Control (balanced BCAAs: Leu 30.4, Ile 19.7, Val 17.4 g/kg)	Leu-L (Leu deficient: 20.9 g/kg)	Liver mtor mRNA expression (Figure 3A)	Not reported numerically	Not reported numerically	3	3	Not estimable	Not estimable	Not estimable	The outcome was presented graphically with Tukey-letter annotations; an exact pairwise effect size was not calculated	Exact group means and SEs were presented only in Figures 1-3 and were not reliably extractable
Wang et al. (2023)	Turbot (Scophthalmus maximus)	BCAA balancing to estimated requirement levels	Control (balanced BCAAs: Leu 30.4, Ile 19.7, Val 17.4 g/kg)	Leu-L (Leu deficient: 20.9 g/kg)	Liver s6ka mRNA expression (Figure 3A)	Not reported numerically	Not reported numerically	3	3	Not estimable	Not estimable	Not estimable	The outcome was presented graphically with Tukey-letter annotations; an exact pairwise effect size was not calculated	Exact group means and SEs were presented only in Figures 1-3 and were not reliably extractable
Wang et al. (2023)	Turbot (Scophthalmus maximus)	BCAA balancing to estimated requirement levels	Control (balanced BCAAs: Leu 30.4, Ile 19.7, Val 17.4 g/kg)	Leu-L (Leu deficient: 20.9 g/kg)	Liver s6kb1a mRNA expression (Figure 3A)	Not reported numerically	Not reported numerically	3	3	Not estimable	Not estimable	Not estimable	The outcome was presented graphically with Tukey-letter annotations; an exact pairwise effect size was not calculated	Exact group means and SEs were presented only in Figures 1-3 and were not reliably extractable
Wang et al. (2023)	Turbot (Scophthalmus maximus)	BCAA balancing to estimated requirement levels	Control (balanced BCAAs: Leu 30.4, Ile 19.7, Val 17.4 g/kg)	Leu-L (Leu deficient: 20.9 g/kg)	Liver s6kb1b mRNA expression (Figure 3A)	Not reported numerically	Not reported numerically	3	3	Not estimable	Not estimable	Not estimable	The outcome was presented graphically with Tukey-letter annotations; an exact pairwise effect size was not calculated	Exact group means and SEs were presented only in Figures 1-3 and were not reliably extractable
Wang et al. (2023)	Turbot (Scophthalmus maximus)	BCAA balancing to estimated requirement levels	Control (balanced BCAAs: Leu 30.4, Ile 19.7, Val 17.4 g/kg)	Leu-L (Leu deficient: 20.9 g/kg)	Liver 4e-bp1 mRNA expression (Figure 3A)	Not reported numerically	Not reported numerically	3	3	Not estimable	Not estimable	Not estimable	The outcome was presented graphically with Tukey-letter annotations; an exact pairwise effect size was not calculated	Exact group means and SEs were presented only in Figures 1-3 and were not reliably extractable
Wang et al. (2023)	Turbot (Scophthalmus maximus)	BCAA balancing to estimated requirement levels	Control (balanced BCAAs: Leu 30.4, Ile 19.7, Val 17.4 g/kg)	Leu-L (Leu deficient: 20.9 g/kg)	Muscle mtor mRNA expression (Figure 3B)	Not reported numerically	Not reported numerically	3	3	Not estimable	Not estimable	Not estimable	The outcome was presented graphically with Tukey-letter annotations; an exact pairwise effect size was not calculated	Exact group means and SEs were presented only in Figures 1-3 and were not reliably extractable
Wang et al. (2023)	Turbot (Scophthalmus maximus)	BCAA balancing to estimated requirement levels	Control (balanced BCAAs: Leu 30.4, Ile 19.7, Val 17.4 g/kg)	Leu-L (Leu deficient: 20.9 g/kg)	Muscle s6ka mRNA expression (Figure 3B)	Not reported numerically	Not reported numerically	3	3	Not estimable	Not estimable	Not estimable	The outcome was presented graphically with Tukey-letter annotations; an exact pairwise effect size was not calculated	Exact group means and SEs were presented only in Figures 1-3 and were not reliably extractable
Wang et al. (2023)	Turbot (Scophthalmus maximus)	BCAA balancing to estimated requirement levels	Control (balanced BCAAs: Leu 30.4, Ile 19.7, Val 17.4 g/kg)	Leu-L (Leu deficient: 20.9 g/kg)	Muscle s6kb1a mRNA expression (Figure 3B)	Not reported numerically	Not reported numerically	3	3	Not estimable	Not estimable	Not estimable	The outcome was presented graphically with Tukey-letter annotations; an exact pairwise effect size was not calculated	Exact group means and SEs were presented only in Figures 1-3 and were not reliably extractable
Wang et al. (2023)	Turbot (Scophthalmus maximus)	BCAA balancing to estimated requirement levels	Control (balanced BCAAs: Leu 30.4, Ile 19.7, Val 17.4 g/kg)	Leu-L (Leu deficient: 20.9 g/kg)	Muscle s6kb1b mRNA expression (Figure 3B)	Not reported numerically	Not reported numerically	3	3	Not estimable	Not estimable	Not estimable	The outcome was presented graphically with Tukey-letter annotations; an exact pairwise effect size was not calculated	Exact group means and SEs were presented only in Figures 1-3 and were not reliably extractable
Wang et al. (2023)	Turbot (Scophthalmus maximus)	BCAA balancing to estimated requirement levels	Control (balanced BCAAs: Leu 30.4, Ile 19.7, Val 17.4 g/kg)	Leu-L (Leu deficient: 20.9 g/kg)	Muscle 4e-bp1 mRNA expression (Figure 3B)	Not reported numerically	Not reported numerically	3	3	Not estimable	Not estimable	Not estimable	The outcome was presented graphically with Tukey-letter annotations; an exact pairwise effect size was not calculated	Exact group means and SEs were presented only in Figures 1-3 and were not reliably extractable
Wang et al. (2023)	Turbot (Scophthalmus maximus)	BCAA balancing to estimated requirement levels	Control (balanced BCAAs: Leu 30.4, Ile 19.7, Val 17.4 g/kg)	Leu-H (Leu excess: 75.5 g/kg)	b0at1 mRNA expression (Figure 1A)	Not reported numerically	Not reported numerically	3	3	Not estimable	Not estimable	Not estimable	The outcome was presented graphically with Tukey-letter annotations; an exact pairwise effect size was not calculated	Exact group means and SEs were presented only in Figures 1-3 and were not reliably extractable
Wang et al. (2023)	Turbot (Scophthalmus maximus)	BCAA balancing to estimated requirement levels	Control (balanced BCAAs: Leu 30.4, Ile 19.7, Val 17.4 g/kg)	Leu-H (Leu excess: 75.5 g/kg)	b0at2 mRNA expression (Figure 1A)	Not reported numerically	Not reported numerically	3	3	Not estimable	Not estimable	Not estimable	The outcome was presented graphically with Tukey-letter annotations; an exact pairwise effect size was not calculated	Exact group means and SEs were presented only in Figures 1-3 and were not reliably extractable
Wang et al. (2023)	Turbot (Scophthalmus maximus)	BCAA balancing to estimated requirement levels	Control (balanced BCAAs: Leu 30.4, Ile 19.7, Val 17.4 g/kg)	Leu-H (Leu excess: 75.5 g/kg)	lat4 mRNA expression (Figure 1A)	Not reported numerically	Not reported numerically	3	3	Not estimable	Not estimable	Not estimable	The outcome was presented graphically with Tukey-letter annotations; an exact pairwise effect size was not calculated	Exact group means and SEs were presented only in Figures 1-3 and were not reliably extractable
Wang et al. (2023)	Turbot (Scophthalmus maximus)	BCAA balancing to estimated requirement levels	Control (balanced BCAAs: Leu 30.4, Ile 19.7, Val 17.4 g/kg)	Leu-H (Leu excess: 75.5 g/kg)	pept1a mRNA expression (Figure 1B)	Not reported numerically	Not reported numerically	3	3	Not estimable	Not estimable	Not estimable	The outcome was presented graphically with Tukey-letter annotations; an exact pairwise effect size was not calculated	Exact group means and SEs were presented only in Figures 1-3 and were not reliably extractable
Wang et al. (2023)	Turbot (Scophthalmus maximus)	BCAA balancing to estimated requirement levels	Control (balanced BCAAs: Leu 30.4, Ile 19.7, Val 17.4 g/kg)	Leu-H (Leu excess: 75.5 g/kg)	pept1b mRNA expression (Figure 1B)	Not reported numerically	Not reported numerically	3	3	Not estimable	Not estimable	Not estimable	The outcome was presented graphically with Tukey-letter annotations; an exact pairwise effect size was not calculated	Exact group means and SEs were presented only in Figures 1-3 and were not reliably extractable
Wang et al. (2023)	Turbot (Scophthalmus maximus)	BCAA balancing to estimated requirement levels	Control (balanced BCAAs: Leu 30.4, Ile 19.7, Val 17.4 g/kg)	Leu-H (Leu excess: 75.5 g/kg)	Muscle bcat2 mRNA expression (Figure 2)	Not reported numerically	Not reported numerically	3	3	Not estimable	Not estimable	Not estimable	The outcome was presented graphically with Tukey-letter annotations; an exact pairwise effect size was not calculated	Exact group means and SEs were presented only in Figures 1-3 and were not reliably extractable
Wang et al. (2023)	Turbot (Scophthalmus maximus)	BCAA balancing to estimated requirement levels	Control (balanced BCAAs: Leu 30.4, Ile 19.7, Val 17.4 g/kg)	Leu-H (Leu excess: 75.5 g/kg)	Liver bckdha mRNA expression (Figure 2)	Not reported numerically	Not reported numerically	3	3	Not estimable	Not estimable	Not estimable	The outcome was presented graphically with Tukey-letter annotations; an exact pairwise effect size was not calculated	Exact group means and SEs were presented only in Figures 1-3 and were not reliably extractable
Wang et al. (2023)	Turbot (Scophthalmus maximus)	BCAA balancing to estimated requirement levels	Control (balanced BCAAs: Leu 30.4, Ile 19.7, Val 17.4 g/kg)	Leu-H (Leu excess: 75.5 g/kg)	Liver bckdhb mRNA expression (Figure 2)	Not reported numerically	Not reported numerically	3	3	Not estimable	Not estimable	Not estimable	The outcome was presented graphically with Tukey-letter annotations; an exact pairwise effect size was not calculated	Exact group means and SEs were presented only in Figures 1-3 and were not reliably extractable
Wang et al. (2023)	Turbot (Scophthalmus maximus)	BCAA balancing to estimated requirement levels	Control (balanced BCAAs: Leu 30.4, Ile 19.7, Val 17.4 g/kg)	Leu-H (Leu excess: 75.5 g/kg)	Liver bckdk mRNA expression (Figure 2)	Not reported numerically	Not reported numerically	3	3	Not estimable	Not estimable	Not estimable	The outcome was presented graphically with Tukey-letter annotations; an exact pairwise effect size was not calculated	Exact group means and SEs were presented only in Figures 1-3 and were not reliably extractable
Wang et al. (2023)	Turbot (Scophthalmus maximus)	BCAA balancing to estimated requirement levels	Control (balanced BCAAs: Leu 30.4, Ile 19.7, Val 17.4 g/kg)	Leu-H (Leu excess: 75.5 g/kg)	Liver mtor mRNA expression (Figure 3A)	Not reported numerically	Not reported numerically	3	3	Not estimable	Not estimable	Not estimable	The outcome was presented graphically with Tukey-letter annotations; an exact pairwise effect size was not calculated	Exact group means and SEs were presented only in Figures 1-3 and were not reliably extractable
Wang et al. (2023)	Turbot (Scophthalmus maximus)	BCAA balancing to estimated requirement levels	Control (balanced BCAAs: Leu 30.4, Ile 19.7, Val 17.4 g/kg)	Leu-H (Leu excess: 75.5 g/kg)	Liver s6ka mRNA expression (Figure 3A)	Not reported numerically	Not reported numerically	3	3	Not estimable	Not estimable	Not estimable	The outcome was presented graphically with Tukey-letter annotations; an exact pairwise effect size was not calculated	Exact group means and SEs were presented only in Figures 1-3 and were not reliably extractable
Wang et al. (2023)	Turbot (Scophthalmus maximus)	BCAA balancing to estimated requirement levels	Control (balanced BCAAs: Leu 30.4, Ile 19.7, Val 17.4 g/kg)	Leu-H (Leu excess: 75.5 g/kg)	Liver s6kb1a mRNA expression (Figure 3A)	Not reported numerically	Not reported numerically	3	3	Not estimable	Not estimable	Not estimable	The outcome was presented graphically with Tukey-letter annotations; an exact pairwise effect size was not calculated	Exact group means and SEs were presented only in Figures 1-3 and were not reliably extractable
Wang et al. (2023)	Turbot (Scophthalmus maximus)	BCAA balancing to estimated requirement levels	Control (balanced BCAAs: Leu 30.4, Ile 19.7, Val 17.4 g/kg)	Leu-H (Leu excess: 75.5 g/kg)	Liver s6kb1b mRNA expression (Figure 3A)	Not reported numerically	Not reported numerically	3	3	Not estimable	Not estimable	Not estimable	The outcome was presented graphically with Tukey-letter annotations; an exact pairwise effect size was not calculated	Exact group means and SEs were presented only in Figures 1-3 and were not reliably extractable
Wang et al. (2023)	Turbot (Scophthalmus maximus)	BCAA balancing to estimated requirement levels	Control (balanced BCAAs: Leu 30.4, Ile 19.7, Val 17.4 g/kg)	Leu-H (Leu excess: 75.5 g/kg)	Liver 4e-bp1 mRNA expression (Figure 3A)	Not reported numerically	Not reported numerically	3	3	Not estimable	Not estimable	Not estimable	The outcome was presented graphically with Tukey-letter annotations; an exact pairwise effect size was not calculated	Exact group means and SEs were presented only in Figures 1-3 and were not reliably extractable
Wang et al. (2023)	Turbot (Scophthalmus maximus)	BCAA balancing to estimated requirement levels	Control (balanced BCAAs: Leu 30.4, Ile 19.7, Val 17.4 g/kg)	Leu-H (Leu excess: 75.5 g/kg)	Muscle mtor mRNA expression (Figure 3B)	Not reported numerically	Not reported numerically	3	3	Not estimable	Not estimable	Not estimable	The outcome was presented graphically with Tukey-letter annotations; an exact pairwise effect size was not calculated	Exact group means and SEs were presented only in Figures 1-3 and were not reliably extractable
Wang et al. (2023)	Turbot (Scophthalmus maximus)	BCAA balancing to estimated requirement levels	Control (balanced BCAAs: Leu 30.4, Ile 19.7, Val 17.4 g/kg)	Leu-H (Leu excess: 75.5 g/kg)	Muscle s6ka mRNA expression (Figure 3B)	Not reported numerically	Not reported numerically	3	3	Not estimable	Not estimable	Not estimable	The outcome was presented graphically with Tukey-letter annotations; an exact pairwise effect size was not calculated	Exact group means and SEs were presented only in Figures 1-3 and were not reliably extractable
Wang et al. (2023)	Turbot (Scophthalmus maximus)	BCAA balancing to estimated requirement levels	Control (balanced BCAAs: Leu 30.4, Ile 19.7, Val 17.4 g/kg)	Leu-H (Leu excess: 75.5 g/kg)	Muscle s6kb1a mRNA expression (Figure 3B)	Not reported numerically	Not reported numerically	3	3	Not estimable	Not estimable	Not estimable	The outcome was presented graphically with Tukey-letter annotations; an exact pairwise effect size was not calculated	Exact group means and SEs were presented only in Figures 1-3 and were not reliably extractable

Study	Species	Strategy	Intervention	Comparator	Outcome and unit	Intervention mean ± SD	Comparator mean ± SD	n intervention (tanks)	n comparator (tanks)	Effect-size metric	Effect size	95% CI	Interpretation	Reason not estimated
Wang et al. (2023)	Turbot (Scophthalmus maximus)	BCAA balancing to estimated requirement levels	Control (balanced BCAAs: Leu 30.4, Ile 19.7, Val 17.4 g/kg)	Leu-H (Leu excess: 75.5 g/kg)	Muscle s6kb1b mRNA expression (Figure 3B)	Not reported numerically	Not reported numerically	3	3	Not estimable	Not estimable	Not estimable	The outcome was presented graphically with Tukey-letter annotations; an exact pairwise effect size was not calculated	Exact group means and SEs were presented only in Figures 1-3 and were not reliably extractable
Wang et al. (2023)	Turbot (Scophthalmus maximus)	BCAA balancing to estimated requirement levels	Control (balanced BCAAs: Leu 30.4, Ile 19.7, Val 17.4 g/kg)	Leu-H (Leu excess: 75.5 g/kg)	Muscle 4e-bp1 mRNA expression (Figure 3B)	Not reported numerically	Not reported numerically	3	3	Not estimable	Not estimable	Not estimable	The outcome was presented graphically with Tukey-letter annotations; an exact pairwise effect size was not calculated	Exact group means and SEs were presented only in Figures 1-3 and were not reliably extractable
Wang et al. (2023)	Turbot (Scophthalmus maximus)	BCAA balancing to estimated requirement levels	Control (balanced BCAAs: Leu 30.4, Ile 19.7, Val 17.4 g/kg)	Ile-H (Ile excess: 52.6 g/kg)	b0at1 mRNA expression (Figure 1A)	Not reported numerically	Not reported numerically	3	3	Not estimable	Not estimable	Not estimable	The outcome was presented graphically with Tukey-letter annotations; an exact pairwise effect size was not calculated	Exact group means and SEs were presented only in Figures 1-3 and were not reliably extractable
Wang et al. (2023)	Turbot (Scophthalmus maximus)	BCAA balancing to estimated requirement levels	Control (balanced BCAAs: Leu 30.4, Ile 19.7, Val 17.4 g/kg)	Ile-H (Ile excess: 52.6 g/kg)	b0at2 mRNA expression (Figure 1A)	Not reported numerically	Not reported numerically	3	3	Not estimable	Not estimable	Not estimable	The outcome was presented graphically with Tukey-letter annotations; an exact pairwise effect size was not calculated	Exact group means and SEs were presented only in Figures 1-3 and were not reliably extractable
Wang et al. (2023)	Turbot (Scophthalmus maximus)	BCAA balancing to estimated requirement levels	Control (balanced BCAAs: Leu 30.4, Ile 19.7, Val 17.4 g/kg)	Ile-H (Ile excess: 52.6 g/kg)	lat4 mRNA expression (Figure 1A)	Not reported numerically	Not reported numerically	3	3	Not estimable	Not estimable	Not estimable	The outcome was presented graphically with Tukey-letter annotations; an exact pairwise effect size was not calculated	Exact group means and SEs were presented only in Figures 1-3 and were not reliably extractable
Wang et al. (2023)	Turbot (Scophthalmus maximus)	BCAA balancing to estimated requirement levels	Control (balanced BCAAs: Leu 30.4, Ile 19.7, Val 17.4 g/kg)	Ile-H (Ile excess: 52.6 g/kg)	pept1a mRNA expression (Figure 1B)	Not reported numerically	Not reported numerically	3	3	Not estimable	Not estimable	Not estimable	The outcome was presented graphically with Tukey-letter annotations; an exact pairwise effect size was not calculated	Exact group means and SEs were presented only in Figures 1-3 and were not reliably extractable
Wang et al. (2023)	Turbot (Scophthalmus maximus)	BCAA balancing to estimated requirement levels	Control (balanced BCAAs: Leu 30.4, Ile 19.7, Val 17.4 g/kg)	Ile-H (Ile excess: 52.6 g/kg)	pept1b mRNA expression (Figure 1B)	Not reported numerically	Not reported numerically	3	3	Not estimable	Not estimable	Not estimable	The outcome was presented graphically with Tukey-letter annotations; an exact pairwise effect size was not calculated	Exact group means and SEs were presented only in Figures 1-3 and were not reliably extractable
Wang et al. (2023)	Turbot (Scophthalmus maximus)	BCAA balancing to estimated requirement levels	Control (balanced BCAAs: Leu 30.4, Ile 19.7, Val 17.4 g/kg)	Ile-H (Ile excess: 52.6 g/kg)	Muscle bcat2 mRNA expression (Figure 2)	Not reported numerically	Not reported numerically	3	3	Not estimable	Not estimable	Not estimable	The outcome was presented graphically with Tukey-letter annotations; an exact pairwise effect size was not calculated	Exact group means and SEs were presented only in Figures 1-3 and were not reliably extractable
Wang et al. (2023)	Turbot (Scophthalmus maximus)	BCAA balancing to estimated requirement levels	Control (balanced BCAAs: Leu 30.4, Ile 19.7, Val 17.4 g/kg)	Ile-H (Ile excess: 52.6 g/kg)	Liver bckdha mRNA expression (Figure 2)	Not reported numerically	Not reported numerically	3	3	Not estimable	Not estimable	Not estimable	The outcome was presented graphically with Tukey-letter annotations; an exact pairwise effect size was not calculated	Exact group means and SEs were presented only in Figures 1-3 and were not reliably extractable
Wang et al. (2023)	Turbot (Scophthalmus maximus)	BCAA balancing to estimated requirement levels	Control (balanced BCAAs: Leu 30.4, Ile 19.7, Val 17.4 g/kg)	Ile-H (Ile excess: 52.6 g/kg)	Liver bckdhb mRNA expression (Figure 2)	Not reported numerically	Not reported numerically	3	3	Not estimable	Not estimable	Not estimable	The outcome was presented graphically with Tukey-letter annotations; an exact pairwise effect size was not calculated	Exact group means and SEs were presented only in Figures 1-3 and were not reliably extractable
Wang et al. (2023)	Turbot (Scophthalmus maximus)	BCAA balancing to estimated requirement levels	Control (balanced BCAAs: Leu 30.4, Ile 19.7, Val 17.4 g/kg)	Ile-H (Ile excess: 52.6 g/kg)	Liver bckdk mRNA expression (Figure 2)	Not reported numerically	Not reported numerically	3	3	Not estimable	Not estimable	Not estimable	The outcome was presented graphically with Tukey-letter annotations; an exact pairwise effect size was not calculated	Exact group means and SEs were presented only in Figures 1-3 and were not reliably extractable
Wang et al. (2023)	Turbot (Scophthalmus maximus)	BCAA balancing to estimated requirement levels	Control (balanced BCAAs: Leu 30.4, Ile 19.7, Val 17.4 g/kg)	Ile-H (Ile excess: 52.6 g/kg)	Liver mtor mRNA expression (Figure 3A)	Not reported numerically	Not reported numerically	3	3	Not estimable	Not estimable	Not estimable	The outcome was presented graphically with Tukey-letter annotations; an exact pairwise effect size was not calculated	Exact group means and SEs were presented only in Figures 1-3 and were not reliably extractable
Wang et al. (2023)	Turbot (Scophthalmus maximus)	BCAA balancing to estimated requirement levels	Control (balanced BCAAs: Leu 30.4, Ile 19.7, Val 17.4 g/kg)	Ile-H (Ile excess: 52.6 g/kg)	Liver s6ka mRNA expression (Figure 3A)	Not reported numerically	Not reported numerically	3	3	Not estimable	Not estimable	Not estimable	The outcome was presented graphically with Tukey-letter annotations; an exact pairwise effect size was not calculated	Exact group means and SEs were presented only in Figures 1-3 and were not reliably extractable
Wang et al. (2023)	Turbot (Scophthalmus maximus)	BCAA balancing to estimated requirement levels	Control (balanced BCAAs: Leu 30.4, Ile 19.7, Val 17.4 g/kg)	Ile-H (Ile excess: 52.6 g/kg)	Liver s6kb1a mRNA expression (Figure 3A)	Not reported numerically	Not reported numerically	3	3	Not estimable	Not estimable	Not estimable	The outcome was presented graphically with Tukey-letter annotations; an exact pairwise effect size was not calculated	Exact group means and SEs were presented only in Figures 1-3 and were not reliably extractable
Wang et al. (2023)	Turbot (Scophthalmus maximus)	BCAA balancing to estimated requirement levels	Control (balanced BCAAs: Leu 30.4, Ile 19.7, Val 17.4 g/kg)	Ile-H (Ile excess: 52.6 g/kg)	Liver s6kb1b mRNA expression (Figure 3A)	Not reported numerically	Not reported numerically	3	3	Not estimable	Not estimable	Not estimable	The outcome was presented graphically with Tukey-letter annotations; an exact pairwise effect size was not calculated	Exact group means and SEs were presented only in Figures 1-3 and were not reliably extractable
Wang et al. (2023)	Turbot (Scophthalmus maximus)	BCAA balancing to estimated requirement levels	Control (balanced BCAAs: Leu 30.4, Ile 19.7, Val 17.4 g/kg)	Ile-H (Ile excess: 52.6 g/kg)	Liver 4e-bp1 mRNA expression (Figure 3A)	Not reported numerically	Not reported numerically	3	3	Not estimable	Not estimable	Not estimable	The outcome was presented graphically with Tukey-letter annotations; an exact pairwise effect size was not calculated	Exact group means and SEs were presented only in Figures 1-3 and were not reliably extractable
Wang et al. (2023)	Turbot (Scophthalmus maximus)	BCAA balancing to estimated requirement levels	Control (balanced BCAAs: Leu 30.4, Ile 19.7, Val 17.4 g/kg)	Ile-H (Ile excess: 52.6 g/kg)	Muscle mtor mRNA expression (Figure 3B)	Not reported numerically	Not reported numerically	3	3	Not estimable	Not estimable	Not estimable	The outcome was presented graphically with Tukey-letter annotations; an exact pairwise effect size was not calculated	Exact group means and SEs were presented only in Figures 1-3 and were not reliably extractable
Wang et al. (2023)	Turbot (Scophthalmus maximus)	BCAA balancing to estimated requirement levels	Control (balanced BCAAs: Leu 30.4, Ile 19.7, Val 17.4 g/kg)	Ile-H (Ile excess: 52.6 g/kg)	Muscle s6ka mRNA expression (Figure 3B)	Not reported numerically	Not reported numerically	3	3	Not estimable	Not estimable	Not estimable	The outcome was presented graphically with Tukey-letter annotations; an exact pairwise effect size was not calculated	Exact group means and SEs were presented only in Figures 1-3 and were not reliably extractable
Wang et al. (2023)	Turbot (Scophthalmus maximus)	BCAA balancing to estimated requirement levels	Control (balanced BCAAs: Leu 30.4, Ile 19.7, Val 17.4 g/kg)	Ile-H (Ile excess: 52.6 g/kg)	Muscle s6kb1a mRNA expression (Figure 3B)	Not reported numerically	Not reported numerically	3	3	Not estimable	Not estimable	Not estimable	The outcome was presented graphically with Tukey-letter annotations; an exact pairwise effect size was not calculated	Exact group means and SEs were presented only in Figures 1-3 and were not reliably extractable
Wang et al. (2023)	Turbot (Scophthalmus maximus)	BCAA balancing to estimated requirement levels	Control (balanced BCAAs: Leu 30.4, Ile 19.7, Val 17.4 g/kg)	Ile-H (Ile excess: 52.6 g/kg)	Muscle s6kb1b mRNA expression (Figure 3B)	Not reported numerically	Not reported numerically	3	3	Not estimable	Not estimable	Not estimable	The outcome was presented graphically with Tukey-letter annotations; an exact pairwise effect size was not calculated	Exact group means and SEs were presented only in Figures 1-3 and were not reliably extractable
Wang et al. (2023)	Turbot (Scophthalmus maximus)	BCAA balancing to estimated requirement levels	Control (balanced BCAAs: Leu 30.4, Ile 19.7, Val 17.4 g/kg)	Ile-H (Ile excess: 52.6 g/kg)	Muscle 4e-bp1 mRNA expression (Figure 3B)	Not reported numerically	Not reported numerically	3	3	Not estimable	Not estimable	Not estimable	The outcome was presented graphically with Tukey-letter annotations; an exact pairwise effect size was not calculated	Exact group means and SEs were presented only in Figures 1-3 and were not reliably extractable
Wang et al. (2023)	Turbot (Scophthalmus maximus)	BCAA balancing to estimated requirement levels	Control (balanced BCAAs: Leu 30.4, Ile 19.7, Val 17.4 g/kg)	Val-H (Val excess: 46.6 g/kg)	b0at1 mRNA expression (Figure 1A)	Not reported numerically	Not reported numerically	3	3	Not estimable	Not estimable	Not estimable	The outcome was presented graphically with Tukey-letter annotations; an exact pairwise effect size was not calculated	Exact group means and SEs were presented only in Figures 1-3 and were not reliably extractable
Wang et al. (2023)	Turbot (Scophthalmus maximus)	BCAA balancing to estimated requirement levels	Control (balanced BCAAs: Leu 30.4, Ile 19.7, Val 17.4 g/kg)	Val-H (Val excess: 46.6 g/kg)	b0at2 mRNA expression (Figure 1A)	Not reported numerically	Not reported numerically	3	3	Not estimable	Not estimable	Not estimable	The outcome was presented graphically with Tukey-letter annotations; an exact pairwise effect size was not calculated	Exact group means and SEs were presented only in Figures 1-3 and were not reliably extractable
Wang et al. (2023)	Turbot (Scophthalmus maximus)	BCAA balancing to estimated requirement levels	Control (balanced BCAAs: Leu 30.4, Ile 19.7, Val 17.4 g/kg)	Val-H (Val excess: 46.6 g/kg)	lat4 mRNA expression (Figure 1A)	Not reported numerically	Not reported numerically	3	3	Not estimable	Not estimable	Not estimable	The outcome was presented graphically with Tukey-letter annotations; an exact pairwise effect size was not calculated	Exact group means and SEs were presented only in Figures 1-3 and were not reliably extractable
Wang et al. (2023)	Turbot (Scophthalmus maximus)	BCAA balancing to estimated requirement levels	Control (balanced BCAAs: Leu 30.4, Ile 19.7, Val 17.4 g/kg)	Val-H (Val excess: 46.6 g/kg)	pept1a mRNA expression (Figure 1B)	Not reported numerically	Not reported numerically	3	3	Not estimable	Not estimable	Not estimable	The outcome was presented graphically with Tukey-letter annotations; an exact pairwise effect size was not calculated	Exact group means and SEs were presented only in Figures 1-3 and were not reliably extractable
Wang et al. (2023)	Turbot (Scophthalmus maximus)	BCAA balancing to estimated requirement levels	Control (balanced BCAAs: Leu 30.4, Ile 19.7, Val 17.4 g/kg)	Val-H (Val excess: 46.6 g/kg)	pept1b mRNA expression (Figure 1B)	Not reported numerically	Not reported numerically	3	3	Not estimable	Not estimable	Not estimable	The outcome was presented graphically with Tukey-letter annotations; an exact pairwise effect size was not calculated	Exact group means and SEs were presented only in Figures 1-3 and were not reliably extractable
Wang et al. (2023)	Turbot (Scophthalmus maximus)	BCAA balancing to estimated requirement levels	Control (balanced BCAAs: Leu 30.4, Ile 19.7, Val 17.4 g/kg)	Val-H (Val excess: 46.6 g/kg)	Muscle bcat2 mRNA expression (Figure 2)	Not reported numerically	Not reported numerically	3	3	Not estimable	Not estimable	Not estimable	The outcome was presented graphically with Tukey-letter annotations; an exact pairwise effect size was not calculated	Exact group means and SEs were presented only in Figures 1-3 and were not reliably extractable
Wang et al. (2023)	Turbot (Scophthalmus maximus)	BCAA balancing to estimated requirement levels	Control (balanced BCAAs: Leu 30.4, Ile 19.7, Val 17.4 g/kg)	Val-H (Val excess: 46.6 g/kg)	Liver bckdha mRNA expression (Figure 2)	Not reported numerically	Not reported numerically	3	3	Not estimable	Not estimable	Not estimable	The outcome was presented graphically with Tukey-letter annotations; an exact pairwise effect size was not calculated	Exact group means and SEs were presented only in Figures 1-3 and were not reliably extractable
Wang et al. (2023)	Turbot (Scophthalmus maximus)	BCAA balancing to estimated requirement levels	Control (balanced BCAAs: Leu 30.4, Ile 19.7, Val 17.4 g/kg)	Val-H (Val excess: 46.6 g/kg)	Liver bckdhb mRNA expression (Figure 2)	Not reported numerically	Not reported numerically	3	3	Not estimable	Not estimable	Not estimable	The outcome was presented graphically with Tukey-letter annotations; an exact pairwise effect size was not calculated	Exact group means and SEs were presented only in Figures 1-3 and were not reliably extractable
Wang et al. (2023)	Turbot (Scophthalmus maximus)	BCAA balancing to estimated requirement levels	Control (balanced BCAAs: Leu 30.4, Ile 19.7, Val 17.4 g/kg)	Val-H (Val excess: 46.6 g/kg)	Liver bckdk mRNA expression (Figure 2)	Not reported numerically	Not reported numerically	3	3	Not estimable	Not estimable	Not estimable	The outcome was presented graphically with Tukey-letter annotations; an exact pairwise effect size was not calculated	Exact group means and SEs were presented only in Figures 1-3 and were not reliably extractable

Study	Species	Strategy	Intervention	Comparator	Outcome and unit	Intervention mean ± SD	Comparator mean ± SD	n intervention (tanks)	n comparator (tanks)	Effect-size metric	Effect size	95% CI	Interpretation	Reason not estimated
Wang et al. (2023)	Turbot (Scophthalmus maximus)	BCAA balancing to estimated requirement levels	Control (balanced BCAAs: Leu 30.4, Ile 19.7, Val 17.4 g/kg)	Val-H (Val excess: 46.6 g/kg)	Liver mtor mRNA expression (Figure 3A)	Not reported numerically	Not reported numerically	3	3	Not estimable	Not estimable	Not estimable	The outcome was presented graphically with Tukey-letter annotations; an exact pairwise effect size was not calculated	Exact group means and SEs were presented only in Figures 1-3 and were not reliably extractable
Wang et al. (2023)	Turbot (Scophthalmus maximus)	BCAA balancing to estimated requirement levels	Control (balanced BCAAs: Leu 30.4, Ile 19.7, Val 17.4 g/kg)	Val-H (Val excess: 46.6 g/kg)	Liver s6ka mRNA expression (Figure 3A)	Not reported numerically	Not reported numerically	3	3	Not estimable	Not estimable	Not estimable	The outcome was presented graphically with Tukey-letter annotations; an exact pairwise effect size was not calculated	Exact group means and SEs were presented only in Figures 1-3 and were not reliably extractable
Wang et al. (2023)	Turbot (Scophthalmus maximus)	BCAA balancing to estimated requirement levels	Control (balanced BCAAs: Leu 30.4, Ile 19.7, Val 17.4 g/kg)	Val-H (Val excess: 46.6 g/kg)	Liver s6kb1a mRNA expression (Figure 3A)	Not reported numerically	Not reported numerically	3	3	Not estimable	Not estimable	Not estimable	The outcome was presented graphically with Tukey-letter annotations; an exact pairwise effect size was not calculated	Exact group means and SEs were presented only in Figures 1-3 and were not reliably extractable
Wang et al. (2023)	Turbot (Scophthalmus maximus)	BCAA balancing to estimated requirement levels	Control (balanced BCAAs: Leu 30.4, Ile 19.7, Val 17.4 g/kg)	Val-H (Val excess: 46.6 g/kg)	Liver s6kb1b mRNA expression (Figure 3A)	Not reported numerically	Not reported numerically	3	3	Not estimable	Not estimable	Not estimable	The outcome was presented graphically with Tukey-letter annotations; an exact pairwise effect size was not calculated	Exact group means and SEs were presented only in Figures 1-3 and were not reliably extractable
Wang et al. (2023)	Turbot (Scophthalmus maximus)	BCAA balancing to estimated requirement levels	Control (balanced BCAAs: Leu 30.4, Ile 19.7, Val 17.4 g/kg)	Val-H (Val excess: 46.6 g/kg)	Liver 4e-bp1 mRNA expression (Figure 3A)	Not reported numerically	Not reported numerically	3	3	Not estimable	Not estimable	Not estimable	The outcome was presented graphically with Tukey-letter annotations; an exact pairwise effect size was not calculated	Exact group means and SEs were presented only in Figures 1-3 and were not reliably extractable
Wang et al. (2023)	Turbot (Scophthalmus maximus)	BCAA balancing to estimated requirement levels	Control (balanced BCAAs: Leu 30.4, Ile 19.7, Val 17.4 g/kg)	Val-H (Val excess: 46.6 g/kg)	Muscle mtor mRNA expression (Figure 3B)	Not reported numerically	Not reported numerically	3	3	Not estimable	Not estimable	Not estimable	The outcome was presented graphically with Tukey-letter annotations; an exact pairwise effect size was not calculated	Exact group means and SEs were presented only in Figures 1-3 and were not reliably extractable
Wang et al. (2023)	Turbot (Scophthalmus maximus)	BCAA balancing to estimated requirement levels	Control (balanced BCAAs: Leu 30.4, Ile 19.7, Val 17.4 g/kg)	Val-H (Val excess: 46.6 g/kg)	Muscle s6ka mRNA expression (Figure 3B)	Not reported numerically	Not reported numerically	3	3	Not estimable	Not estimable	Not estimable	The outcome was presented graphically with Tukey-letter annotations; an exact pairwise effect size was not calculated	Exact group means and SEs were presented only in Figures 1-3 and were not reliably extractable
Wang et al. (2023)	Turbot (Scophthalmus maximus)	BCAA balancing to estimated requirement levels	Control (balanced BCAAs: Leu 30.4, Ile 19.7, Val 17.4 g/kg)	Val-H (Val excess: 46.6 g/kg)	Muscle s6kb1a mRNA expression (Figure 3B)	Not reported numerically	Not reported numerically	3	3	Not estimable	Not estimable	Not estimable	The outcome was presented graphically with Tukey-letter annotations; an exact pairwise effect size was not calculated	Exact group means and SEs were presented only in Figures 1-3 and were not reliably extractable
Wang et al. (2023)	Turbot (Scophthalmus maximus)	BCAA balancing to estimated requirement levels	Control (balanced BCAAs: Leu 30.4, Ile 19.7, Val 17.4 g/kg)	Val-H (Val excess: 46.6 g/kg)	Muscle s6kb1b mRNA expression (Figure 3B)	Not reported numerically	Not reported numerically	3	3	Not estimable	Not estimable	Not estimable	The outcome was presented graphically with Tukey-letter annotations; an exact pairwise effect size was not calculated	Exact group means and SEs were presented only in Figures 1-3 and were not reliably extractable
Wang et al. (2023)	Turbot (Scophthalmus maximus)	BCAA balancing to estimated requirement levels	Control (balanced BCAAs: Leu 30.4, Ile 19.7, Val 17.4 g/kg)	Val-H (Val excess: 46.6 g/kg)	Muscle 4e-bp1 mRNA expression (Figure 3B)	Not reported numerically	Not reported numerically	3	3	Not estimable	Not estimable	Not estimable	The outcome was presented graphically with Tukey-letter annotations; an exact pairwise effect size was not calculated	Exact group means and SEs were presented only in Figures 1-3 and were not reliably extractable

Note: Values in Tables 4-8 were reported as mean ± SE (n = 3 tanks); SD was calculated as SE × sqrt(3) before computing Hedges' g. Positive effect sizes indicate higher values in the balanced control. This direction is favorable for growth, survival, FE, PER, and PPV, but is descriptive for feed intake, composition, and free BCAA concentrations. Gene-expression outcomes in Figures 1-3 were not estimated because exact numerical means and SEs were unavailable. Pairwise 95% CIs are unadjusted and may not match the original Tukey test.

Source: Wang et al. (2023), https://doi.org/10.3390/fishes8110534

Study 9. Shi et al. (2024): hydroxyproline-supplemented versus unsupplemented 10% fishmeal diets

Study	Species	Strategy	Intervention	Comparator	Outcome and unit	Intervention mean ± SD	Comparator mean ± SD	n intervention (tanks)	n comparator (tanks)	Effect-size metric	Effect size	95% CI	Interpretation	Reason not estimated
Shi et al. (2024)	Pacific white shrimp (Litopenaeus vannamei)	Hydroxyproline supplementation in a 10% fishmeal diet	LF2 (10% fishmeal; 0.2% hydroxyproline)	LF0 (10% fishmeal; 0% hydroxyproline)	Final body weight (g)	8.890 ± 0.265	8.670 ± 0.480	3	3	Hedges' g	0.45	-1.18 to 2.09	Higher, more favorable value with hydroxyproline; 95% CI included 0 (imprecise estimate)	Not applicable - effect size estimated after converting SEM to SD
Shi et al. (2024)	Pacific white shrimp (Litopenaeus vannamei)	Hydroxyproline supplementation in a 10% fishmeal diet	LF2 (10% fishmeal; 0.2% hydroxyproline)	LF0 (10% fishmeal; 0% hydroxyproline)	Weight gain rate (%)	2679.120 ± 83.518	2610.340 ± 150.941	3	3	Hedges' g	0.45	-1.18 to 2.08	Higher, more favorable value with hydroxyproline; 95% CI included 0 (imprecise estimate)	Not applicable - effect size estimated after converting SEM to SD
Shi et al. (2024)	Pacific white shrimp (Litopenaeus vannamei)	Hydroxyproline supplementation in a 10% fishmeal diet	LF2 (10% fishmeal; 0.2% hydroxyproline)	LF0 (10% fishmeal; 0% hydroxyproline)	Specific growth rate (%/day)	6.400 ± 0.059	6.340 ± 0.107	3	3	Hedges' g	0.55	-1.09 to 2.20	Higher, more favorable value with hydroxyproline; 95% CI included 0 (imprecise estimate)	Not applicable - effect size estimated after converting SEM to SD
Shi et al. (2024)	Pacific white shrimp (Litopenaeus vannamei)	Hydroxyproline supplementation in a 10% fishmeal diet	LF2 (10% fishmeal; 0.2% hydroxyproline)	LF0 (10% fishmeal; 0% hydroxyproline)	Survival rate (%)	96.670 ± 2.887	95.330 ± 4.619	3	3	Hedges' g	0.28	-1.33 to 1.89	Higher, more favorable value with hydroxyproline; 95% CI included 0 (imprecise estimate)	Not applicable - effect size estimated after converting SEM to SD
Shi et al. (2024)	Pacific white shrimp (Litopenaeus vannamei)	Hydroxyproline supplementation in a 10% fishmeal diet	LF2 (10% fishmeal; 0.2% hydroxyproline)	LF0 (10% fishmeal; 0% hydroxyproline)	Feed conversion ratio	1.260 ± 0.048	1.350 ± 0.094	3	3	Hedges' g (direction reversed)	0.97	-0.77 to 2.70	Lower, more favorable value with hydroxyproline; 95% CI included 0 (imprecise estimate)	Not applicable - effect size estimated after converting SEM to SD
Shi et al. (2024)	Pacific white shrimp (Litopenaeus vannamei)	Hydroxyproline supplementation in a 10% fishmeal diet	LF2 (10% fishmeal; 0.2% hydroxyproline)	LF0 (10% fishmeal; 0% hydroxyproline)	Feed intake (g/shrimp)	10.780 ± 0.289	11.240 ± 0.154	3	3	Hedges' g	-1.59	-3.53 to 0.35	Lower measured value with hydroxyproline; 95% CI included 0 (imprecise estimate)	Not applicable - effect size estimated after converting SEM to SD
Shi et al. (2024)	Pacific white shrimp (Litopenaeus vannamei)	Hydroxyproline supplementation in a 10% fishmeal diet	LF2 (10% fishmeal; 0.2% hydroxyproline)	LF0 (10% fishmeal; 0% hydroxyproline)	Meat yield (%)	40.890 ± 0.360	42.990 ± 0.748	3	3	Hedges' g	-2.86	-5.41 to -0.31	Lower, less favorable value with hydroxyproline; 95% CI excluded 0	Not applicable - effect size estimated after converting SEM to SD
Shi et al. (2024)	Pacific white shrimp (Litopenaeus vannamei)	Hydroxyproline supplementation in a 10% fishmeal diet	LF2 (10% fishmeal; 0.2% hydroxyproline)	LF0 (10% fishmeal; 0% hydroxyproline)	Hardness (N)	957.940 ± 23.341	791.160 ± 26.618	3	3	Hedges' g	5.33	1.30 to 9.36	Higher, more favorable value with hydroxyproline; 95% CI excluded 0	Not applicable - effect size estimated after converting SEM to SD
Shi et al. (2024)	Pacific white shrimp (Litopenaeus vannamei)	Hydroxyproline supplementation in a 10% fishmeal diet	LF2 (10% fishmeal; 0.2% hydroxyproline)	LF0 (10% fishmeal; 0% hydroxyproline)	Springiness (mm)	0.600 ± 0.024	0.540 ± 0.029	3	3	Hedges' g	1.78	-0.24 to 3.80	Higher, more favorable value with hydroxyproline; 95% CI included 0 (imprecise estimate)	Not applicable - effect size estimated after converting SEM to SD
Shi et al. (2024)	Pacific white shrimp (Litopenaeus vannamei)	Hydroxyproline supplementation in a 10% fishmeal diet	LF2 (10% fishmeal; 0.2% hydroxyproline)	LF0 (10% fishmeal; 0% hydroxyproline)	Chewiness (mJ)	227.440 ± 46.991	244.310 ± 23.485	3	3	Hedges' g	-0.36	-1.98 to 1.26	Lower, less favorable value with hydroxyproline; 95% CI included 0 (imprecise estimate)	Not applicable - effect size estimated after converting SEM to SD
Shi et al. (2024)	Pacific white shrimp (Litopenaeus vannamei)	Hydroxyproline supplementation in a 10% fishmeal diet	LF2 (10% fishmeal; 0.2% hydroxyproline)	LF0 (10% fishmeal; 0% hydroxyproline)	Cohesiveness (N)	0.500 ± 0.024	0.460 ± 0.023	3	3	Hedges' g	1.37	-0.49 to 3.23	Higher, more favorable value with hydroxyproline; 95% CI included 0 (imprecise estimate)	Not applicable - effect size estimated after converting SEM to SD
Shi et al. (2024)	Pacific white shrimp (Litopenaeus vannamei)	Hydroxyproline supplementation in a 10% fishmeal diet	LF2 (10% fishmeal; 0.2% hydroxyproline)	LF0 (10% fishmeal; 0% hydroxyproline)	Cooking loss (%)	7.920 ± 0.743	11.660 ± 1.252	3	3	Hedges' g (direction reversed)	2.91	0.33 to 5.48	Lower, more favorable value with hydroxyproline; 95% CI excluded 0	Not applicable - effect size estimated after converting SEM to SD
Shi et al. (2024)	Pacific white shrimp (Litopenaeus vannamei)	Hydroxyproline supplementation in a 10% fishmeal diet	LF2 (10% fishmeal; 0.2% hydroxyproline)	LF0 (10% fishmeal; 0% hydroxyproline)	Freezing loss (%)	1.130 ± 0.035	1.270 ± 0.010	3	3	Hedges' g (direction reversed)	4.38	0.95 to 7.81	Lower, more favorable value with hydroxyproline; 95% CI excluded 0	Not applicable - effect size estimated after converting SEM to SD
Shi et al. (2024)	Pacific white shrimp (Litopenaeus vannamei)	Hydroxyproline supplementation in a 10% fishmeal diet	LF4 (10% fishmeal; 0.4% hydroxyproline)	LF0 (10% fishmeal; 0% hydroxyproline)	Final body weight (g)	9.290 ± 0.199	8.670 ± 0.480	3	3	Hedges' g	1.35	-0.50 to 3.20	Higher, more favorable value with hydroxyproline; 95% CI included 0 (imprecise estimate)	Not applicable - effect size estimated after converting SEM to SD
Shi et al. (2024)	Pacific white shrimp (Litopenaeus vannamei)	Hydroxyproline supplementation in a 10% fishmeal diet	LF4 (10% fishmeal; 0.4% hydroxyproline)	LF0 (10% fishmeal; 0% hydroxyproline)	Weight gain rate (%)	2803.860 ± 61.063	2610.340 ± 150.941	3	3	Hedges' g	1.34	-0.51 to 3.20	Higher, more favorable value with hydroxyproline; 95% CI included 0 (imprecise estimate)	Not applicable - effect size estimated after converting SEM to SD
Shi et al. (2024)	Pacific white shrimp (Litopenaeus vannamei)	Hydroxyproline supplementation in a 10% fishmeal diet	LF4 (10% fishmeal; 0.4% hydroxyproline)	LF0 (10% fishmeal; 0% hydroxyproline)	Specific growth rate (%/day)	6.480 ± 0.038	6.340 ± 0.107	3	3	Hedges' g	1.39	-0.48 to 3.26	Higher, more favorable value with hydroxyproline; 95% CI included 0 (imprecise estimate)	Not applicable - effect size estimated after converting SEM to SD
Shi et al. (2024)	Pacific white shrimp (Litopenaeus vannamei)	Hydroxyproline supplementation in a 10% fishmeal diet	LF4 (10% fishmeal; 0.4% hydroxyproline)	LF0 (10% fishmeal; 0% hydroxyproline)	Survival rate (%)	96.000 ± 1.732	95.330 ± 4.619	3	3	Hedges' g	0.15	-1.45 to 1.76	Higher, more favorable value with hydroxyproline; 95% CI included 0 (imprecise estimate)	Not applicable - effect size estimated after converting SEM to SD
Shi et al. (2024)	Pacific white shrimp (Litopenaeus vannamei)	Hydroxyproline supplementation in a 10% fishmeal diet	LF4 (10% fishmeal; 0.4% hydroxyproline)	LF0 (10% fishmeal; 0% hydroxyproline)	Feed conversion ratio	1.220 ± 0.069	1.350 ± 0.094	3	3	Hedges' g (direction reversed)	1.26	-0.56 to 3.09	Lower, more favorable value with hydroxyproline; 95% CI included 0 (imprecise estimate)	Not applicable - effect size estimated after converting SEM to SD
Shi et al. (2024)	Pacific white shrimp (Litopenaeus vannamei)	Hydroxyproline supplementation in a 10% fishmeal diet	LF4 (10% fishmeal; 0.4% hydroxyproline)	LF0 (10% fishmeal; 0% hydroxyproline)	Feed intake (g/shrimp)	10.910 ± 0.412	11.240 ± 0.154	3	3	Hedges' g	-0.85	-2.55 to 0.86	Lower measured value with hydroxyproline; 95% CI included 0 (imprecise estimate)	Not applicable - effect size estimated after converting SEM to SD
Shi et al. (2024)	Pacific white shrimp (Litopenaeus vannamei)	Hydroxyproline supplementation in a 10% fishmeal diet	LF4 (10% fishmeal; 0.4% hydroxyproline)	LF0 (10% fishmeal; 0% hydroxyproline)	Meat yield (%)	46.220 ± 0.516	42.990 ± 0.748	3	3	Hedges' g	4.02	0.81 to 7.23	Higher, more favorable value with hydroxyproline; 95% CI excluded 0	Not applicable - effect size estimated after converting SEM to SD
Shi et al. (2024)	Pacific white shrimp (Litopenaeus vannamei)	Hydroxyproline supplementation in a 10% fishmeal diet	LF4 (10% fishmeal; 0.4% hydroxyproline)	LF0 (10% fishmeal; 0% hydroxyproline)	Hardness (N)	1000.430 ± 65.336	791.160 ± 26.618	3	3	Hedges' g	3.36	0.53 to 6.18	Higher, more favorable value with hydroxyproline; 95% CI excluded 0	Not applicable - effect size estimated after converting SEM to SD
Shi et al. (2024)	Pacific white shrimp (Litopenaeus vannamei)	Hydroxyproline supplementation in a 10% fishmeal diet	LF4 (10% fishmeal; 0.4% hydroxyproline)	LF0 (10% fishmeal; 0% hydroxyproline)	Springiness (mm)	0.660 ± 0.017	0.540 ± 0.029	3	3	Hedges' g	3.97	0.79 to 7.16	Higher, more favorable value with hydroxyproline; 95% CI excluded 0	Not applicable - effect size estimated after converting SEM to SD
Shi et al. (2024)	Pacific white shrimp (Litopenaeus vannamei)	Hydroxyproline supplementation in a 10% fishmeal diet	LF4 (10% fishmeal; 0.4% hydroxyproline)	LF0 (10% fishmeal; 0% hydroxyproline)	Chewiness (mJ)	374.080 ± 49.263	244.310 ± 23.485	3	3	Hedges' g	2.69	0.23 to 5.15	Higher, more favorable value with hydroxyproline; 95% CI excluded 0	Not applicable - effect size estimated after converting SEM to SD
Shi et al. (2024)	Pacific white shrimp (Litopenaeus vannamei)	Hydroxyproline supplementation in a 10% fishmeal diet	LF4 (10% fishmeal; 0.4% hydroxyproline)	LF0 (10% fishmeal; 0% hydroxyproline)	Cohesiveness (N)	0.560 ± 0.016	0.460 ± 0.023	3	3	Hedges' g	4.13	0.85 to 7.41	Higher, more favorable value with hydroxyproline; 95% CI excluded 0	Not applicable - effect size estimated after converting SEM to SD
Shi et al. (2024)	Pacific white shrimp (Litopenaeus vannamei)	Hydroxyproline supplementation in a 10% fishmeal diet	LF4 (10% fishmeal; 0.4% hydroxyproline)	LF0 (10% fishmeal; 0% hydroxyproline)	Cooking loss (%)	7.490 ± 1.438	11.660 ± 1.252	3	3	Hedges' g (direction reversed)	2.47	0.13 to 4.82	Lower, more favorable value with hydroxyproline; 95% CI excluded 0	Not applicable - effect size estimated after converting SEM to SD
Shi et al. (2024)	Pacific white shrimp (Litopenaeus vannamei)	Hydroxyproline supplementation in a 10% fishmeal diet	LF4 (10% fishmeal; 0.4% hydroxyproline)	LF0 (10% fishmeal; 0% hydroxyproline)	Freezing loss (%)	0.490 ± 0.139	1.270 ± 0.010	3	3	Hedges' g (direction reversed)	6.35	1.67 to 11.03	Lower, more favorable value with hydroxyproline; 95% CI excluded 0	Not applicable - effect size estimated after converting SEM to SD
Shi et al. (2024)	Pacific white shrimp (Litopenaeus vannamei)	Hydroxyproline supplementation in a 10% fishmeal diet	LF6 (10% fishmeal; 0.6% hydroxyproline)	LF0 (10% fishmeal; 0% hydroxyproline)	Final body weight (g)	8.570 ± 0.187	8.670 ± 0.480	3	3	Hedges' g	-0.22	-1.83 to 1.39	Lower, less favorable value with hydroxyproline; 95% CI included 0 (imprecise estimate)	Not applicable - effect size estimated after converting SEM to SD
Shi et al. (2024)	Pacific white shrimp (Litopenaeus vannamei)	Hydroxyproline supplementation in a 10% fishmeal diet	LF6 (10% fishmeal; 0.6% hydroxyproline)	LF0 (10% fishmeal; 0% hydroxyproline)	Weight gain rate (%)	2579.530 ± 57.958	2610.340 ± 150.941	3	3	Hedges' g	-0.22	-1.82 to 1.39	Lower, less favorable value with hydroxyproline; 95% CI included 0 (imprecise estimate)	Not applicable - effect size estimated after converting SEM to SD
Shi et al. (2024)	Pacific white shrimp (Litopenaeus vannamei)	Hydroxyproline supplementation in a 10% fishmeal diet	LF6 (10% fishmeal; 0.6% hydroxyproline)	LF0 (10% fishmeal; 0% hydroxyproline)	Specific growth rate (%/day)	6.320 ± 0.040	6.340 ± 0.107	3	3	Hedges' g	-0.2	-1.80 to 1.41	Lower, less favorable value with hydroxyproline; 95% CI included 0 (imprecise estimate)	Not applicable - effect size estimated after converting SEM to SD
Shi et al. (2024)	Pacific white shrimp (Litopenaeus vannamei)	Hydroxyproline supplementation in a 10% fishmeal diet	LF6 (10% fishmeal; 0.6% hydroxyproline)	LF0 (10% fishmeal; 0% hydroxyproline)	Survival rate (%)	94.330 ± 6.028	95.330 ± 4.619	3	3	Hedges' g	-0.15	-1.75 to 1.45	Lower, less favorable value with hydroxyproline; 95% CI included 0 (imprecise estimate)	Not applicable - effect size estimated after converting SEM to SD

Study	Species	Strategy	Intervention	Comparator	Outcome and unit	Intervention mean ± SD	Comparator mean ± SD	n intervention (tanks)	n comparator (tanks)	Effect-size metric	Effect size	95% CI	Interpretation	Reason not estimated
Shi et al. (2024)	Pacific white shrimp (Litopenaeus vannamei)	Hydroxyproline supplementation in a 10% fishmeal diet	LF6 (10% fishmeal; 0.6% hydroxyproline)	LF0 (10% fishmeal; 0% hydroxyproline)	Feed conversion ratio	1.290 ± 0.071	1.350 ± 0.094	3	3	Hedges' g (direction reversed)	0.58	-1.07 to 2.23	Lower, more favorable value with hydroxyproline; 95% CI included 0 (imprecise estimate)	Not applicable - effect size estimated after converting SEM to SD
Shi et al. (2024)	Pacific white shrimp (Litopenaeus vannamei)	Hydroxyproline supplementation in a 10% fishmeal diet	LF6 (10% fishmeal; 0.6% hydroxyproline)	LF0 (10% fishmeal; 0% hydroxyproline)	Feed intake (g/shrimp)	10.660 ± 0.433	11.240 ± 0.154	3	3	Hedges' g	-1.43	-3.31 to 0.45	Lower measured value with hydroxyproline; 95% CI included 0 (imprecise estimate)	Not applicable - effect size estimated after converting SEM to SD
Shi et al. (2024)	Pacific white shrimp (Litopenaeus vannamei)	Hydroxyproline supplementation in a 10% fishmeal diet	LF6 (10% fishmeal; 0.6% hydroxyproline)	LF0 (10% fishmeal; 0% hydroxyproline)	Meat yield (%)	44.500 ± 0.284	42.990 ± 0.748	3	3	Hedges' g	2.13	-0.04 to 4.31	Higher, more favorable value with hydroxyproline; 95% CI included 0 (imprecise estimate)	Not applicable - effect size estimated after converting SEM to SD
Shi et al. (2024)	Pacific white shrimp (Litopenaeus vannamei)	Hydroxyproline supplementation in a 10% fishmeal diet	LF6 (10% fishmeal; 0.6% hydroxyproline)	LF0 (10% fishmeal; 0% hydroxyproline)	Hardness (N)	886.670 ± 90.657	791.160 ± 26.618	3	3	Hedges' g	1.14	-0.64 to 2.93	Higher, more favorable value with hydroxyproline; 95% CI included 0 (imprecise estimate)	Not applicable - effect size estimated after converting SEM to SD
Shi et al. (2024)	Pacific white shrimp (Litopenaeus vannamei)	Hydroxyproline supplementation in a 10% fishmeal diet	LF6 (10% fishmeal; 0.6% hydroxyproline)	LF0 (10% fishmeal; 0% hydroxyproline)	Springiness (mm)	0.650 ± 0.023	0.540 ± 0.029	3	3	Hedges' g	3.36	0.53 to 6.18	Higher, more favorable value with hydroxyproline; 95% CI excluded 0	Not applicable - effect size estimated after converting SEM to SD
Shi et al. (2024)	Pacific white shrimp (Litopenaeus vannamei)	Hydroxyproline supplementation in a 10% fishmeal diet	LF6 (10% fishmeal; 0.6% hydroxyproline)	LF0 (10% fishmeal; 0% hydroxyproline)	Chewiness (mJ)	349.980 ± 36.214	244.310 ± 23.485	3	3	Hedges' g	2.77	0.27 to 5.27	Higher, more favorable value with hydroxyproline; 95% CI excluded 0	Not applicable - effect size estimated after converting SEM to SD
Shi et al. (2024)	Pacific white shrimp (Litopenaeus vannamei)	Hydroxyproline supplementation in a 10% fishmeal diet	LF6 (10% fishmeal; 0.6% hydroxyproline)	LF0 (10% fishmeal; 0% hydroxyproline)	Cohesiveness (N)	0.530 ± 0.014	0.460 ± 0.023	3	3	Hedges' g	3.0	0.37 to 5.62	Higher, more favorable value with hydroxyproline; 95% CI excluded 0	Not applicable - effect size estimated after converting SEM to SD
Shi et al. (2024)	Pacific white shrimp (Litopenaeus vannamei)	Hydroxyproline supplementation in a 10% fishmeal diet	LF6 (10% fishmeal; 0.6% hydroxyproline)	LF0 (10% fishmeal; 0% hydroxyproline)	Cooking loss (%)	8.310 ± 0.490	11.660 ± 1.252	3	3	Hedges' g (direction reversed)	2.82	0.29 to 5.34	Lower, more favorable value with hydroxyproline; 95% CI excluded 0	Not applicable - effect size estimated after converting SEM to SD
Shi et al. (2024)	Pacific white shrimp (Litopenaeus vannamei)	Hydroxyproline supplementation in a 10% fishmeal diet	LF6 (10% fishmeal; 0.6% hydroxyproline)	LF0 (10% fishmeal; 0% hydroxyproline)	Freezing loss (%)	0.940 ± 0.263	1.270 ± 0.010	3	3	Hedges' g (direction reversed)	1.42	-0.46 to 3.29	Lower, more favorable value with hydroxyproline; 95% CI included 0 (imprecise estimate)	Not applicable - effect size estimated after converting SEM to SD
Shi et al. (2024)	Pacific white shrimp (Litopenaeus vannamei)	Hydroxyproline supplementation in a 10% fishmeal diet	LF8 (10% fishmeal; 0.8% hydroxyproline)	LF0 (10% fishmeal; 0% hydroxyproline)	Final body weight (g)	8.360 ± 0.296	8.670 ± 0.480	3	3	Hedges' g	-0.62	-2.28 to 1.04	Lower, less favorable value with hydroxyproline; 95% CI included 0 (imprecise estimate)	Not applicable - effect size estimated after converting SEM to SD
Shi et al. (2024)	Pacific white shrimp (Litopenaeus vannamei)	Hydroxyproline supplementation in a 10% fishmeal diet	LF8 (10% fishmeal; 0.8% hydroxyproline)	LF0 (10% fishmeal; 0% hydroxyproline)	Weight gain rate (%)	2509.970 ± 92.926	2610.340 ± 150.941	3	3	Hedges' g	-0.64	-2.30 to 1.02	Lower, less favorable value with hydroxyproline; 95% CI included 0 (imprecise estimate)	Not applicable - effect size estimated after converting SEM to SD
Shi et al. (2024)	Pacific white shrimp (Litopenaeus vannamei)	Hydroxyproline supplementation in a 10% fishmeal diet	LF8 (10% fishmeal; 0.8% hydroxyproline)	LF0 (10% fishmeal; 0% hydroxyproline)	Specific growth rate (%/day)	6.270 ± 0.064	6.340 ± 0.107	3	3	Hedges' g	-0.63	-2.29 to 1.03	Lower, less favorable value with hydroxyproline; 95% CI included 0 (imprecise estimate)	Not applicable - effect size estimated after converting SEM to SD
Shi et al. (2024)	Pacific white shrimp (Litopenaeus vannamei)	Hydroxyproline supplementation in a 10% fishmeal diet	LF8 (10% fishmeal; 0.8% hydroxyproline)	LF0 (10% fishmeal; 0% hydroxyproline)	Survival rate (%)	93.670 ± 4.041	95.330 ± 4.619	3	3	Hedges' g	-0.31	-1.92 to 1.31	Lower, less favorable value with hydroxyproline; 95% CI included 0 (imprecise estimate)	Not applicable - effect size estimated after converting SEM to SD
Shi et al. (2024)	Pacific white shrimp (Litopenaeus vannamei)	Hydroxyproline supplementation in a 10% fishmeal diet	LF8 (10% fishmeal; 0.8% hydroxyproline)	LF0 (10% fishmeal; 0% hydroxyproline)	Feed conversion ratio	1.400 ± 0.071	1.350 ± 0.094	3	3	Hedges' g (direction reversed)	-0.48	-2.12 to 1.15	Higher, less favorable value with hydroxyproline; 95% CI included 0 (imprecise estimate)	Not applicable - effect size estimated after converting SEM to SD
Shi et al. (2024)	Pacific white shrimp (Litopenaeus vannamei)	Hydroxyproline supplementation in a 10% fishmeal diet	LF8 (10% fishmeal; 0.8% hydroxyproline)	LF0 (10% fishmeal; 0% hydroxyproline)	Feed intake (g/shrimp)	11.210 ± 0.589	11.240 ± 0.154	3	3	Hedges' g	-0.06	-1.66 to 1.55	Lower measured value with hydroxyproline; 95% CI included 0 (imprecise estimate)	Not applicable - effect size estimated after converting SEM to SD
Shi et al. (2024)	Pacific white shrimp (Litopenaeus vannamei)	Hydroxyproline supplementation in a 10% fishmeal diet	LF8 (10% fishmeal; 0.8% hydroxyproline)	LF0 (10% fishmeal; 0% hydroxyproline)	Meat yield (%)	40.750 ± 0.386	42.990 ± 0.748	3	3	Hedges' g	-3.01	-5.64 to -0.38	Lower, less favorable value with hydroxyproline; 95% CI excluded 0	Not applicable - effect size estimated after converting SEM to SD
Shi et al. (2024)	Pacific white shrimp (Litopenaeus vannamei)	Hydroxyproline supplementation in a 10% fishmeal diet	LF8 (10% fishmeal; 0.8% hydroxyproline)	LF0 (10% fishmeal; 0% hydroxyproline)	Hardness (N)	768.930 ± 60.781	791.160 ± 26.618	3	3	Hedges' g	-0.38	-2.00 to 1.24	Lower, less favorable value with hydroxyproline; 95% CI included 0 (imprecise estimate)	Not applicable - effect size estimated after converting SEM to SD
Shi et al. (2024)	Pacific white shrimp (Litopenaeus vannamei)	Hydroxyproline supplementation in a 10% fishmeal diet	LF8 (10% fishmeal; 0.8% hydroxyproline)	LF0 (10% fishmeal; 0% hydroxyproline)	Springiness (mm)	0.630 ± 0.026	0.540 ± 0.029	3	3	Hedges' g	2.59	0.19 to 5.00	Higher, more favorable value with hydroxyproline; 95% CI excluded 0	Not applicable - effect size estimated after converting SEM to SD
Shi et al. (2024)	Pacific white shrimp (Litopenaeus vannamei)	Hydroxyproline supplementation in a 10% fishmeal diet	LF8 (10% fishmeal; 0.8% hydroxyproline)	LF0 (10% fishmeal; 0% hydroxyproline)	Chewiness (mJ)	352.210 ± 29.476	244.310 ± 23.485	3	3	Hedges' g	3.24	0.48 to 6.00	Higher, more favorable value with hydroxyproline; 95% CI excluded 0	Not applicable - effect size estimated after converting SEM to SD
Shi et al. (2024)	Pacific white shrimp (Litopenaeus vannamei)	Hydroxyproline supplementation in a 10% fishmeal diet	LF8 (10% fishmeal; 0.8% hydroxyproline)	LF0 (10% fishmeal; 0% hydroxyproline)	Cohesiveness (N)	0.530 ± 0.019	0.460 ± 0.023	3	3	Hedges' g	2.68	0.23 to 5.14	Higher, more favorable value with hydroxyproline; 95% CI excluded 0	Not applicable - effect size estimated after converting SEM to SD
Shi et al. (2024)	Pacific white shrimp (Litopenaeus vannamei)	Hydroxyproline supplementation in a 10% fishmeal diet	LF8 (10% fishmeal; 0.8% hydroxyproline)	LF0 (10% fishmeal; 0% hydroxyproline)	Cooking loss (%)	8.560 ± 0.743	11.660 ± 1.252	3	3	Hedges' g (direction reversed)	2.41	0.10 to 4.72	Lower, more favorable value with hydroxyproline; 95% CI excluded 0	Not applicable - effect size estimated after converting SEM to SD
Shi et al. (2024)	Pacific white shrimp (Litopenaeus vannamei)	Hydroxyproline supplementation in a 10% fishmeal diet	LF8 (10% fishmeal; 0.8% hydroxyproline)	LF0 (10% fishmeal; 0% hydroxyproline)	Freezing loss (%)	1.220 ± 0.236	1.270 ± 0.010	3	3	Hedges' g (direction reversed)	0.24	-1.37 to 1.85	Lower, more favorable value with hydroxyproline; 95% CI included 0 (imprecise estimate)	Not applicable - effect size estimated after converting SEM to SD
Shi et al. (2024)	Pacific white shrimp (Litopenaeus vannamei)	Hydroxyproline supplementation in a 10% fishmeal diet	LF2 (10% fishmeal; 0.2% hydroxyproline)	LF0 (10% fishmeal; 0% hydroxyproline)	Muscle moisture	Not available	Not available	3	3	Not estimable	Not estimable	Not estimable	The article reported no significant difference, but exact group data were not available in the uploaded PDF	Values were reported in Supplementary Table S4, which was not included in the uploaded file
Shi et al. (2024)	Pacific white shrimp (Litopenaeus vannamei)	Hydroxyproline supplementation in a 10% fishmeal diet	LF2 (10% fishmeal; 0.2% hydroxyproline)	LF0 (10% fishmeal; 0% hydroxyproline)	Muscle crude protein	Not available	Not available	3	3	Not estimable	Not estimable	Not estimable	The article reported no significant difference, but exact group data were not available in the uploaded PDF	Values were reported in Supplementary Table S4, which was not included in the uploaded file
Shi et al. (2024)	Pacific white shrimp (Litopenaeus vannamei)	Hydroxyproline supplementation in a 10% fishmeal diet	LF2 (10% fishmeal; 0.2% hydroxyproline)	LF0 (10% fishmeal; 0% hydroxyproline)	Muscle crude lipid	Not available	Not available	3	3	Not estimable	Not estimable	Not estimable	The article reported no significant difference, but exact group data were not available in the uploaded PDF	Values were reported in Supplementary Table S4, which was not included in the uploaded file
Shi et al. (2024)	Pacific white shrimp (Litopenaeus vannamei)	Hydroxyproline supplementation in a 10% fishmeal diet	LF4 (10% fishmeal; 0.4% hydroxyproline)	LF0 (10% fishmeal; 0% hydroxyproline)	Muscle moisture	Not available	Not available	3	3	Not estimable	Not estimable	Not estimable	The article reported no significant difference, but exact group data were not available in the uploaded PDF	Values were reported in Supplementary Table S4, which was not included in the uploaded file
Shi et al. (2024)	Pacific white shrimp (Litopenaeus vannamei)	Hydroxyproline supplementation in a 10% fishmeal diet	LF4 (10% fishmeal; 0.4% hydroxyproline)	LF0 (10% fishmeal; 0% hydroxyproline)	Muscle crude protein	Not available	Not available	3	3	Not estimable	Not estimable	Not estimable	The article reported no significant difference, but exact group data were not available in the uploaded PDF	Values were reported in Supplementary Table S4, which was not included in the uploaded file
Shi et al. (2024)	Pacific white shrimp (Litopenaeus vannamei)	Hydroxyproline supplementation in a 10% fishmeal diet	LF4 (10% fishmeal; 0.4% hydroxyproline)	LF0 (10% fishmeal; 0% hydroxyproline)	Muscle crude lipid	Not available	Not available	3	3	Not estimable	Not estimable	Not estimable	The article reported no significant difference, but exact group data were not available in the uploaded PDF	Values were reported in Supplementary Table S4, which was not included in the uploaded file
Shi et al. (2024)	Pacific white shrimp (Litopenaeus vannamei)	Hydroxyproline supplementation in a 10% fishmeal diet	LF6 (10% fishmeal; 0.6% hydroxyproline)	LF0 (10% fishmeal; 0% hydroxyproline)	Muscle moisture	Not available	Not available	3	3	Not estimable	Not estimable	Not estimable	The article reported no significant difference, but exact group data were not available in the uploaded PDF	Values were reported in Supplementary Table S4, which was not included in the uploaded file
Shi et al. (2024)	Pacific white shrimp (Litopenaeus vannamei)	Hydroxyproline supplementation in a 10% fishmeal diet	LF6 (10% fishmeal; 0.6% hydroxyproline)	LF0 (10% fishmeal; 0% hydroxyproline)	Muscle crude protein	Not available	Not available	3	3	Not estimable	Not estimable	Not estimable	The article reported no significant difference, but exact group data were not available in the uploaded PDF	Values were reported in Supplementary Table S4, which was not included in the uploaded file
Shi et al. (2024)	Pacific white shrimp (Litopenaeus vannamei)	Hydroxyproline supplementation in a 10% fishmeal diet	LF6 (10% fishmeal; 0.6% hydroxyproline)	LF0 (10% fishmeal; 0% hydroxyproline)	Muscle crude lipid	Not available	Not available	3	3	Not estimable	Not estimable	Not estimable	The article reported no significant difference, but exact group data were not available in the uploaded PDF	Values were reported in Supplementary Table S4, which was not included in the uploaded file

Study	Species	Strategy	Intervention	Comparator	Outcome and unit	Intervention mean ± SD	Comparator mean ± SD	n interventio n (tanks)	n comparato r (tanks)	Effect-size metric	Effect size	95% CI	Interpretation	Reason not estimated
Shi et al. (2024)	Pacific white shrimp (Litopenaeus vannamei)	Hydroxyproline supplementation in a 10% fishmeal diet	LF8 (10% fishmeal; 0.8% hydroxyproline)	LF0 (10% fishmeal; 0% hydroxyproline)	Muscle moisture	Not available	Not available	3	3	Not estimable	Not estimable	Not estimable	The article reported no significant difference, but exact group data were not available in the uploaded PDF	Values were reported in Supplementary Table S4, which was not included in the uploaded file
Shi et al. (2024)	Pacific white shrimp (Litopenaeus vannamei)	Hydroxyproline supplementation in a 10% fishmeal diet	LF8 (10% fishmeal; 0.8% hydroxyproline)	LF0 (10% fishmeal; 0% hydroxyproline)	Muscle crude protein	Not available	Not available	3	3	Not estimable	Not estimable	Not estimable	The article reported no significant difference, but exact group data were not available in the uploaded PDF	Values were reported in Supplementary Table S4, which was not included in the uploaded file
Shi et al. (2024)	Pacific white shrimp (Litopenaeus vannamei)	Hydroxyproline supplementation in a 10% fishmeal diet	LF8 (10% fishmeal; 0.8% hydroxyproline)	LF0 (10% fishmeal; 0% hydroxyproline)	Muscle crude lipid	Not available	Not available	3	3	Not estimable	Not estimable	Not estimable	The article reported no significant difference, but exact group data were not available in the uploaded PDF	Values were reported in Supplementary Table S4, which was not included in the uploaded file
Shi et al. (2024)	Pacific white shrimp (Litopenaeus vannamei)	Hydroxyproline supplementation in a 10% fishmeal diet	LF2 (10% fishmeal; 0.2% hydroxyproline)	LF0 (10% fishmeal; 0% hydroxyproline)	Hemolymph prolyl 4-hydroxylases content (Figure 1A)	Not reported numerically	Not reported numerically	3	3	Not estimable	Not estimable	Not estimable	The outcome was shown graphically with significance annotations; an exact effect size was not calculated	Exact group means and SEMs were presented only graphically and were not reliably extractable
Shi et al. (2024)	Pacific white shrimp (Litopenaeus vannamei)	Hydroxyproline supplementation in a 10% fishmeal diet	LF2 (10% fishmeal; 0.2% hydroxyproline)	LF0 (10% fishmeal; 0% hydroxyproline)	Hepatopancreas prolyl 4-hydroxylases content (Figure 1B)	Not reported numerically	Not reported numerically	3	3	Not estimable	Not estimable	Not estimable	The outcome was shown graphically with significance annotations; an exact effect size was not calculated	Exact group means and SEMs were presented only graphically and were not reliably extractable
Shi et al. (2024)	Pacific white shrimp (Litopenaeus vannamei)	Hydroxyproline supplementation in a 10% fishmeal diet	LF2 (10% fishmeal; 0.2% hydroxyproline)	LF0 (10% fishmeal; 0% hydroxyproline)	Muscle prolyl 4-hydroxylases content (Figure 1C)	Not reported numerically	Not reported numerically	3	3	Not estimable	Not estimable	Not estimable	The outcome was shown graphically with significance annotations; an exact effect size was not calculated	Exact group means and SEMs were presented only graphically and were not reliably extractable
Shi et al. (2024)	Pacific white shrimp (Litopenaeus vannamei)	Hydroxyproline supplementation in a 10% fishmeal diet	LF2 (10% fishmeal; 0.2% hydroxyproline)	LF0 (10% fishmeal; 0% hydroxyproline)	Hemolymph hydroxyproline content (Figure 1D)	Not reported numerically	Not reported numerically	3	3	Not estimable	Not estimable	Not estimable	The outcome was shown graphically with significance annotations; an exact effect size was not calculated	Exact group means and SEMs were presented only graphically and were not reliably extractable
Shi et al. (2024)	Pacific white shrimp (Litopenaeus vannamei)	Hydroxyproline supplementation in a 10% fishmeal diet	LF2 (10% fishmeal; 0.2% hydroxyproline)	LF0 (10% fishmeal; 0% hydroxyproline)	Hepatopancreas hydroxyproline content (Figure 1E)	Not reported numerically	Not reported numerically	3	3	Not estimable	Not estimable	Not estimable	The outcome was shown graphically with significance annotations; an exact effect size was not calculated	Exact group means and SEMs were presented only graphically and were not reliably extractable
Shi et al. (2024)	Pacific white shrimp (Litopenaeus vannamei)	Hydroxyproline supplementation in a 10% fishmeal diet	LF2 (10% fishmeal; 0.2% hydroxyproline)	LF0 (10% fishmeal; 0% hydroxyproline)	Muscle hydroxyproline content (Figure 1F)	Not reported numerically	Not reported numerically	3	3	Not estimable	Not estimable	Not estimable	The outcome was shown graphically with significance annotations; an exact effect size was not calculated	Exact group means and SEMs were presented only graphically and were not reliably extractable
Shi et al. (2024)	Pacific white shrimp (Litopenaeus vannamei)	Hydroxyproline supplementation in a 10% fishmeal diet	LF2 (10% fishmeal; 0.2% hydroxyproline)	LF0 (10% fishmeal; 0% hydroxyproline)	Hemolymph collagen content (Figure 1G)	Not reported numerically	Not reported numerically	3	3	Not estimable	Not estimable	Not estimable	The outcome was shown graphically with significance annotations; an exact effect size was not calculated	Exact group means and SEMs were presented only graphically and were not reliably extractable
Shi et al. (2024)	Pacific white shrimp (Litopenaeus vannamei)	Hydroxyproline supplementation in a 10% fishmeal diet	LF2 (10% fishmeal; 0.2% hydroxyproline)	LF0 (10% fishmeal; 0% hydroxyproline)	Hepatopancreas collagen content (Figure 1H)	Not reported numerically	Not reported numerically	3	3	Not estimable	Not estimable	Not estimable	The outcome was shown graphically with significance annotations; an exact effect size was not calculated	Exact group means and SEMs were presented only graphically and were not reliably extractable
Shi et al. (2024)	Pacific white shrimp (Litopenaeus vannamei)	Hydroxyproline supplementation in a 10% fishmeal diet	LF2 (10% fishmeal; 0.2% hydroxyproline)	LF0 (10% fishmeal; 0% hydroxyproline)	Muscle collagen content (Figure 1I)	Not reported numerically	Not reported numerically	3	3	Not estimable	Not estimable	Not estimable	The outcome was shown graphically with significance annotations; an exact effect size was not calculated	Exact group means and SEMs were presented only graphically and were not reliably extractable
Shi et al. (2024)	Pacific white shrimp (Litopenaeus vannamei)	Hydroxyproline supplementation in a 10% fishmeal diet	LF2 (10% fishmeal; 0.2% hydroxyproline)	LF0 (10% fishmeal; 0% hydroxyproline)	Muscle smyhc5 mRNA expression (Figure 2A)	Not reported numerically	Not reported numerically	3	3	Not estimable	Not estimable	Not estimable	The outcome was shown graphically with significance annotations; an exact effect size was not calculated	Exact group means and SEMs were presented only graphically and were not reliably extractable
Shi et al. (2024)	Pacific white shrimp (Litopenaeus vannamei)	Hydroxyproline supplementation in a 10% fishmeal diet	LF2 (10% fishmeal; 0.2% hydroxyproline)	LF0 (10% fishmeal; 0% hydroxyproline)	Muscle col1a1 mRNA expression (Figure 2A)	Not reported numerically	Not reported numerically	3	3	Not estimable	Not estimable	Not estimable	The outcome was shown graphically with significance annotations; an exact effect size was not calculated	Exact group means and SEMs were presented only graphically and were not reliably extractable
Shi et al. (2024)	Pacific white shrimp (Litopenaeus vannamei)	Hydroxyproline supplementation in a 10% fishmeal diet	LF2 (10% fishmeal; 0.2% hydroxyproline)	LF0 (10% fishmeal; 0% hydroxyproline)	Muscle col1a2 mRNA expression (Figure 2A)	Not reported numerically	Not reported numerically	3	3	Not estimable	Not estimable	Not estimable	The outcome was shown graphically with significance annotations; an exact effect size was not calculated	Exact group means and SEMs were presented only graphically and were not reliably extractable
Shi et al. (2024)	Pacific white shrimp (Litopenaeus vannamei)	Hydroxyproline supplementation in a 10% fishmeal diet	LF2 (10% fishmeal; 0.2% hydroxyproline)	LF0 (10% fishmeal; 0% hydroxyproline)	Muscle smyhc1 mRNA expression (Figure 2A)	Not reported numerically	Not reported numerically	3	3	Not estimable	Not estimable	Not estimable	The outcome was shown graphically with significance annotations; an exact effect size was not calculated	Exact group means and SEMs were presented only graphically and were not reliably extractable
Shi et al. (2024)	Pacific white shrimp (Litopenaeus vannamei)	Hydroxyproline supplementation in a 10% fishmeal diet	LF2 (10% fishmeal; 0.2% hydroxyproline)	LF0 (10% fishmeal; 0% hydroxyproline)	Muscle smyhc2 mRNA expression (Figure 2A)	Not reported numerically	Not reported numerically	3	3	Not estimable	Not estimable	Not estimable	The outcome was shown graphically with significance annotations; an exact effect size was not calculated	Exact group means and SEMs were presented only graphically and were not reliably extractable
Shi et al. (2024)	Pacific white shrimp (Litopenaeus vannamei)	Hydroxyproline supplementation in a 10% fishmeal diet	LF2 (10% fishmeal; 0.2% hydroxyproline)	LF0 (10% fishmeal; 0% hydroxyproline)	Muscle smyhc6a mRNA expression (Figure 2A)	Not reported numerically	Not reported numerically	3	3	Not estimable	Not estimable	Not estimable	The outcome was shown graphically with significance annotations; an exact effect size was not calculated	Exact group means and SEMs were presented only graphically and were not reliably extractable
Shi et al. (2024)	Pacific white shrimp (Litopenaeus vannamei)	Hydroxyproline supplementation in a 10% fishmeal diet	LF2 (10% fishmeal; 0.2% hydroxyproline)	LF0 (10% fishmeal; 0% hydroxyproline)	Muscle smyhc15 mRNA expression (Figure 2A)	Not reported numerically	Not reported numerically	3	3	Not estimable	Not estimable	Not estimable	The outcome was shown graphically with significance annotations; an exact effect size was not calculated	Exact group means and SEMs were presented only graphically and were not reliably extractable
Shi et al. (2024)	Pacific white shrimp (Litopenaeus vannamei)	Hydroxyproline supplementation in a 10% fishmeal diet	LF2 (10% fishmeal; 0.2% hydroxyproline)	LF0 (10% fishmeal; 0% hydroxyproline)	Muscle igf-1 mRNA expression (Figure 2B)	Not reported numerically	Not reported numerically	3	3	Not estimable	Not estimable	Not estimable	The outcome was shown graphically with significance annotations; an exact effect size was not calculated	Exact group means and SEMs were presented only graphically and were not reliably extractable
Shi et al. (2024)	Pacific white shrimp (Litopenaeus vannamei)	Hydroxyproline supplementation in a 10% fishmeal diet	LF2 (10% fishmeal; 0.2% hydroxyproline)	LF0 (10% fishmeal; 0% hydroxyproline)	Muscle tgf-b mRNA expression (Figure 2B)	Not reported numerically	Not reported numerically	3	3	Not estimable	Not estimable	Not estimable	The outcome was shown graphically with significance annotations; an exact effect size was not calculated	Exact group means and SEMs were presented only graphically and were not reliably extractable
Shi et al. (2024)	Pacific white shrimp (Litopenaeus vannamei)	Hydroxyproline supplementation in a 10% fishmeal diet	LF2 (10% fishmeal; 0.2% hydroxyproline)	LF0 (10% fishmeal; 0% hydroxyproline)	Muscle tor mRNA expression (Figure 2B)	Not reported numerically	Not reported numerically	3	3	Not estimable	Not estimable	Not estimable	The outcome was shown graphically with significance annotations; an exact effect size was not calculated	Exact group means and SEMs were presented only graphically and were not reliably extractable
Shi et al. (2024)	Pacific white shrimp (Litopenaeus vannamei)	Hydroxyproline supplementation in a 10% fishmeal diet	LF2 (10% fishmeal; 0.2% hydroxyproline)	LF0 (10% fishmeal; 0% hydroxyproline)	Hepatopancreas col1a1 mRNA expression (Figure 2C)	Not reported numerically	Not reported numerically	3	3	Not estimable	Not estimable	Not estimable	The outcome was shown graphically with significance annotations; an exact effect size was not calculated	Exact group means and SEMs were presented only graphically and were not reliably extractable
Shi et al. (2024)	Pacific white shrimp (Litopenaeus vannamei)	Hydroxyproline supplementation in a 10% fishmeal diet	LF2 (10% fishmeal; 0.2% hydroxyproline)	LF0 (10% fishmeal; 0% hydroxyproline)	Hepatopancreas col1a2 mRNA expression (Figure 2C)	Not reported numerically	Not reported numerically	3	3	Not estimable	Not estimable	Not estimable	The outcome was shown graphically with significance annotations; an exact effect size was not calculated	Exact group means and SEMs were presented only graphically and were not reliably extractable
Shi et al. (2024)	Pacific white shrimp (Litopenaeus vannamei)	Hydroxyproline supplementation in a 10% fishmeal diet	LF2 (10% fishmeal; 0.2% hydroxyproline)	LF0 (10% fishmeal; 0% hydroxyproline)	Hepatopancreas smyhc5 mRNA expression (Figure 2C)	Not reported numerically	Not reported numerically	3	3	Not estimable	Not estimable	Not estimable	The outcome was shown graphically with significance annotations; an exact effect size was not calculated	Exact group means and SEMs were presented only graphically and were not reliably extractable
Shi et al. (2024)	Pacific white shrimp (Litopenaeus vannamei)	Hydroxyproline supplementation in a 10% fishmeal diet	LF2 (10% fishmeal; 0.2% hydroxyproline)	LF0 (10% fishmeal; 0% hydroxyproline)	Hepatopancreas smyhc6a mRNA expression (Figure 2C)	Not reported numerically	Not reported numerically	3	3	Not estimable	Not estimable	Not estimable	The outcome was shown graphically with significance annotations; an exact effect size was not calculated	Exact group means and SEMs were presented only graphically and were not reliably extractable
Shi et al. (2024)	Pacific white shrimp (Litopenaeus vannamei)	Hydroxyproline supplementation in a 10% fishmeal diet	LF2 (10% fishmeal; 0.2% hydroxyproline)	LF0 (10% fishmeal; 0% hydroxyproline)	Hepatopancreas tgf-b mRNA expression (Figure 2D)	Not reported numerically	Not reported numerically	3	3	Not estimable	Not estimable	Not estimable	The outcome was shown graphically with significance annotations; an exact effect size was not calculated	Exact group means and SEMs were presented only graphically and were not reliably extractable
Shi et al. (2024)	Pacific white shrimp (Litopenaeus vannamei)	Hydroxyproline supplementation in a 10% fishmeal diet	LF2 (10% fishmeal; 0.2% hydroxyproline)	LF0 (10% fishmeal; 0% hydroxyproline)	Hepatopancreas tor mRNA expression (Figure 2D)	Not reported numerically	Not reported numerically	3	3	Not estimable	Not estimable	Not estimable	The outcome was shown graphically with significance annotations; an exact effect size was not calculated	Exact group means and SEMs were presented only graphically and were not reliably extractable
Shi et al. (2024)	Pacific white shrimp (Litopenaeus vannamei)	Hydroxyproline supplementation in a 10% fishmeal diet	LF2 (10% fishmeal; 0.2% hydroxyproline)	LF0 (10% fishmeal; 0% hydroxyproline)	Hepatopancreas igf-1 mRNA expression (Figure 2D)	Not reported numerically	Not reported numerically	3	3	Not estimable	Not estimable	Not estimable	The outcome was shown graphically with significance annotations; an exact effect size was not calculated	Exact group means and SEMs were presented only graphically and were not reliably extractable
Shi et al. (2024)	Pacific white shrimp (Litopenaeus vannamei)	Hydroxyproline supplementation in a 10% fishmeal diet	LF2 (10% fishmeal; 0.2% hydroxyproline)	LF0 (10% fishmeal; 0% hydroxyproline)	Myofiber diameter (Figure 3C)	Not reported numerically	Not reported numerically	3	3	Not estimable	Not estimable	Not estimable	The outcome was shown graphically with significance annotations; an exact effect size was not calculated	Exact group means and SEMs were presented only graphically and were not reliably extractable
Shi et al. (2024)	Pacific white shrimp (Litopenaeus vannamei)	Hydroxyproline supplementation in a 10% fishmeal diet	LF2 (10% fishmeal; 0.2% hydroxyproline)	LF0 (10% fishmeal; 0% hydroxyproline)	Sarcomere diameter (Figure 3C)	Not reported numerically	Not reported numerically	3	3	Not estimable	Not estimable	Not estimable	Not reported for this intervention group	This outcome was not reported for the selected hydroxyproline dose; the figure included LF0 and LF4 only

Study	Species	Strategy	Intervention	Comparator	Outcome and unit	Intervention mean ± SD	Comparator mean ± SD	n intervention (tanks)	n comparator (tanks)	Effect-size metric	Effect size	95% CI	Interpretation	Reason not estimated
Shi et al. (2024)	Pacific white shrimp (Litopenaeus vannamei)	Hydroxyproline supplementation in a 10% fishmeal diet	LF2 (10% fishmeal; 0.2% hydroxyproline)	LF0 (10% fishmeal; 0% hydroxyproline)	P-4E-BP1 protein expression (Figure 4B)	Not reported numerically	Not reported numerically	3	3	Not estimable	Not estimable	Not estimable	Not reported for this intervention group	This outcome was not reported for the selected hydroxyproline dose; the figure included LF0 and LF4 only
Shi et al. (2024)	Pacific white shrimp (Litopenaeus vannamei)	Hydroxyproline supplementation in a 10% fishmeal diet	LF2 (10% fishmeal; 0.2% hydroxyproline)	LF0 (10% fishmeal; 0% hydroxyproline)	4E-BP1 protein expression (Figure 4C)	Not reported numerically	Not reported numerically	3	3	Not estimable	Not estimable	Not estimable	Not reported for this intervention group	This outcome was not reported for the selected hydroxyproline dose; the figure included LF0 and LF4 only
Shi et al. (2024)	Pacific white shrimp (Litopenaeus vannamei)	Hydroxyproline supplementation in a 10% fishmeal diet	LF2 (10% fishmeal; 0.2% hydroxyproline)	LF0 (10% fishmeal; 0% hydroxyproline)	P-AKT protein expression (Figure 4D)	Not reported numerically	Not reported numerically	3	3	Not estimable	Not estimable	Not estimable	Not reported for this intervention group	This outcome was not reported for the selected hydroxyproline dose; the figure included LF0 and LF4 only
Shi et al. (2024)	Pacific white shrimp (Litopenaeus vannamei)	Hydroxyproline supplementation in a 10% fishmeal diet	LF2 (10% fishmeal; 0.2% hydroxyproline)	LF0 (10% fishmeal; 0% hydroxyproline)	AKT protein expression (Figure 4E)	Not reported numerically	Not reported numerically	3	3	Not estimable	Not estimable	Not estimable	Not reported for this intervention group	This outcome was not reported for the selected hydroxyproline dose; the figure included LF0 and LF4 only
Shi et al. (2024)	Pacific white shrimp (Litopenaeus vannamei)	Hydroxyproline supplementation in a 10% fishmeal diet	LF2 (10% fishmeal; 0.2% hydroxyproline)	LF0 (10% fishmeal; 0% hydroxyproline)	P-4E-BP1/4E-BP1 ratio (Figure 4F)	Not reported numerically	Not reported numerically	3	3	Not estimable	Not estimable	Not estimable	Not reported for this intervention group	This outcome was not reported for the selected hydroxyproline dose; the figure included LF0 and LF4 only
Shi et al. (2024)	Pacific white shrimp (Litopenaeus vannamei)	Hydroxyproline supplementation in a 10% fishmeal diet	LF2 (10% fishmeal; 0.2% hydroxyproline)	LF0 (10% fishmeal; 0% hydroxyproline)	P-AKT/AKT ratio (Figure 4G)	Not reported numerically	Not reported numerically	3	3	Not estimable	Not estimable	Not estimable	Not reported for this intervention group	This outcome was not reported for the selected hydroxyproline dose; the figure included LF0 and LF4 only
Shi et al. (2024)	Pacific white shrimp (Litopenaeus vannamei)	Hydroxyproline supplementation in a 10% fishmeal diet	LF4 (10% fishmeal; 0.4% hydroxyproline)	LF0 (10% fishmeal; 0% hydroxyproline)	Hemolymph prolyl 4-hydroxylases content (Figure 1A)	Not reported numerically	Not reported numerically	3	3	Not estimable	Not estimable	Not estimable	The outcome was shown graphically with significance annotations; an exact effect size was not calculated	Exact group means and SEMs were presented only graphically and were not reliably extractable
Shi et al. (2024)	Pacific white shrimp (Litopenaeus vannamei)	Hydroxyproline supplementation in a 10% fishmeal diet	LF4 (10% fishmeal; 0.4% hydroxyproline)	LF0 (10% fishmeal; 0% hydroxyproline)	Hepatopancreas prolyl 4-hydroxylases content (Figure 1B)	Not reported numerically	Not reported numerically	3	3	Not estimable	Not estimable	Not estimable	The outcome was shown graphically with significance annotations; an exact effect size was not calculated	Exact group means and SEMs were presented only graphically and were not reliably extractable
Shi et al. (2024)	Pacific white shrimp (Litopenaeus vannamei)	Hydroxyproline supplementation in a 10% fishmeal diet	LF4 (10% fishmeal; 0.4% hydroxyproline)	LF0 (10% fishmeal; 0% hydroxyproline)	Muscle prolyl 4-hydroxylases content (Figure 1C)	Not reported numerically	Not reported numerically	3	3	Not estimable	Not estimable	Not estimable	The outcome was shown graphically with significance annotations; an exact effect size was not calculated	Exact group means and SEMs were presented only graphically and were not reliably extractable
Shi et al. (2024)	Pacific white shrimp (Litopenaeus vannamei)	Hydroxyproline supplementation in a 10% fishmeal diet	LF4 (10% fishmeal; 0.4% hydroxyproline)	LF0 (10% fishmeal; 0% hydroxyproline)	Hemolymph hydroxyproline content (Figure 1D)	Not reported numerically	Not reported numerically	3	3	Not estimable	Not estimable	Not estimable	The outcome was shown graphically with significance annotations; an exact effect size was not calculated	Exact group means and SEMs were presented only graphically and were not reliably extractable
Shi et al. (2024)	Pacific white shrimp (Litopenaeus vannamei)	Hydroxyproline supplementation in a 10% fishmeal diet	LF4 (10% fishmeal; 0.4% hydroxyproline)	LF0 (10% fishmeal; 0% hydroxyproline)	Hepatopancreas hydroxyproline content (Figure 1E)	Not reported numerically	Not reported numerically	3	3	Not estimable	Not estimable	Not estimable	The outcome was shown graphically with significance annotations; an exact effect size was not calculated	Exact group means and SEMs were presented only graphically and were not reliably extractable
Shi et al. (2024)	Pacific white shrimp (Litopenaeus vannamei)	Hydroxyproline supplementation in a 10% fishmeal diet	LF4 (10% fishmeal; 0.4% hydroxyproline)	LF0 (10% fishmeal; 0% hydroxyproline)	Muscle hydroxyproline content (Figure 1F)	Not reported numerically	Not reported numerically	3	3	Not estimable	Not estimable	Not estimable	The outcome was shown graphically with significance annotations; an exact effect size was not calculated	Exact group means and SEMs were presented only graphically and were not reliably extractable
Shi et al. (2024)	Pacific white shrimp (Litopenaeus vannamei)	Hydroxyproline supplementation in a 10% fishmeal diet	LF4 (10% fishmeal; 0.4% hydroxyproline)	LF0 (10% fishmeal; 0% hydroxyproline)	Hemolymph collagen content (Figure 1G)	Not reported numerically	Not reported numerically	3	3	Not estimable	Not estimable	Not estimable	The outcome was shown graphically with significance annotations; an exact effect size was not calculated	Exact group means and SEMs were presented only graphically and were not reliably extractable
Shi et al. (2024)	Pacific white shrimp (Litopenaeus vannamei)	Hydroxyproline supplementation in a 10% fishmeal diet	LF4 (10% fishmeal; 0.4% hydroxyproline)	LF0 (10% fishmeal; 0% hydroxyproline)	Hepatopancreas collagen content (Figure 1H)	Not reported numerically	Not reported numerically	3	3	Not estimable	Not estimable	Not estimable	The outcome was shown graphically with significance annotations; an exact effect size was not calculated	Exact group means and SEMs were presented only graphically and were not reliably extractable
Shi et al. (2024)	Pacific white shrimp (Litopenaeus vannamei)	Hydroxyproline supplementation in a 10% fishmeal diet	LF4 (10% fishmeal; 0.4% hydroxyproline)	LF0 (10% fishmeal; 0% hydroxyproline)	Muscle collagen content (Figure 1I)	Not reported numerically	Not reported numerically	3	3	Not estimable	Not estimable	Not estimable	The outcome was shown graphically with significance annotations; an exact effect size was not calculated	Exact group means and SEMs were presented only graphically and were not reliably extractable
Shi et al. (2024)	Pacific white shrimp (Litopenaeus vannamei)	Hydroxyproline supplementation in a 10% fishmeal diet	LF4 (10% fishmeal; 0.4% hydroxyproline)	LF0 (10% fishmeal; 0% hydroxyproline)	Muscle smyhc5 mRNA expression (Figure 2A)	Not reported numerically	Not reported numerically	3	3	Not estimable	Not estimable	Not estimable	The outcome was shown graphically with significance annotations; an exact effect size was not calculated	Exact group means and SEMs were presented only graphically and were not reliably extractable
Shi et al. (2024)	Pacific white shrimp (Litopenaeus vannamei)	Hydroxyproline supplementation in a 10% fishmeal diet	LF4 (10% fishmeal; 0.4% hydroxyproline)	LF0 (10% fishmeal; 0% hydroxyproline)	Muscle col1a1 mRNA expression (Figure 2A)	Not reported numerically	Not reported numerically	3	3	Not estimable	Not estimable	Not estimable	The outcome was shown graphically with significance annotations; an exact effect size was not calculated	Exact group means and SEMs were presented only graphically and were not reliably extractable
Shi et al. (2024)	Pacific white shrimp (Litopenaeus vannamei)	Hydroxyproline supplementation in a 10% fishmeal diet	LF4 (10% fishmeal; 0.4% hydroxyproline)	LF0 (10% fishmeal; 0% hydroxyproline)	Muscle col1a2 mRNA expression (Figure 2A)	Not reported numerically	Not reported numerically	3	3	Not estimable	Not estimable	Not estimable	The outcome was shown graphically with significance annotations; an exact effect size was not calculated	Exact group means and SEMs were presented only graphically and were not reliably extractable
Shi et al. (2024)	Pacific white shrimp (Litopenaeus vannamei)	Hydroxyproline supplementation in a 10% fishmeal diet	LF4 (10% fishmeal; 0.4% hydroxyproline)	LF0 (10% fishmeal; 0% hydroxyproline)	Muscle smyhc1 mRNA expression (Figure 2A)	Not reported numerically	Not reported numerically	3	3	Not estimable	Not estimable	Not estimable	The outcome was shown graphically with significance annotations; an exact effect size was not calculated	Exact group means and SEMs were presented only graphically and were not reliably extractable
Shi et al. (2024)	Pacific white shrimp (Litopenaeus vannamei)	Hydroxyproline supplementation in a 10% fishmeal diet	LF4 (10% fishmeal; 0.4% hydroxyproline)	LF0 (10% fishmeal; 0% hydroxyproline)	Muscle smyhc2 mRNA expression (Figure 2A)	Not reported numerically	Not reported numerically	3	3	Not estimable	Not estimable	Not estimable	The outcome was shown graphically with significance annotations; an exact effect size was not calculated	Exact group means and SEMs were presented only graphically and were not reliably extractable
Shi et al. (2024)	Pacific white shrimp (Litopenaeus vannamei)	Hydroxyproline supplementation in a 10% fishmeal diet	LF4 (10% fishmeal; 0.4% hydroxyproline)	LF0 (10% fishmeal; 0% hydroxyproline)	Muscle smyhc6a mRNA expression (Figure 2A)	Not reported numerically	Not reported numerically	3	3	Not estimable	Not estimable	Not estimable	The outcome was shown graphically with significance annotations; an exact effect size was not calculated	Exact group means and SEMs were presented only graphically and were not reliably extractable
Shi et al. (2024)	Pacific white shrimp (Litopenaeus vannamei)	Hydroxyproline supplementation in a 10% fishmeal diet	LF4 (10% fishmeal; 0.4% hydroxyproline)	LF0 (10% fishmeal; 0% hydroxyproline)	Muscle smyhc15 mRNA expression (Figure 2A)	Not reported numerically	Not reported numerically	3	3	Not estimable	Not estimable	Not estimable	The outcome was shown graphically with significance annotations; an exact effect size was not calculated	Exact group means and SEMs were presented only graphically and were not reliably extractable
Shi et al. (2024)	Pacific white shrimp (Litopenaeus vannamei)	Hydroxyproline supplementation in a 10% fishmeal diet	LF4 (10% fishmeal; 0.4% hydroxyproline)	LF0 (10% fishmeal; 0% hydroxyproline)	Muscle igf-1 mRNA expression (Figure 2B)	Not reported numerically	Not reported numerically	3	3	Not estimable	Not estimable	Not estimable	The outcome was shown graphically with significance annotations; an exact effect size was not calculated	Exact group means and SEMs were presented only graphically and were not reliably extractable
Shi et al. (2024)	Pacific white shrimp (Litopenaeus vannamei)	Hydroxyproline supplementation in a 10% fishmeal diet	LF4 (10% fishmeal; 0.4% hydroxyproline)	LF0 (10% fishmeal; 0% hydroxyproline)	Muscle tgf-b mRNA expression (Figure 2B)	Not reported numerically	Not reported numerically	3	3	Not estimable	Not estimable	Not estimable	The outcome was shown graphically with significance annotations; an exact effect size was not calculated	Exact group means and SEMs were presented only graphically and were not reliably extractable
Shi et al. (2024)	Pacific white shrimp (Litopenaeus vannamei)	Hydroxyproline supplementation in a 10% fishmeal diet	LF4 (10% fishmeal; 0.4% hydroxyproline)	LF0 (10% fishmeal; 0% hydroxyproline)	Muscle tor mRNA expression (Figure 2B)	Not reported numerically	Not reported numerically	3	3	Not estimable	Not estimable	Not estimable	The outcome was shown graphically with significance annotations; an exact effect size was not calculated	Exact group means and SEMs were presented only graphically and were not reliably extractable
Shi et al. (2024)	Pacific white shrimp (Litopenaeus vannamei)	Hydroxyproline supplementation in a 10% fishmeal diet	LF4 (10% fishmeal; 0.4% hydroxyproline)	LF0 (10% fishmeal; 0% hydroxyproline)	Hepatopancreas col1a1 mRNA expression (Figure 2C)	Not reported numerically	Not reported numerically	3	3	Not estimable	Not estimable	Not estimable	The outcome was shown graphically with significance annotations; an exact effect size was not calculated	Exact group means and SEMs were presented only graphically and were not reliably extractable
Shi et al. (2024)	Pacific white shrimp (Litopenaeus vannamei)	Hydroxyproline supplementation in a 10% fishmeal diet	LF4 (10% fishmeal; 0.4% hydroxyproline)	LF0 (10% fishmeal; 0% hydroxyproline)	Hepatopancreas col1a2 mRNA expression (Figure 2C)	Not reported numerically	Not reported numerically	3	3	Not estimable	Not estimable	Not estimable	The outcome was shown graphically with significance annotations; an exact effect size was not calculated	Exact group means and SEMs were presented only graphically and were not reliably extractable
Shi et al. (2024)	Pacific white shrimp (Litopenaeus vannamei)	Hydroxyproline supplementation in a 10% fishmeal diet	LF4 (10% fishmeal; 0.4% hydroxyproline)	LF0 (10% fishmeal; 0% hydroxyproline)	Hepatopancreas smyhc5 mRNA expression (Figure 2C)	Not reported numerically	Not reported numerically	3	3	Not estimable	Not estimable	Not estimable	The outcome was shown graphically with significance annotations; an exact effect size was not calculated	Exact group means and SEMs were presented only graphically and were not reliably extractable
Shi et al. (2024)	Pacific white shrimp (Litopenaeus vannamei)	Hydroxyproline supplementation in a 10% fishmeal diet	LF4 (10% fishmeal; 0.4% hydroxyproline)	LF0 (10% fishmeal; 0% hydroxyproline)	Hepatopancreas smyhc6a mRNA expression (Figure 2C)	Not reported numerically	Not reported numerically	3	3	Not estimable	Not estimable	Not estimable	The outcome was shown graphically with significance annotations; an exact effect size was not calculated	Exact group means and SEMs were presented only graphically and were not reliably extractable
Shi et al. (2024)	Pacific white shrimp (Litopenaeus vannamei)	Hydroxyproline supplementation in a 10% fishmeal diet	LF4 (10% fishmeal; 0.4% hydroxyproline)	LF0 (10% fishmeal; 0% hydroxyproline)	Hepatopancreas tgf-b mRNA expression (Figure 2D)	Not reported numerically	Not reported numerically	3	3	Not estimable	Not estimable	Not estimable	The outcome was shown graphically with significance annotations; an exact effect size was not calculated	Exact group means and SEMs were presented only graphically and were not reliably extractable
Shi et al. (2024)	Pacific white shrimp (Litopenaeus vannamei)	Hydroxyproline supplementation in a 10% fishmeal diet	LF4 (10% fishmeal; 0.4% hydroxyproline)	LF0 (10% fishmeal; 0% hydroxyproline)	Hepatopancreas tor mRNA expression (Figure 2D)	Not reported numerically	Not reported numerically	3	3	Not estimable	Not estimable	Not estimable	The outcome was shown graphically with significance annotations; an exact effect size was not calculated	Exact group means and SEMs were presented only graphically and were not reliably extractable

Study	Species	Strategy	Intervention	Comparator	Outcome and unit	Intervention mean ± SD	Comparator mean ± SD	n intervention (tanks)	n comparator (tanks)	Effect-size metric	Effect size	95% CI	Interpretation	Reason not estimated
Shi et al. (2024)	Pacific white shrimp (Litopenaeus vannamei)	Hydroxyproline supplementation in a 10% fishmeal diet	LF4 (10% fishmeal; 0.4% hydroxyproline)	LF0 (10% fishmeal; 0% hydroxyproline)	Hepatopancreas igf-1 mRNA expression (Figure 2D)	Not reported numerically	Not reported numerically	3	3	Not estimable	Not estimable	Not estimable	The outcome was shown graphically with significance annotations; an exact effect size was not calculated	Exact group means and SEMs were presented only graphically and were not reliably extractable
Shi et al. (2024)	Pacific white shrimp (Litopenaeus vannamei)	Hydroxyproline supplementation in a 10% fishmeal diet	LF4 (10% fishmeal; 0.4% hydroxyproline)	LF0 (10% fishmeal; 0% hydroxyproline)	Myofiber diameter (Figure 3C)	Not reported numerically	Not reported numerically	3	3	Not estimable	Not estimable	Not estimable	The outcome was shown graphically with significance annotations; an exact effect size was not calculated	Exact group means and SEMs were presented only graphically and were not reliably extractable
Shi et al. (2024)	Pacific white shrimp (Litopenaeus vannamei)	Hydroxyproline supplementation in a 10% fishmeal diet	LF4 (10% fishmeal; 0.4% hydroxyproline)	LF0 (10% fishmeal; 0% hydroxyproline)	Sarcomere diameter (Figure 3C)	Not reported numerically	Not reported numerically	3	3	Not estimable	Not estimable	Not estimable	The outcome was shown graphically with significance annotations; an exact effect size was not calculated	Exact group means and SEMs were presented only graphically and were not reliably extractable
Shi et al. (2024)	Pacific white shrimp (Litopenaeus vannamei)	Hydroxyproline supplementation in a 10% fishmeal diet	LF4 (10% fishmeal; 0.4% hydroxyproline)	LF0 (10% fishmeal; 0% hydroxyproline)	P-4E-BP1 protein expression (Figure 4B)	Not reported numerically	Not reported numerically	3	3	Not estimable	Not estimable	Not estimable	The outcome was shown graphically with significance annotations; an exact effect size was not calculated	Exact group means and SEMs were presented only graphically and were not reliably extractable
Shi et al. (2024)	Pacific white shrimp (Litopenaeus vannamei)	Hydroxyproline supplementation in a 10% fishmeal diet	LF4 (10% fishmeal; 0.4% hydroxyproline)	LF0 (10% fishmeal; 0% hydroxyproline)	4E-BP1 protein expression (Figure 4C)	Not reported numerically	Not reported numerically	3	3	Not estimable	Not estimable	Not estimable	The outcome was shown graphically with significance annotations; an exact effect size was not calculated	Exact group means and SEMs were presented only graphically and were not reliably extractable
Shi et al. (2024)	Pacific white shrimp (Litopenaeus vannamei)	Hydroxyproline supplementation in a 10% fishmeal diet	LF4 (10% fishmeal; 0.4% hydroxyproline)	LF0 (10% fishmeal; 0% hydroxyproline)	P-AKT protein expression (Figure 4D)	Not reported numerically	Not reported numerically	3	3	Not estimable	Not estimable	Not estimable	The outcome was shown graphically with significance annotations; an exact effect size was not calculated	Exact group means and SEMs were presented only graphically and were not reliably extractable
Shi et al. (2024)	Pacific white shrimp (Litopenaeus vannamei)	Hydroxyproline supplementation in a 10% fishmeal diet	LF4 (10% fishmeal; 0.4% hydroxyproline)	LF0 (10% fishmeal; 0% hydroxyproline)	AKT protein expression (Figure 4E)	Not reported numerically	Not reported numerically	3	3	Not estimable	Not estimable	Not estimable	The outcome was shown graphically with significance annotations; an exact effect size was not calculated	Exact group means and SEMs were presented only graphically and were not reliably extractable
Shi et al. (2024)	Pacific white shrimp (Litopenaeus vannamei)	Hydroxyproline supplementation in a 10% fishmeal diet	LF4 (10% fishmeal; 0.4% hydroxyproline)	LF0 (10% fishmeal; 0% hydroxyproline)	P-4E-BP1/4E-BP1 ratio (Figure 4F)	Not reported numerically	Not reported numerically	3	3	Not estimable	Not estimable	Not estimable	The outcome was shown graphically with significance annotations; an exact effect size was not calculated	Exact group means and SEMs were presented only graphically and were not reliably extractable
Shi et al. (2024)	Pacific white shrimp (Litopenaeus vannamei)	Hydroxyproline supplementation in a 10% fishmeal diet	LF4 (10% fishmeal; 0.4% hydroxyproline)	LF0 (10% fishmeal; 0% hydroxyproline)	P-AKT/AKT ratio (Figure 4G)	Not reported numerically	Not reported numerically	3	3	Not estimable	Not estimable	Not estimable	The outcome was shown graphically with significance annotations; an exact effect size was not calculated	Exact group means and SEMs were presented only graphically and were not reliably extractable
Shi et al. (2024)	Pacific white shrimp (Litopenaeus vannamei)	Hydroxyproline supplementation in a 10% fishmeal diet	LF6 (10% fishmeal; 0.6% hydroxyproline)	LF0 (10% fishmeal; 0% hydroxyproline)	Hemolymph prolyl 4-hydroxylases content (Figure 1A)	Not reported numerically	Not reported numerically	3	3	Not estimable	Not estimable	Not estimable	The outcome was shown graphically with significance annotations; an exact effect size was not calculated	Exact group means and SEMs were presented only graphically and were not reliably extractable
Shi et al. (2024)	Pacific white shrimp (Litopenaeus vannamei)	Hydroxyproline supplementation in a 10% fishmeal diet	LF6 (10% fishmeal; 0.6% hydroxyproline)	LF0 (10% fishmeal; 0% hydroxyproline)	Hepatopancreas prolyl 4-hydroxylases content (Figure 1B)	Not reported numerically	Not reported numerically	3	3	Not estimable	Not estimable	Not estimable	The outcome was shown graphically with significance annotations; an exact effect size was not calculated	Exact group means and SEMs were presented only graphically and were not reliably extractable
Shi et al. (2024)	Pacific white shrimp (Litopenaeus vannamei)	Hydroxyproline supplementation in a 10% fishmeal diet	LF6 (10% fishmeal; 0.6% hydroxyproline)	LF0 (10% fishmeal; 0% hydroxyproline)	Muscle prolyl 4-hydroxylases content (Figure 1C)	Not reported numerically	Not reported numerically	3	3	Not estimable	Not estimable	Not estimable	The outcome was shown graphically with significance annotations; an exact effect size was not calculated	Exact group means and SEMs were presented only graphically and were not reliably extractable
Shi et al. (2024)	Pacific white shrimp (Litopenaeus vannamei)	Hydroxyproline supplementation in a 10% fishmeal diet	LF6 (10% fishmeal; 0.6% hydroxyproline)	LF0 (10% fishmeal; 0% hydroxyproline)	Hemolymph hydroxyproline content (Figure 1D)	Not reported numerically	Not reported numerically	3	3	Not estimable	Not estimable	Not estimable	The outcome was shown graphically with significance annotations; an exact effect size was not calculated	Exact group means and SEMs were presented only graphically and were not reliably extractable
Shi et al. (2024)	Pacific white shrimp (Litopenaeus vannamei)	Hydroxyproline supplementation in a 10% fishmeal diet	LF6 (10% fishmeal; 0.6% hydroxyproline)	LF0 (10% fishmeal; 0% hydroxyproline)	Hepatopancreas hydroxyproline content (Figure 1E)	Not reported numerically	Not reported numerically	3	3	Not estimable	Not estimable	Not estimable	The outcome was shown graphically with significance annotations; an exact effect size was not calculated	Exact group means and SEMs were presented only graphically and were not reliably extractable
Shi et al. (2024)	Pacific white shrimp (Litopenaeus vannamei)	Hydroxyproline supplementation in a 10% fishmeal diet	LF6 (10% fishmeal; 0.6% hydroxyproline)	LF0 (10% fishmeal; 0% hydroxyproline)	Muscle hydroxyproline content (Figure 1F)	Not reported numerically	Not reported numerically	3	3	Not estimable	Not estimable	Not estimable	The outcome was shown graphically with significance annotations; an exact effect size was not calculated	Exact group means and SEMs were presented only graphically and were not reliably extractable
Shi et al. (2024)	Pacific white shrimp (Litopenaeus vannamei)	Hydroxyproline supplementation in a 10% fishmeal diet	LF6 (10% fishmeal; 0.6% hydroxyproline)	LF0 (10% fishmeal; 0% hydroxyproline)	Hemolymph collagen content (Figure 1G)	Not reported numerically	Not reported numerically	3	3	Not estimable	Not estimable	Not estimable	The outcome was shown graphically with significance annotations; an exact effect size was not calculated	Exact group means and SEMs were presented only graphically and were not reliably extractable
Shi et al. (2024)	Pacific white shrimp (Litopenaeus vannamei)	Hydroxyproline supplementation in a 10% fishmeal diet	LF6 (10% fishmeal; 0.6% hydroxyproline)	LF0 (10% fishmeal; 0% hydroxyproline)	Hepatopancreas collagen content (Figure 1H)	Not reported numerically	Not reported numerically	3	3	Not estimable	Not estimable	Not estimable	The outcome was shown graphically with significance annotations; an exact effect size was not calculated	Exact group means and SEMs were presented only graphically and were not reliably extractable
Shi et al. (2024)	Pacific white shrimp (Litopenaeus vannamei)	Hydroxyproline supplementation in a 10% fishmeal diet	LF6 (10% fishmeal; 0.6% hydroxyproline)	LF0 (10% fishmeal; 0% hydroxyproline)	Muscle collagen content (Figure 1I)	Not reported numerically	Not reported numerically	3	3	Not estimable	Not estimable	Not estimable	The outcome was shown graphically with significance annotations; an exact effect size was not calculated	Exact group means and SEMs were presented only graphically and were not reliably extractable
Shi et al. (2024)	Pacific white shrimp (Litopenaeus vannamei)	Hydroxyproline supplementation in a 10% fishmeal diet	LF6 (10% fishmeal; 0.6% hydroxyproline)	LF0 (10% fishmeal; 0% hydroxyproline)	Muscle smyhc5 mRNA expression (Figure 2A)	Not reported numerically	Not reported numerically	3	3	Not estimable	Not estimable	Not estimable	The outcome was shown graphically with significance annotations; an exact effect size was not calculated	Exact group means and SEMs were presented only graphically and were not reliably extractable
Shi et al. (2024)	Pacific white shrimp (Litopenaeus vannamei)	Hydroxyproline supplementation in a 10% fishmeal diet	LF6 (10% fishmeal; 0.6% hydroxyproline)	LF0 (10% fishmeal; 0% hydroxyproline)	Muscle col1a1 mRNA expression (Figure 2A)	Not reported numerically	Not reported numerically	3	3	Not estimable	Not estimable	Not estimable	The outcome was shown graphically with significance annotations; an exact effect size was not calculated	Exact group means and SEMs were presented only graphically and were not reliably extractable
Shi et al. (2024)	Pacific white shrimp (Litopenaeus vannamei)	Hydroxyproline supplementation in a 10% fishmeal diet	LF6 (10% fishmeal; 0.6% hydroxyproline)	LF0 (10% fishmeal; 0% hydroxyproline)	Muscle col1a2 mRNA expression (Figure 2A)	Not reported numerically	Not reported numerically	3	3	Not estimable	Not estimable	Not estimable	The outcome was shown graphically with significance annotations; an exact effect size was not calculated	Exact group means and SEMs were presented only graphically and were not reliably extractable
Shi et al. (2024)	Pacific white shrimp (Litopenaeus vannamei)	Hydroxyproline supplementation in a 10% fishmeal diet	LF6 (10% fishmeal; 0.6% hydroxyproline)	LF0 (10% fishmeal; 0% hydroxyproline)	Muscle smyhc1 mRNA expression (Figure 2A)	Not reported numerically	Not reported numerically	3	3	Not estimable	Not estimable	Not estimable	The outcome was shown graphically with significance annotations; an exact effect size was not calculated	Exact group means and SEMs were presented only graphically and were not reliably extractable
Shi et al. (2024)	Pacific white shrimp (Litopenaeus vannamei)	Hydroxyproline supplementation in a 10% fishmeal diet	LF6 (10% fishmeal; 0.6% hydroxyproline)	LF0 (10% fishmeal; 0% hydroxyproline)	Muscle smyhc2 mRNA expression (Figure 2A)	Not reported numerically	Not reported numerically	3	3	Not estimable	Not estimable	Not estimable	The outcome was shown graphically with significance annotations; an exact effect size was not calculated	Exact group means and SEMs were presented only graphically and were not reliably extractable
Shi et al. (2024)	Pacific white shrimp (Litopenaeus vannamei)	Hydroxyproline supplementation in a 10% fishmeal diet	LF6 (10% fishmeal; 0.6% hydroxyproline)	LF0 (10% fishmeal; 0% hydroxyproline)	Muscle smyhc6a mRNA expression (Figure 2A)	Not reported numerically	Not reported numerically	3	3	Not estimable	Not estimable	Not estimable	The outcome was shown graphically with significance annotations; an exact effect size was not calculated	Exact group means and SEMs were presented only graphically and were not reliably extractable
Shi et al. (2024)	Pacific white shrimp (Litopenaeus vannamei)	Hydroxyproline supplementation in a 10% fishmeal diet	LF6 (10% fishmeal; 0.6% hydroxyproline)	LF0 (10% fishmeal; 0% hydroxyproline)	Muscle smyhc15 mRNA expression (Figure 2A)	Not reported numerically	Not reported numerically	3	3	Not estimable	Not estimable	Not estimable	The outcome was shown graphically with significance annotations; an exact effect size was not calculated	Exact group means and SEMs were presented only graphically and were not reliably extractable
Shi et al. (2024)	Pacific white shrimp (Litopenaeus vannamei)	Hydroxyproline supplementation in a 10% fishmeal diet	LF6 (10% fishmeal; 0.6% hydroxyproline)	LF0 (10% fishmeal; 0% hydroxyproline)	Muscle igf-1 mRNA expression (Figure 2B)	Not reported numerically	Not reported numerically	3	3	Not estimable	Not estimable	Not estimable	The outcome was shown graphically with significance annotations; an exact effect size was not calculated	Exact group means and SEMs were presented only graphically and were not reliably extractable
Shi et al. (2024)	Pacific white shrimp (Litopenaeus vannamei)	Hydroxyproline supplementation in a 10% fishmeal diet	LF6 (10% fishmeal; 0.6% hydroxyproline)	LF0 (10% fishmeal; 0% hydroxyproline)	Muscle tgf-b mRNA expression (Figure 2B)	Not reported numerically	Not reported numerically	3	3	Not estimable	Not estimable	Not estimable	The outcome was shown graphically with significance annotations; an exact effect size was not calculated	Exact group means and SEMs were presented only graphically and were not reliably extractable
Shi et al. (2024)	Pacific white shrimp (Litopenaeus vannamei)	Hydroxyproline supplementation in a 10% fishmeal diet	LF6 (10% fishmeal; 0.6% hydroxyproline)	LF0 (10% fishmeal; 0% hydroxyproline)	Muscle tor mRNA expression (Figure 2B)	Not reported numerically	Not reported numerically	3	3	Not estimable	Not estimable	Not estimable	The outcome was shown graphically with significance annotations; an exact effect size was not calculated	Exact group means and SEMs were presented only graphically and were not reliably extractable
Shi et al. (2024)	Pacific white shrimp (Litopenaeus vannamei)	Hydroxyproline supplementation in a 10% fishmeal diet	LF6 (10% fishmeal; 0.6% hydroxyproline)	LF0 (10% fishmeal; 0% hydroxyproline)	Hepatopancreas col1a1 mRNA expression (Figure 2C)	Not reported numerically	Not reported numerically	3	3	Not estimable	Not estimable	Not estimable	The outcome was shown graphically with significance annotations; an exact effect size was not calculated	Exact group means and SEMs were presented only graphically and were not reliably extractable
Shi et al. (2024)	Pacific white shrimp (Litopenaeus vannamei)	Hydroxyproline supplementation in a 10% fishmeal diet	LF6 (10% fishmeal; 0.6% hydroxyproline)	LF0 (10% fishmeal; 0% hydroxyproline)	Hepatopancreas col1a2 mRNA expression (Figure 2C)	Not reported numerically	Not reported numerically	3	3	Not estimable	Not estimable	Not estimable	The outcome was shown graphically with significance annotations; an exact effect size was not calculated	Exact group means and SEMs were presented only graphically and were not reliably extractable
Shi et al. (2024)	Pacific white shrimp (Litopenaeus vannamei)	Hydroxyproline supplementation in a 10% fishmeal diet	LF6 (10% fishmeal; 0.6% hydroxyproline)	LF0 (10% fishmeal; 0% hydroxyproline)	Hepatopancreas smyhc5 mRNA expression (Figure 2C)	Not reported numerically	Not reported numerically	3	3	Not estimable	Not estimable	Not estimable	The outcome was shown graphically with significance annotations; an exact effect size was not calculated	Exact group means and SEMs were presented only graphically and were not reliably extractable

Study	Species	Strategy	Intervention	Comparator	Outcome and unit	Intervention mean ± SD	Comparator mean ± SD	n intervention (tanks)	n comparator (tanks)	Effect-size metric	Effect size	95% CI	Interpretation	Reason not estimated
Shi et al. (2024)	Pacific white shrimp (Litopenaeus vannamei)	Hydroxyproline supplementation in a 10% fishmeal diet	LF6 (10% fishmeal; 0.6% hydroxyproline)	LF0 (10% fishmeal; 0% hydroxyproline)	Hepatopancreas smyhc6a mRNA expression (Figure 2C)	Not reported numerically	Not reported numerically	3	3	Not estimable	Not estimable	Not estimable	The outcome was shown graphically with significance annotations; an exact effect size was not calculated	Exact group means and SEMs were presented only graphically and were not reliably extractable
Shi et al. (2024)	Pacific white shrimp (Litopenaeus vannamei)	Hydroxyproline supplementation in a 10% fishmeal diet	LF6 (10% fishmeal; 0.6% hydroxyproline)	LF0 (10% fishmeal; 0% hydroxyproline)	Hepatopancreas tgf-b mRNA expression (Figure 2D)	Not reported numerically	Not reported numerically	3	3	Not estimable	Not estimable	Not estimable	The outcome was shown graphically with significance annotations; an exact effect size was not calculated	Exact group means and SEMs were presented only graphically and were not reliably extractable
Shi et al. (2024)	Pacific white shrimp (Litopenaeus vannamei)	Hydroxyproline supplementation in a 10% fishmeal diet	LF6 (10% fishmeal; 0.6% hydroxyproline)	LF0 (10% fishmeal; 0% hydroxyproline)	Hepatopancreas tor mRNA expression (Figure 2D)	Not reported numerically	Not reported numerically	3	3	Not estimable	Not estimable	Not estimable	The outcome was shown graphically with significance annotations; an exact effect size was not calculated	Exact group means and SEMs were presented only graphically and were not reliably extractable
Shi et al. (2024)	Pacific white shrimp (Litopenaeus vannamei)	Hydroxyproline supplementation in a 10% fishmeal diet	LF6 (10% fishmeal; 0.6% hydroxyproline)	LF0 (10% fishmeal; 0% hydroxyproline)	Hepatopancreas igf-1 mRNA expression (Figure 2D)	Not reported numerically	Not reported numerically	3	3	Not estimable	Not estimable	Not estimable	The outcome was shown graphically with significance annotations; an exact effect size was not calculated	Exact group means and SEMs were presented only graphically and were not reliably extractable
Shi et al. (2024)	Pacific white shrimp (Litopenaeus vannamei)	Hydroxyproline supplementation in a 10% fishmeal diet	LF6 (10% fishmeal; 0.6% hydroxyproline)	LF0 (10% fishmeal; 0% hydroxyproline)	Myofiber diameter (Figure 3C)	Not reported numerically	Not reported numerically	3	3	Not estimable	Not estimable	Not estimable	The outcome was shown graphically with significance annotations; an exact effect size was not calculated	Exact group means and SEMs were presented only graphically and were not reliably extractable
Shi et al. (2024)	Pacific white shrimp (Litopenaeus vannamei)	Hydroxyproline supplementation in a 10% fishmeal diet	LF6 (10% fishmeal; 0.6% hydroxyproline)	LF0 (10% fishmeal; 0% hydroxyproline)	Sarcomere diameter (Figure 3C)	Not reported numerically	Not reported numerically	3	3	Not estimable	Not estimable	Not estimable	Not reported for this intervention group	This outcome was not reported for the selected hydroxyproline dose; the figure included LF0 and LF4 only
Shi et al. (2024)	Pacific white shrimp (Litopenaeus vannamei)	Hydroxyproline supplementation in a 10% fishmeal diet	LF6 (10% fishmeal; 0.6% hydroxyproline)	LF0 (10% fishmeal; 0% hydroxyproline)	P-4E-BP1 protein expression (Figure 4B)	Not reported numerically	Not reported numerically	3	3	Not estimable	Not estimable	Not estimable	Not reported for this intervention group	This outcome was not reported for the selected hydroxyproline dose; the figure included LF0 and LF4 only
Shi et al. (2024)	Pacific white shrimp (Litopenaeus vannamei)	Hydroxyproline supplementation in a 10% fishmeal diet	LF6 (10% fishmeal; 0.6% hydroxyproline)	LF0 (10% fishmeal; 0% hydroxyproline)	4E-BP1 protein expression (Figure 4C)	Not reported numerically	Not reported numerically	3	3	Not estimable	Not estimable	Not estimable	Not reported for this intervention group	This outcome was not reported for the selected hydroxyproline dose; the figure included LF0 and LF4 only
Shi et al. (2024)	Pacific white shrimp (Litopenaeus vannamei)	Hydroxyproline supplementation in a 10% fishmeal diet	LF6 (10% fishmeal; 0.6% hydroxyproline)	LF0 (10% fishmeal; 0% hydroxyproline)	P-AKT protein expression (Figure 4D)	Not reported numerically	Not reported numerically	3	3	Not estimable	Not estimable	Not estimable	Not reported for this intervention group	This outcome was not reported for the selected hydroxyproline dose; the figure included LF0 and LF4 only
Shi et al. (2024)	Pacific white shrimp (Litopenaeus vannamei)	Hydroxyproline supplementation in a 10% fishmeal diet	LF6 (10% fishmeal; 0.6% hydroxyproline)	LF0 (10% fishmeal; 0% hydroxyproline)	AKT protein expression (Figure 4E)	Not reported numerically	Not reported numerically	3	3	Not estimable	Not estimable	Not estimable	Not reported for this intervention group	This outcome was not reported for the selected hydroxyproline dose; the figure included LF0 and LF4 only
Shi et al. (2024)	Pacific white shrimp (Litopenaeus vannamei)	Hydroxyproline supplementation in a 10% fishmeal diet	LF6 (10% fishmeal; 0.6% hydroxyproline)	LF0 (10% fishmeal; 0% hydroxyproline)	P-4E-BP1/4E-BP1 ratio (Figure 4F)	Not reported numerically	Not reported numerically	3	3	Not estimable	Not estimable	Not estimable	Not reported for this intervention group	This outcome was not reported for the selected hydroxyproline dose; the figure included LF0 and LF4 only
Shi et al. (2024)	Pacific white shrimp (Litopenaeus vannamei)	Hydroxyproline supplementation in a 10% fishmeal diet	LF6 (10% fishmeal; 0.6% hydroxyproline)	LF0 (10% fishmeal; 0% hydroxyproline)	P-AKT/AKT ratio (Figure 4G)	Not reported numerically	Not reported numerically	3	3	Not estimable	Not estimable	Not estimable	Not reported for this intervention group	This outcome was not reported for the selected hydroxyproline dose; the figure included LF0 and LF4 only
Shi et al. (2024)	Pacific white shrimp (Litopenaeus vannamei)	Hydroxyproline supplementation in a 10% fishmeal diet	LF8 (10% fishmeal; 0.8% hydroxyproline)	LF0 (10% fishmeal; 0% hydroxyproline)	Hemolymph prolyl 4-hydroxylases content (Figure 1A)	Not reported numerically	Not reported numerically	3	3	Not estimable	Not estimable	Not estimable	The outcome was shown graphically with significance annotations; an exact effect size was not calculated	Exact group means and SEMs were presented only graphically and were not reliably extractable
Shi et al. (2024)	Pacific white shrimp (Litopenaeus vannamei)	Hydroxyproline supplementation in a 10% fishmeal diet	LF8 (10% fishmeal; 0.8% hydroxyproline)	LF0 (10% fishmeal; 0% hydroxyproline)	Hepatopancreas prolyl 4-hydroxylases content (Figure 1B)	Not reported numerically	Not reported numerically	3	3	Not estimable	Not estimable	Not estimable	The outcome was shown graphically with significance annotations; an exact effect size was not calculated	Exact group means and SEMs were presented only graphically and were not reliably extractable
Shi et al. (2024)	Pacific white shrimp (Litopenaeus vannamei)	Hydroxyproline supplementation in a 10% fishmeal diet	LF8 (10% fishmeal; 0.8% hydroxyproline)	LF0 (10% fishmeal; 0% hydroxyproline)	Muscle prolyl 4-hydroxylases content (Figure 1C)	Not reported numerically	Not reported numerically	3	3	Not estimable	Not estimable	Not estimable	The outcome was shown graphically with significance annotations; an exact effect size was not calculated	Exact group means and SEMs were presented only graphically and were not reliably extractable
Shi et al. (2024)	Pacific white shrimp (Litopenaeus vannamei)	Hydroxyproline supplementation in a 10% fishmeal diet	LF8 (10% fishmeal; 0.8% hydroxyproline)	LF0 (10% fishmeal; 0% hydroxyproline)	Hemolymph hydroxyproline content (Figure 1D)	Not reported numerically	Not reported numerically	3	3	Not estimable	Not estimable	Not estimable	The outcome was shown graphically with significance annotations; an exact effect size was not calculated	Exact group means and SEMs were presented only graphically and were not reliably extractable
Shi et al. (2024)	Pacific white shrimp (Litopenaeus vannamei)	Hydroxyproline supplementation in a 10% fishmeal diet	LF8 (10% fishmeal; 0.8% hydroxyproline)	LF0 (10% fishmeal; 0% hydroxyproline)	Hepatopancreas hydroxyproline content (Figure 1E)	Not reported numerically	Not reported numerically	3	3	Not estimable	Not estimable	Not estimable	The outcome was shown graphically with significance annotations; an exact effect size was not calculated	Exact group means and SEMs were presented only graphically and were not reliably extractable
Shi et al. (2024)	Pacific white shrimp (Litopenaeus vannamei)	Hydroxyproline supplementation in a 10% fishmeal diet	LF8 (10% fishmeal; 0.8% hydroxyproline)	LF0 (10% fishmeal; 0% hydroxyproline)	Muscle hydroxyproline content (Figure 1F)	Not reported numerically	Not reported numerically	3	3	Not estimable	Not estimable	Not estimable	The outcome was shown graphically with significance annotations; an exact effect size was not calculated	Exact group means and SEMs were presented only graphically and were not reliably extractable
Shi et al. (2024)	Pacific white shrimp (Litopenaeus vannamei)	Hydroxyproline supplementation in a 10% fishmeal diet	LF8 (10% fishmeal; 0.8% hydroxyproline)	LF0 (10% fishmeal; 0% hydroxyproline)	Hemolymph collagen content (Figure 1G)	Not reported numerically	Not reported numerically	3	3	Not estimable	Not estimable	Not estimable	The outcome was shown graphically with significance annotations; an exact effect size was not calculated	Exact group means and SEMs were presented only graphically and were not reliably extractable
Shi et al. (2024)	Pacific white shrimp (Litopenaeus vannamei)	Hydroxyproline supplementation in a 10% fishmeal diet	LF8 (10% fishmeal; 0.8% hydroxyproline)	LF0 (10% fishmeal; 0% hydroxyproline)	Hepatopancreas collagen content (Figure 1H)	Not reported numerically	Not reported numerically	3	3	Not estimable	Not estimable	Not estimable	The outcome was shown graphically with significance annotations; an exact effect size was not calculated	Exact group means and SEMs were presented only graphically and were not reliably extractable
Shi et al. (2024)	Pacific white shrimp (Litopenaeus vannamei)	Hydroxyproline supplementation in a 10% fishmeal diet	LF8 (10% fishmeal; 0.8% hydroxyproline)	LF0 (10% fishmeal; 0% hydroxyproline)	Muscle collagen content (Figure 1I)	Not reported numerically	Not reported numerically	3	3	Not estimable	Not estimable	Not estimable	The outcome was shown graphically with significance annotations; an exact effect size was not calculated	Exact group means and SEMs were presented only graphically and were not reliably extractable
Shi et al. (2024)	Pacific white shrimp (Litopenaeus vannamei)	Hydroxyproline supplementation in a 10% fishmeal diet	LF8 (10% fishmeal; 0.8% hydroxyproline)	LF0 (10% fishmeal; 0% hydroxyproline)	Muscle smyhc5 mRNA expression (Figure 2A)	Not reported numerically	Not reported numerically	3	3	Not estimable	Not estimable	Not estimable	The outcome was shown graphically with significance annotations; an exact effect size was not calculated	Exact group means and SEMs were presented only graphically and were not reliably extractable
Shi et al. (2024)	Pacific white shrimp (Litopenaeus vannamei)	Hydroxyproline supplementation in a 10% fishmeal diet	LF8 (10% fishmeal; 0.8% hydroxyproline)	LF0 (10% fishmeal; 0% hydroxyproline)	Muscle col1a1 mRNA expression (Figure 2A)	Not reported numerically	Not reported numerically	3	3	Not estimable	Not estimable	Not estimable	The outcome was shown graphically with significance annotations; an exact effect size was not calculated	Exact group means and SEMs were presented only graphically and were not reliably extractable
Shi et al. (2024)	Pacific white shrimp (Litopenaeus vannamei)	Hydroxyproline supplementation in a 10% fishmeal diet	LF8 (10% fishmeal; 0.8% hydroxyproline)	LF0 (10% fishmeal; 0% hydroxyproline)	Muscle col1a2 mRNA expression (Figure 2A)	Not reported numerically	Not reported numerically	3	3	Not estimable	Not estimable	Not estimable	The outcome was shown graphically with significance annotations; an exact effect size was not calculated	Exact group means and SEMs were presented only graphically and were not reliably extractable
Shi et al. (2024)	Pacific white shrimp (Litopenaeus vannamei)	Hydroxyproline supplementation in a 10% fishmeal diet	LF8 (10% fishmeal; 0.8% hydroxyproline)	LF0 (10% fishmeal; 0% hydroxyproline)	Muscle smyhc1 mRNA expression (Figure 2A)	Not reported numerically	Not reported numerically	3	3	Not estimable	Not estimable	Not estimable	The outcome was shown graphically with significance annotations; an exact effect size was not calculated	Exact group means and SEMs were presented only graphically and were not reliably extractable
Shi et al. (2024)	Pacific white shrimp (Litopenaeus vannamei)	Hydroxyproline supplementation in a 10% fishmeal diet	LF8 (10% fishmeal; 0.8% hydroxyproline)	LF0 (10% fishmeal; 0% hydroxyproline)	Muscle smyhc2 mRNA expression (Figure 2A)	Not reported numerically	Not reported numerically	3	3	Not estimable	Not estimable	Not estimable	The outcome was shown graphically with significance annotations; an exact effect size was not calculated	Exact group means and SEMs were presented only graphically and were not reliably extractable
Shi et al. (2024)	Pacific white shrimp (Litopenaeus vannamei)	Hydroxyproline supplementation in a 10% fishmeal diet	LF8 (10% fishmeal; 0.8% hydroxyproline)	LF0 (10% fishmeal; 0% hydroxyproline)	Muscle smyhc6a mRNA expression (Figure 2A)	Not reported numerically	Not reported numerically	3	3	Not estimable	Not estimable	Not estimable	The outcome was shown graphically with significance annotations; an exact effect size was not calculated	Exact group means and SEMs were presented only graphically and were not reliably extractable
Shi et al. (2024)	Pacific white shrimp (Litopenaeus vannamei)	Hydroxyproline supplementation in a 10% fishmeal diet	LF8 (10% fishmeal; 0.8% hydroxyproline)	LF0 (10% fishmeal; 0% hydroxyproline)	Muscle smyhc15 mRNA expression (Figure 2A)	Not reported numerically	Not reported numerically	3	3	Not estimable	Not estimable	Not estimable	The outcome was shown graphically with significance annotations; an exact effect size was not calculated	Exact group means and SEMs were presented only graphically and were not reliably extractable
Shi et al. (2024)	Pacific white shrimp (Litopenaeus vannamei)	Hydroxyproline supplementation in a 10% fishmeal diet	LF8 (10% fishmeal; 0.8% hydroxyproline)	LF0 (10% fishmeal; 0% hydroxyproline)	Muscle igf-1 mRNA expression (Figure 2B)	Not reported numerically	Not reported numerically	3	3	Not estimable	Not estimable	Not estimable	The outcome was shown graphically with significance annotations; an exact effect size was not calculated	Exact group means and SEMs were presented only graphically and were not reliably extractable
Shi et al. (2024)	Pacific white shrimp (Litopenaeus vannamei)	Hydroxyproline supplementation in a 10% fishmeal diet	LF8 (10% fishmeal; 0.8% hydroxyproline)	LF0 (10% fishmeal; 0% hydroxyproline)	Muscle tgf-b mRNA expression (Figure 2B)	Not reported numerically	Not reported numerically	3	3	Not estimable	Not estimable	Not estimable	The outcome was shown graphically with significance annotations; an exact effect size was not calculated	Exact group means and SEMs were presented only graphically and were not reliably extractable
Shi et al. (2024)	Pacific white shrimp (Litopenaeus vannamei)	Hydroxyproline supplementation in a 10% fishmeal diet	LF8 (10% fishmeal; 0.8% hydroxyproline)	LF0 (10% fishmeal; 0% hydroxyproline)	Muscle tor mRNA expression (Figure 2B)	Not reported numerically	Not reported numerically	3	3	Not estimable	Not estimable	Not estimable	The outcome was shown graphically with significance annotations; an exact effect size was not calculated	Exact group means and SEMs were presented only graphically and were not reliably extractable

Study	Species	Strategy	Intervention	Comparator	Outcome and unit	Intervention mean ± SD	Comparator mean ± SD	n intervention (tanks)	n comparator (tanks)	Effect-size metric	Effect size	95% CI	Interpretation	Reason not estimated
Shi et al. (2024)	Pacific white shrimp (Litopenaeus vannamei)	Hydroxyproline supplementation in a 10% fishmeal diet	LF8 (10% fishmeal; 0.8% hydroxyproline)	LF0 (10% fishmeal; 0% hydroxyproline)	Hepatopancreas col1a1 mRNA expression (Figure 2C)	Not reported numerically	Not reported numerically	3	3	Not estimable	Not estimable	Not estimable	The outcome was shown graphically with significance annotations; an exact effect size was not calculated	Exact group means and SEMs were presented only graphically and were not reliably extractable
Shi et al. (2024)	Pacific white shrimp (Litopenaeus vannamei)	Hydroxyproline supplementation in a 10% fishmeal diet	LF8 (10% fishmeal; 0.8% hydroxyproline)	LF0 (10% fishmeal; 0% hydroxyproline)	Hepatopancreas col1a2 mRNA expression (Figure 2C)	Not reported numerically	Not reported numerically	3	3	Not estimable	Not estimable	Not estimable	The outcome was shown graphically with significance annotations; an exact effect size was not calculated	Exact group means and SEMs were presented only graphically and were not reliably extractable
Shi et al. (2024)	Pacific white shrimp (Litopenaeus vannamei)	Hydroxyproline supplementation in a 10% fishmeal diet	LF8 (10% fishmeal; 0.8% hydroxyproline)	LF0 (10% fishmeal; 0% hydroxyproline)	Hepatopancreas smyhc5 mRNA expression (Figure 2C)	Not reported numerically	Not reported numerically	3	3	Not estimable	Not estimable	Not estimable	The outcome was shown graphically with significance annotations; an exact effect size was not calculated	Exact group means and SEMs were presented only graphically and were not reliably extractable
Shi et al. (2024)	Pacific white shrimp (Litopenaeus vannamei)	Hydroxyproline supplementation in a 10% fishmeal diet	LF8 (10% fishmeal; 0.8% hydroxyproline)	LF0 (10% fishmeal; 0% hydroxyproline)	Hepatopancreas smyhc6a mRNA expression (Figure 2C)	Not reported numerically	Not reported numerically	3	3	Not estimable	Not estimable	Not estimable	The outcome was shown graphically with significance annotations; an exact effect size was not calculated	Exact group means and SEMs were presented only graphically and were not reliably extractable
Shi et al. (2024)	Pacific white shrimp (Litopenaeus vannamei)	Hydroxyproline supplementation in a 10% fishmeal diet	LF8 (10% fishmeal; 0.8% hydroxyproline)	LF0 (10% fishmeal; 0% hydroxyproline)	Hepatopancreas tgf-b mRNA expression (Figure 2D)	Not reported numerically	Not reported numerically	3	3	Not estimable	Not estimable	Not estimable	The outcome was shown graphically with significance annotations; an exact effect size was not calculated	Exact group means and SEMs were presented only graphically and were not reliably extractable
Shi et al. (2024)	Pacific white shrimp (Litopenaeus vannamei)	Hydroxyproline supplementation in a 10% fishmeal diet	LF8 (10% fishmeal; 0.8% hydroxyproline)	LF0 (10% fishmeal; 0% hydroxyproline)	Hepatopancreas tor mRNA expression (Figure 2D)	Not reported numerically	Not reported numerically	3	3	Not estimable	Not estimable	Not estimable	The outcome was shown graphically with significance annotations; an exact effect size was not calculated	Exact group means and SEMs were presented only graphically and were not reliably extractable
Shi et al. (2024)	Pacific white shrimp (Litopenaeus vannamei)	Hydroxyproline supplementation in a 10% fishmeal diet	LF8 (10% fishmeal; 0.8% hydroxyproline)	LF0 (10% fishmeal; 0% hydroxyproline)	Hepatopancreas igf-1 mRNA expression (Figure 2D)	Not reported numerically	Not reported numerically	3	3	Not estimable	Not estimable	Not estimable	The outcome was shown graphically with significance annotations; an exact effect size was not calculated	Exact group means and SEMs were presented only graphically and were not reliably extractable
Shi et al. (2024)	Pacific white shrimp (Litopenaeus vannamei)	Hydroxyproline supplementation in a 10% fishmeal diet	LF8 (10% fishmeal; 0.8% hydroxyproline)	LF0 (10% fishmeal; 0% hydroxyproline)	Myofiber diameter (Figure 3C)	Not reported numerically	Not reported numerically	3	3	Not estimable	Not estimable	Not estimable	The outcome was shown graphically with significance annotations; an exact effect size was not calculated	Exact group means and SEMs were presented only graphically and were not reliably extractable
Shi et al. (2024)	Pacific white shrimp (Litopenaeus vannamei)	Hydroxyproline supplementation in a 10% fishmeal diet	LF8 (10% fishmeal; 0.8% hydroxyproline)	LF0 (10% fishmeal; 0% hydroxyproline)	Sarcomere diameter (Figure 3C)	Not reported numerically	Not reported numerically	3	3	Not estimable	Not estimable	Not estimable	Not reported for this intervention group	This outcome was not reported for the selected hydroxyproline dose; the figure included LF0 and LF4 only
Shi et al. (2024)	Pacific white shrimp (Litopenaeus vannamei)	Hydroxyproline supplementation in a 10% fishmeal diet	LF8 (10% fishmeal; 0.8% hydroxyproline)	LF0 (10% fishmeal; 0% hydroxyproline)	P-4E-BP1 protein expression (Figure 4B)	Not reported numerically	Not reported numerically	3	3	Not estimable	Not estimable	Not estimable	Not reported for this intervention group	This outcome was not reported for the selected hydroxyproline dose; the figure included LF0 and LF4 only
Shi et al. (2024)	Pacific white shrimp (Litopenaeus vannamei)	Hydroxyproline supplementation in a 10% fishmeal diet	LF8 (10% fishmeal; 0.8% hydroxyproline)	LF0 (10% fishmeal; 0% hydroxyproline)	4E-BP1 protein expression (Figure 4C)	Not reported numerically	Not reported numerically	3	3	Not estimable	Not estimable	Not estimable	Not reported for this intervention group	This outcome was not reported for the selected hydroxyproline dose; the figure included LF0 and LF4 only
Shi et al. (2024)	Pacific white shrimp (Litopenaeus vannamei)	Hydroxyproline supplementation in a 10% fishmeal diet	LF8 (10% fishmeal; 0.8% hydroxyproline)	LF0 (10% fishmeal; 0% hydroxyproline)	P-AKT protein expression (Figure 4D)	Not reported numerically	Not reported numerically	3	3	Not estimable	Not estimable	Not estimable	Not reported for this intervention group	This outcome was not reported for the selected hydroxyproline dose; the figure included LF0 and LF4 only
Shi et al. (2024)	Pacific white shrimp (Litopenaeus vannamei)	Hydroxyproline supplementation in a 10% fishmeal diet	LF8 (10% fishmeal; 0.8% hydroxyproline)	LF0 (10% fishmeal; 0% hydroxyproline)	AKT protein expression (Figure 4E)	Not reported numerically	Not reported numerically	3	3	Not estimable	Not estimable	Not estimable	Not reported for this intervention group	This outcome was not reported for the selected hydroxyproline dose; the figure included LF0 and LF4 only
Shi et al. (2024)	Pacific white shrimp (Litopenaeus vannamei)	Hydroxyproline supplementation in a 10% fishmeal diet	LF8 (10% fishmeal; 0.8% hydroxyproline)	LF0 (10% fishmeal; 0% hydroxyproline)	P-4E-BP1/4E-BP1 ratio (Figure 4F)	Not reported numerically	Not reported numerically	3	3	Not estimable	Not estimable	Not estimable	Not reported for this intervention group	This outcome was not reported for the selected hydroxyproline dose; the figure included LF0 and LF4 only
Shi et al. (2024)	Pacific white shrimp (Litopenaeus vannamei)	Hydroxyproline supplementation in a 10% fishmeal diet	LF8 (10% fishmeal; 0.8% hydroxyproline)	LF0 (10% fishmeal; 0% hydroxyproline)	P-AKT/AKT ratio (Figure 4G)	Not reported numerically	Not reported numerically	3	3	Not estimable	Not estimable	Not estimable	Not reported for this intervention group	This outcome was not reported for the selected hydroxyproline dose; the figure included LF0 and LF4 only

Note: Tables 1 and 2 report mean ± SEM and state n = 3, although the abstract and methods describe four replicates. Effect-size calculations therefore use n = 3 and convert SEM to SD as SD = SEM × sqrt(3). Positive values favor hydroxyproline; FCR, cooking loss, and freezing loss were direction-reversed because lower values are favorable. The 25% fishmeal diet was an external benchmark and was not included in the primary low-fishmeal comparisons. Figure-only and unavailable supplementary outcomes were marked not estimable. Pairwise confidence intervals are unadjusted.

Source: Shi et al. (2024), https://doi.org/10.1016/j.aninu.2024.01.013

Study 10. Cao et al. (2022): graded hydroxyproline supplementation versus the 0.09% Hyp control diet

Study	Species	Strategy	Intervention	Comparator	Outcome and unit	Intervention mean ± SD	Comparator mean ± SD	n intervention (independent tanks)	n comparator (independent tanks)	Effect-size metric	Effect size	95% CI	Interpretation	Reason not estimated
Cao et al. (2022)	Improved triploid crucian carp (Carassius auratus triploid)	Hydroxyproline supplementation in a plant-protein-based diet	Hyp 0.39% (0.40% crystalline Hyp added)	Hyp 0.09% (control; no crystalline Hyp added)	Final body weight (g)	43.95 ± 2.29	41.94 ± 1.85	3	3	Hedges' g (SE-to-SD; CI based on independent tanks)	0.77	-0.91 to 2.46	Higher, more favorable value with hydroxyproline; 95% CI included 0 (imprecise estimate)	Not applicable - effect size estimated; see Notes for subsampling treatment
Cao et al. (2022)	Improved triploid crucian carp (Carassius auratus triploid)	Hydroxyproline supplementation in a plant-protein-based diet	Hyp 0.39% (0.40% crystalline Hyp added)	Hyp 0.09% (control; no crystalline Hyp added)	Weight gain rate (%)	192.92 ± 15.97	179.76 ± 13.48	3	3	Hedges' g (SE-to-SD; CI based on independent tanks)	0.71	-0.96 to 2.39	Higher, more favorable value with hydroxyproline; 95% CI included 0 (imprecise estimate)	Not applicable - effect size estimated; see Notes for subsampling treatment
Cao et al. (2022)	Improved triploid crucian carp (Carassius auratus triploid)	Hydroxyproline supplementation in a plant-protein-based diet	Hyp 0.39% (0.40% crystalline Hyp added)	Hyp 0.09% (control; no crystalline Hyp added)	Specific growth rate (%/day)	1.92 ± 0.10	1.84 ± 0.09	3	3	Hedges' g (SE-to-SD; CI based on independent tanks)	0.67	-1.00 to 2.34	Higher, more favorable value with hydroxyproline; 95% CI included 0 (imprecise estimate)	Not applicable - effect size estimated; see Notes for subsampling treatment
Cao et al. (2022)	Improved triploid crucian carp (Carassius auratus triploid)	Hydroxyproline supplementation in a plant-protein-based diet	Hyp 0.39% (0.40% crystalline Hyp added)	Hyp 0.09% (control; no crystalline Hyp added)	Feed efficiency (%)	47.21 ± 4.50	44.60 ± 3.00	3	3	Hedges' g (SE-to-SD; CI based on independent tanks)	0.55	-1.10 to 2.19	Higher, more favorable value with hydroxyproline; 95% CI included 0 (imprecise estimate)	Not applicable - effect size estimated; see Notes for subsampling treatment
Cao et al. (2022)	Improved triploid crucian carp (Carassius auratus triploid)	Hydroxyproline supplementation in a plant-protein-based diet	Hyp 0.39% (0.40% crystalline Hyp added)	Hyp 0.09% (control; no crystalline Hyp added)	Feeding rate (% body weight/day)	3.43 ± 0.14	3.54 ± 0.10	3	3	Hedges' g (SE-to-SD; CI based on independent tanks)	0.72	-0.96 to 2.39	Lower, more favorable value with hydroxyproline; 95% CI included 0 (imprecise estimate)	Not applicable - effect size estimated; see Notes for subsampling treatment
Cao et al. (2022)	Improved triploid crucian carp (Carassius auratus triploid)	Hydroxyproline supplementation in a plant-protein-based diet	Hyp 0.39% (0.40% crystalline Hyp added)	Hyp 0.09% (control; no crystalline Hyp added)	Protein efficiency ratio (%)	141.04 ± 18.10	128.49 ± 8.61	3	3	Hedges' g (SE-to-SD; CI based on independent tanks)	0.71	-0.97 to 2.38	Higher, more favorable value with hydroxyproline; 95% CI included 0 (imprecise estimate)	Not applicable - effect size estimated; see Notes for subsampling treatment
Cao et al. (2022)	Improved triploid crucian carp (Carassius auratus triploid)	Hydroxyproline supplementation in a plant-protein-based diet	Hyp 0.39% (0.40% crystalline Hyp added)	Hyp 0.09% (control; no crystalline Hyp added)	Nitrogen retention efficiency (%)	23.40 ± 3.45	22.71 ± 1.47	3	3	Hedges' g (SE-to-SD; CI based on independent tanks)	0.21	-1.40 to 1.82	Higher, more favorable value with hydroxyproline; 95% CI included 0 (imprecise estimate)	Not applicable - effect size estimated; see Notes for subsampling treatment
Cao et al. (2022)	Improved triploid crucian carp (Carassius auratus triploid)	Hydroxyproline supplementation in a plant-protein-based diet	Hyp 0.39% (0.40% crystalline Hyp added)	Hyp 0.09% (control; no crystalline Hyp added)	Total nitrogen waste output (g/kg weight gain)	43.12 ± 4.59	45.57 ± 1.59	3	3	Hedges' g (SE-to-SD; CI based on independent tanks)	0.57	-1.08 to 2.22	Lower, more favorable value with hydroxyproline; 95% CI included 0 (imprecise estimate)	Not applicable - effect size estimated; see Notes for subsampling treatment
Cao et al. (2022)	Improved triploid crucian carp (Carassius auratus triploid)	Hydroxyproline supplementation in a plant-protein-based diet	Hyp 0.39% (0.40% crystalline Hyp added)	Hyp 0.09% (control; no crystalline Hyp added)	Survival rate (%)	100.00 ± 0.00	100.00 ± 0.00	3	3	Not estimable	Not estimable	Not estimable	No observed mean difference and no within-group variance	Both groups had zero reported SE; standardized effect size is undefined
Cao et al. (2022)	Improved triploid crucian carp (Carassius auratus triploid)	Hydroxyproline supplementation in a plant-protein-based diet	Hyp 0.39% (0.40% crystalline Hyp added)	Hyp 0.09% (control; no crystalline Hyp added)	Whole-body crude protein (% fresh weight)	15.98 ± 0.19	15.72 ± 0.57	3	3	Hedges' g (SE-to-SD; CI based on independent tanks)	0.49	-1.15 to 2.12	Higher measured value with hydroxyproline; 95% CI included 0 (imprecise estimate)	Not applicable - effect size estimated; see Notes for subsampling treatment
Cao et al. (2022)	Improved triploid crucian carp (Carassius auratus triploid)	Hydroxyproline supplementation in a plant-protein-based diet	Hyp 0.39% (0.40% crystalline Hyp added)	Hyp 0.09% (control; no crystalline Hyp added)	Whole-body crude lipid (% fresh weight)	11.18 ± 3.48	11.04 ± 0.87	3	3	Hedges' g (SE-to-SD; CI based on independent tanks)	0.04	-1.56 to 1.64	Higher measured value with hydroxyproline; 95% CI included 0 (imprecise estimate)	Not applicable - effect size estimated; see Notes for subsampling treatment
Cao et al. (2022)	Improved triploid crucian carp (Carassius auratus triploid)	Hydroxyproline supplementation in a plant-protein-based diet	Hyp 0.39% (0.40% crystalline Hyp added)	Hyp 0.09% (control; no crystalline Hyp added)	Whole-body ash (% fresh weight)	2.47 ± 0.21	2.85 ± 0.28	3	3	Hedges' g (SE-to-SD; CI based on independent tanks)	-1.24	-3.06 to 0.58	Lower measured value with hydroxyproline; 95% CI included 0 (imprecise estimate)	Not applicable - effect size estimated; see Notes for subsampling treatment
Cao et al. (2022)	Improved triploid crucian carp (Carassius auratus triploid)	Hydroxyproline supplementation in a plant-protein-based diet	Hyp 0.39% (0.40% crystalline Hyp added)	Hyp 0.09% (control; no crystalline Hyp added)	Whole-body moisture (% fresh weight)	68.15 ± 0.80	69.85 ± 0.42	3	3	Hedges' g (SE-to-SD; CI based on independent tanks)	-2.14	-4.32 to 0.04	Lower measured value with hydroxyproline; 95% CI included 0 (imprecise estimate)	Not applicable - effect size estimated; see Notes for subsampling treatment
Cao et al. (2022)	Improved triploid crucian carp (Carassius auratus triploid)	Hydroxyproline supplementation in a plant-protein-based diet	Hyp 0.39% (0.40% crystalline Hyp added)	Hyp 0.09% (control; no crystalline Hyp added)	Dorsal-muscle crude protein (% fresh weight)	17.69 ± 0.19	17.06 ± 0.21	3	3	Hedges' g (SE-to-SD; CI based on independent tanks)	2.53	0.16 to 4.90	Higher measured value with hydroxyproline; 95% CI excluded 0	Not applicable - effect size estimated; see Notes for subsampling treatment
Cao et al. (2022)	Improved triploid crucian carp (Carassius auratus triploid)	Hydroxyproline supplementation in a plant-protein-based diet	Hyp 0.39% (0.40% crystalline Hyp added)	Hyp 0.09% (control; no crystalline Hyp added)	Dorsal-muscle crude lipid (% fresh weight)	1.24 ± 0.12	1.32 ± 0.12	3	3	Hedges' g (SE-to-SD; CI based on independent tanks)	-0.53	-2.17 to 1.11	Lower measured value with hydroxyproline; 95% CI included 0 (imprecise estimate)	Not applicable - effect size estimated; see Notes for subsampling treatment
Cao et al. (2022)	Improved triploid crucian carp (Carassius auratus triploid)	Hydroxyproline supplementation in a plant-protein-based diet	Hyp 0.39% (0.40% crystalline Hyp added)	Hyp 0.09% (control; no crystalline Hyp added)	Dorsal-muscle ash (% fresh weight)	1.38 ± 0.10	1.44 ± 0.10	3	3	Hedges' g (SE-to-SD; CI based on independent tanks)	-0.46	-2.09 to 1.17	Lower measured value with hydroxyproline; 95% CI included 0 (imprecise estimate)	Not applicable - effect size estimated; see Notes for subsampling treatment
Cao et al. (2022)	Improved triploid crucian carp (Carassius auratus triploid)	Hydroxyproline supplementation in a plant-protein-based diet	Hyp 0.39% (0.40% crystalline Hyp added)	Hyp 0.09% (control; no crystalline Hyp added)	Dorsal-muscle moisture (% fresh weight)	76.23 ± 0.73	75.65 ± 0.16	3	3	Hedges' g (SE-to-SD; CI based on independent tanks)	0.88	-0.83 to 2.60	Higher measured value with hydroxyproline; 95% CI included 0 (imprecise estimate)	Not applicable - effect size estimated; see Notes for subsampling treatment
Cao et al. (2022)	Improved triploid crucian carp (Carassius auratus triploid)	Hydroxyproline supplementation in a plant-protein-based diet	Hyp 0.39% (0.40% crystalline Hyp added)	Hyp 0.09% (control; no crystalline Hyp added)	Threonine (% dry matter)	2.86 ± 0.26	2.62 ± 0.16	3	3	Hedges' g (SE-to-SD; CI based on independent tanks)	0.9	-0.82 to 2.61	Higher measured value with hydroxyproline; 95% CI included 0 (imprecise estimate)	Not applicable - effect size estimated; see Notes for subsampling treatment
Cao et al. (2022)	Improved triploid crucian carp (Carassius auratus triploid)	Hydroxyproline supplementation in a plant-protein-based diet	Hyp 0.39% (0.40% crystalline Hyp added)	Hyp 0.09% (control; no crystalline Hyp added)	Phenylalanine (% dry matter)	3.16 ± 0.31	2.95 ± 0.19	3	3	Hedges' g (SE-to-SD; CI based on independent tanks)	0.65	-1.01 to 2.31	Higher measured value with hydroxyproline; 95% CI included 0 (imprecise estimate)	Not applicable - effect size estimated; see Notes for subsampling treatment
Cao et al. (2022)	Improved triploid crucian carp (Carassius auratus triploid)	Hydroxyproline supplementation in a plant-protein-based diet	Hyp 0.39% (0.40% crystalline Hyp added)	Hyp 0.09% (control; no crystalline Hyp added)	Valine (% dry matter)	3.13 ± 0.26	2.98 ± 0.19	3	3	Hedges' g (SE-to-SD; CI based on independent tanks)	0.53	-1.11 to 2.17	Higher measured value with hydroxyproline; 95% CI included 0 (imprecise estimate)	Not applicable - effect size estimated; see Notes for subsampling treatment
Cao et al. (2022)	Improved triploid crucian carp (Carassius auratus triploid)	Hydroxyproline supplementation in a plant-protein-based diet	Hyp 0.39% (0.40% crystalline Hyp added)	Hyp 0.09% (control; no crystalline Hyp added)	Isoleucine (% dry matter)	3.21 ± 0.28	3.03 ± 0.17	3	3	Hedges' g (SE-to-SD; CI based on independent tanks)	0.62	-1.03 to 2.28	Higher measured value with hydroxyproline; 95% CI included 0 (imprecise estimate)	Not applicable - effect size estimated; see Notes for subsampling treatment
Cao et al. (2022)	Improved triploid crucian carp (Carassius auratus triploid)	Hydroxyproline supplementation in a plant-protein-based diet	Hyp 0.39% (0.40% crystalline Hyp added)	Hyp 0.09% (control; no crystalline Hyp added)	Leucine (% dry matter)	5.68 ± 0.50	5.36 ± 0.31	3	3	Hedges' g (SE-to-SD; CI based on independent tanks)	0.61	-1.04 to 2.27	Higher measured value with hydroxyproline; 95% CI included 0 (imprecise estimate)	Not applicable - effect size estimated; see Notes for subsampling treatment
Cao et al. (2022)	Improved triploid crucian carp (Carassius auratus triploid)	Hydroxyproline supplementation in a plant-protein-based diet	Hyp 0.39% (0.40% crystalline Hyp added)	Hyp 0.09% (control; no crystalline Hyp added)	Methionine (% dry matter)	1.96 ± 0.19	1.72 ± 0.05	3	3	Hedges' g (SE-to-SD; CI based on independent tanks)	1.37	-0.49 to 3.24	Higher measured value with hydroxyproline; 95% CI included 0 (imprecise estimate)	Not applicable - effect size estimated; see Notes for subsampling treatment
Cao et al. (2022)	Improved triploid crucian carp (Carassius auratus triploid)	Hydroxyproline supplementation in a plant-protein-based diet	Hyp 0.39% (0.40% crystalline Hyp added)	Hyp 0.09% (control; no crystalline Hyp added)	Arginine (% dry matter)	3.94 ± 0.38	3.67 ± 0.23	3	3	Hedges' g (SE-to-SD; CI based on independent tanks)	0.69	-0.98 to 2.36	Higher measured value with hydroxyproline; 95% CI included 0 (imprecise estimate)	Not applicable - effect size estimated; see Notes for subsampling treatment
Cao et al. (2022)	Improved triploid crucian carp (Carassius auratus triploid)	Hydroxyproline supplementation in a plant-protein-based diet	Hyp 0.39% (0.40% crystalline Hyp added)	Hyp 0.09% (control; no crystalline Hyp added)	Lysine (% dry matter)	7.07 ± 0.74	6.01 ± 0.50	3	3	Hedges' g (SE-to-SD; CI based on independent tanks)	1.33	-0.51 to 3.18	Higher measured value with hydroxyproline; 95% CI included 0 (imprecise estimate)	Not applicable - effect size estimated; see Notes for subsampling treatment
Cao et al. (2022)	Improved triploid crucian carp (Carassius auratus triploid)	Hydroxyproline supplementation in a plant-protein-based diet	Hyp 0.39% (0.40% crystalline Hyp added)	Hyp 0.09% (control; no crystalline Hyp added)	Histidine (% dry matter)	3.42 ± 0.28	3.21 ± 0.24	3	3	Hedges' g (SE-to-SD; CI based on independent tanks)	0.65	-1.02 to 2.31	Higher measured value with hydroxyproline; 95% CI included 0 (imprecise estimate)	Not applicable - effect size estimated; see Notes for subsampling treatment
Cao et al. (2022)	Improved triploid crucian carp (Carassius auratus triploid)	Hydroxyproline supplementation in a plant-protein-based diet	Hyp 0.39% (0.40% crystalline Hyp added)	Hyp 0.09% (control; no crystalline Hyp added)	Aspartic acid (% dry matter)	7.23 ± 0.69	6.84 ± 0.43	3	3	Hedges' g (SE-to-SD; CI based on independent tanks)	0.54	-1.10 to 2.18	Higher measured value with hydroxyproline; 95% CI included 0 (imprecise estimate)	Not applicable - effect size estimated; see Notes for subsampling treatment
Cao et al. (2022)	Improved triploid crucian carp (Carassius auratus triploid)	Hydroxyproline supplementation in a plant-protein-based diet	Hyp 0.39% (0.40% crystalline Hyp added)	Hyp 0.09% (control; no crystalline Hyp added)	Serine (% dry matter)	2.86 ± 0.26	2.74 ± 0.16	3	3	Hedges' g (SE-to-SD; CI based on independent tanks)	0.45	-1.18 to 2.08	Higher measured value with hydroxyproline; 95% CI included 0 (imprecise estimate)	Not applicable - effect size estimated; see Notes for subsampling treatment
Cao et al. (2022)	Improved triploid crucian carp (Carassius auratus triploid)	Hydroxyproline supplementation in a plant-protein-based diet	Hyp 0.39% (0.40% crystalline Hyp added)	Hyp 0.09% (control; no crystalline Hyp added)	Glutamic acid (% dry matter)	10.19 ± 1.00	9.74 ± 0.57	3	3	Hedges' g (SE-to-SD; CI based on independent tanks)	0.44	-1.19 to 2.07	Higher measured value with hydroxyproline; 95% CI included 0 (imprecise estimate)	Not applicable - effect size estimated; see Notes for subsampling treatment
Cao et al. (2022)	Improved triploid crucian carp (Carassius auratus triploid)	Hydroxyproline supplementation in a plant-protein-based diet	Hyp 0.39% (0.40% crystalline Hyp added)	Hyp 0.09% (control; no crystalline Hyp added)	Glycine (% dry matter)	2.95 ± 0.24	2.93 ± 0.10	3	3	Hedges' g (SE-to-SD; CI based on independent tanks)	0.09	-1.52 to 1.69	Higher measured value with hydroxyproline; 95% CI included 0 (imprecise estimate)	Not applicable - effect size estimated; see Notes for subsampling treatment

Study	Species	Strategy	Intervention	Comparator	Outcome and unit	Intervention mean ± SD	Comparator mean ± SD	n intervention (independent tanks)	n comparator (independent tanks)	Effect-size metric	Effect size	95% CI	Interpretation	Reason not estimated
Cao et al. (2022)	Improved triploid crucian carp (Carassius auratus triploid)	Hydroxyproline supplementation in a plant-protein-based diet	Hyp 0.39% (0.40% crystalline Hyp added)	Hyp 0.09% (control; no crystalline Hyp added)	Alanine (% dry matter)	3.45 ± 0.35	3.27 ± 0.17	3	3	Hedges' g (SE-to-SD; CI based on independent tanks)	0.53	-1.12 to 2.17	Higher measured value with hydroxyproline; 95% CI included 0 (imprecise estimate)	Not applicable - effect size estimated; see Notes for subsampling treatment
Cao et al. (2022)	Improved triploid crucian carp (Carassius auratus triploid)	Hydroxyproline supplementation in a plant-protein-based diet	Hyp 0.39% (0.40% crystalline Hyp added)	Hyp 0.09% (control; no crystalline Hyp added)	Tyrosine (% dry matter)	2.27 ± 0.19	2.15 ± 0.14	3	3	Hedges' g (SE-to-SD; CI based on independent tanks)	0.58	-1.07 to 2.23	Higher measured value with hydroxyproline; 95% CI included 0 (imprecise estimate)	Not applicable - effect size estimated; see Notes for subsampling treatment
Cao et al. (2022)	Improved triploid crucian carp (Carassius auratus triploid)	Hydroxyproline supplementation in a plant-protein-based diet	Hyp 0.39% (0.40% crystalline Hyp added)	Hyp 0.09% (control; no crystalline Hyp added)	Proline (% dry matter)	2.09 ± 0.14	2.10 ± 0.09	3	3	Hedges' g (SE-to-SD; CI based on independent tanks)	-0.07	-1.67 to 1.53	Lower measured value with hydroxyproline; 95% CI included 0 (imprecise estimate)	Not applicable - effect size estimated; see Notes for subsampling treatment
Cao et al. (2022)	Improved triploid crucian carp (Carassius auratus triploid)	Hydroxyproline supplementation in a plant-protein-based diet	Hyp 0.39% (0.40% crystalline Hyp added)	Hyp 0.09% (control; no crystalline Hyp added)	Hardness (N)	4.11 ± 0.72	3.67 ± 0.51	3	3	Hedges' g (SE-to-SD; CI based on independent tanks)	0.56	-1.08 to 2.21	Higher, more favorable value with hydroxyproline; 95% CI included 0 (imprecise estimate)	Not applicable - effect size estimated; see Notes for subsampling treatment
Cao et al. (2022)	Improved triploid crucian carp (Carassius auratus triploid)	Hydroxyproline supplementation in a plant-protein-based diet	Hyp 0.39% (0.40% crystalline Hyp added)	Hyp 0.09% (control; no crystalline Hyp added)	Springiness (mm)	0.540 ± 0.090	0.510 ± 0.120	3	3	Hedges' g (SE-to-SD; CI based on independent tanks)	0.23	-1.38 to 1.83	Higher, more favorable value with hydroxyproline; 95% CI included 0 (imprecise estimate)	Not applicable - effect size estimated; see Notes for subsampling treatment
Cao et al. (2022)	Improved triploid crucian carp (Carassius auratus triploid)	Hydroxyproline supplementation in a plant-protein-based diet	Hyp 0.39% (0.40% crystalline Hyp added)	Hyp 0.09% (control; no crystalline Hyp added)	Chewiness (mJ)	0.600 ± 0.240	0.600 ± 0.210	3	3	Hedges' g (SE-to-SD; CI based on independent tanks)	0.0	-1.60 to 1.60	No observed mean difference; 95% CI included 0 (imprecise estimate)	Not applicable - effect size estimated; see Notes for subsampling treatment
Cao et al. (2022)	Improved triploid crucian carp (Carassius auratus triploid)	Hydroxyproline supplementation in a plant-protein-based diet	Hyp 0.39% (0.40% crystalline Hyp added)	Hyp 0.09% (control; no crystalline Hyp added)	Adhesiveness (N·mm)	0.032 ± 0.015	0.031 ± 0.012	3	3	Hedges' g (SE-to-SD; CI based on independent tanks)	0.06	-1.54 to 1.66	Higher measured value with hydroxyproline; 95% CI included 0 (imprecise estimate)	Not applicable - effect size estimated; see Notes for subsampling treatment
Cao et al. (2022)	Improved triploid crucian carp (Carassius auratus triploid)	Hydroxyproline supplementation in a plant-protein-based diet	Hyp 0.39% (0.40% crystalline Hyp added)	Hyp 0.09% (control; no crystalline Hyp added)	Cohesiveness (%)	0.370 ± 0.090	0.380 ± 0.090	3	3	Hedges' g (SE-to-SD; CI based on independent tanks)	-0.09	-1.69 to 1.51	Lower measured value with hydroxyproline; 95% CI included 0 (imprecise estimate)	Not applicable - effect size estimated; see Notes for subsampling treatment
Cao et al. (2022)	Improved triploid crucian carp (Carassius auratus triploid)	Hydroxyproline supplementation in a plant-protein-based diet	Hyp 0.39% (0.40% crystalline Hyp added)	Hyp 0.09% (control; no crystalline Hyp added)	Gumminess (g·mm)	1.16 ± 0.42	1.04 ± 0.36	3	3	Hedges' g (SE-to-SD; CI based on independent tanks)	0.25	-1.36 to 1.85	Higher, more favorable value with hydroxyproline; 95% CI included 0 (imprecise estimate)	Not applicable - effect size estimated; see Notes for subsampling treatment
Cao et al. (2022)	Improved triploid crucian carp (Carassius auratus triploid)	Hydroxyproline supplementation in a plant-protein-based diet	Hyp 0.39% (0.40% crystalline Hyp added)	Hyp 0.09% (control; no crystalline Hyp added)	Muscle hydroxyproline (mg/g)	1.37 ± 0.02	0.910 ± 0.245	3	3	Hedges' g (SE-to-SD; CI based on independent tanks)	2.11	-0.06 to 4.28	Higher, more favorable value with hydroxyproline; 95% CI included 0 (imprecise estimate)	Not applicable - effect size estimated; see Notes for subsampling treatment
Cao et al. (2022)	Improved triploid crucian carp (Carassius auratus triploid)	Hydroxyproline supplementation in a plant-protein-based diet	Hyp 0.39% (0.40% crystalline Hyp added)	Hyp 0.09% (control; no crystalline Hyp added)	Skin hydroxyproline (mg/g)	10.33 ± 0.76	8.77 ± 2.28	3	3	Hedges' g (SE-to-SD; CI based on independent tanks)	0.74	-0.94 to 2.41	Higher, more favorable value with hydroxyproline; 95% CI included 0 (imprecise estimate)	Not applicable - effect size estimated; see Notes for subsampling treatment
Cao et al. (2022)	Improved triploid crucian carp (Carassius auratus triploid)	Hydroxyproline supplementation in a plant-protein-based diet	Hyp 0.39% (0.40% crystalline Hyp added)	Hyp 0.09% (control; no crystalline Hyp added)	Liver hydroxyproline (μg/g)	553.99 ± 177.81	256.88 ± 13.94	3	3	Hedges' g (SE-to-SD; CI based on independent tanks)	1.88	-0.18 to 3.95	Higher, more favorable value with hydroxyproline; 95% CI included 0 (imprecise estimate)	Not applicable - effect size estimated; see Notes for subsampling treatment
Cao et al. (2022)	Improved triploid crucian carp (Carassius auratus triploid)	Hydroxyproline supplementation in a plant-protein-based diet	Hyp 0.39% (0.40% crystalline Hyp added)	Hyp 0.09% (control; no crystalline Hyp added)	Vertebral hydroxyproline (mg/g)	7.62 ± 1.20	7.39 ± 0.42	3	3	Hedges' g (SE-to-SD; CI based on independent tanks)	0.2	-1.40 to 1.81	Higher, more favorable value with hydroxyproline; 95% CI included 0 (imprecise estimate)	Not applicable - effect size estimated; see Notes for subsampling treatment
Cao et al. (2022)	Improved triploid crucian carp (Carassius auratus triploid)	Hydroxyproline supplementation in a plant-protein-based diet	Hyp 0.39% (0.40% crystalline Hyp added)	Hyp 0.09% (control; no crystalline Hyp added)	Plasma hydroxyproline (mg/mL)	119.84 ± 18.54	100.00 ± 23.56	3	3	Hedges' g (SE-to-SD; CI based on independent tanks)	0.75	-0.93 to 2.43	Higher, more favorable value with hydroxyproline; 95% CI included 0 (imprecise estimate)	Not applicable - effect size estimated; see Notes for subsampling treatment
Cao et al. (2022)	Improved triploid crucian carp (Carassius auratus triploid)	Hydroxyproline supplementation in a plant-protein-based diet	Hyp 0.39% (0.40% crystalline Hyp added)	Hyp 0.09% (control; no crystalline Hyp added)	Type I collagen (ng/mg protein)	5.53 ± 0.88	4.88 ± 0.05	3	3	Hedges' g (SE-to-SD; CI based on independent tanks)	0.83	-0.87 to 2.53	Higher, more favorable value with hydroxyproline; 95% CI included 0 (imprecise estimate)	Not applicable - effect size estimated; see Notes for subsampling treatment
Cao et al. (2022)	Improved triploid crucian carp (Carassius auratus triploid)	Hydroxyproline supplementation in a plant-protein-based diet	Hyp 0.39% (0.40% crystalline Hyp added)	Hyp 0.09% (control; no crystalline Hyp added)	Pyridinium crosslink (ng/mg protein)	28.76 ± 1.47	25.74 ± 2.60	3	3	Hedges' g (SE-to-SD; CI based on independent tanks)	1.15	-0.64 to 2.93	Higher, more favorable value with hydroxyproline; 95% CI included 0 (imprecise estimate)	Not applicable - effect size estimated; see Notes for subsampling treatment
Cao et al. (2022)	Improved triploid crucian carp (Carassius auratus triploid)	Hydroxyproline supplementation in a plant-protein-based diet	Hyp 0.39% (0.40% crystalline Hyp added)	Hyp 0.09% (control; no crystalline Hyp added)	Prolyl 4-hydroxylase activity (ng/mg protein)	0.740 ± 0.073	0.660 ± 0.073	3	3	Hedges' g (SE-to-SD; CI based on independent tanks)	0.87	-0.84 to 2.58	Higher, more favorable value with hydroxyproline; 95% CI included 0 (imprecise estimate)	Not applicable - effect size estimated; see Notes for subsampling treatment
Cao et al. (2022)	Improved triploid crucian carp (Carassius auratus triploid)	Hydroxyproline supplementation in a plant-protein-based diet	Hyp 0.39% (0.40% crystalline Hyp added)	Hyp 0.09% (control; no crystalline Hyp added)	Lysyl hydroxylase activity (ng/mg protein)	0.142 ± 0.007	0.136 ± 0.012	3	3	Hedges' g (SE-to-SD; CI based on independent tanks)	0.48	-1.16 to 2.11	Higher, more favorable value with hydroxyproline; 95% CI included 0 (imprecise estimate)	Not applicable - effect size estimated; see Notes for subsampling treatment
Cao et al. (2022)	Improved triploid crucian carp (Carassius auratus triploid)	Hydroxyproline supplementation in a plant-protein-based diet	Hyp 0.39% (0.40% crystalline Hyp added)	Hyp 0.09% (control; no crystalline Hyp added)	Muscle fiber diameter (μm)	55.84 ± 4.85	47.49 ± 4.75	3	3	Hedges' g (SE-to-SD; CI based on independent tanks)	1.39	-0.48 to 3.26	Higher, more favorable value with hydroxyproline; 95% CI included 0 (imprecise estimate)	Not applicable - effect size estimated; see Notes for subsampling treatment
Cao et al. (2022)	Improved triploid crucian carp (Carassius auratus triploid)	Hydroxyproline supplementation in a plant-protein-based diet	Hyp 0.39% (0.40% crystalline Hyp added)	Hyp 0.09% (control; no crystalline Hyp added)	Muscle fiber density (n/mm^2)	175.00 ± 17.37	158.67 ± 15.77	3	3	Hedges' g (SE-to-SD; CI based on independent tanks)	0.79	-0.90 to 2.48	Higher, more favorable value with hydroxyproline; 95% CI included 0 (imprecise estimate)	Not applicable - effect size estimated; see Notes for subsampling treatment
Cao et al. (2022)	Improved triploid crucian carp (Carassius auratus triploid)	Hydroxyproline supplementation in a plant-protein-based diet	Hyp 0.76% (0.80% crystalline Hyp added)	Hyp 0.09% (control; no crystalline Hyp added)	Final body weight (g)	46.79 ± 1.07	41.94 ± 1.85	3	3	Hedges' g (SE-to-SD; CI based on independent tanks)	2.56	0.17 to 4.95	Higher, more favorable value with hydroxyproline; 95% CI excluded 0	Not applicable - effect size estimated; see Notes for subsampling treatment
Cao et al. (2022)	Improved triploid crucian carp (Carassius auratus triploid)	Hydroxyproline supplementation in a plant-protein-based diet	Hyp 0.76% (0.80% crystalline Hyp added)	Hyp 0.09% (control; no crystalline Hyp added)	Weight gain rate (%)	212.10 ± 6.13	179.76 ± 13.48	3	3	Hedges' g (SE-to-SD; CI based on independent tanks)	2.47	0.13 to 4.82	Higher, more favorable value with hydroxyproline; 95% CI excluded 0	Not applicable - effect size estimated; see Notes for subsampling treatment
Cao et al. (2022)	Improved triploid crucian carp (Carassius auratus triploid)	Hydroxyproline supplementation in a plant-protein-based diet	Hyp 0.76% (0.80% crystalline Hyp added)	Hyp 0.09% (control; no crystalline Hyp added)	Specific growth rate (%/day)	2.03 ± 0.03	1.84 ± 0.09	3	3	Hedges' g (SE-to-SD; CI based on independent tanks)	2.3	0.04 to 4.57	Higher, more favorable value with hydroxyproline; 95% CI excluded 0	Not applicable - effect size estimated; see Notes for subsampling treatment
Cao et al. (2022)	Improved triploid crucian carp (Carassius auratus triploid)	Hydroxyproline supplementation in a plant-protein-based diet	Hyp 0.76% (0.80% crystalline Hyp added)	Hyp 0.09% (control; no crystalline Hyp added)	Feed efficiency (%)	52.68 ± 1.49	44.60 ± 3.00	3	3	Hedges' g (SE-to-SD; CI based on independent tanks)	2.73	0.25 to 5.21	Higher, more favorable value with hydroxyproline; 95% CI excluded 0	Not applicable - effect size estimated; see Notes for subsampling treatment
Cao et al. (2022)	Improved triploid crucian carp (Carassius auratus triploid)	Hydroxyproline supplementation in a plant-protein-based diet	Hyp 0.76% (0.80% crystalline Hyp added)	Hyp 0.09% (control; no crystalline Hyp added)	Feeding rate (% body weight/day)	3.26 ± 0.05	3.54 ± 0.10	3	3	Hedges' g (SE-to-SD; CI based on independent tanks)	2.73	0.25 to 5.20	Lower, more favorable value with hydroxyproline; 95% CI excluded 0	Not applicable - effect size estimated; see Notes for subsampling treatment
Cao et al. (2022)	Improved triploid crucian carp (Carassius auratus triploid)	Hydroxyproline supplementation in a plant-protein-based diet	Hyp 0.76% (0.80% crystalline Hyp added)	Hyp 0.09% (control; no crystalline Hyp added)	Protein efficiency ratio (%)	154.97 ± 4.38	128.49 ± 8.61	3	3	Hedges' g (SE-to-SD; CI based on independent tanks)	3.1	0.42 to 5.78	Higher, more favorable value with hydroxyproline; 95% CI excluded 0	Not applicable - effect size estimated; see Notes for subsampling treatment
Cao et al. (2022)	Improved triploid crucian carp (Carassius auratus triploid)	Hydroxyproline supplementation in a plant-protein-based diet	Hyp 0.76% (0.80% crystalline Hyp added)	Hyp 0.09% (control; no crystalline Hyp added)	Nitrogen retention efficiency (%)	27.05 ± 1.89	22.71 ± 1.47	3	3	Hedges' g (SE-to-SD; CI based on independent tanks)	2.05	-0.09 to 4.19	Higher, more favorable value with hydroxyproline; 95% CI included 0 (imprecise estimate)	Not applicable - effect size estimated; see Notes for subsampling treatment
Cao et al. (2022)	Improved triploid crucian carp (Carassius auratus triploid)	Hydroxyproline supplementation in a plant-protein-based diet	Hyp 0.76% (0.80% crystalline Hyp added)	Hyp 0.09% (control; no crystalline Hyp added)	Total nitrogen waste output (g/kg weight gain)	38.77 ± 1.23	45.57 ± 1.59	3	3	Hedges' g (SE-to-SD; CI based on independent tanks)	3.82	0.73 to 6.92	Lower, more favorable value with hydroxyproline; 95% CI excluded 0	Not applicable - effect size estimated; see Notes for subsampling treatment
Cao et al. (2022)	Improved triploid crucian carp (Carassius auratus triploid)	Hydroxyproline supplementation in a plant-protein-based diet	Hyp 0.76% (0.80% crystalline Hyp added)	Hyp 0.09% (control; no crystalline Hyp added)	Survival rate (%)	100.00 ± 0.00	100.00 ± 0.00	3	3	Not estimable	Not estimable	Not estimable	No observed mean difference and no within-group variance	Both groups had zero reported SE; standardized effect size is undefined
Cao et al. (2022)	Improved triploid crucian carp (Carassius auratus triploid)	Hydroxyproline supplementation in a plant-protein-based diet	Hyp 0.76% (0.80% crystalline Hyp added)	Hyp 0.09% (control; no crystalline Hyp added)	Whole-body crude protein (% fresh weight)	16.35 ± 1.23	15.72 ± 0.57	3	3	Hedges' g (SE-to-SD; CI based on independent tanks)	0.53	-1.12 to 2.17	Higher measured value with hydroxyproline; 95% CI included 0 (imprecise estimate)	Not applicable - effect size estimated; see Notes for subsampling treatment
Cao et al. (2022)	Improved triploid crucian carp (Carassius auratus triploid)	Hydroxyproline supplementation in a plant-protein-based diet	Hyp 0.76% (0.80% crystalline Hyp added)	Hyp 0.09% (control; no crystalline Hyp added)	Whole-body crude lipid (% fresh weight)	12.10 ± 0.95	11.04 ± 0.87	3	3	Hedges' g (SE-to-SD; CI based on independent tanks)	0.93	-0.79 to 2.66	Higher measured value with hydroxyproline; 95% CI included 0 (imprecise estimate)	Not applicable - effect size estimated; see Notes for subsampling treatment

Study	Species	Strategy	Intervention	Comparator	Outcome and unit	Intervention mean ± SD	Comparator mean ± SD	n intervention (independent tanks)	n comparator (independent tanks)	Effect-size metric	Effect size	95% CI	Interpretation	Reason not estimated
Cao et al. (2022)	Improved triploid crucian carp (Carassius auratus triploid)	Hydroxyproline supplementation in a plant-protein-based diet	Hyp 0.76% (0.80% crystalline Hyp added)	Hyp 0.09% (control; no crystalline Hyp added)	Whole-body ash (% fresh weight)	2.52 ± 0.12	2.85 ± 0.28	3	3	Hedges' g (SE-to-SD; CI based on independent tanks)	-1.23	-3.05 to 0.58	Lower measured value with hydroxyproline; 95% CI included 0 (imprecise estimate)	Not applicable - effect size estimated; see Notes for subsampling treatment
Cao et al. (2022)	Improved triploid crucian carp (Carassius auratus triploid)	Hydroxyproline supplementation in a plant-protein-based diet	Hyp 0.76% (0.80% crystalline Hyp added)	Hyp 0.09% (control; no crystalline Hyp added)	Whole-body moisture (% fresh weight)	68.22 ± 0.66	69.85 ± 0.42	3	3	Hedges' g (SE-to-SD; CI based on independent tanks)	-2.37	-4.66 to -0.08	Lower measured value with hydroxyproline; 95% CI excluded 0	Not applicable - effect size estimated; see Notes for subsampling treatment
Cao et al. (2022)	Improved triploid crucian carp (Carassius auratus triploid)	Hydroxyproline supplementation in a plant-protein-based diet	Hyp 0.76% (0.80% crystalline Hyp added)	Hyp 0.09% (control; no crystalline Hyp added)	Dorsal-muscle crude protein (% fresh weight)	18.55 ± 1.26	17.06 ± 0.21	3	3	Hedges' g (SE-to-SD; CI based on independent tanks)	1.32	-0.53 to 3.16	Higher measured value with hydroxyproline; 95% CI included 0 (imprecise estimate)	Not applicable - effect size estimated; see Notes for subsampling treatment
Cao et al. (2022)	Improved triploid crucian carp (Carassius auratus triploid)	Hydroxyproline supplementation in a plant-protein-based diet	Hyp 0.76% (0.80% crystalline Hyp added)	Hyp 0.09% (control; no crystalline Hyp added)	Dorsal-muscle crude lipid (% fresh weight)	1.28 ± 0.10	1.32 ± 0.12	3	3	Hedges' g (SE-to-SD; CI based on independent tanks)	-0.28	-1.90 to 1.33	Lower measured value with hydroxyproline; 95% CI included 0 (imprecise estimate)	Not applicable - effect size estimated; see Notes for subsampling treatment
Cao et al. (2022)	Improved triploid crucian carp (Carassius auratus triploid)	Hydroxyproline supplementation in a plant-protein-based diet	Hyp 0.76% (0.80% crystalline Hyp added)	Hyp 0.09% (control; no crystalline Hyp added)	Dorsal-muscle ash (% fresh weight)	1.42 ± 0.07	1.44 ± 0.10	3	3	Hedges' g (SE-to-SD; CI based on independent tanks)	-0.18	-1.79 to 1.42	Lower measured value with hydroxyproline; 95% CI included 0 (imprecise estimate)	Not applicable - effect size estimated; see Notes for subsampling treatment
Cao et al. (2022)	Improved triploid crucian carp (Carassius auratus triploid)	Hydroxyproline supplementation in a plant-protein-based diet	Hyp 0.76% (0.80% crystalline Hyp added)	Hyp 0.09% (control; no crystalline Hyp added)	Dorsal-muscle moisture (% fresh weight)	76.00 ± 0.23	75.65 ± 0.16	3	3	Hedges' g (SE-to-SD; CI based on independent tanks)	1.45	-0.44 to 3.33	Higher measured value with hydroxyproline; 95% CI included 0 (imprecise estimate)	Not applicable - effect size estimated; see Notes for subsampling treatment
Cao et al. (2022)	Improved triploid crucian carp (Carassius auratus triploid)	Hydroxyproline supplementation in a plant-protein-based diet	Hyp 0.76% (0.80% crystalline Hyp added)	Hyp 0.09% (control; no crystalline Hyp added)	Threonine (% dry matter)	3.01 ± 0.14	2.62 ± 0.16	3	3	Hedges' g (SE-to-SD; CI based on independent tanks)	2.12	-0.05 to 4.29	Higher measured value with hydroxyproline; 95% CI included 0 (imprecise estimate)	Not applicable - effect size estimated; see Notes for subsampling treatment
Cao et al. (2022)	Improved triploid crucian carp (Carassius auratus triploid)	Hydroxyproline supplementation in a plant-protein-based diet	Hyp 0.76% (0.80% crystalline Hyp added)	Hyp 0.09% (control; no crystalline Hyp added)	Phenylalanine (% dry matter)	3.29 ± 0.21	2.95 ± 0.19	3	3	Hedges' g (SE-to-SD; CI based on independent tanks)	1.36	-0.49 to 3.22	Higher measured value with hydroxyproline; 95% CI included 0 (imprecise estimate)	Not applicable - effect size estimated; see Notes for subsampling treatment
Cao et al. (2022)	Improved triploid crucian carp (Carassius auratus triploid)	Hydroxyproline supplementation in a plant-protein-based diet	Hyp 0.76% (0.80% crystalline Hyp added)	Hyp 0.09% (control; no crystalline Hyp added)	Valine (% dry matter)	3.27 ± 0.17	2.98 ± 0.19	3	3	Hedges' g (SE-to-SD; CI based on independent tanks)	1.27	-0.55 to 3.10	Higher measured value with hydroxyproline; 95% CI included 0 (imprecise estimate)	Not applicable - effect size estimated; see Notes for subsampling treatment
Cao et al. (2022)	Improved triploid crucian carp (Carassius auratus triploid)	Hydroxyproline supplementation in a plant-protein-based diet	Hyp 0.76% (0.80% crystalline Hyp added)	Hyp 0.09% (control; no crystalline Hyp added)	Isoleucine (% dry matter)	3.37 ± 0.19	3.03 ± 0.17	3	3	Hedges' g (SE-to-SD; CI based on independent tanks)	1.49	-0.41 to 3.40	Higher measured value with hydroxyproline; 95% CI included 0 (imprecise estimate)	Not applicable - effect size estimated; see Notes for subsampling treatment
Cao et al. (2022)	Improved triploid crucian carp (Carassius auratus triploid)	Hydroxyproline supplementation in a plant-protein-based diet	Hyp 0.76% (0.80% crystalline Hyp added)	Hyp 0.09% (control; no crystalline Hyp added)	Leucine (% dry matter)	5.96 ± 0.31	5.36 ± 0.31	3	3	Hedges' g (SE-to-SD; CI based on independent tanks)	1.54	-0.38 to 3.46	Higher measured value with hydroxyproline; 95% CI included 0 (imprecise estimate)	Not applicable - effect size estimated; see Notes for subsampling treatment
Cao et al. (2022)	Improved triploid crucian carp (Carassius auratus triploid)	Hydroxyproline supplementation in a plant-protein-based diet	Hyp 0.76% (0.80% crystalline Hyp added)	Hyp 0.09% (control; no crystalline Hyp added)	Methionine (% dry matter)	2.05 ± 0.10	1.72 ± 0.05	3	3	Hedges' g (SE-to-SD; CI based on independent tanks)	3.21	0.47 to 5.96	Higher measured value with hydroxyproline; 95% CI excluded 0	Not applicable - effect size estimated; see Notes for subsampling treatment
Cao et al. (2022)	Improved triploid crucian carp (Carassius auratus triploid)	Hydroxyproline supplementation in a plant-protein-based diet	Hyp 0.76% (0.80% crystalline Hyp added)	Hyp 0.09% (control; no crystalline Hyp added)	Arginine (% dry matter)	4.12 ± 0.21	3.67 ± 0.23	3	3	Hedges' g (SE-to-SD; CI based on independent tanks)	1.66	-0.31 to 3.63	Higher measured value with hydroxyproline; 95% CI included 0 (imprecise estimate)	Not applicable - effect size estimated; see Notes for subsampling treatment
Cao et al. (2022)	Improved triploid crucian carp (Carassius auratus triploid)	Hydroxyproline supplementation in a plant-protein-based diet	Hyp 0.76% (0.80% crystalline Hyp added)	Hyp 0.09% (control; no crystalline Hyp added)	Lysine (% dry matter)	7.19 ± 0.71	6.01 ± 0.50	3	3	Hedges' g (SE-to-SD; CI based on independent tanks)	1.53	-0.39 to 3.46	Higher measured value with hydroxyproline; 95% CI included 0 (imprecise estimate)	Not applicable - effect size estimated; see Notes for subsampling treatment
Cao et al. (2022)	Improved triploid crucian carp (Carassius auratus triploid)	Hydroxyproline supplementation in a plant-protein-based diet	Hyp 0.76% (0.80% crystalline Hyp added)	Hyp 0.09% (control; no crystalline Hyp added)	Histidine (% dry matter)	3.51 ± 0.17	3.21 ± 0.24	3	3	Hedges' g (SE-to-SD; CI based on independent tanks)	1.14	-0.65 to 2.92	Higher measured value with hydroxyproline; 95% CI included 0 (imprecise estimate)	Not applicable - effect size estimated; see Notes for subsampling treatment
Cao et al. (2022)	Improved triploid crucian carp (Carassius auratus triploid)	Hydroxyproline supplementation in a plant-protein-based diet	Hyp 0.76% (0.80% crystalline Hyp added)	Hyp 0.09% (control; no crystalline Hyp added)	Aspartic acid (% dry matter)	7.58 ± 0.42	6.84 ± 0.43	3	3	Hedges' g (SE-to-SD; CI based on independent tanks)	1.39	-0.47 to 3.26	Higher measured value with hydroxyproline; 95% CI included 0 (imprecise estimate)	Not applicable - effect size estimated; see Notes for subsampling treatment
Cao et al. (2022)	Improved triploid crucian carp (Carassius auratus triploid)	Hydroxyproline supplementation in a plant-protein-based diet	Hyp 0.76% (0.80% crystalline Hyp added)	Hyp 0.09% (control; no crystalline Hyp added)	Serine (% dry matter)	3.00 ± 0.14	2.74 ± 0.16	3	3	Hedges' g (SE-to-SD; CI based on independent tanks)	1.41	-0.46 to 3.29	Higher measured value with hydroxyproline; 95% CI included 0 (imprecise estimate)	Not applicable - effect size estimated; see Notes for subsampling treatment
Cao et al. (2022)	Improved triploid crucian carp (Carassius auratus triploid)	Hydroxyproline supplementation in a plant-protein-based diet	Hyp 0.76% (0.80% crystalline Hyp added)	Hyp 0.09% (control; no crystalline Hyp added)	Glutamic acid (% dry matter)	10.75 ± 0.57	9.74 ± 0.57	3	3	Hedges' g (SE-to-SD; CI based on independent tanks)	1.41	-0.46 to 3.29	Higher measured value with hydroxyproline; 95% CI included 0 (imprecise estimate)	Not applicable - effect size estimated; see Notes for subsampling treatment
Cao et al. (2022)	Improved triploid crucian carp (Carassius auratus triploid)	Hydroxyproline supplementation in a plant-protein-based diet	Hyp 0.76% (0.80% crystalline Hyp added)	Hyp 0.09% (control; no crystalline Hyp added)	Glycine (% dry matter)	3.07 ± 0.09	2.93 ± 0.10	3	3	Hedges' g (SE-to-SD; CI based on independent tanks)	1.17	-0.62 to 2.97	Higher measured value with hydroxyproline; 95% CI included 0 (imprecise estimate)	Not applicable - effect size estimated; see Notes for subsampling treatment
Cao et al. (2022)	Improved triploid crucian carp (Carassius auratus triploid)	Hydroxyproline supplementation in a plant-protein-based diet	Hyp 0.76% (0.80% crystalline Hyp added)	Hyp 0.09% (control; no crystalline Hyp added)	Alanine (% dry matter)	3.63 ± 0.17	3.27 ± 0.17	3	3	Hedges' g (SE-to-SD; CI based on independent tanks)	1.66	-0.31 to 3.63	Higher measured value with hydroxyproline; 95% CI included 0 (imprecise estimate)	Not applicable - effect size estimated; see Notes for subsampling treatment
Cao et al. (2022)	Improved triploid crucian carp (Carassius auratus triploid)	Hydroxyproline supplementation in a plant-protein-based diet	Hyp 0.76% (0.80% crystalline Hyp added)	Hyp 0.09% (control; no crystalline Hyp added)	Tyrosine (% dry matter)	2.38 ± 0.12	2.15 ± 0.14	3	3	Hedges' g (SE-to-SD; CI based on independent tanks)	1.41	-0.46 to 3.29	Higher measured value with hydroxyproline; 95% CI included 0 (imprecise estimate)	Not applicable - effect size estimated; see Notes for subsampling treatment
Cao et al. (2022)	Improved triploid crucian carp (Carassius auratus triploid)	Hydroxyproline supplementation in a plant-protein-based diet	Hyp 0.76% (0.80% crystalline Hyp added)	Hyp 0.09% (control; no crystalline Hyp added)	Proline (% dry matter)	2.16 ± 0.07	2.10 ± 0.09	3	3	Hedges' g (SE-to-SD; CI based on independent tanks)	0.61	-1.04 to 2.27	Higher measured value with hydroxyproline; 95% CI included 0 (imprecise estimate)	Not applicable - effect size estimated; see Notes for subsampling treatment
Cao et al. (2022)	Improved triploid crucian carp (Carassius auratus triploid)	Hydroxyproline supplementation in a plant-protein-based diet	Hyp 0.76% (0.80% crystalline Hyp added)	Hyp 0.09% (control; no crystalline Hyp added)	Hardness (N)	3.89 ± 0.24	3.67 ± 0.51	3	3	Hedges' g (SE-to-SD; CI based on independent tanks)	0.44	-1.19 to 2.07	Higher, more favorable value with hydroxyproline; 95% CI included 0 (imprecise estimate)	Not applicable - effect size estimated; see Notes for subsampling treatment
Cao et al. (2022)	Improved triploid crucian carp (Carassius auratus triploid)	Hydroxyproline supplementation in a plant-protein-based diet	Hyp 0.76% (0.80% crystalline Hyp added)	Hyp 0.09% (control; no crystalline Hyp added)	Springiness (mm)	0.630 ± 0.120	0.510 ± 0.120	3	3	Hedges' g (SE-to-SD; CI based on independent tanks)	0.8	-0.89 to 2.49	Higher, more favorable value with hydroxyproline; 95% CI included 0 (imprecise estimate)	Not applicable - effect size estimated; see Notes for subsampling treatment
Cao et al. (2022)	Improved triploid crucian carp (Carassius auratus triploid)	Hydroxyproline supplementation in a plant-protein-based diet	Hyp 0.76% (0.80% crystalline Hyp added)	Hyp 0.09% (control; no crystalline Hyp added)	Chewiness (mJ)	0.680 ± 0.270	0.600 ± 0.210	3	3	Hedges' g (SE-to-SD; CI based on independent tanks)	0.26	-1.35 to 1.88	Higher, more favorable value with hydroxyproline; 95% CI included 0 (imprecise estimate)	Not applicable - effect size estimated; see Notes for subsampling treatment
Cao et al. (2022)	Improved triploid crucian carp (Carassius auratus triploid)	Hydroxyproline supplementation in a plant-protein-based diet	Hyp 0.76% (0.80% crystalline Hyp added)	Hyp 0.09% (control; no crystalline Hyp added)	Adhesiveness (N·mm)	0.037 ± 0.012	0.031 ± 0.012	3	3	Hedges' g (SE-to-SD; CI based on independent tanks)	0.4	-1.22 to 2.02	Higher measured value with hydroxyproline; 95% CI included 0 (imprecise estimate)	Not applicable - effect size estimated; see Notes for subsampling treatment
Cao et al. (2022)	Improved triploid crucian carp (Carassius auratus triploid)	Hydroxyproline supplementation in a plant-protein-based diet	Hyp 0.76% (0.80% crystalline Hyp added)	Hyp 0.09% (control; no crystalline Hyp added)	Cohesiveness (%)	0.390 ± 0.060	0.380 ± 0.090	3	3	Hedges' g (SE-to-SD; CI based on independent tanks)	0.1	-1.50 to 1.71	Higher measured value with hydroxyproline; 95% CI included 0 (imprecise estimate)	Not applicable - effect size estimated; see Notes for subsampling treatment
Cao et al. (2022)	Improved triploid crucian carp (Carassius auratus triploid)	Hydroxyproline supplementation in a plant-protein-based diet	Hyp 0.76% (0.80% crystalline Hyp added)	Hyp 0.09% (control; no crystalline Hyp added)	Gumminess (g·mm)	1.20 ± 0.33	1.04 ± 0.36	3	3	Hedges' g (SE-to-SD; CI based on independent tanks)	0.37	-1.25 to 1.99	Higher, more favorable value with hydroxyproline; 95% CI included 0 (imprecise estimate)	Not applicable - effect size estimated; see Notes for subsampling treatment
Cao et al. (2022)	Improved triploid crucian carp (Carassius auratus triploid)	Hydroxyproline supplementation in a plant-protein-based diet	Hyp 0.76% (0.80% crystalline Hyp added)	Hyp 0.09% (control; no crystalline Hyp added)	Muscle hydroxyproline (mg/g)	1.64 ± 0.47	0.910 ± 0.245	3	3	Hedges' g (SE-to-SD; CI based on independent tanks)	1.57	-0.36 to 3.51	Higher, more favorable value with hydroxyproline; 95% CI included 0 (imprecise estimate)	Not applicable - effect size estimated; see Notes for subsampling treatment
Cao et al. (2022)	Improved triploid crucian carp (Carassius auratus triploid)	Hydroxyproline supplementation in a plant-protein-based diet	Hyp 0.76% (0.80% crystalline Hyp added)	Hyp 0.09% (control; no crystalline Hyp added)	Skin hydroxyproline (mg/g)	13.15 ± 2.69	8.77 ± 2.28	3	3	Hedges' g (SE-to-SD; CI based on independent tanks)	1.4	-0.47 to 3.28	Higher, more favorable value with hydroxyproline; 95% CI included 0 (imprecise estimate)	Not applicable - effect size estimated; see Notes for subsampling treatment
Cao et al. (2022)	Improved triploid crucian carp (Carassius auratus triploid)	Hydroxyproline supplementation in a plant-protein-based diet	Hyp 0.76% (0.80% crystalline Hyp added)	Hyp 0.09% (control; no crystalline Hyp added)	Liver hydroxyproline (μg/g)	671.96 ± 253.38	256.88 ± 13.94	3	3	Hedges' g (SE-to-SD; CI based on independent tanks)	1.85	-0.20 to 3.90	Higher, more favorable value with hydroxyproline; 95% CI included 0 (imprecise estimate)	Not applicable - effect size estimated; see Notes for subsampling treatment

Study	Species	Strategy	Intervention	Comparator	Outcome and unit	Intervention mean ± SD	Comparator mean ± SD	n intervention (independent tanks)	n comparator (independent tanks)	Effect-size metric	Effect size	95% CI	Interpretation	Reason not estimated
Cao et al. (2022)	Improved triploid crucian carp (Carassius auratus triploid)	Hydroxyproline supplementation in a plant-protein-based diet	Hyp 0.76% (0.80% crystalline Hyp added)	Hyp 0.09% (control; no crystalline Hyp added)	Vertebral hydroxyproline (mg/g)	8.45 ± 1.79	7.39 ± 0.42	3	3	Hedges' g (SE-to-SD; CI based on independent tanks)	0.65	-1.01 to 2.32	Higher, more favorable value with hydroxyproline; 95% CI included 0 (imprecise estimate)	Not applicable - effect size estimated; see Notes for subsampling treatment
Cao et al. (2022)	Improved triploid crucian carp (Carassius auratus triploid)	Hydroxyproline supplementation in a plant-protein-based diet	Hyp 0.76% (0.80% crystalline Hyp added)	Hyp 0.09% (control; no crystalline Hyp added)	Plasma hydroxyproline (mg/mL)	124.60 ± 29.42	100.00 ± 23.56	3	3	Hedges' g (SE-to-SD; CI based on independent tanks)	0.74	-0.94 to 2.42	Higher, more favorable value with hydroxyproline; 95% CI included 0 (imprecise estimate)	Not applicable - effect size estimated; see Notes for subsampling treatment
Cao et al. (2022)	Improved triploid crucian carp (Carassius auratus triploid)	Hydroxyproline supplementation in a plant-protein-based diet	Hyp 0.76% (0.80% crystalline Hyp added)	Hyp 0.09% (control; no crystalline Hyp added)	Type I collagen (ng/mg protein)	5.34 ± 1.10	4.88 ± 0.05	3	3	Hedges' g (SE-to-SD; CI based on independent tanks)	0.47	-1.16 to 2.11	Higher, more favorable value with hydroxyproline; 95% CI included 0 (imprecise estimate)	Not applicable - effect size estimated; see Notes for subsampling treatment
Cao et al. (2022)	Improved triploid crucian carp (Carassius auratus triploid)	Hydroxyproline supplementation in a plant-protein-based diet	Hyp 0.76% (0.80% crystalline Hyp added)	Hyp 0.09% (control; no crystalline Hyp added)	Pyridinium crosslink (ng/mg protein)	26.91 ± 3.97	25.74 ± 2.60	3	3	Hedges' g (SE-to-SD; CI based on independent tanks)	0.28	-1.33 to 1.89	Higher, more favorable value with hydroxyproline; 95% CI included 0 (imprecise estimate)	Not applicable - effect size estimated; see Notes for subsampling treatment
Cao et al. (2022)	Improved triploid crucian carp (Carassius auratus triploid)	Hydroxyproline supplementation in a plant-protein-based diet	Hyp 0.76% (0.80% crystalline Hyp added)	Hyp 0.09% (control; no crystalline Hyp added)	Prolyl 4-hydroxylase activity (ng/mg protein)	0.750 ± 0.098	0.660 ± 0.073	3	3	Hedges' g (SE-to-SD; CI based on independent tanks)	0.83	-0.87 to 2.53	Higher, more favorable value with hydroxyproline; 95% CI included 0 (imprecise estimate)	Not applicable - effect size estimated; see Notes for subsampling treatment
Cao et al. (2022)	Improved triploid crucian carp (Carassius auratus triploid)	Hydroxyproline supplementation in a plant-protein-based diet	Hyp 0.76% (0.80% crystalline Hyp added)	Hyp 0.09% (control; no crystalline Hyp added)	Lysyl hydroxylase activity (ng/mg protein)	0.157 ± 0.017	0.136 ± 0.012	3	3	Hedges' g (SE-to-SD; CI based on independent tanks)	1.13	-0.65 to 2.91	Higher, more favorable value with hydroxyproline; 95% CI included 0 (imprecise estimate)	Not applicable - effect size estimated; see Notes for subsampling treatment
Cao et al. (2022)	Improved triploid crucian carp (Carassius auratus triploid)	Hydroxyproline supplementation in a plant-protein-based diet	Hyp 0.76% (0.80% crystalline Hyp added)	Hyp 0.09% (control; no crystalline Hyp added)	Muscle fiber diameter (μm)	61.48 ± 3.33	47.49 ± 4.75	3	3	Hedges' g (SE-to-SD; CI based on independent tanks)	2.73	0.25 to 5.20	Higher, more favorable value with hydroxyproline; 95% CI excluded 0	Not applicable - effect size estimated; see Notes for subsampling treatment
Cao et al. (2022)	Improved triploid crucian carp (Carassius auratus triploid)	Hydroxyproline supplementation in a plant-protein-based diet	Hyp 0.76% (0.80% crystalline Hyp added)	Hyp 0.09% (control; no crystalline Hyp added)	Muscle fiber density (n/mm^2)	221.67 ± 44.09	158.67 ± 15.77	3	3	Hedges' g (SE-to-SD; CI based on independent tanks)	1.52	-0.39 to 3.44	Higher, more favorable value with hydroxyproline; 95% CI included 0 (imprecise estimate)	Not applicable - effect size estimated; see Notes for subsampling treatment
Cao et al. (2022)	Improved triploid crucian carp (Carassius auratus triploid)	Hydroxyproline supplementation in a plant-protein-based diet	Hyp 1.14% (1.20% crystalline Hyp added)	Hyp 0.09% (control; no crystalline Hyp added)	Final body weight (g)	46.58 ± 1.00	41.94 ± 1.85	3	3	Hedges' g (SE-to-SD; CI based on independent tanks)	2.49	0.14 to 4.84	Higher, more favorable value with hydroxyproline; 95% CI excluded 0	Not applicable - effect size estimated; see Notes for subsampling treatment
Cao et al. (2022)	Improved triploid crucian carp (Carassius auratus triploid)	Hydroxyproline supplementation in a plant-protein-based diet	Hyp 1.14% (1.20% crystalline Hyp added)	Hyp 0.09% (control; no crystalline Hyp added)	Weight gain rate (%)	209.49 ± 7.10	179.76 ± 13.48	3	3	Hedges' g (SE-to-SD; CI based on independent tanks)	2.21	-0.01 to 4.42	Higher, more favorable value with hydroxyproline; 95% CI included 0 (imprecise estimate)	Not applicable - effect size estimated; see Notes for subsampling treatment
Cao et al. (2022)	Improved triploid crucian carp (Carassius auratus triploid)	Hydroxyproline supplementation in a plant-protein-based diet	Hyp 1.14% (1.20% crystalline Hyp added)	Hyp 0.09% (control; no crystalline Hyp added)	Specific growth rate (%/day)	2.02 ± 0.03	1.84 ± 0.09	3	3	Hedges' g (SE-to-SD; CI based on independent tanks)	2.18	-0.02 to 4.39	Higher, more favorable value with hydroxyproline; 95% CI included 0 (imprecise estimate)	Not applicable - effect size estimated; see Notes for subsampling treatment
Cao et al. (2022)	Improved triploid crucian carp (Carassius auratus triploid)	Hydroxyproline supplementation in a plant-protein-based diet	Hyp 1.14% (1.20% crystalline Hyp added)	Hyp 0.09% (control; no crystalline Hyp added)	Feed efficiency (%)	52.43 ± 1.82	44.60 ± 3.00	3	3	Hedges' g (SE-to-SD; CI based on independent tanks)	2.53	0.15 to 4.90	Higher, more favorable value with hydroxyproline; 95% CI excluded 0	Not applicable - effect size estimated; see Notes for subsampling treatment
Cao et al. (2022)	Improved triploid crucian carp (Carassius auratus triploid)	Hydroxyproline supplementation in a plant-protein-based diet	Hyp 1.14% (1.20% crystalline Hyp added)	Hyp 0.09% (control; no crystalline Hyp added)	Feeding rate (% body weight/day)	3.25 ± 0.07	3.54 ± 0.10	3	3	Hedges' g (SE-to-SD; CI based on independent tanks)	2.63	0.20 to 5.05	Lower, more favorable value with hydroxyproline; 95% CI excluded 0	Not applicable - effect size estimated; see Notes for subsampling treatment
Cao et al. (2022)	Improved triploid crucian carp (Carassius auratus triploid)	Hydroxyproline supplementation in a plant-protein-based diet	Hyp 1.14% (1.20% crystalline Hyp added)	Hyp 0.09% (control; no crystalline Hyp added)	Protein efficiency ratio (%)	153.57 ± 5.33	128.49 ± 8.61	3	3	Hedges' g (SE-to-SD; CI based on independent tanks)	2.8	0.29 to 5.32	Higher, more favorable value with hydroxyproline; 95% CI excluded 0	Not applicable - effect size estimated; see Notes for subsampling treatment
Cao et al. (2022)	Improved triploid crucian carp (Carassius auratus triploid)	Hydroxyproline supplementation in a plant-protein-based diet	Hyp 1.14% (1.20% crystalline Hyp added)	Hyp 0.09% (control; no crystalline Hyp added)	Nitrogen retention efficiency (%)	25.63 ± 1.45	22.71 ± 1.47	3	3	Hedges' g (SE-to-SD; CI based on independent tanks)	1.6	-0.35 to 3.54	Higher, more favorable value with hydroxyproline; 95% CI included 0 (imprecise estimate)	Not applicable - effect size estimated; see Notes for subsampling treatment
Cao et al. (2022)	Improved triploid crucian carp (Carassius auratus triploid)	Hydroxyproline supplementation in a plant-protein-based diet	Hyp 1.14% (1.20% crystalline Hyp added)	Hyp 0.09% (control; no crystalline Hyp added)	Total nitrogen waste output (g/kg weight gain)	39.66 ± 1.51	45.57 ± 1.59	3	3	Hedges' g (SE-to-SD; CI based on independent tanks)	3.05	0.40 to 5.70	Lower, more favorable value with hydroxyproline; 95% CI excluded 0	Not applicable - effect size estimated; see Notes for subsampling treatment
Cao et al. (2022)	Improved triploid crucian carp (Carassius auratus triploid)	Hydroxyproline supplementation in a plant-protein-based diet	Hyp 1.14% (1.20% crystalline Hyp added)	Hyp 0.09% (control; no crystalline Hyp added)	Survival rate (%)	100.00 ± 0.00	100.00 ± 0.00	3	3	Not estimable	Not estimable	Not estimable	No observed mean difference and no within-group variance	Both groups had zero reported SE; standardized effect size is undefined
Cao et al. (2022)	Improved triploid crucian carp (Carassius auratus triploid)	Hydroxyproline supplementation in a plant-protein-based diet	Hyp 1.14% (1.20% crystalline Hyp added)	Hyp 0.09% (control; no crystalline Hyp added)	Whole-body crude protein (% fresh weight)	16.59 ± 1.02	15.72 ± 0.57	3	3	Hedges' g (SE-to-SD; CI based on independent tanks)	0.84	-0.86 to 2.54	Higher measured value with hydroxyproline; 95% CI included 0 (imprecise estimate)	Not applicable - effect size estimated; see Notes for subsampling treatment
Cao et al. (2022)	Improved triploid crucian carp (Carassius auratus triploid)	Hydroxyproline supplementation in a plant-protein-based diet	Hyp 1.14% (1.20% crystalline Hyp added)	Hyp 0.09% (control; no crystalline Hyp added)	Whole-body crude lipid (% fresh weight)	12.17 ± 2.01	11.04 ± 0.87	3	3	Hedges' g (SE-to-SD; CI based on independent tanks)	0.58	-1.07 to 2.24	Higher measured value with hydroxyproline; 95% CI included 0 (imprecise estimate)	Not applicable - effect size estimated; see Notes for subsampling treatment
Cao et al. (2022)	Improved triploid crucian carp (Carassius auratus triploid)	Hydroxyproline supplementation in a plant-protein-based diet	Hyp 1.14% (1.20% crystalline Hyp added)	Hyp 0.09% (control; no crystalline Hyp added)	Whole-body ash (% fresh weight)	2.71 ± 0.14	2.85 ± 0.28	3	3	Hedges' g (SE-to-SD; CI based on independent tanks)	-0.51	-2.15 to 1.13	Lower measured value with hydroxyproline; 95% CI included 0 (imprecise estimate)	Not applicable - effect size estimated; see Notes for subsampling treatment
Cao et al. (2022)	Improved triploid crucian carp (Carassius auratus triploid)	Hydroxyproline supplementation in a plant-protein-based diet	Hyp 1.14% (1.20% crystalline Hyp added)	Hyp 0.09% (control; no crystalline Hyp added)	Whole-body moisture (% fresh weight)	68.18 ± 1.37	69.85 ± 0.42	3	3	Hedges' g (SE-to-SD; CI based on independent tanks)	-1.32	-3.16 to 0.52	Lower measured value with hydroxyproline; 95% CI included 0 (imprecise estimate)	Not applicable - effect size estimated; see Notes for subsampling treatment
Cao et al. (2022)	Improved triploid crucian carp (Carassius auratus triploid)	Hydroxyproline supplementation in a plant-protein-based diet	Hyp 1.14% (1.20% crystalline Hyp added)	Hyp 0.09% (control; no crystalline Hyp added)	Dorsal-muscle crude protein (% fresh weight)	17.79 ± 0.33	17.06 ± 0.21	3	3	Hedges' g (SE-to-SD; CI based on independent tanks)	2.12	-0.05 to 4.30	Higher measured value with hydroxyproline; 95% CI included 0 (imprecise estimate)	Not applicable - effect size estimated; see Notes for subsampling treatment
Cao et al. (2022)	Improved triploid crucian carp (Carassius auratus triploid)	Hydroxyproline supplementation in a plant-protein-based diet	Hyp 1.14% (1.20% crystalline Hyp added)	Hyp 0.09% (control; no crystalline Hyp added)	Dorsal-muscle crude lipid (% fresh weight)	1.30 ± 0.07	1.32 ± 0.12	3	3	Hedges' g (SE-to-SD; CI based on independent tanks)	-0.16	-1.77 to 1.44	Lower measured value with hydroxyproline; 95% CI included 0 (imprecise estimate)	Not applicable - effect size estimated; see Notes for subsampling treatment
Cao et al. (2022)	Improved triploid crucian carp (Carassius auratus triploid)	Hydroxyproline supplementation in a plant-protein-based diet	Hyp 1.14% (1.20% crystalline Hyp added)	Hyp 0.09% (control; no crystalline Hyp added)	Dorsal-muscle ash (% fresh weight)	1.38 ± 0.21	1.44 ± 0.10	3	3	Hedges' g (SE-to-SD; CI based on independent tanks)	-0.29	-1.91 to 1.32	Lower measured value with hydroxyproline; 95% CI included 0 (imprecise estimate)	Not applicable - effect size estimated; see Notes for subsampling treatment
Cao et al. (2022)	Improved triploid crucian carp (Carassius auratus triploid)	Hydroxyproline supplementation in a plant-protein-based diet	Hyp 1.14% (1.20% crystalline Hyp added)	Hyp 0.09% (control; no crystalline Hyp added)	Dorsal-muscle moisture (% fresh weight)	76.55 ± 0.99	75.65 ± 0.16	3	3	Hedges' g (SE-to-SD; CI based on independent tanks)	1.02	-0.73 to 2.77	Higher measured value with hydroxyproline; 95% CI included 0 (imprecise estimate)	Not applicable - effect size estimated; see Notes for subsampling treatment
Cao et al. (2022)	Improved triploid crucian carp (Carassius auratus triploid)	Hydroxyproline supplementation in a plant-protein-based diet	Hyp 1.14% (1.20% crystalline Hyp added)	Hyp 0.09% (control; no crystalline Hyp added)	Threonine (% dry matter)	2.87 ± 0.05	2.62 ± 0.16	3	3	Hedges' g (SE-to-SD; CI based on independent tanks)	1.72	-0.27 to 3.72	Higher measured value with hydroxyproline; 95% CI included 0 (imprecise estimate)	Not applicable - effect size estimated; see Notes for subsampling treatment
Cao et al. (2022)	Improved triploid crucian carp (Carassius auratus triploid)	Hydroxyproline supplementation in a plant-protein-based diet	Hyp 1.14% (1.20% crystalline Hyp added)	Hyp 0.09% (control; no crystalline Hyp added)	Phenylalanine (% dry matter)	3.24 ± 0.05	2.95 ± 0.19	3	3	Hedges' g (SE-to-SD; CI based on independent tanks)	1.66	-0.31 to 3.63	Higher measured value with hydroxyproline; 95% CI included 0 (imprecise estimate)	Not applicable - effect size estimated; see Notes for subsampling treatment
Cao et al. (2022)	Improved triploid crucian carp (Carassius auratus triploid)	Hydroxyproline supplementation in a plant-protein-based diet	Hyp 1.14% (1.20% crystalline Hyp added)	Hyp 0.09% (control; no crystalline Hyp added)	Valine (% dry matter)	3.19 ± 0.02	2.98 ± 0.19	3	3	Hedges' g (SE-to-SD; CI based on independent tanks)	1.24	-0.58 to 3.06	Higher measured value with hydroxyproline; 95% CI included 0 (imprecise estimate)	Not applicable - effect size estimated; see Notes for subsampling treatment
Cao et al. (2022)	Improved triploid crucian carp (Carassius auratus triploid)	Hydroxyproline supplementation in a plant-protein-based diet	Hyp 1.14% (1.20% crystalline Hyp added)	Hyp 0.09% (control; no crystalline Hyp added)	Isoleucine (% dry matter)	3.29 ± 0.03	3.03 ± 0.17	3	3	Hedges' g (SE-to-SD; CI based on independent tanks)	1.67	-0.31 to 3.64	Higher measured value with hydroxyproline; 95% CI included 0 (imprecise estimate)	Not applicable - effect size estimated; see Notes for subsampling treatment
Cao et al. (2022)	Improved triploid crucian carp (Carassius auratus triploid)	Hydroxyproline supplementation in a plant-protein-based diet	Hyp 1.14% (1.20% crystalline Hyp added)	Hyp 0.09% (control; no crystalline Hyp added)	Leucine (% dry matter)	5.80 ± 0.07	5.36 ± 0.31	3	3	Hedges' g (SE-to-SD; CI based on independent tanks)	1.56	-0.37 to 3.49	Higher measured value with hydroxyproline; 95% CI included 0 (imprecise estimate)	Not applicable - effect size estimated; see Notes for subsampling treatment
Cao et al. (2022)	Improved triploid crucian carp (Carassius auratus triploid)	Hydroxyproline supplementation in a plant-protein-based diet	Hyp 1.14% (1.20% crystalline Hyp added)	Hyp 0.09% (control; no crystalline Hyp added)	Methionine (% dry matter)	1.97 ± 0.03	1.72 ± 0.05	3	3	Hedges' g (SE-to-SD; CI based on independent tanks)	4.53	1.01 to 8.05	Higher measured value with hydroxyproline; 95% CI excluded 0	Not applicable - effect size estimated; see Notes for subsampling treatment

Study	Species	Strategy	Intervention	Comparator	Outcome and unit	Intervention mean ± SD	Comparator mean ± SD	n intervention (independent tanks)	n comparator (independent tanks)	Effect-size metric	Effect size	95% CI	Interpretation	Reason not estimated
Cao et al. (2022)	Improved triploid crucian carp (Carassius auratus triploid)	Hydroxyproline supplementation in a plant-protein-based diet	Hyp 1.14% (1.20% crystalline Hyp added)	Hyp 0.09% (control; no crystalline Hyp added)	Arginine (% dry matter)	4.05 ± 0.07	3.67 ± 0.23	3	3	Hedges' g (SE-to-SD; CI based on independent tanks)	1.82	-0.21 to 3.86	Higher measured value with hydroxyproline; 95% CI included 0 (imprecise estimate)	Not applicable - effect size estimated; see Notes for subsampling treatment
Cao et al. (2022)	Improved triploid crucian carp (Carassius auratus triploid)	Hydroxyproline supplementation in a plant-protein-based diet	Hyp 1.14% (1.20% crystalline Hyp added)	Hyp 0.09% (control; no crystalline Hyp added)	Lysine (% dry matter)	7.23 ± 0.17	6.01 ± 0.50	3	3	Hedges' g (SE-to-SD; CI based on independent tanks)	2.6	0.19 to 5.01	Higher measured value with hydroxyproline; 95% CI excluded 0	Not applicable - effect size estimated; see Notes for subsampling treatment
Cao et al. (2022)	Improved triploid crucian carp (Carassius auratus triploid)	Hydroxyproline supplementation in a plant-protein-based diet	Hyp 1.14% (1.20% crystalline Hyp added)	Hyp 0.09% (control; no crystalline Hyp added)	Histidine (% dry matter)	3.54 ± 0.14	3.21 ± 0.24	3	3	Hedges' g (SE-to-SD; CI based on independent tanks)	1.34	-0.51 to 3.19	Higher measured value with hydroxyproline; 95% CI included 0 (imprecise estimate)	Not applicable - effect size estimated; see Notes for subsampling treatment
Cao et al. (2022)	Improved triploid crucian carp (Carassius auratus triploid)	Hydroxyproline supplementation in a plant-protein-based diet	Hyp 1.14% (1.20% crystalline Hyp added)	Hyp 0.09% (control; no crystalline Hyp added)	Aspartic acid (% dry matter)	7.35 ± 0.12	6.84 ± 0.43	3	3	Hedges' g (SE-to-SD; CI based on independent tanks)	1.28	-0.55 to 3.11	Higher measured value with hydroxyproline; 95% CI included 0 (imprecise estimate)	Not applicable - effect size estimated; see Notes for subsampling treatment
Cao et al. (2022)	Improved triploid crucian carp (Carassius auratus triploid)	Hydroxyproline supplementation in a plant-protein-based diet	Hyp 1.14% (1.20% crystalline Hyp added)	Hyp 0.09% (control; no crystalline Hyp added)	Serine (% dry matter)	2.91 ± 0.03	2.74 ± 0.16	3	3	Hedges' g (SE-to-SD; CI based on independent tanks)	1.2	-0.60 to 3.01	Higher measured value with hydroxyproline; 95% CI included 0 (imprecise estimate)	Not applicable - effect size estimated; see Notes for subsampling treatment
Cao et al. (2022)	Improved triploid crucian carp (Carassius auratus triploid)	Hydroxyproline supplementation in a plant-protein-based diet	Hyp 1.14% (1.20% crystalline Hyp added)	Hyp 0.09% (control; no crystalline Hyp added)	Glutamic acid (% dry matter)	10.41 ± 0.17	9.74 ± 0.57	3	3	Hedges' g (SE-to-SD; CI based on independent tanks)	1.27	-0.56 to 3.10	Higher measured value with hydroxyproline; 95% CI included 0 (imprecise estimate)	Not applicable - effect size estimated; see Notes for subsampling treatment
Cao et al. (2022)	Improved triploid crucian carp (Carassius auratus triploid)	Hydroxyproline supplementation in a plant-protein-based diet	Hyp 1.14% (1.20% crystalline Hyp added)	Hyp 0.09% (control; no crystalline Hyp added)	Glycine (% dry matter)	3.05 ± 0.10	2.93 ± 0.10	3	3	Hedges' g (SE-to-SD; CI based on independent tanks)	0.92	-0.80 to 2.65	Higher measured value with hydroxyproline; 95% CI included 0 (imprecise estimate)	Not applicable - effect size estimated; see Notes for subsampling treatment
Cao et al. (2022)	Improved triploid crucian carp (Carassius auratus triploid)	Hydroxyproline supplementation in a plant-protein-based diet	Hyp 1.14% (1.20% crystalline Hyp added)	Hyp 0.09% (control; no crystalline Hyp added)	Alanine (% dry matter)	3.53 ± 0.07	3.27 ± 0.17	3	3	Hedges' g (SE-to-SD; CI based on independent tanks)	1.58	-0.36 to 3.51	Higher measured value with hydroxyproline; 95% CI included 0 (imprecise estimate)	Not applicable - effect size estimated; see Notes for subsampling treatment
Cao et al. (2022)	Improved triploid crucian carp (Carassius auratus triploid)	Hydroxyproline supplementation in a plant-protein-based diet	Hyp 1.14% (1.20% crystalline Hyp added)	Hyp 0.09% (control; no crystalline Hyp added)	Tyrosine (% dry matter)	2.31 ± 0.02	2.15 ± 0.14	3	3	Hedges' g (SE-to-SD; CI based on independent tanks)	1.3	-0.54 to 3.13	Higher measured value with hydroxyproline; 95% CI included 0 (imprecise estimate)	Not applicable - effect size estimated; see Notes for subsampling treatment
Cao et al. (2022)	Improved triploid crucian carp (Carassius auratus triploid)	Hydroxyproline supplementation in a plant-protein-based diet	Hyp 1.14% (1.20% crystalline Hyp added)	Hyp 0.09% (control; no crystalline Hyp added)	Proline (% dry matter)	2.15 ± 0.05	2.10 ± 0.09	3	3	Hedges' g (SE-to-SD; CI based on independent tanks)	0.56	-1.09 to 2.21	Higher measured value with hydroxyproline; 95% CI included 0 (imprecise estimate)	Not applicable - effect size estimated; see Notes for subsampling treatment
Cao et al. (2022)	Improved triploid crucian carp (Carassius auratus triploid)	Hydroxyproline supplementation in a plant-protein-based diet	Hyp 1.14% (1.20% crystalline Hyp added)	Hyp 0.09% (control; no crystalline Hyp added)	Hardness (N)	4.12 ± 0.33	3.67 ± 0.51	3	3	Hedges' g (SE-to-SD; CI based on independent tanks)	0.84	-0.86 to 2.54	Higher, more favorable value with hydroxyproline; 95% CI included 0 (imprecise estimate)	Not applicable - effect size estimated; see Notes for subsampling treatment
Cao et al. (2022)	Improved triploid crucian carp (Carassius auratus triploid)	Hydroxyproline supplementation in a plant-protein-based diet	Hyp 1.14% (1.20% crystalline Hyp added)	Hyp 0.09% (control; no crystalline Hyp added)	Springiness (mm)	0.540 ± 0.060	0.510 ± 0.120	3	3	Hedges' g (SE-to-SD; CI based on independent tanks)	0.25	-1.36 to 1.86	Higher, more favorable value with hydroxyproline; 95% CI included 0 (imprecise estimate)	Not applicable - effect size estimated; see Notes for subsampling treatment
Cao et al. (2022)	Improved triploid crucian carp (Carassius auratus triploid)	Hydroxyproline supplementation in a plant-protein-based diet	Hyp 1.14% (1.20% crystalline Hyp added)	Hyp 0.09% (control; no crystalline Hyp added)	Chewiness (mJ)	0.550 ± 0.180	0.600 ± 0.210	3	3	Hedges' g (SE-to-SD; CI based on independent tanks)	-0.2	-1.81 to 1.40	Lower, less favorable value with hydroxyproline; 95% CI included 0 (imprecise estimate)	Not applicable - effect size estimated; see Notes for subsampling treatment
Cao et al. (2022)	Improved triploid crucian carp (Carassius auratus triploid)	Hydroxyproline supplementation in a plant-protein-based diet	Hyp 1.14% (1.20% crystalline Hyp added)	Hyp 0.09% (control; no crystalline Hyp added)	Adhesiveness (N·mm)	0.031 ± 0.009	0.031 ± 0.012	3	3	Hedges' g (SE-to-SD; CI based on independent tanks)	0.0	-1.60 to 1.60	No observed mean difference; 95% CI included 0 (imprecise estimate)	Not applicable - effect size estimated; see Notes for subsampling treatment
Cao et al. (2022)	Improved triploid crucian carp (Carassius auratus triploid)	Hydroxyproline supplementation in a plant-protein-based diet	Hyp 1.14% (1.20% crystalline Hyp added)	Hyp 0.09% (control; no crystalline Hyp added)	Cohesiveness (%)	0.360 ± 0.060	0.380 ± 0.090	3	3	Hedges' g (SE-to-SD; CI based on independent tanks)	-0.21	-1.82 to 1.40	Lower measured value with hydroxyproline; 95% CI included 0 (imprecise estimate)	Not applicable - effect size estimated; see Notes for subsampling treatment
Cao et al. (2022)	Improved triploid crucian carp (Carassius auratus triploid)	Hydroxyproline supplementation in a plant-protein-based diet	Hyp 1.14% (1.20% crystalline Hyp added)	Hyp 0.09% (control; no crystalline Hyp added)	Gumminess (g·mm)	1.10 ± 0.30	1.04 ± 0.36	3	3	Hedges' g (SE-to-SD; CI based on independent tanks)	0.14	-1.46 to 1.75	Higher, more favorable value with hydroxyproline; 95% CI included 0 (imprecise estimate)	Not applicable - effect size estimated; see Notes for subsampling treatment
Cao et al. (2022)	Improved triploid crucian carp (Carassius auratus triploid)	Hydroxyproline supplementation in a plant-protein-based diet	Hyp 1.14% (1.20% crystalline Hyp added)	Hyp 0.09% (control; no crystalline Hyp added)	Muscle hydroxyproline (mg/g)	1.41 ± 0.39	0.910 ± 0.245	3	3	Hedges' g (SE-to-SD; CI based on independent tanks)	1.22	-0.59 to 3.04	Higher, more favorable value with hydroxyproline; 95% CI included 0 (imprecise estimate)	Not applicable - effect size estimated; see Notes for subsampling treatment
Cao et al. (2022)	Improved triploid crucian carp (Carassius auratus triploid)	Hydroxyproline supplementation in a plant-protein-based diet	Hyp 1.14% (1.20% crystalline Hyp added)	Hyp 0.09% (control; no crystalline Hyp added)	Skin hydroxyproline (mg/g)	11.98 ± 2.30	8.77 ± 2.28	3	3	Hedges' g (SE-to-SD; CI based on independent tanks)	1.12	-0.66 to 2.90	Higher, more favorable value with hydroxyproline; 95% CI included 0 (imprecise estimate)	Not applicable - effect size estimated; see Notes for subsampling treatment
Cao et al. (2022)	Improved triploid crucian carp (Carassius auratus triploid)	Hydroxyproline supplementation in a plant-protein-based diet	Hyp 1.14% (1.20% crystalline Hyp added)	Hyp 0.09% (control; no crystalline Hyp added)	Liver hydroxyproline (μg/g)	567.93 ± 479.39	256.88 ± 13.94	3	3	Hedges' g (SE-to-SD; CI based on independent tanks)	0.73	-0.95 to 2.41	Higher, more favorable value with hydroxyproline; 95% CI included 0 (imprecise estimate)	Not applicable - effect size estimated; see Notes for subsampling treatment
Cao et al. (2022)	Improved triploid crucian carp (Carassius auratus triploid)	Hydroxyproline supplementation in a plant-protein-based diet	Hyp 1.14% (1.20% crystalline Hyp added)	Hyp 0.09% (control; no crystalline Hyp added)	Vertebral hydroxyproline (mg/g)	9.47 ± 2.18	7.39 ± 0.42	3	3	Hedges' g (SE-to-SD; CI based on independent tanks)	1.06	-0.70 to 2.82	Higher, more favorable value with hydroxyproline; 95% CI included 0 (imprecise estimate)	Not applicable - effect size estimated; see Notes for subsampling treatment
Cao et al. (2022)	Improved triploid crucian carp (Carassius auratus triploid)	Hydroxyproline supplementation in a plant-protein-based diet	Hyp 1.14% (1.20% crystalline Hyp added)	Hyp 0.09% (control; no crystalline Hyp added)	Plasma hydroxyproline (mg/mL)	141.27 ± 26.16	100.00 ± 23.56	3	3	Hedges' g (SE-to-SD; CI based on independent tanks)	1.33	-0.52 to 3.17	Higher, more favorable value with hydroxyproline; 95% CI included 0 (imprecise estimate)	Not applicable - effect size estimated; see Notes for subsampling treatment
Cao et al. (2022)	Improved triploid crucian carp (Carassius auratus triploid)	Hydroxyproline supplementation in a plant-protein-based diet	Hyp 1.14% (1.20% crystalline Hyp added)	Hyp 0.09% (control; no crystalline Hyp added)	Type I collagen (ng/mg protein)	5.13 ± 0.59	4.88 ± 0.05	3	3	Hedges' g (SE-to-SD; CI based on independent tanks)	0.48	-1.15 to 2.11	Higher, more favorable value with hydroxyproline; 95% CI included 0 (imprecise estimate)	Not applicable - effect size estimated; see Notes for subsampling treatment
Cao et al. (2022)	Improved triploid crucian carp (Carassius auratus triploid)	Hydroxyproline supplementation in a plant-protein-based diet	Hyp 1.14% (1.20% crystalline Hyp added)	Hyp 0.09% (control; no crystalline Hyp added)	Pyridinium crosslink (ng/mg protein)	26.73 ± 4.02	25.74 ± 2.60	3	3	Hedges' g (SE-to-SD; CI based on independent tanks)	0.23	-1.37 to 1.84	Higher, more favorable value with hydroxyproline; 95% CI included 0 (imprecise estimate)	Not applicable - effect size estimated; see Notes for subsampling treatment
Cao et al. (2022)	Improved triploid crucian carp (Carassius auratus triploid)	Hydroxyproline supplementation in a plant-protein-based diet	Hyp 1.14% (1.20% crystalline Hyp added)	Hyp 0.09% (control; no crystalline Hyp added)	Prolyl 4-hydroxylase activity (ng/mg protein)	0.830 ± 0.122	0.660 ± 0.073	3	3	Hedges' g (SE-to-SD; CI based on independent tanks)	1.35	-0.51 to 3.20	Higher, more favorable value with hydroxyproline; 95% CI included 0 (imprecise estimate)	Not applicable - effect size estimated; see Notes for subsampling treatment
Cao et al. (2022)	Improved triploid crucian carp (Carassius auratus triploid)	Hydroxyproline supplementation in a plant-protein-based diet	Hyp 1.14% (1.20% crystalline Hyp added)	Hyp 0.09% (control; no crystalline Hyp added)	Lysyl hydroxylase activity (ng/mg protein)	0.154 ± 0.012	0.136 ± 0.012	3	3	Hedges' g (SE-to-SD; CI based on independent tanks)	1.18	-0.62 to 2.97	Higher, more favorable value with hydroxyproline; 95% CI included 0 (imprecise estimate)	Not applicable - effect size estimated; see Notes for subsampling treatment
Cao et al. (2022)	Improved triploid crucian carp (Carassius auratus triploid)	Hydroxyproline supplementation in a plant-protein-based diet	Hyp 1.14% (1.20% crystalline Hyp added)	Hyp 0.09% (control; no crystalline Hyp added)	Muscle fiber diameter (μm)	58.12 ± 2.77	47.49 ± 4.75	3	3	Hedges' g (SE-to-SD; CI based on independent tanks)	2.19	-0.02 to 4.39	Higher, more favorable value with hydroxyproline; 95% CI included 0 (imprecise estimate)	Not applicable - effect size estimated; see Notes for subsampling treatment
Cao et al. (2022)	Improved triploid crucian carp (Carassius auratus triploid)	Hydroxyproline supplementation in a plant-protein-based diet	Hyp 1.14% (1.20% crystalline Hyp added)	Hyp 0.09% (control; no crystalline Hyp added)	Muscle fiber density (n/mm^2)	218.00 ± 46.74	158.67 ± 15.77	3	3	Hedges' g (SE-to-SD; CI based on independent tanks)	1.36	-0.50 to 3.22	Higher, more favorable value with hydroxyproline; 95% CI included 0 (imprecise estimate)	Not applicable - effect size estimated; see Notes for subsampling treatment
Cao et al. (2022)	Improved triploid crucian carp (Carassius auratus triploid)	Hydroxyproline supplementation in a plant-protein-based diet	Hyp 1.53% (1.60% crystalline Hyp added)	Hyp 0.09% (control; no crystalline Hyp added)	Final body weight (g)	46.76 ± 1.25	41.94 ± 1.85	3	3	Hedges' g (SE-to-SD; CI based on independent tanks)	2.44	0.11 to 4.77	Higher, more favorable value with hydroxyproline; 95% CI excluded 0	Not applicable - effect size estimated; see Notes for subsampling treatment
Cao et al. (2022)	Improved triploid crucian carp (Carassius auratus triploid)	Hydroxyproline supplementation in a plant-protein-based diet	Hyp 1.53% (1.60% crystalline Hyp added)	Hyp 0.09% (control; no crystalline Hyp added)	Weight gain rate (%)	211.16 ± 8.45	179.76 ± 13.48	3	3	Hedges' g (SE-to-SD; CI based on independent tanks)	2.23	0.01 to 4.46	Higher, more favorable value with hydroxyproline; 95% CI excluded 0	Not applicable - effect size estimated; see Notes for subsampling treatment
Cao et al. (2022)	Improved triploid crucian carp (Carassius auratus triploid)	Hydroxyproline supplementation in a plant-protein-based diet	Hyp 1.53% (1.60% crystalline Hyp added)	Hyp 0.09% (control; no crystalline Hyp added)	Specific growth rate (%/day)	2.03 ± 0.05	1.84 ± 0.09	3	3	Hedges' g (SE-to-SD; CI based on independent tanks)	2.13	-0.05 to 4.30	Higher, more favorable value with hydroxyproline; 95% CI included 0 (imprecise estimate)	Not applicable - effect size estimated; see Notes for subsampling treatment
Cao et al. (2022)	Improved triploid crucian carp (Carassius auratus triploid)	Hydroxyproline supplementation in a plant-protein-based diet	Hyp 1.53% (1.60% crystalline Hyp added)	Hyp 0.09% (control; no crystalline Hyp added)	Feed efficiency (%)	53.90 ± 2.11	44.60 ± 3.00	3	3	Hedges' g (SE-to-SD; CI based on independent tanks)	2.87	0.32 to 5.42	Higher, more favorable value with hydroxyproline; 95% CI excluded 0	Not applicable - effect size estimated; see Notes for subsampling treatment

Study	Species	Strategy	Intervention	Comparator	Outcome and unit	Intervention mean ± SD	Comparator mean ± SD	n intervention (independent tanks)	n comparator (independent tanks)	Effect-size metric	Effect size	95% CI	Interpretation	Reason not estimated
Cao et al. (2022)	Improved triploid crucian carp (Carassius auratus triploid)	Hydroxyproline supplementation in a plant-protein-based diet	Hyp 1.53% (1.60% crystalline Hyp added)	Hyp 0.09% (control; no crystalline Hyp added)	Feeding rate (% body weight/day)	3.18 ± 0.07	3.54 ± 0.10	3	3	Hedges' g (SE-to-SD; CI based on independent tanks)	3.26	0.49 to 6.03	Lower, more favorable value with hydroxyproline; 95% CI excluded 0	Not applicable - effect size estimated; see Notes for subsampling treatment
Cao et al. (2022)	Improved triploid crucian carp (Carassius auratus triploid)	Hydroxyproline supplementation in a plant-protein-based diet	Hyp 1.53% (1.60% crystalline Hyp added)	Hyp 0.09% (control; no crystalline Hyp added)	Protein efficiency ratio (%)	158.47 ± 6.22	128.49 ± 8.61	3	3	Hedges' g (SE-to-SD; CI based on independent tanks)	3.19	0.46 to 5.93	Higher, more favorable value with hydroxyproline; 95% CI excluded 0	Not applicable - effect size estimated; see Notes for subsampling treatment
Cao et al. (2022)	Improved triploid crucian carp (Carassius auratus triploid)	Hydroxyproline supplementation in a plant-protein-based diet	Hyp 1.53% (1.60% crystalline Hyp added)	Hyp 0.09% (control; no crystalline Hyp added)	Nitrogen retention efficiency (%)	25.67 ± 0.83	22.71 ± 1.47	3	3	Hedges' g (SE-to-SD; CI based on independent tanks)	1.98	-0.13 to 4.09	Higher, more favorable value with hydroxyproline; 95% CI included 0 (imprecise estimate)	Not applicable - effect size estimated; see Notes for subsampling treatment
Cao et al. (2022)	Improved triploid crucian carp (Carassius auratus triploid)	Hydroxyproline supplementation in a plant-protein-based diet	Hyp 1.53% (1.60% crystalline Hyp added)	Hyp 0.09% (control; no crystalline Hyp added)	Total nitrogen waste output (g/kg weight gain)	38.56 ± 1.30	45.57 ± 1.59	3	3	Hedges' g (SE-to-SD; CI based on independent tanks)	3.86	0.74 to 6.97	Lower, more favorable value with hydroxyproline; 95% CI excluded 0	Not applicable - effect size estimated; see Notes for subsampling treatment
Cao et al. (2022)	Improved triploid crucian carp (Carassius auratus triploid)	Hydroxyproline supplementation in a plant-protein-based diet	Hyp 1.53% (1.60% crystalline Hyp added)	Hyp 0.09% (control; no crystalline Hyp added)	Survival rate (%)	100.00 ± 0.00	100.00 ± 0.00	3	3	Not estimable	Not estimable	Not estimable	No observed mean difference and no within-group variance	Both groups had zero reported SE; standardized effect size is undefined
Cao et al. (2022)	Improved triploid crucian carp (Carassius auratus triploid)	Hydroxyproline supplementation in a plant-protein-based diet	Hyp 1.53% (1.60% crystalline Hyp added)	Hyp 0.09% (control; no crystalline Hyp added)	Whole-body crude protein (% fresh weight)	16.01 ± 0.28	15.72 ± 0.57	3	3	Hedges' g (SE-to-SD; CI based on independent tanks)	0.52	-1.12 to 2.16	Higher measured value with hydroxyproline; 95% CI included 0 (imprecise estimate)	Not applicable - effect size estimated; see Notes for subsampling treatment
Cao et al. (2022)	Improved triploid crucian carp (Carassius auratus triploid)	Hydroxyproline supplementation in a plant-protein-based diet	Hyp 1.53% (1.60% crystalline Hyp added)	Hyp 0.09% (control; no crystalline Hyp added)	Whole-body crude lipid (% fresh weight)	13.06 ± 1.58	11.04 ± 0.87	3	3	Hedges' g (SE-to-SD; CI based on independent tanks)	1.27	-0.56 to 3.10	Higher measured value with hydroxyproline; 95% CI included 0 (imprecise estimate)	Not applicable - effect size estimated; see Notes for subsampling treatment
Cao et al. (2022)	Improved triploid crucian carp (Carassius auratus triploid)	Hydroxyproline supplementation in a plant-protein-based diet	Hyp 1.53% (1.60% crystalline Hyp added)	Hyp 0.09% (control; no crystalline Hyp added)	Whole-body ash (% fresh weight)	2.63 ± 0.09	2.85 ± 0.28	3	3	Hedges' g (SE-to-SD; CI based on independent tanks)	-0.86	-2.56 to 0.85	Lower measured value with hydroxyproline; 95% CI included 0 (imprecise estimate)	Not applicable - effect size estimated; see Notes for subsampling treatment
Cao et al. (2022)	Improved triploid crucian carp (Carassius auratus triploid)	Hydroxyproline supplementation in a plant-protein-based diet	Hyp 1.53% (1.60% crystalline Hyp added)	Hyp 0.09% (control; no crystalline Hyp added)	Whole-body moisture (% fresh weight)	67.94 ± 1.68	69.85 ± 0.42	3	3	Hedges' g (SE-to-SD; CI based on independent tanks)	-1.25	-3.07 to 0.57	Lower measured value with hydroxyproline; 95% CI included 0 (imprecise estimate)	Not applicable - effect size estimated; see Notes for subsampling treatment
Cao et al. (2022)	Improved triploid crucian carp (Carassius auratus triploid)	Hydroxyproline supplementation in a plant-protein-based diet	Hyp 1.53% (1.60% crystalline Hyp added)	Hyp 0.09% (control; no crystalline Hyp added)	Dorsal-muscle crude protein (% fresh weight)	17.57 ± 0.48	17.06 ± 0.21	3	3	Hedges' g (SE-to-SD; CI based on independent tanks)	1.09	-0.68 to 2.86	Higher measured value with hydroxyproline; 95% CI included 0 (imprecise estimate)	Not applicable - effect size estimated; see Notes for subsampling treatment
Cao et al. (2022)	Improved triploid crucian carp (Carassius auratus triploid)	Hydroxyproline supplementation in a plant-protein-based diet	Hyp 1.53% (1.60% crystalline Hyp added)	Hyp 0.09% (control; no crystalline Hyp added)	Dorsal-muscle crude lipid (% fresh weight)	1.25 ± 0.14	1.32 ± 0.12	3	3	Hedges' g (SE-to-SD; CI based on independent tanks)	-0.43	-2.06 to 1.20	Lower measured value with hydroxyproline; 95% CI included 0 (imprecise estimate)	Not applicable - effect size estimated; see Notes for subsampling treatment
Cao et al. (2022)	Improved triploid crucian carp (Carassius auratus triploid)	Hydroxyproline supplementation in a plant-protein-based diet	Hyp 1.53% (1.60% crystalline Hyp added)	Hyp 0.09% (control; no crystalline Hyp added)	Dorsal-muscle ash (% fresh weight)	1.39 ± 0.07	1.44 ± 0.10	3	3	Hedges' g (SE-to-SD; CI based on independent tanks)	-0.45	-2.08 to 1.18	Lower measured value with hydroxyproline; 95% CI included 0 (imprecise estimate)	Not applicable - effect size estimated; see Notes for subsampling treatment
Cao et al. (2022)	Improved triploid crucian carp (Carassius auratus triploid)	Hydroxyproline supplementation in a plant-protein-based diet	Hyp 1.53% (1.60% crystalline Hyp added)	Hyp 0.09% (control; no crystalline Hyp added)	Dorsal-muscle moisture (% fresh weight)	75.92 ± 1.66	75.65 ± 0.16	3	3	Hedges' g (SE-to-SD; CI based on independent tanks)	0.18	-1.42 to 1.79	Higher measured value with hydroxyproline; 95% CI included 0 (imprecise estimate)	Not applicable - effect size estimated; see Notes for subsampling treatment
Cao et al. (2022)	Improved triploid crucian carp (Carassius auratus triploid)	Hydroxyproline supplementation in a plant-protein-based diet	Hyp 1.53% (1.60% crystalline Hyp added)	Hyp 0.09% (control; no crystalline Hyp added)	Threonine (% dry matter)	2.89 ± 0.05	2.62 ± 0.16	3	3	Hedges' g (SE-to-SD; CI based on independent tanks)	1.86	-0.20 to 3.91	Higher measured value with hydroxyproline; 95% CI included 0 (imprecise estimate)	Not applicable - effect size estimated; see Notes for subsampling treatment
Cao et al. (2022)	Improved triploid crucian carp (Carassius auratus triploid)	Hydroxyproline supplementation in a plant-protein-based diet	Hyp 1.53% (1.60% crystalline Hyp added)	Hyp 0.09% (control; no crystalline Hyp added)	Phenylalanine (% dry matter)	3.23 ± 0.16	2.95 ± 0.19	3	3	Hedges' g (SE-to-SD; CI based on independent tanks)	1.29	-0.55 to 3.12	Higher measured value with hydroxyproline; 95% CI included 0 (imprecise estimate)	Not applicable - effect size estimated; see Notes for subsampling treatment
Cao et al. (2022)	Improved triploid crucian carp (Carassius auratus triploid)	Hydroxyproline supplementation in a plant-protein-based diet	Hyp 1.53% (1.60% crystalline Hyp added)	Hyp 0.09% (control; no crystalline Hyp added)	Valine (% dry matter)	3.24 ± 0.09	2.98 ± 0.19	3	3	Hedges' g (SE-to-SD; CI based on independent tanks)	1.41	-0.47 to 3.28	Higher measured value with hydroxyproline; 95% CI included 0 (imprecise estimate)	Not applicable - effect size estimated; see Notes for subsampling treatment
Cao et al. (2022)	Improved triploid crucian carp (Carassius auratus triploid)	Hydroxyproline supplementation in a plant-protein-based diet	Hyp 1.53% (1.60% crystalline Hyp added)	Hyp 0.09% (control; no crystalline Hyp added)	Isoleucine (% dry matter)	3.31 ± 0.12	3.03 ± 0.17	3	3	Hedges' g (SE-to-SD; CI based on independent tanks)	1.5	-0.41 to 3.41	Higher measured value with hydroxyproline; 95% CI included 0 (imprecise estimate)	Not applicable - effect size estimated; see Notes for subsampling treatment
Cao et al. (2022)	Improved triploid crucian carp (Carassius auratus triploid)	Hydroxyproline supplementation in a plant-protein-based diet	Hyp 1.53% (1.60% crystalline Hyp added)	Hyp 0.09% (control; no crystalline Hyp added)	Leucine (% dry matter)	5.84 ± 0.19	5.36 ± 0.31	3	3	Hedges' g (SE-to-SD; CI based on independent tanks)	1.49	-0.42 to 3.39	Higher measured value with hydroxyproline; 95% CI included 0 (imprecise estimate)	Not applicable - effect size estimated; see Notes for subsampling treatment
Cao et al. (2022)	Improved triploid crucian carp (Carassius auratus triploid)	Hydroxyproline supplementation in a plant-protein-based diet	Hyp 1.53% (1.60% crystalline Hyp added)	Hyp 0.09% (control; no crystalline Hyp added)	Methionine (% dry matter)	1.97 ± 0.05	1.72 ± 0.05	3	3	Hedges' g (SE-to-SD; CI based on independent tanks)	3.85	0.74 to 6.96	Higher measured value with hydroxyproline; 95% CI excluded 0	Not applicable - effect size estimated; see Notes for subsampling treatment
Cao et al. (2022)	Improved triploid crucian carp (Carassius auratus triploid)	Hydroxyproline supplementation in a plant-protein-based diet	Hyp 1.53% (1.60% crystalline Hyp added)	Hyp 0.09% (control; no crystalline Hyp added)	Arginine (% dry matter)	4.02 ± 0.16	3.67 ± 0.23	3	3	Hedges' g (SE-to-SD; CI based on independent tanks)	1.45	-0.44 to 3.33	Higher measured value with hydroxyproline; 95% CI included 0 (imprecise estimate)	Not applicable - effect size estimated; see Notes for subsampling treatment
Cao et al. (2022)	Improved triploid crucian carp (Carassius auratus triploid)	Hydroxyproline supplementation in a plant-protein-based diet	Hyp 1.53% (1.60% crystalline Hyp added)	Hyp 0.09% (control; no crystalline Hyp added)	Lysine (% dry matter)	6.95 ± 0.64	6.01 ± 0.50	3	3	Hedges' g (SE-to-SD; CI based on independent tanks)	1.31	-0.53 to 3.14	Higher measured value with hydroxyproline; 95% CI included 0 (imprecise estimate)	Not applicable - effect size estimated; see Notes for subsampling treatment
Cao et al. (2022)	Improved triploid crucian carp (Carassius auratus triploid)	Hydroxyproline supplementation in a plant-protein-based diet	Hyp 1.53% (1.60% crystalline Hyp added)	Hyp 0.09% (control; no crystalline Hyp added)	Histidine (% dry matter)	3.57 ± 0.16	3.21 ± 0.24	3	3	Hedges' g (SE-to-SD; CI based on independent tanks)	1.41	-0.46 to 3.29	Higher measured value with hydroxyproline; 95% CI included 0 (imprecise estimate)	Not applicable - effect size estimated; see Notes for subsampling treatment
Cao et al. (2022)	Improved triploid crucian carp (Carassius auratus triploid)	Hydroxyproline supplementation in a plant-protein-based diet	Hyp 1.53% (1.60% crystalline Hyp added)	Hyp 0.09% (control; no crystalline Hyp added)	Aspartic acid (% dry matter)	7.40 ± 0.14	6.84 ± 0.43	3	3	Hedges' g (SE-to-SD; CI based on independent tanks)	1.39	-0.48 to 3.26	Higher measured value with hydroxyproline; 95% CI included 0 (imprecise estimate)	Not applicable - effect size estimated; see Notes for subsampling treatment
Cao et al. (2022)	Improved triploid crucian carp (Carassius auratus triploid)	Hydroxyproline supplementation in a plant-protein-based diet	Hyp 1.53% (1.60% crystalline Hyp added)	Hyp 0.09% (control; no crystalline Hyp added)	Serine (% dry matter)	2.95 ± 0.09	2.74 ± 0.16	3	3	Hedges' g (SE-to-SD; CI based on independent tanks)	1.33	-0.52 to 3.18	Higher measured value with hydroxyproline; 95% CI included 0 (imprecise estimate)	Not applicable - effect size estimated; see Notes for subsampling treatment
Cao et al. (2022)	Improved triploid crucian carp (Carassius auratus triploid)	Hydroxyproline supplementation in a plant-protein-based diet	Hyp 1.53% (1.60% crystalline Hyp added)	Hyp 0.09% (control; no crystalline Hyp added)	Glutamic acid (% dry matter)	10.54 ± 0.17	9.74 ± 0.57	3	3	Hedges' g (SE-to-SD; CI based on independent tanks)	1.52	-0.40 to 3.43	Higher measured value with hydroxyproline; 95% CI included 0 (imprecise estimate)	Not applicable - effect size estimated; see Notes for subsampling treatment
Cao et al. (2022)	Improved triploid crucian carp (Carassius auratus triploid)	Hydroxyproline supplementation in a plant-protein-based diet	Hyp 1.53% (1.60% crystalline Hyp added)	Hyp 0.09% (control; no crystalline Hyp added)	Glycine (% dry matter)	3.06 ± 0.05	2.93 ± 0.10	3	3	Hedges' g (SE-to-SD; CI based on independent tanks)	1.27	-0.56 to 3.09	Higher measured value with hydroxyproline; 95% CI included 0 (imprecise estimate)	Not applicable - effect size estimated; see Notes for subsampling treatment
Cao et al. (2022)	Improved triploid crucian carp (Carassius auratus triploid)	Hydroxyproline supplementation in a plant-protein-based diet	Hyp 1.53% (1.60% crystalline Hyp added)	Hyp 0.09% (control; no crystalline Hyp added)	Alanine (% dry matter)	3.53 ± 0.09	3.27 ± 0.17	3	3	Hedges' g (SE-to-SD; CI based on independent tanks)	1.52	-0.40 to 3.43	Higher measured value with hydroxyproline; 95% CI included 0 (imprecise estimate)	Not applicable - effect size estimated; see Notes for subsampling treatment
Cao et al. (2022)	Improved triploid crucian carp (Carassius auratus triploid)	Hydroxyproline supplementation in a plant-protein-based diet	Hyp 1.53% (1.60% crystalline Hyp added)	Hyp 0.09% (control; no crystalline Hyp added)	Tyrosine (% dry matter)	2.33 ± 0.09	2.15 ± 0.14	3	3	Hedges' g (SE-to-SD; CI based on independent tanks)	1.25	-0.57 to 3.06	Higher measured value with hydroxyproline; 95% CI included 0 (imprecise estimate)	Not applicable - effect size estimated; see Notes for subsampling treatment
Cao et al. (2022)	Improved triploid crucian carp (Carassius auratus triploid)	Hydroxyproline supplementation in a plant-protein-based diet	Hyp 1.53% (1.60% crystalline Hyp added)	Hyp 0.09% (control; no crystalline Hyp added)	Proline (% dry matter)	2.18 ± 0.05	2.10 ± 0.09	3	3	Hedges' g (SE-to-SD; CI based on independent tanks)	0.9	-0.82 to 2.61	Higher measured value with hydroxyproline; 95% CI included 0 (imprecise estimate)	Not applicable - effect size estimated; see Notes for subsampling treatment
Cao et al. (2022)	Improved triploid crucian carp (Carassius auratus triploid)	Hydroxyproline supplementation in a plant-protein-based diet	Hyp 1.53% (1.60% crystalline Hyp added)	Hyp 0.09% (control; no crystalline Hyp added)	Hardness (N)	4.16 ± 0.24	3.67 ± 0.51	3	3	Hedges' g (SE-to-SD; CI based on independent tanks)	0.98	-0.76 to 2.72	Higher, more favorable value with hydroxyproline; 95% CI included 0 (imprecise estimate)	Not applicable - effect size estimated; see Notes for subsampling treatment
Cao et al. (2022)	Improved triploid crucian carp (Carassius auratus triploid)	Hydroxyproline supplementation in a plant-protein-based diet	Hyp 1.53% (1.60% crystalline Hyp added)	Hyp 0.09% (control; no crystalline Hyp added)	Springiness (mm)	0.640 ± 0.150	0.510 ± 0.120	3	3	Hedges' g (SE-to-SD; CI based on independent tanks)	0.77	-0.92 to 2.45	Higher, more favorable value with hydroxyproline; 95% CI included 0 (imprecise estimate)	Not applicable - effect size estimated; see Notes for subsampling treatment

Study	Species	Strategy	Intervention	Comparator	Outcome and unit	Intervention mean ± SD	Comparator mean ± SD	n intervention (independent tanks)	n comparator (independent tanks)	Effect-size metric	Effect size	95% CI	Interpretation	Reason not estimated
Cao et al. (2022)	Improved triploid crucian carp (Carassius auratus triploid)	Hydroxyproline supplementation in a plant-protein-based diet	Hyp 1.53% (1.60% crystalline Hyp added)	Hyp 0.09% (control; no crystalline Hyp added)	Chewiness (mJ)	0.930 ± 0.390	0.600 ± 0.210	3	3	Hedges' g (SE-to-SD; CI based on independent tanks)	0.84	-0.86 to 2.55	Higher, more favorable value with hydroxyproline; 95% CI included 0 (imprecise estimate)	Not applicable - effect size estimated; see Notes for subsampling treatment
Cao et al. (2022)	Improved triploid crucian carp (Carassius auratus triploid)	Hydroxyproline supplementation in a plant-protein-based diet	Hyp 1.53% (1.60% crystalline Hyp added)	Hyp 0.09% (control; no crystalline Hyp added)	Adhesiveness (N·mm)	0.027 ± 0.009	0.031 ± 0.012	3	3	Hedges' g (SE-to-SD; CI based on independent tanks)	-0.3	-1.92 to 1.31	Lower measured value with hydroxyproline; 95% CI included 0 (imprecise estimate)	Not applicable - effect size estimated; see Notes for subsampling treatment
Cao et al. (2022)	Improved triploid crucian carp (Carassius auratus triploid)	Hydroxyproline supplementation in a plant-protein-based diet	Hyp 1.53% (1.60% crystalline Hyp added)	Hyp 0.09% (control; no crystalline Hyp added)	Cohesiveness (%)	0.430 ± 0.090	0.380 ± 0.090	3	3	Hedges' g (SE-to-SD; CI based on independent tanks)	0.44	-1.19 to 2.07	Higher measured value with hydroxyproline; 95% CI included 0 (imprecise estimate)	Not applicable - effect size estimated; see Notes for subsampling treatment
Cao et al. (2022)	Improved triploid crucian carp (Carassius auratus triploid)	Hydroxyproline supplementation in a plant-protein-based diet	Hyp 1.53% (1.60% crystalline Hyp added)	Hyp 0.09% (control; no crystalline Hyp added)	Gumminess (g·mm)	1.54 ± 0.42	1.04 ± 0.36	3	3	Hedges' g (SE-to-SD; CI based on independent tanks)	1.02	-0.73 to 2.77	Higher, more favorable value with hydroxyproline; 95% CI included 0 (imprecise estimate)	Not applicable - effect size estimated; see Notes for subsampling treatment
Cao et al. (2022)	Improved triploid crucian carp (Carassius auratus triploid)	Hydroxyproline supplementation in a plant-protein-based diet	Hyp 1.53% (1.60% crystalline Hyp added)	Hyp 0.09% (control; no crystalline Hyp added)	Muscle hydroxyproline (mg/g)	1.46 ± 0.42	0.910 ± 0.245	3	3	Hedges' g (SE-to-SD; CI based on independent tanks)	1.29	-0.54 to 3.12	Higher, more favorable value with hydroxyproline; 95% CI included 0 (imprecise estimate)	Not applicable - effect size estimated; see Notes for subsampling treatment
Cao et al. (2022)	Improved triploid crucian carp (Carassius auratus triploid)	Hydroxyproline supplementation in a plant-protein-based diet	Hyp 1.53% (1.60% crystalline Hyp added)	Hyp 0.09% (control; no crystalline Hyp added)	Skin hydroxyproline (mg/g)	9.66 ± 2.30	8.77 ± 2.28	3	3	Hedges' g (SE-to-SD; CI based on independent tanks)	0.31	-1.30 to 1.93	Higher, more favorable value with hydroxyproline; 95% CI included 0 (imprecise estimate)	Not applicable - effect size estimated; see Notes for subsampling treatment
Cao et al. (2022)	Improved triploid crucian carp (Carassius auratus triploid)	Hydroxyproline supplementation in a plant-protein-based diet	Hyp 1.53% (1.60% crystalline Hyp added)	Hyp 0.09% (control; no crystalline Hyp added)	Liver hydroxyproline (μg/g)	430.59 ± 114.39	256.88 ± 13.94	3	3	Hedges' g (SE-to-SD; CI based on independent tanks)	1.71	-0.28 to 3.69	Higher, more favorable value with hydroxyproline; 95% CI included 0 (imprecise estimate)	Not applicable - effect size estimated; see Notes for subsampling treatment
Cao et al. (2022)	Improved triploid crucian carp (Carassius auratus triploid)	Hydroxyproline supplementation in a plant-protein-based diet	Hyp 1.53% (1.60% crystalline Hyp added)	Hyp 0.09% (control; no crystalline Hyp added)	Vertebral hydroxyproline (mg/g)	9.04 ± 2.89	7.39 ± 0.42	3	3	Hedges' g (SE-to-SD; CI based on independent tanks)	0.64	-1.02 to 2.30	Higher, more favorable value with hydroxyproline; 95% CI included 0 (imprecise estimate)	Not applicable - effect size estimated; see Notes for subsampling treatment
Cao et al. (2022)	Improved triploid crucian carp (Carassius auratus triploid)	Hydroxyproline supplementation in a plant-protein-based diet	Hyp 1.53% (1.60% crystalline Hyp added)	Hyp 0.09% (control; no crystalline Hyp added)	Plasma hydroxyproline (mg/mL)	142.86 ± 11.66	100.00 ± 23.56	3	3	Hedges' g (SE-to-SD; CI based on independent tanks)	1.84	-0.20 to 3.89	Higher, more favorable value with hydroxyproline; 95% CI included 0 (imprecise estimate)	Not applicable - effect size estimated; see Notes for subsampling treatment
Cao et al. (2022)	Improved triploid crucian carp (Carassius auratus triploid)	Hydroxyproline supplementation in a plant-protein-based diet	Hyp 1.53% (1.60% crystalline Hyp added)	Hyp 0.09% (control; no crystalline Hyp added)	Type I collagen (ng/mg protein)	6.02 ± 0.66	4.88 ± 0.05	3	3	Hedges' g (SE-to-SD; CI based on independent tanks)	1.94	-0.15 to 4.04	Higher, more favorable value with hydroxyproline; 95% CI included 0 (imprecise estimate)	Not applicable - effect size estimated; see Notes for subsampling treatment
Cao et al. (2022)	Improved triploid crucian carp (Carassius auratus triploid)	Hydroxyproline supplementation in a plant-protein-based diet	Hyp 1.53% (1.60% crystalline Hyp added)	Hyp 0.09% (control; no crystalline Hyp added)	Pyridinium crosslink (ng/mg protein)	30.37 ± 2.87	25.74 ± 2.60	3	3	Hedges' g (SE-to-SD; CI based on independent tanks)	1.35	-0.50 to 3.21	Higher, more favorable value with hydroxyproline; 95% CI included 0 (imprecise estimate)	Not applicable - effect size estimated; see Notes for subsampling treatment
Cao et al. (2022)	Improved triploid crucian carp (Carassius auratus triploid)	Hydroxyproline supplementation in a plant-protein-based diet	Hyp 1.53% (1.60% crystalline Hyp added)	Hyp 0.09% (control; no crystalline Hyp added)	Prolyl 4-hydroxylase activity (ng/mg protein)	0.810 ± 0.171	0.660 ± 0.073	3	3	Hedges' g (SE-to-SD; CI based on independent tanks)	0.91	-0.81 to 2.63	Higher, more favorable value with hydroxyproline; 95% CI included 0 (imprecise estimate)	Not applicable - effect size estimated; see Notes for subsampling treatment
Cao et al. (2022)	Improved triploid crucian carp (Carassius auratus triploid)	Hydroxyproline supplementation in a plant-protein-based diet	Hyp 1.53% (1.60% crystalline Hyp added)	Hyp 0.09% (control; no crystalline Hyp added)	Lysyl hydroxylase activity (ng/mg protein)	0.154 ± 0.010	0.136 ± 0.012	3	3	Hedges' g (SE-to-SD; CI based on independent tanks)	1.3	-0.54 to 3.13	Higher, more favorable value with hydroxyproline; 95% CI included 0 (imprecise estimate)	Not applicable - effect size estimated; see Notes for subsampling treatment
Cao et al. (2022)	Improved triploid crucian carp (Carassius auratus triploid)	Hydroxyproline supplementation in a plant-protein-based diet	Hyp 1.53% (1.60% crystalline Hyp added)	Hyp 0.09% (control; no crystalline Hyp added)	Muscle fiber diameter (μm)	56.39 ± 3.16	47.49 ± 4.75	3	3	Hedges' g (SE-to-SD; CI based on independent tanks)	1.76	-0.25 to 3.78	Higher, more favorable value with hydroxyproline; 95% CI included 0 (imprecise estimate)	Not applicable - effect size estimated; see Notes for subsampling treatment
Cao et al. (2022)	Improved triploid crucian carp (Carassius auratus triploid)	Hydroxyproline supplementation in a plant-protein-based diet	Hyp 1.53% (1.60% crystalline Hyp added)	Hyp 0.09% (control; no crystalline Hyp added)	Muscle fiber density (n/mm²)	184.67 ± 47.47	158.67 ± 15.77	3	3	Hedges' g (SE-to-SD; CI based on independent tanks)	0.59	-1.06 to 2.24	Higher, more favorable value with hydroxyproline; 95% CI included 0 (imprecise estimate)	Not applicable - effect size estimated; see Notes for subsampling treatment
Cao et al. (2022)	Improved triploid crucian carp (Carassius auratus triploid)	Hydroxyproline supplementation in a plant-protein-based diet	Hyp 1.90% (2.00% crystalline Hyp added)	Hyp 0.09% (control; no crystalline Hyp added)	Final body weight (g)	46.68 ± 1.20	41.94 ± 1.85	3	3	Hedges' g (SE-to-SD; CI based on independent tanks)	2.43	0.11 to 4.76	Higher, more favorable value with hydroxyproline; 95% CI excluded 0	Not applicable - effect size estimated; see Notes for subsampling treatment
Cao et al. (2022)	Improved triploid crucian carp (Carassius auratus triploid)	Hydroxyproline supplementation in a plant-protein-based diet	Hyp 1.90% (2.00% crystalline Hyp added)	Hyp 0.09% (control; no crystalline Hyp added)	Weight gain rate (%)	211.12 ± 7.88	179.76 ± 13.48	3	3	Hedges' g (SE-to-SD; CI based on independent tanks)	2.27	0.03 to 4.52	Higher, more favorable value with hydroxyproline; 95% CI excluded 0	Not applicable - effect size estimated; see Notes for subsampling treatment
Cao et al. (2022)	Improved triploid crucian carp (Carassius auratus triploid)	Hydroxyproline supplementation in a plant-protein-based diet	Hyp 1.90% (2.00% crystalline Hyp added)	Hyp 0.09% (control; no crystalline Hyp added)	Specific growth rate (%/day)	2.03 ± 0.03	1.84 ± 0.09	3	3	Hedges' g (SE-to-SD; CI based on independent tanks)	2.3	0.04 to 4.57	Higher, more favorable value with hydroxyproline; 95% CI excluded 0	Not applicable - effect size estimated; see Notes for subsampling treatment
Cao et al. (2022)	Improved triploid crucian carp (Carassius auratus triploid)	Hydroxyproline supplementation in a plant-protein-based diet	Hyp 1.90% (2.00% crystalline Hyp added)	Hyp 0.09% (control; no crystalline Hyp added)	Feed efficiency (%)	52.62 ± 1.80	44.60 ± 3.00	3	3	Hedges' g (SE-to-SD; CI based on independent tanks)	2.6	0.19 to 5.00	Higher, more favorable value with hydroxyproline; 95% CI excluded 0	Not applicable - effect size estimated; see Notes for subsampling treatment
Cao et al. (2022)	Improved triploid crucian carp (Carassius auratus triploid)	Hydroxyproline supplementation in a plant-protein-based diet	Hyp 1.90% (2.00% crystalline Hyp added)	Hyp 0.09% (control; no crystalline Hyp added)	Feeding rate (% body weight/day)	3.25 ± 0.05	3.54 ± 0.10	3	3	Hedges' g (SE-to-SD; CI based on independent tanks)	2.82	0.30 to 5.35	Lower, more favorable value with hydroxyproline; 95% CI excluded 0	Not applicable - effect size estimated; see Notes for subsampling treatment
Cao et al. (2022)	Improved triploid crucian carp (Carassius auratus triploid)	Hydroxyproline supplementation in a plant-protein-based diet	Hyp 1.90% (2.00% crystalline Hyp added)	Hyp 0.09% (control; no crystalline Hyp added)	Protein efficiency ratio (%)	155.99 ± 5.33	128.49 ± 8.61	3	3	Hedges' g (SE-to-SD; CI based on independent tanks)	3.07	0.41 to 5.74	Higher, more favorable value with hydroxyproline; 95% CI excluded 0	Not applicable - effect size estimated; see Notes for subsampling treatment
Cao et al. (2022)	Improved triploid crucian carp (Carassius auratus triploid)	Hydroxyproline supplementation in a plant-protein-based diet	Hyp 1.90% (2.00% crystalline Hyp added)	Hyp 0.09% (control; no crystalline Hyp added)	Nitrogen retention efficiency (%)	25.35 ± 2.04	22.71 ± 1.47	3	3	Hedges' g (SE-to-SD; CI based on independent tanks)	1.19	-0.61 to 2.98	Higher, more favorable value with hydroxyproline; 95% CI included 0 (imprecise estimate)	Not applicable - effect size estimated; see Notes for subsampling treatment
Cao et al. (2022)	Improved triploid crucian carp (Carassius auratus triploid)	Hydroxyproline supplementation in a plant-protein-based diet	Hyp 1.90% (2.00% crystalline Hyp added)	Hyp 0.09% (control; no crystalline Hyp added)	Total nitrogen waste output (g/kg weight gain)	39.33 ± 1.68	45.57 ± 1.59	3	3	Hedges' g (SE-to-SD; CI based on independent tanks)	3.05	0.40 to 5.70	Lower, more favorable value with hydroxyproline; 95% CI excluded 0	Not applicable - effect size estimated; see Notes for subsampling treatment
Cao et al. (2022)	Improved triploid crucian carp (Carassius auratus triploid)	Hydroxyproline supplementation in a plant-protein-based diet	Hyp 1.90% (2.00% crystalline Hyp added)	Hyp 0.09% (control; no crystalline Hyp added)	Survival rate (%)	100.00 ± 0.00	100.00 ± 0.00	3	3	Not estimable	Not estimable	Not estimable	No observed mean difference and no within-group variance	Both groups had zero reported SE; standardized effect size is undefined
Cao et al. (2022)	Improved triploid crucian carp (Carassius auratus triploid)	Hydroxyproline supplementation in a plant-protein-based diet	Hyp 1.90% (2.00% crystalline Hyp added)	Hyp 0.09% (control; no crystalline Hyp added)	Whole-body crude protein (% fresh weight)	15.68 ± 0.10	15.72 ± 0.57	3	3	Hedges' g (SE-to-SD; CI based on independent tanks)	-0.08	-1.68 to 1.52	Lower measured value with hydroxyproline; 95% CI included 0 (imprecise estimate)	Not applicable - effect size estimated; see Notes for subsampling treatment
Cao et al. (2022)	Improved triploid crucian carp (Carassius auratus triploid)	Hydroxyproline supplementation in a plant-protein-based diet	Hyp 1.90% (2.00% crystalline Hyp added)	Hyp 0.09% (control; no crystalline Hyp added)	Whole-body crude lipid (% fresh weight)	13.57 ± 2.32	11.04 ± 0.87	3	3	Hedges' g (SE-to-SD; CI based on independent tanks)	1.16	-0.63 to 2.94	Higher measured value with hydroxyproline; 95% CI included 0 (imprecise estimate)	Not applicable - effect size estimated; see Notes for subsampling treatment
Cao et al. (2022)	Improved triploid crucian carp (Carassius auratus triploid)	Hydroxyproline supplementation in a plant-protein-based diet	Hyp 1.90% (2.00% crystalline Hyp added)	Hyp 0.09% (control; no crystalline Hyp added)	Whole-body ash (% fresh weight)	2.58 ± 0.14	2.85 ± 0.28	3	3	Hedges' g (SE-to-SD; CI based on independent tanks)	-0.99	-2.73 to 0.75	Lower measured value with hydroxyproline; 95% CI included 0 (imprecise estimate)	Not applicable - effect size estimated; see Notes for subsampling treatment
Cao et al. (2022)	Improved triploid crucian carp (Carassius auratus triploid)	Hydroxyproline supplementation in a plant-protein-based diet	Hyp 1.90% (2.00% crystalline Hyp added)	Hyp 0.09% (control; no crystalline Hyp added)	Whole-body moisture (% fresh weight)	67.30 ± 2.11	69.85 ± 0.42	3	3	Hedges' g (SE-to-SD; CI based on independent tanks)	-1.34	-3.19 to 0.51	Lower measured value with hydroxyproline; 95% CI included 0 (imprecise estimate)	Not applicable - effect size estimated; see Notes for subsampling treatment
Cao et al. (2022)	Improved triploid crucian carp (Carassius auratus triploid)	Hydroxyproline supplementation in a plant-protein-based diet	Hyp 1.90% (2.00% crystalline Hyp added)	Hyp 0.09% (control; no crystalline Hyp added)	Dorsal-muscle crude protein (% fresh weight)	17.26 ± 0.95	17.06 ± 0.21	3	3	Hedges' g (SE-to-SD; CI based on independent tanks)	0.23	-1.38 to 1.84	Higher measured value with hydroxyproline; 95% CI included 0 (imprecise estimate)	Not applicable - effect size estimated; see Notes for subsampling treatment
Cao et al. (2022)	Improved triploid crucian carp (Carassius auratus triploid)	Hydroxyproline supplementation in a plant-protein-based diet	Hyp 1.90% (2.00% crystalline Hyp added)	Hyp 0.09% (control; no crystalline Hyp added)	Dorsal-muscle crude lipid (% fresh weight)	1.20 ± 0.07	1.32 ± 0.12	3	3	Hedges' g (SE-to-SD; CI based on independent tanks)	-0.97	-2.71 to 0.76	Lower measured value with hydroxyproline; 95% CI included 0 (imprecise estimate)	Not applicable - effect size estimated; see Notes for subsampling treatment
Cao et al. (2022)	Improved triploid crucian carp (Carassius auratus triploid)	Hydroxyproline supplementation in a plant-protein-based diet	Hyp 1.90% (2.00% crystalline Hyp added)	Hyp 0.09% (control; no crystalline Hyp added)	Dorsal-muscle ash (% fresh weight)	1.51 ± 0.02	1.44 ± 0.10	3	3	Hedges' g (SE-to-SD; CI based on independent tanks)	0.75	-0.93 to 2.43	Higher measured value with hydroxyproline; 95% CI included 0 (imprecise estimate)	Not applicable - effect size estimated; see Notes for subsampling treatment

Study	Species	Strategy	Intervention	Comparator	Outcome and unit	Intervention mean ± SD	Comparator mean ± SD	n intervention (independent tanks)	n comparator (independent tanks)	Effect-size metric	Effect size	95% CI	Interpretation	Reason not estimated
Cao et al. (2022)	Improved triploid crucian carp (Carassius auratus triploid)	Hydroxyproline supplementation in a plant-protein-based diet	Hyp 1.90% (2.00% crystalline Hyp added)	Hyp 0.09% (control; no crystalline Hyp added)	Dorsal-muscle moisture (% fresh weight)	75.69 ± 1.30	75.65 ± 0.16	3	3	Hedges' g (SE-to-SD; CI based on independent tanks)	0.03	-1.57 to 1.64	Higher measured value with hydroxyproline; 95% CI included 0 (imprecise estimate)	Not applicable - effect size estimated; see Notes for subsampling treatment
Cao et al. (2022)	Improved triploid crucian carp (Carassius auratus triploid)	Hydroxyproline supplementation in a plant-protein-based diet	Hyp 1.90% (2.00% crystalline Hyp added)	Hyp 0.09% (control; no crystalline Hyp added)	Threonine (% dry matter)	2.65 ± 0.07	2.62 ± 0.16	3	3	Hedges' g (SE-to-SD; CI based on independent tanks)	0.2	-1.41 to 1.81	Higher measured value with hydroxyproline; 95% CI included 0 (imprecise estimate)	Not applicable - effect size estimated; see Notes for subsampling treatment
Cao et al. (2022)	Improved triploid crucian carp (Carassius auratus triploid)	Hydroxyproline supplementation in a plant-protein-based diet	Hyp 1.90% (2.00% crystalline Hyp added)	Hyp 0.09% (control; no crystalline Hyp added)	Phenylalanine (% dry matter)	2.95 ± 0.14	2.95 ± 0.19	3	3	Hedges' g (SE-to-SD; CI based on independent tanks)	0.0	-1.60 to 1.60	No observed mean difference; 95% CI included 0 (imprecise estimate)	Not applicable - effect size estimated; see Notes for subsampling treatment
Cao et al. (2022)	Improved triploid crucian carp (Carassius auratus triploid)	Hydroxyproline supplementation in a plant-protein-based diet	Hyp 1.90% (2.00% crystalline Hyp added)	Hyp 0.09% (control; no crystalline Hyp added)	Valine (% dry matter)	2.97 ± 0.12	2.98 ± 0.19	3	3	Hedges' g (SE-to-SD; CI based on independent tanks)	-0.05	-1.65 to 1.55	Lower measured value with hydroxyproline; 95% CI included 0 (imprecise estimate)	Not applicable - effect size estimated; see Notes for subsampling treatment
Cao et al. (2022)	Improved triploid crucian carp (Carassius auratus triploid)	Hydroxyproline supplementation in a plant-protein-based diet	Hyp 1.90% (2.00% crystalline Hyp added)	Hyp 0.09% (control; no crystalline Hyp added)	Isoleucine (% dry matter)	3.02 ± 0.14	3.03 ± 0.17	3	3	Hedges' g (SE-to-SD; CI based on independent tanks)	-0.05	-1.65 to 1.55	Lower measured value with hydroxyproline; 95% CI included 0 (imprecise estimate)	Not applicable - effect size estimated; see Notes for subsampling treatment
Cao et al. (2022)	Improved triploid crucian carp (Carassius auratus triploid)	Hydroxyproline supplementation in a plant-protein-based diet	Hyp 1.90% (2.00% crystalline Hyp added)	Hyp 0.09% (control; no crystalline Hyp added)	Leucine (% dry matter)	5.34 ± 0.35	5.36 ± 0.31	3	3	Hedges' g (SE-to-SD; CI based on independent tanks)	-0.05	-1.65 to 1.55	Lower measured value with hydroxyproline; 95% CI included 0 (imprecise estimate)	Not applicable - effect size estimated; see Notes for subsampling treatment
Cao et al. (2022)	Improved triploid crucian carp (Carassius auratus triploid)	Hydroxyproline supplementation in a plant-protein-based diet	Hyp 1.90% (2.00% crystalline Hyp added)	Hyp 0.09% (control; no crystalline Hyp added)	Methionine (% dry matter)	1.56 ± 0.47	1.72 ± 0.05	3	3	Hedges' g (SE-to-SD; CI based on independent tanks)	-0.38	-2.01 to 1.24	Lower measured value with hydroxyproline; 95% CI included 0 (imprecise estimate)	Not applicable - effect size estimated; see Notes for subsampling treatment
Cao et al. (2022)	Improved triploid crucian carp (Carassius auratus triploid)	Hydroxyproline supplementation in a plant-protein-based diet	Hyp 1.90% (2.00% crystalline Hyp added)	Hyp 0.09% (control; no crystalline Hyp added)	Arginine (% dry matter)	3.71 ± 0.10	3.67 ± 0.23	3	3	Hedges' g (SE-to-SD; CI based on independent tanks)	0.18	-1.42 to 1.79	Higher measured value with hydroxyproline; 95% CI included 0 (imprecise estimate)	Not applicable - effect size estimated; see Notes for subsampling treatment
Cao et al. (2022)	Improved triploid crucian carp (Carassius auratus triploid)	Hydroxyproline supplementation in a plant-protein-based diet	Hyp 1.90% (2.00% crystalline Hyp added)	Hyp 0.09% (control; no crystalline Hyp added)	Lysine (% dry matter)	6.17 ± 0.71	6.01 ± 0.50	3	3	Hedges' g (SE-to-SD; CI based on independent tanks)	0.21	-1.40 to 1.81	Higher measured value with hydroxyproline; 95% CI included 0 (imprecise estimate)	Not applicable - effect size estimated; see Notes for subsampling treatment
Cao et al. (2022)	Improved triploid crucian carp (Carassius auratus triploid)	Hydroxyproline supplementation in a plant-protein-based diet	Hyp 1.90% (2.00% crystalline Hyp added)	Hyp 0.09% (control; no crystalline Hyp added)	Histidine (% dry matter)	3.16 ± 0.09	3.21 ± 0.24	3	3	Hedges' g (SE-to-SD; CI based on independent tanks)	-0.22	-1.83 to 1.39	Lower measured value with hydroxyproline; 95% CI included 0 (imprecise estimate)	Not applicable - effect size estimated; see Notes for subsampling treatment
Cao et al. (2022)	Improved triploid crucian carp (Carassius auratus triploid)	Hydroxyproline supplementation in a plant-protein-based diet	Hyp 1.90% (2.00% crystalline Hyp added)	Hyp 0.09% (control; no crystalline Hyp added)	Aspartic acid (% dry matter)	6.85 ± 0.55	6.84 ± 0.43	3	3	Hedges' g (SE-to-SD; CI based on independent tanks)	0.02	-1.58 to 1.62	Higher measured value with hydroxyproline; 95% CI included 0 (imprecise estimate)	Not applicable - effect size estimated; see Notes for subsampling treatment
Cao et al. (2022)	Improved triploid crucian carp (Carassius auratus triploid)	Hydroxyproline supplementation in a plant-protein-based diet	Hyp 1.90% (2.00% crystalline Hyp added)	Hyp 0.09% (control; no crystalline Hyp added)	Serine (% dry matter)	2.72 ± 0.07	2.74 ± 0.16	3	3	Hedges' g (SE-to-SD; CI based on independent tanks)	-0.13	-1.74 to 1.47	Lower measured value with hydroxyproline; 95% CI included 0 (imprecise estimate)	Not applicable - effect size estimated; see Notes for subsampling treatment
Cao et al. (2022)	Improved triploid crucian carp (Carassius auratus triploid)	Hydroxyproline supplementation in a plant-protein-based diet	Hyp 1.90% (2.00% crystalline Hyp added)	Hyp 0.09% (control; no crystalline Hyp added)	Glutamic acid (% dry matter)	9.71 ± 0.83	9.74 ± 0.57	3	3	Hedges' g (SE-to-SD; CI based on independent tanks)	-0.03	-1.63 to 1.57	Lower measured value with hydroxyproline; 95% CI included 0 (imprecise estimate)	Not applicable - effect size estimated; see Notes for subsampling treatment
Cao et al. (2022)	Improved triploid crucian carp (Carassius auratus triploid)	Hydroxyproline supplementation in a plant-protein-based diet	Hyp 1.90% (2.00% crystalline Hyp added)	Hyp 0.09% (control; no crystalline Hyp added)	Glycine (% dry matter)	2.93 ± 0.02	2.93 ± 0.10	3	3	Hedges' g (SE-to-SD; CI based on independent tanks)	0.0	-1.60 to 1.60	No observed mean difference; 95% CI included 0 (imprecise estimate)	Not applicable - effect size estimated; see Notes for subsampling treatment
Cao et al. (2022)	Improved triploid crucian carp (Carassius auratus triploid)	Hydroxyproline supplementation in a plant-protein-based diet	Hyp 1.90% (2.00% crystalline Hyp added)	Hyp 0.09% (control; no crystalline Hyp added)	Alanine (% dry matter)	3.26 ± 0.14	3.27 ± 0.17	3	3	Hedges' g (SE-to-SD; CI based on independent tanks)	-0.05	-1.65 to 1.55	Lower measured value with hydroxyproline; 95% CI included 0 (imprecise estimate)	Not applicable - effect size estimated; see Notes for subsampling treatment
Cao et al. (2022)	Improved triploid crucian carp (Carassius auratus triploid)	Hydroxyproline supplementation in a plant-protein-based diet	Hyp 1.90% (2.00% crystalline Hyp added)	Hyp 0.09% (control; no crystalline Hyp added)	Tyrosine (% dry matter)	2.16 ± 0.03	2.15 ± 0.14	3	3	Hedges' g (SE-to-SD; CI based on independent tanks)	0.08	-1.52 to 1.68	Higher measured value with hydroxyproline; 95% CI included 0 (imprecise estimate)	Not applicable - effect size estimated; see Notes for subsampling treatment
Cao et al. (2022)	Improved triploid crucian carp (Carassius auratus triploid)	Hydroxyproline supplementation in a plant-protein-based diet	Hyp 1.90% (2.00% crystalline Hyp added)	Hyp 0.09% (control; no crystalline Hyp added)	Proline (% dry matter)	2.06 ± 0.07	2.10 ± 0.09	3	3	Hedges' g (SE-to-SD; CI based on independent tanks)	-0.41	-2.03 to 1.22	Lower measured value with hydroxyproline; 95% CI included 0 (imprecise estimate)	Not applicable - effect size estimated; see Notes for subsampling treatment
Cao et al. (2022)	Improved triploid crucian carp (Carassius auratus triploid)	Hydroxyproline supplementation in a plant-protein-based diet	Hyp 1.90% (2.00% crystalline Hyp added)	Hyp 0.09% (control; no crystalline Hyp added)	Hardness (N)	4.30 ± 0.33	3.67 ± 0.51	3	3	Hedges' g (SE-to-SD; CI based on independent tanks)	1.17	-0.62 to 2.97	Higher, more favorable value with hydroxyproline; 95% CI included 0 (imprecise estimate)	Not applicable - effect size estimated; see Notes for subsampling treatment
Cao et al. (2022)	Improved triploid crucian carp (Carassius auratus triploid)	Hydroxyproline supplementation in a plant-protein-based diet	Hyp 1.90% (2.00% crystalline Hyp added)	Hyp 0.09% (control; no crystalline Hyp added)	Springiness (mm)	0.600 ± 0.120	0.510 ± 0.120	3	3	Hedges' g (SE-to-SD; CI based on independent tanks)	0.6	-1.05 to 2.25	Higher, more favorable value with hydroxyproline; 95% CI included 0 (imprecise estimate)	Not applicable - effect size estimated; see Notes for subsampling treatment
Cao et al. (2022)	Improved triploid crucian carp (Carassius auratus triploid)	Hydroxyproline supplementation in a plant-protein-based diet	Hyp 1.90% (2.00% crystalline Hyp added)	Hyp 0.09% (control; no crystalline Hyp added)	Chewiness (mJ)	0.670 ± 0.300	0.600 ± 0.210	3	3	Hedges' g (SE-to-SD; CI based on independent tanks)	0.22	-1.39 to 1.82	Higher, more favorable value with hydroxyproline; 95% CI included 0 (imprecise estimate)	Not applicable - effect size estimated; see Notes for subsampling treatment
Cao et al. (2022)	Improved triploid crucian carp (Carassius auratus triploid)	Hydroxyproline supplementation in a plant-protein-based diet	Hyp 1.90% (2.00% crystalline Hyp added)	Hyp 0.09% (control; no crystalline Hyp added)	Adhesiveness (N·mm)	0.031 ± 0.015	0.031 ± 0.012	3	3	Hedges' g (SE-to-SD; CI based on independent tanks)	0.0	-1.60 to 1.60	No observed mean difference; 95% CI included 0 (imprecise estimate)	Not applicable - effect size estimated; see Notes for subsampling treatment
Cao et al. (2022)	Improved triploid crucian carp (Carassius auratus triploid)	Hydroxyproline supplementation in a plant-protein-based diet	Hyp 1.90% (2.00% crystalline Hyp added)	Hyp 0.09% (control; no crystalline Hyp added)	Cohesiveness (%)	0.370 ± 0.090	0.380 ± 0.090	3	3	Hedges' g (SE-to-SD; CI based on independent tanks)	-0.09	-1.69 to 1.51	Lower measured value with hydroxyproline; 95% CI included 0 (imprecise estimate)	Not applicable - effect size estimated; see Notes for subsampling treatment
Cao et al. (2022)	Improved triploid crucian carp (Carassius auratus triploid)	Hydroxyproline supplementation in a plant-protein-based diet	Hyp 1.90% (2.00% crystalline Hyp added)	Hyp 0.09% (control; no crystalline Hyp added)	Gumminess (g·mm)	1.13 ± 0.48	1.04 ± 0.36	3	3	Hedges' g (SE-to-SD; CI based on independent tanks)	0.17	-1.43 to 1.77	Higher, more favorable value with hydroxyproline; 95% CI included 0 (imprecise estimate)	Not applicable - effect size estimated; see Notes for subsampling treatment
Cao et al. (2022)	Improved triploid crucian carp (Carassius auratus triploid)	Hydroxyproline supplementation in a plant-protein-based diet	Hyp 1.90% (2.00% crystalline Hyp added)	Hyp 0.09% (control; no crystalline Hyp added)	Muscle hydroxyproline (mg/g)	1.44 ± 0.59	0.910 ± 0.245	3	3	Hedges' g (SE-to-SD; CI based on independent tanks)	0.94	-0.79 to 2.67	Higher, more favorable value with hydroxyproline; 95% CI included 0 (imprecise estimate)	Not applicable - effect size estimated; see Notes for subsampling treatment
Cao et al. (2022)	Improved triploid crucian carp (Carassius auratus triploid)	Hydroxyproline supplementation in a plant-protein-based diet	Hyp 1.90% (2.00% crystalline Hyp added)	Hyp 0.09% (control; no crystalline Hyp added)	Skin hydroxyproline (mg/g)	13.99 ± 3.72	8.77 ± 2.28	3	3	Hedges' g (SE-to-SD; CI based on independent tanks)	1.35	-0.50 to 3.21	Higher, more favorable value with hydroxyproline; 95% CI included 0 (imprecise estimate)	Not applicable - effect size estimated; see Notes for subsampling treatment
Cao et al. (2022)	Improved triploid crucian carp (Carassius auratus triploid)	Hydroxyproline supplementation in a plant-protein-based diet	Hyp 1.90% (2.00% crystalline Hyp added)	Hyp 0.09% (control; no crystalline Hyp added)	Liver hydroxyproline (μg/g)	456.84 ± 180.99	256.88 ± 13.94	3	3	Hedges' g (SE-to-SD; CI based on independent tanks)	1.25	-0.57 to 3.06	Higher, more favorable value with hydroxyproline; 95% CI included 0 (imprecise estimate)	Not applicable - effect size estimated; see Notes for subsampling treatment
Cao et al. (2022)	Improved triploid crucian carp (Carassius auratus triploid)	Hydroxyproline supplementation in a plant-protein-based diet	Hyp 1.90% (2.00% crystalline Hyp added)	Hyp 0.09% (control; no crystalline Hyp added)	Vertebral hydroxyproline (mg/g)	10.50 ± 0.98	7.39 ± 0.42	3	3	Hedges' g (SE-to-SD; CI based on independent tanks)	3.31	0.51 to 6.10	Higher, more favorable value with hydroxyproline; 95% CI excluded 0	Not applicable - effect size estimated; see Notes for subsampling treatment
Cao et al. (2022)	Improved triploid crucian carp (Carassius auratus triploid)	Hydroxyproline supplementation in a plant-protein-based diet	Hyp 1.90% (2.00% crystalline Hyp added)	Hyp 0.09% (control; no crystalline Hyp added)	Plasma hydroxyproline (mg/mL)	170.63 ± 22.93	100.00 ± 23.56	3	3	Hedges' g (SE-to-SD; CI based on independent tanks)	2.43	0.11 to 4.75	Higher, more favorable value with hydroxyproline; 95% CI excluded 0	Not applicable - effect size estimated; see Notes for subsampling treatment
Cao et al. (2022)	Improved triploid crucian carp (Carassius auratus triploid)	Hydroxyproline supplementation in a plant-protein-based diet	Hyp 1.90% (2.00% crystalline Hyp added)	Hyp 0.09% (control; no crystalline Hyp added)	Type I collagen (ng/mg protein)	5.98 ± 0.66	4.88 ± 0.05	3	3	Hedges' g (SE-to-SD; CI based on independent tanks)	1.88	-0.19 to 3.94	Higher, more favorable value with hydroxyproline; 95% CI included 0 (imprecise estimate)	Not applicable - effect size estimated; see Notes for subsampling treatment
Cao et al. (2022)	Improved triploid crucian carp (Carassius auratus triploid)	Hydroxyproline supplementation in a plant-protein-based diet	Hyp 1.90% (2.00% crystalline Hyp added)	Hyp 0.09% (control; no crystalline Hyp added)	Pyridinium crosslink (ng/mg protein)	30.79 ± 2.89	25.74 ± 2.60	3	3	Hedges' g (SE-to-SD; CI based on independent tanks)	1.47	-0.43 to 3.37	Higher, more favorable value with hydroxyproline; 95% CI included 0 (imprecise estimate)	Not applicable - effect size estimated; see Notes for subsampling treatment
Cao et al. (2022)	Improved triploid crucian carp (Carassius auratus triploid)	Hydroxyproline supplementation in a plant-protein-based diet	Hyp 1.90% (2.00% crystalline Hyp added)	Hyp 0.09% (control; no crystalline Hyp added)	Prolyl 4-hydroxylase activity (ng/mg protein)	0.840 ± 0.122	0.660 ± 0.073	3	3	Hedges' g (SE-to-SD; CI based on independent tanks)	1.43	-0.45 to 3.31	Higher, more favorable value with hydroxyproline; 95% CI included 0 (imprecise estimate)	Not applicable - effect size estimated; see Notes for subsampling treatment

Study	Species	Strategy	Intervention	Comparator	Outcome and unit	Intervention mean ± SD	Comparator mean ± SD	n intervention (independent tanks)	n comparator (independent tanks)	Effect-size metric	Effect size	95% CI	Interpretation	Reason not estimated
Cao et al. (2022)	Improved triploid crucian carp (Carassius auratus triploid)	Hydroxyproline supplementation in a plant-protein-based diet	Hyp 1.90% (2.00% crystalline Hyp added)	Hyp 0.09% (control; no crystalline Hyp added)	Lysyl hydroxylase activity (ng/mg protein)	0.161 ± 0.032	0.136 ± 0.012	3	3	Hedges' g (SE-to-SD; CI based on independent tanks)	0.83	-0.87 to 2.53	Higher, more favorable value with hydroxyproline; 95% CI included 0 (imprecise estimate)	Not applicable - effect size estimated; see Notes for subsampling treatment
Cao et al. (2022)	Improved triploid crucian carp (Carassius auratus triploid)	Hydroxyproline supplementation in a plant-protein-based diet	Hyp 1.90% (2.00% crystalline Hyp added)	Hyp 0.09% (control; no crystalline Hyp added)	Muscle fiber diameter (μm)	49.14 ± 4.38	47.49 ± 4.75	3	3	Hedges' g (SE-to-SD; CI based on independent tanks)	0.29	-1.32 to 1.90	Higher, more favorable value with hydroxyproline; 95% CI included 0 (imprecise estimate)	Not applicable - effect size estimated; see Notes for subsampling treatment
Cao et al. (2022)	Improved triploid crucian carp (Carassius auratus triploid)	Hydroxyproline supplementation in a plant-protein-based diet	Hyp 1.90% (2.00% crystalline Hyp added)	Hyp 0.09% (control; no crystalline Hyp added)	Muscle fiber density (n/mm^2)	167.67 ± 14.84	158.67 ± 15.77	3	3	Hedges' g (SE-to-SD; CI based on independent tanks)	0.47	-1.16 to 2.10	Higher, more favorable value with hydroxyproline; 95% CI included 0 (imprecise estimate)	Not applicable - effect size estimated; see Notes for subsampling treatment
Cao et al. (2022)	Improved triploid crucian carp (Carassius auratus triploid)	Hydroxyproline supplementation in a plant-protein-based diet	Hyp 0.39% (0.40% crystalline Hyp added)	Hyp 0.09% (control; no crystalline Hyp added)	Muscle col1a1 mRNA expression (Figure 3)	Not reported numerically	Not reported numerically	3	3	Not estimable	Not estimable	Not estimable	The outcome was presented graphically with significance annotations; an exact pairwise effect size was not calculated	Exact group means and SEs were presented only in Figures 3-5 and were not reliably extractable
Cao et al. (2022)	Improved triploid crucian carp (Carassius auratus triploid)	Hydroxyproline supplementation in a plant-protein-based diet	Hyp 0.39% (0.40% crystalline Hyp added)	Hyp 0.09% (control; no crystalline Hyp added)	Muscle col1a2 mRNA expression (Figure 3)	Not reported numerically	Not reported numerically	3	3	Not estimable	Not estimable	Not estimable	The outcome was presented graphically with significance annotations; an exact pairwise effect size was not calculated	Exact group means and SEs were presented only in Figures 3-5 and were not reliably extractable
Cao et al. (2022)	Improved triploid crucian carp (Carassius auratus triploid)	Hydroxyproline supplementation in a plant-protein-based diet	Hyp 0.39% (0.40% crystalline Hyp added)	Hyp 0.09% (control; no crystalline Hyp added)	Muscle p4hα1 mRNA expression (Figure 3)	Not reported numerically	Not reported numerically	3	3	Not estimable	Not estimable	Not estimable	The outcome was presented graphically with significance annotations; an exact pairwise effect size was not calculated	Exact group means and SEs were presented only in Figures 3-5 and were not reliably extractable
Cao et al. (2022)	Improved triploid crucian carp (Carassius auratus triploid)	Hydroxyproline supplementation in a plant-protein-based diet	Hyp 0.39% (0.40% crystalline Hyp added)	Hyp 0.09% (control; no crystalline Hyp added)	Muscle p4hβ mRNA expression (Figure 3)	Not reported numerically	Not reported numerically	3	3	Not estimable	Not estimable	Not estimable	The outcome was presented graphically with significance annotations; an exact pairwise effect size was not calculated	Exact group means and SEs were presented only in Figures 3-5 and were not reliably extractable
Cao et al. (2022)	Improved triploid crucian carp (Carassius auratus triploid)	Hydroxyproline supplementation in a plant-protein-based diet	Hyp 0.39% (0.40% crystalline Hyp added)	Hyp 0.09% (control; no crystalline Hyp added)	Muscle smad4 mRNA expression (Figure 4)	Not reported numerically	Not reported numerically	3	3	Not estimable	Not estimable	Not estimable	The outcome was presented graphically with significance annotations; an exact pairwise effect size was not calculated	Exact group means and SEs were presented only in Figures 3-5 and were not reliably extractable
Cao et al. (2022)	Improved triploid crucian carp (Carassius auratus triploid)	Hydroxyproline supplementation in a plant-protein-based diet	Hyp 0.39% (0.40% crystalline Hyp added)	Hyp 0.09% (control; no crystalline Hyp added)	Muscle smad5 mRNA expression (Figure 4)	Not reported numerically	Not reported numerically	3	3	Not estimable	Not estimable	Not estimable	The outcome was presented graphically with significance annotations; an exact pairwise effect size was not calculated	Exact group means and SEs were presented only in Figures 3-5 and were not reliably extractable
Cao et al. (2022)	Improved triploid crucian carp (Carassius auratus triploid)	Hydroxyproline supplementation in a plant-protein-based diet	Hyp 0.39% (0.40% crystalline Hyp added)	Hyp 0.09% (control; no crystalline Hyp added)	Muscle smad9 mRNA expression (Figure 4)	Not reported numerically	Not reported numerically	3	3	Not estimable	Not estimable	Not estimable	The outcome was presented graphically with significance annotations; an exact pairwise effect size was not calculated	Exact group means and SEs were presented only in Figures 3-5 and were not reliably extractable
Cao et al. (2022)	Improved triploid crucian carp (Carassius auratus triploid)	Hydroxyproline supplementation in a plant-protein-based diet	Hyp 0.39% (0.40% crystalline Hyp added)	Hyp 0.09% (control; no crystalline Hyp added)	Muscle tgf-β mRNA expression (Figure 4)	Not reported numerically	Not reported numerically	3	3	Not estimable	Not estimable	Not estimable	The outcome was presented graphically with significance annotations; an exact pairwise effect size was not calculated	Exact group means and SEs were presented only in Figures 3-5 and were not reliably extractable
Cao et al. (2022)	Improved triploid crucian carp (Carassius auratus triploid)	Hydroxyproline supplementation in a plant-protein-based diet	Hyp 0.39% (0.40% crystalline Hyp added)	Hyp 0.09% (control; no crystalline Hyp added)	Muscle igf-1 mRNA expression (Figure 5)	Not reported numerically	Not reported numerically	3	3	Not estimable	Not estimable	Not estimable	The outcome was presented graphically with significance annotations; an exact pairwise effect size was not calculated	Exact group means and SEs were presented only in Figures 3-5 and were not reliably extractable
Cao et al. (2022)	Improved triploid crucian carp (Carassius auratus triploid)	Hydroxyproline supplementation in a plant-protein-based diet	Hyp 0.39% (0.40% crystalline Hyp added)	Hyp 0.09% (control; no crystalline Hyp added)	Muscle tor mRNA expression (Figure 5)	Not reported numerically	Not reported numerically	3	3	Not estimable	Not estimable	Not estimable	The outcome was presented graphically with significance annotations; an exact pairwise effect size was not calculated	Exact group means and SEs were presented only in Figures 3-5 and were not reliably extractable
Cao et al. (2022)	Improved triploid crucian carp (Carassius auratus triploid)	Hydroxyproline supplementation in a plant-protein-based diet	Hyp 0.39% (0.40% crystalline Hyp added)	Hyp 0.09% (control; no crystalline Hyp added)	Muscle myod mRNA expression (Figure 5)	Not reported numerically	Not reported numerically	3	3	Not estimable	Not estimable	Not estimable	The outcome was presented graphically with significance annotations; an exact pairwise effect size was not calculated	Exact group means and SEs were presented only in Figures 3-5 and were not reliably extractable
Cao et al. (2022)	Improved triploid crucian carp (Carassius auratus triploid)	Hydroxyproline supplementation in a plant-protein-based diet	Hyp 0.39% (0.40% crystalline Hyp added)	Hyp 0.09% (control; no crystalline Hyp added)	Muscle myf5 mRNA expression (Figure 5)	Not reported numerically	Not reported numerically	3	3	Not estimable	Not estimable	Not estimable	The outcome was presented graphically with significance annotations; an exact pairwise effect size was not calculated	Exact group means and SEs were presented only in Figures 3-5 and were not reliably extractable
Cao et al. (2022)	Improved triploid crucian carp (Carassius auratus triploid)	Hydroxyproline supplementation in a plant-protein-based diet	Hyp 0.39% (0.40% crystalline Hyp added)	Hyp 0.09% (control; no crystalline Hyp added)	Muscle myhc mRNA expression (Figure 5)	Not reported numerically	Not reported numerically	3	3	Not estimable	Not estimable	Not estimable	The outcome was presented graphically with significance annotations; an exact pairwise effect size was not calculated	Exact group means and SEs were presented only in Figures 3-5 and were not reliably extractable
Cao et al. (2022)	Improved triploid crucian carp (Carassius auratus triploid)	Hydroxyproline supplementation in a plant-protein-based diet	Hyp 0.76% (0.80% crystalline Hyp added)	Hyp 0.09% (control; no crystalline Hyp added)	Muscle col1a1 mRNA expression (Figure 3)	Not reported numerically	Not reported numerically	3	3	Not estimable	Not estimable	Not estimable	The outcome was presented graphically with significance annotations; an exact pairwise effect size was not calculated	Exact group means and SEs were presented only in Figures 3-5 and were not reliably extractable
Cao et al. (2022)	Improved triploid crucian carp (Carassius auratus triploid)	Hydroxyproline supplementation in a plant-protein-based diet	Hyp 0.76% (0.80% crystalline Hyp added)	Hyp 0.09% (control; no crystalline Hyp added)	Muscle col1a2 mRNA expression (Figure 3)	Not reported numerically	Not reported numerically	3	3	Not estimable	Not estimable	Not estimable	The outcome was presented graphically with significance annotations; an exact pairwise effect size was not calculated	Exact group means and SEs were presented only in Figures 3-5 and were not reliably extractable
Cao et al. (2022)	Improved triploid crucian carp (Carassius auratus triploid)	Hydroxyproline supplementation in a plant-protein-based diet	Hyp 0.76% (0.80% crystalline Hyp added)	Hyp 0.09% (control; no crystalline Hyp added)	Muscle p4hα1 mRNA expression (Figure 3)	Not reported numerically	Not reported numerically	3	3	Not estimable	Not estimable	Not estimable	The outcome was presented graphically with significance annotations; an exact pairwise effect size was not calculated	Exact group means and SEs were presented only in Figures 3-5 and were not reliably extractable
Cao et al. (2022)	Improved triploid crucian carp (Carassius auratus triploid)	Hydroxyproline supplementation in a plant-protein-based diet	Hyp 0.76% (0.80% crystalline Hyp added)	Hyp 0.09% (control; no crystalline Hyp added)	Muscle p4hβ mRNA expression (Figure 3)	Not reported numerically	Not reported numerically	3	3	Not estimable	Not estimable	Not estimable	The outcome was presented graphically with significance annotations; an exact pairwise effect size was not calculated	Exact group means and SEs were presented only in Figures 3-5 and were not reliably extractable
Cao et al. (2022)	Improved triploid crucian carp (Carassius auratus triploid)	Hydroxyproline supplementation in a plant-protein-based diet	Hyp 0.76% (0.80% crystalline Hyp added)	Hyp 0.09% (control; no crystalline Hyp added)	Muscle smad4 mRNA expression (Figure 4)	Not reported numerically	Not reported numerically	3	3	Not estimable	Not estimable	Not estimable	The outcome was presented graphically with significance annotations; an exact pairwise effect size was not calculated	Exact group means and SEs were presented only in Figures 3-5 and were not reliably extractable
Cao et al. (2022)	Improved triploid crucian carp (Carassius auratus triploid)	Hydroxyproline supplementation in a plant-protein-based diet	Hyp 0.76% (0.80% crystalline Hyp added)	Hyp 0.09% (control; no crystalline Hyp added)	Muscle smad5 mRNA expression (Figure 4)	Not reported numerically	Not reported numerically	3	3	Not estimable	Not estimable	Not estimable	The outcome was presented graphically with significance annotations; an exact pairwise effect size was not calculated	Exact group means and SEs were presented only in Figures 3-5 and were not reliably extractable
Cao et al. (2022)	Improved triploid crucian carp (Carassius auratus triploid)	Hydroxyproline supplementation in a plant-protein-based diet	Hyp 0.76% (0.80% crystalline Hyp added)	Hyp 0.09% (control; no crystalline Hyp added)	Muscle smad9 mRNA expression (Figure 4)	Not reported numerically	Not reported numerically	3	3	Not estimable	Not estimable	Not estimable	The outcome was presented graphically with significance annotations; an exact pairwise effect size was not calculated	Exact group means and SEs were presented only in Figures 3-5 and were not reliably extractable
Cao et al. (2022)	Improved triploid crucian carp (Carassius auratus triploid)	Hydroxyproline supplementation in a plant-protein-based diet	Hyp 0.76% (0.80% crystalline Hyp added)	Hyp 0.09% (control; no crystalline Hyp added)	Muscle tgf-β mRNA expression (Figure 4)	Not reported numerically	Not reported numerically	3	3	Not estimable	Not estimable	Not estimable	The outcome was presented graphically with significance annotations; an exact pairwise effect size was not calculated	Exact group means and SEs were presented only in Figures 3-5 and were not reliably extractable
Cao et al. (2022)	Improved triploid crucian carp (Carassius auratus triploid)	Hydroxyproline supplementation in a plant-protein-based diet	Hyp 0.76% (0.80% crystalline Hyp added)	Hyp 0.09% (control; no crystalline Hyp added)	Muscle igf-1 mRNA expression (Figure 5)	Not reported numerically	Not reported numerically	3	3	Not estimable	Not estimable	Not estimable	The outcome was presented graphically with significance annotations; an exact pairwise effect size was not calculated	Exact group means and SEs were presented only in Figures 3-5 and were not reliably extractable
Cao et al. (2022)	Improved triploid crucian carp (Carassius auratus triploid)	Hydroxyproline supplementation in a plant-protein-based diet	Hyp 0.76% (0.80% crystalline Hyp added)	Hyp 0.09% (control; no crystalline Hyp added)	Muscle tor mRNA expression (Figure 5)	Not reported numerically	Not reported numerically	3	3	Not estimable	Not estimable	Not estimable	The outcome was presented graphically with significance annotations; an exact pairwise effect size was not calculated	Exact group means and SEs were presented only in Figures 3-5 and were not reliably extractable
Cao et al. (2022)	Improved triploid crucian carp (Carassius auratus triploid)	Hydroxyproline supplementation in a plant-protein-based diet	Hyp 0.76% (0.80% crystalline Hyp added)	Hyp 0.09% (control; no crystalline Hyp added)	Muscle myod mRNA expression (Figure 5)	Not reported numerically	Not reported numerically	3	3	Not estimable	Not estimable	Not estimable	The outcome was presented graphically with significance annotations; an exact pairwise effect size was not calculated	Exact group means and SEs were presented only in Figures 3-5 and were not reliably extractable
Cao et al. (2022)	Improved triploid crucian carp (Carassius auratus triploid)	Hydroxyproline supplementation in a plant-protein-based diet	Hyp 0.76% (0.80% crystalline Hyp added)	Hyp 0.09% (control; no crystalline Hyp added)	Muscle myf5 mRNA expression (Figure 5)	Not reported numerically	Not reported numerically	3	3	Not estimable	Not estimable	Not estimable	The outcome was presented graphically with significance annotations; an exact pairwise effect size was not calculated	Exact group means and SEs were presented only in Figures 3-5 and were not reliably extractable
Cao et al. (2022)	Improved triploid crucian carp (Carassius auratus triploid)	Hydroxyproline supplementation in a plant-protein-based diet	Hyp 0.76% (0.80% crystalline Hyp added)	Hyp 0.09% (control; no crystalline Hyp added)	Muscle myhc mRNA expression (Figure 5)	Not reported numerically	Not reported numerically	3	3	Not estimable	Not estimable	Not estimable	The outcome was presented graphically with significance annotations; an exact pairwise effect size was not calculated	Exact group means and SEs were presented only in Figures 3-5 and were not reliably extractable
Cao et al. (2022)	Improved triploid crucian carp (Carassius auratus triploid)	Hydroxyproline supplementation in a plant-protein-based diet	Hyp 1.14% (1.20% crystalline Hyp added)	Hyp 0.09% (control; no crystalline Hyp added)	Muscle col1a1 mRNA expression (Figure 3)	Not reported numerically	Not reported numerically	3	3	Not estimable	Not estimable	Not estimable	The outcome was presented graphically with significance annotations; an exact pairwise effect size was not calculated	Exact group means and SEs were presented only in Figures 3-5 and were not reliably extractable
Cao et al. (2022)	Improved triploid crucian carp (Carassius auratus triploid)	Hydroxyproline supplementation in a plant-protein-based diet	Hyp 1.14% (1.20% crystalline Hyp added)	Hyp 0.09% (control; no crystalline Hyp added)	Muscle col1a2 mRNA expression (Figure 3)	Not reported numerically	Not reported numerically	3	3	Not estimable	Not estimable	Not estimable	The outcome was presented graphically with significance annotations; an exact pairwise effect size was not calculated	Exact group means and SEs were presented only in Figures 3-5 and were not reliably extractable

Study	Species	Strategy	Intervention	Comparator	Outcome and unit	Intervention mean ± SD	Comparator mean ± SD	n intervention (independent tanks)	n comparator (independent tanks)	Effect-size metric	Effect size	95% CI	Interpretation	Reason not estimated
Cao et al. (2022)	Improved triploid crucian carp (Carassius auratus triploid)	Hydroxyproline supplementation in a plant-protein-based diet	Hyp 1.14% (1.20% crystalline Hyp added)	Hyp 0.09% (control; no crystalline Hyp added)	Muscle p4hα1 mRNA expression (Figure 3)	Not reported numerically	Not reported numerically	3	3	Not estimable	Not estimable	Not estimable	The outcome was presented graphically with significance annotations; an exact pairwise effect size was not calculated	Exact group means and SEs were presented only in Figures 3-5 and were not reliably extractable
Cao et al. (2022)	Improved triploid crucian carp (Carassius auratus triploid)	Hydroxyproline supplementation in a plant-protein-based diet	Hyp 1.14% (1.20% crystalline Hyp added)	Hyp 0.09% (control; no crystalline Hyp added)	Muscle p4hβ mRNA expression (Figure 3)	Not reported numerically	Not reported numerically	3	3	Not estimable	Not estimable	Not estimable	The outcome was presented graphically with significance annotations; an exact pairwise effect size was not calculated	Exact group means and SEs were presented only in Figures 3-5 and were not reliably extractable
Cao et al. (2022)	Improved triploid crucian carp (Carassius auratus triploid)	Hydroxyproline supplementation in a plant-protein-based diet	Hyp 1.14% (1.20% crystalline Hyp added)	Hyp 0.09% (control; no crystalline Hyp added)	Muscle smad4 mRNA expression (Figure 4)	Not reported numerically	Not reported numerically	3	3	Not estimable	Not estimable	Not estimable	The outcome was presented graphically with significance annotations; an exact pairwise effect size was not calculated	Exact group means and SEs were presented only in Figures 3-5 and were not reliably extractable
Cao et al. (2022)	Improved triploid crucian carp (Carassius auratus triploid)	Hydroxyproline supplementation in a plant-protein-based diet	Hyp 1.14% (1.20% crystalline Hyp added)	Hyp 0.09% (control; no crystalline Hyp added)	Muscle smad5 mRNA expression (Figure 4)	Not reported numerically	Not reported numerically	3	3	Not estimable	Not estimable	Not estimable	The outcome was presented graphically with significance annotations; an exact pairwise effect size was not calculated	Exact group means and SEs were presented only in Figures 3-5 and were not reliably extractable
Cao et al. (2022)	Improved triploid crucian carp (Carassius auratus triploid)	Hydroxyproline supplementation in a plant-protein-based diet	Hyp 1.14% (1.20% crystalline Hyp added)	Hyp 0.09% (control; no crystalline Hyp added)	Muscle smad9 mRNA expression (Figure 4)	Not reported numerically	Not reported numerically	3	3	Not estimable	Not estimable	Not estimable	The outcome was presented graphically with significance annotations; an exact pairwise effect size was not calculated	Exact group means and SEs were presented only in Figures 3-5 and were not reliably extractable
Cao et al. (2022)	Improved triploid crucian carp (Carassius auratus triploid)	Hydroxyproline supplementation in a plant-protein-based diet	Hyp 1.14% (1.20% crystalline Hyp added)	Hyp 0.09% (control; no crystalline Hyp added)	Muscle tgf-β mRNA expression (Figure 4)	Not reported numerically	Not reported numerically	3	3	Not estimable	Not estimable	Not estimable	The outcome was presented graphically with significance annotations; an exact pairwise effect size was not calculated	Exact group means and SEs were presented only in Figures 3-5 and were not reliably extractable
Cao et al. (2022)	Improved triploid crucian carp (Carassius auratus triploid)	Hydroxyproline supplementation in a plant-protein-based diet	Hyp 1.14% (1.20% crystalline Hyp added)	Hyp 0.09% (control; no crystalline Hyp added)	Muscle igf-1 mRNA expression (Figure 5)	Not reported numerically	Not reported numerically	3	3	Not estimable	Not estimable	Not estimable	The outcome was presented graphically with significance annotations; an exact pairwise effect size was not calculated	Exact group means and SEs were presented only in Figures 3-5 and were not reliably extractable
Cao et al. (2022)	Improved triploid crucian carp (Carassius auratus triploid)	Hydroxyproline supplementation in a plant-protein-based diet	Hyp 1.14% (1.20% crystalline Hyp added)	Hyp 0.09% (control; no crystalline Hyp added)	Muscle tor mRNA expression (Figure 5)	Not reported numerically	Not reported numerically	3	3	Not estimable	Not estimable	Not estimable	The outcome was presented graphically with significance annotations; an exact pairwise effect size was not calculated	Exact group means and SEs were presented only in Figures 3-5 and were not reliably extractable
Cao et al. (2022)	Improved triploid crucian carp (Carassius auratus triploid)	Hydroxyproline supplementation in a plant-protein-based diet	Hyp 1.14% (1.20% crystalline Hyp added)	Hyp 0.09% (control; no crystalline Hyp added)	Muscle myod mRNA expression (Figure 5)	Not reported numerically	Not reported numerically	3	3	Not estimable	Not estimable	Not estimable	The outcome was presented graphically with significance annotations; an exact pairwise effect size was not calculated	Exact group means and SEs were presented only in Figures 3-5 and were not reliably extractable
Cao et al. (2022)	Improved triploid crucian carp (Carassius auratus triploid)	Hydroxyproline supplementation in a plant-protein-based diet	Hyp 1.14% (1.20% crystalline Hyp added)	Hyp 0.09% (control; no crystalline Hyp added)	Muscle myf5 mRNA expression (Figure 5)	Not reported numerically	Not reported numerically	3	3	Not estimable	Not estimable	Not estimable	The outcome was presented graphically with significance annotations; an exact pairwise effect size was not calculated	Exact group means and SEs were presented only in Figures 3-5 and were not reliably extractable
Cao et al. (2022)	Improved triploid crucian carp (Carassius auratus triploid)	Hydroxyproline supplementation in a plant-protein-based diet	Hyp 1.14% (1.20% crystalline Hyp added)	Hyp 0.09% (control; no crystalline Hyp added)	Muscle myhc mRNA expression (Figure 5)	Not reported numerically	Not reported numerically	3	3	Not estimable	Not estimable	Not estimable	The outcome was presented graphically with significance annotations; an exact pairwise effect size was not calculated	Exact group means and SEs were presented only in Figures 3-5 and were not reliably extractable
Cao et al. (2022)	Improved triploid crucian carp (Carassius auratus triploid)	Hydroxyproline supplementation in a plant-protein-based diet	Hyp 1.53% (1.60% crystalline Hyp added)	Hyp 0.09% (control; no crystalline Hyp added)	Muscle col1a1 mRNA expression (Figure 3)	Not reported numerically	Not reported numerically	3	3	Not estimable	Not estimable	Not estimable	The outcome was presented graphically with significance annotations; an exact pairwise effect size was not calculated	Exact group means and SEs were presented only in Figures 3-5 and were not reliably extractable
Cao et al. (2022)	Improved triploid crucian carp (Carassius auratus triploid)	Hydroxyproline supplementation in a plant-protein-based diet	Hyp 1.53% (1.60% crystalline Hyp added)	Hyp 0.09% (control; no crystalline Hyp added)	Muscle col1a2 mRNA expression (Figure 3)	Not reported numerically	Not reported numerically	3	3	Not estimable	Not estimable	Not estimable	The outcome was presented graphically with significance annotations; an exact pairwise effect size was not calculated	Exact group means and SEs were presented only in Figures 3-5 and were not reliably extractable
Cao et al. (2022)	Improved triploid crucian carp (Carassius auratus triploid)	Hydroxyproline supplementation in a plant-protein-based diet	Hyp 1.53% (1.60% crystalline Hyp added)	Hyp 0.09% (control; no crystalline Hyp added)	Muscle p4hα1 mRNA expression (Figure 3)	Not reported numerically	Not reported numerically	3	3	Not estimable	Not estimable	Not estimable	The outcome was presented graphically with significance annotations; an exact pairwise effect size was not calculated	Exact group means and SEs were presented only in Figures 3-5 and were not reliably extractable
Cao et al. (2022)	Improved triploid crucian carp (Carassius auratus triploid)	Hydroxyproline supplementation in a plant-protein-based diet	Hyp 1.53% (1.60% crystalline Hyp added)	Hyp 0.09% (control; no crystalline Hyp added)	Muscle p4hβ mRNA expression (Figure 3)	Not reported numerically	Not reported numerically	3	3	Not estimable	Not estimable	Not estimable	The outcome was presented graphically with significance annotations; an exact pairwise effect size was not calculated	Exact group means and SEs were presented only in Figures 3-5 and were not reliably extractable
Cao et al. (2022)	Improved triploid crucian carp (Carassius auratus triploid)	Hydroxyproline supplementation in a plant-protein-based diet	Hyp 1.53% (1.60% crystalline Hyp added)	Hyp 0.09% (control; no crystalline Hyp added)	Muscle smad4 mRNA expression (Figure 4)	Not reported numerically	Not reported numerically	3	3	Not estimable	Not estimable	Not estimable	The outcome was presented graphically with significance annotations; an exact pairwise effect size was not calculated	Exact group means and SEs were presented only in Figures 3-5 and were not reliably extractable
Cao et al. (2022)	Improved triploid crucian carp (Carassius auratus triploid)	Hydroxyproline supplementation in a plant-protein-based diet	Hyp 1.53% (1.60% crystalline Hyp added)	Hyp 0.09% (control; no crystalline Hyp added)	Muscle smad5 mRNA expression (Figure 4)	Not reported numerically	Not reported numerically	3	3	Not estimable	Not estimable	Not estimable	The outcome was presented graphically with significance annotations; an exact pairwise effect size was not calculated	Exact group means and SEs were presented only in Figures 3-5 and were not reliably extractable
Cao et al. (2022)	Improved triploid crucian carp (Carassius auratus triploid)	Hydroxyproline supplementation in a plant-protein-based diet	Hyp 1.53% (1.60% crystalline Hyp added)	Hyp 0.09% (control; no crystalline Hyp added)	Muscle smad9 mRNA expression (Figure 4)	Not reported numerically	Not reported numerically	3	3	Not estimable	Not estimable	Not estimable	The outcome was presented graphically with significance annotations; an exact pairwise effect size was not calculated	Exact group means and SEs were presented only in Figures 3-5 and were not reliably extractable
Cao et al. (2022)	Improved triploid crucian carp (Carassius auratus triploid)	Hydroxyproline supplementation in a plant-protein-based diet	Hyp 1.53% (1.60% crystalline Hyp added)	Hyp 0.09% (control; no crystalline Hyp added)	Muscle tgf-β mRNA expression (Figure 4)	Not reported numerically	Not reported numerically	3	3	Not estimable	Not estimable	Not estimable	The outcome was presented graphically with significance annotations; an exact pairwise effect size was not calculated	Exact group means and SEs were presented only in Figures 3-5 and were not reliably extractable
Cao et al. (2022)	Improved triploid crucian carp (Carassius auratus triploid)	Hydroxyproline supplementation in a plant-protein-based diet	Hyp 1.53% (1.60% crystalline Hyp added)	Hyp 0.09% (control; no crystalline Hyp added)	Muscle igf-1 mRNA expression (Figure 5)	Not reported numerically	Not reported numerically	3	3	Not estimable	Not estimable	Not estimable	The outcome was presented graphically with significance annotations; an exact pairwise effect size was not calculated	Exact group means and SEs were presented only in Figures 3-5 and were not reliably extractable
Cao et al. (2022)	Improved triploid crucian carp (Carassius auratus triploid)	Hydroxyproline supplementation in a plant-protein-based diet	Hyp 1.53% (1.60% crystalline Hyp added)	Hyp 0.09% (control; no crystalline Hyp added)	Muscle tor mRNA expression (Figure 5)	Not reported numerically	Not reported numerically	3	3	Not estimable	Not estimable	Not estimable	The outcome was presented graphically with significance annotations; an exact pairwise effect size was not calculated	Exact group means and SEs were presented only in Figures 3-5 and were not reliably extractable
Cao et al. (2022)	Improved triploid crucian carp (Carassius auratus triploid)	Hydroxyproline supplementation in a plant-protein-based diet	Hyp 1.53% (1.60% crystalline Hyp added)	Hyp 0.09% (control; no crystalline Hyp added)	Muscle myod mRNA expression (Figure 5)	Not reported numerically	Not reported numerically	3	3	Not estimable	Not estimable	Not estimable	The outcome was presented graphically with significance annotations; an exact pairwise effect size was not calculated	Exact group means and SEs were presented only in Figures 3-5 and were not reliably extractable
Cao et al. (2022)	Improved triploid crucian carp (Carassius auratus triploid)	Hydroxyproline supplementation in a plant-protein-based diet	Hyp 1.53% (1.60% crystalline Hyp added)	Hyp 0.09% (control; no crystalline Hyp added)	Muscle myf5 mRNA expression (Figure 5)	Not reported numerically	Not reported numerically	3	3	Not estimable	Not estimable	Not estimable	The outcome was presented graphically with significance annotations; an exact pairwise effect size was not calculated	Exact group means and SEs were presented only in Figures 3-5 and were not reliably extractable
Cao et al. (2022)	Improved triploid crucian carp (Carassius auratus triploid)	Hydroxyproline supplementation in a plant-protein-based diet	Hyp 1.53% (1.60% crystalline Hyp added)	Hyp 0.09% (control; no crystalline Hyp added)	Muscle myhc mRNA expression (Figure 5)	Not reported numerically	Not reported numerically	3	3	Not estimable	Not estimable	Not estimable	The outcome was presented graphically with significance annotations; an exact pairwise effect size was not calculated	Exact group means and SEs were presented only in Figures 3-5 and were not reliably extractable
Cao et al. (2022)	Improved triploid crucian carp (Carassius auratus triploid)	Hydroxyproline supplementation in a plant-protein-based diet	Hyp 1.90% (2.00% crystalline Hyp added)	Hyp 0.09% (control; no crystalline Hyp added)	Muscle col1a1 mRNA expression (Figure 3)	Not reported numerically	Not reported numerically	3	3	Not estimable	Not estimable	Not estimable	The outcome was presented graphically with significance annotations; an exact pairwise effect size was not calculated	Exact group means and SEs were presented only in Figures 3-5 and were not reliably extractable
Cao et al. (2022)	Improved triploid crucian carp (Carassius auratus triploid)	Hydroxyproline supplementation in a plant-protein-based diet	Hyp 1.90% (2.00% crystalline Hyp added)	Hyp 0.09% (control; no crystalline Hyp added)	Muscle col1a2 mRNA expression (Figure 3)	Not reported numerically	Not reported numerically	3	3	Not estimable	Not estimable	Not estimable	The outcome was presented graphically with significance annotations; an exact pairwise effect size was not calculated	Exact group means and SEs were presented only in Figures 3-5 and were not reliably extractable
Cao et al. (2022)	Improved triploid crucian carp (Carassius auratus triploid)	Hydroxyproline supplementation in a plant-protein-based diet	Hyp 1.90% (2.00% crystalline Hyp added)	Hyp 0.09% (control; no crystalline Hyp added)	Muscle p4hα1 mRNA expression (Figure 3)	Not reported numerically	Not reported numerically	3	3	Not estimable	Not estimable	Not estimable	The outcome was presented graphically with significance annotations; an exact pairwise effect size was not calculated	Exact group means and SEs were presented only in Figures 3-5 and were not reliably extractable
Cao et al. (2022)	Improved triploid crucian carp (Carassius auratus triploid)	Hydroxyproline supplementation in a plant-protein-based diet	Hyp 1.90% (2.00% crystalline Hyp added)	Hyp 0.09% (control; no crystalline Hyp added)	Muscle p4hβ mRNA expression (Figure 3)	Not reported numerically	Not reported numerically	3	3	Not estimable	Not estimable	Not estimable	The outcome was presented graphically with significance annotations; an exact pairwise effect size was not calculated	Exact group means and SEs were presented only in Figures 3-5 and were not reliably extractable
Cao et al. (2022)	Improved triploid crucian carp (Carassius auratus triploid)	Hydroxyproline supplementation in a plant-protein-based diet	Hyp 1.90% (2.00% crystalline Hyp added)	Hyp 0.09% (control; no crystalline Hyp added)	Muscle smad4 mRNA expression (Figure 4)	Not reported numerically	Not reported numerically	3	3	Not estimable	Not estimable	Not estimable	The outcome was presented graphically with significance annotations; an exact pairwise effect size was not calculated	Exact group means and SEs were presented only in Figures 3-5 and were not reliably extractable
Cao et al. (2022)	Improved triploid crucian carp (Carassius auratus triploid)	Hydroxyproline supplementation in a plant-protein-based diet	Hyp 1.90% (2.00% crystalline Hyp added)	Hyp 0.09% (control; no crystalline Hyp added)	Muscle smad5 mRNA expression (Figure 4)	Not reported numerically	Not reported numerically	3	3	Not estimable	Not estimable	Not estimable	The outcome was presented graphically with significance annotations; an exact pairwise effect size was not calculated	Exact group means and SEs were presented only in Figures 3-5 and were not reliably extractable
Cao et al. (2022)	Improved triploid crucian carp (Carassius auratus triploid)	Hydroxyproline supplementation in a plant-protein-based diet	Hyp 1.90% (2.00% crystalline Hyp added)	Hyp 0.09% (control; no crystalline Hyp added)	Muscle smad9 mRNA expression (Figure 4)	Not reported numerically	Not reported numerically	3	3	Not estimable	Not estimable	Not estimable	The outcome was presented graphically with significance annotations; an exact pairwise effect size was not calculated	Exact group means and SEs were presented only in Figures 3-5 and were not reliably extractable

Study	Species	Strategy	Intervention	Comparator	Outcome and unit	Intervention mean ± SD	Comparator mean ± SD	n intervention (independent tanks)	n comparator (independent tanks)	Effect-size metric	Effect size	95% CI	Interpretation	Reason not estimated
Cao et al. (2022)	Improved triploid crucian carp (Carassius auratus triploid)	Hydroxyproline supplementation in a plant-protein-based diet	Hyp 1.90% (2.00% crystalline Hyp added)	Hyp 0.09% (control; no crystalline Hyp added)	Muscle tgf-β mRNA expression (Figure 4)	Not reported numerically	Not reported numerically	3	3	Not estimable	Not estimable	Not estimable	The outcome was presented graphically with significance annotations; an exact pairwise effect size was not calculated	Exact group means and SEs were presented only in Figures 3-5 and were not reliably extractable
Cao et al. (2022)	Improved triploid crucian carp (Carassius auratus triploid)	Hydroxyproline supplementation in a plant-protein-based diet	Hyp 1.90% (2.00% crystalline Hyp added)	Hyp 0.09% (control; no crystalline Hyp added)	Muscle igf-1 mRNA expression (Figure 5)	Not reported numerically	Not reported numerically	3	3	Not estimable	Not estimable	Not estimable	The outcome was presented graphically with significance annotations; an exact pairwise effect size was not calculated	Exact group means and SEs were presented only in Figures 3-5 and were not reliably extractable
Cao et al. (2022)	Improved triploid crucian carp (Carassius auratus triploid)	Hydroxyproline supplementation in a plant-protein-based diet	Hyp 1.90% (2.00% crystalline Hyp added)	Hyp 0.09% (control; no crystalline Hyp added)	Muscle tor mRNA expression (Figure 5)	Not reported numerically	Not reported numerically	3	3	Not estimable	Not estimable	Not estimable	The outcome was presented graphically with significance annotations; an exact pairwise effect size was not calculated	Exact group means and SEs were presented only in Figures 3-5 and were not reliably extractable
Cao et al. (2022)	Improved triploid crucian carp (Carassius auratus triploid)	Hydroxyproline supplementation in a plant-protein-based diet	Hyp 1.90% (2.00% crystalline Hyp added)	Hyp 0.09% (control; no crystalline Hyp added)	Muscle myod mRNA expression (Figure 5)	Not reported numerically	Not reported numerically	3	3	Not estimable	Not estimable	Not estimable	The outcome was presented graphically with significance annotations; an exact pairwise effect size was not calculated	Exact group means and SEs were presented only in Figures 3-5 and were not reliably extractable
Cao et al. (2022)	Improved triploid crucian carp (Carassius auratus triploid)	Hydroxyproline supplementation in a plant-protein-based diet	Hyp 1.90% (2.00% crystalline Hyp added)	Hyp 0.09% (control; no crystalline Hyp added)	Muscle myf5 mRNA expression (Figure 5)	Not reported numerically	Not reported numerically	3	3	Not estimable	Not estimable	Not estimable	The outcome was presented graphically with significance annotations; an exact pairwise effect size was not calculated	Exact group means and SEs were presented only in Figures 3-5 and were not reliably extractable
Cao et al. (2022)	Improved triploid crucian carp (Carassius auratus triploid)	Hydroxyproline supplementation in a plant-protein-based diet	Hyp 1.90% (2.00% crystalline Hyp added)	Hyp 0.09% (control; no crystalline Hyp added)	Muscle myhc mRNA expression (Figure 5)	Not reported numerically	Not reported numerically	3	3	Not estimable	Not estimable	Not estimable	The outcome was presented graphically with significance annotations; an exact pairwise effect size was not calculated	Exact group means and SEs were presented only in Figures 3-5 and were not reliably extractable

Note: The replicate tank was treated as the independent unit (n = 3 per diet). Reported SEs were converted to SDs using the reported analytical n: 3 for Tables 4-6, 9 for Table 7, 6 for Tables 8-9, and an inferred n = 6 for Table 10 based on two fish per tank. For subsampled outcomes, Hedges' g point estimates use the reported observation-level SD, whereas CI variance uses three independent tanks. Feeding rate and total nitrogen waste were direction-reversed. Gene-expression outcomes were not estimated because exact means and SEs were available only graphically. All pairwise CIs are unadjusted.

Source: Cao et al. (2022), https://doi.org/10.3389/fphys.2022.913800

Study 11. Yoo and Bai (2014): matched phytase-supplemented versus unsupplemented soybean-meal diets

Study	Species	Strategy	Intervention	Comparator	Outcome and unit	Intervention mean ± pooled SD	Comparator mean ± pooled SD	n intervention (tanks)	n comparator (tanks)	Effect-size metric	Effect size	95% CI	Interpretation	Reason not estimated
Yoo and Bai (2014)	Olive flounder (Paralichthys olivaceus)	Microbial phytase supplementation in soybean-meal-based low-fishmeal diets	S30P1000 (30% replacement; 1000 U phytase/kg; analyzed 987 U/kg)	S30 (30% FM-protein replacement by soybean meal; no phytase)	Apparent digestibility coefficient of phosphorus (%)	91.0 ± 12.57	58.2 ± 12.57	3	3	Hedges' g using study-wide pooled SD	2.47	0.70 to 4.23	Higher, more favorable value with phytase; unadjusted 95% CI excluded 0	Not applicable - effect size estimated from reported pooled SEM
Yoo and Bai (2014)	Olive flounder (Paralichthys olivaceus)	Microbial phytase supplementation in soybean-meal-based low-fishmeal diets	S30P1000 (30% replacement; 1000 U phytase/kg; analyzed 987 U/kg)	S30 (30% FM-protein replacement by soybean meal; no phytase)	Apparent digestibility coefficient of dry matter (%)	66.9 ± 1.85	64.1 ± 1.85	3	3	Hedges' g using study-wide pooled SD	1.43	-0.17 to 3.03	Higher, more favorable value with phytase; unadjusted 95% CI included 0	Not applicable - effect size estimated from reported pooled SEM
Yoo and Bai (2014)	Olive flounder (Paralichthys olivaceus)	Microbial phytase supplementation in soybean-meal-based low-fishmeal diets	S30P1000 (30% replacement; 1000 U phytase/kg; analyzed 987 U/kg)	S30 (30% FM-protein replacement by soybean meal; no phytase)	Apparent digestibility coefficient of crude protein (%)	89.9 ± 1.84	84.9 ± 1.84	3	3	Hedges' g using study-wide pooled SD	2.57	0.79 to 4.36	Higher, more favorable value with phytase; unadjusted 95% CI excluded 0	Not applicable - effect size estimated from reported pooled SEM
Yoo and Bai (2014)	Olive flounder (Paralichthys olivaceus)	Microbial phytase supplementation in soybean-meal-based low-fishmeal diets	S30P1000 (30% replacement; 1000 U phytase/kg; analyzed 987 U/kg)	S30 (30% FM-protein replacement by soybean meal; no phytase)	Weight gain (%)	281.0 ± 5.63	261.0 ± 5.63	3	3	Hedges' g using study-wide pooled SD	3.36	1.40 to 5.32	Higher, more favorable value with phytase; unadjusted 95% CI excluded 0	Not applicable - effect size estimated from reported pooled SEM
Yoo and Bai (2014)	Olive flounder (Paralichthys olivaceus)	Microbial phytase supplementation in soybean-meal-based low-fishmeal diets	S30P1000 (30% replacement; 1000 U phytase/kg; analyzed 987 U/kg)	S30 (30% FM-protein replacement by soybean meal; no phytase)	Specific growth rate (%/day)	2.42 ± 0.03	2.29 ± 0.03	3	3	Hedges' g using study-wide pooled SD	3.55	1.54 to 5.55	Higher, more favorable value with phytase; unadjusted 95% CI excluded 0	Not applicable - effect size estimated from reported pooled SEM
Yoo and Bai (2014)	Olive flounder (Paralichthys olivaceus)	Microbial phytase supplementation in soybean-meal-based low-fishmeal diets	S30P1000 (30% replacement; 1000 U phytase/kg; analyzed 987 U/kg)	S30 (30% FM-protein replacement by soybean meal; no phytase)	Feed efficiency ratio (%)	106.0 ± 1.97	101.0 ± 1.97	3	3	Hedges' g using study-wide pooled SD	2.39	0.64 to 4.15	Higher, more favorable value with phytase; unadjusted 95% CI excluded 0	Not applicable - effect size estimated from reported pooled SEM
Yoo and Bai (2014)	Olive flounder (Paralichthys olivaceus)	Microbial phytase supplementation in soybean-meal-based low-fishmeal diets	S30P1000 (30% replacement; 1000 U phytase/kg; analyzed 987 U/kg)	S30 (30% FM-protein replacement by soybean meal; no phytase)	Protein efficiency ratio	2.10 ± 0.03	2.07 ± 0.03	3	3	Hedges' g using study-wide pooled SD	0.82	-0.72 to 2.36	Higher, more favorable value with phytase; unadjusted 95% CI included 0	Not applicable - effect size estimated from reported pooled SEM
Yoo and Bai (2014)	Olive flounder (Paralichthys olivaceus)	Microbial phytase supplementation in soybean-meal-based low-fishmeal diets	S30P1000 (30% replacement; 1000 U phytase/kg; analyzed 987 U/kg)	S30 (30% FM-protein replacement by soybean meal; no phytase)	Hepatosomatic index (%)	2.11 ± 0.16	2.29 ± 0.16	3	3	Hedges' g using study-wide pooled SD	-1.09	-2.66 to 0.47	Lower measured value with phytase; unadjusted 95% CI included 0	Not applicable - effect size estimated from reported pooled SEM
Yoo and Bai (2014)	Olive flounder (Paralichthys olivaceus)	Microbial phytase supplementation in soybean-meal-based low-fishmeal diets	S30P1000 (30% replacement; 1000 U phytase/kg; analyzed 987 U/kg)	S30 (30% FM-protein replacement by soybean meal; no phytase)	Condition factor	1.77 ± 0.03	1.17 ± 0.03	3	3	Hedges' g using study-wide pooled SD	16.38	10.12 to 22.63	Higher measured value with phytase; unadjusted 95% CI excluded 0	Not applicable - effect size estimated from reported pooled SEM
Yoo and Bai (2014)	Olive flounder (Paralichthys olivaceus)	Microbial phytase supplementation in soybean-meal-based low-fishmeal diets	S30P1000 (30% replacement; 1000 U phytase/kg; analyzed 987 U/kg)	S30 (30% FM-protein replacement by soybean meal; no phytase)	Survival rate (%)	96.7 ± 1.78	96.7 ± 1.78	3	3	Hedges' g using study-wide pooled SD	0.0	-1.51 to 1.51	No observed mean difference; unadjusted 95% CI included 0	Not applicable - effect size estimated from reported pooled SEM
Yoo and Bai (2014)	Olive flounder (Paralichthys olivaceus)	Microbial phytase supplementation in soybean-meal-based low-fishmeal diets	S30P1000 (30% replacement; 1000 U phytase/kg; analyzed 987 U/kg)	S30 (30% FM-protein replacement by soybean meal; no phytase)	Hematocrit (%)	48.2 ± 0.68	46.6 ± 0.68	3	3	Hedges' g using study-wide pooled SD	2.24	0.51 to 3.96	Higher measured value with phytase; unadjusted 95% CI excluded 0	Not applicable - effect size estimated from reported pooled SEM
Yoo and Bai (2014)	Olive flounder (Paralichthys olivaceus)	Microbial phytase supplementation in soybean-meal-based low-fishmeal diets	S30P1000 (30% replacement; 1000 U phytase/kg; analyzed 987 U/kg)	S30 (30% FM-protein replacement by soybean meal; no phytase)	Hemoglobin (g/100 mL)	5.50 ± 0.23	6.32 ± 0.23	3	3	Hedges' g using study-wide pooled SD	-3.44	-5.42 to -1.46	Lower measured value with phytase; unadjusted 95% CI excluded 0	Not applicable - effect size estimated from reported pooled SEM
Yoo and Bai (2014)	Olive flounder (Paralichthys olivaceus)	Microbial phytase supplementation in soybean-meal-based low-fishmeal diets	S30P1000 (30% replacement; 1000 U phytase/kg; analyzed 987 U/kg)	S30 (30% FM-protein replacement by soybean meal; no phytase)	Moisture (% as-is)	74.5 ± 0.33	73.7 ± 0.33	3	3	Hedges' g using study-wide pooled SD	2.3	0.56 to 4.03	Higher measured value with phytase; unadjusted 95% CI excluded 0	Not applicable - effect size estimated from reported pooled SEM
Yoo and Bai (2014)	Olive flounder (Paralichthys olivaceus)	Microbial phytase supplementation in soybean-meal-based low-fishmeal diets	S30P1000 (30% replacement; 1000 U phytase/kg; analyzed 987 U/kg)	S30 (30% FM-protein replacement by soybean meal; no phytase)	Crude protein (% as-is)	17.5 ± 0.24	17.7 ± 0.24	3	3	Hedges' g using study-wide pooled SD	-0.78	-2.32 to 0.76	Lower measured value with phytase; unadjusted 95% CI included 0	Not applicable - effect size estimated from reported pooled SEM
Yoo and Bai (2014)	Olive flounder (Paralichthys olivaceus)	Microbial phytase supplementation in soybean-meal-based low-fishmeal diets	S30P1000 (30% replacement; 1000 U phytase/kg; analyzed 987 U/kg)	S30 (30% FM-protein replacement by soybean meal; no phytase)	Crude lipid (% as-is)	4.56 ± 0.23	4.64 ± 0.23	3	3	Hedges' g using study-wide pooled SD	-0.34	-1.85 to 1.18	Lower measured value with phytase; unadjusted 95% CI included 0	Not applicable - effect size estimated from reported pooled SEM
Yoo and Bai (2014)	Olive flounder (Paralichthys olivaceus)	Microbial phytase supplementation in soybean-meal-based low-fishmeal diets	S30P1000 (30% replacement; 1000 U phytase/kg; analyzed 987 U/kg)	S30 (30% FM-protein replacement by soybean meal; no phytase)	Ash (% as-is)	5.77 ± 0.28	6.43 ± 0.28	3	3	Hedges' g using study-wide pooled SD	-2.25	-3.98 to -0.52	Lower measured value with phytase; unadjusted 95% CI excluded 0	Not applicable - effect size estimated from reported pooled SEM
Yoo and Bai (2014)	Olive flounder (Paralichthys olivaceus)	Microbial phytase supplementation in soybean-meal-based low-fishmeal diets	S30P2000 (30% replacement; 2000 U phytase/kg; analyzed 2013 U/kg)	S30 (30% FM-protein replacement by soybean meal; no phytase)	Apparent digestibility coefficient of phosphorus (%)	90.7 ± 12.57	58.2 ± 12.57	3	3	Hedges' g using study-wide pooled SD	2.44	0.68 to 4.21	Higher, more favorable value with phytase; unadjusted 95% CI excluded 0	Not applicable - effect size estimated from reported pooled SEM
Yoo and Bai (2014)	Olive flounder (Paralichthys olivaceus)	Microbial phytase supplementation in soybean-meal-based low-fishmeal diets	S30P2000 (30% replacement; 2000 U phytase/kg; analyzed 2013 U/kg)	S30 (30% FM-protein replacement by soybean meal; no phytase)	Apparent digestibility coefficient of dry matter (%)	66.5 ± 1.85	64.1 ± 1.85	3	3	Hedges' g using study-wide pooled SD	1.22	-0.36 to 2.80	Higher, more favorable value with phytase; unadjusted 95% CI included 0	Not applicable - effect size estimated from reported pooled SEM
Yoo and Bai (2014)	Olive flounder (Paralichthys olivaceus)	Microbial phytase supplementation in soybean-meal-based low-fishmeal diets	S30P2000 (30% replacement; 2000 U phytase/kg; analyzed 2013 U/kg)	S30 (30% FM-protein replacement by soybean meal; no phytase)	Apparent digestibility coefficient of crude protein (%)	89.2 ± 1.84	84.9 ± 1.84	3	3	Hedges' g using study-wide pooled SD	2.21	0.49 to 3.94	Higher, more favorable value with phytase; unadjusted 95% CI excluded 0	Not applicable - effect size estimated from reported pooled SEM
Yoo and Bai (2014)	Olive flounder (Paralichthys olivaceus)	Microbial phytase supplementation in soybean-meal-based low-fishmeal diets	S30P2000 (30% replacement; 2000 U phytase/kg; analyzed 2013 U/kg)	S30 (30% FM-protein replacement by soybean meal; no phytase)	Weight gain (%)	275.0 ± 5.63	261.0 ± 5.63	3	3	Hedges' g using study-wide pooled SD	2.35	0.61 to 4.10	Higher, more favorable value with phytase; unadjusted 95% CI excluded 0	Not applicable - effect size estimated from reported pooled SEM
Yoo and Bai (2014)	Olive flounder (Paralichthys olivaceus)	Microbial phytase supplementation in soybean-meal-based low-fishmeal diets	S30P2000 (30% replacement; 2000 U phytase/kg; analyzed 2013 U/kg)	S30 (30% FM-protein replacement by soybean meal; no phytase)	Specific growth rate (%/day)	2.40 ± 0.03	2.29 ± 0.03	3	3	Hedges' g using study-wide pooled SD	3.0	1.12 to 4.88	Higher, more favorable value with phytase; unadjusted 95% CI excluded 0	Not applicable - effect size estimated from reported pooled SEM
Yoo and Bai (2014)	Olive flounder (Paralichthys olivaceus)	Microbial phytase supplementation in soybean-meal-based low-fishmeal diets	S30P2000 (30% replacement; 2000 U phytase/kg; analyzed 2013 U/kg)	S30 (30% FM-protein replacement by soybean meal; no phytase)	Feed efficiency ratio (%)	102.0 ± 1.97	101.0 ± 1.97	3	3	Hedges' g using study-wide pooled SD	0.48	-1.04 to 2.00	Higher, more favorable value with phytase; unadjusted 95% CI included 0	Not applicable - effect size estimated from reported pooled SEM
Yoo and Bai (2014)	Olive flounder (Paralichthys olivaceus)	Microbial phytase supplementation in soybean-meal-based low-fishmeal diets	S30P2000 (30% replacement; 2000 U phytase/kg; analyzed 2013 U/kg)	S30 (30% FM-protein replacement by soybean meal; no phytase)	Protein efficiency ratio	2.03 ± 0.03	2.07 ± 0.03	3	3	Hedges' g using study-wide pooled SD	-1.09	-2.66 to 0.47	Lower, less favorable value with phytase; unadjusted 95% CI included 0	Not applicable - effect size estimated from reported pooled SEM
Yoo and Bai (2014)	Olive flounder (Paralichthys olivaceus)	Microbial phytase supplementation in soybean-meal-based low-fishmeal diets	S30P2000 (30% replacement; 2000 U phytase/kg; analyzed 2013 U/kg)	S30 (30% FM-protein replacement by soybean meal; no phytase)	Hepatosomatic index (%)	2.19 ± 0.16	2.29 ± 0.16	3	3	Hedges' g using study-wide pooled SD	-0.61	-2.14 to 0.92	Lower measured value with phytase; unadjusted 95% CI included 0	Not applicable - effect size estimated from reported pooled SEM
Yoo and Bai (2014)	Olive flounder (Paralichthys olivaceus)	Microbial phytase supplementation in soybean-meal-based low-fishmeal diets	S30P2000 (30% replacement; 2000 U phytase/kg; analyzed 2013 U/kg)	S30 (30% FM-protein replacement by soybean meal; no phytase)	Condition factor	1.09 ± 0.03	1.17 ± 0.03	3	3	Hedges' g using study-wide pooled SD	-2.18	-3.90 to -0.47	Lower measured value with phytase; unadjusted 95% CI excluded 0	Not applicable - effect size estimated from reported pooled SEM
Yoo and Bai (2014)	Olive flounder (Paralichthys olivaceus)	Microbial phytase supplementation in soybean-meal-based low-fishmeal diets	S30P2000 (30% replacement; 2000 U phytase/kg; analyzed 2013 U/kg)	S30 (30% FM-protein replacement by soybean meal; no phytase)	Survival rate (%)	95.0 ± 1.78	96.7 ± 1.78	3	3	Hedges' g using study-wide pooled SD	-0.9	-2.45 to 0.65	Lower, less favorable value with phytase; unadjusted 95% CI included 0	Not applicable - effect size estimated from reported pooled SEM
Yoo and Bai (2014)	Olive flounder (Paralichthys olivaceus)	Microbial phytase supplementation in soybean-meal-based low-fishmeal diets	S30P2000 (30% replacement; 2000 U phytase/kg; analyzed 2013 U/kg)	S30 (30% FM-protein replacement by soybean meal; no phytase)	Hematocrit (%)	48.7 ± 0.68	46.6 ± 0.68	3	3	Hedges' g using study-wide pooled SD	2.94	1.08 to 4.80	Higher measured value with phytase; unadjusted 95% CI excluded 0	Not applicable - effect size estimated from reported pooled SEM
Yoo and Bai (2014)	Olive flounder (Paralichthys olivaceus)	Microbial phytase supplementation in soybean-meal-based low-fishmeal diets	S30P2000 (30% replacement; 2000 U phytase/kg; analyzed 2013 U/kg)	S30 (30% FM-protein replacement by soybean meal; no phytase)	Hemoglobin (g/100 mL)	6.54 ± 0.23	6.32 ± 0.23	3	3	Hedges' g using study-wide pooled SD	0.92	-0.63 to 2.48	Higher measured value with phytase; unadjusted 95% CI included 0	Not applicable - effect size estimated from reported pooled SEM
Yoo and Bai (2014)	Olive flounder (Paralichthys olivaceus)	Microbial phytase supplementation in soybean-meal-based low-fishmeal diets	S30P2000 (30% replacement; 2000 U phytase/kg; analyzed 2013 U/kg)	S30 (30% FM-protein replacement by soybean meal; no phytase)	Moisture (% as-is)	74.0 ± 0.33	73.7 ± 0.33	3	3	Hedges' g using study-wide pooled SD	0.86	-0.68 to 2.41	Higher measured value with phytase; unadjusted 95% CI included 0	Not applicable - effect size estimated from reported pooled SEM
Yoo and Bai (2014)	Olive flounder (Paralichthys olivaceus)	Microbial phytase supplementation in soybean-meal-based low-fishmeal diets	S30P2000 (30% replacement; 2000 U phytase/kg; analyzed 2013 U/kg)	S30 (30% FM-protein replacement by soybean meal; no phytase)	Crude protein (% as-is)	17.2 ± 0.24	17.7 ± 0.24	3	3	Hedges' g using study-wide pooled SD	-1.95	-3.63 to -0.27	Lower measured value with phytase; unadjusted 95% CI excluded 0	Not applicable - effect size estimated from reported pooled SEM

Study	Species	Strategy	Intervention	Comparator	Outcome and unit	Intervention mean ± pooled SD	Comparator mean ± pooled SD	n intervention (tanks)	n comparator (tanks)	Effect-size metric	Effect size	95% CI	Interpretation	Reason not estimated
Yoo and Bai (2014)	Olive flounder (Paralichthys olivaceus)	Microbial phytase supplementation in soybean-meal-based low-fishmeal diets	S30P2000 (30% replacement; 2000 U phytase/kg; analyzed 2013 U/kg)	S30 (30% FM-protein replacement by soybean meal; no phytase)	Crude lipid (% as-is)	4.43 ± 0.23	4.64 ± 0.23	3	3	Hedges' g using study-wide pooled SD	-0.88	-2.43 to 0.67	Lower measured value with phytase; unadjusted 95% CI included 0	Not applicable - effect size estimated from reported pooled SEM
Yoo and Bai (2014)	Olive flounder (Paralichthys olivaceus)	Microbial phytase supplementation in soybean-meal-based low-fishmeal diets	S30P2000 (30% replacement; 2000 U phytase/kg; analyzed 2013 U/kg)	S30 (30% FM-protein replacement by soybean meal; no phytase)	Ash (% as-is)	6.02 ± 0.28	6.43 ± 0.28	3	3	Hedges' g using study-wide pooled SD	-1.4	-3.00 to 0.20	Lower measured value with phytase; unadjusted 95% CI included 0	Not applicable - effect size estimated from reported pooled SEM
Yoo and Bai (2014)	Olive flounder (Paralichthys olivaceus)	Microbial phytase supplementation in soybean-meal-based low-fishmeal diets	S40P1000 (40% replacement; 1000 U phytase/kg; analyzed 1036 U/kg)	S40 (40% FM-protein replacement by soybean meal; no phytase)	Apparent digestibility coefficient of phosphorus (%)	86.7 ± 12.57	53.6 ± 12.57	3	3	Hedges' g using study-wide pooled SD	2.49	0.72 to 4.26	Higher, more favorable value with phytase; unadjusted 95% CI excluded 0	Not applicable - effect size estimated from reported pooled SEM
Yoo and Bai (2014)	Olive flounder (Paralichthys olivaceus)	Microbial phytase supplementation in soybean-meal-based low-fishmeal diets	S40P1000 (40% replacement; 1000 U phytase/kg; analyzed 1036 U/kg)	S40 (40% FM-protein replacement by soybean meal; no phytase)	Apparent digestibility coefficient of dry matter (%)	58.8 ± 1.85	56.8 ± 1.85	3	3	Hedges' g using study-wide pooled SD	1.02	-0.54 to 2.58	Higher, more favorable value with phytase; unadjusted 95% CI included 0	Not applicable - effect size estimated from reported pooled SEM
Yoo and Bai (2014)	Olive flounder (Paralichthys olivaceus)	Microbial phytase supplementation in soybean-meal-based low-fishmeal diets	S40P1000 (40% replacement; 1000 U phytase/kg; analyzed 1036 U/kg)	S40 (40% FM-protein replacement by soybean meal; no phytase)	Apparent digestibility coefficient of crude protein (%)	85.4 ± 1.84	81.4 ± 1.84	3	3	Hedges' g using study-wide pooled SD	2.06	0.37 to 3.75	Higher, more favorable value with phytase; unadjusted 95% CI excluded 0	Not applicable - effect size estimated from reported pooled SEM
Yoo and Bai (2014)	Olive flounder (Paralichthys olivaceus)	Microbial phytase supplementation in soybean-meal-based low-fishmeal diets	S40P1000 (40% replacement; 1000 U phytase/kg; analyzed 1036 U/kg)	S40 (40% FM-protein replacement by soybean meal; no phytase)	Weight gain (%)	236.0 ± 5.63	235.0 ± 5.63	3	3	Hedges' g using study-wide pooled SD	0.17	-1.35 to 1.68	Higher, more favorable value with phytase; unadjusted 95% CI included 0	Not applicable - effect size estimated from reported pooled SEM
Yoo and Bai (2014)	Olive flounder (Paralichthys olivaceus)	Microbial phytase supplementation in soybean-meal-based low-fishmeal diets	S40P1000 (40% replacement; 1000 U phytase/kg; analyzed 1036 U/kg)	S40 (40% FM-protein replacement by soybean meal; no phytase)	Specific growth rate (%/day)	2.16 ± 0.03	2.16 ± 0.03	3	3	Hedges' g using study-wide pooled SD	0.0	-1.51 to 1.51	No observed mean difference; unadjusted 95% CI included 0	Not applicable - effect size estimated from reported pooled SEM
Yoo and Bai (2014)	Olive flounder (Paralichthys olivaceus)	Microbial phytase supplementation in soybean-meal-based low-fishmeal diets	S40P1000 (40% replacement; 1000 U phytase/kg; analyzed 1036 U/kg)	S40 (40% FM-protein replacement by soybean meal; no phytase)	Feed efficiency ratio (%)	93.9 ± 1.97	91.6 ± 1.97	3	3	Hedges' g using study-wide pooled SD	1.1	-0.47 to 2.67	Higher, more favorable value with phytase; unadjusted 95% CI included 0	Not applicable - effect size estimated from reported pooled SEM
Yoo and Bai (2014)	Olive flounder (Paralichthys olivaceus)	Microbial phytase supplementation in soybean-meal-based low-fishmeal diets	S40P1000 (40% replacement; 1000 U phytase/kg; analyzed 1036 U/kg)	S40 (40% FM-protein replacement by soybean meal; no phytase)	Protein efficiency ratio	1.88 ± 0.03	1.85 ± 0.03	3	3	Hedges' g using study-wide pooled SD	0.82	-0.72 to 2.36	Higher, more favorable value with phytase; unadjusted 95% CI included 0	Not applicable - effect size estimated from reported pooled SEM
Yoo and Bai (2014)	Olive flounder (Paralichthys olivaceus)	Microbial phytase supplementation in soybean-meal-based low-fishmeal diets	S40P1000 (40% replacement; 1000 U phytase/kg; analyzed 1036 U/kg)	S40 (40% FM-protein replacement by soybean meal; no phytase)	Hepatosomatic index (%)	2.00 ± 0.16	2.28 ± 0.16	3	3	Hedges' g using study-wide pooled SD	-1.7	-3.34 to -0.06	Lower measured value with phytase; unadjusted 95% CI excluded 0	Not applicable - effect size estimated from reported pooled SEM
Yoo and Bai (2014)	Olive flounder (Paralichthys olivaceus)	Microbial phytase supplementation in soybean-meal-based low-fishmeal diets	S40P1000 (40% replacement; 1000 U phytase/kg; analyzed 1036 U/kg)	S40 (40% FM-protein replacement by soybean meal; no phytase)	Condition factor	1.02 ± 0.03	1.09 ± 0.03	3	3	Hedges' g using study-wide pooled SD	-1.91	-3.58 to -0.24	Lower measured value with phytase; unadjusted 95% CI excluded 0	Not applicable - effect size estimated from reported pooled SEM
Yoo and Bai (2014)	Olive flounder (Paralichthys olivaceus)	Microbial phytase supplementation in soybean-meal-based low-fishmeal diets	S40P1000 (40% replacement; 1000 U phytase/kg; analyzed 1036 U/kg)	S40 (40% FM-protein replacement by soybean meal; no phytase)	Survival rate (%)	96.7 ± 1.78	100.0 ± 1.78	3	3	Hedges' g using study-wide pooled SD	-1.75	-3.39 to -0.10	Lower, less favorable value with phytase; unadjusted 95% CI excluded 0	Not applicable - effect size estimated from reported pooled SEM
Yoo and Bai (2014)	Olive flounder (Paralichthys olivaceus)	Microbial phytase supplementation in soybean-meal-based low-fishmeal diets	S40P1000 (40% replacement; 1000 U phytase/kg; analyzed 1036 U/kg)	S40 (40% FM-protein replacement by soybean meal; no phytase)	Hematocrit (%)	48.6 ± 0.68	47.4 ± 0.68	3	3	Hedges' g using study-wide pooled SD	1.68	0.04 to 3.32	Higher measured value with phytase; unadjusted 95% CI excluded 0	Not applicable - effect size estimated from reported pooled SEM
Yoo and Bai (2014)	Olive flounder (Paralichthys olivaceus)	Microbial phytase supplementation in soybean-meal-based low-fishmeal diets	S40P1000 (40% replacement; 1000 U phytase/kg; analyzed 1036 U/kg)	S40 (40% FM-protein replacement by soybean meal; no phytase)	Hemoglobin (g/100 mL)	6.33 ± 0.23	6.62 ± 0.23	3	3	Hedges' g using study-wide pooled SD	-1.22	-2.80 to 0.36	Lower measured value with phytase; unadjusted 95% CI included 0	Not applicable - effect size estimated from reported pooled SEM
Yoo and Bai (2014)	Olive flounder (Paralichthys olivaceus)	Microbial phytase supplementation in soybean-meal-based low-fishmeal diets	S40P1000 (40% replacement; 1000 U phytase/kg; analyzed 1036 U/kg)	S40 (40% FM-protein replacement by soybean meal; no phytase)	Moisture (% as-is)	74.6 ± 0.33	74.6 ± 0.33	3	3	Hedges' g using study-wide pooled SD	0.0	-1.51 to 1.51	No observed mean difference; unadjusted 95% CI included 0	Not applicable - effect size estimated from reported pooled SEM
Yoo and Bai (2014)	Olive flounder (Paralichthys olivaceus)	Microbial phytase supplementation in soybean-meal-based low-fishmeal diets	S40P1000 (40% replacement; 1000 U phytase/kg; analyzed 1036 U/kg)	S40 (40% FM-protein replacement by soybean meal; no phytase)	Crude protein (% as-is)	17.9 ± 0.24	18.1 ± 0.24	3	3	Hedges' g using study-wide pooled SD	-0.78	-2.32 to 0.76	Lower measured value with phytase; unadjusted 95% CI included 0	Not applicable - effect size estimated from reported pooled SEM
Yoo and Bai (2014)	Olive flounder (Paralichthys olivaceus)	Microbial phytase supplementation in soybean-meal-based low-fishmeal diets	S40P1000 (40% replacement; 1000 U phytase/kg; analyzed 1036 U/kg)	S40 (40% FM-protein replacement by soybean meal; no phytase)	Crude lipid (% as-is)	3.88 ± 0.23	4.05 ± 0.23	3	3	Hedges' g using study-wide pooled SD	-0.71	-2.25 to 0.82	Lower measured value with phytase; unadjusted 95% CI included 0	Not applicable - effect size estimated from reported pooled SEM
Yoo and Bai (2014)	Olive flounder (Paralichthys olivaceus)	Microbial phytase supplementation in soybean-meal-based low-fishmeal diets	S40P1000 (40% replacement; 1000 U phytase/kg; analyzed 1036 U/kg)	S40 (40% FM-protein replacement by soybean meal; no phytase)	Ash (% as-is)	5.56 ± 0.28	6.28 ± 0.28	3	3	Hedges' g using study-wide pooled SD	-2.46	-4.22 to -0.69	Lower measured value with phytase; unadjusted 95% CI excluded 0	Not applicable - effect size estimated from reported pooled SEM
Yoo and Bai (2014)	Olive flounder (Paralichthys olivaceus)	Microbial phytase supplementation in soybean-meal-based low-fishmeal diets	S40P2000 (40% replacement; 2000 U phytase/kg; analyzed 2018 U/kg)	S40 (40% FM-protein replacement by soybean meal; no phytase)	Apparent digestibility coefficient of phosphorus (%)	85.3 ± 12.57	53.6 ± 12.57	3	3	Hedges' g using study-wide pooled SD	2.38	0.63 to 4.14	Higher, more favorable value with phytase; unadjusted 95% CI excluded 0	Not applicable - effect size estimated from reported pooled SEM
Yoo and Bai (2014)	Olive flounder (Paralichthys olivaceus)	Microbial phytase supplementation in soybean-meal-based low-fishmeal diets	S40P2000 (40% replacement; 2000 U phytase/kg; analyzed 2018 U/kg)	S40 (40% FM-protein replacement by soybean meal; no phytase)	Apparent digestibility coefficient of dry matter (%)	58.0 ± 1.85	56.8 ± 1.85	3	3	Hedges' g using study-wide pooled SD	0.61	-0.92 to 2.14	Higher, more favorable value with phytase; unadjusted 95% CI included 0	Not applicable - effect size estimated from reported pooled SEM
Yoo and Bai (2014)	Olive flounder (Paralichthys olivaceus)	Microbial phytase supplementation in soybean-meal-based low-fishmeal diets	S40P2000 (40% replacement; 2000 U phytase/kg; analyzed 2018 U/kg)	S40 (40% FM-protein replacement by soybean meal; no phytase)	Apparent digestibility coefficient of crude protein (%)	84.2 ± 1.84	81.4 ± 1.84	3	3	Hedges' g using study-wide pooled SD	1.44	-0.16 to 3.05	Higher, more favorable value with phytase; unadjusted 95% CI included 0	Not applicable - effect size estimated from reported pooled SEM
Yoo and Bai (2014)	Olive flounder (Paralichthys olivaceus)	Microbial phytase supplementation in soybean-meal-based low-fishmeal diets	S40P2000 (40% replacement; 2000 U phytase/kg; analyzed 2018 U/kg)	S40 (40% FM-protein replacement by soybean meal; no phytase)	Weight gain (%)	235.0 ± 5.63	235.0 ± 5.63	3	3	Hedges' g using study-wide pooled SD	0.0	-1.51 to 1.51	No observed mean difference; unadjusted 95% CI included 0	Not applicable - effect size estimated from reported pooled SEM
Yoo and Bai (2014)	Olive flounder (Paralichthys olivaceus)	Microbial phytase supplementation in soybean-meal-based low-fishmeal diets	S40P2000 (40% replacement; 2000 U phytase/kg; analyzed 2018 U/kg)	S40 (40% FM-protein replacement by soybean meal; no phytase)	Specific growth rate (%/day)	2.16 ± 0.03	2.16 ± 0.03	3	3	Hedges' g using study-wide pooled SD	0.0	-1.51 to 1.51	No observed mean difference; unadjusted 95% CI included 0	Not applicable - effect size estimated from reported pooled SEM
Yoo and Bai (2014)	Olive flounder (Paralichthys olivaceus)	Microbial phytase supplementation in soybean-meal-based low-fishmeal diets	S40P2000 (40% replacement; 2000 U phytase/kg; analyzed 2018 U/kg)	S40 (40% FM-protein replacement by soybean meal; no phytase)	Feed efficiency ratio (%)	93.9 ± 1.97	91.6 ± 1.97	3	3	Hedges' g using study-wide pooled SD	1.1	-0.47 to 2.67	Higher, more favorable value with phytase; unadjusted 95% CI included 0	Not applicable - effect size estimated from reported pooled SEM
Yoo and Bai (2014)	Olive flounder (Paralichthys olivaceus)	Microbial phytase supplementation in soybean-meal-based low-fishmeal diets	S40P2000 (40% replacement; 2000 U phytase/kg; analyzed 2018 U/kg)	S40 (40% FM-protein replacement by soybean meal; no phytase)	Protein efficiency ratio	1.88 ± 0.03	1.85 ± 0.03	3	3	Hedges' g using study-wide pooled SD	0.82	-0.72 to 2.36	Higher, more favorable value with phytase; unadjusted 95% CI included 0	Not applicable - effect size estimated from reported pooled SEM
Yoo and Bai (2014)	Olive flounder (Paralichthys olivaceus)	Microbial phytase supplementation in soybean-meal-based low-fishmeal diets	S40P2000 (40% replacement; 2000 U phytase/kg; analyzed 2018 U/kg)	S40 (40% FM-protein replacement by soybean meal; no phytase)	Hepatosomatic index (%)	2.37 ± 0.16	2.28 ± 0.16	3	3	Hedges' g using study-wide pooled SD	0.55	-0.98 to 2.07	Higher measured value with phytase; unadjusted 95% CI included 0	Not applicable - effect size estimated from reported pooled SEM
Yoo and Bai (2014)	Olive flounder (Paralichthys olivaceus)	Microbial phytase supplementation in soybean-meal-based low-fishmeal diets	S40P2000 (40% replacement; 2000 U phytase/kg; analyzed 2018 U/kg)	S40 (40% FM-protein replacement by soybean meal; no phytase)	Condition factor	1.04 ± 0.03	1.09 ± 0.03	3	3	Hedges' g using study-wide pooled SD	-1.36	-2.96 to 0.23	Lower measured value with phytase; unadjusted 95% CI included 0	Not applicable - effect size estimated from reported pooled SEM
Yoo and Bai (2014)	Olive flounder (Paralichthys olivaceus)	Microbial phytase supplementation in soybean-meal-based low-fishmeal diets	S40P2000 (40% replacement; 2000 U phytase/kg; analyzed 2018 U/kg)	S40 (40% FM-protein replacement by soybean meal; no phytase)	Survival rate (%)	86.7 ± 1.78	100.0 ± 1.78	3	3	Hedges' g using study-wide pooled SD	-7.05	-10.07 to -4.03	Lower, less favorable value with phytase; unadjusted 95% CI excluded 0	Not applicable - effect size estimated from reported pooled SEM
Yoo and Bai (2014)	Olive flounder (Paralichthys olivaceus)	Microbial phytase supplementation in soybean-meal-based low-fishmeal diets	S40P2000 (40% replacement; 2000 U phytase/kg; analyzed 2018 U/kg)	S40 (40% FM-protein replacement by soybean meal; no phytase)	Hematocrit (%)	46.5 ± 0.68	47.4 ± 0.68	3	3	Hedges' g using study-wide pooled SD	-1.26	-2.84 to 0.32	Lower measured value with phytase; unadjusted 95% CI included 0	Not applicable - effect size estimated from reported pooled SEM
Yoo and Bai (2014)	Olive flounder (Paralichthys olivaceus)	Microbial phytase supplementation in soybean-meal-based low-fishmeal diets	S40P2000 (40% replacement; 2000 U phytase/kg; analyzed 2018 U/kg)	S40 (40% FM-protein replacement by soybean meal; no phytase)	Hemoglobin (g/100 mL)	6.43 ± 0.23	6.62 ± 0.23	3	3	Hedges' g using study-wide pooled SD	-0.8	-2.34 to 0.74	Lower measured value with phytase; unadjusted 95% CI included 0	Not applicable - effect size estimated from reported pooled SEM
Yoo and Bai (2014)	Olive flounder (Paralichthys olivaceus)	Microbial phytase supplementation in soybean-meal-based low-fishmeal diets	S40P2000 (40% replacement; 2000 U phytase/kg; analyzed 2018 U/kg)	S40 (40% FM-protein replacement by soybean meal; no phytase)	Moisture (% as-is)	75.2 ± 0.33	74.6 ± 0.33	3	3	Hedges' g using study-wide pooled SD	1.72	0.08 to 3.37	Higher measured value with phytase; unadjusted 95% CI excluded 0	Not applicable - effect size estimated from reported pooled SEM

Study	Species	Strategy	Intervention	Comparator	Outcome and unit	Intervention mean ± pooled SD	Comparator mean ± pooled SD	n intervention (tanks)	n comparator (tanks)	Effect-size metric	Effect size	95% CI	Interpretation	Reason not estimated
Yoo and Bai (2014)	Olive flounder (Paralichthys olivaceus)	Microbial phytase supplementation in soybean-meal-based low-fishmeal diets	S40P2000 (40% replacement; 2000 U phytase/kg; analyzed 2018 U/kg)	S40 (40% FM-protein replacement by soybean meal; no phytase)	Crude protein (% as-is)	17.9 ± 0.24	18.1 ± 0.24	3	3	Hedges' g using study-wide pooled SD	-0.78	-2.32 to 0.76	Lower measured value with phytase; unadjusted 95% CI included 0	Not applicable - effect size estimated from reported pooled SEM
Yoo and Bai (2014)	Olive flounder (Paralichthys olivaceus)	Microbial phytase supplementation in soybean-meal-based low-fishmeal diets	S40P2000 (40% replacement; 2000 U phytase/kg; analyzed 2018 U/kg)	S40 (40% FM-protein replacement by soybean meal; no phytase)	Crude lipid (% as-is)	3.96 ± 0.23	4.05 ± 0.23	3	3	Hedges' g using study-wide pooled SD	-0.38	-1.90 to 1.14	Lower measured value with phytase; unadjusted 95% CI included 0	Not applicable - effect size estimated from reported pooled SEM
Yoo and Bai (2014)	Olive flounder (Paralichthys olivaceus)	Microbial phytase supplementation in soybean-meal-based low-fishmeal diets	S40P2000 (40% replacement; 2000 U phytase/kg; analyzed 2018 U/kg)	S40 (40% FM-protein replacement by soybean meal; no phytase)	Ash (% as-is)	5.97 ± 0.28	6.28 ± 0.28	3	3	Hedges' g using study-wide pooled SD	-1.06	-2.62 to 0.51	Lower measured value with phytase; unadjusted 95% CI included 0	Not applicable - effect size estimated from reported pooled SEM

Note: The article reported one pooled SEM for each outcome across all seven diets. Pooled SD was calculated as pooled SEM × sqrt(3), and the same SD was used for both groups in each matched comparison. Hedges' g used residual df = 14 from seven treatments × (three tanks - 1). Positive values indicate higher values with phytase. Pairwise 95% CIs are descriptive and unadjusted and may not reproduce the original one-way ANOVA/HSD lettering.

Source: Yoo and Bai (2014), https://doi.org/10.5657/FAS.2014.0319

Study 12. Terrey et al. (2024): PHY-750 versus the negative control and inorganic-P benchmark

Study	Species	Strategy	Intervention	Comparator	Outcome and unit	Intervention mean ± SD	Comparator mean ± SD	n intervention (cages)	n comparator (cages)	Effect-size metric	Effect size	95% CI	Interpretation	Reason not estimated
Terrey et al. (2024)	Atlantic salmon (Salmo salar)	Microbial phytase supplementation in a high-plant-meal diet	PHY-750 (high-plant diet; 750 OTU/kg target, 795 OTU/kg analyzed)	NC (high-plant diet; no phytase or supplemental MCP)	Final body weight (g)	398.0 ± 6.0	379.0 ± 8.0	3	3	Hedges' g	2.15	0.19 to 4.11	Higher, more favorable value with phytase; raw-scale 95% CI excluded 0	Not applicable - effect size estimated from reported mean ± SD
Terrey et al. (2024)	Atlantic salmon (Salmo salar)	Microbial phytase supplementation in a high-plant-meal diet	PHY-750 (high-plant diet; 750 OTU/kg target, 795 OTU/kg analyzed)	NC (high-plant diet; no phytase or supplemental MCP)	Specific growth rate (% body weight/day)	1.47 ± 0.02	1.38 ± 0.04	3	3	Hedges' g	2.28	0.24 to 4.31	Higher, more favorable value with phytase; raw-scale 95% CI excluded 0	Not applicable - effect size estimated from reported mean ± SD
Terrey et al. (2024)	Atlantic salmon (Salmo salar)	Microbial phytase supplementation in a high-plant-meal diet	PHY-750 (high-plant diet; 750 OTU/kg target, 795 OTU/kg analyzed)	NC (high-plant diet; no phytase or supplemental MCP)	Feed conversion ratio (kg feed/kg gain)	0.930 ± 0.010	1.03 ± 0.02	3	3	Hedges' g	5.06	1.33 to 8.79	Lower, more favorable value with phytase; raw-scale 95% CI excluded 0	Not applicable - effect size estimated from reported mean ± SD
Terrey et al. (2024)	Atlantic salmon (Salmo salar)	Microbial phytase supplementation in a high-plant-meal diet	PHY-750 (high-plant diet; 750 OTU/kg target, 795 OTU/kg analyzed)	NC (high-plant diet; no phytase or supplemental MCP)	Feed intake (% body weight/day)	1.28 ± 0.01	1.34 ± 0.01	3	3	Hedges' g	-4.8	-8.36 to -1.24	Lower measured value with phytase; raw-scale 95% CI excluded 0	Not applicable - effect size estimated from reported mean ± SD
Terrey et al. (2024)	Atlantic salmon (Salmo salar)	Microbial phytase supplementation in a high-plant-meal diet	PHY-750 (high-plant diet; 750 OTU/kg target, 795 OTU/kg analyzed)	NC (high-plant diet; no phytase or supplemental MCP)	Protein efficiency ratio (kg gain/kg protein)	2.28 ± 0.03	2.07 ± 0.03	3	3	Hedges' g	5.6	1.51 to 9.69	Higher, more favorable value with phytase; raw-scale 95% CI excluded 0	Not applicable - effect size estimated from reported mean ± SD
Terrey et al. (2024)	Atlantic salmon (Salmo salar)	Microbial phytase supplementation in a high-plant-meal diet	PHY-750 (high-plant diet; 750 OTU/kg target, 795 OTU/kg analyzed)	NC (high-plant diet; no phytase or supplemental MCP)	Apparent digestibility coefficient of dry matter (%)	82.8 ± 0.1	82.9 ± 0.2	3	3	Hedges' g	-0.51	-1.83 to 0.82	Lower, less favorable value with phytase; raw-scale 95% CI included 0	Not applicable - effect size estimated from reported mean ± SD
Terrey et al. (2024)	Atlantic salmon (Salmo salar)	Microbial phytase supplementation in a high-plant-meal diet	PHY-750 (high-plant diet; 750 OTU/kg target, 795 OTU/kg analyzed)	NC (high-plant diet; no phytase or supplemental MCP)	Apparent digestibility coefficient of phosphorus (%)	69.5 ± 2.0	39.3 ± 1.8	3	3	Hedges' g	12.7	3.81 to 21.59	Higher, more favorable value with phytase; raw-scale 95% CI excluded 0	Not applicable - effect size estimated from reported mean ± SD
Terrey et al. (2024)	Atlantic salmon (Salmo salar)	Microbial phytase supplementation in a high-plant-meal diet	PHY-750 (high-plant diet; 750 OTU/kg target, 795 OTU/kg analyzed)	NC (high-plant diet; no phytase or supplemental MCP)	Apparent digestibility coefficient of phytate (%)	41.1 ± 2.0	35.4 ± 1.0	3	3	Hedges' g	2.88	0.51 to 5.26	Higher, more favorable value with phytase; raw-scale 95% CI excluded 0	Not applicable - effect size estimated from reported mean ± SD
Terrey et al. (2024)	Atlantic salmon (Salmo salar)	Microbial phytase supplementation in a high-plant-meal diet	PHY-750 (high-plant diet; 750 OTU/kg target, 795 OTU/kg analyzed)	NC (high-plant diet; no phytase or supplemental MCP)	Whole-body phosphorus (%)	0.330 ± 0.010	0.300 ± 0.010	3	3	Hedges' g	2.4	0.30 to 4.50	Higher measured value with phytase; raw-scale 95% CI excluded 0	Not applicable - effect size estimated from reported mean ± SD
Terrey et al. (2024)	Atlantic salmon (Salmo salar)	Microbial phytase supplementation in a high-plant-meal diet	PHY-750 (high-plant diet; 750 OTU/kg target, 795 OTU/kg analyzed)	NC (high-plant diet; no phytase or supplemental MCP)	Bone ash (%)	8.12 ± 0.24	4.88 ± 0.24	3	3	Hedges' g	10.8	3.21 to 18.39	Higher, more favorable value with phytase; raw-scale 95% CI excluded 0	Not applicable - effect size estimated from reported mean ± SD
Terrey et al. (2024)	Atlantic salmon (Salmo salar)	Microbial phytase supplementation in a high-plant-meal diet	PHY-750 (high-plant diet; 750 OTU/kg target, 795 OTU/kg analyzed)	NC (high-plant diet; no phytase or supplemental MCP)	Phosphorus retention (%)	36.6 ± 1.9	24.4 ± 2.0	3	3	Hedges' g	5.0	1.31 to 8.70	Higher, more favorable value with phytase; raw-scale 95% CI excluded 0	Not applicable - effect size estimated from reported mean ± SD
Terrey et al. (2024)	Atlantic salmon (Salmo salar)	Microbial phytase supplementation in a high-plant-meal diet	PHY-750 (high-plant diet; 750 OTU/kg target, 795 OTU/kg analyzed)	PC (high-plant diet; 2% monocalcium phosphate; no phytase)	Final body weight (g)	398.0 ± 6.0	397.0 ± 6.0	3	3	Hedges' g	0.13	-1.15 to 1.42	Higher, more favorable value with phytase; raw-scale 95% CI included 0	Not applicable - effect size estimated from reported mean ± SD
Terrey et al. (2024)	Atlantic salmon (Salmo salar)	Microbial phytase supplementation in a high-plant-meal diet	PHY-750 (high-plant diet; 750 OTU/kg target, 795 OTU/kg analyzed)	PC (high-plant diet; 2% monocalcium phosphate; no phytase)	Specific growth rate (% body weight/day)	1.47 ± 0.02	1.46 ± 0.01	3	3	Hedges' g	0.51	-0.82 to 1.83	Higher, more favorable value with phytase; raw-scale 95% CI included 0	Not applicable - effect size estimated from reported mean ± SD
Terrey et al. (2024)	Atlantic salmon (Salmo salar)	Microbial phytase supplementation in a high-plant-meal diet	PHY-750 (high-plant diet; 750 OTU/kg target, 795 OTU/kg analyzed)	PC (high-plant diet; 2% monocalcium phosphate; no phytase)	Feed conversion ratio (kg feed/kg gain)	0.930 ± 0.010	0.940 ± 0.020	3	3	Hedges' g	0.51	-0.82 to 1.83	Lower, more favorable value with phytase; raw-scale 95% CI included 0	Not applicable - effect size estimated from reported mean ± SD
Terrey et al. (2024)	Atlantic salmon (Salmo salar)	Microbial phytase supplementation in a high-plant-meal diet	PHY-750 (high-plant diet; 750 OTU/kg target, 795 OTU/kg analyzed)	PC (high-plant diet; 2% monocalcium phosphate; no phytase)	Feed intake (% body weight/day)	1.28 ± 0.01	1.28 ± 0.04	3	3	Hedges' g	0.0	-1.28 to 1.28	No observed mean difference; raw-scale 95% CI included 0	Not applicable - effect size estimated from reported mean ± SD
Terrey et al. (2024)	Atlantic salmon (Salmo salar)	Microbial phytase supplementation in a high-plant-meal diet	PHY-750 (high-plant diet; 750 OTU/kg target, 795 OTU/kg analyzed)	PC (high-plant diet; 2% monocalcium phosphate; no phytase)	Protein efficiency ratio (kg gain/kg protein)	2.28 ± 0.03	2.27 ± 0.06	3	3	Hedges' g	0.17	-1.12 to 1.45	Higher, more favorable value with phytase; raw-scale 95% CI included 0	Not applicable - effect size estimated from reported mean ± SD
Terrey et al. (2024)	Atlantic salmon (Salmo salar)	Microbial phytase supplementation in a high-plant-meal diet	PHY-750 (high-plant diet; 750 OTU/kg target, 795 OTU/kg analyzed)	PC (high-plant diet; 2% monocalcium phosphate; no phytase)	Apparent digestibility coefficient of dry matter (%)	82.8 ± 0.1	83.1 ± 0.1	3	3	Hedges' g	-2.4	-4.50 to -0.30	Lower, less favorable value with phytase; raw-scale 95% CI excluded 0	Not applicable - effect size estimated from reported mean ± SD
Terrey et al. (2024)	Atlantic salmon (Salmo salar)	Microbial phytase supplementation in a high-plant-meal diet	PHY-750 (high-plant diet; 750 OTU/kg target, 795 OTU/kg analyzed)	PC (high-plant diet; 2% monocalcium phosphate; no phytase)	Apparent digestibility coefficient of phosphorus (%)	69.5 ± 2.0	64.8 ± 1.7	3	3	Hedges' g	2.03	0.13 to 3.93	Higher, more favorable value with phytase; raw-scale 95% CI excluded 0	Not applicable - effect size estimated from reported mean ± SD
Terrey et al. (2024)	Atlantic salmon (Salmo salar)	Microbial phytase supplementation in a high-plant-meal diet	PHY-750 (high-plant diet; 750 OTU/kg target, 795 OTU/kg analyzed)	PC (high-plant diet; 2% monocalcium phosphate; no phytase)	Apparent digestibility coefficient of phytate (%)	41.1 ± 2.0	34.4 ± 2.5	3	3	Hedges' g	2.37	0.29 to 4.45	Higher, more favorable value with phytase; raw-scale 95% CI excluded 0	Not applicable - effect size estimated from reported mean ± SD
Terrey et al. (2024)	Atlantic salmon (Salmo salar)	Microbial phytase supplementation in a high-plant-meal diet	PHY-750 (high-plant diet; 750 OTU/kg target, 795 OTU/kg analyzed)	PC (high-plant diet; 2% monocalcium phosphate; no phytase)	Whole-body phosphorus (%)	0.330 ± 0.010	0.310 ± 0.030	3	3	Hedges' g	0.72	-0.66 to 2.09	Higher measured value with phytase; raw-scale 95% CI included 0	Not applicable - effect size estimated from reported mean ± SD
Terrey et al. (2024)	Atlantic salmon (Salmo salar)	Microbial phytase supplementation in a high-plant-meal diet	PHY-750 (high-plant diet; 750 OTU/kg target, 795 OTU/kg analyzed)	PC (high-plant diet; 2% monocalcium phosphate; no phytase)	Bone ash (%)	8.12 ± 0.24	8.16 ± 0.37	3	3	Hedges' g	-0.1	-1.38 to 1.18	Lower, less favorable value with phytase; raw-scale 95% CI included 0	Not applicable - effect size estimated from reported mean ± SD
Terrey et al. (2024)	Atlantic salmon (Salmo salar)	Microbial phytase supplementation in a high-plant-meal diet	PHY-750 (high-plant diet; 750 OTU/kg target, 795 OTU/kg analyzed)	PC (high-plant diet; 2% monocalcium phosphate; no phytase)	Phosphorus retention (%)	36.6 ± 1.9	20.5 ± 4.7	3	3	Hedges' g	3.59	0.79 to 6.39	Higher, more favorable value with phytase; raw-scale 95% CI excluded 0	Not applicable - effect size estimated from reported mean ± SD
Terrey et al. (2024)	Atlantic salmon (Salmo salar)	Microbial phytase supplementation in a high-plant-meal diet	PHY-750 (high-plant diet; 750 OTU/kg target, 795 OTU/kg analyzed)	NC (high-plant diet; no phytase or supplemental MCP)	Daily phosphorus gain (Figure 1)	Not reported numerically	Not reported numerically	3	3	Not estimable	Not estimable	Not estimable	The outcome was presented as a stacked bar chart with error bars and significance letters	Exact group means and SDs were not reported numerically in Figure 1
Terrey et al. (2024)	Atlantic salmon (Salmo salar)	Microbial phytase supplementation in a high-plant-meal diet	PHY-750 (high-plant diet; 750 OTU/kg target, 795 OTU/kg analyzed)	NC (high-plant diet; no phytase or supplemental MCP)	Daily faecal phosphorus loss (Figure 1)	Not reported numerically	Not reported numerically	3	3	Not estimable	Not estimable	Not estimable	The outcome was presented as a stacked bar chart with error bars and significance letters	Exact group means and SDs were not reported numerically in Figure 1
Terrey et al. (2024)	Atlantic salmon (Salmo salar)	Microbial phytase supplementation in a high-plant-meal diet	PHY-750 (high-plant diet; 750 OTU/kg target, 795 OTU/kg analyzed)	NC (high-plant diet; no phytase or supplemental MCP)	Daily metabolic phosphorus loss (Figure 1)	Not reported numerically	Not reported numerically	3	3	Not estimable	Not estimable	Not estimable	The outcome was presented as a stacked bar chart with error bars and significance letters	Exact group means and SDs were not reported numerically in Figure 1
Terrey et al. (2024)	Atlantic salmon (Salmo salar)	Microbial phytase supplementation in a high-plant-meal diet	PHY-750 (high-plant diet; 750 OTU/kg target, 795 OTU/kg analyzed)	PC (high-plant diet; 2% monocalcium phosphate; no phytase)	Daily phosphorus gain (Figure 1)	Not reported numerically	Not reported numerically	3	3	Not estimable	Not estimable	Not estimable	The outcome was presented as a stacked bar chart with error bars and significance letters	Exact group means and SDs were not reported numerically in Figure 1
Terrey et al. (2024)	Atlantic salmon (Salmo salar)	Microbial phytase supplementation in a high-plant-meal diet	PHY-750 (high-plant diet; 750 OTU/kg target, 795 OTU/kg analyzed)	PC (high-plant diet; 2% monocalcium phosphate; no phytase)	Daily faecal phosphorus loss (Figure 1)	Not reported numerically	Not reported numerically	3	3	Not estimable	Not estimable	Not estimable	The outcome was presented as a stacked bar chart with error bars and significance letters	Exact group means and SDs were not reported numerically in Figure 1
Terrey et al. (2024)	Atlantic salmon (Salmo salar)	Microbial phytase supplementation in a high-plant-meal diet	PHY-750 (high-plant diet; 750 OTU/kg target, 795 OTU/kg analyzed)	PC (high-plant diet; 2% monocalcium phosphate; no phytase)	Daily metabolic phosphorus loss (Figure 1)	Not reported numerically	Not reported numerically	3	3	Not estimable	Not estimable	Not estimable	The outcome was presented as a stacked bar chart with error bars and significance letters	Exact group means and SDs were not reported numerically in Figure 1

Note: Values are reported mean ± SD from three independent sea cages per diet. PHY-750 versus NC is the primary phytase comparison; PHY-750 versus PC is an inorganic-phosphorus benchmark. FCR was direction-reversed because lower values are favorable. Percentage outcomes were analyzed after arcsine-square-root transformation in the original study, whereas the effect sizes here use the published raw-scale means and SDs. Figure 1 phosphorus-balance outcomes were not estimable because exact group means and SDs were not reported. Pairwise 95% CIs are unadjusted.

Source: Terrey et al. (2024), https://doi.org/10.1007/s10499-023-01295-1

Study 13. Li et al. (2019): matched phytase, protease, and combined-enzyme comparisons

Study	Species	Strategy	Intervention	Comparator	Outcome and unit	Intervention mean ± SD	Comparator mean ± SD	n intervention (independent tanks)	n comparator (independent tanks)	Effect-size metric	Effect size	95% CI	Interpretation	Reason not estimated
Li et al. (2019)	Hybrid tilapia (Oreochromis niloticus × Oreochromis aureus)	Phytase and/or protease supplementation in low-phosphorus and fishmeal-free diets	LP + Ph (LP diet + 400 mg/kg phytase)	LP (60 g/kg fishmeal; 12 g/kg MCP; no enzyme)	Final body weight (g)	104.5 ± 1.3	93.3 ± 5.7	3	3	Hedges' g	2.17	0.19 to 4.14	Higher, more favorable value with enzyme supplementation; unadjusted 95% CI excluded 0	Not applicable - effect size estimated from reported mean ± SD
Li et al. (2019)	Hybrid tilapia (Oreochromis niloticus × Oreochromis aureus)	Phytase and/or protease supplementation in low-phosphorus and fishmeal-free diets	LP + Ph (LP diet + 400 mg/kg phytase)	LP (60 g/kg fishmeal; 12 g/kg MCP; no enzyme)	Weight gain (%)	1250.9 ± 27.9	1104.9 ± 81.1	3	3	Hedges' g	1.93	0.08 to 3.78	Higher, more favorable value with enzyme supplementation; unadjusted 95% CI excluded 0	Not applicable - effect size estimated from reported mean ± SD
Li et al. (2019)	Hybrid tilapia (Oreochromis niloticus × Oreochromis aureus)	Phytase and/or protease supplementation in low-phosphorus and fishmeal-free diets	LP + Ph (LP diet + 400 mg/kg phytase)	LP (60 g/kg fishmeal; 12 g/kg MCP; no enzyme)	Feed conversion ratio	1.17 ± 0.02	1.29 ± 0.07	3	3	Hedges' g	1.86	0.05 to 3.68	Lower, more favorable value with enzyme supplementation; unadjusted 95% CI excluded 0	Not applicable - effect size estimated from reported mean ± SD
Li et al. (2019)	Hybrid tilapia (Oreochromis niloticus × Oreochromis aureus)	Phytase and/or protease supplementation in low-phosphorus and fishmeal-free diets	LP + Ph (LP diet + 400 mg/kg phytase)	LP (60 g/kg fishmeal; 12 g/kg MCP; no enzyme)	Hepatosomatic index (%)	1.50 ± 0.60	1.50 ± 0.30	3	3	Hedges' g	0.0	-1.28 to 1.28	No observed mean difference; unadjusted 95% CI included 0	Not applicable - effect size estimated from reported mean ± SD
Li et al. (2019)	Hybrid tilapia (Oreochromis niloticus × Oreochromis aureus)	Phytase and/or protease supplementation in low-phosphorus and fishmeal-free diets	LP + Ph (LP diet + 400 mg/kg phytase)	LP (60 g/kg fishmeal; 12 g/kg MCP; no enzyme)	Viscerosomatic index (%)	8.10 ± 0.40	8.20 ± 1.30	3	3	Hedges' g	-0.08	-1.36 to 1.20	Lower measured value with enzyme supplementation; unadjusted 95% CI included 0	Not applicable - effect size estimated from reported mean ± SD
Li et al. (2019)	Hybrid tilapia (Oreochromis niloticus × Oreochromis aureus)	Phytase and/or protease supplementation in low-phosphorus and fishmeal-free diets	LP + Ph (LP diet + 400 mg/kg phytase)	LP (60 g/kg fishmeal; 12 g/kg MCP; no enzyme)	Condition factor (%)	6.80 ± 0.90	7.30 ± 1.50	3	3	Hedges' g	-0.32	-1.62 to 0.98	Lower measured value with enzyme supplementation; unadjusted 95% CI included 0	Not applicable - effect size estimated from reported mean ± SD
Li et al. (2019)	Hybrid tilapia (Oreochromis niloticus × Oreochromis aureus)	Phytase and/or protease supplementation in low-phosphorus and fishmeal-free diets	LP + Ph (LP diet + 400 mg/kg phytase)	LP (60 g/kg fishmeal; 12 g/kg MCP; no enzyme)	Moisture (g/kg fresh tissue)	727.8 ± 14.6	723.1 ± 15.7	3	3	Hedges' g	0.25	-1.04 to 1.54	Higher measured value with enzyme supplementation; unadjusted 95% CI included 0	Not applicable - effect size estimated from reported mean ± SD
Li et al. (2019)	Hybrid tilapia (Oreochromis niloticus × Oreochromis aureus)	Phytase and/or protease supplementation in low-phosphorus and fishmeal-free diets	LP + Ph (LP diet + 400 mg/kg phytase)	LP (60 g/kg fishmeal; 12 g/kg MCP; no enzyme)	Ash (g/kg fresh tissue)	41.0 ± 4.2	39.6 ± 3.1	3	3	Hedges' g	0.3	-0.99 to 1.60	Higher, more favorable value with enzyme supplementation; unadjusted 95% CI included 0	Not applicable - effect size estimated from reported mean ± SD
Li et al. (2019)	Hybrid tilapia (Oreochromis niloticus × Oreochromis aureus)	Phytase and/or protease supplementation in low-phosphorus and fishmeal-free diets	LP + Ph (LP diet + 400 mg/kg phytase)	LP (60 g/kg fishmeal; 12 g/kg MCP; no enzyme)	Crude protein (g/kg fresh tissue)	167.8 ± 11.3	165.6 ± 9.4	3	3	Hedges' g	0.17	-1.12 to 1.45	Higher, more favorable value with enzyme supplementation; unadjusted 95% CI included 0	Not applicable - effect size estimated from reported mean ± SD
Li et al. (2019)	Hybrid tilapia (Oreochromis niloticus × Oreochromis aureus)	Phytase and/or protease supplementation in low-phosphorus and fishmeal-free diets	LP + Ph (LP diet + 400 mg/kg phytase)	LP (60 g/kg fishmeal; 12 g/kg MCP; no enzyme)	Crude lipid (g/kg fresh tissue)	56.5 ± 5.9	64.6 ± 7.1	3	3	Hedges' g	0.99	-0.46 to 2.45	Lower, more favorable value with enzyme supplementation; unadjusted 95% CI included 0	Not applicable - effect size estimated from reported mean ± SD
Li et al. (2019)	Hybrid tilapia (Oreochromis niloticus × Oreochromis aureus)	Phytase and/or protease supplementation in low-phosphorus and fishmeal-free diets	LP + Ph (LP diet + 400 mg/kg phytase)	LP (60 g/kg fishmeal; 12 g/kg MCP; no enzyme)	Calcium (g/kg fresh tissue)	8.50 ± 0.70	8.60 ± 0.70	3	3	Hedges' g	-0.11	-1.40 to 1.17	Lower, less favorable value with enzyme supplementation; unadjusted 95% CI included 0	Not applicable - effect size estimated from reported mean ± SD
Li et al. (2019)	Hybrid tilapia (Oreochromis niloticus × Oreochromis aureus)	Phytase and/or protease supplementation in low-phosphorus and fishmeal-free diets	LP + Ph (LP diet + 400 mg/kg phytase)	LP (60 g/kg fishmeal; 12 g/kg MCP; no enzyme)	Phosphorus (g/kg fresh tissue)	6.40 ± 0.50	6.10 ± 0.50	3	3	Hedges' g	0.48	-0.84 to 1.80	Higher, more favorable value with enzyme supplementation; unadjusted 95% CI included 0	Not applicable - effect size estimated from reported mean ± SD
Li et al. (2019)	Hybrid tilapia (Oreochromis niloticus × Oreochromis aureus)	Phytase and/or protease supplementation in low-phosphorus and fishmeal-free diets	LP + Ph (LP diet + 400 mg/kg phytase)	LP (60 g/kg fishmeal; 12 g/kg MCP; no enzyme)	Lipid retention (%)	75.5 ± 3.0	80.5 ± 2.6	3	3	Hedges' g	-1.42	-3.04 to 0.19	Lower, less favorable value with enzyme supplementation; unadjusted 95% CI included 0	Not applicable - effect size estimated from reported mean ± SD
Li et al. (2019)	Hybrid tilapia (Oreochromis niloticus × Oreochromis aureus)	Phytase and/or protease supplementation in low-phosphorus and fishmeal-free diets	LP + Ph (LP diet + 400 mg/kg phytase)	LP (60 g/kg fishmeal; 12 g/kg MCP; no enzyme)	Protein retention (%)	46.8 ± 1.9	41.7 ± 1.8	3	3	Hedges' g	2.2	0.21 to 4.20	Higher, more favorable value with enzyme supplementation; unadjusted 95% CI excluded 0	Not applicable - effect size estimated from reported mean ± SD
Li et al. (2019)	Hybrid tilapia (Oreochromis niloticus × Oreochromis aureus)	Phytase and/or protease supplementation in low-phosphorus and fishmeal-free diets	LP + Ph (LP diet + 400 mg/kg phytase)	LP (60 g/kg fishmeal; 12 g/kg MCP; no enzyme)	Calcium retention (%)	102.3 ± 7.2	92.6 ± 5.8	3	3	Hedges' g	1.19	-0.33 to 2.71	Higher, more favorable value with enzyme supplementation; unadjusted 95% CI included 0	Not applicable - effect size estimated from reported mean ± SD
Li et al. (2019)	Hybrid tilapia (Oreochromis niloticus × Oreochromis aureus)	Phytase and/or protease supplementation in low-phosphorus and fishmeal-free diets	LP + Ph (LP diet + 400 mg/kg phytase)	LP (60 g/kg fishmeal; 12 g/kg MCP; no enzyme)	Phosphorus retention (%)	45.6 ± 2.0	40.7 ± 1.4	3	3	Hedges' g	2.27	0.24 to 4.30	Higher, more favorable value with enzyme supplementation; unadjusted 95% CI excluded 0	Not applicable - effect size estimated from reported mean ± SD
Li et al. (2019)	Hybrid tilapia (Oreochromis niloticus × Oreochromis aureus)	Phytase and/or protease supplementation in low-phosphorus and fishmeal-free diets	LP + Ph (LP diet + 400 mg/kg phytase)	LP (60 g/kg fishmeal; 12 g/kg MCP; no enzyme)	Dry matter digestibility (%)	56.5 ± 0.6	54.2 ± 1.1	3	3	Hedges' g	2.08	0.15 to 4.00	Higher, more favorable value with enzyme supplementation; unadjusted 95% CI excluded 0	Not applicable - effect size estimated from reported mean ± SD
Li et al. (2019)	Hybrid tilapia (Oreochromis niloticus × Oreochromis aureus)	Phytase and/or protease supplementation in low-phosphorus and fishmeal-free diets	LP + Ph (LP diet + 400 mg/kg phytase)	LP (60 g/kg fishmeal; 12 g/kg MCP; no enzyme)	Crude protein digestibility (%)	83.8 ± 0.7	82.2 ± 0.6	3	3	Hedges' g	1.96	0.10 to 3.83	Higher, more favorable value with enzyme supplementation; unadjusted 95% CI excluded 0	Not applicable - effect size estimated from reported mean ± SD
Li et al. (2019)	Hybrid tilapia (Oreochromis niloticus × Oreochromis aureus)	Phytase and/or protease supplementation in low-phosphorus and fishmeal-free diets	LP + Ph (LP diet + 400 mg/kg phytase)	LP (60 g/kg fishmeal; 12 g/kg MCP; no enzyme)	Calcium digestibility (%)	51.2 ± 1.5	46.4 ± 0.9	3	3	Hedges' g	3.1	0.60 to 5.61	Higher, more favorable value with enzyme supplementation; unadjusted 95% CI excluded 0	Not applicable - effect size estimated from reported mean ± SD
Li et al. (2019)	Hybrid tilapia (Oreochromis niloticus × Oreochromis aureus)	Phytase and/or protease supplementation in low-phosphorus and fishmeal-free diets	LP + Ph (LP diet + 400 mg/kg phytase)	LP (60 g/kg fishmeal; 12 g/kg MCP; no enzyme)	Phosphorus digestibility (%)	60.9 ± 0.5	57.2 ± 1.1	3	3	Hedges' g	3.46	0.74 to 6.19	Higher, more favorable value with enzyme supplementation; unadjusted 95% CI excluded 0	Not applicable - effect size estimated from reported mean ± SD
Li et al. (2019)	Hybrid tilapia (Oreochromis niloticus × Oreochromis aureus)	Phytase and/or protease supplementation in low-phosphorus and fishmeal-free diets	LP + Ph (LP diet + 400 mg/kg phytase)	LP (60 g/kg fishmeal; 12 g/kg MCP; no enzyme)	Anterior-intestinal villus height (µm)	369.0 ± 25.2	325.3 ± 28.5	3	3	Hedges' g	1.3	-0.27 to 2.86	Higher, more favorable value with enzyme supplementation; unadjusted 95% CI included 0	Not applicable - effect size estimated from reported mean ± SD
Li et al. (2019)	Hybrid tilapia (Oreochromis niloticus × Oreochromis aureus)	Phytase and/or protease supplementation in low-phosphorus and fishmeal-free diets	LP + Ph (LP diet + 400 mg/kg phytase)	LP (60 g/kg fishmeal; 12 g/kg MCP; no enzyme)	Anterior-intestinal villus width (µm)	88.9 ± 7.3	81.3 ± 9.2	3	3	Hedges' g	0.73	-0.64 to 2.11	Higher measured value with enzyme supplementation; unadjusted 95% CI included 0	Not applicable - effect size estimated from reported mean ± SD
Li et al. (2019)	Hybrid tilapia (Oreochromis niloticus × Oreochromis aureus)	Phytase and/or protease supplementation in low-phosphorus and fishmeal-free diets	FMF + Pr (FMF diet + 175 mg/kg protease)	FMF (fishmeal-free; 18 g/kg MCP; no enzyme)	Final body weight (g)	97.9 ± 0.8	89.3 ± 1.9	3	3	Hedges' g	4.72	1.21 to 8.23	Higher, more favorable value with enzyme supplementation; unadjusted 95% CI excluded 0	Not applicable - effect size estimated from reported mean ± SD

Study	Species	Strategy	Intervention	Comparator	Outcome and unit	Intervention mean ± SD	Comparator mean ± SD	n intervention (independent tanks)	n comparator (independent tanks)	Effect-size metric	Effect size	95% CI	Interpretation	Reason not estimated
Li et al. (2019)	Hybrid tilapia (Oreochromis niloticus × Oreochromis aureus)	Phytase and/or protease supplementation in low-phosphorus and fishmeal-free diets	FMF + Pr (FMF diet + 175 mg/kg protease)	FMF (fishmeal-free; 18 g/kg MCP; no enzyme)	Weight gain (%)	1169.8 ± 11.4	1052.8 ± 24.4	3	3	Hedges' g	4.92	1.28 to 8.55	Higher, more favorable value with enzyme supplementation; unadjusted 95% CI excluded 0	Not applicable - effect size estimated from reported mean ± SD
Li et al. (2019)	Hybrid tilapia (Oreochromis niloticus × Oreochromis aureus)	Phytase and/or protease supplementation in low-phosphorus and fishmeal-free diets	FMF + Pr (FMF diet + 175 mg/kg protease)	FMF (fishmeal-free; 18 g/kg MCP; no enzyme)	Feed conversion ratio	1.25 ± 0.01	1.35 ± 0.02	3	3	Hedges' g	5.06	1.33 to 8.79	Lower, more favorable value with enzyme supplementation; unadjusted 95% CI excluded 0	Not applicable - effect size estimated from reported mean ± SD
Li et al. (2019)	Hybrid tilapia (Oreochromis niloticus × Oreochromis aureus)	Phytase and/or protease supplementation in low-phosphorus and fishmeal-free diets	FMF + Pr (FMF diet + 175 mg/kg protease)	FMF (fishmeal-free; 18 g/kg MCP; no enzyme)	Hepatosomatic index (%)	1.50 ± 0.30	1.50 ± 0.40	3	3	Hedges' g	0.0	-1.28 to 1.28	No observed mean difference; unadjusted 95% CI included 0	Not applicable - effect size estimated from reported mean ± SD
Li et al. (2019)	Hybrid tilapia (Oreochromis niloticus × Oreochromis aureus)	Phytase and/or protease supplementation in low-phosphorus and fishmeal-free diets	FMF + Pr (FMF diet + 175 mg/kg protease)	FMF (fishmeal-free; 18 g/kg MCP; no enzyme)	Viscerosomatic index (%)	8.00 ± 0.80	7.90 ± 1.00	3	3	Hedges' g	0.09	-1.19 to 1.37	Higher measured value with enzyme supplementation; unadjusted 95% CI included 0	Not applicable - effect size estimated from reported mean ± SD
Li et al. (2019)	Hybrid tilapia (Oreochromis niloticus × Oreochromis aureus)	Phytase and/or protease supplementation in low-phosphorus and fishmeal-free diets	FMF + Pr (FMF diet + 175 mg/kg protease)	FMF (fishmeal-free; 18 g/kg MCP; no enzyme)	Condition factor (%)	6.90 ± 0.60	6.90 ± 0.60	3	3	Hedges' g	0.0	-1.28 to 1.28	No observed mean difference; unadjusted 95% CI included 0	Not applicable - effect size estimated from reported mean ± SD
Li et al. (2019)	Hybrid tilapia (Oreochromis niloticus × Oreochromis aureus)	Phytase and/or protease supplementation in low-phosphorus and fishmeal-free diets	FMF + Pr (FMF diet + 175 mg/kg protease)	FMF (fishmeal-free; 18 g/kg MCP; no enzyme)	Moisture (g/kg fresh tissue)	728.0 ± 11.0	727.8 ± 13.0	3	3	Hedges' g	0.01	-1.27 to 1.29	Higher measured value with enzyme supplementation; unadjusted 95% CI included 0	Not applicable - effect size estimated from reported mean ± SD
Li et al. (2019)	Hybrid tilapia (Oreochromis niloticus × Oreochromis aureus)	Phytase and/or protease supplementation in low-phosphorus and fishmeal-free diets	FMF + Pr (FMF diet + 175 mg/kg protease)	FMF (fishmeal-free; 18 g/kg MCP; no enzyme)	Ash (g/kg fresh tissue)	39.7 ± 3.5	40.3 ± 4.0	3	3	Hedges' g	-0.13	-1.41 to 1.16	Lower, less favorable value with enzyme supplementation; unadjusted 95% CI included 0	Not applicable - effect size estimated from reported mean ± SD
Li et al. (2019)	Hybrid tilapia (Oreochromis niloticus × Oreochromis aureus)	Phytase and/or protease supplementation in low-phosphorus and fishmeal-free diets	FMF + Pr (FMF diet + 175 mg/kg protease)	FMF (fishmeal-free; 18 g/kg MCP; no enzyme)	Crude protein (g/kg fresh tissue)	165.4 ± 8.2	172.1 ± 18.1	3	3	Hedges' g	-0.38	-1.69 to 0.93	Lower, less favorable value with enzyme supplementation; unadjusted 95% CI included 0	Not applicable - effect size estimated from reported mean ± SD
Li et al. (2019)	Hybrid tilapia (Oreochromis niloticus × Oreochromis aureus)	Phytase and/or protease supplementation in low-phosphorus and fishmeal-free diets	FMF + Pr (FMF diet + 175 mg/kg protease)	FMF (fishmeal-free; 18 g/kg MCP; no enzyme)	Crude lipid (g/kg fresh tissue)	54.7 ± 5.3	62.9 ± 6.5	3	3	Hedges' g	1.11	-0.39 to 2.60	Lower, more favorable value with enzyme supplementation; unadjusted 95% CI included 0	Not applicable - effect size estimated from reported mean ± SD
Li et al. (2019)	Hybrid tilapia (Oreochromis niloticus × Oreochromis aureus)	Phytase and/or protease supplementation in low-phosphorus and fishmeal-free diets	FMF + Pr (FMF diet + 175 mg/kg protease)	FMF (fishmeal-free; 18 g/kg MCP; no enzyme)	Calcium (g/kg fresh tissue)	8.10 ± 0.80	8.50 ± 1.00	3	3	Hedges' g	-0.35	-1.66 to 0.95	Lower, less favorable value with enzyme supplementation; unadjusted 95% CI included 0	Not applicable - effect size estimated from reported mean ± SD
Li et al. (2019)	Hybrid tilapia (Oreochromis niloticus × Oreochromis aureus)	Phytase and/or protease supplementation in low-phosphorus and fishmeal-free diets	FMF + Pr (FMF diet + 175 mg/kg protease)	FMF (fishmeal-free; 18 g/kg MCP; no enzyme)	Phosphorus (g/kg fresh tissue)	6.50 ± 0.60	6.10 ± 0.40	3	3	Hedges' g	0.63	-0.72 to 1.98	Higher, more favorable value with enzyme supplementation; unadjusted 95% CI included 0	Not applicable - effect size estimated from reported mean ± SD
Li et al. (2019)	Hybrid tilapia (Oreochromis niloticus × Oreochromis aureus)	Phytase and/or protease supplementation in low-phosphorus and fishmeal-free diets	FMF + Pr (FMF diet + 175 mg/kg protease)	FMF (fishmeal-free; 18 g/kg MCP; no enzyme)	Lipid retention (%)	70.1 ± 2.3	75.3 ± 3.1	3	3	Hedges' g	-1.52	-3.18 to 0.14	Lower, less favorable value with enzyme supplementation; unadjusted 95% CI included 0	Not applicable - effect size estimated from reported mean ± SD
Li et al. (2019)	Hybrid tilapia (Oreochromis niloticus × Oreochromis aureus)	Phytase and/or protease supplementation in low-phosphorus and fishmeal-free diets	FMF + Pr (FMF diet + 175 mg/kg protease)	FMF (fishmeal-free; 18 g/kg MCP; no enzyme)	Protein retention (%)	43.8 ± 1.2	40.6 ± 1.8	3	3	Hedges' g	1.67	-0.05 to 3.40	Higher, more favorable value with enzyme supplementation; unadjusted 95% CI included 0	Not applicable - effect size estimated from reported mean ± SD
Li et al. (2019)	Hybrid tilapia (Oreochromis niloticus × Oreochromis aureus)	Phytase and/or protease supplementation in low-phosphorus and fishmeal-free diets	FMF + Pr (FMF diet + 175 mg/kg protease)	FMF (fishmeal-free; 18 g/kg MCP; no enzyme)	Calcium retention (%)	111.7 ± 6.7	106.7 ± 7.3	3	3	Hedges' g	0.57	-0.77 to 1.91	Higher, more favorable value with enzyme supplementation; unadjusted 95% CI included 0	Not applicable - effect size estimated from reported mean ± SD
Li et al. (2019)	Hybrid tilapia (Oreochromis niloticus × Oreochromis aureus)	Phytase and/or protease supplementation in low-phosphorus and fishmeal-free diets	FMF + Pr (FMF diet + 175 mg/kg protease)	FMF (fishmeal-free; 18 g/kg MCP; no enzyme)	Phosphorus retention (%)	43.9 ± 2.2	38.0 ± 1.9	3	3	Hedges' g	2.3	0.25 to 4.34	Higher, more favorable value with enzyme supplementation; unadjusted 95% CI excluded 0	Not applicable - effect size estimated from reported mean ± SD
Li et al. (2019)	Hybrid tilapia (Oreochromis niloticus × Oreochromis aureus)	Phytase and/or protease supplementation in low-phosphorus and fishmeal-free diets	FMF + Pr (FMF diet + 175 mg/kg protease)	FMF (fishmeal-free; 18 g/kg MCP; no enzyme)	Dry matter digestibility (%)	57.2 ± 1.1	53.0 ± 0.4	3	3	Hedges' g	4.06	0.97 to 7.15	Higher, more favorable value with enzyme supplementation; unadjusted 95% CI excluded 0	Not applicable - effect size estimated from reported mean ± SD
Li et al. (2019)	Hybrid tilapia (Oreochromis niloticus × Oreochromis aureus)	Phytase and/or protease supplementation in low-phosphorus and fishmeal-free diets	FMF + Pr (FMF diet + 175 mg/kg protease)	FMF (fishmeal-free; 18 g/kg MCP; no enzyme)	Crude protein digestibility (%)	83.1 ± 0.3	81.2 ± 0.5	3	3	Hedges' g	3.69	0.83 to 6.54	Higher, more favorable value with enzyme supplementation; unadjusted 95% CI excluded 0	Not applicable - effect size estimated from reported mean ± SD
Li et al. (2019)	Hybrid tilapia (Oreochromis niloticus × Oreochromis aureus)	Phytase and/or protease supplementation in low-phosphorus and fishmeal-free diets	FMF + Pr (FMF diet + 175 mg/kg protease)	FMF (fishmeal-free; 18 g/kg MCP; no enzyme)	Calcium digestibility (%)	48.9 ± 1.6	48.0 ± 0.9	3	3	Hedges' g	0.55	-0.78 to 1.89	Higher, more favorable value with enzyme supplementation; unadjusted 95% CI included 0	Not applicable - effect size estimated from reported mean ± SD
Li et al. (2019)	Hybrid tilapia (Oreochromis niloticus × Oreochromis aureus)	Phytase and/or protease supplementation in low-phosphorus and fishmeal-free diets	FMF + Pr (FMF diet + 175 mg/kg protease)	FMF (fishmeal-free; 18 g/kg MCP; no enzyme)	Phosphorus digestibility (%)	62.4 ± 1.3	58.3 ± 1.2	3	3	Hedges' g	2.62	0.40 to 4.84	Higher, more favorable value with enzyme supplementation; unadjusted 95% CI excluded 0	Not applicable - effect size estimated from reported mean ± SD
Li et al. (2019)	Hybrid tilapia (Oreochromis niloticus × Oreochromis aureus)	Phytase and/or protease supplementation in low-phosphorus and fishmeal-free diets	FMF + Pr (FMF diet + 175 mg/kg protease)	FMF (fishmeal-free; 18 g/kg MCP; no enzyme)	Anterior-intestinal villus height (µm)	405.8 ± 34.4	322.0 ± 37.1	3	3	Hedges' g	1.87	0.05 to 3.70	Higher, more favorable value with enzyme supplementation; unadjusted 95% CI excluded 0	Not applicable - effect size estimated from reported mean ± SD
Li et al. (2019)	Hybrid tilapia (Oreochromis niloticus × Oreochromis aureus)	Phytase and/or protease supplementation in low-phosphorus and fishmeal-free diets	FMF + Pr (FMF diet + 175 mg/kg protease)	FMF (fishmeal-free; 18 g/kg MCP; no enzyme)	Anterior-intestinal villus width (µm)	88.7 ± 9.5	78.5 ± 7.4	3	3	Hedges' g	0.96	-0.48 to 2.40	Higher measured value with enzyme supplementation; unadjusted 95% CI included 0	Not applicable - effect size estimated from reported mean ± SD
Li et al. (2019)	Hybrid tilapia (Oreochromis niloticus × Oreochromis aureus)	Phytase and/or protease supplementation in low-phosphorus and fishmeal-free diets	LP-FMF + Ph + Pr (LP-FMF diet + phytase + protease)	LP-FMF (fishmeal-free; 12 g/kg MCP; no enzyme)	Final body weight (g)	94.5 ± 0.1	84.4 ± 1.9	3	3	Hedges' g	6.01	1.65 to 10.36	Higher, more favorable value with enzyme supplementation; unadjusted 95% CI excluded 0	Not applicable - effect size estimated from reported mean ± SD
Li et al. (2019)	Hybrid tilapia (Oreochromis niloticus × Oreochromis aureus)	Phytase and/or protease supplementation in low-phosphorus and fishmeal-free diets	LP-FMF + Ph + Pr (LP-FMF diet + phytase + protease)	LP-FMF (fishmeal-free; 12 g/kg MCP; no enzyme)	Weight gain (%)	1127.8 ± 2.8	997.8 ± 28.7	3	3	Hedges' g	5.1	1.34 to 8.86	Higher, more favorable value with enzyme supplementation; unadjusted 95% CI excluded 0	Not applicable - effect size estimated from reported mean ± SD

Study	Species	Strategy	Intervention	Comparator	Outcome and unit	Intervention mean ± SD	Comparator mean ± SD	n intervention (independent tanks)	n comparator (independent tanks)	Effect-size metric	Effect size	95% CI	Interpretation	Reason not estimated
Li et al. (2019)	Hybrid tilapia (Oreochromis niloticus × Oreochromis aureus)	Phytase and/or protease supplementation in low-phosphorus and fishmeal-free diets	LP-FMF + Ph + Pr (LP-FMF diet + phytase + protease)	LP-FMF (fishmeal-free; 12 g/kg MCP; no enzyme)	Feed conversion ratio	1.30 ± 0.01	1.41 ± 0.03	3	3	Hedges' g	3.94	0.92 to 6.95	Lower, more favorable value with enzyme supplementation; unadjusted 95% CI excluded 0	Not applicable - effect size estimated from reported mean ± SD
Li et al. (2019)	Hybrid tilapia (Oreochromis niloticus × Oreochromis aureus)	Phytase and/or protease supplementation in low-phosphorus and fishmeal-free diets	LP-FMF + Ph + Pr (LP-FMF diet + phytase + protease)	LP-FMF (fishmeal-free; 12 g/kg MCP; no enzyme)	Hepatosomatic index (%)	1.60 ± 0.50	1.60 ± 0.30	3	3	Hedges' g	0.0	-1.28 to 1.28	No observed mean difference; unadjusted 95% CI included 0	Not applicable - effect size estimated from reported mean ± SD
Li et al. (2019)	Hybrid tilapia (Oreochromis niloticus × Oreochromis aureus)	Phytase and/or protease supplementation in low-phosphorus and fishmeal-free diets	LP-FMF + Ph + Pr (LP-FMF diet + phytase + protease)	LP-FMF (fishmeal-free; 12 g/kg MCP; no enzyme)	Viscerosomatic index (%)	8.70 ± 0.80	8.50 ± 0.80	3	3	Hedges' g	0.2	-1.09 to 1.49	Higher measured value with enzyme supplementation; unadjusted 95% CI included 0	Not applicable - effect size estimated from reported mean ± SD
Li et al. (2019)	Hybrid tilapia (Oreochromis niloticus × Oreochromis aureus)	Phytase and/or protease supplementation in low-phosphorus and fishmeal-free diets	LP-FMF + Ph + Pr (LP-FMF diet + phytase + protease)	LP-FMF (fishmeal-free; 12 g/kg MCP; no enzyme)	Condition factor (%)	6.80 ± 0.30	7.70 ± 0.90	3	3	Hedges' g	-1.07	-2.55 to 0.41	Lower measured value with enzyme supplementation; unadjusted 95% CI included 0	Not applicable - effect size estimated from reported mean ± SD
Li et al. (2019)	Hybrid tilapia (Oreochromis niloticus × Oreochromis aureus)	Phytase and/or protease supplementation in low-phosphorus and fishmeal-free diets	LP-FMF + Ph + Pr (LP-FMF diet + phytase + protease)	LP-FMF (fishmeal-free; 12 g/kg MCP; no enzyme)	Moisture (g/kg fresh tissue)	724.4 ± 13.1	726.4 ± 1.4	3	3	Hedges' g	-0.17	-1.46 to 1.11	Lower measured value with enzyme supplementation; unadjusted 95% CI included 0	Not applicable - effect size estimated from reported mean ± SD
Li et al. (2019)	Hybrid tilapia (Oreochromis niloticus × Oreochromis aureus)	Phytase and/or protease supplementation in low-phosphorus and fishmeal-free diets	LP-FMF + Ph + Pr (LP-FMF diet + phytase + protease)	LP-FMF (fishmeal-free; 12 g/kg MCP; no enzyme)	Ash (g/kg fresh tissue)	38.0 ± 3.7	34.4 ± 2.7	3	3	Hedges' g	0.89	-0.53 to 2.31	Higher, more favorable value with enzyme supplementation; unadjusted 95% CI included 0	Not applicable - effect size estimated from reported mean ± SD
Li et al. (2019)	Hybrid tilapia (Oreochromis niloticus × Oreochromis aureus)	Phytase and/or protease supplementation in low-phosphorus and fishmeal-free diets	LP-FMF + Ph + Pr (LP-FMF diet + phytase + protease)	LP-FMF (fishmeal-free; 12 g/kg MCP; no enzyme)	Crude protein (g/kg fresh tissue)	165.4 ± 6.7	170.0 ± 5.6	3	3	Hedges' g	-0.6	-1.94 to 0.75	Lower, less favorable value with enzyme supplementation; unadjusted 95% CI included 0	Not applicable - effect size estimated from reported mean ± SD
Li et al. (2019)	Hybrid tilapia (Oreochromis niloticus × Oreochromis aureus)	Phytase and/or protease supplementation in low-phosphorus and fishmeal-free diets	LP-FMF + Ph + Pr (LP-FMF diet + phytase + protease)	LP-FMF (fishmeal-free; 12 g/kg MCP; no enzyme)	Crude lipid (g/kg fresh tissue)	62.3 ± 5.4	62.5 ± 3.0	3	3	Hedges' g	0.04	-1.24 to 1.32	Lower, more favorable value with enzyme supplementation; unadjusted 95% CI included 0	Not applicable - effect size estimated from reported mean ± SD
Li et al. (2019)	Hybrid tilapia (Oreochromis niloticus × Oreochromis aureus)	Phytase and/or protease supplementation in low-phosphorus and fishmeal-free diets	LP-FMF + Ph + Pr (LP-FMF diet + phytase + protease)	LP-FMF (fishmeal-free; 12 g/kg MCP; no enzyme)	Calcium (g/kg fresh tissue)	8.00 ± 1.10	7.20 ± 0.30	3	3	Hedges' g	0.79	-0.60 to 2.19	Higher, more favorable value with enzyme supplementation; unadjusted 95% CI included 0	Not applicable - effect size estimated from reported mean ± SD
Li et al. (2019)	Hybrid tilapia (Oreochromis niloticus × Oreochromis aureus)	Phytase and/or protease supplementation in low-phosphorus and fishmeal-free diets	LP-FMF + Ph + Pr (LP-FMF diet + phytase + protease)	LP-FMF (fishmeal-free; 12 g/kg MCP; no enzyme)	Phosphorus (g/kg fresh tissue)	6.20 ± 0.60	5.30 ± 0.40	3	3	Hedges' g	1.41	-0.20 to 3.02	Higher, more favorable value with enzyme supplementation; unadjusted 95% CI included 0	Not applicable - effect size estimated from reported mean ± SD
Li et al. (2019)	Hybrid tilapia (Oreochromis niloticus × Oreochromis aureus)	Phytase and/or protease supplementation in low-phosphorus and fishmeal-free diets	LP-FMF + Ph + Pr (LP-FMF diet + phytase + protease)	LP-FMF (fishmeal-free; 12 g/kg MCP; no enzyme)	Lipid retention (%)	74.9 ± 3.0	71.9 ± 2.5	3	3	Hedges' g	0.87	-0.55 to 2.28	Higher, more favorable value with enzyme supplementation; unadjusted 95% CI included 0	Not applicable - effect size estimated from reported mean ± SD
Li et al. (2019)	Hybrid tilapia (Oreochromis niloticus × Oreochromis aureus)	Phytase and/or protease supplementation in low-phosphorus and fishmeal-free diets	LP-FMF + Ph + Pr (LP-FMF diet + phytase + protease)	LP-FMF (fishmeal-free; 12 g/kg MCP; no enzyme)	Protein retention (%)	41.0 ± 0.8	37.9 ± 1.0	3	3	Hedges' g	2.74	0.45 to 5.03	Higher, more favorable value with enzyme supplementation; unadjusted 95% CI excluded 0	Not applicable - effect size estimated from reported mean ± SD
Li et al. (2019)	Hybrid tilapia (Oreochromis niloticus × Oreochromis aureus)	Phytase and/or protease supplementation in low-phosphorus and fishmeal-free diets	LP-FMF + Ph + Pr (LP-FMF diet + phytase + protease)	LP-FMF (fishmeal-free; 12 g/kg MCP; no enzyme)	Calcium retention (%)	124.5 ± 5.3	107.2 ± 8.1	3	3	Hedges' g	2.02	0.12 to 3.92	Higher, more favorable value with enzyme supplementation; unadjusted 95% CI excluded 0	Not applicable - effect size estimated from reported mean ± SD
Li et al. (2019)	Hybrid tilapia (Oreochromis niloticus × Oreochromis aureus)	Phytase and/or protease supplementation in low-phosphorus and fishmeal-free diets	LP-FMF + Ph + Pr (LP-FMF diet + phytase + protease)	LP-FMF (fishmeal-free; 12 g/kg MCP; no enzyme)	Phosphorus retention (%)	43.5 ± 1.5	35.1 ± 1.6	3	3	Hedges' g	4.33	1.07 to 7.60	Higher, more favorable value with enzyme supplementation; unadjusted 95% CI excluded 0	Not applicable - effect size estimated from reported mean ± SD
Li et al. (2019)	Hybrid tilapia (Oreochromis niloticus × Oreochromis aureus)	Phytase and/or protease supplementation in low-phosphorus and fishmeal-free diets	LP-FMF + Ph + Pr (LP-FMF diet + phytase + protease)	LP-FMF (fishmeal-free; 12 g/kg MCP; no enzyme)	Dry matter digestibility (%)	56.9 ± 0.5	53.9 ± 1.3	3	3	Hedges' g	2.44	0.32 to 4.56	Higher, more favorable value with enzyme supplementation; unadjusted 95% CI excluded 0	Not applicable - effect size estimated from reported mean ± SD
Li et al. (2019)	Hybrid tilapia (Oreochromis niloticus × Oreochromis aureus)	Phytase and/or protease supplementation in low-phosphorus and fishmeal-free diets	LP-FMF + Ph + Pr (LP-FMF diet + phytase + protease)	LP-FMF (fishmeal-free; 12 g/kg MCP; no enzyme)	Crude protein digestibility (%)	82.9 ± 0.3	81.5 ± 0.6	3	3	Hedges' g	2.36	0.28 to 4.44	Higher, more favorable value with enzyme supplementation; unadjusted 95% CI excluded 0	Not applicable - effect size estimated from reported mean ± SD
Li et al. (2019)	Hybrid tilapia (Oreochromis niloticus × Oreochromis aureus)	Phytase and/or protease supplementation in low-phosphorus and fishmeal-free diets	LP-FMF + Ph + Pr (LP-FMF diet + phytase + protease)	LP-FMF (fishmeal-free; 12 g/kg MCP; no enzyme)	Calcium digestibility (%)	48.0 ± 1.9	40.4 ± 1.2	3	3	Hedges' g	3.83	0.88 to 6.77	Higher, more favorable value with enzyme supplementation; unadjusted 95% CI excluded 0	Not applicable - effect size estimated from reported mean ± SD
Li et al. (2019)	Hybrid tilapia (Oreochromis niloticus × Oreochromis aureus)	Phytase and/or protease supplementation in low-phosphorus and fishmeal-free diets	LP-FMF + Ph + Pr (LP-FMF diet + phytase + protease)	LP-FMF (fishmeal-free; 12 g/kg MCP; no enzyme)	Phosphorus digestibility (%)	61.0 ± 0.6	55.7 ± 1.5	3	3	Hedges' g	3.71	0.84 to 6.58	Higher, more favorable value with enzyme supplementation; unadjusted 95% CI excluded 0	Not applicable - effect size estimated from reported mean ± SD
Li et al. (2019)	Hybrid tilapia (Oreochromis niloticus × Oreochromis aureus)	Phytase and/or protease supplementation in low-phosphorus and fishmeal-free diets	LP-FMF + Ph + Pr (LP-FMF diet + phytase + protease)	LP-FMF (fishmeal-free; 12 g/kg MCP; no enzyme)	Anterior-intestinal villus height (µm)	385.7 ± 32.7	316.5 ± 40.9	3	3	Hedges' g	1.5	-0.15 to 3.14	Higher, more favorable value with enzyme supplementation; unadjusted 95% CI included 0	Not applicable - effect size estimated from reported mean ± SD
Li et al. (2019)	Hybrid tilapia (Oreochromis niloticus × Oreochromis aureus)	Phytase and/or protease supplementation in low-phosphorus and fishmeal-free diets	LP-FMF + Ph + Pr (LP-FMF diet + phytase + protease)	LP-FMF (fishmeal-free; 12 g/kg MCP; no enzyme)	Anterior-intestinal villus width (µm)	84.9 ± 6.2	90.1 ± 8.3	3	3	Hedges' g	-0.57	-1.91 to 0.77	Lower measured value with enzyme supplementation; unadjusted 95% CI included 0	Not applicable - effect size estimated from reported mean ± SD
Li et al. (2019)	Hybrid tilapia (Oreochromis niloticus × Oreochromis aureus)	Phytase and/or protease supplementation in low-phosphorus and fishmeal-free diets	LP + Ph (LP diet + 400 mg/kg phytase)	LP (60 g/kg fishmeal; 12 g/kg MCP; no enzyme)	Survival rate (%)	100.0; SD not reported	100.0; SD not reported	3	3	Not estimable	Not estimable	Not estimable	No observed survival difference	All groups had 100% survival and no dispersion was reported; standardized effect size is undefined.
Li et al. (2019)	Hybrid tilapia (Oreochromis niloticus × Oreochromis aureus)	Phytase and/or protease supplementation in low-phosphorus and fishmeal-free diets	FMF + Pr (FMF diet + 175 mg/kg protease)	FMF (fishmeal-free; 18 g/kg MCP; no enzyme)	Survival rate (%)	100.0; SD not reported	100.0; SD not reported	3	3	Not estimable	Not estimable	Not estimable	No observed survival difference	All groups had 100% survival and no dispersion was reported; standardized effect size is undefined.
Li et al. (2019)	Hybrid tilapia (Oreochromis niloticus × Oreochromis aureus)	Phytase and/or protease supplementation in low-phosphorus and fishmeal-free diets	LP-FMF + Ph + Pr (LP-FMF diet + phytase + protease)	LP-FMF (fishmeal-free; 12 g/kg MCP; no enzyme)	Survival rate (%)	100.0; SD not reported	100.0; SD not reported	3	3	Not estimable	Not estimable	Not estimable	No observed survival difference	All groups had 100% survival and no dispersion was reported; standardized effect size is undefined.

Note: The tank was treated as the independent experimental unit (n = 3 per group). Reported SDs were used directly. For whole-body composition and intestinal morphology, fish were subsampled within tanks; reported individual-level SDs were retained for the standardized mean difference, while CI precision was based on three independent tanks. FCR and whole-body crude lipid were direction-reversed. Survival was not estimable because all groups had 100% survival and no dispersion was reported. Pairwise 95% CIs are unadjusted.

Source: Li et al. (2019), https://doi.org/10.1111/anu.12828

Study 14. Sajjadi and Carter (2004): matched phytase comparisons with and without supplemental inorganic phosphorus

Study	Species	Strategy	Intervention	Comparator	Outcome and unit	Intervention mean ± SD	Comparator mean ± SD	n intervention (tanks)	n comparator (tanks)	Effect-size metric	Effect size	95% CI	Interpretation	Reason not estimated
Sajjadi and Carter (2004)	Atlantic salmon (Salmo salar)	Phytase supplementation in a canola-meal-based, available-phosphorus-deficient diet	CP (2000 U phytase/kg; no supplemental inorganic phosphorus)	C (no phytase; no supplemental inorganic phosphorus)	Final body weight (g)	307.20 ± 5.72	292.70 ± 14.90	3	3	Hedges' g	1.03	-0.44 to 2.49	Higher, more favorable value with phytase; unadjusted 95% CI included 0	Not applicable - effect size estimated after converting SEM to SD
Sajjadi and Carter (2004)	Atlantic salmon (Salmo salar)	Phytase supplementation in a canola-meal-based, available-phosphorus-deficient diet	CP (2000 U phytase/kg; no supplemental inorganic phosphorus)	C (no phytase; no supplemental inorganic phosphorus)	Weight gain (g)	206.90 ± 5.72	192.30 ± 14.03	3	3	Hedges' g	1.09	-0.40 to 2.58	Higher, more favorable value with phytase; unadjusted 95% CI included 0	Not applicable - effect size estimated after converting SEM to SD
Sajjadi and Carter (2004)	Atlantic salmon (Salmo salar)	Phytase supplementation in a canola-meal-based, available-phosphorus-deficient diet	CP (2000 U phytase/kg; no supplemental inorganic phosphorus)	C (no phytase; no supplemental inorganic phosphorus)	Total feed intake (kg dry matter)	4.15 ± 0.12	3.88 ± 0.16	3	3	Hedges' g	1.55	-0.12 to 3.22	Higher measured value with phytase; unadjusted 95% CI included 0	Not applicable - effect size estimated after converting SEM to SD
Sajjadi and Carter (2004)	Atlantic salmon (Salmo salar)	Phytase supplementation in a canola-meal-based, available-phosphorus-deficient diet	CP (2000 U phytase/kg; no supplemental inorganic phosphorus)	C (no phytase; no supplemental inorganic phosphorus)	Feed efficiency ratio (g gain/g dry feed)	0.99 ± 0.02	0.99 ± 0.02	3	3	Hedges' g	0.0	-1.28 to 1.28	No observed mean difference; unadjusted 95% CI included 0	Not applicable - effect size estimated after converting SEM to SD
Sajjadi and Carter (2004)	Atlantic salmon (Salmo salar)	Phytase supplementation in a canola-meal-based, available-phosphorus-deficient diet	CP (2000 U phytase/kg; no supplemental inorganic phosphorus)	C (no phytase; no supplemental inorganic phosphorus)	Dry matter (% wet weight)	31.87 ± 0.43	31.74 ± 0.90	3	3	Hedges' g	0.15	-1.14 to 1.43	Higher measured value with phytase; unadjusted 95% CI included 0	Not applicable - effect size estimated after converting SEM to SD
Sajjadi and Carter (2004)	Atlantic salmon (Salmo salar)	Phytase supplementation in a canola-meal-based, available-phosphorus-deficient diet	CP (2000 U phytase/kg; no supplemental inorganic phosphorus)	C (no phytase; no supplemental inorganic phosphorus)	Crude protein (% wet weight)	17.90 ± 0.24	18.12 ± 0.33	3	3	Hedges' g	-0.61	-1.96 to 0.74	Lower measured value with phytase; unadjusted 95% CI included 0	Not applicable - effect size estimated after converting SEM to SD
Sajjadi and Carter (2004)	Atlantic salmon (Salmo salar)	Phytase supplementation in a canola-meal-based, available-phosphorus-deficient diet	CP (2000 U phytase/kg; no supplemental inorganic phosphorus)	C (no phytase; no supplemental inorganic phosphorus)	Total lipid (% wet weight)	10.66 ± 0.28	10.39 ± 0.21	3	3	Hedges' g	0.88	-0.54 to 2.30	Higher measured value with phytase; unadjusted 95% CI included 0	Not applicable - effect size estimated after converting SEM to SD
Sajjadi and Carter (2004)	Atlantic salmon (Salmo salar)	Phytase supplementation in a canola-meal-based, available-phosphorus-deficient diet	CP (2000 U phytase/kg; no supplemental inorganic phosphorus)	C (no phytase; no supplemental inorganic phosphorus)	Ash (% wet weight)	2.00 ± 0.02	1.91 ± 0.03	3	3	Hedges' g	2.63	0.40 to 4.86	Higher measured value with phytase; unadjusted 95% CI excluded 0	Not applicable - effect size estimated after converting SEM to SD
Sajjadi and Carter (2004)	Atlantic salmon (Salmo salar)	Phytase supplementation in a canola-meal-based, available-phosphorus-deficient diet	CP (2000 U phytase/kg; no supplemental inorganic phosphorus)	C (no phytase; no supplemental inorganic phosphorus)	Whole-body phosphorus (% wet weight)	1.33 ± 0.03	1.21 ± 0.03	3	3	Hedges' g	2.77	0.46 to 5.08	Higher, more favorable value with phytase; unadjusted 95% CI excluded 0	Not applicable - effect size estimated after converting SEM to SD
Sajjadi and Carter (2004)	Atlantic salmon (Salmo salar)	Phytase supplementation in a canola-meal-based, available-phosphorus-deficient diet	CP (2000 U phytase/kg; no supplemental inorganic phosphorus)	C (no phytase; no supplemental inorganic phosphorus)	Bone ash (%)	50.15 ± 1.33	46.53 ± 1.30	3	3	Hedges' g	2.2	0.21 to 4.19	Higher, more favorable value with phytase; unadjusted 95% CI excluded 0	Not applicable - effect size estimated after converting SEM to SD
Sajjadi and Carter (2004)	Atlantic salmon (Salmo salar)	Phytase supplementation in a canola-meal-based, available-phosphorus-deficient diet	CP (2000 U phytase/kg; no supplemental inorganic phosphorus)	C (no phytase; no supplemental inorganic phosphorus)	Bone phosphorus (%)	10.62 ± 0.17	9.78 ± 0.40	3	3	Hedges' g	2.19	0.20 to 4.17	Higher, more favorable value with phytase; unadjusted 95% CI excluded 0	Not applicable - effect size estimated after converting SEM to SD
Sajjadi and Carter (2004)	Atlantic salmon (Salmo salar)	Phytase supplementation in a canola-meal-based, available-phosphorus-deficient diet	CP (2000 U phytase/kg; no supplemental inorganic phosphorus)	C (no phytase; no supplemental inorganic phosphorus)	Apparent digestibility coefficient of crude protein (%)	94.89 ± 0.33	95.09 ± 0.23	3	3	Hedges' g	-0.57	-1.91 to 0.77	Lower, less favorable value with phytase; unadjusted 95% CI included 0	Not applicable - effect size estimated after converting SEM to SD
Sajjadi and Carter (2004)	Atlantic salmon (Salmo salar)	Phytase supplementation in a canola-meal-based, available-phosphorus-deficient diet	CP (2000 U phytase/kg; no supplemental inorganic phosphorus)	C (no phytase; no supplemental inorganic phosphorus)	Apparent digestibility coefficient of energy (%)	86.24 ± 0.43	84.76 ± 0.52	3	3	Hedges' g	2.48	0.34 to 4.62	Higher, more favorable value with phytase; unadjusted 95% CI excluded 0	Not applicable - effect size estimated after converting SEM to SD
Sajjadi and Carter (2004)	Atlantic salmon (Salmo salar)	Phytase supplementation in a canola-meal-based, available-phosphorus-deficient diet	CP (2000 U phytase/kg; no supplemental inorganic phosphorus)	C (no phytase; no supplemental inorganic phosphorus)	Apparent digestibility coefficient of phosphorus (%)	74.06 ± 0.74	63.84 ± 2.13	3	3	Hedges' g	5.12	1.35 to 8.90	Higher, more favorable value with phytase; unadjusted 95% CI excluded 0	Not applicable - effect size estimated after converting SEM to SD
Sajjadi and Carter (2004)	Atlantic salmon (Salmo salar)	Phytase supplementation in a canola-meal-based, available-phosphorus-deficient diet	CSP (2000 U phytase/kg + 10 g/kg dibasic sodium phosphate)	CS (no phytase; 10 g/kg dibasic sodium phosphate)	Final body weight (g)	313.00 ± 9.87	320.80 ± 18.71	3	3	Hedges' g	-0.42	-1.73 to 0.90	Lower, less favorable value with phytase; unadjusted 95% CI included 0	Not applicable - effect size estimated after converting SEM to SD
Sajjadi and Carter (2004)	Atlantic salmon (Salmo salar)	Phytase supplementation in a canola-meal-based, available-phosphorus-deficient diet	CSP (2000 U phytase/kg + 10 g/kg dibasic sodium phosphate)	CS (no phytase; 10 g/kg dibasic sodium phosphate)	Weight gain (g)	211.60 ± 10.57	220.30 ± 18.53	3	3	Hedges' g	-0.46	-1.78 to 0.86	Lower, less favorable value with phytase; unadjusted 95% CI included 0	Not applicable - effect size estimated after converting SEM to SD
Sajjadi and Carter (2004)	Atlantic salmon (Salmo salar)	Phytase supplementation in a canola-meal-based, available-phosphorus-deficient diet	CSP (2000 U phytase/kg + 10 g/kg dibasic sodium phosphate)	CS (no phytase; 10 g/kg dibasic sodium phosphate)	Total feed intake (kg dry matter)	4.10 ± 0.26	4.39 ± 0.31	3	3	Hedges' g	-0.81	-2.21 to 0.59	Lower measured value with phytase; unadjusted 95% CI included 0	Not applicable - effect size estimated after converting SEM to SD
Sajjadi and Carter (2004)	Atlantic salmon (Salmo salar)	Phytase supplementation in a canola-meal-based, available-phosphorus-deficient diet	CSP (2000 U phytase/kg + 10 g/kg dibasic sodium phosphate)	CS (no phytase; 10 g/kg dibasic sodium phosphate)	Feed efficiency ratio (g gain/g dry feed)	1.00 ± 0.07	1.00 ± 0.02	3	3	Hedges' g	0.0	-1.28 to 1.28	No observed mean difference; unadjusted 95% CI included 0	Not applicable - effect size estimated after converting SEM to SD
Sajjadi and Carter (2004)	Atlantic salmon (Salmo salar)	Phytase supplementation in a canola-meal-based, available-phosphorus-deficient diet	CSP (2000 U phytase/kg + 10 g/kg dibasic sodium phosphate)	CS (no phytase; 10 g/kg dibasic sodium phosphate)	Dry matter (% wet weight)	31.83 ± 0.74	31.67 ± 0.47	3	3	Hedges' g	0.21	-1.08 to 1.49	Higher measured value with phytase; unadjusted 95% CI included 0	Not applicable - effect size estimated after converting SEM to SD
Sajjadi and Carter (2004)	Atlantic salmon (Salmo salar)	Phytase supplementation in a canola-meal-based, available-phosphorus-deficient diet	CSP (2000 U phytase/kg + 10 g/kg dibasic sodium phosphate)	CS (no phytase; 10 g/kg dibasic sodium phosphate)	Crude protein (% wet weight)	18.12 ± 0.47	17.73 ± 0.52	3	3	Hedges' g	0.63	-0.72 to 1.98	Higher measured value with phytase; unadjusted 95% CI included 0	Not applicable - effect size estimated after converting SEM to SD
Sajjadi and Carter (2004)	Atlantic salmon (Salmo salar)	Phytase supplementation in a canola-meal-based, available-phosphorus-deficient diet	CSP (2000 U phytase/kg + 10 g/kg dibasic sodium phosphate)	CS (no phytase; 10 g/kg dibasic sodium phosphate)	Total lipid (% wet weight)	10.58 ± 0.40	10.89 ± 0.36	3	3	Hedges' g	-0.65	-2.01 to 0.71	Lower measured value with phytase; unadjusted 95% CI included 0	Not applicable - effect size estimated after converting SEM to SD
Sajjadi and Carter (2004)	Atlantic salmon (Salmo salar)	Phytase supplementation in a canola-meal-based, available-phosphorus-deficient diet	CSP (2000 U phytase/kg + 10 g/kg dibasic sodium phosphate)	CS (no phytase; 10 g/kg dibasic sodium phosphate)	Ash (% wet weight)	2.01 ± 0.02	2.00 ± 0.07	3	3	Hedges' g	0.16	-1.13 to 1.44	Higher measured value with phytase; unadjusted 95% CI included 0	Not applicable - effect size estimated after converting SEM to SD
Sajjadi and Carter (2004)	Atlantic salmon (Salmo salar)	Phytase supplementation in a canola-meal-based, available-phosphorus-deficient diet	CSP (2000 U phytase/kg + 10 g/kg dibasic sodium phosphate)	CS (no phytase; 10 g/kg dibasic sodium phosphate)	Whole-body phosphorus (% wet weight)	1.32 ± 0.03	1.32 ± 0.03	3	3	Hedges' g	0.0	-1.28 to 1.28	No observed mean difference; unadjusted 95% CI included 0	Not applicable - effect size estimated after converting SEM to SD
Sajjadi and Carter (2004)	Atlantic salmon (Salmo salar)	Phytase supplementation in a canola-meal-based, available-phosphorus-deficient diet	CSP (2000 U phytase/kg + 10 g/kg dibasic sodium phosphate)	CS (no phytase; 10 g/kg dibasic sodium phosphate)	Bone ash (%)	50.52 ± 0.43	50.38 ± 1.33	3	3	Hedges' g	0.11	-1.17 to 1.40	Higher, more favorable value with phytase; unadjusted 95% CI included 0	Not applicable - effect size estimated after converting SEM to SD
Sajjadi and Carter (2004)	Atlantic salmon (Salmo salar)	Phytase supplementation in a canola-meal-based, available-phosphorus-deficient diet	CSP (2000 U phytase/kg + 10 g/kg dibasic sodium phosphate)	CS (no phytase; 10 g/kg dibasic sodium phosphate)	Bone phosphorus (%)	10.71 ± 0.19	10.59 ± 0.14	3	3	Hedges' g	0.58	-0.76 to 1.92	Higher, more favorable value with phytase; unadjusted 95% CI included 0	Not applicable - effect size estimated after converting SEM to SD
Sajjadi and Carter (2004)	Atlantic salmon (Salmo salar)	Phytase supplementation in a canola-meal-based, available-phosphorus-deficient diet	CSP (2000 U phytase/kg + 10 g/kg dibasic sodium phosphate)	CS (no phytase; 10 g/kg dibasic sodium phosphate)	Apparent digestibility coefficient of crude protein (%)	94.95 ± 0.14	94.90 ± 0.14	3	3	Hedges' g	0.29	-1.01 to 1.58	Higher, more favorable value with phytase; unadjusted 95% CI included 0	Not applicable - effect size estimated after converting SEM to SD
Sajjadi and Carter (2004)	Atlantic salmon (Salmo salar)	Phytase supplementation in a canola-meal-based, available-phosphorus-deficient diet	CSP (2000 U phytase/kg + 10 g/kg dibasic sodium phosphate)	CS (no phytase; 10 g/kg dibasic sodium phosphate)	Apparent digestibility coefficient of energy (%)	84.52 ± 1.00	83.90 ± 0.74	3	3	Hedges' g	0.56	-0.78 to 1.90	Higher, more favorable value with phytase; unadjusted 95% CI included 0	Not applicable - effect size estimated after converting SEM to SD
Sajjadi and Carter (2004)	Atlantic salmon (Salmo salar)	Phytase supplementation in a canola-meal-based, available-phosphorus-deficient diet	CSP (2000 U phytase/kg + 10 g/kg dibasic sodium phosphate)	CS (no phytase; 10 g/kg dibasic sodium phosphate)	Apparent digestibility coefficient of phosphorus (%)	69.15 ± 1.18	68.33 ± 1.78	3	3	Hedges' g	0.43	-0.88 to 1.75	Higher, more favorable value with phytase; unadjusted 95% CI included 0	Not applicable - effect size estimated after converting SEM to SD
Sajjadi and Carter (2004)	Atlantic salmon (Salmo salar)	Phytase supplementation in a canola-meal-based, available-phosphorus-deficient diet	CP (2000 U phytase/kg; no supplemental inorganic phosphorus)	C (no phytase; no supplemental inorganic phosphorus)	Survival rate (%)	100.0; SD not reported	100.0; SD not reported	3	3	Not estimable	Not estimable	Not estimable	No exact standardized effect could be calculated	All groups had 100% survival; no dispersion was reported, so a standardized effect size is undefined.
Sajjadi and Carter (2004)	Atlantic salmon (Salmo salar)	Phytase supplementation in a canola-meal-based, available-phosphorus-deficient diet	CP (2000 U phytase/kg; no supplemental inorganic phosphorus)	C (no phytase; no supplemental inorganic phosphorus)	Phosphorus retention efficiency (Figure 1)	Not reported numerically	Not reported numerically	3	3	Not estimable	Not estimable	Not estimable	No exact standardized effect could be calculated	The outcome was reported graphically without exact treatment means and SEMs.

Study	Species	Strategy	Intervention	Comparator	Outcome and unit	Intervention mean ± SD	Comparator mean ± SD	n intervention (tanks)	n comparator (tanks)	Effect-size metric	Effect size	95% CI	Interpretation	Reason not estimated
Sajjadi and Carter (2004)	Atlantic salmon (Salmo salar)	Phytase supplementation in a canola-meal-based, available-phosphorus-deficient diet	CP (2000 U phytase/kg; no supplemental inorganic phosphorus)	C (no phytase; no supplemental inorganic phosphorus)	Phosphorus load (Figure 2)	Not reported numerically	Not reported numerically	3	3	Not estimable	Not estimable	Not estimable	No exact standardized effect could be calculated	The outcome was reported graphically without exact treatment means and SEMs.
Sajjadi and Carter (2004)	Atlantic salmon (Salmo salar)	Phytase supplementation in a canola-meal-based, available-phosphorus-deficient diet	CSP (2000 U phytase/kg + 10 g/kg dibasic sodium phosphate)	CS (no phytase; 10 g/kg dibasic sodium phosphate)	Survival rate (%)	100.0; SD not reported	100.0; SD not reported	3	3	Not estimable	Not estimable	Not estimable	No exact standardized effect could be calculated	All groups had 100% survival; no dispersion was reported, so a standardized effect size is undefined.
Sajjadi and Carter (2004)	Atlantic salmon (Salmo salar)	Phytase supplementation in a canola-meal-based, available-phosphorus-deficient diet	CSP (2000 U phytase/kg + 10 g/kg dibasic sodium phosphate)	CS (no phytase; 10 g/kg dibasic sodium phosphate)	Phosphorus retention efficiency (Figure 1)	Not reported numerically	Not reported numerically	3	3	Not estimable	Not estimable	Not estimable	No exact standardized effect could be calculated	The outcome was reported graphically without exact treatment means and SEMs.
Sajjadi and Carter (2004)	Atlantic salmon (Salmo salar)	Phytase supplementation in a canola-meal-based, available-phosphorus-deficient diet	CSP (2000 U phytase/kg + 10 g/kg dibasic sodium phosphate)	CS (no phytase; 10 g/kg dibasic sodium phosphate)	Phosphorus load (Figure 2)	Not reported numerically	Not reported numerically	3	3	Not estimable	Not estimable	Not estimable	No exact standardized effect could be calculated	The outcome was reported graphically without exact treatment means and SEMs.

Note: Reported SEMs were converted to SD using SD = SEM × sqrt(3), with three independent tanks per diet. CP versus C is the phytase effect without supplemental inorganic phosphorus; CSP versus CS is the phytase effect with inorganic phosphorus. Survival and the graph-only phosphorus retention/load outcomes were not estimable. The uploaded PDF was an abstract/preview, so table values were reconstructed from an accessible full-text rendering. Pairwise 95% CIs are descriptive and unadjusted and do not replace the original 2 × 2 factorial analysis.

Source: Sajjadi and Carter (2004), https://doi.org/10.1016/j.aquaculture.2004.07.003

Study 15. Carter and Sajjadi (2011): graded direct phytase supplementation and phytase pre-treatment of SPC

Study	Species	Strategy	Intervention	Comparator	Outcome and unit	Intervention mean +/- SD	Comparator mean +/- SD	n intervention (tanks)	n comparator (tanks)	Effect-size metric	Effect size	95% CI	Interpretation	Reason not estimated
Carter and Sajjadi (2011)	Atlantic salmon (Salmo salar)	Direct phytase supplementation or phytase pre-treatment of soy protein concentrate in a low-fishmeal diet	Direct phytase 250 U/kg diet	Control (0 U phytase/kg; untreated SPC)	Final body weight (g)	84.32 +/- 1.45	82.35 +/- 1.42	3	3	Hedges' g	1.1	-0.39 to 2.58	Higher, more favorable value with phytase; unadjusted 95% CI included 0	Not applicable - effect size estimated after converting SEM to SD
Carter and Sajjadi (2011)	Atlantic salmon (Salmo salar)	Direct phytase supplementation or phytase pre-treatment of soy protein concentrate in a low-fishmeal diet	Direct phytase 250 U/kg diet	Control (0 U phytase/kg; untreated SPC)	Weight gain (g)	38.59 +/- 4.36	36.77 +/- 2.75	3	3	Hedges' g	0.4	-0.91 to 1.71	Higher, more favorable value with phytase; unadjusted 95% CI included 0	Not applicable - effect size estimated after converting SEM to SD
Carter and Sajjadi (2011)	Atlantic salmon (Salmo salar)	Direct phytase supplementation or phytase pre-treatment of soy protein concentrate in a low-fishmeal diet	Direct phytase 250 U/kg diet	Control (0 U phytase/kg; untreated SPC)	Total feed intake (kg dry matter)	1.040 +/- 0.005	1.000 +/- 0.016	3	3	Hedges' g	2.75	0.46 to 5.05	Higher measured value with phytase; unadjusted 95% CI excluded 0	Not applicable - effect size estimated after converting SEM to SD
Carter and Sajjadi (2011)	Atlantic salmon (Salmo salar)	Direct phytase supplementation or phytase pre-treatment of soy protein concentrate in a low-fishmeal diet	Direct phytase 250 U/kg diet	Control (0 U phytase/kg; untreated SPC)	Feed efficiency ratio (g gain/g dry feed)	0.740 +/- 0.090	0.740 +/- 0.045	3	3	Hedges' g	0.0	-1.28 to 1.28	No observed mean difference; unadjusted 95% CI included 0	Not applicable - effect size estimated after converting SEM to SD
Carter and Sajjadi (2011)	Atlantic salmon (Salmo salar)	Direct phytase supplementation or phytase pre-treatment of soy protein concentrate in a low-fishmeal diet	Direct phytase 250 U/kg diet	Control (0 U phytase/kg; untreated SPC)	Dry matter (% wet weight)	33.83 +/- 0.17	33.81 +/- 0.43	3	3	Hedges' g	0.05	-1.23 to 1.33	Higher measured value with phytase; unadjusted 95% CI included 0	Not applicable - effect size estimated after converting SEM to SD
Carter and Sajjadi (2011)	Atlantic salmon (Salmo salar)	Direct phytase supplementation or phytase pre-treatment of soy protein concentrate in a low-fishmeal diet	Direct phytase 250 U/kg diet	Control (0 U phytase/kg; untreated SPC)	Crude protein (% wet weight)	17.42 +/- 0.14	17.21 +/- 0.33	3	3	Hedges' g	0.67	-0.70 to 2.03	Higher measured value with phytase; unadjusted 95% CI included 0	Not applicable - effect size estimated after converting SEM to SD
Carter and Sajjadi (2011)	Atlantic salmon (Salmo salar)	Direct phytase supplementation or phytase pre-treatment of soy protein concentrate in a low-fishmeal diet	Direct phytase 250 U/kg diet	Control (0 U phytase/kg; untreated SPC)	Total lipid (% wet weight)	10.66 +/- 0.26	10.60 +/- 0.21	3	3	Hedges' g	0.2	-1.08 to 1.49	Higher measured value with phytase; unadjusted 95% CI included 0	Not applicable - effect size estimated after converting SEM to SD
Carter and Sajjadi (2011)	Atlantic salmon (Salmo salar)	Direct phytase supplementation or phytase pre-treatment of soy protein concentrate in a low-fishmeal diet	Direct phytase 250 U/kg diet	Control (0 U phytase/kg; untreated SPC)	Ash (% wet weight)	5.630 +/- 0.225	5.450 +/- 0.139	3	3	Hedges' g	0.77	-0.62 to 2.16	Higher, more favorable value with phytase; unadjusted 95% CI included 0	Not applicable - effect size estimated after converting SEM to SD
Carter and Sajjadi (2011)	Atlantic salmon (Salmo salar)	Direct phytase supplementation or phytase pre-treatment of soy protein concentrate in a low-fishmeal diet	Direct phytase 250 U/kg diet	Control (0 U phytase/kg; untreated SPC)	Whole-body phosphorus (% wet weight)	1.290 +/- 0.010	1.230 +/- 0.019	3	3	Hedges' g	3.13	0.61 to 5.65	Higher, more favorable value with phytase; unadjusted 95% CI excluded 0	Not applicable - effect size estimated after converting SEM to SD
Carter and Sajjadi (2011)	Atlantic salmon (Salmo salar)	Direct phytase supplementation or phytase pre-treatment of soy protein concentrate in a low-fishmeal diet	Direct phytase 250 U/kg diet	Control (0 U phytase/kg; untreated SPC)	Bone ash (% bone weight)	45.85 +/- 1.85	44.58 +/- 0.24	3	3	Hedges' g	0.77	-0.62 to 2.16	Higher, more favorable value with phytase; unadjusted 95% CI included 0	Not applicable - effect size estimated after converting SEM to SD
Carter and Sajjadi (2011)	Atlantic salmon (Salmo salar)	Direct phytase supplementation or phytase pre-treatment of soy protein concentrate in a low-fishmeal diet	Direct phytase 250 U/kg diet	Control (0 U phytase/kg; untreated SPC)	Bone phosphorus (% bone weight)	10.53 +/- 0.09	10.54 +/- 0.31	3	3	Hedges' g	-0.03	-1.32 to 1.25	Lower, less favorable value with phytase; unadjusted 95% CI included 0	Not applicable - effect size estimated after converting SEM to SD
Carter and Sajjadi (2011)	Atlantic salmon (Salmo salar)	Direct phytase supplementation or phytase pre-treatment of soy protein concentrate in a low-fishmeal diet	Direct phytase 250 U/kg diet	Control (0 U phytase/kg; untreated SPC)	Apparent digestibility of calcium (%)	0.580 +/- 8.262	-15.51 +/- 10.12	3	3	Hedges' g	1.39	-0.21 to 3.00	Higher, more favorable value with phytase; unadjusted 95% CI included 0	Not applicable - effect size estimated after converting SEM to SD
Carter and Sajjadi (2011)	Atlantic salmon (Salmo salar)	Direct phytase supplementation or phytase pre-treatment of soy protein concentrate in a low-fishmeal diet	Direct phytase 250 U/kg diet	Control (0 U phytase/kg; untreated SPC)	Apparent digestibility of magnesium (%)	56.54 +/- 3.78	53.56 +/- 5.01	3	3	Hedges' g	0.54	-0.80 to 1.87	Higher, more favorable value with phytase; unadjusted 95% CI included 0	Not applicable - effect size estimated after converting SEM to SD
Carter and Sajjadi (2011)	Atlantic salmon (Salmo salar)	Direct phytase supplementation or phytase pre-treatment of soy protein concentrate in a low-fishmeal diet	Direct phytase 250 U/kg diet	Control (0 U phytase/kg; untreated SPC)	Apparent digestibility of copper (%)	48.57 +/- 5.75	55.07 +/- 1.35	3	3	Hedges' g	-1.24	-2.79 to 0.30	Lower, less favorable value with phytase; unadjusted 95% CI included 0	Not applicable - effect size estimated after converting SEM to SD
Carter and Sajjadi (2011)	Atlantic salmon (Salmo salar)	Direct phytase supplementation or phytase pre-treatment of soy protein concentrate in a low-fishmeal diet	Direct phytase 250 U/kg diet	Control (0 U phytase/kg; untreated SPC)	Apparent digestibility of manganese (%)	17.57 +/- 8.61	18.34 +/- 8.61	3	3	Hedges' g	-0.07	-1.35 to 1.21	Lower, less favorable value with phytase; unadjusted 95% CI included 0	Not applicable - effect size estimated after converting SEM to SD
Carter and Sajjadi (2011)	Atlantic salmon (Salmo salar)	Direct phytase supplementation or phytase pre-treatment of soy protein concentrate in a low-fishmeal diet	Direct phytase 250 U/kg diet	Control (0 U phytase/kg; untreated SPC)	Apparent digestibility of strontium (%)	10.44 +/- 7.97	7.930 +/- 8.245	3	3	Hedges' g	0.25	-1.04 to 1.54	Higher, more favorable value with phytase; unadjusted 95% CI included 0	Not applicable - effect size estimated after converting SEM to SD
Carter and Sajjadi (2011)	Atlantic salmon (Salmo salar)	Direct phytase supplementation or phytase pre-treatment of soy protein concentrate in a low-fishmeal diet	Direct phytase 250 U/kg diet	Control (0 U phytase/kg; untreated SPC)	Apparent digestibility of zinc (%)	61.35 +/- 2.03	58.18 +/- 4.14	3	3	Hedges' g	0.78	-0.61 to 2.17	Higher, more favorable value with phytase; unadjusted 95% CI included 0	Not applicable - effect size estimated after converting SEM to SD
Carter and Sajjadi (2011)	Atlantic salmon (Salmo salar)	Direct phytase supplementation or phytase pre-treatment of soy protein concentrate in a low-fishmeal diet	Direct phytase 500 U/kg diet	Control (0 U phytase/kg; untreated SPC)	Final body weight (g)	84.50 +/- 1.47	82.35 +/- 1.42	3	3	Hedges' g	1.19	-0.33 to 2.71	Higher, more favorable value with phytase; unadjusted 95% CI included 0	Not applicable - effect size estimated after converting SEM to SD
Carter and Sajjadi (2011)	Atlantic salmon (Salmo salar)	Direct phytase supplementation or phytase pre-treatment of soy protein concentrate in a low-fishmeal diet	Direct phytase 500 U/kg diet	Control (0 U phytase/kg; untreated SPC)	Weight gain (g)	39.09 +/- 5.82	36.77 +/- 2.75	3	3	Hedges' g	0.41	-0.90 to 1.72	Higher, more favorable value with phytase; unadjusted 95% CI included 0	Not applicable - effect size estimated after converting SEM to SD

Study	Species	Strategy	Intervention	Comparator	Outcome and unit	Intervention mean +/- SD	Comparator mean +/- SD	n intervention (tanks)	n comparator (tanks)	Effect-size metric	Effect size	95% CI	Interpretation	Reason not estimated
Carter and Sajjadi (2011)	Atlantic salmon (Salmo salar)	Direct phytase supplementation or phytase pre-treatment of soy protein concentrate in a low-fishmeal diet	Direct phytase 500 U/kg diet	Control (0 U phytase/kg; untreated SPC)	Total feed intake (kg dry matter)	1.020 +/- 0.003	1.000 +/- 0.016	3	3	Hedges' g	1.42	-0.20 to 3.03	Higher measured value with phytase; unadjusted 95% CI included 0	Not applicable - effect size estimated after converting SEM to SD
Carter and Sajjadi (2011)	Atlantic salmon (Salmo salar)	Direct phytase supplementation or phytase pre-treatment of soy protein concentrate in a low-fishmeal diet	Direct phytase 500 U/kg diet	Control (0 U phytase/kg; untreated SPC)	Feed efficiency ratio (g gain/g dry feed)	0.770 +/- 0.111	0.740 +/- 0.045	3	3	Hedges' g	0.28	-1.01 to 1.58	Higher, more favorable value with phytase; unadjusted 95% CI included 0	Not applicable - effect size estimated after converting SEM to SD
Carter and Sajjadi (2011)	Atlantic salmon (Salmo salar)	Direct phytase supplementation or phytase pre-treatment of soy protein concentrate in a low-fishmeal diet	Direct phytase 500 U/kg diet	Control (0 U phytase/kg; untreated SPC)	Dry matter (% wet weight)	33.71 +/- 0.26	33.81 +/- 0.43	3	3	Hedges' g	-0.22	-1.51 to 1.07	Lower measured value with phytase; unadjusted 95% CI included 0	Not applicable - effect size estimated after converting SEM to SD
Carter and Sajjadi (2011)	Atlantic salmon (Salmo salar)	Direct phytase supplementation or phytase pre-treatment of soy protein concentrate in a low-fishmeal diet	Direct phytase 500 U/kg diet	Control (0 U phytase/kg; untreated SPC)	Crude protein (% wet weight)	17.27 +/- 0.12	17.21 +/- 0.33	3	3	Hedges' g	0.19	-1.09 to 1.48	Higher measured value with phytase; unadjusted 95% CI included 0	Not applicable - effect size estimated after converting SEM to SD
Carter and Sajjadi (2011)	Atlantic salmon (Salmo salar)	Direct phytase supplementation or phytase pre-treatment of soy protein concentrate in a low-fishmeal diet	Direct phytase 500 U/kg diet	Control (0 U phytase/kg; untreated SPC)	Total lipid (% wet weight)	10.51 +/- 0.40	10.60 +/- 0.21	3	3	Hedges' g	-0.23	-1.52 to 1.06	Lower measured value with phytase; unadjusted 95% CI included 0	Not applicable - effect size estimated after converting SEM to SD
Carter and Sajjadi (2011)	Atlantic salmon (Salmo salar)	Direct phytase supplementation or phytase pre-treatment of soy protein concentrate in a low-fishmeal diet	Direct phytase 500 U/kg diet	Control (0 U phytase/kg; untreated SPC)	Ash (% wet weight)	5.990 +/- 0.294	5.450 +/- 0.139	3	3	Hedges' g	1.88	0.05 to 3.70	Higher, more favorable value with phytase; unadjusted 95% CI excluded 0	Not applicable - effect size estimated after converting SEM to SD
Carter and Sajjadi (2011)	Atlantic salmon (Salmo salar)	Direct phytase supplementation or phytase pre-treatment of soy protein concentrate in a low-fishmeal diet	Direct phytase 500 U/kg diet	Control (0 U phytase/kg; untreated SPC)	Whole-body phosphorus (% wet weight)	1.320 +/- 0.057	1.230 +/- 0.019	3	3	Hedges' g	1.69	-0.05 to 3.43	Higher, more favorable value with phytase; unadjusted 95% CI included 0	Not applicable - effect size estimated after converting SEM to SD
Carter and Sajjadi (2011)	Atlantic salmon (Salmo salar)	Direct phytase supplementation or phytase pre-treatment of soy protein concentrate in a low-fishmeal diet	Direct phytase 500 U/kg diet	Control (0 U phytase/kg; untreated SPC)	Bone ash (% bone weight)	46.65 +/- 1.11	44.58 +/- 0.24	3	3	Hedges' g	2.06	0.14 to 3.98	Higher, more favorable value with phytase; unadjusted 95% CI excluded 0	Not applicable - effect size estimated after converting SEM to SD
Carter and Sajjadi (2011)	Atlantic salmon (Salmo salar)	Direct phytase supplementation or phytase pre-treatment of soy protein concentrate in a low-fishmeal diet	Direct phytase 500 U/kg diet	Control (0 U phytase/kg; untreated SPC)	Bone phosphorus (% bone weight)	10.85 +/- 0.10	10.54 +/- 0.31	3	3	Hedges' g	1.07	-0.41 to 2.55	Higher, more favorable value with phytase; unadjusted 95% CI included 0	Not applicable - effect size estimated after converting SEM to SD
Carter and Sajjadi (2011)	Atlantic salmon (Salmo salar)	Direct phytase supplementation or phytase pre-treatment of soy protein concentrate in a low-fishmeal diet	Direct phytase 500 U/kg diet	Control (0 U phytase/kg; untreated SPC)	Apparent digestibility of calcium (%)	-0.170 +/- 4.988	-15.51 +/- 10.12	3	3	Hedges' g	1.54	-0.13 to 3.21	Higher, more favorable value with phytase; unadjusted 95% CI included 0	Not applicable - effect size estimated after converting SEM to SD
Carter and Sajjadi (2011)	Atlantic salmon (Salmo salar)	Direct phytase supplementation or phytase pre-treatment of soy protein concentrate in a low-fishmeal diet	Direct phytase 500 U/kg diet	Control (0 U phytase/kg; untreated SPC)	Apparent digestibility of magnesium (%)	56.99 +/- 3.64	53.56 +/- 5.01	3	3	Hedges' g	0.63	-0.72 to 1.98	Higher, more favorable value with phytase; unadjusted 95% CI included 0	Not applicable - effect size estimated after converting SEM to SD
Carter and Sajjadi (2011)	Atlantic salmon (Salmo salar)	Direct phytase supplementation or phytase pre-treatment of soy protein concentrate in a low-fishmeal diet	Direct phytase 500 U/kg diet	Control (0 U phytase/kg; untreated SPC)	Apparent digestibility of copper (%)	49.09 +/- 2.94	55.07 +/- 1.35	3	3	Hedges' g	-2.09	-4.02 to -0.16	Lower, less favorable value with phytase; unadjusted 95% CI excluded 0	Not applicable - effect size estimated after converting SEM to SD
Carter and Sajjadi (2011)	Atlantic salmon (Salmo salar)	Direct phytase supplementation or phytase pre-treatment of soy protein concentrate in a low-fishmeal diet	Direct phytase 500 U/kg diet	Control (0 U phytase/kg; untreated SPC)	Apparent digestibility of manganese (%)	16.84 +/- 3.33	18.34 +/- 8.61	3	3	Hedges' g	-0.18	-1.47 to 1.10	Lower, less favorable value with phytase; unadjusted 95% CI included 0	Not applicable - effect size estimated after converting SEM to SD
Carter and Sajjadi (2011)	Atlantic salmon (Salmo salar)	Direct phytase supplementation or phytase pre-treatment of soy protein concentrate in a low-fishmeal diet	Direct phytase 500 U/kg diet	Control (0 U phytase/kg; untreated SPC)	Apparent digestibility of strontium (%)	7.960 +/- 3.741	7.930 +/- 8.245	3	3	Hedges' g	0.0	-1.28 to 1.28	Higher, more favorable value with phytase; unadjusted 95% CI included 0	Not applicable - effect size estimated after converting SEM to SD
Carter and Sajjadi (2011)	Atlantic salmon (Salmo salar)	Direct phytase supplementation or phytase pre-treatment of soy protein concentrate in a low-fishmeal diet	Direct phytase 500 U/kg diet	Control (0 U phytase/kg; untreated SPC)	Apparent digestibility of zinc (%)	67.88 +/- 1.59	58.18 +/- 4.14	3	3	Hedges' g	2.47	0.33 to 4.61	Higher, more favorable value with phytase; unadjusted 95% CI excluded 0	Not applicable - effect size estimated after converting SEM to SD
Carter and Sajjadi (2011)	Atlantic salmon (Salmo salar)	Direct phytase supplementation or phytase pre-treatment of soy protein concentrate in a low-fishmeal diet	Direct phytase 1000 U/kg diet	Control (0 U phytase/kg; untreated SPC)	Final body weight (g)	89.10 +/- 1.54	82.35 +/- 1.42	3	3	Hedges' g	3.64	0.81 to 6.47	Higher, more favorable value with phytase; unadjusted 95% CI excluded 0	Not applicable - effect size estimated after converting SEM to SD
Carter and Sajjadi (2011)	Atlantic salmon (Salmo salar)	Direct phytase supplementation or phytase pre-treatment of soy protein concentrate in a low-fishmeal diet	Direct phytase 1000 U/kg diet	Control (0 U phytase/kg; untreated SPC)	Weight gain (g)	43.48 +/- 0.21	36.77 +/- 2.75	3	3	Hedges' g	2.75	0.45 to 5.04	Higher, more favorable value with phytase; unadjusted 95% CI excluded 0	Not applicable - effect size estimated after converting SEM to SD
Carter and Sajjadi (2011)	Atlantic salmon (Salmo salar)	Direct phytase supplementation or phytase pre-treatment of soy protein concentrate in a low-fishmeal diet	Direct phytase 1000 U/kg diet	Control (0 U phytase/kg; untreated SPC)	Total feed intake (kg dry matter)	1.140 +/- 0.005	1.000 +/- 0.016	3	3	Hedges' g	9.64	2.84 to 16.44	Higher measured value with phytase; unadjusted 95% CI excluded 0	Not applicable - effect size estimated after converting SEM to SD
Carter and Sajjadi (2011)	Atlantic salmon (Salmo salar)	Direct phytase supplementation or phytase pre-treatment of soy protein concentrate in a low-fishmeal diet	Direct phytase 1000 U/kg diet	Control (0 U phytase/kg; untreated SPC)	Feed efficiency ratio (g gain/g dry feed)	0.760 +/- 0.003	0.740 +/- 0.045	3	3	Hedges' g	0.5	-0.83 to 1.83	Higher, more favorable value with phytase; unadjusted 95% CI included 0	Not applicable - effect size estimated after converting SEM to SD

Study	Species	Strategy	Intervention	Comparator	Outcome and unit	Intervention mean +/- SD	Comparator mean +/- SD	n intervention (tanks)	n comparator (tanks)	Effect-size metric	Effect size	95% CI	Interpretation	Reason not estimated
Carter and Sajjadi (2011)	Atlantic salmon (Salmo salar)	Direct phytase supplementation or phytase pre-treatment of soy protein concentrate in a low-fishmeal diet	Direct phytase 1000 U/kg diet	Control (0 U phytase/kg; untreated SPC)	Dry matter (% wet weight)	33.66 +/- 0.19	33.81 +/- 0.43	3	3	Hedges' g	-0.36	-1.66 to 0.95	Lower measured value with phytase; unadjusted 95% CI included 0	Not applicable - effect size estimated after converting SEM to SD
Carter and Sajjadi (2011)	Atlantic salmon (Salmo salar)	Direct phytase supplementation or phytase pre-treatment of soy protein concentrate in a low-fishmeal diet	Direct phytase 1000 U/kg diet	Control (0 U phytase/kg; untreated SPC)	Crude protein (% wet weight)	17.02 +/- 0.16	17.21 +/- 0.33	3	3	Hedges' g	-0.59	-1.93 to 0.75	Lower measured value with phytase; unadjusted 95% CI included 0	Not applicable - effect size estimated after converting SEM to SD
Carter and Sajjadi (2011)	Atlantic salmon (Salmo salar)	Direct phytase supplementation or phytase pre-treatment of soy protein concentrate in a low-fishmeal diet	Direct phytase 1000 U/kg diet	Control (0 U phytase/kg; untreated SPC)	Total lipid (% wet weight)	10.32 +/- 0.36	10.60 +/- 0.21	3	3	Hedges' g	-0.76	-2.14 to 0.63	Lower measured value with phytase; unadjusted 95% CI included 0	Not applicable - effect size estimated after converting SEM to SD
Carter and Sajjadi (2011)	Atlantic salmon (Salmo salar)	Direct phytase supplementation or phytase pre-treatment of soy protein concentrate in a low-fishmeal diet	Direct phytase 1000 U/kg diet	Control (0 U phytase/kg; untreated SPC)	Ash (% wet weight)	6.570 +/- 0.398	5.450 +/- 0.139	3	3	Hedges' g	3.0	0.56 to 5.45	Higher, more favorable value with phytase; unadjusted 95% CI excluded 0	Not applicable - effect size estimated after converting SEM to SD
Carter and Sajjadi (2011)	Atlantic salmon (Salmo salar)	Direct phytase supplementation or phytase pre-treatment of soy protein concentrate in a low-fishmeal diet	Direct phytase 1000 U/kg diet	Control (0 U phytase/kg; untreated SPC)	Whole-body phosphorus (% wet weight)	1.370 +/- 0.010	1.230 +/- 0.019	3	3	Hedges' g	7.3	2.08 to 12.52	Higher, more favorable value with phytase; unadjusted 95% CI excluded 0	Not applicable - effect size estimated after converting SEM to SD
Carter and Sajjadi (2011)	Atlantic salmon (Salmo salar)	Direct phytase supplementation or phytase pre-treatment of soy protein concentrate in a low-fishmeal diet	Direct phytase 1000 U/kg diet	Control (0 U phytase/kg; untreated SPC)	Bone ash (% bone weight)	51.26 +/- 0.21	44.58 +/- 0.24	3	3	Hedges' g	23.66	7.22 to 40.11	Higher, more favorable value with phytase; unadjusted 95% CI excluded 0	Not applicable - effect size estimated after converting SEM to SD
Carter and Sajjadi (2011)	Atlantic salmon (Salmo salar)	Direct phytase supplementation or phytase pre-treatment of soy protein concentrate in a low-fishmeal diet	Direct phytase 1000 U/kg diet	Control (0 U phytase/kg; untreated SPC)	Bone phosphorus (% bone weight)	11.21 +/- 0.07	10.54 +/- 0.31	3	3	Hedges' g	2.37	0.29 to 4.46	Higher, more favorable value with phytase; unadjusted 95% CI excluded 0	Not applicable - effect size estimated after converting SEM to SD
Carter and Sajjadi (2011)	Atlantic salmon (Salmo salar)	Direct phytase supplementation or phytase pre-treatment of soy protein concentrate in a low-fishmeal diet	Direct phytase 1000 U/kg diet	Control (0 U phytase/kg; untreated SPC)	Apparent digestibility of calcium (%)	1.210 +/- 9.613	-15.51 +/- 10.12	3	3	Hedges' g	1.36	-0.23 to 2.94	Higher, more favorable value with phytase; unadjusted 95% CI included 0	Not applicable - effect size estimated after converting SEM to SD
Carter and Sajjadi (2011)	Atlantic salmon (Salmo salar)	Direct phytase supplementation or phytase pre-treatment of soy protein concentrate in a low-fishmeal diet	Direct phytase 1000 U/kg diet	Control (0 U phytase/kg; untreated SPC)	Apparent digestibility of magnesium (%)	59.99 +/- 1.89	53.56 +/- 5.01	3	3	Hedges' g	1.36	-0.23 to 2.95	Higher, more favorable value with phytase; unadjusted 95% CI included 0	Not applicable - effect size estimated after converting SEM to SD
Carter and Sajjadi (2011)	Atlantic salmon (Salmo salar)	Direct phytase supplementation or phytase pre-treatment of soy protein concentrate in a low-fishmeal diet	Direct phytase 1000 U/kg diet	Control (0 U phytase/kg; untreated SPC)	Apparent digestibility of copper (%)	37.44 +/- 36.44	55.07 +/- 1.35	3	3	Hedges' g	-0.55	-1.88 to 0.79	Lower, less favorable value with phytase; unadjusted 95% CI included 0	Not applicable - effect size estimated after converting SEM to SD
Carter and Sajjadi (2011)	Atlantic salmon (Salmo salar)	Direct phytase supplementation or phytase pre-treatment of soy protein concentrate in a low-fishmeal diet	Direct phytase 1000 U/kg diet	Control (0 U phytase/kg; untreated SPC)	Apparent digestibility of manganese (%)	21.71 +/- 3.53	18.34 +/- 8.61	3	3	Hedges' g	0.41	-0.90 to 1.72	Higher, more favorable value with phytase; unadjusted 95% CI included 0	Not applicable - effect size estimated after converting SEM to SD
Carter and Sajjadi (2011)	Atlantic salmon (Salmo salar)	Direct phytase supplementation or phytase pre-treatment of soy protein concentrate in a low-fishmeal diet	Direct phytase 1000 U/kg diet	Control (0 U phytase/kg; untreated SPC)	Apparent digestibility of strontium (%)	10.07 +/- 4.83	7.930 +/- 8.245	3	3	Hedges' g	0.25	-1.04 to 1.55	Higher, more favorable value with phytase; unadjusted 95% CI included 0	Not applicable - effect size estimated after converting SEM to SD
Carter and Sajjadi (2011)	Atlantic salmon (Salmo salar)	Direct phytase supplementation or phytase pre-treatment of soy protein concentrate in a low-fishmeal diet	Direct phytase 1000 U/kg diet	Control (0 U phytase/kg; untreated SPC)	Apparent digestibility of zinc (%)	75.23 +/- 0.59	58.18 +/- 4.14	3	3	Hedges' g	4.61	1.17 to 8.06	Higher, more favorable value with phytase; unadjusted 95% CI excluded 0	Not applicable - effect size estimated after converting SEM to SD
Carter and Sajjadi (2011)	Atlantic salmon (Salmo salar)	Direct phytase supplementation or phytase pre-treatment of soy protein concentrate in a low-fishmeal diet	Direct phytase 4000 U/kg diet	Control (0 U phytase/kg; untreated SPC)	Final body weight (g)	93.49 +/- 1.61	82.35 +/- 1.42	3	3	Hedges' g	5.87	1.61 to 10.13	Higher, more favorable value with phytase; unadjusted 95% CI excluded 0	Not applicable - effect size estimated after converting SEM to SD
Carter and Sajjadi (2011)	Atlantic salmon (Salmo salar)	Direct phytase supplementation or phytase pre-treatment of soy protein concentrate in a low-fishmeal diet	Direct phytase 4000 U/kg diet	Control (0 U phytase/kg; untreated SPC)	Weight gain (g)	47.89 +/- 1.49	36.77 +/- 2.75	3	3	Hedges' g	4.02	0.95 to 7.08	Higher, more favorable value with phytase; unadjusted 95% CI excluded 0	Not applicable - effect size estimated after converting SEM to SD
Carter and Sajjadi (2011)	Atlantic salmon (Salmo salar)	Direct phytase supplementation or phytase pre-treatment of soy protein concentrate in a low-fishmeal diet	Direct phytase 4000 U/kg diet	Control (0 U phytase/kg; untreated SPC)	Total feed intake (kg dry matter)	1.180 +/- 0.005	1.000 +/- 0.016	3	3	Hedges' g	12.39	3.71 to 21.08	Higher measured value with phytase; unadjusted 95% CI excluded 0	Not applicable - effect size estimated after converting SEM to SD
Carter and Sajjadi (2011)	Atlantic salmon (Salmo salar)	Direct phytase supplementation or phytase pre-treatment of soy protein concentrate in a low-fishmeal diet	Direct phytase 4000 U/kg diet	Control (0 U phytase/kg; untreated SPC)	Feed efficiency ratio (g gain/g dry feed)	0.810 +/- 0.028	0.740 +/- 0.045	3	3	Hedges' g	1.5	-0.15 to 3.15	Higher, more favorable value with phytase; unadjusted 95% CI included 0	Not applicable - effect size estimated after converting SEM to SD
Carter and Sajjadi (2011)	Atlantic salmon (Salmo salar)	Direct phytase supplementation or phytase pre-treatment of soy protein concentrate in a low-fishmeal diet	Direct phytase 4000 U/kg diet	Control (0 U phytase/kg; untreated SPC)	Dry matter (% wet weight)	33.68 +/- 0.12	33.81 +/- 0.43	3	3	Hedges' g	-0.33	-1.63 to 0.97	Lower measured value with phytase; unadjusted 95% CI included 0	Not applicable - effect size estimated after converting SEM to SD
Carter and Sajjadi (2011)	Atlantic salmon (Salmo salar)	Direct phytase supplementation or phytase pre-treatment of soy protein concentrate in a low-fishmeal diet	Direct phytase 4000 U/kg diet	Control (0 U phytase/kg; untreated SPC)	Crude protein (% wet weight)	17.34 +/- 0.33	17.21 +/- 0.33	3	3	Hedges' g	0.32	-0.98 to 1.61	Higher measured value with phytase; unadjusted 95% CI included 0	Not applicable - effect size estimated after converting SEM to SD

Study	Species	Strategy	Intervention	Comparator	Outcome and unit	Intervention mean +/- SD	Comparator mean +/- SD	n intervention (tanks)	n comparator (tanks)	Effect-size metric	Effect size	95% CI	Interpretation	Reason not estimated
Carter and Sajjadi (2011)	Atlantic salmon (Salmo salar)	Direct phytase supplementation or phytase pre-treatment of soy protein concentrate in a low-fishmeal diet	Direct phytase 4000 U/kg diet	Control (0 U phytase/kg; untreated SPC)	Total lipid (% wet weight)	10.44 +/- 0.36	10.60 +/- 0.21	3	3	Hedges' g	-0.43	-1.75 to 0.88	Lower measured value with phytase; unadjusted 95% CI included 0	Not applicable - effect size estimated after converting SEM to SD
Carter and Sajjadi (2011)	Atlantic salmon (Salmo salar)	Direct phytase supplementation or phytase pre-treatment of soy protein concentrate in a low-fishmeal diet	Direct phytase 4000 U/kg diet	Control (0 U phytase/kg; untreated SPC)	Ash (% wet weight)	6.820 +/- 0.502	5.450 +/- 0.139	3	3	Hedges' g	2.97	0.55 to 5.40	Higher, more favorable value with phytase; unadjusted 95% CI excluded 0	Not applicable - effect size estimated after converting SEM to SD
Carter and Sajjadi (2011)	Atlantic salmon (Salmo salar)	Direct phytase supplementation or phytase pre-treatment of soy protein concentrate in a low-fishmeal diet	Direct phytase 4000 U/kg diet	Control (0 U phytase/kg; untreated SPC)	Whole-body phosphorus (% wet weight)	1.430 +/- 0.024	1.230 +/- 0.019	3	3	Hedges' g	7.34	2.09 to 12.58	Higher, more favorable value with phytase; unadjusted 95% CI excluded 0	Not applicable - effect size estimated after converting SEM to SD
Carter and Sajjadi (2011)	Atlantic salmon (Salmo salar)	Direct phytase supplementation or phytase pre-treatment of soy protein concentrate in a low-fishmeal diet	Direct phytase 4000 U/kg diet	Control (0 U phytase/kg; untreated SPC)	Bone ash (% bone weight)	52.13 +/- 0.74	44.58 +/- 0.24	3	3	Hedges' g	10.91	3.24 to 18.57	Higher, more favorable value with phytase; unadjusted 95% CI excluded 0	Not applicable - effect size estimated after converting SEM to SD
Carter and Sajjadi (2011)	Atlantic salmon (Salmo salar)	Direct phytase supplementation or phytase pre-treatment of soy protein concentrate in a low-fishmeal diet	Direct phytase 4000 U/kg diet	Control (0 U phytase/kg; untreated SPC)	Bone phosphorus (% bone weight)	11.68 +/- 0.14	10.54 +/- 0.31	3	3	Hedges' g	3.78	0.86 to 6.70	Higher, more favorable value with phytase; unadjusted 95% CI excluded 0	Not applicable - effect size estimated after converting SEM to SD
Carter and Sajjadi (2011)	Atlantic salmon (Salmo salar)	Direct phytase supplementation or phytase pre-treatment of soy protein concentrate in a low-fishmeal diet	Direct phytase 4000 U/kg diet	Control (0 U phytase/kg; untreated SPC)	Apparent digestibility of calcium (%)	11.90 +/- 1.32	-15.51 +/- 10.12	3	3	Hedges' g	3.04	0.57 to 5.51	Higher, more favorable value with phytase; unadjusted 95% CI excluded 0	Not applicable - effect size estimated after converting SEM to SD
Carter and Sajjadi (2011)	Atlantic salmon (Salmo salar)	Direct phytase supplementation or phytase pre-treatment of soy protein concentrate in a low-fishmeal diet	Direct phytase 4000 U/kg diet	Control (0 U phytase/kg; untreated SPC)	Apparent digestibility of magnesium (%)	68.82 +/- 3.97	53.56 +/- 5.01	3	3	Hedges' g	2.7	0.43 to 4.97	Higher, more favorable value with phytase; unadjusted 95% CI excluded 0	Not applicable - effect size estimated after converting SEM to SD
Carter and Sajjadi (2011)	Atlantic salmon (Salmo salar)	Direct phytase supplementation or phytase pre-treatment of soy protein concentrate in a low-fishmeal diet	Direct phytase 4000 U/kg diet	Control (0 U phytase/kg; untreated SPC)	Apparent digestibility of copper (%)	47.71 +/- 8.73	55.07 +/- 1.35	3	3	Hedges' g	-0.94	-2.38 to 0.49	Lower, less favorable value with phytase; unadjusted 95% CI included 0	Not applicable - effect size estimated after converting SEM to SD
Carter and Sajjadi (2011)	Atlantic salmon (Salmo salar)	Direct phytase supplementation or phytase pre-treatment of soy protein concentrate in a low-fishmeal diet	Direct phytase 4000 U/kg diet	Control (0 U phytase/kg; untreated SPC)	Apparent digestibility of manganese (%)	31.88 +/- 9.47	18.34 +/- 8.61	3	3	Hedges' g	1.2	-0.33 to 2.72	Higher, more favorable value with phytase; unadjusted 95% CI included 0	Not applicable - effect size estimated after converting SEM to SD
Carter and Sajjadi (2011)	Atlantic salmon (Salmo salar)	Direct phytase supplementation or phytase pre-treatment of soy protein concentrate in a low-fishmeal diet	Direct phytase 4000 U/kg diet	Control (0 U phytase/kg; untreated SPC)	Apparent digestibility of strontium (%)	18.43 +/- 7.57	7.930 +/- 8.245	3	3	Hedges' g	1.06	-0.42 to 2.54	Higher, more favorable value with phytase; unadjusted 95% CI included 0	Not applicable - effect size estimated after converting SEM to SD
Carter and Sajjadi (2011)	Atlantic salmon (Salmo salar)	Direct phytase supplementation or phytase pre-treatment of soy protein concentrate in a low-fishmeal diet	Direct phytase 4000 U/kg diet	Control (0 U phytase/kg; untreated SPC)	Apparent digestibility of zinc (%)	80.82 +/- 4.16	58.18 +/- 4.14	3	3	Hedges' g	4.37	1.08 to 7.65	Higher, more favorable value with phytase; unadjusted 95% CI excluded 0	Not applicable - effect size estimated after converting SEM to SD
Carter and Sajjadi (2011)	Atlantic salmon (Salmo salar)	Direct phytase supplementation or phytase pre-treatment of soy protein concentrate in a low-fishmeal diet	Pre-treated SPC, equivalent to 250 U/kg diet (417 U/kg SPC)	Control (0 U phytase/kg; untreated SPC)	Final body weight (g)	94.62 +/- 1.65	82.35 +/- 1.42	3	3	Hedges' g	6.39	1.78 to 10.99	Higher, more favorable value with phytase; unadjusted 95% CI excluded 0	Not applicable - effect size estimated after converting SEM to SD
Carter and Sajjadi (2011)	Atlantic salmon (Salmo salar)	Direct phytase supplementation or phytase pre-treatment of soy protein concentrate in a low-fishmeal diet	Pre-treated SPC, equivalent to 250 U/kg diet (417 U/kg SPC)	Control (0 U phytase/kg; untreated SPC)	Weight gain (g)	49.26 +/- 2.62	36.77 +/- 2.75	3	3	Hedges' g	3.72	0.84 to 6.60	Higher, more favorable value with phytase; unadjusted 95% CI excluded 0	Not applicable - effect size estimated after converting SEM to SD
Carter and Sajjadi (2011)	Atlantic salmon (Salmo salar)	Direct phytase supplementation or phytase pre-treatment of soy protein concentrate in a low-fishmeal diet	Pre-treated SPC, equivalent to 250 U/kg diet (417 U/kg SPC)	Control (0 U phytase/kg; untreated SPC)	Total feed intake (kg dry matter)	1.230 +/- 0.087	1.000 +/- 0.016	3	3	Hedges' g	2.96	0.54 to 5.37	Higher measured value with phytase; unadjusted 95% CI excluded 0	Not applicable - effect size estimated after converting SEM to SD
Carter and Sajjadi (2011)	Atlantic salmon (Salmo salar)	Direct phytase supplementation or phytase pre-treatment of soy protein concentrate in a low-fishmeal diet	Pre-treated SPC, equivalent to 250 U/kg diet (417 U/kg SPC)	Control (0 U phytase/kg; untreated SPC)	Feed efficiency ratio (g gain/g dry feed)	0.800 +/- 0.019	0.740 +/- 0.045	3	3	Hedges' g	1.39	-0.21 to 2.99	Higher, more favorable value with phytase; unadjusted 95% CI included 0	Not applicable - effect size estimated after converting SEM to SD
Carter and Sajjadi (2011)	Atlantic salmon (Salmo salar)	Direct phytase supplementation or phytase pre-treatment of soy protein concentrate in a low-fishmeal diet	Pre-treated SPC, equivalent to 250 U/kg diet (417 U/kg SPC)	Control (0 U phytase/kg; untreated SPC)	Dry matter (% wet weight)	33.67 +/- 0.40	33.81 +/- 0.43	3	3	Hedges' g	-0.27	-1.56 to 1.02	Lower measured value with phytase; unadjusted 95% CI included 0	Not applicable - effect size estimated after converting SEM to SD
Carter and Sajjadi (2011)	Atlantic salmon (Salmo salar)	Direct phytase supplementation or phytase pre-treatment of soy protein concentrate in a low-fishmeal diet	Pre-treated SPC, equivalent to 250 U/kg diet (417 U/kg SPC)	Control (0 U phytase/kg; untreated SPC)	Crude protein (% wet weight)	17.19 +/- 0.12	17.21 +/- 0.33	3	3	Hedges' g	-0.06	-1.35 to 1.22	Lower measured value with phytase; unadjusted 95% CI included 0	Not applicable - effect size estimated after converting SEM to SD
Carter and Sajjadi (2011)	Atlantic salmon (Salmo salar)	Direct phytase supplementation or phytase pre-treatment of soy protein concentrate in a low-fishmeal diet	Pre-treated SPC, equivalent to 250 U/kg diet (417 U/kg SPC)	Control (0 U phytase/kg; untreated SPC)	Total lipid (% wet weight)	10.56 +/- 0.33	10.60 +/- 0.21	3	3	Hedges' g	-0.12	-1.40 to 1.17	Lower measured value with phytase; unadjusted 95% CI included 0	Not applicable - effect size estimated after converting SEM to SD
Carter and Sajjadi (2011)	Atlantic salmon (Salmo salar)	Direct phytase supplementation or phytase pre-treatment of soy protein concentrate in a low-fishmeal diet	Pre-treated SPC, equivalent to 250 U/kg diet (417 U/kg SPC)	Control (0 U phytase/kg; untreated SPC)	Ash (% wet weight)	6.600 +/- 0.121	5.450 +/- 0.139	3	3	Hedges' g	7.07	2.01 to 12.13	Higher, more favorable value with phytase; unadjusted 95% CI excluded 0	Not applicable - effect size estimated after converting SEM to SD

Study	Species	Strategy	Intervention	Comparator	Outcome and unit	Intervention mean +/- SD	Comparator mean +/- SD	n intervention (tanks)	n comparator (tanks)	Effect-size metric	Effect size	95% CI	Interpretation	Reason not estimated
Carter and Sajjadi (2011)	Atlantic salmon (Salmo salar)	Direct phytase supplementation or phytase pre-treatment of soy protein concentrate in a low-fishmeal diet	Pre-treated SPC, equivalent to 250 U/kg diet (417 U/kg SPC)	Control (0 U phytase/kg; untreated SPC)	Whole-body phosphorus (% wet weight)	1.390 +/- 0.019	1.230 +/- 0.019	3	3	Hedges' g	6.72	1.89 to 11.55	Higher, more favorable value with phytase; unadjusted 95% CI excluded 0	Not applicable - effect size estimated after converting SEM to SD
Carter and Sajjadi (2011)	Atlantic salmon (Salmo salar)	Direct phytase supplementation or phytase pre-treatment of soy protein concentrate in a low-fishmeal diet	Pre-treated SPC, equivalent to 250 U/kg diet (417 U/kg SPC)	Control (0 U phytase/kg; untreated SPC)	Bone ash (% bone weight)	51.93 +/- 0.24	44.58 +/- 0.24	3	3	Hedges' g	24.25	7.40 to 41.10	Higher, more favorable value with phytase; unadjusted 95% CI excluded 0	Not applicable - effect size estimated after converting SEM to SD
Carter and Sajjadi (2011)	Atlantic salmon (Salmo salar)	Direct phytase supplementation or phytase pre-treatment of soy protein concentrate in a low-fishmeal diet	Pre-treated SPC, equivalent to 250 U/kg diet (417 U/kg SPC)	Control (0 U phytase/kg; untreated SPC)	Bone phosphorus (% bone weight)	11.45 +/- 0.09	10.54 +/- 0.31	3	3	Hedges' g	3.18	0.63 to 5.73	Higher, more favorable value with phytase; unadjusted 95% CI excluded 0	Not applicable - effect size estimated after converting SEM to SD
Carter and Sajjadi (2011)	Atlantic salmon (Salmo salar)	Direct phytase supplementation or phytase pre-treatment of soy protein concentrate in a low-fishmeal diet	Pre-treated SPC, equivalent to 250 U/kg diet (417 U/kg SPC)	Control (0 U phytase/kg; untreated SPC)	Apparent digestibility of calcium (%)	13.75 +/- 8.71	-15.51 +/- 10.12	3	3	Hedges' g	2.48	0.34 to 4.62	Higher, more favorable value with phytase; unadjusted 95% CI excluded 0	Not applicable - effect size estimated after converting SEM to SD
Carter and Sajjadi (2011)	Atlantic salmon (Salmo salar)	Direct phytase supplementation or phytase pre-treatment of soy protein concentrate in a low-fishmeal diet	Pre-treated SPC, equivalent to 250 U/kg diet (417 U/kg SPC)	Control (0 U phytase/kg; untreated SPC)	Apparent digestibility of magnesium (%)	64.21 +/- 4.33	53.56 +/- 5.01	3	3	Hedges' g	1.82	0.02 to 3.62	Higher, more favorable value with phytase; unadjusted 95% CI excluded 0	Not applicable - effect size estimated after converting SEM to SD
Carter and Sajjadi (2011)	Atlantic salmon (Salmo salar)	Direct phytase supplementation or phytase pre-treatment of soy protein concentrate in a low-fishmeal diet	Pre-treated SPC, equivalent to 250 U/kg diet (417 U/kg SPC)	Control (0 U phytase/kg; untreated SPC)	Apparent digestibility of copper (%)	51.00 +/- 3.83	55.07 +/- 1.35	3	3	Hedges' g	-1.13	-2.64 to 0.37	Lower, less favorable value with phytase; unadjusted 95% CI included 0	Not applicable - effect size estimated after converting SEM to SD
Carter and Sajjadi (2011)	Atlantic salmon (Salmo salar)	Direct phytase supplementation or phytase pre-treatment of soy protein concentrate in a low-fishmeal diet	Pre-treated SPC, equivalent to 250 U/kg diet (417 U/kg SPC)	Control (0 U phytase/kg; untreated SPC)	Apparent digestibility of manganese (%)	20.46 +/- 5.44	18.34 +/- 8.61	3	3	Hedges' g	0.24	-1.06 to 1.53	Higher, more favorable value with phytase; unadjusted 95% CI included 0	Not applicable - effect size estimated after converting SEM to SD
Carter and Sajjadi (2011)	Atlantic salmon (Salmo salar)	Direct phytase supplementation or phytase pre-treatment of soy protein concentrate in a low-fishmeal diet	Pre-treated SPC, equivalent to 250 U/kg diet (417 U/kg SPC)	Control (0 U phytase/kg; untreated SPC)	Apparent digestibility of strontium (%)	11.76 +/- 3.76	7.930 +/- 8.245	3	3	Hedges' g	0.48	-0.84 to 1.80	Higher, more favorable value with phytase; unadjusted 95% CI included 0	Not applicable - effect size estimated after converting SEM to SD
Carter and Sajjadi (2011)	Atlantic salmon (Salmo salar)	Direct phytase supplementation or phytase pre-treatment of soy protein concentrate in a low-fishmeal diet	Pre-treated SPC, equivalent to 250 U/kg diet (417 U/kg SPC)	Control (0 U phytase/kg; untreated SPC)	Apparent digestibility of zinc (%)	81.29 +/- 1.35	58.18 +/- 4.14	3	3	Hedges' g	6.0	1.65 to 10.36	Higher, more favorable value with phytase; unadjusted 95% CI excluded 0	Not applicable - effect size estimated after converting SEM to SD
Carter and Sajjadi (2011)	Atlantic salmon (Salmo salar)	Direct phytase supplementation or phytase pre-treatment of soy protein concentrate in a low-fishmeal diet	Pre-treated SPC, equivalent to 500 U/kg diet (833 U/kg SPC)	Control (0 U phytase/kg; untreated SPC)	Final body weight (g)	94.74 +/- 1.65	82.35 +/- 1.42	3	3	Hedges' g	6.45	1.80 to 11.10	Higher, more favorable value with phytase; unadjusted 95% CI excluded 0	Not applicable - effect size estimated after converting SEM to SD
Carter and Sajjadi (2011)	Atlantic salmon (Salmo salar)	Direct phytase supplementation or phytase pre-treatment of soy protein concentrate in a low-fishmeal diet	Pre-treated SPC, equivalent to 500 U/kg diet (833 U/kg SPC)	Control (0 U phytase/kg; untreated SPC)	Weight gain (g)	49.21 +/- 2.10	36.77 +/- 2.75	3	3	Hedges' g	4.07	0.97 to 7.16	Higher, more favorable value with phytase; unadjusted 95% CI excluded 0	Not applicable - effect size estimated after converting SEM to SD
Carter and Sajjadi (2011)	Atlantic salmon (Salmo salar)	Direct phytase supplementation or phytase pre-treatment of soy protein concentrate in a low-fishmeal diet	Pre-treated SPC, equivalent to 500 U/kg diet (833 U/kg SPC)	Control (0 U phytase/kg; untreated SPC)	Total feed intake (kg dry matter)	1.210 +/- 0.052	1.000 +/- 0.016	3	3	Hedges' g	4.38	1.09 to 7.67	Higher measured value with phytase; unadjusted 95% CI excluded 0	Not applicable - effect size estimated after converting SEM to SD
Carter and Sajjadi (2011)	Atlantic salmon (Salmo salar)	Direct phytase supplementation or phytase pre-treatment of soy protein concentrate in a low-fishmeal diet	Pre-treated SPC, equivalent to 500 U/kg diet (833 U/kg SPC)	Control (0 U phytase/kg; untreated SPC)	Feed efficiency ratio (g gain/g dry feed)	0.810 +/- 0.019	0.740 +/- 0.045	3	3	Hedges' g	1.62	-0.08 to 3.32	Higher, more favorable value with phytase; unadjusted 95% CI included 0	Not applicable - effect size estimated after converting SEM to SD
Carter and Sajjadi (2011)	Atlantic salmon (Salmo salar)	Direct phytase supplementation or phytase pre-treatment of soy protein concentrate in a low-fishmeal diet	Pre-treated SPC, equivalent to 500 U/kg diet (833 U/kg SPC)	Control (0 U phytase/kg; untreated SPC)	Dry matter (% wet weight)	33.72 +/- 0.12	33.81 +/- 0.43	3	3	Hedges' g	-0.23	-1.52 to 1.06	Lower measured value with phytase; unadjusted 95% CI included 0	Not applicable - effect size estimated after converting SEM to SD
Carter and Sajjadi (2011)	Atlantic salmon (Salmo salar)	Direct phytase supplementation or phytase pre-treatment of soy protein concentrate in a low-fishmeal diet	Pre-treated SPC, equivalent to 500 U/kg diet (833 U/kg SPC)	Control (0 U phytase/kg; untreated SPC)	Crude protein (% wet weight)	16.96 +/- 0.05	17.21 +/- 0.33	3	3	Hedges' g	-0.85	-2.26 to 0.56	Lower measured value with phytase; unadjusted 95% CI included 0	Not applicable - effect size estimated after converting SEM to SD
Carter and Sajjadi (2011)	Atlantic salmon (Salmo salar)	Direct phytase supplementation or phytase pre-treatment of soy protein concentrate in a low-fishmeal diet	Pre-treated SPC, equivalent to 500 U/kg diet (833 U/kg SPC)	Control (0 U phytase/kg; untreated SPC)	Total lipid (% wet weight)	10.38 +/- 0.12	10.60 +/- 0.21	3	3	Hedges' g	-1.03	-2.50 to 0.43	Lower measured value with phytase; unadjusted 95% CI included 0	Not applicable - effect size estimated after converting SEM to SD
Carter and Sajjadi (2011)	Atlantic salmon (Salmo salar)	Direct phytase supplementation or phytase pre-treatment of soy protein concentrate in a low-fishmeal diet	Pre-treated SPC, equivalent to 500 U/kg diet (833 U/kg SPC)	Control (0 U phytase/kg; untreated SPC)	Ash (% wet weight)	6.680 +/- 0.225	5.450 +/- 0.139	3	3	Hedges' g	5.26	1.40 to 9.13	Higher, more favorable value with phytase; unadjusted 95% CI excluded 0	Not applicable - effect size estimated after converting SEM to SD
Carter and Sajjadi (2011)	Atlantic salmon (Salmo salar)	Direct phytase supplementation or phytase pre-treatment of soy protein concentrate in a low-fishmeal diet	Pre-treated SPC, equivalent to 500 U/kg diet (833 U/kg SPC)	Control (0 U phytase/kg; untreated SPC)	Whole-body phosphorus (% wet weight)	1.370 +/- 0.024	1.230 +/- 0.019	3	3	Hedges' g	5.14	1.35 to 8.92	Higher, more favorable value with phytase; unadjusted 95% CI excluded 0	Not applicable - effect size estimated after converting SEM to SD
Carter and Sajjadi (2011)	Atlantic salmon (Salmo salar)	Direct phytase supplementation or phytase pre-treatment of soy protein concentrate in a low-fishmeal diet	Pre-treated SPC, equivalent to 500 U/kg diet (833 U/kg SPC)	Control (0 U phytase/kg; untreated SPC)	Bone ash (% bone weight)	52.13 +/- 0.40	44.58 +/- 0.24	3	3	Hedges' g	18.32	5.56 to 31.07	Higher, more favorable value with phytase; unadjusted 95% CI excluded 0	Not applicable - effect size estimated after converting SEM to SD

Study	Species	Strategy	Intervention	Comparator	Outcome and unit	Intervention mean +/- SD	Comparator mean +/- SD	n intervention (tanks)	n comparator (tanks)	Effect-size metric	Effect size	95% CI	Interpretation	Reason not estimated
Carter and Sajjadi (2011)	Atlantic salmon (Salmo salar)	Direct phytase supplementation or phytase pre-treatment of soy protein concentrate in a low-fishmeal diet	Pre-treated SPC, equivalent to 500 U/kg diet (833 U/kg SPC)	Control (0 U phytase/kg; untreated SPC)	Bone phosphorus (% bone weight)	11.82 +/- 0.54	10.54 +/- 0.31	3	3	Hedges' g	2.33	0.27 to 4.39	Higher, more favorable value with phytase; unadjusted 95% CI excluded 0	Not applicable - effect size estimated after converting SEM to SD
Carter and Sajjadi (2011)	Atlantic salmon (Salmo salar)	Direct phytase supplementation or phytase pre-treatment of soy protein concentrate in a low-fishmeal diet	Pre-treated SPC, equivalent to 500 U/kg diet (833 U/kg SPC)	Control (0 U phytase/kg; untreated SPC)	Apparent digestibility of calcium (%)	-4.270 +/- 20.992	-15.51 +/- 10.12	3	3	Hedges' g	0.55	-0.79 to 1.88	Higher, more favorable value with phytase; unadjusted 95% CI included 0	Not applicable - effect size estimated after converting SEM to SD
Carter and Sajjadi (2011)	Atlantic salmon (Salmo salar)	Direct phytase supplementation or phytase pre-treatment of soy protein concentrate in a low-fishmeal diet	Pre-treated SPC, equivalent to 500 U/kg diet (833 U/kg SPC)	Control (0 U phytase/kg; untreated SPC)	Apparent digestibility of magnesium (%)	64.44 +/- 2.13	53.56 +/- 5.01	3	3	Hedges' g	2.26	0.24 to 4.29	Higher, more favorable value with phytase; unadjusted 95% CI excluded 0	Not applicable - effect size estimated after converting SEM to SD
Carter and Sajjadi (2011)	Atlantic salmon (Salmo salar)	Direct phytase supplementation or phytase pre-treatment of soy protein concentrate in a low-fishmeal diet	Pre-treated SPC, equivalent to 500 U/kg diet (833 U/kg SPC)	Control (0 U phytase/kg; untreated SPC)	Apparent digestibility of copper (%)	53.77 +/- 10.79	55.07 +/- 1.35	3	3	Hedges' g	-0.14	-1.42 to 1.15	Lower, less favorable value with phytase; unadjusted 95% CI included 0	Not applicable - effect size estimated after converting SEM to SD
Carter and Sajjadi (2011)	Atlantic salmon (Salmo salar)	Direct phytase supplementation or phytase pre-treatment of soy protein concentrate in a low-fishmeal diet	Pre-treated SPC, equivalent to 500 U/kg diet (833 U/kg SPC)	Control (0 U phytase/kg; untreated SPC)	Apparent digestibility of manganese (%)	16.44 +/- 11.14	18.34 +/- 8.61	3	3	Hedges' g	-0.15	-1.44 to 1.13	Lower, less favorable value with phytase; unadjusted 95% CI included 0	Not applicable - effect size estimated after converting SEM to SD
Carter and Sajjadi (2011)	Atlantic salmon (Salmo salar)	Direct phytase supplementation or phytase pre-treatment of soy protein concentrate in a low-fishmeal diet	Pre-treated SPC, equivalent to 500 U/kg diet (833 U/kg SPC)	Control (0 U phytase/kg; untreated SPC)	Apparent digestibility of strontium (%)	3.470 +/- 11.466	7.930 +/- 8.245	3	3	Hedges' g	-0.36	-1.66 to 0.95	Lower, less favorable value with phytase; unadjusted 95% CI included 0	Not applicable - effect size estimated after converting SEM to SD
Carter and Sajjadi (2011)	Atlantic salmon (Salmo salar)	Direct phytase supplementation or phytase pre-treatment of soy protein concentrate in a low-fishmeal diet	Pre-treated SPC, equivalent to 500 U/kg diet (833 U/kg SPC)	Control (0 U phytase/kg; untreated SPC)	Apparent digestibility of zinc (%)	77.25 +/- 2.29	58.18 +/- 4.14	3	3	Hedges' g	4.56	1.15 to 7.97	Higher, more favorable value with phytase; unadjusted 95% CI excluded 0	Not applicable - effect size estimated after converting SEM to SD
Carter and Sajjadi (2011)	Atlantic salmon (Salmo salar)	Direct phytase supplementation or phytase pre-treatment of soy protein concentrate in a low-fishmeal diet	Pre-treated SPC, equivalent to 1000 U/kg diet (1667 U/kg SPC)	Control (0 U phytase/kg; untreated SPC)	Final body weight (g)	94.46 +/- 1.63	82.35 +/- 1.42	3	3	Hedges' g	6.34	1.76 to 10.92	Higher, more favorable value with phytase; unadjusted 95% CI excluded 0	Not applicable - effect size estimated after converting SEM to SD
Carter and Sajjadi (2011)	Atlantic salmon (Salmo salar)	Direct phytase supplementation or phytase pre-treatment of soy protein concentrate in a low-fishmeal diet	Pre-treated SPC, equivalent to 1000 U/kg diet (1667 U/kg SPC)	Control (0 U phytase/kg; untreated SPC)	Weight gain (g)	48.93 +/- 3.24	36.77 +/- 2.75	3	3	Hedges' g	3.24	0.65 to 5.82	Higher, more favorable value with phytase; unadjusted 95% CI excluded 0	Not applicable - effect size estimated after converting SEM to SD
Carter and Sajjadi (2011)	Atlantic salmon (Salmo salar)	Direct phytase supplementation or phytase pre-treatment of soy protein concentrate in a low-fishmeal diet	Pre-treated SPC, equivalent to 1000 U/kg diet (1667 U/kg SPC)	Control (0 U phytase/kg; untreated SPC)	Total feed intake (kg dry matter)	1.240 +/- 0.139	1.000 +/- 0.016	3	3	Hedges' g	1.95	0.09 to 3.81	Higher measured value with phytase; unadjusted 95% CI excluded 0	Not applicable - effect size estimated after converting SEM to SD
Carter and Sajjadi (2011)	Atlantic salmon (Salmo salar)	Direct phytase supplementation or phytase pre-treatment of soy protein concentrate in a low-fishmeal diet	Pre-treated SPC, equivalent to 1000 U/kg diet (1667 U/kg SPC)	Control (0 U phytase/kg; untreated SPC)	Feed efficiency ratio (g gain/g dry feed)	0.790 +/- 0.074	0.740 +/- 0.045	3	3	Hedges' g	0.65	-0.71 to 2.01	Higher, more favorable value with phytase; unadjusted 95% CI included 0	Not applicable - effect size estimated after converting SEM to SD
Carter and Sajjadi (2011)	Atlantic salmon (Salmo salar)	Direct phytase supplementation or phytase pre-treatment of soy protein concentrate in a low-fishmeal diet	Pre-treated SPC, equivalent to 1000 U/kg diet (1667 U/kg SPC)	Control (0 U phytase/kg; untreated SPC)	Dry matter (% wet weight)	33.52 +/- 0.23	33.81 +/- 0.43	3	3	Hedges' g	-0.67	-2.03 to 0.69	Lower measured value with phytase; unadjusted 95% CI included 0	Not applicable - effect size estimated after converting SEM to SD
Carter and Sajjadi (2011)	Atlantic salmon (Salmo salar)	Direct phytase supplementation or phytase pre-treatment of soy protein concentrate in a low-fishmeal diet	Pre-treated SPC, equivalent to 1000 U/kg diet (1667 U/kg SPC)	Control (0 U phytase/kg; untreated SPC)	Crude protein (% wet weight)	17.20 +/- 0.31	17.21 +/- 0.33	3	3	Hedges' g	-0.02	-1.31 to 1.26	Lower measured value with phytase; unadjusted 95% CI included 0	Not applicable - effect size estimated after converting SEM to SD
Carter and Sajjadi (2011)	Atlantic salmon (Salmo salar)	Direct phytase supplementation or phytase pre-treatment of soy protein concentrate in a low-fishmeal diet	Pre-treated SPC, equivalent to 1000 U/kg diet (1667 U/kg SPC)	Control (0 U phytase/kg; untreated SPC)	Total lipid (% wet weight)	10.69 +/- 0.21	10.60 +/- 0.21	3	3	Hedges' g	0.35	-0.96 to 1.65	Higher measured value with phytase; unadjusted 95% CI included 0	Not applicable - effect size estimated after converting SEM to SD
Carter and Sajjadi (2011)	Atlantic salmon (Salmo salar)	Direct phytase supplementation or phytase pre-treatment of soy protein concentrate in a low-fishmeal diet	Pre-treated SPC, equivalent to 1000 U/kg diet (1667 U/kg SPC)	Control (0 U phytase/kg; untreated SPC)	Ash (% wet weight)	6.850 +/- 0.398	5.450 +/- 0.139	3	3	Hedges' g	3.76	0.86 to 6.66	Higher, more favorable value with phytase; unadjusted 95% CI excluded 0	Not applicable - effect size estimated after converting SEM to SD
Carter and Sajjadi (2011)	Atlantic salmon (Salmo salar)	Direct phytase supplementation or phytase pre-treatment of soy protein concentrate in a low-fishmeal diet	Pre-treated SPC, equivalent to 1000 U/kg diet (1667 U/kg SPC)	Control (0 U phytase/kg; untreated SPC)	Whole-body phosphorus (% wet weight)	1.430 +/- 0.014	1.230 +/- 0.019	3	3	Hedges' g	9.6	2.83 to 16.38	Higher, more favorable value with phytase; unadjusted 95% CI excluded 0	Not applicable - effect size estimated after converting SEM to SD
Carter and Sajjadi (2011)	Atlantic salmon (Salmo salar)	Direct phytase supplementation or phytase pre-treatment of soy protein concentrate in a low-fishmeal diet	Pre-treated SPC, equivalent to 1000 U/kg diet (1667 U/kg SPC)	Control (0 U phytase/kg; untreated SPC)	Bone ash (% bone weight)	51.57 +/- 1.40	44.58 +/- 0.24	3	3	Hedges' g	5.55	1.50 to 9.61	Higher, more favorable value with phytase; unadjusted 95% CI excluded 0	Not applicable - effect size estimated after converting SEM to SD
Carter and Sajjadi (2011)	Atlantic salmon (Salmo salar)	Direct phytase supplementation or phytase pre-treatment of soy protein concentrate in a low-fishmeal diet	Pre-treated SPC, equivalent to 1000 U/kg diet (1667 U/kg SPC)	Control (0 U phytase/kg; untreated SPC)	Bone phosphorus (% bone weight)	11.52 +/- 0.07	10.54 +/- 0.31	3	3	Hedges' g	3.47	0.75 to 6.20	Higher, more favorable value with phytase; unadjusted 95% CI excluded 0	Not applicable - effect size estimated after converting SEM to SD
Carter and Sajjadi (2011)	Atlantic salmon (Salmo salar)	Direct phytase supplementation or phytase pre-treatment of soy protein concentrate in a low-fishmeal diet	Pre-treated SPC, equivalent to 1000 U/kg diet (1667 U/kg SPC)	Control (0 U phytase/kg; untreated SPC)	Apparent digestibility of calcium (%)	2.630 +/- 6.616	-15.51 +/- 10.12	3	3	Hedges' g	1.7	-0.04 to 3.44	Higher, more favorable value with phytase; unadjusted 95% CI included 0	Not applicable - effect size estimated after converting SEM to SD

Study	Species	Strategy	Intervention	Comparator	Outcome and unit	Intervention mean +/- SD	Comparator mean +/- SD	n intervention (tanks)	n comparator (tanks)	Effect-size metric	Effect size	95% CI	Interpretation	Reason not estimated
Carter and Sajjadi (2011)	Atlantic salmon (Salmo salar)	Direct phytase supplementation or phytase pre-treatment of soy protein concentrate in a low-fishmeal diet	Pre-treated SPC, equivalent to 1000 U/kg diet (1667 U/kg SPC)	Control (0 U phytase/kg; untreated SPC)	Apparent digestibility of magnesium (%)	67.86 +/- 1.09	53.56 +/- 5.01	3	3	Hedges' g	3.16	0.62 to 5.69	Higher, more favorable value with phytase; unadjusted 95% CI excluded 0	Not applicable - effect size estimated after converting SEM to SD
Carter and Sajjadi (2011)	Atlantic salmon (Salmo salar)	Direct phytase supplementation or phytase pre-treatment of soy protein concentrate in a low-fishmeal diet	Pre-treated SPC, equivalent to 1000 U/kg diet (1667 U/kg SPC)	Control (0 U phytase/kg; untreated SPC)	Apparent digestibility of copper (%)	51.07 +/- 2.89	55.07 +/- 1.35	3	3	Hedges' g	-1.42	-3.03 to 0.20	Lower, less favorable value with phytase; unadjusted 95% CI included 0	Not applicable - effect size estimated after converting SEM to SD
Carter and Sajjadi (2011)	Atlantic salmon (Salmo salar)	Direct phytase supplementation or phytase pre-treatment of soy protein concentrate in a low-fishmeal diet	Pre-treated SPC, equivalent to 1000 U/kg diet (1667 U/kg SPC)	Control (0 U phytase/kg; untreated SPC)	Apparent digestibility of manganese (%)	32.22 +/- 11.54	18.34 +/- 8.61	3	3	Hedges' g	1.09	-0.40 to 2.58	Higher, more favorable value with phytase; unadjusted 95% CI included 0	Not applicable - effect size estimated after converting SEM to SD
Carter and Sajjadi (2011)	Atlantic salmon (Salmo salar)	Direct phytase supplementation or phytase pre-treatment of soy protein concentrate in a low-fishmeal diet	Pre-treated SPC, equivalent to 1000 U/kg diet (1667 U/kg SPC)	Control (0 U phytase/kg; untreated SPC)	Apparent digestibility of strontium (%)	11.65 +/- 1.25	7.930 +/- 8.245	3	3	Hedges' g	0.5	-0.82 to 1.83	Higher, more favorable value with phytase; unadjusted 95% CI included 0	Not applicable - effect size estimated after converting SEM to SD
Carter and Sajjadi (2011)	Atlantic salmon (Salmo salar)	Direct phytase supplementation or phytase pre-treatment of soy protein concentrate in a low-fishmeal diet	Pre-treated SPC, equivalent to 1000 U/kg diet (1667 U/kg SPC)	Control (0 U phytase/kg; untreated SPC)	Apparent digestibility of zinc (%)	81.29 +/- 0.48	58.18 +/- 4.14	3	3	Hedges' g	6.27	1.74 to 10.80	Higher, more favorable value with phytase; unadjusted 95% CI excluded 0	Not applicable - effect size estimated after converting SEM to SD
Carter and Sajjadi (2011)	Atlantic salmon (Salmo salar)	Direct phytase supplementation or phytase pre-treatment of soy protein concentrate in a low-fishmeal diet	Direct phytase 250 U/kg diet	Control (0 U phytase/kg; untreated SPC)	Apparent digestibility of phosphorus (%)	55.70 +/- 3.14	45.43 +/- 3.57	3	3	Hedges' g	2.45	0.32 to 4.57	Higher, more favorable value with phytase; unadjusted 95% CI excluded 0	Not applicable - exact mean +/- SEM values were reported in the abstract
Carter and Sajjadi (2011)	Atlantic salmon (Salmo salar)	Direct phytase supplementation or phytase pre-treatment of soy protein concentrate in a low-fishmeal diet	Direct phytase 500 U/kg diet	Control (0 U phytase/kg; untreated SPC)	Apparent digestibility of phosphorus (%)	Not reported numerically	45.43 +/- 3.57	3	3	Not estimable	Not estimable	Not estimable	Figure 3 indicates higher phosphorus digestibility, but an exact standardized effect was not calculated	Exact mean and SEM for this intervention were not reported numerically
Carter and Sajjadi (2011)	Atlantic salmon (Salmo salar)	Direct phytase supplementation or phytase pre-treatment of soy protein concentrate in a low-fishmeal diet	Direct phytase 1000 U/kg diet	Control (0 U phytase/kg; untreated SPC)	Apparent digestibility of phosphorus (%)	Not reported numerically	45.43 +/- 3.57	3	3	Not estimable	Not estimable	Not estimable	Figure 3 indicates higher phosphorus digestibility, but an exact standardized effect was not calculated	Exact mean and SEM for this intervention were not reported numerically
Carter and Sajjadi (2011)	Atlantic salmon (Salmo salar)	Direct phytase supplementation or phytase pre-treatment of soy protein concentrate in a low-fishmeal diet	Direct phytase 4000 U/kg diet	Control (0 U phytase/kg; untreated SPC)	Apparent digestibility of phosphorus (%)	80.87 +/- 3.67	45.43 +/- 3.57	3	3	Hedges' g	7.83	2.26 to 13.41	Higher, more favorable value with phytase; unadjusted 95% CI excluded 0	Not applicable - exact mean +/- SEM values were reported in the abstract
Carter and Sajjadi (2011)	Atlantic salmon (Salmo salar)	Direct phytase supplementation or phytase pre-treatment of soy protein concentrate in a low-fishmeal diet	Pre-treated SPC, equivalent to 250 U/kg diet (417 U/kg SPC)	Control (0 U phytase/kg; untreated SPC)	Apparent digestibility of phosphorus (%)	Not reported numerically	45.43 +/- 3.57	3	3	Not estimable	Not estimable	Not estimable	Figure 3 indicates higher phosphorus digestibility, but an exact standardized effect was not calculated	Exact mean and SEM for this intervention were not reported numerically
Carter and Sajjadi (2011)	Atlantic salmon (Salmo salar)	Direct phytase supplementation or phytase pre-treatment of soy protein concentrate in a low-fishmeal diet	Pre-treated SPC, equivalent to 500 U/kg diet (833 U/kg SPC)	Control (0 U phytase/kg; untreated SPC)	Apparent digestibility of phosphorus (%)	Not reported numerically	45.43 +/- 3.57	3	3	Not estimable	Not estimable	Not estimable	Figure 3 indicates higher phosphorus digestibility, but an exact standardized effect was not calculated	Exact mean and SEM for this intervention were not reported numerically
Carter and Sajjadi (2011)	Atlantic salmon (Salmo salar)	Direct phytase supplementation or phytase pre-treatment of soy protein concentrate in a low-fishmeal diet	Pre-treated SPC, equivalent to 1000 U/kg diet (1667 U/kg SPC)	Control (0 U phytase/kg; untreated SPC)	Apparent digestibility of phosphorus (%)	Not reported numerically	45.43 +/- 3.57	3	3	Not estimable	Not estimable	Not estimable	Figure 3 indicates higher phosphorus digestibility, but an exact standardized effect was not calculated	Exact mean and SEM for this intervention were not reported numerically
Carter and Sajjadi (2011)	Atlantic salmon (Salmo salar)	Direct phytase supplementation or phytase pre-treatment of soy protein concentrate in a low-fishmeal diet	Direct phytase 250 U/kg diet	Control (0 U phytase/kg; untreated SPC)	Survival rate (%)	Not reported by treatment	Not reported by treatment	3	3	Not estimable	Not estimable	Not estimable	The study reported no significant survival difference	Only a treatment range of 96-100% was reported; exact group values and dispersion were unavailable
Carter and Sajjadi (2011)	Atlantic salmon (Salmo salar)	Direct phytase supplementation or phytase pre-treatment of soy protein concentrate in a low-fishmeal diet	Direct phytase 500 U/kg diet	Control (0 U phytase/kg; untreated SPC)	Survival rate (%)	Not reported by treatment	Not reported by treatment	3	3	Not estimable	Not estimable	Not estimable	The study reported no significant survival difference	Only a treatment range of 96-100% was reported; exact group values and dispersion were unavailable
Carter and Sajjadi (2011)	Atlantic salmon (Salmo salar)	Direct phytase supplementation or phytase pre-treatment of soy protein concentrate in a low-fishmeal diet	Direct phytase 1000 U/kg diet	Control (0 U phytase/kg; untreated SPC)	Survival rate (%)	Not reported by treatment	Not reported by treatment	3	3	Not estimable	Not estimable	Not estimable	The study reported no significant survival difference	Only a treatment range of 96-100% was reported; exact group values and dispersion were unavailable
Carter and Sajjadi (2011)	Atlantic salmon (Salmo salar)	Direct phytase supplementation or phytase pre-treatment of soy protein concentrate in a low-fishmeal diet	Direct phytase 4000 U/kg diet	Control (0 U phytase/kg; untreated SPC)	Survival rate (%)	Not reported by treatment	Not reported by treatment	3	3	Not estimable	Not estimable	Not estimable	The study reported no significant survival difference	Only a treatment range of 96-100% was reported; exact group values and dispersion were unavailable
Carter and Sajjadi (2011)	Atlantic salmon (Salmo salar)	Direct phytase supplementation or phytase pre-treatment of soy protein concentrate in a low-fishmeal diet	Pre-treated SPC, equivalent to 250 U/kg diet (417 U/kg SPC)	Control (0 U phytase/kg; untreated SPC)	Survival rate (%)	Not reported by treatment	Not reported by treatment	3	3	Not estimable	Not estimable	Not estimable	The study reported no significant survival difference	Only a treatment range of 96-100% was reported; exact group values and dispersion were unavailable
Carter and Sajjadi (2011)	Atlantic salmon (Salmo salar)	Direct phytase supplementation or phytase pre-treatment of soy protein concentrate in a low-fishmeal diet	Pre-treated SPC, equivalent to 500 U/kg diet (833 U/kg SPC)	Control (0 U phytase/kg; untreated SPC)	Survival rate (%)	Not reported by treatment	Not reported by treatment	3	3	Not estimable	Not estimable	Not estimable	The study reported no significant survival difference	Only a treatment range of 96-100% was reported; exact group values and dispersion were unavailable
Carter and Sajjadi (2011)	Atlantic salmon (Salmo salar)	Direct phytase supplementation or phytase pre-treatment of soy protein concentrate in a low-fishmeal diet	Pre-treated SPC, equivalent to 1000 U/kg diet (1667 U/kg SPC)	Control (0 U phytase/kg; untreated SPC)	Survival rate (%)	Not reported by treatment	Not reported by treatment	3	3	Not estimable	Not estimable	Not estimable	The study reported no significant survival difference	Only a treatment range of 96-100% was reported; exact group values and dispersion were unavailable

Note: Tables 3-5 report mean +/- SEM from three independent tanks per diet; SD was calculated as SEM x sqrt(3). Direct phytase doses and phytase-pre-treated SPC diets were each compared with the untreated control. Exact phosphorus-digestibility values were available only for the control, direct 250 U/kg, and direct 4000 U/kg groups; other Figure 3 comparisons were marked not estimable. Survival was also not estimable because exact treatment values and dispersion were not tabulated. Pairwise 95% CIs are descriptive and unadjusted.

Source: Carter and Sajjadi (2011), https://doi.org/10.1007/s10499-010-9358-z

Study 16. Gunathilaka et al. (2021): shrimp protein hydrolysate and krill meal supplementation in red seabream

Study	Species	Strategy	Intervention	Comparator	Outcome and unit	Intervention mean +/- SD	Comparator mean +/- SD	n intervention (tanks)	n comparator (tanks)	Effect-size metric	Effect size	95% CI	Interpretation	Reason not estimated
Gunathilaka et al. (2021)	Red seabream (Pagrus major)	Shrimp protein hydrolysate or krill meal supplementation in a low-fishmeal diet	KM (20% fishmeal + 5% krill meal)	LFM (25% fishmeal + 21% soy protein concentrate)	Final body weight (g)	63.20 +/- 3.51	52.90 +/- 1.68	3	3	Hedges' g	2.99	0.56 to 5.43	Higher, more favorable value with supplementation; unadjusted 95% CI excluded 0	Not applicable - effect size estimated from reported mean +/- SD
Gunathilaka et al. (2021)	Red seabream (Pagrus major)	Shrimp protein hydrolysate or krill meal supplementation in a low-fishmeal diet	KM (20% fishmeal + 5% krill meal)	LFM (25% fishmeal + 21% soy protein concentrate)	Weight gain (%)	649.00 +/- 47.90	520.00 +/- 15.60	3	3	Hedges' g	2.9	0.52 to 5.28	Higher, more favorable value with supplementation; unadjusted 95% CI excluded 0	Not applicable - effect size estimated from reported mean +/- SD
Gunathilaka et al. (2021)	Red seabream (Pagrus major)	Shrimp protein hydrolysate or krill meal supplementation in a low-fishmeal diet	KM (20% fishmeal + 5% krill meal)	LFM (25% fishmeal + 21% soy protein concentrate)	Feed intake (g/fish)	97.60 +/- 5.48	93.10 +/- 0.91	3	3	Hedges' g	0.92	-0.51 to 2.35	Higher measured value with supplementation; unadjusted 95% CI included 0	Not applicable - effect size estimated from reported mean +/- SD
Gunathilaka et al. (2021)	Red seabream (Pagrus major)	Shrimp protein hydrolysate or krill meal supplementation in a low-fishmeal diet	KM (20% fishmeal + 5% krill meal)	LFM (25% fishmeal + 21% soy protein concentrate)	Feed conversion ratio	1.740 +/- 0.140	2.010 +/- 0.050	3	3	Hedges' g (direction reversed)	2.05	0.14 to 3.97	Lower, more favorable value with supplementation; unadjusted 95% CI excluded 0	Not applicable - effect size estimated from reported mean +/- SD
Gunathilaka et al. (2021)	Red seabream (Pagrus major)	Shrimp protein hydrolysate or krill meal supplementation in a low-fishmeal diet	KM (20% fishmeal + 5% krill meal)	LFM (25% fishmeal + 21% soy protein concentrate)	Protein efficiency ratio	1.190 +/- 0.090	1.060 +/- 0.030	3	3	Hedges' g	1.55	-0.12 to 3.22	Higher, more favorable value with supplementation; unadjusted 95% CI included 0	Not applicable - effect size estimated from reported mean +/- SD
Gunathilaka et al. (2021)	Red seabream (Pagrus major)	Shrimp protein hydrolysate or krill meal supplementation in a low-fishmeal diet	KM (20% fishmeal + 5% krill meal)	LFM (25% fishmeal + 21% soy protein concentrate)	Specific growth rate (%/day)	1.920 +/- 0.060	1.740 +/- 0.020	3	3	Hedges' g	3.22	0.65 to 5.79	Higher, more favorable value with supplementation; unadjusted 95% CI excluded 0	Not applicable - effect size estimated from reported mean +/- SD
Gunathilaka et al. (2021)	Red seabream (Pagrus major)	Shrimp protein hydrolysate or krill meal supplementation in a low-fishmeal diet	KM (20% fishmeal + 5% krill meal)	LFM (25% fishmeal + 21% soy protein concentrate)	Liver IGF-I relative mRNA expression	0.820 +/- 0.070	0.300 +/- 0.050	3	3	Hedges' g	6.84	1.93 to 11.75	Higher, more favorable value with supplementation; unadjusted 95% CI excluded 0	Not applicable - effect size estimated from reported mean +/- SD
Gunathilaka et al. (2021)	Red seabream (Pagrus major)	Shrimp protein hydrolysate or krill meal supplementation in a low-fishmeal diet	KM (20% fishmeal + 5% krill meal)	LFM (25% fishmeal + 21% soy protein concentrate)	Survival rate (%)	84.40 +/- 5.09	84.40 +/- 1.92	3	3	Hedges' g	0.0	-1.28 to 1.28	No observed mean difference; unadjusted 95% CI included 0	Not applicable - effect size estimated from reported mean +/- SD
Gunathilaka et al. (2021)	Red seabream (Pagrus major)	Shrimp protein hydrolysate or krill meal supplementation in a low-fishmeal diet	KM (20% fishmeal + 5% krill meal)	LFM (25% fishmeal + 21% soy protein concentrate)	Nitro blue tetrazolium activity (OD)	0.840 +/- 0.070	0.710 +/- 0.040	3	3	Hedges' g	1.82	0.03 to 3.62	Higher, more favorable value with supplementation; unadjusted 95% CI excluded 0	Not applicable - effect size estimated from reported mean +/- SD
Gunathilaka et al. (2021)	Red seabream (Pagrus major)	Shrimp protein hydrolysate or krill meal supplementation in a low-fishmeal diet	KM (20% fishmeal + 5% krill meal)	LFM (25% fishmeal + 21% soy protein concentrate)	Myeloperoxidase activity (OD)	1.760 +/- 0.440	1.360 +/- 0.110	3	3	Hedges' g	1.0	-0.46 to 2.45	Higher measured value with supplementation; unadjusted 95% CI included 0	Not applicable - effect size estimated from reported mean +/- SD
Gunathilaka et al. (2021)	Red seabream (Pagrus major)	Shrimp protein hydrolysate or krill meal supplementation in a low-fishmeal diet	KM (20% fishmeal + 5% krill meal)	LFM (25% fishmeal + 21% soy protein concentrate)	Superoxide dismutase activity (% inhibition)	74.60 +/- 2.20	63.30 +/- 2.70	3	3	Hedges' g	3.67	0.82 to 6.52	Higher, more favorable value with supplementation; unadjusted 95% CI excluded 0	Not applicable - effect size estimated from reported mean +/- SD
Gunathilaka et al. (2021)	Red seabream (Pagrus major)	Shrimp protein hydrolysate or krill meal supplementation in a low-fishmeal diet	KM (20% fishmeal + 5% krill meal)	LFM (25% fishmeal + 21% soy protein concentrate)	Glutathione peroxidase activity (mU/mL)	95.50 +/- 20.00	75.90 +/- 7.90	3	3	Hedges' g	1.03	-0.43 to 2.50	Higher, more favorable value with supplementation; unadjusted 95% CI included 0	Not applicable - effect size estimated from reported mean +/- SD
Gunathilaka et al. (2021)	Red seabream (Pagrus major)	Shrimp protein hydrolysate or krill meal supplementation in a low-fishmeal diet	KM (20% fishmeal + 5% krill meal)	LFM (25% fishmeal + 21% soy protein concentrate)	Catalase activity (mU/mL)	1.190 +/- 0.070	1.050 +/- 0.090	3	3	Hedges' g	1.39	-0.21 to 2.99	Higher, more favorable value with supplementation; unadjusted 95% CI included 0	Not applicable - effect size estimated from reported mean +/- SD
Gunathilaka et al. (2021)	Red seabream (Pagrus major)	Shrimp protein hydrolysate or krill meal supplementation in a low-fishmeal diet	KM (20% fishmeal + 5% krill meal)	LFM (25% fishmeal + 21% soy protein concentrate)	Hematocrit (%)	37.60 +/- 2.80	32.70 +/- 0.90	3	3	Hedges' g	1.88	0.06 to 3.71	Higher, more favorable value with supplementation; unadjusted 95% CI excluded 0	Not applicable - effect size estimated from reported mean +/- SD
Gunathilaka et al. (2021)	Red seabream (Pagrus major)	Shrimp protein hydrolysate or krill meal supplementation in a low-fishmeal diet	KM (20% fishmeal + 5% krill meal)	LFM (25% fishmeal + 21% soy protein concentrate)	Hemoglobin (g/dL)	6.960 +/- 0.770	4.860 +/- 1.060	3	3	Hedges' g	1.81	0.02 to 3.61	Higher, more favorable value with supplementation; unadjusted 95% CI excluded 0	Not applicable - effect size estimated from reported mean +/- SD
Gunathilaka et al. (2021)	Red seabream (Pagrus major)	Shrimp protein hydrolysate or krill meal supplementation in a low-fishmeal diet	KM (20% fishmeal + 5% krill meal)	LFM (25% fishmeal + 21% soy protein concentrate)	Total protein (g/dL)	1.680 +/- 0.470	1.430 +/- 0.040	3	3	Hedges' g	0.6	-0.75 to 1.95	Higher measured value with supplementation; unadjusted 95% CI included 0	Not applicable - effect size estimated from reported mean +/- SD
Gunathilaka et al. (2021)	Red seabream (Pagrus major)	Shrimp protein hydrolysate or krill meal supplementation in a low-fishmeal diet	KM (20% fishmeal + 5% krill meal)	LFM (25% fishmeal + 21% soy protein concentrate)	Glucose (mg/dL)	55.80 +/- 3.80	55.50 +/- 1.60	3	3	Hedges' g	0.08	-1.20 to 1.36	Higher measured value with supplementation; unadjusted 95% CI included 0	Not applicable - effect size estimated from reported mean +/- SD
Gunathilaka et al. (2021)	Red seabream (Pagrus major)	Shrimp protein hydrolysate or krill meal supplementation in a low-fishmeal diet	KM (20% fishmeal + 5% krill meal)	LFM (25% fishmeal + 21% soy protein concentrate)	Total cholesterol (mg/dL)	217.00 +/- 13.00	210.00 +/- 6.00	3	3	Hedges' g	0.55	-0.78 to 1.89	Higher measured value with supplementation; unadjusted 95% CI included 0	Not applicable - effect size estimated from reported mean +/- SD
Gunathilaka et al. (2021)	Red seabream (Pagrus major)	Shrimp protein hydrolysate or krill meal supplementation in a low-fishmeal diet	KM (20% fishmeal + 5% krill meal)	LFM (25% fishmeal + 21% soy protein concentrate)	Dry matter (%)	32.90 +/- 0.60	32.70 +/- 1.20	3	3	Hedges' g	0.17	-1.12 to 1.45	Higher measured value with supplementation; unadjusted 95% CI included 0	Not applicable - effect size estimated from reported mean +/- SD
Gunathilaka et al. (2021)	Red seabream (Pagrus major)	Shrimp protein hydrolysate or krill meal supplementation in a low-fishmeal diet	KM (20% fishmeal + 5% krill meal)	LFM (25% fishmeal + 21% soy protein concentrate)	Crude protein (% dry matter)	53.60 +/- 1.40	51.80 +/- 0.30	3	3	Hedges' g	1.42	-0.19 to 3.04	Higher measured value with supplementation; unadjusted 95% CI included 0	Not applicable - effect size estimated from reported mean +/- SD
Gunathilaka et al. (2021)	Red seabream (Pagrus major)	Shrimp protein hydrolysate or krill meal supplementation in a low-fishmeal diet	KM (20% fishmeal + 5% krill meal)	LFM (25% fishmeal + 21% soy protein concentrate)	Crude lipid (% dry matter)	33.40 +/- 2.10	32.80 +/- 2.50	3	3	Hedges' g	0.21	-1.08 to 1.50	Higher measured value with supplementation; unadjusted 95% CI included 0	Not applicable - effect size estimated from reported mean +/- SD
Gunathilaka et al. (2021)	Red seabream (Pagrus major)	Shrimp protein hydrolysate or krill meal supplementation in a low-fishmeal diet	KM (20% fishmeal + 5% krill meal)	LFM (25% fishmeal + 21% soy protein concentrate)	Ash (% dry matter)	13.70 +/- 1.40	11.40 +/- 0.60	3	3	Hedges' g	1.71	-0.04 to 3.45	Higher measured value with supplementation; unadjusted 95% CI included 0	Not applicable - effect size estimated from reported mean +/- SD
Gunathilaka et al. (2021)	Red seabream (Pagrus major)	Shrimp protein hydrolysate or krill meal supplementation in a low-fishmeal diet	SH (20.2% fishmeal + 5% shrimp protein hydrolysate)	LFM (25% fishmeal + 21% soy protein concentrate)	Final body weight (g)	76.30 +/- 4.79	52.90 +/- 1.68	3	3	Hedges' g	5.22	1.38 to 9.05	Higher, more favorable value with supplementation; unadjusted 95% CI excluded 0	Not applicable - effect size estimated from reported mean +/- SD
Gunathilaka et al. (2021)	Red seabream (Pagrus major)	Shrimp protein hydrolysate or krill meal supplementation in a low-fishmeal diet	SH (20.2% fishmeal + 5% shrimp protein hydrolysate)	LFM (25% fishmeal + 21% soy protein concentrate)	Weight gain (%)	810.00 +/- 60.70	520.00 +/- 15.60	3	3	Hedges' g	5.24	1.39 to 9.08	Higher, more favorable value with supplementation; unadjusted 95% CI excluded 0	Not applicable - effect size estimated from reported mean +/- SD
Gunathilaka et al. (2021)	Red seabream (Pagrus major)	Shrimp protein hydrolysate or krill meal supplementation in a low-fishmeal diet	SH (20.2% fishmeal + 5% shrimp protein hydrolysate)	LFM (25% fishmeal + 21% soy protein concentrate)	Feed intake (g/fish)	95.10 +/- 6.46	93.10 +/- 0.91	3	3	Hedges' g	0.35	-0.96 to 1.65	Higher measured value with supplementation; unadjusted 95% CI included 0	Not applicable - effect size estimated from reported mean +/- SD
Gunathilaka et al. (2021)	Red seabream (Pagrus major)	Shrimp protein hydrolysate or krill meal supplementation in a low-fishmeal diet	SH (20.2% fishmeal + 5% shrimp protein hydrolysate)	LFM (25% fishmeal + 21% soy protein concentrate)	Feed conversion ratio	1.510 +/- 0.050	2.010 +/- 0.050	3	3	Hedges' g (direction reversed)	8.0	2.31 to 13.69	Lower, more favorable value with supplementation; unadjusted 95% CI excluded 0	Not applicable - effect size estimated from reported mean +/- SD
Gunathilaka et al. (2021)	Red seabream (Pagrus major)	Shrimp protein hydrolysate or krill meal supplementation in a low-fishmeal diet	SH (20.2% fishmeal + 5% shrimp protein hydrolysate)	LFM (25% fishmeal + 21% soy protein concentrate)	Protein efficiency ratio	1.390 +/- 0.050	1.060 +/- 0.030	3	3	Hedges' g	6.4	1.78 to 11.02	Higher, more favorable value with supplementation; unadjusted 95% CI excluded 0	Not applicable - effect size estimated from reported mean +/- SD
Gunathilaka et al. (2021)	Red seabream (Pagrus major)	Shrimp protein hydrolysate or krill meal supplementation in a low-fishmeal diet	SH (20.2% fishmeal + 5% shrimp protein hydrolysate)	LFM (25% fishmeal + 21% soy protein concentrate)	Specific growth rate (%/day)	2.100 +/- 0.060	1.740 +/- 0.020	3	3	Hedges' g	6.44	1.80 to 11.08	Higher, more favorable value with supplementation; unadjusted 95% CI excluded 0	Not applicable - effect size estimated from reported mean +/- SD
Gunathilaka et al. (2021)	Red seabream (Pagrus major)	Shrimp protein hydrolysate or krill meal supplementation in a low-fishmeal diet	SH (20.2% fishmeal + 5% shrimp protein hydrolysate)	LFM (25% fishmeal + 21% soy protein concentrate)	Liver IGF-I relative mRNA expression	1.120 +/- 0.070	0.300 +/- 0.050	3	3	Hedges' g	10.78	3.20 to 18.37	Higher, more favorable value with supplementation; unadjusted 95% CI excluded 0	Not applicable - effect size estimated from reported mean +/- SD
Gunathilaka et al. (2021)	Red seabream (Pagrus major)	Shrimp protein hydrolysate or krill meal supplementation in a low-fishmeal diet	SH (20.2% fishmeal + 5% shrimp protein hydrolysate)	LFM (25% fishmeal + 21% soy protein concentrate)	Survival rate (%)	90.00 +/- 3.33	84.40 +/- 1.92	3	3	Hedges' g	1.65	-0.07 to 3.36	Higher, more favorable value with supplementation; unadjusted 95% CI included 0	Not applicable - effect size estimated from reported mean +/- SD
Gunathilaka et al. (2021)	Red seabream (Pagrus major)	Shrimp protein hydrolysate or krill meal supplementation in a low-fishmeal diet	SH (20.2% fishmeal + 5% shrimp protein hydrolysate)	LFM (25% fishmeal + 21% soy protein concentrate)	Nitro blue tetrazolium activity (OD)	1.020 +/- 0.060	0.710 +/- 0.040	3	3	Hedges' g	4.86	1.26 to 8.47	Higher, more favorable value with supplementation; unadjusted 95% CI excluded 0	Not applicable - effect size estimated from reported mean +/- SD

Study	Species	Strategy	Intervention	Comparator	Outcome and unit	Intervention mean +/- SD	Comparator mean +/- SD	n intervention (tanks)	n comparator (tanks)	Effect-size metric	Effect size	95% CI	Interpretation	Reason not estimated
Gunathilaka et al. (2021)	Red seabream (Pagrus major)	Shrimp protein hydrolysate or krill meal supplementation in a low-fishmeal diet	SH (20.2% fishmeal + 5% shrimp protein hydrolysate)	LFM (25% fishmeal + 21% soy protein concentrate)	Myeloperoxidase activity (OD)	1.810 +/- 0.090	1.360 +/- 0.110	3	3	Hedges' g	3.58	0.79 to 6.38	Higher measured value with supplementation; unadjusted 95% CI excluded 0	Not applicable - effect size estimated from reported mean +/- SD
Gunathilaka et al. (2021)	Red seabream (Pagrus major)	Shrimp protein hydrolysate or krill meal supplementation in a low-fishmeal diet	SH (20.2% fishmeal + 5% shrimp protein hydrolysate)	LFM (25% fishmeal + 21% soy protein concentrate)	Superoxide dismutase activity (% inhibition)	81.60 +/- 4.10	63.30 +/- 2.70	3	3	Hedges' g	4.22	1.03 to 7.41	Higher, more favorable value with supplementation; unadjusted 95% CI excluded 0	Not applicable - effect size estimated from reported mean +/- SD
Gunathilaka et al. (2021)	Red seabream (Pagrus major)	Shrimp protein hydrolysate or krill meal supplementation in a low-fishmeal diet	SH (20.2% fishmeal + 5% shrimp protein hydrolysate)	LFM (25% fishmeal + 21% soy protein concentrate)	Glutathione peroxidase activity (mU/mL)	116.00 +/- 8.80	75.90 +/- 7.90	3	3	Hedges' g	3.84	0.89 to 6.79	Higher, more favorable value with supplementation; unadjusted 95% CI excluded 0	Not applicable - effect size estimated from reported mean +/- SD
Gunathilaka et al. (2021)	Red seabream (Pagrus major)	Shrimp protein hydrolysate or krill meal supplementation in a low-fishmeal diet	SH (20.2% fishmeal + 5% shrimp protein hydrolysate)	LFM (25% fishmeal + 21% soy protein concentrate)	Catalase activity (mU/mL)	1.410 +/- 0.140	1.050 +/- 0.090	3	3	Hedges' g	2.45	0.32 to 4.57	Higher, more favorable value with supplementation; unadjusted 95% CI excluded 0	Not applicable - effect size estimated from reported mean +/- SD
Gunathilaka et al. (2021)	Red seabream (Pagrus major)	Shrimp protein hydrolysate or krill meal supplementation in a low-fishmeal diet	SH (20.2% fishmeal + 5% shrimp protein hydrolysate)	LFM (25% fishmeal + 21% soy protein concentrate)	Hematocrit (%)	35.80 +/- 1.30	32.70 +/- 0.90	3	3	Hedges' g	2.22	0.22 to 4.22	Higher, more favorable value with supplementation; unadjusted 95% CI excluded 0	Not applicable - effect size estimated from reported mean +/- SD
Gunathilaka et al. (2021)	Red seabream (Pagrus major)	Shrimp protein hydrolysate or krill meal supplementation in a low-fishmeal diet	SH (20.2% fishmeal + 5% shrimp protein hydrolysate)	LFM (25% fishmeal + 21% soy protein concentrate)	Hemoglobin (g/dL)	6.580 +/- 0.270	4.860 +/- 1.060	3	3	Hedges' g	1.78	0.00 to 3.56	Higher, more favorable value with supplementation; unadjusted 95% CI excluded 0	Not applicable - effect size estimated from reported mean +/- SD
Gunathilaka et al. (2021)	Red seabream (Pagrus major)	Shrimp protein hydrolysate or krill meal supplementation in a low-fishmeal diet	SH (20.2% fishmeal + 5% shrimp protein hydrolysate)	LFM (25% fishmeal + 21% soy protein concentrate)	Total protein (g/dL)	1.940 +/- 0.090	1.430 +/- 0.040	3	3	Hedges' g	5.86	1.60 to 10.12	Higher measured value with supplementation; unadjusted 95% CI excluded 0	Not applicable - effect size estimated from reported mean +/- SD
Gunathilaka et al. (2021)	Red seabream (Pagrus major)	Shrimp protein hydrolysate or krill meal supplementation in a low-fishmeal diet	SH (20.2% fishmeal + 5% shrimp protein hydrolysate)	LFM (25% fishmeal + 21% soy protein concentrate)	Glucose (mg/dL)	57.60 +/- 3.00	55.50 +/- 1.60	3	3	Hedges' g	0.7	-0.67 to 2.07	Higher measured value with supplementation; unadjusted 95% CI included 0	Not applicable - effect size estimated from reported mean +/- SD
Gunathilaka et al. (2021)	Red seabream (Pagrus major)	Shrimp protein hydrolysate or krill meal supplementation in a low-fishmeal diet	SH (20.2% fishmeal + 5% shrimp protein hydrolysate)	LFM (25% fishmeal + 21% soy protein concentrate)	Total cholesterol (mg/dL)	231.00 +/- 13.00	210.00 +/- 6.00	3	3	Hedges' g	1.66	-0.06 to 3.38	Higher measured value with supplementation; unadjusted 95% CI included 0	Not applicable - effect size estimated from reported mean +/- SD
Gunathilaka et al. (2021)	Red seabream (Pagrus major)	Shrimp protein hydrolysate or krill meal supplementation in a low-fishmeal diet	SH (20.2% fishmeal + 5% shrimp protein hydrolysate)	LFM (25% fishmeal + 21% soy protein concentrate)	Dry matter (%)	33.10 +/- 1.59	32.70 +/- 1.20	3	3	Hedges' g	0.23	-1.06 to 1.52	Higher measured value with supplementation; unadjusted 95% CI included 0	Not applicable - effect size estimated from reported mean +/- SD
Gunathilaka et al. (2021)	Red seabream (Pagrus major)	Shrimp protein hydrolysate or krill meal supplementation in a low-fishmeal diet	SH (20.2% fishmeal + 5% shrimp protein hydrolysate)	LFM (25% fishmeal + 21% soy protein concentrate)	Crude protein (% dry matter)	54.00 +/- 0.20	51.80 +/- 0.30	3	3	Hedges' g	6.9	1.95 to 11.86	Higher measured value with supplementation; unadjusted 95% CI excluded 0	Not applicable - effect size estimated from reported mean +/- SD
Gunathilaka et al. (2021)	Red seabream (Pagrus major)	Shrimp protein hydrolysate or krill meal supplementation in a low-fishmeal diet	SH (20.2% fishmeal + 5% shrimp protein hydrolysate)	LFM (25% fishmeal + 21% soy protein concentrate)	Crude lipid (% dry matter)	33.80 +/- 0.80	32.80 +/- 2.50	3	3	Hedges' g	0.43	-0.88 to 1.75	Higher measured value with supplementation; unadjusted 95% CI included 0	Not applicable - effect size estimated from reported mean +/- SD
Gunathilaka et al. (2021)	Red seabream (Pagrus major)	Shrimp protein hydrolysate or krill meal supplementation in a low-fishmeal diet	SH (20.2% fishmeal + 5% shrimp protein hydrolysate)	LFM (25% fishmeal + 21% soy protein concentrate)	Ash (% dry matter)	13.80 +/- 0.80	11.40 +/- 0.60	3	3	Hedges' g	2.72	0.44 to 4.99	Higher measured value with supplementation; unadjusted 95% CI excluded 0	Not applicable - effect size estimated from reported mean +/- SD
Gunathilaka et al. (2021)	Red seabream (Pagrus major)	Shrimp protein hydrolysate or krill meal supplementation in a low-fishmeal diet	KM (20% fishmeal + 5% krill meal)	HFM (40% fishmeal reference diet)	Final body weight (g)	63.20 +/- 3.51	65.00 +/- 2.55	3	3	Hedges' g	-0.47	-1.79 to 0.85	Lower, less favorable value with supplementation; unadjusted 95% CI included 0	Not applicable - effect size estimated from reported mean +/- SD
Gunathilaka et al. (2021)	Red seabream (Pagrus major)	Shrimp protein hydrolysate or krill meal supplementation in a low-fishmeal diet	KM (20% fishmeal + 5% krill meal)	HFM (40% fishmeal reference diet)	Weight gain (%)	649.00 +/- 47.90	666.00 +/- 29.40	3	3	Hedges' g	-0.34	-1.64 to 0.96	Lower, less favorable value with supplementation; unadjusted 95% CI included 0	Not applicable - effect size estimated from reported mean +/- SD
Gunathilaka et al. (2021)	Red seabream (Pagrus major)	Shrimp protein hydrolysate or krill meal supplementation in a low-fishmeal diet	KM (20% fishmeal + 5% krill meal)	HFM (40% fishmeal reference diet)	Feed intake (g/fish)	97.60 +/- 5.48	91.30 +/- 3.94	3	3	Hedges' g	1.06	-0.42 to 2.53	Higher measured value with supplementation; unadjusted 95% CI included 0	Not applicable - effect size estimated from reported mean +/- SD
Gunathilaka et al. (2021)	Red seabream (Pagrus major)	Shrimp protein hydrolysate or krill meal supplementation in a low-fishmeal diet	KM (20% fishmeal + 5% krill meal)	HFM (40% fishmeal reference diet)	Feed conversion ratio	1.740 +/- 0.140	1.600 +/- 0.080	3	3	Hedges' g (direction reversed)	-0.98	-2.43 to 0.47	Higher, less favorable value with supplementation; unadjusted 95% CI included 0	Not applicable - effect size estimated from reported mean +/- SD
Gunathilaka et al. (2021)	Red seabream (Pagrus major)	Shrimp protein hydrolysate or krill meal supplementation in a low-fishmeal diet	KM (20% fishmeal + 5% krill meal)	HFM (40% fishmeal reference diet)	Protein efficiency ratio	1.190 +/- 0.090	1.330 +/- 0.070	3	3	Hedges' g	-1.39	-2.99 to 0.21	Lower, less favorable value with supplementation; unadjusted 95% CI included 0	Not applicable - effect size estimated from reported mean +/- SD
Gunathilaka et al. (2021)	Red seabream (Pagrus major)	Shrimp protein hydrolysate or krill meal supplementation in a low-fishmeal diet	KM (20% fishmeal + 5% krill meal)	HFM (40% fishmeal reference diet)	Specific growth rate (%/day)	1.920 +/- 0.060	1.940 +/- 0.040	3	3	Hedges' g	-0.31	-1.61 to 0.98	Lower, less favorable value with supplementation; unadjusted 95% CI included 0	Not applicable - effect size estimated from reported mean +/- SD
Gunathilaka et al. (2021)	Red seabream (Pagrus major)	Shrimp protein hydrolysate or krill meal supplementation in a low-fishmeal diet	KM (20% fishmeal + 5% krill meal)	HFM (40% fishmeal reference diet)	Liver IGF-I relative mRNA expression	0.820 +/- 0.070	1.000 +/- 0.000	3	3	Hedges' g	-2.91	-5.30 to -0.52	Lower, less favorable value with supplementation; unadjusted 95% CI excluded 0	Not applicable - effect size estimated from reported mean +/- SD
Gunathilaka et al. (2021)	Red seabream (Pagrus major)	Shrimp protein hydrolysate or krill meal supplementation in a low-fishmeal diet	KM (20% fishmeal + 5% krill meal)	HFM (40% fishmeal reference diet)	Survival rate (%)	84.40 +/- 5.09	90.00 +/- 5.77	3	3	Hedges' g	-0.82	-2.23 to 0.58	Lower, less favorable value with supplementation; unadjusted 95% CI included 0	Not applicable - effect size estimated from reported mean +/- SD
Gunathilaka et al. (2021)	Red seabream (Pagrus major)	Shrimp protein hydrolysate or krill meal supplementation in a low-fishmeal diet	KM (20% fishmeal + 5% krill meal)	HFM (40% fishmeal reference diet)	Nitro blue tetrazolium activity (OD)	0.840 +/- 0.070	0.950 +/- 0.070	3	3	Hedges' g	-1.26	-2.81 to 0.29	Lower, less favorable value with supplementation; unadjusted 95% CI included 0	Not applicable - effect size estimated from reported mean +/- SD
Gunathilaka et al. (2021)	Red seabream (Pagrus major)	Shrimp protein hydrolysate or krill meal supplementation in a low-fishmeal diet	KM (20% fishmeal + 5% krill meal)	HFM (40% fishmeal reference diet)	Myeloperoxidase activity (OD)	1.760 +/- 0.440	1.660 +/- 0.130	3	3	Hedges' g	0.25	-1.05 to 1.54	Higher measured value with supplementation; unadjusted 95% CI included 0	Not applicable - effect size estimated from reported mean +/- SD
Gunathilaka et al. (2021)	Red seabream (Pagrus major)	Shrimp protein hydrolysate or krill meal supplementation in a low-fishmeal diet	KM (20% fishmeal + 5% krill meal)	HFM (40% fishmeal reference diet)	Superoxide dismutase activity (% inhibition)	74.60 +/- 2.20	74.80 +/- 0.30	3	3	Hedges' g	-0.1	-1.38 to 1.18	Lower, less favorable value with supplementation; unadjusted 95% CI included 0	Not applicable - effect size estimated from reported mean +/- SD
Gunathilaka et al. (2021)	Red seabream (Pagrus major)	Shrimp protein hydrolysate or krill meal supplementation in a low-fishmeal diet	KM (20% fishmeal + 5% krill meal)	HFM (40% fishmeal reference diet)	Glutathione peroxidase activity (mU/mL)	95.50 +/- 20.00	109.00 +/- 17.00	3	3	Hedges' g	-0.58	-1.92 to 0.76	Lower, less favorable value with supplementation; unadjusted 95% CI included 0	Not applicable - effect size estimated from reported mean +/- SD
Gunathilaka et al. (2021)	Red seabream (Pagrus major)	Shrimp protein hydrolysate or krill meal supplementation in a low-fishmeal diet	KM (20% fishmeal + 5% krill meal)	HFM (40% fishmeal reference diet)	Catalase activity (mU/mL)	1.190 +/- 0.070	1.350 +/- 0.090	3	3	Hedges' g	-1.59	-3.28 to 0.10	Lower, less favorable value with supplementation; unadjusted 95% CI included 0	Not applicable - effect size estimated from reported mean +/- SD
Gunathilaka et al. (2021)	Red seabream (Pagrus major)	Shrimp protein hydrolysate or krill meal supplementation in a low-fishmeal diet	KM (20% fishmeal + 5% krill meal)	HFM (40% fishmeal reference diet)	Hematocrit (%)	37.60 +/- 2.80	37.60 +/- 4.00	3	3	Hedges' g	0.0	-1.28 to 1.28	No observed mean difference; unadjusted 95% CI included 0	Not applicable - effect size estimated from reported mean +/- SD
Gunathilaka et al. (2021)	Red seabream (Pagrus major)	Shrimp protein hydrolysate or krill meal supplementation in a low-fishmeal diet	KM (20% fishmeal + 5% krill meal)	HFM (40% fishmeal reference diet)	Hemoglobin (g/dL)	6.960 +/- 0.770	5.890 +/- 0.200	3	3	Hedges' g	1.52	-0.14 to 3.18	Higher, more favorable value with supplementation; unadjusted 95% CI included 0	Not applicable - effect size estimated from reported mean +/- SD
Gunathilaka et al. (2021)	Red seabream (Pagrus major)	Shrimp protein hydrolysate or krill meal supplementation in a low-fishmeal diet	KM (20% fishmeal + 5% krill meal)	HFM (40% fishmeal reference diet)	Total protein (g/dL)	1.680 +/- 0.470	1.870 +/- 0.150	3	3	Hedges' g	-0.44	-1.75 to 0.88	Lower measured value with supplementation; unadjusted 95% CI included 0	Not applicable - effect size estimated from reported mean +/- SD
Gunathilaka et al. (2021)	Red seabream (Pagrus major)	Shrimp protein hydrolysate or krill meal supplementation in a low-fishmeal diet	KM (20% fishmeal + 5% krill meal)	HFM (40% fishmeal reference diet)	Glucose (mg/dL)	55.80 +/- 3.80	57.60 +/- 1.00	3	3	Hedges' g	-0.52	-1.85 to 0.81	Lower measured value with supplementation; unadjusted 95% CI included 0	Not applicable - effect size estimated from reported mean +/- SD
Gunathilaka et al. (2021)	Red seabream (Pagrus major)	Shrimp protein hydrolysate or krill meal supplementation in a low-fishmeal diet	KM (20% fishmeal + 5% krill meal)	HFM (40% fishmeal reference diet)	Total cholesterol (mg/dL)	217.00 +/- 13.00	220.00 +/- 12.00	3	3	Hedges' g	-0.19	-1.48 to 1.10	Lower measured value with supplementation; unadjusted 95% CI included 0	Not applicable - effect size estimated from reported mean +/- SD
Gunathilaka et al. (2021)	Red seabream (Pagrus major)	Shrimp protein hydrolysate or krill meal supplementation in a low-fishmeal diet	KM (20% fishmeal + 5% krill meal)	HFM (40% fishmeal reference diet)	Dry matter (%)	32.90 +/- 0.60	30.00 +/- 0.41	3	3	Hedges' g	4.51	1.13 to 7.90	Higher measured value with supplementation; unadjusted 95% CI excluded 0	Not applicable - effect size estimated from reported mean +/- SD

Study	Species	Strategy	Intervention	Comparator	Outcome and unit	Intervention mean +/- SD	Comparator mean +/- SD	n intervention (tanks)	n comparator (tanks)	Effect-size metric	Effect size	95% CI	Interpretation	Reason not estimated
Gunathilaka et al. (2021)	Red seabream (Pagrus major)	Shrimp protein hydrolysate or krill meal supplementation in a low-fishmeal diet	KM (20% fishmeal + 5% krill meal)	HFM (40% fishmeal reference diet)	Crude protein (% dry matter)	53.60 +/- 1.40	53.60 +/- 0.40	3	3	Hedges' g	0.0	-1.28 to 1.28	No observed mean difference; unadjusted 95% CI included 0	Not applicable - effect size estimated from reported mean +/- SD
Gunathilaka et al. (2021)	Red seabream (Pagrus major)	Shrimp protein hydrolysate or krill meal supplementation in a low-fishmeal diet	KM (20% fishmeal + 5% krill meal)	HFM (40% fishmeal reference diet)	Crude lipid (% dry matter)	33.40 +/- 2.10	34.10 +/- 2.50	3	3	Hedges' g	-0.24	-1.53 to 1.05	Lower measured value with supplementation; unadjusted 95% CI included 0	Not applicable - effect size estimated from reported mean +/- SD
Gunathilaka et al. (2021)	Red seabream (Pagrus major)	Shrimp protein hydrolysate or krill meal supplementation in a low-fishmeal diet	KM (20% fishmeal + 5% krill meal)	HFM (40% fishmeal reference diet)	Ash (% dry matter)	13.70 +/- 1.40	13.90 +/- 1.00	3	3	Hedges' g	-0.13	-1.42 to 1.15	Lower measured value with supplementation; unadjusted 95% CI included 0	Not applicable - effect size estimated from reported mean +/- SD
Gunathilaka et al. (2021)	Red seabream (Pagrus major)	Shrimp protein hydrolysate or krill meal supplementation in a low-fishmeal diet	SH (20.2% fishmeal + 5% shrimp protein hydrolysate)	HFM (40% fishmeal reference diet)	Final body weight (g)	76.30 +/- 4.79	65.00 +/- 2.55	3	3	Hedges' g	2.36	0.28 to 4.43	Higher, more favorable value with supplementation; unadjusted 95% CI excluded 0	Not applicable - effect size estimated from reported mean +/- SD
Gunathilaka et al. (2021)	Red seabream (Pagrus major)	Shrimp protein hydrolysate or krill meal supplementation in a low-fishmeal diet	SH (20.2% fishmeal + 5% shrimp protein hydrolysate)	HFM (40% fishmeal reference diet)	Weight gain (%)	810.00 +/- 60.70	666.00 +/- 29.40	3	3	Hedges' g	2.42	0.31 to 4.52	Higher, more favorable value with supplementation; unadjusted 95% CI excluded 0	Not applicable - effect size estimated from reported mean +/- SD
Gunathilaka et al. (2021)	Red seabream (Pagrus major)	Shrimp protein hydrolysate or krill meal supplementation in a low-fishmeal diet	SH (20.2% fishmeal + 5% shrimp protein hydrolysate)	HFM (40% fishmeal reference diet)	Feed intake (g/fish)	95.10 +/- 6.46	91.30 +/- 3.94	3	3	Hedges' g	0.57	-0.77 to 1.91	Higher measured value with supplementation; unadjusted 95% CI included 0	Not applicable - effect size estimated from reported mean +/- SD
Gunathilaka et al. (2021)	Red seabream (Pagrus major)	Shrimp protein hydrolysate or krill meal supplementation in a low-fishmeal diet	SH (20.2% fishmeal + 5% shrimp protein hydrolysate)	HFM (40% fishmeal reference diet)	Feed conversion ratio	1.510 +/- 0.050	1.600 +/- 0.080	3	3	Hedges' g (direction reversed)	1.08	-0.40 to 2.56	Lower, more favorable value with supplementation; unadjusted 95% CI included 0	Not applicable - effect size estimated from reported mean +/- SD
Gunathilaka et al. (2021)	Red seabream (Pagrus major)	Shrimp protein hydrolysate or krill meal supplementation in a low-fishmeal diet	SH (20.2% fishmeal + 5% shrimp protein hydrolysate)	HFM (40% fishmeal reference diet)	Protein efficiency ratio	1.390 +/- 0.050	1.330 +/- 0.070	3	3	Hedges' g	0.79	-0.60 to 2.18	Higher, more favorable value with supplementation; unadjusted 95% CI included 0	Not applicable - effect size estimated from reported mean +/- SD
Gunathilaka et al. (2021)	Red seabream (Pagrus major)	Shrimp protein hydrolysate or krill meal supplementation in a low-fishmeal diet	SH (20.2% fishmeal + 5% shrimp protein hydrolysate)	HFM (40% fishmeal reference diet)	Specific growth rate (%/day)	2.100 +/- 0.060	1.940 +/- 0.040	3	3	Hedges' g	2.51	0.35 to 4.67	Higher, more favorable value with supplementation; unadjusted 95% CI excluded 0	Not applicable - effect size estimated from reported mean +/- SD
Gunathilaka et al. (2021)	Red seabream (Pagrus major)	Shrimp protein hydrolysate or krill meal supplementation in a low-fishmeal diet	SH (20.2% fishmeal + 5% shrimp protein hydrolysate)	HFM (40% fishmeal reference diet)	Liver IGF-I relative mRNA expression	1.120 +/- 0.070	1.000 +/- 0.000	3	3	Hedges' g	1.94	0.08 to 3.80	Higher, more favorable value with supplementation; unadjusted 95% CI excluded 0	Not applicable - effect size estimated from reported mean +/- SD
Gunathilaka et al. (2021)	Red seabream (Pagrus major)	Shrimp protein hydrolysate or krill meal supplementation in a low-fishmeal diet	SH (20.2% fishmeal + 5% shrimp protein hydrolysate)	HFM (40% fishmeal reference diet)	Survival rate (%)	90.00 +/- 3.33	90.00 +/- 5.77	3	3	Hedges' g	0.0	-1.28 to 1.28	No observed mean difference; unadjusted 95% CI included 0	Not applicable - effect size estimated from reported mean +/- SD
Gunathilaka et al. (2021)	Red seabream (Pagrus major)	Shrimp protein hydrolysate or krill meal supplementation in a low-fishmeal diet	SH (20.2% fishmeal + 5% shrimp protein hydrolysate)	HFM (40% fishmeal reference diet)	Nitro blue tetrazolium activity (OD)	1.020 +/- 0.060	0.950 +/- 0.070	3	3	Hedges' g	0.86	-0.55 to 2.27	Higher, more favorable value with supplementation; unadjusted 95% CI included 0	Not applicable - effect size estimated from reported mean +/- SD
Gunathilaka et al. (2021)	Red seabream (Pagrus major)	Shrimp protein hydrolysate or krill meal supplementation in a low-fishmeal diet	SH (20.2% fishmeal + 5% shrimp protein hydrolysate)	HFM (40% fishmeal reference diet)	Myeloperoxidase activity (OD)	1.810 +/- 0.090	1.660 +/- 0.130	3	3	Hedges' g	1.07	-0.41 to 2.55	Higher measured value with supplementation; unadjusted 95% CI included 0	Not applicable - effect size estimated from reported mean +/- SD
Gunathilaka et al. (2021)	Red seabream (Pagrus major)	Shrimp protein hydrolysate or krill meal supplementation in a low-fishmeal diet	SH (20.2% fishmeal + 5% shrimp protein hydrolysate)	HFM (40% fishmeal reference diet)	Superoxide dismutase activity (% inhibition)	81.60 +/- 4.10	74.80 +/- 0.30	3	3	Hedges' g	1.87	0.05 to 3.69	Higher, more favorable value with supplementation; unadjusted 95% CI excluded 0	Not applicable - effect size estimated from reported mean +/- SD
Gunathilaka et al. (2021)	Red seabream (Pagrus major)	Shrimp protein hydrolysate or krill meal supplementation in a low-fishmeal diet	SH (20.2% fishmeal + 5% shrimp protein hydrolysate)	HFM (40% fishmeal reference diet)	Glutathione peroxidase activity (mU/mL)	116.00 +/- 8.80	109.00 +/- 17.00	3	3	Hedges' g	0.41	-0.90 to 1.73	Higher, more favorable value with supplementation; unadjusted 95% CI included 0	Not applicable - effect size estimated from reported mean +/- SD
Gunathilaka et al. (2021)	Red seabream (Pagrus major)	Shrimp protein hydrolysate or krill meal supplementation in a low-fishmeal diet	SH (20.2% fishmeal + 5% shrimp protein hydrolysate)	HFM (40% fishmeal reference diet)	Catalase activity (mU/mL)	1.410 +/- 0.140	1.350 +/- 0.090	3	3	Hedges' g	0.41	-0.90 to 1.72	Higher, more favorable value with supplementation; unadjusted 95% CI included 0	Not applicable - effect size estimated from reported mean +/- SD
Gunathilaka et al. (2021)	Red seabream (Pagrus major)	Shrimp protein hydrolysate or krill meal supplementation in a low-fishmeal diet	SH (20.2% fishmeal + 5% shrimp protein hydrolysate)	HFM (40% fishmeal reference diet)	Hematocrit (%)	35.80 +/- 1.30	37.60 +/- 4.00	3	3	Hedges' g	-0.48	-1.81 to 0.84	Lower, less favorable value with supplementation; unadjusted 95% CI included 0	Not applicable - effect size estimated from reported mean +/- SD
Gunathilaka et al. (2021)	Red seabream (Pagrus major)	Shrimp protein hydrolysate or krill meal supplementation in a low-fishmeal diet	SH (20.2% fishmeal + 5% shrimp protein hydrolysate)	HFM (40% fishmeal reference diet)	Hemoglobin (g/dL)	6.580 +/- 0.270	5.890 +/- 0.200	3	3	Hedges' g	2.32	0.27 to 4.38	Higher, more favorable value with supplementation; unadjusted 95% CI excluded 0	Not applicable - effect size estimated from reported mean +/- SD
Gunathilaka et al. (2021)	Red seabream (Pagrus major)	Shrimp protein hydrolysate or krill meal supplementation in a low-fishmeal diet	SH (20.2% fishmeal + 5% shrimp protein hydrolysate)	HFM (40% fishmeal reference diet)	Total protein (g/dL)	1.940 +/- 0.090	1.870 +/- 0.150	3	3	Hedges' g	0.45	-0.87 to 1.77	Higher measured value with supplementation; unadjusted 95% CI included 0	Not applicable - effect size estimated from reported mean +/- SD
Gunathilaka et al. (2021)	Red seabream (Pagrus major)	Shrimp protein hydrolysate or krill meal supplementation in a low-fishmeal diet	SH (20.2% fishmeal + 5% shrimp protein hydrolysate)	HFM (40% fishmeal reference diet)	Glucose (mg/dL)	57.60 +/- 3.00	57.60 +/- 1.00	3	3	Hedges' g	0.0	-1.28 to 1.28	No observed mean difference; unadjusted 95% CI included 0	Not applicable - effect size estimated from reported mean +/- SD
Gunathilaka et al. (2021)	Red seabream (Pagrus major)	Shrimp protein hydrolysate or krill meal supplementation in a low-fishmeal diet	SH (20.2% fishmeal + 5% shrimp protein hydrolysate)	HFM (40% fishmeal reference diet)	Total cholesterol (mg/dL)	231.00 +/- 13.00	220.00 +/- 12.00	3	3	Hedges' g	0.7	-0.67 to 2.07	Higher measured value with supplementation; unadjusted 95% CI included 0	Not applicable - effect size estimated from reported mean +/- SD
Gunathilaka et al. (2021)	Red seabream (Pagrus major)	Shrimp protein hydrolysate or krill meal supplementation in a low-fishmeal diet	SH (20.2% fishmeal + 5% shrimp protein hydrolysate)	HFM (40% fishmeal reference diet)	Dry matter (%)	33.10 +/- 1.59	30.00 +/- 0.41	3	3	Hedges' g	2.14	0.18 to 4.09	Higher measured value with supplementation; unadjusted 95% CI excluded 0	Not applicable - effect size estimated from reported mean +/- SD
Gunathilaka et al. (2021)	Red seabream (Pagrus major)	Shrimp protein hydrolysate or krill meal supplementation in a low-fishmeal diet	SH (20.2% fishmeal + 5% shrimp protein hydrolysate)	HFM (40% fishmeal reference diet)	Crude protein (% dry matter)	54.00 +/- 0.20	53.60 +/- 0.40	3	3	Hedges' g	1.01	-0.45 to 2.47	Higher measured value with supplementation; unadjusted 95% CI included 0	Not applicable - effect size estimated from reported mean +/- SD
Gunathilaka et al. (2021)	Red seabream (Pagrus major)	Shrimp protein hydrolysate or krill meal supplementation in a low-fishmeal diet	SH (20.2% fishmeal + 5% shrimp protein hydrolysate)	HFM (40% fishmeal reference diet)	Crude lipid (% dry matter)	33.80 +/- 0.80	34.10 +/- 2.50	3	3	Hedges' g	-0.13	-1.41 to 1.15	Lower measured value with supplementation; unadjusted 95% CI included 0	Not applicable - effect size estimated from reported mean +/- SD
Gunathilaka et al. (2021)	Red seabream (Pagrus major)	Shrimp protein hydrolysate or krill meal supplementation in a low-fishmeal diet	SH (20.2% fishmeal + 5% shrimp protein hydrolysate)	HFM (40% fishmeal reference diet)	Ash (% dry matter)	13.80 +/- 0.80	13.90 +/- 1.00	3	3	Hedges' g	-0.09	-1.37 to 1.19	Lower measured value with supplementation; unadjusted 95% CI included 0	Not applicable - effect size estimated from reported mean +/- SD
Gunathilaka et al. (2021)	Red seabream (Pagrus major)	Shrimp protein hydrolysate or krill meal supplementation in a low-fishmeal diet	KM (20% fishmeal + 5% krill meal)	LFM (25% fishmeal + 21% soy protein concentrate)	Apparent digestibility coefficient of protein (%)	Not reported numerically	Not reported numerically	3	3	Not estimable	Not estimable	Not estimable	Figure 1 shows the direction and Tukey lettering, but an exact standardized effect was not calculated	Exact group means and SDs were available only graphically
Gunathilaka et al. (2021)	Red seabream (Pagrus major)	Shrimp protein hydrolysate or krill meal supplementation in a low-fishmeal diet	KM (20% fishmeal + 5% krill meal)	LFM (25% fishmeal + 21% soy protein concentrate)	Apparent digestibility coefficient of dry matter (%)	Not reported numerically	Not reported numerically	3	3	Not estimable	Not estimable	Not estimable	Figure 1 shows the direction and Tukey lettering, but an exact standardized effect was not calculated	Exact group means and SDs were available only graphically
Gunathilaka et al. (2021)	Red seabream (Pagrus major)	Shrimp protein hydrolysate or krill meal supplementation in a low-fishmeal diet	SH (20.2% fishmeal + 5% shrimp protein hydrolysate)	LFM (25% fishmeal + 21% soy protein concentrate)	Apparent digestibility coefficient of protein (%)	Not reported numerically	Not reported numerically	3	3	Not estimable	Not estimable	Not estimable	Figure 1 shows the direction and Tukey lettering, but an exact standardized effect was not calculated	Exact group means and SDs were available only graphically
Gunathilaka et al. (2021)	Red seabream (Pagrus major)	Shrimp protein hydrolysate or krill meal supplementation in a low-fishmeal diet	SH (20.2% fishmeal + 5% shrimp protein hydrolysate)	LFM (25% fishmeal + 21% soy protein concentrate)	Apparent digestibility coefficient of dry matter (%)	Not reported numerically	Not reported numerically	3	3	Not estimable	Not estimable	Not estimable	Figure 1 shows the direction and Tukey lettering, but an exact standardized effect was not calculated	Exact group means and SDs were available only graphically
Gunathilaka et al. (2021)	Red seabream (Pagrus major)	Shrimp protein hydrolysate or krill meal supplementation in a low-fishmeal diet	KM (20% fishmeal + 5% krill meal)	HFM (40% fishmeal reference diet)	Apparent digestibility coefficient of protein (%)	Not reported numerically	Not reported numerically	3	3	Not estimable	Not estimable	Not estimable	Figure 1 shows the direction and Tukey lettering, but an exact standardized effect was not calculated	Exact group means and SDs were available only graphically
Gunathilaka et al. (2021)	Red seabream (Pagrus major)	Shrimp protein hydrolysate or krill meal supplementation in a low-fishmeal diet	KM (20% fishmeal + 5% krill meal)	HFM (40% fishmeal reference diet)	Apparent digestibility coefficient of dry matter (%)	Not reported numerically	Not reported numerically	3	3	Not estimable	Not estimable	Not estimable	Figure 1 shows the direction and Tukey lettering, but an exact standardized effect was not calculated	Exact group means and SDs were available only graphically
Gunathilaka et al. (2021)	Red seabream (Pagrus major)	Shrimp protein hydrolysate or krill meal supplementation in a low-fishmeal diet	SH (20.2% fishmeal + 5% shrimp protein hydrolysate)	HFM (40% fishmeal reference diet)	Apparent digestibility coefficient of protein (%)	Not reported numerically	Not reported numerically	3	3	Not estimable	Not estimable	Not estimable	Figure 1 shows the direction and Tukey lettering, but an exact standardized effect was not calculated	Exact group means and SDs were available only graphically

Study	Species	Strategy	Intervention	Comparator	Outcome and unit	Intervention mean +/- SD	Comparator mean +/- SD	n intervention (tanks)	n comparator (tanks)	Effect-size metric	Effect size	95% CI	Interpretation	Reason not estimated
Gunathilaka et al. (2021)	Red seabream (Pagrus major)	Shrimp protein hydrolysate or krill meal supplementation in a low-fishmeal diet	SH (20.2% fishmeal + 5% shrimp protein hydrolysate)	HFM (40% fishmeal reference diet)	Apparent digestibility coefficient of dry matter (%)	Not reported numerically	Not reported numerically	3	3	Not estimable	Not estimable	Not estimable	Figure 1 shows the direction and Tukey lettering, but an exact standardized effect was not calculated	Exact group means and SDs were available only graphically

Note: Values in Tables 3-5 are mean +/- SD from three independent tanks per diet. KM and SH were each compared with the LFM basal diet and with the HFM reference diet. FCR was direction-reversed because lower values are favorable. Protein and dry-matter digestibility outcomes in Figure 1 were marked not estimable because exact group means and SDs were not printed. Pairwise 95% CIs are descriptive and unadjusted.

Source: Gunathilaka et al. (2021), https://doi.org/10.47853/FAS.2021.e11

Study 17. Khosravi et al. (2018): shrimp, tilapia, and krill protein hydrolysate supplementation in juvenile olive flounder

Study	Species	Strategy	Intervention	Comparator	Outcome and unit	Intervention mean +/- SD	Comparator mean +/- SD	n intervention (tanks)	n comparator (tanks)	Effect-size metric	Effect size	95% CI	Interpretation	Reason not estimated
Khosravi et al. (2018)	Olive flounder (Paralichthys olivaceus)	Shrimp, tilapia, or krill protein hydrolysate supplementation in a low-fishmeal, high-SPC diet	SH (24.2% fishmeal + 3.34% shrimp hydrolysate)	LFM (27.5% fishmeal + 22% soy protein concentrate)	Final body weight (g)	117.00 +/- 6.10	99.00 +/- 6.80	3	3	Hedges' g	2.23	0.22 to 4.24	Higher, more favorable value with supplementation; unadjusted 95% CI excluded 0	Not applicable - effect size estimated from reported mean +/- SD
Khosravi et al. (2018)	Olive flounder (Paralichthys olivaceus)	Shrimp, tilapia, or krill protein hydrolysate supplementation in a low-fishmeal, high-SPC diet	SH (24.2% fishmeal + 3.34% shrimp hydrolysate)	LFM (27.5% fishmeal + 22% soy protein concentrate)	Weight gain (%)	673.00 +/- 43.40	552.00 +/- 43.30	3	3	Hedges' g	2.23	0.22 to 4.24	Higher, more favorable value with supplementation; unadjusted 95% CI excluded 0	Not applicable - effect size estimated from reported mean +/- SD
Khosravi et al. (2018)	Olive flounder (Paralichthys olivaceus)	Shrimp, tilapia, or krill protein hydrolysate supplementation in a low-fishmeal, high-SPC diet	SH (24.2% fishmeal + 3.34% shrimp hydrolysate)	LFM (27.5% fishmeal + 22% soy protein concentrate)	Specific growth rate (%/day)	2.840 +/- 0.080	2.600 +/- 0.090	3	3	Hedges' g	2.25	0.23 to 4.28	Higher, more favorable value with supplementation; unadjusted 95% CI excluded 0	Not applicable - effect size estimated from reported mean +/- SD
Khosravi et al. (2018)	Olive flounder (Paralichthys olivaceus)	Shrimp, tilapia, or krill protein hydrolysate supplementation in a low-fishmeal, high-SPC diet	SH (24.2% fishmeal + 3.34% shrimp hydrolysate)	LFM (27.5% fishmeal + 22% soy protein concentrate)	Feed intake (g/fish)	107.00 +/- 1.00	96.00 +/- 3.60	3	3	Hedges' g	3.33	0.69 to 5.97	Higher measured value with supplementation; unadjusted 95% CI excluded 0	Not applicable - effect size estimated from reported mean +/- SD
Khosravi et al. (2018)	Olive flounder (Paralichthys olivaceus)	Shrimp, tilapia, or krill protein hydrolysate supplementation in a low-fishmeal, high-SPC diet	SH (24.2% fishmeal + 3.34% shrimp hydrolysate)	LFM (27.5% fishmeal + 22% soy protein concentrate)	Feed conversion ratio	1.140 +/- 0.050	1.270 +/- 0.060	3	3	Hedges' g (direction reversed)	1.88	0.06 to 3.71	Lower, more favorable value with supplementation; unadjusted 95% CI excluded 0	Not applicable - effect size estimated from reported mean +/- SD
Khosravi et al. (2018)	Olive flounder (Paralichthys olivaceus)	Shrimp, tilapia, or krill protein hydrolysate supplementation in a low-fishmeal, high-SPC diet	SH (24.2% fishmeal + 3.34% shrimp hydrolysate)	LFM (27.5% fishmeal + 22% soy protein concentrate)	Protein efficiency ratio	1.730 +/- 0.080	1.560 +/- 0.080	3	3	Hedges' g	1.7	-0.04 to 3.44	Higher, more favorable value with supplementation; unadjusted 95% CI included 0	Not applicable - effect size estimated from reported mean +/- SD
Khosravi et al. (2018)	Olive flounder (Paralichthys olivaceus)	Shrimp, tilapia, or krill protein hydrolysate supplementation in a low-fishmeal, high-SPC diet	SH (24.2% fishmeal + 3.34% shrimp hydrolysate)	LFM (27.5% fishmeal + 22% soy protein concentrate)	Survival rate (%)	91.40 +/- 6.70	89.20 +/- 1.90	3	3	Hedges' g	0.36	-0.95 to 1.66	Higher, more favorable value with supplementation; unadjusted 95% CI included 0	Not applicable - effect size estimated from reported mean +/- SD
Khosravi et al. (2018)	Olive flounder (Paralichthys olivaceus)	Shrimp, tilapia, or krill protein hydrolysate supplementation in a low-fishmeal, high-SPC diet	SH (24.2% fishmeal + 3.34% shrimp hydrolysate)	LFM (27.5% fishmeal + 22% soy protein concentrate)	Apparent digestibility coefficient of dry matter (%)	77.50 +/- 5.60	71.80 +/- 3.60	3	3	Hedges' g	0.97	-0.48 to 2.41	Higher, more favorable value with supplementation; unadjusted 95% CI included 0	Not applicable - effect size estimated from reported mean +/- SD
Khosravi et al. (2018)	Olive flounder (Paralichthys olivaceus)	Shrimp, tilapia, or krill protein hydrolysate supplementation in a low-fishmeal, high-SPC diet	SH (24.2% fishmeal + 3.34% shrimp hydrolysate)	LFM (27.5% fishmeal + 22% soy protein concentrate)	Apparent digestibility coefficient of protein (%)	93.20 +/- 1.50	88.00 +/- 1.50	3	3	Hedges' g	2.77	0.46 to 5.08	Higher, more favorable value with supplementation; unadjusted 95% CI excluded 0	Not applicable - effect size estimated from reported mean +/- SD
Khosravi et al. (2018)	Olive flounder (Paralichthys olivaceus)	Shrimp, tilapia, or krill protein hydrolysate supplementation in a low-fishmeal, high-SPC diet	SH (24.2% fishmeal + 3.34% shrimp hydrolysate)	LFM (27.5% fishmeal + 22% soy protein concentrate)	Hematocrit (%)	29.30 +/- 2.08	30.80 +/- 1.70	3	3	Hedges' g	-0.63	-1.98 to 0.72	Lower, less favorable value with supplementation; unadjusted 95% CI included 0	Not applicable - effect size estimated from reported mean +/- SD
Khosravi et al. (2018)	Olive flounder (Paralichthys olivaceus)	Shrimp, tilapia, or krill protein hydrolysate supplementation in a low-fishmeal, high-SPC diet	SH (24.2% fishmeal + 3.34% shrimp hydrolysate)	LFM (27.5% fishmeal + 22% soy protein concentrate)	Hemoglobin (g/dL)	5.070 +/- 0.370	5.360 +/- 0.090	3	3	Hedges' g	-0.86	-2.27 to 0.55	Lower, less favorable value with supplementation; unadjusted 95% CI included 0	Not applicable - effect size estimated from reported mean +/- SD
Khosravi et al. (2018)	Olive flounder (Paralichthys olivaceus)	Shrimp, tilapia, or krill protein hydrolysate supplementation in a low-fishmeal, high-SPC diet	SH (24.2% fishmeal + 3.34% shrimp hydrolysate)	LFM (27.5% fishmeal + 22% soy protein concentrate)	Glucose (mg/dL)	26.80 +/- 3.40	29.30 +/- 5.10	3	3	Hedges' g	-0.46	-1.78 to 0.86	Lower measured value with supplementation; unadjusted 95% CI included 0	Not applicable - effect size estimated from reported mean +/- SD
Khosravi et al. (2018)	Olive flounder (Paralichthys olivaceus)	Shrimp, tilapia, or krill protein hydrolysate supplementation in a low-fishmeal, high-SPC diet	SH (24.2% fishmeal + 3.34% shrimp hydrolysate)	LFM (27.5% fishmeal + 22% soy protein concentrate)	Triglyceride (mg/dL)	346.00 +/- 59.00	399.00 +/- 44.00	3	3	Hedges' g	-0.81	-2.21 to 0.58	Lower measured value with supplementation; unadjusted 95% CI included 0	Not applicable - effect size estimated from reported mean +/- SD
Khosravi et al. (2018)	Olive flounder (Paralichthys olivaceus)	Shrimp, tilapia, or krill protein hydrolysate supplementation in a low-fishmeal, high-SPC diet	SH (24.2% fishmeal + 3.34% shrimp hydrolysate)	LFM (27.5% fishmeal + 22% soy protein concentrate)	Total protein (g/dL)	4.040 +/- 0.120	4.020 +/- 0.120	3	3	Hedges' g	0.13	-1.15 to 1.42	Higher measured value with supplementation; unadjusted 95% CI included 0	Not applicable - effect size estimated from reported mean +/- SD
Khosravi et al. (2018)	Olive flounder (Paralichthys olivaceus)	Shrimp, tilapia, or krill protein hydrolysate supplementation in a low-fishmeal, high-SPC diet	SH (24.2% fishmeal + 3.34% shrimp hydrolysate)	LFM (27.5% fishmeal + 22% soy protein concentrate)	Total cholesterol (mg/dL)	262.00 +/- 48.00	302.00 +/- 35.00	3	3	Hedges' g	-0.76	-2.15 to 0.62	Lower measured value with supplementation; unadjusted 95% CI included 0	Not applicable - effect size estimated from reported mean +/- SD
Khosravi et al. (2018)	Olive flounder (Paralichthys olivaceus)	Shrimp, tilapia, or krill protein hydrolysate supplementation in a low-fishmeal, high-SPC diet	SH (24.2% fishmeal + 3.34% shrimp hydrolysate)	LFM (27.5% fishmeal + 22% soy protein concentrate)	Aspartate aminotransferase activity (U/L)	30.90 +/- 7.10	35.20 +/- 6.70	3	3	Hedges' g	-0.5	-1.82 to 0.83	Lower measured value with supplementation; unadjusted 95% CI included 0	Not applicable - effect size estimated from reported mean +/- SD
Khosravi et al. (2018)	Olive flounder (Paralichthys olivaceus)	Shrimp, tilapia, or krill protein hydrolysate supplementation in a low-fishmeal, high-SPC diet	SH (24.2% fishmeal + 3.34% shrimp hydrolysate)	LFM (27.5% fishmeal + 22% soy protein concentrate)	Alanine aminotransferase activity (U/L)	6.630 +/- 0.200	7.000 +/- 0.380	3	3	Hedges' g	-0.97	-2.42 to 0.47	Lower measured value with supplementation; unadjusted 95% CI included 0	Not applicable - effect size estimated from reported mean +/- SD
Khosravi et al. (2018)	Olive flounder (Paralichthys olivaceus)	Shrimp, tilapia, or krill protein hydrolysate supplementation in a low-fishmeal, high-SPC diet	SH (24.2% fishmeal + 3.34% shrimp hydrolysate)	LFM (27.5% fishmeal + 22% soy protein concentrate)	Moisture (% wet basis)	71.10 +/- 0.30	71.10 +/- 0.20	3	3	Hedges' g	0.0	-1.28 to 1.28	No observed mean difference; unadjusted 95% CI included 0	Not applicable - effect size estimated from reported mean +/- SD
Khosravi et al. (2018)	Olive flounder (Paralichthys olivaceus)	Shrimp, tilapia, or krill protein hydrolysate supplementation in a low-fishmeal, high-SPC diet	SH (24.2% fishmeal + 3.34% shrimp hydrolysate)	LFM (27.5% fishmeal + 22% soy protein concentrate)	Crude protein (% wet basis)	18.80 +/- 0.40	18.70 +/- 0.10	3	3	Hedges' g	0.27	-1.02 to 1.57	Higher measured value with supplementation; unadjusted 95% CI included 0	Not applicable - effect size estimated from reported mean +/- SD
Khosravi et al. (2018)	Olive flounder (Paralichthys olivaceus)	Shrimp, tilapia, or krill protein hydrolysate supplementation in a low-fishmeal, high-SPC diet	SH (24.2% fishmeal + 3.34% shrimp hydrolysate)	LFM (27.5% fishmeal + 22% soy protein concentrate)	Crude lipid (% wet basis)	6.360 +/- 0.290	5.580 +/- 0.260	3	3	Hedges' g	2.27	0.24 to 4.29	Higher measured value with supplementation; unadjusted 95% CI excluded 0	Not applicable - effect size estimated from reported mean +/- SD
Khosravi et al. (2018)	Olive flounder (Paralichthys olivaceus)	Shrimp, tilapia, or krill protein hydrolysate supplementation in a low-fishmeal, high-SPC diet	SH (24.2% fishmeal + 3.34% shrimp hydrolysate)	LFM (27.5% fishmeal + 22% soy protein concentrate)	Ash (% wet basis)	3.340 +/- 0.190	3.410 +/- 0.070	3	3	Hedges' g	-0.39	-1.70 to 0.92	Lower measured value with supplementation; unadjusted 95% CI included 0	Not applicable - effect size estimated from reported mean +/- SD
Khosravi et al. (2018)	Olive flounder (Paralichthys olivaceus)	Shrimp, tilapia, or krill protein hydrolysate supplementation in a low-fishmeal, high-SPC diet	SH (24.2% fishmeal + 3.34% shrimp hydrolysate)	LFM (27.5% fishmeal + 22% soy protein concentrate)	Goblet cell count (cells)	1235.00 +/- 115.00	935.00 +/- 127.00	3	3	Hedges' g	1.98	0.10 to 3.86	Higher, more favorable value with supplementation; unadjusted 95% CI excluded 0	Not applicable - effect size estimated from reported mean +/- SD
Khosravi et al. (2018)	Olive flounder (Paralichthys olivaceus)	Shrimp, tilapia, or krill protein hydrolysate supplementation in a low-fishmeal, high-SPC diet	SH (24.2% fishmeal + 3.34% shrimp hydrolysate)	LFM (27.5% fishmeal + 22% soy protein concentrate)	Villus height (µm)	508.00 +/- 28.00	398.00 +/- 33.00	3	3	Hedges' g	2.88	0.51 to 5.24	Higher, more favorable value with supplementation; unadjusted 95% CI excluded 0	Not applicable - effect size estimated from reported mean +/- SD
Khosravi et al. (2018)	Olive flounder (Paralichthys olivaceus)	Shrimp, tilapia, or krill protein hydrolysate supplementation in a low-fishmeal, high-SPC diet	SH (24.2% fishmeal + 3.34% shrimp hydrolysate)	LFM (27.5% fishmeal + 22% soy protein concentrate)	Enterocyte height (µm)	36.40 +/- 0.80	30.40 +/- 2.50	3	3	Hedges' g	2.59	0.38 to 4.79	Higher, more favorable value with supplementation; unadjusted 95% CI excluded 0	Not applicable - effect size estimated from reported mean +/- SD
Khosravi et al. (2018)	Olive flounder (Paralichthys olivaceus)	Shrimp, tilapia, or krill protein hydrolysate supplementation in a low-fishmeal, high-SPC diet	SH (24.2% fishmeal + 3.34% shrimp hydrolysate)	LFM (27.5% fishmeal + 22% soy protein concentrate)	Nitroblue tetrazolium activity (OD)	0.750 +/- 0.020	0.690 +/- 0.020	3	3	Hedges' g	2.4	0.30 to 4.50	Higher, more favorable value with supplementation; unadjusted 95% CI excluded 0	Not applicable - effect size estimated from reported mean +/- SD
Khosravi et al. (2018)	Olive flounder (Paralichthys olivaceus)	Shrimp, tilapia, or krill protein hydrolysate supplementation in a low-fishmeal, high-SPC diet	SH (24.2% fishmeal + 3.34% shrimp hydrolysate)	LFM (27.5% fishmeal + 22% soy protein concentrate)	Total immunoglobulin (mg/mL)	23.20 +/- 0.50	18.50 +/- 0.80	3	3	Hedges' g	5.64	1.53 to 9.75	Higher, more favorable value with supplementation; unadjusted 95% CI excluded 0	Not applicable - effect size estimated from reported mean +/- SD
Khosravi et al. (2018)	Olive flounder (Paralichthys olivaceus)	Shrimp, tilapia, or krill protein hydrolysate supplementation in a low-fishmeal, high-SPC diet	SH (24.2% fishmeal + 3.34% shrimp hydrolysate)	LFM (27.5% fishmeal + 22% soy protein concentrate)	Lysozyme activity (µg/mL)	28.10 +/- 1.20	21.20 +/- 1.60	3	3	Hedges' g	3.9	0.91 to 6.90	Higher, more favorable value with supplementation; unadjusted 95% CI excluded 0	Not applicable - effect size estimated from reported mean +/- SD
Khosravi et al. (2018)	Olive flounder (Paralichthys olivaceus)	Shrimp, tilapia, or krill protein hydrolysate supplementation in a low-fishmeal, high-SPC diet	SH (24.2% fishmeal + 3.34% shrimp hydrolysate)	LFM (27.5% fishmeal + 22% soy protein concentrate)	Myeloperoxidase level	2.080 +/- 0.070	1.980 +/- 0.110	3	3	Hedges' g	0.87	-0.55 to 2.28	Higher measured value with supplementation; unadjusted 95% CI included 0	Not applicable - effect size estimated from reported mean +/- SD
Khosravi et al. (2018)	Olive flounder (Paralichthys olivaceus)	Shrimp, tilapia, or krill protein hydrolysate supplementation in a low-fishmeal, high-SPC diet	SH (24.2% fishmeal + 3.34% shrimp hydrolysate)	LFM (27.5% fishmeal + 22% soy protein concentrate)	Antiprotease activity (% inhibition)	33.40 +/- 3.60	32.40 +/- 2.50	3	3	Hedges' g	0.26	-1.03 to 1.55	Higher measured value with supplementation; unadjusted 95% CI included 0	Not applicable - effect size estimated from reported mean +/- SD
Khosravi et al. (2018)	Olive flounder (Paralichthys olivaceus)	Shrimp, tilapia, or krill protein hydrolysate supplementation in a low-fishmeal, high-SPC diet	SH (24.2% fishmeal + 3.34% shrimp hydrolysate)	LFM (27.5% fishmeal + 22% soy protein concentrate)	Superoxide dismutase activity (% inhibition)	78.70 +/- 3.70	67.70 +/- 1.70	3	3	Hedges' g	3.06	0.58 to 5.53	Higher, more favorable value with supplementation; unadjusted 95% CI excluded 0	Not applicable - effect size estimated from reported mean +/- SD
Khosravi et al. (2018)	Olive flounder (Paralichthys olivaceus)	Shrimp, tilapia, or krill protein hydrolysate supplementation in a low-fishmeal, high-SPC diet	SH (24.2% fishmeal + 3.34% shrimp hydrolysate)	LFM (27.5% fishmeal + 22% soy protein concentrate)	Glutathione peroxidase activity (mU/mL)	15.40 +/- 0.60	14.60 +/- 0.60	3	3	Hedges' g	1.07	-0.41 to 2.54	Higher, more favorable value with supplementation; unadjusted 95% CI included 0	Not applicable - effect size estimated from reported mean +/- SD

Study	Species	Strategy	Intervention	Comparator	Outcome and unit	Intervention mean +/- SD	Comparator mean +/- SD	n intervention (tanks)	n comparator (tanks)	Effect-size metric	Effect size	95% CI	Interpretation	Reason not estimated
Khosravi et al. (2018)	Olive flounder (Paralichthys olivaceus)	Shrimp, tilapia, or krill protein hydrolysate supplementation in a low-fishmeal, high-SPC diet	TH (24.1% fishmeal + 2.88% tilapia hydrolysate)	LFM (27.5% fishmeal + 22% soy protein concentrate)	Final body weight (g)	120.00 +/- 3.40	99.00 +/- 6.80	3	3	Hedges' g	3.13	0.61 to 5.64	Higher, more favorable value with supplementation; unadjusted 95% CI excluded 0	Not applicable - effect size estimated from reported mean +/- SD
Khosravi et al. (2018)	Olive flounder (Paralichthys olivaceus)	Shrimp, tilapia, or krill protein hydrolysate supplementation in a low-fishmeal, high-SPC diet	TH (24.1% fishmeal + 2.88% tilapia hydrolysate)	LFM (27.5% fishmeal + 22% soy protein concentrate)	Weight gain (%)	693.00 +/- 21.80	552.00 +/- 43.30	3	3	Hedges' g	3.29	0.68 to 5.91	Higher, more favorable value with supplementation; unadjusted 95% CI excluded 0	Not applicable - effect size estimated from reported mean +/- SD
Khosravi et al. (2018)	Olive flounder (Paralichthys olivaceus)	Shrimp, tilapia, or krill protein hydrolysate supplementation in a low-fishmeal, high-SPC diet	TH (24.1% fishmeal + 2.88% tilapia hydrolysate)	LFM (27.5% fishmeal + 22% soy protein concentrate)	Specific growth rate (%/day)	2.880 +/- 0.040	2.600 +/- 0.090	3	3	Hedges' g	3.22	0.65 to 5.79	Higher, more favorable value with supplementation; unadjusted 95% CI excluded 0	Not applicable - effect size estimated from reported mean +/- SD
Khosravi et al. (2018)	Olive flounder (Paralichthys olivaceus)	Shrimp, tilapia, or krill protein hydrolysate supplementation in a low-fishmeal, high-SPC diet	TH (24.1% fishmeal + 2.88% tilapia hydrolysate)	LFM (27.5% fishmeal + 22% soy protein concentrate)	Feed intake (g/fish)	107.00 +/- 1.80	96.00 +/- 3.60	3	3	Hedges' g	3.09	0.60 to 5.59	Higher measured value with supplementation; unadjusted 95% CI excluded 0	Not applicable - effect size estimated from reported mean +/- SD
Khosravi et al. (2018)	Olive flounder (Paralichthys olivaceus)	Shrimp, tilapia, or krill protein hydrolysate supplementation in a low-fishmeal, high-SPC diet	TH (24.1% fishmeal + 2.88% tilapia hydrolysate)	LFM (27.5% fishmeal + 22% soy protein concentrate)	Feed conversion ratio	1.110 +/- 0.050	1.270 +/- 0.060	3	3	Hedges' g (direction reversed)	2.32	0.26 to 4.37	Lower, more favorable value with supplementation; unadjusted 95% CI excluded 0	Not applicable - effect size estimated from reported mean +/- SD
Khosravi et al. (2018)	Olive flounder (Paralichthys olivaceus)	Shrimp, tilapia, or krill protein hydrolysate supplementation in a low-fishmeal, high-SPC diet	TH (24.1% fishmeal + 2.88% tilapia hydrolysate)	LFM (27.5% fishmeal + 22% soy protein concentrate)	Protein efficiency ratio	1.780 +/- 0.080	1.560 +/- 0.080	3	3	Hedges' g	2.2	0.21 to 4.19	Higher, more favorable value with supplementation; unadjusted 95% CI excluded 0	Not applicable - effect size estimated from reported mean +/- SD
Khosravi et al. (2018)	Olive flounder (Paralichthys olivaceus)	Shrimp, tilapia, or krill protein hydrolysate supplementation in a low-fishmeal, high-SPC diet	TH (24.1% fishmeal + 2.88% tilapia hydrolysate)	LFM (27.5% fishmeal + 22% soy protein concentrate)	Survival rate (%)	92.50 +/- 4.90	89.20 +/- 1.90	3	3	Hedges' g	0.71	-0.66 to 2.08	Higher, more favorable value with supplementation; unadjusted 95% CI included 0	Not applicable - effect size estimated from reported mean +/- SD
Khosravi et al. (2018)	Olive flounder (Paralichthys olivaceus)	Shrimp, tilapia, or krill protein hydrolysate supplementation in a low-fishmeal, high-SPC diet	TH (24.1% fishmeal + 2.88% tilapia hydrolysate)	LFM (27.5% fishmeal + 22% soy protein concentrate)	Apparent digestibility coefficient of dry matter (%)	81.90 +/- 2.70	71.80 +/- 3.60	3	3	Hedges' g	2.54	0.36 to 4.72	Higher, more favorable value with supplementation; unadjusted 95% CI excluded 0	Not applicable - effect size estimated from reported mean +/- SD
Khosravi et al. (2018)	Olive flounder (Paralichthys olivaceus)	Shrimp, tilapia, or krill protein hydrolysate supplementation in a low-fishmeal, high-SPC diet	TH (24.1% fishmeal + 2.88% tilapia hydrolysate)	LFM (27.5% fishmeal + 22% soy protein concentrate)	Apparent digestibility coefficient of protein (%)	94.70 +/- 0.80	88.00 +/- 1.50	3	3	Hedges' g	4.46	1.11 to 7.80	Higher, more favorable value with supplementation; unadjusted 95% CI excluded 0	Not applicable - effect size estimated from reported mean +/- SD
Khosravi et al. (2018)	Olive flounder (Paralichthys olivaceus)	Shrimp, tilapia, or krill protein hydrolysate supplementation in a low-fishmeal, high-SPC diet	TH (24.1% fishmeal + 2.88% tilapia hydrolysate)	LFM (27.5% fishmeal + 22% soy protein concentrate)	Hematocrit (%)	29.60 +/- 2.30	30.80 +/- 1.70	3	3	Hedges' g	-0.47	-1.80 to 0.85	Lower, less favorable value with supplementation; unadjusted 95% CI included 0	Not applicable - effect size estimated from reported mean +/- SD
Khosravi et al. (2018)	Olive flounder (Paralichthys olivaceus)	Shrimp, tilapia, or krill protein hydrolysate supplementation in a low-fishmeal, high-SPC diet	TH (24.1% fishmeal + 2.88% tilapia hydrolysate)	LFM (27.5% fishmeal + 22% soy protein concentrate)	Hemoglobin (g/dL)	5.260 +/- 0.450	5.360 +/- 0.090	3	3	Hedges' g	-0.25	-1.54 to 1.05	Lower, less favorable value with supplementation; unadjusted 95% CI included 0	Not applicable - effect size estimated from reported mean +/- SD
Khosravi et al. (2018)	Olive flounder (Paralichthys olivaceus)	Shrimp, tilapia, or krill protein hydrolysate supplementation in a low-fishmeal, high-SPC diet	TH (24.1% fishmeal + 2.88% tilapia hydrolysate)	LFM (27.5% fishmeal + 22% soy protein concentrate)	Glucose (mg/dL)	26.40 +/- 2.70	29.30 +/- 5.10	3	3	Hedges' g	-0.57	-1.91 to 0.77	Lower measured value with supplementation; unadjusted 95% CI included 0	Not applicable - effect size estimated from reported mean +/- SD
Khosravi et al. (2018)	Olive flounder (Paralichthys olivaceus)	Shrimp, tilapia, or krill protein hydrolysate supplementation in a low-fishmeal, high-SPC diet	TH (24.1% fishmeal + 2.88% tilapia hydrolysate)	LFM (27.5% fishmeal + 22% soy protein concentrate)	Triglyceride (mg/dL)	379.00 +/- 53.00	399.00 +/- 44.00	3	3	Hedges' g	-0.33	-1.63 to 0.97	Lower measured value with supplementation; unadjusted 95% CI included 0	Not applicable - effect size estimated from reported mean +/- SD
Khosravi et al. (2018)	Olive flounder (Paralichthys olivaceus)	Shrimp, tilapia, or krill protein hydrolysate supplementation in a low-fishmeal, high-SPC diet	TH (24.1% fishmeal + 2.88% tilapia hydrolysate)	LFM (27.5% fishmeal + 22% soy protein concentrate)	Total protein (g/dL)	4.290 +/- 0.670	4.020 +/- 0.120	3	3	Hedges' g	0.45	-0.87 to 1.77	Higher measured value with supplementation; unadjusted 95% CI included 0	Not applicable - effect size estimated from reported mean +/- SD
Khosravi et al. (2018)	Olive flounder (Paralichthys olivaceus)	Shrimp, tilapia, or krill protein hydrolysate supplementation in a low-fishmeal, high-SPC diet	TH (24.1% fishmeal + 2.88% tilapia hydrolysate)	LFM (27.5% fishmeal + 22% soy protein concentrate)	Total cholesterol (mg/dL)	296.00 +/- 60.00	302.00 +/- 35.00	3	3	Hedges' g	-0.1	-1.38 to 1.18	Lower measured value with supplementation; unadjusted 95% CI included 0	Not applicable - effect size estimated from reported mean +/- SD
Khosravi et al. (2018)	Olive flounder (Paralichthys olivaceus)	Shrimp, tilapia, or krill protein hydrolysate supplementation in a low-fishmeal, high-SPC diet	TH (24.1% fishmeal + 2.88% tilapia hydrolysate)	LFM (27.5% fishmeal + 22% soy protein concentrate)	Aspartate aminotransferase activity (U/L)	33.20 +/- 5.10	35.20 +/- 6.70	3	3	Hedges' g	-0.27	-1.56 to 1.03	Lower measured value with supplementation; unadjusted 95% CI included 0	Not applicable - effect size estimated from reported mean +/- SD
Khosravi et al. (2018)	Olive flounder (Paralichthys olivaceus)	Shrimp, tilapia, or krill protein hydrolysate supplementation in a low-fishmeal, high-SPC diet	TH (24.1% fishmeal + 2.88% tilapia hydrolysate)	LFM (27.5% fishmeal + 22% soy protein concentrate)	Alanine aminotransferase activity (U/L)	6.360 +/- 0.390	7.000 +/- 0.380	3	3	Hedges' g	-1.33	-2.91 to 0.25	Lower measured value with supplementation; unadjusted 95% CI included 0	Not applicable - effect size estimated from reported mean +/- SD
Khosravi et al. (2018)	Olive flounder (Paralichthys olivaceus)	Shrimp, tilapia, or krill protein hydrolysate supplementation in a low-fishmeal, high-SPC diet	TH (24.1% fishmeal + 2.88% tilapia hydrolysate)	LFM (27.5% fishmeal + 22% soy protein concentrate)	Moisture (% wet basis)	70.70 +/- 0.70	71.10 +/- 0.20	3	3	Hedges' g	-0.62	-1.97 to 0.73	Lower measured value with supplementation; unadjusted 95% CI included 0	Not applicable - effect size estimated from reported mean +/- SD
Khosravi et al. (2018)	Olive flounder (Paralichthys olivaceus)	Shrimp, tilapia, or krill protein hydrolysate supplementation in a low-fishmeal, high-SPC diet	TH (24.1% fishmeal + 2.88% tilapia hydrolysate)	LFM (27.5% fishmeal + 22% soy protein concentrate)	Crude protein (% wet basis)	18.60 +/- 0.20	18.70 +/- 0.10	3	3	Hedges' g	-0.51	-1.83 to 0.82	Lower measured value with supplementation; unadjusted 95% CI included 0	Not applicable - effect size estimated from reported mean +/- SD
Khosravi et al. (2018)	Olive flounder (Paralichthys olivaceus)	Shrimp, tilapia, or krill protein hydrolysate supplementation in a low-fishmeal, high-SPC diet	TH (24.1% fishmeal + 2.88% tilapia hydrolysate)	LFM (27.5% fishmeal + 22% soy protein concentrate)	Crude lipid (% wet basis)	6.390 +/- 0.400	5.580 +/- 0.260	3	3	Hedges' g	1.92	0.07 to 3.77	Higher measured value with supplementation; unadjusted 95% CI excluded 0	Not applicable - effect size estimated from reported mean +/- SD
Khosravi et al. (2018)	Olive flounder (Paralichthys olivaceus)	Shrimp, tilapia, or krill protein hydrolysate supplementation in a low-fishmeal, high-SPC diet	TH (24.1% fishmeal + 2.88% tilapia hydrolysate)	LFM (27.5% fishmeal + 22% soy protein concentrate)	Ash (% wet basis)	3.280 +/- 0.210	3.410 +/- 0.070	3	3	Hedges' g	-0.66	-2.02 to 0.70	Lower measured value with supplementation; unadjusted 95% CI included 0	Not applicable - effect size estimated from reported mean +/- SD
Khosravi et al. (2018)	Olive flounder (Paralichthys olivaceus)	Shrimp, tilapia, or krill protein hydrolysate supplementation in a low-fishmeal, high-SPC diet	TH (24.1% fishmeal + 2.88% tilapia hydrolysate)	LFM (27.5% fishmeal + 22% soy protein concentrate)	Goblet cell count (cells)	1223.00 +/- 186.00	935.00 +/- 127.00	3	3	Hedges' g	1.45	-0.18 to 3.07	Higher, more favorable value with supplementation; unadjusted 95% CI included 0	Not applicable - effect size estimated from reported mean +/- SD
Khosravi et al. (2018)	Olive flounder (Paralichthys olivaceus)	Shrimp, tilapia, or krill protein hydrolysate supplementation in a low-fishmeal, high-SPC diet	TH (24.1% fishmeal + 2.88% tilapia hydrolysate)	LFM (27.5% fishmeal + 22% soy protein concentrate)	Villus height (µm)	502.00 +/- 40.00	398.00 +/- 33.00	3	3	Hedges' g	2.27	0.24 to 4.30	Higher, more favorable value with supplementation; unadjusted 95% CI excluded 0	Not applicable - effect size estimated from reported mean +/- SD
Khosravi et al. (2018)	Olive flounder (Paralichthys olivaceus)	Shrimp, tilapia, or krill protein hydrolysate supplementation in a low-fishmeal, high-SPC diet	TH (24.1% fishmeal + 2.88% tilapia hydrolysate)	LFM (27.5% fishmeal + 22% soy protein concentrate)	Enterocyte height (µm)	35.80 +/- 0.40	30.40 +/- 2.50	3	3	Hedges' g	2.41	0.31 to 4.52	Higher, more favorable value with supplementation; unadjusted 95% CI excluded 0	Not applicable - effect size estimated from reported mean +/- SD
Khosravi et al. (2018)	Olive flounder (Paralichthys olivaceus)	Shrimp, tilapia, or krill protein hydrolysate supplementation in a low-fishmeal, high-SPC diet	TH (24.1% fishmeal + 2.88% tilapia hydrolysate)	LFM (27.5% fishmeal + 22% soy protein concentrate)	Nitroblue tetrazolium activity (OD)	0.800 +/- 0.050	0.690 +/- 0.020	3	3	Hedges' g	2.31	0.26 to 4.36	Higher, more favorable value with supplementation; unadjusted 95% CI excluded 0	Not applicable - effect size estimated from reported mean +/- SD
Khosravi et al. (2018)	Olive flounder (Paralichthys olivaceus)	Shrimp, tilapia, or krill protein hydrolysate supplementation in a low-fishmeal, high-SPC diet	TH (24.1% fishmeal + 2.88% tilapia hydrolysate)	LFM (27.5% fishmeal + 22% soy protein concentrate)	Total immunoglobulin (mg/mL)	21.90 +/- 1.60	18.50 +/- 0.80	3	3	Hedges' g	2.15	0.19 to 4.11	Higher, more favorable value with supplementation; unadjusted 95% CI excluded 0	Not applicable - effect size estimated from reported mean +/- SD
Khosravi et al. (2018)	Olive flounder (Paralichthys olivaceus)	Shrimp, tilapia, or krill protein hydrolysate supplementation in a low-fishmeal, high-SPC diet	TH (24.1% fishmeal + 2.88% tilapia hydrolysate)	LFM (27.5% fishmeal + 22% soy protein concentrate)	Lysozyme activity (µg/mL)	26.40 +/- 1.30	21.20 +/- 1.60	3	3	Hedges' g	2.85	0.50 to 5.21	Higher, more favorable value with supplementation; unadjusted 95% CI excluded 0	Not applicable - effect size estimated from reported mean +/- SD
Khosravi et al. (2018)	Olive flounder (Paralichthys olivaceus)	Shrimp, tilapia, or krill protein hydrolysate supplementation in a low-fishmeal, high-SPC diet	TH (24.1% fishmeal + 2.88% tilapia hydrolysate)	LFM (27.5% fishmeal + 22% soy protein concentrate)	Myeloperoxidase level	2.080 +/- 0.220	1.980 +/- 0.110	3	3	Hedges' g	0.46	-0.86 to 1.78	Higher measured value with supplementation; unadjusted 95% CI included 0	Not applicable - effect size estimated from reported mean +/- SD
Khosravi et al. (2018)	Olive flounder (Paralichthys olivaceus)	Shrimp, tilapia, or krill protein hydrolysate supplementation in a low-fishmeal, high-SPC diet	TH (24.1% fishmeal + 2.88% tilapia hydrolysate)	LFM (27.5% fishmeal + 22% soy protein concentrate)	Antiprotease activity (% inhibition)	33.30 +/- 1.90	32.40 +/- 2.50	3	3	Hedges' g	0.32	-0.98 to 1.62	Higher measured value with supplementation; unadjusted 95% CI included 0	Not applicable - effect size estimated from reported mean +/- SD
Khosravi et al. (2018)	Olive flounder (Paralichthys olivaceus)	Shrimp, tilapia, or krill protein hydrolysate supplementation in a low-fishmeal, high-SPC diet	TH (24.1% fishmeal + 2.88% tilapia hydrolysate)	LFM (27.5% fishmeal + 22% soy protein concentrate)	Superoxide dismutase activity (% inhibition)	75.20 +/- 2.70	67.70 +/- 1.70	3	3	Hedges' g	2.66	0.42 to 4.90	Higher, more favorable value with supplementation; unadjusted 95% CI excluded 0	Not applicable - effect size estimated from reported mean +/- SD
Khosravi et al. (2018)	Olive flounder (Paralichthys olivaceus)	Shrimp, tilapia, or krill protein hydrolysate supplementation in a low-fishmeal, high-SPC diet	TH (24.1% fishmeal + 2.88% tilapia hydrolysate)	LFM (27.5% fishmeal + 22% soy protein concentrate)	Glutathione peroxidase activity (mU/mL)	16.80 +/- 0.80	14.60 +/- 0.60	3	3	Hedges' g	2.49	0.34 to 4.64	Higher, more favorable value with supplementation; unadjusted 95% CI excluded 0	Not applicable - effect size estimated from reported mean +/- SD
Khosravi et al. (2018)	Olive flounder (Paralichthys olivaceus)	Shrimp, tilapia, or krill protein hydrolysate supplementation in a low-fishmeal, high-SPC diet	KH (24.1% fishmeal + 3.12% krill hydrolysate)	LFM (27.5% fishmeal + 22% soy protein concentrate)	Final body weight (g)	127.00 +/- 8.10	99.00 +/- 6.80	3	3	Hedges' g	3.0	0.56 to 5.43	Higher, more favorable value with supplementation; unadjusted 95% CI excluded 0	Not applicable - effect size estimated from reported mean +/- SD

Study	Species	Strategy	Intervention	Comparator	Outcome and unit	Intervention mean +/- SD	Comparator mean +/- SD	n intervention (tanks)	n comparator (tanks)	Effect-size metric	Effect size	95% CI	Interpretation	Reason not estimated
Khosravi et al. (2018)	Olive flounder (Paralichthys olivaceus)	Shrimp, tilapia, or krill protein hydrolysate supplementation in a low-fishmeal, high-SPC diet	KH (24.1% fishmeal + 3.12% krill hydrolysate)	LFM (27.5% fishmeal + 22% soy protein concentrate)	Weight gain (%)	742.00 +/- 50.60	552.00 +/- 43.30	3	3	Hedges' g	3.23	0.65 to 5.80	Higher, more favorable value with supplementation; unadjusted 95% CI excluded 0	Not applicable - effect size estimated from reported mean +/- SD
Khosravi et al. (2018)	Olive flounder (Paralichthys olivaceus)	Shrimp, tilapia, or krill protein hydrolysate supplementation in a low-fishmeal, high-SPC diet	KH (24.1% fishmeal + 3.12% krill hydrolysate)	LFM (27.5% fishmeal + 22% soy protein concentrate)	Specific growth rate (%/day)	2.960 +/- 0.080	2.600 +/- 0.090	3	3	Hedges' g	3.38	0.71 to 6.05	Higher, more favorable value with supplementation; unadjusted 95% CI excluded 0	Not applicable - effect size estimated from reported mean +/- SD
Khosravi et al. (2018)	Olive flounder (Paralichthys olivaceus)	Shrimp, tilapia, or krill protein hydrolysate supplementation in a low-fishmeal, high-SPC diet	KH (24.1% fishmeal + 3.12% krill hydrolysate)	LFM (27.5% fishmeal + 22% soy protein concentrate)	Feed intake (g/fish)	111.00 +/- 1.20	96.00 +/- 3.60	3	3	Hedges' g	4.47	1.12 to 7.83	Higher measured value with supplementation; unadjusted 95% CI excluded 0	Not applicable - effect size estimated from reported mean +/- SD
Khosravi et al. (2018)	Olive flounder (Paralichthys olivaceus)	Shrimp, tilapia, or krill protein hydrolysate supplementation in a low-fishmeal, high-SPC diet	KH (24.1% fishmeal + 3.12% krill hydrolysate)	LFM (27.5% fishmeal + 22% soy protein concentrate)	Feed conversion ratio	1.070 +/- 0.060	1.270 +/- 0.060	3	3	Hedges' g (direction reversed)	2.67	0.42 to 4.91	Lower, more favorable value with supplementation; unadjusted 95% CI excluded 0	Not applicable - effect size estimated from reported mean +/- SD
Khosravi et al. (2018)	Olive flounder (Paralichthys olivaceus)	Shrimp, tilapia, or krill protein hydrolysate supplementation in a low-fishmeal, high-SPC diet	KH (24.1% fishmeal + 3.12% krill hydrolysate)	LFM (27.5% fishmeal + 22% soy protein concentrate)	Protein efficiency ratio	1.830 +/- 0.100	1.560 +/- 0.080	3	3	Hedges' g	2.39	0.29 to 4.48	Higher, more favorable value with supplementation; unadjusted 95% CI excluded 0	Not applicable - effect size estimated from reported mean +/- SD
Khosravi et al. (2018)	Olive flounder (Paralichthys olivaceus)	Shrimp, tilapia, or krill protein hydrolysate supplementation in a low-fishmeal, high-SPC diet	KH (24.1% fishmeal + 3.12% krill hydrolysate)	LFM (27.5% fishmeal + 22% soy protein concentrate)	Survival rate (%)	91.40 +/- 4.90	89.20 +/- 1.90	3	3	Hedges' g	0.47	-0.85 to 1.80	Higher, more favorable value with supplementation; unadjusted 95% CI included 0	Not applicable - effect size estimated from reported mean +/- SD
Khosravi et al. (2018)	Olive flounder (Paralichthys olivaceus)	Shrimp, tilapia, or krill protein hydrolysate supplementation in a low-fishmeal, high-SPC diet	KH (24.1% fishmeal + 3.12% krill hydrolysate)	LFM (27.5% fishmeal + 22% soy protein concentrate)	Apparent digestibility coefficient of dry matter (%)	82.70 +/- 0.80	71.80 +/- 3.60	3	3	Hedges' g	3.34	0.70 to 5.99	Higher, more favorable value with supplementation; unadjusted 95% CI excluded 0	Not applicable - effect size estimated from reported mean +/- SD
Khosravi et al. (2018)	Olive flounder (Paralichthys olivaceus)	Shrimp, tilapia, or krill protein hydrolysate supplementation in a low-fishmeal, high-SPC diet	KH (24.1% fishmeal + 3.12% krill hydrolysate)	LFM (27.5% fishmeal + 22% soy protein concentrate)	Apparent digestibility coefficient of protein (%)	94.20 +/- 0.40	88.00 +/- 1.50	3	3	Hedges' g	4.52	1.14 to 7.90	Higher, more favorable value with supplementation; unadjusted 95% CI excluded 0	Not applicable - effect size estimated from reported mean +/- SD
Khosravi et al. (2018)	Olive flounder (Paralichthys olivaceus)	Shrimp, tilapia, or krill protein hydrolysate supplementation in a low-fishmeal, high-SPC diet	KH (24.1% fishmeal + 3.12% krill hydrolysate)	LFM (27.5% fishmeal + 22% soy protein concentrate)	Hematocrit (%)	30.40 +/- 2.20	30.80 +/- 1.70	3	3	Hedges' g	-0.16	-1.45 to 1.12	Lower, less favorable value with supplementation; unadjusted 95% CI included 0	Not applicable - effect size estimated from reported mean +/- SD
Khosravi et al. (2018)	Olive flounder (Paralichthys olivaceus)	Shrimp, tilapia, or krill protein hydrolysate supplementation in a low-fishmeal, high-SPC diet	KH (24.1% fishmeal + 3.12% krill hydrolysate)	LFM (27.5% fishmeal + 22% soy protein concentrate)	Hemoglobin (g/dL)	5.670 +/- 0.610	5.360 +/- 0.090	3	3	Hedges' g	0.57	-0.77 to 1.91	Higher, more favorable value with supplementation; unadjusted 95% CI included 0	Not applicable - effect size estimated from reported mean +/- SD
Khosravi et al. (2018)	Olive flounder (Paralichthys olivaceus)	Shrimp, tilapia, or krill protein hydrolysate supplementation in a low-fishmeal, high-SPC diet	KH (24.1% fishmeal + 3.12% krill hydrolysate)	LFM (27.5% fishmeal + 22% soy protein concentrate)	Glucose (mg/dL)	28.30 +/- 2.40	29.30 +/- 5.10	3	3	Hedges' g	-0.2	-1.49 to 1.09	Lower measured value with supplementation; unadjusted 95% CI included 0	Not applicable - effect size estimated from reported mean +/- SD
Khosravi et al. (2018)	Olive flounder (Paralichthys olivaceus)	Shrimp, tilapia, or krill protein hydrolysate supplementation in a low-fishmeal, high-SPC diet	KH (24.1% fishmeal + 3.12% krill hydrolysate)	LFM (27.5% fishmeal + 22% soy protein concentrate)	Triglyceride (mg/dL)	381.00 +/- 48.00	399.00 +/- 44.00	3	3	Hedges' g	-0.31	-1.61 to 0.99	Lower measured value with supplementation; unadjusted 95% CI included 0	Not applicable - effect size estimated from reported mean +/- SD
Khosravi et al. (2018)	Olive flounder (Paralichthys olivaceus)	Shrimp, tilapia, or krill protein hydrolysate supplementation in a low-fishmeal, high-SPC diet	KH (24.1% fishmeal + 3.12% krill hydrolysate)	LFM (27.5% fishmeal + 22% soy protein concentrate)	Total protein (g/dL)	4.300 +/- 0.470	4.020 +/- 0.120	3	3	Hedges' g	0.65	-0.70 to 2.01	Higher measured value with supplementation; unadjusted 95% CI included 0	Not applicable - effect size estimated from reported mean +/- SD
Khosravi et al. (2018)	Olive flounder (Paralichthys olivaceus)	Shrimp, tilapia, or krill protein hydrolysate supplementation in a low-fishmeal, high-SPC diet	KH (24.1% fishmeal + 3.12% krill hydrolysate)	LFM (27.5% fishmeal + 22% soy protein concentrate)	Total cholesterol (mg/dL)	284.00 +/- 19.00	302.00 +/- 35.00	3	3	Hedges' g	-0.51	-1.84 to 0.82	Lower measured value with supplementation; unadjusted 95% CI included 0	Not applicable - effect size estimated from reported mean +/- SD
Khosravi et al. (2018)	Olive flounder (Paralichthys olivaceus)	Shrimp, tilapia, or krill protein hydrolysate supplementation in a low-fishmeal, high-SPC diet	KH (24.1% fishmeal + 3.12% krill hydrolysate)	LFM (27.5% fishmeal + 22% soy protein concentrate)	Aspartate aminotransferase activity (U/L)	28.80 +/- 5.90	35.20 +/- 6.70	3	3	Hedges' g	-0.81	-2.21 to 0.59	Lower measured value with supplementation; unadjusted 95% CI included 0	Not applicable - effect size estimated from reported mean +/- SD
Khosravi et al. (2018)	Olive flounder (Paralichthys olivaceus)	Shrimp, tilapia, or krill protein hydrolysate supplementation in a low-fishmeal, high-SPC diet	KH (24.1% fishmeal + 3.12% krill hydrolysate)	LFM (27.5% fishmeal + 22% soy protein concentrate)	Alanine aminotransferase activity (U/L)	5.870 +/- 0.260	7.000 +/- 0.380	3	3	Hedges' g	-2.78	-5.09 to -0.47	Lower measured value with supplementation; unadjusted 95% CI excluded 0	Not applicable - effect size estimated from reported mean +/- SD
Khosravi et al. (2018)	Olive flounder (Paralichthys olivaceus)	Shrimp, tilapia, or krill protein hydrolysate supplementation in a low-fishmeal, high-SPC diet	KH (24.1% fishmeal + 3.12% krill hydrolysate)	LFM (27.5% fishmeal + 22% soy protein concentrate)	Moisture (% wet basis)	71.20 +/- 0.40	71.10 +/- 0.20	3	3	Hedges' g	0.25	-1.04 to 1.55	Higher measured value with supplementation; unadjusted 95% CI included 0	Not applicable - effect size estimated from reported mean +/- SD
Khosravi et al. (2018)	Olive flounder (Paralichthys olivaceus)	Shrimp, tilapia, or krill protein hydrolysate supplementation in a low-fishmeal, high-SPC diet	KH (24.1% fishmeal + 3.12% krill hydrolysate)	LFM (27.5% fishmeal + 22% soy protein concentrate)	Crude protein (% wet basis)	18.70 +/- 0.10	18.70 +/- 0.10	3	3	Hedges' g	0.0	-1.28 to 1.28	No observed mean difference; unadjusted 95% CI included 0	Not applicable - effect size estimated from reported mean +/- SD
Khosravi et al. (2018)	Olive flounder (Paralichthys olivaceus)	Shrimp, tilapia, or krill protein hydrolysate supplementation in a low-fishmeal, high-SPC diet	KH (24.1% fishmeal + 3.12% krill hydrolysate)	LFM (27.5% fishmeal + 22% soy protein concentrate)	Crude lipid (% wet basis)	6.060 +/- 0.130	5.580 +/- 0.260	3	3	Hedges' g	1.87	0.05 to 3.69	Higher measured value with supplementation; unadjusted 95% CI excluded 0	Not applicable - effect size estimated from reported mean +/- SD
Khosravi et al. (2018)	Olive flounder (Paralichthys olivaceus)	Shrimp, tilapia, or krill protein hydrolysate supplementation in a low-fishmeal, high-SPC diet	KH (24.1% fishmeal + 3.12% krill hydrolysate)	LFM (27.5% fishmeal + 22% soy protein concentrate)	Ash (% wet basis)	3.280 +/- 0.050	3.410 +/- 0.070	3	3	Hedges' g	-1.71	-3.45 to 0.03	Lower measured value with supplementation; unadjusted 95% CI included 0	Not applicable - effect size estimated from reported mean +/- SD
Khosravi et al. (2018)	Olive flounder (Paralichthys olivaceus)	Shrimp, tilapia, or krill protein hydrolysate supplementation in a low-fishmeal, high-SPC diet	KH (24.1% fishmeal + 3.12% krill hydrolysate)	LFM (27.5% fishmeal + 22% soy protein concentrate)	Goblet cell count (cells)	1392.00 +/- 111.00	935.00 +/- 127.00	3	3	Hedges' g	3.07	0.59 to 5.55	Higher, more favorable value with supplementation; unadjusted 95% CI excluded 0	Not applicable - effect size estimated from reported mean +/- SD
Khosravi et al. (2018)	Olive flounder (Paralichthys olivaceus)	Shrimp, tilapia, or krill protein hydrolysate supplementation in a low-fishmeal, high-SPC diet	KH (24.1% fishmeal + 3.12% krill hydrolysate)	LFM (27.5% fishmeal + 22% soy protein concentrate)	Villus height (µm)	502.00 +/- 12.00	398.00 +/- 33.00	3	3	Hedges' g	3.35	0.70 to 6.00	Higher, more favorable value with supplementation; unadjusted 95% CI excluded 0	Not applicable - effect size estimated from reported mean +/- SD
Khosravi et al. (2018)	Olive flounder (Paralichthys olivaceus)	Shrimp, tilapia, or krill protein hydrolysate supplementation in a low-fishmeal, high-SPC diet	KH (24.1% fishmeal + 3.12% krill hydrolysate)	LFM (27.5% fishmeal + 22% soy protein concentrate)	Enterocyte height (µm)	36.30 +/- 0.60	30.40 +/- 2.50	3	3	Hedges' g	2.6	0.39 to 4.80	Higher, more favorable value with supplementation; unadjusted 95% CI excluded 0	Not applicable - effect size estimated from reported mean +/- SD
Khosravi et al. (2018)	Olive flounder (Paralichthys olivaceus)	Shrimp, tilapia, or krill protein hydrolysate supplementation in a low-fishmeal, high-SPC diet	KH (24.1% fishmeal + 3.12% krill hydrolysate)	LFM (27.5% fishmeal + 22% soy protein concentrate)	Nitroblue tetrazolium activity (OD)	0.800 +/- 0.030	0.690 +/- 0.020	3	3	Hedges' g	3.45	0.74 to 6.16	Higher, more favorable value with supplementation; unadjusted 95% CI excluded 0	Not applicable - effect size estimated from reported mean +/- SD
Khosravi et al. (2018)	Olive flounder (Paralichthys olivaceus)	Shrimp, tilapia, or krill protein hydrolysate supplementation in a low-fishmeal, high-SPC diet	KH (24.1% fishmeal + 3.12% krill hydrolysate)	LFM (27.5% fishmeal + 22% soy protein concentrate)	Total immunoglobulin (mg/mL)	24.60 +/- 2.60	18.50 +/- 0.80	3	3	Hedges' g	2.54	0.36 to 4.71	Higher, more favorable value with supplementation; unadjusted 95% CI excluded 0	Not applicable - effect size estimated from reported mean +/- SD
Khosravi et al. (2018)	Olive flounder (Paralichthys olivaceus)	Shrimp, tilapia, or krill protein hydrolysate supplementation in a low-fishmeal, high-SPC diet	KH (24.1% fishmeal + 3.12% krill hydrolysate)	LFM (27.5% fishmeal + 22% soy protein concentrate)	Lysozyme activity (µg/mL)	27.30 +/- 1.10	21.20 +/- 1.60	3	3	Hedges' g	3.55	0.78 to 6.33	Higher, more favorable value with supplementation; unadjusted 95% CI excluded 0	Not applicable - effect size estimated from reported mean +/- SD
Khosravi et al. (2018)	Olive flounder (Paralichthys olivaceus)	Shrimp, tilapia, or krill protein hydrolysate supplementation in a low-fishmeal, high-SPC diet	KH (24.1% fishmeal + 3.12% krill hydrolysate)	LFM (27.5% fishmeal + 22% soy protein concentrate)	Myeloperoxidase level	2.260 +/- 0.210	1.980 +/- 0.110	3	3	Hedges' g	1.34	-0.24 to 2.92	Higher measured value with supplementation; unadjusted 95% CI included 0	Not applicable - effect size estimated from reported mean +/- SD
Khosravi et al. (2018)	Olive flounder (Paralichthys olivaceus)	Shrimp, tilapia, or krill protein hydrolysate supplementation in a low-fishmeal, high-SPC diet	KH (24.1% fishmeal + 3.12% krill hydrolysate)	LFM (27.5% fishmeal + 22% soy protein concentrate)	Antiprotease activity (% inhibition)	35.80 +/- 0.30	32.40 +/- 2.50	3	3	Hedges' g	1.53	-0.13 to 3.19	Higher measured value with supplementation; unadjusted 95% CI included 0	Not applicable - effect size estimated from reported mean +/- SD
Khosravi et al. (2018)	Olive flounder (Paralichthys olivaceus)	Shrimp, tilapia, or krill protein hydrolysate supplementation in a low-fishmeal, high-SPC diet	KH (24.1% fishmeal + 3.12% krill hydrolysate)	LFM (27.5% fishmeal + 22% soy protein concentrate)	Superoxide dismutase activity (% inhibition)	72.10 +/- 3.60	67.70 +/- 1.70	3	3	Hedges' g	1.25	-0.30 to 2.80	Higher, more favorable value with supplementation; unadjusted 95% CI included 0	Not applicable - effect size estimated from reported mean +/- SD
Khosravi et al. (2018)	Olive flounder (Paralichthys olivaceus)	Shrimp, tilapia, or krill protein hydrolysate supplementation in a low-fishmeal, high-SPC diet	KH (24.1% fishmeal + 3.12% krill hydrolysate)	LFM (27.5% fishmeal + 22% soy protein concentrate)	Glutathione peroxidase activity (mU/mL)	15.80 +/- 1.60	14.60 +/- 0.60	3	3	Hedges' g	0.79	-0.60 to 2.19	Higher, more favorable value with supplementation; unadjusted 95% CI included 0	Not applicable - effect size estimated from reported mean +/- SD
Khosravi et al. (2018)	Olive flounder (Paralichthys olivaceus)	Shrimp, tilapia, or krill protein hydrolysate supplementation in a low-fishmeal, high-SPC diet	SH (24.2% fishmeal + 3.34% shrimp hydrolysate)	HFM (55% fishmeal reference diet)	Final body weight (g)	117.00 +/- 6.10	121.00 +/- 0.60	3	3	Hedges' g	-0.74	-2.12 to 0.64	Lower, less favorable value with supplementation; unadjusted 95% CI included 0	Not applicable - effect size estimated from reported mean +/- SD
Khosravi et al. (2018)	Olive flounder (Paralichthys olivaceus)	Shrimp, tilapia, or krill protein hydrolysate supplementation in a low-fishmeal, high-SPC diet	SH (24.2% fishmeal + 3.34% shrimp hydrolysate)	HFM (55% fishmeal reference diet)	Weight gain (%)	673.00 +/- 43.40	699.00 +/- 7.30	3	3	Hedges' g	-0.67	-2.03 to 0.69	Lower, less favorable value with supplementation; unadjusted 95% CI included 0	Not applicable - effect size estimated from reported mean +/- SD

Study	Species	Strategy	Intervention	Comparator	Outcome and unit	Intervention mean +/- SD	Comparator mean +/- SD	n intervention (tanks)	n comparator (tanks)	Effect-size metric	Effect size	95% CI	Interpretation	Reason not estimated
Khosravi et al. (2018)	Olive flounder (Paralichthys olivaceus)	Shrimp, tilapia, or krill protein hydrolysate supplementation in a low-fishmeal, high-SPC diet	SH (24.2% fishmeal + 3.34% shrimp hydrolysate)	HFM (55% fishmeal reference diet)	Specific growth rate (%/day)	2.840 +/- 0.080	2.890 +/- 0.010	3	3	Hedges' g	-0.7	-2.07 to 0.67	Lower, less favorable value with supplementation; unadjusted 95% CI included 0	Not applicable - effect size estimated from reported mean +/- SD
Khosravi et al. (2018)	Olive flounder (Paralichthys olivaceus)	Shrimp, tilapia, or krill protein hydrolysate supplementation in a low-fishmeal, high-SPC diet	SH (24.2% fishmeal + 3.34% shrimp hydrolysate)	HFM (55% fishmeal reference diet)	Feed intake (g/fish)	107.00 +/- 1.00	113.00 +/- 1.20	3	3	Hedges' g	-4.35	-7.62 to -1.07	Lower measured value with supplementation; unadjusted 95% CI excluded 0	Not applicable - effect size estimated from reported mean +/- SD
Khosravi et al. (2018)	Olive flounder (Paralichthys olivaceus)	Shrimp, tilapia, or krill protein hydrolysate supplementation in a low-fishmeal, high-SPC diet	SH (24.2% fishmeal + 3.34% shrimp hydrolysate)	HFM (55% fishmeal reference diet)	Feed conversion ratio	1.140 +/- 0.050	1.180 +/- 0.030	3	3	Hedges' g (direction reversed)	0.78	-0.61 to 2.16	Lower, more favorable value with supplementation; unadjusted 95% CI included 0	Not applicable - effect size estimated from reported mean +/- SD
Khosravi et al. (2018)	Olive flounder (Paralichthys olivaceus)	Shrimp, tilapia, or krill protein hydrolysate supplementation in a low-fishmeal, high-SPC diet	SH (24.2% fishmeal + 3.34% shrimp hydrolysate)	HFM (55% fishmeal reference diet)	Protein efficiency ratio	1.730 +/- 0.080	1.660 +/- 0.040	3	3	Hedges' g	0.89	-0.53 to 2.31	Higher, more favorable value with supplementation; unadjusted 95% CI included 0	Not applicable - effect size estimated from reported mean +/- SD
Khosravi et al. (2018)	Olive flounder (Paralichthys olivaceus)	Shrimp, tilapia, or krill protein hydrolysate supplementation in a low-fishmeal, high-SPC diet	SH (24.2% fishmeal + 3.34% shrimp hydrolysate)	HFM (55% fishmeal reference diet)	Survival rate (%)	91.40 +/- 6.70	83.90 +/- 3.20	3	3	Hedges' g	1.14	-0.36 to 2.65	Higher, more favorable value with supplementation; unadjusted 95% CI included 0	Not applicable - effect size estimated from reported mean +/- SD
Khosravi et al. (2018)	Olive flounder (Paralichthys olivaceus)	Shrimp, tilapia, or krill protein hydrolysate supplementation in a low-fishmeal, high-SPC diet	SH (24.2% fishmeal + 3.34% shrimp hydrolysate)	HFM (55% fishmeal reference diet)	Apparent digestibility coefficient of dry matter (%)	77.50 +/- 5.60	81.20 +/- 0.80	3	3	Hedges' g	-0.74	-2.12 to 0.64	Lower, less favorable value with supplementation; unadjusted 95% CI included 0	Not applicable - effect size estimated from reported mean +/- SD
Khosravi et al. (2018)	Olive flounder (Paralichthys olivaceus)	Shrimp, tilapia, or krill protein hydrolysate supplementation in a low-fishmeal, high-SPC diet	SH (24.2% fishmeal + 3.34% shrimp hydrolysate)	HFM (55% fishmeal reference diet)	Apparent digestibility coefficient of protein (%)	93.20 +/- 1.50	94.40 +/- 0.50	3	3	Hedges' g	-0.86	-2.27 to 0.55	Lower, less favorable value with supplementation; unadjusted 95% CI included 0	Not applicable - effect size estimated from reported mean +/- SD
Khosravi et al. (2018)	Olive flounder (Paralichthys olivaceus)	Shrimp, tilapia, or krill protein hydrolysate supplementation in a low-fishmeal, high-SPC diet	SH (24.2% fishmeal + 3.34% shrimp hydrolysate)	HFM (55% fishmeal reference diet)	Hematocrit (%)	29.30 +/- 2.08	32.20 +/- 3.30	3	3	Hedges' g	-0.84	-2.25 to 0.57	Lower, less favorable value with supplementation; unadjusted 95% CI included 0	Not applicable - effect size estimated from reported mean +/- SD
Khosravi et al. (2018)	Olive flounder (Paralichthys olivaceus)	Shrimp, tilapia, or krill protein hydrolysate supplementation in a low-fishmeal, high-SPC diet	SH (24.2% fishmeal + 3.34% shrimp hydrolysate)	HFM (55% fishmeal reference diet)	Hemoglobin (g/dL)	5.070 +/- 0.370	5.590 +/- 0.130	3	3	Hedges' g	-1.5	-3.15 to 0.15	Lower, less favorable value with supplementation; unadjusted 95% CI included 0	Not applicable - effect size estimated from reported mean +/- SD
Khosravi et al. (2018)	Olive flounder (Paralichthys olivaceus)	Shrimp, tilapia, or krill protein hydrolysate supplementation in a low-fishmeal, high-SPC diet	SH (24.2% fishmeal + 3.34% shrimp hydrolysate)	HFM (55% fishmeal reference diet)	Glucose (mg/dL)	26.80 +/- 3.40	28.90 +/- 3.30	3	3	Hedges' g	-0.5	-1.83 to 0.83	Lower measured value with supplementation; unadjusted 95% CI included 0	Not applicable - effect size estimated from reported mean +/- SD
Khosravi et al. (2018)	Olive flounder (Paralichthys olivaceus)	Shrimp, tilapia, or krill protein hydrolysate supplementation in a low-fishmeal, high-SPC diet	SH (24.2% fishmeal + 3.34% shrimp hydrolysate)	HFM (55% fishmeal reference diet)	Triglyceride (mg/dL)	346.00 +/- 59.00	378.00 +/- 21.00	3	3	Hedges' g	-0.58	-1.92 to 0.76	Lower measured value with supplementation; unadjusted 95% CI included 0	Not applicable - effect size estimated from reported mean +/- SD
Khosravi et al. (2018)	Olive flounder (Paralichthys olivaceus)	Shrimp, tilapia, or krill protein hydrolysate supplementation in a low-fishmeal, high-SPC diet	SH (24.2% fishmeal + 3.34% shrimp hydrolysate)	HFM (55% fishmeal reference diet)	Total protein (g/dL)	4.040 +/- 0.120	4.020 +/- 0.440	3	3	Hedges' g	0.05	-1.23 to 1.33	Higher measured value with supplementation; unadjusted 95% CI included 0	Not applicable - effect size estimated from reported mean +/- SD
Khosravi et al. (2018)	Olive flounder (Paralichthys olivaceus)	Shrimp, tilapia, or krill protein hydrolysate supplementation in a low-fishmeal, high-SPC diet	SH (24.2% fishmeal + 3.34% shrimp hydrolysate)	HFM (55% fishmeal reference diet)	Total cholesterol (mg/dL)	262.00 +/- 48.00	307.00 +/- 55.00	3	3	Hedges' g	-0.7	-2.07 to 0.67	Lower measured value with supplementation; unadjusted 95% CI included 0	Not applicable - effect size estimated from reported mean +/- SD
Khosravi et al. (2018)	Olive flounder (Paralichthys olivaceus)	Shrimp, tilapia, or krill protein hydrolysate supplementation in a low-fishmeal, high-SPC diet	SH (24.2% fishmeal + 3.34% shrimp hydrolysate)	HFM (55% fishmeal reference diet)	Aspartate aminotransferase activity (U/L)	30.90 +/- 7.10	32.00 +/- 1.70	3	3	Hedges' g	-0.17	-1.46 to 1.12	Lower measured value with supplementation; unadjusted 95% CI included 0	Not applicable - effect size estimated from reported mean +/- SD
Khosravi et al. (2018)	Olive flounder (Paralichthys olivaceus)	Shrimp, tilapia, or krill protein hydrolysate supplementation in a low-fishmeal, high-SPC diet	SH (24.2% fishmeal + 3.34% shrimp hydrolysate)	HFM (55% fishmeal reference diet)	Alanine aminotransferase activity (U/L)	6.630 +/- 0.200	6.240 +/- 0.380	3	3	Hedges' g	1.03	-0.44 to 2.49	Higher measured value with supplementation; unadjusted 95% CI included 0	Not applicable - effect size estimated from reported mean +/- SD
Khosravi et al. (2018)	Olive flounder (Paralichthys olivaceus)	Shrimp, tilapia, or krill protein hydrolysate supplementation in a low-fishmeal, high-SPC diet	SH (24.2% fishmeal + 3.34% shrimp hydrolysate)	HFM (55% fishmeal reference diet)	Moisture (% wet basis)	71.10 +/- 0.30	70.50 +/- 0.40	3	3	Hedges' g	1.36	-0.23 to 2.95	Higher measured value with supplementation; unadjusted 95% CI included 0	Not applicable - effect size estimated from reported mean +/- SD
Khosravi et al. (2018)	Olive flounder (Paralichthys olivaceus)	Shrimp, tilapia, or krill protein hydrolysate supplementation in a low-fishmeal, high-SPC diet	SH (24.2% fishmeal + 3.34% shrimp hydrolysate)	HFM (55% fishmeal reference diet)	Crude protein (% wet basis)	18.80 +/- 0.40	18.80 +/- 0.20	3	3	Hedges' g	0.0	-1.28 to 1.28	No observed mean difference; unadjusted 95% CI included 0	Not applicable - effect size estimated from reported mean +/- SD
Khosravi et al. (2018)	Olive flounder (Paralichthys olivaceus)	Shrimp, tilapia, or krill protein hydrolysate supplementation in a low-fishmeal, high-SPC diet	SH (24.2% fishmeal + 3.34% shrimp hydrolysate)	HFM (55% fishmeal reference diet)	Crude lipid (% wet basis)	6.360 +/- 0.290	6.430 +/- 0.200	3	3	Hedges' g	-0.22	-1.51 to 1.06	Lower measured value with supplementation; unadjusted 95% CI included 0	Not applicable - effect size estimated from reported mean +/- SD
Khosravi et al. (2018)	Olive flounder (Paralichthys olivaceus)	Shrimp, tilapia, or krill protein hydrolysate supplementation in a low-fishmeal, high-SPC diet	SH (24.2% fishmeal + 3.34% shrimp hydrolysate)	HFM (55% fishmeal reference diet)	Ash (% wet basis)	3.340 +/- 0.190	3.450 +/- 0.000	3	3	Hedges' g	-0.66	-2.01 to 0.70	Lower measured value with supplementation; unadjusted 95% CI included 0	Not applicable - effect size estimated from reported mean +/- SD
Khosravi et al. (2018)	Olive flounder (Paralichthys olivaceus)	Shrimp, tilapia, or krill protein hydrolysate supplementation in a low-fishmeal, high-SPC diet	SH (24.2% fishmeal + 3.34% shrimp hydrolysate)	HFM (55% fishmeal reference diet)	Goblet cell count (cells)	1235.00 +/- 115.00	1324.00 +/- 176.00	3	3	Hedges' g	-0.48	-1.80 to 0.84	Lower, less favorable value with supplementation; unadjusted 95% CI included 0	Not applicable - effect size estimated from reported mean +/- SD
Khosravi et al. (2018)	Olive flounder (Paralichthys olivaceus)	Shrimp, tilapia, or krill protein hydrolysate supplementation in a low-fishmeal, high-SPC diet	SH (24.2% fishmeal + 3.34% shrimp hydrolysate)	HFM (55% fishmeal reference diet)	Villus height (µm)	508.00 +/- 28.00	505.00 +/- 45.00	3	3	Hedges' g	0.06	-1.22 to 1.35	Higher, more favorable value with supplementation; unadjusted 95% CI included 0	Not applicable - effect size estimated from reported mean +/- SD
Khosravi et al. (2018)	Olive flounder (Paralichthys olivaceus)	Shrimp, tilapia, or krill protein hydrolysate supplementation in a low-fishmeal, high-SPC diet	SH (24.2% fishmeal + 3.34% shrimp hydrolysate)	HFM (55% fishmeal reference diet)	Enterocyte height (µm)	36.40 +/- 0.80	39.10 +/- 1.80	3	3	Hedges' g	-1.55	-3.22 to 0.12	Lower, less favorable value with supplementation; unadjusted 95% CI included 0	Not applicable - effect size estimated from reported mean +/- SD
Khosravi et al. (2018)	Olive flounder (Paralichthys olivaceus)	Shrimp, tilapia, or krill protein hydrolysate supplementation in a low-fishmeal, high-SPC diet	SH (24.2% fishmeal + 3.34% shrimp hydrolysate)	HFM (55% fishmeal reference diet)	Nitroblue tetrazolium activity (OD)	0.750 +/- 0.020	0.710 +/- 0.050	3	3	Hedges' g	0.84	-0.57 to 2.25	Higher, more favorable value with supplementation; unadjusted 95% CI included 0	Not applicable - effect size estimated from reported mean +/- SD
Khosravi et al. (2018)	Olive flounder (Paralichthys olivaceus)	Shrimp, tilapia, or krill protein hydrolysate supplementation in a low-fishmeal, high-SPC diet	SH (24.2% fishmeal + 3.34% shrimp hydrolysate)	HFM (55% fishmeal reference diet)	Total immunoglobulin (mg/mL)	23.20 +/- 0.50	21.70 +/- 1.10	3	3	Hedges' g	1.4	-0.20 to 3.01	Higher, more favorable value with supplementation; unadjusted 95% CI included 0	Not applicable - effect size estimated from reported mean +/- SD
Khosravi et al. (2018)	Olive flounder (Paralichthys olivaceus)	Shrimp, tilapia, or krill protein hydrolysate supplementation in a low-fishmeal, high-SPC diet	SH (24.2% fishmeal + 3.34% shrimp hydrolysate)	HFM (55% fishmeal reference diet)	Lysozyme activity (µg/mL)	28.10 +/- 1.20	24.50 +/- 2.20	3	3	Hedges' g	1.63	-0.08 to 3.33	Higher, more favorable value with supplementation; unadjusted 95% CI included 0	Not applicable - effect size estimated from reported mean +/- SD
Khosravi et al. (2018)	Olive flounder (Paralichthys olivaceus)	Shrimp, tilapia, or krill protein hydrolysate supplementation in a low-fishmeal, high-SPC diet	SH (24.2% fishmeal + 3.34% shrimp hydrolysate)	HFM (55% fishmeal reference diet)	Myeloperoxidase level	2.080 +/- 0.070	1.950 +/- 0.300	3	3	Hedges' g	0.48	-0.84 to 1.80	Higher measured value with supplementation; unadjusted 95% CI included 0	Not applicable - effect size estimated from reported mean +/- SD
Khosravi et al. (2018)	Olive flounder (Paralichthys olivaceus)	Shrimp, tilapia, or krill protein hydrolysate supplementation in a low-fishmeal, high-SPC diet	SH (24.2% fishmeal + 3.34% shrimp hydrolysate)	HFM (55% fishmeal reference diet)	Antiprotease activity (% inhibition)	33.40 +/- 3.60	32.00 +/- 2.40	3	3	Hedges' g	0.37	-0.94 to 1.67	Higher measured value with supplementation; unadjusted 95% CI included 0	Not applicable - effect size estimated from reported mean +/- SD
Khosravi et al. (2018)	Olive flounder (Paralichthys olivaceus)	Shrimp, tilapia, or krill protein hydrolysate supplementation in a low-fishmeal, high-SPC diet	SH (24.2% fishmeal + 3.34% shrimp hydrolysate)	HFM (55% fishmeal reference diet)	Superoxide dismutase activity (% inhibition)	78.70 +/- 3.70	67.50 +/- 3.30	3	3	Hedges' g	2.56	0.37 to 4.74	Higher, more favorable value with supplementation; unadjusted 95% CI excluded 0	Not applicable - effect size estimated from reported mean +/- SD
Khosravi et al. (2018)	Olive flounder (Paralichthys olivaceus)	Shrimp, tilapia, or krill protein hydrolysate supplementation in a low-fishmeal, high-SPC diet	SH (24.2% fishmeal + 3.34% shrimp hydrolysate)	HFM (55% fishmeal reference diet)	Glutathione peroxidase activity (mU/mL)	15.40 +/- 0.60	16.70 +/- 0.40	3	3	Hedges' g	-2.04	-3.95 to -0.13	Lower, less favorable value with supplementation; unadjusted 95% CI excluded 0	Not applicable - effect size estimated from reported mean +/- SD
Khosravi et al. (2018)	Olive flounder (Paralichthys olivaceus)	Shrimp, tilapia, or krill protein hydrolysate supplementation in a low-fishmeal, high-SPC diet	TH (24.1% fishmeal + 2.88% tilapia hydrolysate)	HFM (55% fishmeal reference diet)	Final body weight (g)	120.00 +/- 3.40	121.00 +/- 0.60	3	3	Hedges' g	-0.33	-1.63 to 0.97	Lower, less favorable value with supplementation; unadjusted 95% CI included 0	Not applicable - effect size estimated from reported mean +/- SD
Khosravi et al. (2018)	Olive flounder (Paralichthys olivaceus)	Shrimp, tilapia, or krill protein hydrolysate supplementation in a low-fishmeal, high-SPC diet	TH (24.1% fishmeal + 2.88% tilapia hydrolysate)	HFM (55% fishmeal reference diet)	Weight gain (%)	693.00 +/- 21.80	699.00 +/- 7.30	3	3	Hedges' g	-0.3	-1.59 to 1.00	Lower, less favorable value with supplementation; unadjusted 95% CI included 0	Not applicable - effect size estimated from reported mean +/- SD
Khosravi et al. (2018)	Olive flounder (Paralichthys olivaceus)	Shrimp, tilapia, or krill protein hydrolysate supplementation in a low-fishmeal, high-SPC diet	TH (24.1% fishmeal + 2.88% tilapia hydrolysate)	HFM (55% fishmeal reference diet)	Specific growth rate (%/day)	2.880 +/- 0.040	2.890 +/- 0.010	3	3	Hedges' g	-0.27	-1.57 to 1.02	Lower, less favorable value with supplementation; unadjusted 95% CI included 0	Not applicable - effect size estimated from reported mean +/- SD

Study	Species	Strategy	Intervention	Comparator	Outcome and unit	Intervention mean +/- SD	Comparator mean +/- SD	n intervention (tanks)	n comparator (tanks)	Effect-size metric	Effect size	95% CI	Interpretation	Reason not estimated
Khosravi et al. (2018)	Olive flounder (Paralichthys olivaceus)	Shrimp, tilapia, or krill protein hydrolysate supplementation in a low-fishmeal, high-SPC diet	TH (24.1% fishmeal + 2.88% tilapia hydrolysate)	HFM (55% fishmeal reference diet)	Feed intake (g/fish)	107.00 +/- 1.80	113.00 +/- 1.20	3	3	Hedges' g	-3.14	-5.66 to -0.61	Lower measured value with supplementation; unadjusted 95% CI excluded 0	Not applicable - effect size estimated from reported mean +/- SD
Khosravi et al. (2018)	Olive flounder (Paralichthys olivaceus)	Shrimp, tilapia, or krill protein hydrolysate supplementation in a low-fishmeal, high-SPC diet	TH (24.1% fishmeal + 2.88% tilapia hydrolysate)	HFM (55% fishmeal reference diet)	Feed conversion ratio	1.110 +/- 0.050	1.180 +/- 0.030	3	3	Hedges' g (direction reversed)	1.36	-0.23 to 2.95	Lower, more favorable value with supplementation; unadjusted 95% CI included 0	Not applicable - effect size estimated from reported mean +/- SD
Khosravi et al. (2018)	Olive flounder (Paralichthys olivaceus)	Shrimp, tilapia, or krill protein hydrolysate supplementation in a low-fishmeal, high-SPC diet	TH (24.1% fishmeal + 2.88% tilapia hydrolysate)	HFM (55% fishmeal reference diet)	Protein efficiency ratio	1.780 +/- 0.080	1.660 +/- 0.040	3	3	Hedges' g	1.52	-0.14 to 3.17	Higher, more favorable value with supplementation; unadjusted 95% CI included 0	Not applicable - effect size estimated from reported mean +/- SD
Khosravi et al. (2018)	Olive flounder (Paralichthys olivaceus)	Shrimp, tilapia, or krill protein hydrolysate supplementation in a low-fishmeal, high-SPC diet	TH (24.1% fishmeal + 2.88% tilapia hydrolysate)	HFM (55% fishmeal reference diet)	Survival rate (%)	92.50 +/- 4.90	83.90 +/- 3.20	3	3	Hedges' g	1.66	-0.06 to 3.38	Higher, more favorable value with supplementation; unadjusted 95% CI included 0	Not applicable - effect size estimated from reported mean +/- SD
Khosravi et al. (2018)	Olive flounder (Paralichthys olivaceus)	Shrimp, tilapia, or krill protein hydrolysate supplementation in a low-fishmeal, high-SPC diet	TH (24.1% fishmeal + 2.88% tilapia hydrolysate)	HFM (55% fishmeal reference diet)	Apparent digestibility coefficient of dry matter (%)	81.90 +/- 2.70	81.20 +/- 0.80	3	3	Hedges' g	0.28	-1.01 to 1.58	Higher, more favorable value with supplementation; unadjusted 95% CI included 0	Not applicable - effect size estimated from reported mean +/- SD
Khosravi et al. (2018)	Olive flounder (Paralichthys olivaceus)	Shrimp, tilapia, or krill protein hydrolysate supplementation in a low-fishmeal, high-SPC diet	TH (24.1% fishmeal + 2.88% tilapia hydrolysate)	HFM (55% fishmeal reference diet)	Apparent digestibility coefficient of protein (%)	94.70 +/- 0.80	94.40 +/- 0.50	3	3	Hedges' g	0.36	-0.94 to 1.66	Higher, more favorable value with supplementation; unadjusted 95% CI included 0	Not applicable - effect size estimated from reported mean +/- SD
Khosravi et al. (2018)	Olive flounder (Paralichthys olivaceus)	Shrimp, tilapia, or krill protein hydrolysate supplementation in a low-fishmeal, high-SPC diet	TH (24.1% fishmeal + 2.88% tilapia hydrolysate)	HFM (55% fishmeal reference diet)	Hematocrit (%)	29.60 +/- 2.30	32.20 +/- 3.30	3	3	Hedges' g	-0.73	-2.11 to 0.65	Lower, less favorable value with supplementation; unadjusted 95% CI included 0	Not applicable - effect size estimated from reported mean +/- SD
Khosravi et al. (2018)	Olive flounder (Paralichthys olivaceus)	Shrimp, tilapia, or krill protein hydrolysate supplementation in a low-fishmeal, high-SPC diet	TH (24.1% fishmeal + 2.88% tilapia hydrolysate)	HFM (55% fishmeal reference diet)	Hemoglobin (g/dL)	5.260 +/- 0.450	5.590 +/- 0.130	3	3	Hedges' g	-0.8	-2.19 to 0.60	Lower, less favorable value with supplementation; unadjusted 95% CI included 0	Not applicable - effect size estimated from reported mean +/- SD
Khosravi et al. (2018)	Olive flounder (Paralichthys olivaceus)	Shrimp, tilapia, or krill protein hydrolysate supplementation in a low-fishmeal, high-SPC diet	TH (24.1% fishmeal + 2.88% tilapia hydrolysate)	HFM (55% fishmeal reference diet)	Glucose (mg/dL)	26.40 +/- 2.70	28.90 +/- 3.30	3	3	Hedges' g	-0.66	-2.02 to 0.70	Lower measured value with supplementation; unadjusted 95% CI included 0	Not applicable - effect size estimated from reported mean +/- SD
Khosravi et al. (2018)	Olive flounder (Paralichthys olivaceus)	Shrimp, tilapia, or krill protein hydrolysate supplementation in a low-fishmeal, high-SPC diet	TH (24.1% fishmeal + 2.88% tilapia hydrolysate)	HFM (55% fishmeal reference diet)	Triglyceride (mg/dL)	379.00 +/- 53.00	378.00 +/- 21.00	3	3	Hedges' g	0.02	-1.26 to 1.30	Higher measured value with supplementation; unadjusted 95% CI included 0	Not applicable - effect size estimated from reported mean +/- SD
Khosravi et al. (2018)	Olive flounder (Paralichthys olivaceus)	Shrimp, tilapia, or krill protein hydrolysate supplementation in a low-fishmeal, high-SPC diet	TH (24.1% fishmeal + 2.88% tilapia hydrolysate)	HFM (55% fishmeal reference diet)	Total protein (g/dL)	4.290 +/- 0.670	4.020 +/- 0.440	3	3	Hedges' g	0.38	-0.93 to 1.69	Higher measured value with supplementation; unadjusted 95% CI included 0	Not applicable - effect size estimated from reported mean +/- SD
Khosravi et al. (2018)	Olive flounder (Paralichthys olivaceus)	Shrimp, tilapia, or krill protein hydrolysate supplementation in a low-fishmeal, high-SPC diet	TH (24.1% fishmeal + 2.88% tilapia hydrolysate)	HFM (55% fishmeal reference diet)	Total cholesterol (mg/dL)	296.00 +/- 60.00	307.00 +/- 55.00	3	3	Hedges' g	-0.15	-1.44 to 1.13	Lower measured value with supplementation; unadjusted 95% CI included 0	Not applicable - effect size estimated from reported mean +/- SD
Khosravi et al. (2018)	Olive flounder (Paralichthys olivaceus)	Shrimp, tilapia, or krill protein hydrolysate supplementation in a low-fishmeal, high-SPC diet	TH (24.1% fishmeal + 2.88% tilapia hydrolysate)	HFM (55% fishmeal reference diet)	Aspartate aminotransferase activity (U/L)	33.20 +/- 5.10	32.00 +/- 1.70	3	3	Hedges' g	0.25	-1.04 to 1.54	Higher measured value with supplementation; unadjusted 95% CI included 0	Not applicable - effect size estimated from reported mean +/- SD
Khosravi et al. (2018)	Olive flounder (Paralichthys olivaceus)	Shrimp, tilapia, or krill protein hydrolysate supplementation in a low-fishmeal, high-SPC diet	TH (24.1% fishmeal + 2.88% tilapia hydrolysate)	HFM (55% fishmeal reference diet)	Alanine aminotransferase activity (U/L)	6.360 +/- 0.390	6.240 +/- 0.380	3	3	Hedges' g	0.25	-1.04 to 1.54	Higher measured value with supplementation; unadjusted 95% CI included 0	Not applicable - effect size estimated from reported mean +/- SD
Khosravi et al. (2018)	Olive flounder (Paralichthys olivaceus)	Shrimp, tilapia, or krill protein hydrolysate supplementation in a low-fishmeal, high-SPC diet	TH (24.1% fishmeal + 2.88% tilapia hydrolysate)	HFM (55% fishmeal reference diet)	Moisture (% wet basis)	70.70 +/- 0.70	70.50 +/- 0.40	3	3	Hedges' g	0.28	-1.01 to 1.58	Higher measured value with supplementation; unadjusted 95% CI included 0	Not applicable - effect size estimated from reported mean +/- SD
Khosravi et al. (2018)	Olive flounder (Paralichthys olivaceus)	Shrimp, tilapia, or krill protein hydrolysate supplementation in a low-fishmeal, high-SPC diet	TH (24.1% fishmeal + 2.88% tilapia hydrolysate)	HFM (55% fishmeal reference diet)	Crude protein (% wet basis)	18.60 +/- 0.20	18.80 +/- 0.20	3	3	Hedges' g	-0.8	-2.20 to 0.60	Lower measured value with supplementation; unadjusted 95% CI included 0	Not applicable - effect size estimated from reported mean +/- SD
Khosravi et al. (2018)	Olive flounder (Paralichthys olivaceus)	Shrimp, tilapia, or krill protein hydrolysate supplementation in a low-fishmeal, high-SPC diet	TH (24.1% fishmeal + 2.88% tilapia hydrolysate)	HFM (55% fishmeal reference diet)	Crude lipid (% wet basis)	6.390 +/- 0.400	6.430 +/- 0.200	3	3	Hedges' g	-0.1	-1.38 to 1.18	Lower measured value with supplementation; unadjusted 95% CI included 0	Not applicable - effect size estimated from reported mean +/- SD
Khosravi et al. (2018)	Olive flounder (Paralichthys olivaceus)	Shrimp, tilapia, or krill protein hydrolysate supplementation in a low-fishmeal, high-SPC diet	TH (24.1% fishmeal + 2.88% tilapia hydrolysate)	HFM (55% fishmeal reference diet)	Ash (% wet basis)	3.280 +/- 0.210	3.450 +/- 0.000	3	3	Hedges' g	-0.92	-2.34 to 0.51	Lower measured value with supplementation; unadjusted 95% CI included 0	Not applicable - effect size estimated from reported mean +/- SD
Khosravi et al. (2018)	Olive flounder (Paralichthys olivaceus)	Shrimp, tilapia, or krill protein hydrolysate supplementation in a low-fishmeal, high-SPC diet	TH (24.1% fishmeal + 2.88% tilapia hydrolysate)	HFM (55% fishmeal reference diet)	Goblet cell count (cells)	1223.00 +/- 186.00	1324.00 +/- 176.00	3	3	Hedges' g	-0.45	-1.76 to 0.87	Lower, less favorable value with supplementation; unadjusted 95% CI included 0	Not applicable - effect size estimated from reported mean +/- SD
Khosravi et al. (2018)	Olive flounder (Paralichthys olivaceus)	Shrimp, tilapia, or krill protein hydrolysate supplementation in a low-fishmeal, high-SPC diet	TH (24.1% fishmeal + 2.88% tilapia hydrolysate)	HFM (55% fishmeal reference diet)	Villus height (µm)	502.00 +/- 40.00	505.00 +/- 45.00	3	3	Hedges' g	-0.06	-1.34 to 1.22	Lower, less favorable value with supplementation; unadjusted 95% CI included 0	Not applicable - effect size estimated from reported mean +/- SD
Khosravi et al. (2018)	Olive flounder (Paralichthys olivaceus)	Shrimp, tilapia, or krill protein hydrolysate supplementation in a low-fishmeal, high-SPC diet	TH (24.1% fishmeal + 2.88% tilapia hydrolysate)	HFM (55% fishmeal reference diet)	Enterocyte height (µm)	35.80 +/- 0.40	39.10 +/- 1.80	3	3	Hedges' g	-2.02	-3.92 to -0.13	Lower, less favorable value with supplementation; unadjusted 95% CI excluded 0	Not applicable - effect size estimated from reported mean +/- SD
Khosravi et al. (2018)	Olive flounder (Paralichthys olivaceus)	Shrimp, tilapia, or krill protein hydrolysate supplementation in a low-fishmeal, high-SPC diet	TH (24.1% fishmeal + 2.88% tilapia hydrolysate)	HFM (55% fishmeal reference diet)	Nitroblue tetrazolium activity (OD)	0.800 +/- 0.050	0.710 +/- 0.050	3	3	Hedges' g	1.44	-0.18 to 3.06	Higher, more favorable value with supplementation; unadjusted 95% CI included 0	Not applicable - effect size estimated from reported mean +/- SD
Khosravi et al. (2018)	Olive flounder (Paralichthys olivaceus)	Shrimp, tilapia, or krill protein hydrolysate supplementation in a low-fishmeal, high-SPC diet	TH (24.1% fishmeal + 2.88% tilapia hydrolysate)	HFM (55% fishmeal reference diet)	Total immunoglobulin (mg/mL)	21.90 +/- 1.60	21.70 +/- 1.10	3	3	Hedges' g	0.12	-1.17 to 1.40	Higher, more favorable value with supplementation; unadjusted 95% CI included 0	Not applicable - effect size estimated from reported mean +/- SD
Khosravi et al. (2018)	Olive flounder (Paralichthys olivaceus)	Shrimp, tilapia, or krill protein hydrolysate supplementation in a low-fishmeal, high-SPC diet	TH (24.1% fishmeal + 2.88% tilapia hydrolysate)	HFM (55% fishmeal reference diet)	Lysozyme activity (µg/mL)	26.40 +/- 1.30	24.50 +/- 2.20	3	3	Hedges' g	0.84	-0.57 to 2.25	Higher, more favorable value with supplementation; unadjusted 95% CI included 0	Not applicable - effect size estimated from reported mean +/- SD
Khosravi et al. (2018)	Olive flounder (Paralichthys olivaceus)	Shrimp, tilapia, or krill protein hydrolysate supplementation in a low-fishmeal, high-SPC diet	TH (24.1% fishmeal + 2.88% tilapia hydrolysate)	HFM (55% fishmeal reference diet)	Myeloperoxidase level	2.080 +/- 0.220	1.950 +/- 0.300	3	3	Hedges' g	0.4	-0.91 to 1.70	Higher measured value with supplementation; unadjusted 95% CI included 0	Not applicable - effect size estimated from reported mean +/- SD
Khosravi et al. (2018)	Olive flounder (Paralichthys olivaceus)	Shrimp, tilapia, or krill protein hydrolysate supplementation in a low-fishmeal, high-SPC diet	TH (24.1% fishmeal + 2.88% tilapia hydrolysate)	HFM (55% fishmeal reference diet)	Antiprotease activity (% inhibition)	33.30 +/- 1.90	32.00 +/- 2.40	3	3	Hedges' g	0.48	-0.84 to 1.80	Higher measured value with supplementation; unadjusted 95% CI included 0	Not applicable - effect size estimated from reported mean +/- SD
Khosravi et al. (2018)	Olive flounder (Paralichthys olivaceus)	Shrimp, tilapia, or krill protein hydrolysate supplementation in a low-fishmeal, high-SPC diet	TH (24.1% fishmeal + 2.88% tilapia hydrolysate)	HFM (55% fishmeal reference diet)	Superoxide dismutase activity (% inhibition)	75.20 +/- 2.70	67.50 +/- 3.30	3	3	Hedges' g	2.04	0.13 to 3.95	Higher, more favorable value with supplementation; unadjusted 95% CI excluded 0	Not applicable - effect size estimated from reported mean +/- SD
Khosravi et al. (2018)	Olive flounder (Paralichthys olivaceus)	Shrimp, tilapia, or krill protein hydrolysate supplementation in a low-fishmeal, high-SPC diet	TH (24.1% fishmeal + 2.88% tilapia hydrolysate)	HFM (55% fishmeal reference diet)	Glutathione peroxidase activity (mU/mL)	16.80 +/- 0.80	16.70 +/- 0.40	3	3	Hedges' g	0.13	-1.16 to 1.41	Higher, more favorable value with supplementation; unadjusted 95% CI included 0	Not applicable - effect size estimated from reported mean +/- SD
Khosravi et al. (2018)	Olive flounder (Paralichthys olivaceus)	Shrimp, tilapia, or krill protein hydrolysate supplementation in a low-fishmeal, high-SPC diet	KH (24.1% fishmeal + 3.12% krill hydrolysate)	HFM (55% fishmeal reference diet)	Final body weight (g)	127.00 +/- 8.10	121.00 +/- 0.60	3	3	Hedges' g	0.84	-0.57 to 2.24	Higher, more favorable value with supplementation; unadjusted 95% CI included 0	Not applicable - effect size estimated from reported mean +/- SD
Khosravi et al. (2018)	Olive flounder (Paralichthys olivaceus)	Shrimp, tilapia, or krill protein hydrolysate supplementation in a low-fishmeal, high-SPC diet	KH (24.1% fishmeal + 3.12% krill hydrolysate)	HFM (55% fishmeal reference diet)	Weight gain (%)	742.00 +/- 50.60	699.00 +/- 7.30	3	3	Hedges' g	0.95	-0.49 to 2.39	Higher, more favorable value with supplementation; unadjusted 95% CI included 0	Not applicable - effect size estimated from reported mean +/- SD
Khosravi et al. (2018)	Olive flounder (Paralichthys olivaceus)	Shrimp, tilapia, or krill protein hydrolysate supplementation in a low-fishmeal, high-SPC diet	KH (24.1% fishmeal + 3.12% krill hydrolysate)	HFM (55% fishmeal reference diet)	Specific growth rate (%/day)	2.960 +/- 0.080	2.890 +/- 0.010	3	3	Hedges' g	0.98	-0.47 to 2.43	Higher, more favorable value with supplementation; unadjusted 95% CI included 0	Not applicable - effect size estimated from reported mean +/- SD
Khosravi et al. (2018)	Olive flounder (Paralichthys olivaceus)	Shrimp, tilapia, or krill protein hydrolysate supplementation in a low-fishmeal, high-SPC diet	KH (24.1% fishmeal + 3.12% krill hydrolysate)	HFM (55% fishmeal reference diet)	Feed intake (g/fish)	111.00 +/- 1.20	113.00 +/- 1.20	3	3	Hedges' g	-1.33	-2.91 to 0.25	Lower measured value with supplementation; unadjusted 95% CI included 0	Not applicable - effect size estimated from reported mean +/- SD

Study	Species	Strategy	Intervention	Comparator	Outcome and unit	Intervention mean +/- SD	Comparator mean +/- SD	n intervention (tanks)	n comparator (tanks)	Effect-size metric	Effect size	95% CI	Interpretation	Reason not estimated
Khosravi et al. (2018)	Olive flounder (Paralichthys olivaceus)	Shrimp, tilapia, or krill protein hydrolysate supplementation in a low-fishmeal, high-SPC diet	KH (24.1% fishmeal + 3.12% krill hydrolysate)	HFM (55% fishmeal reference diet)	Feed conversion ratio	1.070 +/- 0.060	1.180 +/- 0.030	3	3	Hedges' g (direction reversed)	1.86	0.04 to 3.67	Lower, more favorable value with supplementation; unadjusted 95% CI excluded 0	Not applicable - effect size estimated from reported mean +/- SD
Khosravi et al. (2018)	Olive flounder (Paralichthys olivaceus)	Shrimp, tilapia, or krill protein hydrolysate supplementation in a low-fishmeal, high-SPC diet	KH (24.1% fishmeal + 3.12% krill hydrolysate)	HFM (55% fishmeal reference diet)	Protein efficiency ratio	1.830 +/- 0.100	1.660 +/- 0.040	3	3	Hedges' g	1.79	0.01 to 3.57	Higher, more favorable value with supplementation; unadjusted 95% CI excluded 0	Not applicable - effect size estimated from reported mean +/- SD
Khosravi et al. (2018)	Olive flounder (Paralichthys olivaceus)	Shrimp, tilapia, or krill protein hydrolysate supplementation in a low-fishmeal, high-SPC diet	KH (24.1% fishmeal + 3.12% krill hydrolysate)	HFM (55% fishmeal reference diet)	Survival rate (%)	91.40 +/- 4.90	83.90 +/- 3.20	3	3	Hedges' g	1.45	-0.18 to 3.08	Higher, more favorable value with supplementation; unadjusted 95% CI included 0	Not applicable - effect size estimated from reported mean +/- SD
Khosravi et al. (2018)	Olive flounder (Paralichthys olivaceus)	Shrimp, tilapia, or krill protein hydrolysate supplementation in a low-fishmeal, high-SPC diet	KH (24.1% fishmeal + 3.12% krill hydrolysate)	HFM (55% fishmeal reference diet)	Apparent digestibility coefficient of dry matter (%)	82.70 +/- 0.80	81.20 +/- 0.80	3	3	Hedges' g	1.5	-0.15 to 3.15	Higher, more favorable value with supplementation; unadjusted 95% CI included 0	Not applicable - effect size estimated from reported mean +/- SD
Khosravi et al. (2018)	Olive flounder (Paralichthys olivaceus)	Shrimp, tilapia, or krill protein hydrolysate supplementation in a low-fishmeal, high-SPC diet	KH (24.1% fishmeal + 3.12% krill hydrolysate)	HFM (55% fishmeal reference diet)	Apparent digestibility coefficient of protein (%)	94.20 +/- 0.40	94.40 +/- 0.50	3	3	Hedges' g	-0.35	-1.66 to 0.95	Lower, less favorable value with supplementation; unadjusted 95% CI included 0	Not applicable - effect size estimated from reported mean +/- SD
Khosravi et al. (2018)	Olive flounder (Paralichthys olivaceus)	Shrimp, tilapia, or krill protein hydrolysate supplementation in a low-fishmeal, high-SPC diet	KH (24.1% fishmeal + 3.12% krill hydrolysate)	HFM (55% fishmeal reference diet)	Hematocrit (%)	30.40 +/- 2.20	32.20 +/- 3.30	3	3	Hedges' g	-0.51	-1.84 to 0.82	Lower, less favorable value with supplementation; unadjusted 95% CI included 0	Not applicable - effect size estimated from reported mean +/- SD
Khosravi et al. (2018)	Olive flounder (Paralichthys olivaceus)	Shrimp, tilapia, or krill protein hydrolysate supplementation in a low-fishmeal, high-SPC diet	KH (24.1% fishmeal + 3.12% krill hydrolysate)	HFM (55% fishmeal reference diet)	Hemoglobin (g/dL)	5.670 +/- 0.610	5.590 +/- 0.130	3	3	Hedges' g	0.15	-1.14 to 1.43	Higher, more favorable value with supplementation; unadjusted 95% CI included 0	Not applicable - effect size estimated from reported mean +/- SD
Khosravi et al. (2018)	Olive flounder (Paralichthys olivaceus)	Shrimp, tilapia, or krill protein hydrolysate supplementation in a low-fishmeal, high-SPC diet	KH (24.1% fishmeal + 3.12% krill hydrolysate)	HFM (55% fishmeal reference diet)	Glucose (mg/dL)	28.30 +/- 2.40	28.90 +/- 3.30	3	3	Hedges' g	-0.17	-1.45 to 1.12	Lower measured value with supplementation; unadjusted 95% CI included 0	Not applicable - effect size estimated from reported mean +/- SD
Khosravi et al. (2018)	Olive flounder (Paralichthys olivaceus)	Shrimp, tilapia, or krill protein hydrolysate supplementation in a low-fishmeal, high-SPC diet	KH (24.1% fishmeal + 3.12% krill hydrolysate)	HFM (55% fishmeal reference diet)	Triglyceride (mg/dL)	381.00 +/- 48.00	378.00 +/- 21.00	3	3	Hedges' g	0.06	-1.22 to 1.35	Higher measured value with supplementation; unadjusted 95% CI included 0	Not applicable - effect size estimated from reported mean +/- SD
Khosravi et al. (2018)	Olive flounder (Paralichthys olivaceus)	Shrimp, tilapia, or krill protein hydrolysate supplementation in a low-fishmeal, high-SPC diet	KH (24.1% fishmeal + 3.12% krill hydrolysate)	HFM (55% fishmeal reference diet)	Total protein (g/dL)	4.300 +/- 0.470	4.020 +/- 0.440	3	3	Hedges' g	0.49	-0.83 to 1.82	Higher measured value with supplementation; unadjusted 95% CI included 0	Not applicable - effect size estimated from reported mean +/- SD
Khosravi et al. (2018)	Olive flounder (Paralichthys olivaceus)	Shrimp, tilapia, or krill protein hydrolysate supplementation in a low-fishmeal, high-SPC diet	KH (24.1% fishmeal + 3.12% krill hydrolysate)	HFM (55% fishmeal reference diet)	Total cholesterol (mg/dL)	284.00 +/- 19.00	307.00 +/- 55.00	3	3	Hedges' g	-0.45	-1.76 to 0.87	Lower measured value with supplementation; unadjusted 95% CI included 0	Not applicable - effect size estimated from reported mean +/- SD
Khosravi et al. (2018)	Olive flounder (Paralichthys olivaceus)	Shrimp, tilapia, or krill protein hydrolysate supplementation in a low-fishmeal, high-SPC diet	KH (24.1% fishmeal + 3.12% krill hydrolysate)	HFM (55% fishmeal reference diet)	Aspartate aminotransferase activity (U/L)	28.80 +/- 5.90	32.00 +/- 1.70	3	3	Hedges' g	-0.59	-1.93 to 0.75	Lower measured value with supplementation; unadjusted 95% CI included 0	Not applicable - effect size estimated from reported mean +/- SD
Khosravi et al. (2018)	Olive flounder (Paralichthys olivaceus)	Shrimp, tilapia, or krill protein hydrolysate supplementation in a low-fishmeal, high-SPC diet	KH (24.1% fishmeal + 3.12% krill hydrolysate)	HFM (55% fishmeal reference diet)	Alanine aminotransferase activity (U/L)	5.870 +/- 0.260	6.240 +/- 0.380	3	3	Hedges' g	-0.91	-2.34 to 0.52	Lower measured value with supplementation; unadjusted 95% CI included 0	Not applicable - effect size estimated from reported mean +/- SD
Khosravi et al. (2018)	Olive flounder (Paralichthys olivaceus)	Shrimp, tilapia, or krill protein hydrolysate supplementation in a low-fishmeal, high-SPC diet	KH (24.1% fishmeal + 3.12% krill hydrolysate)	HFM (55% fishmeal reference diet)	Moisture (% wet basis)	71.20 +/- 0.40	70.50 +/- 0.40	3	3	Hedges' g	1.4	-0.21 to 3.01	Higher measured value with supplementation; unadjusted 95% CI included 0	Not applicable - effect size estimated from reported mean +/- SD
Khosravi et al. (2018)	Olive flounder (Paralichthys olivaceus)	Shrimp, tilapia, or krill protein hydrolysate supplementation in a low-fishmeal, high-SPC diet	KH (24.1% fishmeal + 3.12% krill hydrolysate)	HFM (55% fishmeal reference diet)	Crude protein (% wet basis)	18.70 +/- 0.10	18.80 +/- 0.20	3	3	Hedges' g	-0.51	-1.83 to 0.82	Lower measured value with supplementation; unadjusted 95% CI included 0	Not applicable - effect size estimated from reported mean +/- SD
Khosravi et al. (2018)	Olive flounder (Paralichthys olivaceus)	Shrimp, tilapia, or krill protein hydrolysate supplementation in a low-fishmeal, high-SPC diet	KH (24.1% fishmeal + 3.12% krill hydrolysate)	HFM (55% fishmeal reference diet)	Crude lipid (% wet basis)	6.060 +/- 0.130	6.430 +/- 0.200	3	3	Hedges' g	-1.75	-3.52 to 0.01	Lower measured value with supplementation; unadjusted 95% CI included 0	Not applicable - effect size estimated from reported mean +/- SD
Khosravi et al. (2018)	Olive flounder (Paralichthys olivaceus)	Shrimp, tilapia, or krill protein hydrolysate supplementation in a low-fishmeal, high-SPC diet	KH (24.1% fishmeal + 3.12% krill hydrolysate)	HFM (55% fishmeal reference diet)	Ash (% wet basis)	3.280 +/- 0.050	3.450 +/- 0.000	3	3	Hedges' g	-3.85	-6.80 to -0.89	Lower measured value with supplementation; unadjusted 95% CI excluded 0	Not applicable - effect size estimated from reported mean +/- SD
Khosravi et al. (2018)	Olive flounder (Paralichthys olivaceus)	Shrimp, tilapia, or krill protein hydrolysate supplementation in a low-fishmeal, high-SPC diet	KH (24.1% fishmeal + 3.12% krill hydrolysate)	HFM (55% fishmeal reference diet)	Goblet cell count (cells)	1392.00 +/- 111.00	1324.00 +/- 176.00	3	3	Hedges' g	0.37	-0.94 to 1.68	Higher, more favorable value with supplementation; unadjusted 95% CI included 0	Not applicable - effect size estimated from reported mean +/- SD
Khosravi et al. (2018)	Olive flounder (Paralichthys olivaceus)	Shrimp, tilapia, or krill protein hydrolysate supplementation in a low-fishmeal, high-SPC diet	KH (24.1% fishmeal + 3.12% krill hydrolysate)	HFM (55% fishmeal reference diet)	Villus height (µm)	502.00 +/- 12.00	505.00 +/- 45.00	3	3	Hedges' g	-0.07	-1.35 to 1.21	Lower, less favorable value with supplementation; unadjusted 95% CI included 0	Not applicable - effect size estimated from reported mean +/- SD
Khosravi et al. (2018)	Olive flounder (Paralichthys olivaceus)	Shrimp, tilapia, or krill protein hydrolysate supplementation in a low-fishmeal, high-SPC diet	KH (24.1% fishmeal + 3.12% krill hydrolysate)	HFM (55% fishmeal reference diet)	Enterocyte height (µm)	36.30 +/- 0.60	39.10 +/- 1.80	3	3	Hedges' g	-1.67	-3.40 to 0.06	Lower, less favorable value with supplementation; unadjusted 95% CI included 0	Not applicable - effect size estimated from reported mean +/- SD
Khosravi et al. (2018)	Olive flounder (Paralichthys olivaceus)	Shrimp, tilapia, or krill protein hydrolysate supplementation in a low-fishmeal, high-SPC diet	KH (24.1% fishmeal + 3.12% krill hydrolysate)	HFM (55% fishmeal reference diet)	Nitroblue tetrazolium activity (OD)	0.800 +/- 0.030	0.710 +/- 0.050	3	3	Hedges' g	1.75	-0.02 to 3.51	Higher, more favorable value with supplementation; unadjusted 95% CI included 0	Not applicable - effect size estimated from reported mean +/- SD
Khosravi et al. (2018)	Olive flounder (Paralichthys olivaceus)	Shrimp, tilapia, or krill protein hydrolysate supplementation in a low-fishmeal, high-SPC diet	KH (24.1% fishmeal + 3.12% krill hydrolysate)	HFM (55% fishmeal reference diet)	Total immunoglobulin (mg/mL)	24.60 +/- 2.60	21.70 +/- 1.10	3	3	Hedges' g	1.16	-0.35 to 2.67	Higher, more favorable value with supplementation; unadjusted 95% CI included 0	Not applicable - effect size estimated from reported mean +/- SD
Khosravi et al. (2018)	Olive flounder (Paralichthys olivaceus)	Shrimp, tilapia, or krill protein hydrolysate supplementation in a low-fishmeal, high-SPC diet	KH (24.1% fishmeal + 3.12% krill hydrolysate)	HFM (55% fishmeal reference diet)	Lysozyme activity (µg/mL)	27.30 +/- 1.10	24.50 +/- 2.20	3	3	Hedges' g	1.29	-0.27 to 2.85	Higher, more favorable value with supplementation; unadjusted 95% CI included 0	Not applicable - effect size estimated from reported mean +/- SD
Khosravi et al. (2018)	Olive flounder (Paralichthys olivaceus)	Shrimp, tilapia, or krill protein hydrolysate supplementation in a low-fishmeal, high-SPC diet	KH (24.1% fishmeal + 3.12% krill hydrolysate)	HFM (55% fishmeal reference diet)	Myeloperoxidase level	2.260 +/- 0.210	1.950 +/- 0.300	3	3	Hedges' g	0.96	-0.48 to 2.40	Higher measured value with supplementation; unadjusted 95% CI included 0	Not applicable - effect size estimated from reported mean +/- SD
Khosravi et al. (2018)	Olive flounder (Paralichthys olivaceus)	Shrimp, tilapia, or krill protein hydrolysate supplementation in a low-fishmeal, high-SPC diet	KH (24.1% fishmeal + 3.12% krill hydrolysate)	HFM (55% fishmeal reference diet)	Antiprotease activity (% inhibition)	35.80 +/- 0.30	32.00 +/- 2.40	3	3	Hedges' g	1.78	0.00 to 3.55	Higher measured value with supplementation; unadjusted 95% CI excluded 0	Not applicable - effect size estimated from reported mean +/- SD
Khosravi et al. (2018)	Olive flounder (Paralichthys olivaceus)	Shrimp, tilapia, or krill protein hydrolysate supplementation in a low-fishmeal, high-SPC diet	KH (24.1% fishmeal + 3.12% krill hydrolysate)	HFM (55% fishmeal reference diet)	Superoxide dismutase activity (% inhibition)	72.10 +/- 3.60	67.50 +/- 3.30	3	3	Hedges' g	1.07	-0.41 to 2.54	Higher, more favorable value with supplementation; unadjusted 95% CI included 0	Not applicable - effect size estimated from reported mean +/- SD
Khosravi et al. (2018)	Olive flounder (Paralichthys olivaceus)	Shrimp, tilapia, or krill protein hydrolysate supplementation in a low-fishmeal, high-SPC diet	KH (24.1% fishmeal + 3.12% krill hydrolysate)	HFM (55% fishmeal reference diet)	Glutathione peroxidase activity (mU/mL)	15.80 +/- 1.60	16.70 +/- 0.40	3	3	Hedges' g	-0.62	-1.97 to 0.73	Lower, less favorable value with supplementation; unadjusted 95% CI included 0	Not applicable - effect size estimated from reported mean +/- SD
Khosravi et al. (2018)	Olive flounder (Paralichthys olivaceus)	Shrimp, tilapia, or krill protein hydrolysate supplementation in a low-fishmeal, high-SPC diet	SH (24.2% fishmeal + 3.34% shrimp hydrolysate)	LFM (27.5% fishmeal + 22% soy protein concentrate)	Final survival after Edwardsiella tarda challenge (%)	Not reported numerically	Not reported numerically	3	3	Not estimable	Not estimable	Not estimable	Figure 1 shows higher final challenge survival with hydrolysate supplementation; an exact standardized effect was not calculated	Exact endpoint means and SDs were available only graphically in Figure 1
Khosravi et al. (2018)	Olive flounder (Paralichthys olivaceus)	Shrimp, tilapia, or krill protein hydrolysate supplementation in a low-fishmeal, high-SPC diet	TH (24.1% fishmeal + 2.88% tilapia hydrolysate)	LFM (27.5% fishmeal + 22% soy protein concentrate)	Final survival after Edwardsiella tarda challenge (%)	Not reported numerically	Not reported numerically	3	3	Not estimable	Not estimable	Not estimable	Figure 1 shows higher final challenge survival with hydrolysate supplementation; an exact standardized effect was not calculated	Exact endpoint means and SDs were available only graphically in Figure 1
Khosravi et al. (2018)	Olive flounder (Paralichthys olivaceus)	Shrimp, tilapia, or krill protein hydrolysate supplementation in a low-fishmeal, high-SPC diet	KH (24.1% fishmeal + 3.12% krill hydrolysate)	LFM (27.5% fishmeal + 22% soy protein concentrate)	Final survival after Edwardsiella tarda challenge (%)	Not reported numerically	Not reported numerically	3	3	Not estimable	Not estimable	Not estimable	Figure 1 shows higher final challenge survival with hydrolysate supplementation; an exact standardized effect was not calculated	Exact endpoint means and SDs were available only graphically in Figure 1
Khosravi et al. (2018)	Olive flounder (Paralichthys olivaceus)	Shrimp, tilapia, or krill protein hydrolysate supplementation in a low-fishmeal, high-SPC diet	SH (24.2% fishmeal + 3.34% shrimp hydrolysate)	HFM (55% fishmeal reference diet)	Final survival after Edwardsiella tarda challenge (%)	Not reported numerically	Not reported numerically	3	3	Not estimable	Not estimable	Not estimable	Figure 1 shows the final challenge-survival direction and Tukey lettering relative to HFM; an exact standardized effect was not calculated	Exact endpoint means and SDs were available only graphically in Figure 1

Study	Species	Strategy	Intervention	Comparator	Outcome and unit	Intervention mean +/- SD	Comparator mean +/- SD	n intervention (tanks)	n comparator (tanks)	Effect-size metric	Effect size	95% CI	Interpretation	Reason not estimated
Khosravi et al. (2018)	Olive flounder (Paralichthys olivaceus)	Shrimp, tilapia, or krill protein hydrolysate supplementation in a low-fishmeal, high-SPC diet	TH (24.1% fishmeal + 2.88% tilapia hydrolysate)	HFM (55% fishmeal reference diet)	Final survival after Edwardsiella tarda challenge (%)	Not reported numerically	Not reported numerically	3	3	Not estimable	Not estimable	Not estimable	Figure 1 shows the final challenge-survival direction and Tukey lettering relative to HFM; an exact standardized effect was not calculated	Exact endpoint means and SDs were available only graphically in Figure 1
Khosravi et al. (2018)	Olive flounder (Paralichthys olivaceus)	Shrimp, tilapia, or krill protein hydrolysate supplementation in a low-fishmeal, high-SPC diet	KH (24.1% fishmeal + 3.12% krill hydrolysate)	HFM (55% fishmeal reference diet)	Final survival after Edwardsiella tarda challenge (%)	Not reported numerically	Not reported numerically	3	3	Not estimable	Not estimable	Not estimable	Figure 1 shows the final challenge-survival direction and Tukey lettering relative to HFM; an exact standardized effect was not calculated	Exact endpoint means and SDs were available only graphically in Figure 1

Note: Values in Tables 4-9 are reported as mean +/- SD for triplicate groups. SH, TH, and KH were each compared with the LFM basal diet and the HFM reference diet. FCR was direction-reversed because lower values are favorable. Final survival after the Edwardsiella tarda challenge was marked not estimable because exact endpoint means and SDs were not printed. Pairwise 95% CIs are descriptive and unadjusted.

Source: Khosravi et al. (2018), https://doi.org/10.1111/jwas.12436

Study 18. Egerton et al. (2020): partly hydrolysed and soluble fish-protein hydrolysates in Atlantic salmon parr

Study	Species	Strategy	Intervention	Comparator	Outcome and unit	Intervention mean +/- SD	Comparator mean +/- SD	n intervention (tanks)	n comparator (tanks)	Effect-size metric	Effect size	95% CI	Interpretation	Reason not estimated
Egerton et al. (2020)	Atlantic salmon parr (Salmo salar)	Fish protein hydrolysate supplementation in an 80% plant-protein, low-fishmeal diet	PHP (80% plant protein + 5% fishmeal + 10% partly hydrolysed FPH)	PL (80% plant protein + 15% fishmeal)	Feed intake (g/fish)	24.81 +/- 1.03	25.44 +/- 1.88	3	3	Hedges' g	-0.33	-1.63 to 0.97	Lower measured value with supplementation; unadjusted 95% CI included 0	Not applicable - effect size estimated from reported mean +/- SD
Egerton et al. (2020)	Atlantic salmon parr (Salmo salar)	Fish protein hydrolysate supplementation in an 80% plant-protein, low-fishmeal diet	PHP (80% plant protein + 5% fishmeal + 10% partly hydrolysed FPH)	PL (80% plant protein + 15% fishmeal)	Feed conversion ratio	0.880 +/- 0.070	1.040 +/- 0.060	3	3	Hedges' g (direction reversed)	1.96	0.10 to 3.83	Lower, more favorable value with supplementation; unadjusted 95% CI excluded 0	Not applicable - effect size estimated from reported mean +/- SD
Egerton et al. (2020)	Atlantic salmon parr (Salmo salar)	Fish protein hydrolysate supplementation in an 80% plant-protein, low-fishmeal diet	PHP (80% plant protein + 5% fishmeal + 10% partly hydrolysed FPH)	PL (80% plant protein + 15% fishmeal)	Protein productive value	3.080 +/- 0.210	2.670 +/- 0.190	3	3	Hedges' g	1.64	-0.07 to 3.35	Higher, more favorable value with supplementation; unadjusted 95% CI included 0	Not applicable - effect size estimated from reported mean +/- SD
Egerton et al. (2020)	Atlantic salmon parr (Salmo salar)	Fish protein hydrolysate supplementation in an 80% plant-protein, low-fishmeal diet	PHP (80% plant protein + 5% fishmeal + 10% partly hydrolysed FPH)	PL (80% plant protein + 15% fishmeal)	Protein efficiency ratio	2.570 +/- 0.130	2.210 +/- 0.130	3	3	Hedges' g	2.22	0.22 to 4.21	Higher, more favorable value with supplementation; unadjusted 95% CI excluded 0	Not applicable - effect size estimated from reported mean +/- SD
Egerton et al. (2020)	Atlantic salmon parr (Salmo salar)	Fish protein hydrolysate supplementation in an 80% plant-protein, low-fishmeal diet	PHP (80% plant protein + 5% fishmeal + 10% partly hydrolysed FPH)	PL (80% plant protein + 15% fishmeal)	Liver weight (mg)	561.67 +/- 28.11	425.29 +/- 25.57	3	3	Hedges' g	4.06	0.97 to 7.15	Higher measured value with supplementation; unadjusted 95% CI excluded 0	Not applicable - effect size estimated from reported mean +/- SD
Egerton et al. (2020)	Atlantic salmon parr (Salmo salar)	Fish protein hydrolysate supplementation in an 80% plant-protein, low-fishmeal diet	PHP (80% plant protein + 5% fishmeal + 10% partly hydrolysed FPH)	PL (80% plant protein + 15% fishmeal)	Hepatosomatic index (%)	1.520 +/- 0.080	1.250 +/- 0.120	3	3	Hedges' g	2.12	0.17 to 4.07	Higher measured value with supplementation; unadjusted 95% CI excluded 0	Not applicable - effect size estimated from reported mean +/- SD
Egerton et al. (2020)	Atlantic salmon parr (Salmo salar)	Fish protein hydrolysate supplementation in an 80% plant-protein, low-fishmeal diet	PHP (80% plant protein + 5% fishmeal + 10% partly hydrolysed FPH)	PL (80% plant protein + 15% fishmeal)	Moisture (% wet basis)	72.12 +/- 3.17	72.81 +/- 3.67	3	3	Hedges' g	-0.16	-1.45 to 1.12	Lower measured value with supplementation; unadjusted 95% CI included 0	Not applicable - effect size estimated from reported mean +/- SD
Egerton et al. (2020)	Atlantic salmon parr (Salmo salar)	Fish protein hydrolysate supplementation in an 80% plant-protein, low-fishmeal diet	PHP (80% plant protein + 5% fishmeal + 10% partly hydrolysed FPH)	PL (80% plant protein + 15% fishmeal)	Crude protein (% wet basis)	17.07 +/- 0.57	16.82 +/- 0.23	3	3	Hedges' g	0.46	-0.86 to 1.78	Higher measured value with supplementation; unadjusted 95% CI included 0	Not applicable - effect size estimated from reported mean +/- SD
Egerton et al. (2020)	Atlantic salmon parr (Salmo salar)	Fish protein hydrolysate supplementation in an 80% plant-protein, low-fishmeal diet	PHP (80% plant protein + 5% fishmeal + 10% partly hydrolysed FPH)	PL (80% plant protein + 15% fishmeal)	Crude lipid (% wet basis)	10.07 +/- 0.79	10.14 +/- 0.78	3	3	Hedges' g	-0.07	-1.35 to 1.21	Lower measured value with supplementation; unadjusted 95% CI included 0	Not applicable - effect size estimated from reported mean +/- SD
Egerton et al. (2020)	Atlantic salmon parr (Salmo salar)	Fish protein hydrolysate supplementation in an 80% plant-protein, low-fishmeal diet	PHP (80% plant protein + 5% fishmeal + 10% partly hydrolysed FPH)	PL (80% plant protein + 15% fishmeal)	Ash (% wet basis)	1.580 +/- 0.120	1.740 +/- 0.110	3	3	Hedges' g	-1.11	-2.61 to 0.38	Lower measured value with supplementation; unadjusted 95% CI included 0	Not applicable - effect size estimated from reported mean +/- SD
Egerton et al. (2020)	Atlantic salmon parr (Salmo salar)	Fish protein hydrolysate supplementation in an 80% plant-protein, low-fishmeal diet	PHP (80% plant protein + 5% fishmeal + 10% partly hydrolysed FPH)	PL (80% plant protein + 15% fishmeal)	Villus height (μm)	435.12 +/- 159.02	448.30 +/- 227.62	3	3	Hedges' g	-0.05	-1.33 to 1.23	Lower, less favorable value with supplementation; unadjusted 95% CI included 0	Not applicable - effect size estimated from reported mean +/- SD
Egerton et al. (2020)	Atlantic salmon parr (Salmo salar)	Fish protein hydrolysate supplementation in an 80% plant-protein, low-fishmeal diet	PHP (80% plant protein + 5% fishmeal + 10% partly hydrolysed FPH)	PL (80% plant protein + 15% fishmeal)	Villus width (μm)	75.09 +/- 14.89	76.44 +/- 22.63	3	3	Hedges' g	-0.06	-1.34 to 1.22	Lower, less favorable value with supplementation; unadjusted 95% CI included 0	Not applicable - effect size estimated from reported mean +/- SD
Egerton et al. (2020)	Atlantic salmon parr (Salmo salar)	Fish protein hydrolysate supplementation in an 80% plant-protein, low-fishmeal diet	PHP (80% plant protein + 5% fishmeal + 10% partly hydrolysed FPH)	PL (80% plant protein + 15% fishmeal)	Villus height-to-width ratio	5.830 +/- 1.800	6.210 +/- 3.350	3	3	Hedges' g	-0.11	-1.40 to 1.17	Lower measured value with supplementation; unadjusted 95% CI included 0	Not applicable - effect size estimated from reported mean +/- SD
Egerton et al. (2020)	Atlantic salmon parr (Salmo salar)	Fish protein hydrolysate supplementation in an 80% plant-protein, low-fishmeal diet	PHP (80% plant protein + 5% fishmeal + 10% partly hydrolysed FPH)	PL (80% plant protein + 15% fishmeal)	Intestinal wall width (μm)	65.57 +/- 23.16	63.18 +/- 29.97	3	3	Hedges' g	0.07	-1.21 to 1.35	Higher measured value with supplementation; unadjusted 95% CI included 0	Not applicable - effect size estimated from reported mean +/- SD
Egerton et al. (2020)	Atlantic salmon parr (Salmo salar)	Fish protein hydrolysate supplementation in an 80% plant-protein, low-fishmeal diet	PHP (80% plant protein + 5% fishmeal + 10% partly hydrolysed FPH)	PL (80% plant protein + 15% fishmeal)	Intestine diameter (mm)	2.080 +/- 0.500	2.100 +/- 0.840	3	3	Hedges' g	-0.02	-1.30 to 1.26	Lower measured value with supplementation; unadjusted 95% CI included 0	Not applicable - effect size estimated from reported mean +/- SD
Egerton et al. (2020)	Atlantic salmon parr (Salmo salar)	Fish protein hydrolysate supplementation in an 80% plant-protein, low-fishmeal diet	PHP (80% plant protein + 5% fishmeal + 10% partly hydrolysed FPH)	PL (80% plant protein + 15% fishmeal)	Interior lumen area (mm^2)	1.460 +/- 0.680	1.630 +/- 1.300	3	3	Hedges' g	-0.13	-1.41 to 1.15	Lower measured value with supplementation; unadjusted 95% CI included 0	Not applicable - effect size estimated from reported mean +/- SD
Egerton et al. (2020)	Atlantic salmon parr (Salmo salar)	Fish protein hydrolysate supplementation in an 80% plant-protein, low-fishmeal diet	PHP (80% plant protein + 5% fishmeal + 10% partly hydrolysed FPH)	PL (80% plant protein + 15% fishmeal)	Internal perimeter (mm)	25.16 +/- 9.29	21.13 +/- 9.46	3	3	Hedges' g	0.34	-0.96 to 1.65	Higher measured value with supplementation; unadjusted 95% CI included 0	Not applicable - effect size estimated from reported mean +/- SD
Egerton et al. (2020)	Atlantic salmon parr (Salmo salar)	Fish protein hydrolysate supplementation in an 80% plant-protein, low-fishmeal diet	PHP (80% plant protein + 5% fishmeal + 10% partly hydrolysed FPH)	PL (80% plant protein + 15% fishmeal)	External perimeter (mm)	6.510 +/- 1.500	6.590 +/- 2.470	3	3	Hedges' g	-0.03	-1.31 to 1.25	Lower measured value with supplementation; unadjusted 95% CI included 0	Not applicable - effect size estimated from reported mean +/- SD
Egerton et al. (2020)	Atlantic salmon parr (Salmo salar)	Fish protein hydrolysate supplementation in an 80% plant-protein, low-fishmeal diet	PHP (80% plant protein + 5% fishmeal + 10% partly hydrolysed FPH)	PL (80% plant protein + 15% fishmeal)	Perimeter ratio (absolute)	3.770 +/- 0.620	3.240 +/- 0.880	3	3	Hedges' g	0.56	-0.78 to 1.89	Higher measured value with supplementation; unadjusted 95% CI included 0	Not applicable - effect size estimated from reported mean +/- SD
Egerton et al. (2020)	Atlantic salmon parr (Salmo salar)	Fish protein hydrolysate supplementation in an 80% plant-protein, low-fishmeal diet	PHP (80% plant protein + 5% fishmeal + 10% partly hydrolysed FPH)	PL (80% plant protein + 15% fishmeal)	Alanine (μg/mL)	307.10 +/- 30.00	265.60 +/- 34.70	3	3	Hedges' g	1.02	-0.44 to 2.49	Higher measured value with supplementation; unadjusted 95% CI included 0	Not applicable - effect size estimated from reported mean +/- SD
Egerton et al. (2020)	Atlantic salmon parr (Salmo salar)	Fish protein hydrolysate supplementation in an 80% plant-protein, low-fishmeal diet	PHP (80% plant protein + 5% fishmeal + 10% partly hydrolysed FPH)	PL (80% plant protein + 15% fishmeal)	Asparagine (μg/mL)	65.30 +/- 17.50	71.30 +/- 13.40	3	3	Hedges' g	-0.31	-1.61 to 0.99	Lower measured value with supplementation; unadjusted 95% CI included 0	Not applicable - effect size estimated from reported mean +/- SD
Egerton et al. (2020)	Atlantic salmon parr (Salmo salar)	Fish protein hydrolysate supplementation in an 80% plant-protein, low-fishmeal diet	PHP (80% plant protein + 5% fishmeal + 10% partly hydrolysed FPH)	PL (80% plant protein + 15% fishmeal)	Cysteine (μg/mL)	36.50 +/- 4.10	34.60 +/- 5.10	3	3	Hedges' g	0.33	-0.97 to 1.63	Higher measured value with supplementation; unadjusted 95% CI included 0	Not applicable - effect size estimated from reported mean +/- SD
Egerton et al. (2020)	Atlantic salmon parr (Salmo salar)	Fish protein hydrolysate supplementation in an 80% plant-protein, low-fishmeal diet	PHP (80% plant protein + 5% fishmeal + 10% partly hydrolysed FPH)	PL (80% plant protein + 15% fishmeal)	GABA (μg/mL)	13.20 +/- 2.10	15.80 +/- 3.00	3	3	Hedges' g	-0.8	-2.20 to 0.59	Lower measured value with supplementation; unadjusted 95% CI included 0	Not applicable - effect size estimated from reported mean +/- SD
Egerton et al. (2020)	Atlantic salmon parr (Salmo salar)	Fish protein hydrolysate supplementation in an 80% plant-protein, low-fishmeal diet	PHP (80% plant protein + 5% fishmeal + 10% partly hydrolysed FPH)	PL (80% plant protein + 15% fishmeal)	Glutamic acid (μg/mL)	269.00 +/- 32.80	238.70 +/- 43.90	3	3	Hedges' g	0.63	-0.73 to 1.98	Higher measured value with supplementation; unadjusted 95% CI included 0	Not applicable - effect size estimated from reported mean +/- SD

Study	Species	Strategy	Intervention	Comparator	Outcome and unit	Intervention mean +/- SD	Comparator mean +/- SD	n intervention (tanks)	n comparator (tanks)	Effect-size metric	Effect size	95% CI	Interpretation	Reason not estimated
Egerton et al. (2020)	Atlantic salmon parr (Salmo salar)	Fish protein hydrolysate supplementation in an 80% plant-protein, low-fishmeal diet	PHP (80% plant protein + 5% fishmeal + 10% partly hydrolysed FPH)	PL (80% plant protein + 15% fishmeal)	Glycine (µg/mL)	175.80 +/- 19.20	174.70 +/- 31.10	3	3	Hedges' g	0.03	-1.25 to 1.31	Higher measured value with supplementation; unadjusted 95% CI included 0	Not applicable - effect size estimated from reported mean +/- SD
Egerton et al. (2020)	Atlantic salmon parr (Salmo salar)	Fish protein hydrolysate supplementation in an 80% plant-protein, low-fishmeal diet	PHP (80% plant protein + 5% fishmeal + 10% partly hydrolysed FPH)	PL (80% plant protein + 15% fishmeal)	Proline (µg/mL)	180.80 +/- 18.80	152.10 +/- 25.50	3	3	Hedges' g	1.02	-0.44 to 2.49	Higher measured value with supplementation; unadjusted 95% CI included 0	Not applicable - effect size estimated from reported mean +/- SD
Egerton et al. (2020)	Atlantic salmon parr (Salmo salar)	Fish protein hydrolysate supplementation in an 80% plant-protein, low-fishmeal diet	PHP (80% plant protein + 5% fishmeal + 10% partly hydrolysed FPH)	PL (80% plant protein + 15% fishmeal)	Serine (µg/mL)	113.80 +/- 15.90	103.70 +/- 17.70	3	3	Hedges' g	0.48	-0.84 to 1.80	Higher measured value with supplementation; unadjusted 95% CI included 0	Not applicable - effect size estimated from reported mean +/- SD
Egerton et al. (2020)	Atlantic salmon parr (Salmo salar)	Fish protein hydrolysate supplementation in an 80% plant-protein, low-fishmeal diet	PHP (80% plant protein + 5% fishmeal + 10% partly hydrolysed FPH)	PL (80% plant protein + 15% fishmeal)	Taurine (µg/mL)	913.50 +/- 159.00	885.80 +/- 140.30	3	3	Hedges' g	0.15	-1.14 to 1.43	Higher measured value with supplementation; unadjusted 95% CI included 0	Not applicable - effect size estimated from reported mean +/- SD
Egerton et al. (2020)	Atlantic salmon parr (Salmo salar)	Fish protein hydrolysate supplementation in an 80% plant-protein, low-fishmeal diet	PHP (80% plant protein + 5% fishmeal + 10% partly hydrolysed FPH)	PL (80% plant protein + 15% fishmeal)	Tyrosine (µg/mL)	41.20 +/- 10.00	26.80 +/- 8.30	3	3	Hedges' g	1.25	-0.29 to 2.80	Higher measured value with supplementation; unadjusted 95% CI included 0	Not applicable - effect size estimated from reported mean +/- SD
Egerton et al. (2020)	Atlantic salmon parr (Salmo salar)	Fish protein hydrolysate supplementation in an 80% plant-protein, low-fishmeal diet	PHP (80% plant protein + 5% fishmeal + 10% partly hydrolysed FPH)	PL (80% plant protein + 15% fishmeal)	Arginine (µg/mL)	152.90 +/- 30.10	131.60 +/- 16.10	3	3	Hedges' g	0.71	-0.66 to 2.08	Higher measured value with supplementation; unadjusted 95% CI included 0	Not applicable - effect size estimated from reported mean +/- SD
Egerton et al. (2020)	Atlantic salmon parr (Salmo salar)	Fish protein hydrolysate supplementation in an 80% plant-protein, low-fishmeal diet	PHP (80% plant protein + 5% fishmeal + 10% partly hydrolysed FPH)	PL (80% plant protein + 15% fishmeal)	Histidine (µg/mL)	39.20 +/- 9.20	35.70 +/- 7.00	3	3	Hedges' g	0.34	-0.96 to 1.64	Higher measured value with supplementation; unadjusted 95% CI included 0	Not applicable - effect size estimated from reported mean +/- SD
Egerton et al. (2020)	Atlantic salmon parr (Salmo salar)	Fish protein hydrolysate supplementation in an 80% plant-protein, low-fishmeal diet	PHP (80% plant protein + 5% fishmeal + 10% partly hydrolysed FPH)	PL (80% plant protein + 15% fishmeal)	Isoleucine (µg/mL)	61.40 +/- 12.30	42.00 +/- 9.50	3	3	Hedges' g	1.41	-0.20 to 3.02	Higher measured value with supplementation; unadjusted 95% CI included 0	Not applicable - effect size estimated from reported mean +/- SD
Egerton et al. (2020)	Atlantic salmon parr (Salmo salar)	Fish protein hydrolysate supplementation in an 80% plant-protein, low-fishmeal diet	PHP (80% plant protein + 5% fishmeal + 10% partly hydrolysed FPH)	PL (80% plant protein + 15% fishmeal)	Leucine (µg/mL)	116.80 +/- 22.50	84.70 +/- 18.20	3	3	Hedges' g	1.25	-0.29 to 2.80	Higher measured value with supplementation; unadjusted 95% CI included 0	Not applicable - effect size estimated from reported mean +/- SD
Egerton et al. (2020)	Atlantic salmon parr (Salmo salar)	Fish protein hydrolysate supplementation in an 80% plant-protein, low-fishmeal diet	PHP (80% plant protein + 5% fishmeal + 10% partly hydrolysed FPH)	PL (80% plant protein + 15% fishmeal)	Lysine (µg/mL)	472.50 +/- 47.50	415.50 +/- 57.40	3	3	Hedges' g	0.87	-0.55 to 2.28	Higher measured value with supplementation; unadjusted 95% CI included 0	Not applicable - effect size estimated from reported mean +/- SD
Egerton et al. (2020)	Atlantic salmon parr (Salmo salar)	Fish protein hydrolysate supplementation in an 80% plant-protein, low-fishmeal diet	PHP (80% plant protein + 5% fishmeal + 10% partly hydrolysed FPH)	PL (80% plant protein + 15% fishmeal)	Methionine (µg/mL)	28.60 +/- 7.20	20.50 +/- 7.40	3	3	Hedges' g	0.89	-0.53 to 2.31	Higher measured value with supplementation; unadjusted 95% CI included 0	Not applicable - effect size estimated from reported mean +/- SD
Egerton et al. (2020)	Atlantic salmon parr (Salmo salar)	Fish protein hydrolysate supplementation in an 80% plant-protein, low-fishmeal diet	PHP (80% plant protein + 5% fishmeal + 10% partly hydrolysed FPH)	PL (80% plant protein + 15% fishmeal)	Phenylalanine (µg/mL)	58.90 +/- 23.20	45.60 +/- 11.90	3	3	Hedges' g	0.58	-0.76 to 1.92	Higher measured value with supplementation; unadjusted 95% CI included 0	Not applicable - effect size estimated from reported mean +/- SD
Egerton et al. (2020)	Atlantic salmon parr (Salmo salar)	Fish protein hydrolysate supplementation in an 80% plant-protein, low-fishmeal diet	PHP (80% plant protein + 5% fishmeal + 10% partly hydrolysed FPH)	PL (80% plant protein + 15% fishmeal)	Threonine (µg/mL)	119.50 +/- 17.60	99.20 +/- 16.20	3	3	Hedges' g	0.96	-0.48 to 2.40	Higher measured value with supplementation; unadjusted 95% CI included 0	Not applicable - effect size estimated from reported mean +/- SD
Egerton et al. (2020)	Atlantic salmon parr (Salmo salar)	Fish protein hydrolysate supplementation in an 80% plant-protein, low-fishmeal diet	PHP (80% plant protein + 5% fishmeal + 10% partly hydrolysed FPH)	PL (80% plant protein + 15% fishmeal)	Tryptophan (µg/mL)	9.500 +/- 1.700	8.900 +/- 4.000	3	3	Hedges' g	0.16	-1.13 to 1.44	Higher measured value with supplementation; unadjusted 95% CI included 0	Not applicable - effect size estimated from reported mean +/- SD
Egerton et al. (2020)	Atlantic salmon parr (Salmo salar)	Fish protein hydrolysate supplementation in an 80% plant-protein, low-fishmeal diet	PHP (80% plant protein + 5% fishmeal + 10% partly hydrolysed FPH)	PL (80% plant protein + 15% fishmeal)	Valine (µg/mL)	177.20 +/- 26.00	143.80 +/- 25.20	3	3	Hedges' g	1.04	-0.43 to 2.51	Higher measured value with supplementation; unadjusted 95% CI included 0	Not applicable - effect size estimated from reported mean +/- SD
Egerton et al. (2020)	Atlantic salmon parr (Salmo salar)	Fish protein hydrolysate supplementation in an 80% plant-protein, low-fishmeal diet	PHP (80% plant protein + 5% fishmeal + 10% partly hydrolysed FPH)	PL (80% plant protein + 15% fishmeal)	Cystic acid (µg/mL)	4.600 +/- 0.700	4.000 +/- 0.900	3	3	Hedges' g	0.6	-0.75 to 1.94	Higher measured value with supplementation; unadjusted 95% CI included 0	Not applicable - effect size estimated from reported mean +/- SD
Egerton et al. (2020)	Atlantic salmon parr (Salmo salar)	Fish protein hydrolysate supplementation in an 80% plant-protein, low-fishmeal diet	PHP (80% plant protein + 5% fishmeal + 10% partly hydrolysed FPH)	PL (80% plant protein + 15% fishmeal)	Total free amino acids (µg/mL)	3357.40 +/- 298.10	3000.80 +/- 402.80	3	3	Hedges' g	0.81	-0.59 to 2.20	Higher measured value with supplementation; unadjusted 95% CI included 0	Not applicable - effect size estimated from reported mean +/- SD
Egerton et al. (2020)	Atlantic salmon parr (Salmo salar)	Fish protein hydrolysate supplementation in an 80% plant-protein, low-fishmeal diet	SPH (80% plant protein + 5% fishmeal + 10% soluble FPH)	PL (80% plant protein + 15% fishmeal)	Feed intake (g/fish)	27.43 +/- 0.50	25.44 +/- 1.88	3	3	Hedges' g	1.16	-0.35 to 2.67	Higher measured value with supplementation; unadjusted 95% CI included 0	Not applicable - effect size estimated from reported mean +/- SD
Egerton et al. (2020)	Atlantic salmon parr (Salmo salar)	Fish protein hydrolysate supplementation in an 80% plant-protein, low-fishmeal diet	SPH (80% plant protein + 5% fishmeal + 10% soluble FPH)	PL (80% plant protein + 15% fishmeal)	Feed conversion ratio	1.050 +/- 0.090	1.040 +/- 0.060	3	3	Hedges' g (direction reversed)	-0.1	-1.39 to 1.18	Higher, less favorable value with supplementation; unadjusted 95% CI included 0	Not applicable - effect size estimated from reported mean +/- SD
Egerton et al. (2020)	Atlantic salmon parr (Salmo salar)	Fish protein hydrolysate supplementation in an 80% plant-protein, low-fishmeal diet	SPH (80% plant protein + 5% fishmeal + 10% soluble FPH)	PL (80% plant protein + 15% fishmeal)	Protein productive value	2.520 +/- 0.190	2.670 +/- 0.190	3	3	Hedges' g	-0.63	-1.98 to 0.72	Lower, less favorable value with supplementation; unadjusted 95% CI included 0	Not applicable - effect size estimated from reported mean +/- SD
Egerton et al. (2020)	Atlantic salmon parr (Salmo salar)	Fish protein hydrolysate supplementation in an 80% plant-protein, low-fishmeal diet	SPH (80% plant protein + 5% fishmeal + 10% soluble FPH)	PL (80% plant protein + 15% fishmeal)	Protein efficiency ratio	2.190 +/- 0.200	2.210 +/- 0.130	3	3	Hedges' g	-0.09	-1.38 to 1.19	Lower, less favorable value with supplementation; unadjusted 95% CI included 0	Not applicable - effect size estimated from reported mean +/- SD
Egerton et al. (2020)	Atlantic salmon parr (Salmo salar)	Fish protein hydrolysate supplementation in an 80% plant-protein, low-fishmeal diet	SPH (80% plant protein + 5% fishmeal + 10% soluble FPH)	PL (80% plant protein + 15% fishmeal)	Liver weight (mg)	484.21 +/- 30.29	425.29 +/- 25.57	3	3	Hedges' g	1.68	-0.05 to 3.41	Higher measured value with supplementation; unadjusted 95% CI included 0	Not applicable - effect size estimated from reported mean +/- SD
Egerton et al. (2020)	Atlantic salmon parr (Salmo salar)	Fish protein hydrolysate supplementation in an 80% plant-protein, low-fishmeal diet	SPH (80% plant protein + 5% fishmeal + 10% soluble FPH)	PL (80% plant protein + 15% fishmeal)	Hepatosomatic index (%)	1.350 +/- 0.050	1.250 +/- 0.120	3	3	Hedges' g	0.87	-0.54 to 2.29	Higher measured value with supplementation; unadjusted 95% CI included 0	Not applicable - effect size estimated from reported mean +/- SD
Egerton et al. (2020)	Atlantic salmon parr (Salmo salar)	Fish protein hydrolysate supplementation in an 80% plant-protein, low-fishmeal diet	SPH (80% plant protein + 5% fishmeal + 10% soluble FPH)	PL (80% plant protein + 15% fishmeal)	Moisture (% wet basis)	74.24 +/- 3.76	72.81 +/- 3.67	3	3	Hedges' g	0.31	-0.99 to 1.61	Higher measured value with supplementation; unadjusted 95% CI included 0	Not applicable - effect size estimated from reported mean +/- SD

Study	Species	Strategy	Intervention	Comparator	Outcome and unit	Intervention mean +/- SD	Comparator mean +/- SD	n intervention (tanks)	n comparator (tanks)	Effect-size metric	Effect size	95% CI	Interpretation	Reason not estimated
Egerton et al. (2020)	Atlantic salmon parr (Salmo salar)	Fish protein hydrolysate supplementation in an 80% plant-protein, low-fishmeal diet	SPH (80% plant protein + 5% fishmeal + 10% soluble FPH)	PL (80% plant protein + 15% fishmeal)	Crude protein (% wet basis)	16.76 +/- 0.32	16.82 +/- 0.23	3	3	Hedges' g	-0.17	-1.46 to 1.11	Lower measured value with supplementation; unadjusted 95% CI included 0	Not applicable - effect size estimated from reported mean +/- SD
Egerton et al. (2020)	Atlantic salmon parr (Salmo salar)	Fish protein hydrolysate supplementation in an 80% plant-protein, low-fishmeal diet	SPH (80% plant protein + 5% fishmeal + 10% soluble FPH)	PL (80% plant protein + 15% fishmeal)	Crude lipid (% wet basis)	9.550 +/- 0.810	10.14 +/- 0.78	3	3	Hedges' g	-0.59	-1.94 to 0.75	Lower measured value with supplementation; unadjusted 95% CI included 0	Not applicable - effect size estimated from reported mean +/- SD
Egerton et al. (2020)	Atlantic salmon parr (Salmo salar)	Fish protein hydrolysate supplementation in an 80% plant-protein, low-fishmeal diet	SPH (80% plant protein + 5% fishmeal + 10% soluble FPH)	PL (80% plant protein + 15% fishmeal)	Ash (% wet basis)	1.630 +/- 0.100	1.740 +/- 0.110	3	3	Hedges' g	-0.84	-2.24 to 0.57	Lower measured value with supplementation; unadjusted 95% CI included 0	Not applicable - effect size estimated from reported mean +/- SD
Egerton et al. (2020)	Atlantic salmon parr (Salmo salar)	Fish protein hydrolysate supplementation in an 80% plant-protein, low-fishmeal diet	SPH (80% plant protein + 5% fishmeal + 10% soluble FPH)	PL (80% plant protein + 15% fishmeal)	Villus height (µm)	514.30 +/- 161.04	448.30 +/- 227.62	3	3	Hedges' g	0.27	-1.03 to 1.56	Higher, more favorable value with supplementation; unadjusted 95% CI included 0	Not applicable - effect size estimated from reported mean +/- SD
Egerton et al. (2020)	Atlantic salmon parr (Salmo salar)	Fish protein hydrolysate supplementation in an 80% plant-protein, low-fishmeal diet	SPH (80% plant protein + 5% fishmeal + 10% soluble FPH)	PL (80% plant protein + 15% fishmeal)	Villus width (µm)	91.67 +/- 20.33	76.44 +/- 22.63	3	3	Hedges' g	0.57	-0.77 to 1.91	Higher, more favorable value with supplementation; unadjusted 95% CI included 0	Not applicable - effect size estimated from reported mean +/- SD
Egerton et al. (2020)	Atlantic salmon parr (Salmo salar)	Fish protein hydrolysate supplementation in an 80% plant-protein, low-fishmeal diet	SPH (80% plant protein + 5% fishmeal + 10% soluble FPH)	PL (80% plant protein + 15% fishmeal)	Villus height-to-width ratio	5.680 +/- 1.600	6.210 +/- 3.350	3	3	Hedges' g	-0.16	-1.45 to 1.12	Lower measured value with supplementation; unadjusted 95% CI included 0	Not applicable - effect size estimated from reported mean +/- SD
Egerton et al. (2020)	Atlantic salmon parr (Salmo salar)	Fish protein hydrolysate supplementation in an 80% plant-protein, low-fishmeal diet	SPH (80% plant protein + 5% fishmeal + 10% soluble FPH)	PL (80% plant protein + 15% fishmeal)	Intestinal wall width (µm)	84.93 +/- 23.62	63.18 +/- 29.97	3	3	Hedges' g	0.64	-0.71 to 2.00	Higher measured value with supplementation; unadjusted 95% CI included 0	Not applicable - effect size estimated from reported mean +/- SD
Egerton et al. (2020)	Atlantic salmon parr (Salmo salar)	Fish protein hydrolysate supplementation in an 80% plant-protein, low-fishmeal diet	SPH (80% plant protein + 5% fishmeal + 10% soluble FPH)	PL (80% plant protein + 15% fishmeal)	Intestine diameter (mm)	2.430 +/- 0.540	2.100 +/- 0.840	3	3	Hedges' g	0.37	-0.93 to 1.68	Higher measured value with supplementation; unadjusted 95% CI included 0	Not applicable - effect size estimated from reported mean +/- SD
Egerton et al. (2020)	Atlantic salmon parr (Salmo salar)	Fish protein hydrolysate supplementation in an 80% plant-protein, low-fishmeal diet	SPH (80% plant protein + 5% fishmeal + 10% soluble FPH)	PL (80% plant protein + 15% fishmeal)	Interior lumen area (mm²)	1.680 +/- 0.710	1.630 +/- 1.300	3	3	Hedges' g	0.04	-1.24 to 1.32	Higher measured value with supplementation; unadjusted 95% CI included 0	Not applicable - effect size estimated from reported mean +/- SD
Egerton et al. (2020)	Atlantic salmon parr (Salmo salar)	Fish protein hydrolysate supplementation in an 80% plant-protein, low-fishmeal diet	SPH (80% plant protein + 5% fishmeal + 10% soluble FPH)	PL (80% plant protein + 15% fishmeal)	Internal perimeter (mm)	27.15 +/- 7.42	21.13 +/- 9.46	3	3	Hedges' g	0.57	-0.77 to 1.91	Higher measured value with supplementation; unadjusted 95% CI included 0	Not applicable - effect size estimated from reported mean +/- SD
Egerton et al. (2020)	Atlantic salmon parr (Salmo salar)	Fish protein hydrolysate supplementation in an 80% plant-protein, low-fishmeal diet	SPH (80% plant protein + 5% fishmeal + 10% soluble FPH)	PL (80% plant protein + 15% fishmeal)	External perimeter (mm)	7.480 +/- 1.500	6.590 +/- 2.470	3	3	Hedges' g	0.35	-0.95 to 1.65	Higher measured value with supplementation; unadjusted 95% CI included 0	Not applicable - effect size estimated from reported mean +/- SD
Egerton et al. (2020)	Atlantic salmon parr (Salmo salar)	Fish protein hydrolysate supplementation in an 80% plant-protein, low-fishmeal diet	SPH (80% plant protein + 5% fishmeal + 10% soluble FPH)	PL (80% plant protein + 15% fishmeal)	Perimeter ratio (absolute)	3.620 +/- 0.550	3.240 +/- 0.880	3	3	Hedges' g	0.41	-0.90 to 1.73	Higher measured value with supplementation; unadjusted 95% CI included 0	Not applicable - effect size estimated from reported mean +/- SD
Egerton et al. (2020)	Atlantic salmon parr (Salmo salar)	Fish protein hydrolysate supplementation in an 80% plant-protein, low-fishmeal diet	SPH (80% plant protein + 5% fishmeal + 10% soluble FPH)	PL (80% plant protein + 15% fishmeal)	Alanine (µg/mL)	314.60 +/- 30.40	265.60 +/- 34.70	3	3	Hedges' g	1.2	-0.33 to 2.73	Higher measured value with supplementation; unadjusted 95% CI included 0	Not applicable - effect size estimated from reported mean +/- SD
Egerton et al. (2020)	Atlantic salmon parr (Salmo salar)	Fish protein hydrolysate supplementation in an 80% plant-protein, low-fishmeal diet	SPH (80% plant protein + 5% fishmeal + 10% soluble FPH)	PL (80% plant protein + 15% fishmeal)	Asparagine (µg/mL)	76.10 +/- 11.30	71.30 +/- 13.40	3	3	Hedges' g	0.31	-0.99 to 1.61	Higher measured value with supplementation; unadjusted 95% CI included 0	Not applicable - effect size estimated from reported mean +/- SD
Egerton et al. (2020)	Atlantic salmon parr (Salmo salar)	Fish protein hydrolysate supplementation in an 80% plant-protein, low-fishmeal diet	SPH (80% plant protein + 5% fishmeal + 10% soluble FPH)	PL (80% plant protein + 15% fishmeal)	Cysteine (µg/mL)	39.50 +/- 5.60	34.60 +/- 5.10	3	3	Hedges' g	0.73	-0.65 to 2.11	Higher measured value with supplementation; unadjusted 95% CI included 0	Not applicable - effect size estimated from reported mean +/- SD
Egerton et al. (2020)	Atlantic salmon parr (Salmo salar)	Fish protein hydrolysate supplementation in an 80% plant-protein, low-fishmeal diet	SPH (80% plant protein + 5% fishmeal + 10% soluble FPH)	PL (80% plant protein + 15% fishmeal)	GABA (µg/mL)	13.50 +/- 1.60	15.80 +/- 3.00	3	3	Hedges' g	-0.77	-2.15 to 0.62	Lower measured value with supplementation; unadjusted 95% CI included 0	Not applicable - effect size estimated from reported mean +/- SD
Egerton et al. (2020)	Atlantic salmon parr (Salmo salar)	Fish protein hydrolysate supplementation in an 80% plant-protein, low-fishmeal diet	SPH (80% plant protein + 5% fishmeal + 10% soluble FPH)	PL (80% plant protein + 15% fishmeal)	Glutamic acid (µg/mL)	248.90 +/- 26.00	238.70 +/- 43.90	3	3	Hedges' g	0.23	-1.06 to 1.52	Higher measured value with supplementation; unadjusted 95% CI included 0	Not applicable - effect size estimated from reported mean +/- SD
Egerton et al. (2020)	Atlantic salmon parr (Salmo salar)	Fish protein hydrolysate supplementation in an 80% plant-protein, low-fishmeal diet	SPH (80% plant protein + 5% fishmeal + 10% soluble FPH)	PL (80% plant protein + 15% fishmeal)	Glycine (µg/mL)	198.70 +/- 19.50	174.70 +/- 31.10	3	3	Hedges' g	0.74	-0.64 to 2.12	Higher measured value with supplementation; unadjusted 95% CI included 0	Not applicable - effect size estimated from reported mean +/- SD
Egerton et al. (2020)	Atlantic salmon parr (Salmo salar)	Fish protein hydrolysate supplementation in an 80% plant-protein, low-fishmeal diet	SPH (80% plant protein + 5% fishmeal + 10% soluble FPH)	PL (80% plant protein + 15% fishmeal)	Proline (µg/mL)	194.10 +/- 16.50	152.10 +/- 25.50	3	3	Hedges' g	1.56	-0.11 to 3.24	Higher measured value with supplementation; unadjusted 95% CI included 0	Not applicable - effect size estimated from reported mean +/- SD
Egerton et al. (2020)	Atlantic salmon parr (Salmo salar)	Fish protein hydrolysate supplementation in an 80% plant-protein, low-fishmeal diet	SPH (80% plant protein + 5% fishmeal + 10% soluble FPH)	PL (80% plant protein + 15% fishmeal)	Serine (µg/mL)	124.00 +/- 8.90	103.70 +/- 17.70	3	3	Hedges' g	1.16	-0.35 to 2.67	Higher measured value with supplementation; unadjusted 95% CI included 0	Not applicable - effect size estimated from reported mean +/- SD
Egerton et al. (2020)	Atlantic salmon parr (Salmo salar)	Fish protein hydrolysate supplementation in an 80% plant-protein, low-fishmeal diet	SPH (80% plant protein + 5% fishmeal + 10% soluble FPH)	PL (80% plant protein + 15% fishmeal)	Taurine (µg/mL)	1065.30 +/- 92.50	885.80 +/- 140.30	3	3	Hedges' g	1.21	-0.32 to 2.74	Higher measured value with supplementation; unadjusted 95% CI included 0	Not applicable - effect size estimated from reported mean +/- SD
Egerton et al. (2020)	Atlantic salmon parr (Salmo salar)	Fish protein hydrolysate supplementation in an 80% plant-protein, low-fishmeal diet	SPH (80% plant protein + 5% fishmeal + 10% soluble FPH)	PL (80% plant protein + 15% fishmeal)	Tyrosine (µg/mL)	32.10 +/- 4.40	26.80 +/- 8.30	3	3	Hedges' g	0.64	-0.72 to 1.99	Higher measured value with supplementation; unadjusted 95% CI included 0	Not applicable - effect size estimated from reported mean +/- SD
Egerton et al. (2020)	Atlantic salmon parr (Salmo salar)	Fish protein hydrolysate supplementation in an 80% plant-protein, low-fishmeal diet	SPH (80% plant protein + 5% fishmeal + 10% soluble FPH)	PL (80% plant protein + 15% fishmeal)	Arginine (µg/mL)	155.50 +/- 13.00	131.60 +/- 16.10	3	3	Hedges' g	1.31	-0.26 to 2.87	Higher measured value with supplementation; unadjusted 95% CI included 0	Not applicable - effect size estimated from reported mean +/- SD
Egerton et al. (2020)	Atlantic salmon parr (Salmo salar)	Fish protein hydrolysate supplementation in an 80% plant-protein, low-fishmeal diet	SPH (80% plant protein + 5% fishmeal + 10% soluble FPH)	PL (80% plant protein + 15% fishmeal)	Histidine (µg/mL)	38.60 +/- 4.10	35.70 +/- 7.00	3	3	Hedges' g	0.4	-0.91 to 1.72	Higher measured value with supplementation; unadjusted 95% CI included 0	Not applicable - effect size estimated from reported mean +/- SD

Study	Species	Strategy	Intervention	Comparator	Outcome and unit	Intervention mean +/- SD	Comparator mean +/- SD	n intervention (tanks)	n comparator (tanks)	Effect-size metric	Effect size	95% CI	Interpretation	Reason not estimated
Egerton et al. (2020)	Atlantic salmon parr (Salmo salar)	Fish protein hydrolysate supplementation in an 80% plant-protein, low-fishmeal diet	SPH (80% plant protein + 5% fishmeal + 10% soluble FPH)	PL (80% plant protein + 15% fishmeal)	Isoleucine (µg/mL)	46.30 +/- 5.00	42.00 +/- 9.50	3	3	Hedges' g	0.45	-0.87 to 1.77	Higher measured value with supplementation; unadjusted 95% CI included 0	Not applicable - effect size estimated from reported mean +/- SD
Egerton et al. (2020)	Atlantic salmon parr (Salmo salar)	Fish protein hydrolysate supplementation in an 80% plant-protein, low-fishmeal diet	SPH (80% plant protein + 5% fishmeal + 10% soluble FPH)	PL (80% plant protein + 15% fishmeal)	Leucine (µg/mL)	99.70 +/- 10.80	84.70 +/- 18.20	3	3	Hedges' g	0.8	-0.59 to 2.20	Higher measured value with supplementation; unadjusted 95% CI included 0	Not applicable - effect size estimated from reported mean +/- SD
Egerton et al. (2020)	Atlantic salmon parr (Salmo salar)	Fish protein hydrolysate supplementation in an 80% plant-protein, low-fishmeal diet	SPH (80% plant protein + 5% fishmeal + 10% soluble FPH)	PL (80% plant protein + 15% fishmeal)	Lysine (µg/mL)	505.50 +/- 36.90	415.50 +/- 57.40	3	3	Hedges' g	1.49	-0.15 to 3.14	Higher measured value with supplementation; unadjusted 95% CI included 0	Not applicable - effect size estimated from reported mean +/- SD
Egerton et al. (2020)	Atlantic salmon parr (Salmo salar)	Fish protein hydrolysate supplementation in an 80% plant-protein, low-fishmeal diet	SPH (80% plant protein + 5% fishmeal + 10% soluble FPH)	PL (80% plant protein + 15% fishmeal)	Methionine (µg/mL)	31.50 +/- 8.30	20.50 +/- 7.40	3	3	Hedges' g	1.12	-0.38 to 2.62	Higher measured value with supplementation; unadjusted 95% CI included 0	Not applicable - effect size estimated from reported mean +/- SD
Egerton et al. (2020)	Atlantic salmon parr (Salmo salar)	Fish protein hydrolysate supplementation in an 80% plant-protein, low-fishmeal diet	SPH (80% plant protein + 5% fishmeal + 10% soluble FPH)	PL (80% plant protein + 15% fishmeal)	Phenylalanine (µg/mL)	57.80 +/- 12.20	45.60 +/- 11.90	3	3	Hedges' g	0.81	-0.59 to 2.21	Higher measured value with supplementation; unadjusted 95% CI included 0	Not applicable - effect size estimated from reported mean +/- SD
Egerton et al. (2020)	Atlantic salmon parr (Salmo salar)	Fish protein hydrolysate supplementation in an 80% plant-protein, low-fishmeal diet	SPH (80% plant protein + 5% fishmeal + 10% soluble FPH)	PL (80% plant protein + 15% fishmeal)	Threonine (µg/mL)	118.00 +/- 9.40	99.20 +/- 16.20	3	3	Hedges' g	1.14	-0.37 to 2.64	Higher measured value with supplementation; unadjusted 95% CI included 0	Not applicable - effect size estimated from reported mean +/- SD
Egerton et al. (2020)	Atlantic salmon parr (Salmo salar)	Fish protein hydrolysate supplementation in an 80% plant-protein, low-fishmeal diet	SPH (80% plant protein + 5% fishmeal + 10% soluble FPH)	PL (80% plant protein + 15% fishmeal)	Tryptophan (µg/mL)	10.90 +/- 2.20	8.900 +/- 4.000	3	3	Hedges' g	0.5	-0.83 to 1.82	Higher measured value with supplementation; unadjusted 95% CI included 0	Not applicable - effect size estimated from reported mean +/- SD
Egerton et al. (2020)	Atlantic salmon parr (Salmo salar)	Fish protein hydrolysate supplementation in an 80% plant-protein, low-fishmeal diet	SPH (80% plant protein + 5% fishmeal + 10% soluble FPH)	PL (80% plant protein + 15% fishmeal)	Valine (µg/mL)	158.00 +/- 17.40	143.80 +/- 25.20	3	3	Hedges' g	0.52	-0.81 to 1.86	Higher measured value with supplementation; unadjusted 95% CI included 0	Not applicable - effect size estimated from reported mean +/- SD
Egerton et al. (2020)	Atlantic salmon parr (Salmo salar)	Fish protein hydrolysate supplementation in an 80% plant-protein, low-fishmeal diet	SPH (80% plant protein + 5% fishmeal + 10% soluble FPH)	PL (80% plant protein + 15% fishmeal)	Cystic acid (µg/mL)	5.100 +/- 0.900	4.000 +/- 0.900	3	3	Hedges' g	0.98	-0.47 to 2.43	Higher measured value with supplementation; unadjusted 95% CI included 0	Not applicable - effect size estimated from reported mean +/- SD
Egerton et al. (2020)	Atlantic salmon parr (Salmo salar)	Fish protein hydrolysate supplementation in an 80% plant-protein, low-fishmeal diet	SPH (80% plant protein + 5% fishmeal + 10% soluble FPH)	PL (80% plant protein + 15% fishmeal)	Total free amino acids (µg/mL)	3533.60 +/- 205.30	3000.80 +/- 402.80	3	3	Hedges' g	1.33	-0.25 to 2.91	Higher measured value with supplementation; unadjusted 95% CI included 0	Not applicable - effect size estimated from reported mean +/- SD
Egerton et al. (2020)	Atlantic salmon parr (Salmo salar)	Fish protein hydrolysate supplementation in an 80% plant-protein, low-fishmeal diet	PHP (80% plant protein + 5% fishmeal + 10% partly hydrolysed FPH)	FM (35% fishmeal reference diet)	Feed intake (g/fish)	24.81 +/- 1.03	26.55 +/- 1.17	3	3	Hedges' g	-1.26	-2.81 to 0.29	Lower measured value with supplementation; unadjusted 95% CI included 0	Not applicable - effect size estimated from reported mean +/- SD
Egerton et al. (2020)	Atlantic salmon parr (Salmo salar)	Fish protein hydrolysate supplementation in an 80% plant-protein, low-fishmeal diet	PHP (80% plant protein + 5% fishmeal + 10% partly hydrolysed FPH)	FM (35% fishmeal reference diet)	Feed conversion ratio	0.880 +/- 0.070	0.920 +/- 0.090	3	3	Hedges' g (direction reversed)	0.4	-0.91 to 1.71	Lower, more favorable value with supplementation; unadjusted 95% CI included 0	Not applicable - effect size estimated from reported mean +/- SD
Egerton et al. (2020)	Atlantic salmon parr (Salmo salar)	Fish protein hydrolysate supplementation in an 80% plant-protein, low-fishmeal diet	PHP (80% plant protein + 5% fishmeal + 10% partly hydrolysed FPH)	FM (35% fishmeal reference diet)	Protein productive value	3.080 +/- 0.210	3.340 +/- 0.430	3	3	Hedges' g	-0.61	-1.96 to 0.73	Lower, less favorable value with supplementation; unadjusted 95% CI included 0	Not applicable - effect size estimated from reported mean +/- SD
Egerton et al. (2020)	Atlantic salmon parr (Salmo salar)	Fish protein hydrolysate supplementation in an 80% plant-protein, low-fishmeal diet	PHP (80% plant protein + 5% fishmeal + 10% partly hydrolysed FPH)	FM (35% fishmeal reference diet)	Protein efficiency ratio	2.570 +/- 0.130	2.480 +/- 0.260	3	3	Hedges' g	0.35	-0.95 to 1.65	Higher, more favorable value with supplementation; unadjusted 95% CI included 0	Not applicable - effect size estimated from reported mean +/- SD
Egerton et al. (2020)	Atlantic salmon parr (Salmo salar)	Fish protein hydrolysate supplementation in an 80% plant-protein, low-fishmeal diet	PHP (80% plant protein + 5% fishmeal + 10% partly hydrolysed FPH)	FM (35% fishmeal reference diet)	Liver weight (mg)	561.67 +/- 28.11	499.67 +/- 17.73	3	3	Hedges' g	2.11	0.17 to 4.05	Higher measured value with supplementation; unadjusted 95% CI excluded 0	Not applicable - effect size estimated from reported mean +/- SD
Egerton et al. (2020)	Atlantic salmon parr (Salmo salar)	Fish protein hydrolysate supplementation in an 80% plant-protein, low-fishmeal diet	PHP (80% plant protein + 5% fishmeal + 10% partly hydrolysed FPH)	FM (35% fishmeal reference diet)	Hepatosomatic index (%)	1.520 +/- 0.080	1.310 +/- 0.030	3	3	Hedges' g	2.78	0.47 to 5.09	Higher measured value with supplementation; unadjusted 95% CI excluded 0	Not applicable - effect size estimated from reported mean +/- SD
Egerton et al. (2020)	Atlantic salmon parr (Salmo salar)	Fish protein hydrolysate supplementation in an 80% plant-protein, low-fishmeal diet	PHP (80% plant protein + 5% fishmeal + 10% partly hydrolysed FPH)	FM (35% fishmeal reference diet)	Moisture (% wet basis)	72.12 +/- 3.17	70.43 +/- 3.15	3	3	Hedges' g	0.43	-0.89 to 1.74	Higher measured value with supplementation; unadjusted 95% CI included 0	Not applicable - effect size estimated from reported mean +/- SD
Egerton et al. (2020)	Atlantic salmon parr (Salmo salar)	Fish protein hydrolysate supplementation in an 80% plant-protein, low-fishmeal diet	PHP (80% plant protein + 5% fishmeal + 10% partly hydrolysed FPH)	FM (35% fishmeal reference diet)	Crude protein (% wet basis)	17.07 +/- 0.57	17.26 +/- 0.43	3	3	Hedges' g	-0.3	-1.60 to 1.00	Lower measured value with supplementation; unadjusted 95% CI included 0	Not applicable - effect size estimated from reported mean +/- SD
Egerton et al. (2020)	Atlantic salmon parr (Salmo salar)	Fish protein hydrolysate supplementation in an 80% plant-protein, low-fishmeal diet	PHP (80% plant protein + 5% fishmeal + 10% partly hydrolysed FPH)	FM (35% fishmeal reference diet)	Crude lipid (% wet basis)	10.07 +/- 0.79	10.31 +/- 0.67	3	3	Hedges' g	-0.26	-1.56 to 1.03	Lower measured value with supplementation; unadjusted 95% CI included 0	Not applicable - effect size estimated from reported mean +/- SD
Egerton et al. (2020)	Atlantic salmon parr (Salmo salar)	Fish protein hydrolysate supplementation in an 80% plant-protein, low-fishmeal diet	PHP (80% plant protein + 5% fishmeal + 10% partly hydrolysed FPH)	FM (35% fishmeal reference diet)	Ash (% wet basis)	1.580 +/- 0.120	2.140 +/- 0.180	3	3	Hedges' g	-2.93	-5.33 to -0.53	Lower measured value with supplementation; unadjusted 95% CI excluded 0	Not applicable - effect size estimated from reported mean +/- SD
Egerton et al. (2020)	Atlantic salmon parr (Salmo salar)	Fish protein hydrolysate supplementation in an 80% plant-protein, low-fishmeal diet	PHP (80% plant protein + 5% fishmeal + 10% partly hydrolysed FPH)	FM (35% fishmeal reference diet)	Villus height (µm)	435.12 +/- 159.02	473.67 +/- 130.92	3	3	Hedges' g	-0.21	-1.50 to 1.08	Lower, less favorable value with supplementation; unadjusted 95% CI included 0	Not applicable - effect size estimated from reported mean +/- SD
Egerton et al. (2020)	Atlantic salmon parr (Salmo salar)	Fish protein hydrolysate supplementation in an 80% plant-protein, low-fishmeal diet	PHP (80% plant protein + 5% fishmeal + 10% partly hydrolysed FPH)	FM (35% fishmeal reference diet)	Villus width (µm)	75.09 +/- 14.89	76.31 +/- 14.92	3	3	Hedges' g	-0.07	-1.35 to 1.22	Lower, less favorable value with supplementation; unadjusted 95% CI included 0	Not applicable - effect size estimated from reported mean +/- SD
Egerton et al. (2020)	Atlantic salmon parr (Salmo salar)	Fish protein hydrolysate supplementation in an 80% plant-protein, low-fishmeal diet	PHP (80% plant protein + 5% fishmeal + 10% partly hydrolysed FPH)	FM (35% fishmeal reference diet)	Villus height-to-width ratio	5.830 +/- 1.800	6.370 +/- 1.970	3	3	Hedges' g	-0.23	-1.52 to 1.06	Lower measured value with supplementation; unadjusted 95% CI included 0	Not applicable - effect size estimated from reported mean +/- SD
Egerton et al. (2020)	Atlantic salmon parr (Salmo salar)	Fish protein hydrolysate supplementation in an 80% plant-protein, low-fishmeal diet	PHP (80% plant protein + 5% fishmeal + 10% partly hydrolysed FPH)	FM (35% fishmeal reference diet)	Intestinal wall width (µm)	65.57 +/- 23.16	58.97 +/- 20.18	3	3	Hedges' g	0.24	-1.05 to 1.53	Higher measured value with supplementation; unadjusted 95% CI included 0	Not applicable - effect size estimated from reported mean +/- SD

Study	Species	Strategy	Intervention	Comparator	Outcome and unit	Intervention mean +/- SD	Comparator mean +/- SD	n intervention (tanks)	n comparator (tanks)	Effect-size metric	Effect size	95% CI	Interpretation	Reason not estimated
Egerton et al. (2020)	Atlantic salmon parr (Salmo salar)	Fish protein hydrolysate supplementation in an 80% plant-protein, low-fishmeal diet	PHP (80% plant protein + 5% fishmeal + 10% partly hydrolysed FPH)	FM (35% fishmeal reference diet)	Intestine diameter (mm)	2.080 +/- 0.500	2.230 +/- 0.560	3	3	Hedges' g	-0.23	-1.52 to 1.06	Lower measured value with supplementation; unadjusted 95% CI included 0	Not applicable - effect size estimated from reported mean +/- SD
Egerton et al. (2020)	Atlantic salmon parr (Salmo salar)	Fish protein hydrolysate supplementation in an 80% plant-protein, low-fishmeal diet	PHP (80% plant protein + 5% fishmeal + 10% partly hydrolysed FPH)	FM (35% fishmeal reference diet)	Interior lumen area (mm²)	1.460 +/- 0.680	1.900 +/- 1.180	3	3	Hedges' g	-0.37	-1.67 to 0.94	Lower measured value with supplementation; unadjusted 95% CI included 0	Not applicable - effect size estimated from reported mean +/- SD
Egerton et al. (2020)	Atlantic salmon parr (Salmo salar)	Fish protein hydrolysate supplementation in an 80% plant-protein, low-fishmeal diet	PHP (80% plant protein + 5% fishmeal + 10% partly hydrolysed FPH)	FM (35% fishmeal reference diet)	Internal perimeter (mm)	25.16 +/- 9.29	24.73 +/- 10.35	3	3	Hedges' g	0.03	-1.25 to 1.32	Higher measured value with supplementation; unadjusted 95% CI included 0	Not applicable - effect size estimated from reported mean +/- SD
Egerton et al. (2020)	Atlantic salmon parr (Salmo salar)	Fish protein hydrolysate supplementation in an 80% plant-protein, low-fishmeal diet	PHP (80% plant protein + 5% fishmeal + 10% partly hydrolysed FPH)	FM (35% fishmeal reference diet)	External perimeter (mm)	6.510 +/- 1.500	7.350 +/- 1.880	3	3	Hedges' g	-0.4	-1.70 to 0.91	Lower measured value with supplementation; unadjusted 95% CI included 0	Not applicable - effect size estimated from reported mean +/- SD
Egerton et al. (2020)	Atlantic salmon parr (Salmo salar)	Fish protein hydrolysate supplementation in an 80% plant-protein, low-fishmeal diet	PHP (80% plant protein + 5% fishmeal + 10% partly hydrolysed FPH)	FM (35% fishmeal reference diet)	Perimeter ratio (absolute)	3.770 +/- 0.620	3.340 +/- 0.810	3	3	Hedges' g	0.48	-0.85 to 1.80	Higher measured value with supplementation; unadjusted 95% CI included 0	Not applicable - effect size estimated from reported mean +/- SD
Egerton et al. (2020)	Atlantic salmon parr (Salmo salar)	Fish protein hydrolysate supplementation in an 80% plant-protein, low-fishmeal diet	PHP (80% plant protein + 5% fishmeal + 10% partly hydrolysed FPH)	FM (35% fishmeal reference diet)	Alanine (µg/mL)	307.10 +/- 30.00	236.90 +/- 34.60	3	3	Hedges' g	1.73	-0.02 to 3.49	Higher measured value with supplementation; unadjusted 95% CI included 0	Not applicable - effect size estimated from reported mean +/- SD
Egerton et al. (2020)	Atlantic salmon parr (Salmo salar)	Fish protein hydrolysate supplementation in an 80% plant-protein, low-fishmeal diet	PHP (80% plant protein + 5% fishmeal + 10% partly hydrolysed FPH)	FM (35% fishmeal reference diet)	Asparagine (µg/mL)	65.30 +/- 17.50	54.00 +/- 10.70	3	3	Hedges' g	0.62	-0.73 to 1.97	Higher measured value with supplementation; unadjusted 95% CI included 0	Not applicable - effect size estimated from reported mean +/- SD
Egerton et al. (2020)	Atlantic salmon parr (Salmo salar)	Fish protein hydrolysate supplementation in an 80% plant-protein, low-fishmeal diet	PHP (80% plant protein + 5% fishmeal + 10% partly hydrolysed FPH)	FM (35% fishmeal reference diet)	Cysteine (µg/mL)	36.50 +/- 4.10	35.40 +/- 7.70	3	3	Hedges' g	0.14	-1.14 to 1.43	Higher measured value with supplementation; unadjusted 95% CI included 0	Not applicable - effect size estimated from reported mean +/- SD
Egerton et al. (2020)	Atlantic salmon parr (Salmo salar)	Fish protein hydrolysate supplementation in an 80% plant-protein, low-fishmeal diet	PHP (80% plant protein + 5% fishmeal + 10% partly hydrolysed FPH)	FM (35% fishmeal reference diet)	GABA (µg/mL)	13.20 +/- 2.10	22.50 +/- 7.70	3	3	Hedges' g	-1.32	-2.89 to 0.25	Lower measured value with supplementation; unadjusted 95% CI included 0	Not applicable - effect size estimated from reported mean +/- SD
Egerton et al. (2020)	Atlantic salmon parr (Salmo salar)	Fish protein hydrolysate supplementation in an 80% plant-protein, low-fishmeal diet	PHP (80% plant protein + 5% fishmeal + 10% partly hydrolysed FPH)	FM (35% fishmeal reference diet)	Glutamic acid (µg/mL)	269.00 +/- 32.80	215.00 +/- 34.50	3	3	Hedges' g	1.28	-0.28 to 2.84	Higher measured value with supplementation; unadjusted 95% CI included 0	Not applicable - effect size estimated from reported mean +/- SD
Egerton et al. (2020)	Atlantic salmon parr (Salmo salar)	Fish protein hydrolysate supplementation in an 80% plant-protein, low-fishmeal diet	PHP (80% plant protein + 5% fishmeal + 10% partly hydrolysed FPH)	FM (35% fishmeal reference diet)	Glycine (µg/mL)	175.80 +/- 19.20	176.00 +/- 19.10	3	3	Hedges' g	-0.01	-1.29 to 1.27	Lower measured value with supplementation; unadjusted 95% CI included 0	Not applicable - effect size estimated from reported mean +/- SD
Egerton et al. (2020)	Atlantic salmon parr (Salmo salar)	Fish protein hydrolysate supplementation in an 80% plant-protein, low-fishmeal diet	PHP (80% plant protein + 5% fishmeal + 10% partly hydrolysed FPH)	FM (35% fishmeal reference diet)	Proline (µg/mL)	180.80 +/- 18.80	140.80 +/- 23.00	3	3	Hedges' g	1.52	-0.14 to 3.18	Higher measured value with supplementation; unadjusted 95% CI included 0	Not applicable - effect size estimated from reported mean +/- SD
Egerton et al. (2020)	Atlantic salmon parr (Salmo salar)	Fish protein hydrolysate supplementation in an 80% plant-protein, low-fishmeal diet	PHP (80% plant protein + 5% fishmeal + 10% partly hydrolysed FPH)	FM (35% fishmeal reference diet)	Serine (µg/mL)	113.80 +/- 15.90	104.20 +/- 10.40	3	3	Hedges' g	0.57	-0.77 to 1.91	Higher measured value with supplementation; unadjusted 95% CI included 0	Not applicable - effect size estimated from reported mean +/- SD
Egerton et al. (2020)	Atlantic salmon parr (Salmo salar)	Fish protein hydrolysate supplementation in an 80% plant-protein, low-fishmeal diet	PHP (80% plant protein + 5% fishmeal + 10% partly hydrolysed FPH)	FM (35% fishmeal reference diet)	Taurine (µg/mL)	913.50 +/- 159.00	1212.10 +/- 153.20	3	3	Hedges' g	-1.53	-3.19 to 0.13	Lower measured value with supplementation; unadjusted 95% CI included 0	Not applicable - effect size estimated from reported mean +/- SD
Egerton et al. (2020)	Atlantic salmon parr (Salmo salar)	Fish protein hydrolysate supplementation in an 80% plant-protein, low-fishmeal diet	PHP (80% plant protein + 5% fishmeal + 10% partly hydrolysed FPH)	FM (35% fishmeal reference diet)	Tyrosine (µg/mL)	41.20 +/- 10.00	26.10 +/- 4.40	3	3	Hedges' g	1.56	-0.11 to 3.24	Higher measured value with supplementation; unadjusted 95% CI included 0	Not applicable - effect size estimated from reported mean +/- SD
Egerton et al. (2020)	Atlantic salmon parr (Salmo salar)	Fish protein hydrolysate supplementation in an 80% plant-protein, low-fishmeal diet	PHP (80% plant protein + 5% fishmeal + 10% partly hydrolysed FPH)	FM (35% fishmeal reference diet)	Arginine (µg/mL)	152.90 +/- 30.10	116.10 +/- 37.20	3	3	Hedges' g	0.87	-0.55 to 2.29	Higher measured value with supplementation; unadjusted 95% CI included 0	Not applicable - effect size estimated from reported mean +/- SD
Egerton et al. (2020)	Atlantic salmon parr (Salmo salar)	Fish protein hydrolysate supplementation in an 80% plant-protein, low-fishmeal diet	PHP (80% plant protein + 5% fishmeal + 10% partly hydrolysed FPH)	FM (35% fishmeal reference diet)	Histidine (µg/mL)	39.20 +/- 9.20	27.20 +/- 4.10	3	3	Hedges' g	1.35	-0.24 to 2.93	Higher measured value with supplementation; unadjusted 95% CI included 0	Not applicable - effect size estimated from reported mean +/- SD
Egerton et al. (2020)	Atlantic salmon parr (Salmo salar)	Fish protein hydrolysate supplementation in an 80% plant-protein, low-fishmeal diet	PHP (80% plant protein + 5% fishmeal + 10% partly hydrolysed FPH)	FM (35% fishmeal reference diet)	Isoleucine (µg/mL)	61.40 +/- 12.30	38.30 +/- 7.30	3	3	Hedges' g	1.83	0.03 to 3.63	Higher measured value with supplementation; unadjusted 95% CI excluded 0	Not applicable - effect size estimated from reported mean +/- SD
Egerton et al. (2020)	Atlantic salmon parr (Salmo salar)	Fish protein hydrolysate supplementation in an 80% plant-protein, low-fishmeal diet	PHP (80% plant protein + 5% fishmeal + 10% partly hydrolysed FPH)	FM (35% fishmeal reference diet)	Leucine (µg/mL)	116.80 +/- 22.50	79.70 +/- 13.40	3	3	Hedges' g	1.6	-0.09 to 3.30	Higher measured value with supplementation; unadjusted 95% CI included 0	Not applicable - effect size estimated from reported mean +/- SD
Egerton et al. (2020)	Atlantic salmon parr (Salmo salar)	Fish protein hydrolysate supplementation in an 80% plant-protein, low-fishmeal diet	PHP (80% plant protein + 5% fishmeal + 10% partly hydrolysed FPH)	FM (35% fishmeal reference diet)	Lysine (µg/mL)	472.50 +/- 47.50	352.30 +/- 112.10	3	3	Hedges' g	1.12	-0.38 to 2.61	Higher measured value with supplementation; unadjusted 95% CI included 0	Not applicable - effect size estimated from reported mean +/- SD
Egerton et al. (2020)	Atlantic salmon parr (Salmo salar)	Fish protein hydrolysate supplementation in an 80% plant-protein, low-fishmeal diet	PHP (80% plant protein + 5% fishmeal + 10% partly hydrolysed FPH)	FM (35% fishmeal reference diet)	Methionine (µg/mL)	28.60 +/- 7.20	28.20 +/- 6.90	3	3	Hedges' g	0.05	-1.24 to 1.33	Higher measured value with supplementation; unadjusted 95% CI included 0	Not applicable - effect size estimated from reported mean +/- SD
Egerton et al. (2020)	Atlantic salmon parr (Salmo salar)	Fish protein hydrolysate supplementation in an 80% plant-protein, low-fishmeal diet	PHP (80% plant protein + 5% fishmeal + 10% partly hydrolysed FPH)	FM (35% fishmeal reference diet)	Phenylalanine (µg/mL)	58.90 +/- 23.20	47.10 +/- 12.00	3	3	Hedges' g	0.51	-0.82 to 1.84	Higher measured value with supplementation; unadjusted 95% CI included 0	Not applicable - effect size estimated from reported mean +/- SD
Egerton et al. (2020)	Atlantic salmon parr (Salmo salar)	Fish protein hydrolysate supplementation in an 80% plant-protein, low-fishmeal diet	PHP (80% plant protein + 5% fishmeal + 10% partly hydrolysed FPH)	FM (35% fishmeal reference diet)	Threonine (µg/mL)	119.50 +/- 17.60	94.40 +/- 11.20	3	3	Hedges' g	1.36	-0.23 to 2.95	Higher measured value with supplementation; unadjusted 95% CI included 0	Not applicable - effect size estimated from reported mean +/- SD
Egerton et al. (2020)	Atlantic salmon parr (Salmo salar)	Fish protein hydrolysate supplementation in an 80% plant-protein, low-fishmeal diet	PHP (80% plant protein + 5% fishmeal + 10% partly hydrolysed FPH)	FM (35% fishmeal reference diet)	Tryptophan (µg/mL)	9.500 +/- 1.700	41.60 +/- 104.70	3	3	Hedges' g	-0.35	-1.65 to 0.96	Lower measured value with supplementation; unadjusted 95% CI included 0	Not applicable - effect size estimated from reported mean +/- SD

Study	Species	Strategy	Intervention	Comparator	Outcome and unit	Intervention mean +/- SD	Comparator mean +/- SD	n intervention (tanks)	n comparator (tanks)	Effect-size metric	Effect size	95% CI	Interpretation	Reason not estimated
Egerton et al. (2020)	Atlantic salmon parr (Salmo salar)	Fish protein hydrolysate supplementation in an 80% plant-protein, low-fishmeal diet	PHP (80% plant protein + 5% fishmeal + 10% partly hydrolysed FPH)	FM (35% fishmeal reference diet)	Valine (µg/mL)	177.20 +/- 26.00	121.40 +/- 12.30	3	3	Hedges' g	2.19	0.21 to 4.18	Higher measured value with supplementation; unadjusted 95% CI excluded 0	Not applicable - effect size estimated from reported mean +/- SD
Egerton et al. (2020)	Atlantic salmon parr (Salmo salar)	Fish protein hydrolysate supplementation in an 80% plant-protein, low-fishmeal diet	PHP (80% plant protein + 5% fishmeal + 10% partly hydrolysed FPH)	FM (35% fishmeal reference diet)	Cystic acid (µg/mL)	4.600 +/- 0.700	4.800 +/- 0.700	3	3	Hedges' g	-0.23	-1.52 to 1.06	Lower measured value with supplementation; unadjusted 95% CI included 0	Not applicable - effect size estimated from reported mean +/- SD
Egerton et al. (2020)	Atlantic salmon parr (Salmo salar)	Fish protein hydrolysate supplementation in an 80% plant-protein, low-fishmeal diet	PHP (80% plant protein + 5% fishmeal + 10% partly hydrolysed FPH)	FM (35% fishmeal reference diet)	Total free amino acids (µg/mL)	3357.40 +/- 298.10	3174.20 +/- 286.80	3	3	Hedges' g	0.5	-0.83 to 1.83	Higher measured value with supplementation; unadjusted 95% CI included 0	Not applicable - effect size estimated from reported mean +/- SD
Egerton et al. (2020)	Atlantic salmon parr (Salmo salar)	Fish protein hydrolysate supplementation in an 80% plant-protein, low-fishmeal diet	SPH (80% plant protein + 5% fishmeal + 10% soluble FPH)	FM (35% fishmeal reference diet)	Feed intake (g/fish)	27.43 +/- 0.50	26.55 +/- 1.17	3	3	Hedges' g	0.78	-0.61 to 2.17	Higher measured value with supplementation; unadjusted 95% CI included 0	Not applicable - effect size estimated from reported mean +/- SD
Egerton et al. (2020)	Atlantic salmon parr (Salmo salar)	Fish protein hydrolysate supplementation in an 80% plant-protein, low-fishmeal diet	SPH (80% plant protein + 5% fishmeal + 10% soluble FPH)	FM (35% fishmeal reference diet)	Feed conversion ratio	1.050 +/- 0.090	0.920 +/- 0.090	3	3	Hedges' g (direction reversed)	-1.16	-2.67 to 0.35	Higher, less favorable value with supplementation; unadjusted 95% CI included 0	Not applicable - effect size estimated from reported mean +/- SD
Egerton et al. (2020)	Atlantic salmon parr (Salmo salar)	Fish protein hydrolysate supplementation in an 80% plant-protein, low-fishmeal diet	SPH (80% plant protein + 5% fishmeal + 10% soluble FPH)	FM (35% fishmeal reference diet)	Protein productive value	2.520 +/- 0.190	3.340 +/- 0.430	3	3	Hedges' g	-1.97	-3.85 to -0.10	Lower, less favorable value with supplementation; unadjusted 95% CI excluded 0	Not applicable - effect size estimated from reported mean +/- SD
Egerton et al. (2020)	Atlantic salmon parr (Salmo salar)	Fish protein hydrolysate supplementation in an 80% plant-protein, low-fishmeal diet	SPH (80% plant protein + 5% fishmeal + 10% soluble FPH)	FM (35% fishmeal reference diet)	Protein efficiency ratio	2.190 +/- 0.200	2.480 +/- 0.260	3	3	Hedges' g	-1.0	-2.46 to 0.46	Lower, less favorable value with supplementation; unadjusted 95% CI included 0	Not applicable - effect size estimated from reported mean +/- SD
Egerton et al. (2020)	Atlantic salmon parr (Salmo salar)	Fish protein hydrolysate supplementation in an 80% plant-protein, low-fishmeal diet	SPH (80% plant protein + 5% fishmeal + 10% soluble FPH)	FM (35% fishmeal reference diet)	Liver weight (mg)	484.21 +/- 30.29	499.67 +/- 17.73	3	3	Hedges' g	-0.5	-1.82 to 0.83	Lower measured value with supplementation; unadjusted 95% CI included 0	Not applicable - effect size estimated from reported mean +/- SD
Egerton et al. (2020)	Atlantic salmon parr (Salmo salar)	Fish protein hydrolysate supplementation in an 80% plant-protein, low-fishmeal diet	SPH (80% plant protein + 5% fishmeal + 10% soluble FPH)	FM (35% fishmeal reference diet)	Hepatosomatic index (%)	1.350 +/- 0.050	1.310 +/- 0.030	3	3	Hedges' g	0.78	-0.61 to 2.16	Higher measured value with supplementation; unadjusted 95% CI included 0	Not applicable - effect size estimated from reported mean +/- SD
Egerton et al. (2020)	Atlantic salmon parr (Salmo salar)	Fish protein hydrolysate supplementation in an 80% plant-protein, low-fishmeal diet	SPH (80% plant protein + 5% fishmeal + 10% soluble FPH)	FM (35% fishmeal reference diet)	Moisture (% wet basis)	74.24 +/- 3.76	70.43 +/- 3.15	3	3	Hedges' g	0.88	-0.54 to 2.30	Higher measured value with supplementation; unadjusted 95% CI included 0	Not applicable - effect size estimated from reported mean +/- SD
Egerton et al. (2020)	Atlantic salmon parr (Salmo salar)	Fish protein hydrolysate supplementation in an 80% plant-protein, low-fishmeal diet	SPH (80% plant protein + 5% fishmeal + 10% soluble FPH)	FM (35% fishmeal reference diet)	Crude protein (% wet basis)	16.76 +/- 0.32	17.26 +/- 0.43	3	3	Hedges' g	-1.06	-2.53 to 0.42	Lower measured value with supplementation; unadjusted 95% CI included 0	Not applicable - effect size estimated from reported mean +/- SD
Egerton et al. (2020)	Atlantic salmon parr (Salmo salar)	Fish protein hydrolysate supplementation in an 80% plant-protein, low-fishmeal diet	SPH (80% plant protein + 5% fishmeal + 10% soluble FPH)	FM (35% fishmeal reference diet)	Crude lipid (% wet basis)	9.550 +/- 0.810	10.31 +/- 0.67	3	3	Hedges' g	-0.82	-2.22 to 0.58	Lower measured value with supplementation; unadjusted 95% CI included 0	Not applicable - effect size estimated from reported mean +/- SD
Egerton et al. (2020)	Atlantic salmon parr (Salmo salar)	Fish protein hydrolysate supplementation in an 80% plant-protein, low-fishmeal diet	SPH (80% plant protein + 5% fishmeal + 10% soluble FPH)	FM (35% fishmeal reference diet)	Ash (% wet basis)	1.630 +/- 0.100	2.140 +/- 0.180	3	3	Hedges' g	-2.8	-5.13 to -0.48	Lower measured value with supplementation; unadjusted 95% CI excluded 0	Not applicable - effect size estimated from reported mean +/- SD
Egerton et al. (2020)	Atlantic salmon parr (Salmo salar)	Fish protein hydrolysate supplementation in an 80% plant-protein, low-fishmeal diet	SPH (80% plant protein + 5% fishmeal + 10% soluble FPH)	FM (35% fishmeal reference diet)	Villus height (µm)	514.30 +/- 161.04	473.67 +/- 130.92	3	3	Hedges' g	0.22	-1.07 to 1.51	Higher, more favorable value with supplementation; unadjusted 95% CI included 0	Not applicable - effect size estimated from reported mean +/- SD
Egerton et al. (2020)	Atlantic salmon parr (Salmo salar)	Fish protein hydrolysate supplementation in an 80% plant-protein, low-fishmeal diet	SPH (80% plant protein + 5% fishmeal + 10% soluble FPH)	FM (35% fishmeal reference diet)	Villus width (µm)	91.67 +/- 20.33	76.31 +/- 14.92	3	3	Hedges' g	0.69	-0.68 to 2.06	Higher, more favorable value with supplementation; unadjusted 95% CI included 0	Not applicable - effect size estimated from reported mean +/- SD
Egerton et al. (2020)	Atlantic salmon parr (Salmo salar)	Fish protein hydrolysate supplementation in an 80% plant-protein, low-fishmeal diet	SPH (80% plant protein + 5% fishmeal + 10% soluble FPH)	FM (35% fishmeal reference diet)	Villus height-to-width ratio	5.680 +/- 1.600	6.370 +/- 1.970	3	3	Hedges' g	-0.31	-1.61 to 0.99	Lower measured value with supplementation; unadjusted 95% CI included 0	Not applicable - effect size estimated from reported mean +/- SD
Egerton et al. (2020)	Atlantic salmon parr (Salmo salar)	Fish protein hydrolysate supplementation in an 80% plant-protein, low-fishmeal diet	SPH (80% plant protein + 5% fishmeal + 10% soluble FPH)	FM (35% fishmeal reference diet)	Intestinal wall width (µm)	84.93 +/- 23.62	58.97 +/- 20.18	3	3	Hedges' g	0.95	-0.49 to 2.38	Higher measured value with supplementation; unadjusted 95% CI included 0	Not applicable - effect size estimated from reported mean +/- SD
Egerton et al. (2020)	Atlantic salmon parr (Salmo salar)	Fish protein hydrolysate supplementation in an 80% plant-protein, low-fishmeal diet	SPH (80% plant protein + 5% fishmeal + 10% soluble FPH)	FM (35% fishmeal reference diet)	Intestine diameter (mm)	2.430 +/- 0.540	2.230 +/- 0.560	3	3	Hedges' g	0.29	-1.01 to 1.59	Higher measured value with supplementation; unadjusted 95% CI included 0	Not applicable - effect size estimated from reported mean +/- SD
Egerton et al. (2020)	Atlantic salmon parr (Salmo salar)	Fish protein hydrolysate supplementation in an 80% plant-protein, low-fishmeal diet	SPH (80% plant protein + 5% fishmeal + 10% soluble FPH)	FM (35% fishmeal reference diet)	Interior lumen area (mm²)	1.680 +/- 0.710	1.900 +/- 1.180	3	3	Hedges' g	-0.18	-1.47 to 1.11	Lower measured value with supplementation; unadjusted 95% CI included 0	Not applicable - effect size estimated from reported mean +/- SD
Egerton et al. (2020)	Atlantic salmon parr (Salmo salar)	Fish protein hydrolysate supplementation in an 80% plant-protein, low-fishmeal diet	SPH (80% plant protein + 5% fishmeal + 10% soluble FPH)	FM (35% fishmeal reference diet)	Internal perimeter (mm)	27.15 +/- 7.42	24.73 +/- 10.35	3	3	Hedges' g	0.21	-1.07 to 1.50	Higher measured value with supplementation; unadjusted 95% CI included 0	Not applicable - effect size estimated from reported mean +/- SD
Egerton et al. (2020)	Atlantic salmon parr (Salmo salar)	Fish protein hydrolysate supplementation in an 80% plant-protein, low-fishmeal diet	SPH (80% plant protein + 5% fishmeal + 10% soluble FPH)	FM (35% fishmeal reference diet)	External perimeter (mm)	7.480 +/- 1.500	7.350 +/- 1.880	3	3	Hedges' g	0.06	-1.22 to 1.34	Higher measured value with supplementation; unadjusted 95% CI included 0	Not applicable - effect size estimated from reported mean +/- SD
Egerton et al. (2020)	Atlantic salmon parr (Salmo salar)	Fish protein hydrolysate supplementation in an 80% plant-protein, low-fishmeal diet	SPH (80% plant protein + 5% fishmeal + 10% soluble FPH)	FM (35% fishmeal reference diet)	Perimeter ratio (absolute)	3.620 +/- 0.550	3.340 +/- 0.810	3	3	Hedges' g	0.32	-0.98 to 1.62	Higher measured value with supplementation; unadjusted 95% CI included 0	Not applicable - effect size estimated from reported mean +/- SD
Egerton et al. (2020)	Atlantic salmon parr (Salmo salar)	Fish protein hydrolysate supplementation in an 80% plant-protein, low-fishmeal diet	SPH (80% plant protein + 5% fishmeal + 10% soluble FPH)	FM (35% fishmeal reference diet)	Alanine (µg/mL)	314.60 +/- 30.40	236.90 +/- 34.60	3	3	Hedges' g	1.91	0.07 to 3.75	Higher measured value with supplementation; unadjusted 95% CI excluded 0	Not applicable - effect size estimated from reported mean +/- SD
Egerton et al. (2020)	Atlantic salmon parr (Salmo salar)	Fish protein hydrolysate supplementation in an 80% plant-protein, low-fishmeal diet	SPH (80% plant protein + 5% fishmeal + 10% soluble FPH)	FM (35% fishmeal reference diet)	Asparagine (µg/mL)	76.10 +/- 11.30	54.00 +/- 10.70	3	3	Hedges' g	1.61	-0.09 to 3.30	Higher measured value with supplementation; unadjusted 95% CI included 0	Not applicable - effect size estimated from reported mean +/- SD

Study	Species	Strategy	Intervention	Comparator	Outcome and unit	Intervention mean +/- SD	Comparator mean +/- SD	n intervention (tanks)	n comparator (tanks)	Effect-size metric	Effect size	95% CI	Interpretation	Reason not estimated
Egerton et al. (2020)	Atlantic salmon parr (Salmo salar)	Fish protein hydrolysate supplementation in an 80% plant-protein, low-fishmeal diet	SPH (80% plant protein + 5% fishmeal + 10% soluble FPH)	FM (35% fishmeal reference diet)	Cysteine (µg/mL)	39.50 +/- 5.60	35.40 +/- 7.70	3	3	Hedges' g	0.49	-0.84 to 1.81	Higher measured value with supplementation; unadjusted 95% CI included 0	Not applicable - effect size estimated from reported mean +/- SD
Egerton et al. (2020)	Atlantic salmon parr (Salmo salar)	Fish protein hydrolysate supplementation in an 80% plant-protein, low-fishmeal diet	SPH (80% plant protein + 5% fishmeal + 10% soluble FPH)	FM (35% fishmeal reference diet)	GABA (µg/mL)	13.50 +/- 1.60	22.50 +/- 7.70	3	3	Hedges' g	-1.29	-2.86 to 0.27	Lower measured value with supplementation; unadjusted 95% CI included 0	Not applicable - effect size estimated from reported mean +/- SD
Egerton et al. (2020)	Atlantic salmon parr (Salmo salar)	Fish protein hydrolysate supplementation in an 80% plant-protein, low-fishmeal diet	SPH (80% plant protein + 5% fishmeal + 10% soluble FPH)	FM (35% fishmeal reference diet)	Glutamic acid (µg/mL)	248.90 +/- 26.00	215.00 +/- 34.50	3	3	Hedges' g	0.89	-0.53 to 2.31	Higher measured value with supplementation; unadjusted 95% CI included 0	Not applicable - effect size estimated from reported mean +/- SD
Egerton et al. (2020)	Atlantic salmon parr (Salmo salar)	Fish protein hydrolysate supplementation in an 80% plant-protein, low-fishmeal diet	SPH (80% plant protein + 5% fishmeal + 10% soluble FPH)	FM (35% fishmeal reference diet)	Glycine (µg/mL)	198.70 +/- 19.50	176.00 +/- 19.10	3	3	Hedges' g	0.94	-0.50 to 2.38	Higher measured value with supplementation; unadjusted 95% CI included 0	Not applicable - effect size estimated from reported mean +/- SD
Egerton et al. (2020)	Atlantic salmon parr (Salmo salar)	Fish protein hydrolysate supplementation in an 80% plant-protein, low-fishmeal diet	SPH (80% plant protein + 5% fishmeal + 10% soluble FPH)	FM (35% fishmeal reference diet)	Proline (µg/mL)	194.10 +/- 16.50	140.80 +/- 23.00	3	3	Hedges' g	2.13	0.18 to 4.08	Higher measured value with supplementation; unadjusted 95% CI excluded 0	Not applicable - effect size estimated from reported mean +/- SD
Egerton et al. (2020)	Atlantic salmon parr (Salmo salar)	Fish protein hydrolysate supplementation in an 80% plant-protein, low-fishmeal diet	SPH (80% plant protein + 5% fishmeal + 10% soluble FPH)	FM (35% fishmeal reference diet)	Serine (µg/mL)	124.00 +/- 8.90	104.20 +/- 10.40	3	3	Hedges' g	1.64	-0.07 to 3.35	Higher measured value with supplementation; unadjusted 95% CI included 0	Not applicable - effect size estimated from reported mean +/- SD
Egerton et al. (2020)	Atlantic salmon parr (Salmo salar)	Fish protein hydrolysate supplementation in an 80% plant-protein, low-fishmeal diet	SPH (80% plant protein + 5% fishmeal + 10% soluble FPH)	FM (35% fishmeal reference diet)	Taurine (µg/mL)	1065.30 +/- 92.50	1212.10 +/- 153.20	3	3	Hedges' g	-0.93	-2.36 to 0.50	Lower measured value with supplementation; unadjusted 95% CI included 0	Not applicable - effect size estimated from reported mean +/- SD
Egerton et al. (2020)	Atlantic salmon parr (Salmo salar)	Fish protein hydrolysate supplementation in an 80% plant-protein, low-fishmeal diet	SPH (80% plant protein + 5% fishmeal + 10% soluble FPH)	FM (35% fishmeal reference diet)	Tyrosine (µg/mL)	32.10 +/- 4.40	26.10 +/- 4.40	3	3	Hedges' g	1.09	-0.40 to 2.58	Higher measured value with supplementation; unadjusted 95% CI included 0	Not applicable - effect size estimated from reported mean +/- SD
Egerton et al. (2020)	Atlantic salmon parr (Salmo salar)	Fish protein hydrolysate supplementation in an 80% plant-protein, low-fishmeal diet	SPH (80% plant protein + 5% fishmeal + 10% soluble FPH)	FM (35% fishmeal reference diet)	Arginine (µg/mL)	155.50 +/- 13.00	116.10 +/- 37.20	3	3	Hedges' g	1.13	-0.37 to 2.63	Higher measured value with supplementation; unadjusted 95% CI included 0	Not applicable - effect size estimated from reported mean +/- SD
Egerton et al. (2020)	Atlantic salmon parr (Salmo salar)	Fish protein hydrolysate supplementation in an 80% plant-protein, low-fishmeal diet	SPH (80% plant protein + 5% fishmeal + 10% soluble FPH)	FM (35% fishmeal reference diet)	Histidine (µg/mL)	38.60 +/- 4.10	27.20 +/- 4.10	3	3	Hedges' g	2.22	0.22 to 4.23	Higher measured value with supplementation; unadjusted 95% CI excluded 0	Not applicable - effect size estimated from reported mean +/- SD
Egerton et al. (2020)	Atlantic salmon parr (Salmo salar)	Fish protein hydrolysate supplementation in an 80% plant-protein, low-fishmeal diet	SPH (80% plant protein + 5% fishmeal + 10% soluble FPH)	FM (35% fishmeal reference diet)	Isoleucine (µg/mL)	46.30 +/- 5.00	38.30 +/- 7.30	3	3	Hedges' g	1.02	-0.44 to 2.49	Higher measured value with supplementation; unadjusted 95% CI included 0	Not applicable - effect size estimated from reported mean +/- SD
Egerton et al. (2020)	Atlantic salmon parr (Salmo salar)	Fish protein hydrolysate supplementation in an 80% plant-protein, low-fishmeal diet	SPH (80% plant protein + 5% fishmeal + 10% soluble FPH)	FM (35% fishmeal reference diet)	Leucine (µg/mL)	99.70 +/- 10.80	79.70 +/- 13.40	3	3	Hedges' g	1.31	-0.26 to 2.89	Higher measured value with supplementation; unadjusted 95% CI included 0	Not applicable - effect size estimated from reported mean +/- SD
Egerton et al. (2020)	Atlantic salmon parr (Salmo salar)	Fish protein hydrolysate supplementation in an 80% plant-protein, low-fishmeal diet	SPH (80% plant protein + 5% fishmeal + 10% soluble FPH)	FM (35% fishmeal reference diet)	Lysine (µg/mL)	505.50 +/- 36.90	352.30 +/- 112.10	3	3	Hedges' g	1.47	-0.17 to 3.10	Higher measured value with supplementation; unadjusted 95% CI included 0	Not applicable - effect size estimated from reported mean +/- SD
Egerton et al. (2020)	Atlantic salmon parr (Salmo salar)	Fish protein hydrolysate supplementation in an 80% plant-protein, low-fishmeal diet	SPH (80% plant protein + 5% fishmeal + 10% soluble FPH)	FM (35% fishmeal reference diet)	Methionine (µg/mL)	31.50 +/- 8.30	28.20 +/- 6.90	3	3	Hedges' g	0.35	-0.96 to 1.65	Higher measured value with supplementation; unadjusted 95% CI included 0	Not applicable - effect size estimated from reported mean +/- SD
Egerton et al. (2020)	Atlantic salmon parr (Salmo salar)	Fish protein hydrolysate supplementation in an 80% plant-protein, low-fishmeal diet	SPH (80% plant protein + 5% fishmeal + 10% soluble FPH)	FM (35% fishmeal reference diet)	Phenylalanine (µg/mL)	57.80 +/- 12.20	47.10 +/- 12.00	3	3	Hedges' g	0.71	-0.66 to 2.08	Higher measured value with supplementation; unadjusted 95% CI included 0	Not applicable - effect size estimated from reported mean +/- SD
Egerton et al. (2020)	Atlantic salmon parr (Salmo salar)	Fish protein hydrolysate supplementation in an 80% plant-protein, low-fishmeal diet	SPH (80% plant protein + 5% fishmeal + 10% soluble FPH)	FM (35% fishmeal reference diet)	Threonine (µg/mL)	118.00 +/- 9.40	94.40 +/- 11.20	3	3	Hedges' g	1.83	0.03 to 3.63	Higher measured value with supplementation; unadjusted 95% CI excluded 0	Not applicable - effect size estimated from reported mean +/- SD
Egerton et al. (2020)	Atlantic salmon parr (Salmo salar)	Fish protein hydrolysate supplementation in an 80% plant-protein, low-fishmeal diet	SPH (80% plant protein + 5% fishmeal + 10% soluble FPH)	FM (35% fishmeal reference diet)	Tryptophan (µg/mL)	10.90 +/- 2.20	41.60 +/- 104.70	3	3	Hedges' g	-0.33	-1.63 to 0.97	Lower measured value with supplementation; unadjusted 95% CI included 0	Not applicable - effect size estimated from reported mean +/- SD
Egerton et al. (2020)	Atlantic salmon parr (Salmo salar)	Fish protein hydrolysate supplementation in an 80% plant-protein, low-fishmeal diet	SPH (80% plant protein + 5% fishmeal + 10% soluble FPH)	FM (35% fishmeal reference diet)	Valine (µg/mL)	158.00 +/- 17.40	121.40 +/- 12.30	3	3	Hedges' g	1.94	0.09 to 3.80	Higher measured value with supplementation; unadjusted 95% CI excluded 0	Not applicable - effect size estimated from reported mean +/- SD
Egerton et al. (2020)	Atlantic salmon parr (Salmo salar)	Fish protein hydrolysate supplementation in an 80% plant-protein, low-fishmeal diet	SPH (80% plant protein + 5% fishmeal + 10% soluble FPH)	FM (35% fishmeal reference diet)	Cystic acid (µg/mL)	5.100 +/- 0.900	4.800 +/- 0.700	3	3	Hedges' g	0.3	-1.00 to 1.59	Higher measured value with supplementation; unadjusted 95% CI included 0	Not applicable - effect size estimated from reported mean +/- SD
Egerton et al. (2020)	Atlantic salmon parr (Salmo salar)	Fish protein hydrolysate supplementation in an 80% plant-protein, low-fishmeal diet	SPH (80% plant protein + 5% fishmeal + 10% soluble FPH)	FM (35% fishmeal reference diet)	Total free amino acids (µg/mL)	3533.60 +/- 205.30	3174.20 +/- 286.80	3	3	Hedges' g	1.15	-0.36 to 2.66	Higher measured value with supplementation; unadjusted 95% CI included 0	Not applicable - effect size estimated from reported mean +/- SD
Egerton et al. (2020)	Atlantic salmon parr (Salmo salar)	Fish protein hydrolysate supplementation in an 80% plant-protein, low-fishmeal diet	PHP (80% plant protein + 5% fishmeal + 10% partly hydrolysed FPH)	PL (80% plant protein + 15% fishmeal)	Mortality count (fish)	13	10	Not available	Not available	Not estimable	Not estimable	Not estimable	Treatment-level mortality count reported, but a risk ratio or standardized mean difference was not calculated	Only treatment-level counts were reported; the denominator and changing number at risk after periodic removals were not available
Egerton et al. (2020)	Atlantic salmon parr (Salmo salar)	Fish protein hydrolysate supplementation in an 80% plant-protein, low-fishmeal diet	PHP (80% plant protein + 5% fishmeal + 10% partly hydrolysed FPH)	PL (80% plant protein + 15% fishmeal)	Apparent digestibility coefficient of dry matter (%)	72.86	70.77	Not available	Not available	Not estimable	Not estimable	Not estimable	Direction can be read from the reported pooled values, but no variance-based effect size was estimable	Faecal material was pooled and only one value per diet was available; no replicated SD/SE was reported
Egerton et al. (2020)	Atlantic salmon parr (Salmo salar)	Fish protein hydrolysate supplementation in an 80% plant-protein, low-fishmeal diet	PHP (80% plant protein + 5% fishmeal + 10% partly hydrolysed FPH)	PL (80% plant protein + 15% fishmeal)	Specific growth rate (%/day)	Not reported	Not reported	Not available	Not available	Not estimable	Not estimable	Not estimable	Not numerically reported	The article discussed SGR trends but did not print group means and SDs
Egerton et al. (2020)	Atlantic salmon parr (Salmo salar)	Fish protein hydrolysate supplementation in an 80% plant-protein, low-fishmeal diet	PHP (80% plant protein + 5% fishmeal + 10% partly hydrolysed FPH)	PL (80% plant protein + 15% fishmeal)	Final body weight (g)	37.03 +/- 0.88	34.14 +/- 3.23	Not available	Not available	Not estimable	Not estimable	Not estimable	Published individual-level summary retained, but a tank-level Hedges' g was not calculated	Measured and analysed at the individual-fish level; the independent-unit sample size and tank-level SD were not reported

Supplementary Table S3

Study	Species	Strategy	Intervention	Comparator	Outcome and unit	Intervention mean +/- SD	Comparator mean +/- SD	n intervention (tanks)	n comparator (tanks)	Effect-size metric	Effect size	95% CI	Interpretation	Reason not estimated
Egerton et al. (2020)	Atlantic salmon parr (Salmo salar)	Fish protein hydrolysate supplementation in an 80% plant-protein, low-fishmeal diet	PHP (80% plant protein + 5% fishmeal + 10% partly hydrolysed FPH)	PL (80% plant protein + 15% fishmeal)	Final fork length (cm)	13.92 +/- 0.03	13.87 +/- 0.37	Not available	Not available	Not estimable	Not estimable	Not estimable	Published individual-level summary retained, but a tank-level Hedges' g was not calculated	Measured and analysed at the individual-fish level; the independent-unit sample size and tank-level SD were not reported
Egerton et al. (2020)	Atlantic salmon parr (Salmo salar)	Fish protein hydrolysate supplementation in an 80% plant-protein, low-fishmeal diet	PHP (80% plant protein + 5% fishmeal + 10% partly hydrolysed FPH)	PL (80% plant protein + 15% fishmeal)	Final condition factor (K)	1.32 +/- 0.03	1.24 +/- 0.04	Not available	Not available	Not estimable	Not estimable	Not estimable	Published individual-level summary retained, but a tank-level Hedges' g was not calculated	Calculated and analysed at the individual-fish level; the independent-unit sample size and tank-level SD were not reported
Egerton et al. (2020)	Atlantic salmon parr (Salmo salar)	Fish protein hydrolysate supplementation in an 80% plant-protein, low-fishmeal diet	PHP (80% plant protein + 5% fishmeal + 10% partly hydrolysed FPH)	PL (80% plant protein + 15% fishmeal)	Gut microbiota Chao1 alpha-diversity index	Figure only	Figure only	Not available	Not available	Not estimable	Not estimable	Not estimable	Figure 3 showed treatment differences, but an exact standardized effect was not calculated	Exact group means and SDs were not printed and the figure displayed sample-level distributions
Egerton et al. (2020)	Atlantic salmon parr (Salmo salar)	Fish protein hydrolysate supplementation in an 80% plant-protein, low-fishmeal diet	PHP (80% plant protein + 5% fishmeal + 10% partly hydrolysed FPH)	PL (80% plant protein + 15% fishmeal)	Gut microbiota Shannon alpha-diversity index	Figure only	Figure only	Not available	Not available	Not estimable	Not estimable	Not estimable	Figure 3 showed treatment differences, but an exact standardized effect was not calculated	Exact group means and SDs were not printed and the figure displayed sample-level distributions
Egerton et al. (2020)	Atlantic salmon parr (Salmo salar)	Fish protein hydrolysate supplementation in an 80% plant-protein, low-fishmeal diet	PHP (80% plant protein + 5% fishmeal + 10% partly hydrolysed FPH)	PL (80% plant protein + 15% fishmeal)	Gut microbiota beta-diversity composition	PCoA/PERMANOVA	PCoA/PERMANOVA	Not available	Not available	Not estimable	Not estimable	Not estimable	Not compatible with pairwise Hedges' g	This was a multivariate PERMANOVA/PCoA outcome rather than a scalar mean with SD
Egerton et al. (2020)	Atlantic salmon parr (Salmo salar)	Fish protein hydrolysate supplementation in an 80% plant-protein, low-fishmeal diet	SPH (80% plant protein + 5% fishmeal + 10% soluble FPH)	PL (80% plant protein + 15% fishmeal)	Mortality count (fish)	6	10	Not available	Not available	Not estimable	Not estimable	Not estimable	Treatment-level mortality count reported, but a risk ratio or standardized mean difference was not calculated	Only treatment-level counts were reported; the denominator and changing number at risk after periodic removals were not available
Egerton et al. (2020)	Atlantic salmon parr (Salmo salar)	Fish protein hydrolysate supplementation in an 80% plant-protein, low-fishmeal diet	SPH (80% plant protein + 5% fishmeal + 10% soluble FPH)	PL (80% plant protein + 15% fishmeal)	Apparent digestibility coefficient of dry matter (%)	80.00	70.77	Not available	Not available	Not estimable	Not estimable	Not estimable	Direction can be read from the reported pooled values, but no variance-based effect size was estimable	Faecal material was pooled and only one value per diet was available; no replicated SD/SE was reported
Egerton et al. (2020)	Atlantic salmon parr (Salmo salar)	Fish protein hydrolysate supplementation in an 80% plant-protein, low-fishmeal diet	SPH (80% plant protein + 5% fishmeal + 10% soluble FPH)	PL (80% plant protein + 15% fishmeal)	Specific growth rate (%/day)	Not reported	Not reported	Not available	Not available	Not estimable	Not estimable	Not estimable	Not numerically reported	The article discussed SGR trends but did not print group means and SDs
Egerton et al. (2020)	Atlantic salmon parr (Salmo salar)	Fish protein hydrolysate supplementation in an 80% plant-protein, low-fishmeal diet	SPH (80% plant protein + 5% fishmeal + 10% soluble FPH)	PL (80% plant protein + 15% fishmeal)	Final body weight (g)	35.71 +/- 1.77	34.14 +/- 3.23	Not available	Not available	Not estimable	Not estimable	Not estimable	Published individual-level summary retained, but a tank-level Hedges' g was not calculated	Measured and analysed at the individual-fish level; the independent-unit sample size and tank-level SD were not reported
Egerton et al. (2020)	Atlantic salmon parr (Salmo salar)	Fish protein hydrolysate supplementation in an 80% plant-protein, low-fishmeal diet	SPH (80% plant protein + 5% fishmeal + 10% soluble FPH)	PL (80% plant protein + 15% fishmeal)	Final fork length (cm)	14.00 +/- 0.35	13.87 +/- 0.37	Not available	Not available	Not estimable	Not estimable	Not estimable	Published individual-level summary retained, but a tank-level Hedges' g was not calculated	Measured and analysed at the individual-fish level; the independent-unit sample size and tank-level SD were not reported
Egerton et al. (2020)	Atlantic salmon parr (Salmo salar)	Fish protein hydrolysate supplementation in an 80% plant-protein, low-fishmeal diet	SPH (80% plant protein + 5% fishmeal + 10% soluble FPH)	PL (80% plant protein + 15% fishmeal)	Final condition factor (K)	1.26 +/- 0.04	1.24 +/- 0.04	Not available	Not available	Not estimable	Not estimable	Not estimable	Published individual-level summary retained, but a tank-level Hedges' g was not calculated	Calculated and analysed at the individual-fish level; the independent-unit sample size and tank-level SD were not reported
Egerton et al. (2020)	Atlantic salmon parr (Salmo salar)	Fish protein hydrolysate supplementation in an 80% plant-protein, low-fishmeal diet	SPH (80% plant protein + 5% fishmeal + 10% soluble FPH)	PL (80% plant protein + 15% fishmeal)	Gut microbiota Chao1 alpha-diversity index	Figure only	Figure only	Not available	Not available	Not estimable	Not estimable	Not estimable	Figure 3 showed treatment differences, but an exact standardized effect was not calculated	Exact group means and SDs were not printed and the figure displayed sample-level distributions
Egerton et al. (2020)	Atlantic salmon parr (Salmo salar)	Fish protein hydrolysate supplementation in an 80% plant-protein, low-fishmeal diet	SPH (80% plant protein + 5% fishmeal + 10% soluble FPH)	PL (80% plant protein + 15% fishmeal)	Gut microbiota Shannon alpha-diversity index	Figure only	Figure only	Not available	Not available	Not estimable	Not estimable	Not estimable	Figure 3 showed treatment differences, but an exact standardized effect was not calculated	Exact group means and SDs were not printed and the figure displayed sample-level distributions
Egerton et al. (2020)	Atlantic salmon parr (Salmo salar)	Fish protein hydrolysate supplementation in an 80% plant-protein, low-fishmeal diet	SPH (80% plant protein + 5% fishmeal + 10% soluble FPH)	PL (80% plant protein + 15% fishmeal)	Gut microbiota beta-diversity composition	PCoA/PERMANOVA	PCoA/PERMANOVA	Not available	Not available	Not estimable	Not estimable	Not estimable	Not compatible with pairwise Hedges' g	This was a multivariate PERMANOVA/PCoA outcome rather than a scalar mean with SD
Egerton et al. (2020)	Atlantic salmon parr (Salmo salar)	Fish protein hydrolysate supplementation in an 80% plant-protein, low-fishmeal diet	PHP (80% plant protein + 5% fishmeal + 10% partly hydrolysed FPH)	FM (35% fishmeal reference diet)	Mortality count (fish)	13	11	Not available	Not available	Not estimable	Not estimable	Not estimable	Treatment-level mortality count reported, but a risk ratio or standardized mean difference was not calculated	Only treatment-level counts were reported; the denominator and changing number at risk after periodic removals were not available
Egerton et al. (2020)	Atlantic salmon parr (Salmo salar)	Fish protein hydrolysate supplementation in an 80% plant-protein, low-fishmeal diet	PHP (80% plant protein + 5% fishmeal + 10% partly hydrolysed FPH)	FM (35% fishmeal reference diet)	Apparent digestibility coefficient of dry matter (%)	72.86	80.83	Not available	Not available	Not estimable	Not estimable	Not estimable	Direction can be read from the reported pooled values, but no variance-based effect size was estimable	Faecal material was pooled and only one value per diet was available; no replicated SD/SE was reported
Egerton et al. (2020)	Atlantic salmon parr (Salmo salar)	Fish protein hydrolysate supplementation in an 80% plant-protein, low-fishmeal diet	PHP (80% plant protein + 5% fishmeal + 10% partly hydrolysed FPH)	FM (35% fishmeal reference diet)	Specific growth rate (%/day)	Not reported	Not reported	Not available	Not available	Not estimable	Not estimable	Not estimable	Not numerically reported	The article discussed SGR trends but did not print group means and SDs
Egerton et al. (2020)	Atlantic salmon parr (Salmo salar)	Fish protein hydrolysate supplementation in an 80% plant-protein, low-fishmeal diet	PHP (80% plant protein + 5% fishmeal + 10% partly hydrolysed FPH)	FM (35% fishmeal reference diet)	Final body weight (g)	37.03 +/- 0.88	38.13 +/- 2.84	Not available	Not available	Not estimable	Not estimable	Not estimable	Published individual-level summary retained, but a tank-level Hedges' g was not calculated	Measured and analysed at the individual-fish level; the independent-unit sample size and tank-level SD were not reported
Egerton et al. (2020)	Atlantic salmon parr (Salmo salar)	Fish protein hydrolysate supplementation in an 80% plant-protein, low-fishmeal diet	PHP (80% plant protein + 5% fishmeal + 10% partly hydrolysed FPH)	FM (35% fishmeal reference diet)	Final fork length (cm)	13.92 +/- 0.03	14.39 +/- 0.34	Not available	Not available	Not estimable	Not estimable	Not estimable	Published individual-level summary retained, but a tank-level Hedges' g was not calculated	Measured and analysed at the individual-fish level; the independent-unit sample size and tank-level SD were not reported
Egerton et al. (2020)	Atlantic salmon parr (Salmo salar)	Fish protein hydrolysate supplementation in an 80% plant-protein, low-fishmeal diet	PHP (80% plant protein + 5% fishmeal + 10% partly hydrolysed FPH)	FM (35% fishmeal reference diet)	Final condition factor (K)	1.32 +/- 0.03	1.23 +/- 0.01	Not available	Not available	Not estimable	Not estimable	Not estimable	Published individual-level summary retained, but a tank-level Hedges' g was not calculated	Calculated and analysed at the individual-fish level; the independent-unit sample size and tank-level SD were not reported
Egerton et al. (2020)	Atlantic salmon parr (Salmo salar)	Fish protein hydrolysate supplementation in an 80% plant-protein, low-fishmeal diet	PHP (80% plant protein + 5% fishmeal + 10% partly hydrolysed FPH)	FM (35% fishmeal reference diet)	Gut microbiota Chao1 alpha-diversity index	Figure only	Figure only	Not available	Not available	Not estimable	Not estimable	Not estimable	Figure 3 showed treatment differences, but an exact standardized effect was not calculated	Exact group means and SDs were not printed and the figure displayed sample-level distributions
Egerton et al. (2020)	Atlantic salmon parr (Salmo salar)	Fish protein hydrolysate supplementation in an 80% plant-protein, low-fishmeal diet	PHP (80% plant protein + 5% fishmeal + 10% partly hydrolysed FPH)	FM (35% fishmeal reference diet)	Gut microbiota Shannon alpha-diversity index	Figure only	Figure only	Not available	Not available	Not estimable	Not estimable	Not estimable	Figure 3 showed treatment differences, but an exact standardized effect was not calculated	Exact group means and SDs were not printed and the figure displayed sample-level distributions
Egerton et al. (2020)	Atlantic salmon parr (Salmo salar)	Fish protein hydrolysate supplementation in an 80% plant-protein, low-fishmeal diet	PHP (80% plant protein + 5% fishmeal + 10% partly hydrolysed FPH)	FM (35% fishmeal reference diet)	Gut microbiota beta-diversity composition	PCoA/PERMANOVA	PCoA/PERMANOVA	Not available	Not available	Not estimable	Not estimable	Not estimable	Not compatible with pairwise Hedges' g	This was a multivariate PERMANOVA/PCoA outcome rather than a scalar mean with SD
Egerton et al. (2020)	Atlantic salmon parr (Salmo salar)	Fish protein hydrolysate supplementation in an 80% plant-protein, low-fishmeal diet	SPH (80% plant protein + 5% fishmeal + 10% soluble FPH)	FM (35% fishmeal reference diet)	Mortality count (fish)	6	11	Not available	Not available	Not estimable	Not estimable	Not estimable	Treatment-level mortality count reported, but a risk ratio or standardized mean difference was not calculated	Only treatment-level counts were reported; the denominator and changing number at risk after periodic removals were not available

Study	Species	Strategy	Intervention	Comparator	Outcome and unit	Intervention mean +/- SD	Comparator mean +/- SD	n intervention (tanks)	n comparator (tanks)	Effect-size metric	Effect size	95% CI	Interpretation	Reason not estimated
Egerton et al. (2020)	Atlantic salmon parr (Salmo salar)	Fish protein hydrolysate supplementation in an 80% plant-protein, low-fishmeal diet	SPH (80% plant protein + 5% fishmeal + 10% soluble FPH)	FM (35% fishmeal reference diet)	Apparent digestibility coefficient of dry matter (%)	80.00	80.83	Not available	Not available	Not estimable	Not estimable	Not estimable	Direction can be read from the reported pooled values, but no variance-based effect size was estimable	Faecal material was pooled and only one value per diet was available; no replicated SD/SE was reported
Egerton et al. (2020)	Atlantic salmon parr (Salmo salar)	Fish protein hydrolysate supplementation in an 80% plant-protein, low-fishmeal diet	SPH (80% plant protein + 5% fishmeal + 10% soluble FPH)	FM (35% fishmeal reference diet)	Specific growth rate (%/day)	Not reported	Not reported	Not available	Not available	Not estimable	Not estimable	Not estimable	Not numerically reported	The article discussed SGR trends but did not print group means and SDs
Egerton et al. (2020)	Atlantic salmon parr (Salmo salar)	Fish protein hydrolysate supplementation in an 80% plant-protein, low-fishmeal diet	SPH (80% plant protein + 5% fishmeal + 10% soluble FPH)	FM (35% fishmeal reference diet)	Final body weight (g)	35.71 +/- 1.77	38.13 +/- 2.84	Not available	Not available	Not estimable	Not estimable	Not estimable	Published individual-level summary retained, but a tank-level Hedges' g was not calculated	Measured and analysed at the individual-fish level; the independent-unit sample size and tank-level SD were not reported
Egerton et al. (2020)	Atlantic salmon parr (Salmo salar)	Fish protein hydrolysate supplementation in an 80% plant-protein, low-fishmeal diet	SPH (80% plant protein + 5% fishmeal + 10% soluble FPH)	FM (35% fishmeal reference diet)	Final fork length (cm)	14.00 +/- 0.35	14.39 +/- 0.34	Not available	Not available	Not estimable	Not estimable	Not estimable	Published individual-level summary retained, but a tank-level Hedges' g was not calculated	Measured and analysed at the individual-fish level; the independent-unit sample size and tank-level SD were not reported
Egerton et al. (2020)	Atlantic salmon parr (Salmo salar)	Fish protein hydrolysate supplementation in an 80% plant-protein, low-fishmeal diet	SPH (80% plant protein + 5% fishmeal + 10% soluble FPH)	FM (35% fishmeal reference diet)	Final condition factor (K)	1.26 +/- 0.04	1.23 +/- 0.01	Not available	Not available	Not estimable	Not estimable	Not estimable	Published individual-level summary retained, but a tank-level Hedges' g was not calculated	Calculated and analysed at the individual-fish level; the independent-unit sample size and tank-level SD were not reported
Egerton et al. (2020)	Atlantic salmon parr (Salmo salar)	Fish protein hydrolysate supplementation in an 80% plant-protein, low-fishmeal diet	SPH (80% plant protein + 5% fishmeal + 10% soluble FPH)	FM (35% fishmeal reference diet)	Gut microbiota Chao1 alpha-diversity index	Figure only	Figure only	Not available	Not available	Not estimable	Not estimable	Not estimable	Figure 3 showed treatment differences, but an exact standardized effect was not calculated	Exact group means and SDs were not printed and the figure displayed sample-level distributions
Egerton et al. (2020)	Atlantic salmon parr (Salmo salar)	Fish protein hydrolysate supplementation in an 80% plant-protein, low-fishmeal diet	SPH (80% plant protein + 5% fishmeal + 10% soluble FPH)	FM (35% fishmeal reference diet)	Gut microbiota Shannon alpha-diversity index	Figure only	Figure only	Not available	Not available	Not estimable	Not estimable	Not estimable	Figure 3 showed treatment differences, but an exact standardized effect was not calculated	Exact group means and SDs were not printed and the figure displayed sample-level distributions
Egerton et al. (2020)	Atlantic salmon parr (Salmo salar)	Fish protein hydrolysate supplementation in an 80% plant-protein, low-fishmeal diet	SPH (80% plant protein + 5% fishmeal + 10% soluble FPH)	FM (35% fishmeal reference diet)	Gut microbiota beta-diversity composition	PCoA/PERMANOVA	PCoA/PERMANOVA	Not available	Not available	Not estimable	Not estimable	Not estimable	Not compatible with pairwise Hedges' g	This was a multivariate PERMANOVA/PCoA outcome rather than a scalar mean with SD

Note: Estimable outcomes used published mean +/- SD and n = 3 independent tanks. PHP and SPH were each compared with the PL basal diet and with the FM reference diet. FCR was direction-reversed because lower values are favorable. Apparent digestibility was not estimable because faeces were pooled without replicated variance. Final weight, length, and condition factor were not assigned tank-level Hedges' g because the article analysed individual fish and did not report tank-level dispersion. Pairwise 95% CIs are descriptive and unadjusted.

Source: Egerton et al. (2020), https://doi.org/10.1038/s41598-020-60325-7

Study 19. Bae et al. (2020): soybean meal, fermented soybean meal, and sesame meal replacing fishmeal in juvenile whiteleg shrimp

Study	Species	Strategy	Intervention	Comparator	Outcome and unit	Intervention mean +/- SD	Comparator mean +/- SD	n intervention (tanks)	n comparator (tanks)	Effect-size metric	Effect size	95% CI	Interpretation	Reason not estimated
Bae et al. (2020)	Whiteleg shrimp (Litopenaeus vannamei)	Soybean meal, fermented soybean meal, or sesame meal replacing 20% or 40% of dietary fishmeal	SB20 (20% fishmeal replacement with soybean meal)	CON (30% fishmeal control diet)	Weight gain (%)	379.00 +/- 18.71	391.00 +/- 18.71	3	3	Hedges' g	-0.51	-1.84 to 0.82	Lower, less favorable value with replacement; unadjusted 95% CI included 0	Not applicable - effect size estimated using reconstructed common SD (pooled SEM x sqrt[3])
Bae et al. (2020)	Whiteleg shrimp (Litopenaeus vannamei)	Soybean meal, fermented soybean meal, or sesame meal replacing 20% or 40% of dietary fishmeal	SB20 (20% fishmeal replacement with soybean meal)	CON (30% fishmeal control diet)	Specific growth rate (%/day)	3.480 +/- 0.087	3.540 +/- 0.087	3	3	Hedges' g	-0.55	-1.89 to 0.78	Lower, less favorable value with replacement; unadjusted 95% CI included 0	Not applicable - effect size estimated using reconstructed common SD (pooled SEM x sqrt[3])
Bae et al. (2020)	Whiteleg shrimp (Litopenaeus vannamei)	Soybean meal, fermented soybean meal, or sesame meal replacing 20% or 40% of dietary fishmeal	SB20 (20% fishmeal replacement with soybean meal)	CON (30% fishmeal control diet)	Feed efficiency (%)	135.00 +/- 5.94	124.00 +/- 5.94	3	3	Hedges' g	1.48	-0.16 to 3.12	Higher, more favorable value with replacement; unadjusted 95% CI included 0	Not applicable - effect size estimated using reconstructed common SD (pooled SEM x sqrt[3])
Bae et al. (2020)	Whiteleg shrimp (Litopenaeus vannamei)	Soybean meal, fermented soybean meal, or sesame meal replacing 20% or 40% of dietary fishmeal	SB20 (20% fishmeal replacement with soybean meal)	CON (30% fishmeal control diet)	Protein efficiency ratio	2.930 +/- 0.139	2.970 +/- 0.139	3	3	Hedges' g	-0.23	-1.52 to 1.06	Lower, less favorable value with replacement; unadjusted 95% CI included 0	Not applicable - effect size estimated using reconstructed common SD (pooled SEM x sqrt[3])
Bae et al. (2020)	Whiteleg shrimp (Litopenaeus vannamei)	Soybean meal, fermented soybean meal, or sesame meal replacing 20% or 40% of dietary fishmeal	SB20 (20% fishmeal replacement with soybean meal)	CON (30% fishmeal control diet)	Survival rate (%)	68.30 +/- 0.74	70.00 +/- 0.74	3	3	Hedges' g	-1.83	-3.63 to -0.03	Lower, less favorable value with replacement; unadjusted 95% CI excluded 0	Not applicable - effect size estimated using reconstructed common SD (pooled SEM x sqrt[3])
Bae et al. (2020)	Whiteleg shrimp (Litopenaeus vannamei)	Soybean meal, fermented soybean meal, or sesame meal replacing 20% or 40% of dietary fishmeal	SB20 (20% fishmeal replacement with soybean meal)	CON (30% fishmeal control diet)	Lysozyme activity (U/L)	1.620 +/- 0.035	1.620 +/- 0.035	3	3	Hedges' g	0.0	-1.28 to 1.28	No observed mean difference; unadjusted 95% CI included 0	Not applicable - effect size estimated using reconstructed common SD (pooled SEM x sqrt[3])
Bae et al. (2020)	Whiteleg shrimp (Litopenaeus vannamei)	Soybean meal, fermented soybean meal, or sesame meal replacing 20% or 40% of dietary fishmeal	SB20 (20% fishmeal replacement with soybean meal)	CON (30% fishmeal control diet)	Superoxide dismutase activity (% inhibition)	84.40 +/- 1.65	87.10 +/- 1.65	3	3	Hedges' g	-1.31	-2.88 to 0.26	Lower, less favorable value with replacement; unadjusted 95% CI included 0	Not applicable - effect size estimated using reconstructed common SD (pooled SEM x sqrt[3])
Bae et al. (2020)	Whiteleg shrimp (Litopenaeus vannamei)	Soybean meal, fermented soybean meal, or sesame meal replacing 20% or 40% of dietary fishmeal	SB20 (20% fishmeal replacement with soybean meal)	CON (30% fishmeal control diet)	Moisture (% dry-matter basis)	6.430 +/- 0.121	6.080 +/- 0.121	3	3	Hedges' g	2.31	0.26 to 4.36	Higher measured value with replacement; unadjusted 95% CI excluded 0	Not applicable - effect size estimated using reconstructed common SD (pooled SEM x sqrt[3])
Bae et al. (2020)	Whiteleg shrimp (Litopenaeus vannamei)	Soybean meal, fermented soybean meal, or sesame meal replacing 20% or 40% of dietary fishmeal	SB20 (20% fishmeal replacement with soybean meal)	CON (30% fishmeal control diet)	Crude protein (% dry-matter basis)	76.00 +/- 0.38	76.30 +/- 0.38	3	3	Hedges' g	-0.63	-1.98 to 0.72	Lower measured value with replacement; unadjusted 95% CI included 0	Not applicable - effect size estimated using reconstructed common SD (pooled SEM x sqrt[3])
Bae et al. (2020)	Whiteleg shrimp (Litopenaeus vannamei)	Soybean meal, fermented soybean meal, or sesame meal replacing 20% or 40% of dietary fishmeal	SB20 (20% fishmeal replacement with soybean meal)	CON (30% fishmeal control diet)	Crude lipid (% dry-matter basis)	1.970 +/- 0.087	1.840 +/- 0.087	3	3	Hedges' g	1.2	-0.33 to 2.73	Higher measured value with replacement; unadjusted 95% CI included 0	Not applicable - effect size estimated using reconstructed common SD (pooled SEM x sqrt[3])
Bae et al. (2020)	Whiteleg shrimp (Litopenaeus vannamei)	Soybean meal, fermented soybean meal, or sesame meal replacing 20% or 40% of dietary fishmeal	SB20 (20% fishmeal replacement with soybean meal)	CON (30% fishmeal control diet)	Ash (% dry-matter basis)	13.70 +/- 0.28	14.20 +/- 0.28	3	3	Hedges' g	-1.44	-3.07 to 0.18	Lower measured value with replacement; unadjusted 95% CI included 0	Not applicable - effect size estimated using reconstructed common SD (pooled SEM x sqrt[3])
Bae et al. (2020)	Whiteleg shrimp (Litopenaeus vannamei)	Soybean meal, fermented soybean meal, or sesame meal replacing 20% or 40% of dietary fishmeal	SB20 (20% fishmeal replacement with soybean meal)	CON (30% fishmeal control diet)	Glucose (mg/dL)	48.20 +/- 7.86	51.70 +/- 7.86	3	3	Hedges' g	-0.36	-1.66 to 0.95	Lower measured value with replacement; unadjusted 95% CI included 0	Not applicable - effect size estimated using reconstructed common SD (pooled SEM x sqrt[3])
Bae et al. (2020)	Whiteleg shrimp (Litopenaeus vannamei)	Soybean meal, fermented soybean meal, or sesame meal replacing 20% or 40% of dietary fishmeal	SB20 (20% fishmeal replacement with soybean meal)	CON (30% fishmeal control diet)	Total protein (g/dL)	10.10 +/- 1.00	10.60 +/- 1.00	3	3	Hedges' g	-0.4	-1.71 to 0.91	Lower measured value with replacement; unadjusted 95% CI included 0	Not applicable - effect size estimated using reconstructed common SD (pooled SEM x sqrt[3])
Bae et al. (2020)	Whiteleg shrimp (Litopenaeus vannamei)	Soybean meal, fermented soybean meal, or sesame meal replacing 20% or 40% of dietary fishmeal	SB20 (20% fishmeal replacement with soybean meal)	CON (30% fishmeal control diet)	Glutamic oxaloacetic transaminase (U/L)	64.80 +/- 7.81	63.30 +/- 7.81	3	3	Hedges' g	0.15	-1.13 to 1.44	Higher measured value with replacement; unadjusted 95% CI included 0	Not applicable - effect size estimated using reconstructed common SD (pooled SEM x sqrt[3])
Bae et al. (2020)	Whiteleg shrimp (Litopenaeus vannamei)	Soybean meal, fermented soybean meal, or sesame meal replacing 20% or 40% of dietary fishmeal	SB20 (20% fishmeal replacement with soybean meal)	CON (30% fishmeal control diet)	Glutamic-pyruvic transaminase (U/L)	13.30 +/- 1.99	15.00 +/- 1.99	3	3	Hedges' g	-0.68	-2.05 to 0.68	Lower measured value with replacement; unadjusted 95% CI included 0	Not applicable - effect size estimated using reconstructed common SD (pooled SEM x sqrt[3])
Bae et al. (2020)	Whiteleg shrimp (Litopenaeus vannamei)	Soybean meal, fermented soybean meal, or sesame meal replacing 20% or 40% of dietary fishmeal	SB40 (40% fishmeal replacement with soybean meal)	CON (30% fishmeal control diet)	Weight gain (%)	370.00 +/- 18.71	391.00 +/- 18.71	3	3	Hedges' g	-0.9	-2.32 to 0.53	Lower, less favorable value with replacement; unadjusted 95% CI included 0	Not applicable - effect size estimated using reconstructed common SD (pooled SEM x sqrt[3])
Bae et al. (2020)	Whiteleg shrimp (Litopenaeus vannamei)	Soybean meal, fermented soybean meal, or sesame meal replacing 20% or 40% of dietary fishmeal	SB40 (40% fishmeal replacement with soybean meal)	CON (30% fishmeal control diet)	Specific growth rate (%/day)	3.440 +/- 0.087	3.540 +/- 0.087	3	3	Hedges' g	-0.92	-2.36 to 0.51	Lower, less favorable value with replacement; unadjusted 95% CI included 0	Not applicable - effect size estimated using reconstructed common SD (pooled SEM x sqrt[3])
Bae et al. (2020)	Whiteleg shrimp (Litopenaeus vannamei)	Soybean meal, fermented soybean meal, or sesame meal replacing 20% or 40% of dietary fishmeal	SB40 (40% fishmeal replacement with soybean meal)	CON (30% fishmeal control diet)	Feed efficiency (%)	126.00 +/- 5.94	124.00 +/- 5.94	3	3	Hedges' g	0.27	-1.02 to 1.56	Higher, more favorable value with replacement; unadjusted 95% CI included 0	Not applicable - effect size estimated using reconstructed common SD (pooled SEM x sqrt[3])
Bae et al. (2020)	Whiteleg shrimp (Litopenaeus vannamei)	Soybean meal, fermented soybean meal, or sesame meal replacing 20% or 40% of dietary fishmeal	SB40 (40% fishmeal replacement with soybean meal)	CON (30% fishmeal control diet)	Protein efficiency ratio	2.800 +/- 0.139	2.970 +/- 0.139	3	3	Hedges' g	-0.98	-2.43 to 0.47	Lower, less favorable value with replacement; unadjusted 95% CI included 0	Not applicable - effect size estimated using reconstructed common SD (pooled SEM x sqrt[3])
Bae et al. (2020)	Whiteleg shrimp (Litopenaeus vannamei)	Soybean meal, fermented soybean meal, or sesame meal replacing 20% or 40% of dietary fishmeal	SB40 (40% fishmeal replacement with soybean meal)	CON (30% fishmeal control diet)	Survival rate (%)	68.30 +/- 0.74	70.00 +/- 0.74	3	3	Hedges' g	-1.83	-3.63 to -0.03	Lower, less favorable value with replacement; unadjusted 95% CI excluded 0	Not applicable - effect size estimated using reconstructed common SD (pooled SEM x sqrt[3])
Bae et al. (2020)	Whiteleg shrimp (Litopenaeus vannamei)	Soybean meal, fermented soybean meal, or sesame meal replacing 20% or 40% of dietary fishmeal	SB40 (40% fishmeal replacement with soybean meal)	CON (30% fishmeal control diet)	Lysozyme activity (U/L)	1.540 +/- 0.035	1.620 +/- 0.035	3	3	Hedges' g	-1.85	-3.66 to -0.04	Lower, less favorable value with replacement; unadjusted 95% CI excluded 0	Not applicable - effect size estimated using reconstructed common SD (pooled SEM x sqrt[3])
Bae et al. (2020)	Whiteleg shrimp (Litopenaeus vannamei)	Soybean meal, fermented soybean meal, or sesame meal replacing 20% or 40% of dietary fishmeal	SB40 (40% fishmeal replacement with soybean meal)	CON (30% fishmeal control diet)	Superoxide dismutase activity (% inhibition)	81.80 +/- 1.65	87.10 +/- 1.65	3	3	Hedges' g	-2.58	-4.77 to -0.38	Lower, less favorable value with replacement; unadjusted 95% CI excluded 0	Not applicable - effect size estimated using reconstructed common SD (pooled SEM x sqrt[3])
Bae et al. (2020)	Whiteleg shrimp (Litopenaeus vannamei)	Soybean meal, fermented soybean meal, or sesame meal replacing 20% or 40% of dietary fishmeal	SB40 (40% fishmeal replacement with soybean meal)	CON (30% fishmeal control diet)	Moisture (% dry-matter basis)	6.120 +/- 0.121	6.080 +/- 0.121	3	3	Hedges' g	0.26	-1.03 to 1.56	Higher measured value with replacement; unadjusted 95% CI included 0	Not applicable - effect size estimated using reconstructed common SD (pooled SEM x sqrt[3])
Bae et al. (2020)	Whiteleg shrimp (Litopenaeus vannamei)	Soybean meal, fermented soybean meal, or sesame meal replacing 20% or 40% of dietary fishmeal	SB40 (40% fishmeal replacement with soybean meal)	CON (30% fishmeal control diet)	Crude protein (% dry-matter basis)	77.10 +/- 0.38	76.30 +/- 0.38	3	3	Hedges' g	1.68	-0.05 to 3.41	Higher measured value with replacement; unadjusted 95% CI included 0	Not applicable - effect size estimated using reconstructed common SD (pooled SEM x sqrt[3])

Study	Species	Strategy	Intervention	Comparator	Outcome and unit	Intervention mean +/- SD	Comparator mean +/- SD	n intervention (tanks)	n comparator (tanks)	Effect-size metric	Effect size	95% CI	Interpretation	Reason not estimated
Bae et al. (2020)	Whiteleg shrimp (Litopenaeus vannamei)	Soybean meal, fermented soybean meal, or sesame meal replacing 20% or 40% of dietary fishmeal	SB40 (40% fishmeal replacement with soybean meal)	CON (30% fishmeal control diet)	Crude lipid (% dry-matter basis)	1.680 +/- 0.087	1.840 +/- 0.087	3	3	Hedges' g	-1.48	-3.12 to 0.16	Lower measured value with replacement; unadjusted 95% CI included 0	Not applicable - effect size estimated using reconstructed common SD (pooled SEM x sqrt[3])
Bae et al. (2020)	Whiteleg shrimp (Litopenaeus vannamei)	Soybean meal, fermented soybean meal, or sesame meal replacing 20% or 40% of dietary fishmeal	SB40 (40% fishmeal replacement with soybean meal)	CON (30% fishmeal control diet)	Ash (% dry-matter basis)	13.70 +/- 0.28	14.20 +/- 0.28	3	3	Hedges' g	-1.44	-3.07 to 0.18	Lower measured value with replacement; unadjusted 95% CI included 0	Not applicable - effect size estimated using reconstructed common SD (pooled SEM x sqrt[3])
Bae et al. (2020)	Whiteleg shrimp (Litopenaeus vannamei)	Soybean meal, fermented soybean meal, or sesame meal replacing 20% or 40% of dietary fishmeal	SB40 (40% fishmeal replacement with soybean meal)	CON (30% fishmeal control diet)	Glucose (mg/dL)	50.00 +/- 7.86	51.70 +/- 7.86	3	3	Hedges' g	-0.17	-1.46 to 1.11	Lower measured value with replacement; unadjusted 95% CI included 0	Not applicable - effect size estimated using reconstructed common SD (pooled SEM x sqrt[3])
Bae et al. (2020)	Whiteleg shrimp (Litopenaeus vannamei)	Soybean meal, fermented soybean meal, or sesame meal replacing 20% or 40% of dietary fishmeal	SB40 (40% fishmeal replacement with soybean meal)	CON (30% fishmeal control diet)	Total protein (g/dL)	10.20 +/- 1.00	10.60 +/- 1.00	3	3	Hedges' g	-0.32	-1.62 to 0.98	Lower measured value with replacement; unadjusted 95% CI included 0	Not applicable - effect size estimated using reconstructed common SD (pooled SEM x sqrt[3])
Bae et al. (2020)	Whiteleg shrimp (Litopenaeus vannamei)	Soybean meal, fermented soybean meal, or sesame meal replacing 20% or 40% of dietary fishmeal	SB40 (40% fishmeal replacement with soybean meal)	CON (30% fishmeal control diet)	Glutamic oxaloacetic transaminase (U/L)	67.20 +/- 7.81	63.30 +/- 7.81	3	3	Hedges' g	0.4	-0.91 to 1.71	Higher measured value with replacement; unadjusted 95% CI included 0	Not applicable - effect size estimated using reconstructed common SD (pooled SEM x sqrt[3])
Bae et al. (2020)	Whiteleg shrimp (Litopenaeus vannamei)	Soybean meal, fermented soybean meal, or sesame meal replacing 20% or 40% of dietary fishmeal	SB40 (40% fishmeal replacement with soybean meal)	CON (30% fishmeal control diet)	Glutamic-pyruvic transaminase (U/L)	15.00 +/- 1.99	15.00 +/- 1.99	3	3	Hedges' g	0.0	-1.28 to 1.28	No observed mean difference; unadjusted 95% CI included 0	Not applicable - effect size estimated using reconstructed common SD (pooled SEM x sqrt[3])
Bae et al. (2020)	Whiteleg shrimp (Litopenaeus vannamei)	Soybean meal, fermented soybean meal, or sesame meal replacing 20% or 40% of dietary fishmeal	ST20 (20% fishmeal replacement with fermented soybean meal)	CON (30% fishmeal control diet)	Weight gain (%)	426.00 +/- 18.71	391.00 +/- 18.71	3	3	Hedges' g	1.5	-0.15 to 3.14	Higher, more favorable value with replacement; unadjusted 95% CI included 0	Not applicable - effect size estimated using reconstructed common SD (pooled SEM x sqrt[3])
Bae et al. (2020)	Whiteleg shrimp (Litopenaeus vannamei)	Soybean meal, fermented soybean meal, or sesame meal replacing 20% or 40% of dietary fishmeal	ST20 (20% fishmeal replacement with fermented soybean meal)	CON (30% fishmeal control diet)	Specific growth rate (%/day)	3.690 +/- 0.087	3.540 +/- 0.087	3	3	Hedges' g	1.39	-0.21 to 2.99	Higher, more favorable value with replacement; unadjusted 95% CI included 0	Not applicable - effect size estimated using reconstructed common SD (pooled SEM x sqrt[3])
Bae et al. (2020)	Whiteleg shrimp (Litopenaeus vannamei)	Soybean meal, fermented soybean meal, or sesame meal replacing 20% or 40% of dietary fishmeal	ST20 (20% fishmeal replacement with fermented soybean meal)	CON (30% fishmeal control diet)	Feed efficiency (%)	115.00 +/- 5.94	124.00 +/- 5.94	3	3	Hedges' g	-1.21	-2.74 to 0.32	Lower, less favorable value with replacement; unadjusted 95% CI included 0	Not applicable - effect size estimated using reconstructed common SD (pooled SEM x sqrt[3])
Bae et al. (2020)	Whiteleg shrimp (Litopenaeus vannamei)	Soybean meal, fermented soybean meal, or sesame meal replacing 20% or 40% of dietary fishmeal	ST20 (20% fishmeal replacement with fermented soybean meal)	CON (30% fishmeal control diet)	Protein efficiency ratio	3.230 +/- 0.139	2.970 +/- 0.139	3	3	Hedges' g	1.5	-0.15 to 3.15	Higher, more favorable value with replacement; unadjusted 95% CI included 0	Not applicable - effect size estimated using reconstructed common SD (pooled SEM x sqrt[3])
Bae et al. (2020)	Whiteleg shrimp (Litopenaeus vannamei)	Soybean meal, fermented soybean meal, or sesame meal replacing 20% or 40% of dietary fishmeal	ST20 (20% fishmeal replacement with fermented soybean meal)	CON (30% fishmeal control diet)	Survival rate (%)	68.30 +/- 0.74	70.00 +/- 0.74	3	3	Hedges' g	-1.83	-3.63 to -0.03	Lower, less favorable value with replacement; unadjusted 95% CI excluded 0	Not applicable - effect size estimated using reconstructed common SD (pooled SEM x sqrt[3])
Bae et al. (2020)	Whiteleg shrimp (Litopenaeus vannamei)	Soybean meal, fermented soybean meal, or sesame meal replacing 20% or 40% of dietary fishmeal	ST20 (20% fishmeal replacement with fermented soybean meal)	CON (30% fishmeal control diet)	Lysozyme activity (U/L)	1.700 +/- 0.035	1.620 +/- 0.035	3	3	Hedges' g	1.85	0.04 to 3.66	Higher, more favorable value with replacement; unadjusted 95% CI excluded 0	Not applicable - effect size estimated using reconstructed common SD (pooled SEM x sqrt[3])
Bae et al. (2020)	Whiteleg shrimp (Litopenaeus vannamei)	Soybean meal, fermented soybean meal, or sesame meal replacing 20% or 40% of dietary fishmeal	ST20 (20% fishmeal replacement with fermented soybean meal)	CON (30% fishmeal control diet)	Superoxide dismutase activity (% inhibition)	87.70 +/- 1.65	87.10 +/- 1.65	3	3	Hedges' g	0.29	-1.00 to 1.59	Higher, more favorable value with replacement; unadjusted 95% CI included 0	Not applicable - effect size estimated using reconstructed common SD (pooled SEM x sqrt[3])
Bae et al. (2020)	Whiteleg shrimp (Litopenaeus vannamei)	Soybean meal, fermented soybean meal, or sesame meal replacing 20% or 40% of dietary fishmeal	ST20 (20% fishmeal replacement with fermented soybean meal)	CON (30% fishmeal control diet)	Moisture (% dry-matter basis)	6.480 +/- 0.121	6.080 +/- 0.121	3	3	Hedges' g	2.64	0.41 to 4.87	Higher measured value with replacement; unadjusted 95% CI excluded 0	Not applicable - effect size estimated using reconstructed common SD (pooled SEM x sqrt[3])
Bae et al. (2020)	Whiteleg shrimp (Litopenaeus vannamei)	Soybean meal, fermented soybean meal, or sesame meal replacing 20% or 40% of dietary fishmeal	ST20 (20% fishmeal replacement with fermented soybean meal)	CON (30% fishmeal control diet)	Crude protein (% dry-matter basis)	76.20 +/- 0.38	76.30 +/- 0.38	3	3	Hedges' g	-0.21	-1.50 to 1.08	Lower measured value with replacement; unadjusted 95% CI included 0	Not applicable - effect size estimated using reconstructed common SD (pooled SEM x sqrt[3])
Bae et al. (2020)	Whiteleg shrimp (Litopenaeus vannamei)	Soybean meal, fermented soybean meal, or sesame meal replacing 20% or 40% of dietary fishmeal	ST20 (20% fishmeal replacement with fermented soybean meal)	CON (30% fishmeal control diet)	Crude lipid (% dry-matter basis)	1.670 +/- 0.087	1.840 +/- 0.087	3	3	Hedges' g	-1.57	-3.25 to 0.11	Lower measured value with replacement; unadjusted 95% CI included 0	Not applicable - effect size estimated using reconstructed common SD (pooled SEM x sqrt[3])
Bae et al. (2020)	Whiteleg shrimp (Litopenaeus vannamei)	Soybean meal, fermented soybean meal, or sesame meal replacing 20% or 40% of dietary fishmeal	ST20 (20% fishmeal replacement with fermented soybean meal)	CON (30% fishmeal control diet)	Ash (% dry-matter basis)	13.20 +/- 0.28	14.20 +/- 0.28	3	3	Hedges' g	-2.89	-5.26 to -0.51	Lower measured value with replacement; unadjusted 95% CI excluded 0	Not applicable - effect size estimated using reconstructed common SD (pooled SEM x sqrt[3])
Bae et al. (2020)	Whiteleg shrimp (Litopenaeus vannamei)	Soybean meal, fermented soybean meal, or sesame meal replacing 20% or 40% of dietary fishmeal	ST20 (20% fishmeal replacement with fermented soybean meal)	CON (30% fishmeal control diet)	Glucose (mg/dL)	52.50 +/- 7.86	51.70 +/- 7.86	3	3	Hedges' g	0.08	-1.20 to 1.36	Higher measured value with replacement; unadjusted 95% CI included 0	Not applicable - effect size estimated using reconstructed common SD (pooled SEM x sqrt[3])
Bae et al. (2020)	Whiteleg shrimp (Litopenaeus vannamei)	Soybean meal, fermented soybean meal, or sesame meal replacing 20% or 40% of dietary fishmeal	ST20 (20% fishmeal replacement with fermented soybean meal)	CON (30% fishmeal control diet)	Total protein (g/dL)	10.30 +/- 1.00	10.60 +/- 1.00	3	3	Hedges' g	-0.24	-1.53 to 1.05	Lower measured value with replacement; unadjusted 95% CI included 0	Not applicable - effect size estimated using reconstructed common SD (pooled SEM x sqrt[3])
Bae et al. (2020)	Whiteleg shrimp (Litopenaeus vannamei)	Soybean meal, fermented soybean meal, or sesame meal replacing 20% or 40% of dietary fishmeal	ST20 (20% fishmeal replacement with fermented soybean meal)	CON (30% fishmeal control diet)	Glutamic oxaloacetic transaminase (U/L)	65.20 +/- 7.81	63.30 +/- 7.81	3	3	Hedges' g	0.19	-1.09 to 1.48	Higher measured value with replacement; unadjusted 95% CI included 0	Not applicable - effect size estimated using reconstructed common SD (pooled SEM x sqrt[3])
Bae et al. (2020)	Whiteleg shrimp (Litopenaeus vannamei)	Soybean meal, fermented soybean meal, or sesame meal replacing 20% or 40% of dietary fishmeal	ST20 (20% fishmeal replacement with fermented soybean meal)	CON (30% fishmeal control diet)	Glutamic-pyruvic transaminase (U/L)	15.30 +/- 1.99	15.00 +/- 1.99	3	3	Hedges' g	0.12	-1.16 to 1.40	Higher measured value with replacement; unadjusted 95% CI included 0	Not applicable - effect size estimated using reconstructed common SD (pooled SEM x sqrt[3])
Bae et al. (2020)	Whiteleg shrimp (Litopenaeus vannamei)	Soybean meal, fermented soybean meal, or sesame meal replacing 20% or 40% of dietary fishmeal	ST40 (40% fishmeal replacement with fermented soybean meal)	CON (30% fishmeal control diet)	Weight gain (%)	380.00 +/- 18.71	391.00 +/- 18.71	3	3	Hedges' g	-0.47	-1.79 to 0.85	Lower, less favorable value with replacement; unadjusted 95% CI included 0	Not applicable - effect size estimated using reconstructed common SD (pooled SEM x sqrt[3])
Bae et al. (2020)	Whiteleg shrimp (Litopenaeus vannamei)	Soybean meal, fermented soybean meal, or sesame meal replacing 20% or 40% of dietary fishmeal	ST40 (40% fishmeal replacement with fermented soybean meal)	CON (30% fishmeal control diet)	Specific growth rate (%/day)	3.470 +/- 0.087	3.540 +/- 0.087	3	3	Hedges' g	-0.65	-2.00 to 0.71	Lower, less favorable value with replacement; unadjusted 95% CI included 0	Not applicable - effect size estimated using reconstructed common SD (pooled SEM x sqrt[3])
Bae et al. (2020)	Whiteleg shrimp (Litopenaeus vannamei)	Soybean meal, fermented soybean meal, or sesame meal replacing 20% or 40% of dietary fishmeal	ST40 (40% fishmeal replacement with fermented soybean meal)	CON (30% fishmeal control diet)	Feed efficiency (%)	130.00 +/- 5.94	124.00 +/- 5.94	3	3	Hedges' g	0.81	-0.59 to 2.21	Higher, more favorable value with replacement; unadjusted 95% CI included 0	Not applicable - effect size estimated using reconstructed common SD (pooled SEM x sqrt[3])

Study	Species	Strategy	Intervention	Comparator	Outcome and unit	Intervention mean +/- SD	Comparator mean +/- SD	n intervention (tanks)	n comparator (tanks)	Effect-size metric	Effect size	95% CI	Interpretation	Reason not estimated
Bae et al. (2020)	Whiteleg shrimp (Litopenaeus vannamei)	Soybean meal, fermented soybean meal, or sesame meal replacing 20% or 40% of dietary fishmeal	ST40 (40% fishmeal replacement with fermented soybean meal)	CON (30% fishmeal control diet)	Protein efficiency ratio	2.880 +/- 0.139	2.970 +/- 0.139	3	3	Hedges' g	-0.52	-1.85 to 0.81	Lower, less favorable value with replacement; unadjusted 95% CI included 0	Not applicable - effect size estimated using reconstructed common SD (pooled SEM x sqrt[3])
Bae et al. (2020)	Whiteleg shrimp (Litopenaeus vannamei)	Soybean meal, fermented soybean meal, or sesame meal replacing 20% or 40% of dietary fishmeal	ST40 (40% fishmeal replacement with fermented soybean meal)	CON (30% fishmeal control diet)	Survival rate (%)	70.00 +/- 0.74	70.00 +/- 0.74	3	3	Hedges' g	0.0	-1.28 to 1.28	No observed mean difference; unadjusted 95% CI included 0	Not applicable - effect size estimated using reconstructed common SD (pooled SEM x sqrt[3])
Bae et al. (2020)	Whiteleg shrimp (Litopenaeus vannamei)	Soybean meal, fermented soybean meal, or sesame meal replacing 20% or 40% of dietary fishmeal	ST40 (40% fishmeal replacement with fermented soybean meal)	CON (30% fishmeal control diet)	Lysozyme activity (U/L)	1.630 +/- 0.035	1.620 +/- 0.035	3	3	Hedges' g	0.23	-1.06 to 1.52	Higher, more favorable value with replacement; unadjusted 95% CI included 0	Not applicable - effect size estimated using reconstructed common SD (pooled SEM x sqrt[3])
Bae et al. (2020)	Whiteleg shrimp (Litopenaeus vannamei)	Soybean meal, fermented soybean meal, or sesame meal replacing 20% or 40% of dietary fishmeal	ST40 (40% fishmeal replacement with fermented soybean meal)	CON (30% fishmeal control diet)	Superoxide dismutase activity (% inhibition)	87.20 +/- 1.65	87.10 +/- 1.65	3	3	Hedges' g	0.05	-1.23 to 1.33	Higher, more favorable value with replacement; unadjusted 95% CI included 0	Not applicable - effect size estimated using reconstructed common SD (pooled SEM x sqrt[3])
Bae et al. (2020)	Whiteleg shrimp (Litopenaeus vannamei)	Soybean meal, fermented soybean meal, or sesame meal replacing 20% or 40% of dietary fishmeal	ST40 (40% fishmeal replacement with fermented soybean meal)	CON (30% fishmeal control diet)	Moisture (% dry-matter basis)	6.420 +/- 0.121	6.080 +/- 0.121	3	3	Hedges' g	2.24	0.23 to 4.26	Higher measured value with replacement; unadjusted 95% CI excluded 0	Not applicable - effect size estimated using reconstructed common SD (pooled SEM x sqrt[3])
Bae et al. (2020)	Whiteleg shrimp (Litopenaeus vannamei)	Soybean meal, fermented soybean meal, or sesame meal replacing 20% or 40% of dietary fishmeal	ST40 (40% fishmeal replacement with fermented soybean meal)	CON (30% fishmeal control diet)	Crude protein (% dry-matter basis)	75.20 +/- 0.38	76.30 +/- 0.38	3	3	Hedges' g	-2.31	-4.36 to -0.26	Lower measured value with replacement; unadjusted 95% CI excluded 0	Not applicable - effect size estimated using reconstructed common SD (pooled SEM x sqrt[3])
Bae et al. (2020)	Whiteleg shrimp (Litopenaeus vannamei)	Soybean meal, fermented soybean meal, or sesame meal replacing 20% or 40% of dietary fishmeal	ST40 (40% fishmeal replacement with fermented soybean meal)	CON (30% fishmeal control diet)	Crude lipid (% dry-matter basis)	1.770 +/- 0.087	1.840 +/- 0.087	3	3	Hedges' g	-0.65	-2.00 to 0.71	Lower measured value with replacement; unadjusted 95% CI included 0	Not applicable - effect size estimated using reconstructed common SD (pooled SEM x sqrt[3])
Bae et al. (2020)	Whiteleg shrimp (Litopenaeus vannamei)	Soybean meal, fermented soybean meal, or sesame meal replacing 20% or 40% of dietary fishmeal	ST40 (40% fishmeal replacement with fermented soybean meal)	CON (30% fishmeal control diet)	Ash (% dry-matter basis)	14.00 +/- 0.28	14.20 +/- 0.28	3	3	Hedges' g	-0.58	-1.92 to 0.76	Lower measured value with replacement; unadjusted 95% CI included 0	Not applicable - effect size estimated using reconstructed common SD (pooled SEM x sqrt[3])
Bae et al. (2020)	Whiteleg shrimp (Litopenaeus vannamei)	Soybean meal, fermented soybean meal, or sesame meal replacing 20% or 40% of dietary fishmeal	ST40 (40% fishmeal replacement with fermented soybean meal)	CON (30% fishmeal control diet)	Glucose (mg/dL)	45.50 +/- 7.86	51.70 +/- 7.86	3	3	Hedges' g	-0.63	-1.98 to 0.72	Lower measured value with replacement; unadjusted 95% CI included 0	Not applicable - effect size estimated using reconstructed common SD (pooled SEM x sqrt[3])
Bae et al. (2020)	Whiteleg shrimp (Litopenaeus vannamei)	Soybean meal, fermented soybean meal, or sesame meal replacing 20% or 40% of dietary fishmeal	ST40 (40% fishmeal replacement with fermented soybean meal)	CON (30% fishmeal control diet)	Total protein (g/dL)	10.20 +/- 1.00	10.60 +/- 1.00	3	3	Hedges' g	-0.32	-1.62 to 0.98	Lower measured value with replacement; unadjusted 95% CI included 0	Not applicable - effect size estimated using reconstructed common SD (pooled SEM x sqrt[3])
Bae et al. (2020)	Whiteleg shrimp (Litopenaeus vannamei)	Soybean meal, fermented soybean meal, or sesame meal replacing 20% or 40% of dietary fishmeal	ST40 (40% fishmeal replacement with fermented soybean meal)	CON (30% fishmeal control diet)	Glutamic oxaloacetic transaminase (U/L)	64.70 +/- 7.81	63.30 +/- 7.81	3	3	Hedges' g	0.14	-1.14 to 1.43	Higher measured value with replacement; unadjusted 95% CI included 0	Not applicable - effect size estimated using reconstructed common SD (pooled SEM x sqrt[3])
Bae et al. (2020)	Whiteleg shrimp (Litopenaeus vannamei)	Soybean meal, fermented soybean meal, or sesame meal replacing 20% or 40% of dietary fishmeal	ST40 (40% fishmeal replacement with fermented soybean meal)	CON (30% fishmeal control diet)	Glutamic-pyruvic transaminase (U/L)	14.50 +/- 1.99	15.00 +/- 1.99	3	3	Hedges' g	-0.2	-1.49 to 1.09	Lower measured value with replacement; unadjusted 95% CI included 0	Not applicable - effect size estimated using reconstructed common SD (pooled SEM x sqrt[3])
Bae et al. (2020)	Whiteleg shrimp (Litopenaeus vannamei)	Soybean meal, fermented soybean meal, or sesame meal replacing 20% or 40% of dietary fishmeal	SM20 (20% fishmeal replacement with sesame meal)	CON (30% fishmeal control diet)	Weight gain (%)	358.00 +/- 18.71	391.00 +/- 18.71	3	3	Hedges' g	-1.41	-3.02 to 0.20	Lower, less favorable value with replacement; unadjusted 95% CI included 0	Not applicable - effect size estimated using reconstructed common SD (pooled SEM x sqrt[3])
Bae et al. (2020)	Whiteleg shrimp (Litopenaeus vannamei)	Soybean meal, fermented soybean meal, or sesame meal replacing 20% or 40% of dietary fishmeal	SM20 (20% fishmeal replacement with sesame meal)	CON (30% fishmeal control diet)	Specific growth rate (%/day)	3.380 +/- 0.087	3.540 +/- 0.087	3	3	Hedges' g	-1.48	-3.12 to 0.16	Lower, less favorable value with replacement; unadjusted 95% CI included 0	Not applicable - effect size estimated using reconstructed common SD (pooled SEM x sqrt[3])
Bae et al. (2020)	Whiteleg shrimp (Litopenaeus vannamei)	Soybean meal, fermented soybean meal, or sesame meal replacing 20% or 40% of dietary fishmeal	SM20 (20% fishmeal replacement with sesame meal)	CON (30% fishmeal control diet)	Feed efficiency (%)	137.00 +/- 5.94	124.00 +/- 5.94	3	3	Hedges' g	1.75	-0.01 to 3.51	Higher, more favorable value with replacement; unadjusted 95% CI included 0	Not applicable - effect size estimated using reconstructed common SD (pooled SEM x sqrt[3])
Bae et al. (2020)	Whiteleg shrimp (Litopenaeus vannamei)	Soybean meal, fermented soybean meal, or sesame meal replacing 20% or 40% of dietary fishmeal	SM20 (20% fishmeal replacement with sesame meal)	CON (30% fishmeal control diet)	Protein efficiency ratio	2.700 +/- 0.139	2.970 +/- 0.139	3	3	Hedges' g	-1.56	-3.23 to 0.12	Lower, less favorable value with replacement; unadjusted 95% CI included 0	Not applicable - effect size estimated using reconstructed common SD (pooled SEM x sqrt[3])
Bae et al. (2020)	Whiteleg shrimp (Litopenaeus vannamei)	Soybean meal, fermented soybean meal, or sesame meal replacing 20% or 40% of dietary fishmeal	SM20 (20% fishmeal replacement with sesame meal)	CON (30% fishmeal control diet)	Survival rate (%)	66.70 +/- 0.74	70.00 +/- 0.74	3	3	Hedges' g	-3.54	-6.31 to -0.77	Lower, less favorable value with replacement; unadjusted 95% CI excluded 0	Not applicable - effect size estimated using reconstructed common SD (pooled SEM x sqrt[3])
Bae et al. (2020)	Whiteleg shrimp (Litopenaeus vannamei)	Soybean meal, fermented soybean meal, or sesame meal replacing 20% or 40% of dietary fishmeal	SM20 (20% fishmeal replacement with sesame meal)	CON (30% fishmeal control diet)	Lysozyme activity (U/L)	1.610 +/- 0.035	1.620 +/- 0.035	3	3	Hedges' g	-0.23	-1.52 to 1.06	Lower, less favorable value with replacement; unadjusted 95% CI included 0	Not applicable - effect size estimated using reconstructed common SD (pooled SEM x sqrt[3])
Bae et al. (2020)	Whiteleg shrimp (Litopenaeus vannamei)	Soybean meal, fermented soybean meal, or sesame meal replacing 20% or 40% of dietary fishmeal	SM20 (20% fishmeal replacement with sesame meal)	CON (30% fishmeal control diet)	Superoxide dismutase activity (% inhibition)	84.90 +/- 1.65	87.10 +/- 1.65	3	3	Hedges' g	-1.07	-2.55 to 0.41	Lower, less favorable value with replacement; unadjusted 95% CI included 0	Not applicable - effect size estimated using reconstructed common SD (pooled SEM x sqrt[3])
Bae et al. (2020)	Whiteleg shrimp (Litopenaeus vannamei)	Soybean meal, fermented soybean meal, or sesame meal replacing 20% or 40% of dietary fishmeal	SM20 (20% fishmeal replacement with sesame meal)	CON (30% fishmeal control diet)	Moisture (% dry-matter basis)	6.190 +/- 0.121	6.080 +/- 0.121	3	3	Hedges' g	0.73	-0.65 to 2.10	Higher measured value with replacement; unadjusted 95% CI included 0	Not applicable - effect size estimated using reconstructed common SD (pooled SEM x sqrt[3])
Bae et al. (2020)	Whiteleg shrimp (Litopenaeus vannamei)	Soybean meal, fermented soybean meal, or sesame meal replacing 20% or 40% of dietary fishmeal	SM20 (20% fishmeal replacement with sesame meal)	CON (30% fishmeal control diet)	Crude protein (% dry-matter basis)	75.70 +/- 0.38	76.30 +/- 0.38	3	3	Hedges' g	-1.26	-2.81 to 0.29	Lower measured value with replacement; unadjusted 95% CI included 0	Not applicable - effect size estimated using reconstructed common SD (pooled SEM x sqrt[3])
Bae et al. (2020)	Whiteleg shrimp (Litopenaeus vannamei)	Soybean meal, fermented soybean meal, or sesame meal replacing 20% or 40% of dietary fishmeal	SM20 (20% fishmeal replacement with sesame meal)	CON (30% fishmeal control diet)	Crude lipid (% dry-matter basis)	1.970 +/- 0.087	1.840 +/- 0.087	3	3	Hedges' g	1.2	-0.33 to 2.73	Higher measured value with replacement; unadjusted 95% CI included 0	Not applicable - effect size estimated using reconstructed common SD (pooled SEM x sqrt[3])
Bae et al. (2020)	Whiteleg shrimp (Litopenaeus vannamei)	Soybean meal, fermented soybean meal, or sesame meal replacing 20% or 40% of dietary fishmeal	SM20 (20% fishmeal replacement with sesame meal)	CON (30% fishmeal control diet)	Ash (% dry-matter basis)	14.10 +/- 0.28	14.20 +/- 0.28	3	3	Hedges' g	-0.29	-1.58 to 1.01	Lower measured value with replacement; unadjusted 95% CI included 0	Not applicable - effect size estimated using reconstructed common SD (pooled SEM x sqrt[3])
Bae et al. (2020)	Whiteleg shrimp (Litopenaeus vannamei)	Soybean meal, fermented soybean meal, or sesame meal replacing 20% or 40% of dietary fishmeal	SM20 (20% fishmeal replacement with sesame meal)	CON (30% fishmeal control diet)	Glucose (mg/dL)	49.00 +/- 7.86	51.70 +/- 7.86	3	3	Hedges' g	-0.27	-1.57 to 1.02	Lower measured value with replacement; unadjusted 95% CI included 0	Not applicable - effect size estimated using reconstructed common SD (pooled SEM x sqrt[3])

Study	Species	Strategy	Intervention	Comparator	Outcome and unit	Intervention mean +/- SD	Comparator mean +/- SD	n intervention (tanks)	n comparator (tanks)	Effect-size metric	Effect size	95% CI	Interpretation	Reason not estimated
Bae et al. (2020)	Whiteleg shrimp (Litopenaeus vannamei)	Soybean meal, fermented soybean meal, or sesame meal replacing 20% or 40% of dietary fishmeal	SM20 (20% fishmeal replacement with sesame meal)	CON (30% fishmeal control diet)	Total protein (g/dL)	9.570 +/- 1.005	10.60 +/- 1.00	3	3	Hedges' g	-0.82	-2.22 to 0.58	Lower measured value with replacement; unadjusted 95% CI included 0	Not applicable - effect size estimated using reconstructed common SD (pooled SEM x sqrt[3])
Bae et al. (2020)	Whiteleg shrimp (Litopenaeus vannamei)	Soybean meal, fermented soybean meal, or sesame meal replacing 20% or 40% of dietary fishmeal	SM20 (20% fishmeal replacement with sesame meal)	CON (30% fishmeal control diet)	Glutamic oxaloacetic transaminase (U/L)	69.80 +/- 7.81	63.30 +/- 7.81	3	3	Hedges' g	0.67	-0.70 to 2.03	Higher measured value with replacement; unadjusted 95% CI included 0	Not applicable - effect size estimated using reconstructed common SD (pooled SEM x sqrt[3])
Bae et al. (2020)	Whiteleg shrimp (Litopenaeus vannamei)	Soybean meal, fermented soybean meal, or sesame meal replacing 20% or 40% of dietary fishmeal	SM20 (20% fishmeal replacement with sesame meal)	CON (30% fishmeal control diet)	Glutamic-pyruvic transaminase (U/L)	15.40 +/- 1.99	15.00 +/- 1.99	3	3	Hedges' g	0.16	-1.12 to 1.45	Higher measured value with replacement; unadjusted 95% CI included 0	Not applicable - effect size estimated using reconstructed common SD (pooled SEM x sqrt[3])
Bae et al. (2020)	Whiteleg shrimp (Litopenaeus vannamei)	Soybean meal, fermented soybean meal, or sesame meal replacing 20% or 40% of dietary fishmeal	SM40 (40% fishmeal replacement with sesame meal)	CON (30% fishmeal control diet)	Weight gain (%)	334.00 +/- 18.71	391.00 +/- 18.71	3	3	Hedges' g	-2.44	-4.56 to -0.32	Lower, less favorable value with replacement; unadjusted 95% CI excluded 0	Not applicable - effect size estimated using reconstructed common SD (pooled SEM x sqrt[3])
Bae et al. (2020)	Whiteleg shrimp (Litopenaeus vannamei)	Soybean meal, fermented soybean meal, or sesame meal replacing 20% or 40% of dietary fishmeal	SM40 (40% fishmeal replacement with sesame meal)	CON (30% fishmeal control diet)	Specific growth rate (%/day)	3.250 +/- 0.087	3.540 +/- 0.087	3	3	Hedges' g	-2.68	-4.93 to -0.42	Lower, less favorable value with replacement; unadjusted 95% CI excluded 0	Not applicable - effect size estimated using reconstructed common SD (pooled SEM x sqrt[3])
Bae et al. (2020)	Whiteleg shrimp (Litopenaeus vannamei)	Soybean meal, fermented soybean meal, or sesame meal replacing 20% or 40% of dietary fishmeal	SM40 (40% fishmeal replacement with sesame meal)	CON (30% fishmeal control diet)	Feed efficiency (%)	106.00 +/- 5.94	124.00 +/- 5.94	3	3	Hedges' g	-2.42	-4.54 to -0.31	Lower, less favorable value with replacement; unadjusted 95% CI excluded 0	Not applicable - effect size estimated using reconstructed common SD (pooled SEM x sqrt[3])
Bae et al. (2020)	Whiteleg shrimp (Litopenaeus vannamei)	Soybean meal, fermented soybean meal, or sesame meal replacing 20% or 40% of dietary fishmeal	SM40 (40% fishmeal replacement with sesame meal)	CON (30% fishmeal control diet)	Protein efficiency ratio	2.530 +/- 0.139	2.970 +/- 0.139	3	3	Hedges' g	-2.54	-4.72 to -0.36	Lower, less favorable value with replacement; unadjusted 95% CI excluded 0	Not applicable - effect size estimated using reconstructed common SD (pooled SEM x sqrt[3])
Bae et al. (2020)	Whiteleg shrimp (Litopenaeus vannamei)	Soybean meal, fermented soybean meal, or sesame meal replacing 20% or 40% of dietary fishmeal	SM40 (40% fishmeal replacement with sesame meal)	CON (30% fishmeal control diet)	Survival rate (%)	68.30 +/- 0.74	70.00 +/- 0.74	3	3	Hedges' g	-1.83	-3.63 to -0.03	Lower, less favorable value with replacement; unadjusted 95% CI excluded 0	Not applicable - effect size estimated using reconstructed common SD (pooled SEM x sqrt[3])
Bae et al. (2020)	Whiteleg shrimp (Litopenaeus vannamei)	Soybean meal, fermented soybean meal, or sesame meal replacing 20% or 40% of dietary fishmeal	SM40 (40% fishmeal replacement with sesame meal)	CON (30% fishmeal control diet)	Lysozyme activity (U/L)	1.550 +/- 0.035	1.620 +/- 0.035	3	3	Hedges' g	-1.62	-3.32 to 0.08	Lower, less favorable value with replacement; unadjusted 95% CI included 0	Not applicable - effect size estimated using reconstructed common SD (pooled SEM x sqrt[3])
Bae et al. (2020)	Whiteleg shrimp (Litopenaeus vannamei)	Soybean meal, fermented soybean meal, or sesame meal replacing 20% or 40% of dietary fishmeal	SM40 (40% fishmeal replacement with sesame meal)	CON (30% fishmeal control diet)	Superoxide dismutase activity (% inhibition)	81.70 +/- 1.65	87.10 +/- 1.65	3	3	Hedges' g	-2.63	-4.85 to -0.40	Lower, less favorable value with replacement; unadjusted 95% CI excluded 0	Not applicable - effect size estimated using reconstructed common SD (pooled SEM x sqrt[3])
Bae et al. (2020)	Whiteleg shrimp (Litopenaeus vannamei)	Soybean meal, fermented soybean meal, or sesame meal replacing 20% or 40% of dietary fishmeal	SM40 (40% fishmeal replacement with sesame meal)	CON (30% fishmeal control diet)	Moisture (% dry-matter basis)	6.020 +/- 0.121	6.080 +/- 0.121	3	3	Hedges' g	-0.4	-1.71 to 0.91	Lower measured value with replacement; unadjusted 95% CI included 0	Not applicable - effect size estimated using reconstructed common SD (pooled SEM x sqrt[3])
Bae et al. (2020)	Whiteleg shrimp (Litopenaeus vannamei)	Soybean meal, fermented soybean meal, or sesame meal replacing 20% or 40% of dietary fishmeal	SM40 (40% fishmeal replacement with sesame meal)	CON (30% fishmeal control diet)	Crude protein (% dry-matter basis)	76.20 +/- 0.38	76.30 +/- 0.38	3	3	Hedges' g	-0.21	-1.50 to 1.08	Lower measured value with replacement; unadjusted 95% CI included 0	Not applicable - effect size estimated using reconstructed common SD (pooled SEM x sqrt[3])
Bae et al. (2020)	Whiteleg shrimp (Litopenaeus vannamei)	Soybean meal, fermented soybean meal, or sesame meal replacing 20% or 40% of dietary fishmeal	SM40 (40% fishmeal replacement with sesame meal)	CON (30% fishmeal control diet)	Crude lipid (% dry-matter basis)	1.980 +/- 0.087	1.840 +/- 0.087	3	3	Hedges' g	1.29	-0.27 to 2.86	Higher measured value with replacement; unadjusted 95% CI included 0	Not applicable - effect size estimated using reconstructed common SD (pooled SEM x sqrt[3])
Bae et al. (2020)	Whiteleg shrimp (Litopenaeus vannamei)	Soybean meal, fermented soybean meal, or sesame meal replacing 20% or 40% of dietary fishmeal	SM40 (40% fishmeal replacement with sesame meal)	CON (30% fishmeal control diet)	Ash (% dry-matter basis)	16.60 +/- 0.28	14.20 +/- 0.28	3	3	Hedges' g	6.93	1.96 to 11.90	Higher measured value with replacement; unadjusted 95% CI excluded 0	Not applicable - effect size estimated using reconstructed common SD (pooled SEM x sqrt[3])
Bae et al. (2020)	Whiteleg shrimp (Litopenaeus vannamei)	Soybean meal, fermented soybean meal, or sesame meal replacing 20% or 40% of dietary fishmeal	SM40 (40% fishmeal replacement with sesame meal)	CON (30% fishmeal control diet)	Glucose (mg/dL)	46.50 +/- 7.86	51.70 +/- 7.86	3	3	Hedges' g	-0.53	-1.86 to 0.80	Lower measured value with replacement; unadjusted 95% CI included 0	Not applicable - effect size estimated using reconstructed common SD (pooled SEM x sqrt[3])
Bae et al. (2020)	Whiteleg shrimp (Litopenaeus vannamei)	Soybean meal, fermented soybean meal, or sesame meal replacing 20% or 40% of dietary fishmeal	SM40 (40% fishmeal replacement with sesame meal)	CON (30% fishmeal control diet)	Total protein (g/dL)	9.600 +/- 1.005	10.60 +/- 1.00	3	3	Hedges' g	-0.8	-2.19 to 0.60	Lower measured value with replacement; unadjusted 95% CI included 0	Not applicable - effect size estimated using reconstructed common SD (pooled SEM x sqrt[3])
Bae et al. (2020)	Whiteleg shrimp (Litopenaeus vannamei)	Soybean meal, fermented soybean meal, or sesame meal replacing 20% or 40% of dietary fishmeal	SM40 (40% fishmeal replacement with sesame meal)	CON (30% fishmeal control diet)	Glutamic oxaloacetic transaminase (U/L)	65.30 +/- 7.81	63.30 +/- 7.81	3	3	Hedges' g	0.2	-1.08 to 1.49	Higher measured value with replacement; unadjusted 95% CI included 0	Not applicable - effect size estimated using reconstructed common SD (pooled SEM x sqrt[3])
Bae et al. (2020)	Whiteleg shrimp (Litopenaeus vannamei)	Soybean meal, fermented soybean meal, or sesame meal replacing 20% or 40% of dietary fishmeal	SM40 (40% fishmeal replacement with sesame meal)	CON (30% fishmeal control diet)	Glutamic-pyruvic transaminase (U/L)	14.30 +/- 1.99	15.00 +/- 1.99	3	3	Hedges' g	-0.28	-1.58 to 1.01	Lower measured value with replacement; unadjusted 95% CI included 0	Not applicable - effect size estimated using reconstructed common SD (pooled SEM x sqrt[3])

Note: Values are published treatment means from three independent tanks per diet. Tables 2-5 provide one pooled SEM per outcome and define pooled SEM = SD/sqrt(n); therefore, a common SD was reconstructed as pooled SEM x sqrt(3). Each replacement diet was compared with the CON diet. Pairwise 95% CIs are descriptive and unadjusted. Table 2 contains an internal feed-efficiency inconsistency: the printed ST20 and SM20 values conflict with the narrative and superscript interpretation; the numerical values were retained exactly as published.

Source: Bae et al. (2020), https://doi.org/10.1186/s41240-020-0148-x

Study 20. Hamidoghli et al. (2023): graded yeast hydrolysate supplementation in juvenile whiteleg shrimp

Study	Species	Strategy	Intervention	Comparator	Outcome and unit	Intervention mean +/- SD	Comparator mean +/- SD	n intervention (tanks)	n comparator (tanks)	Effect-size metric	Effect size	95% CI	Interpretation	Reason not estimated
Hamidoghli et al. (2023)	Whiteleg shrimp (Litopenaeus vannamei)	Graded yeast hydrolysate supplementation in a 10% fishmeal diet	YH0.5 (10% fishmeal + 0.5% yeast hydrolysate)	CON (10% fishmeal, no yeast hydrolysate)	Final body weight (g)	2.280 +/- 0.150	2.290 +/- 0.130	3	3	Hedges' g	-0.06	-1.34 to 1.22	Lower, less favorable value with supplementation; unadjusted 95% CI included 0	Not applicable - effect size estimated from reported mean +/- SD
Hamidoghli et al. (2023)	Whiteleg shrimp (Litopenaeus vannamei)	Graded yeast hydrolysate supplementation in a 10% fishmeal diet	YH0.5 (10% fishmeal + 0.5% yeast hydrolysate)	CON (10% fishmeal, no yeast hydrolysate)	Weight gain (%)	426.70 +/- 30.61	434.27 +/- 33.25	3	3	Hedges' g	-0.19	-1.48 to 1.10	Lower, less favorable value with supplementation; unadjusted 95% CI included 0	Not applicable - effect size estimated from reported mean +/- SD
Hamidoghli et al. (2023)	Whiteleg shrimp (Litopenaeus vannamei)	Graded yeast hydrolysate supplementation in a 10% fishmeal diet	YH0.5 (10% fishmeal + 0.5% yeast hydrolysate)	CON (10% fishmeal, no yeast hydrolysate)	Specific growth rate (%/day)	3.460 +/- 0.120	3.490 +/- 0.130	3	3	Hedges' g	-0.19	-1.48 to 1.10	Lower, less favorable value with supplementation; unadjusted 95% CI included 0	Not applicable - effect size estimated from reported mean +/- SD
Hamidoghli et al. (2023)	Whiteleg shrimp (Litopenaeus vannamei)	Graded yeast hydrolysate supplementation in a 10% fishmeal diet	YH0.5 (10% fishmeal + 0.5% yeast hydrolysate)	CON (10% fishmeal, no yeast hydrolysate)	Feed efficiency (%)	34.39 +/- 0.77	36.56 +/- 3.55	3	3	Hedges' g	-0.68	-2.04 to 0.69	Lower, less favorable value with supplementation; unadjusted 95% CI included 0	Not applicable - effect size estimated from reported mean +/- SD
Hamidoghli et al. (2023)	Whiteleg shrimp (Litopenaeus vannamei)	Graded yeast hydrolysate supplementation in a 10% fishmeal diet	YH0.5 (10% fishmeal + 0.5% yeast hydrolysate)	CON (10% fishmeal, no yeast hydrolysate)	Protein efficiency ratio	0.940 +/- 0.030	1.000 +/- 0.090	3	3	Hedges' g	-0.72	-2.09 to 0.66	Lower, less favorable value with supplementation; unadjusted 95% CI included 0	Not applicable - effect size estimated from reported mean +/- SD
Hamidoghli et al. (2023)	Whiteleg shrimp (Litopenaeus vannamei)	Graded yeast hydrolysate supplementation in a 10% fishmeal diet	YH0.5 (10% fishmeal + 0.5% yeast hydrolysate)	CON (10% fishmeal, no yeast hydrolysate)	Survival rate (%)	81.67 +/- 4.71	90.00 +/- 4.08	3	3	Hedges' g	-1.51	-3.17 to 0.14	Lower, less favorable value with supplementation; unadjusted 95% CI included 0	Not applicable - effect size estimated from reported mean +/- SD
Hamidoghli et al. (2023)	Whiteleg shrimp (Litopenaeus vannamei)	Graded yeast hydrolysate supplementation in a 10% fishmeal diet	YH0.5 (10% fishmeal + 0.5% yeast hydrolysate)	CON (10% fishmeal, no yeast hydrolysate)	Moisture (% wet weight)	76.07 +/- 0.44	75.83 +/- 0.42	3	3	Hedges' g	0.45	-0.87 to 1.76	Higher measured value with supplementation; unadjusted 95% CI included 0	Not applicable - effect size estimated from reported mean +/- SD
Hamidoghli et al. (2023)	Whiteleg shrimp (Litopenaeus vannamei)	Graded yeast hydrolysate supplementation in a 10% fishmeal diet	YH0.5 (10% fishmeal + 0.5% yeast hydrolysate)	CON (10% fishmeal, no yeast hydrolysate)	Crude protein (% wet weight)	18.58 +/- 0.03	18.56 +/- 0.06	3	3	Hedges' g	0.34	-0.96 to 1.64	Higher measured value with supplementation; unadjusted 95% CI included 0	Not applicable - effect size estimated from reported mean +/- SD
Hamidoghli et al. (2023)	Whiteleg shrimp (Litopenaeus vannamei)	Graded yeast hydrolysate supplementation in a 10% fishmeal diet	YH0.5 (10% fishmeal + 0.5% yeast hydrolysate)	CON (10% fishmeal, no yeast hydrolysate)	Crude lipid (% wet weight)	1.310 +/- 0.040	1.290 +/- 0.050	3	3	Hedges' g	0.35	-0.95 to 1.66	Higher measured value with supplementation; unadjusted 95% CI included 0	Not applicable - effect size estimated from reported mean +/- SD
Hamidoghli et al. (2023)	Whiteleg shrimp (Litopenaeus vannamei)	Graded yeast hydrolysate supplementation in a 10% fishmeal diet	YH0.5 (10% fishmeal + 0.5% yeast hydrolysate)	CON (10% fishmeal, no yeast hydrolysate)	Crude ash (% wet weight)	3.440 +/- 0.040	3.430 +/- 0.020	3	3	Hedges' g	0.25	-1.04 to 1.55	Higher measured value with supplementation; unadjusted 95% CI included 0	Not applicable - effect size estimated from reported mean +/- SD
Hamidoghli et al. (2023)	Whiteleg shrimp (Litopenaeus vannamei)	Graded yeast hydrolysate supplementation in a 10% fishmeal diet	YH0.5 (10% fishmeal + 0.5% yeast hydrolysate)	CON (10% fishmeal, no yeast hydrolysate)	Aspartate aminotransferase (U/L)	12.67 +/- 3.79	10.67 +/- 2.08	3	3	Hedges' g	0.52	-0.81 to 1.85	Higher measured value with supplementation; unadjusted 95% CI included 0	Not applicable - effect size estimated from reported mean +/- SD
Hamidoghli et al. (2023)	Whiteleg shrimp (Litopenaeus vannamei)	Graded yeast hydrolysate supplementation in a 10% fishmeal diet	YH0.5 (10% fishmeal + 0.5% yeast hydrolysate)	CON (10% fishmeal, no yeast hydrolysate)	Alanine aminotransferase (U/L)	9.670 +/- 1.530	9.000 +/- 1.730	3	3	Hedges' g	0.33	-0.97 to 1.63	Higher measured value with supplementation; unadjusted 95% CI included 0	Not applicable - effect size estimated from reported mean +/- SD
Hamidoghli et al. (2023)	Whiteleg shrimp (Litopenaeus vannamei)	Graded yeast hydrolysate supplementation in a 10% fishmeal diet	YH0.5 (10% fishmeal + 0.5% yeast hydrolysate)	CON (10% fishmeal, no yeast hydrolysate)	Glucose (mg/dL)	61.33 +/- 15.01	57.33 +/- 11.15	3	3	Hedges' g	0.24	-1.05 to 1.53	Higher measured value with supplementation; unadjusted 95% CI included 0	Not applicable - effect size estimated from reported mean +/- SD
Hamidoghli et al. (2023)	Whiteleg shrimp (Litopenaeus vannamei)	Graded yeast hydrolysate supplementation in a 10% fishmeal diet	YH0.5 (10% fishmeal + 0.5% yeast hydrolysate)	CON (10% fishmeal, no yeast hydrolysate)	Superoxide dismutase activity (% inhibition)	34.37 +/- 5.88	43.60 +/- 7.96	3	3	Hedges' g	-1.06	-2.53 to 0.42	Lower, less favorable value with supplementation; unadjusted 95% CI included 0	Not applicable - effect size estimated from reported mean +/- SD
Hamidoghli et al. (2023)	Whiteleg shrimp (Litopenaeus vannamei)	Graded yeast hydrolysate supplementation in a 10% fishmeal diet	YH0.5 (10% fishmeal + 0.5% yeast hydrolysate)	CON (10% fishmeal, no yeast hydrolysate)	Lysozyme activity (U/mL)	0.076 +/- 0.010	0.082 +/- 0.010	3	3	Hedges' g	-0.48	-1.80 to 0.84	Lower, less favorable value with supplementation; unadjusted 95% CI included 0	Not applicable - effect size estimated from reported mean +/- SD
Hamidoghli et al. (2023)	Whiteleg shrimp (Litopenaeus vannamei)	Graded yeast hydrolysate supplementation in a 10% fishmeal diet	YH0.5 (10% fishmeal + 0.5% yeast hydrolysate)	CON (10% fishmeal, no yeast hydrolysate)	Villus length (µm)	29.80 +/- 2.10	33.80 +/- 1.12	3	3	Hedges' g	-1.9	-3.74 to -0.06	Lower, less favorable value with supplementation; unadjusted 95% CI excluded 0	Not applicable - effect size estimated from reported mean +/- SD
Hamidoghli et al. (2023)	Whiteleg shrimp (Litopenaeus vannamei)	Graded yeast hydrolysate supplementation in a 10% fishmeal diet	YH0.5 (10% fishmeal + 0.5% yeast hydrolysate)	CON (10% fishmeal, no yeast hydrolysate)	Muscular layer thickness (µm)	49.96 +/- 2.26	47.02 +/- 1.50	3	3	Hedges' g	1.23	-0.31 to 2.76	Higher, more favorable value with supplementation; unadjusted 95% CI included 0	Not applicable - effect size estimated from reported mean +/- SD
Hamidoghli et al. (2023)	Whiteleg shrimp (Litopenaeus vannamei)	Graded yeast hydrolysate supplementation in a 10% fishmeal diet	YH1 (10% fishmeal + 1% yeast hydrolysate)	CON (10% fishmeal, no yeast hydrolysate)	Final body weight (g)	2.450 +/- 0.270	2.290 +/- 0.130	3	3	Hedges' g	0.6	-0.74 to 1.95	Higher, more favorable value with supplementation; unadjusted 95% CI included 0	Not applicable - effect size estimated from reported mean +/- SD
Hamidoghli et al. (2023)	Whiteleg shrimp (Litopenaeus vannamei)	Graded yeast hydrolysate supplementation in a 10% fishmeal diet	YH1 (10% fishmeal + 1% yeast hydrolysate)	CON (10% fishmeal, no yeast hydrolysate)	Weight gain (%)	464.70 +/- 59.43	434.27 +/- 33.25	3	3	Hedges' g	0.51	-0.82 to 1.83	Higher, more favorable value with supplementation; unadjusted 95% CI included 0	Not applicable - effect size estimated from reported mean +/- SD
Hamidoghli et al. (2023)	Whiteleg shrimp (Litopenaeus vannamei)	Graded yeast hydrolysate supplementation in a 10% fishmeal diet	YH1 (10% fishmeal + 1% yeast hydrolysate)	CON (10% fishmeal, no yeast hydrolysate)	Specific growth rate (%/day)	3.590 +/- 0.230	3.490 +/- 0.130	3	3	Hedges' g	0.43	-0.89 to 1.74	Higher, more favorable value with supplementation; unadjusted 95% CI included 0	Not applicable - effect size estimated from reported mean +/- SD
Hamidoghli et al. (2023)	Whiteleg shrimp (Litopenaeus vannamei)	Graded yeast hydrolysate supplementation in a 10% fishmeal diet	YH1 (10% fishmeal + 1% yeast hydrolysate)	CON (10% fishmeal, no yeast hydrolysate)	Feed efficiency (%)	36.57 +/- 2.97	36.56 +/- 3.55	3	3	Hedges' g	0.0	-1.28 to 1.28	Higher, more favorable value with supplementation; unadjusted 95% CI included 0	Not applicable - effect size estimated from reported mean +/- SD
Hamidoghli et al. (2023)	Whiteleg shrimp (Litopenaeus vannamei)	Graded yeast hydrolysate supplementation in a 10% fishmeal diet	YH1 (10% fishmeal + 1% yeast hydrolysate)	CON (10% fishmeal, no yeast hydrolysate)	Protein efficiency ratio	0.980 +/- 0.080	1.000 +/- 0.090	3	3	Hedges' g	-0.19	-1.47 to 1.10	Lower, less favorable value with supplementation; unadjusted 95% CI included 0	Not applicable - effect size estimated from reported mean +/- SD
Hamidoghli et al. (2023)	Whiteleg shrimp (Litopenaeus vannamei)	Graded yeast hydrolysate supplementation in a 10% fishmeal diet	YH1 (10% fishmeal + 1% yeast hydrolysate)	CON (10% fishmeal, no yeast hydrolysate)	Survival rate (%)	80.00 +/- 7.07	90.00 +/- 4.08	3	3	Hedges' g	-1.39	-2.99 to 0.21	Lower, less favorable value with supplementation; unadjusted 95% CI included 0	Not applicable - effect size estimated from reported mean +/- SD
Hamidoghli et al. (2023)	Whiteleg shrimp (Litopenaeus vannamei)	Graded yeast hydrolysate supplementation in a 10% fishmeal diet	YH1 (10% fishmeal + 1% yeast hydrolysate)	CON (10% fishmeal, no yeast hydrolysate)	Moisture (% wet weight)	75.82 +/- 0.17	75.83 +/- 0.42	3	3	Hedges' g	-0.02	-1.31 to 1.26	Lower measured value with supplementation; unadjusted 95% CI included 0	Not applicable - effect size estimated from reported mean +/- SD
Hamidoghli et al. (2023)	Whiteleg shrimp (Litopenaeus vannamei)	Graded yeast hydrolysate supplementation in a 10% fishmeal diet	YH1 (10% fishmeal + 1% yeast hydrolysate)	CON (10% fishmeal, no yeast hydrolysate)	Crude protein (% wet weight)	18.53 +/- 0.06	18.56 +/- 0.06	3	3	Hedges' g	-0.4	-1.71 to 0.91	Lower measured value with supplementation; unadjusted 95% CI included 0	Not applicable - effect size estimated from reported mean +/- SD
Hamidoghli et al. (2023)	Whiteleg shrimp (Litopenaeus vannamei)	Graded yeast hydrolysate supplementation in a 10% fishmeal diet	YH1 (10% fishmeal + 1% yeast hydrolysate)	CON (10% fishmeal, no yeast hydrolysate)	Crude lipid (% wet weight)	1.350 +/- 0.050	1.290 +/- 0.050	3	3	Hedges' g	0.96	-0.48 to 2.40	Higher measured value with supplementation; unadjusted 95% CI included 0	Not applicable - effect size estimated from reported mean +/- SD
Hamidoghli et al. (2023)	Whiteleg shrimp (Litopenaeus vannamei)	Graded yeast hydrolysate supplementation in a 10% fishmeal diet	YH1 (10% fishmeal + 1% yeast hydrolysate)	CON (10% fishmeal, no yeast hydrolysate)	Crude ash (% wet weight)	3.420 +/- 0.020	3.430 +/- 0.020	3	3	Hedges' g	-0.4	-1.71 to 0.91	Lower measured value with supplementation; unadjusted 95% CI included 0	Not applicable - effect size estimated from reported mean +/- SD
Hamidoghli et al. (2023)	Whiteleg shrimp (Litopenaeus vannamei)	Graded yeast hydrolysate supplementation in a 10% fishmeal diet	YH1 (10% fishmeal + 1% yeast hydrolysate)	CON (10% fishmeal, no yeast hydrolysate)	Aspartate aminotransferase (U/L)	13.67 +/- 2.89	10.67 +/- 2.08	3	3	Hedges' g	0.95	-0.49 to 2.39	Higher measured value with supplementation; unadjusted 95% CI included 0	Not applicable - effect size estimated from reported mean +/- SD
Hamidoghli et al. (2023)	Whiteleg shrimp (Litopenaeus vannamei)	Graded yeast hydrolysate supplementation in a 10% fishmeal diet	YH1 (10% fishmeal + 1% yeast hydrolysate)	CON (10% fishmeal, no yeast hydrolysate)	Alanine aminotransferase (U/L)	9.670 +/- 1.530	9.000 +/- 1.730	3	3	Hedges' g	0.33	-0.97 to 1.63	Higher measured value with supplementation; unadjusted 95% CI included 0	Not applicable - effect size estimated from reported mean +/- SD
Hamidoghli et al. (2023)	Whiteleg shrimp (Litopenaeus vannamei)	Graded yeast hydrolysate supplementation in a 10% fishmeal diet	YH1 (10% fishmeal + 1% yeast hydrolysate)	CON (10% fishmeal, no yeast hydrolysate)	Glucose (mg/dL)	57.67 +/- 8.14	57.33 +/- 11.15	3	3	Hedges' g	0.03	-1.25 to 1.31	Higher measured value with supplementation; unadjusted 95% CI included 0	Not applicable - effect size estimated from reported mean +/- SD
Hamidoghli et al. (2023)	Whiteleg shrimp (Litopenaeus vannamei)	Graded yeast hydrolysate supplementation in a 10% fishmeal diet	YH1 (10% fishmeal + 1% yeast hydrolysate)	CON (10% fishmeal, no yeast hydrolysate)	Superoxide dismutase activity (% inhibition)	39.66 +/- 4.64	43.60 +/- 7.96	3	3	Hedges' g	-0.48	-1.81 to 0.84	Lower, less favorable value with supplementation; unadjusted 95% CI included 0	Not applicable - effect size estimated from reported mean +/- SD

Study	Species	Strategy	Intervention	Comparator	Outcome and unit	Intervention mean +/- SD	Comparator mean +/- SD	n intervention (tanks)	n comparator (tanks)	Effect-size metric	Effect size	95% CI	Interpretation	Reason not estimated
Hamidoghli et al. (2023)	Whiteleg shrimp (Litopenaeus vannamei)	Graded yeast hydrolysate supplementation in a 10% fishmeal diet	YH1 (10% fishmeal + 1% yeast hydrolysate)	CON (10% fishmeal, no yeast hydrolysate)	Lysozyme activity (U/mL)	0.073 +/- 0.030	0.082 +/- 0.010	3	3	Hedges' g	-0.32	-1.62 to 0.98	Lower, less favorable value with supplementation; unadjusted 95% CI included 0	Not applicable - effect size estimated from reported mean +/- SD
Hamidoghli et al. (2023)	Whiteleg shrimp (Litopenaeus vannamei)	Graded yeast hydrolysate supplementation in a 10% fishmeal diet	YH1 (10% fishmeal + 1% yeast hydrolysate)	CON (10% fishmeal, no yeast hydrolysate)	Villus length (µm)	36.48 +/- 2.33	33.80 +/- 1.12	3	3	Hedges' g	1.17	-0.34 to 2.69	Higher, more favorable value with supplementation; unadjusted 95% CI included 0	Not applicable - effect size estimated from reported mean +/- SD
Hamidoghli et al. (2023)	Whiteleg shrimp (Litopenaeus vannamei)	Graded yeast hydrolysate supplementation in a 10% fishmeal diet	YH1 (10% fishmeal + 1% yeast hydrolysate)	CON (10% fishmeal, no yeast hydrolysate)	Muscular layer thickness (µm)	50.35 +/- 1.33	47.02 +/- 1.50	3	3	Hedges' g	1.88	0.05 to 3.71	Higher, more favorable value with supplementation; unadjusted 95% CI excluded 0	Not applicable - effect size estimated from reported mean +/- SD
Hamidoghli et al. (2023)	Whiteleg shrimp (Litopenaeus vannamei)	Graded yeast hydrolysate supplementation in a 10% fishmeal diet	YH2 (10% fishmeal + 2% yeast hydrolysate)	CON (10% fishmeal, no yeast hydrolysate)	Final body weight (g)	2.330 +/- 0.130	2.290 +/- 0.130	3	3	Hedges' g	0.25	-1.05 to 1.54	Higher, more favorable value with supplementation; unadjusted 95% CI included 0	Not applicable - effect size estimated from reported mean +/- SD
Hamidoghli et al. (2023)	Whiteleg shrimp (Litopenaeus vannamei)	Graded yeast hydrolysate supplementation in a 10% fishmeal diet	YH2 (10% fishmeal + 2% yeast hydrolysate)	CON (10% fishmeal, no yeast hydrolysate)	Weight gain (%)	439.17 +/- 23.86	434.27 +/- 33.25	3	3	Hedges' g	0.14	-1.15 to 1.42	Higher, more favorable value with supplementation; unadjusted 95% CI included 0	Not applicable - effect size estimated from reported mean +/- SD
Hamidoghli et al. (2023)	Whiteleg shrimp (Litopenaeus vannamei)	Graded yeast hydrolysate supplementation in a 10% fishmeal diet	YH2 (10% fishmeal + 2% yeast hydrolysate)	CON (10% fishmeal, no yeast hydrolysate)	Specific growth rate (%/day)	3.510 +/- 0.090	3.490 +/- 0.130	3	3	Hedges' g	0.14	-1.14 to 1.43	Higher, more favorable value with supplementation; unadjusted 95% CI included 0	Not applicable - effect size estimated from reported mean +/- SD
Hamidoghli et al. (2023)	Whiteleg shrimp (Litopenaeus vannamei)	Graded yeast hydrolysate supplementation in a 10% fishmeal diet	YH2 (10% fishmeal + 2% yeast hydrolysate)	CON (10% fishmeal, no yeast hydrolysate)	Feed efficiency (%)	35.17 +/- 1.65	36.56 +/- 3.55	3	3	Hedges' g	-0.4	-1.71 to 0.91	Lower, less favorable value with supplementation; unadjusted 95% CI included 0	Not applicable - effect size estimated from reported mean +/- SD
Hamidoghli et al. (2023)	Whiteleg shrimp (Litopenaeus vannamei)	Graded yeast hydrolysate supplementation in a 10% fishmeal diet	YH2 (10% fishmeal + 2% yeast hydrolysate)	CON (10% fishmeal, no yeast hydrolysate)	Protein efficiency ratio	0.970 +/- 0.030	1.000 +/- 0.090	3	3	Hedges' g	-0.36	-1.66 to 0.95	Lower, less favorable value with supplementation; unadjusted 95% CI included 0	Not applicable - effect size estimated from reported mean +/- SD
Hamidoghli et al. (2023)	Whiteleg shrimp (Litopenaeus vannamei)	Graded yeast hydrolysate supplementation in a 10% fishmeal diet	YH2 (10% fishmeal + 2% yeast hydrolysate)	CON (10% fishmeal, no yeast hydrolysate)	Survival rate (%)	78.33 +/- 4.71	90.00 +/- 4.08	3	3	Hedges' g	-2.12	-4.07 to -0.17	Lower, less favorable value with supplementation; unadjusted 95% CI excluded 0	Not applicable - effect size estimated from reported mean +/- SD
Hamidoghli et al. (2023)	Whiteleg shrimp (Litopenaeus vannamei)	Graded yeast hydrolysate supplementation in a 10% fishmeal diet	YH2 (10% fishmeal + 2% yeast hydrolysate)	CON (10% fishmeal, no yeast hydrolysate)	Moisture (% wet weight)	75.78 +/- 0.41	75.83 +/- 0.42	3	3	Hedges' g	-0.1	-1.38 to 1.19	Lower measured value with supplementation; unadjusted 95% CI included 0	Not applicable - effect size estimated from reported mean +/- SD
Hamidoghli et al. (2023)	Whiteleg shrimp (Litopenaeus vannamei)	Graded yeast hydrolysate supplementation in a 10% fishmeal diet	YH2 (10% fishmeal + 2% yeast hydrolysate)	CON (10% fishmeal, no yeast hydrolysate)	Crude protein (% wet weight)	18.53 +/- 0.01	18.56 +/- 0.06	3	3	Hedges' g	-0.56	-1.90 to 0.78	Lower measured value with supplementation; unadjusted 95% CI included 0	Not applicable - effect size estimated from reported mean +/- SD
Hamidoghli et al. (2023)	Whiteleg shrimp (Litopenaeus vannamei)	Graded yeast hydrolysate supplementation in a 10% fishmeal diet	YH2 (10% fishmeal + 2% yeast hydrolysate)	CON (10% fishmeal, no yeast hydrolysate)	Crude lipid (% wet weight)	1.300 +/- 0.060	1.290 +/- 0.050	3	3	Hedges' g	0.14	-1.14 to 1.43	Higher measured value with supplementation; unadjusted 95% CI included 0	Not applicable - effect size estimated from reported mean +/- SD
Hamidoghli et al. (2023)	Whiteleg shrimp (Litopenaeus vannamei)	Graded yeast hydrolysate supplementation in a 10% fishmeal diet	YH2 (10% fishmeal + 2% yeast hydrolysate)	CON (10% fishmeal, no yeast hydrolysate)	Crude ash (% wet weight)	3.400 +/- 0.010	3.430 +/- 0.020	3	3	Hedges' g	-1.52	-3.17 to 0.14	Lower measured value with supplementation; unadjusted 95% CI included 0	Not applicable - effect size estimated from reported mean +/- SD
Hamidoghli et al. (2023)	Whiteleg shrimp (Litopenaeus vannamei)	Graded yeast hydrolysate supplementation in a 10% fishmeal diet	YH2 (10% fishmeal + 2% yeast hydrolysate)	CON (10% fishmeal, no yeast hydrolysate)	Aspartate aminotransferase (U/L)	11.67 +/- 2.52	10.67 +/- 2.08	3	3	Hedges' g	0.35	-0.96 to 1.65	Higher measured value with supplementation; unadjusted 95% CI included 0	Not applicable - effect size estimated from reported mean +/- SD
Hamidoghli et al. (2023)	Whiteleg shrimp (Litopenaeus vannamei)	Graded yeast hydrolysate supplementation in a 10% fishmeal diet	YH2 (10% fishmeal + 2% yeast hydrolysate)	CON (10% fishmeal, no yeast hydrolysate)	Alanine aminotransferase (U/L)	10.33 +/- 2.08	9.000 +/- 1.730	3	3	Hedges' g	0.56	-0.78 to 1.89	Higher measured value with supplementation; unadjusted 95% CI included 0	Not applicable - effect size estimated from reported mean +/- SD
Hamidoghli et al. (2023)	Whiteleg shrimp (Litopenaeus vannamei)	Graded yeast hydrolysate supplementation in a 10% fishmeal diet	YH2 (10% fishmeal + 2% yeast hydrolysate)	CON (10% fishmeal, no yeast hydrolysate)	Glucose (mg/dL)	58.67 +/- 9.02	57.33 +/- 11.15	3	3	Hedges' g	0.11	-1.18 to 1.39	Higher measured value with supplementation; unadjusted 95% CI included 0	Not applicable - effect size estimated from reported mean +/- SD
Hamidoghli et al. (2023)	Whiteleg shrimp (Litopenaeus vannamei)	Graded yeast hydrolysate supplementation in a 10% fishmeal diet	YH2 (10% fishmeal + 2% yeast hydrolysate)	CON (10% fishmeal, no yeast hydrolysate)	Superoxide dismutase activity (% inhibition)	46.85 +/- 4.67	43.60 +/- 7.96	3	3	Hedges' g	0.4	-0.91 to 1.71	Higher, more favorable value with supplementation; unadjusted 95% CI included 0	Not applicable - effect size estimated from reported mean +/- SD
Hamidoghli et al. (2023)	Whiteleg shrimp (Litopenaeus vannamei)	Graded yeast hydrolysate supplementation in a 10% fishmeal diet	YH2 (10% fishmeal + 2% yeast hydrolysate)	CON (10% fishmeal, no yeast hydrolysate)	Lysozyme activity (U/mL)	0.080 +/- 0.030	0.082 +/- 0.010	3	3	Hedges' g	-0.07	-1.35 to 1.21	Lower, less favorable value with supplementation; unadjusted 95% CI included 0	Not applicable - effect size estimated from reported mean +/- SD
Hamidoghli et al. (2023)	Whiteleg shrimp (Litopenaeus vannamei)	Graded yeast hydrolysate supplementation in a 10% fishmeal diet	YH2 (10% fishmeal + 2% yeast hydrolysate)	CON (10% fishmeal, no yeast hydrolysate)	Villus length (µm)	30.05 +/- 1.16	33.80 +/- 1.12	3	3	Hedges' g	-2.63	-4.86 to -0.40	Lower, less favorable value with supplementation; unadjusted 95% CI excluded 0	Not applicable - effect size estimated from reported mean +/- SD
Hamidoghli et al. (2023)	Whiteleg shrimp (Litopenaeus vannamei)	Graded yeast hydrolysate supplementation in a 10% fishmeal diet	YH2 (10% fishmeal + 2% yeast hydrolysate)	CON (10% fishmeal, no yeast hydrolysate)	Muscular layer thickness (µm)	50.62 +/- 1.24	47.02 +/- 1.50	3	3	Hedges' g	2.09	0.16 to 4.03	Higher, more favorable value with supplementation; unadjusted 95% CI excluded 0	Not applicable - effect size estimated from reported mean +/- SD
Hamidoghli et al. (2023)	Whiteleg shrimp (Litopenaeus vannamei)	Graded yeast hydrolysate supplementation in a 10% fishmeal diet	YH4 (10% fishmeal + 4% yeast hydrolysate)	CON (10% fishmeal, no yeast hydrolysate)	Final body weight (g)	2.580 +/- 0.050	2.290 +/- 0.130	3	3	Hedges' g	2.36	0.28 to 4.43	Higher, more favorable value with supplementation; unadjusted 95% CI excluded 0	Not applicable - effect size estimated from reported mean +/- SD
Hamidoghli et al. (2023)	Whiteleg shrimp (Litopenaeus vannamei)	Graded yeast hydrolysate supplementation in a 10% fishmeal diet	YH4 (10% fishmeal + 4% yeast hydrolysate)	CON (10% fishmeal, no yeast hydrolysate)	Weight gain (%)	492.08 +/- 4.89	434.27 +/- 33.25	3	3	Hedges' g	1.95	0.09 to 3.81	Higher, more favorable value with supplementation; unadjusted 95% CI excluded 0	Not applicable - effect size estimated from reported mean +/- SD
Hamidoghli et al. (2023)	Whiteleg shrimp (Litopenaeus vannamei)	Graded yeast hydrolysate supplementation in a 10% fishmeal diet	YH4 (10% fishmeal + 4% yeast hydrolysate)	CON (10% fishmeal, no yeast hydrolysate)	Specific growth rate (%/day)	3.710 +/- 0.020	3.490 +/- 0.130	3	3	Hedges' g	1.89	0.06 to 3.73	Higher, more favorable value with supplementation; unadjusted 95% CI excluded 0	Not applicable - effect size estimated from reported mean +/- SD
Hamidoghli et al. (2023)	Whiteleg shrimp (Litopenaeus vannamei)	Graded yeast hydrolysate supplementation in a 10% fishmeal diet	YH4 (10% fishmeal + 4% yeast hydrolysate)	CON (10% fishmeal, no yeast hydrolysate)	Feed efficiency (%)	38.78 +/- 4.58	36.56 +/- 3.55	3	3	Hedges' g	0.43	-0.88 to 1.75	Higher, more favorable value with supplementation; unadjusted 95% CI included 0	Not applicable - effect size estimated from reported mean +/- SD
Hamidoghli et al. (2023)	Whiteleg shrimp (Litopenaeus vannamei)	Graded yeast hydrolysate supplementation in a 10% fishmeal diet	YH4 (10% fishmeal + 4% yeast hydrolysate)	CON (10% fishmeal, no yeast hydrolysate)	Protein efficiency ratio	1.050 +/- 0.090	1.000 +/- 0.090	3	3	Hedges' g	0.44	-0.87 to 1.76	Higher, more favorable value with supplementation; unadjusted 95% CI included 0	Not applicable - effect size estimated from reported mean +/- SD
Hamidoghli et al. (2023)	Whiteleg shrimp (Litopenaeus vannamei)	Graded yeast hydrolysate supplementation in a 10% fishmeal diet	YH4 (10% fishmeal + 4% yeast hydrolysate)	CON (10% fishmeal, no yeast hydrolysate)	Survival rate (%)	83.33 +/- 10.27	90.00 +/- 4.08	3	3	Hedges' g	-0.68	-2.05 to 0.68	Lower, less favorable value with supplementation; unadjusted 95% CI included 0	Not applicable - effect size estimated from reported mean +/- SD
Hamidoghli et al. (2023)	Whiteleg shrimp (Litopenaeus vannamei)	Graded yeast hydrolysate supplementation in a 10% fishmeal diet	YH4 (10% fishmeal + 4% yeast hydrolysate)	CON (10% fishmeal, no yeast hydrolysate)	Moisture (% wet weight)	76.01 +/- 0.58	75.83 +/- 0.42	3	3	Hedges' g	0.28	-1.01 to 1.58	Higher measured value with supplementation; unadjusted 95% CI included 0	Not applicable - effect size estimated from reported mean +/- SD
Hamidoghli et al. (2023)	Whiteleg shrimp (Litopenaeus vannamei)	Graded yeast hydrolysate supplementation in a 10% fishmeal diet	YH4 (10% fishmeal + 4% yeast hydrolysate)	CON (10% fishmeal, no yeast hydrolysate)	Crude protein (% wet weight)	18.54 +/- 0.02	18.56 +/- 0.06	3	3	Hedges' g	-0.36	-1.66 to 0.95	Lower measured value with supplementation; unadjusted 95% CI included 0	Not applicable - effect size estimated from reported mean +/- SD
Hamidoghli et al. (2023)	Whiteleg shrimp (Litopenaeus vannamei)	Graded yeast hydrolysate supplementation in a 10% fishmeal diet	YH4 (10% fishmeal + 4% yeast hydrolysate)	CON (10% fishmeal, no yeast hydrolysate)	Crude lipid (% wet weight)	1.320 +/- 0.050	1.290 +/- 0.050	3	3	Hedges' g	0.48	-0.84 to 1.80	Higher measured value with supplementation; unadjusted 95% CI included 0	Not applicable - effect size estimated from reported mean +/- SD
Hamidoghli et al. (2023)	Whiteleg shrimp (Litopenaeus vannamei)	Graded yeast hydrolysate supplementation in a 10% fishmeal diet	YH4 (10% fishmeal + 4% yeast hydrolysate)	CON (10% fishmeal, no yeast hydrolysate)	Crude ash (% wet weight)	3.450 +/- 0.030	3.430 +/- 0.020	3	3	Hedges' g	0.63	-0.72 to 1.98	Higher measured value with supplementation; unadjusted 95% CI included 0	Not applicable - effect size estimated from reported mean +/- SD
Hamidoghli et al. (2023)	Whiteleg shrimp (Litopenaeus vannamei)	Graded yeast hydrolysate supplementation in a 10% fishmeal diet	YH4 (10% fishmeal + 4% yeast hydrolysate)	CON (10% fishmeal, no yeast hydrolysate)	Aspartate aminotransferase (U/L)	11.33 +/- 3.21	10.67 +/- 2.08	3	3	Hedges' g	0.2	-1.09 to 1.48	Higher measured value with supplementation; unadjusted 95% CI included 0	Not applicable - effect size estimated from reported mean +/- SD
Hamidoghli et al. (2023)	Whiteleg shrimp (Litopenaeus vannamei)	Graded yeast hydrolysate supplementation in a 10% fishmeal diet	YH4 (10% fishmeal + 4% yeast hydrolysate)	CON (10% fishmeal, no yeast hydrolysate)	Alanine aminotransferase (U/L)	9.330 +/- 0.580	9.000 +/- 1.730	3	3	Hedges' g	0.2	-1.08 to 1.49	Higher measured value with supplementation; unadjusted 95% CI included 0	Not applicable - effect size estimated from reported mean +/- SD

Study	Species	Strategy	Intervention	Comparator	Outcome and unit	Intervention mean +/- SD	Comparator mean +/- SD	n intervention (tanks)	n comparator (tanks)	Effect-size metric	Effect size	95% CI	Interpretation	Reason not estimated
Hamidoghli et al. (2023)	Whiteleg shrimp (Litopenaeus vannamei)	Graded yeast hydrolysate supplementation in a 10% fishmeal diet	YH4 (10% fishmeal + 4% yeast hydrolysate)	CON (10% fishmeal, no yeast hydrolysate)	Glucose (mg/dL)	55.67 +/- 8.74	57.33 +/- 11.15	3	3	Hedges' g	-0.13	-1.42 to 1.15	Lower measured value with supplementation; unadjusted 95% CI included 0	Not applicable - effect size estimated from reported mean +/- SD
Hamidoghli et al. (2023)	Whiteleg shrimp (Litopenaeus vannamei)	Graded yeast hydrolysate supplementation in a 10% fishmeal diet	YH4 (10% fishmeal + 4% yeast hydrolysate)	CON (10% fishmeal, no yeast hydrolysate)	Superoxide dismutase activity (% inhibition)	45.03 +/- 0.94	43.60 +/- 7.96	3	3	Hedges' g	0.2	-1.09 to 1.49	Higher, more favorable value with supplementation; unadjusted 95% CI included 0	Not applicable - effect size estimated from reported mean +/- SD
Hamidoghli et al. (2023)	Whiteleg shrimp (Litopenaeus vannamei)	Graded yeast hydrolysate supplementation in a 10% fishmeal diet	YH4 (10% fishmeal + 4% yeast hydrolysate)	CON (10% fishmeal, no yeast hydrolysate)	Lysozyme activity (U/mL)	0.073 +/- 0.030	0.082 +/- 0.010	3	3	Hedges' g	-0.32	-1.62 to 0.98	Lower, less favorable value with supplementation; unadjusted 95% CI included 0	Not applicable - effect size estimated from reported mean +/- SD
Hamidoghli et al. (2023)	Whiteleg shrimp (Litopenaeus vannamei)	Graded yeast hydrolysate supplementation in a 10% fishmeal diet	YH4 (10% fishmeal + 4% yeast hydrolysate)	CON (10% fishmeal, no yeast hydrolysate)	Villus length (µm)	55.43 +/- 4.79	33.80 +/- 1.12	3	3	Hedges' g	4.97	1.30 to 8.65	Higher, more favorable value with supplementation; unadjusted 95% CI excluded 0	Not applicable - effect size estimated from reported mean +/- SD
Hamidoghli et al. (2023)	Whiteleg shrimp (Litopenaeus vannamei)	Graded yeast hydrolysate supplementation in a 10% fishmeal diet	YH4 (10% fishmeal + 4% yeast hydrolysate)	CON (10% fishmeal, no yeast hydrolysate)	Muscular layer thickness (µm)	59.35 +/- 2.37	47.02 +/- 1.50	3	3	Hedges' g	4.97	1.30 to 8.65	Higher, more favorable value with supplementation; unadjusted 95% CI excluded 0	Not applicable - effect size estimated from reported mean +/- SD
Hamidoghli et al. (2023)	Whiteleg shrimp (Litopenaeus vannamei)	Graded yeast hydrolysate supplementation in a 10% fishmeal diet	YH0.5 (10% fishmeal + 0.5% yeast hydrolysate)	FM25 (25% fishmeal positive-control diet)	Final body weight (g)	2.280 +/- 0.150	2.870 +/- 0.080	3	3	Hedges' g	-3.93	-6.93 to -0.92	Lower, less favorable value with supplementation; unadjusted 95% CI excluded 0	Not applicable - effect size estimated from reported mean +/- SD
Hamidoghli et al. (2023)	Whiteleg shrimp (Litopenaeus vannamei)	Graded yeast hydrolysate supplementation in a 10% fishmeal diet	YH0.5 (10% fishmeal + 0.5% yeast hydrolysate)	FM25 (25% fishmeal positive-control diet)	Weight gain (%)	426.70 +/- 30.61	554.21 +/- 18.31	3	3	Hedges' g	-4.04	-7.13 to -0.96	Lower, less favorable value with supplementation; unadjusted 95% CI excluded 0	Not applicable - effect size estimated from reported mean +/- SD
Hamidoghli et al. (2023)	Whiteleg shrimp (Litopenaeus vannamei)	Graded yeast hydrolysate supplementation in a 10% fishmeal diet	YH0.5 (10% fishmeal + 0.5% yeast hydrolysate)	FM25 (25% fishmeal positive-control diet)	Specific growth rate (%/day)	3.460 +/- 0.120	3.910 +/- 0.060	3	3	Hedges' g	-3.79	-6.72 to -0.87	Lower, less favorable value with supplementation; unadjusted 95% CI excluded 0	Not applicable - effect size estimated from reported mean +/- SD
Hamidoghli et al. (2023)	Whiteleg shrimp (Litopenaeus vannamei)	Graded yeast hydrolysate supplementation in a 10% fishmeal diet	YH0.5 (10% fishmeal + 0.5% yeast hydrolysate)	FM25 (25% fishmeal positive-control diet)	Feed efficiency (%)	34.39 +/- 0.77	46.53 +/- 0.76	3	3	Hedges' g	-12.7	-21.59 to -3.81	Lower, less favorable value with supplementation; unadjusted 95% CI excluded 0	Not applicable - effect size estimated from reported mean +/- SD
Hamidoghli et al. (2023)	Whiteleg shrimp (Litopenaeus vannamei)	Graded yeast hydrolysate supplementation in a 10% fishmeal diet	YH0.5 (10% fishmeal + 0.5% yeast hydrolysate)	FM25 (25% fishmeal positive-control diet)	Protein efficiency ratio	0.940 +/- 0.030	1.190 +/- 0.020	3	3	Hedges' g	-7.84	-13.43 to -2.26	Lower, less favorable value with supplementation; unadjusted 95% CI excluded 0	Not applicable - effect size estimated from reported mean +/- SD
Hamidoghli et al. (2023)	Whiteleg shrimp (Litopenaeus vannamei)	Graded yeast hydrolysate supplementation in a 10% fishmeal diet	YH0.5 (10% fishmeal + 0.5% yeast hydrolysate)	FM25 (25% fishmeal positive-control diet)	Survival rate (%)	81.67 +/- 4.71	95.00 +/- 0.00	3	3	Hedges' g	-3.2	-5.76 to -0.64	Lower, less favorable value with supplementation; unadjusted 95% CI excluded 0	Not applicable - effect size estimated from reported mean +/- SD
Hamidoghli et al. (2023)	Whiteleg shrimp (Litopenaeus vannamei)	Graded yeast hydrolysate supplementation in a 10% fishmeal diet	YH0.5 (10% fishmeal + 0.5% yeast hydrolysate)	FM25 (25% fishmeal positive-control diet)	Moisture (% wet weight)	76.07 +/- 0.44	76.06 +/- 0.51	3	3	Hedges' g	0.02	-1.26 to 1.30	Higher measured value with supplementation; unadjusted 95% CI included 0	Not applicable - effect size estimated from reported mean +/- SD
Hamidoghli et al. (2023)	Whiteleg shrimp (Litopenaeus vannamei)	Graded yeast hydrolysate supplementation in a 10% fishmeal diet	YH0.5 (10% fishmeal + 0.5% yeast hydrolysate)	FM25 (25% fishmeal positive-control diet)	Crude protein (% wet weight)	18.58 +/- 0.03	18.55 +/- 0.02	3	3	Hedges' g	0.94	-0.50 to 2.38	Higher measured value with supplementation; unadjusted 95% CI included 0	Not applicable - effect size estimated from reported mean +/- SD
Hamidoghli et al. (2023)	Whiteleg shrimp (Litopenaeus vannamei)	Graded yeast hydrolysate supplementation in a 10% fishmeal diet	YH0.5 (10% fishmeal + 0.5% yeast hydrolysate)	FM25 (25% fishmeal positive-control diet)	Crude lipid (% wet weight)	1.310 +/- 0.040	1.300 +/- 0.030	3	3	Hedges' g	0.23	-1.06 to 1.52	Higher measured value with supplementation; unadjusted 95% CI included 0	Not applicable - effect size estimated from reported mean +/- SD
Hamidoghli et al. (2023)	Whiteleg shrimp (Litopenaeus vannamei)	Graded yeast hydrolysate supplementation in a 10% fishmeal diet	YH0.5 (10% fishmeal + 0.5% yeast hydrolysate)	FM25 (25% fishmeal positive-control diet)	Crude ash (% wet weight)	3.440 +/- 0.040	3.390 +/- 0.020	3	3	Hedges' g	1.26	-0.29 to 2.82	Higher measured value with supplementation; unadjusted 95% CI included 0	Not applicable - effect size estimated from reported mean +/- SD
Hamidoghli et al. (2023)	Whiteleg shrimp (Litopenaeus vannamei)	Graded yeast hydrolysate supplementation in a 10% fishmeal diet	YH0.5 (10% fishmeal + 0.5% yeast hydrolysate)	FM25 (25% fishmeal positive-control diet)	Aspartate aminotransferase (U/L)	12.67 +/- 3.79	9.330 +/- 1.150	3	3	Hedges' g	0.95	-0.49 to 2.39	Higher measured value with supplementation; unadjusted 95% CI included 0	Not applicable - effect size estimated from reported mean +/- SD
Hamidoghli et al. (2023)	Whiteleg shrimp (Litopenaeus vannamei)	Graded yeast hydrolysate supplementation in a 10% fishmeal diet	YH0.5 (10% fishmeal + 0.5% yeast hydrolysate)	FM25 (25% fishmeal positive-control diet)	Alanine aminotransferase (U/L)	9.670 +/- 1.530	9.000 +/- 1.000	3	3	Hedges' g	0.41	-0.90 to 1.73	Higher measured value with supplementation; unadjusted 95% CI included 0	Not applicable - effect size estimated from reported mean +/- SD
Hamidoghli et al. (2023)	Whiteleg shrimp (Litopenaeus vannamei)	Graded yeast hydrolysate supplementation in a 10% fishmeal diet	YH0.5 (10% fishmeal + 0.5% yeast hydrolysate)	FM25 (25% fishmeal positive-control diet)	Glucose (mg/dL)	61.33 +/- 15.01	53.33 +/- 7.09	3	3	Hedges' g	0.55	-0.79 to 1.88	Higher measured value with supplementation; unadjusted 95% CI included 0	Not applicable - effect size estimated from reported mean +/- SD
Hamidoghli et al. (2023)	Whiteleg shrimp (Litopenaeus vannamei)	Graded yeast hydrolysate supplementation in a 10% fishmeal diet	YH0.5 (10% fishmeal + 0.5% yeast hydrolysate)	FM25 (25% fishmeal positive-control diet)	Superoxide dismutase activity (% inhibition)	34.37 +/- 5.88	43.38 +/- 4.34	3	3	Hedges' g	-1.39	-3.00 to 0.21	Lower, less favorable value with supplementation; unadjusted 95% CI included 0	Not applicable - effect size estimated from reported mean +/- SD
Hamidoghli et al. (2023)	Whiteleg shrimp (Litopenaeus vannamei)	Graded yeast hydrolysate supplementation in a 10% fishmeal diet	YH0.5 (10% fishmeal + 0.5% yeast hydrolysate)	FM25 (25% fishmeal positive-control diet)	Lysozyme activity (U/mL)	0.076 +/- 0.010	0.077 +/- 0.030	3	3	Hedges' g	-0.04	-1.32 to 1.24	Lower, less favorable value with supplementation; unadjusted 95% CI included 0	Not applicable - effect size estimated from reported mean +/- SD
Hamidoghli et al. (2023)	Whiteleg shrimp (Litopenaeus vannamei)	Graded yeast hydrolysate supplementation in a 10% fishmeal diet	YH0.5 (10% fishmeal + 0.5% yeast hydrolysate)	FM25 (25% fishmeal positive-control diet)	Villus length (µm)	29.80 +/- 2.10	54.08 +/- 1.34	3	3	Hedges' g	-11.03	-18.78 to -3.28	Lower, less favorable value with supplementation; unadjusted 95% CI excluded 0	Not applicable - effect size estimated from reported mean +/- SD
Hamidoghli et al. (2023)	Whiteleg shrimp (Litopenaeus vannamei)	Graded yeast hydrolysate supplementation in a 10% fishmeal diet	YH0.5 (10% fishmeal + 0.5% yeast hydrolysate)	FM25 (25% fishmeal positive-control diet)	Muscular layer thickness (µm)	49.96 +/- 2.26	65.03 +/- 3.73	3	3	Hedges' g	-3.91	-6.91 to -0.91	Lower, less favorable value with supplementation; unadjusted 95% CI excluded 0	Not applicable - effect size estimated from reported mean +/- SD
Hamidoghli et al. (2023)	Whiteleg shrimp (Litopenaeus vannamei)	Graded yeast hydrolysate supplementation in a 10% fishmeal diet	YH1 (10% fishmeal + 1% yeast hydrolysate)	FM25 (25% fishmeal positive-control diet)	Final body weight (g)	2.450 +/- 0.270	2.870 +/- 0.080	3	3	Hedges' g	-1.69	-3.42 to 0.05	Lower, less favorable value with supplementation; unadjusted 95% CI included 0	Not applicable - effect size estimated from reported mean +/- SD
Hamidoghli et al. (2023)	Whiteleg shrimp (Litopenaeus vannamei)	Graded yeast hydrolysate supplementation in a 10% fishmeal diet	YH1 (10% fishmeal + 1% yeast hydrolysate)	FM25 (25% fishmeal positive-control diet)	Weight gain (%)	464.70 +/- 59.43	554.21 +/- 18.31	3	3	Hedges' g	-1.63	-3.34 to 0.08	Lower, less favorable value with supplementation; unadjusted 95% CI included 0	Not applicable - effect size estimated from reported mean +/- SD
Hamidoghli et al. (2023)	Whiteleg shrimp (Litopenaeus vannamei)	Graded yeast hydrolysate supplementation in a 10% fishmeal diet	YH1 (10% fishmeal + 1% yeast hydrolysate)	FM25 (25% fishmeal positive-control diet)	Specific growth rate (%/day)	3.590 +/- 0.230	3.910 +/- 0.060	3	3	Hedges' g	-1.52	-3.18 to 0.14	Lower, less favorable value with supplementation; unadjusted 95% CI included 0	Not applicable - effect size estimated from reported mean +/- SD
Hamidoghli et al. (2023)	Whiteleg shrimp (Litopenaeus vannamei)	Graded yeast hydrolysate supplementation in a 10% fishmeal diet	YH1 (10% fishmeal + 1% yeast hydrolysate)	FM25 (25% fishmeal positive-control diet)	Feed efficiency (%)	36.57 +/- 2.97	46.53 +/- 0.76	3	3	Hedges' g	-3.68	-6.53 to -0.82	Lower, less favorable value with supplementation; unadjusted 95% CI excluded 0	Not applicable - effect size estimated from reported mean +/- SD
Hamidoghli et al. (2023)	Whiteleg shrimp (Litopenaeus vannamei)	Graded yeast hydrolysate supplementation in a 10% fishmeal diet	YH1 (10% fishmeal + 1% yeast hydrolysate)	FM25 (25% fishmeal positive-control diet)	Protein efficiency ratio	0.980 +/- 0.080	1.190 +/- 0.020	3	3	Hedges' g	-2.88	-5.25 to -0.51	Lower, less favorable value with supplementation; unadjusted 95% CI excluded 0	Not applicable - effect size estimated from reported mean +/- SD
Hamidoghli et al. (2023)	Whiteleg shrimp (Litopenaeus vannamei)	Graded yeast hydrolysate supplementation in a 10% fishmeal diet	YH1 (10% fishmeal + 1% yeast hydrolysate)	FM25 (25% fishmeal positive-control diet)	Survival rate (%)	80.00 +/- 7.07	95.00 +/- 0.00	3	3	Hedges' g	-2.4	-4.50 to -0.30	Lower, less favorable value with supplementation; unadjusted 95% CI excluded 0	Not applicable - effect size estimated from reported mean +/- SD
Hamidoghli et al. (2023)	Whiteleg shrimp (Litopenaeus vannamei)	Graded yeast hydrolysate supplementation in a 10% fishmeal diet	YH1 (10% fishmeal + 1% yeast hydrolysate)	FM25 (25% fishmeal positive-control diet)	Moisture (% wet weight)	75.82 +/- 0.17	76.06 +/- 0.51	3	3	Hedges' g	-0.51	-1.83 to 0.82	Lower measured value with supplementation; unadjusted 95% CI included 0	Not applicable - effect size estimated from reported mean +/- SD
Hamidoghli et al. (2023)	Whiteleg shrimp (Litopenaeus vannamei)	Graded yeast hydrolysate supplementation in a 10% fishmeal diet	YH1 (10% fishmeal + 1% yeast hydrolysate)	FM25 (25% fishmeal positive-control diet)	Crude protein (% wet weight)	18.53 +/- 0.06	18.55 +/- 0.02	3	3	Hedges' g	-0.36	-1.66 to 0.95	Lower measured value with supplementation; unadjusted 95% CI included 0	Not applicable - effect size estimated from reported mean +/- SD
Hamidoghli et al. (2023)	Whiteleg shrimp (Litopenaeus vannamei)	Graded yeast hydrolysate supplementation in a 10% fishmeal diet	YH1 (10% fishmeal + 1% yeast hydrolysate)	FM25 (25% fishmeal positive-control diet)	Crude lipid (% wet weight)	1.350 +/- 0.050	1.300 +/- 0.030	3	3	Hedges' g	0.97	-0.48 to 2.42	Higher measured value with supplementation; unadjusted 95% CI included 0	Not applicable - effect size estimated from reported mean +/- SD
Hamidoghli et al. (2023)	Whiteleg shrimp (Litopenaeus vannamei)	Graded yeast hydrolysate supplementation in a 10% fishmeal diet	YH1 (10% fishmeal + 1% yeast hydrolysate)	FM25 (25% fishmeal positive-control diet)	Crude ash (% wet weight)	3.420 +/- 0.020	3.390 +/- 0.020	3	3	Hedges' g	1.2	-0.33 to 2.73	Higher measured value with supplementation; unadjusted 95% CI included 0	Not applicable - effect size estimated from reported mean +/- SD

Study	Species	Strategy	Intervention	Comparator	Outcome and unit	Intervention mean +/- SD	Comparator mean +/- SD	n intervention (tanks)	n comparator (tanks)	Effect-size metric	Effect size	95% CI	Interpretation	Reason not estimated
Hamidoghli et al. (2023)	Whiteleg shrimp (Litopenaeus vannamei)	Graded yeast hydrolysate supplementation in a 10% fishmeal diet	YH1 (10% fishmeal + 1% yeast hydrolysate)	FM25 (25% fishmeal positive-control diet)	Aspartate aminotransferase (U/L)	13.67 +/- 2.89	9.330 +/- 1.150	3	3	Hedges' g	1.58	-0.11 to 3.26	Higher measured value with supplementation; unadjusted 95% CI included 0	Not applicable - effect size estimated from reported mean +/- SD
Hamidoghli et al. (2023)	Whiteleg shrimp (Litopenaeus vannamei)	Graded yeast hydrolysate supplementation in a 10% fishmeal diet	YH1 (10% fishmeal + 1% yeast hydrolysate)	FM25 (25% fishmeal positive-control diet)	Alanine aminotransferase (U/L)	9.670 +/- 1.530	9.000 +/- 1.000	3	3	Hedges' g	0.41	-0.90 to 1.73	Higher measured value with supplementation; unadjusted 95% CI included 0	Not applicable - effect size estimated from reported mean +/- SD
Hamidoghli et al. (2023)	Whiteleg shrimp (Litopenaeus vannamei)	Graded yeast hydrolysate supplementation in a 10% fishmeal diet	YH1 (10% fishmeal + 1% yeast hydrolysate)	FM25 (25% fishmeal positive-control diet)	Glucose (mg/dL)	57.67 +/- 8.14	53.33 +/- 7.09	3	3	Hedges' g	0.45	-0.86 to 1.77	Higher measured value with supplementation; unadjusted 95% CI included 0	Not applicable - effect size estimated from reported mean +/- SD
Hamidoghli et al. (2023)	Whiteleg shrimp (Litopenaeus vannamei)	Graded yeast hydrolysate supplementation in a 10% fishmeal diet	YH1 (10% fishmeal + 1% yeast hydrolysate)	FM25 (25% fishmeal positive-control diet)	Superoxide dismutase activity (% inhibition)	39.66 +/- 4.64	43.38 +/- 4.34	3	3	Hedges' g	-0.66	-2.02 to 0.70	Lower, less favorable value with supplementation; unadjusted 95% CI included 0	Not applicable - effect size estimated from reported mean +/- SD
Hamidoghli et al. (2023)	Whiteleg shrimp (Litopenaeus vannamei)	Graded yeast hydrolysate supplementation in a 10% fishmeal diet	YH1 (10% fishmeal + 1% yeast hydrolysate)	FM25 (25% fishmeal positive-control diet)	Lysozyme activity (U/mL)	0.073 +/- 0.030	0.077 +/- 0.030	3	3	Hedges' g	-0.11	-1.39 to 1.18	Lower, less favorable value with supplementation; unadjusted 95% CI included 0	Not applicable - effect size estimated from reported mean +/- SD
Hamidoghli et al. (2023)	Whiteleg shrimp (Litopenaeus vannamei)	Graded yeast hydrolysate supplementation in a 10% fishmeal diet	YH1 (10% fishmeal + 1% yeast hydrolysate)	FM25 (25% fishmeal positive-control diet)	Villus length (μm)	36.48 +/- 2.33	54.08 +/- 1.34	3	3	Hedges' g	-7.41	-12.70 to -2.12	Lower, less favorable value with supplementation; unadjusted 95% CI excluded 0	Not applicable - effect size estimated from reported mean +/- SD
Hamidoghli et al. (2023)	Whiteleg shrimp (Litopenaeus vannamei)	Graded yeast hydrolysate supplementation in a 10% fishmeal diet	YH1 (10% fishmeal + 1% yeast hydrolysate)	FM25 (25% fishmeal positive-control diet)	Muscular layer thickness (μm)	50.35 +/- 1.33	65.03 +/- 3.73	3	3	Hedges' g	-4.19	-7.37 to -1.02	Lower, less favorable value with supplementation; unadjusted 95% CI excluded 0	Not applicable - effect size estimated from reported mean +/- SD
Hamidoghli et al. (2023)	Whiteleg shrimp (Litopenaeus vannamei)	Graded yeast hydrolysate supplementation in a 10% fishmeal diet	YH2 (10% fishmeal + 2% yeast hydrolysate)	FM25 (25% fishmeal positive-control diet)	Final body weight (g)	2.330 +/- 0.130	2.870 +/- 0.080	3	3	Hedges' g	-4.0	-7.06 to -0.95	Lower, less favorable value with supplementation; unadjusted 95% CI excluded 0	Not applicable - effect size estimated from reported mean +/- SD
Hamidoghli et al. (2023)	Whiteleg shrimp (Litopenaeus vannamei)	Graded yeast hydrolysate supplementation in a 10% fishmeal diet	YH2 (10% fishmeal + 2% yeast hydrolysate)	FM25 (25% fishmeal positive-control diet)	Weight gain (%)	439.17 +/- 23.86	554.21 +/- 18.31	3	3	Hedges' g	-4.33	-7.59 to -1.07	Lower, less favorable value with supplementation; unadjusted 95% CI excluded 0	Not applicable - effect size estimated from reported mean +/- SD
Hamidoghli et al. (2023)	Whiteleg shrimp (Litopenaeus vannamei)	Graded yeast hydrolysate supplementation in a 10% fishmeal diet	YH2 (10% fishmeal + 2% yeast hydrolysate)	FM25 (25% fishmeal positive-control diet)	Specific growth rate (%/day)	3.510 +/- 0.090	3.910 +/- 0.060	3	3	Hedges' g	-4.18	-7.35 to -1.01	Lower, less favorable value with supplementation; unadjusted 95% CI excluded 0	Not applicable - effect size estimated from reported mean +/- SD
Hamidoghli et al. (2023)	Whiteleg shrimp (Litopenaeus vannamei)	Graded yeast hydrolysate supplementation in a 10% fishmeal diet	YH2 (10% fishmeal + 2% yeast hydrolysate)	FM25 (25% fishmeal positive-control diet)	Feed efficiency (%)	35.17 +/- 1.65	46.53 +/- 0.76	3	3	Hedges' g	-7.07	-12.14 to -2.01	Lower, less favorable value with supplementation; unadjusted 95% CI excluded 0	Not applicable - effect size estimated from reported mean +/- SD
Hamidoghli et al. (2023)	Whiteleg shrimp (Litopenaeus vannamei)	Graded yeast hydrolysate supplementation in a 10% fishmeal diet	YH2 (10% fishmeal + 2% yeast hydrolysate)	FM25 (25% fishmeal positive-control diet)	Protein efficiency ratio	0.970 +/- 0.030	1.190 +/- 0.020	3	3	Hedges' g	-6.9	-11.86 to -1.95	Lower, less favorable value with supplementation; unadjusted 95% CI excluded 0	Not applicable - effect size estimated from reported mean +/- SD
Hamidoghli et al. (2023)	Whiteleg shrimp (Litopenaeus vannamei)	Graded yeast hydrolysate supplementation in a 10% fishmeal diet	YH2 (10% fishmeal + 2% yeast hydrolysate)	FM25 (25% fishmeal positive-control diet)	Survival rate (%)	78.33 +/- 4.71	95.00 +/- 0.00	3	3	Hedges' g	-4.0	-7.06 to -0.95	Lower, less favorable value with supplementation; unadjusted 95% CI excluded 0	Not applicable - effect size estimated from reported mean +/- SD
Hamidoghli et al. (2023)	Whiteleg shrimp (Litopenaeus vannamei)	Graded yeast hydrolysate supplementation in a 10% fishmeal diet	YH2 (10% fishmeal + 2% yeast hydrolysate)	FM25 (25% fishmeal positive-control diet)	Moisture (% wet weight)	75.78 +/- 0.41	76.06 +/- 0.51	3	3	Hedges' g	-0.48	-1.81 to 0.84	Lower measured value with supplementation; unadjusted 95% CI included 0	Not applicable - effect size estimated from reported mean +/- SD
Hamidoghli et al. (2023)	Whiteleg shrimp (Litopenaeus vannamei)	Graded yeast hydrolysate supplementation in a 10% fishmeal diet	YH2 (10% fishmeal + 2% yeast hydrolysate)	FM25 (25% fishmeal positive-control diet)	Crude protein (% wet weight)	18.53 +/- 0.01	18.55 +/- 0.02	3	3	Hedges' g	-1.01	-2.47 to 0.45	Lower measured value with supplementation; unadjusted 95% CI included 0	Not applicable - effect size estimated from reported mean +/- SD
Hamidoghli et al. (2023)	Whiteleg shrimp (Litopenaeus vannamei)	Graded yeast hydrolysate supplementation in a 10% fishmeal diet	YH2 (10% fishmeal + 2% yeast hydrolysate)	FM25 (25% fishmeal positive-control diet)	Crude lipid (% wet weight)	1.300 +/- 0.060	1.300 +/- 0.030	3	3	Hedges' g	0.0	-1.28 to 1.28	No observed mean difference; unadjusted 95% CI included 0	Not applicable - effect size estimated from reported mean +/- SD
Hamidoghli et al. (2023)	Whiteleg shrimp (Litopenaeus vannamei)	Graded yeast hydrolysate supplementation in a 10% fishmeal diet	YH2 (10% fishmeal + 2% yeast hydrolysate)	FM25 (25% fishmeal positive-control diet)	Crude ash (% wet weight)	3.400 +/- 0.010	3.390 +/- 0.020	3	3	Hedges' g	0.51	-0.82 to 1.83	Higher measured value with supplementation; unadjusted 95% CI included 0	Not applicable - effect size estimated from reported mean +/- SD
Hamidoghli et al. (2023)	Whiteleg shrimp (Litopenaeus vannamei)	Graded yeast hydrolysate supplementation in a 10% fishmeal diet	YH2 (10% fishmeal + 2% yeast hydrolysate)	FM25 (25% fishmeal positive-control diet)	Aspartate aminotransferase (U/L)	11.67 +/- 2.52	9.330 +/- 1.150	3	3	Hedges' g	0.96	-0.49 to 2.40	Higher measured value with supplementation; unadjusted 95% CI included 0	Not applicable - effect size estimated from reported mean +/- SD
Hamidoghli et al. (2023)	Whiteleg shrimp (Litopenaeus vannamei)	Graded yeast hydrolysate supplementation in a 10% fishmeal diet	YH2 (10% fishmeal + 2% yeast hydrolysate)	FM25 (25% fishmeal positive-control diet)	Alanine aminotransferase (U/L)	10.33 +/- 2.08	9.000 +/- 1.000	3	3	Hedges' g	0.65	-0.71 to 2.01	Higher measured value with supplementation; unadjusted 95% CI included 0	Not applicable - effect size estimated from reported mean +/- SD
Hamidoghli et al. (2023)	Whiteleg shrimp (Litopenaeus vannamei)	Graded yeast hydrolysate supplementation in a 10% fishmeal diet	YH2 (10% fishmeal + 2% yeast hydrolysate)	FM25 (25% fishmeal positive-control diet)	Glucose (mg/dL)	58.67 +/- 9.02	53.33 +/- 7.09	3	3	Hedges' g	0.53	-0.80 to 1.86	Higher measured value with supplementation; unadjusted 95% CI included 0	Not applicable - effect size estimated from reported mean +/- SD
Hamidoghli et al. (2023)	Whiteleg shrimp (Litopenaeus vannamei)	Graded yeast hydrolysate supplementation in a 10% fishmeal diet	YH2 (10% fishmeal + 2% yeast hydrolysate)	FM25 (25% fishmeal positive-control diet)	Superoxide dismutase activity (% inhibition)	46.85 +/- 4.67	43.38 +/- 4.34	3	3	Hedges' g	0.62	-0.73 to 1.97	Higher, more favorable value with supplementation; unadjusted 95% CI included 0	Not applicable - effect size estimated from reported mean +/- SD
Hamidoghli et al. (2023)	Whiteleg shrimp (Litopenaeus vannamei)	Graded yeast hydrolysate supplementation in a 10% fishmeal diet	YH2 (10% fishmeal + 2% yeast hydrolysate)	FM25 (25% fishmeal positive-control diet)	Lysozyme activity (U/mL)	0.080 +/- 0.030	0.077 +/- 0.030	3	3	Hedges' g	0.08	-1.20 to 1.36	Higher, more favorable value with supplementation; unadjusted 95% CI included 0	Not applicable - effect size estimated from reported mean +/- SD
Hamidoghli et al. (2023)	Whiteleg shrimp (Litopenaeus vannamei)	Graded yeast hydrolysate supplementation in a 10% fishmeal diet	YH2 (10% fishmeal + 2% yeast hydrolysate)	FM25 (25% fishmeal positive-control diet)	Villus length (μm)	30.05 +/- 1.16	54.08 +/- 1.34	3	3	Hedges' g	-15.34	-26.05 to -4.63	Lower, less favorable value with supplementation; unadjusted 95% CI excluded 0	Not applicable - effect size estimated from reported mean +/- SD
Hamidoghli et al. (2023)	Whiteleg shrimp (Litopenaeus vannamei)	Graded yeast hydrolysate supplementation in a 10% fishmeal diet	YH2 (10% fishmeal + 2% yeast hydrolysate)	FM25 (25% fishmeal positive-control diet)	Muscular layer thickness (μm)	50.62 +/- 1.24	65.03 +/- 3.73	3	3	Hedges' g	-4.15	-7.29 to -1.00	Lower, less favorable value with supplementation; unadjusted 95% CI excluded 0	Not applicable - effect size estimated from reported mean +/- SD
Hamidoghli et al. (2023)	Whiteleg shrimp (Litopenaeus vannamei)	Graded yeast hydrolysate supplementation in a 10% fishmeal diet	YH4 (10% fishmeal + 4% yeast hydrolysate)	FM25 (25% fishmeal positive-control diet)	Final body weight (g)	2.580 +/- 0.050	2.870 +/- 0.080	3	3	Hedges' g	-3.48	-6.21 to -0.75	Lower, less favorable value with supplementation; unadjusted 95% CI excluded 0	Not applicable - effect size estimated from reported mean +/- SD
Hamidoghli et al. (2023)	Whiteleg shrimp (Litopenaeus vannamei)	Graded yeast hydrolysate supplementation in a 10% fishmeal diet	YH4 (10% fishmeal + 4% yeast hydrolysate)	FM25 (25% fishmeal positive-control diet)	Weight gain (%)	492.08 +/- 4.89	554.21 +/- 18.31	3	3	Hedges' g	-3.71	-6.58 to -0.84	Lower, less favorable value with supplementation; unadjusted 95% CI excluded 0	Not applicable - effect size estimated from reported mean +/- SD
Hamidoghli et al. (2023)	Whiteleg shrimp (Litopenaeus vannamei)	Graded yeast hydrolysate supplementation in a 10% fishmeal diet	YH4 (10% fishmeal + 4% yeast hydrolysate)	FM25 (25% fishmeal positive-control diet)	Specific growth rate (%/day)	3.710 +/- 0.020	3.910 +/- 0.060	3	3	Hedges' g	-3.58	-6.37 to -0.79	Lower, less favorable value with supplementation; unadjusted 95% CI excluded 0	Not applicable - effect size estimated from reported mean +/- SD
Hamidoghli et al. (2023)	Whiteleg shrimp (Litopenaeus vannamei)	Graded yeast hydrolysate supplementation in a 10% fishmeal diet	YH4 (10% fishmeal + 4% yeast hydrolysate)	FM25 (25% fishmeal positive-control diet)	Feed efficiency (%)	38.78 +/- 4.58	46.53 +/- 0.76	3	3	Hedges' g	-1.89	-3.72 to -0.06	Lower, less favorable value with supplementation; unadjusted 95% CI excluded 0	Not applicable - effect size estimated from reported mean +/- SD
Hamidoghli et al. (2023)	Whiteleg shrimp (Litopenaeus vannamei)	Graded yeast hydrolysate supplementation in a 10% fishmeal diet	YH4 (10% fishmeal + 4% yeast hydrolysate)	FM25 (25% fishmeal positive-control diet)	Protein efficiency ratio	1.050 +/- 0.090	1.190 +/- 0.020	3	3	Hedges' g	-1.72	-3.47 to 0.03	Lower, less favorable value with supplementation; unadjusted 95% CI included 0	Not applicable - effect size estimated from reported mean +/- SD
Hamidoghli et al. (2023)	Whiteleg shrimp (Litopenaeus vannamei)	Graded yeast hydrolysate supplementation in a 10% fishmeal diet	YH4 (10% fishmeal + 4% yeast hydrolysate)	FM25 (25% fishmeal positive-control diet)	Survival rate (%)	83.33 +/- 10.27	95.00 +/- 0.00	3	3	Hedges' g	-1.29	-2.85 to 0.27	Lower, less favorable value with supplementation; unadjusted 95% CI included 0	Not applicable - effect size estimated from reported mean +/- SD
Hamidoghli et al. (2023)	Whiteleg shrimp (Litopenaeus vannamei)	Graded yeast hydrolysate supplementation in a 10% fishmeal diet	YH4 (10% fishmeal + 4% yeast hydrolysate)	FM25 (25% fishmeal positive-control diet)	Moisture (% wet weight)	76.01 +/- 0.58	76.06 +/- 0.51	3	3	Hedges' g	-0.07	-1.35 to 1.21	Lower measured value with supplementation; unadjusted 95% CI included 0	Not applicable - effect size estimated from reported mean +/- SD
Hamidoghli et al. (2023)	Whiteleg shrimp (Litopenaeus vannamei)	Graded yeast hydrolysate supplementation in a 10% fishmeal diet	YH4 (10% fishmeal + 4% yeast hydrolysate)	FM25 (25% fishmeal positive-control diet)	Crude protein (% wet weight)	18.54 +/- 0.02	18.55 +/- 0.02	3	3	Hedges' g	-0.4	-1.71 to 0.91	Lower measured value with supplementation; unadjusted 95% CI included 0	Not applicable - effect size estimated from reported mean +/- SD

Study	Species	Strategy	Intervention	Comparator	Outcome and unit	Intervention mean +/- SD	Comparator mean +/- SD	n intervention (tanks)	n comparator (tanks)	Effect-size metric	Effect size	95% CI	Interpretation	Reason not estimated
Hamidoghli et al. (2023)	Whiteleg shrimp (Litopenaeus vannamei)	Graded yeast hydrolysate supplementation in a 10% fishmeal diet	YH4 (10% fishmeal + 4% yeast hydrolysate)	FM25 (25% fishmeal positive-control diet)	Crude lipid (% wet weight)	1.320 +/- 0.050	1.300 +/- 0.030	3	3	Hedges' g	0.39	-0.92 to 1.70	Higher measured value with supplementation; unadjusted 95% CI included 0	Not applicable - effect size estimated from reported mean +/- SD
Hamidoghli et al. (2023)	Whiteleg shrimp (Litopenaeus vannamei)	Graded yeast hydrolysate supplementation in a 10% fishmeal diet	YH4 (10% fishmeal + 4% yeast hydrolysate)	FM25 (25% fishmeal positive-control diet)	Crude ash (% wet weight)	3.450 +/- 0.030	3.390 +/- 0.020	3	3	Hedges' g	1.88	0.05 to 3.71	Higher measured value with supplementation; unadjusted 95% CI excluded 0	Not applicable - effect size estimated from reported mean +/- SD
Hamidoghli et al. (2023)	Whiteleg shrimp (Litopenaeus vannamei)	Graded yeast hydrolysate supplementation in a 10% fishmeal diet	YH4 (10% fishmeal + 4% yeast hydrolysate)	FM25 (25% fishmeal positive-control diet)	Aspartate aminotransferase (U/L)	11.33 +/- 3.21	9.330 +/- 1.150	3	3	Hedges' g	0.66	-0.70 to 2.02	Higher measured value with supplementation; unadjusted 95% CI included 0	Not applicable - effect size estimated from reported mean +/- SD
Hamidoghli et al. (2023)	Whiteleg shrimp (Litopenaeus vannamei)	Graded yeast hydrolysate supplementation in a 10% fishmeal diet	YH4 (10% fishmeal + 4% yeast hydrolysate)	FM25 (25% fishmeal positive-control diet)	Alanine aminotransferase (U/L)	9.330 +/- 0.580	9.000 +/- 1.000	3	3	Hedges' g	0.32	-0.98 to 1.62	Higher measured value with supplementation; unadjusted 95% CI included 0	Not applicable - effect size estimated from reported mean +/- SD
Hamidoghli et al. (2023)	Whiteleg shrimp (Litopenaeus vannamei)	Graded yeast hydrolysate supplementation in a 10% fishmeal diet	YH4 (10% fishmeal + 4% yeast hydrolysate)	FM25 (25% fishmeal positive-control diet)	Glucose (mg/dL)	55.67 +/- 8.74	53.33 +/- 7.09	3	3	Hedges' g	0.24	-1.06 to 1.53	Higher measured value with supplementation; unadjusted 95% CI included 0	Not applicable - effect size estimated from reported mean +/- SD
Hamidoghli et al. (2023)	Whiteleg shrimp (Litopenaeus vannamei)	Graded yeast hydrolysate supplementation in a 10% fishmeal diet	YH4 (10% fishmeal + 4% yeast hydrolysate)	FM25 (25% fishmeal positive-control diet)	Superoxide dismutase activity (% inhibition)	45.03 +/- 0.94	43.38 +/- 4.34	3	3	Hedges' g	0.42	-0.89 to 1.73	Higher, more favorable value with supplementation; unadjusted 95% CI included 0	Not applicable - effect size estimated from reported mean +/- SD
Hamidoghli et al. (2023)	Whiteleg shrimp (Litopenaeus vannamei)	Graded yeast hydrolysate supplementation in a 10% fishmeal diet	YH4 (10% fishmeal + 4% yeast hydrolysate)	FM25 (25% fishmeal positive-control diet)	Lysozyme activity (U/mL)	0.073 +/- 0.030	0.077 +/- 0.030	3	3	Hedges' g	-0.11	-1.39 to 1.18	Lower, less favorable value with supplementation; unadjusted 95% CI included 0	Not applicable - effect size estimated from reported mean +/- SD
Hamidoghli et al. (2023)	Whiteleg shrimp (Litopenaeus vannamei)	Graded yeast hydrolysate supplementation in a 10% fishmeal diet	YH4 (10% fishmeal + 4% yeast hydrolysate)	FM25 (25% fishmeal positive-control diet)	Villus length (µm)	55.43 +/- 4.79	54.08 +/- 1.34	3	3	Hedges' g	0.31	-0.99 to 1.60	Higher, more favorable value with supplementation; unadjusted 95% CI included 0	Not applicable - effect size estimated from reported mean +/- SD
Hamidoghli et al. (2023)	Whiteleg shrimp (Litopenaeus vannamei)	Graded yeast hydrolysate supplementation in a 10% fishmeal diet	YH4 (10% fishmeal + 4% yeast hydrolysate)	FM25 (25% fishmeal positive-control diet)	Muscular layer thickness (µm)	59.35 +/- 2.37	65.03 +/- 3.73	3	3	Hedges' g	-1.45	-3.08 to 0.18	Lower, less favorable value with supplementation; unadjusted 95% CI included 0	Not applicable - effect size estimated from reported mean +/- SD
Hamidoghli et al. (2023)	Whiteleg shrimp (Litopenaeus vannamei)	Graded yeast hydrolysate supplementation in a 10% fishmeal diet	YH0.5 (10% fishmeal + 0.5% yeast hydrolysate)	CON (10% fishmeal, no yeast hydrolysate)	Cumulative survival after Vibrio parahaemolyticus challenge (%)	Not reported numerically	Not reported numerically	3	3	Not estimable	Not estimable	Not estimable	Figure 2 shows time-specific survival and significance lettering, but an exact standardized effect was not calculated	Exact group means and SDs at the selected challenge endpoint were available only graphically
Hamidoghli et al. (2023)	Whiteleg shrimp (Litopenaeus vannamei)	Graded yeast hydrolysate supplementation in a 10% fishmeal diet	YH1 (10% fishmeal + 1% yeast hydrolysate)	CON (10% fishmeal, no yeast hydrolysate)	Cumulative survival after Vibrio parahaemolyticus challenge (%)	Not reported numerically	Not reported numerically	3	3	Not estimable	Not estimable	Not estimable	Figure 2 shows time-specific survival and significance lettering, but an exact standardized effect was not calculated	Exact group means and SDs at the selected challenge endpoint were available only graphically
Hamidoghli et al. (2023)	Whiteleg shrimp (Litopenaeus vannamei)	Graded yeast hydrolysate supplementation in a 10% fishmeal diet	YH2 (10% fishmeal + 2% yeast hydrolysate)	CON (10% fishmeal, no yeast hydrolysate)	Cumulative survival after Vibrio parahaemolyticus challenge (%)	Not reported numerically	Not reported numerically	3	3	Not estimable	Not estimable	Not estimable	Figure 2 shows time-specific survival and significance lettering, but an exact standardized effect was not calculated	Exact group means and SDs at the selected challenge endpoint were available only graphically
Hamidoghli et al. (2023)	Whiteleg shrimp (Litopenaeus vannamei)	Graded yeast hydrolysate supplementation in a 10% fishmeal diet	YH4 (10% fishmeal + 4% yeast hydrolysate)	CON (10% fishmeal, no yeast hydrolysate)	Cumulative survival after Vibrio parahaemolyticus challenge (%)	Not reported numerically	Not reported numerically	3	3	Not estimable	Not estimable	Not estimable	Figure 2 shows time-specific survival and significance lettering, but an exact standardized effect was not calculated	Exact group means and SDs at the selected challenge endpoint were available only graphically
Hamidoghli et al. (2023)	Whiteleg shrimp (Litopenaeus vannamei)	Graded yeast hydrolysate supplementation in a 10% fishmeal diet	YH0.5 (10% fishmeal + 0.5% yeast hydrolysate)	FM25 (25% fishmeal positive-control diet)	Cumulative survival after Vibrio parahaemolyticus challenge (%)	Not reported numerically	Not reported numerically	3	3	Not estimable	Not estimable	Not estimable	Figure 2 shows time-specific survival and significance lettering, but an exact standardized effect was not calculated	Exact group means and SDs at the selected challenge endpoint were available only graphically
Hamidoghli et al. (2023)	Whiteleg shrimp (Litopenaeus vannamei)	Graded yeast hydrolysate supplementation in a 10% fishmeal diet	YH1 (10% fishmeal + 1% yeast hydrolysate)	FM25 (25% fishmeal positive-control diet)	Cumulative survival after Vibrio parahaemolyticus challenge (%)	Not reported numerically	Not reported numerically	3	3	Not estimable	Not estimable	Not estimable	Figure 2 shows time-specific survival and significance lettering, but an exact standardized effect was not calculated	Exact group means and SDs at the selected challenge endpoint were available only graphically
Hamidoghli et al. (2023)	Whiteleg shrimp (Litopenaeus vannamei)	Graded yeast hydrolysate supplementation in a 10% fishmeal diet	YH2 (10% fishmeal + 2% yeast hydrolysate)	FM25 (25% fishmeal positive-control diet)	Cumulative survival after Vibrio parahaemolyticus challenge (%)	Not reported numerically	Not reported numerically	3	3	Not estimable	Not estimable	Not estimable	Figure 2 shows time-specific survival and significance lettering, but an exact standardized effect was not calculated	Exact group means and SDs at the selected challenge endpoint were available only graphically
Hamidoghli et al. (2023)	Whiteleg shrimp (Litopenaeus vannamei)	Graded yeast hydrolysate supplementation in a 10% fishmeal diet	YH4 (10% fishmeal + 4% yeast hydrolysate)	FM25 (25% fishmeal positive-control diet)	Cumulative survival after Vibrio parahaemolyticus challenge (%)	Not reported numerically	Not reported numerically	3	3	Not estimable	Not estimable	Not estimable	Figure 2 shows time-specific survival and significance lettering, but an exact standardized effect was not calculated	Exact group means and SDs at the selected challenge endpoint were available only graphically

Note: Values in Tables 3-7 are mean +/- SD from three independent tanks per diet. Each yeast-hydrolysate dose was compared with the 10% fishmeal CON diet and the 25% fishmeal FM25 reference diet. Initial body weight was excluded as a baseline measure. Cumulative survival after the Vibrio parahaemolyticus challenge was marked not estimable because exact endpoint means and SDs were not printed. Pairwise 95% CIs are descriptive and unadjusted.

Source: Hamidoghli et al. (2023), https://doi.org/10.3390/ani13111877

Study 21. Niu et al. (2019): multi-strain probiotic supplementation in a low-fishmeal diet for juvenile olive flounder

Niu et al. (2019) - numerical effect sizes cannot yet be estimated because the uploaded file is an article preview

Full article tables or figures are required before Hedges' g and 95% confidence intervals can be calculated.

Study	Species	Strategy	Intervention	Comparator	Outcome and unit	Intervention mean +/- SD	Comparator mean +/- SD	n intervention (tanks)	n comparator (tanks)	Effect-size metric	Effect size	95% CI	Interpretation	Reason not estimated
Niu et al. (2019)	Juvenile olive flounder (Paralichthys olivaceus)	Multi-strain probiotic supplementation in a diet replacing 30% of fishmeal protein	Pro (FM30 supplemented with 10^8-10^9 CFU/kg multi-strain probiotic)	FM30 (30% of fishmeal protein replaced by mixed plant and animal proteins)	Growth performance outcomes	Not available in the uploaded preview	Not available in the uploaded preview	3	3	Not estimable	Not estimable	Not estimable	No significant difference reported for this comparison.	The uploaded Study 21 file is a ScienceDirect article preview rather than the full paper. Exact treatment means and SD/SE values from the article tables and figures were not available.
Niu et al. (2019)	Juvenile olive flounder (Paralichthys olivaceus)	Multi-strain probiotic supplementation in a diet replacing 30% of fishmeal protein	Pro (FM30 supplemented with 10^8-10^9 CFU/kg multi-strain probiotic)	FM30 (30% of fishmeal protein replaced by mixed plant and animal proteins)	Feed efficiency	Not available in the uploaded preview	Not available in the uploaded preview	3	3	Not estimable	Not estimable	Not estimable	No significant difference reported for this comparison.	The uploaded Study 21 file is a ScienceDirect article preview rather than the full paper. Exact treatment means and SD/SE values from the article tables and figures were not available.
Niu et al. (2019)	Juvenile olive flounder (Paralichthys olivaceus)	Multi-strain probiotic supplementation in a diet replacing 30% of fishmeal protein	Pro (FM30 supplemented with 10^8-10^9 CFU/kg multi-strain probiotic)	FM30 (30% of fishmeal protein replaced by mixed plant and animal proteins)	Survival rate	Not available in the uploaded preview	Not available in the uploaded preview	3	3	Not estimable	Not estimable	Not estimable	No significant difference reported for this comparison.	The uploaded Study 21 file is a ScienceDirect article preview rather than the full paper. Exact treatment means and SD/SE values from the article tables and figures were not available.
Niu et al. (2019)	Juvenile olive flounder (Paralichthys olivaceus)	Multi-strain probiotic supplementation in a diet replacing 30% of fishmeal protein	Pro (FM30 supplemented with 10^8-10^9 CFU/kg multi-strain probiotic)	FM30 (30% of fishmeal protein replaced by mixed plant and animal proteins)	Lipid retention	Not available in the uploaded preview	Not available in the uploaded preview	3	3	Not estimable	Not estimable	Not estimable	Pro significantly improved lipid retention relative to FM30.	The uploaded Study 21 file is a ScienceDirect article preview rather than the full paper. Exact treatment means and SD/SE values from the article tables and figures were not available.
Niu et al. (2019)	Juvenile olive flounder (Paralichthys olivaceus)	Multi-strain probiotic supplementation in a diet replacing 30% of fishmeal protein	Pro (FM30 supplemented with 10^8-10^9 CFU/kg multi-strain probiotic)	FM30 (30% of fishmeal protein replaced by mixed plant and animal proteins)	Other nutrient-utilization outcomes	Not available in the uploaded preview	Not available in the uploaded preview	3	3	Not estimable	Not estimable	Not estimable	No significant difference reported apart from lipid retention.	The uploaded Study 21 file is a ScienceDirect article preview rather than the full paper. Exact treatment means and SD/SE values from the article tables and figures were not available.
Niu et al. (2019)	Juvenile olive flounder (Paralichthys olivaceus)	Multi-strain probiotic supplementation in a diet replacing 30% of fishmeal protein	Pro (FM30 supplemented with 10^8-10^9 CFU/kg multi-strain probiotic)	FM30 (30% of fishmeal protein replaced by mixed plant and animal proteins)	Whole-body proximate composition	Not available in the uploaded preview	Not available in the uploaded preview	3	3	Not estimable	Not estimable	Not estimable	No significant difference reported for this comparison.	The uploaded Study 21 file is a ScienceDirect article preview rather than the full paper. Exact treatment means and SD/SE values from the article tables and figures were not available.
Niu et al. (2019)	Juvenile olive flounder (Paralichthys olivaceus)	Multi-strain probiotic supplementation in a diet replacing 30% of fishmeal protein	Pro (FM30 supplemented with 10^8-10^9 CFU/kg multi-strain probiotic)	FM30 (30% of fishmeal protein replaced by mixed plant and animal proteins)	Fillet proximate composition	Not available in the uploaded preview	Not available in the uploaded preview	3	3	Not estimable	Not estimable	Not estimable	No significant difference reported for this comparison.	The uploaded Study 21 file is a ScienceDirect article preview rather than the full paper. Exact treatment means and SD/SE values from the article tables and figures were not available.
Niu et al. (2019)	Juvenile olive flounder (Paralichthys olivaceus)	Multi-strain probiotic supplementation in a diet replacing 30% of fishmeal protein	Pro (FM30 supplemented with 10^8-10^9 CFU/kg multi-strain probiotic)	FM30 (30% of fishmeal protein replaced by mixed plant and animal proteins)	Myeloperoxidase activity	Not available in the uploaded preview	Not available in the uploaded preview	3	3	Not estimable	Not estimable	Not estimable	Pro was significantly higher than FM30.	The uploaded Study 21 file is a ScienceDirect article preview rather than the full paper. Exact treatment means and SD/SE values from the article tables and figures were not available.
Niu et al. (2019)	Juvenile olive flounder (Paralichthys olivaceus)	Multi-strain probiotic supplementation in a diet replacing 30% of fishmeal protein	Pro (FM30 supplemented with 10^8-10^9 CFU/kg multi-strain probiotic)	FM30 (30% of fishmeal protein replaced by mixed plant and animal proteins)	Lysozyme activity	Not available in the uploaded preview	Not available in the uploaded preview	3	3	Not estimable	Not estimable	Not estimable	Pro was significantly higher than FM30.	The uploaded Study 21 file is a ScienceDirect article preview rather than the full paper. Exact treatment means and SD/SE values from the article tables and figures were not available.
Niu et al. (2019)	Juvenile olive flounder (Paralichthys olivaceus)	Multi-strain probiotic supplementation in a diet replacing 30% of fishmeal protein	Pro (FM30 supplemented with 10^8-10^9 CFU/kg multi-strain probiotic)	FM30 (30% of fishmeal protein replaced by mixed plant and animal proteins)	Glutathione peroxidase activity	Not available in the uploaded preview	Not available in the uploaded preview	3	3	Not estimable	Not estimable	Not estimable	Pro was significantly higher than FM30.	The uploaded Study 21 file is a ScienceDirect article preview rather than the full paper. Exact treatment means and SD/SE values from the article tables and figures were not available.
Niu et al. (2019)	Juvenile olive flounder (Paralichthys olivaceus)	Multi-strain probiotic supplementation in a diet replacing 30% of fishmeal protein	Pro (FM30 supplemented with 10^8-10^9 CFU/kg multi-strain probiotic)	FM30 (30% of fishmeal protein replaced by mixed plant and animal proteins)	Liver IL-1beta expression	Not available in the uploaded preview	Not available in the uploaded preview	3	3	Not estimable	Not estimable	Not estimable	Pro was markedly upregulated relative to FM30.	The uploaded Study 21 file is a ScienceDirect article preview rather than the full paper. Exact treatment means and SD/SE values from the article tables and figures were not available.
Niu et al. (2019)	Juvenile olive flounder (Paralichthys olivaceus)	Multi-strain probiotic supplementation in a diet replacing 30% of fishmeal protein	Pro (FM30 supplemented with 10^8-10^9 CFU/kg multi-strain probiotic)	FM30 (30% of fishmeal protein replaced by mixed plant and animal proteins)	Liver IL-6 expression	Not available in the uploaded preview	Not available in the uploaded preview	3	3	Not estimable	Not estimable	Not estimable	Pro was markedly upregulated relative to FM30.	The uploaded Study 21 file is a ScienceDirect article preview rather than the full paper. Exact treatment means and SD/SE values from the article tables and figures were not available.
Niu et al. (2019)	Juvenile olive flounder (Paralichthys olivaceus)	Multi-strain probiotic supplementation in a diet replacing 30% of fishmeal protein	Pro (FM30 supplemented with 10^8-10^9 CFU/kg multi-strain probiotic)	FM30 (30% of fishmeal protein replaced by mixed plant and animal proteins)	Liver TNF-alpha expression	Not available in the uploaded preview	Not available in the uploaded preview	3	3	Not estimable	Not estimable	Not estimable	Pro was markedly upregulated relative to FM30.	The uploaded Study 21 file is a ScienceDirect article preview rather than the full paper. Exact treatment means and SD/SE values from the article tables and figures were not available.
Niu et al. (2019)	Juvenile olive flounder (Paralichthys olivaceus)	Multi-strain probiotic supplementation in a diet replacing 30% of fishmeal protein	Pro (FM30 supplemented with 10^8-10^9 CFU/kg multi-strain probiotic)	FM30 (30% of fishmeal protein replaced by mixed plant and animal proteins)	Lactobacillus relative abundance	Not available in the uploaded preview	Not available in the uploaded preview	3	3	Not estimable	Not estimable	Not estimable	Probiotic supplementation enriched Lactobacillus abundance; exact pairwise numerical data were unavailable.	The uploaded Study 21 file is a ScienceDirect article preview rather than the full paper. Exact treatment means and SD/SE values from the article tables and figures were not available.
Niu et al. (2019)	Juvenile olive flounder (Paralichthys olivaceus)	Multi-strain probiotic supplementation in a diet replacing 30% of fishmeal protein	Pro (FM30 supplemented with 10^8-10^9 CFU/kg multi-strain probiotic)	FM30 (30% of fishmeal protein replaced by mixed plant and animal proteins)	Gut microbial community composition	Not available in the uploaded preview	Not available in the uploaded preview	3	3	Not estimable	Not estimable	Not estimable	Probiotic supplementation modulated gut microbiota; no scalar effect size could be calculated.	The uploaded Study 21 file is a ScienceDirect article preview rather than the full paper. Exact treatment means and SD/SE values from the article tables and figures were not available.
Niu et al. (2019)	Juvenile olive flounder (Paralichthys olivaceus)	Multi-strain probiotic supplementation in a diet replacing 30% of fishmeal protein	Pro (FM30 supplemented with 10^8-10^9 CFU/kg multi-strain probiotic)	FM30 (30% of fishmeal protein replaced by mixed plant and animal proteins)	Predicted lipid-metabolism gene functions	Not available in the uploaded preview	Not available in the uploaded preview	3	3	Not estimable	Not estimable	Not estimable	Probiotic supplementation enriched the reported predicted microbial functions.	The uploaded Study 21 file is a ScienceDirect article preview rather than the full paper. Exact treatment means and SD/SE values from the article tables and figures were not available.
Niu et al. (2019)	Juvenile olive flounder (Paralichthys olivaceus)	Multi-strain probiotic supplementation in a diet replacing 30% of fishmeal protein	Pro (FM30 supplemented with 10^8-10^9 CFU/kg multi-strain probiotic)	FM30 (30% of fishmeal protein replaced by mixed plant and animal proteins)	Predicted carbohydrate-metabolism gene functions	Not available in the uploaded preview	Not available in the uploaded preview	3	3	Not estimable	Not estimable	Not estimable	Probiotic supplementation enriched the reported predicted microbial functions.	The uploaded Study 21 file is a ScienceDirect article preview rather than the full paper. Exact treatment means and SD/SE values from the article tables and figures were not available.
Niu et al. (2019)	Juvenile olive flounder (Paralichthys olivaceus)	Multi-strain probiotic supplementation in a diet replacing 30% of fishmeal protein	Pro (FM30 supplemented with 10^8-10^9 CFU/kg multi-strain probiotic)	Con (fishmeal-based reference diet)	Growth performance outcomes	Not available in the uploaded preview	Not available in the uploaded preview	3	3	Not estimable	Not estimable	Not estimable	No significant difference reported for this comparison.	The uploaded Study 21 file is a ScienceDirect article preview rather than the full paper. Exact treatment means and SD/SE values from the article tables and figures were not available.

Study	Species	Strategy	Intervention	Comparator	Outcome and unit	Intervention mean +/- SD	Comparator mean +/- SD	n intervention (tanks)	n comparator (tanks)	Effect-size metric	Effect size	95% CI	Interpretation	Reason not estimated
Niu et al. (2019)	Juvenile olive flounder (Paralichthys olivaceus)	Multi-strain probiotic supplementation in a diet replacing 30% of fishmeal protein	Pro (FM30 supplemented with 10^8-10^9 CFU/kg multi-strain probiotic)	Con (fishmeal-based reference diet)	Feed efficiency	Not available in the uploaded preview	Not available in the uploaded preview	3	3	Not estimable	Not estimable	Not estimable	No significant difference reported for this comparison.	The uploaded Study 21 file is a ScienceDirect article preview rather than the full paper. Exact treatment means and SD/SE values from the article tables and figures were not available.
Niu et al. (2019)	Juvenile olive flounder (Paralichthys olivaceus)	Multi-strain probiotic supplementation in a diet replacing 30% of fishmeal protein	Pro (FM30 supplemented with 10^8-10^9 CFU/kg multi-strain probiotic)	Con (fishmeal-based reference diet)	Survival rate	Not available in the uploaded preview	Not available in the uploaded preview	3	3	Not estimable	Not estimable	Not estimable	No significant difference reported for this comparison.	The uploaded Study 21 file is a ScienceDirect article preview rather than the full paper. Exact treatment means and SD/SE values from the article tables and figures were not available.
Niu et al. (2019)	Juvenile olive flounder (Paralichthys olivaceus)	Multi-strain probiotic supplementation in a diet replacing 30% of fishmeal protein	Pro (FM30 supplemented with 10^8-10^9 CFU/kg multi-strain probiotic)	Con (fishmeal-based reference diet)	Lipid retention	Not available in the uploaded preview	Not available in the uploaded preview	3	3	Not estimable	Not estimable	Not estimable	Pro was reported as comparable with the fishmeal-based Con diet.	The uploaded Study 21 file is a ScienceDirect article preview rather than the full paper. Exact treatment means and SD/SE values from the article tables and figures were not available.
Niu et al. (2019)	Juvenile olive flounder (Paralichthys olivaceus)	Multi-strain probiotic supplementation in a diet replacing 30% of fishmeal protein	Pro (FM30 supplemented with 10^8-10^9 CFU/kg multi-strain probiotic)	Con (fishmeal-based reference diet)	Other nutrient-utilization outcomes	Not available in the uploaded preview	Not available in the uploaded preview	3	3	Not estimable	Not estimable	Not estimable	No significant difference reported apart from lipid retention.	The uploaded Study 21 file is a ScienceDirect article preview rather than the full paper. Exact treatment means and SD/SE values from the article tables and figures were not available.
Niu et al. (2019)	Juvenile olive flounder (Paralichthys olivaceus)	Multi-strain probiotic supplementation in a diet replacing 30% of fishmeal protein	Pro (FM30 supplemented with 10^8-10^9 CFU/kg multi-strain probiotic)	Con (fishmeal-based reference diet)	Whole-body proximate composition	Not available in the uploaded preview	Not available in the uploaded preview	3	3	Not estimable	Not estimable	Not estimable	No significant difference reported for this comparison.	The uploaded Study 21 file is a ScienceDirect article preview rather than the full paper. Exact treatment means and SD/SE values from the article tables and figures were not available.
Niu et al. (2019)	Juvenile olive flounder (Paralichthys olivaceus)	Multi-strain probiotic supplementation in a diet replacing 30% of fishmeal protein	Pro (FM30 supplemented with 10^8-10^9 CFU/kg multi-strain probiotic)	Con (fishmeal-based reference diet)	Fillet proximate composition	Not available in the uploaded preview	Not available in the uploaded preview	3	3	Not estimable	Not estimable	Not estimable	No significant difference reported for this comparison.	The uploaded Study 21 file is a ScienceDirect article preview rather than the full paper. Exact treatment means and SD/SE values from the article tables and figures were not available.
Niu et al. (2019)	Juvenile olive flounder (Paralichthys olivaceus)	Multi-strain probiotic supplementation in a diet replacing 30% of fishmeal protein	Pro (FM30 supplemented with 10^8-10^9 CFU/kg multi-strain probiotic)	Con (fishmeal-based reference diet)	Myeloperoxidase activity	Not available in the uploaded preview	Not available in the uploaded preview	3	3	Not estimable	Not estimable	Not estimable	The uploaded preview did not state this pairwise comparison explicitly.	The uploaded Study 21 file is a ScienceDirect article preview rather than the full paper. Exact treatment means and SD/SE values from the article tables and figures were not available.
Niu et al. (2019)	Juvenile olive flounder (Paralichthys olivaceus)	Multi-strain probiotic supplementation in a diet replacing 30% of fishmeal protein	Pro (FM30 supplemented with 10^8-10^9 CFU/kg multi-strain probiotic)	Con (fishmeal-based reference diet)	Lysozyme activity	Not available in the uploaded preview	Not available in the uploaded preview	3	3	Not estimable	Not estimable	Not estimable	The uploaded preview did not state this pairwise comparison explicitly.	The uploaded Study 21 file is a ScienceDirect article preview rather than the full paper. Exact treatment means and SD/SE values from the article tables and figures were not available.
Niu et al. (2019)	Juvenile olive flounder (Paralichthys olivaceus)	Multi-strain probiotic supplementation in a diet replacing 30% of fishmeal protein	Pro (FM30 supplemented with 10^8-10^9 CFU/kg multi-strain probiotic)	Con (fishmeal-based reference diet)	Glutathione peroxidase activity	Not available in the uploaded preview	Not available in the uploaded preview	3	3	Not estimable	Not estimable	Not estimable	Pro was significantly higher than Con.	The uploaded Study 21 file is a ScienceDirect article preview rather than the full paper. Exact treatment means and SD/SE values from the article tables and figures were not available.
Niu et al. (2019)	Juvenile olive flounder (Paralichthys olivaceus)	Multi-strain probiotic supplementation in a diet replacing 30% of fishmeal protein	Pro (FM30 supplemented with 10^8-10^9 CFU/kg multi-strain probiotic)	Con (fishmeal-based reference diet)	Liver IL-1beta expression	Not available in the uploaded preview	Not available in the uploaded preview	3	3	Not estimable	Not estimable	Not estimable	Pro was markedly upregulated relative to Con.	The uploaded Study 21 file is a ScienceDirect article preview rather than the full paper. Exact treatment means and SD/SE values from the article tables and figures were not available.
Niu et al. (2019)	Juvenile olive flounder (Paralichthys olivaceus)	Multi-strain probiotic supplementation in a diet replacing 30% of fishmeal protein	Pro (FM30 supplemented with 10^8-10^9 CFU/kg multi-strain probiotic)	Con (fishmeal-based reference diet)	Liver IL-6 expression	Not available in the uploaded preview	Not available in the uploaded preview	3	3	Not estimable	Not estimable	Not estimable	Pro was markedly upregulated relative to Con.	The uploaded Study 21 file is a ScienceDirect article preview rather than the full paper. Exact treatment means and SD/SE values from the article tables and figures were not available.
Niu et al. (2019)	Juvenile olive flounder (Paralichthys olivaceus)	Multi-strain probiotic supplementation in a diet replacing 30% of fishmeal protein	Pro (FM30 supplemented with 10^8-10^9 CFU/kg multi-strain probiotic)	Con (fishmeal-based reference diet)	Liver TNF-alpha expression	Not available in the uploaded preview	Not available in the uploaded preview	3	3	Not estimable	Not estimable	Not estimable	Pro was markedly upregulated relative to Con.	The uploaded Study 21 file is a ScienceDirect article preview rather than the full paper. Exact treatment means and SD/SE values from the article tables and figures were not available.
Niu et al. (2019)	Juvenile olive flounder (Paralichthys olivaceus)	Multi-strain probiotic supplementation in a diet replacing 30% of fishmeal protein	Pro (FM30 supplemented with 10^8-10^9 CFU/kg multi-strain probiotic)	Con (fishmeal-based reference diet)	Lactobacillus relative abundance	Not available in the uploaded preview	Not available in the uploaded preview	3	3	Not estimable	Not estimable	Not estimable	Probiotic supplementation enriched Lactobacillus abundance; exact pairwise numerical data were unavailable.	The uploaded Study 21 file is a ScienceDirect article preview rather than the full paper. Exact treatment means and SD/SE values from the article tables and figures were not available.
Niu et al. (2019)	Juvenile olive flounder (Paralichthys olivaceus)	Multi-strain probiotic supplementation in a diet replacing 30% of fishmeal protein	Pro (FM30 supplemented with 10^8-10^9 CFU/kg multi-strain probiotic)	Con (fishmeal-based reference diet)	Gut microbial community composition	Not available in the uploaded preview	Not available in the uploaded preview	3	3	Not estimable	Not estimable	Not estimable	Probiotic supplementation modulated gut microbiota; no scalar effect size could be calculated.	The uploaded Study 21 file is a ScienceDirect article preview rather than the full paper. Exact treatment means and SD/SE values from the article tables and figures were not available.
Niu et al. (2019)	Juvenile olive flounder (Paralichthys olivaceus)	Multi-strain probiotic supplementation in a diet replacing 30% of fishmeal protein	Pro (FM30 supplemented with 10^8-10^9 CFU/kg multi-strain probiotic)	Con (fishmeal-based reference diet)	Predicted lipid-metabolism gene functions	Not available in the uploaded preview	Not available in the uploaded preview	3	3	Not estimable	Not estimable	Not estimable	Probiotic supplementation enriched the reported predicted microbial functions.	The uploaded Study 21 file is a ScienceDirect article preview rather than the full paper. Exact treatment means and SD/SE values from the article tables and figures were not available.
Niu et al. (2019)	Juvenile olive flounder (Paralichthys olivaceus)	Multi-strain probiotic supplementation in a diet replacing 30% of fishmeal protein	Pro (FM30 supplemented with 10^8-10^9 CFU/kg multi-strain probiotic)	Con (fishmeal-based reference diet)	Predicted carbohydrate-metabolism gene functions	Not available in the uploaded preview	Not available in the uploaded preview	3	3	Not estimable	Not estimable	Not estimable	Probiotic supplementation enriched the reported predicted microbial functions.	The uploaded Study 21 file is a ScienceDirect article preview rather than the full paper. Exact treatment means and SD/SE values from the article tables and figures were not available.
Niu et al. (2019)	Juvenile olive flounder (Paralichthys olivaceus)	Multi-strain probiotic supplementation in a diet replacing 30% of fishmeal protein	FM30 (30% of fishmeal protein replaced by mixed plant and animal proteins)	Con (fishmeal-based reference diet)	Growth performance outcomes	Not available in the uploaded preview	Not available in the uploaded preview	3	3	Not estimable	Not estimable	Not estimable	No significant difference reported for this comparison.	The uploaded Study 21 file is a ScienceDirect article preview rather than the full paper. Exact treatment means and SD/SE values from the article tables and figures were not available.
Niu et al. (2019)	Juvenile olive flounder (Paralichthys olivaceus)	Multi-strain probiotic supplementation in a diet replacing 30% of fishmeal protein	FM30 (30% of fishmeal protein replaced by mixed plant and animal proteins)	Con (fishmeal-based reference diet)	Feed efficiency	Not available in the uploaded preview	Not available in the uploaded preview	3	3	Not estimable	Not estimable	Not estimable	No significant difference reported for this comparison.	The uploaded Study 21 file is a ScienceDirect article preview rather than the full paper. Exact treatment means and SD/SE values from the article tables and figures were not available.
Niu et al. (2019)	Juvenile olive flounder (Paralichthys olivaceus)	Multi-strain probiotic supplementation in a diet replacing 30% of fishmeal protein	FM30 (30% of fishmeal protein replaced by mixed plant and animal proteins)	Con (fishmeal-based reference diet)	Survival rate	Not available in the uploaded preview	Not available in the uploaded preview	3	3	Not estimable	Not estimable	Not estimable	No significant difference reported for this comparison.	The uploaded Study 21 file is a ScienceDirect article preview rather than the full paper. Exact treatment means and SD/SE values from the article tables and figures were not available.
Niu et al. (2019)	Juvenile olive flounder (Paralichthys olivaceus)	Multi-strain probiotic supplementation in a diet replacing 30% of fishmeal protein	FM30 (30% of fishmeal protein replaced by mixed plant and animal proteins)	Con (fishmeal-based reference diet)	Lipid retention	Not available in the uploaded preview	Not available in the uploaded preview	3	3	Not estimable	Not estimable	Not estimable	FM30 had significantly lower lipid retention than Con.	The uploaded Study 21 file is a ScienceDirect article preview rather than the full paper. Exact treatment means and SD/SE values from the article tables and figures were not available.

Study	Species	Strategy	Intervention	Comparator	Outcome and unit	Intervention mean +/- SD	Comparator mean +/- SD	n intervention (tanks)	n comparator (tanks)	Effect-size metric	Effect size	95% CI	Interpretation	Reason not estimated
Niu et al. (2019)	Juvenile olive flounder (Paralichthys olivaceus)	Multi-strain probiotic supplementation in a diet replacing 30% of fishmeal protein	FM30 (30% of fishmeal protein replaced by mixed plant and animal proteins)	Con (fishmeal-based reference diet)	Other nutrient-utilization outcomes	Not available in the uploaded preview	Not available in the uploaded preview	3	3	Not estimable	Not estimable	Not estimable	No significant difference reported apart from lipid retention.	The uploaded Study 21 file is a ScienceDirect article preview rather than the full paper. Exact treatment means and SD/SE values from the article tables and figures were not available.
Niu et al. (2019)	Juvenile olive flounder (Paralichthys olivaceus)	Multi-strain probiotic supplementation in a diet replacing 30% of fishmeal protein	FM30 (30% of fishmeal protein replaced by mixed plant and animal proteins)	Con (fishmeal-based reference diet)	Whole-body proximate composition	Not available in the uploaded preview	Not available in the uploaded preview	3	3	Not estimable	Not estimable	Not estimable	No significant difference reported for this comparison.	The uploaded Study 21 file is a ScienceDirect article preview rather than the full paper. Exact treatment means and SD/SE values from the article tables and figures were not available.
Niu et al. (2019)	Juvenile olive flounder (Paralichthys olivaceus)	Multi-strain probiotic supplementation in a diet replacing 30% of fishmeal protein	FM30 (30% of fishmeal protein replaced by mixed plant and animal proteins)	Con (fishmeal-based reference diet)	Fillet proximate composition	Not available in the uploaded preview	Not available in the uploaded preview	3	3	Not estimable	Not estimable	Not estimable	No significant difference reported for this comparison.	The uploaded Study 21 file is a ScienceDirect article preview rather than the full paper. Exact treatment means and SD/SE values from the article tables and figures were not available.
Niu et al. (2019)	Juvenile olive flounder (Paralichthys olivaceus)	Multi-strain probiotic supplementation in a diet replacing 30% of fishmeal protein	FM30 (30% of fishmeal protein replaced by mixed plant and animal proteins)	Con (fishmeal-based reference diet)	Myeloperoxidase activity	Not available in the uploaded preview	Not available in the uploaded preview	3	3	Not estimable	Not estimable	Not estimable	The uploaded preview did not state this pairwise comparison explicitly.	The uploaded Study 21 file is a ScienceDirect article preview rather than the full paper. Exact treatment means and SD/SE values from the article tables and figures were not available.
Niu et al. (2019)	Juvenile olive flounder (Paralichthys olivaceus)	Multi-strain probiotic supplementation in a diet replacing 30% of fishmeal protein	FM30 (30% of fishmeal protein replaced by mixed plant and animal proteins)	Con (fishmeal-based reference diet)	Lysozyme activity	Not available in the uploaded preview	Not available in the uploaded preview	3	3	Not estimable	Not estimable	Not estimable	The uploaded preview did not state this pairwise comparison explicitly.	The uploaded Study 21 file is a ScienceDirect article preview rather than the full paper. Exact treatment means and SD/SE values from the article tables and figures were not available.
Niu et al. (2019)	Juvenile olive flounder (Paralichthys olivaceus)	Multi-strain probiotic supplementation in a diet replacing 30% of fishmeal protein	FM30 (30% of fishmeal protein replaced by mixed plant and animal proteins)	Con (fishmeal-based reference diet)	Glutathione peroxidase activity	Not available in the uploaded preview	Not available in the uploaded preview	3	3	Not estimable	Not estimable	Not estimable	FM30 was significantly lower than Con.	The uploaded Study 21 file is a ScienceDirect article preview rather than the full paper. Exact treatment means and SD/SE values from the article tables and figures were not available.
Niu et al. (2019)	Juvenile olive flounder (Paralichthys olivaceus)	Multi-strain probiotic supplementation in a diet replacing 30% of fishmeal protein	FM30 (30% of fishmeal protein replaced by mixed plant and animal proteins)	Con (fishmeal-based reference diet)	Liver IL-1beta expression	Not available in the uploaded preview	Not available in the uploaded preview	3	3	Not estimable	Not estimable	Not estimable	The uploaded preview did not state the Con-FM30 pairwise result explicitly.	The uploaded Study 21 file is a ScienceDirect article preview rather than the full paper. Exact treatment means and SD/SE values from the article tables and figures were not available.
Niu et al. (2019)	Juvenile olive flounder (Paralichthys olivaceus)	Multi-strain probiotic supplementation in a diet replacing 30% of fishmeal protein	FM30 (30% of fishmeal protein replaced by mixed plant and animal proteins)	Con (fishmeal-based reference diet)	Liver IL-6 expression	Not available in the uploaded preview	Not available in the uploaded preview	3	3	Not estimable	Not estimable	Not estimable	The uploaded preview did not state the Con-FM30 pairwise result explicitly.	The uploaded Study 21 file is a ScienceDirect article preview rather than the full paper. Exact treatment means and SD/SE values from the article tables and figures were not available.
Niu et al. (2019)	Juvenile olive flounder (Paralichthys olivaceus)	Multi-strain probiotic supplementation in a diet replacing 30% of fishmeal protein	FM30 (30% of fishmeal protein replaced by mixed plant and animal proteins)	Con (fishmeal-based reference diet)	Liver TNF-alpha expression	Not available in the uploaded preview	Not available in the uploaded preview	3	3	Not estimable	Not estimable	Not estimable	The uploaded preview did not state the Con-FM30 pairwise result explicitly.	The uploaded Study 21 file is a ScienceDirect article preview rather than the full paper. Exact treatment means and SD/SE values from the article tables and figures were not available.
Niu et al. (2019)	Juvenile olive flounder (Paralichthys olivaceus)	Multi-strain probiotic supplementation in a diet replacing 30% of fishmeal protein	FM30 (30% of fishmeal protein replaced by mixed plant and animal proteins)	Con (fishmeal-based reference diet)	Lactobacillus relative abundance	Not available in the uploaded preview	Not available in the uploaded preview	3	3	Not estimable	Not estimable	Not estimable	The uploaded preview did not state the Con-FM30 pairwise result explicitly.	The uploaded Study 21 file is a ScienceDirect article preview rather than the full paper. Exact treatment means and SD/SE values from the article tables and figures were not available.
Niu et al. (2019)	Juvenile olive flounder (Paralichthys olivaceus)	Multi-strain probiotic supplementation in a diet replacing 30% of fishmeal protein	FM30 (30% of fishmeal protein replaced by mixed plant and animal proteins)	Con (fishmeal-based reference diet)	Gut microbial community composition	Not available in the uploaded preview	Not available in the uploaded preview	3	3	Not estimable	Not estimable	Not estimable	The uploaded preview did not state the Con-FM30 pairwise result explicitly.	The uploaded Study 21 file is a ScienceDirect article preview rather than the full paper. Exact treatment means and SD/SE values from the article tables and figures were not available.
Niu et al. (2019)	Juvenile olive flounder (Paralichthys olivaceus)	Multi-strain probiotic supplementation in a diet replacing 30% of fishmeal protein	FM30 (30% of fishmeal protein replaced by mixed plant and animal proteins)	Con (fishmeal-based reference diet)	Predicted lipid-metabolism gene functions	Not available in the uploaded preview	Not available in the uploaded preview	3	3	Not estimable	Not estimable	Not estimable	The uploaded preview did not state the Con-FM30 pairwise result explicitly.	The uploaded Study 21 file is a ScienceDirect article preview rather than the full paper. Exact treatment means and SD/SE values from the article tables and figures were not available.
Niu et al. (2019)	Juvenile olive flounder (Paralichthys olivaceus)	Multi-strain probiotic supplementation in a diet replacing 30% of fishmeal protein	FM30 (30% of fishmeal protein replaced by mixed plant and animal proteins)	Con (fishmeal-based reference diet)	Predicted carbohydrate-metabolism gene functions	Not available in the uploaded preview	Not available in the uploaded preview	3	3	Not estimable	Not estimable	Not estimable	The uploaded preview did not state the Con-FM30 pairwise result explicitly.	The uploaded Study 21 file is a ScienceDirect article preview rather than the full paper. Exact treatment means and SD/SE values from the article tables and figures were not available.

Note: The tank is the independent unit (n = 3 per diet). The uploaded Study 21 document contains a ScienceDirect preview, abstract, and section snippets rather than the full numerical tables. Therefore, no treatment means, SD/SE values, Hedges' g estimates, or 95% confidence intervals were invented. The qualitative interpretations reproduce only relationships explicitly described in the preview.
Source: Niu et al. (2019), https://doi.org/10.1016/j.fsi.2019.07.056

Study 22. Zhao et al. (2023): tributyrin and alanyl-glutamine supplementation in high-soybean-meal diets for largemouth bass

Study	Species	Strategy	Intervention	Comparator	Outcome and unit	Intervention mean +/- SD	Comparator mean +/- SD	n interventio n (tanks)	n comparator (tanks)	Effect-size metric	Effect size	95% CI	Interpretation	Reason not estimated
Zhao et al. (2023)	Largemouth bass (Micropterus salmoides)	Tributyrin or alanyl-glutamine supplementation in a diet containing 41.3% soybean meal and 35% fishmeal	AGn1 (SM diet + 1% alanyl-glutamine)	SM (41.3% soybean-meal negative-control diet)	Survival rate (%)	93.33 +/- 7.64	80.00 +/- 26.47	3	3	Hedges' g	0.55	-0.79 to 1.88	Higher, more favorable value with supplementation; unadjusted 95% CI included 0	Not applicable - reported SE converted to SD as SE x sqrt(3)
Zhao et al. (2023)	Largemouth bass (Micropterus salmoides)	Tributyrin or alanyl-glutamine supplementation in a diet containing 41.3% soybean meal and 35% fishmeal	AGn1 (SM diet + 1% alanyl-glutamine)	SM (41.3% soybean-meal negative-control diet)	Weight gain (%)	189.05 +/- 28.56	179.85 +/- 13.77	3	3	Hedges' g	0.33	-0.97 to 1.63	Higher, more favorable value with supplementation; unadjusted 95% CI included 0	Not applicable - reported SE converted to SD as SE x sqrt(3)
Zhao et al. (2023)	Largemouth bass (Micropterus salmoides)	Tributyrin or alanyl-glutamine supplementation in a diet containing 41.3% soybean meal and 35% fishmeal	AGn1 (SM diet + 1% alanyl-glutamine)	SM (41.3% soybean-meal negative-control diet)	Specific growth rate (%/day)	1.890 +/- 0.173	1.840 +/- 0.087	3	3	Hedges' g	0.29	-1.00 to 1.59	Higher, more favorable value with supplementation; unadjusted 95% CI included 0	Not applicable - reported SE converted to SD as SE x sqrt(3)
Zhao et al. (2023)	Largemouth bass (Micropterus salmoides)	Tributyrin or alanyl-glutamine supplementation in a diet containing 41.3% soybean meal and 35% fishmeal	AGn1 (SM diet + 1% alanyl-glutamine)	SM (41.3% soybean-meal negative-control diet)	Feed conversion ratio	0.990 +/- 0.052	1.300 +/- 0.225	3	3	Hedges' g (direction reversed)	1.52	-0.14 to 3.17	Lower, more favorable value with supplementation; unadjusted 95% CI included 0	Not applicable - reported SE converted to SD as SE x sqrt(3)
Zhao et al. (2023)	Largemouth bass (Micropterus salmoides)	Tributyrin or alanyl-glutamine supplementation in a diet containing 41.3% soybean meal and 35% fishmeal	AGn1 (SM diet + 1% alanyl-glutamine)	SM (41.3% soybean-meal negative-control diet)	Hepatosomatic index (%)	1.540 +/- 0.121	1.850 +/- 0.104	3	3	Hedges' g	-2.2	-4.19 to -0.21	Lower measured value with supplementation; unadjusted 95% CI excluded 0	Not applicable - reported SE converted to SD as SE x sqrt(3)
Zhao et al. (2023)	Largemouth bass (Micropterus salmoides)	Tributyrin or alanyl-glutamine supplementation in a diet containing 41.3% soybean meal and 35% fishmeal	AGn1 (SM diet + 1% alanyl-glutamine)	SM (41.3% soybean-meal negative-control diet)	Viscerosomatic index (%)	7.770 +/- 0.935	7.890 +/- 0.364	3	3	Hedges' g	-0.14	-1.42 to 1.15	Lower measured value with supplementation; unadjusted 95% CI included 0	Not applicable - reported SE converted to SD as SE x sqrt(3)
Zhao et al. (2023)	Largemouth bass (Micropterus salmoides)	Tributyrin or alanyl-glutamine supplementation in a diet containing 41.3% soybean meal and 35% fishmeal	AGn1 (SM diet + 1% alanyl-glutamine)	SM (41.3% soybean-meal negative-control diet)	Crude protein (%)	17.00 +/- 0.16	17.01 +/- 0.09	3	3	Hedges' g	-0.06	-1.34 to 1.22	Lower measured value with supplementation; unadjusted 95% CI included 0	Not applicable - reported SE converted to SD as SE x sqrt(3)
Zhao et al. (2023)	Largemouth bass (Micropterus salmoides)	Tributyrin or alanyl-glutamine supplementation in a diet containing 41.3% soybean meal and 35% fishmeal	AGn1 (SM diet + 1% alanyl-glutamine)	SM (41.3% soybean-meal negative-control diet)	Crude lipid (%)	4.510 +/- 0.087	4.620 +/- 0.087	3	3	Hedges' g	-1.02	-2.48 to 0.44	Lower measured value with supplementation; unadjusted 95% CI included 0	Not applicable - reported SE converted to SD as SE x sqrt(3)
Zhao et al. (2023)	Largemouth bass (Micropterus salmoides)	Tributyrin or alanyl-glutamine supplementation in a diet containing 41.3% soybean meal and 35% fishmeal	AGn1 (SM diet + 1% alanyl-glutamine)	SM (41.3% soybean-meal negative-control diet)	Crude ash (%)	3.890 +/- 0.069	3.890 +/- 0.069	3	3	Hedges' g	0.0	-1.28 to 1.28	No observed mean difference; unadjusted 95% CI included 0	Not applicable - reported SE converted to SD as SE x sqrt(3)
Zhao et al. (2023)	Largemouth bass (Micropterus salmoides)	Tributyrin or alanyl-glutamine supplementation in a diet containing 41.3% soybean meal and 35% fishmeal	AGn1 (SM diet + 1% alanyl-glutamine)	SM (41.3% soybean-meal negative-control diet)	Moisture (%)	70.46 +/- 1.14	70.81 +/- 0.48	3	3	Hedges' g	-0.32	-1.62 to 0.98	Lower measured value with supplementation; unadjusted 95% CI included 0	Not applicable - reported SE converted to SD as SE x sqrt(3)
Zhao et al. (2023)	Largemouth bass (Micropterus salmoides)	Tributyrin or alanyl-glutamine supplementation in a diet containing 41.3% soybean meal and 35% fishmeal	AGn1 (SM diet + 1% alanyl-glutamine)	SM (41.3% soybean-meal negative-control diet)	Foregut villus height (µm)	1037.50 +/- 3.95	871.04 +/- 32.87	3	3	Hedges' g	5.69	1.54 to 9.83	Higher, more favorable value with supplementation; unadjusted 95% CI excluded 0	Not applicable - reported SE converted to SD as SE x sqrt(3)
Zhao et al. (2023)	Largemouth bass (Micropterus salmoides)	Tributyrin or alanyl-glutamine supplementation in a diet containing 41.3% soybean meal and 35% fishmeal	AGn1 (SM diet + 1% alanyl-glutamine)	SM (41.3% soybean-meal negative-control diet)	Foregut crypt depth (µm)	28.20 +/- 1.39	41.60 +/- 4.02	3	3	Hedges' g (direction reversed)	3.57	0.78 to 6.35	Lower, more favorable value with supplementation; unadjusted 95% CI excluded 0	Not applicable - reported SE converted to SD as SE x sqrt(3)
Zhao et al. (2023)	Largemouth bass (Micropterus salmoides)	Tributyrin or alanyl-glutamine supplementation in a diet containing 41.3% soybean meal and 35% fishmeal	AGn1 (SM diet + 1% alanyl-glutamine)	SM (41.3% soybean-meal negative-control diet)	Foregut mucosal thickness (µm)	1068.34 +/- 4.50	915.59 +/- 28.94	3	3	Hedges' g	5.9	1.62 to 10.18	Higher, more favorable value with supplementation; unadjusted 95% CI excluded 0	Not applicable - reported SE converted to SD as SE x sqrt(3)
Zhao et al. (2023)	Largemouth bass (Micropterus salmoides)	Tributyrin or alanyl-glutamine supplementation in a diet containing 41.3% soybean meal and 35% fishmeal	AGn1 (SM diet + 1% alanyl-glutamine)	SM (41.3% soybean-meal negative-control diet)	Midgut villus height (µm)	765.88 +/- 9.51	557.82 +/- 53.71	3	3	Hedges' g	4.32	1.06 to 7.57	Higher, more favorable value with supplementation; unadjusted 95% CI excluded 0	Not applicable - reported SE converted to SD as SE x sqrt(3)
Zhao et al. (2023)	Largemouth bass (Micropterus salmoides)	Tributyrin or alanyl-glutamine supplementation in a diet containing 41.3% soybean meal and 35% fishmeal	AGn1 (SM diet + 1% alanyl-glutamine)	SM (41.3% soybean-meal negative-control diet)	Midgut crypt depth (µm)	30.37 +/- 2.29	42.69 +/- 1.51	3	3	Hedges' g (direction reversed)	5.09	1.34 to 8.84	Lower, more favorable value with supplementation; unadjusted 95% CI excluded 0	Not applicable - reported SE converted to SD as SE x sqrt(3)
Zhao et al. (2023)	Largemouth bass (Micropterus salmoides)	Tributyrin or alanyl-glutamine supplementation in a diet containing 41.3% soybean meal and 35% fishmeal	AGn1 (SM diet + 1% alanyl-glutamine)	SM (41.3% soybean-meal negative-control diet)	Midgut mucosal thickness (µm)	798.91 +/- 11.02	598.25 +/- 54.33	3	3	Hedges' g	4.09	0.98 to 7.21	Higher, more favorable value with supplementation; unadjusted 95% CI excluded 0	Not applicable - reported SE converted to SD as SE x sqrt(3)
Zhao et al. (2023)	Largemouth bass (Micropterus salmoides)	Tributyrin or alanyl-glutamine supplementation in a diet containing 41.3% soybean meal and 35% fishmeal	AGn1 (SM diet + 1% alanyl-glutamine)	SM (41.3% soybean-meal negative-control diet)	Hindgut villus height (µm)	654.64 +/- 23.24	509.46 +/- 8.07	3	3	Hedges' g	6.68	1.88 to 11.48	Higher, more favorable value with supplementation; unadjusted 95% CI excluded 0	Not applicable - reported SE converted to SD as SE x sqrt(3)
Zhao et al. (2023)	Largemouth bass (Micropterus salmoides)	Tributyrin or alanyl-glutamine supplementation in a diet containing 41.3% soybean meal and 35% fishmeal	AGn1 (SM diet + 1% alanyl-glutamine)	SM (41.3% soybean-meal negative-control diet)	Hindgut crypt depth (µm)	47.90 +/- 4.40	53.25 +/- 5.42	3	3	Hedges' g (direction reversed)	0.87	-0.55 to 2.28	Lower, more favorable value with supplementation; unadjusted 95% CI included 0	Not applicable - reported SE converted to SD as SE x sqrt(3)
Zhao et al. (2023)	Largemouth bass (Micropterus salmoides)	Tributyrin or alanyl-glutamine supplementation in a diet containing 41.3% soybean meal and 35% fishmeal	AGn1 (SM diet + 1% alanyl-glutamine)	SM (41.3% soybean-meal negative-control diet)	Hindgut mucosal thickness (µm)	705.28 +/- 27.59	559.31 +/- 4.33	3	3	Hedges' g	5.91	1.62 to 10.21	Higher, more favorable value with supplementation; unadjusted 95% CI excluded 0	Not applicable - reported SE converted to SD as SE x sqrt(3)
Zhao et al. (2023)	Largemouth bass (Micropterus salmoides)	Tributyrin or alanyl-glutamine supplementation in a diet containing 41.3% soybean meal and 35% fishmeal	AGn2 (SM diet + 2% alanyl-glutamine)	SM (41.3% soybean-meal negative-control diet)	Survival rate (%)	100.00 +/- 0.00	80.00 +/- 26.47	3	3	Hedges' g	0.85	-0.56 to 2.27	Higher, more favorable value with supplementation; unadjusted 95% CI included 0	Not applicable - reported SE converted to SD as SE x sqrt(3)
Zhao et al. (2023)	Largemouth bass (Micropterus salmoides)	Tributyrin or alanyl-glutamine supplementation in a diet containing 41.3% soybean meal and 35% fishmeal	AGn2 (SM diet + 2% alanyl-glutamine)	SM (41.3% soybean-meal negative-control diet)	Weight gain (%)	212.70 +/- 20.06	179.85 +/- 13.77	3	3	Hedges' g	1.53	-0.13 to 3.19	Higher, more favorable value with supplementation; unadjusted 95% CI included 0	Not applicable - reported SE converted to SD as SE x sqrt(3)
Zhao et al. (2023)	Largemouth bass (Micropterus salmoides)	Tributyrin or alanyl-glutamine supplementation in a diet containing 41.3% soybean meal and 35% fishmeal	AGn2 (SM diet + 2% alanyl-glutamine)	SM (41.3% soybean-meal negative-control diet)	Specific growth rate (%/day)	2.000 +/- 0.069	1.840 +/- 0.087	3	3	Hedges' g	1.63	-0.08 to 3.34	Higher, more favorable value with supplementation; unadjusted 95% CI included 0	Not applicable - reported SE converted to SD as SE x sqrt(3)
Zhao et al. (2023)	Largemouth bass (Micropterus salmoides)	Tributyrin or alanyl-glutamine supplementation in a diet containing 41.3% soybean meal and 35% fishmeal	AGn2 (SM diet + 2% alanyl-glutamine)	SM (41.3% soybean-meal negative-control diet)	Feed conversion ratio	0.880 +/- 0.139	1.300 +/- 0.225	3	3	Hedges' g (direction reversed)	1.8	0.01 to 3.58	Lower, more favorable value with supplementation; unadjusted 95% CI excluded 0	Not applicable - reported SE converted to SD as SE x sqrt(3)
Zhao et al. (2023)	Largemouth bass (Micropterus salmoides)	Tributyrin or alanyl-glutamine supplementation in a diet containing 41.3% soybean meal and 35% fishmeal	AGn2 (SM diet + 2% alanyl-glutamine)	SM (41.3% soybean-meal negative-control diet)	Hepatosomatic index (%)	1.050 +/- 0.450	1.850 +/- 0.104	3	3	Hedges' g	-1.96	-3.82 to -0.09	Lower measured value with supplementation; unadjusted 95% CI excluded 0	Not applicable - reported SE converted to SD as SE x sqrt(3)

Study	Species	Strategy	Intervention	Comparator	Outcome and unit	Intervention mean +/- SD	Comparator mean +/- SD	n intervention (tanks)	n comparator (tanks)	Effect-size metric	Effect size	95% CI	Interpretation	Reason not estimated
Zhao et al. (2023)	Largemouth bass (Micropterus salmoides)	Tributyrin or alanyl-glutamine supplementation in a diet containing 41.3% soybean meal and 35% fishmeal	AGn2 (SM diet + 2% alanyl-glutamine)	SM (41.3% soybean-meal negative-control diet)	Viscerosomatic index (%)	6.460 +/- 0.849	7.890 +/- 0.364	3	3	Hedges' g	-1.75	-3.52 to 0.01	Lower measured value with supplementation; unadjusted 95% CI included 0	Not applicable - reported SE converted to SD as SE x sqrt(3)
Zhao et al. (2023)	Largemouth bass (Micropterus salmoides)	Tributyrin or alanyl-glutamine supplementation in a diet containing 41.3% soybean meal and 35% fishmeal	AGn2 (SM diet + 2% alanyl-glutamine)	SM (41.3% soybean-meal negative-control diet)	Crude protein (%)	17.09 +/- 0.19	17.01 +/- 0.09	3	3	Hedges' g	0.43	-0.88 to 1.75	Higher measured value with supplementation; unadjusted 95% CI included 0	Not applicable - reported SE converted to SD as SE x sqrt(3)
Zhao et al. (2023)	Largemouth bass (Micropterus salmoides)	Tributyrin or alanyl-glutamine supplementation in a diet containing 41.3% soybean meal and 35% fishmeal	AGn2 (SM diet + 2% alanyl-glutamine)	SM (41.3% soybean-meal negative-control diet)	Crude lipid (%)	4.550 +/- 0.104	4.620 +/- 0.087	3	3	Hedges' g	-0.59	-1.93 to 0.76	Lower measured value with supplementation; unadjusted 95% CI included 0	Not applicable - reported SE converted to SD as SE x sqrt(3)
Zhao et al. (2023)	Largemouth bass (Micropterus salmoides)	Tributyrin or alanyl-glutamine supplementation in a diet containing 41.3% soybean meal and 35% fishmeal	AGn2 (SM diet + 2% alanyl-glutamine)	SM (41.3% soybean-meal negative-control diet)	Crude ash (%)	3.950 +/- 0.035	3.890 +/- 0.069	3	3	Hedges' g	0.88	-0.54 to 2.29	Higher measured value with supplementation; unadjusted 95% CI included 0	Not applicable - reported SE converted to SD as SE x sqrt(3)
Zhao et al. (2023)	Largemouth bass (Micropterus salmoides)	Tributyrin or alanyl-glutamine supplementation in a diet containing 41.3% soybean meal and 35% fishmeal	AGn2 (SM diet + 2% alanyl-glutamine)	SM (41.3% soybean-meal negative-control diet)	Moisture (%)	70.50 +/- 1.04	70.81 +/- 0.48	3	3	Hedges' g	-0.31	-1.60 to 0.99	Lower measured value with supplementation; unadjusted 95% CI included 0	Not applicable - reported SE converted to SD as SE x sqrt(3)
Zhao et al. (2023)	Largemouth bass (Micropterus salmoides)	Tributyrin or alanyl-glutamine supplementation in a diet containing 41.3% soybean meal and 35% fishmeal	AGn2 (SM diet + 2% alanyl-glutamine)	SM (41.3% soybean-meal negative-control diet)	Foregut villus height (µm)	1020.00 +/- 2.81	871.04 +/- 32.87	3	3	Hedges' g	5.11	1.34 to 8.87	Higher, more favorable value with supplementation; unadjusted 95% CI excluded 0	Not applicable - reported SE converted to SD as SE x sqrt(3)
Zhao et al. (2023)	Largemouth bass (Micropterus salmoides)	Tributyrin or alanyl-glutamine supplementation in a diet containing 41.3% soybean meal and 35% fishmeal	AGn2 (SM diet + 2% alanyl-glutamine)	SM (41.3% soybean-meal negative-control diet)	Foregut crypt depth (µm)	26.28 +/- 1.78	41.60 +/- 4.02	3	3	Hedges' g (direction reversed)	3.94	0.93 to 6.96	Lower, more favorable value with supplementation; unadjusted 95% CI excluded 0	Not applicable - reported SE converted to SD as SE x sqrt(3)
Zhao et al. (2023)	Largemouth bass (Micropterus salmoides)	Tributyrin or alanyl-glutamine supplementation in a diet containing 41.3% soybean meal and 35% fishmeal	AGn2 (SM diet + 2% alanyl-glutamine)	SM (41.3% soybean-meal negative-control diet)	Foregut mucosal thickness (µm)	1048.83 +/- 1.58	915.59 +/- 28.94	3	3	Hedges' g	5.2	1.38 to 9.03	Higher, more favorable value with supplementation; unadjusted 95% CI excluded 0	Not applicable - reported SE converted to SD as SE x sqrt(3)
Zhao et al. (2023)	Largemouth bass (Micropterus salmoides)	Tributyrin or alanyl-glutamine supplementation in a diet containing 41.3% soybean meal and 35% fishmeal	AGn2 (SM diet + 2% alanyl-glutamine)	SM (41.3% soybean-meal negative-control diet)	Midgut villus height (µm)	1001.75 +/- 42.61	557.82 +/- 53.71	3	3	Hedges' g	7.33	2.09 to 12.56	Higher, more favorable value with supplementation; unadjusted 95% CI excluded 0	Not applicable - reported SE converted to SD as SE x sqrt(3)
Zhao et al. (2023)	Largemouth bass (Micropterus salmoides)	Tributyrin or alanyl-glutamine supplementation in a diet containing 41.3% soybean meal and 35% fishmeal	AGn2 (SM diet + 2% alanyl-glutamine)	SM (41.3% soybean-meal negative-control diet)	Midgut crypt depth (µm)	25.11 +/- 3.29	42.69 +/- 1.51	3	3	Hedges' g (direction reversed)	5.5	1.48 to 9.51	Lower, more favorable value with supplementation; unadjusted 95% CI excluded 0	Not applicable - reported SE converted to SD as SE x sqrt(3)
Zhao et al. (2023)	Largemouth bass (Micropterus salmoides)	Tributyrin or alanyl-glutamine supplementation in a diet containing 41.3% soybean meal and 35% fishmeal	AGn2 (SM diet + 2% alanyl-glutamine)	SM (41.3% soybean-meal negative-control diet)	Midgut mucosal thickness (µm)	1052.34 +/- 44.05	598.25 +/- 54.33	3	3	Hedges' g	7.34	2.10 to 12.59	Higher, more favorable value with supplementation; unadjusted 95% CI excluded 0	Not applicable - reported SE converted to SD as SE x sqrt(3)
Zhao et al. (2023)	Largemouth bass (Micropterus salmoides)	Tributyrin or alanyl-glutamine supplementation in a diet containing 41.3% soybean meal and 35% fishmeal	AGn2 (SM diet + 2% alanyl-glutamine)	SM (41.3% soybean-meal negative-control diet)	Hindgut villus height (µm)	734.28 +/- 40.79	509.46 +/- 8.07	3	3	Hedges' g	6.12	1.69 to 10.55	Higher, more favorable value with supplementation; unadjusted 95% CI excluded 0	Not applicable - reported SE converted to SD as SE x sqrt(3)
Zhao et al. (2023)	Largemouth bass (Micropterus salmoides)	Tributyrin or alanyl-glutamine supplementation in a diet containing 41.3% soybean meal and 35% fishmeal	AGn2 (SM diet + 2% alanyl-glutamine)	SM (41.3% soybean-meal negative-control diet)	Hindgut crypt depth (µm)	27.47 +/- 1.82	53.25 +/- 5.42	3	3	Hedges' g (direction reversed)	5.1	1.34 to 8.86	Lower, more favorable value with supplementation; unadjusted 95% CI excluded 0	Not applicable - reported SE converted to SD as SE x sqrt(3)
Zhao et al. (2023)	Largemouth bass (Micropterus salmoides)	Tributyrin or alanyl-glutamine supplementation in a diet containing 41.3% soybean meal and 35% fishmeal	AGn2 (SM diet + 2% alanyl-glutamine)	SM (41.3% soybean-meal negative-control diet)	Hindgut mucosal thickness (µm)	788.92 +/- 45.69	559.31 +/- 4.33	3	3	Hedges' g	5.66	1.53 to 9.79	Higher, more favorable value with supplementation; unadjusted 95% CI excluded 0	Not applicable - reported SE converted to SD as SE x sqrt(3)
Zhao et al. (2023)	Largemouth bass (Micropterus salmoides)	Tributyrin or alanyl-glutamine supplementation in a diet containing 41.3% soybean meal and 35% fishmeal	TB0.1 (SM diet + 0.1% tributyrin)	SM (41.3% soybean-meal negative-control diet)	Survival rate (%)	80.00 +/- 30.41	80.00 +/- 26.47	3	3	Hedges' g	0.0	-1.28 to 1.28	No observed mean difference; unadjusted 95% CI included 0	Not applicable - reported SE converted to SD as SE x sqrt(3)
Zhao et al. (2023)	Largemouth bass (Micropterus salmoides)	Tributyrin or alanyl-glutamine supplementation in a diet containing 41.3% soybean meal and 35% fishmeal	TB0.1 (SM diet + 0.1% tributyrin)	SM (41.3% soybean-meal negative-control diet)	Weight gain (%)	164.22 +/- 24.99	179.85 +/- 13.77	3	3	Hedges' g	-0.62	-1.97 to 0.73	Lower, less favorable value with supplementation; unadjusted 95% CI included 0	Not applicable - reported SE converted to SD as SE x sqrt(3)
Zhao et al. (2023)	Largemouth bass (Micropterus salmoides)	Tributyrin or alanyl-glutamine supplementation in a diet containing 41.3% soybean meal and 35% fishmeal	TB0.1 (SM diet + 0.1% tributyrin)	SM (41.3% soybean-meal negative-control diet)	Specific growth rate (%/day)	1.730 +/- 0.173	1.840 +/- 0.087	3	3	Hedges' g	-0.64	-2.00 to 0.71	Lower, less favorable value with supplementation; unadjusted 95% CI included 0	Not applicable - reported SE converted to SD as SE x sqrt(3)
Zhao et al. (2023)	Largemouth bass (Micropterus salmoides)	Tributyrin or alanyl-glutamine supplementation in a diet containing 41.3% soybean meal and 35% fishmeal	TB0.1 (SM diet + 0.1% tributyrin)	SM (41.3% soybean-meal negative-control diet)	Feed conversion ratio	1.610 +/- 0.554	1.300 +/- 0.225	3	3	Hedges' g (direction reversed)	-0.59	-1.93 to 0.76	Higher, less favorable value with supplementation; unadjusted 95% CI included 0	Not applicable - reported SE converted to SD as SE x sqrt(3)
Zhao et al. (2023)	Largemouth bass (Micropterus salmoides)	Tributyrin or alanyl-glutamine supplementation in a diet containing 41.3% soybean meal and 35% fishmeal	TB0.1 (SM diet + 0.1% tributyrin)	SM (41.3% soybean-meal negative-control diet)	Hepatosomatic index (%)	1.870 +/- 0.139	1.850 +/- 0.104	3	3	Hedges' g	0.13	-1.15 to 1.41	Higher measured value with supplementation; unadjusted 95% CI included 0	Not applicable - reported SE converted to SD as SE x sqrt(3)
Zhao et al. (2023)	Largemouth bass (Micropterus salmoides)	Tributyrin or alanyl-glutamine supplementation in a diet containing 41.3% soybean meal and 35% fishmeal	TB0.1 (SM diet + 0.1% tributyrin)	SM (41.3% soybean-meal negative-control diet)	Viscerosomatic index (%)	8.240 +/- 0.260	7.890 +/- 0.364	3	3	Hedges' g	0.89	-0.53 to 2.31	Higher measured value with supplementation; unadjusted 95% CI included 0	Not applicable - reported SE converted to SD as SE x sqrt(3)
Zhao et al. (2023)	Largemouth bass (Micropterus salmoides)	Tributyrin or alanyl-glutamine supplementation in a diet containing 41.3% soybean meal and 35% fishmeal	TB0.1 (SM diet + 0.1% tributyrin)	SM (41.3% soybean-meal negative-control diet)	Crude protein (%)	16.96 +/- 0.14	17.01 +/- 0.09	3	3	Hedges' g	-0.35	-1.65 to 0.96	Lower measured value with supplementation; unadjusted 95% CI included 0	Not applicable - reported SE converted to SD as SE x sqrt(3)
Zhao et al. (2023)	Largemouth bass (Micropterus salmoides)	Tributyrin or alanyl-glutamine supplementation in a diet containing 41.3% soybean meal and 35% fishmeal	TB0.1 (SM diet + 0.1% tributyrin)	SM (41.3% soybean-meal negative-control diet)	Crude lipid (%)	4.650 +/- 0.104	4.620 +/- 0.087	3	3	Hedges' g	0.25	-1.04 to 1.54	Higher measured value with supplementation; unadjusted 95% CI included 0	Not applicable - reported SE converted to SD as SE x sqrt(3)
Zhao et al. (2023)	Largemouth bass (Micropterus salmoides)	Tributyrin or alanyl-glutamine supplementation in a diet containing 41.3% soybean meal and 35% fishmeal	TB0.1 (SM diet + 0.1% tributyrin)	SM (41.3% soybean-meal negative-control diet)	Crude ash (%)	3.880 +/- 0.052	3.890 +/- 0.069	3	3	Hedges' g	-0.13	-1.41 to 1.15	Lower measured value with supplementation; unadjusted 95% CI included 0	Not applicable - reported SE converted to SD as SE x sqrt(3)
Zhao et al. (2023)	Largemouth bass (Micropterus salmoides)	Tributyrin or alanyl-glutamine supplementation in a diet containing 41.3% soybean meal and 35% fishmeal	TB0.1 (SM diet + 0.1% tributyrin)	SM (41.3% soybean-meal negative-control diet)	Moisture (%)	71.05 +/- 0.80	70.81 +/- 0.48	3	3	Hedges' g	0.29	-1.00 to 1.59	Higher measured value with supplementation; unadjusted 95% CI included 0	Not applicable - reported SE converted to SD as SE x sqrt(3)

Study	Species	Strategy	Intervention	Comparator	Outcome and unit	Intervention mean +/- SD	Comparator mean +/- SD	n intervention (tanks)	n comparator (tanks)	Effect-size metric	Effect size	95% CI	Interpretation	Reason not estimated
Zhao et al. (2023)	Largemouth bass (Micropterus salmoides)	Tributyrin or alanyl-glutamine supplementation in a diet containing 41.3% soybean meal and 35% fishmeal	TB0.1 (SM diet + 0.1% tributyrin)	SM (41.3% soybean-meal negative-control diet)	Foregut villus height (µm)	984.83 +/- 8.19	871.04 +/- 32.87	3	3	Hedges' g	3.8	0.87 to 6.73	Higher, more favorable value with supplementation; unadjusted 95% CI excluded 0	Not applicable - reported SE converted to SD as SE x sqrt(3)
Zhao et al. (2023)	Largemouth bass (Micropterus salmoides)	Tributyrin or alanyl-glutamine supplementation in a diet containing 41.3% soybean meal and 35% fishmeal	TB0.1 (SM diet + 0.1% tributyrin)	SM (41.3% soybean-meal negative-control diet)	Foregut crypt depth (µm)	25.82 +/- 1.11	41.60 +/- 4.02	3	3	Hedges' g (direction reversed)	4.28	1.05 to 7.52	Lower, more favorable value with supplementation; unadjusted 95% CI excluded 0	Not applicable - reported SE converted to SD as SE x sqrt(3)
Zhao et al. (2023)	Largemouth bass (Micropterus salmoides)	Tributyrin or alanyl-glutamine supplementation in a diet containing 41.3% soybean meal and 35% fishmeal	TB0.1 (SM diet + 0.1% tributyrin)	SM (41.3% soybean-meal negative-control diet)	Foregut mucosal thickness (µm)	1013.56 +/- 7.81	915.59 +/- 28.94	3	3	Hedges' g	3.7	0.83 to 6.56	Higher, more favorable value with supplementation; unadjusted 95% CI excluded 0	Not applicable - reported SE converted to SD as SE x sqrt(3)
Zhao et al. (2023)	Largemouth bass (Micropterus salmoides)	Tributyrin or alanyl-glutamine supplementation in a diet containing 41.3% soybean meal and 35% fishmeal	TB0.1 (SM diet + 0.1% tributyrin)	SM (41.3% soybean-meal negative-control diet)	Midgut villus height (µm)	857.00 +/- 11.33	557.82 +/- 53.71	3	3	Hedges' g	6.17	1.71 to 10.63	Higher, more favorable value with supplementation; unadjusted 95% CI excluded 0	Not applicable - reported SE converted to SD as SE x sqrt(3)
Zhao et al. (2023)	Largemouth bass (Micropterus salmoides)	Tributyrin or alanyl-glutamine supplementation in a diet containing 41.3% soybean meal and 35% fishmeal	TB0.1 (SM diet + 0.1% tributyrin)	SM (41.3% soybean-meal negative-control diet)	Midgut crypt depth (µm)	38.16 +/- 1.32	42.69 +/- 1.51	3	3	Hedges' g (direction reversed)	2.56	0.37 to 4.75	Lower, more favorable value with supplementation; unadjusted 95% CI excluded 0	Not applicable - reported SE converted to SD as SE x sqrt(3)
Zhao et al. (2023)	Largemouth bass (Micropterus salmoides)	Tributyrin or alanyl-glutamine supplementation in a diet containing 41.3% soybean meal and 35% fishmeal	TB0.1 (SM diet + 0.1% tributyrin)	SM (41.3% soybean-meal negative-control diet)	Midgut mucosal thickness (µm)	898.38 +/- 10.76	598.25 +/- 54.33	3	3	Hedges' g	6.13	1.69 to 10.57	Higher, more favorable value with supplementation; unadjusted 95% CI excluded 0	Not applicable - reported SE converted to SD as SE x sqrt(3)
Zhao et al. (2023)	Largemouth bass (Micropterus salmoides)	Tributyrin or alanyl-glutamine supplementation in a diet containing 41.3% soybean meal and 35% fishmeal	TB0.1 (SM diet + 0.1% tributyrin)	SM (41.3% soybean-meal negative-control diet)	Hindgut villus height (µm)	730.90 +/- 39.79	509.46 +/- 8.07	3	3	Hedges' g	6.17	1.71 to 10.64	Higher, more favorable value with supplementation; unadjusted 95% CI excluded 0	Not applicable - reported SE converted to SD as SE x sqrt(3)
Zhao et al. (2023)	Largemouth bass (Micropterus salmoides)	Tributyrin or alanyl-glutamine supplementation in a diet containing 41.3% soybean meal and 35% fishmeal	TB0.1 (SM diet + 0.1% tributyrin)	SM (41.3% soybean-meal negative-control diet)	Hindgut crypt depth (µm)	34.47 +/- 2.23	53.25 +/- 5.42	3	3	Hedges' g (direction reversed)	3.62	0.80 to 6.44	Lower, more favorable value with supplementation; unadjusted 95% CI excluded 0	Not applicable - reported SE converted to SD as SE x sqrt(3)
Zhao et al. (2023)	Largemouth bass (Micropterus salmoides)	Tributyrin or alanyl-glutamine supplementation in a diet containing 41.3% soybean meal and 35% fishmeal	TB0.1 (SM diet + 0.1% tributyrin)	SM (41.3% soybean-meal negative-control diet)	Hindgut mucosal thickness (µm)	768.20 +/- 41.21	559.31 +/- 4.33	3	3	Hedges' g	5.7	1.55 to 9.86	Higher, more favorable value with supplementation; unadjusted 95% CI excluded 0	Not applicable - reported SE converted to SD as SE x sqrt(3)
Zhao et al. (2023)	Largemouth bass (Micropterus salmoides)	Tributyrin or alanyl-glutamine supplementation in a diet containing 41.3% soybean meal and 35% fishmeal	TB0.2 (SM diet + 0.2% tributyrin)	SM (41.3% soybean-meal negative-control diet)	Survival rate (%)	86.67 +/- 18.93	80.00 +/- 26.47	3	3	Hedges' g	0.23	-1.06 to 1.52	Higher, more favorable value with supplementation; unadjusted 95% CI included 0	Not applicable - reported SE converted to SD as SE x sqrt(3)
Zhao et al. (2023)	Largemouth bass (Micropterus salmoides)	Tributyrin or alanyl-glutamine supplementation in a diet containing 41.3% soybean meal and 35% fishmeal	TB0.2 (SM diet + 0.2% tributyrin)	SM (41.3% soybean-meal negative-control diet)	Weight gain (%)	185.28 +/- 25.51	179.85 +/- 13.77	3	3	Hedges' g	0.21	-1.08 to 1.50	Higher, more favorable value with supplementation; unadjusted 95% CI included 0	Not applicable - reported SE converted to SD as SE x sqrt(3)
Zhao et al. (2023)	Largemouth bass (Micropterus salmoides)	Tributyrin or alanyl-glutamine supplementation in a diet containing 41.3% soybean meal and 35% fishmeal	TB0.2 (SM diet + 0.2% tributyrin)	SM (41.3% soybean-meal negative-control diet)	Specific growth rate (%/day)	1.870 +/- 0.156	1.840 +/- 0.087	3	3	Hedges' g	0.19	-1.10 to 1.48	Higher, more favorable value with supplementation; unadjusted 95% CI included 0	Not applicable - reported SE converted to SD as SE x sqrt(3)
Zhao et al. (2023)	Largemouth bass (Micropterus salmoides)	Tributyrin or alanyl-glutamine supplementation in a diet containing 41.3% soybean meal and 35% fishmeal	TB0.2 (SM diet + 0.2% tributyrin)	SM (41.3% soybean-meal negative-control diet)	Feed conversion ratio	0.900 +/- 0.104	1.300 +/- 0.225	3	3	Hedges' g (direction reversed)	1.82	0.03 to 3.62	Lower, more favorable value with supplementation; unadjusted 95% CI excluded 0	Not applicable - reported SE converted to SD as SE x sqrt(3)
Zhao et al. (2023)	Largemouth bass (Micropterus salmoides)	Tributyrin or alanyl-glutamine supplementation in a diet containing 41.3% soybean meal and 35% fishmeal	TB0.2 (SM diet + 0.2% tributyrin)	SM (41.3% soybean-meal negative-control diet)	Hepatosomatic index (%)	1.550 +/- 0.121	1.850 +/- 0.104	3	3	Hedges' g	-2.13	-4.08 to -0.17	Lower measured value with supplementation; unadjusted 95% CI excluded 0	Not applicable - reported SE converted to SD as SE x sqrt(3)
Zhao et al. (2023)	Largemouth bass (Micropterus salmoides)	Tributyrin or alanyl-glutamine supplementation in a diet containing 41.3% soybean meal and 35% fishmeal	TB0.2 (SM diet + 0.2% tributyrin)	SM (41.3% soybean-meal negative-control diet)	Viscerosomatic index (%)	7.310 +/- 0.901	7.890 +/- 0.364	3	3	Hedges' g	-0.68	-2.04 to 0.69	Lower measured value with supplementation; unadjusted 95% CI included 0	Not applicable - reported SE converted to SD as SE x sqrt(3)
Zhao et al. (2023)	Largemouth bass (Micropterus salmoides)	Tributyrin or alanyl-glutamine supplementation in a diet containing 41.3% soybean meal and 35% fishmeal	TB0.2 (SM diet + 0.2% tributyrin)	SM (41.3% soybean-meal negative-control diet)	Crude protein (%)	16.95 +/- 0.10	17.01 +/- 0.09	3	3	Hedges' g	-0.5	-1.83 to 0.82	Lower measured value with supplementation; unadjusted 95% CI included 0	Not applicable - reported SE converted to SD as SE x sqrt(3)
Zhao et al. (2023)	Largemouth bass (Micropterus salmoides)	Tributyrin or alanyl-glutamine supplementation in a diet containing 41.3% soybean meal and 35% fishmeal	TB0.2 (SM diet + 0.2% tributyrin)	SM (41.3% soybean-meal negative-control diet)	Crude lipid (%)	4.640 +/- 0.104	4.620 +/- 0.087	3	3	Hedges' g	0.17	-1.12 to 1.45	Higher measured value with supplementation; unadjusted 95% CI included 0	Not applicable - reported SE converted to SD as SE x sqrt(3)
Zhao et al. (2023)	Largemouth bass (Micropterus salmoides)	Tributyrin or alanyl-glutamine supplementation in a diet containing 41.3% soybean meal and 35% fishmeal	TB0.2 (SM diet + 0.2% tributyrin)	SM (41.3% soybean-meal negative-control diet)	Crude ash (%)	3.870 +/- 0.069	3.890 +/- 0.069	3	3	Hedges' g	-0.23	-1.52 to 1.06	Lower measured value with supplementation; unadjusted 95% CI included 0	Not applicable - reported SE converted to SD as SE x sqrt(3)
Zhao et al. (2023)	Largemouth bass (Micropterus salmoides)	Tributyrin or alanyl-glutamine supplementation in a diet containing 41.3% soybean meal and 35% fishmeal	TB0.2 (SM diet + 0.2% tributyrin)	SM (41.3% soybean-meal negative-control diet)	Moisture (%)	70.65 +/- 1.18	70.81 +/- 0.48	3	3	Hedges' g	-0.14	-1.43 to 1.14	Lower measured value with supplementation; unadjusted 95% CI included 0	Not applicable - reported SE converted to SD as SE x sqrt(3)
Zhao et al. (2023)	Largemouth bass (Micropterus salmoides)	Tributyrin or alanyl-glutamine supplementation in a diet containing 41.3% soybean meal and 35% fishmeal	TB0.2 (SM diet + 0.2% tributyrin)	SM (41.3% soybean-meal negative-control diet)	Foregut villus height (µm)	953.03 +/- 14.79	871.04 +/- 32.87	3	3	Hedges' g	2.57	0.38 to 4.77	Higher, more favorable value with supplementation; unadjusted 95% CI excluded 0	Not applicable - reported SE converted to SD as SE x sqrt(3)
Zhao et al. (2023)	Largemouth bass (Micropterus salmoides)	Tributyrin or alanyl-glutamine supplementation in a diet containing 41.3% soybean meal and 35% fishmeal	TB0.2 (SM diet + 0.2% tributyrin)	SM (41.3% soybean-meal negative-control diet)	Foregut crypt depth (µm)	21.01 +/- 1.65	41.60 +/- 4.02	3	3	Hedges' g (direction reversed)	5.36	1.43 to 9.30	Lower, more favorable value with supplementation; unadjusted 95% CI excluded 0	Not applicable - reported SE converted to SD as SE x sqrt(3)
Zhao et al. (2023)	Largemouth bass (Micropterus salmoides)	Tributyrin or alanyl-glutamine supplementation in a diet containing 41.3% soybean meal and 35% fishmeal	TB0.2 (SM diet + 0.2% tributyrin)	SM (41.3% soybean-meal negative-control diet)	Foregut mucosal thickness (µm)	977.75 +/- 15.52	915.59 +/- 28.94	3	3	Hedges' g	2.14	0.18 to 4.10	Higher, more favorable value with supplementation; unadjusted 95% CI excluded 0	Not applicable - reported SE converted to SD as SE x sqrt(3)
Zhao et al. (2023)	Largemouth bass (Micropterus salmoides)	Tributyrin or alanyl-glutamine supplementation in a diet containing 41.3% soybean meal and 35% fishmeal	TB0.2 (SM diet + 0.2% tributyrin)	SM (41.3% soybean-meal negative-control diet)	Midgut villus height (µm)	855.89 +/- 11.03	557.82 +/- 53.71	3	3	Hedges' g	6.15	1.70 to 10.60	Higher, more favorable value with supplementation; unadjusted 95% CI excluded 0	Not applicable - reported SE converted to SD as SE x sqrt(3)
Zhao et al. (2023)	Largemouth bass (Micropterus salmoides)	Tributyrin or alanyl-glutamine supplementation in a diet containing 41.3% soybean meal and 35% fishmeal	TB0.2 (SM diet + 0.2% tributyrin)	SM (41.3% soybean-meal negative-control diet)	Midgut crypt depth (µm)	36.71 +/- 1.84	42.69 +/- 1.51	3	3	Hedges' g (direction reversed)	2.85	0.50 to 5.20	Lower, more favorable value with supplementation; unadjusted 95% CI excluded 0	Not applicable - reported SE converted to SD as SE x sqrt(3)

Study	Species	Strategy	Intervention	Comparator	Outcome and unit	Intervention mean +/- SD	Comparator mean +/- SD	n intervention (tanks)	n comparator (tanks)	Effect-size metric	Effect size	95% CI	Interpretation	Reason not estimated
Zhao et al. (2023)	Largemouth bass (Micropterus salmoides)	Tributyrin or alanyl-glutamine supplementation in a diet containing 41.3% soybean meal and 35% fishmeal	TB0.2 (SM diet + 0.2% tributyrin)	SM (41.3% soybean-meal negative-control diet)	Midgut mucosal thickness (µm)	896.37 +/- 10.32	598.25 +/- 54.33	3	3	Hedges' g	6.1	1.68 to 10.51	Higher, more favorable value with supplementation; unadjusted 95% CI excluded 0	Not applicable - reported SE converted to SD as SE x sqrt(3)
Zhao et al. (2023)	Largemouth bass (Micropterus salmoides)	Tributyrin or alanyl-glutamine supplementation in a diet containing 41.3% soybean meal and 35% fishmeal	TB0.2 (SM diet + 0.2% tributyrin)	SM (41.3% soybean-meal negative-control diet)	Hindgut villus height (µm)	673.45 +/- 27.75	509.46 +/- 8.07	3	3	Hedges' g	6.42	1.79 to 11.05	Higher, more favorable value with supplementation; unadjusted 95% CI excluded 0	Not applicable - reported SE converted to SD as SE x sqrt(3)
Zhao et al. (2023)	Largemouth bass (Micropterus salmoides)	Tributyrin or alanyl-glutamine supplementation in a diet containing 41.3% soybean meal and 35% fishmeal	TB0.2 (SM diet + 0.2% tributyrin)	SM (41.3% soybean-meal negative-control diet)	Hindgut crypt depth (µm)	25.11 +/- 3.29	53.25 +/- 5.42	3	3	Hedges' g (direction reversed)	5.02	1.31 to 8.73	Lower, more favorable value with supplementation; unadjusted 95% CI excluded 0	Not applicable - reported SE converted to SD as SE x sqrt(3)
Zhao et al. (2023)	Largemouth bass (Micropterus salmoides)	Tributyrin or alanyl-glutamine supplementation in a diet containing 41.3% soybean meal and 35% fishmeal	TB0.2 (SM diet + 0.2% tributyrin)	SM (41.3% soybean-meal negative-control diet)	Hindgut mucosal thickness (µm)	729.37 +/- 32.81	559.31 +/- 4.33	3	3	Hedges' g	5.81	1.59 to 10.04	Higher, more favorable value with supplementation; unadjusted 95% CI excluded 0	Not applicable - reported SE converted to SD as SE x sqrt(3)
Zhao et al. (2023)	Largemouth bass (Micropterus salmoides)	Tributyrin or alanyl-glutamine supplementation in a diet containing 41.3% soybean meal and 35% fishmeal	AGn1 (SM diet + 1% alanyl-glutamine)	PM (34.8% peanut-meal positive-control diet)	Survival rate (%)	93.33 +/- 7.64	76.67 +/- 17.56	3	3	Hedges' g	0.98	-0.47 to 2.43	Higher, more favorable value with supplementation; unadjusted 95% CI included 0	Not applicable - reported SE converted to SD as SE x sqrt(3)
Zhao et al. (2023)	Largemouth bass (Micropterus salmoides)	Tributyrin or alanyl-glutamine supplementation in a diet containing 41.3% soybean meal and 35% fishmeal	AGn1 (SM diet + 1% alanyl-glutamine)	PM (34.8% peanut-meal positive-control diet)	Weight gain (%)	189.05 +/- 28.56	185.51 +/- 16.97	3	3	Hedges' g	0.12	-1.16 to 1.40	Higher, more favorable value with supplementation; unadjusted 95% CI included 0	Not applicable - reported SE converted to SD as SE x sqrt(3)
Zhao et al. (2023)	Largemouth bass (Micropterus salmoides)	Tributyrin or alanyl-glutamine supplementation in a diet containing 41.3% soybean meal and 35% fishmeal	AGn1 (SM diet + 1% alanyl-glutamine)	PM (34.8% peanut-meal positive-control diet)	Specific growth rate (%/day)	1.890 +/- 0.173	1.870 +/- 0.104	3	3	Hedges' g	0.11	-1.17 to 1.39	Higher, more favorable value with supplementation; unadjusted 95% CI included 0	Not applicable - reported SE converted to SD as SE x sqrt(3)
Zhao et al. (2023)	Largemouth bass (Micropterus salmoides)	Tributyrin or alanyl-glutamine supplementation in a diet containing 41.3% soybean meal and 35% fishmeal	AGn1 (SM diet + 1% alanyl-glutamine)	PM (34.8% peanut-meal positive-control diet)	Feed conversion ratio	0.990 +/- 0.052	1.170 +/- 0.208	3	3	Hedges' g (direction reversed)	0.95	-0.49 to 2.39	Lower, more favorable value with supplementation; unadjusted 95% CI included 0	Not applicable - reported SE converted to SD as SE x sqrt(3)
Zhao et al. (2023)	Largemouth bass (Micropterus salmoides)	Tributyrin or alanyl-glutamine supplementation in a diet containing 41.3% soybean meal and 35% fishmeal	AGn1 (SM diet + 1% alanyl-glutamine)	PM (34.8% peanut-meal positive-control diet)	Hepatosomatic index (%)	1.540 +/- 0.121	1.710 +/- 0.294	3	3	Hedges' g	-0.6	-1.95 to 0.74	Lower measured value with supplementation; unadjusted 95% CI included 0	Not applicable - reported SE converted to SD as SE x sqrt(3)
Zhao et al. (2023)	Largemouth bass (Micropterus salmoides)	Tributyrin or alanyl-glutamine supplementation in a diet containing 41.3% soybean meal and 35% fishmeal	AGn1 (SM diet + 1% alanyl-glutamine)	PM (34.8% peanut-meal positive-control diet)	Viscerosomatic index (%)	7.770 +/- 0.935	7.560 +/- 0.312	3	3	Hedges' g	0.24	-1.05 to 1.53	Higher measured value with supplementation; unadjusted 95% CI included 0	Not applicable - reported SE converted to SD as SE x sqrt(3)
Zhao et al. (2023)	Largemouth bass (Micropterus salmoides)	Tributyrin or alanyl-glutamine supplementation in a diet containing 41.3% soybean meal and 35% fishmeal	AGn1 (SM diet + 1% alanyl-glutamine)	PM (34.8% peanut-meal positive-control diet)	Crude protein (%)	17.00 +/- 0.16	17.24 +/- 0.05	3	3	Hedges' g	-1.65	-3.37 to 0.07	Lower measured value with supplementation; unadjusted 95% CI included 0	Not applicable - reported SE converted to SD as SE x sqrt(3)
Zhao et al. (2023)	Largemouth bass (Micropterus salmoides)	Tributyrin or alanyl-glutamine supplementation in a diet containing 41.3% soybean meal and 35% fishmeal	AGn1 (SM diet + 1% alanyl-glutamine)	PM (34.8% peanut-meal positive-control diet)	Crude lipid (%)	4.510 +/- 0.087	4.480 +/- 0.139	3	3	Hedges' g	0.21	-1.08 to 1.50	Higher measured value with supplementation; unadjusted 95% CI included 0	Not applicable - reported SE converted to SD as SE x sqrt(3)
Zhao et al. (2023)	Largemouth bass (Micropterus salmoides)	Tributyrin or alanyl-glutamine supplementation in a diet containing 41.3% soybean meal and 35% fishmeal	AGn1 (SM diet + 1% alanyl-glutamine)	PM (34.8% peanut-meal positive-control diet)	Crude ash (%)	3.890 +/- 0.069	3.860 +/- 0.052	3	3	Hedges' g	0.39	-0.92 to 1.70	Higher measured value with supplementation; unadjusted 95% CI included 0	Not applicable - reported SE converted to SD as SE x sqrt(3)
Zhao et al. (2023)	Largemouth bass (Micropterus salmoides)	Tributyrin or alanyl-glutamine supplementation in a diet containing 41.3% soybean meal and 35% fishmeal	AGn1 (SM diet + 1% alanyl-glutamine)	PM (34.8% peanut-meal positive-control diet)	Moisture (%)	70.46 +/- 1.14	70.59 +/- 1.44	3	3	Hedges' g	-0.08	-1.36 to 1.20	Lower measured value with supplementation; unadjusted 95% CI included 0	Not applicable - reported SE converted to SD as SE x sqrt(3)
Zhao et al. (2023)	Largemouth bass (Micropterus salmoides)	Tributyrin or alanyl-glutamine supplementation in a diet containing 41.3% soybean meal and 35% fishmeal	AGn1 (SM diet + 1% alanyl-glutamine)	PM (34.8% peanut-meal positive-control diet)	Foregut villus height (µm)	1037.50 +/- 3.95	1015.58 +/- 1.92	3	3	Hedges' g	5.65	1.53 to 9.76	Higher, more favorable value with supplementation; unadjusted 95% CI excluded 0	Not applicable - reported SE converted to SD as SE x sqrt(3)
Zhao et al. (2023)	Largemouth bass (Micropterus salmoides)	Tributyrin or alanyl-glutamine supplementation in a diet containing 41.3% soybean meal and 35% fishmeal	AGn1 (SM diet + 1% alanyl-glutamine)	PM (34.8% peanut-meal positive-control diet)	Foregut crypt depth (µm)	28.20 +/- 1.39	30.66 +/- 1.66	3	3	Hedges' g (direction reversed)	1.29	-0.27 to 2.85	Lower, more favorable value with supplementation; unadjusted 95% CI included 0	Not applicable - reported SE converted to SD as SE x sqrt(3)
Zhao et al. (2023)	Largemouth bass (Micropterus salmoides)	Tributyrin or alanyl-glutamine supplementation in a diet containing 41.3% soybean meal and 35% fishmeal	AGn1 (SM diet + 1% alanyl-glutamine)	PM (34.8% peanut-meal positive-control diet)	Foregut mucosal thickness (µm)	1068.34 +/- 4.50	1049.49 +/- 1.59	3	3	Hedges' g	4.46	1.12 to 7.81	Higher, more favorable value with supplementation; unadjusted 95% CI excluded 0	Not applicable - reported SE converted to SD as SE x sqrt(3)
Zhao et al. (2023)	Largemouth bass (Micropterus salmoides)	Tributyrin or alanyl-glutamine supplementation in a diet containing 41.3% soybean meal and 35% fishmeal	AGn1 (SM diet + 1% alanyl-glutamine)	PM (34.8% peanut-meal positive-control diet)	Midgut villus height (µm)	765.88 +/- 9.51	855.50 +/- 37.38	3	3	Hedges' g	-2.63	-4.86 to -0.40	Lower, less favorable value with supplementation; unadjusted 95% CI excluded 0	Not applicable - reported SE converted to SD as SE x sqrt(3)
Zhao et al. (2023)	Largemouth bass (Micropterus salmoides)	Tributyrin or alanyl-glutamine supplementation in a diet containing 41.3% soybean meal and 35% fishmeal	AGn1 (SM diet + 1% alanyl-glutamine)	PM (34.8% peanut-meal positive-control diet)	Midgut crypt depth (µm)	30.37 +/- 2.29	47.06 +/- 2.37	3	3	Hedges' g (direction reversed)	5.73	1.56 to 9.90	Lower, more favorable value with supplementation; unadjusted 95% CI excluded 0	Not applicable - reported SE converted to SD as SE x sqrt(3)
Zhao et al. (2023)	Largemouth bass (Micropterus salmoides)	Tributyrin or alanyl-glutamine supplementation in a diet containing 41.3% soybean meal and 35% fishmeal	AGn1 (SM diet + 1% alanyl-glutamine)	PM (34.8% peanut-meal positive-control diet)	Midgut mucosal thickness (µm)	798.91 +/- 11.02	900.77 +/- 37.36	3	3	Hedges' g	-2.96	-5.38 to -0.54	Lower, less favorable value with supplementation; unadjusted 95% CI excluded 0	Not applicable - reported SE converted to SD as SE x sqrt(3)
Zhao et al. (2023)	Largemouth bass (Micropterus salmoides)	Tributyrin or alanyl-glutamine supplementation in a diet containing 41.3% soybean meal and 35% fishmeal	AGn1 (SM diet + 1% alanyl-glutamine)	PM (34.8% peanut-meal positive-control diet)	Hindgut villus height (µm)	654.64 +/- 23.24	547.99 +/- 2.88	3	3	Hedges' g	5.15	1.36 to 8.94	Higher, more favorable value with supplementation; unadjusted 95% CI excluded 0	Not applicable - reported SE converted to SD as SE x sqrt(3)
Zhao et al. (2023)	Largemouth bass (Micropterus salmoides)	Tributyrin or alanyl-glutamine supplementation in a diet containing 41.3% soybean meal and 35% fishmeal	AGn1 (SM diet + 1% alanyl-glutamine)	PM (34.8% peanut-meal positive-control diet)	Hindgut crypt depth (µm)	47.90 +/- 4.40	51.52 +/- 5.07	3	3	Hedges' g (direction reversed)	0.61	-0.74 to 1.96	Lower, more favorable value with supplementation; unadjusted 95% CI included 0	Not applicable - reported SE converted to SD as SE x sqrt(3)
Zhao et al. (2023)	Largemouth bass (Micropterus salmoides)	Tributyrin or alanyl-glutamine supplementation in a diet containing 41.3% soybean meal and 35% fishmeal	AGn1 (SM diet + 1% alanyl-glutamine)	PM (34.8% peanut-meal positive-control diet)	Hindgut mucosal thickness (µm)	705.28 +/- 27.59	578.56 +/- 1.68	3	3	Hedges' g	5.19	1.37 to 9.00	Higher, more favorable value with supplementation; unadjusted 95% CI excluded 0	Not applicable - reported SE converted to SD as SE x sqrt(3)
Zhao et al. (2023)	Largemouth bass (Micropterus salmoides)	Tributyrin or alanyl-glutamine supplementation in a diet containing 41.3% soybean meal and 35% fishmeal	AGn2 (SM diet + 2% alanyl-glutamine)	PM (34.8% peanut-meal positive-control diet)	Survival rate (%)	100.00 +/- 0.00	76.67 +/- 17.56	3	3	Hedges' g	1.5	-0.15 to 3.15	Higher, more favorable value with supplementation; unadjusted 95% CI included 0	Not applicable - reported SE converted to SD as SE x sqrt(3)

Study	Species	Strategy	Intervention	Comparator	Outcome and unit	Intervention mean +/- SD	Comparator mean +/- SD	n intervention (tanks)	n comparator (tanks)	Effect-size metric	Effect size	95% CI	Interpretation	Reason not estimated
Zhao et al. (2023)	Largemouth bass (Micropterus salmoides)	Tributyrin or alanyl-glutamine supplementation in a diet containing 41.3% soybean meal and 35% fishmeal	AGn2 (SM diet + 2% alanyl-glutamine)	PM (34.8% peanut-meal positive-control diet)	Weight gain (%)	212.70 +/- 20.06	185.51 +/- 16.97	3	3	Hedges' g	1.17	-0.34 to 2.69	Higher, more favorable value with supplementation; unadjusted 95% CI included 0	Not applicable - reported SE converted to SD as SE x sqrt(3)
Zhao et al. (2023)	Largemouth bass (Micropterus salmoides)	Tributyrin or alanyl-glutamine supplementation in a diet containing 41.3% soybean meal and 35% fishmeal	AGn2 (SM diet + 2% alanyl-glutamine)	PM (34.8% peanut-meal positive-control diet)	Specific growth rate (%/day)	2.000 +/- 0.069	1.870 +/- 0.104	3	3	Hedges' g	1.18	-0.34 to 2.70	Higher, more favorable value with supplementation; unadjusted 95% CI included 0	Not applicable - reported SE converted to SD as SE x sqrt(3)
Zhao et al. (2023)	Largemouth bass (Micropterus salmoides)	Tributyrin or alanyl-glutamine supplementation in a diet containing 41.3% soybean meal and 35% fishmeal	AGn2 (SM diet + 2% alanyl-glutamine)	PM (34.8% peanut-meal positive-control diet)	Feed conversion ratio	0.880 +/- 0.139	1.170 +/- 0.208	3	3	Hedges' g (direction reversed)	1.31	-0.26 to 2.88	Lower, more favorable value with supplementation; unadjusted 95% CI included 0	Not applicable - reported SE converted to SD as SE x sqrt(3)
Zhao et al. (2023)	Largemouth bass (Micropterus salmoides)	Tributyrin or alanyl-glutamine supplementation in a diet containing 41.3% soybean meal and 35% fishmeal	AGn2 (SM diet + 2% alanyl-glutamine)	PM (34.8% peanut-meal positive-control diet)	Hepatosomatic index (%)	1.050 +/- 0.450	1.710 +/- 0.294	3	3	Hedges' g	-1.39	-2.99 to 0.21	Lower measured value with supplementation; unadjusted 95% CI included 0	Not applicable - reported SE converted to SD as SE x sqrt(3)
Zhao et al. (2023)	Largemouth bass (Micropterus salmoides)	Tributyrin or alanyl-glutamine supplementation in a diet containing 41.3% soybean meal and 35% fishmeal	AGn2 (SM diet + 2% alanyl-glutamine)	PM (34.8% peanut-meal positive-control diet)	Viscerosomatic index (%)	6.460 +/- 0.849	7.560 +/- 0.312	3	3	Hedges' g	-1.38	-2.97 to 0.22	Lower measured value with supplementation; unadjusted 95% CI included 0	Not applicable - reported SE converted to SD as SE x sqrt(3)
Zhao et al. (2023)	Largemouth bass (Micropterus salmoides)	Tributyrin or alanyl-glutamine supplementation in a diet containing 41.3% soybean meal and 35% fishmeal	AGn2 (SM diet + 2% alanyl-glutamine)	PM (34.8% peanut-meal positive-control diet)	Crude protein (%)	17.09 +/- 0.19	17.24 +/- 0.05	3	3	Hedges' g	-0.86	-2.27 to 0.55	Lower measured value with supplementation; unadjusted 95% CI included 0	Not applicable - reported SE converted to SD as SE x sqrt(3)
Zhao et al. (2023)	Largemouth bass (Micropterus salmoides)	Tributyrin or alanyl-glutamine supplementation in a diet containing 41.3% soybean meal and 35% fishmeal	AGn2 (SM diet + 2% alanyl-glutamine)	PM (34.8% peanut-meal positive-control diet)	Crude lipid (%)	4.550 +/- 0.104	4.480 +/- 0.139	3	3	Hedges' g	0.46	-0.86 to 1.78	Higher measured value with supplementation; unadjusted 95% CI included 0	Not applicable - reported SE converted to SD as SE x sqrt(3)
Zhao et al. (2023)	Largemouth bass (Micropterus salmoides)	Tributyrin or alanyl-glutamine supplementation in a diet containing 41.3% soybean meal and 35% fishmeal	AGn2 (SM diet + 2% alanyl-glutamine)	PM (34.8% peanut-meal positive-control diet)	Crude ash (%)	3.950 +/- 0.035	3.860 +/- 0.052	3	3	Hedges' g	1.63	-0.08 to 3.34	Higher measured value with supplementation; unadjusted 95% CI included 0	Not applicable - reported SE converted to SD as SE x sqrt(3)
Zhao et al. (2023)	Largemouth bass (Micropterus salmoides)	Tributyrin or alanyl-glutamine supplementation in a diet containing 41.3% soybean meal and 35% fishmeal	AGn2 (SM diet + 2% alanyl-glutamine)	PM (34.8% peanut-meal positive-control diet)	Moisture (%)	70.50 +/- 1.04	70.59 +/- 1.44	3	3	Hedges' g	-0.06	-1.34 to 1.22	Lower measured value with supplementation; unadjusted 95% CI included 0	Not applicable - reported SE converted to SD as SE x sqrt(3)
Zhao et al. (2023)	Largemouth bass (Micropterus salmoides)	Tributyrin or alanyl-glutamine supplementation in a diet containing 41.3% soybean meal and 35% fishmeal	AGn2 (SM diet + 2% alanyl-glutamine)	PM (34.8% peanut-meal positive-control diet)	Foregut villus height (µm)	1020.00 +/- 2.81	1015.58 +/- 1.92	3	3	Hedges' g	1.47	-0.17 to 3.11	Higher, more favorable value with supplementation; unadjusted 95% CI included 0	Not applicable - reported SE converted to SD as SE x sqrt(3)
Zhao et al. (2023)	Largemouth bass (Micropterus salmoides)	Tributyrin or alanyl-glutamine supplementation in a diet containing 41.3% soybean meal and 35% fishmeal	AGn2 (SM diet + 2% alanyl-glutamine)	PM (34.8% peanut-meal positive-control diet)	Foregut crypt depth (µm)	26.28 +/- 1.78	30.66 +/- 1.66	3	3	Hedges' g (direction reversed)	2.03	0.13 to 3.94	Lower, more favorable value with supplementation; unadjusted 95% CI excluded 0	Not applicable - reported SE converted to SD as SE x sqrt(3)
Zhao et al. (2023)	Largemouth bass (Micropterus salmoides)	Tributyrin or alanyl-glutamine supplementation in a diet containing 41.3% soybean meal and 35% fishmeal	AGn2 (SM diet + 2% alanyl-glutamine)	PM (34.8% peanut-meal positive-control diet)	Foregut mucosal thickness (µm)	1048.83 +/- 1.58	1049.49 +/- 1.59	3	3	Hedges' g	-0.33	-1.63 to 0.97	Lower, less favorable value with supplementation; unadjusted 95% CI included 0	Not applicable - reported SE converted to SD as SE x sqrt(3)
Zhao et al. (2023)	Largemouth bass (Micropterus salmoides)	Tributyrin or alanyl-glutamine supplementation in a diet containing 41.3% soybean meal and 35% fishmeal	AGn2 (SM diet + 2% alanyl-glutamine)	PM (34.8% peanut-meal positive-control diet)	Midgut villus height (µm)	1001.75 +/- 42.61	855.50 +/- 37.38	3	3	Hedges' g	2.92	0.53 to 5.31	Higher, more favorable value with supplementation; unadjusted 95% CI excluded 0	Not applicable - reported SE converted to SD as SE x sqrt(3)
Zhao et al. (2023)	Largemouth bass (Micropterus salmoides)	Tributyrin or alanyl-glutamine supplementation in a diet containing 41.3% soybean meal and 35% fishmeal	AGn2 (SM diet + 2% alanyl-glutamine)	PM (34.8% peanut-meal positive-control diet)	Midgut crypt depth (µm)	25.11 +/- 3.29	47.06 +/- 2.37	3	3	Hedges' g (direction reversed)	6.12	1.69 to 10.55	Lower, more favorable value with supplementation; unadjusted 95% CI excluded 0	Not applicable - reported SE converted to SD as SE x sqrt(3)
Zhao et al. (2023)	Largemouth bass (Micropterus salmoides)	Tributyrin or alanyl-glutamine supplementation in a diet containing 41.3% soybean meal and 35% fishmeal	AGn2 (SM diet + 2% alanyl-glutamine)	PM (34.8% peanut-meal positive-control diet)	Midgut mucosal thickness (µm)	1052.34 +/- 44.05	900.77 +/- 37.36	3	3	Hedges' g	2.97	0.55 to 5.39	Higher, more favorable value with supplementation; unadjusted 95% CI excluded 0	Not applicable - reported SE converted to SD as SE x sqrt(3)
Zhao et al. (2023)	Largemouth bass (Micropterus salmoides)	Tributyrin or alanyl-glutamine supplementation in a diet containing 41.3% soybean meal and 35% fishmeal	AGn2 (SM diet + 2% alanyl-glutamine)	PM (34.8% peanut-meal positive-control diet)	Hindgut villus height (µm)	734.28 +/- 40.79	547.99 +/- 2.88	3	3	Hedges' g	5.15	1.36 to 8.95	Higher, more favorable value with supplementation; unadjusted 95% CI excluded 0	Not applicable - reported SE converted to SD as SE x sqrt(3)
Zhao et al. (2023)	Largemouth bass (Micropterus salmoides)	Tributyrin or alanyl-glutamine supplementation in a diet containing 41.3% soybean meal and 35% fishmeal	AGn2 (SM diet + 2% alanyl-glutamine)	PM (34.8% peanut-meal positive-control diet)	Hindgut crypt depth (µm)	27.47 +/- 1.82	51.52 +/- 5.07	3	3	Hedges' g (direction reversed)	5.05	1.32 to 8.77	Lower, more favorable value with supplementation; unadjusted 95% CI excluded 0	Not applicable - reported SE converted to SD as SE x sqrt(3)
Zhao et al. (2023)	Largemouth bass (Micropterus salmoides)	Tributyrin or alanyl-glutamine supplementation in a diet containing 41.3% soybean meal and 35% fishmeal	AGn2 (SM diet + 2% alanyl-glutamine)	PM (34.8% peanut-meal positive-control diet)	Hindgut mucosal thickness (µm)	788.92 +/- 45.69	578.56 +/- 1.68	3	3	Hedges' g	5.21	1.38 to 9.03	Higher, more favorable value with supplementation; unadjusted 95% CI excluded 0	Not applicable - reported SE converted to SD as SE x sqrt(3)
Zhao et al. (2023)	Largemouth bass (Micropterus salmoides)	Tributyrin or alanyl-glutamine supplementation in a diet containing 41.3% soybean meal and 35% fishmeal	TB0.1 (SM diet + 0.1% tributyrin)	PM (34.8% peanut-meal positive-control diet)	Survival rate (%)	80.00 +/- 30.41	76.67 +/- 17.56	3	3	Hedges' g	0.11	-1.18 to 1.39	Higher, more favorable value with supplementation; unadjusted 95% CI included 0	Not applicable - reported SE converted to SD as SE x sqrt(3)
Zhao et al. (2023)	Largemouth bass (Micropterus salmoides)	Tributyrin or alanyl-glutamine supplementation in a diet containing 41.3% soybean meal and 35% fishmeal	TB0.1 (SM diet + 0.1% tributyrin)	PM (34.8% peanut-meal positive-control diet)	Weight gain (%)	164.22 +/- 24.99	185.51 +/- 16.97	3	3	Hedges' g	-0.8	-2.19 to 0.60	Lower, less favorable value with supplementation; unadjusted 95% CI included 0	Not applicable - reported SE converted to SD as SE x sqrt(3)
Zhao et al. (2023)	Largemouth bass (Micropterus salmoides)	Tributyrin or alanyl-glutamine supplementation in a diet containing 41.3% soybean meal and 35% fishmeal	TB0.1 (SM diet + 0.1% tributyrin)	PM (34.8% peanut-meal positive-control diet)	Specific growth rate (%/day)	1.730 +/- 0.173	1.870 +/- 0.104	3	3	Hedges' g	-0.78	-2.17 to 0.61	Lower, less favorable value with supplementation; unadjusted 95% CI included 0	Not applicable - reported SE converted to SD as SE x sqrt(3)
Zhao et al. (2023)	Largemouth bass (Micropterus salmoides)	Tributyrin or alanyl-glutamine supplementation in a diet containing 41.3% soybean meal and 35% fishmeal	TB0.1 (SM diet + 0.1% tributyrin)	PM (34.8% peanut-meal positive-control diet)	Feed conversion ratio	1.610 +/- 0.554	1.170 +/- 0.208	3	3	Hedges' g (direction reversed)	-0.84	-2.25 to 0.57	Higher, less favorable value with supplementation; unadjusted 95% CI included 0	Not applicable - reported SE converted to SD as SE x sqrt(3)
Zhao et al. (2023)	Largemouth bass (Micropterus salmoides)	Tributyrin or alanyl-glutamine supplementation in a diet containing 41.3% soybean meal and 35% fishmeal	TB0.1 (SM diet + 0.1% tributyrin)	PM (34.8% peanut-meal positive-control diet)	Hepatosomatic index (%)	1.870 +/- 0.139	1.710 +/- 0.294	3	3	Hedges' g	0.56	-0.78 to 1.89	Higher measured value with supplementation; unadjusted 95% CI included 0	Not applicable - reported SE converted to SD as SE x sqrt(3)
Zhao et al. (2023)	Largemouth bass (Micropterus salmoides)	Tributyrin or alanyl-glutamine supplementation in a diet containing 41.3% soybean meal and 35% fishmeal	TB0.1 (SM diet + 0.1% tributyrin)	PM (34.8% peanut-meal positive-control diet)	Viscerosomatic index (%)	8.240 +/- 0.260	7.560 +/- 0.312	3	3	Hedges' g	1.9	0.06 to 3.73	Higher measured value with supplementation; unadjusted 95% CI excluded 0	Not applicable - reported SE converted to SD as SE x sqrt(3)

Study	Species	Strategy	Intervention	Comparator	Outcome and unit	Intervention mean +/- SD	Comparator mean +/- SD	n intervention (tanks)	n comparator (tanks)	Effect-size metric	Effect size	95% CI	Interpretation	Reason not estimated
Zhao et al. (2023)	Largemouth bass (Micropterus salmoides)	Tributyrin or alanyl-glutamine supplementation in a diet containing 41.3% soybean meal and 35% fishmeal	TB0.1 (SM diet + 0.1% tributyrin)	PM (34.8% peanut-meal positive-control diet)	Crude protein (%)	16.96 +/- 0.14	17.24 +/- 0.05	3	3	Hedges' g	-2.14	-4.10 to -0.18	Lower measured value with supplementation; unadjusted 95% CI excluded 0	Not applicable - reported SE converted to SD as SE x sqrt(3)
Zhao et al. (2023)	Largemouth bass (Micropterus salmoides)	Tributyrin or alanyl-glutamine supplementation in a diet containing 41.3% soybean meal and 35% fishmeal	TB0.1 (SM diet + 0.1% tributyrin)	PM (34.8% peanut-meal positive-control diet)	Crude lipid (%)	4.650 +/- 0.104	4.480 +/- 0.139	3	3	Hedges' g	1.11	-0.38 to 2.60	Higher measured value with supplementation; unadjusted 95% CI included 0	Not applicable - reported SE converted to SD as SE x sqrt(3)
Zhao et al. (2023)	Largemouth bass (Micropterus salmoides)	Tributyrin or alanyl-glutamine supplementation in a diet containing 41.3% soybean meal and 35% fishmeal	TB0.1 (SM diet + 0.1% tributyrin)	PM (34.8% peanut-meal positive-control diet)	Crude ash (%)	3.880 +/- 0.052	3.860 +/- 0.052	3	3	Hedges' g	0.31	-0.99 to 1.61	Higher measured value with supplementation; unadjusted 95% CI included 0	Not applicable - reported SE converted to SD as SE x sqrt(3)
Zhao et al. (2023)	Largemouth bass (Micropterus salmoides)	Tributyrin or alanyl-glutamine supplementation in a diet containing 41.3% soybean meal and 35% fishmeal	TB0.1 (SM diet + 0.1% tributyrin)	PM (34.8% peanut-meal positive-control diet)	Moisture (%)	71.05 +/- 0.80	70.59 +/- 1.44	3	3	Hedges' g	0.32	-0.98 to 1.62	Higher measured value with supplementation; unadjusted 95% CI included 0	Not applicable - reported SE converted to SD as SE x sqrt(3)
Zhao et al. (2023)	Largemouth bass (Micropterus salmoides)	Tributyrin or alanyl-glutamine supplementation in a diet containing 41.3% soybean meal and 35% fishmeal	TB0.1 (SM diet + 0.1% tributyrin)	PM (34.8% peanut-meal positive-control diet)	Foregut villus height (μm)	984.83 +/- 8.19	1015.58 +/- 1.92	3	3	Hedges' g	-4.13	-7.27 to -1.00	Lower, less favorable value with supplementation; unadjusted 95% CI excluded 0	Not applicable - reported SE converted to SD as SE x sqrt(3)
Zhao et al. (2023)	Largemouth bass (Micropterus salmoides)	Tributyrin or alanyl-glutamine supplementation in a diet containing 41.3% soybean meal and 35% fishmeal	TB0.1 (SM diet + 0.1% tributyrin)	PM (34.8% peanut-meal positive-control diet)	Foregut crypt depth (μm)	25.82 +/- 1.11	30.66 +/- 1.66	3	3	Hedges' g (direction reversed)	2.74	0.45 to 5.03	Lower, more favorable value with supplementation; unadjusted 95% CI excluded 0	Not applicable - reported SE converted to SD as SE x sqrt(3)
Zhao et al. (2023)	Largemouth bass (Micropterus salmoides)	Tributyrin or alanyl-glutamine supplementation in a diet containing 41.3% soybean meal and 35% fishmeal	TB0.1 (SM diet + 0.1% tributyrin)	PM (34.8% peanut-meal positive-control diet)	Foregut mucosal thickness (μm)	1013.56 +/- 7.81	1049.49 +/- 1.59	3	3	Hedges' g	-5.1	-8.86 to -1.34	Lower, less favorable value with supplementation; unadjusted 95% CI excluded 0	Not applicable - reported SE converted to SD as SE x sqrt(3)
Zhao et al. (2023)	Largemouth bass (Micropterus salmoides)	Tributyrin or alanyl-glutamine supplementation in a diet containing 41.3% soybean meal and 35% fishmeal	TB0.1 (SM diet + 0.1% tributyrin)	PM (34.8% peanut-meal positive-control diet)	Midgut villus height (μm)	857.00 +/- 11.33	855.50 +/- 37.38	3	3	Hedges' g	0.04	-1.24 to 1.32	Higher, more favorable value with supplementation; unadjusted 95% CI included 0	Not applicable - reported SE converted to SD as SE x sqrt(3)
Zhao et al. (2023)	Largemouth bass (Micropterus salmoides)	Tributyrin or alanyl-glutamine supplementation in a diet containing 41.3% soybean meal and 35% fishmeal	TB0.1 (SM diet + 0.1% tributyrin)	PM (34.8% peanut-meal positive-control diet)	Midgut crypt depth (μm)	38.16 +/- 1.32	47.06 +/- 2.37	3	3	Hedges' g (direction reversed)	3.71	0.84 to 6.58	Lower, more favorable value with supplementation; unadjusted 95% CI excluded 0	Not applicable - reported SE converted to SD as SE x sqrt(3)
Zhao et al. (2023)	Largemouth bass (Micropterus salmoides)	Tributyrin or alanyl-glutamine supplementation in a diet containing 41.3% soybean meal and 35% fishmeal	TB0.1 (SM diet + 0.1% tributyrin)	PM (34.8% peanut-meal positive-control diet)	Midgut mucosal thickness (μm)	898.38 +/- 10.76	900.77 +/- 37.36	3	3	Hedges' g	-0.07	-1.35 to 1.21	Lower, less favorable value with supplementation; unadjusted 95% CI included 0	Not applicable - reported SE converted to SD as SE x sqrt(3)
Zhao et al. (2023)	Largemouth bass (Micropterus salmoides)	Tributyrin or alanyl-glutamine supplementation in a diet containing 41.3% soybean meal and 35% fishmeal	TB0.1 (SM diet + 0.1% tributyrin)	PM (34.8% peanut-meal positive-control diet)	Hindgut villus height (μm)	730.90 +/- 39.79	547.99 +/- 2.88	3	3	Hedges' g	5.19	1.37 to 9.00	Higher, more favorable value with supplementation; unadjusted 95% CI excluded 0	Not applicable - reported SE converted to SD as SE x sqrt(3)
Zhao et al. (2023)	Largemouth bass (Micropterus salmoides)	Tributyrin or alanyl-glutamine supplementation in a diet containing 41.3% soybean meal and 35% fishmeal	TB0.1 (SM diet + 0.1% tributyrin)	PM (34.8% peanut-meal positive-control diet)	Hindgut crypt depth (μm)	34.47 +/- 2.23	51.52 +/- 5.07	3	3	Hedges' g (direction reversed)	3.48	0.75 to 6.21	Lower, more favorable value with supplementation; unadjusted 95% CI excluded 0	Not applicable - reported SE converted to SD as SE x sqrt(3)
Zhao et al. (2023)	Largemouth bass (Micropterus salmoides)	Tributyrin or alanyl-glutamine supplementation in a diet containing 41.3% soybean meal and 35% fishmeal	TB0.1 (SM diet + 0.1% tributyrin)	PM (34.8% peanut-meal positive-control diet)	Hindgut mucosal thickness (μm)	768.20 +/- 41.21	578.56 +/- 1.68	3	3	Hedges' g	5.2	1.38 to 9.03	Higher, more favorable value with supplementation; unadjusted 95% CI excluded 0	Not applicable - reported SE converted to SD as SE x sqrt(3)
Zhao et al. (2023)	Largemouth bass (Micropterus salmoides)	Tributyrin or alanyl-glutamine supplementation in a diet containing 41.3% soybean meal and 35% fishmeal	TB0.2 (SM diet + 0.2% tributyrin)	PM (34.8% peanut-meal positive-control diet)	Survival rate (%)	86.67 +/- 18.93	76.67 +/- 17.56	3	3	Hedges' g	0.44	-0.88 to 1.75	Higher, more favorable value with supplementation; unadjusted 95% CI included 0	Not applicable - reported SE converted to SD as SE x sqrt(3)
Zhao et al. (2023)	Largemouth bass (Micropterus salmoides)	Tributyrin or alanyl-glutamine supplementation in a diet containing 41.3% soybean meal and 35% fishmeal	TB0.2 (SM diet + 0.2% tributyrin)	PM (34.8% peanut-meal positive-control diet)	Weight gain (%)	185.28 +/- 25.51	185.51 +/- 16.97	3	3	Hedges' g	-0.01	-1.29 to 1.27	Lower, less favorable value with supplementation; unadjusted 95% CI included 0	Not applicable - reported SE converted to SD as SE x sqrt(3)
Zhao et al. (2023)	Largemouth bass (Micropterus salmoides)	Tributyrin or alanyl-glutamine supplementation in a diet containing 41.3% soybean meal and 35% fishmeal	TB0.2 (SM diet + 0.2% tributyrin)	PM (34.8% peanut-meal positive-control diet)	Specific growth rate (%/day)	1.870 +/- 0.156	1.870 +/- 0.104	3	3	Hedges' g	0.0	-1.28 to 1.28	No observed mean difference; unadjusted 95% CI included 0	Not applicable - reported SE converted to SD as SE x sqrt(3)
Zhao et al. (2023)	Largemouth bass (Micropterus salmoides)	Tributyrin or alanyl-glutamine supplementation in a diet containing 41.3% soybean meal and 35% fishmeal	TB0.2 (SM diet + 0.2% tributyrin)	PM (34.8% peanut-meal positive-control diet)	Feed conversion ratio	0.900 +/- 0.104	1.170 +/- 0.208	3	3	Hedges' g (direction reversed)	1.31	-0.26 to 2.89	Lower, more favorable value with supplementation; unadjusted 95% CI included 0	Not applicable - reported SE converted to SD as SE x sqrt(3)
Zhao et al. (2023)	Largemouth bass (Micropterus salmoides)	Tributyrin or alanyl-glutamine supplementation in a diet containing 41.3% soybean meal and 35% fishmeal	TB0.2 (SM diet + 0.2% tributyrin)	PM (34.8% peanut-meal positive-control diet)	Hepatosomatic index (%)	1.550 +/- 0.121	1.710 +/- 0.294	3	3	Hedges' g	-0.57	-1.91 to 0.77	Lower measured value with supplementation; unadjusted 95% CI included 0	Not applicable - reported SE converted to SD as SE x sqrt(3)
Zhao et al. (2023)	Largemouth bass (Micropterus salmoides)	Tributyrin or alanyl-glutamine supplementation in a diet containing 41.3% soybean meal and 35% fishmeal	TB0.2 (SM diet + 0.2% tributyrin)	PM (34.8% peanut-meal positive-control diet)	Viscerosomatic index (%)	7.310 +/- 0.901	7.560 +/- 0.312	3	3	Hedges' g	-0.3	-1.59 to 1.00	Lower measured value with supplementation; unadjusted 95% CI included 0	Not applicable - reported SE converted to SD as SE x sqrt(3)
Zhao et al. (2023)	Largemouth bass (Micropterus salmoides)	Tributyrin or alanyl-glutamine supplementation in a diet containing 41.3% soybean meal and 35% fishmeal	TB0.2 (SM diet + 0.2% tributyrin)	PM (34.8% peanut-meal positive-control diet)	Crude protein (%)	16.95 +/- 0.10	17.24 +/- 0.05	3	3	Hedges' g	-2.82	-5.16 to -0.49	Lower measured value with supplementation; unadjusted 95% CI excluded 0	Not applicable - reported SE converted to SD as SE x sqrt(3)
Zhao et al. (2023)	Largemouth bass (Micropterus salmoides)	Tributyrin or alanyl-glutamine supplementation in a diet containing 41.3% soybean meal and 35% fishmeal	TB0.2 (SM diet + 0.2% tributyrin)	PM (34.8% peanut-meal positive-control diet)	Crude lipid (%)	4.640 +/- 0.104	4.480 +/- 0.139	3	3	Hedges' g	1.05	-0.43 to 2.52	Higher measured value with supplementation; unadjusted 95% CI included 0	Not applicable - reported SE converted to SD as SE x sqrt(3)
Zhao et al. (2023)	Largemouth bass (Micropterus salmoides)	Tributyrin or alanyl-glutamine supplementation in a diet containing 41.3% soybean meal and 35% fishmeal	TB0.2 (SM diet + 0.2% tributyrin)	PM (34.8% peanut-meal positive-control diet)	Crude ash (%)	3.870 +/- 0.069	3.860 +/- 0.052	3	3	Hedges' g	0.13	-1.15 to 1.41	Higher measured value with supplementation; unadjusted 95% CI included 0	Not applicable - reported SE converted to SD as SE x sqrt(3)
Zhao et al. (2023)	Largemouth bass (Micropterus salmoides)	Tributyrin or alanyl-glutamine supplementation in a diet containing 41.3% soybean meal and 35% fishmeal	TB0.2 (SM diet + 0.2% tributyrin)	PM (34.8% peanut-meal positive-control diet)	Moisture (%)	70.65 +/- 1.18	70.59 +/- 1.44	3	3	Hedges' g	0.04	-1.24 to 1.32	Higher measured value with supplementation; unadjusted 95% CI included 0	Not applicable - reported SE converted to SD as SE x sqrt(3)
Zhao et al. (2023)	Largemouth bass (Micropterus salmoides)	Tributyrin or alanyl-glutamine supplementation in a diet containing 41.3% soybean meal and 35% fishmeal	TB0.2 (SM diet + 0.2% tributyrin)	PM (34.8% peanut-meal positive-control diet)	Foregut villus height (μm)	953.03 +/- 14.79	1015.58 +/- 1.92	3	3	Hedges' g	-4.74	-8.27 to -1.22	Lower, less favorable value with supplementation; unadjusted 95% CI excluded 0	Not applicable - reported SE converted to SD as SE x sqrt(3)

Study	Species	Strategy	Intervention	Comparator	Outcome and unit	Intervention mean +/- SD	Comparator mean +/- SD	n intervention (tanks)	n comparator (tanks)	Effect-size metric	Effect size	95% CI	Interpretation	Reason not estimated
Zhao et al. (2023)	Largemouth bass (Micropterus salmoides)	Tributyrin or alanyl-glutamine supplementation in a diet containing 41.3% soybean meal and 35% fishmeal	TB0.2 (SM diet + 0.2% tributyrin)	PM (34.8% peanut-meal positive-control diet)	Foregut crypt depth (µm)	21.01 +/- 1.65	30.66 +/- 1.66	3	3	Hedges' g (direction reversed)	4.67	1.19 to 8.15	Lower, more favorable value with supplementation; unadjusted 95% CI excluded 0	Not applicable - reported SE converted to SD as SE x sqrt(3)
Zhao et al. (2023)	Largemouth bass (Micropterus salmoides)	Tributyrin or alanyl-glutamine supplementation in a diet containing 41.3% soybean meal and 35% fishmeal	TB0.2 (SM diet + 0.2% tributyrin)	PM (34.8% peanut-meal positive-control diet)	Foregut mucosal thickness (µm)	977.75 +/- 15.52	1049.49 +/- 1.59	3	3	Hedges' g	-5.2	-9.03 to -1.38	Lower, less favorable value with supplementation; unadjusted 95% CI excluded 0	Not applicable - reported SE converted to SD as SE x sqrt(3)
Zhao et al. (2023)	Largemouth bass (Micropterus salmoides)	Tributyrin or alanyl-glutamine supplementation in a diet containing 41.3% soybean meal and 35% fishmeal	TB0.2 (SM diet + 0.2% tributyrin)	PM (34.8% peanut-meal positive-control diet)	Midgut villus height (µm)	855.89 +/- 11.03	855.50 +/- 37.38	3	3	Hedges' g	0.01	-1.27 to 1.29	Higher, more favorable value with supplementation; unadjusted 95% CI included 0	Not applicable - reported SE converted to SD as SE x sqrt(3)
Zhao et al. (2023)	Largemouth bass (Micropterus salmoides)	Tributyrin or alanyl-glutamine supplementation in a diet containing 41.3% soybean meal and 35% fishmeal	TB0.2 (SM diet + 0.2% tributyrin)	PM (34.8% peanut-meal positive-control diet)	Midgut crypt depth (µm)	36.71 +/- 1.84	47.06 +/- 2.37	3	3	Hedges' g (direction reversed)	3.9	0.91 to 6.90	Lower, more favorable value with supplementation; unadjusted 95% CI excluded 0	Not applicable - reported SE converted to SD as SE x sqrt(3)
Zhao et al. (2023)	Largemouth bass (Micropterus salmoides)	Tributyrin or alanyl-glutamine supplementation in a diet containing 41.3% soybean meal and 35% fishmeal	TB0.2 (SM diet + 0.2% tributyrin)	PM (34.8% peanut-meal positive-control diet)	Midgut mucosal thickness (µm)	896.37 +/- 10.32	900.77 +/- 37.36	3	3	Hedges' g	-0.13	-1.41 to 1.15	Lower, less favorable value with supplementation; unadjusted 95% CI included 0	Not applicable - reported SE converted to SD as SE x sqrt(3)
Zhao et al. (2023)	Largemouth bass (Micropterus salmoides)	Tributyrin or alanyl-glutamine supplementation in a diet containing 41.3% soybean meal and 35% fishmeal	TB0.2 (SM diet + 0.2% tributyrin)	PM (34.8% peanut-meal positive-control diet)	Hindgut villus height (µm)	673.45 +/- 27.75	547.99 +/- 2.88	3	3	Hedges' g	5.09	1.34 to 8.84	Higher, more favorable value with supplementation; unadjusted 95% CI excluded 0	Not applicable - reported SE converted to SD as SE x sqrt(3)
Zhao et al. (2023)	Largemouth bass (Micropterus salmoides)	Tributyrin or alanyl-glutamine supplementation in a diet containing 41.3% soybean meal and 35% fishmeal	TB0.2 (SM diet + 0.2% tributyrin)	PM (34.8% peanut-meal positive-control diet)	Hindgut crypt depth (µm)	25.11 +/- 3.29	51.52 +/- 5.07	3	3	Hedges' g (direction reversed)	4.94	1.29 to 8.59	Lower, more favorable value with supplementation; unadjusted 95% CI excluded 0	Not applicable - reported SE converted to SD as SE x sqrt(3)
Zhao et al. (2023)	Largemouth bass (Micropterus salmoides)	Tributyrin or alanyl-glutamine supplementation in a diet containing 41.3% soybean meal and 35% fishmeal	TB0.2 (SM diet + 0.2% tributyrin)	PM (34.8% peanut-meal positive-control diet)	Hindgut mucosal thickness (µm)	729.37 +/- 32.81	578.56 +/- 1.68	3	3	Hedges' g	5.19	1.37 to 9.01	Higher, more favorable value with supplementation; unadjusted 95% CI excluded 0	Not applicable - reported SE converted to SD as SE x sqrt(3)
Zhao et al. (2023)	Largemouth bass (Micropterus salmoides)	Tributyrin or alanyl-glutamine supplementation in a diet containing 41.3% soybean meal and 35% fishmeal	AGn1 (SM diet + 1% alanyl-glutamine)	SM (41.3% soybean-meal negative-control diet)	Foregut trypsin activity (U/mg protein)	Not reported numerically	Not reported numerically	3	3	Not estimable	Not estimable	Not estimable	AGn1 was reported to increase this enzyme activity relative to SM; the exact mean and SE were not printed.	Exact treatment means and SE/SD values were available only graphically in Figure 1A
Zhao et al. (2023)	Largemouth bass (Micropterus salmoides)	Tributyrin or alanyl-glutamine supplementation in a diet containing 41.3% soybean meal and 35% fishmeal	AGn1 (SM diet + 1% alanyl-glutamine)	SM (41.3% soybean-meal negative-control diet)	Midgut trypsin activity (U/mg protein)	Not reported numerically	Not reported numerically	3	3	Not estimable	Not estimable	Not estimable	AGn1 was reported to increase this enzyme activity relative to SM; the exact mean and SE were not printed.	Exact treatment means and SE/SD values were available only graphically in Figure 1A
Zhao et al. (2023)	Largemouth bass (Micropterus salmoides)	Tributyrin or alanyl-glutamine supplementation in a diet containing 41.3% soybean meal and 35% fishmeal	AGn1 (SM diet + 1% alanyl-glutamine)	SM (41.3% soybean-meal negative-control diet)	Hindgut trypsin activity (U/mg protein)	Not reported numerically	Not reported numerically	3	3	Not estimable	Not estimable	Not estimable	AGn1 was reported to increase this enzyme activity relative to SM; the exact mean and SE were not printed.	Exact treatment means and SE/SD values were available only graphically in Figure 1A
Zhao et al. (2023)	Largemouth bass (Micropterus salmoides)	Tributyrin or alanyl-glutamine supplementation in a diet containing 41.3% soybean meal and 35% fishmeal	AGn1 (SM diet + 1% alanyl-glutamine)	SM (41.3% soybean-meal negative-control diet)	Foregut lipase activity (U/mg protein)	Not reported numerically	Not reported numerically	3	3	Not estimable	Not estimable	Not estimable	AGn1 was reported to increase this enzyme activity relative to SM; the exact mean and SE were not printed.	Exact treatment means and SE/SD values were available only graphically in Figure 1B
Zhao et al. (2023)	Largemouth bass (Micropterus salmoides)	Tributyrin or alanyl-glutamine supplementation in a diet containing 41.3% soybean meal and 35% fishmeal	AGn1 (SM diet + 1% alanyl-glutamine)	SM (41.3% soybean-meal negative-control diet)	Midgut lipase activity (U/mg protein)	Not reported numerically	Not reported numerically	3	3	Not estimable	Not estimable	Not estimable	AGn1 was reported to increase this enzyme activity relative to SM; the exact mean and SE were not printed.	Exact treatment means and SE/SD values were available only graphically in Figure 1B
Zhao et al. (2023)	Largemouth bass (Micropterus salmoides)	Tributyrin or alanyl-glutamine supplementation in a diet containing 41.3% soybean meal and 35% fishmeal	AGn1 (SM diet + 1% alanyl-glutamine)	SM (41.3% soybean-meal negative-control diet)	Hindgut lipase activity (U/mg protein)	Not reported numerically	Not reported numerically	3	3	Not estimable	Not estimable	Not estimable	AGn1 was reported to increase this enzyme activity relative to SM; the exact mean and SE were not printed.	Exact treatment means and SE/SD values were available only graphically in Figure 1B
Zhao et al. (2023)	Largemouth bass (Micropterus salmoides)	Tributyrin or alanyl-glutamine supplementation in a diet containing 41.3% soybean meal and 35% fishmeal	AGn1 (SM diet + 1% alanyl-glutamine)	SM (41.3% soybean-meal negative-control diet)	Foregut amylase activity (U/mg protein)	Not reported numerically	Not reported numerically	3	3	Not estimable	Not estimable	Not estimable	AGn1 was reported to increase foregut amylase relative to SM; the exact mean and SE were not printed.	Exact treatment means and SE/SD values were available only graphically in Figure 1C
Zhao et al. (2023)	Largemouth bass (Micropterus salmoides)	Tributyrin or alanyl-glutamine supplementation in a diet containing 41.3% soybean meal and 35% fishmeal	AGn1 (SM diet + 1% alanyl-glutamine)	SM (41.3% soybean-meal negative-control diet)	Midgut amylase activity (U/mg protein)	Not reported numerically	Not reported numerically	3	3	Not estimable	Not estimable	Not estimable	AGn1 was reported to decrease this amylase activity relative to SM; the exact mean and SE were not printed.	Exact treatment means and SE/SD values were available only graphically in Figure 1C
Zhao et al. (2023)	Largemouth bass (Micropterus salmoides)	Tributyrin or alanyl-glutamine supplementation in a diet containing 41.3% soybean meal and 35% fishmeal	AGn1 (SM diet + 1% alanyl-glutamine)	SM (41.3% soybean-meal negative-control diet)	Hindgut amylase activity (U/mg protein)	Not reported numerically	Not reported numerically	3	3	Not estimable	Not estimable	Not estimable	AGn1 was reported to decrease this amylase activity relative to SM; the exact mean and SE were not printed.	Exact treatment means and SE/SD values were available only graphically in Figure 1C
Zhao et al. (2023)	Largemouth bass (Micropterus salmoides)	Tributyrin or alanyl-glutamine supplementation in a diet containing 41.3% soybean meal and 35% fishmeal	AGn1 (SM diet + 1% alanyl-glutamine)	SM (41.3% soybean-meal negative-control diet)	Intestinal 4E-BP relative expression	Not reported numerically	Not reported numerically	3	3	Not estimable	Not estimable	Not estimable	AGn1 was reported to increase this expression outcome relative to SM; the exact mean and SE were not printed.	Exact treatment means and SE/SD values were available only graphically in Figure 5A
Zhao et al. (2023)	Largemouth bass (Micropterus salmoides)	Tributyrin or alanyl-glutamine supplementation in a diet containing 41.3% soybean meal and 35% fishmeal	AGn1 (SM diet + 1% alanyl-glutamine)	SM (41.3% soybean-meal negative-control diet)	Intestinal TOR relative expression	Not reported numerically	Not reported numerically	3	3	Not estimable	Not estimable	Not estimable	AGn1 was reported to increase this expression outcome relative to SM; the exact mean and SE were not printed.	Exact treatment means and SE/SD values were available only graphically in Figure 5B
Zhao et al. (2023)	Largemouth bass (Micropterus salmoides)	Tributyrin or alanyl-glutamine supplementation in a diet containing 41.3% soybean meal and 35% fishmeal	AGn1 (SM diet + 1% alanyl-glutamine)	SM (41.3% soybean-meal negative-control diet)	Intestinal ACC relative expression	Not reported numerically	Not reported numerically	3	3	Not estimable	Not estimable	Not estimable	AGn1 was reported to decrease this expression outcome relative to SM; the exact mean and SE were not printed.	Exact treatment means and SE/SD values were available only graphically in Figure 5C
Zhao et al. (2023)	Largemouth bass (Micropterus salmoides)	Tributyrin or alanyl-glutamine supplementation in a diet containing 41.3% soybean meal and 35% fishmeal	AGn1 (SM diet + 1% alanyl-glutamine)	SM (41.3% soybean-meal negative-control diet)	Intestinal AMPK-alpha relative expression	Not reported numerically	Not reported numerically	3	3	Not estimable	Not estimable	Not estimable	No significant difference among experimental groups was reported for this outcome; the exact mean and SE were not printed.	Exact treatment means and SE/SD values were available only graphically in Figure 5D
Zhao et al. (2023)	Largemouth bass (Micropterus salmoides)	Tributyrin or alanyl-glutamine supplementation in a diet containing 41.3% soybean meal and 35% fishmeal	AGn1 (SM diet + 1% alanyl-glutamine)	SM (41.3% soybean-meal negative-control diet)	Intestinal TNF-alpha relative expression	Not reported numerically	Not reported numerically	3	3	Not estimable	Not estimable	Not estimable	AGn1 was reported to decrease this expression outcome relative to SM; the exact mean and SE were not printed.	Exact treatment means and SE/SD values were available only graphically in Figure 6A
Zhao et al. (2023)	Largemouth bass (Micropterus salmoides)	Tributyrin or alanyl-glutamine supplementation in a diet containing 41.3% soybean meal and 35% fishmeal	AGn1 (SM diet + 1% alanyl-glutamine)	SM (41.3% soybean-meal negative-control diet)	Intestinal IL-1beta relative expression	Not reported numerically	Not reported numerically	3	3	Not estimable	Not estimable	Not estimable	AGn1 was reported to decrease this expression outcome relative to SM; the exact mean and SE were not printed.	Exact treatment means and SE/SD values were available only graphically in Figure 6B
Zhao et al. (2023)	Largemouth bass (Micropterus salmoides)	Tributyrin or alanyl-glutamine supplementation in a diet containing 41.3% soybean meal and 35% fishmeal	AGn1 (SM diet + 1% alanyl-glutamine)	SM (41.3% soybean-meal negative-control diet)	Intestinal TGF-beta1 relative expression	Not reported numerically	Not reported numerically	3	3	Not estimable	Not estimable	Not estimable	No significant difference among experimental groups was reported for this outcome; the exact mean and SE were not printed.	Exact treatment means and SE/SD values were available only graphically in Figure 6C

Study	Species	Strategy	Intervention	Comparator	Outcome and unit	Intervention mean +/- SD	Comparator mean +/- SD	n intervention (tanks)	n comparator (tanks)	Effect-size metric	Effect size	95% CI	Interpretation	Reason not estimated
Zhao et al. (2023)	Largemouth bass (Micropterus salmoides)	Tributyrin or alanyl-glutamine supplementation in a diet containing 41.3% soybean meal and 35% fishmeal	AGn1 (SM diet + 1% alanyl-glutamine)	SM (41.3% soybean-meal negative-control diet)	Intestinal TGF-beta2 relative expression	Not reported numerically	Not reported numerically	3	3	Not estimable	Not estimable	Not estimable	No significant difference among experimental groups was reported for this outcome; the exact mean and SE were not printed.	Exact treatment means and SE/SD values were available only graphically in Figure 6D
Zhao et al. (2023)	Largemouth bass (Micropterus salmoides)	Tributyrin or alanyl-glutamine supplementation in a diet containing 41.3% soybean meal and 35% fishmeal	AGn1 (SM diet + 1% alanyl-glutamine)	SM (41.3% soybean-meal negative-control diet)	Intestinal caspase-3 relative expression	Not reported numerically	Not reported numerically	3	3	Not estimable	Not estimable	Not estimable	AGn1 was reported to decrease this expression outcome relative to SM; the exact mean and SE were not printed.	Exact treatment means and SE/SD values were available only graphically in Figure 7A
Zhao et al. (2023)	Largemouth bass (Micropterus salmoides)	Tributyrin or alanyl-glutamine supplementation in a diet containing 41.3% soybean meal and 35% fishmeal	AGn1 (SM diet + 1% alanyl-glutamine)	SM (41.3% soybean-meal negative-control diet)	Intestinal caspase-8 relative expression	Not reported numerically	Not reported numerically	3	3	Not estimable	Not estimable	Not estimable	AGn1 was reported to decrease this expression outcome relative to SM; the exact mean and SE were not printed.	Exact treatment means and SE/SD values were available only graphically in Figure 7B
Zhao et al. (2023)	Largemouth bass (Micropterus salmoides)	Tributyrin or alanyl-glutamine supplementation in a diet containing 41.3% soybean meal and 35% fishmeal	AGn1 (SM diet + 1% alanyl-glutamine)	SM (41.3% soybean-meal negative-control diet)	Intestinal caspase-9 relative expression	Not reported numerically	Not reported numerically	3	3	Not estimable	Not estimable	Not estimable	AGn1 was reported to decrease this expression outcome relative to SM; the exact mean and SE were not printed.	Exact treatment means and SE/SD values were available only graphically in Figure 7C
Zhao et al. (2023)	Largemouth bass (Micropterus salmoides)	Tributyrin or alanyl-glutamine supplementation in a diet containing 41.3% soybean meal and 35% fishmeal	AGn2 (SM diet + 2% alanyl-glutamine)	SM (41.3% soybean-meal negative-control diet)	Foregut trypsin activity (U/mg protein)	Not reported numerically	Not reported numerically	3	3	Not estimable	Not estimable	Not estimable	AGn2 was reported to increase this enzyme activity relative to SM; the exact mean and SE were not printed.	Exact treatment means and SE/SD values were available only graphically in Figure 1A
Zhao et al. (2023)	Largemouth bass (Micropterus salmoides)	Tributyrin or alanyl-glutamine supplementation in a diet containing 41.3% soybean meal and 35% fishmeal	AGn2 (SM diet + 2% alanyl-glutamine)	SM (41.3% soybean-meal negative-control diet)	Midgut trypsin activity (U/mg protein)	Not reported numerically	Not reported numerically	3	3	Not estimable	Not estimable	Not estimable	AGn2 was reported to increase this enzyme activity relative to SM; the exact mean and SE were not printed.	Exact treatment means and SE/SD values were available only graphically in Figure 1A
Zhao et al. (2023)	Largemouth bass (Micropterus salmoides)	Tributyrin or alanyl-glutamine supplementation in a diet containing 41.3% soybean meal and 35% fishmeal	AGn2 (SM diet + 2% alanyl-glutamine)	SM (41.3% soybean-meal negative-control diet)	Hindgut trypsin activity (U/mg protein)	Not reported numerically	Not reported numerically	3	3	Not estimable	Not estimable	Not estimable	AGn2 was reported to increase this enzyme activity relative to SM; the exact mean and SE were not printed.	Exact treatment means and SE/SD values were available only graphically in Figure 1A
Zhao et al. (2023)	Largemouth bass (Micropterus salmoides)	Tributyrin or alanyl-glutamine supplementation in a diet containing 41.3% soybean meal and 35% fishmeal	AGn2 (SM diet + 2% alanyl-glutamine)	SM (41.3% soybean-meal negative-control diet)	Foregut lipase activity (U/mg protein)	Not reported numerically	Not reported numerically	3	3	Not estimable	Not estimable	Not estimable	AGn2 was reported to increase this enzyme activity relative to SM; the exact mean and SE were not printed.	Exact treatment means and SE/SD values were available only graphically in Figure 1B
Zhao et al. (2023)	Largemouth bass (Micropterus salmoides)	Tributyrin or alanyl-glutamine supplementation in a diet containing 41.3% soybean meal and 35% fishmeal	AGn2 (SM diet + 2% alanyl-glutamine)	SM (41.3% soybean-meal negative-control diet)	Midgut lipase activity (U/mg protein)	Not reported numerically	Not reported numerically	3	3	Not estimable	Not estimable	Not estimable	AGn2 was reported to increase this enzyme activity relative to SM; the exact mean and SE were not printed.	Exact treatment means and SE/SD values were available only graphically in Figure 1B
Zhao et al. (2023)	Largemouth bass (Micropterus salmoides)	Tributyrin or alanyl-glutamine supplementation in a diet containing 41.3% soybean meal and 35% fishmeal	AGn2 (SM diet + 2% alanyl-glutamine)	SM (41.3% soybean-meal negative-control diet)	Hindgut lipase activity (U/mg protein)	Not reported numerically	Not reported numerically	3	3	Not estimable	Not estimable	Not estimable	AGn2 was reported to increase this enzyme activity relative to SM; the exact mean and SE were not printed.	Exact treatment means and SE/SD values were available only graphically in Figure 1B
Zhao et al. (2023)	Largemouth bass (Micropterus salmoides)	Tributyrin or alanyl-glutamine supplementation in a diet containing 41.3% soybean meal and 35% fishmeal	AGn2 (SM diet + 2% alanyl-glutamine)	SM (41.3% soybean-meal negative-control diet)	Foregut amylase activity (U/mg protein)	Not reported numerically	Not reported numerically	3	3	Not estimable	Not estimable	Not estimable	AGn2 was reported to increase foregut amylase relative to SM; the exact mean and SE were not printed.	Exact treatment means and SE/SD values were available only graphically in Figure 1C
Zhao et al. (2023)	Largemouth bass (Micropterus salmoides)	Tributyrin or alanyl-glutamine supplementation in a diet containing 41.3% soybean meal and 35% fishmeal	AGn2 (SM diet + 2% alanyl-glutamine)	SM (41.3% soybean-meal negative-control diet)	Midgut amylase activity (U/mg protein)	Not reported numerically	Not reported numerically	3	3	Not estimable	Not estimable	Not estimable	AGn2 was reported to decrease this amylase activity relative to SM; the exact mean and SE were not printed.	Exact treatment means and SE/SD values were available only graphically in Figure 1C
Zhao et al. (2023)	Largemouth bass (Micropterus salmoides)	Tributyrin or alanyl-glutamine supplementation in a diet containing 41.3% soybean meal and 35% fishmeal	AGn2 (SM diet + 2% alanyl-glutamine)	SM (41.3% soybean-meal negative-control diet)	Hindgut amylase activity (U/mg protein)	Not reported numerically	Not reported numerically	3	3	Not estimable	Not estimable	Not estimable	AGn2 was reported to decrease this amylase activity relative to SM; the exact mean and SE were not printed.	Exact treatment means and SE/SD values were available only graphically in Figure 1C
Zhao et al. (2023)	Largemouth bass (Micropterus salmoides)	Tributyrin or alanyl-glutamine supplementation in a diet containing 41.3% soybean meal and 35% fishmeal	AGn2 (SM diet + 2% alanyl-glutamine)	SM (41.3% soybean-meal negative-control diet)	Intestinal 4E-BP relative expression	Not reported numerically	Not reported numerically	3	3	Not estimable	Not estimable	Not estimable	AGn2 was reported to increase this expression outcome relative to SM; the exact mean and SE were not printed.	Exact treatment means and SE/SD values were available only graphically in Figure 5A
Zhao et al. (2023)	Largemouth bass (Micropterus salmoides)	Tributyrin or alanyl-glutamine supplementation in a diet containing 41.3% soybean meal and 35% fishmeal	AGn2 (SM diet + 2% alanyl-glutamine)	SM (41.3% soybean-meal negative-control diet)	Intestinal TOR relative expression	Not reported numerically	Not reported numerically	3	3	Not estimable	Not estimable	Not estimable	AGn2 was reported to increase this expression outcome relative to SM; the exact mean and SE were not printed.	Exact treatment means and SE/SD values were available only graphically in Figure 5B
Zhao et al. (2023)	Largemouth bass (Micropterus salmoides)	Tributyrin or alanyl-glutamine supplementation in a diet containing 41.3% soybean meal and 35% fishmeal	AGn2 (SM diet + 2% alanyl-glutamine)	SM (41.3% soybean-meal negative-control diet)	Intestinal ACC relative expression	Not reported numerically	Not reported numerically	3	3	Not estimable	Not estimable	Not estimable	AGn2 was reported to decrease this expression outcome relative to SM; the exact mean and SE were not printed.	Exact treatment means and SE/SD values were available only graphically in Figure 5C
Zhao et al. (2023)	Largemouth bass (Micropterus salmoides)	Tributyrin or alanyl-glutamine supplementation in a diet containing 41.3% soybean meal and 35% fishmeal	AGn2 (SM diet + 2% alanyl-glutamine)	SM (41.3% soybean-meal negative-control diet)	Intestinal AMPK-alpha relative expression	Not reported numerically	Not reported numerically	3	3	Not estimable	Not estimable	Not estimable	No significant difference among experimental groups was reported for this outcome; the exact mean and SE were not printed.	Exact treatment means and SE/SD values were available only graphically in Figure 5D
Zhao et al. (2023)	Largemouth bass (Micropterus salmoides)	Tributyrin or alanyl-glutamine supplementation in a diet containing 41.3% soybean meal and 35% fishmeal	AGn2 (SM diet + 2% alanyl-glutamine)	SM (41.3% soybean-meal negative-control diet)	Intestinal TNF-alpha relative expression	Not reported numerically	Not reported numerically	3	3	Not estimable	Not estimable	Not estimable	AGn2 was reported to decrease this expression outcome relative to SM; the exact mean and SE were not printed.	Exact treatment means and SE/SD values were available only graphically in Figure 6A
Zhao et al. (2023)	Largemouth bass (Micropterus salmoides)	Tributyrin or alanyl-glutamine supplementation in a diet containing 41.3% soybean meal and 35% fishmeal	AGn2 (SM diet + 2% alanyl-glutamine)	SM (41.3% soybean-meal negative-control diet)	Intestinal IL-1beta relative expression	Not reported numerically	Not reported numerically	3	3	Not estimable	Not estimable	Not estimable	AGn2 was reported to decrease this expression outcome relative to SM; the exact mean and SE were not printed.	Exact treatment means and SE/SD values were available only graphically in Figure 6B
Zhao et al. (2023)	Largemouth bass (Micropterus salmoides)	Tributyrin or alanyl-glutamine supplementation in a diet containing 41.3% soybean meal and 35% fishmeal	AGn2 (SM diet + 2% alanyl-glutamine)	SM (41.3% soybean-meal negative-control diet)	Intestinal TGF-beta1 relative expression	Not reported numerically	Not reported numerically	3	3	Not estimable	Not estimable	Not estimable	No significant difference among experimental groups was reported for this outcome; the exact mean and SE were not printed.	Exact treatment means and SE/SD values were available only graphically in Figure 6C
Zhao et al. (2023)	Largemouth bass (Micropterus salmoides)	Tributyrin or alanyl-glutamine supplementation in a diet containing 41.3% soybean meal and 35% fishmeal	AGn2 (SM diet + 2% alanyl-glutamine)	SM (41.3% soybean-meal negative-control diet)	Intestinal TGF-beta2 relative expression	Not reported numerically	Not reported numerically	3	3	Not estimable	Not estimable	Not estimable	No significant difference among experimental groups was reported for this outcome; the exact mean and SE were not printed.	Exact treatment means and SE/SD values were available only graphically in Figure 6D
Zhao et al. (2023)	Largemouth bass (Micropterus salmoides)	Tributyrin or alanyl-glutamine supplementation in a diet containing 41.3% soybean meal and 35% fishmeal	AGn2 (SM diet + 2% alanyl-glutamine)	SM (41.3% soybean-meal negative-control diet)	Intestinal caspase-3 relative expression	Not reported numerically	Not reported numerically	3	3	Not estimable	Not estimable	Not estimable	AGn2 was reported to decrease this expression outcome relative to SM; the exact mean and SE were not printed.	Exact treatment means and SE/SD values were available only graphically in Figure 7A
Zhao et al. (2023)	Largemouth bass (Micropterus salmoides)	Tributyrin or alanyl-glutamine supplementation in a diet containing 41.3% soybean meal and 35% fishmeal	AGn2 (SM diet + 2% alanyl-glutamine)	SM (41.3% soybean-meal negative-control diet)	Intestinal caspase-8 relative expression	Not reported numerically	Not reported numerically	3	3	Not estimable	Not estimable	Not estimable	AGn2 was reported to decrease this expression outcome relative to SM; the exact mean and SE were not printed.	Exact treatment means and SE/SD values were available only graphically in Figure 7B
Zhao et al. (2023)	Largemouth bass (Micropterus salmoides)	Tributyrin or alanyl-glutamine supplementation in a diet containing 41.3% soybean meal and 35% fishmeal	AGn2 (SM diet + 2% alanyl-glutamine)	SM (41.3% soybean-meal negative-control diet)	Intestinal caspase-9 relative expression	Not reported numerically	Not reported numerically	3	3	Not estimable	Not estimable	Not estimable	AGn2 was reported to decrease this expression outcome relative to SM; the exact mean and SE were not printed.	Exact treatment means and SE/SD values were available only graphically in Figure 7C

Study	Species	Strategy	Intervention	Comparator	Outcome and unit	Intervention mean +/- SD	Comparator mean +/- SD	n interventio n (tanks)	n comparator (tanks)	Effect-size metric	Effect size	95% CI	Interpretation	Reason not estimated
Zhao et al. (2023)	Largemouth bass (Micropterus salmoides)	Tributyrin or alanyl-glutamine supplementation in a diet containing 41.3% soybean meal and 35% fishmeal	TB0.1 (SM diet + 0.1% tributyrin)	SM (41.3% soybean-meal negative-control diet)	Foregut trypsin activity (U/mg protein)	Not reported numerically	Not reported numerically	3	3	Not estimable	Not estimable	Not estimable	TB0.1 was reported to increase this enzyme activity relative to SM; the exact mean and SE were not printed.	Exact treatment means and SE/SD values were available only graphically in Figure 1A
Zhao et al. (2023)	Largemouth bass (Micropterus salmoides)	Tributyrin or alanyl-glutamine supplementation in a diet containing 41.3% soybean meal and 35% fishmeal	TB0.1 (SM diet + 0.1% tributyrin)	SM (41.3% soybean-meal negative-control diet)	Midgut trypsin activity (U/mg protein)	Not reported numerically	Not reported numerically	3	3	Not estimable	Not estimable	Not estimable	TB0.1 was reported to increase this enzyme activity relative to SM; the exact mean and SE were not printed.	Exact treatment means and SE/SD values were available only graphically in Figure 1A
Zhao et al. (2023)	Largemouth bass (Micropterus salmoides)	Tributyrin or alanyl-glutamine supplementation in a diet containing 41.3% soybean meal and 35% fishmeal	TB0.1 (SM diet + 0.1% tributyrin)	SM (41.3% soybean-meal negative-control diet)	Hindgut trypsin activity (U/mg protein)	Not reported numerically	Not reported numerically	3	3	Not estimable	Not estimable	Not estimable	TB0.1 was reported to increase this enzyme activity relative to SM; the exact mean and SE were not printed.	Exact treatment means and SE/SD values were available only graphically in Figure 1A
Zhao et al. (2023)	Largemouth bass (Micropterus salmoides)	Tributyrin or alanyl-glutamine supplementation in a diet containing 41.3% soybean meal and 35% fishmeal	TB0.1 (SM diet + 0.1% tributyrin)	SM (41.3% soybean-meal negative-control diet)	Foregut lipase activity (U/mg protein)	Not reported numerically	Not reported numerically	3	3	Not estimable	Not estimable	Not estimable	TB0.1 was reported to increase this enzyme activity relative to SM; the exact mean and SE were not printed.	Exact treatment means and SE/SD values were available only graphically in Figure 1B
Zhao et al. (2023)	Largemouth bass (Micropterus salmoides)	Tributyrin or alanyl-glutamine supplementation in a diet containing 41.3% soybean meal and 35% fishmeal	TB0.1 (SM diet + 0.1% tributyrin)	SM (41.3% soybean-meal negative-control diet)	Midgut lipase activity (U/mg protein)	Not reported numerically	Not reported numerically	3	3	Not estimable	Not estimable	Not estimable	TB0.1 was reported to increase this enzyme activity relative to SM; the exact mean and SE were not printed.	Exact treatment means and SE/SD values were available only graphically in Figure 1B
Zhao et al. (2023)	Largemouth bass (Micropterus salmoides)	Tributyrin or alanyl-glutamine supplementation in a diet containing 41.3% soybean meal and 35% fishmeal	TB0.1 (SM diet + 0.1% tributyrin)	SM (41.3% soybean-meal negative-control diet)	Hindgut lipase activity (U/mg protein)	Not reported numerically	Not reported numerically	3	3	Not estimable	Not estimable	Not estimable	TB0.1 was reported to increase this enzyme activity relative to SM; the exact mean and SE were not printed.	Exact treatment means and SE/SD values were available only graphically in Figure 1B
Zhao et al. (2023)	Largemouth bass (Micropterus salmoides)	Tributyrin or alanyl-glutamine supplementation in a diet containing 41.3% soybean meal and 35% fishmeal	TB0.1 (SM diet + 0.1% tributyrin)	SM (41.3% soybean-meal negative-control diet)	Foregut amylase activity (U/mg protein)	Not reported numerically	Not reported numerically	3	3	Not estimable	Not estimable	Not estimable	TB0.1 was reported to increase foregut amylase relative to SM; the exact mean and SE were not printed.	Exact treatment means and SE/SD values were available only graphically in Figure 1C
Zhao et al. (2023)	Largemouth bass (Micropterus salmoides)	Tributyrin or alanyl-glutamine supplementation in a diet containing 41.3% soybean meal and 35% fishmeal	TB0.1 (SM diet + 0.1% tributyrin)	SM (41.3% soybean-meal negative-control diet)	Midgut amylase activity (U/mg protein)	Not reported numerically	Not reported numerically	3	3	Not estimable	Not estimable	Not estimable	TB0.1 was reported to decrease this amylase activity relative to SM; the exact mean and SE were not printed.	Exact treatment means and SE/SD values were available only graphically in Figure 1C
Zhao et al. (2023)	Largemouth bass (Micropterus salmoides)	Tributyrin or alanyl-glutamine supplementation in a diet containing 41.3% soybean meal and 35% fishmeal	TB0.1 (SM diet + 0.1% tributyrin)	SM (41.3% soybean-meal negative-control diet)	Hindgut amylase activity (U/mg protein)	Not reported numerically	Not reported numerically	3	3	Not estimable	Not estimable	Not estimable	TB0.1 was reported to decrease this amylase activity relative to SM; the exact mean and SE were not printed.	Exact treatment means and SE/SD values were available only graphically in Figure 1C
Zhao et al. (2023)	Largemouth bass (Micropterus salmoides)	Tributyrin or alanyl-glutamine supplementation in a diet containing 41.3% soybean meal and 35% fishmeal	TB0.1 (SM diet + 0.1% tributyrin)	SM (41.3% soybean-meal negative-control diet)	Intestinal 4E-BP relative expression	Not reported numerically	Not reported numerically	3	3	Not estimable	Not estimable	Not estimable	TB0.1 was not significantly different from SM for this expression outcome; the exact mean and SE were not printed.	Exact treatment means and SE/SD values were available only graphically in Figure 5A
Zhao et al. (2023)	Largemouth bass (Micropterus salmoides)	Tributyrin or alanyl-glutamine supplementation in a diet containing 41.3% soybean meal and 35% fishmeal	TB0.1 (SM diet + 0.1% tributyrin)	SM (41.3% soybean-meal negative-control diet)	Intestinal TOR relative expression	Not reported numerically	Not reported numerically	3	3	Not estimable	Not estimable	Not estimable	TB0.1 was not significantly different from SM for this expression outcome; the exact mean and SE were not printed.	Exact treatment means and SE/SD values were available only graphically in Figure 5B
Zhao et al. (2023)	Largemouth bass (Micropterus salmoides)	Tributyrin or alanyl-glutamine supplementation in a diet containing 41.3% soybean meal and 35% fishmeal	TB0.1 (SM diet + 0.1% tributyrin)	SM (41.3% soybean-meal negative-control diet)	Intestinal ACC relative expression	Not reported numerically	Not reported numerically	3	3	Not estimable	Not estimable	Not estimable	TB0.1 was reported to decrease this expression outcome relative to SM; the exact mean and SE were not printed.	Exact treatment means and SE/SD values were available only graphically in Figure 5C
Zhao et al. (2023)	Largemouth bass (Micropterus salmoides)	Tributyrin or alanyl-glutamine supplementation in a diet containing 41.3% soybean meal and 35% fishmeal	TB0.1 (SM diet + 0.1% tributyrin)	SM (41.3% soybean-meal negative-control diet)	Intestinal AMPK-alpha relative expression	Not reported numerically	Not reported numerically	3	3	Not estimable	Not estimable	Not estimable	No significant difference among experimental groups was reported for this outcome; the exact mean and SE were not printed.	Exact treatment means and SE/SD values were available only graphically in Figure 5D
Zhao et al. (2023)	Largemouth bass (Micropterus salmoides)	Tributyrin or alanyl-glutamine supplementation in a diet containing 41.3% soybean meal and 35% fishmeal	TB0.1 (SM diet + 0.1% tributyrin)	SM (41.3% soybean-meal negative-control diet)	Intestinal TNF-alpha relative expression	Not reported numerically	Not reported numerically	3	3	Not estimable	Not estimable	Not estimable	TB0.1 was reported to decrease this expression outcome relative to SM; the exact mean and SE were not printed.	Exact treatment means and SE/SD values were available only graphically in Figure 6A
Zhao et al. (2023)	Largemouth bass (Micropterus salmoides)	Tributyrin or alanyl-glutamine supplementation in a diet containing 41.3% soybean meal and 35% fishmeal	TB0.1 (SM diet + 0.1% tributyrin)	SM (41.3% soybean-meal negative-control diet)	Intestinal IL-1beta relative expression	Not reported numerically	Not reported numerically	3	3	Not estimable	Not estimable	Not estimable	TB0.1 was reported to decrease this expression outcome relative to SM; the exact mean and SE were not printed.	Exact treatment means and SE/SD values were available only graphically in Figure 6B
Zhao et al. (2023)	Largemouth bass (Micropterus salmoides)	Tributyrin or alanyl-glutamine supplementation in a diet containing 41.3% soybean meal and 35% fishmeal	TB0.1 (SM diet + 0.1% tributyrin)	SM (41.3% soybean-meal negative-control diet)	Intestinal TGF-beta1 relative expression	Not reported numerically	Not reported numerically	3	3	Not estimable	Not estimable	Not estimable	No significant difference among experimental groups was reported for this outcome; the exact mean and SE were not printed.	Exact treatment means and SE/SD values were available only graphically in Figure 6C
Zhao et al. (2023)	Largemouth bass (Micropterus salmoides)	Tributyrin or alanyl-glutamine supplementation in a diet containing 41.3% soybean meal and 35% fishmeal	TB0.1 (SM diet + 0.1% tributyrin)	SM (41.3% soybean-meal negative-control diet)	Intestinal TGF-beta2 relative expression	Not reported numerically	Not reported numerically	3	3	Not estimable	Not estimable	Not estimable	No significant difference among experimental groups was reported for this outcome; the exact mean and SE were not printed.	Exact treatment means and SE/SD values were available only graphically in Figure 6D
Zhao et al. (2023)	Largemouth bass (Micropterus salmoides)	Tributyrin or alanyl-glutamine supplementation in a diet containing 41.3% soybean meal and 35% fishmeal	TB0.1 (SM diet + 0.1% tributyrin)	SM (41.3% soybean-meal negative-control diet)	Intestinal caspase-3 relative expression	Not reported numerically	Not reported numerically	3	3	Not estimable	Not estimable	Not estimable	TB0.1 was reported to decrease this expression outcome relative to SM; the exact mean and SE were not printed.	Exact treatment means and SE/SD values were available only graphically in Figure 7A
Zhao et al. (2023)	Largemouth bass (Micropterus salmoides)	Tributyrin or alanyl-glutamine supplementation in a diet containing 41.3% soybean meal and 35% fishmeal	TB0.1 (SM diet + 0.1% tributyrin)	SM (41.3% soybean-meal negative-control diet)	Intestinal caspase-8 relative expression	Not reported numerically	Not reported numerically	3	3	Not estimable	Not estimable	Not estimable	TB0.1 was reported to decrease this expression outcome relative to SM; the exact mean and SE were not printed.	Exact treatment means and SE/SD values were available only graphically in Figure 7B
Zhao et al. (2023)	Largemouth bass (Micropterus salmoides)	Tributyrin or alanyl-glutamine supplementation in a diet containing 41.3% soybean meal and 35% fishmeal	TB0.1 (SM diet + 0.1% tributyrin)	SM (41.3% soybean-meal negative-control diet)	Intestinal caspase-9 relative expression	Not reported numerically	Not reported numerically	3	3	Not estimable	Not estimable	Not estimable	TB0.1 was reported to decrease this expression outcome relative to SM; the exact mean and SE were not printed.	Exact treatment means and SE/SD values were available only graphically in Figure 7C
Zhao et al. (2023)	Largemouth bass (Micropterus salmoides)	Tributyrin or alanyl-glutamine supplementation in a diet containing 41.3% soybean meal and 35% fishmeal	TB0.2 (SM diet + 0.2% tributyrin)	SM (41.3% soybean-meal negative-control diet)	Foregut trypsin activity (U/mg protein)	Not reported numerically	Not reported numerically	3	3	Not estimable	Not estimable	Not estimable	TB0.2 was reported to increase this enzyme activity relative to SM; the exact mean and SE were not printed.	Exact treatment means and SE/SD values were available only graphically in Figure 1A
Zhao et al. (2023)	Largemouth bass (Micropterus salmoides)	Tributyrin or alanyl-glutamine supplementation in a diet containing 41.3% soybean meal and 35% fishmeal	TB0.2 (SM diet + 0.2% tributyrin)	SM (41.3% soybean-meal negative-control diet)	Midgut trypsin activity (U/mg protein)	Not reported numerically	Not reported numerically	3	3	Not estimable	Not estimable	Not estimable	TB0.2 was reported to increase this enzyme activity relative to SM; the exact mean and SE were not printed.	Exact treatment means and SE/SD values were available only graphically in Figure 1A
Zhao et al. (2023)	Largemouth bass (Micropterus salmoides)	Tributyrin or alanyl-glutamine supplementation in a diet containing 41.3% soybean meal and 35% fishmeal	TB0.2 (SM diet + 0.2% tributyrin)	SM (41.3% soybean-meal negative-control diet)	Hindgut trypsin activity (U/mg protein)	Not reported numerically	Not reported numerically	3	3	Not estimable	Not estimable	Not estimable	TB0.2 was reported to increase this enzyme activity relative to SM; the exact mean and SE were not printed.	Exact treatment means and SE/SD values were available only graphically in Figure 1A
Zhao et al. (2023)	Largemouth bass (Micropterus salmoides)	Tributyrin or alanyl-glutamine supplementation in a diet containing 41.3% soybean meal and 35% fishmeal	TB0.2 (SM diet + 0.2% tributyrin)	SM (41.3% soybean-meal negative-control diet)	Foregut lipase activity (U/mg protein)	Not reported numerically	Not reported numerically	3	3	Not estimable	Not estimable	Not estimable	TB0.2 was reported to increase this enzyme activity relative to SM; the exact mean and SE were not printed.	Exact treatment means and SE/SD values were available only graphically in Figure 1B

Study	Species	Strategy	Intervention	Comparator	Outcome and unit	Intervention mean +/- SD	Comparator mean +/- SD	n intervention (tanks)	n comparator (tanks)	Effect-size metric	Effect size	95% CI	Interpretation	Reason not estimated
Zhao et al. (2023)	Largemouth bass (Micropterus salmoides)	Tributyrin or alanyl-glutamine supplementation in a diet containing 41.3% soybean meal and 35% fishmeal	TB0.2 (SM diet + 0.2% tributyrin)	SM (41.3% soybean-meal negative-control diet)	Midgut lipase activity (U/mg protein)	Not reported numerically	Not reported numerically	3	3	Not estimable	Not estimable	Not estimable	TB0.2 was reported to increase this enzyme activity relative to SM; the exact mean and SE were not printed.	Exact treatment means and SE/SD values were available only graphically in Figure 1B
Zhao et al. (2023)	Largemouth bass (Micropterus salmoides)	Tributyrin or alanyl-glutamine supplementation in a diet containing 41.3% soybean meal and 35% fishmeal	TB0.2 (SM diet + 0.2% tributyrin)	SM (41.3% soybean-meal negative-control diet)	Hindgut lipase activity (U/mg protein)	Not reported numerically	Not reported numerically	3	3	Not estimable	Not estimable	Not estimable	TB0.2 was reported to increase this enzyme activity relative to SM; the exact mean and SE were not printed.	Exact treatment means and SE/SD values were available only graphically in Figure 1B
Zhao et al. (2023)	Largemouth bass (Micropterus salmoides)	Tributyrin or alanyl-glutamine supplementation in a diet containing 41.3% soybean meal and 35% fishmeal	TB0.2 (SM diet + 0.2% tributyrin)	SM (41.3% soybean-meal negative-control diet)	Foregut amylase activity (U/mg protein)	Not reported numerically	Not reported numerically	3	3	Not estimable	Not estimable	Not estimable	TB0.2 was reported to increase foregut amylase relative to SM; the exact mean and SE were not printed.	Exact treatment means and SE/SD values were available only graphically in Figure 1C
Zhao et al. (2023)	Largemouth bass (Micropterus salmoides)	Tributyrin or alanyl-glutamine supplementation in a diet containing 41.3% soybean meal and 35% fishmeal	TB0.2 (SM diet + 0.2% tributyrin)	SM (41.3% soybean-meal negative-control diet)	Midgut amylase activity (U/mg protein)	Not reported numerically	Not reported numerically	3	3	Not estimable	Not estimable	Not estimable	TB0.2 was reported to decrease this amylase activity relative to SM; the exact mean and SE were not printed.	Exact treatment means and SE/SD values were available only graphically in Figure 1C
Zhao et al. (2023)	Largemouth bass (Micropterus salmoides)	Tributyrin or alanyl-glutamine supplementation in a diet containing 41.3% soybean meal and 35% fishmeal	TB0.2 (SM diet + 0.2% tributyrin)	SM (41.3% soybean-meal negative-control diet)	Hindgut amylase activity (U/mg protein)	Not reported numerically	Not reported numerically	3	3	Not estimable	Not estimable	Not estimable	TB0.2 was reported to decrease this amylase activity relative to SM; the exact mean and SE were not printed.	Exact treatment means and SE/SD values were available only graphically in Figure 1C
Zhao et al. (2023)	Largemouth bass (Micropterus salmoides)	Tributyrin or alanyl-glutamine supplementation in a diet containing 41.3% soybean meal and 35% fishmeal	TB0.2 (SM diet + 0.2% tributyrin)	SM (41.3% soybean-meal negative-control diet)	Intestinal 4E-BP relative expression	Not reported numerically	Not reported numerically	3	3	Not estimable	Not estimable	Not estimable	TB0.2 was reported to increase this expression outcome relative to SM; the exact mean and SE were not printed.	Exact treatment means and SE/SD values were available only graphically in Figure 5A
Zhao et al. (2023)	Largemouth bass (Micropterus salmoides)	Tributyrin or alanyl-glutamine supplementation in a diet containing 41.3% soybean meal and 35% fishmeal	TB0.2 (SM diet + 0.2% tributyrin)	SM (41.3% soybean-meal negative-control diet)	Intestinal TOR relative expression	Not reported numerically	Not reported numerically	3	3	Not estimable	Not estimable	Not estimable	TB0.2 was reported to increase this expression outcome relative to SM; the exact mean and SE were not printed.	Exact treatment means and SE/SD values were available only graphically in Figure 5B
Zhao et al. (2023)	Largemouth bass (Micropterus salmoides)	Tributyrin or alanyl-glutamine supplementation in a diet containing 41.3% soybean meal and 35% fishmeal	TB0.2 (SM diet + 0.2% tributyrin)	SM (41.3% soybean-meal negative-control diet)	Intestinal ACC relative expression	Not reported numerically	Not reported numerically	3	3	Not estimable	Not estimable	Not estimable	TB0.2 was reported to decrease this expression outcome relative to SM; the exact mean and SE were not printed.	Exact treatment means and SE/SD values were available only graphically in Figure 5C
Zhao et al. (2023)	Largemouth bass (Micropterus salmoides)	Tributyrin or alanyl-glutamine supplementation in a diet containing 41.3% soybean meal and 35% fishmeal	TB0.2 (SM diet + 0.2% tributyrin)	SM (41.3% soybean-meal negative-control diet)	Intestinal AMPK-alpha relative expression	Not reported numerically	Not reported numerically	3	3	Not estimable	Not estimable	Not estimable	No significant difference among experimental groups was reported for this outcome; the exact mean and SE were not printed.	Exact treatment means and SE/SD values were available only graphically in Figure 5D
Zhao et al. (2023)	Largemouth bass (Micropterus salmoides)	Tributyrin or alanyl-glutamine supplementation in a diet containing 41.3% soybean meal and 35% fishmeal	TB0.2 (SM diet + 0.2% tributyrin)	SM (41.3% soybean-meal negative-control diet)	Intestinal TNF-alpha relative expression	Not reported numerically	Not reported numerically	3	3	Not estimable	Not estimable	Not estimable	TB0.2 was reported to decrease this expression outcome relative to SM; the exact mean and SE were not printed.	Exact treatment means and SE/SD values were available only graphically in Figure 6A
Zhao et al. (2023)	Largemouth bass (Micropterus salmoides)	Tributyrin or alanyl-glutamine supplementation in a diet containing 41.3% soybean meal and 35% fishmeal	TB0.2 (SM diet + 0.2% tributyrin)	SM (41.3% soybean-meal negative-control diet)	Intestinal IL-1beta relative expression	Not reported numerically	Not reported numerically	3	3	Not estimable	Not estimable	Not estimable	TB0.2 was reported to decrease this expression outcome relative to SM; the exact mean and SE were not printed.	Exact treatment means and SE/SD values were available only graphically in Figure 6B
Zhao et al. (2023)	Largemouth bass (Micropterus salmoides)	Tributyrin or alanyl-glutamine supplementation in a diet containing 41.3% soybean meal and 35% fishmeal	TB0.2 (SM diet + 0.2% tributyrin)	SM (41.3% soybean-meal negative-control diet)	Intestinal TGF-beta1 relative expression	Not reported numerically	Not reported numerically	3	3	Not estimable	Not estimable	Not estimable	No significant difference among experimental groups was reported for this outcome; the exact mean and SE were not printed.	Exact treatment means and SE/SD values were available only graphically in Figure 6C
Zhao et al. (2023)	Largemouth bass (Micropterus salmoides)	Tributyrin or alanyl-glutamine supplementation in a diet containing 41.3% soybean meal and 35% fishmeal	TB0.2 (SM diet + 0.2% tributyrin)	SM (41.3% soybean-meal negative-control diet)	Intestinal TGF-beta2 relative expression	Not reported numerically	Not reported numerically	3	3	Not estimable	Not estimable	Not estimable	No significant difference among experimental groups was reported for this outcome; the exact mean and SE were not printed.	Exact treatment means and SE/SD values were available only graphically in Figure 6D
Zhao et al. (2023)	Largemouth bass (Micropterus salmoides)	Tributyrin or alanyl-glutamine supplementation in a diet containing 41.3% soybean meal and 35% fishmeal	TB0.2 (SM diet + 0.2% tributyrin)	SM (41.3% soybean-meal negative-control diet)	Intestinal caspase-3 relative expression	Not reported numerically	Not reported numerically	3	3	Not estimable	Not estimable	Not estimable	TB0.2 was reported to decrease this expression outcome relative to SM; the exact mean and SE were not printed.	Exact treatment means and SE/SD values were available only graphically in Figure 7A
Zhao et al. (2023)	Largemouth bass (Micropterus salmoides)	Tributyrin or alanyl-glutamine supplementation in a diet containing 41.3% soybean meal and 35% fishmeal	TB0.2 (SM diet + 0.2% tributyrin)	SM (41.3% soybean-meal negative-control diet)	Intestinal caspase-8 relative expression	Not reported numerically	Not reported numerically	3	3	Not estimable	Not estimable	Not estimable	TB0.2 was reported to decrease this expression outcome relative to SM; the exact mean and SE were not printed.	Exact treatment means and SE/SD values were available only graphically in Figure 7B
Zhao et al. (2023)	Largemouth bass (Micropterus salmoides)	Tributyrin or alanyl-glutamine supplementation in a diet containing 41.3% soybean meal and 35% fishmeal	TB0.2 (SM diet + 0.2% tributyrin)	SM (41.3% soybean-meal negative-control diet)	Intestinal caspase-9 relative expression	Not reported numerically	Not reported numerically	3	3	Not estimable	Not estimable	Not estimable	TB0.2 was reported to decrease this expression outcome relative to SM; the exact mean and SE were not printed.	Exact treatment means and SE/SD values were available only graphically in Figure 7C
Zhao et al. (2023)	Largemouth bass (Micropterus salmoides)	Tributyrin or alanyl-glutamine supplementation in a diet containing 41.3% soybean meal and 35% fishmeal	AGn1 (SM diet + 1% alanyl-glutamine)	PM (34.8% peanut-meal positive-control diet)	Foregut trypsin activity (U/mg protein)	Not reported numerically	Not reported numerically	3	3	Not estimable	Not estimable	Not estimable	The figure shows means, error bars, and significance lettering, but the article does not provide exact numerical values for this benchmark comparison.	Exact treatment means and SE/SD values were available only graphically in Figure 1A
Zhao et al. (2023)	Largemouth bass (Micropterus salmoides)	Tributyrin or alanyl-glutamine supplementation in a diet containing 41.3% soybean meal and 35% fishmeal	AGn1 (SM diet + 1% alanyl-glutamine)	PM (34.8% peanut-meal positive-control diet)	Midgut trypsin activity (U/mg protein)	Not reported numerically	Not reported numerically	3	3	Not estimable	Not estimable	Not estimable	The figure shows means, error bars, and significance lettering, but the article does not provide exact numerical values for this benchmark comparison.	Exact treatment means and SE/SD values were available only graphically in Figure 1A
Zhao et al. (2023)	Largemouth bass (Micropterus salmoides)	Tributyrin or alanyl-glutamine supplementation in a diet containing 41.3% soybean meal and 35% fishmeal	AGn1 (SM diet + 1% alanyl-glutamine)	PM (34.8% peanut-meal positive-control diet)	Hindgut trypsin activity (U/mg protein)	Not reported numerically	Not reported numerically	3	3	Not estimable	Not estimable	Not estimable	The figure shows means, error bars, and significance lettering, but the article does not provide exact numerical values for this benchmark comparison.	Exact treatment means and SE/SD values were available only graphically in Figure 1A
Zhao et al. (2023)	Largemouth bass (Micropterus salmoides)	Tributyrin or alanyl-glutamine supplementation in a diet containing 41.3% soybean meal and 35% fishmeal	AGn1 (SM diet + 1% alanyl-glutamine)	PM (34.8% peanut-meal positive-control diet)	Foregut lipase activity (U/mg protein)	Not reported numerically	Not reported numerically	3	3	Not estimable	Not estimable	Not estimable	The figure shows means, error bars, and significance lettering, but the article does not provide exact numerical values for this benchmark comparison.	Exact treatment means and SE/SD values were available only graphically in Figure 1B
Zhao et al. (2023)	Largemouth bass (Micropterus salmoides)	Tributyrin or alanyl-glutamine supplementation in a diet containing 41.3% soybean meal and 35% fishmeal	AGn1 (SM diet + 1% alanyl-glutamine)	PM (34.8% peanut-meal positive-control diet)	Midgut lipase activity (U/mg protein)	Not reported numerically	Not reported numerically	3	3	Not estimable	Not estimable	Not estimable	The figure shows means, error bars, and significance lettering, but the article does not provide exact numerical values for this benchmark comparison.	Exact treatment means and SE/SD values were available only graphically in Figure 1B
Zhao et al. (2023)	Largemouth bass (Micropterus salmoides)	Tributyrin or alanyl-glutamine supplementation in a diet containing 41.3% soybean meal and 35% fishmeal	AGn1 (SM diet + 1% alanyl-glutamine)	PM (34.8% peanut-meal positive-control diet)	Hindgut lipase activity (U/mg protein)	Not reported numerically	Not reported numerically	3	3	Not estimable	Not estimable	Not estimable	The figure shows means, error bars, and significance lettering, but the article does not provide exact numerical values for this benchmark comparison.	Exact treatment means and SE/SD values were available only graphically in Figure 1B
Zhao et al. (2023)	Largemouth bass (Micropterus salmoides)	Tributyrin or alanyl-glutamine supplementation in a diet containing 41.3% soybean meal and 35% fishmeal	AGn1 (SM diet + 1% alanyl-glutamine)	PM (34.8% peanut-meal positive-control diet)	Foregut amylase activity (U/mg protein)	Not reported numerically	Not reported numerically	3	3	Not estimable	Not estimable	Not estimable	The figure shows means, error bars, and significance lettering, but the article does not provide exact numerical values for this benchmark comparison.	Exact treatment means and SE/SD values were available only graphically in Figure 1C
Zhao et al. (2023)	Largemouth bass (Micropterus salmoides)	Tributyrin or alanyl-glutamine supplementation in a diet containing 41.3% soybean meal and 35% fishmeal	AGn1 (SM diet + 1% alanyl-glutamine)	PM (34.8% peanut-meal positive-control diet)	Midgut amylase activity (U/mg protein)	Not reported numerically	Not reported numerically	3	3	Not estimable	Not estimable	Not estimable	The figure shows means, error bars, and significance lettering, but the article does not provide exact numerical values for this benchmark comparison.	Exact treatment means and SE/SD values were available only graphically in Figure 1C

Study	Species	Strategy	Intervention	Comparator	Outcome and unit	Intervention mean +/- SD	Comparator mean +/- SD	n intervention (tanks)	n comparator (tanks)	Effect-size metric	Effect size	95% CI	Interpretation	Reason not estimated
Zhao et al. (2023)	Largemouth bass (Micropterus salmoides)	Tributyrin or alanyl-glutamine supplementation in a diet containing 41.3% soybean meal and 35% fishmeal	AGn1 (SM diet + 1% alanyl-glutamine)	PM (34.8% peanut-meal positive-control diet)	Hindgut amylase activity (U/mg protein)	Not reported numerically	Not reported numerically	3	3	Not estimable	Not estimable	Not estimable	The figure shows means, error bars, and significance lettering, but the article does not provide exact numerical values for this benchmark comparison.	Exact treatment means and SE/SD values were available only graphically in Figure 1C
Zhao et al. (2023)	Largemouth bass (Micropterus salmoides)	Tributyrin or alanyl-glutamine supplementation in a diet containing 41.3% soybean meal and 35% fishmeal	AGn1 (SM diet + 1% alanyl-glutamine)	PM (34.8% peanut-meal positive-control diet)	Intestinal 4E-BP relative expression	Not reported numerically	Not reported numerically	3	3	Not estimable	Not estimable	Not estimable	The figure shows means, error bars, and significance lettering, but the article does not provide exact numerical values for this benchmark comparison.	Exact treatment means and SE/SD values were available only graphically in Figure 5A
Zhao et al. (2023)	Largemouth bass (Micropterus salmoides)	Tributyrin or alanyl-glutamine supplementation in a diet containing 41.3% soybean meal and 35% fishmeal	AGn1 (SM diet + 1% alanyl-glutamine)	PM (34.8% peanut-meal positive-control diet)	Intestinal TOR relative expression	Not reported numerically	Not reported numerically	3	3	Not estimable	Not estimable	Not estimable	The figure shows means, error bars, and significance lettering, but the article does not provide exact numerical values for this benchmark comparison.	Exact treatment means and SE/SD values were available only graphically in Figure 5B
Zhao et al. (2023)	Largemouth bass (Micropterus salmoides)	Tributyrin or alanyl-glutamine supplementation in a diet containing 41.3% soybean meal and 35% fishmeal	AGn1 (SM diet + 1% alanyl-glutamine)	PM (34.8% peanut-meal positive-control diet)	Intestinal ACC relative expression	Not reported numerically	Not reported numerically	3	3	Not estimable	Not estimable	Not estimable	The figure shows means, error bars, and significance lettering, but the article does not provide exact numerical values for this benchmark comparison.	Exact treatment means and SE/SD values were available only graphically in Figure 5C
Zhao et al. (2023)	Largemouth bass (Micropterus salmoides)	Tributyrin or alanyl-glutamine supplementation in a diet containing 41.3% soybean meal and 35% fishmeal	AGn1 (SM diet + 1% alanyl-glutamine)	PM (34.8% peanut-meal positive-control diet)	Intestinal AMPK-alpha relative expression	Not reported numerically	Not reported numerically	3	3	Not estimable	Not estimable	Not estimable	The figure shows means, error bars, and significance lettering, but the article does not provide exact numerical values for this benchmark comparison.	Exact treatment means and SE/SD values were available only graphically in Figure 5D
Zhao et al. (2023)	Largemouth bass (Micropterus salmoides)	Tributyrin or alanyl-glutamine supplementation in a diet containing 41.3% soybean meal and 35% fishmeal	AGn1 (SM diet + 1% alanyl-glutamine)	PM (34.8% peanut-meal positive-control diet)	Intestinal TNF-alpha relative expression	Not reported numerically	Not reported numerically	3	3	Not estimable	Not estimable	Not estimable	The figure shows means, error bars, and significance lettering, but the article does not provide exact numerical values for this benchmark comparison.	Exact treatment means and SE/SD values were available only graphically in Figure 6A
Zhao et al. (2023)	Largemouth bass (Micropterus salmoides)	Tributyrin or alanyl-glutamine supplementation in a diet containing 41.3% soybean meal and 35% fishmeal	AGn1 (SM diet + 1% alanyl-glutamine)	PM (34.8% peanut-meal positive-control diet)	Intestinal IL-1beta relative expression	Not reported numerically	Not reported numerically	3	3	Not estimable	Not estimable	Not estimable	The figure shows means, error bars, and significance lettering, but the article does not provide exact numerical values for this benchmark comparison.	Exact treatment means and SE/SD values were available only graphically in Figure 6B
Zhao et al. (2023)	Largemouth bass (Micropterus salmoides)	Tributyrin or alanyl-glutamine supplementation in a diet containing 41.3% soybean meal and 35% fishmeal	AGn1 (SM diet + 1% alanyl-glutamine)	PM (34.8% peanut-meal positive-control diet)	Intestinal TGF-beta1 relative expression	Not reported numerically	Not reported numerically	3	3	Not estimable	Not estimable	Not estimable	The figure shows means, error bars, and significance lettering, but the article does not provide exact numerical values for this benchmark comparison.	Exact treatment means and SE/SD values were available only graphically in Figure 6C
Zhao et al. (2023)	Largemouth bass (Micropterus salmoides)	Tributyrin or alanyl-glutamine supplementation in a diet containing 41.3% soybean meal and 35% fishmeal	AGn1 (SM diet + 1% alanyl-glutamine)	PM (34.8% peanut-meal positive-control diet)	Intestinal TGF-beta2 relative expression	Not reported numerically	Not reported numerically	3	3	Not estimable	Not estimable	Not estimable	The figure shows means, error bars, and significance lettering, but the article does not provide exact numerical values for this benchmark comparison.	Exact treatment means and SE/SD values were available only graphically in Figure 6D
Zhao et al. (2023)	Largemouth bass (Micropterus salmoides)	Tributyrin or alanyl-glutamine supplementation in a diet containing 41.3% soybean meal and 35% fishmeal	AGn1 (SM diet + 1% alanyl-glutamine)	PM (34.8% peanut-meal positive-control diet)	Intestinal caspase-3 relative expression	Not reported numerically	Not reported numerically	3	3	Not estimable	Not estimable	Not estimable	The figure shows means, error bars, and significance lettering, but the article does not provide exact numerical values for this benchmark comparison.	Exact treatment means and SE/SD values were available only graphically in Figure 7A
Zhao et al. (2023)	Largemouth bass (Micropterus salmoides)	Tributyrin or alanyl-glutamine supplementation in a diet containing 41.3% soybean meal and 35% fishmeal	AGn1 (SM diet + 1% alanyl-glutamine)	PM (34.8% peanut-meal positive-control diet)	Intestinal caspase-8 relative expression	Not reported numerically	Not reported numerically	3	3	Not estimable	Not estimable	Not estimable	The figure shows means, error bars, and significance lettering, but the article does not provide exact numerical values for this benchmark comparison.	Exact treatment means and SE/SD values were available only graphically in Figure 7B
Zhao et al. (2023)	Largemouth bass (Micropterus salmoides)	Tributyrin or alanyl-glutamine supplementation in a diet containing 41.3% soybean meal and 35% fishmeal	AGn1 (SM diet + 1% alanyl-glutamine)	PM (34.8% peanut-meal positive-control diet)	Intestinal caspase-9 relative expression	Not reported numerically	Not reported numerically	3	3	Not estimable	Not estimable	Not estimable	The figure shows means, error bars, and significance lettering, but the article does not provide exact numerical values for this benchmark comparison.	Exact treatment means and SE/SD values were available only graphically in Figure 7C
Zhao et al. (2023)	Largemouth bass (Micropterus salmoides)	Tributyrin or alanyl-glutamine supplementation in a diet containing 41.3% soybean meal and 35% fishmeal	AGn2 (SM diet + 2% alanyl-glutamine)	PM (34.8% peanut-meal positive-control diet)	Foregut trypsin activity (U/mg protein)	Not reported numerically	Not reported numerically	3	3	Not estimable	Not estimable	Not estimable	The figure shows means, error bars, and significance lettering, but the article does not provide exact numerical values for this benchmark comparison.	Exact treatment means and SE/SD values were available only graphically in Figure 1A
Zhao et al. (2023)	Largemouth bass (Micropterus salmoides)	Tributyrin or alanyl-glutamine supplementation in a diet containing 41.3% soybean meal and 35% fishmeal	AGn2 (SM diet + 2% alanyl-glutamine)	PM (34.8% peanut-meal positive-control diet)	Midgut trypsin activity (U/mg protein)	Not reported numerically	Not reported numerically	3	3	Not estimable	Not estimable	Not estimable	The figure shows means, error bars, and significance lettering, but the article does not provide exact numerical values for this benchmark comparison.	Exact treatment means and SE/SD values were available only graphically in Figure 1A
Zhao et al. (2023)	Largemouth bass (Micropterus salmoides)	Tributyrin or alanyl-glutamine supplementation in a diet containing 41.3% soybean meal and 35% fishmeal	AGn2 (SM diet + 2% alanyl-glutamine)	PM (34.8% peanut-meal positive-control diet)	Hindgut trypsin activity (U/mg protein)	Not reported numerically	Not reported numerically	3	3	Not estimable	Not estimable	Not estimable	The figure shows means, error bars, and significance lettering, but the article does not provide exact numerical values for this benchmark comparison.	Exact treatment means and SE/SD values were available only graphically in Figure 1A
Zhao et al. (2023)	Largemouth bass (Micropterus salmoides)	Tributyrin or alanyl-glutamine supplementation in a diet containing 41.3% soybean meal and 35% fishmeal	AGn2 (SM diet + 2% alanyl-glutamine)	PM (34.8% peanut-meal positive-control diet)	Foregut lipase activity (U/mg protein)	Not reported numerically	Not reported numerically	3	3	Not estimable	Not estimable	Not estimable	The figure shows means, error bars, and significance lettering, but the article does not provide exact numerical values for this benchmark comparison.	Exact treatment means and SE/SD values were available only graphically in Figure 1B
Zhao et al. (2023)	Largemouth bass (Micropterus salmoides)	Tributyrin or alanyl-glutamine supplementation in a diet containing 41.3% soybean meal and 35% fishmeal	AGn2 (SM diet + 2% alanyl-glutamine)	PM (34.8% peanut-meal positive-control diet)	Midgut lipase activity (U/mg protein)	Not reported numerically	Not reported numerically	3	3	Not estimable	Not estimable	Not estimable	The figure shows means, error bars, and significance lettering, but the article does not provide exact numerical values for this benchmark comparison.	Exact treatment means and SE/SD values were available only graphically in Figure 1B
Zhao et al. (2023)	Largemouth bass (Micropterus salmoides)	Tributyrin or alanyl-glutamine supplementation in a diet containing 41.3% soybean meal and 35% fishmeal	AGn2 (SM diet + 2% alanyl-glutamine)	PM (34.8% peanut-meal positive-control diet)	Hindgut lipase activity (U/mg protein)	Not reported numerically	Not reported numerically	3	3	Not estimable	Not estimable	Not estimable	The figure shows means, error bars, and significance lettering, but the article does not provide exact numerical values for this benchmark comparison.	Exact treatment means and SE/SD values were available only graphically in Figure 1B
Zhao et al. (2023)	Largemouth bass (Micropterus salmoides)	Tributyrin or alanyl-glutamine supplementation in a diet containing 41.3% soybean meal and 35% fishmeal	AGn2 (SM diet + 2% alanyl-glutamine)	PM (34.8% peanut-meal positive-control diet)	Foregut amylase activity (U/mg protein)	Not reported numerically	Not reported numerically	3	3	Not estimable	Not estimable	Not estimable	The figure shows means, error bars, and significance lettering, but the article does not provide exact numerical values for this benchmark comparison.	Exact treatment means and SE/SD values were available only graphically in Figure 1C
Zhao et al. (2023)	Largemouth bass (Micropterus salmoides)	Tributyrin or alanyl-glutamine supplementation in a diet containing 41.3% soybean meal and 35% fishmeal	AGn2 (SM diet + 2% alanyl-glutamine)	PM (34.8% peanut-meal positive-control diet)	Midgut amylase activity (U/mg protein)	Not reported numerically	Not reported numerically	3	3	Not estimable	Not estimable	Not estimable	The figure shows means, error bars, and significance lettering, but the article does not provide exact numerical values for this benchmark comparison.	Exact treatment means and SE/SD values were available only graphically in Figure 1C
Zhao et al. (2023)	Largemouth bass (Micropterus salmoides)	Tributyrin or alanyl-glutamine supplementation in a diet containing 41.3% soybean meal and 35% fishmeal	AGn2 (SM diet + 2% alanyl-glutamine)	PM (34.8% peanut-meal positive-control diet)	Hindgut amylase activity (U/mg protein)	Not reported numerically	Not reported numerically	3	3	Not estimable	Not estimable	Not estimable	The figure shows means, error bars, and significance lettering, but the article does not provide exact numerical values for this benchmark comparison.	Exact treatment means and SE/SD values were available only graphically in Figure 1C
Zhao et al. (2023)	Largemouth bass (Micropterus salmoides)	Tributyrin or alanyl-glutamine supplementation in a diet containing 41.3% soybean meal and 35% fishmeal	AGn2 (SM diet + 2% alanyl-glutamine)	PM (34.8% peanut-meal positive-control diet)	Intestinal 4E-BP relative expression	Not reported numerically	Not reported numerically	3	3	Not estimable	Not estimable	Not estimable	The figure shows means, error bars, and significance lettering, but the article does not provide exact numerical values for this benchmark comparison.	Exact treatment means and SE/SD values were available only graphically in Figure 5A
Zhao et al. (2023)	Largemouth bass (Micropterus salmoides)	Tributyrin or alanyl-glutamine supplementation in a diet containing 41.3% soybean meal and 35% fishmeal	AGn2 (SM diet + 2% alanyl-glutamine)	PM (34.8% peanut-meal positive-control diet)	Intestinal TOR relative expression	Not reported numerically	Not reported numerically	3	3	Not estimable	Not estimable	Not estimable	The figure shows means, error bars, and significance lettering, but the article does not provide exact numerical values for this benchmark comparison.	Exact treatment means and SE/SD values were available only graphically in Figure 5B
Zhao et al. (2023)	Largemouth bass (Micropterus salmoides)	Tributyrin or alanyl-glutamine supplementation in a diet containing 41.3% soybean meal and 35% fishmeal	AGn2 (SM diet + 2% alanyl-glutamine)	PM (34.8% peanut-meal positive-control diet)	Intestinal ACC relative expression	Not reported numerically	Not reported numerically	3	3	Not estimable	Not estimable	Not estimable	The figure shows means, error bars, and significance lettering, but the article does not provide exact numerical values for this benchmark comparison.	Exact treatment means and SE/SD values were available only graphically in Figure 5C

Study	Species	Strategy	Intervention	Comparator	Outcome and unit	Intervention mean +/- SD	Comparator mean +/- SD	n intervention (tanks)	n comparator (tanks)	Effect-size metric	Effect size	95% CI	Interpretation	Reason not estimated
Zhao et al. (2023)	Largemouth bass (Micropterus salmoides)	Tributyrin or alanyl-glutamine supplementation in a diet containing 41.3% soybean meal and 35% fishmeal	AGn2 (SM diet + 2% alanyl-glutamine)	PM (34.8% peanut-meal positive-control diet)	Intestinal AMPK-alpha relative expression	Not reported numerically	Not reported numerically	3	3	Not estimable	Not estimable	Not estimable	The figure shows means, error bars, and significance lettering, but the article does not provide exact numerical values for this benchmark comparison.	Exact treatment means and SE/SD values were available only graphically in Figure 5D
Zhao et al. (2023)	Largemouth bass (Micropterus salmoides)	Tributyrin or alanyl-glutamine supplementation in a diet containing 41.3% soybean meal and 35% fishmeal	AGn2 (SM diet + 2% alanyl-glutamine)	PM (34.8% peanut-meal positive-control diet)	Intestinal TNF-alpha relative expression	Not reported numerically	Not reported numerically	3	3	Not estimable	Not estimable	Not estimable	The figure shows means, error bars, and significance lettering, but the article does not provide exact numerical values for this benchmark comparison.	Exact treatment means and SE/SD values were available only graphically in Figure 6A
Zhao et al. (2023)	Largemouth bass (Micropterus salmoides)	Tributyrin or alanyl-glutamine supplementation in a diet containing 41.3% soybean meal and 35% fishmeal	AGn2 (SM diet + 2% alanyl-glutamine)	PM (34.8% peanut-meal positive-control diet)	Intestinal IL-1beta relative expression	Not reported numerically	Not reported numerically	3	3	Not estimable	Not estimable	Not estimable	The figure shows means, error bars, and significance lettering, but the article does not provide exact numerical values for this benchmark comparison.	Exact treatment means and SE/SD values were available only graphically in Figure 6B
Zhao et al. (2023)	Largemouth bass (Micropterus salmoides)	Tributyrin or alanyl-glutamine supplementation in a diet containing 41.3% soybean meal and 35% fishmeal	AGn2 (SM diet + 2% alanyl-glutamine)	PM (34.8% peanut-meal positive-control diet)	Intestinal TGF-beta1 relative expression	Not reported numerically	Not reported numerically	3	3	Not estimable	Not estimable	Not estimable	The figure shows means, error bars, and significance lettering, but the article does not provide exact numerical values for this benchmark comparison.	Exact treatment means and SE/SD values were available only graphically in Figure 6C
Zhao et al. (2023)	Largemouth bass (Micropterus salmoides)	Tributyrin or alanyl-glutamine supplementation in a diet containing 41.3% soybean meal and 35% fishmeal	AGn2 (SM diet + 2% alanyl-glutamine)	PM (34.8% peanut-meal positive-control diet)	Intestinal TGF-beta2 relative expression	Not reported numerically	Not reported numerically	3	3	Not estimable	Not estimable	Not estimable	The figure shows means, error bars, and significance lettering, but the article does not provide exact numerical values for this benchmark comparison.	Exact treatment means and SE/SD values were available only graphically in Figure 6D
Zhao et al. (2023)	Largemouth bass (Micropterus salmoides)	Tributyrin or alanyl-glutamine supplementation in a diet containing 41.3% soybean meal and 35% fishmeal	AGn2 (SM diet + 2% alanyl-glutamine)	PM (34.8% peanut-meal positive-control diet)	Intestinal caspase-3 relative expression	Not reported numerically	Not reported numerically	3	3	Not estimable	Not estimable	Not estimable	The figure shows means, error bars, and significance lettering, but the article does not provide exact numerical values for this benchmark comparison.	Exact treatment means and SE/SD values were available only graphically in Figure 7A
Zhao et al. (2023)	Largemouth bass (Micropterus salmoides)	Tributyrin or alanyl-glutamine supplementation in a diet containing 41.3% soybean meal and 35% fishmeal	AGn2 (SM diet + 2% alanyl-glutamine)	PM (34.8% peanut-meal positive-control diet)	Intestinal caspase-8 relative expression	Not reported numerically	Not reported numerically	3	3	Not estimable	Not estimable	Not estimable	The figure shows means, error bars, and significance lettering, but the article does not provide exact numerical values for this benchmark comparison.	Exact treatment means and SE/SD values were available only graphically in Figure 7B
Zhao et al. (2023)	Largemouth bass (Micropterus salmoides)	Tributyrin or alanyl-glutamine supplementation in a diet containing 41.3% soybean meal and 35% fishmeal	AGn2 (SM diet + 2% alanyl-glutamine)	PM (34.8% peanut-meal positive-control diet)	Intestinal caspase-9 relative expression	Not reported numerically	Not reported numerically	3	3	Not estimable	Not estimable	Not estimable	The figure shows means, error bars, and significance lettering, but the article does not provide exact numerical values for this benchmark comparison.	Exact treatment means and SE/SD values were available only graphically in Figure 7C
Zhao et al. (2023)	Largemouth bass (Micropterus salmoides)	Tributyrin or alanyl-glutamine supplementation in a diet containing 41.3% soybean meal and 35% fishmeal	TB0.1 (SM diet + 0.1% tributyrin)	PM (34.8% peanut-meal positive-control diet)	Foregut trypsin activity (U/mg protein)	Not reported numerically	Not reported numerically	3	3	Not estimable	Not estimable	Not estimable	The figure shows means, error bars, and significance lettering, but the article does not provide exact numerical values for this benchmark comparison.	Exact treatment means and SE/SD values were available only graphically in Figure 1A
Zhao et al. (2023)	Largemouth bass (Micropterus salmoides)	Tributyrin or alanyl-glutamine supplementation in a diet containing 41.3% soybean meal and 35% fishmeal	TB0.1 (SM diet + 0.1% tributyrin)	PM (34.8% peanut-meal positive-control diet)	Midgut trypsin activity (U/mg protein)	Not reported numerically	Not reported numerically	3	3	Not estimable	Not estimable	Not estimable	The figure shows means, error bars, and significance lettering, but the article does not provide exact numerical values for this benchmark comparison.	Exact treatment means and SE/SD values were available only graphically in Figure 1A
Zhao et al. (2023)	Largemouth bass (Micropterus salmoides)	Tributyrin or alanyl-glutamine supplementation in a diet containing 41.3% soybean meal and 35% fishmeal	TB0.1 (SM diet + 0.1% tributyrin)	PM (34.8% peanut-meal positive-control diet)	Hindgut trypsin activity (U/mg protein)	Not reported numerically	Not reported numerically	3	3	Not estimable	Not estimable	Not estimable	The figure shows means, error bars, and significance lettering, but the article does not provide exact numerical values for this benchmark comparison.	Exact treatment means and SE/SD values were available only graphically in Figure 1A
Zhao et al. (2023)	Largemouth bass (Micropterus salmoides)	Tributyrin or alanyl-glutamine supplementation in a diet containing 41.3% soybean meal and 35% fishmeal	TB0.1 (SM diet + 0.1% tributyrin)	PM (34.8% peanut-meal positive-control diet)	Foregut lipase activity (U/mg protein)	Not reported numerically	Not reported numerically	3	3	Not estimable	Not estimable	Not estimable	The figure shows means, error bars, and significance lettering, but the article does not provide exact numerical values for this benchmark comparison.	Exact treatment means and SE/SD values were available only graphically in Figure 1B
Zhao et al. (2023)	Largemouth bass (Micropterus salmoides)	Tributyrin or alanyl-glutamine supplementation in a diet containing 41.3% soybean meal and 35% fishmeal	TB0.1 (SM diet + 0.1% tributyrin)	PM (34.8% peanut-meal positive-control diet)	Midgut lipase activity (U/mg protein)	Not reported numerically	Not reported numerically	3	3	Not estimable	Not estimable	Not estimable	The figure shows means, error bars, and significance lettering, but the article does not provide exact numerical values for this benchmark comparison.	Exact treatment means and SE/SD values were available only graphically in Figure 1B
Zhao et al. (2023)	Largemouth bass (Micropterus salmoides)	Tributyrin or alanyl-glutamine supplementation in a diet containing 41.3% soybean meal and 35% fishmeal	TB0.1 (SM diet + 0.1% tributyrin)	PM (34.8% peanut-meal positive-control diet)	Hindgut lipase activity (U/mg protein)	Not reported numerically	Not reported numerically	3	3	Not estimable	Not estimable	Not estimable	The figure shows means, error bars, and significance lettering, but the article does not provide exact numerical values for this benchmark comparison.	Exact treatment means and SE/SD values were available only graphically in Figure 1B
Zhao et al. (2023)	Largemouth bass (Micropterus salmoides)	Tributyrin or alanyl-glutamine supplementation in a diet containing 41.3% soybean meal and 35% fishmeal	TB0.1 (SM diet + 0.1% tributyrin)	PM (34.8% peanut-meal positive-control diet)	Foregut amylase activity (U/mg protein)	Not reported numerically	Not reported numerically	3	3	Not estimable	Not estimable	Not estimable	The figure shows means, error bars, and significance lettering, but the article does not provide exact numerical values for this benchmark comparison.	Exact treatment means and SE/SD values were available only graphically in Figure 1C
Zhao et al. (2023)	Largemouth bass (Micropterus salmoides)	Tributyrin or alanyl-glutamine supplementation in a diet containing 41.3% soybean meal and 35% fishmeal	TB0.1 (SM diet + 0.1% tributyrin)	PM (34.8% peanut-meal positive-control diet)	Midgut amylase activity (U/mg protein)	Not reported numerically	Not reported numerically	3	3	Not estimable	Not estimable	Not estimable	The figure shows means, error bars, and significance lettering, but the article does not provide exact numerical values for this benchmark comparison.	Exact treatment means and SE/SD values were available only graphically in Figure 1C
Zhao et al. (2023)	Largemouth bass (Micropterus salmoides)	Tributyrin or alanyl-glutamine supplementation in a diet containing 41.3% soybean meal and 35% fishmeal	TB0.1 (SM diet + 0.1% tributyrin)	PM (34.8% peanut-meal positive-control diet)	Hindgut amylase activity (U/mg protein)	Not reported numerically	Not reported numerically	3	3	Not estimable	Not estimable	Not estimable	The figure shows means, error bars, and significance lettering, but the article does not provide exact numerical values for this benchmark comparison.	Exact treatment means and SE/SD values were available only graphically in Figure 1C
Zhao et al. (2023)	Largemouth bass (Micropterus salmoides)	Tributyrin or alanyl-glutamine supplementation in a diet containing 41.3% soybean meal and 35% fishmeal	TB0.1 (SM diet + 0.1% tributyrin)	PM (34.8% peanut-meal positive-control diet)	Intestinal 4E-BP relative expression	Not reported numerically	Not reported numerically	3	3	Not estimable	Not estimable	Not estimable	The figure shows means, error bars, and significance lettering, but the article does not provide exact numerical values for this benchmark comparison.	Exact treatment means and SE/SD values were available only graphically in Figure 5A
Zhao et al. (2023)	Largemouth bass (Micropterus salmoides)	Tributyrin or alanyl-glutamine supplementation in a diet containing 41.3% soybean meal and 35% fishmeal	TB0.1 (SM diet + 0.1% tributyrin)	PM (34.8% peanut-meal positive-control diet)	Intestinal TOR relative expression	Not reported numerically	Not reported numerically	3	3	Not estimable	Not estimable	Not estimable	The figure shows means, error bars, and significance lettering, but the article does not provide exact numerical values for this benchmark comparison.	Exact treatment means and SE/SD values were available only graphically in Figure 5B
Zhao et al. (2023)	Largemouth bass (Micropterus salmoides)	Tributyrin or alanyl-glutamine supplementation in a diet containing 41.3% soybean meal and 35% fishmeal	TB0.1 (SM diet + 0.1% tributyrin)	PM (34.8% peanut-meal positive-control diet)	Intestinal ACC relative expression	Not reported numerically	Not reported numerically	3	3	Not estimable	Not estimable	Not estimable	The figure shows means, error bars, and significance lettering, but the article does not provide exact numerical values for this benchmark comparison.	Exact treatment means and SE/SD values were available only graphically in Figure 5C
Zhao et al. (2023)	Largemouth bass (Micropterus salmoides)	Tributyrin or alanyl-glutamine supplementation in a diet containing 41.3% soybean meal and 35% fishmeal	TB0.1 (SM diet + 0.1% tributyrin)	PM (34.8% peanut-meal positive-control diet)	Intestinal AMPK-alpha relative expression	Not reported numerically	Not reported numerically	3	3	Not estimable	Not estimable	Not estimable	The figure shows means, error bars, and significance lettering, but the article does not provide exact numerical values for this benchmark comparison.	Exact treatment means and SE/SD values were available only graphically in Figure 5D
Zhao et al. (2023)	Largemouth bass (Micropterus salmoides)	Tributyrin or alanyl-glutamine supplementation in a diet containing 41.3% soybean meal and 35% fishmeal	TB0.1 (SM diet + 0.1% tributyrin)	PM (34.8% peanut-meal positive-control diet)	Intestinal TNF-alpha relative expression	Not reported numerically	Not reported numerically	3	3	Not estimable	Not estimable	Not estimable	The figure shows means, error bars, and significance lettering, but the article does not provide exact numerical values for this benchmark comparison.	Exact treatment means and SE/SD values were available only graphically in Figure 6A
Zhao et al. (2023)	Largemouth bass (Micropterus salmoides)	Tributyrin or alanyl-glutamine supplementation in a diet containing 41.3% soybean meal and 35% fishmeal	TB0.1 (SM diet + 0.1% tributyrin)	PM (34.8% peanut-meal positive-control diet)	Intestinal IL-1beta relative expression	Not reported numerically	Not reported numerically	3	3	Not estimable	Not estimable	Not estimable	The figure shows means, error bars, and significance lettering, but the article does not provide exact numerical values for this benchmark comparison.	Exact treatment means and SE/SD values were available only graphically in Figure 6B
Zhao et al. (2023)	Largemouth bass (Micropterus salmoides)	Tributyrin or alanyl-glutamine supplementation in a diet containing 41.3% soybean meal and 35% fishmeal	TB0.1 (SM diet + 0.1% tributyrin)	PM (34.8% peanut-meal positive-control diet)	Intestinal TGF-beta1 relative expression	Not reported numerically	Not reported numerically	3	3	Not estimable	Not estimable	Not estimable	The figure shows means, error bars, and significance lettering, but the article does not provide exact numerical values for this benchmark comparison.	Exact treatment means and SE/SD values were available only graphically in Figure 6C

Study	Species	Strategy	Intervention	Comparator	Outcome and unit	Intervention mean +/- SD	Comparator mean +/- SD	n intervention (tanks)	n comparator (tanks)	Effect-size metric	Effect size	95% CI	Interpretation	Reason not estimated
Zhao et al. (2023)	Largemouth bass (Micropterus salmoides)	Tributyrin or alanyl-glutamine supplementation in a diet containing 41.3% soybean meal and 35% fishmeal	TB0.1 (SM diet + 0.1% tributyrin)	PM (34.8% peanut-meal positive-control diet)	Intestinal TGF-beta2 relative expression	Not reported numerically	Not reported numerically	3	3	Not estimable	Not estimable	Not estimable	The figure shows means, error bars, and significance lettering, but the article does not provide exact numerical values for this benchmark comparison.	Exact treatment means and SE/SD values were available only graphically in Figure 6D
Zhao et al. (2023)	Largemouth bass (Micropterus salmoides)	Tributyrin or alanyl-glutamine supplementation in a diet containing 41.3% soybean meal and 35% fishmeal	TB0.1 (SM diet + 0.1% tributyrin)	PM (34.8% peanut-meal positive-control diet)	Intestinal caspase-3 relative expression	Not reported numerically	Not reported numerically	3	3	Not estimable	Not estimable	Not estimable	The figure shows means, error bars, and significance lettering, but the article does not provide exact numerical values for this benchmark comparison.	Exact treatment means and SE/SD values were available only graphically in Figure 7A
Zhao et al. (2023)	Largemouth bass (Micropterus salmoides)	Tributyrin or alanyl-glutamine supplementation in a diet containing 41.3% soybean meal and 35% fishmeal	TB0.1 (SM diet + 0.1% tributyrin)	PM (34.8% peanut-meal positive-control diet)	Intestinal caspase-8 relative expression	Not reported numerically	Not reported numerically	3	3	Not estimable	Not estimable	Not estimable	The figure shows means, error bars, and significance lettering, but the article does not provide exact numerical values for this benchmark comparison.	Exact treatment means and SE/SD values were available only graphically in Figure 7B
Zhao et al. (2023)	Largemouth bass (Micropterus salmoides)	Tributyrin or alanyl-glutamine supplementation in a diet containing 41.3% soybean meal and 35% fishmeal	TB0.1 (SM diet + 0.1% tributyrin)	PM (34.8% peanut-meal positive-control diet)	Intestinal caspase-9 relative expression	Not reported numerically	Not reported numerically	3	3	Not estimable	Not estimable	Not estimable	The figure shows means, error bars, and significance lettering, but the article does not provide exact numerical values for this benchmark comparison.	Exact treatment means and SE/SD values were available only graphically in Figure 7C
Zhao et al. (2023)	Largemouth bass (Micropterus salmoides)	Tributyrin or alanyl-glutamine supplementation in a diet containing 41.3% soybean meal and 35% fishmeal	TB0.2 (SM diet + 0.2% tributyrin)	PM (34.8% peanut-meal positive-control diet)	Foregut trypsin activity (U/mg protein)	Not reported numerically	Not reported numerically	3	3	Not estimable	Not estimable	Not estimable	The figure shows means, error bars, and significance lettering, but the article does not provide exact numerical values for this benchmark comparison.	Exact treatment means and SE/SD values were available only graphically in Figure 1A
Zhao et al. (2023)	Largemouth bass (Micropterus salmoides)	Tributyrin or alanyl-glutamine supplementation in a diet containing 41.3% soybean meal and 35% fishmeal	TB0.2 (SM diet + 0.2% tributyrin)	PM (34.8% peanut-meal positive-control diet)	Midgut trypsin activity (U/mg protein)	Not reported numerically	Not reported numerically	3	3	Not estimable	Not estimable	Not estimable	The figure shows means, error bars, and significance lettering, but the article does not provide exact numerical values for this benchmark comparison.	Exact treatment means and SE/SD values were available only graphically in Figure 1A
Zhao et al. (2023)	Largemouth bass (Micropterus salmoides)	Tributyrin or alanyl-glutamine supplementation in a diet containing 41.3% soybean meal and 35% fishmeal	TB0.2 (SM diet + 0.2% tributyrin)	PM (34.8% peanut-meal positive-control diet)	Hindgut trypsin activity (U/mg protein)	Not reported numerically	Not reported numerically	3	3	Not estimable	Not estimable	Not estimable	The figure shows means, error bars, and significance lettering, but the article does not provide exact numerical values for this benchmark comparison.	Exact treatment means and SE/SD values were available only graphically in Figure 1A
Zhao et al. (2023)	Largemouth bass (Micropterus salmoides)	Tributyrin or alanyl-glutamine supplementation in a diet containing 41.3% soybean meal and 35% fishmeal	TB0.2 (SM diet + 0.2% tributyrin)	PM (34.8% peanut-meal positive-control diet)	Foregut lipase activity (U/mg protein)	Not reported numerically	Not reported numerically	3	3	Not estimable	Not estimable	Not estimable	The figure shows means, error bars, and significance lettering, but the article does not provide exact numerical values for this benchmark comparison.	Exact treatment means and SE/SD values were available only graphically in Figure 1B
Zhao et al. (2023)	Largemouth bass (Micropterus salmoides)	Tributyrin or alanyl-glutamine supplementation in a diet containing 41.3% soybean meal and 35% fishmeal	TB0.2 (SM diet + 0.2% tributyrin)	PM (34.8% peanut-meal positive-control diet)	Midgut lipase activity (U/mg protein)	Not reported numerically	Not reported numerically	3	3	Not estimable	Not estimable	Not estimable	The figure shows means, error bars, and significance lettering, but the article does not provide exact numerical values for this benchmark comparison.	Exact treatment means and SE/SD values were available only graphically in Figure 1B
Zhao et al. (2023)	Largemouth bass (Micropterus salmoides)	Tributyrin or alanyl-glutamine supplementation in a diet containing 41.3% soybean meal and 35% fishmeal	TB0.2 (SM diet + 0.2% tributyrin)	PM (34.8% peanut-meal positive-control diet)	Hindgut lipase activity (U/mg protein)	Not reported numerically	Not reported numerically	3	3	Not estimable	Not estimable	Not estimable	The figure shows means, error bars, and significance lettering, but the article does not provide exact numerical values for this benchmark comparison.	Exact treatment means and SE/SD values were available only graphically in Figure 1B
Zhao et al. (2023)	Largemouth bass (Micropterus salmoides)	Tributyrin or alanyl-glutamine supplementation in a diet containing 41.3% soybean meal and 35% fishmeal	TB0.2 (SM diet + 0.2% tributyrin)	PM (34.8% peanut-meal positive-control diet)	Foregut amylase activity (U/mg protein)	Not reported numerically	Not reported numerically	3	3	Not estimable	Not estimable	Not estimable	The figure shows means, error bars, and significance lettering, but the article does not provide exact numerical values for this benchmark comparison.	Exact treatment means and SE/SD values were available only graphically in Figure 1C
Zhao et al. (2023)	Largemouth bass (Micropterus salmoides)	Tributyrin or alanyl-glutamine supplementation in a diet containing 41.3% soybean meal and 35% fishmeal	TB0.2 (SM diet + 0.2% tributyrin)	PM (34.8% peanut-meal positive-control diet)	Midgut amylase activity (U/mg protein)	Not reported numerically	Not reported numerically	3	3	Not estimable	Not estimable	Not estimable	The figure shows means, error bars, and significance lettering, but the article does not provide exact numerical values for this benchmark comparison.	Exact treatment means and SE/SD values were available only graphically in Figure 1C
Zhao et al. (2023)	Largemouth bass (Micropterus salmoides)	Tributyrin or alanyl-glutamine supplementation in a diet containing 41.3% soybean meal and 35% fishmeal	TB0.2 (SM diet + 0.2% tributyrin)	PM (34.8% peanut-meal positive-control diet)	Hindgut amylase activity (U/mg protein)	Not reported numerically	Not reported numerically	3	3	Not estimable	Not estimable	Not estimable	The figure shows means, error bars, and significance lettering, but the article does not provide exact numerical values for this benchmark comparison.	Exact treatment means and SE/SD values were available only graphically in Figure 1C
Zhao et al. (2023)	Largemouth bass (Micropterus salmoides)	Tributyrin or alanyl-glutamine supplementation in a diet containing 41.3% soybean meal and 35% fishmeal	TB0.2 (SM diet + 0.2% tributyrin)	PM (34.8% peanut-meal positive-control diet)	Intestinal 4E-BP relative expression	Not reported numerically	Not reported numerically	3	3	Not estimable	Not estimable	Not estimable	The figure shows means, error bars, and significance lettering, but the article does not provide exact numerical values for this benchmark comparison.	Exact treatment means and SE/SD values were available only graphically in Figure 5A
Zhao et al. (2023)	Largemouth bass (Micropterus salmoides)	Tributyrin or alanyl-glutamine supplementation in a diet containing 41.3% soybean meal and 35% fishmeal	TB0.2 (SM diet + 0.2% tributyrin)	PM (34.8% peanut-meal positive-control diet)	Intestinal TOR relative expression	Not reported numerically	Not reported numerically	3	3	Not estimable	Not estimable	Not estimable	The figure shows means, error bars, and significance lettering, but the article does not provide exact numerical values for this benchmark comparison.	Exact treatment means and SE/SD values were available only graphically in Figure 5B
Zhao et al. (2023)	Largemouth bass (Micropterus salmoides)	Tributyrin or alanyl-glutamine supplementation in a diet containing 41.3% soybean meal and 35% fishmeal	TB0.2 (SM diet + 0.2% tributyrin)	PM (34.8% peanut-meal positive-control diet)	Intestinal ACC relative expression	Not reported numerically	Not reported numerically	3	3	Not estimable	Not estimable	Not estimable	The figure shows means, error bars, and significance lettering, but the article does not provide exact numerical values for this benchmark comparison.	Exact treatment means and SE/SD values were available only graphically in Figure 5C
Zhao et al. (2023)	Largemouth bass (Micropterus salmoides)	Tributyrin or alanyl-glutamine supplementation in a diet containing 41.3% soybean meal and 35% fishmeal	TB0.2 (SM diet + 0.2% tributyrin)	PM (34.8% peanut-meal positive-control diet)	Intestinal AMPK-alpha relative expression	Not reported numerically	Not reported numerically	3	3	Not estimable	Not estimable	Not estimable	The figure shows means, error bars, and significance lettering, but the article does not provide exact numerical values for this benchmark comparison.	Exact treatment means and SE/SD values were available only graphically in Figure 5D
Zhao et al. (2023)	Largemouth bass (Micropterus salmoides)	Tributyrin or alanyl-glutamine supplementation in a diet containing 41.3% soybean meal and 35% fishmeal	TB0.2 (SM diet + 0.2% tributyrin)	PM (34.8% peanut-meal positive-control diet)	Intestinal TNF-alpha relative expression	Not reported numerically	Not reported numerically	3	3	Not estimable	Not estimable	Not estimable	The figure shows means, error bars, and significance lettering, but the article does not provide exact numerical values for this benchmark comparison.	Exact treatment means and SE/SD values were available only graphically in Figure 6A
Zhao et al. (2023)	Largemouth bass (Micropterus salmoides)	Tributyrin or alanyl-glutamine supplementation in a diet containing 41.3% soybean meal and 35% fishmeal	TB0.2 (SM diet + 0.2% tributyrin)	PM (34.8% peanut-meal positive-control diet)	Intestinal IL-1beta relative expression	Not reported numerically	Not reported numerically	3	3	Not estimable	Not estimable	Not estimable	The figure shows means, error bars, and significance lettering, but the article does not provide exact numerical values for this benchmark comparison.	Exact treatment means and SE/SD values were available only graphically in Figure 6B
Zhao et al. (2023)	Largemouth bass (Micropterus salmoides)	Tributyrin or alanyl-glutamine supplementation in a diet containing 41.3% soybean meal and 35% fishmeal	TB0.2 (SM diet + 0.2% tributyrin)	PM (34.8% peanut-meal positive-control diet)	Intestinal TGF-beta1 relative expression	Not reported numerically	Not reported numerically	3	3	Not estimable	Not estimable	Not estimable	The figure shows means, error bars, and significance lettering, but the article does not provide exact numerical values for this benchmark comparison.	Exact treatment means and SE/SD values were available only graphically in Figure 6C
Zhao et al. (2023)	Largemouth bass (Micropterus salmoides)	Tributyrin or alanyl-glutamine supplementation in a diet containing 41.3% soybean meal and 35% fishmeal	TB0.2 (SM diet + 0.2% tributyrin)	PM (34.8% peanut-meal positive-control diet)	Intestinal TGF-beta2 relative expression	Not reported numerically	Not reported numerically	3	3	Not estimable	Not estimable	Not estimable	The figure shows means, error bars, and significance lettering, but the article does not provide exact numerical values for this benchmark comparison.	Exact treatment means and SE/SD values were available only graphically in Figure 6D
Zhao et al. (2023)	Largemouth bass (Micropterus salmoides)	Tributyrin or alanyl-glutamine supplementation in a diet containing 41.3% soybean meal and 35% fishmeal	TB0.2 (SM diet + 0.2% tributyrin)	PM (34.8% peanut-meal positive-control diet)	Intestinal caspase-3 relative expression	Not reported numerically	Not reported numerically	3	3	Not estimable	Not estimable	Not estimable	The figure shows means, error bars, and significance lettering, but the article does not provide exact numerical values for this benchmark comparison.	Exact treatment means and SE/SD values were available only graphically in Figure 7A
Zhao et al. (2023)	Largemouth bass (Micropterus salmoides)	Tributyrin or alanyl-glutamine supplementation in a diet containing 41.3% soybean meal and 35% fishmeal	TB0.2 (SM diet + 0.2% tributyrin)	PM (34.8% peanut-meal positive-control diet)	Intestinal caspase-8 relative expression	Not reported numerically	Not reported numerically	3	3	Not estimable	Not estimable	Not estimable	The figure shows means, error bars, and significance lettering, but the article does not provide exact numerical values for this benchmark comparison.	Exact treatment means and SE/SD values were available only graphically in Figure 7B
Zhao et al. (2023)	Largemouth bass (Micropterus salmoides)	Tributyrin or alanyl-glutamine supplementation in a diet containing 41.3% soybean meal and 35% fishmeal	TB0.2 (SM diet + 0.2% tributyrin)	PM (34.8% peanut-meal positive-control diet)	Intestinal caspase-9 relative expression	Not reported numerically	Not reported numerically	3	3	Not estimable	Not estimable	Not estimable	The figure shows means, error bars, and significance lettering, but the article does not provide exact numerical values for this benchmark comparison.	Exact treatment means and SE/SD values were available only graphically in Figure 7C

Note: Tables 3-5 report mean +/- SE from three independent tanks per diet; SD was reconstructed as SE x sqrt(3). Each additive diet was compared with the soybean-meal negative control and the peanut-meal positive control. FCR and crypt depth were direction-reversed because lower values were treated as favorable. Digestive-enzyme and gene-expression outcomes were marked not estimable because exact values were available only in Figures 1 and 5-7. Pairwise 95% CIs are descriptive and unadjusted.

Source: Zhao et al. (2023), https://doi.org/10.3389/fimmu.2023.1140678

Study 23. Teodosio et al. (2021): eco-diets and a multi-additive dietary package for gilthead seabream under low temperature

Study	Species	Strategy	Intervention	Comparator	Outcome and unit	Intervention mean +/- SD	Comparator mean +/- SD	n intervention (tanks)	n comparator (tanks)	Effect-size metric	Effect size	95% CI	Interpretation	Reason not estimated
Teodosio et al. (2021)	Gilthead seabream (Sparus aurata)	Low-protein, low-fishmeal eco-diets, with or without a multi-additive package, under low-temperature rearing	ECOSup (42.0% protein; 7.5% fishmeal; 15% poultry meal; betaine, krill meal, soy lecithin, macroalgae and taurine)	ECO (42.0% protein; 10% fishmeal; 15% poultry meal)	Apparent digestibility coefficient of dry matter (%)	69.6 +/- 1.9	64.9 +/- 1.8	3	3	Hedges' g	2.03	0.13 to 3.93	Higher, more favorable value with the intervention diet; unadjusted 95% CI excluded 0	Not applicable - effect size estimated from reported mean +/- SD
Teodosio et al. (2021)	Gilthead seabream (Sparus aurata)	Low-protein, low-fishmeal eco-diets, with or without a multi-additive package, under low-temperature rearing	ECOSup (42.0% protein; 7.5% fishmeal; 15% poultry meal; betaine, krill meal, soy lecithin, macroalgae and taurine)	ECO (42.0% protein; 10% fishmeal; 15% poultry meal)	Apparent digestibility coefficient of protein (%)	92.3 +/- 1.1	90.0 +/- 3.2	3	3	Hedges' g	0.77	-0.62 to 2.16	Higher, more favorable value with the intervention diet; unadjusted 95% CI included 0	Not applicable - effect size estimated from reported mean +/- SD
Teodosio et al. (2021)	Gilthead seabream (Sparus aurata)	Low-protein, low-fishmeal eco-diets, with or without a multi-additive package, under low-temperature rearing	ECOSup (42.0% protein; 7.5% fishmeal; 15% poultry meal; betaine, krill meal, soy lecithin, macroalgae and taurine)	ECO (42.0% protein; 10% fishmeal; 15% poultry meal)	Apparent digestibility coefficient of fat (%)	96.2 +/- 0.7	94.6 +/- 1.4	3	3	Hedges' g	1.16	-0.35 to 2.67	Higher, more favorable value with the intervention diet; unadjusted 95% CI included 0	Not applicable - effect size estimated from reported mean +/- SD
Teodosio et al. (2021)	Gilthead seabream (Sparus aurata)	Low-protein, low-fishmeal eco-diets, with or without a multi-additive package, under low-temperature rearing	ECOSup (42.0% protein; 7.5% fishmeal; 15% poultry meal; betaine, krill meal, soy lecithin, macroalgae and taurine)	ECO (42.0% protein; 10% fishmeal; 15% poultry meal)	Apparent digestibility coefficient of phosphorus (%)	56.3 +/- 2.2	45.9 +/- 1.9	3	3	Hedges' g	4.05	0.96 to 7.13	Higher, more favorable value with the intervention diet; unadjusted 95% CI excluded 0	Not applicable - effect size estimated from reported mean +/- SD
Teodosio et al. (2021)	Gilthead seabream (Sparus aurata)	Low-protein, low-fishmeal eco-diets, with or without a multi-additive package, under low-temperature rearing	ECOSup (42.0% protein; 7.5% fishmeal; 15% poultry meal; betaine, krill meal, soy lecithin, macroalgae and taurine)	ECO (42.0% protein; 10% fishmeal; 15% poultry meal)	Apparent digestibility coefficient of energy (%)	88.1 +/- 0.7	85.5 +/- 1.3	3	3	Hedges' g	1.99	0.11 to 3.88	Higher, more favorable value with the intervention diet; unadjusted 95% CI excluded 0	Not applicable - effect size estimated from reported mean +/- SD
Teodosio et al. (2021)	Gilthead seabream (Sparus aurata)	Low-protein, low-fishmeal eco-diets, with or without a multi-additive package, under low-temperature rearing	ECOSup (42.0% protein; 7.5% fishmeal; 15% poultry meal; betaine, krill meal, soy lecithin, macroalgae and taurine)	ECO (42.0% protein; 10% fishmeal; 15% poultry meal)	Apparent digestibility coefficient of Arginine (%)	83.1 +/- 2.3	79.4 +/- 1.4	3	3	Hedges' g	1.55	-0.12 to 3.23	Higher, more favorable value with the intervention diet; unadjusted 95% CI included 0	Not applicable - effect size estimated from reported mean +/- SD
Teodosio et al. (2021)	Gilthead seabream (Sparus aurata)	Low-protein, low-fishmeal eco-diets, with or without a multi-additive package, under low-temperature rearing	ECOSup (42.0% protein; 7.5% fishmeal; 15% poultry meal; betaine, krill meal, soy lecithin, macroalgae and taurine)	ECO (42.0% protein; 10% fishmeal; 15% poultry meal)	Apparent digestibility coefficient of Histidine (%)	88.6 +/- 1.4	86.8 +/- 0.5	3	3	Hedges' g	1.37	-0.22 to 2.96	Higher, more favorable value with the intervention diet; unadjusted 95% CI included 0	Not applicable - effect size estimated from reported mean +/- SD
Teodosio et al. (2021)	Gilthead seabream (Sparus aurata)	Low-protein, low-fishmeal eco-diets, with or without a multi-additive package, under low-temperature rearing	ECOSup (42.0% protein; 7.5% fishmeal; 15% poultry meal; betaine, krill meal, soy lecithin, macroalgae and taurine)	ECO (42.0% protein; 10% fishmeal; 15% poultry meal)	Apparent digestibility coefficient of Lysine (%)	89.4 +/- 1.4	86.2 +/- 0.8	3	3	Hedges' g	2.25	0.23 to 4.26	Higher, more favorable value with the intervention diet; unadjusted 95% CI excluded 0	Not applicable - effect size estimated from reported mean +/- SD
Teodosio et al. (2021)	Gilthead seabream (Sparus aurata)	Low-protein, low-fishmeal eco-diets, with or without a multi-additive package, under low-temperature rearing	ECOSup (42.0% protein; 7.5% fishmeal; 15% poultry meal; betaine, krill meal, soy lecithin, macroalgae and taurine)	ECO (42.0% protein; 10% fishmeal; 15% poultry meal)	Apparent digestibility coefficient of Threonine (%)	85.8 +/- 1.4	83.8 +/- 1.2	3	3	Hedges' g	1.23	-0.31 to 2.76	Higher, more favorable value with the intervention diet; unadjusted 95% CI included 0	Not applicable - effect size estimated from reported mean +/- SD
Teodosio et al. (2021)	Gilthead seabream (Sparus aurata)	Low-protein, low-fishmeal eco-diets, with or without a multi-additive package, under low-temperature rearing	ECOSup (42.0% protein; 7.5% fishmeal; 15% poultry meal; betaine, krill meal, soy lecithin, macroalgae and taurine)	ECO (42.0% protein; 10% fishmeal; 15% poultry meal)	Apparent digestibility coefficient of Isoleucine (%)	91.0 +/- 1.1	89.6 +/- 0.7	3	3	Hedges' g	1.21	-0.32 to 2.75	Higher, more favorable value with the intervention diet; unadjusted 95% CI included 0	Not applicable - effect size estimated from reported mean +/- SD
Teodosio et al. (2021)	Gilthead seabream (Sparus aurata)	Low-protein, low-fishmeal eco-diets, with or without a multi-additive package, under low-temperature rearing	ECOSup (42.0% protein; 7.5% fishmeal; 15% poultry meal; betaine, krill meal, soy lecithin, macroalgae and taurine)	ECO (42.0% protein; 10% fishmeal; 15% poultry meal)	Apparent digestibility coefficient of Leucine (%)	89.1 +/- 1.4	89.4 +/- 0.6	3	3	Hedges' g	-0.22	-1.51 to 1.07	Lower, less favorable value with the intervention diet; unadjusted 95% CI included 0	Not applicable - effect size estimated from reported mean +/- SD
Teodosio et al. (2021)	Gilthead seabream (Sparus aurata)	Low-protein, low-fishmeal eco-diets, with or without a multi-additive package, under low-temperature rearing	ECOSup (42.0% protein; 7.5% fishmeal; 15% poultry meal; betaine, krill meal, soy lecithin, macroalgae and taurine)	ECO (42.0% protein; 10% fishmeal; 15% poultry meal)	Apparent digestibility coefficient of Valine (%)	91.0 +/- 1.2	89.3 +/- 0.6	3	3	Hedges' g	1.43	-0.19 to 3.05	Higher, more favorable value with the intervention diet; unadjusted 95% CI included 0	Not applicable - effect size estimated from reported mean +/- SD
Teodosio et al. (2021)	Gilthead seabream (Sparus aurata)	Low-protein, low-fishmeal eco-diets, with or without a multi-additive package, under low-temperature rearing	ECOSup (42.0% protein; 7.5% fishmeal; 15% poultry meal; betaine, krill meal, soy lecithin, macroalgae and taurine)	ECO (42.0% protein; 10% fishmeal; 15% poultry meal)	Apparent digestibility coefficient of Methionine (%)	89.3 +/- 1.2	90.3 +/- 0.6	3	3	Hedges' g	-0.84	-2.25 to 0.56	Lower, less favorable value with the intervention diet; unadjusted 95% CI included 0	Not applicable - effect size estimated from reported mean +/- SD
Teodosio et al. (2021)	Gilthead seabream (Sparus aurata)	Low-protein, low-fishmeal eco-diets, with or without a multi-additive package, under low-temperature rearing	ECOSup (42.0% protein; 7.5% fishmeal; 15% poultry meal; betaine, krill meal, soy lecithin, macroalgae and taurine)	ECO (42.0% protein; 10% fishmeal; 15% poultry meal)	Apparent digestibility coefficient of Phenylalanine (%)	89.6 +/- 1.4	88.8 +/- 0.7	3	3	Hedges' g	0.58	-0.76 to 1.92	Higher, more favorable value with the intervention diet; unadjusted 95% CI included 0	Not applicable - effect size estimated from reported mean +/- SD
Teodosio et al. (2021)	Gilthead seabream (Sparus aurata)	Low-protein, low-fishmeal eco-diets, with or without a multi-additive package, under low-temperature rearing	ECOSup (42.0% protein; 7.5% fishmeal; 15% poultry meal; betaine, krill meal, soy lecithin, macroalgae and taurine)	ECO (42.0% protein; 10% fishmeal; 15% poultry meal)	Apparent digestibility coefficient of Cystine (%)	85.9 +/- 2.2	88.5 +/- 0.8	3	3	Hedges' g	-1.26	-2.80 to 0.29	Lower, less favorable value with the intervention diet; unadjusted 95% CI included 0	Not applicable - effect size estimated from reported mean +/- SD
Teodosio et al. (2021)	Gilthead seabream (Sparus aurata)	Low-protein, low-fishmeal eco-diets, with or without a multi-additive package, under low-temperature rearing	ECOSup (42.0% protein; 7.5% fishmeal; 15% poultry meal; betaine, krill meal, soy lecithin, macroalgae and taurine)	ECO (42.0% protein; 10% fishmeal; 15% poultry meal)	Apparent digestibility coefficient of Tyrosine (%)	92.1 +/- 0.9	92.7 +/- 0.6	3	3	Hedges' g	-0.63	-1.98 to 0.72	Lower, less favorable value with the intervention diet; unadjusted 95% CI included 0	Not applicable - effect size estimated from reported mean +/- SD
Teodosio et al. (2021)	Gilthead seabream (Sparus aurata)	Low-protein, low-fishmeal eco-diets, with or without a multi-additive package, under low-temperature rearing	ECOSup (42.0% protein; 7.5% fishmeal; 15% poultry meal; betaine, krill meal, soy lecithin, macroalgae and taurine)	ECO (42.0% protein; 10% fishmeal; 15% poultry meal)	Apparent digestibility coefficient of Aspartic acid + asparagine (%)	75.5 +/- 3.4	71.3 +/- 2.0	3	3	Hedges' g	1.2	-0.32 to 2.73	Higher, more favorable value with the intervention diet; unadjusted 95% CI included 0	Not applicable - effect size estimated from reported mean +/- SD
Teodosio et al. (2021)	Gilthead seabream (Sparus aurata)	Low-protein, low-fishmeal eco-diets, with or without a multi-additive package, under low-temperature rearing	ECOSup (42.0% protein; 7.5% fishmeal; 15% poultry meal; betaine, krill meal, soy lecithin, macroalgae and taurine)	ECO (42.0% protein; 10% fishmeal; 15% poultry meal)	Apparent digestibility coefficient of Glutamic acid + glutamine (%)	88.8 +/- 1.3	87.6 +/- 0.3	3	3	Hedges' g	1.02	-0.44 to 2.48	Higher, more favorable value with the intervention diet; unadjusted 95% CI included 0	Not applicable - effect size estimated from reported mean +/- SD
Teodosio et al. (2021)	Gilthead seabream (Sparus aurata)	Low-protein, low-fishmeal eco-diets, with or without a multi-additive package, under low-temperature rearing	ECOSup (42.0% protein; 7.5% fishmeal; 15% poultry meal; betaine, krill meal, soy lecithin, macroalgae and taurine)	ECO (42.0% protein; 10% fishmeal; 15% poultry meal)	Apparent digestibility coefficient of Alanine (%)	90.6 +/- 1.1	88.4 +/- 0.6	3	3	Hedges' g	1.99	0.11 to 3.87	Higher, more favorable value with the intervention diet; unadjusted 95% CI excluded 0	Not applicable - effect size estimated from reported mean +/- SD
Teodosio et al. (2021)	Gilthead seabream (Sparus aurata)	Low-protein, low-fishmeal eco-diets, with or without a multi-additive package, under low-temperature rearing	ECOSup (42.0% protein; 7.5% fishmeal; 15% poultry meal; betaine, krill meal, soy lecithin, macroalgae and taurine)	ECO (42.0% protein; 10% fishmeal; 15% poultry meal)	Apparent digestibility coefficient of Glycine (%)	94.8 +/- 0.7	93.1 +/- 0.4	3	3	Hedges' g	2.39	0.29 to 4.48	Higher, more favorable value with the intervention diet; unadjusted 95% CI excluded 0	Not applicable - effect size estimated from reported mean +/- SD
Teodosio et al. (2021)	Gilthead seabream (Sparus aurata)	Low-protein, low-fishmeal eco-diets, with or without a multi-additive package, under low-temperature rearing	ECOSup (42.0% protein; 7.5% fishmeal; 15% poultry meal; betaine, krill meal, soy lecithin, macroalgae and taurine)	ECO (42.0% protein; 10% fishmeal; 15% poultry meal)	Apparent digestibility coefficient of Proline (%)	89.7 +/- 1.2	88.7 +/- 0.8	3	3	Hedges' g	0.78	-0.61 to 2.18	Higher, more favorable value with the intervention diet; unadjusted 95% CI included 0	Not applicable - effect size estimated from reported mean +/- SD
Teodosio et al. (2021)	Gilthead seabream (Sparus aurata)	Low-protein, low-fishmeal eco-diets, with or without a multi-additive package, under low-temperature rearing	ECOSup (42.0% protein; 7.5% fishmeal; 15% poultry meal; betaine, krill meal, soy lecithin, macroalgae and taurine)	ECO (42.0% protein; 10% fishmeal; 15% poultry meal)	Apparent digestibility coefficient of Serine (%)	84.6 +/- 2.2	83.1 +/- 1.2	3	3	Hedges' g	0.68	-0.69 to 2.04	Higher, more favorable value with the intervention diet; unadjusted 95% CI included 0	Not applicable - effect size estimated from reported mean +/- SD
Teodosio et al. (2021)	Gilthead seabream (Sparus aurata)	Low-protein, low-fishmeal eco-diets, with or without a multi-additive package, under low-temperature rearing	ECOSup (42.0% protein; 7.5% fishmeal; 15% poultry meal; betaine, krill meal, soy lecithin, macroalgae and taurine)	ECO (42.0% protein; 10% fishmeal; 15% poultry meal)	Apparent digestibility coefficient of Taurine (%)	87.3 +/- 1.3	85.1 +/- 1.5	3	3	Hedges' g	1.25	-0.29 to 2.80	Higher, more favorable value with the intervention diet; unadjusted 95% CI included 0	Not applicable - effect size estimated from reported mean +/- SD
Teodosio et al. (2021)	Gilthead seabream (Sparus aurata)	Low-protein, low-fishmeal eco-diets, with or without a multi-additive package, under low-temperature rearing	ECOSup (42.0% protein; 7.5% fishmeal; 15% poultry meal; betaine, krill meal, soy lecithin, macroalgae and taurine)	ECO (42.0% protein; 10% fishmeal; 15% poultry meal)	Weight gain (%)	22.3 +/- 3.0	18.5 +/- 6.6	3	3	Hedges' g	0.59	-0.75 to 1.94	Higher, more favorable value with the intervention diet; unadjusted 95% CI included 0	Not applicable - effect size estimated from reported mean +/- SD

Study	Species	Strategy	Intervention	Comparator	Outcome and unit	Intervention mean +/- SD	Comparator mean +/- SD	n intervention (tanks)	n comparator (tanks)	Effect-size metric	Effect size	95% CI	Interpretation	Reason not estimated
Teodosio et al. (2021)	Gilthead seabream (Sparus aurata)	Low-protein, low-fishmeal eco-diets, with or without a multi-additive package, under low-temperature rearing	ECOSup (42.0% protein; 7.5% fishmeal; 15% poultry meal; betaine, krill meal, soy lecithin, macroalgae and taurine)	ECO (42.0% protein; 10% fishmeal; 15% poultry meal)	Thermal growth coefficient	0.040 +/- 0.010	0.020 +/- 0.010	3	3	Hedges' g	1.6	-0.09 to 3.29	Higher, more favorable value with the intervention diet; unadjusted 95% CI included 0	Not applicable - effect size estimated from reported mean +/- SD
Teodosio et al. (2021)	Gilthead seabream (Sparus aurata)	Low-protein, low-fishmeal eco-diets, with or without a multi-additive package, under low-temperature rearing	ECOSup (42.0% protein; 7.5% fishmeal; 15% poultry meal; betaine, krill meal, soy lecithin, macroalgae and taurine)	ECO (42.0% protein; 10% fishmeal; 15% poultry meal)	Daily voluntary feed intake (%/day)	0.480 +/- 0.040	0.440 +/- 0.040	3	3	Hedges' g	0.8	-0.60 to 2.20	Higher, more favorable value with the intervention diet; unadjusted 95% CI included 0	Not applicable - effect size estimated from reported mean +/- SD
Teodosio et al. (2021)	Gilthead seabream (Sparus aurata)	Low-protein, low-fishmeal eco-diets, with or without a multi-additive package, under low-temperature rearing	ECOSup (42.0% protein; 7.5% fishmeal; 15% poultry meal; betaine, krill meal, soy lecithin, macroalgae and taurine)	ECO (42.0% protein; 10% fishmeal; 15% poultry meal)	Feed conversion ratio	1.90 +/- 0.20	2.10 +/- 0.30	3	3	Hedges' g (direction reversed)	0.63	-0.72 to 1.98	Lower, more favorable value with the intervention diet; unadjusted 95% CI included 0	Not applicable - effect size estimated from reported mean +/- SD
Teodosio et al. (2021)	Gilthead seabream (Sparus aurata)	Low-protein, low-fishmeal eco-diets, with or without a multi-additive package, under low-temperature rearing	ECOSup (42.0% protein; 7.5% fishmeal; 15% poultry meal; betaine, krill meal, soy lecithin, macroalgae and taurine)	ECO (42.0% protein; 10% fishmeal; 15% poultry meal)	Protein efficiency ratio	1.30 +/- 0.10	1.20 +/- 0.20	3	3	Hedges' g	0.51	-0.82 to 1.83	Higher, more favorable value with the intervention diet; unadjusted 95% CI included 0	Not applicable - effect size estimated from reported mean +/- SD
Teodosio et al. (2021)	Gilthead seabream (Sparus aurata)	Low-protein, low-fishmeal eco-diets, with or without a multi-additive package, under low-temperature rearing	ECOSup (42.0% protein; 7.5% fishmeal; 15% poultry meal; betaine, krill meal, soy lecithin, macroalgae and taurine)	ECO (42.0% protein; 10% fishmeal; 15% poultry meal)	Moisture (% wet weight)	65.1 +/- 1.2	65.5 +/- 1.5	3	3	Hedges' g	-0.24	-1.53 to 1.06	Lower measured value with the intervention diet; unadjusted 95% CI included 0	Not applicable - effect size estimated from reported mean +/- SD
Teodosio et al. (2021)	Gilthead seabream (Sparus aurata)	Low-protein, low-fishmeal eco-diets, with or without a multi-additive package, under low-temperature rearing	ECOSup (42.0% protein; 7.5% fishmeal; 15% poultry meal; betaine, krill meal, soy lecithin, macroalgae and taurine)	ECO (42.0% protein; 10% fishmeal; 15% poultry meal)	Ash (% wet weight)	4.00 +/- 0.80	4.00 +/- 0.40	3	3	Hedges' g	0.0	-1.28 to 1.28	No observed mean difference; unadjusted 95% CI included 0	Not applicable - effect size estimated from reported mean +/- SD
Teodosio et al. (2021)	Gilthead seabream (Sparus aurata)	Low-protein, low-fishmeal eco-diets, with or without a multi-additive package, under low-temperature rearing	ECOSup (42.0% protein; 7.5% fishmeal; 15% poultry meal; betaine, krill meal, soy lecithin, macroalgae and taurine)	ECO (42.0% protein; 10% fishmeal; 15% poultry meal)	Protein (% wet weight)	16.5 +/- 0.3	16.7 +/- 0.8	3	3	Hedges' g	-0.26	-1.56 to 1.03	Lower measured value with the intervention diet; unadjusted 95% CI included 0	Not applicable - effect size estimated from reported mean +/- SD
Teodosio et al. (2021)	Gilthead seabream (Sparus aurata)	Low-protein, low-fishmeal eco-diets, with or without a multi-additive package, under low-temperature rearing	ECOSup (42.0% protein; 7.5% fishmeal; 15% poultry meal; betaine, krill meal, soy lecithin, macroalgae and taurine)	ECO (42.0% protein; 10% fishmeal; 15% poultry meal)	Fat (% wet weight)	12.8 +/- 0.9	12.4 +/- 0.7	3	3	Hedges' g	0.4	-0.91 to 1.71	Higher measured value with the intervention diet; unadjusted 95% CI included 0	Not applicable - effect size estimated from reported mean +/- SD
Teodosio et al. (2021)	Gilthead seabream (Sparus aurata)	Low-protein, low-fishmeal eco-diets, with or without a multi-additive package, under low-temperature rearing	ECOSup (42.0% protein; 7.5% fishmeal; 15% poultry meal; betaine, krill meal, soy lecithin, macroalgae and taurine)	ECO (42.0% protein; 10% fishmeal; 15% poultry meal)	Phosphorus (% wet weight)	0.900 +/- 0.100	0.900 +/- 0.100	3	3	Hedges' g	0.0	-1.28 to 1.28	No observed mean difference; unadjusted 95% CI included 0	Not applicable - effect size estimated from reported mean +/- SD
Teodosio et al. (2021)	Gilthead seabream (Sparus aurata)	Low-protein, low-fishmeal eco-diets, with or without a multi-additive package, under low-temperature rearing	ECOSup (42.0% protein; 7.5% fishmeal; 15% poultry meal; betaine, krill meal, soy lecithin, macroalgae and taurine)	ECO (42.0% protein; 10% fishmeal; 15% poultry meal)	Energy (MJ/kg)	8.10 +/- 0.40	8.10 +/- 0.50	3	3	Hedges' g	0.0	-1.28 to 1.28	No observed mean difference; unadjusted 95% CI included 0	Not applicable - effect size estimated from reported mean +/- SD
Teodosio et al. (2021)	Gilthead seabream (Sparus aurata)	Low-protein, low-fishmeal eco-diets, with or without a multi-additive package, under low-temperature rearing	ECOSup (42.0% protein; 7.5% fishmeal; 15% poultry meal; betaine, krill meal, soy lecithin, macroalgae and taurine)	ECO (42.0% protein; 10% fishmeal; 15% poultry meal)	Liver protein (% dry weight)	32.1 +/- 1.6	35.4 +/- 5.2	3	3	Hedges' g	-0.69	-2.05 to 0.68	Lower measured value with the intervention diet; unadjusted 95% CI included 0	Not applicable - effect size estimated from reported mean +/- SD
Teodosio et al. (2021)	Gilthead seabream (Sparus aurata)	Low-protein, low-fishmeal eco-diets, with or without a multi-additive package, under low-temperature rearing	ECOSup (42.0% protein; 7.5% fishmeal; 15% poultry meal; betaine, krill meal, soy lecithin, macroalgae and taurine)	ECO (42.0% protein; 10% fishmeal; 15% poultry meal)	Liver fat (% dry weight)	22.1 +/- 4.8	21.0 +/- 5.3	3	3	Hedges' g	0.17	-1.11 to 1.46	Higher measured value with the intervention diet; unadjusted 95% CI included 0	Not applicable - effect size estimated from reported mean +/- SD
Teodosio et al. (2021)	Gilthead seabream (Sparus aurata)	Low-protein, low-fishmeal eco-diets, with or without a multi-additive package, under low-temperature rearing	ECOSup (42.0% protein; 7.5% fishmeal; 15% poultry meal; betaine, krill meal, soy lecithin, macroalgae and taurine)	ECO (42.0% protein; 10% fishmeal; 15% poultry meal)	Faecal nitrogen loss (mg N/kg/day)	24.6 +/- 1.9	29.3 +/- 2.7	3	3	Hedges' g (direction reversed)	1.61	-0.09 to 3.31	Lower, more favorable value with the intervention diet; unadjusted 95% CI included 0	Not applicable - effect size estimated from reported mean +/- SD
Teodosio et al. (2021)	Gilthead seabream (Sparus aurata)	Low-protein, low-fishmeal eco-diets, with or without a multi-additive package, under low-temperature rearing	ECOSup (42.0% protein; 7.5% fishmeal; 15% poultry meal; betaine, krill meal, soy lecithin, macroalgae and taurine)	COM (44.2% protein; 27.5% fishmeal commercial-like reference)	Apparent digestibility coefficient of dry matter (%)	69.6 +/- 1.9	67.7 +/- 1.3	3	3	Hedges' g	0.93	-0.50 to 2.37	Higher, more favorable value with the intervention diet; unadjusted 95% CI included 0	Not applicable - effect size estimated from reported mean +/- SD
Teodosio et al. (2021)	Gilthead seabream (Sparus aurata)	Low-protein, low-fishmeal eco-diets, with or without a multi-additive package, under low-temperature rearing	ECOSup (42.0% protein; 7.5% fishmeal; 15% poultry meal; betaine, krill meal, soy lecithin, macroalgae and taurine)	COM (44.2% protein; 27.5% fishmeal commercial-like reference)	Apparent digestibility coefficient of protein (%)	92.3 +/- 1.1	89.0 +/- 1.5	3	3	Hedges' g	2.01	0.12 to 3.90	Higher, more favorable value with the intervention diet; unadjusted 95% CI excluded 0	Not applicable - effect size estimated from reported mean +/- SD
Teodosio et al. (2021)	Gilthead seabream (Sparus aurata)	Low-protein, low-fishmeal eco-diets, with or without a multi-additive package, under low-temperature rearing	ECOSup (42.0% protein; 7.5% fishmeal; 15% poultry meal; betaine, krill meal, soy lecithin, macroalgae and taurine)	COM (44.2% protein; 27.5% fishmeal commercial-like reference)	Apparent digestibility coefficient of fat (%)	96.2 +/- 0.7	95.4 +/- 0.8	3	3	Hedges' g	0.85	-0.56 to 2.26	Higher, more favorable value with the intervention diet; unadjusted 95% CI included 0	Not applicable - effect size estimated from reported mean +/- SD
Teodosio et al. (2021)	Gilthead seabream (Sparus aurata)	Low-protein, low-fishmeal eco-diets, with or without a multi-additive package, under low-temperature rearing	ECOSup (42.0% protein; 7.5% fishmeal; 15% poultry meal; betaine, krill meal, soy lecithin, macroalgae and taurine)	COM (44.2% protein; 27.5% fishmeal commercial-like reference)	Apparent digestibility coefficient of phosphorus (%)	56.3 +/- 2.2	54.6 +/- 1.6	3	3	Hedges' g	0.71	-0.66 to 2.08	Higher, more favorable value with the intervention diet; unadjusted 95% CI included 0	Not applicable - effect size estimated from reported mean +/- SD
Teodosio et al. (2021)	Gilthead seabream (Sparus aurata)	Low-protein, low-fishmeal eco-diets, with or without a multi-additive package, under low-temperature rearing	ECOSup (42.0% protein; 7.5% fishmeal; 15% poultry meal; betaine, krill meal, soy lecithin, macroalgae and taurine)	COM (44.2% protein; 27.5% fishmeal commercial-like reference)	Apparent digestibility coefficient of energy (%)	88.1 +/- 0.7	85.8 +/- 0.9	3	3	Hedges' g	2.28	0.25 to 4.32	Higher, more favorable value with the intervention diet; unadjusted 95% CI excluded 0	Not applicable - effect size estimated from reported mean +/- SD
Teodosio et al. (2021)	Gilthead seabream (Sparus aurata)	Low-protein, low-fishmeal eco-diets, with or without a multi-additive package, under low-temperature rearing	ECOSup (42.0% protein; 7.5% fishmeal; 15% poultry meal; betaine, krill meal, soy lecithin, macroalgae and taurine)	COM (44.2% protein; 27.5% fishmeal commercial-like reference)	Apparent digestibility coefficient of Arginine (%)	83.1 +/- 2.3	85.6 +/- 0.7	3	3	Hedges' g	-1.18	-2.69 to 0.34	Lower, less favorable value with the intervention diet; unadjusted 95% CI included 0	Not applicable - effect size estimated from reported mean +/- SD
Teodosio et al. (2021)	Gilthead seabream (Sparus aurata)	Low-protein, low-fishmeal eco-diets, with or without a multi-additive package, under low-temperature rearing	ECOSup (42.0% protein; 7.5% fishmeal; 15% poultry meal; betaine, krill meal, soy lecithin, macroalgae and taurine)	COM (44.2% protein; 27.5% fishmeal commercial-like reference)	Apparent digestibility coefficient of Histidine (%)	88.6 +/- 1.4	88.8 +/- 0.8	3	3	Hedges' g	-0.14	-1.42 to 1.14	Lower, less favorable value with the intervention diet; unadjusted 95% CI included 0	Not applicable - effect size estimated from reported mean +/- SD
Teodosio et al. (2021)	Gilthead seabream (Sparus aurata)	Low-protein, low-fishmeal eco-diets, with or without a multi-additive package, under low-temperature rearing	ECOSup (42.0% protein; 7.5% fishmeal; 15% poultry meal; betaine, krill meal, soy lecithin, macroalgae and taurine)	COM (44.2% protein; 27.5% fishmeal commercial-like reference)	Apparent digestibility coefficient of Lysine (%)	89.4 +/- 1.4	88.5 +/- 0.5	3	3	Hedges' g	0.68	-0.68 to 2.05	Higher, more favorable value with the intervention diet; unadjusted 95% CI included 0	Not applicable - effect size estimated from reported mean +/- SD
Teodosio et al. (2021)	Gilthead seabream (Sparus aurata)	Low-protein, low-fishmeal eco-diets, with or without a multi-additive package, under low-temperature rearing	ECOSup (42.0% protein; 7.5% fishmeal; 15% poultry meal; betaine, krill meal, soy lecithin, macroalgae and taurine)	COM (44.2% protein; 27.5% fishmeal commercial-like reference)	Apparent digestibility coefficient of Threonine (%)	85.8 +/- 1.4	87.5 +/- 0.4	3	3	Hedges' g	-1.32	-2.89 to 0.25	Lower, less favorable value with the intervention diet; unadjusted 95% CI included 0	Not applicable - effect size estimated from reported mean +/- SD
Teodosio et al. (2021)	Gilthead seabream (Sparus aurata)	Low-protein, low-fishmeal eco-diets, with or without a multi-additive package, under low-temperature rearing	ECOSup (42.0% protein; 7.5% fishmeal; 15% poultry meal; betaine, krill meal, soy lecithin, macroalgae and taurine)	COM (44.2% protein; 27.5% fishmeal commercial-like reference)	Apparent digestibility coefficient of Isoleucine (%)	91.0 +/- 1.1	91.7 +/- 0.3	3	3	Hedges' g	-0.69	-2.06 to 0.67	Lower, less favorable value with the intervention diet; unadjusted 95% CI included 0	Not applicable - effect size estimated from reported mean +/- SD
Teodosio et al. (2021)	Gilthead seabream (Sparus aurata)	Low-protein, low-fishmeal eco-diets, with or without a multi-additive package, under low-temperature rearing	ECOSup (42.0% protein; 7.5% fishmeal; 15% poultry meal; betaine, krill meal, soy lecithin, macroalgae and taurine)	COM (44.2% protein; 27.5% fishmeal commercial-like reference)	Apparent digestibility coefficient of Leucine (%)	89.1 +/- 1.4	91.2 +/- 0.2	3	3	Hedges' g	-1.68	-3.41 to 0.05	Lower, less favorable value with the intervention diet; unadjusted 95% CI included 0	Not applicable - effect size estimated from reported mean +/- SD

Study	Species	Strategy	Intervention	Comparator	Outcome and unit	Intervention mean +/- SD	Comparator mean +/- SD	n intervention (tanks)	n comparator (tanks)	Effect-size metric	Effect size	95% CI	Interpretation	Reason not estimated
Teodosio et al. (2021)	Gilthead seabream (Sparus aurata)	Low-protein, low-fishmeal eco-diets, with or without a multi-additive package, under low-temperature rearing	ECOSup (42.0% protein; 7.5% fishmeal; 15% poultry meal; betaine, krill meal, soy lecithin, macroalgae and taurine)	COM (44.2% protein; 27.5% fishmeal commercial-like reference)	Apparent digestibility coefficient of Valine (%)	91.0 +/- 1.2	91.3 +/- 0.3	3	3	Hedges' g	-0.27	-1.57 to 1.02	Lower, less favorable value with the intervention diet; unadjusted 95% CI included 0	Not applicable - effect size estimated from reported mean +/- SD
Teodosio et al. (2021)	Gilthead seabream (Sparus aurata)	Low-protein, low-fishmeal eco-diets, with or without a multi-additive package, under low-temperature rearing	ECOSup (42.0% protein; 7.5% fishmeal; 15% poultry meal; betaine, krill meal, soy lecithin, macroalgae and taurine)	COM (44.2% protein; 27.5% fishmeal commercial-like reference)	Apparent digestibility coefficient of Methionine (%)	89.3 +/- 1.2	91.3 +/- 0.5	3	3	Hedges' g	-1.74	-3.50 to 0.02	Lower, less favorable value with the intervention diet; unadjusted 95% CI included 0	Not applicable - effect size estimated from reported mean +/- SD
Teodosio et al. (2021)	Gilthead seabream (Sparus aurata)	Low-protein, low-fishmeal eco-diets, with or without a multi-additive package, under low-temperature rearing	ECOSup (42.0% protein; 7.5% fishmeal; 15% poultry meal; betaine, krill meal, soy lecithin, macroalgae and taurine)	COM (44.2% protein; 27.5% fishmeal commercial-like reference)	Apparent digestibility coefficient of Phenylalanine (%)	89.6 +/- 1.4	91.9 +/- 0.3	3	3	Hedges' g	-1.82	-3.61 to -0.02	Lower, less favorable value with the intervention diet; unadjusted 95% CI excluded 0	Not applicable - effect size estimated from reported mean +/- SD
Teodosio et al. (2021)	Gilthead seabream (Sparus aurata)	Low-protein, low-fishmeal eco-diets, with or without a multi-additive package, under low-temperature rearing	ECOSup (42.0% protein; 7.5% fishmeal; 15% poultry meal; betaine, krill meal, soy lecithin, macroalgae and taurine)	COM (44.2% protein; 27.5% fishmeal commercial-like reference)	Apparent digestibility coefficient of Cystine (%)	85.9 +/- 2.2	88.6 +/- 0.7	3	3	Hedges' g	-1.32	-2.90 to 0.25	Lower, less favorable value with the intervention diet; unadjusted 95% CI included 0	Not applicable - effect size estimated from reported mean +/- SD
Teodosio et al. (2021)	Gilthead seabream (Sparus aurata)	Low-protein, low-fishmeal eco-diets, with or without a multi-additive package, under low-temperature rearing	ECOSup (42.0% protein; 7.5% fishmeal; 15% poultry meal; betaine, krill meal, soy lecithin, macroalgae and taurine)	COM (44.2% protein; 27.5% fishmeal commercial-like reference)	Apparent digestibility coefficient of Tyrosine (%)	92.1 +/- 0.9	93.6 +/- 0.3	3	3	Hedges' g	-1.79	-3.57 to -0.01	Lower, less favorable value with the intervention diet; unadjusted 95% CI excluded 0	Not applicable - effect size estimated from reported mean +/- SD
Teodosio et al. (2021)	Gilthead seabream (Sparus aurata)	Low-protein, low-fishmeal eco-diets, with or without a multi-additive package, under low-temperature rearing	ECOSup (42.0% protein; 7.5% fishmeal; 15% poultry meal; betaine, krill meal, soy lecithin, macroalgae and taurine)	COM (44.2% protein; 27.5% fishmeal commercial-like reference)	Apparent digestibility coefficient of Aspartic acid + asparagine (%)	75.5 +/- 3.4	74.8 +/- 1.5	3	3	Hedges' g	0.21	-1.08 to 1.50	Higher, more favorable value with the intervention diet; unadjusted 95% CI included 0	Not applicable - effect size estimated from reported mean +/- SD
Teodosio et al. (2021)	Gilthead seabream (Sparus aurata)	Low-protein, low-fishmeal eco-diets, with or without a multi-additive package, under low-temperature rearing	ECOSup (42.0% protein; 7.5% fishmeal; 15% poultry meal; betaine, krill meal, soy lecithin, macroalgae and taurine)	COM (44.2% protein; 27.5% fishmeal commercial-like reference)	Apparent digestibility coefficient of Glutamic acid + glutamine (%)	88.8 +/- 1.3	90.5 +/- 0.5	3	3	Hedges' g	-1.38	-2.98 to 0.22	Lower, less favorable value with the intervention diet; unadjusted 95% CI included 0	Not applicable - effect size estimated from reported mean +/- SD
Teodosio et al. (2021)	Gilthead seabream (Sparus aurata)	Low-protein, low-fishmeal eco-diets, with or without a multi-additive package, under low-temperature rearing	ECOSup (42.0% protein; 7.5% fishmeal; 15% poultry meal; betaine, krill meal, soy lecithin, macroalgae and taurine)	COM (44.2% protein; 27.5% fishmeal commercial-like reference)	Apparent digestibility coefficient of Alanine (%)	90.6 +/- 1.1	91.0 +/- 0.2	3	3	Hedges' g	-0.4	-1.72 to 0.91	Lower, less favorable value with the intervention diet; unadjusted 95% CI included 0	Not applicable - effect size estimated from reported mean +/- SD
Teodosio et al. (2021)	Gilthead seabream (Sparus aurata)	Low-protein, low-fishmeal eco-diets, with or without a multi-additive package, under low-temperature rearing	ECOSup (42.0% protein; 7.5% fishmeal; 15% poultry meal; betaine, krill meal, soy lecithin, macroalgae and taurine)	COM (44.2% protein; 27.5% fishmeal commercial-like reference)	Apparent digestibility coefficient of Glycine (%)	94.8 +/- 0.7	95.2 +/- 0.2	3	3	Hedges' g	-0.62	-1.97 to 0.73	Lower, less favorable value with the intervention diet; unadjusted 95% CI included 0	Not applicable - effect size estimated from reported mean +/- SD
Teodosio et al. (2021)	Gilthead seabream (Sparus aurata)	Low-protein, low-fishmeal eco-diets, with or without a multi-additive package, under low-temperature rearing	ECOSup (42.0% protein; 7.5% fishmeal; 15% poultry meal; betaine, krill meal, soy lecithin, macroalgae and taurine)	COM (44.2% protein; 27.5% fishmeal commercial-like reference)	Apparent digestibility coefficient of Proline (%)	89.7 +/- 1.2	91.6 +/- 0.3	3	3	Hedges' g	-1.74	-3.50 to 0.02	Lower, less favorable value with the intervention diet; unadjusted 95% CI included 0	Not applicable - effect size estimated from reported mean +/- SD
Teodosio et al. (2021)	Gilthead seabream (Sparus aurata)	Low-protein, low-fishmeal eco-diets, with or without a multi-additive package, under low-temperature rearing	ECOSup (42.0% protein; 7.5% fishmeal; 15% poultry meal; betaine, krill meal, soy lecithin, macroalgae and taurine)	COM (44.2% protein; 27.5% fishmeal commercial-like reference)	Apparent digestibility coefficient of Serine (%)	84.6 +/- 2.2	86.1 +/- 0.3	3	3	Hedges' g	-0.76	-2.15 to 0.62	Lower, less favorable value with the intervention diet; unadjusted 95% CI included 0	Not applicable - effect size estimated from reported mean +/- SD
Teodosio et al. (2021)	Gilthead seabream (Sparus aurata)	Low-protein, low-fishmeal eco-diets, with or without a multi-additive package, under low-temperature rearing	ECOSup (42.0% protein; 7.5% fishmeal; 15% poultry meal; betaine, krill meal, soy lecithin, macroalgae and taurine)	COM (44.2% protein; 27.5% fishmeal commercial-like reference)	Apparent digestibility coefficient of Taurine (%)	87.3 +/- 1.3	85.2 +/- 0.7	3	3	Hedges' g	1.61	-0.09 to 3.31	Higher, more favorable value with the intervention diet; unadjusted 95% CI included 0	Not applicable - effect size estimated from reported mean +/- SD
Teodosio et al. (2021)	Gilthead seabream (Sparus aurata)	Low-protein, low-fishmeal eco-diets, with or without a multi-additive package, under low-temperature rearing	ECOSup (42.0% protein; 7.5% fishmeal; 15% poultry meal; betaine, krill meal, soy lecithin, macroalgae and taurine)	COM (44.2% protein; 27.5% fishmeal commercial-like reference)	Weight gain (%)	22.3 +/- 3.0	24.6 +/- 3.0	3	3	Hedges' g	-0.61	-1.96 to 0.74	Lower, less favorable value with the intervention diet; unadjusted 95% CI included 0	Not applicable - effect size estimated from reported mean +/- SD
Teodosio et al. (2021)	Gilthead seabream (Sparus aurata)	Low-protein, low-fishmeal eco-diets, with or without a multi-additive package, under low-temperature rearing	ECOSup (42.0% protein; 7.5% fishmeal; 15% poultry meal; betaine, krill meal, soy lecithin, macroalgae and taurine)	COM (44.2% protein; 27.5% fishmeal commercial-like reference)	Thermal growth coefficient	0.040 +/- 0.010	0.040 +/- 0.010	3	3	Hedges' g	0.0	-1.28 to 1.28	No observed mean difference; unadjusted 95% CI included 0	Not applicable - effect size estimated from reported mean +/- SD
Teodosio et al. (2021)	Gilthead seabream (Sparus aurata)	Low-protein, low-fishmeal eco-diets, with or without a multi-additive package, under low-temperature rearing	ECOSup (42.0% protein; 7.5% fishmeal; 15% poultry meal; betaine, krill meal, soy lecithin, macroalgae and taurine)	COM (44.2% protein; 27.5% fishmeal commercial-like reference)	Daily voluntary feed intake (%/day)	0.480 +/- 0.040	0.510 +/- 0.020	3	3	Hedges' g	-0.76	-2.14 to 0.63	Lower, less favorable value with the intervention diet; unadjusted 95% CI included 0	Not applicable - effect size estimated from reported mean +/- SD
Teodosio et al. (2021)	Gilthead seabream (Sparus aurata)	Low-protein, low-fishmeal eco-diets, with or without a multi-additive package, under low-temperature rearing	ECOSup (42.0% protein; 7.5% fishmeal; 15% poultry meal; betaine, krill meal, soy lecithin, macroalgae and taurine)	COM (44.2% protein; 27.5% fishmeal commercial-like reference)	Feed conversion ratio	1.90 +/- 0.20	1.80 +/- 0.10	3	3	Hedges' g (direction reversed)	-0.51	-1.83 to 0.82	Higher, less favorable value with the intervention diet; unadjusted 95% CI included 0	Not applicable - effect size estimated from reported mean +/- SD
Teodosio et al. (2021)	Gilthead seabream (Sparus aurata)	Low-protein, low-fishmeal eco-diets, with or without a multi-additive package, under low-temperature rearing	ECOSup (42.0% protein; 7.5% fishmeal; 15% poultry meal; betaine, krill meal, soy lecithin, macroalgae and taurine)	COM (44.2% protein; 27.5% fishmeal commercial-like reference)	Protein efficiency ratio	1.30 +/- 0.10	1.30 +/- 0.10	3	3	Hedges' g	0.0	-1.28 to 1.28	No observed mean difference; unadjusted 95% CI included 0	Not applicable - effect size estimated from reported mean +/- SD
Teodosio et al. (2021)	Gilthead seabream (Sparus aurata)	Low-protein, low-fishmeal eco-diets, with or without a multi-additive package, under low-temperature rearing	ECOSup (42.0% protein; 7.5% fishmeal; 15% poultry meal; betaine, krill meal, soy lecithin, macroalgae and taurine)	COM (44.2% protein; 27.5% fishmeal commercial-like reference)	Moisture (% wet weight)	65.1 +/- 1.2	66.0 +/- 0.3	3	3	Hedges' g	-0.82	-2.22 to 0.58	Lower measured value with the intervention diet; unadjusted 95% CI included 0	Not applicable - effect size estimated from reported mean +/- SD
Teodosio et al. (2021)	Gilthead seabream (Sparus aurata)	Low-protein, low-fishmeal eco-diets, with or without a multi-additive package, under low-temperature rearing	ECOSup (42.0% protein; 7.5% fishmeal; 15% poultry meal; betaine, krill meal, soy lecithin, macroalgae and taurine)	COM (44.2% protein; 27.5% fishmeal commercial-like reference)	Ash (% wet weight)	4.00 +/- 0.80	3.30 +/- 0.50	3	3	Hedges' g	0.84	-0.57 to 2.25	Higher measured value with the intervention diet; unadjusted 95% CI included 0	Not applicable - effect size estimated from reported mean +/- SD
Teodosio et al. (2021)	Gilthead seabream (Sparus aurata)	Low-protein, low-fishmeal eco-diets, with or without a multi-additive package, under low-temperature rearing	ECOSup (42.0% protein; 7.5% fishmeal; 15% poultry meal; betaine, krill meal, soy lecithin, macroalgae and taurine)	COM (44.2% protein; 27.5% fishmeal commercial-like reference)	Protein (% wet weight)	16.5 +/- 0.3	16.6 +/- 0.4	3	3	Hedges' g	-0.23	-1.52 to 1.06	Lower measured value with the intervention diet; unadjusted 95% CI included 0	Not applicable - effect size estimated from reported mean +/- SD
Teodosio et al. (2021)	Gilthead seabream (Sparus aurata)	Low-protein, low-fishmeal eco-diets, with or without a multi-additive package, under low-temperature rearing	ECOSup (42.0% protein; 7.5% fishmeal; 15% poultry meal; betaine, krill meal, soy lecithin, macroalgae and taurine)	COM (44.2% protein; 27.5% fishmeal commercial-like reference)	Fat (% wet weight)	12.8 +/- 0.9	12.3 +/- 0.9	3	3	Hedges' g	0.44	-0.87 to 1.76	Higher measured value with the intervention diet; unadjusted 95% CI included 0	Not applicable - effect size estimated from reported mean +/- SD
Teodosio et al. (2021)	Gilthead seabream (Sparus aurata)	Low-protein, low-fishmeal eco-diets, with or without a multi-additive package, under low-temperature rearing	ECOSup (42.0% protein; 7.5% fishmeal; 15% poultry meal; betaine, krill meal, soy lecithin, macroalgae and taurine)	COM (44.2% protein; 27.5% fishmeal commercial-like reference)	Phosphorus (% wet weight)	0.900 +/- 0.100	0.800 +/- 0.100	3	3	Hedges' g	0.8	-0.60 to 2.20	Higher measured value with the intervention diet; unadjusted 95% CI included 0	Not applicable - effect size estimated from reported mean +/- SD
Teodosio et al. (2021)	Gilthead seabream (Sparus aurata)	Low-protein, low-fishmeal eco-diets, with or without a multi-additive package, under low-temperature rearing	ECOSup (42.0% protein; 7.5% fishmeal; 15% poultry meal; betaine, krill meal, soy lecithin, macroalgae and taurine)	COM (44.2% protein; 27.5% fishmeal commercial-like reference)	Energy (MJ/kg)	8.10 +/- 0.40	7.80 +/- 0.10	3	3	Hedges' g	0.82	-0.58 to 2.22	Higher measured value with the intervention diet; unadjusted 95% CI included 0	Not applicable - effect size estimated from reported mean +/- SD
Teodosio et al. (2021)	Gilthead seabream (Sparus aurata)	Low-protein, low-fishmeal eco-diets, with or without a multi-additive package, under low-temperature rearing	ECOSup (42.0% protein; 7.5% fishmeal; 15% poultry meal; betaine, krill meal, soy lecithin, macroalgae and taurine)	COM (44.2% protein; 27.5% fishmeal commercial-like reference)	Liver protein (% dry weight)	32.1 +/- 1.6	35.6 +/- 3.0	3	3	Hedges' g	-1.16	-2.68 to 0.35	Lower measured value with the intervention diet; unadjusted 95% CI included 0	Not applicable - effect size estimated from reported mean +/- SD

Supplementary Table S3

Study	Species	Strategy	Intervention	Comparator	Outcome and unit	Intervention mean +/- SD	Comparator mean +/- SD	n intervention (tanks)	n comparator (tanks)	Effect-size metric	Effect size	95% CI	Interpretation	Reason not estimated
Teodosio et al. (2021)	Gilthead seabream (Sparus aurata)	Low-protein, low-fishmeal eco-diets, with or without a multi-additive package, under low-temperature rearing	ECOSup (42.0% protein; 7.5% fishmeal; 15% poultry meal; betaine, krill meal, soy lecithin, macroalgae and taurine)	COM (44.2% protein; 27.5% fishmeal commercial-like reference)	Liver fat (% dry weight)	22.1 +/- 4.8	29.1 +/- 3.4	3	3	Hedges' g	-1.35	-2.93 to 0.24	Lower measured value with the intervention diet; unadjusted 95% CI included 0	Not applicable - effect size estimated from reported mean +/- SD
Teodosio et al. (2021)	Gilthead seabream (Sparus aurata)	Low-protein, low-fishmeal eco-diets, with or without a multi-additive package, under low-temperature rearing	ECOSup (42.0% protein; 7.5% fishmeal; 15% poultry meal; betaine, krill meal, soy lecithin, macroalgae and taurine)	COM (44.2% protein; 27.5% fishmeal commercial-like reference)	Faecal nitrogen loss (mg N/kg/day)	24.6 +/- 1.9	39.2 +/- 1.3	3	3	Hedges' g (direction reversed)	7.17	2.04 to 12.31	Lower, more favorable value with the intervention diet; unadjusted 95% CI excluded 0	Not applicable - effect size estimated from reported mean +/- SD
Teodosio et al. (2021)	Gilthead seabream (Sparus aurata)	Low-protein, low-fishmeal eco-diets, with or without a multi-additive package, under low-temperature rearing	ECO (42.0% protein; 10% fishmeal; 15% poultry meal)	COM (44.2% protein; 27.5% fishmeal commercial-like reference)	Apparent digestibility coefficient of dry matter (%)	64.9 +/- 1.8	67.7 +/- 1.3	3	3	Hedges' g	-1.43	-3.04 to 0.19	Lower, less favorable value with the intervention diet; unadjusted 95% CI included 0	Not applicable - effect size estimated from reported mean +/- SD
Teodosio et al. (2021)	Gilthead seabream (Sparus aurata)	Low-protein, low-fishmeal eco-diets, with or without a multi-additive package, under low-temperature rearing	ECO (42.0% protein; 10% fishmeal; 15% poultry meal)	COM (44.2% protein; 27.5% fishmeal commercial-like reference)	Apparent digestibility coefficient of protein (%)	90.0 +/- 3.2	89.0 +/- 1.5	3	3	Hedges' g	0.32	-0.98 to 1.62	Higher, more favorable value with the intervention diet; unadjusted 95% CI included 0	Not applicable - effect size estimated from reported mean +/- SD
Teodosio et al. (2021)	Gilthead seabream (Sparus aurata)	Low-protein, low-fishmeal eco-diets, with or without a multi-additive package, under low-temperature rearing	ECO (42.0% protein; 10% fishmeal; 15% poultry meal)	COM (44.2% protein; 27.5% fishmeal commercial-like reference)	Apparent digestibility coefficient of fat (%)	94.6 +/- 1.4	95.4 +/- 0.8	3	3	Hedges' g	-0.56	-1.90 to 0.78	Lower, less favorable value with the intervention diet; unadjusted 95% CI included 0	Not applicable - effect size estimated from reported mean +/- SD
Teodosio et al. (2021)	Gilthead seabream (Sparus aurata)	Low-protein, low-fishmeal eco-diets, with or without a multi-additive package, under low-temperature rearing	ECO (42.0% protein; 10% fishmeal; 15% poultry meal)	COM (44.2% protein; 27.5% fishmeal commercial-like reference)	Apparent digestibility coefficient of phosphorus (%)	45.9 +/- 1.9	54.6 +/- 1.6	3	3	Hedges' g	-3.96	-6.99 to -0.93	Lower, less favorable value with the intervention diet; unadjusted 95% CI excluded 0	Not applicable - effect size estimated from reported mean +/- SD
Teodosio et al. (2021)	Gilthead seabream (Sparus aurata)	Low-protein, low-fishmeal eco-diets, with or without a multi-additive package, under low-temperature rearing	ECO (42.0% protein; 10% fishmeal; 15% poultry meal)	COM (44.2% protein; 27.5% fishmeal commercial-like reference)	Apparent digestibility coefficient of energy (%)	85.5 +/- 1.3	85.8 +/- 0.9	3	3	Hedges' g	-0.21	-1.50 to 1.07	Lower, less favorable value with the intervention diet; unadjusted 95% CI included 0	Not applicable - effect size estimated from reported mean +/- SD
Teodosio et al. (2021)	Gilthead seabream (Sparus aurata)	Low-protein, low-fishmeal eco-diets, with or without a multi-additive package, under low-temperature rearing	ECO (42.0% protein; 10% fishmeal; 15% poultry meal)	COM (44.2% protein; 27.5% fishmeal commercial-like reference)	Apparent digestibility coefficient of Arginine (%)	79.4 +/- 1.4	85.6 +/- 0.7	3	3	Hedges' g	-4.48	-7.84 to -1.12	Lower, less favorable value with the intervention diet; unadjusted 95% CI excluded 0	Not applicable - effect size estimated from reported mean +/- SD
Teodosio et al. (2021)	Gilthead seabream (Sparus aurata)	Low-protein, low-fishmeal eco-diets, with or without a multi-additive package, under low-temperature rearing	ECO (42.0% protein; 10% fishmeal; 15% poultry meal)	COM (44.2% protein; 27.5% fishmeal commercial-like reference)	Apparent digestibility coefficient of Histidine (%)	86.8 +/- 0.5	88.8 +/- 0.8	3	3	Hedges' g	-2.4	-4.50 to -0.30	Lower, less favorable value with the intervention diet; unadjusted 95% CI excluded 0	Not applicable - effect size estimated from reported mean +/- SD
Teodosio et al. (2021)	Gilthead seabream (Sparus aurata)	Low-protein, low-fishmeal eco-diets, with or without a multi-additive package, under low-temperature rearing	ECO (42.0% protein; 10% fishmeal; 15% poultry meal)	COM (44.2% protein; 27.5% fishmeal commercial-like reference)	Apparent digestibility coefficient of Lysine (%)	86.2 +/- 0.8	88.5 +/- 0.5	3	3	Hedges' g	-2.76	-5.06 to -0.46	Lower, less favorable value with the intervention diet; unadjusted 95% CI excluded 0	Not applicable - effect size estimated from reported mean +/- SD
Teodosio et al. (2021)	Gilthead seabream (Sparus aurata)	Low-protein, low-fishmeal eco-diets, with or without a multi-additive package, under low-temperature rearing	ECO (42.0% protein; 10% fishmeal; 15% poultry meal)	COM (44.2% protein; 27.5% fishmeal commercial-like reference)	Apparent digestibility coefficient of Threonine (%)	83.8 +/- 1.2	87.5 +/- 0.4	3	3	Hedges' g	-3.31	-5.94 to -0.68	Lower, less favorable value with the intervention diet; unadjusted 95% CI excluded 0	Not applicable - effect size estimated from reported mean +/- SD
Teodosio et al. (2021)	Gilthead seabream (Sparus aurata)	Low-protein, low-fishmeal eco-diets, with or without a multi-additive package, under low-temperature rearing	ECO (42.0% protein; 10% fishmeal; 15% poultry meal)	COM (44.2% protein; 27.5% fishmeal commercial-like reference)	Apparent digestibility coefficient of Isoleucine (%)	89.6 +/- 0.7	91.7 +/- 0.3	3	3	Hedges' g	-3.12	-5.63 to -0.61	Lower, less favorable value with the intervention diet; unadjusted 95% CI excluded 0	Not applicable - effect size estimated from reported mean +/- SD
Teodosio et al. (2021)	Gilthead seabream (Sparus aurata)	Low-protein, low-fishmeal eco-diets, with or without a multi-additive package, under low-temperature rearing	ECO (42.0% protein; 10% fishmeal; 15% poultry meal)	COM (44.2% protein; 27.5% fishmeal commercial-like reference)	Apparent digestibility coefficient of Leucine (%)	89.4 +/- 0.6	91.2 +/- 0.2	3	3	Hedges' g	-3.22	-5.79 to -0.65	Lower, less favorable value with the intervention diet; unadjusted 95% CI excluded 0	Not applicable - effect size estimated from reported mean +/- SD
Teodosio et al. (2021)	Gilthead seabream (Sparus aurata)	Low-protein, low-fishmeal eco-diets, with or without a multi-additive package, under low-temperature rearing	ECO (42.0% protein; 10% fishmeal; 15% poultry meal)	COM (44.2% protein; 27.5% fishmeal commercial-like reference)	Apparent digestibility coefficient of Valine (%)	89.3 +/- 0.6	91.3 +/- 0.3	3	3	Hedges' g	-3.37	-6.04 to -0.71	Lower, less favorable value with the intervention diet; unadjusted 95% CI excluded 0	Not applicable - effect size estimated from reported mean +/- SD
Teodosio et al. (2021)	Gilthead seabream (Sparus aurata)	Low-protein, low-fishmeal eco-diets, with or without a multi-additive package, under low-temperature rearing	ECO (42.0% protein; 10% fishmeal; 15% poultry meal)	COM (44.2% protein; 27.5% fishmeal commercial-like reference)	Apparent digestibility coefficient of Methionine (%)	90.3 +/- 0.6	91.3 +/- 0.5	3	3	Hedges' g	-1.45	-3.08 to 0.18	Lower, less favorable value with the intervention diet; unadjusted 95% CI included 0	Not applicable - effect size estimated from reported mean +/- SD
Teodosio et al. (2021)	Gilthead seabream (Sparus aurata)	Low-protein, low-fishmeal eco-diets, with or without a multi-additive package, under low-temperature rearing	ECO (42.0% protein; 10% fishmeal; 15% poultry meal)	COM (44.2% protein; 27.5% fishmeal commercial-like reference)	Apparent digestibility coefficient of Phenylalanine (%)	88.8 +/- 0.7	91.9 +/- 0.3	3	3	Hedges' g	-4.61	-8.04 to -1.17	Lower, less favorable value with the intervention diet; unadjusted 95% CI excluded 0	Not applicable - effect size estimated from reported mean +/- SD
Teodosio et al. (2021)	Gilthead seabream (Sparus aurata)	Low-protein, low-fishmeal eco-diets, with or without a multi-additive package, under low-temperature rearing	ECO (42.0% protein; 10% fishmeal; 15% poultry meal)	COM (44.2% protein; 27.5% fishmeal commercial-like reference)	Apparent digestibility coefficient of Cystine (%)	88.5 +/- 0.8	88.6 +/- 0.7	3	3	Hedges' g	-0.11	-1.39 to 1.18	Lower, less favorable value with the intervention diet; unadjusted 95% CI included 0	Not applicable - effect size estimated from reported mean +/- SD
Teodosio et al. (2021)	Gilthead seabream (Sparus aurata)	Low-protein, low-fishmeal eco-diets, with or without a multi-additive package, under low-temperature rearing	ECO (42.0% protein; 10% fishmeal; 15% poultry meal)	COM (44.2% protein; 27.5% fishmeal commercial-like reference)	Apparent digestibility coefficient of Tyrosine (%)	92.7 +/- 0.6	93.6 +/- 0.3	3	3	Hedges' g	-1.52	-3.17 to 0.14	Lower, less favorable value with the intervention diet; unadjusted 95% CI included 0	Not applicable - effect size estimated from reported mean +/- SD
Teodosio et al. (2021)	Gilthead seabream (Sparus aurata)	Low-protein, low-fishmeal eco-diets, with or without a multi-additive package, under low-temperature rearing	ECO (42.0% protein; 10% fishmeal; 15% poultry meal)	COM (44.2% protein; 27.5% fishmeal commercial-like reference)	Apparent digestibility coefficient of Aspartic acid + asparagine (%)	71.3 +/- 2.0	74.8 +/- 1.5	3	3	Hedges' g	-1.58	-3.27 to 0.10	Lower, less favorable value with the intervention diet; unadjusted 95% CI included 0	Not applicable - effect size estimated from reported mean +/- SD
Teodosio et al. (2021)	Gilthead seabream (Sparus aurata)	Low-protein, low-fishmeal eco-diets, with or without a multi-additive package, under low-temperature rearing	ECO (42.0% protein; 10% fishmeal; 15% poultry meal)	COM (44.2% protein; 27.5% fishmeal commercial-like reference)	Apparent digestibility coefficient of Glutamic acid + glutamine (%)	87.6 +/- 0.3	90.5 +/- 0.5	3	3	Hedges' g	-5.63	-9.73 to -1.52	Lower, less favorable value with the intervention diet; unadjusted 95% CI excluded 0	Not applicable - effect size estimated from reported mean +/- SD
Teodosio et al. (2021)	Gilthead seabream (Sparus aurata)	Low-protein, low-fishmeal eco-diets, with or without a multi-additive package, under low-temperature rearing	ECO (42.0% protein; 10% fishmeal; 15% poultry meal)	COM (44.2% protein; 27.5% fishmeal commercial-like reference)	Apparent digestibility coefficient of Alanine (%)	88.4 +/- 0.6	91.0 +/- 0.2	3	3	Hedges' g	-4.65	-8.12 to -1.18	Lower, less favorable value with the intervention diet; unadjusted 95% CI excluded 0	Not applicable - effect size estimated from reported mean +/- SD
Teodosio et al. (2021)	Gilthead seabream (Sparus aurata)	Low-protein, low-fishmeal eco-diets, with or without a multi-additive package, under low-temperature rearing	ECO (42.0% protein; 10% fishmeal; 15% poultry meal)	COM (44.2% protein; 27.5% fishmeal commercial-like reference)	Apparent digestibility coefficient of Glycine (%)	93.1 +/- 0.4	95.2 +/- 0.2	3	3	Hedges' g	-5.31	-9.21 to -1.41	Lower, less favorable value with the intervention diet; unadjusted 95% CI excluded 0	Not applicable - effect size estimated from reported mean +/- SD
Teodosio et al. (2021)	Gilthead seabream (Sparus aurata)	Low-protein, low-fishmeal eco-diets, with or without a multi-additive package, under low-temperature rearing	ECO (42.0% protein; 10% fishmeal; 15% poultry meal)	COM (44.2% protein; 27.5% fishmeal commercial-like reference)	Apparent digestibility coefficient of Proline (%)	88.7 +/- 0.8	91.6 +/- 0.3	3	3	Hedges' g	-3.84	-6.79 to -0.89	Lower, less favorable value with the intervention diet; unadjusted 95% CI excluded 0	Not applicable - effect size estimated from reported mean +/- SD
Teodosio et al. (2021)	Gilthead seabream (Sparus aurata)	Low-protein, low-fishmeal eco-diets, with or without a multi-additive package, under low-temperature rearing	ECO (42.0% protein; 10% fishmeal; 15% poultry meal)	COM (44.2% protein; 27.5% fishmeal commercial-like reference)	Apparent digestibility coefficient of Serine (%)	83.1 +/- 1.2	86.1 +/- 0.3	3	3	Hedges' g	-2.74	-5.04 to -0.45	Lower, less favorable value with the intervention diet; unadjusted 95% CI excluded 0	Not applicable - effect size estimated from reported mean +/- SD

Study	Species	Strategy	Intervention	Comparator	Outcome and unit	Intervention mean +/- SD	Comparator mean +/- SD	n intervention (tanks)	n comparator (tanks)	Effect-size metric	Effect size	95% CI	Interpretation	Reason not estimated
Teodosio et al. (2021)	Gilthead seabream (Sparus aurata)	Low-protein, low-fishmeal eco-diets, with or without a multi-additive package, under low-temperature rearing	ECO (42.0% protein; 10% fishmeal; 15% poultry meal)	COM (44.2% protein; 27.5% fishmeal commercial-like reference)	Apparent digestibility coefficient of Taurine (%)	85.1 +/- 1.5	85.2 +/- 0.7	3	3	Hedges' g	-0.07	-1.35 to 1.21	Lower, less favorable value with the intervention diet; unadjusted 95% CI included 0	Not applicable - effect size estimated from reported mean +/- SD
Teodosio et al. (2021)	Gilthead seabream (Sparus aurata)	Low-protein, low-fishmeal eco-diets, with or without a multi-additive package, under low-temperature rearing	ECO (42.0% protein; 10% fishmeal; 15% poultry meal)	COM (44.2% protein; 27.5% fishmeal commercial-like reference)	Weight gain (%)	18.5 +/- 6.6	24.6 +/- 3.0	3	3	Hedges' g	-0.95	-2.39 to 0.49	Lower, less favorable value with the intervention diet; unadjusted 95% CI included 0	Not applicable - effect size estimated from reported mean +/- SD
Teodosio et al. (2021)	Gilthead seabream (Sparus aurata)	Low-protein, low-fishmeal eco-diets, with or without a multi-additive package, under low-temperature rearing	ECO (42.0% protein; 10% fishmeal; 15% poultry meal)	COM (44.2% protein; 27.5% fishmeal commercial-like reference)	Thermal growth coefficient	0.020 +/- 0.010	0.040 +/- 0.010	3	3	Hedges' g	-1.6	-3.29 to 0.09	Lower, less favorable value with the intervention diet; unadjusted 95% CI included 0	Not applicable - effect size estimated from reported mean +/- SD
Teodosio et al. (2021)	Gilthead seabream (Sparus aurata)	Low-protein, low-fishmeal eco-diets, with or without a multi-additive package, under low-temperature rearing	ECO (42.0% protein; 10% fishmeal; 15% poultry meal)	COM (44.2% protein; 27.5% fishmeal commercial-like reference)	Daily voluntary feed intake (%/day)	0.440 +/- 0.040	0.510 +/- 0.020	3	3	Hedges' g	-1.77	-3.54 to 0.00	Lower, less favorable value with the intervention diet; unadjusted 95% CI included 0	Not applicable - effect size estimated from reported mean +/- SD
Teodosio et al. (2021)	Gilthead seabream (Sparus aurata)	Low-protein, low-fishmeal eco-diets, with or without a multi-additive package, under low-temperature rearing	ECO (42.0% protein; 10% fishmeal; 15% poultry meal)	COM (44.2% protein; 27.5% fishmeal commercial-like reference)	Feed conversion ratio	2.10 +/- 0.30	1.80 +/- 0.10	3	3	Hedges' g (direction reversed)	-1.07	-2.55 to 0.41	Higher, less favorable value with the intervention diet; unadjusted 95% CI included 0	Not applicable - effect size estimated from reported mean +/- SD
Teodosio et al. (2021)	Gilthead seabream (Sparus aurata)	Low-protein, low-fishmeal eco-diets, with or without a multi-additive package, under low-temperature rearing	ECO (42.0% protein; 10% fishmeal; 15% poultry meal)	COM (44.2% protein; 27.5% fishmeal commercial-like reference)	Protein efficiency ratio	1.20 +/- 0.20	1.30 +/- 0.10	3	3	Hedges' g	-0.51	-1.83 to 0.82	Lower, less favorable value with the intervention diet; unadjusted 95% CI included 0	Not applicable - effect size estimated from reported mean +/- SD
Teodosio et al. (2021)	Gilthead seabream (Sparus aurata)	Low-protein, low-fishmeal eco-diets, with or without a multi-additive package, under low-temperature rearing	ECO (42.0% protein; 10% fishmeal; 15% poultry meal)	COM (44.2% protein; 27.5% fishmeal commercial-like reference)	Moisture (% wet weight)	65.5 +/- 1.5	66.0 +/- 0.3	3	3	Hedges' g	-0.37	-1.68 to 0.94	Lower measured value with the intervention diet; unadjusted 95% CI included 0	Not applicable - effect size estimated from reported mean +/- SD
Teodosio et al. (2021)	Gilthead seabream (Sparus aurata)	Low-protein, low-fishmeal eco-diets, with or without a multi-additive package, under low-temperature rearing	ECO (42.0% protein; 10% fishmeal; 15% poultry meal)	COM (44.2% protein; 27.5% fishmeal commercial-like reference)	Ash (% wet weight)	4.00 +/- 0.40	3.30 +/- 0.50	3	3	Hedges' g	1.24	-0.30 to 2.78	Higher measured value with the intervention diet; unadjusted 95% CI included 0	Not applicable - effect size estimated from reported mean +/- SD
Teodosio et al. (2021)	Gilthead seabream (Sparus aurata)	Low-protein, low-fishmeal eco-diets, with or without a multi-additive package, under low-temperature rearing	ECO (42.0% protein; 10% fishmeal; 15% poultry meal)	COM (44.2% protein; 27.5% fishmeal commercial-like reference)	Protein (% wet weight)	16.7 +/- 0.8	16.6 +/- 0.4	3	3	Hedges' g	0.13	-1.16 to 1.41	Higher measured value with the intervention diet; unadjusted 95% CI included 0	Not applicable - effect size estimated from reported mean +/- SD
Teodosio et al. (2021)	Gilthead seabream (Sparus aurata)	Low-protein, low-fishmeal eco-diets, with or without a multi-additive package, under low-temperature rearing	ECO (42.0% protein; 10% fishmeal; 15% poultry meal)	COM (44.2% protein; 27.5% fishmeal commercial-like reference)	Fat (% wet weight)	12.4 +/- 0.7	12.3 +/- 0.9	3	3	Hedges' g	0.1	-1.18 to 1.38	Higher measured value with the intervention diet; unadjusted 95% CI included 0	Not applicable - effect size estimated from reported mean +/- SD
Teodosio et al. (2021)	Gilthead seabream (Sparus aurata)	Low-protein, low-fishmeal eco-diets, with or without a multi-additive package, under low-temperature rearing	ECO (42.0% protein; 10% fishmeal; 15% poultry meal)	COM (44.2% protein; 27.5% fishmeal commercial-like reference)	Phosphorus (% wet weight)	0.900 +/- 0.100	0.800 +/- 0.100	3	3	Hedges' g	0.8	-0.60 to 2.20	Higher measured value with the intervention diet; unadjusted 95% CI included 0	Not applicable - effect size estimated from reported mean +/- SD
Teodosio et al. (2021)	Gilthead seabream (Sparus aurata)	Low-protein, low-fishmeal eco-diets, with or without a multi-additive package, under low-temperature rearing	ECO (42.0% protein; 10% fishmeal; 15% poultry meal)	COM (44.2% protein; 27.5% fishmeal commercial-like reference)	Energy (MJ/kg)	8.10 +/- 0.50	7.80 +/- 0.10	3	3	Hedges' g	0.67	-0.70 to 2.03	Higher measured value with the intervention diet; unadjusted 95% CI included 0	Not applicable - effect size estimated from reported mean +/- SD
Teodosio et al. (2021)	Gilthead seabream (Sparus aurata)	Low-protein, low-fishmeal eco-diets, with or without a multi-additive package, under low-temperature rearing	ECO (42.0% protein; 10% fishmeal; 15% poultry meal)	COM (44.2% protein; 27.5% fishmeal commercial-like reference)	Liver protein (% dry weight)	35.4 +/- 5.2	35.6 +/- 3.0	3	3	Hedges' g	-0.04	-1.32 to 1.24	Lower measured value with the intervention diet; unadjusted 95% CI included 0	Not applicable - effect size estimated from reported mean +/- SD
Teodosio et al. (2021)	Gilthead seabream (Sparus aurata)	Low-protein, low-fishmeal eco-diets, with or without a multi-additive package, under low-temperature rearing	ECO (42.0% protein; 10% fishmeal; 15% poultry meal)	COM (44.2% protein; 27.5% fishmeal commercial-like reference)	Liver fat (% dry weight)	21.0 +/- 5.3	29.1 +/- 3.4	3	3	Hedges' g	-1.46	-3.09 to 0.17	Lower measured value with the intervention diet; unadjusted 95% CI included 0	Not applicable - effect size estimated from reported mean +/- SD
Teodosio et al. (2021)	Gilthead seabream (Sparus aurata)	Low-protein, low-fishmeal eco-diets, with or without a multi-additive package, under low-temperature rearing	ECO (42.0% protein; 10% fishmeal; 15% poultry meal)	COM (44.2% protein; 27.5% fishmeal commercial-like reference)	Faecal nitrogen loss (mg N/kg/day)	29.3 +/- 2.7	39.2 +/- 1.3	3	3	Hedges' g (direction reversed)	3.74	0.85 to 6.63	Lower, more favorable value with the intervention diet; unadjusted 95% CI excluded 0	Not applicable - effect size estimated from reported mean +/- SD
Teodosio et al. (2021)	Gilthead seabream (Sparus aurata)	Low-protein, low-fishmeal eco-diets, with or without a multi-additive package, under low-temperature rearing	ECOSup (42.0% protein; 7.5% fishmeal; 15% poultry meal; betaine, krill meal, soy lecithin, macroalgae and taurine)	ECO (42.0% protein; 10% fishmeal; 15% poultry meal)	Nitrogen gain (mg N/kg/day)	Exact value not printed	59.2 +/- 22.8	3	3	Not estimable	Not estimable	Not estimable	The paper described the ECOSup value as intermediate but did not provide the exact mean and SD.	The ECOSup value was described only as intermediate; its exact mean and SD were not printed.
Teodosio et al. (2021)	Gilthead seabream (Sparus aurata)	Low-protein, low-fishmeal eco-diets, with or without a multi-additive package, under low-temperature rearing	ECOSup (42.0% protein; 7.5% fishmeal; 15% poultry meal; betaine, krill meal, soy lecithin, macroalgae and taurine)	ECO (42.0% protein; 10% fishmeal; 15% poultry meal)	Metabolic nitrogen loss (mg N/kg/day)	Exact value not printed	203.9 +/- 24.8	3	3	Not estimable	Not estimable	Not estimable	The paper described the ECOSup value as intermediate but did not provide the exact mean and SD.	The ECOSup value was described only as intermediate; its exact mean and SD were not printed.
Teodosio et al. (2021)	Gilthead seabream (Sparus aurata)	Low-protein, low-fishmeal eco-diets, with or without a multi-additive package, under low-temperature rearing	ECOSup (42.0% protein; 7.5% fishmeal; 15% poultry meal; betaine, krill meal, soy lecithin, macroalgae and taurine)	COM (44.2% protein; 27.5% fishmeal commercial-like reference)	Nitrogen gain (mg N/kg/day)	Exact value not printed	72.7 +/- 15.4	3	3	Not estimable	Not estimable	Not estimable	The paper described the ECOSup value as intermediate but did not provide the exact mean and SD.	The ECOSup value was described only as intermediate; its exact mean and SD were not printed.
Teodosio et al. (2021)	Gilthead seabream (Sparus aurata)	Low-protein, low-fishmeal eco-diets, with or without a multi-additive package, under low-temperature rearing	ECOSup (42.0% protein; 7.5% fishmeal; 15% poultry meal; betaine, krill meal, soy lecithin, macroalgae and taurine)	COM (44.2% protein; 27.5% fishmeal commercial-like reference)	Metabolic nitrogen loss (mg N/kg/day)	Exact value not printed	245.2 +/- 23.2	3	3	Not estimable	Not estimable	Not estimable	The paper described the ECOSup value as intermediate but did not provide the exact mean and SD.	The ECOSup value was described only as intermediate; its exact mean and SD were not printed.
Teodosio et al. (2021)	Gilthead seabream (Sparus aurata)	Low-protein, low-fishmeal eco-diets, with or without a multi-additive package, under low-temperature rearing	ECO (42.0% protein; 10% fishmeal; 15% poultry meal)	COM (44.2% protein; 27.5% fishmeal commercial-like reference)	Nitrogen gain (mg N/kg/day)	59.2 +/- 22.8	72.7 +/- 15.4	3	3	Hedges' g	-0.56	-1.89 to 0.78	Lower, less favorable value with the intervention diet; unadjusted 95% CI included 0	Not applicable - effect size estimated from reported mean +/- SD
Teodosio et al. (2021)	Gilthead seabream (Sparus aurata)	Low-protein, low-fishmeal eco-diets, with or without a multi-additive package, under low-temperature rearing	ECO (42.0% protein; 10% fishmeal; 15% poultry meal)	COM (44.2% protein; 27.5% fishmeal commercial-like reference)	Metabolic nitrogen loss (mg N/kg/day)	203.9 +/- 24.8	245.2 +/- 23.2	3	3	Hedges' g (direction reversed)	1.38	-0.22 to 2.97	Lower, more favorable value with the intervention diet; unadjusted 95% CI included 0	Not applicable - effect size estimated from reported mean +/- SD
Teodosio et al. (2021)	Gilthead seabream (Sparus aurata)	Low-protein, low-fishmeal eco-diets, with or without a multi-additive package, under low-temperature rearing	ECOSup (42.0% protein; 7.5% fishmeal; 15% poultry meal; betaine, krill meal, soy lecithin, macroalgae and taurine)	ECO (42.0% protein; 10% fishmeal; 15% poultry meal)	Final body weight (g)	191.4 +/- 22.8	179.7 +/- 29.2	3	3	Not estimable at tank level	Not estimable	Not estimable	Higher final body weight is generally favorable.	The reported SD was based on individual fish (20 fish per tank; n = 60 individual fish per diet); tank-level SD was not reported.
Teodosio et al. (2021)	Gilthead seabream (Sparus aurata)	Low-protein, low-fishmeal eco-diets, with or without a multi-additive package, under low-temperature rearing	ECOSup (42.0% protein; 7.5% fishmeal; 15% poultry meal; betaine, krill meal, soy lecithin, macroalgae and taurine)	ECO (42.0% protein; 10% fishmeal; 15% poultry meal)	Condition factor	1.60 +/- 0.10	1.60 +/- 0.10	3	3	Not estimable at tank level	Not estimable	Not estimable	No dietary-treatment difference was reported.	The reported SD was based on individual fish (10 fish per tank; n = 30 individual fish per diet); tank-level SD was not reported.
Teodosio et al. (2021)	Gilthead seabream (Sparus aurata)	Low-protein, low-fishmeal eco-diets, with or without a multi-additive package, under low-temperature rearing	ECOSup (42.0% protein; 7.5% fishmeal; 15% poultry meal; betaine, krill meal, soy lecithin, macroalgae and taurine)	ECO (42.0% protein; 10% fishmeal; 15% poultry meal)	Hepatosomatic index (%)	2.50 +/- 0.40	2.30 +/- 0.40	3	3	Not estimable at tank level	Not estimable	Not estimable	ECOSup was significantly higher than COM; the authors related this to higher hepatic glycogen reserves.	The reported SD was based on individual fish (5 fish per tank; n = 15 individual fish per diet); tank-level SD was not reported.

Supplementary Table S3

Study	Species	Strategy	Intervention	Comparator	Outcome and unit	Intervention mean +/- SD	Comparator mean +/- SD	n intervention (tanks)	n comparator (tanks)	Effect-size metric	Effect size	95% CI	Interpretation	Reason not estimated
Teodosio et al. (2021)	Gilthead seabream (Sparus aurata)	Low-protein, low-fishmeal eco-diets, with or without a multi-additive package, under low-temperature rearing	ECOSup (42.0% protein; 7.5% fishmeal; 15% poultry meal; betaine, krill meal, soy lecithin, macroalgae and taurine)	ECO (42.0% protein; 10% fishmeal; 15% poultry meal)	Viscerosomatic index (%)	4.00 +/- 0.60	4.40 +/- 1.00	3	3	Not estimable at tank level	Not estimable	Not estimable	ECOSup was significantly lower than COM; the authors described this as positive from a consumer perspective.	The reported SD was based on individual fish (5 fish per tank; n = 15 individual fish per diet); tank-level SD was not reported.
Teodosio et al. (2021)	Gilthead seabream (Sparus aurata)	Low-protein, low-fishmeal eco-diets, with or without a multi-additive package, under low-temperature rearing	ECOSup (42.0% protein; 7.5% fishmeal; 15% poultry meal; betaine, krill meal, soy lecithin, macroalgae and taurine)	COM (44.2% protein; 27.5% fishmeal commercial-like reference)	Final body weight (g)	191.4 +/- 22.8	193.7 +/- 28.8	3	3	Not estimable at tank level	Not estimable	Not estimable	Higher final body weight is generally favorable.	The reported SD was based on individual fish (20 fish per tank; n = 60 individual fish per diet); tank-level SD was not reported.
Teodosio et al. (2021)	Gilthead seabream (Sparus aurata)	Low-protein, low-fishmeal eco-diets, with or without a multi-additive package, under low-temperature rearing	ECOSup (42.0% protein; 7.5% fishmeal; 15% poultry meal; betaine, krill meal, soy lecithin, macroalgae and taurine)	COM (44.2% protein; 27.5% fishmeal commercial-like reference)	Condition factor	1.60 +/- 0.10	1.60 +/- 0.10	3	3	Not estimable at tank level	Not estimable	Not estimable	No dietary-treatment difference was reported.	The reported SD was based on individual fish (10 fish per tank; n = 30 individual fish per diet); tank-level SD was not reported.
Teodosio et al. (2021)	Gilthead seabream (Sparus aurata)	Low-protein, low-fishmeal eco-diets, with or without a multi-additive package, under low-temperature rearing	ECOSup (42.0% protein; 7.5% fishmeal; 15% poultry meal; betaine, krill meal, soy lecithin, macroalgae and taurine)	COM (44.2% protein; 27.5% fishmeal commercial-like reference)	Hepatosomatic index (%)	2.50 +/- 0.40	2.20 +/- 0.30	3	3	Not estimable at tank level	Not estimable	Not estimable	ECOSup was significantly higher than COM; the authors related this to higher hepatic glycogen reserves.	The reported SD was based on individual fish (5 fish per tank; n = 15 individual fish per diet); tank-level SD was not reported.
Teodosio et al. (2021)	Gilthead seabream (Sparus aurata)	Low-protein, low-fishmeal eco-diets, with or without a multi-additive package, under low-temperature rearing	ECOSup (42.0% protein; 7.5% fishmeal; 15% poultry meal; betaine, krill meal, soy lecithin, macroalgae and taurine)	COM (44.2% protein; 27.5% fishmeal commercial-like reference)	Viscerosomatic index (%)	4.00 +/- 0.60	5.10 +/- 0.70	3	3	Not estimable at tank level	Not estimable	Not estimable	ECOSup was significantly lower than COM; the authors described this as positive from a consumer perspective.	The reported SD was based on individual fish (5 fish per tank; n = 15 individual fish per diet); tank-level SD was not reported.
Teodosio et al. (2021)	Gilthead seabream (Sparus aurata)	Low-protein, low-fishmeal eco-diets, with or without a multi-additive package, under low-temperature rearing	ECO (42.0% protein; 10% fishmeal; 15% poultry meal)	COM (44.2% protein; 27.5% fishmeal commercial-like reference)	Final body weight (g)	179.7 +/- 29.2	193.7 +/- 28.8	3	3	Not estimable at tank level	Not estimable	Not estimable	Higher final body weight is generally favorable.	The reported SD was based on individual fish (20 fish per tank; n = 60 individual fish per diet); tank-level SD was not reported.
Teodosio et al. (2021)	Gilthead seabream (Sparus aurata)	Low-protein, low-fishmeal eco-diets, with or without a multi-additive package, under low-temperature rearing	ECO (42.0% protein; 10% fishmeal; 15% poultry meal)	COM (44.2% protein; 27.5% fishmeal commercial-like reference)	Condition factor	1.60 +/- 0.10	1.60 +/- 0.10	3	3	Not estimable at tank level	Not estimable	Not estimable	No dietary-treatment difference was reported.	The reported SD was based on individual fish (10 fish per tank; n = 30 individual fish per diet); tank-level SD was not reported.
Teodosio et al. (2021)	Gilthead seabream (Sparus aurata)	Low-protein, low-fishmeal eco-diets, with or without a multi-additive package, under low-temperature rearing	ECO (42.0% protein; 10% fishmeal; 15% poultry meal)	COM (44.2% protein; 27.5% fishmeal commercial-like reference)	Hepatosomatic index (%)	2.30 +/- 0.40	2.20 +/- 0.30	3	3	Not estimable at tank level	Not estimable	Not estimable	ECOSup was significantly higher than COM; the authors related this to higher hepatic glycogen reserves.	The reported SD was based on individual fish (5 fish per tank; n = 15 individual fish per diet); tank-level SD was not reported.
Teodosio et al. (2021)	Gilthead seabream (Sparus aurata)	Low-protein, low-fishmeal eco-diets, with or without a multi-additive package, under low-temperature rearing	ECO (42.0% protein; 10% fishmeal; 15% poultry meal)	COM (44.2% protein; 27.5% fishmeal commercial-like reference)	Viscerosomatic index (%)	4.40 +/- 1.00	5.10 +/- 0.70	3	3	Not estimable at tank level	Not estimable	Not estimable	ECOSup was significantly lower than COM; the authors described this as positive from a consumer perspective.	The reported SD was based on individual fish (5 fish per tank; n = 15 individual fish per diet); tank-level SD was not reported.
Teodosio et al. (2021)	Gilthead seabream (Sparus aurata)	Low-protein, low-fishmeal eco-diets, with or without a multi-additive package, under low-temperature rearing	ECOSup (42.0% protein; 7.5% fishmeal; 15% poultry meal; betaine, krill meal, soy lecithin, macroalgae and taurine)	ECO (42.0% protein; 10% fishmeal; 15% poultry meal)	Survival rate (%)	Approximately 98%; exact diet-specific value not printed	Approximately 98%; exact diet-specific value not printed	3	3	Not estimable	Not estimable	Not estimable	Survival was high and not significantly affected by dietary treatment.	Only an approximate overall survival value was reported.
Teodosio et al. (2021)	Gilthead seabream (Sparus aurata)	Low-protein, low-fishmeal eco-diets, with or without a multi-additive package, under low-temperature rearing	ECOSup (42.0% protein; 7.5% fishmeal; 15% poultry meal; betaine, krill meal, soy lecithin, macroalgae and taurine)	ECO (42.0% protein; 10% fishmeal; 15% poultry meal)	Nutrient and energy retention	Results not shown	Results not shown	3	3	Not estimable	Not estimable	Not estimable	The authors reported no significant dietary-treatment effect.	Exact treatment means and SDs were not presented.
Teodosio et al. (2021)	Gilthead seabream (Sparus aurata)	Low-protein, low-fishmeal eco-diets, with or without a multi-additive package, under low-temperature rearing	ECOSup (42.0% protein; 7.5% fishmeal; 15% poultry meal; betaine, krill meal, soy lecithin, macroalgae and taurine)	COM (44.2% protein; 27.5% fishmeal commercial-like reference)	Survival rate (%)	Approximately 98%; exact diet-specific value not printed	Approximately 98%; exact diet-specific value not printed	3	3	Not estimable	Not estimable	Not estimable	Survival was high and not significantly affected by dietary treatment.	Only an approximate overall survival value was reported.
Teodosio et al. (2021)	Gilthead seabream (Sparus aurata)	Low-protein, low-fishmeal eco-diets, with or without a multi-additive package, under low-temperature rearing	ECOSup (42.0% protein; 7.5% fishmeal; 15% poultry meal; betaine, krill meal, soy lecithin, macroalgae and taurine)	COM (44.2% protein; 27.5% fishmeal commercial-like reference)	Nutrient and energy retention	Results not shown	Results not shown	3	3	Not estimable	Not estimable	Not estimable	The authors reported no significant dietary-treatment effect.	Exact treatment means and SDs were not presented.
Teodosio et al. (2021)	Gilthead seabream (Sparus aurata)	Low-protein, low-fishmeal eco-diets, with or without a multi-additive package, under low-temperature rearing	ECO (42.0% protein; 10% fishmeal; 15% poultry meal)	COM (44.2% protein; 27.5% fishmeal commercial-like reference)	Survival rate (%)	Approximately 98%; exact diet-specific value not printed	Approximately 98%; exact diet-specific value not printed	3	3	Not estimable	Not estimable	Not estimable	Survival was high and not significantly affected by dietary treatment.	Only an approximate overall survival value was reported.
Teodosio et al. (2021)	Gilthead seabream (Sparus aurata)	Low-protein, low-fishmeal eco-diets, with or without a multi-additive package, under low-temperature rearing	ECO (42.0% protein; 10% fishmeal; 15% poultry meal)	COM (44.2% protein; 27.5% fishmeal commercial-like reference)	Nutrient and energy retention	Results not shown	Results not shown	3	3	Not estimable	Not estimable	Not estimable	The authors reported no significant dietary-treatment effect.	Exact treatment means and SDs were not presented.

Note: Estimable outcomes use mean +/- SD and three independent tanks per diet. ECOSup versus ECO represents a complete dietary-package comparison rather than a single-additive contrast. FCR and nitrogen losses were direction-reversed. Individual-fish FBW, K, HSI and VSI summaries were retained but not converted to tank-level Hedges' g because tank-level SDs were unavailable. Pairwise 95% CIs are descriptive and unadjusted.

Source: Teodosio et al. (2021), https://doi.org/10.1016/j.aquaculture.2021.736494; accepted manuscript: https://arxiv.org/abs/2111.03565

Abbreviations: CI, confidence interval; DM, dry matter; FCR, feed conversion ratio; FM, fishmeal; GPx, glutathione peroxidase; IGF-I, insulin-like growth factor I; MPO, myeloperoxidase; NBT, nitroblue tetrazolium; PER, protein efficiency ratio; SD, standard deviation; SGR, specific growth rate; SOD, superoxide dismutase.
