# Supplementary material for: Nutritional Balancing Strategies in Low-Fishmeal Aquafeeds: A PRISMA 2020-Based Systematic Review Beyond Lysine and Methionine Supplementation
Source: Vet Sci. 2026 Jul 30;13(8):762. doi: 10.3390/vetsci13080762 (PMC13517252; doi:10.3390/vetsci13080762)
Supplement: Supplementary file 1 [file vetsci-13-00762-s001.zip › Supplementary_Table_S4.pdf]

Table S4. Search-strategy validation and retrieval audit of all 23 studies included in the systematic review.

The final search strategy was evaluated during manuscript revision using two complementary procedures. First, eight known eligible studies were selected as benchmark studies to test whether the final search strategy captured the principal intervention and outcome concepts represented in the review. Second, all 23 eligible studies were audited to confirm that they were captured by the combined search process and retained in the final eligible set. The audit documents capture by the combined database, citation-tracking, reference-list, Google Scholar, and publisher-platform searches; it does not imply that every record was indexed in or retrieved from every database searched.

A. Known-item validation using benchmark studies

Benchmark study	Concept domain represented	Key search concepts tested	Captured by final combined search	Validation result
Yamamoto et al. (2019) [35]	Essential amino acids beyond lysine and methionine	low fishmeal; histidine; yellowtail; growth; tissue free amino acids	Yes	Retrieved
Gunathilaka et al. (2019) [33]	Functional amino acids and non-protein nitrogenous compounds	low fishmeal; taurine; red seabream; growth; feed utilization; low temperature	Yes	Retrieved
Gunathilaka et al. (2021) [41]	Palatability and feed-intake enhancement	low fishmeal; shrimp protein hydrolysate; krill meal; red seabream; digestibility	Yes	Retrieved
Li et al. (2019) [49]	Digestibility improvement and enzyme supplementation	fishmeal-free; phytase; protease; tilapia; growth; nutrient utilization	Yes	Retrieved
Terrey et al. (2024) [37]	Mineral and phosphorus balancing	high plant meal; microbial phytase; Atlantic salmon; phosphorus digestibility; retention	Yes	Retrieved
Bae et al. (2020) [38]	Fermented ingredients and antioxidant/immune outcomes	fishmeal replacement; fermented soybean meal; whiteleg shrimp; antioxidant; lysozyme	Yes	Retrieved
Watson et al. (2013) [45]	Lipid-associated nutrient balancing	plant protein; taurine; alternative lipid sources; gilthead seabream; growth	Yes	Retrieved
Niu et al. (2019) [42]	Gut-health and microbiome-targeted strategies	low fishmeal; multi-strain probiotics; olive flounder; immunity; gut microbiota	Yes	Retrieved

Interpretation: all eight benchmark studies were captured by the final combined search process, supporting the sensitivity of the search across the principal nutritional-balancing concepts. This check confirms that the search could retrieve known eligible studies but does not, by itself, demonstrate that the evidence base was completely exhaustive.

B. Retrieval audit of all studies included in the qualitative synthesis

No.	Included study	Primary nutritional-balancing category	Species and diet context	Captured in final eligible set	Benchmark study
1	Gunathilaka et al. (2019) [33]	Functional amino acids: taurine	Red seabream; soybean-meal-based low-fishmeal diet; low-temperature season	Yes	Yes
2	Yoo and Bai (2014) [34]	Phytase and phosphorus balancing	Juvenile olive flounder; soybean-meal-based diet	Yes	No
3	Yamamoto et al. (2019) [35]	Essential amino acids: histidine	Fingerling yellowtail; fishmeal-based and low-fishmeal diets	Yes	Yes
4	Ramos-Pinto et al. (2019) [36]	Essential amino acids: tryptophan	Juvenile gilthead seabream; fishmeal-free diet	Yes	No
5	Terrey et al. (2024) [37]	Phytase and phosphorus balancing	Atlantic salmon; high-plant-meal diet	Yes	Yes
6	Bae et al. (2020) [38]	Fermented plant protein and health support	Juvenile whiteleg shrimp; plant proteins used as fishmeal replacers	Yes	Yes
7	Hamidoghli et al. (2023) [39]	Yeast hydrolysate and gut-health support	Whiteleg shrimp; low-fishmeal diet	Yes	No
8	Ramos-Pinto et al. (2021) [40]	Essential/functional amino acids: histidine, threonine, taurine	Gilthead seabream; short-term amino-acid supplementation	Yes	No
9	Gunathilaka et al. (2021) [41]	Protein hydrolysates and attractant-rich ingredients	Red seabream; low-fishmeal diet; shrimp hydrolysate and krill meal	Yes	Yes
10	Niu et al. (2019) [42]	Probiotics and microbiome-targeted strategy	Juvenile olive flounder; low-fishmeal diet	Yes	Yes
11	Kim et al. (2017) [43]	Functional amino acids: taurine	Olive flounder; low-water-temperature feeding	Yes	No
12	Kotzamanis et al. (2020) [44]	Taurine and product-quality support	European seabass; plant-protein-based diet	Yes	No
13	Watson et al. (2013) [45]	Lipid-associated nutrient balancing with taurine	Juvenile gilthead seabream; plant protein and alternative lipid sources	Yes	Yes
14	Wang et al. (2023) [46]	Essential amino acids: branched-chain amino acids	Turbot; imbalanced leucine, isoleucine, and valine supply	Yes	No
15	Shi et al. (2024) [47]	Hydroxyproline and collagen-related nutrients	Juvenile Pacific white shrimp; low-fishmeal diet	Yes	No
16	Cao et al. (2022) [48]	Hydroxyproline and collagen-related nutrients	Triploid crucian carp; hydroxyproline supplementation	Yes	No

No.	Included study	Primary nutritional-balancing category	Species and diet context	Captured in final eligible set	Benchmark study
17	Li et al. (2019) [49]	Digestibility improvement: phytase and protease	Hybrid tilapia; low-phosphorus, fishmeal-free diet	Yes	Yes
18	Sajjadi and Carter (2004) [50]	Phytase and phosphorus utilization	Atlantic salmon; canola-meal-based diet	Yes	No
19	Carter and Sajjadi (2011) [51]	Phytase-treated low-fishmeal formulation	Atlantic salmon; soy protein concentrate with graded phytase	Yes	No
20	Khosravi et al. (2018) [52]	Protein hydrolysates and health support	Juvenile olive flounder; low-fishmeal diet	Yes	No
21	Egerton et al. (2020) [53]	Fish protein hydrolysate supplementation	Atlantic salmon; plant-protein replacement of fishmeal	Yes	No
22	Zhao et al. (2023) [54]	Gut-functional additives	Largemouth bass; high-soybean-meal diet; tributyrin and alanyl-glutamine	Yes	No
23	Teodosio et al. (2021) [55]	Integrated nutritional strategy under environmental stress	Gilthead seabream; low-temperature conditions	Yes	No

Audit interpretation: all 23 studies included in the qualitative synthesis were present in the final eligible set. The “Benchmark study” column identifies the eight studies used specifically for the known-item validation; the remaining studies were included in the full-study retrieval audit.

C. Independent review of the search strategy

Search component reviewed	Review criterion	Outcome of independent review
Population and diet concepts	Coverage of fish, shrimp, crustaceans, aquaculture species, low-fishmeal, fishmeal-free, fishmeal-replacement, plant-protein, and alternative-protein terminology.	Relevant population and diet-context terms were included.
Nutritional-balancing interventions	Coverage of amino acids beyond lysine and methionine, taurine, phytase, minerals, vitamins, antioxidants, hydrolysates, fermented ingredients, enzymes, probiotics, prebiotics, nucleotides, organic acids, microbiota, and gut-health strategies.	The principal intervention concepts and relevant synonyms were included.
Outcome concepts	Coverage of growth, feed intake, feed efficiency, FCR, digestibility, nutrient retention, blood biochemistry, immunity, antioxidant status, intestinal condition, microbiota, stress tolerance, disease resistance, and product quality.	The predefined outcome domains were adequately represented.
Boolean structure and parentheses	Use of OR within concept blocks, AND between concept blocks, and parentheses to preserve the intended logic.	The Boolean structure was appropriate and internally consistent.
Phrase searching and terminology	Use of quotation marks for multiword terms and inclusion of wording variants such as “fishmeal” and “fish meal.”	Phrase searching and terminology variants were applied where supported.
Database-specific adaptation	Adaptation of field tags, phrase searching, operators, and syntax to the requirements of each database or platform.	Database-specific syntax and field restrictions were checked and adapted.
Known-item sensitivity	Capture of benchmark studies representing the principal nutritional-balancing concepts.	All eight benchmark studies were captured by the final combined search process.
Full-study retrieval audit	Confirmation that every study included in the qualitative synthesis was present in the final eligible set.	All 23 included studies were confirmed in the final eligible set.
Overall assessment	Sensitivity and conceptual coverage of the final search strategy in relation to the review questions and eligibility criteria.	The final strategy was considered sufficiently sensitive and appropriate for the systematic review.

Abbreviations: FCR, feed conversion ratio.

Note: The audit confirms presence in the final eligible set and does not imply that every study was indexed in or retrieved from every database searched.
