# Supplementary material for: Nutritional Balancing Strategies in Low-Fishmeal Aquafeeds: A PRISMA 2020-Based Systematic Review Beyond Lysine and Methionine Supplementation
Source: Vet Sci. 2026 Jul 30;13(8):762. doi: 10.3390/vetsci13080762 (PMC13517252; doi:10.3390/vetsci13080762)
Supplement: Supplementary file 1 [file vetsci-13-00762-s001.zip › Supplementary_Table_S5.pdf]

Supplementary Table S5. Exploratory subgroup classification and descriptive response patterns according to taxonomic group, reported life stage, and primary diet matrix.

Panel A. Study/comparison-level subgroup classification.

Study	Species	Taxonomic group	Reported life stage	Primary diet matrix	Strategy	Representative intervention–comparator comparison	Outcome	Hedges' g (95% CI)	CI+
Gunathilaka et al. (2019)	Red seabream	Marine non-salmonid finfish	Not explicitly specified	Soy-dominant	Taurine	1% taurine at 50% FM replacement vs matched unsupplemented diet	WG	2.10 (-0.06, 4.26)	No
Kim et al. (2017)	Olive flounder	Marine non-salmonid finfish	Juvenile	Other practical/mixed matrix	Taurine under low temperature	1.5% taurine vs control	SGR	1.37 (-0.41, 3.15)	No
Kotzamanis et al. (2020)	European seabass	Marine non-salmonid finfish	Not explicitly specified	Mixed plant-protein/fishmeal-free	Taurine	20 g/kg taurine vs low-FM control	SGR	1.56 (-0.26, 3.39)	No
Watson et al. (2013)	Gilthead seabream	Marine non-salmonid finfish	Juvenile	Mixed plant-protein/fishmeal-free	Alternative lipid-source balancing	Canola oil + DHA + ARA vs fish-oil control	SGR	-0.22 (-2.18, 1.75)	No
Yamamoto et al. (2019)	Yellowtail	Marine non-salmonid finfish	Fingerling	Other practical/mixed matrix	Histidine	High histidine in 15% FM diet vs matched low-FM diet	SGR	0.99 (-1.09, 3.06)	No
Ramos-Pinto et al. (2019)	Gilthead seabream	Marine non-salmonid finfish	Juvenile	Mixed plant-protein/fishmeal-free	Tryptophan	Additional tryptophan vs amino-acid-balanced fishmeal-free control	Performance outcomes	NE	NE
Ramos-Pinto et al. (2021)	Gilthead seabream	Marine non-salmonid finfish	Not explicitly specified	Other practical/mixed matrix	Threonine	0.75% threonine vs basal diet	RGR (4 weeks)	-0.06 (-1.66, 1.55)	No
Ramos-Pinto et al. (2021)	Gilthead seabream	Marine non-salmonid finfish	Not explicitly specified	Other practical/mixed matrix	Taurine	0.50% taurine vs basal diet	RGR (4 weeks)	-0.34 (-1.95, 1.27)	No
Ramos-Pinto et al. (2021)	Gilthead seabream	Marine non-salmonid finfish	Not explicitly specified	Other practical/mixed matrix	Histidine	0.40% histidine vs basal diet	RGR (4 weeks)	-0.20 (-1.80, 1.40)	No
Wang et al. (2023)	Turbot	Marine non-salmonid finfish	Not explicitly specified	Mixed plant-protein/fishmeal-free	BCAA balancing	Balanced BCAAs vs leucine-deficient diet	SGR	1.31 (-0.53, 3.15)	No
Shi et al. (2024)	Whiteleg shrimp	Crustaceans	Juvenile	Other practical/mixed matrix	Hydroxyproline	0.8% hydroxyproline vs 0% hydroxyproline	SGR	-0.63 (-2.29, 1.03)	No
Cao et al. (2022)	Triploid crucian carp	Freshwater finfish	Not explicitly specified	Mixed plant-protein/fishmeal-free	Hydroxyproline	2.0% crystalline hydroxyproline vs control	SGR	2.30 (0.04, 4.57)	Yes
Yoo and Bai (2014)	Olive flounder	Marine non-salmonid finfish	Juvenile	Soy-dominant	Phytase	2000 U/kg phytase at 40% FM replacement vs matched unsupplemented diet	SGR	0.00 (-1.51, 1.51)	No
Terrey et al. (2024)	Atlantic salmon	Salmonids	Not explicitly specified	Mixed plant-protein/fishmeal-free	Phytase	750 OTU/kg phytase vs high-plant negative control	SGR	2.28 (0.24, 4.31)	Yes
Li et al. (2019)	Hybrid tilapia	Freshwater finfish	Not explicitly specified	Mixed plant-protein/fishmeal-free	Phytase + protease	Phytase + protease in fishmeal-free diet vs unsupplemented diet	WG	5.10 (1.34, 8.86)	Yes
Sajjadi and Carter (2004)	Atlantic salmon	Salmonids	Not explicitly specified	Mixed plant-protein/fishmeal-free	Phytase	2000 U/kg phytase without inorganic P vs unsupplemented control	WG	1.09 (-0.40, 2.58)	No
Carter and Sajjadi (2011)	Atlantic salmon	Salmonids	Not explicitly specified	Soy-dominant	Phytase	Direct phytase at 4000 U/kg vs untreated-SPC control	WG	4.02 (0.95, 7.08)	Yes
Gunathilaka et al. (2021)	Red seabream	Marine non-salmonid finfish	Not explicitly specified	Soy-dominant	Shrimp protein hydrolysate	5% shrimp protein hydrolysate vs low-FM/SPC basal diet	SGR	6.44 (1.80, 11.08)	Yes
Khosravi et al. (2018)	Olive flounder	Marine non-salmonid finfish	Juvenile	Soy-dominant	Shrimp protein hydrolysate	Shrimp hydrolysate vs low-FM/high-SPC basal diet	SGR	2.25 (0.23, 4.28)	Yes
Egerton et al. (2020)	Atlantic salmon	Salmonids	Parr	Mixed plant-protein/fishmeal-free	Fish protein hydrolysate	Partly hydrolysed FPH vs plant-rich basal diet	FCR (direction reversed)	1.96 (0.10, 3.83)	Yes
Bae et al. (2020)	Whiteleg shrimp	Crustaceans	Juvenile	Soy-dominant	Fermented soybean meal	40% FM replacement with fermented soybean meal vs FM control	SGR	-0.65 (-2.00, 0.71)	No
Hamidoghli et al. (2023)	Whiteleg shrimp	Crustaceans	Juvenile	Other practical/mixed matrix	Yeast hydrolysate	4% yeast hydrolysate vs 10% FM control	SGR	1.89 (0.06, 3.73)	Yes

Study	Species	Taxonomic group	Reported life stage	Primary diet matrix	Strategy	Representative intervention–comparator comparison	Outcome	Hedges' g (95% CI)	CI+
Niu et al. (2019)	Olive flounder	Marine non-salmonid finfish	Juvenile	Other practical/mixed matrix	Multi-strain probiotic	Probiotic-supplemented low-FM diet vs unsupplemented diet	Performance outcomes	NE	NE
Zhao et al. (2023)	Largemouth bass	Freshwater finfish	Not explicitly specified	Soy-dominant	Alanyl-glutamine	2% alanyl-glutamine vs high-SBM control	SGR	1.63 (-0.08, 3.34)	No
Zhao et al. (2023)	Largemouth bass	Freshwater finfish	Not explicitly specified	Soy-dominant	Tributyrin	0.2% tributyrin vs high-SBM control	SGR	0.19 (-1.10, 1.48)	No
Teodosio et al. (2021)	Gilthead seabream	Marine non-salmonid finfish	Not explicitly specified	Other practical/mixed matrix	Multi-additive package under low temperature	Supplemented eco-diet vs unsupplemented eco-diet	TGC	1.60 (-0.09, 3.29)	No

Panel B. Descriptive subgroup summary based on the 24 representative performance comparisons shown in Figure 5.

Subgroup dimension	Subgroup	Studies, n	Representative comparisons, n	Hedges' g range	CI+, n/N	Exploratory interpretation
Taxonomic group	Marine non-salmonid finfish	13	13	−0.34 to 6.44	2/13	Largest and most diverse evidence group; wide range reflects multiple strategies and matrices rather than a uniform taxonomic response.
Taxonomic group	Salmonids	4	4	1.09 to 4.02	3/4	Mostly favorable estimates, but comparisons were concentrated in phytase and fish-protein-hydrolysate studies.
Taxonomic group	Freshwater finfish	3	4	0.19 to 5.10	2/4	Favorable responses occurred in selected hydroxyproline, enzyme, and gut-functional comparisons; evidence remained sparse.
Taxonomic group	Crustaceans	3	3	−0.65 to 1.89	1/3	Mixed performance responses; benefits were more frequently reported in gut, immune, disease-resistance, or product-quality domains.
Reported life stage	Juvenile, fingerling, or parr	11	9	−0.65 to 2.25	3/9	The evidence base was dominated by early production stages; two explicitly juvenile studies were not quantitatively estimable.
Reported life stage	Not explicitly specified	12	15	−0.34 to 6.44	5/15	This is a reporting-status subgroup, not a biological adult-stage comparator; no adequate adult or broodstock subgroup was available.
Primary diet matrix	Soy-dominant	7	8	−0.65 to 6.44	3/8	Highly variable responses across phytase, hydrolysate, fermented-soy, taurine, and gut-functional strategies.
Primary diet matrix	Mixed plant-protein or fishmeal-free	9	8	−0.22 to 5.10	4/8	More favorable effects were observed when the intervention addressed a defined limitation, especially phosphorus availability or protein-hydrolysate supply.
Primary diet matrix	Other practical or mixed alternative-protein	7	8	−0.63 to 1.89	1/8	Heterogeneous practical models, including low-temperature, amino-acid, shrimp, yeast, probiotic, and poultry-meal formulations; estimates were mostly imprecise.
Overall	All included studies	23	24	−0.65 to 6.44	8/24	Most estimates favored the intervention directionally, but confidence intervals were frequently wide and no effects were pooled.

Abbreviations: ARA, arachidonic acid; BCAA, branched-chain amino acid; CI, confidence interval; CI+, comparison whose 95% confidence interval excluded zero in favor of the intervention; DHA, docosahexaenoic acid; FCR, feed conversion ratio; FM, fishmeal; FPH, fish protein hydrolysate; NE, not estimable; P, phosphorus; RGR, relative growth rate; SBM, soybean meal; SGR, specific growth rate; SPC, soy protein concentrate; TGC, thermal growth coefficient; WG, weight gain.

Effect sizes are the same representative performance-related estimates used in Figure 5 and were not statistically pooled. Positive Hedges' g values favor the intervention; the direction of FCR was reversed so that positive values indicate improvement.

The 24 representative comparisons were derived from 21 studies. Ramos-Pinto et al. (2021) contributed three amino-acid comparisons, and Zhao et al. (2023) contributed two gut-functional comparisons; comparisons sharing a common control should not be interpreted as independent studies.

Reported life stage was classified only when an explicit juvenile, fingerling, or parr descriptor was available in the review dataset; life stage was not inferred solely from body weight.

Diet-matrix categories were broad exploratory classifications based on the dominant replacement or challenge component. Soy-dominant formulations contained soybean meal or soy protein concentrate as the principal matrix; mixed plant-protein/fishmeal-free formulations contained high-plant blends, canola meal, or fishmeal-free plant-based formulations; other practical/mixed matrices included practical low-fishmeal, low-temperature, shrimp, yeast, probiotic, and poultry-meal models.

Kim et al. (2017) was retained because it was part of the existing review dataset; however, all experimental diets in that study contained 50% fishmeal. Its inclusion should therefore be considered a sensitivity or scope issue rather than direct evidence from a low-fishmeal model.

Performance effect sizes were not estimable for Ramos-Pinto et al. (2019) and Niu et al. (2019) because exact numerical means, variance estimates, or independent experimental-unit information were unavailable in the supplied reports.
